# Supplementary material for: Structural adaptations for enhanced translation kinetics in evolved ribosomes
Source: Nucleic Acids Res. 2026 Jul 22;54(14):gkag703. doi: 10.1093/nar/gkag703 (PMC13389312; doi:10.1093/nar/gkag703)
Supplement: gkag703_Supplemental_Files [file gkag703_supplemental_files.zip › PDB_validation.zip!/VC_S4.4_D_1000305225_val-report-full-annotate_P1.pdf]

Full wwPDB EM Validation Report (i)

Feb 20, 2026 – 12:24 PM EST

PDB ID	:	11DG / pdb_000011dg
EMDB ID	:	EMD-75634
Title	:	Chimeric Escherichia coli 70S ribosome containing an evolved Vibrio cholerae 16S rRNA (VC-S4.4)
Deposited on	:	2026-02-18
Resolution	:	3.00 Å(reported)

This wwPDB validation report is for manuscript review

This is a Full wwPDB EM Validation Report.

This report is produced by the wwPDB biocuration pipeline after annotation of the structure.

We welcome your comments at *validation@mail.wwpdb.org*
A user guide is available at
https://www.wwpdb.org/validation/2017/EMValidationReportHelp
with specific help available everywhere you see the (i) symbol.

The types of validation reports are described at
http://www.wwpdb.org/validation/2017/FAQs#types.

The following versions of software and data (see references (i)) were used in the production of this report:

EMDB validation analysis	:	0.0.1.dev132
Mogul	:	2022.3.0, CSD as543be (2022)
MolProbity	:	4-5-2 with Phenix2.0
Percentile statistics	:	20231227.v01 (using entries in the PDB archive December 27th 2023)
EM percentile statistics	:	202505.v01 (Using data in the EMDB archive up until May 2025)
MapQ	:	1.9.13
Ideal geometry (proteins)	:	Engh & Huber (2001)
Ideal geometry (DNA, RNA)	:	Parkinson et al. (1996)

For Manuscript Review

1 Overall quality at a glance (i)

The following experimental techniques were used to determine the structure:
ELECTRON MICROSCOPY

The reported resolution of this entry is 3.00 Å.

Percentile scores (ranging between 0-100) for global validation metrics of the entry are shown in the following graphic. The table shows the number of entries on which the scores are based.

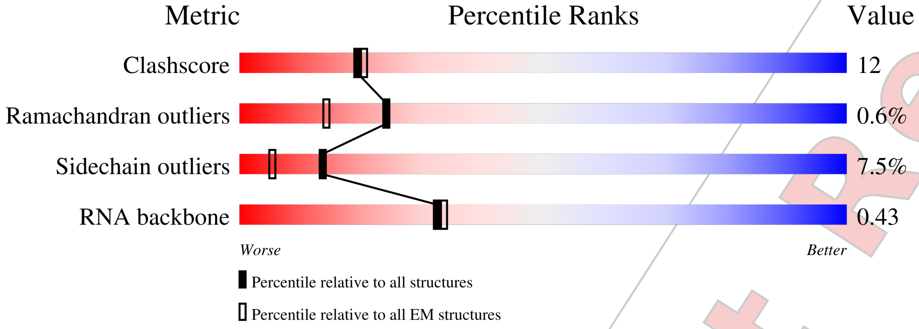

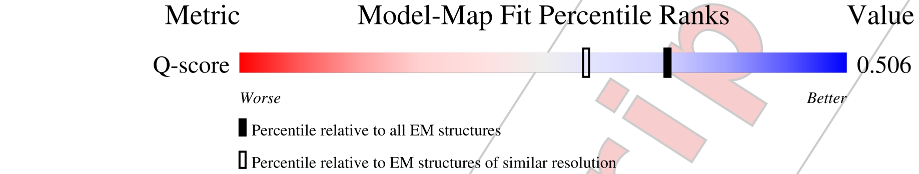

Metric	Whole archive (#Entries)	EM structures (#Entries)	Similar EM resolution (#Entries, resolution range(Å))
Clashscore	210492	15764	-
Ramachandran outliers	207382	16835	-
Sidechain outliers	206894	16415	-
RNA backbone	6643	2191	-
Q-score	-	25397	14081 (2.50 - 3.50)

The table below summarises the geometric issues observed across the polymeric chains and their fit to the map. The red, orange, yellow and green segments of the bar indicate the fraction of residues that contain outliers for >=3, 2, 1 and 0 types of geometric quality criteria respectively. A grey segment represents the fraction of residues that are not modelled. The numeric value for each fraction is indicated below the corresponding segment, with a dot representing fractions <=5% The upper red bar (where present) indicates the fraction of residues that have poor fit to the EM map (all-atom inclusion < 40%). The numeric value is given above the bar.

Mol	Chain	Length	Quality of chain
1	0	55	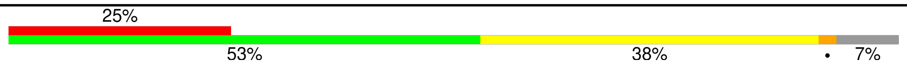

Continued on next page...

WORLDWIDE PDB PROTEIN DATA BANK
For Manuscript Review

Continued from previous page...

Mol	Chain	Length	Quality of chain
2	1	46	• 91% 9%
3	2	65	6% 68% 29% • •
4	3	38	29% 34% 58% 8%
5	4	70	39% 56% 29% • 14%
6	5	2	50% 50%
7	A	1517	• 46% 34% 19% •
8	B	241	24% 57% 33% • 7%
9	C	233	12% 50% 34% • 12%
10	D	206	13% 53% 39% 5% •
11	E	167	• 66% 25% • • 7%
12	F	135	26% 39% 34% • 24%
13	G	179	30% 50% 32% • 15%
14	H	130	• 71% 27% • •
15	I	130	8% 56% 38% • •
16	J	103	36% 48% 41% 6% • 5%
17	K	129	• 59% 32% 9%
18	L	124	• 62% 33% • •
19	M	118	32% 47% 43% 7% •
20	N	101	5% 69% 27% • •
21	O	89	• 71% 25% • • •
22	P	82	7% 65% 30% • •
23	Q	84	• 62% 24% 7% • 6%
24	R	75	16% 48% 40% 12%
25	S	92	28% 45% 37% 9% • 9%
26	T	87	5% 71% 25% • •

Continued on next page...

For Manuscript Review

Continued from previous page...

Mol	Chain	Length	Quality of chain
27	U	71	59% / 65% 31% • •
28	a	2904	• / 66% 24% 5% 5%
29	b	120	16% / 21% 56% 22% •
30	c	273	85% 14% •
31	d	209	• / 75% 22% •
32	e	201	6% / 69% 30% •
33	f	179	46% / 49% 48% • •
34	g	177	34% / 38% 55% 7% •
35	h	149	• / 23% • 72%
36	i	142	5% / 68% 29% •
37	j	123	75% 23% •
38	k	144	12% / 67% 33% •
39	l	136	• / 70% 28% •
40	m	127	76% 17% 7%
41	n	117	42% / 50% 45% • •
42	o	115	• / 84% 13% • •
43	p	118	8% / 64% 31% • •
44	q	103	14% / 57% 42% •
45	r	110	• / 89% 11%
46	s	100	78% 14% • 7%
47	t	104	• / 73% 23% • •
48	u	94	37% / 37% 56% 6%
49	v	85	11% / 53% 34% 5% 8%
50	w	78	78% 19% • •
51	x	63	• / 78% 21% •

Continued on next page...

For Manuscript Review

Continued from previous page...

Mol	Chain	Length	Quality of chain
52	y	59	7% 75% 24% •
53	z	57	• 79% 19% •

The following table lists non-polymeric compounds, carbohydrate monomers and non-standard residues in protein, DNA, RNA chains that are outliers for geometric or electron-density-fit criteria:

Mol	Type	Chain	Res	Chirality	Geometry	Clashes	Electron density
17	BH2	K	119	X	-	-	-

For Manuscript Review

2 Entry composition (i)

There are 57 unique types of molecules in this entry. The entry contains 138477 atoms, of which 0 are hydrogens and 0 are deuteriums.

In the tables below, the AltConf column contains the number of residues with at least one atom in alternate conformation and the Trace column contains the number of residues modelled with at most 2 atoms.

- Molecule 1 is a protein called 50S ribosomal protein L33.

Mol	Chain	Residues	Atoms				AltConf	Trace
			Total	C	N	O		
1	0	51	417	269	76	72	0	0

- Molecule 2 is a protein called Large ribosomal subunit protein bL34.

Mol	Chain	Residues	Atoms					AltConf	Trace
			Total	C	N	O	S		
2	1	46	377	228	90	57	2	0	0

- Molecule 3 is a protein called 50S ribosomal protein L35.

Mol	Chain	Residues	Atoms					AltConf	Trace
			Total	C	N	O	S		
3	2	64	504	323	105	74	2	0	0

- Molecule 4 is a protein called Large ribosomal subunit protein bL36A.

Mol	Chain	Residues	Atoms					AltConf	Trace
			Total	C	N	O	S		
4	3	38	302	185	65	48	4	0	0

- Molecule 5 is a protein called 50S ribosomal protein L31.

Mol	Chain	Residues	Atoms					AltConf	Trace
			Total	C	N	O	S		
5	4	60	480	299	90	85	6	0	0

- Molecule 6 is a RNA chain called E-site tRNA.

Mol	Chain	Residues	Atoms					AltConf	Trace
			Total	C	N	O	P		
6	5	2	42	19	8	13	2	0	0

For Manuscript Review

- Molecule 7 is a RNA chain called 16S ribosomal RNA.

Mol	Chain	Residues	Atoms					AltConf	Trace
7	A	1517	Total<br>32564	C<br>14534	N<br>5976	O<br>10537	P<br>1517	0	0

- Molecule 8 is a protein called Small ribosomal subunit protein uS2.

Mol	Chain	Residues	Atoms					AltConf	Trace
8	B	224	Total<br>1753	C<br>1109	N<br>315	O<br>321	S<br>8	0	0

- Molecule 9 is a protein called 30S ribosomal protein S3.

Mol	Chain	Residues	Atoms					AltConf	Trace
9	C	206	Total<br>1624	C<br>1028	N<br>305	O<br>288	S<br>3	0	0

- Molecule 10 is a protein called Small ribosomal subunit protein uS4.

Mol	Chain	Residues	Atoms					AltConf	Trace
10	D	205	Total<br>1642	C<br>1026	N<br>315	O<br>297	S<br>4	0	0

- Molecule 11 is a protein called Small ribosomal subunit protein uS5.

Mol	Chain	Residues	Atoms					AltConf	Trace
11	E	156	Total<br>1152	C<br>717	N<br>217	O<br>212	S<br>6	0	0

- Molecule 12 is a protein called Small ribosomal subunit protein bS6.

Mol	Chain	Residues	Atoms					AltConf	Trace
12	F	103	Total<br>839	C<br>530	N<br>151	O<br>151	S<br>7	0	0

- Molecule 13 is a protein called Small ribosomal subunit protein uS7.

Mol	Chain	Residues	Atoms					AltConf	Trace
13	G	153	Total<br>1203	C<br>750	N<br>231	O<br>218	S<br>4	0	0

- Molecule 14 is a protein called Small ribosomal subunit protein uS8.

For Manuscript Review

Mol	Chain	Residues	Atoms					AltConf	Trace
			Total	C	N	O	S		
14	H	129	978	616	173	183	6	0	0

- Molecule 15 is a protein called Small ribosomal subunit protein uS9.

Mol	Chain	Residues	Atoms					AltConf	Trace
			Total	C	N	O	S		
15	I	127	1022	634	206	179	3	0	0

- Molecule 16 is a protein called Small ribosomal subunit protein uS10.

Mol	Chain	Residues	Atoms					AltConf	Trace
			Total	C	N	O	S		
16	J	98	786	493	150	142	1	0	0

- Molecule 17 is a protein called Small ribosomal subunit protein uS11.

Mol	Chain	Residues	Atoms					AltConf	Trace
			Total	C	N	O	S		
17	K	117	876	540	173	160	3	0	0

There is a discrepancy between the modelled and reference sequences:

Chain	Residue	Modelled	Actual	Comment	Reference
K	119	BH2	ASN	conflict	UNP A7ZSI6

- Molecule 18 is a protein called 30S ribosomal protein S12.

Mol	Chain	Residues	Atoms					AltConf	Trace
			Total	C	N	O	S		
18	L	123	957	591	196	165	5	0	0

- Molecule 19 is a protein called Small ribosomal subunit protein uS13.

Mol	Chain	Residues	Atoms					AltConf	Trace
			Total	C	N	O	S		
19	M	115	891	552	179	157	3	0	0

- Molecule 20 is a protein called Small ribosomal subunit protein uS14.

For Manuscript Review

Mol	Chain	Residues	Atoms					AltConf	Trace
20	N	100	Total 805	C 499	N 164	O 139	S 3	0	0

- Molecule 21 is a protein called Small ribosomal subunit protein uS15.

Mol	Chain	Residues	Atoms					AltConf	Trace
21	O	88	Total 714	C 439	N 144	O 130	S 1	0	0

- Molecule 22 is a protein called Small ribosomal subunit protein bS16.

Mol	Chain	Residues	Atoms					AltConf	Trace
22	P	81	Total 643	C 403	N 127	O 112	S 1	0	0

- Molecule 23 is a protein called Small ribosomal subunit protein uS17.

Mol	Chain	Residues	Atoms					AltConf	Trace
23	Q	79	Total 641	C 406	N 120	O 112	S 3	0	0

- Molecule 24 is a protein called Small ribosomal subunit protein bS18.

Mol	Chain	Residues	Atoms					AltConf	Trace
24	R	66	Total 544	C 345	N 102	O 96	S 1	0	0

- Molecule 25 is a protein called Small ribosomal subunit protein uS19.

Mol	Chain	Residues	Atoms					AltConf	Trace
25	S	84	Total 668	C 427	N 127	O 112	S 2	0	0

- Molecule 26 is a protein called Small ribosomal subunit protein bS20.

Mol	Chain	Residues	Atoms					AltConf	Trace
26	T	86	Total 669	C 412	N 138	O 115	S 4	0	0

There is a discrepancy between the modelled and reference sequences:

For Manuscript Review

Chain	Residue	Modelled	Actual	Comment	Reference
T	58	CYS	VAL	conflict	UNP A7ZHB2

- Molecule 27 is a protein called Small ribosomal subunit protein bS21.

Mol	Chain	Residues	Atoms					AltConf	Trace
27	U	70	Total<br>589	C<br>366	N<br>125	O<br>97	S<br>1	0	0

- Molecule 28 is a RNA chain called 23S ribosomal RNA.

Mol	Chain	Residues	Atoms					AltConf	Trace
28	a	2753	Total<br>59130	C<br>26384	N<br>10897	O<br>19096	P<br>2753	0	0

- Molecule 29 is a RNA chain called 5S ribosomal RNA.

Mol	Chain	Residues	Atoms					AltConf	Trace
29	b	119	Total<br>2549	C<br>1135	N<br>466	O<br>829	P<br>119	0	0

- Molecule 30 is a protein called 50S ribosomal protein L2.

Mol	Chain	Residues	Atoms					AltConf	Trace
30	c	271	Total<br>2082	C<br>1288	N<br>423	O<br>364	S<br>7	0	0

- Molecule 31 is a protein called 50S ribosomal protein L3.

Mol	Chain	Residues	Atoms					AltConf	Trace
31	d	209	Total<br>1566	C<br>980	N<br>288	O<br>294	S<br>4	0	0

- Molecule 32 is a protein called Large ribosomal subunit protein uL4.

Mol	Chain	Residues	Atoms					AltConf	Trace
32	e	201	Total<br>1552	C<br>974	N<br>283	O<br>290	S<br>5	0	0

- Molecule 33 is a protein called Large ribosomal subunit protein uL5.

For Manuscript Review

Mol	Chain	Residues	Atoms					AltConf	Trace
			Total	C	N	O	S		
33	f	177	1410	899	249	256	6	0	0

- Molecule 34 is a protein called Large ribosomal subunit protein uL6.

Mol	Chain	Residues	Atoms					AltConf	Trace
			Total	C	N	O	S		
34	g	176	1323	832	243	246	2	0	0

- Molecule 35 is a protein called Large ribosomal subunit protein bL9.

Mol	Chain	Residues	Atoms					AltConf	Trace
			Total	C	N	O	S		
35	h	41	303	194	54	54	1	0	0

- Molecule 36 is a protein called Large ribosomal subunit protein uL13.

Mol	Chain	Residues	Atoms					AltConf	Trace
			Total	C	N	O	S		
36	i	142	1129	714	212	199	4	0	0

- Molecule 37 is a protein called Large ribosomal subunit protein uL14.

Mol	Chain	Residues	Atoms					AltConf	Trace
			Total	C	N	O	S		
37	j	123	946	593	181	166	6	0	0

- Molecule 38 is a protein called Large ribosomal subunit protein uL15.

Mol	Chain	Residues	Atoms					AltConf	Trace
			Total	C	N	O	S		
38	k	144	1053	654	207	190	2	0	0

- Molecule 39 is a protein called Large ribosomal subunit protein uL16.

Mol	Chain	Residues	Atoms					AltConf	Trace
			Total	C	N	O	S		
39	l	136	1075	686	205	177	7	0	0

- Molecule 40 is a protein called Large ribosomal subunit protein bL17.

For Manuscript Review

Mol	Chain	Residues	Atoms					AltConf	Trace
			Total	C	N	O	S		
40	m	118	945	585	194	161	5	0	0

- Molecule 41 is a protein called Large ribosomal subunit protein uL18.

Mol	Chain	Residues	Atoms				AltConf	Trace
			Total	C	N	O		
41	n	116	892	552	178	162	0	0

- Molecule 42 is a protein called Large ribosomal subunit protein bL19.

Mol	Chain	Residues	Atoms					AltConf	Trace
			Total	C	N	O	S		
42	o	114	917	574	179	163	1	0	0

- Molecule 43 is a protein called 50S ribosomal protein L20.

Mol	Chain	Residues	Atoms				AltConf	Trace
			Total	C	N	O		
43	p	117	947	604	192	151	0	0

- Molecule 44 is a protein called Large ribosomal subunit protein bL21.

Mol	Chain	Residues	Atoms					AltConf	Trace
			Total	C	N	O	S		
44	q	103	816	516	153	145	2	0	0

- Molecule 45 is a protein called Large ribosomal subunit protein uL22.

Mol	Chain	Residues	Atoms					AltConf	Trace
			Total	C	N	O	S		
45	r	110	857	532	166	156	3	0	0

- Molecule 46 is a protein called 50S ribosomal protein L23.

Mol	Chain	Residues	Atoms					AltConf	Trace
			Total	C	N	O	S		
46	s	93	738	466	139	131	2	0	0

- Molecule 47 is a protein called 50S ribosomal protein L24.

For Manuscript Review

Mol	Chain	Residues	Atoms				AltConf	Trace
			Total	C	N	O		
47	t	102	779	492	146	141	0	0

- Molecule 48 is a protein called Large ribosomal subunit protein bL25.

Mol	Chain	Residues	Atoms					AltConf	Trace
			Total	C	N	O	S		
48	u	94	753	479	137	134	3	0	0

- Molecule 49 is a protein called 50S ribosomal protein L27.

Mol	Chain	Residues	Atoms					AltConf	Trace
			Total	C	N	O	S		
49	v	78	586	362	116	107	1	0	0

- Molecule 50 is a protein called 50S ribosomal protein L28.

Mol	Chain	Residues	Atoms					AltConf	Trace
			Total	C	N	O	S		
50	w	77	625	388	129	106	2	0	0

- Molecule 51 is a protein called Large ribosomal subunit protein uL29.

Mol	Chain	Residues	Atoms					AltConf	Trace
			Total	C	N	O	S		
51	x	62	501	308	98	94	1	0	0

- Molecule 52 is a protein called 50S ribosomal protein L30.

Mol	Chain	Residues	Atoms					AltConf	Trace
			Total	C	N	O	S		
52	y	58	449	281	87	79	2	0	0

- Molecule 53 is a protein called 50S ribosomal protein L32.

Mol	Chain	Residues	Atoms					AltConf	Trace
			Total	C	N	O	S		
53	z	56	444	269	94	80	1	0	0

- Molecule 54 is ZINC ION (CCD ID: ZN) (formula: Zn).

For Manuscript Review

Mol	Chain	Residues	Atoms		AltConf
54	3	1	Total 1	Zn 1	0
54	4	1	Total 1	Zn 1	0

- Molecule 55 is MAGNESIUM ION (CCD ID: MG) (formula: Mg).

Mol	Chain	Residues	Atoms		AltConf
55	A	55	Total 55	Mg 55	0
55	Q	1	Total 1	Mg 1	0
55	a	207	Total 207	Mg 207	0
55	b	5	Total 5	Mg 5	0
55	c	1	Total 1	Mg 1	0
55	m	1	Total 1	Mg 1	0
55	n	1	Total 1	Mg 1	0
55	z	1	Total 1	Mg 1	0

- Molecule 56 is SPERMIDINE (CCD ID: SPD) (formula: $C_7H_{19}N_3$).

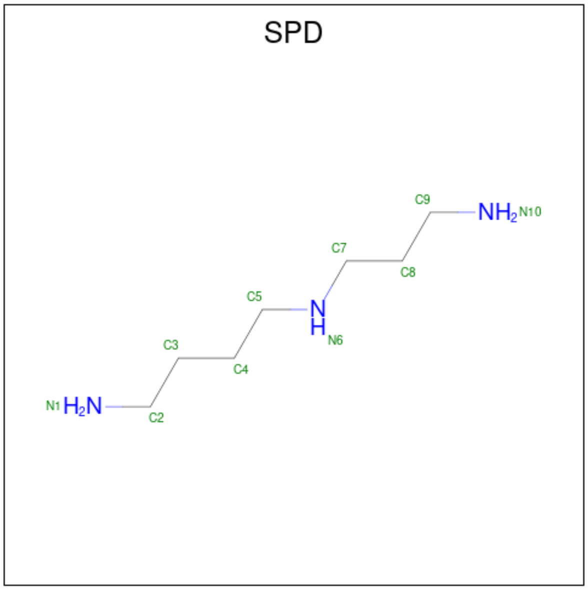

For Manuscript Review

Mol	Chain	Residues	Atoms	AltConf
56	a	1	Total C N<br>10 7 3	0
56	a	1	Total C N<br>10 7 3	0
56	a	1	Total C N<br>10 7 3	0
56	a	1	Total C N<br>10 7 3	0
56	a	1	Total C N<br>10 7 3	0
56	a	1	Total C N<br>10 7 3	0
56	a	1	Total C N<br>10 7 3	0
56	a	1	Total C N<br>10 7 3	0
56	a	1	Total C N<br>10 7 3	0
56	a	1	Total C N<br>10 7 3	0
56	a	1	Total C N<br>10 7 3	0
56	a	1	Total C N<br>10 7 3	0
56	a	1	Total C N<br>10 7 3	0
56	a	1	Total C N<br>10 7 3	0

- Molecule 57 is SPERMINE (CCD ID: SPM) (formula: $C_{10}H_{26}N_4$).

For Manuscript Review

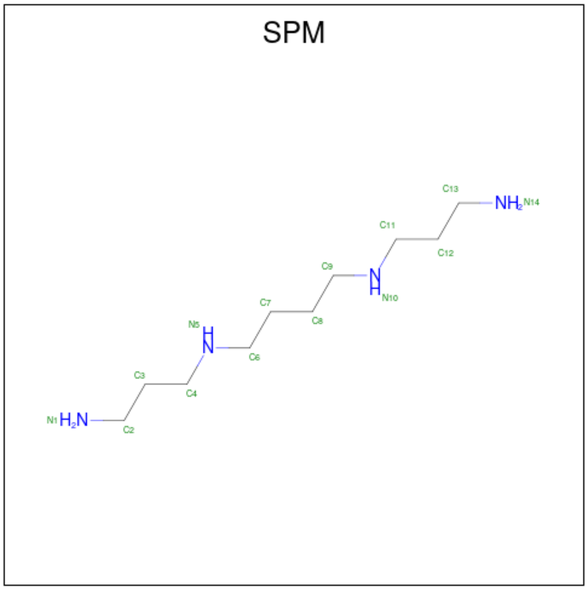

Mol	Chain	Residues	Atoms			AltConf
			Total	C	N	
57	a	1	14	10	4	0

For Manuscript Review

3 Residue-property plots

These plots are drawn for all protein, RNA, DNA and oligosaccharide chains in the entry. The first graphic for a chain summarises the proportions of the various outlier classes displayed in the second graphic. The second graphic shows the sequence view annotated by issues in geometry and atom inclusion in map density. Residues are color-coded according to the number of geometric quality criteria for which they contain at least one outlier: green = 0, yellow = 1, orange = 2 and red = 3 or more. A red diamond above a residue indicates a poor fit to the EM map for this residue (all-atom inclusion < 40%). Stretches of 2 or more consecutive residues without any outlier are shown as a green connector. Residues present in the sample, but not in the model, are shown in grey.

- Molecule 1: 50S ribosomal protein L33

Chain 0:

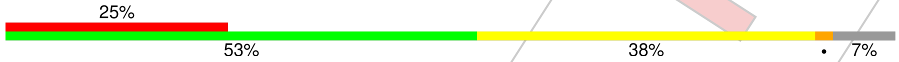

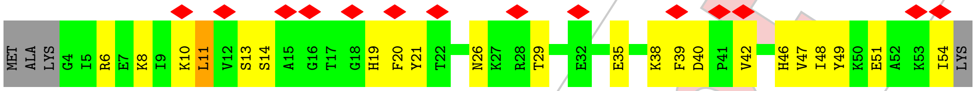

- Molecule 2: Large ribosomal subunit protein bL34

Chain 1:

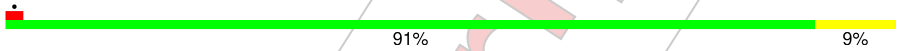

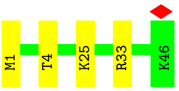

- Molecule 3: 50S ribosomal protein L35

Chain 2:

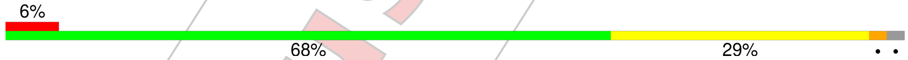

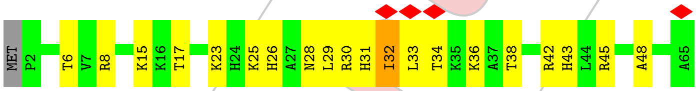

- Molecule 4: Large ribosomal subunit protein bL36A

Chain 3:

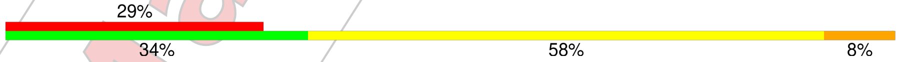

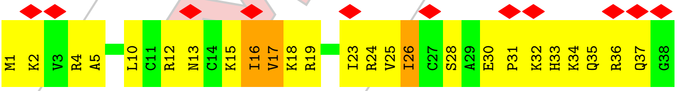

- Molecule 5: 50S ribosomal protein L31

Chain 4:

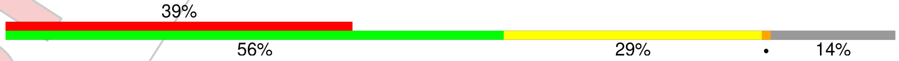

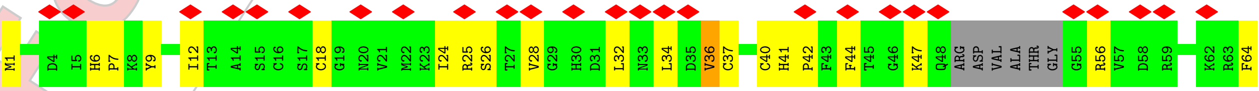

For Manuscript Review

N65 I66 PRO GLY SER LYS

• Molecule 6: E-site tRNA

Chain 5: 50% 50%

C75 A76

• Molecule 7: 16S ribosomal RNA

Chain A: 46% 34% 19%

A2 A3 U4 U5 G6 A7 A8 G9 A10 G11 G15 A16 U17 C18 G22 A28 A32 A33 C34 G35 C36 U37 G38 G39 C40 G41 G42 C43 C47 C48 A51 G61 U62 C63 G64 A65 G66 C67 G68 G69 G72 C73 A74 C75 A76 G77 A78 G79 G80 A81 A82 C83

U84 U85 G86 U87 U88 C89 C90 U91 U92 G93 G94 G95 U96 G97 G98 C99 G100 A101 G102 G105 C106 G107 G108 A109 A116 A120 U121 G122 C123 C124 G127 G128 A129 A130 A131 U132 U133 G134 C135 C136 A143 G144 G145 G146 A152 C153 C154 U157 A162 C163 G164 A165

U166 G167 A171 A172 U173 C176 G177 C178 A179 U180 A181 A182 C183 C184 U185 C186 A192 C193 C194 A195 A196 A197 G198 C199 A200 G201 G202 G203 G204 C214 C215 U216 C219 G220 C221 U222 A223 G227 A228 U229 A230 U231 G232 C235 A236 G237 G238 U239 G240 U245 A246 G247

G251 G254 G255 U256 G259 G260 U261 A262 G266 C267 U268 C272 A273 A274 C280 G281 A282 U283 C284 C285 C286 G289 C295 U296 G297 A298 G299 A300 G301 A306 U323 G324 A325 G326 A327 C328 A329 C330 G331 G332 U333 C334 G337 A338 A344 C345 G346 G347 G348

G351 C352 A353 G354 G362 A363 A364 U365 A366 U367 C372 A373 A374 U375 G376 G377 A378 C379 G380 C381 A382 A383 G384 C385 C386 G391 C392 A393 A397 C401 G402 C403 G404 U405 A406 U407 G410 A411 C412 A413 A414 A415 G416 U420 U421 C422 G423 G424 G425 C426 U427 G428

U429 A430 A431 A432 A435 C436 U437 U438 U439 C440 A441 G442 G445 G446 A447 A448 G449 G450 A451 A452 G453 G454 U455 G456 G457 U458 A459 A460 A461 G462 C463 U464 A465 A466 U467 A468 C469 C470 U471 U472 A473 A474 U475 C476 A477 U480 G481 A482 C483 G484 U485 U486 A487 C488 C489 U490 A491

C492 A493 G497 A498 A499 G500 C501 A502 G505 G506 C507 U508 C511 U512 C513 C514 G515 U516 G517 C518 G521 C522 A523 G524 C525 C526 G527 A532 A533 U534 A535 C536 G537 G538 A539 G540 U543 G544 A547 G548 C549 G550 U555 C556 A559 A563 C564 U565 A566

A573 A574 G575 C576 G577 C578 U586 G587 G588 U589 G592 A596 G597 U598 C599 A600 G601 A602 U603 G604 U605 G606 A607 A608 A609 G610 C611 C612 C613 C614 G615 C616 G617 C618 U619 C620 A621 A622 G626 C632 G633 U637 U638 G639 A642 C643 U644 U652 A653 G654 U657

A658 C659 U660 G661 U662 A663 G664 A665 A673 G674 A675 U678 U679 G683 A687 G690 A695 U707 A712 G713 G714 A715 A716 U717 A718 C719 C720 G721 G722 U723 G724 A728 C736 C737 C738 C744 G745 A746 A747 U748 A749 C750 U751 G752 A755 C756 U757

C764 G765 A777 G778 C779 A780 A781 A782 A787 U793 A794 G799 G800 U801 A802 G812 A815 A816 C817 G818 A819 U820 G821 A825 C826 U827 A831 G832 G836 U837 G838 C839 G847 C848 G849 U850 G851 A860 C868 G869 A872 G877 A878 C879 C880 G881

G885 G886 G887 G890 U891 A892 C893 G902 A903 A908 A909 C912 A913 A914 U921 G922 A923 G926 G927 G933 C934 A935 A938 G944 G951 U952 G953 U957 A958 A959 U960 U961 C962 G963 A964 U965 G966 C967 A968 A969 C970 G971 C972 G973 A974 A975 G976 A977

For Manuscript Review

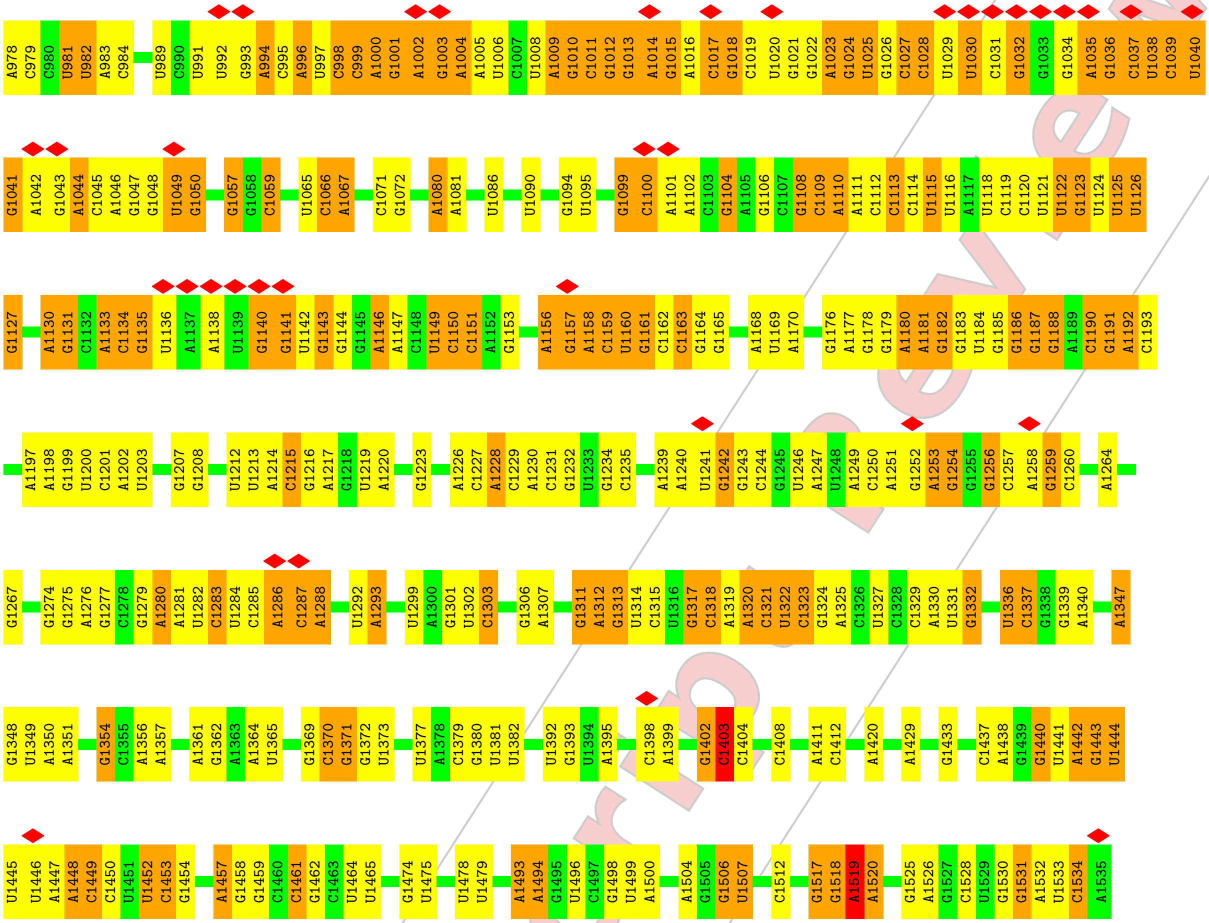

• Molecule 8: Small ribosomal subunit protein uS2

Chain B:

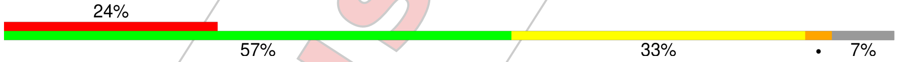

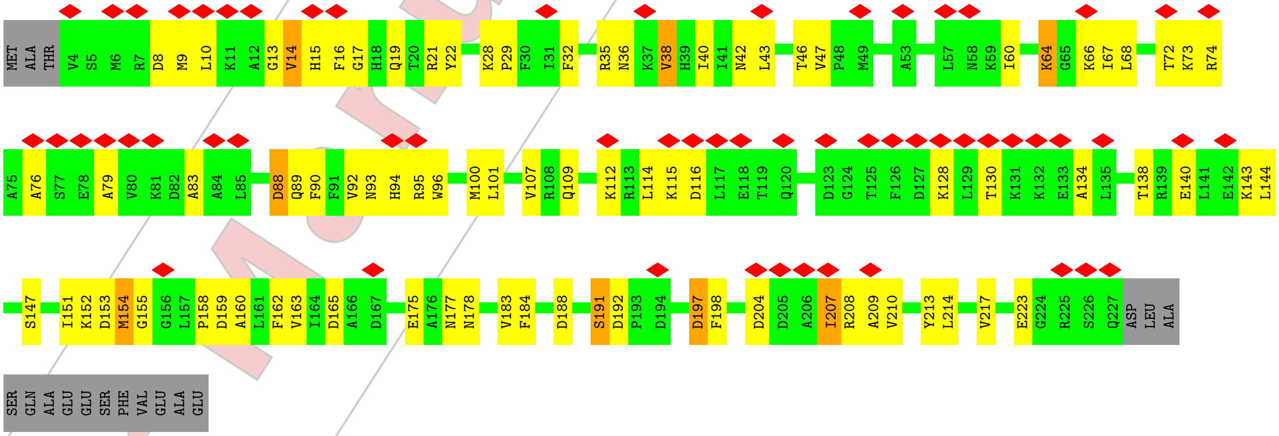

• Molecule 9: 30S ribosomal protein S3

Chain C:

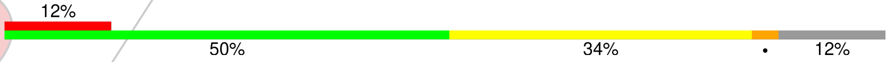

For Manuscript Review

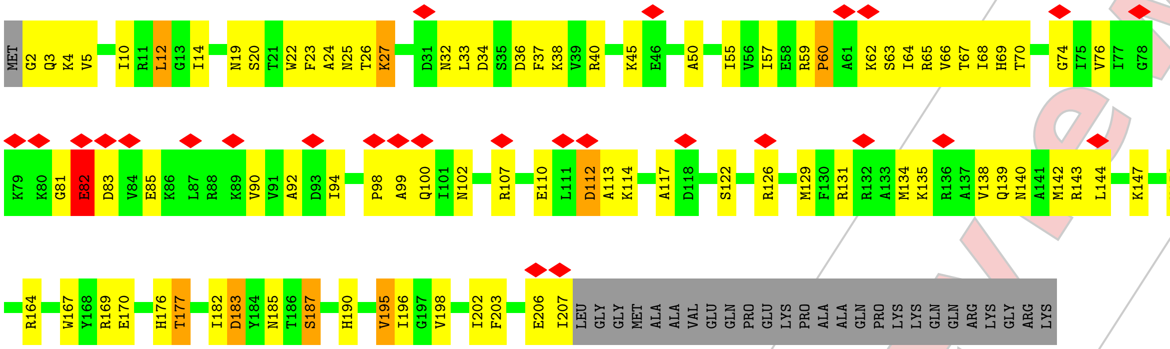

- Molecule 10: Small ribosomal subunit protein uS4

Chain D:

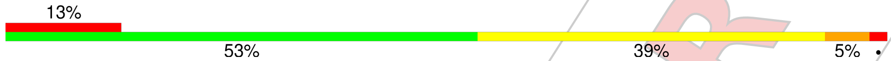

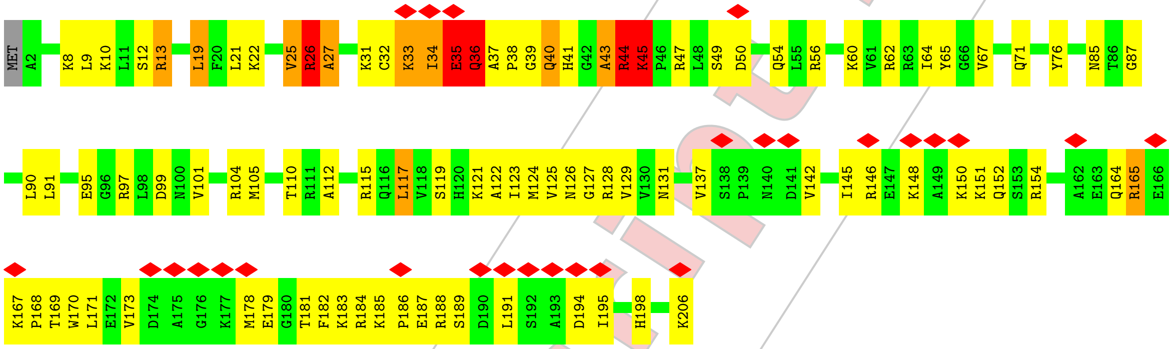

- Molecule 11: Small ribosomal subunit protein uS5

Chain E:

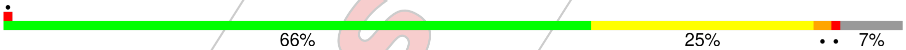

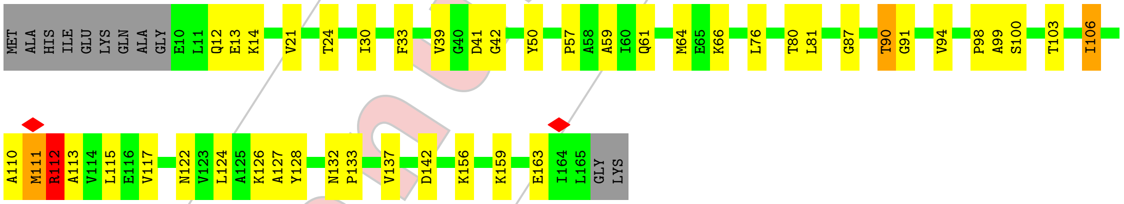

- Molecule 12: Small ribosomal subunit protein bS6

Chain F:

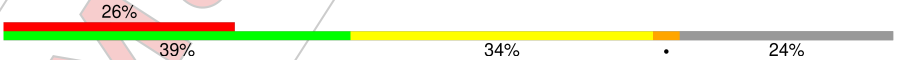

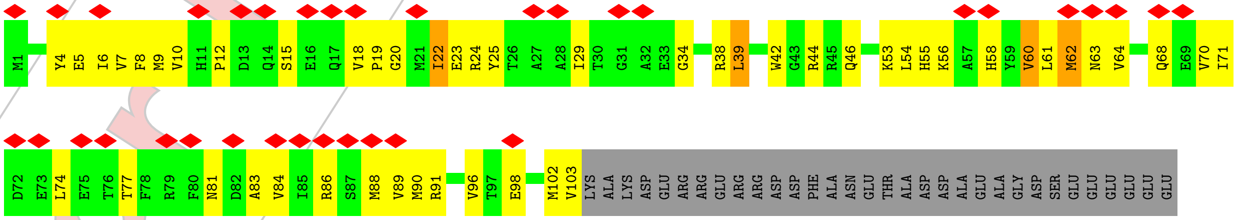

- Molecule 13: Small ribosomal subunit protein uS7

For Manuscript Review

Chain G: 30% | 50% | 32% | • | 15%

MET P2 R3 R4 I7 G8 Q9 R10 K11 I12 L13 P14 D15 P16 K17 F18 G19 K25 I29 L30 D33 G34 K35 T38 A39 E40 S41 I42 S45 A46 L47 E48 T49 L50 S54 G55 K56 L59 E60 A61 F62 E63 V64 A65 L66 E67 N68 V69 R70 P71 T72 V73

E74 V75 K76 S77 R78 R79 V80 G81 G82 S83 T84 Y85 Q86 V87 P88 V89 E90 V91 R92 P93 V94 R95 R96 M101 V105 R111 G112 D113 M116 A121 N122 E123 L124 S125 D126 A127 N130 K131 G132 T133 A134 V135 K136 K137 R138 E139 D140 V141 H142 R143 M144 A145 E146 A147

N148 K149 A150 H153 Y154 ARG TRP LEU SER LEU ARG SER PHE SER HIS GLN ALA GLY ALA SER SER LYS GLN PRO ALA LEU GLY TYR LEU ASN

• Molecule 14: Small ribosomal subunit protein uS8

Chain H: • | 71% | 27% | • •

MET S2 I7 A8 D9 M10 L11 N16 G17 Q18 K22 T26 S29 L32 I36 L40 K41 E42 E43 E47 D54 T55 K56 L59 E60 K64 Y65 K69 A70 S74 I75 V78 S79 D90 E91 L92 P93 I101 D113 G122

I126 C127 A130

• Molecule 15: Small ribosomal subunit protein uS9

Chain I: 8% | 56% | 38% | • •

MET ALA GLU N4 Q5 T9 G10 R11 R12 K13 S14 A17 R18 K22 P23 G24 N25 G26 K27 I28 V29 I30 S34 L35 E42 T43 A44 R45 M46 V47 V48 R49 Q50 P51 L52 E53 L54 V55 D56 E59 K60 L61 D62 L63 V67 I72 S73 G74 Q75 I79

R85 A86 L87 M88 E89 Y90 D91 E92 S93 L94 R95 L98 R99 F103 D107 A108 R109 Q110 V111 E112 R113 K114 K115 L118 R119 K120 A121 R122 R123 R124 F127 S128 K129 R130

• Molecule 16: Small ribosomal subunit protein uS10

Chain J: 36% | 48% | 41% | 6% | • | 5%

MET GLN ASN GLN R5 I6 R7 I8 R9 D14 L17 I18 D19 Q20 A21 T22 A23 E24 I25 V26 E27 T28 A29 K30 R31 T32 G33 A34 Q35 V36 R37 G38 P39 I40 P41 L42 E47 V51 L52 I53 S54 V57 N58 K59 D63 Q64 H70 L71 R72 L73 V74 D75 I76

V77 E78 P79 T80 E81 K82 T83 V84 D85 A86 L87 M88 R89 L90 D91 L92 A93 A94 V98 Q99 I100 S101 L102 GLY

• Molecule 17: Small ribosomal subunit protein uS11

Chain K: • | 59% | 32% | 9%

For Manuscript Review

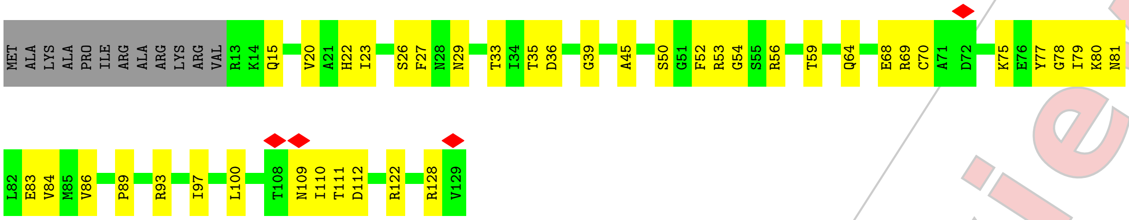

• Molecule 18: 30S ribosomal protein S12

Chain L:

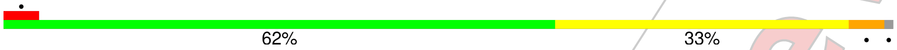

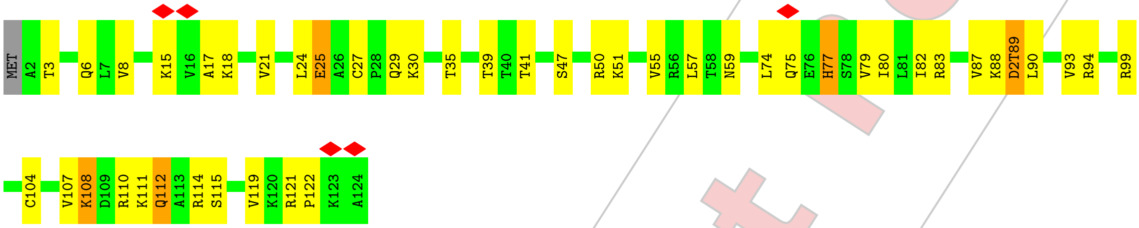

• Molecule 19: Small ribosomal subunit protein uS13

Chain M:

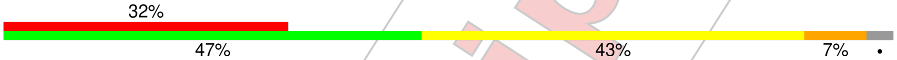

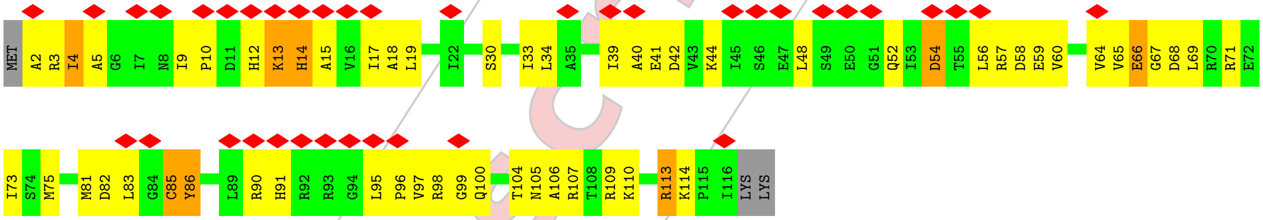

• Molecule 20: Small ribosomal subunit protein uS14

Chain N:

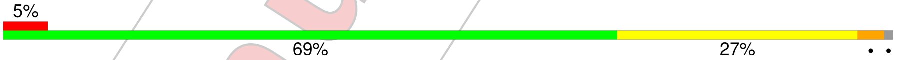

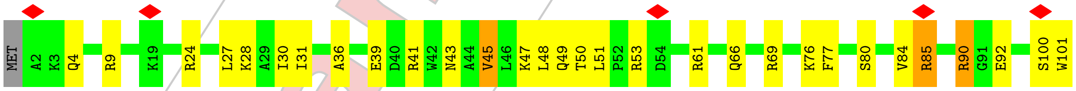

• Molecule 21: Small ribosomal subunit protein uS15

Chain O:

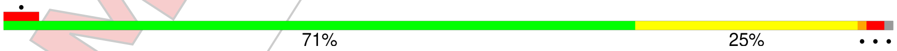

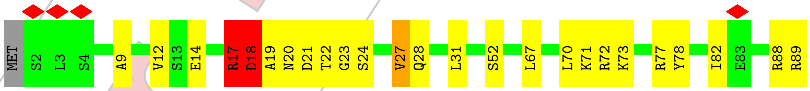

• Molecule 22: Small ribosomal subunit protein bS16

Chain P:

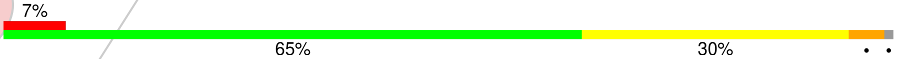

For Manuscript Review

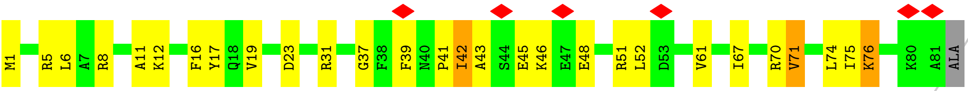

• Molecule 23: Small ribosomal subunit protein uS17

Chain Q:
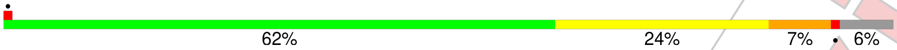

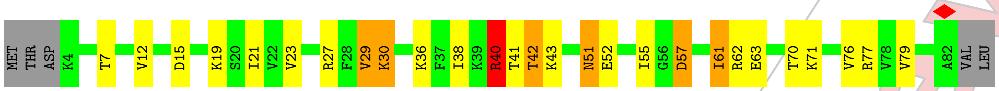

• Molecule 24: Small ribosomal subunit protein bS18

Chain R:
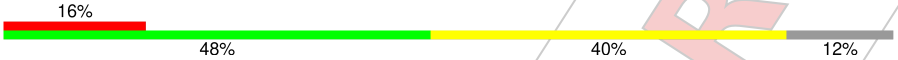

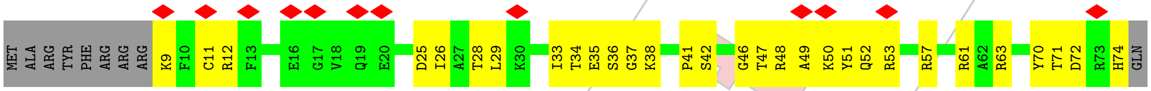

• Molecule 25: Small ribosomal subunit protein uS19

Chain S:
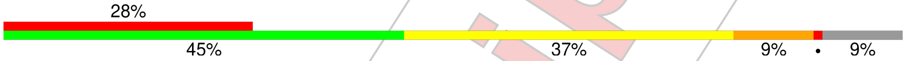

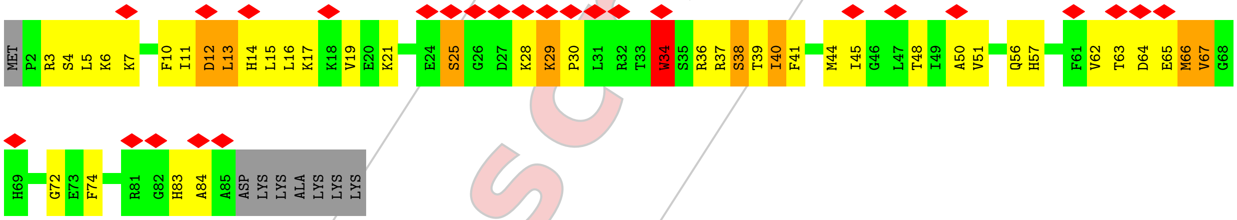

• Molecule 26: Small ribosomal subunit protein bS20

Chain T:
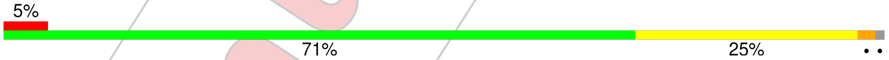

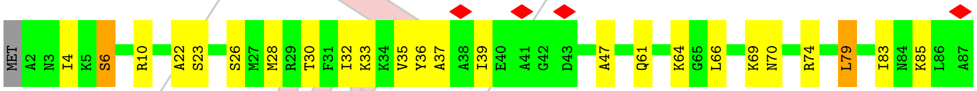

• Molecule 27: Small ribosomal subunit protein bS21

Chain U:
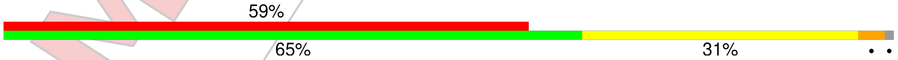

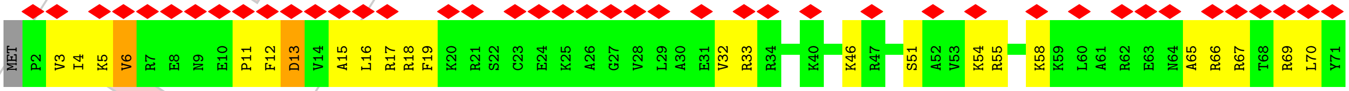

• Molecule 28: 23S ribosomal RNA

Chain a:
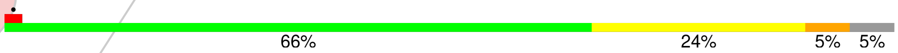

For Manuscript Review

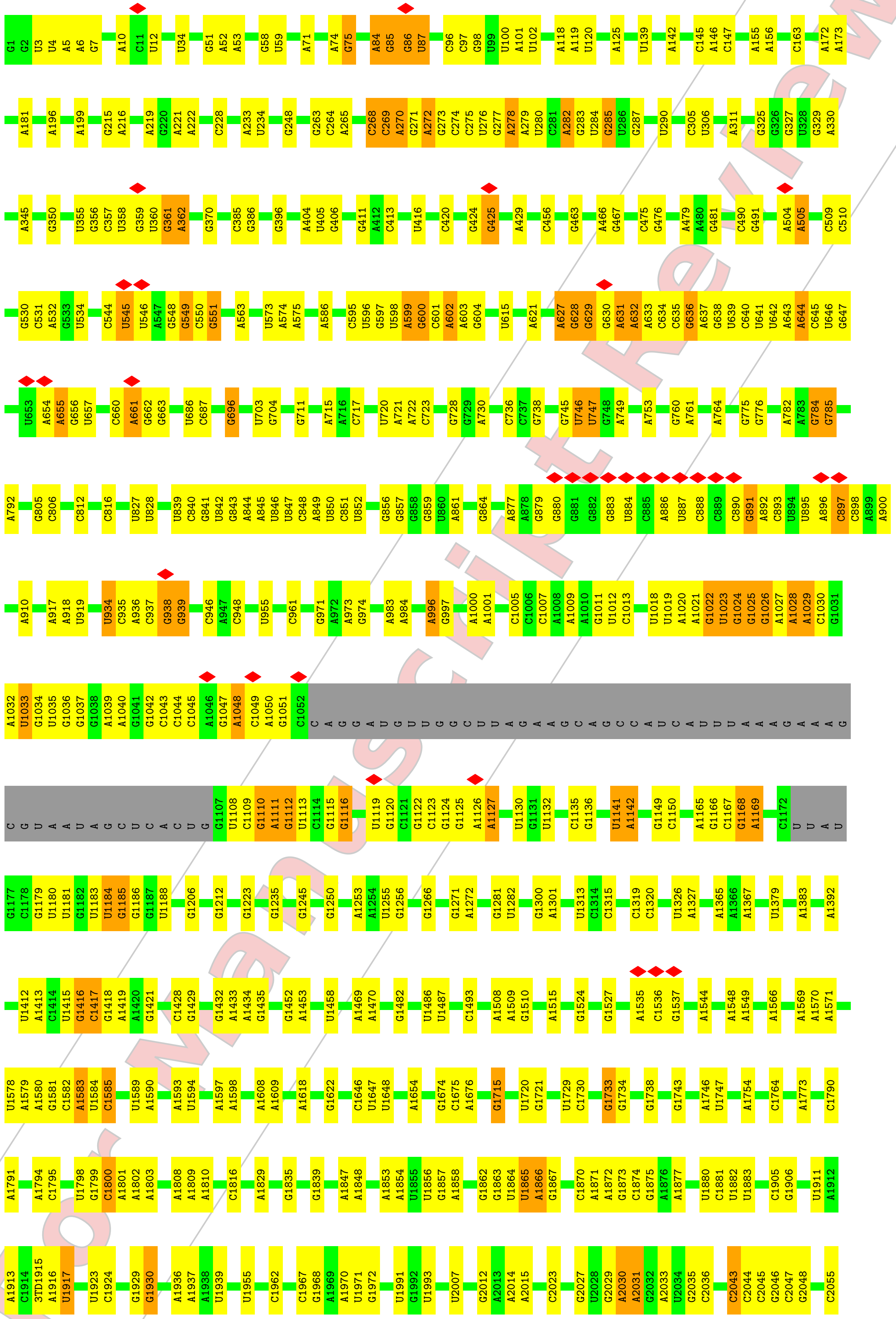

For Manuscript Review

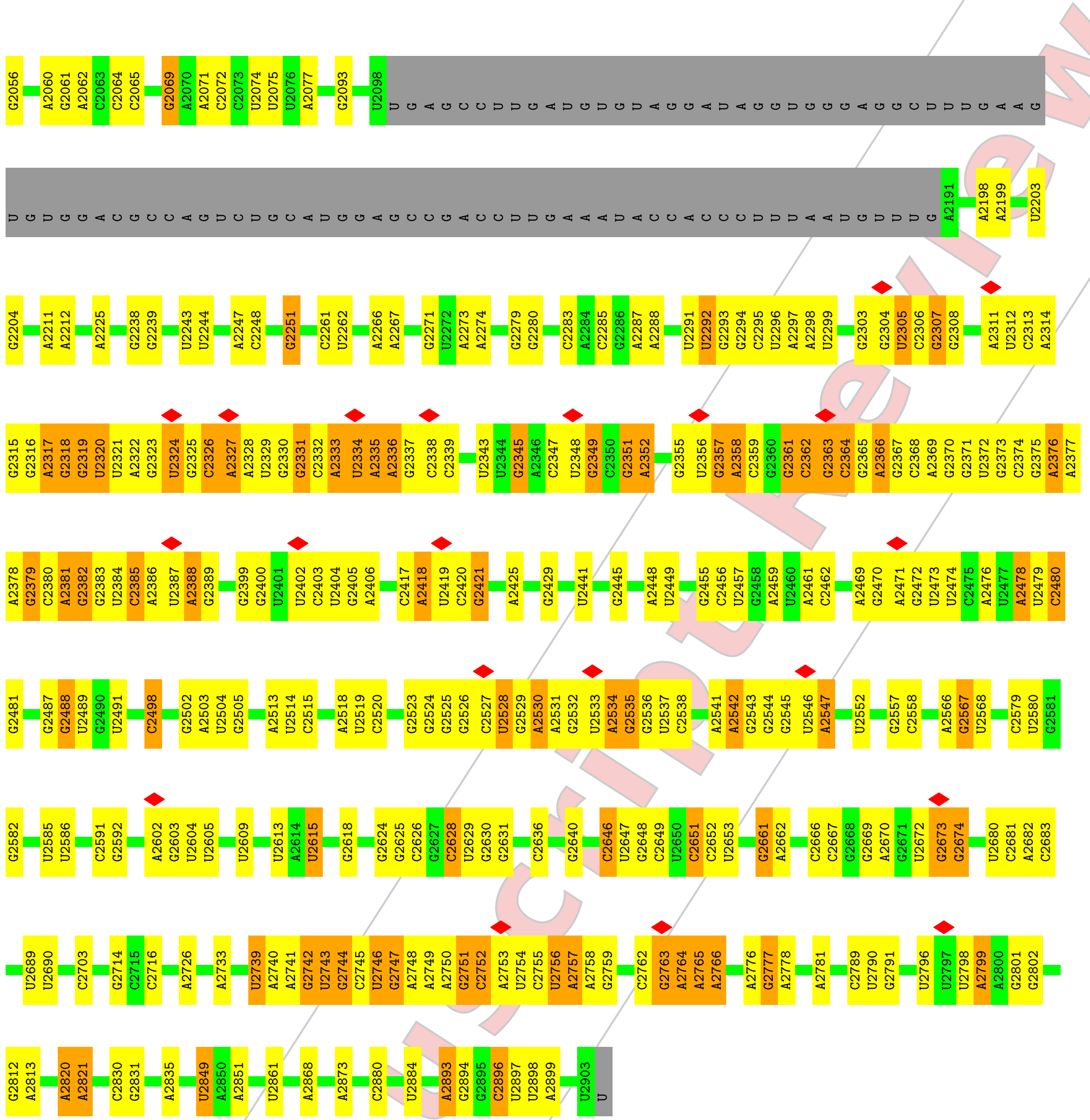

- Molecule 29: 5S ribosomal RNA

Chain b:

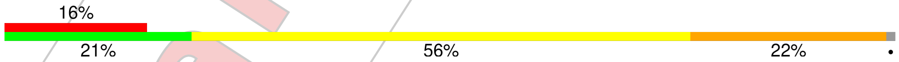

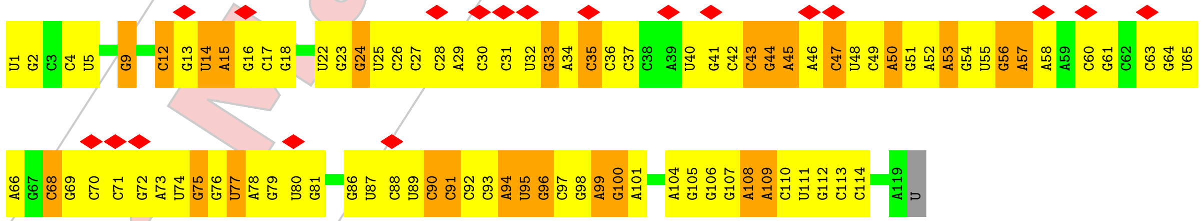

- Molecule 30: 50S ribosomal protein L2

Chain c:

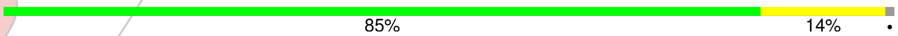

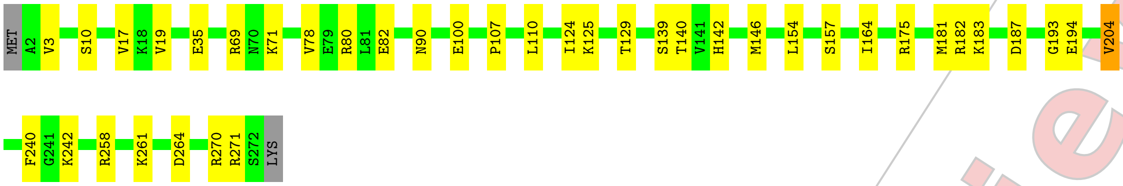

- Molecule 31: 50S ribosomal protein L3

Chain d:

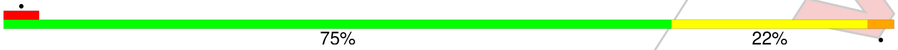

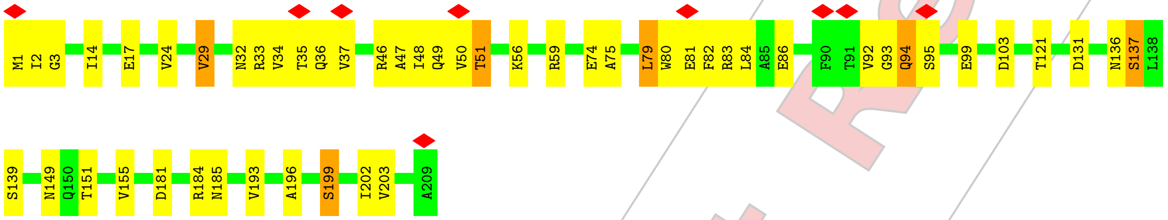

- Molecule 32: Large ribosomal subunit protein uL4

Chain e:

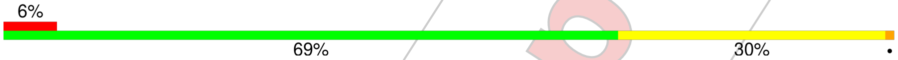

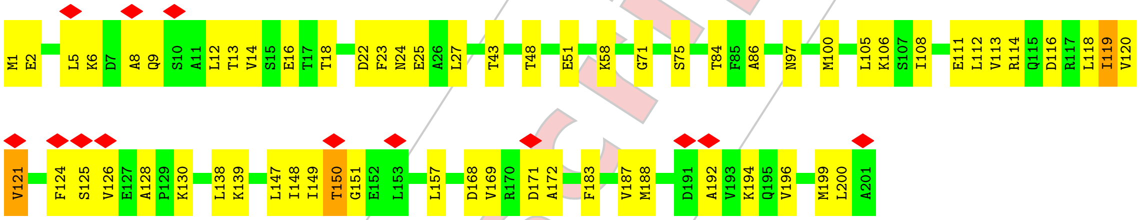

- Molecule 33: Large ribosomal subunit protein uL5

Chain f:

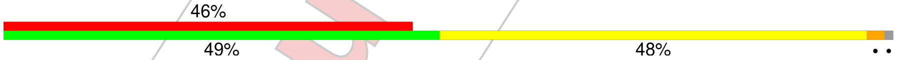

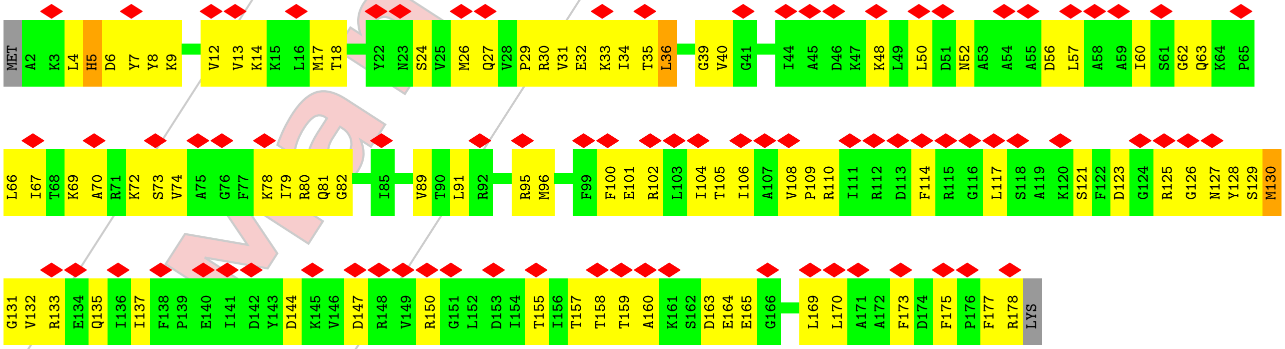

- Molecule 34: Large ribosomal subunit protein uL6

Chain g:

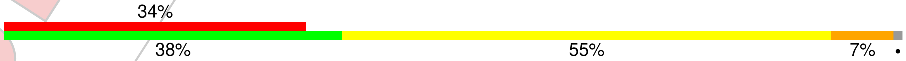

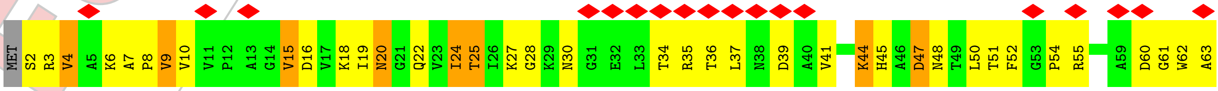

For Manuscript Review

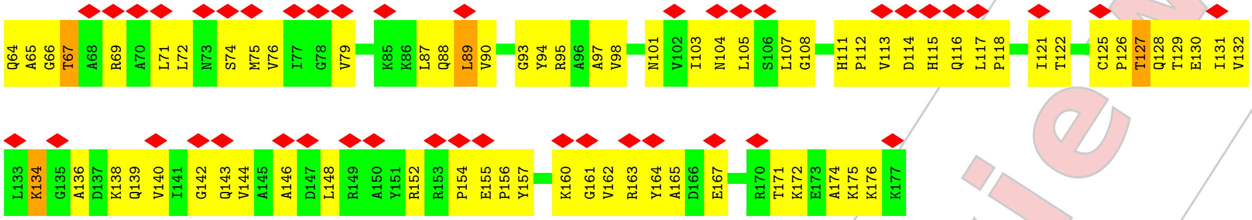

• Molecule 35: Large ribosomal subunit protein bL9

Chain h:

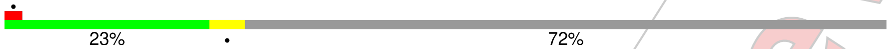

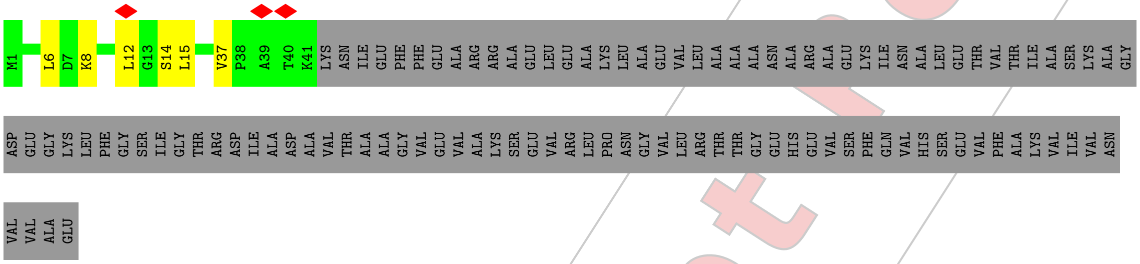

• Molecule 36: Large ribosomal subunit protein uL13

Chain i:

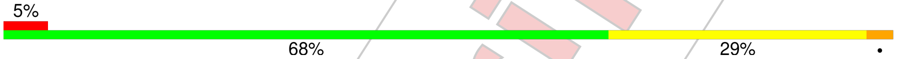

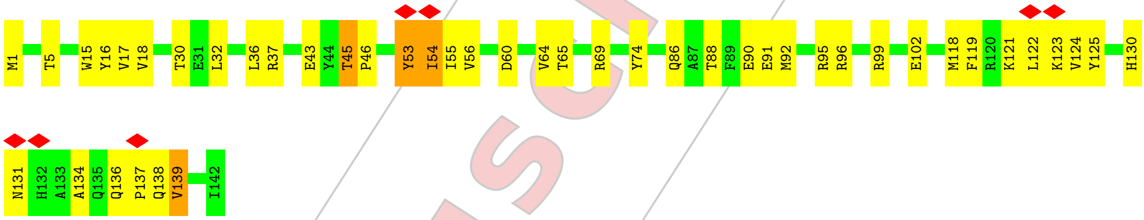

• Molecule 37: Large ribosomal subunit protein uL14

Chain j:

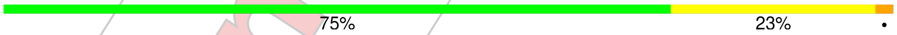

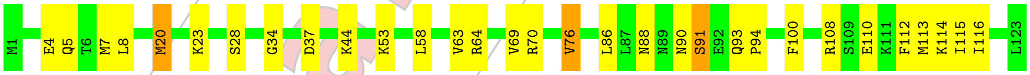

• Molecule 38: Large ribosomal subunit protein uL15

Chain k:

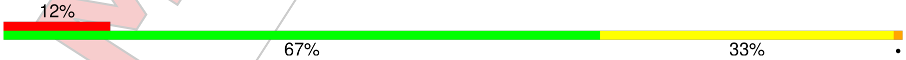

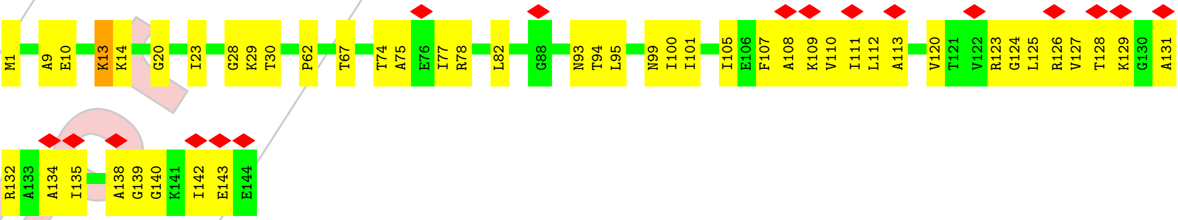

• Molecule 39: Large ribosomal subunit protein uL16

For Manuscript Review

Chain l:

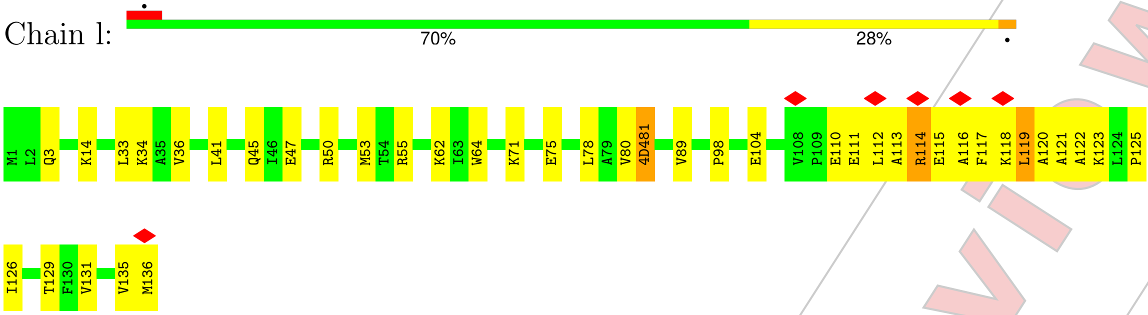

• Molecule 40: Large ribosomal subunit protein bL17

Chain m:

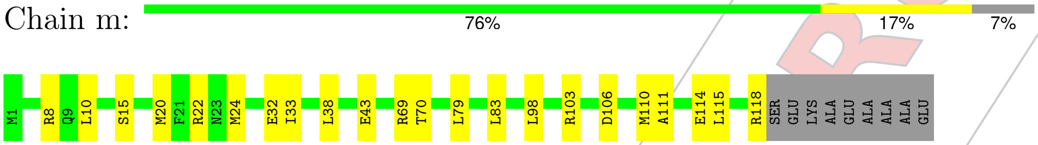

• Molecule 41: Large ribosomal subunit protein uL18

Chain n:

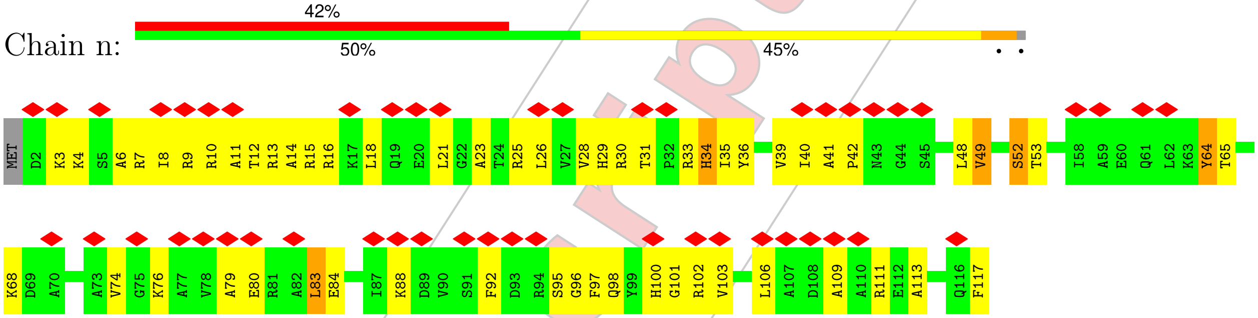

• Molecule 42: Large ribosomal subunit protein bL19

Chain o:

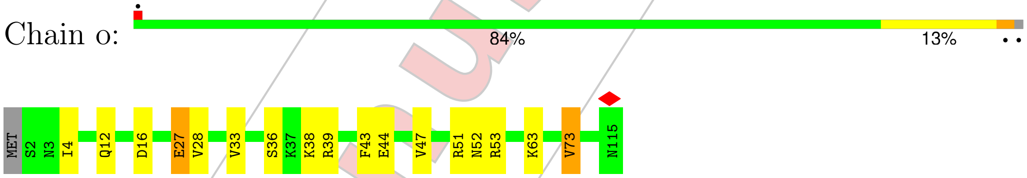

• Molecule 43: 50S ribosomal protein L20

Chain p:

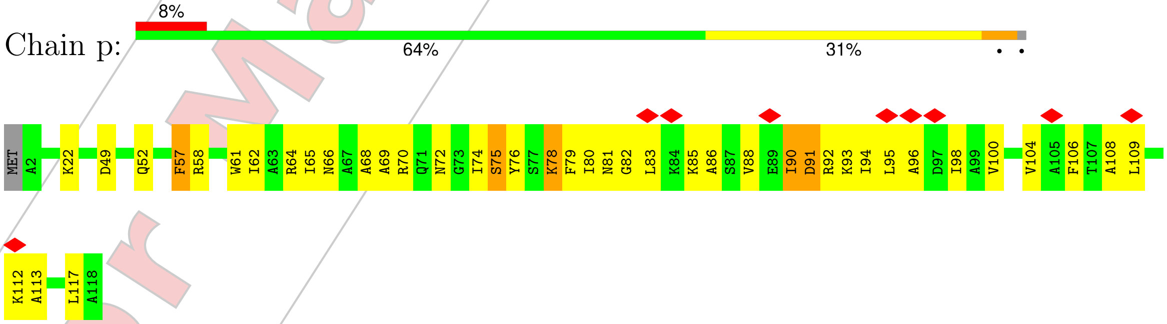

• Molecule 44: Large ribosomal subunit protein bL21

Chain q:

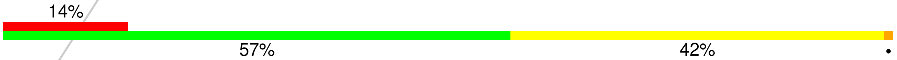

For Manuscript Review

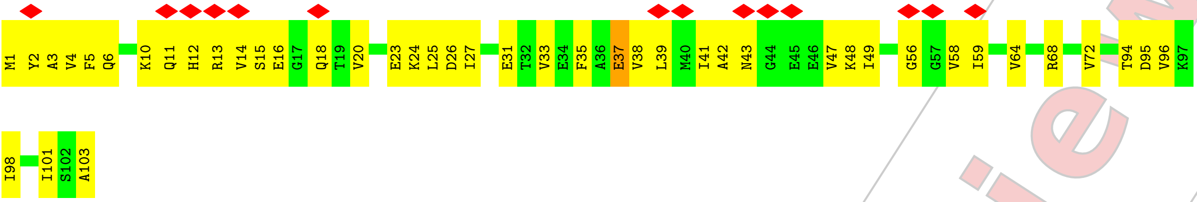

• Molecule 45: Large ribosomal subunit protein uL22

Chain r:

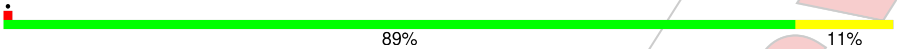

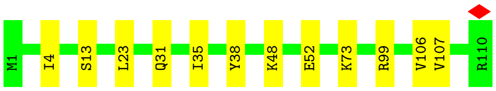

• Molecule 46: 50S ribosomal protein L23

Chain s:

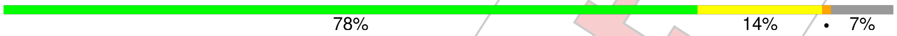

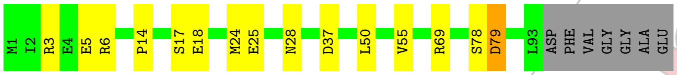

• Molecule 47: 50S ribosomal protein L24

Chain t:

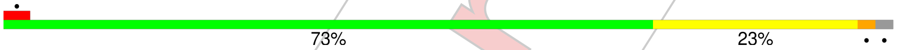

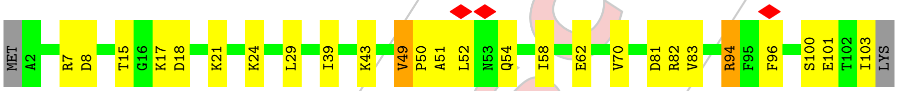

• Molecule 48: Large ribosomal subunit protein bL25

Chain u:

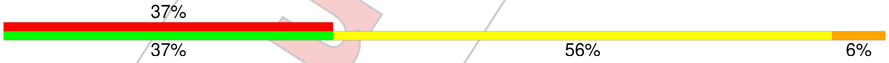

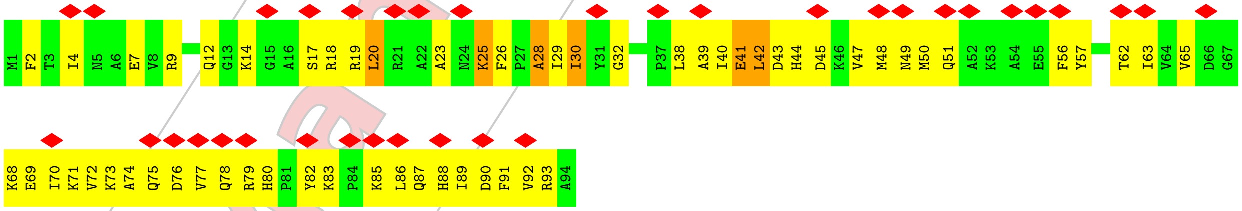

• Molecule 49: 50S ribosomal protein L27

Chain v:

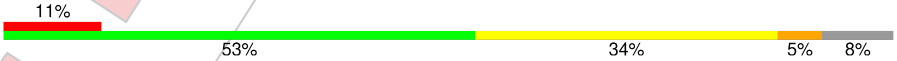

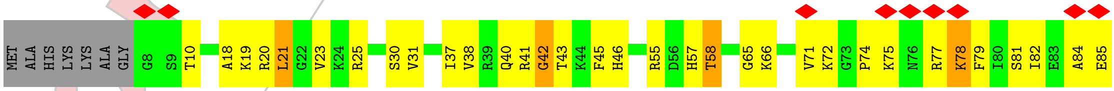

• Molecule 50: 50S ribosomal protein L28

Chain w:

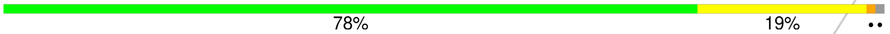

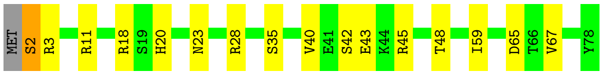

• Molecule 51: Large ribosomal subunit protein uL29

Chain x:

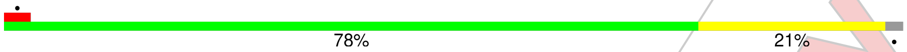

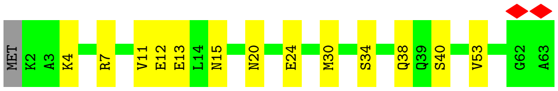

• Molecule 52: 50S ribosomal protein L30

Chain y:

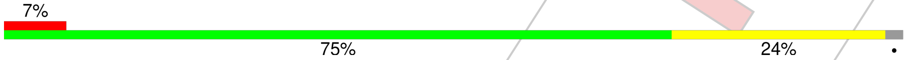

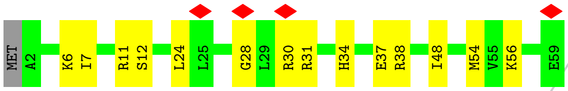

• Molecule 53: 50S ribosomal protein L32

Chain z:

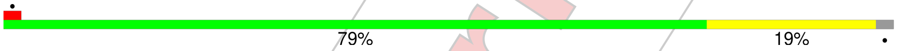

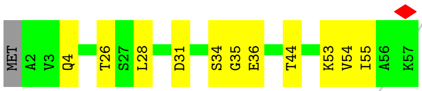

For Manuscript Review

4 Experimental information (i)

Property	Value	Source
EM reconstruction method	SINGLE PARTICLE	Depositor
Imposed symmetry	POINT, Not provided	
Number of particles used	450287	Depositor
Resolution determination method	FSC 0.143 CUT-OFF	Depositor
CTF correction method	PHASE FLIPPING AND AMPLITUDE CORRECTION	Depositor
Microscope	TFS GLACIOS	Depositor
Voltage (kV)	200	Depositor
Electron dose $(e^-/\text{Å}^2)$	60	Depositor
Minimum defocus (nm)	500	Depositor
Maximum defocus (nm)	2500	Depositor
Magnification	Not provided	
Image detector	FEI FALCON IV (4k x 4k)	Depositor
Maximum map value	43.573	Depositor
Minimum map value	-23.645	Depositor
Average map value	0.000	Depositor
Map value standard deviation	1.000	Depositor
Recommended contour level	2.8	Depositor
Map size (Å)	579.36, 579.36, 579.36	wwPDB
Map dimensions	480, 480, 480	wwPDB
Map angles (°)	90.0, 90.0, 90.0	wwPDB
Pixel spacing (Å)	1.207, 1.207, 1.207	Depositor

For Manuscript Review

5 Model quality (i)

5.1 Standard geometry (i)

Bond lengths and bond angles in the following residue types are not validated in this section: OMG, 4OC, UR3, 5MC, MS6, OMC, MG, D2T, 2MA, MA6, 4D4, 2MG, G7M, MEQ, H2U, 6MZ, OMU, SPM, 3TD, 1MG, PSU, ZN, SPD, BH2, 5MU

The Z score for a bond length (or angle) is the number of standard deviations the observed value is removed from the expected value. A bond length (or angle) with $|Z| > 5$ is considered an outlier worth inspection. RMSZ is the root-mean-square of all Z scores of the bond lengths (or angles).

Mol	Chain	Bond lengths		Bond angles	
		RMSZ	#|Z| >5	RMSZ	#|Z| >5
1	0	0.25	0/424	0.58	1/565 (0.2%)
2	1	0.24	0/380	0.38	0/498
3	2	0.27	0/513	0.45	0/676
4	3	0.33	0/303	0.76	0/397
5	4	0.23	0/488	0.49	0/649
6	5	0.10	0/46	0.09	0/69
7	A	0.25	0/36183	0.45	10/56432 (0.0%)
8	B	0.26	0/1784	0.64	2/2403 (0.1%)
9	C	0.24	0/1651	0.63	1/2225 (0.0%)
10	D	0.41	0/1664	0.80	2/2226 (0.1%)
11	E	0.25	0/1165	0.65	1/1568 (0.1%)
12	F	0.25	0/858	0.60	0/1160
13	G	0.55	2/1219 (0.2%)	0.92	4/1635 (0.2%)
14	H	0.22	0/988	0.59	1/1326 (0.1%)
15	I	0.22	0/1034	0.61	0/1375
16	J	0.55	1/796 (0.1%)	0.99	3/1077 (0.3%)
17	K	0.20	0/884	0.54	0/1191
18	L	0.21	0/960	0.52	1/1286 (0.1%)
19	M	0.24	0/900	0.78	2/1204 (0.2%)
20	N	0.21	0/817	0.59	0/1088
21	O	0.37	0/722	0.55	1/964 (0.1%)
22	P	0.24	0/653	0.65	0/877
23	Q	0.30	0/650	0.71	0/871
24	R	0.19	0/553	0.55	0/742
25	S	0.26	0/685	0.75	1/922 (0.1%)
26	T	0.20	0/675	0.58	1/893 (0.1%)
27	U	0.24	0/597	0.55	0/792
28	a	0.26	0/65651	0.36	0/102413
29	b	0.26	0/2850	0.43	0/4444
30	c	0.22	0/2121	0.36	0/2852
31	d	0.26	0/1576	0.47	0/2119

For Manuscript Review

Mol	Chain	Bond lengths		Bond angles	
		RMSZ	#|Z| >5	RMSZ	#|Z| >5
32	e	0.24	0/1571	0.48	0/2113
33	f	0.21	0/1434	0.41	0/1926
34	g	0.22	0/1343	0.53	0/1816
35	h	0.20	0/306	0.54	0/413
36	i	0.29	0/1152	0.54	0/1551
37	j	0.22	0/955	0.37	0/1279
38	k	0.23	0/1062	0.46	0/1413
39	l	0.26	0/1073	0.54	0/1433
40	m	0.23	0/958	0.39	0/1281
41	n	0.53	1/902 (0.1%)	0.47	0/1209
42	o	0.21	0/929	0.35	0/1242
43	p	0.36	0/960	0.66	0/1278
44	q	0.27	0/829	0.56	0/1107
45	r	0.20	0/864	0.29	0/1156
46	s	0.21	0/744	0.42	0/994
47	t	0.21	0/787	0.49	0/1051
48	u	0.33	1/766 (0.1%)	0.63	0/1025
49	v	0.29	0/593	0.67	1/785 (0.1%)
50	w	0.20	0/635	0.35	0/848
51	x	0.22	0/502	0.52	0/667
52	y	0.25	0/453	0.54	0/605
53	z	0.21	0/450	0.37	0/599
All	All	0.27	5/149058 (0.0%)	0.45	32/222730 (0.0%)

Chiral center outliers are detected by calculating the chiral volume of a chiral center and verifying if the center is modelled as a planar moiety or with the opposite hand.A planarity outlier is detected by checking planarity of atoms in a peptide group, atoms in a mainchain group or atoms of a sidechain that are expected to be planar.

Mol	Chain	#Chirality outliers	#Planarity outliers
10	D	0	3
11	E	0	1
16	J	0	1
17	K	1	0
20	N	0	1
21	O	0	1
23	Q	0	1
34	g	0	1
39	l	0	3
All	All	1	12

All (5) bond length outliers are listed below:

For Manuscript Review

Mol	Chain	Res	Type	Atoms	Z	Observed(Å)	Ideal(Å)
13	G	14	PRO	CG-CD	-15.01	0.99	1.50
41	n	34	HIS	C-N	-13.82	1.25	1.33
16	J	79	PRO	CG-CD	-12.36	1.08	1.50
13	G	14	PRO	N-CD	6.37	1.56	1.47
48	u	28	ALA	C-N	-5.12	1.30	1.33

All (32) bond angle outliers are listed below:

Mol	Chain	Res	Type	Atoms	Z	Observed(^{o})	Ideal(^{o})
13	G	14	PRO	N-CD-CG	-18.57	75.34	103.20
16	J	79	PRO	N-CD-CG	-16.56	78.36	103.20
16	J	79	PRO	CA-CB-CG	-12.96	79.88	104.50
13	G	14	PRO	CA-CB-CG	-11.90	81.90	104.50
13	G	14	PRO	CA-N-CD	-9.30	98.98	112.00
13	G	14	PRO	CB-CG-CD	8.77	134.16	106.10
7	A	413	A	P-O3'-C3'	-8.73	107.11	120.20
7	A	422	C	P-O3'-C3'	-8.48	107.48	120.20
7	A	425	G	P-O3'-C3'	-8.29	107.77	120.20
7	A	415	A	P-O3'-C3'	-8.05	108.13	120.20
11	E	106	ILE	N-CA-C	-7.83	104.94	111.91
7	A	1004	A	C2'-C3'-O3'	7.23	120.34	109.50
10	D	39	GLY	CA-C-O	-6.84	117.52	122.45
16	J	19	ASP	N-CA-C	-6.80	104.13	113.18
7	A	420	U	P-O3'-C3'	-6.77	110.04	120.20
26	T	66	LEU	N-CA-C	-6.54	106.51	114.75
1	0	11	LEU	CA-CB-CG	6.41	138.72	116.30
9	C	82	GLU	CA-CB-CG	6.33	126.75	114.10
7	A	424	G	P-O3'-C3'	-6.15	110.97	120.20
18	L	25	GLU	CA-CB-CG	5.70	125.50	114.10
25	S	34	TRP	N-CA-C	-5.62	107.67	114.75
19	M	86	TYR	N-CA-C	-5.55	105.49	114.09
14	H	93	PRO	CA-N-CD	-5.53	104.26	112.00
7	A	412	C	P-O3'-C3'	-5.51	111.93	120.20
21	O	22	THR	N-CA-C	-5.35	106.92	113.50
10	D	19	LEU	N-CA-C	-5.32	106.57	113.16
8	B	207	ILE	N-CA-C	-5.27	108.70	113.71
8	B	107	VAL	N-CA-C	-5.26	108.37	113.53
49	v	42	GLY	N-CA-C	-5.26	106.11	112.48
19	M	66	GLU	CB-CA-C	-5.23	110.56	116.63
7	A	423	G	C2'-C3'-O3'	5.22	117.33	109.50
7	A	421	U	P-O3'-C3'	-5.13	112.50	120.20

All (1) chirality outliers are listed below:

For Manuscript Review

Mol	Chain	Res	Type	Atom
17	K	119	BH2	CB

All (12) planarity outliers are listed below:

Mol	Chain	Res	Type	Group
10	D	13	ARG	Sidechain
10	D	26	ARG	Sidechain
10	D	44	ARG	Sidechain
11	E	112	ARG	Sidechain
16	J	78	GLU	Peptide
20	N	85	ARG	Sidechain
21	O	17	ARG	Sidechain
23	Q	40	ARG	Sidechain
34	g	47	ASP	Peptide
39	l	119	LEU	Peptide
39	l	80	VAL	Mainchain
39	l	81	4D4	Mainchain

5.2 Too-close contacts (i)

In the following table, the Non-H and H(model) columns list the number of non-hydrogen atoms and hydrogen atoms in the chain respectively. The H(added) column lists the number of hydrogen atoms added and optimized by MolProbity. The Clashes column lists the number of clashes within the asymmetric unit, whereas Symm-Clashes lists symmetry-related clashes.

Mol	Chain	Non-H	H(model)	H(added)	Clashes	Symm-Clashes
1	0	417	0	451	21	0
2	1	377	0	418	5	0
3	2	504	0	572	18	0
4	3	302	0	340	31	0
5	4	480	0	478	22	0
6	5	42	0	23	0	0
7	A	32564	0	16407	523	0
8	B	1753	0	1780	58	0
9	C	1624	0	1696	63	0
10	D	1642	0	1704	73	0
11	E	1152	0	1196	29	0
12	F	839	0	833	42	0
13	G	1203	0	1254	49	0
14	H	978	0	1031	24	0
15	I	1022	0	1070	46	0
16	J	786	0	828	36	0

Continued on next page...

For Manuscript Review

Continued from previous page...

Mol	Chain	Non-H	H(model)	H(added)	Clashes	Symm-Clashes
17	K	876	0	882	35	0
18	L	957	0	1016	27	0
19	M	891	0	952	60	0
20	N	805	0	844	28	0
21	O	714	0	734	12	0
22	P	643	0	661	23	0
23	Q	641	0	682	16	0
24	R	544	0	565	28	0
25	S	668	0	693	43	0
26	T	669	0	715	17	0
27	U	589	0	629	27	0
28	a	59130	0	29768	760	0
29	b	2549	0	1291	121	0
30	c	2082	0	2154	19	0
31	d	1566	0	1617	46	0
32	e	1552	0	1619	56	0
33	f	1410	0	1444	79	0
34	g	1323	0	1371	106	0
35	h	303	0	327	4	0
36	i	1129	0	1162	66	0
37	j	946	0	1023	20	0
38	k	1053	0	1129	49	0
39	l	1075	0	1145	33	0
40	m	945	0	989	11	0
41	n	892	0	923	55	0
42	o	917	0	962	9	0
43	p	947	0	1019	67	0
44	q	816	0	839	45	0
45	r	857	0	922	6	0
46	s	738	0	807	8	0
47	t	779	0	831	22	0
48	u	753	0	780	77	0
49	v	586	0	596	48	0
50	w	625	0	652	5	0
51	x	501	0	531	7	0
52	y	449	0	488	11	0
53	z	444	0	458	7	0
54	3	1	0	0	0	0
54	4	1	0	0	0	0
55	A	55	0	0	0	0
55	Q	1	0	0	0	0
55	a	207	0	0	0	0

Continued on next page...

For Manuscript Review

Continued from previous page...

Mol	Chain	Non-H	H(model)	H(added)	Clashes	Symm-Clashes
55	b	5	0	0	0	0
55	c	1	0	0	0	0
55	m	1	0	0	0	0
55	n	1	0	0	0	0
55	z	1	0	0	0	0
56	a	140	0	266	12	0
57	a	14	0	26	1	0
All	All	138477	0	93593	2821	0

The all-atom clashscore is defined as the number of clashes found per 1000 atoms (including hydrogen atoms). The all-atom clashscore for this structure is 12.

All (2821) close contacts within the same asymmetric unit are listed below, sorted by their clash magnitude.

Atom-1	Atom-2	Interatomic distance (Å)	Clash overlap (Å)
29:b:78:A:H62	29:b:98:G:N2	1.20	1.38
7:A:998:C:C6	7:A:1044:A:C5’	2.07	1.37
28:a:1418:G:H21	28:a:1580:A:N6	1.23	1.37
28:a:1020:A:N6	28:a:1141:U:C2	2.00	1.29
7:A:73:C:N4	7:A:96:U:H3	1.30	1.29
28:a:2739:U:H3	28:a:2764:A:N6	1.28	1.28
29:b:77:U:H3	29:b:99:A:N6	1.31	1.28
29:b:78:A:N6	29:b:98:G:H21	1.30	1.28
28:a:1418:G:N2	28:a:1580:A:H62	1.31	1.25
7:A:998:C:C2’	7:A:999:C:O2	1.90	1.19
28:a:2352:A:N6	28:a:2365:G:H21	1.42	1.18
7:A:998:C:H2’	7:A:999:C:O2	1.41	1.17
28:a:2352:A:H62	28:a:2365:G:N2	1.39	1.17
7:A:998:C:C6	7:A:1044:A:H5’	1.81	1.13
28:a:2472:G:H21	28:a:2478:A:N6	1.46	1.12
3:2:33:LEU:HB2	3:2:36:LYS:HG3	1.41	1.02
7:A:998:C:C6	7:A:1044:A:H5”	1.89	1.02
7:A:998:C:O2’	7:A:999:C:O2	1.77	1.02
28:a:2472:G:N2	28:a:2478:A:H62	1.59	1.01
29:b:76:G:N1	29:b:100:G:O6	1.92	1.01
29:b:77:U:O4	29:b:99:A:N7	1.94	1.01
7:A:836:G:H1	7:A:850:U:H3	1.06	1.01
28:a:996:A:H4’	44:q:11:GLN:HG3	1.42	0.99
28:a:600:G:H1	28:a:657:U:H3	1.04	0.99
28:a:2739:U:O4	28:a:2764:A:N7	1.97	0.97
28:a:1039:A:H2	28:a:1116:G:H1	0.99	0.96

Continued on next page...

For Manuscript Review

Continued from previous page...

Atom-1	Atom-2	Interatomic distance (Å)	Clash overlap (Å)
36:i:53:TYR:HA	36:i:121:LYS:HB3	1.47	0.96
28:a:2345:G:O6	28:a:2371:G:N1	1.98	0.95
3:2:31:HIS:HD2	3:2:32:ILE:HG13	1.31	0.95
28:a:2648:G:O6	28:a:2672:U:O4	1.85	0.94
7:A:998:C:C5	7:A:1044:A:H5”	2.02	0.94
28:a:600:G:O6	28:a:657:U:O4	1.85	0.94
10:D:8:LYS:HE3	10:D:21:LEU:HB3	1.47	0.93
7:A:1151:C:H1’	16:J:41:PRO:HB3	1.47	0.93
29:b:77:U:N3	29:b:99:A:N6	1.97	0.93
7:A:1023:A:H1’	7:A:1024:G:H5’	1.49	0.91
28:a:2331:G:N2	28:a:2336:A:O2’	2.03	0.91
7:A:998:C:C5	7:A:1044:A:C5’	2.54	0.91
43:p:81:ASN:O	43:p:85:LYS:HB3	1.70	0.91
28:a:2356:U:H4’	49:v:20:ARG:HE	1.35	0.90
28:a:2519:U:O4	28:a:2542:A:N6	2.04	0.90
28:a:2351:G:H21	28:a:2366:A:H8	1.17	0.90
7:A:73:C:N4	7:A:96:U:N3	2.11	0.90
28:a:2523:G:H21	28:a:2764:A:H1’	1.37	0.90
32:e:121:VAL:HG21	32:e:187:VAL:HG23	1.53	0.89
4:3:2:LYS:O	4:3:36:ARG:N	2.04	0.89
28:a:2472:G:H21	28:a:2478:A:H62	0.92	0.89
28:a:1020:A:C6	28:a:1141:U:N3	2.41	0.89
28:a:284:U:H3	28:a:356:G:H1	0.90	0.89
28:a:1027:A:H2’	28:a:1028:A:H8	1.36	0.89
29:b:77:U:C2	29:b:99:A:N6	2.40	0.88
7:A:736:C:O2’	12:F:89:VAL:O	1.92	0.88
28:a:2305:U:H5”	33:f:131:GLY:HA3	1.55	0.88
7:A:998:C:H2’	7:A:999:C:C2	2.08	0.88
5:4:64:PHE:O	25:S:7:LYS:NZ	2.07	0.87
39:l:113:ALA:O	39:l:117:PHE:N	2.08	0.87
28:a:2262:U:O2’	28:a:2329:U:O2’	1.93	0.87
7:A:765:G:N1	7:A:812:G:N3	2.23	0.86
31:d:37:VAL:H	31:d:92:VAL:HG22	1.40	0.86
28:a:424:G:N1	28:a:425:G:N3	2.24	0.86
28:a:1027:A:H2’	28:a:1028:A:C8	2.12	0.85
28:a:1183:U:OP1	52:y:31:ARG:NH2	2.10	0.85
10:D:146:ARG:HE	10:D:178:MET:HG3	1.41	0.85
28:a:1418:G:N2	28:a:1580:A:N6	2.02	0.85
28:a:2757:A:N1	34:g:67:THR:OG1	2.09	0.84
7:A:1130:A:O2’	15:I:18:ARG:NH2	2.11	0.84
36:i:16:TYR:N	36:i:53:TYR:O	2.11	0.84

Continued on next page...

For Manuscript Review

Continued from previous page...

Atom-1	Atom-2	Interatomic distance (Å)	Clash overlap (Å)
32:e:97:ASN:HB2	32:e:100:MET:HG3	1.60	0.84
29:b:9:G:O6	29:b:111:U:O2	1.95	0.84
7:A:484:G:H4’	7:A:485:U:H5’	1.59	0.83
28:a:85:G:H2’	28:a:86:G:H4’	1.59	0.83
7:A:1125:U:H5’	16:J:37:ARG:HH11	1.42	0.83
10:D:178:MET:HG2	10:D:179:GLU:HG2	1.59	0.83
28:a:1583:A:O2’	28:a:1585:C:N4	2.12	0.83
28:a:2336:A:N6	49:v:57:HIS:HD2	1.77	0.83
7:A:1280:A:H8	7:A:1283:C:H42	1.24	0.82
4:3:4:ARG:N	4:3:36:ARG:O	2.12	0.82
7:A:778:G:H21	17:K:122:ARG:HD3	1.42	0.82
28:a:2261:C:H4’	28:a:2387:U:H2’	1.61	0.82
28:a:646:U:O4	28:a:2368:C:O2’	1.96	0.82
7:A:35:G:H1	7:A:549:C:H5	1.27	0.82
7:A:1001:G:O2’	7:A:1041:G:O6	1.97	0.82
29:b:78:A:N6	29:b:98:G:N2	2.05	0.82
18:L:121:ARG:HD2	18:L:122:PRO:HD2	1.62	0.82
7:A:609:A:O2’	7:A:610:G:O5’	1.98	0.81
48:u:78:GLN:HB2	48:u:88:HIS:HB3	1.60	0.81
11:E:13:GLU:HG2	11:E:64:MET:HE1	1.62	0.81
19:M:54:ASP:HA	19:M:57:ARG:HE	1.45	0.81
43:p:95:LEU:HA	43:p:98:ILE:HD12	1.63	0.81
49:v:72:LYS:HD2	49:v:79:PHE:HB2	1.61	0.81
28:a:1020:A:N6	28:a:1141:U:N3	2.28	0.81
43:p:68:ALA:O	43:p:72:ASN:ND2	2.14	0.81
28:a:643:A:H61	28:a:2370:G:H4’	1.45	0.80
29:b:40:U:H1’	29:b:45:A:H61	1.47	0.80
28:a:97:C:N4	28:a:98:G:O6	2.13	0.80
28:a:2519:U:N3	28:a:2541:A:N1	2.28	0.80
28:a:2525:G:H2’	28:a:2526:G:C8	2.15	0.80
39:l:47:GLU:OE1	39:l:50:ARG:NH1	2.15	0.80
7:A:1158:A:H61	7:A:1182:G:H5’	1.47	0.80
28:a:2796:U:H3	28:a:2799:A:H61	1.28	0.79
7:A:1252:G:H3’	7:A:1253:A:H5’	1.63	0.79
28:a:2262:U:HO2’	28:a:2329:U:HO2’	1.28	0.79
49:v:66:LYS:NZ	49:v:85:GLU:OE1	2.15	0.79
27:U:6:VAL:HG12	27:U:18:ARG:HH22	1.47	0.79
29:b:31:C:H1’	29:b:53:A:H61	1.46	0.79
7:A:998:C:H6	7:A:1044:A:C5’	1.94	0.79
39:l:114:ARG:O	39:l:118:LYS:N	2.16	0.79
12:F:38:ARG:HD3	12:F:98:GLU:H	1.46	0.79

Continued on next page...

Continued from previous page...

Atom-1	Atom-2	Interatomic distance (Å)	Clash overlap (Å)
28:a:2297:A:H62	28:a:2319:G:H21	1.28	0.79
34:g:126:PRO:HG3	34:g:132:VAL:HG23	1.64	0.79
38:k:82:LEU:HD21	38:k:120:VAL:HG21	1.64	0.79
28:a:1028:A:C2	28:a:2487:G:H4'	2.17	0.79
28:a:1036:G:O6	28:a:1119:U:O2	2.00	0.79
28:a:643:A:N6	28:a:2370:G:O2'	2.16	0.79
31:d:50:VAL:N	31:d:80:TRP:O	2.16	0.78
28:a:636:G:H5"	38:k:128:THR:HB	1.63	0.78
48:u:9:ARG:NH1	48:u:17:SER:OG	2.17	0.78
1:0:19:HIS:ND1	1:0:49:TYR:OH	2.16	0.78
7:A:683:G:N2	17:K:39:GLY:O	2.17	0.78
28:a:660:C:H2'	28:a:661:A:C8	2.19	0.78
39:l:119:LEU:HD22	39:l:123:LYS:HE2	1.63	0.78
7:A:61:G:N2	7:A:105:G:H22	1.82	0.78
47:t:96:PHE:HB2	47:t:101:GLU:HG3	1.64	0.78
4:3:1:MET:HB3	4:3:35:GLN:HA	1.66	0.77
28:a:1880:U:C4	28:a:1881:C:N4	2.52	0.77
28:a:277:G:N2	28:a:361:G:OP2	2.18	0.77
32:e:196:VAL:HA	32:e:199:MET:HE2	1.65	0.77
36:i:15:TRP:HA	36:i:53:TYR:H	1.48	0.77
48:u:32:GLY:O	48:u:93:ARG:NH1	2.16	0.77
9:C:65:ARG:HG3	9:C:100:GLN:HG2	1.65	0.77
28:a:877:A:O2'	28:a:900:A:N6	2.16	0.77
29:b:66:A:OP2	29:b:108:A:N6	2.17	0.77
41:n:18:LEU:HD22	41:n:25:ARG:HG3	1.67	0.77
29:b:76:G:OP1	48:u:17:SER:OG	2.03	0.77
18:L:30:LYS:HE3	18:L:59:ASN:HD22	1.48	0.77
19:M:30:SER:O	19:M:34:LEU:HB2	1.84	0.77
38:k:110:VAL:O	38:k:128:THR:OG1	2.03	0.76
39:l:116:ALA:HA	39:l:119:LEU:HB2	1.65	0.76
7:A:599:C:H2'	7:A:600:A:H8	1.49	0.76
40:m:103:ARG:HD3	40:m:110:MET:HE2	1.64	0.76
7:A:143:A:H2	7:A:220:G:H1	1.30	0.76
7:A:1321:C:O2'	7:A:1322:U:O5'	2.03	0.76
48:u:78:GLN:N	48:u:88:HIS:O	2.18	0.76
36:i:69:ARG:NH1	36:i:90:GLU:OE2	2.19	0.76
28:a:1020:A:N1	28:a:1141:U:C4	2.53	0.76
28:a:1035:U:O2	28:a:1120:G:O6	2.03	0.76
39:l:118:LYS:O	39:l:122:ALA:N	2.12	0.76
1:0:21:TYR:OH	1:0:39:PHE:O	2.04	0.76
28:a:2306:C:H42	33:f:39:GLY:HA3	1.51	0.76

Continued on next page...

For Manuscript Review

Continued from previous page...

Atom-1	Atom-2	Interatomic distance (Å)	Clash overlap (Å)
28:a:2345:G:O6	28:a:2371:G:C6	2.39	0.76
53:z:54:VAL:HG12	53:z:55:ILE:HG23	1.67	0.76
28:a:1168:G:N2	28:a:1181:U:O2	2.14	0.76
4:3:5:ALA:HA	4:3:37:GLN:HG2	1.68	0.76
4:3:19:ARG:NH1	28:a:2754:U:O2'	2.19	0.76
7:A:82:A:H3'	7:A:83:C:H4'	1.68	0.76
7:A:448:A:H2'	7:A:449:G:H4'	1.68	0.75
36:i:54:ILE:N	36:i:121:LYS:O	2.16	0.75
43:p:93:LYS:NZ	44:q:11:GLN:O	2.19	0.75
4:3:32:LYS:O	4:3:35:GLN:NE2	2.18	0.75
28:a:2630:G:H2'	28:a:2631:G:H8	1.49	0.75
7:A:66:G:O6	7:A:102:G:N2	2.19	0.75
10:D:142:VAL:HG22	10:D:181:THR:HG22	1.67	0.75
28:a:2336:A:N6	49:v:57:HIS:CD2	2.53	0.75
23:Q:57:ASP:N	23:Q:57:ASP:OD1	2.20	0.75
28:a:1235:G:OP1	56:a:6211:SPD:N1	2.20	0.75
28:a:2333:A:OP2	49:v:77:ARG:NH2	2.20	0.75
34:g:39:ASP:O	34:g:55:ARG:NH1	2.20	0.75
39:l:115:GLU:O	39:l:119:LEU:N	2.17	0.75
34:g:87:LEU:HD22	34:g:148:LEU:HB2	1.67	0.74
38:k:78:ARG:HB2	38:k:78:ARG:HH11	1.52	0.74
28:a:1023:U:H2'	28:a:1024:G:H5'	1.69	0.74
41:n:33:ARG:NH2	41:n:65:THR:OG1	2.19	0.74
43:p:61:TRP:CE2	43:p:93:LYS:HB2	2.23	0.74
7:A:981:U:H3'	7:A:982:U:H5"	1.70	0.74
28:a:2648:G:H1	28:a:2672:U:H3	0.82	0.74
7:A:67:C:H42	7:A:101:A:H61	1.33	0.74
28:a:1029:A:H61	28:a:1125:G:H1'	1.52	0.74
28:a:2750:A:H5"	34:g:3:ARG:HH12	1.51	0.74
7:A:1186:G:N2	20:N:101:TRP:O	2.20	0.74
32:e:6:LYS:HD3	32:e:119:ILE:HD12	1.69	0.74
50:w:43:GLU:OE1	50:w:45:ARG:NH1	2.20	0.74
7:A:129:A:H2	7:A:232:G:H22	1.36	0.73
39:l:36:VAL:HA	48:u:82:TYR:HB2	1.70	0.73
32:e:112:LEU:HG	32:e:118:LEU:HB2	1.70	0.73
36:i:36:LEU:O	36:i:121:LYS:NZ	2.22	0.73
28:a:285:G:O6	28:a:355:U:O2	2.07	0.73
28:a:631:A:O2'	28:a:632:A:OP1	2.06	0.73
28:a:2334:U:H1'	41:n:13:ARG:HD3	1.70	0.73
38:k:10:GLU:N	38:k:10:GLU:OE2	2.20	0.73
8:B:13:GLY:O	8:B:17:GLY:N	2.17	0.73

Continued on next page...

For Manuscript Review

Continued from previous page...

Atom-1	Atom-2	Interatomic distance (Å)	Clash overlap (Å)
18:L:114:ARG:HB3	18:L:119:VAL:HB	1.70	0.73
28:a:84:A:H3'	47:t:7:ARG:HH22	1.50	0.73
29:b:66:A:N6	29:b:108:A:OP2	2.21	0.73
7:A:61:G:H22	7:A:105:G:H1	1.36	0.73
28:a:2525:G:H2'	28:a:2526:G:H8	1.51	0.73
28:a:2741:A:H2'	28:a:2742:G:H8	1.53	0.73
36:i:55:ILE:HG23	36:i:124:VAL:HA	1.69	0.73
13:G:92:ARG:HE	13:G:94:VAL:HG12	1.53	0.73
39:l:64:TRP:HB2	39:l:104:GLU:HB2	1.70	0.73
3:2:31:HIS:CD2	3:2:32:ILE:HG13	2.20	0.73
10:D:33:LYS:O	10:D:35:GLU:N	2.21	0.73
28:a:2741:A:H2'	28:a:2742:G:C8	2.24	0.73
41:n:31:THR:O	41:n:102:ARG:NH1	2.18	0.73
8:B:114:LEU:HD12	8:B:144:LEU:HB3	1.70	0.72
38:k:134:ALA:O	38:k:138:ALA:N	2.20	0.72
36:i:17:VAL:HA	36:i:55:ILE:O	1.89	0.72
28:a:1417:C:H2'	28:a:1418:G:C8	2.24	0.72
29:b:27:C:H2'	29:b:28:C:C5	2.23	0.72
49:v:65:GLY:HA3	49:v:84:ALA:HA	1.71	0.72
28:a:2329:U:H2'	28:a:2330:G:C8	2.24	0.72
38:k:78:ARG:HE	38:k:113:ALA:HB3	1.54	0.72
32:e:112:LEU:HD23	32:e:118:LEU:HD12	1.71	0.72
33:f:30:ARG:NH1	33:f:31:VAL:O	2.23	0.72
7:A:393:A:O2'	7:A:483:C:OP1	2.06	0.72
7:A:1059:C:H4'	20:N:85:ARG:NH2	2.04	0.72
10:D:148:LYS:HG3	10:D:150:LYS:HG2	1.72	0.72
28:a:505:A:OP2	56:a:6211:SPD:N1	2.23	0.72
7:A:176:C:H3'	7:A:177:G:H21	1.55	0.71
28:a:550:C:H2'	28:a:551:G:H8	1.54	0.71
49:v:25:ARG:HD2	49:v:31:VAL:HG12	1.72	0.71
15:I:129:LYS:HD2	15:I:130:ARG:HH21	1.55	0.71
28:a:2525:G:O2'	28:a:2742:G:O2'	2.04	0.71
28:a:2750:A:O2'	28:a:2752:C:N4	2.23	0.71
48:u:7:GLU:HA	48:u:65:VAL:HG21	1.71	0.71
7:A:1506:G:O2'	7:A:1507:U:OP2	2.09	0.71
28:a:1880:U:H2'	28:a:1881:C:C6	2.26	0.71
28:a:2740:A:N7	28:a:2764:A:N6	2.39	0.71
8:B:66:LYS:HB2	8:B:158:PRO:HA	1.73	0.71
9:C:34:ASP:OD2	9:C:38:LYS:NZ	2.24	0.71
28:a:2297:A:H62	28:a:2319:G:N2	1.88	0.71
28:a:2744:G:H21	34:g:143:GLN:HE22	1.35	0.71

Continued on next page...

For Manuscript Review

Continued from previous page...

Atom-1	Atom-2	Interatomic distance (Å)	Clash overlap (Å)
7:A:383:A:H5'	7:A:384:G:H5'	1.71	0.71
28:a:630:G:N1	28:a:632:A:OP2	2.23	0.71
7:A:998:C:C6	7:A:1044:A:O5'	2.43	0.71
22:P:48:GLU:OE1	22:P:51:ARG:NH1	2.23	0.71
36:i:96:ARG:HD3	36:i:99:ARG:HD3	1.71	0.71
43:p:58:ARG:HA	43:p:61:TRP:CE3	2.25	0.71
28:a:1039:A:N1	28:a:1116:G:O6	2.24	0.71
7:A:959:A:HO2'	7:A:984:C:HO2'	1.37	0.71
7:A:1158:A:N7	7:A:1178:G:N2	2.39	0.71
29:b:74:U:H2'	48:u:29:ILE:HD13	1.71	0.71
33:f:117:LEU:H	33:f:177:PHE:HA	1.55	0.71
7:A:440:C:OP1	10:D:121:LYS:NZ	2.23	0.71
28:a:2386:A:OP1	49:v:55:ARG:NH2	2.23	0.71
28:a:2530:A:OP1	28:a:2535:G:N2	2.24	0.71
39:l:114:ARG:HA	39:l:117:PHE:HB2	1.73	0.71
43:p:61:TRP:HA	43:p:96:ALA:HB2	1.71	0.71
28:a:2317:A:H3'	28:a:2318:G:C8	2.25	0.70
28:a:2329:U:H2'	28:a:2330:G:H8	1.55	0.70
13:G:145:ALA:O	13:G:149:LYS:NZ	2.25	0.70
26:T:69:LYS:H	26:T:69:LYS:HD2	1.55	0.70
36:i:118:MET:HA	36:i:121:LYS:HG3	1.73	0.70
7:A:459:A:H61	7:A:473:A:H61	1.39	0.70
43:p:66:ASN:HD21	43:p:70:ARG:HH21	1.39	0.70
25:S:29:LYS:HB3	25:S:30:PRO:HD2	1.74	0.70
36:i:15:TRP:CG	36:i:53:TYR:HB2	2.26	0.70
48:u:76:ASP:O	48:u:90:ASP:N	2.18	0.70
7:A:415:A:H2'	7:A:416:G:C8	2.26	0.70
7:A:836:G:O6	7:A:850:U:O4	2.09	0.70
12:F:9:MET:N	12:F:9:MET:SD	2.65	0.70
24:R:50:LYS:HD3	24:R:53:ARG:HH22	1.56	0.70
28:a:1040:A:N1	28:a:1115:G:O6	2.25	0.70
28:a:2683:C:O2	37:j:70:ARG:NH2	2.22	0.70
47:t:96:PHE:N	47:t:101:GLU:O	2.22	0.70
1:0:39:PHE:HA	1:0:46:HIS:HA	1.73	0.70
28:a:1050:A:H2'	28:a:1051:G:H8	1.56	0.70
7:A:74:A:N6	7:A:95:G:OP1	2.25	0.70
28:a:1418:G:H21	28:a:1580:A:H62	0.72	0.70
29:b:29:A:H2	29:b:57:A:H61	1.38	0.70
41:n:41:ALA:HB2	41:n:48:LEU:HD11	1.72	0.70
19:M:69:LEU:O	19:M:73:ILE:HG13	1.92	0.70
28:a:1028:A:H62	28:a:1126:A:N6	1.90	0.70

Continued on next page...

For Manuscript Review

Continued from previous page...

Atom-1	Atom-2	Interatomic distance (Å)	Clash overlap (Å)
34:g:74:SER:OG	34:g:138:LYS:NZ	2.22	0.70
3:2:45:ARG:NH1	28:a:2349:G:OP1	2.24	0.69
25:S:3:ARG:HE	25:S:10:PHE:HB2	1.57	0.69
28:a:1023:U:OP2	28:a:1025:G:O2'	2.10	0.69
39:l:110:GLU:O	39:l:114:ARG:N	2.25	0.69
20:N:27:LEU:HA	20:N:30:ILE:HD12	1.74	0.69
28:a:1880:U:N3	28:a:1881:C:C4	2.61	0.69
31:d:51:THR:HB	31:d:79:LEU:HD13	1.75	0.69
32:e:168:ASP:HB2	32:e:183:PHE:HE2	1.57	0.69
7:A:120:A:H2'	7:A:122:G:H8	1.56	0.69
7:A:673:A:H2'	7:A:674:G:C8	2.28	0.69
7:A:933:G:OP1	13:G:4:ARG:NH1	2.25	0.69
28:a:2292:U:OP1	28:a:2378:A:N6	2.26	0.69
39:l:135:VAL:HG23	39:l:136:MET:HG2	1.74	0.69
7:A:484:G:H4'	7:A:485:U:C5'	2.22	0.69
28:a:634:C:H2'	28:a:635:C:C6	2.27	0.69
28:a:2739:U:N3	28:a:2764:A:N6	2.09	0.69
46:s:69:ARG:NH1	46:s:69:ARG:HB2	2.07	0.69
7:A:37:U:H3	7:A:397:A:H61	1.39	0.69
10:D:105:MET:HG3	10:D:171:LEU:HD13	1.75	0.69
49:v:43:THR:HG22	49:v:57:HIS:HB3	1.75	0.69
7:A:449:G:OP1	7:A:450:G:N2	2.25	0.69
13:G:150:ALA:HB1	17:K:59:THR:HG21	1.75	0.69
29:b:80:U:O2	29:b:96:G:O6	2.09	0.69
34:g:127:THR:OG1	34:g:128:GLN:N	2.25	0.69
29:b:14:U:H4'	29:b:69:G:H22	1.57	0.69
9:C:65:ARG:NH1	9:C:100:GLN:OE1	2.26	0.68
48:u:75:GLN:HB2	48:u:92:VAL:HG23	1.75	0.68
48:u:80:HIS:HB3	48:u:83:LYS:HZ3	1.58	0.68
19:M:13:LYS:O	19:M:13:LYS:HD2	1.93	0.68
28:a:1417:C:H2'	28:a:1418:G:H8	1.58	0.68
28:a:1039:A:C2	28:a:1116:G:N1	2.53	0.68
28:a:1042:G:O6	28:a:1113:U:O4	2.10	0.68
28:a:2747:G:N1	28:a:2756:U:OP1	2.27	0.68
34:g:103:ILE:HD12	34:g:115:HIS:HB3	1.76	0.68
27:U:66:ARG:HH11	27:U:67:ARG:HH12	1.42	0.68
39:l:75:GLU:N	39:l:75:GLU:OE2	2.26	0.68
7:A:1159:C:OP1	7:A:1182:G:N1	2.26	0.68
8:B:9:MET:HE3	8:B:14:VAL:HG21	1.73	0.68
29:b:77:U:H3	29:b:99:A:H62	0.76	0.68
33:f:32:GLU:N	33:f:157:THR:O	2.25	0.68

Continued on next page...

For Manuscript Review

Continued from previous page...

Atom-1	Atom-2	Interatomic distance (Å)	Clash overlap (Å)
7:A:1067:A:N6	7:A:1110:A:OP2	2.26	0.68
28:a:325:G:O6	56:a:6214:SPD:N1	2.26	0.68
28:a:661:A:H2'	28:a:662:G:H5'	1.74	0.68
29:b:63:C:H2'	29:b:64:G:H8	1.59	0.68
7:A:100:G:N2	7:A:152:A:O2'	2.25	0.68
28:a:1125:G:H3'	28:a:1126:A:H2'	1.76	0.68
48:u:42:LEU:HD13	48:u:43:ASP:H	1.57	0.68
7:A:1442:A:H2'	7:A:1443:G:H5'	1.76	0.68
7:A:484:G:H5'	7:A:486:U:H5"	1.75	0.67
7:A:921:U:O2	11:E:24:THR:OG1	2.12	0.67
7:A:1531:G:N7	27:U:46:LYS:NZ	2.41	0.67
28:a:636:G:OP1	38:k:129:LYS:N	2.26	0.67
32:e:150:THR:O	32:e:172:ALA:N	2.23	0.67
33:f:31:VAL:HA	33:f:158:THR:HA	1.77	0.67
7:A:239:U:O3'	7:A:240:G:H8	1.78	0.67
36:i:1:MET:HE2	44:q:13:ARG:H	1.59	0.67
51:x:11:VAL:O	51:x:15:ASN:ND2	2.24	0.67
48:u:9:ARG:O	48:u:12:GLN:NE2	2.23	0.67
28:a:268:C:N3	28:a:269:C:H5	1.92	0.67
41:n:34:HIS:O	41:n:102:ARG:NH2	2.27	0.67
28:a:1583:A:HO2'	28:a:1585:C:H41	1.41	0.67
32:e:113:VAL:HG23	32:e:118:LEU:HD13	1.77	0.67
7:A:413:A:H5'	7:A:414:A:H8	1.59	0.67
7:A:1223:G:OP2	7:A:1323:C:N4	2.21	0.67
19:M:95:LEU:HA	19:M:110:LYS:HB2	1.77	0.67
28:a:629:G:H2'	28:a:630:G:O4'	1.95	0.67
28:a:2351:G:N2	28:a:2366:A:H8	1.91	0.67
15:I:28:ILE:HG23	15:I:63:LEU:HD12	1.76	0.66
20:N:47:LYS:O	20:N:50:THR:OG1	2.13	0.66
27:U:13:ASP:OD1	27:U:13:ASP:N	2.28	0.66
29:b:92:C:OP1	48:u:18:ARG:NH2	2.15	0.66
7:A:714:G:H2'	7:A:715:A:C8	2.30	0.66
28:a:1043:C:O2	28:a:1112:G:N2	2.16	0.66
34:g:126:PRO:HB2	34:g:127:THR:HG22	1.78	0.66
36:i:17:VAL:HG12	36:i:137:PRO:HB2	1.77	0.66
28:a:327:G:N7	56:a:6214:SPD:N10	2.42	0.66
28:a:1168:G:O6	28:a:1181:U:O4	2.13	0.66
32:e:2:GLU:OE2	32:e:12:LEU:N	2.27	0.66
38:k:110:VAL:HB	38:k:127:VAL:HG13	1.77	0.66
41:n:30:ARG:NH1	41:n:100:HIS:O	2.24	0.66
5:4:37:CYS:O	5:4:41:HIS:HB2	1.95	0.66

Continued on next page...

For Manuscript Review

Continued from previous page...

Atom-1	Atom-2	Interatomic distance (Å)	Clash overlap (Å)
28:a:1027:A:C2'	28:a:1028:A:H8	2.07	0.66
41:n:29:HIS:HD1	41:n:97:PHE:HZ	1.44	0.66
48:u:83:LYS:HB3	48:u:85:LYS:HG3	1.76	0.66
13:G:140:ASP:HA	13:G:143:ARG:HE	1.61	0.66
28:a:2751:G:OP1	28:a:2751:G:N2	2.29	0.66
45:r:48:LYS:O	45:r:52:GLU:HG3	1.95	0.66
7:A:501:C:H2'	7:A:502:A:C8	2.30	0.66
9:C:36:ASP:HB3	9:C:40:ARG:HH11	1.61	0.66
18:L:57:LEU:HD21	18:L:82:ILE:HD11	1.78	0.66
41:n:25:ARG:HB2	41:n:40:ILE:HB	1.78	0.66
43:p:83:LEU:HA	43:p:112:LYS:NZ	2.10	0.66
53:z:34:SER:OG	53:z:36:GLU:OE1	2.14	0.66
14:H:32:LEU:O	14:H:36:ILE:HD12	1.95	0.66
49:v:75:LYS:HD3	49:v:77:ARG:HB2	1.78	0.66
7:A:194:C:H2'	7:A:195:A:H4'	1.78	0.66
48:u:79:ARG:HA	48:u:86:LEU:HA	1.77	0.66
7:A:998:C:H6	7:A:1044:A:H5'	1.54	0.66
8:B:188:ASP:HB2	8:B:204:ASP:HB3	1.77	0.66
28:a:1881:C:H2'	28:a:1882:U:C6	2.30	0.66
28:a:1881:C:H2'	28:a:1882:U:H6	1.59	0.66
28:a:2333:A:H1'	41:n:9:ARG:NH2	2.10	0.66
38:k:108:ALA:O	38:k:125:LEU:HB3	1.96	0.66
43:p:69:ALA:HB2	43:p:106:PHE:HZ	1.61	0.66
15:I:23:PRO:HA	15:I:61:LEU:HD12	1.77	0.65
28:a:2324:U:C2	28:a:2331:G:O6	2.48	0.65
36:i:15:TRP:CD2	36:i:53:TYR:HB2	2.31	0.65
47:t:50:PRO:O	47:t:54:GLN:NE2	2.29	0.65
34:g:39:ASP:OD1	34:g:55:ARG:NH1	2.30	0.65
14:H:29:SER:HB2	14:H:59:LEU:HB2	1.78	0.65
28:a:1020:A:C6	28:a:1141:U:C2	2.80	0.65
28:a:1392:A:N6	46:s:18:GLU:OE1	2.29	0.65
29:b:50:A:N6	29:b:51:G:O6	2.29	0.65
40:m:114:GLU:OE1	40:m:118:ARG:NH1	2.30	0.65
28:a:2261:C:O2'	28:a:2387:U:O2	2.12	0.65
30:c:71:LYS:NZ	30:c:100:GLU:OE2	2.26	0.65
7:A:376:G:H2'	7:A:377:G:H8	1.61	0.65
28:a:2303:G:O2'	33:f:121:SER:O	2.15	0.65
28:a:2319:G:H1'	28:a:2320:U:C5	2.32	0.65
44:q:14:VAL:HG11	44:q:20:VAL:HG13	1.79	0.65
1:0:8:LYS:NZ	28:a:2420:C:OP1	2.22	0.65
28:a:2748:A:H4'	34:g:4:VAL:HG11	1.78	0.65

Continued on next page...

For Manuscript Review

Continued from previous page...

Atom-1	Atom-2	Interatomic distance (Å)	Clash overlap (Å)
36:i:15:TRP:HA	36:i:53:TYR:N	2.10	0.65
8:B:9:MET:HG3	8:B:209:ALA:HB2	1.79	0.65
9:C:113:ALA:HB3	9:C:185:ASN:HB2	1.79	0.65
30:c:124:ILE:HD12	30:c:124:ILE:O	1.97	0.65
7:A:891:U:HO2’	7:A:892:A:H8	1.45	0.65
28:a:549:G:H2’	28:a:550:C:C6	2.32	0.65
28:a:2293:G:H4’	41:n:98:GLN:CD	2.21	0.65
15:I:72:ILE:HD12	15:I:73:SER:H	1.62	0.64
28:a:2528:U:N3	28:a:2536:G:N1	2.44	0.64
29:b:78:A:N6	29:b:98:G:C2	2.61	0.64
32:e:121:VAL:HG23	32:e:188:MET:H	1.61	0.64
44:q:10:LYS:C	44:q:11:GLN:HG2	2.22	0.64
31:d:149:ASN:O	31:d:151:THR:N	2.29	0.64
43:p:61:TRP:CG	43:p:93:LYS:HA	2.31	0.64
48:u:77:VAL:HA	48:u:89:ILE:HA	1.80	0.64
7:A:136:C:H41	7:A:227:G:H22	1.44	0.64
7:A:157:U:H3	7:A:164:G:H1	1.45	0.64
29:b:29:A:O2’	29:b:58:A:N6	2.23	0.64
7:A:1267:G:N2	7:A:1327:U:O2’	2.29	0.64
11:E:12:GLN:OE1	11:E:14:LYS:NZ	2.26	0.64
28:a:1021:A:OP1	28:a:1122:G:O2’	2.16	0.64
28:a:84:A:N1	28:a:98:G:O2’	2.30	0.64
28:a:1418:G:N2	28:a:1580:A:C6	2.65	0.64
28:a:2318:G:H2’	28:a:2319:G:C2	2.33	0.64
5:4:56:ARG:HE	25:S:65:GLU:H	1.45	0.64
11:E:13:GLU:N	11:E:13:GLU:OE1	2.30	0.64
28:a:1021:A:H5’	28:a:1022:G:OP2	1.98	0.64
28:a:2355:G:H2’	28:a:2356:U:C6	2.33	0.64
28:a:996:A:C4’	44:q:11:GLN:HG3	2.24	0.64
33:f:48:LYS:NZ	33:f:48:LYS:O	2.28	0.64
38:k:108:ALA:N	38:k:125:LEU:HD22	2.12	0.64
32:e:196:VAL:O	32:e:200:LEU:HG	1.98	0.64
47:t:62:GLU:N	47:t:62:GLU:OE2	2.31	0.64
19:M:113:ARG:H	19:M:113:ARG:HD3	1.63	0.64
12:F:70:VAL:O	12:F:74:LEU:HD12	1.97	0.63
19:M:41:GLU:HG2	19:M:41:GLU:O	1.98	0.63
29:b:28:C:H2’	29:b:29:A:O4’	1.98	0.63
7:A:1533:U:H4’	7:A:1534:C:H5’	1.79	0.63
2:1:33:ARG:NH1	28:a:467:G:OP1	2.31	0.63
10:D:87:GLY:HA3	10:D:198:HIS:CD2	2.34	0.63
13:G:56:LYS:HB3	13:G:60:GLU:HG2	1.80	0.63

Continued on next page...

For Manuscript Review

Continued from previous page...

Atom-1	Atom-2	Interatomic distance (Å)	Clash overlap (Å)
42:o:33:VAL:HG22	42:o:38:LYS:HZ3	1.63	0.63
28:a:840:C:H2'	28:a:841:G:H8	1.64	0.63
28:a:2630:G:H2'	28:a:2631:G:C8	2.34	0.63
43:p:65:ILE:HG12	43:p:95:LEU:HD22	1.81	0.63
7:A:437:U:O5'	10:D:154:ARG:NH1	2.23	0.63
28:a:544:C:N4	28:a:548:G:O6	2.31	0.63
28:a:1048:A:N1	28:a:1112:G:O2'	2.24	0.63
29:b:27:C:N3	29:b:28:C:N4	2.46	0.63
29:b:79:G:N7	48:u:14:LYS:NZ	2.44	0.63
29:b:94:A:OP1	48:u:19:ARG:NE	2.31	0.63
7:A:410:G:H21	7:A:432:A:H62	1.46	0.63
8:B:73:LYS:HB3	8:B:76:ALA:HB3	1.81	0.63
28:a:2523:G:N2	28:a:2764:A:H1'	2.12	0.63
34:g:164:TYR:HB2	34:g:167:GLU:HB2	1.81	0.63
47:t:50:PRO:HA	47:t:54:GLN:HG3	1.81	0.63
7:A:642:A:HO2'	7:A:643:C:H6	1.45	0.63
7:A:778:G:N2	17:K:122:ARG:HD3	2.12	0.63
8:B:143:LYS:O	8:B:147:SER:OG	2.14	0.63
8:B:162:PHE:HD1	8:B:184:PHE:HE2	1.46	0.63
9:C:5:VAL:HG21	9:C:10:ILE:HD11	1.80	0.63
19:M:67:GLY:O	19:M:71:ARG:HG3	1.99	0.63
8:B:13:GLY:HA2	8:B:16:PHE:HB2	1.80	0.63
10:D:184:ARG:HG2	10:D:186:PRO:HD3	1.81	0.63
28:a:600:G:C6	28:a:657:U:O4	2.52	0.63
28:a:848:C:H2'	28:a:849:A:H8	1.63	0.63
28:a:2356:U:N3	28:a:2357:G:N7	2.46	0.63
28:a:2893:A:H1'	28:a:2894:G:C6	2.34	0.63
29:b:34:A:C6	29:b:44:G:H1'	2.34	0.63
33:f:30:ARG:O	33:f:159:THR:N	2.31	0.63
36:i:96:ARG:HG2	36:i:96:ARG:HH11	1.64	0.63
38:k:74:THR:HG22	38:k:107:PHE:HB2	1.81	0.63
41:n:3:LYS:O	41:n:7:ARG:HB2	1.98	0.63
41:n:76:LYS:HD3	41:n:109:ALA:HB1	1.80	0.63
7:A:123:C:H42	7:A:238:G:H1	1.47	0.62
11:E:111:MET:HG2	11:E:133:PRO:HA	1.81	0.62
17:K:20:VAL:HG13	17:K:83:GLU:HG2	1.81	0.62
27:U:5:LYS:O	27:U:18:ARG:NH1	2.32	0.62
34:g:105:LEU:HB2	34:g:113:VAL:HB	1.81	0.62
7:A:1253:A:H2	7:A:1370:C:H1'	1.64	0.62
9:C:182:ILE:HD13	9:C:203:PHE:HB2	1.80	0.62
19:M:12:HIS:O	19:M:15:ALA:N	2.29	0.62

Continued on next page...

For Manuscript Review

Continued from previous page...

Atom-1	Atom-2	Interatomic distance (Å)	Clash overlap (Å)
36:i:18:VAL:HA	36:i:139:VAL:HG13	1.80	0.62
40:m:22:ARG:HD2	40:m:70:THR:H	1.63	0.62
42:o:27:GLU:HB2	42:o:44:GLU:HG3	1.80	0.62
1:0:11:LEU:HD23	1:0:49:TYR:CD2	2.34	0.62
3:2:42:ARG:HG3	28:a:2349:G:OP2	1.99	0.62
7:A:41:G:H1	7:A:401:C:H5	1.47	0.62
9:C:153:VAL:HG22	9:C:198:VAL:HG22	1.81	0.62
28:a:284:U:O2	28:a:356:G:N2	2.25	0.62
28:a:597:G:H2'	28:a:598:U:O4'	1.99	0.62
28:a:1036:G:C6	28:a:1119:U:O2	2.52	0.62
29:b:14:U:OP2	29:b:70:C:O2'	2.17	0.62
49:v:18:ALA:O	49:v:20:ARG:NH1	2.32	0.62
7:A:472:U:H2'	7:A:473:A:H8	1.64	0.62
19:M:75:MET:O	19:M:75:MET:HE3	1.99	0.62
24:R:34:THR:HG22	24:R:36:SER:H	1.64	0.62
28:a:938:G:C2	28:a:939:G:N7	2.67	0.62
29:b:17:C:H2'	29:b:18:G:C8	2.35	0.62
41:n:111:ARG:NH2	41:n:117:PHE:O	2.32	0.62
7:A:619:U:H5	10:D:131:ASN:HB3	1.64	0.62
11:E:41:ASP:OD1	11:E:42:GLY:N	2.33	0.62
28:a:1581:G:H2'	28:a:1582:C:C6	2.35	0.62
28:a:2307:G:N2	28:a:2311:A:O2'	2.32	0.62
29:b:80:U:O2	29:b:96:G:C6	2.52	0.62
7:A:1121:U:H2'	7:A:1122:U:C6	2.35	0.62
10:D:35:GLU:O	10:D:36:GLN:C	2.42	0.62
13:G:111:ARG:NH1	13:G:113:ASP:OD2	2.32	0.62
28:a:1416:G:O2'	28:a:1417:C:OP2	2.13	0.62
28:a:2043:C:N4	28:a:2625:G:N2	2.47	0.62
29:b:86:G:N1	29:b:88:C:O4'	2.33	0.62
5:4:24:ILE:HD11	33:f:102:ARG:HG2	1.82	0.62
28:a:534:U:O2'	43:p:49:ASP:OD2	2.12	0.62
30:c:142:HIS:ND1	30:c:193:GLY:O	2.28	0.62
7:A:404:G:OP2	10:D:115:ARG:NH2	2.33	0.62
12:F:6:ILE:HG12	12:F:89:VAL:HG22	1.82	0.62
28:a:816:C:H5'	28:a:1166:G:H1'	1.81	0.62
28:a:1039:A:H2	28:a:1116:G:N1	1.83	0.62
28:a:1125:G:H2'	28:a:1126:A:C8	2.34	0.62
28:a:1315:C:O2'	28:a:1392:A:N3	2.32	0.62
28:a:2334:U:H5'	41:n:12:THR:HB	1.82	0.62
28:a:2336:A:H61	49:v:57:HIS:CD2	2.16	0.62
4:3:24:ARG:HA	4:3:36:ARG:HA	1.80	0.62

Continued on next page...

For Manuscript Review

Continued from previous page...

Atom-1	Atom-2	Interatomic distance (Å)	Clash overlap (Å)
7:A:1228:A:H8	25:S:84:ALA:HB2	1.65	0.62
28:a:1166:G:O6	28:a:1184:U:C4	2.52	0.62
28:a:2334:U:H5”	41:n:9:ARG:HH22	1.64	0.62
28:a:2545:G:H2’	28:a:2546:U:O4’	2.00	0.62
36:i:122:LEU:O	36:i:123:LYS:HD3	2.00	0.62
45:r:31:GLN:O	45:r:35:ILE:HG13	2.00	0.62
7:A:713:G:H2’	7:A:714:G:C8	2.35	0.62
28:a:1028:A:N1	28:a:2487:G:O2’	2.32	0.62
36:i:54:ILE:HG23	36:i:122:LEU:HA	1.81	0.62
37:j:112:PHE:HB3	37:j:115:ILE:HD12	1.82	0.62
41:n:36:TYR:HA	41:n:52:SER:HB2	1.81	0.62
7:A:998:C:C5	7:A:1044:A:O5’	2.53	0.61
28:a:1802:A:H2’	28:a:1803:A:C8	2.35	0.61
28:a:2666:C:N4	34:g:108:GLY:O	2.33	0.61
33:f:128:TYR:OH	33:f:130:MET:SD	2.57	0.61
7:A:1039:C:H1’	7:A:1040:U:H2’	1.82	0.61
19:M:95:LEU:HD12	19:M:96:PRO:HD2	1.81	0.61
23:Q:76:VAL:HG12	23:Q:77:ARG:HG2	1.81	0.61
28:a:1880:U:H2’	28:a:1881:C:H6	1.65	0.61
31:d:34:VAL:HG22	31:d:50:VAL:HB	1.83	0.61
7:A:346:G:OP1	42:o:39:ARG:NH2	2.32	0.61
7:A:642:A:O2’	7:A:643:C:O5’	2.17	0.61
7:A:653:A:H3’	7:A:654:G:H21	1.63	0.61
8:B:184:PHE:HB2	8:B:198:PHE:HB2	1.81	0.61
28:a:1021:A:C6	28:a:1023:U:C4	2.88	0.61
34:g:98:VAL:HG21	34:g:131:ILE:HG21	1.81	0.61
36:i:53:TYR:CD1	36:i:121:LYS:HD2	2.35	0.61
7:A:548:G:O2’	7:A:549:C:OP2	2.14	0.61
28:a:2758:A:N1	34:g:139:GLN:NE2	2.48	0.61
34:g:101:ASN:HA	34:g:117:LEU:HB2	1.82	0.61
43:p:64:ARG:CZ	43:p:100:VAL:HG21	2.30	0.61
43:p:61:TRP:CD2	43:p:93:LYS:HB2	2.35	0.61
4:3:25:VAL:CG1	4:3:35:GLN:H	2.12	0.61
4:3:25:VAL:HG13	4:3:35:GLN:H	1.65	0.61
7:A:1012:G:H21	7:A:1013:G:H8	1.48	0.61
7:A:1108:G:H22	7:A:1111:A:H62	1.48	0.61
7:A:1302:U:O2’	7:A:1303:C:OP1	2.18	0.61
15:I:127:PHE:HE2	15:I:129:LYS:HE2	1.65	0.61
16:J:79:PRO:HG2	16:J:79:PRO:O	1.97	0.61
28:a:2524:G:C5	28:a:2525:G:C8	2.88	0.61
28:a:2740:A:C5	28:a:2741:A:C5	2.89	0.61

Continued on next page...

For Manuscript Review

Continued from previous page...

Atom-1	Atom-2	Interatomic distance (Å)	Clash overlap (Å)
31:d:33:ARG:HA	31:d:94:GLN:O	2.01	0.61
38:k:78:ARG:HB2	38:k:78:ARG:NH1	2.14	0.61
13:G:127:ALA:HA	13:G:131:LYS:HB2	1.82	0.61
28:a:1024:G:OP2	28:a:1025:G:H5"	2.01	0.61
28:a:2519:U:C4	28:a:2542:A:N6	2.68	0.61
38:k:74:THR:HG21	38:k:126:ARG:HH12	1.65	0.61
7:A:202:G:O2'	7:A:468:A:O2'	2.18	0.61
8:B:153:ASP:O	8:B:155:GLY:N	2.34	0.61
19:M:64:VAL:HB	19:M:68:ASP:HB3	1.83	0.61
7:A:28:A:O2'	7:A:296:U:OP1	2.15	0.61
15:I:5:GLN:HB2	15:I:22:LYS:HZ2	1.66	0.61
31:d:50:VAL:HG11	31:d:82:PHE:CZ	2.35	0.61
7:A:507:C:H3'	7:A:508:U:H5'	1.83	0.60
7:A:1315:C:OP2	25:S:4:SER:OG	2.12	0.60
28:a:917:A:HO2'	29:b:99:A:HO2'	1.23	0.60
7:A:1356:A:H2'	7:A:1357:A:H8	1.64	0.60
9:C:92:ALA:HB2	9:C:98:PRO:HG3	1.82	0.60
28:a:476:G:N1	28:a:479:A:OP2	2.32	0.60
7:A:492:C:H2'	7:A:493:A:C8	2.37	0.60
7:A:1312:A:H61	7:A:1327:U:H3	1.49	0.60
7:A:1318:C:C4	20:N:53:ARG:HD3	2.36	0.60
10:D:19:LEU:HG	10:D:64:ILE:HD12	1.83	0.60
28:a:2336:A:H61	49:v:57:HIS:HD2	1.48	0.60
28:a:2472:G:N2	28:a:2478:A:N6	2.28	0.60
31:d:74:GLU:N	31:d:74:GLU:OE2	2.34	0.60
36:i:53:TYR:CA	36:i:121:LYS:HB3	2.28	0.60
43:p:88:VAL:HG13	43:p:90:ILE:HG13	1.83	0.60
7:A:885:G:N7	7:A:912:C:N4	2.49	0.60
7:A:994:A:H61	7:A:1044:A:H2	1.49	0.60
46:s:6:ARG:NH2	46:s:37:ASP:OD1	2.33	0.60
12:F:20:GLY:O	12:F:24:ARG:HD3	2.02	0.60
28:a:2741:A:H62	28:a:2763:G:H21	1.49	0.60
26:T:35:VAL:HG22	26:T:39:ILE:HD11	1.84	0.60
28:a:603:A:H4'	28:a:604:G:H4'	1.83	0.60
28:a:1028:A:H62	28:a:1126:A:H62	1.47	0.60
28:a:2315:G:O4'	33:f:127:ASN:ND2	2.35	0.60
43:p:91:ASP:OD1	43:p:91:ASP:N	2.31	0.60
7:A:994:A:N6	7:A:1044:A:H2	2.00	0.60
15:I:44:ALA:O	15:I:48:VAL:HG12	2.02	0.60
28:a:634:C:C4	28:a:635:C:N4	2.69	0.60
33:f:35:THR:HB	33:f:155:THR:HB	1.84	0.60

Continued on next page...

For Manuscript Review

Continued from previous page...

Atom-1	Atom-2	Interatomic distance (Å)	Clash overlap (Å)
7:A:1264:A:H61	7:A:1274:G:H1’	1.66	0.60
8:B:35:ARG:HB3	8:B:38:VAL:HG12	1.83	0.60
10:D:110:THR:HG22	10:D:112:ALA:H	1.67	0.60
22:P:17:TYR:HD2	22:P:42:ILE:HD11	1.66	0.60
33:f:8:TYR:HA	33:f:12:VAL:HB	1.82	0.60
43:p:91:ASP:O	43:p:95:LEU:N	2.35	0.60
49:v:46:HIS:N	49:v:78:LYS:O	2.29	0.60
23:Q:62:ARG:NH1	23:Q:63:GLU:O	2.35	0.60
29:b:70:C:H2’	29:b:71:C:C6	2.36	0.60
48:u:75:GLN:HB3	48:u:90:ASP:HB3	1.84	0.60
1:0:13:SER:OG	1:0:14:SER:N	2.32	0.60
7:A:72:G:H1	7:A:97:G:H21	1.50	0.60
28:a:2355:G:O2’	28:a:2356:U:O4’	2.13	0.60
29:b:92:C:O5’	48:u:18:ARG:NH1	2.34	0.60
7:A:362:G:N2	7:A:365:U:OP2	2.32	0.59
28:a:2313:C:H2’	28:a:2314:A:H8	1.66	0.59
28:a:2541:A:OP2	56:a:6210:SPD:N1	2.35	0.59
32:e:5:LEU:HB2	32:e:9:GLN:N	2.17	0.59
39:l:113:ALA:HA	39:l:116:ALA:HB3	1.84	0.59
20:N:47:LYS:HZ2	20:N:48:LEU:HD13	1.67	0.59
28:a:2298:A:H61	28:a:2318:G:H1’	1.67	0.59
28:a:2333:A:O5’	28:a:2335:A:H1’	2.01	0.59
30:c:3:VAL:HG12	30:c:19:VAL:HG22	1.83	0.59
34:g:44:LYS:HG2	34:g:45:HIS:H	1.67	0.59
44:q:15:SER:HB2	44:q:18:GLN:HB2	1.83	0.59
48:u:57:TYR:OH	48:u:79:ARG:NH1	2.21	0.59
7:A:363:A:N6	18:L:27:CYS:SG	2.75	0.59
28:a:1589:U:H2’	28:a:1590:A:C8	2.36	0.59
39:l:45:GLN:NE2	39:l:125:PRO:HD3	2.18	0.59
41:n:29:HIS:HA	41:n:97:PHE:CE2	2.37	0.59
48:u:4:ILE:N	48:u:62:THR:O	2.35	0.59
5:4:37:CYS:HB2	5:4:40:CYS:SG	2.42	0.59
7:A:1108:G:N2	7:A:1111:A:H62	2.00	0.59
28:a:2640:G:OP1	36:i:96:ARG:NH2	2.26	0.59
39:l:116:ALA:O	39:l:120:ALA:N	2.35	0.59
12:F:4:TYR:O	12:F:63:ASN:ND2	2.36	0.59
32:e:148:ILE:HG23	32:e:169:VAL:HA	1.84	0.59
33:f:69:LYS:HB3	33:f:82:GLY:HA2	1.83	0.59
34:g:155:GLU:N	34:g:160:LYS:O	2.32	0.59
7:A:195:A:H2’	7:A:196:A:C5	2.37	0.59
23:Q:19:LYS:H	23:Q:51:ASN:ND2	2.01	0.59

Continued on next page...

For Manuscript Review

Continued from previous page...

Atom-1	Atom-2	Interatomic distance (Å)	Clash overlap (Å)
28:a:628:G:H2'	28:a:629:G:O4'	2.03	0.59
28:a:2746:U:H4'	34:g:138:LYS:HG2	1.84	0.59
34:g:44:LYS:HD2	34:g:51:THR:H	1.67	0.59
7:A:615:G:H2'	7:A:616:G:H5'	1.84	0.59
26:T:26:SER:O	26:T:30:THR:HG23	2.03	0.59
28:a:2324:U:O3'	28:a:2337:G:H5"	2.03	0.59
15:I:17:ALA:HB2	15:I:67:VAL:HG23	1.85	0.59
19:M:10:PRO:HB3	19:M:13:LYS:HG2	1.85	0.59
28:a:2352:A:H62	28:a:2365:G:H21	0.70	0.59
28:a:2630:G:C4	28:a:2894:G:C6	2.90	0.59
33:f:91:LEU:HD12	33:f:95:ARG:HB3	1.83	0.59
7:A:473:A:OP1	22:P:76:LYS:NZ	2.30	0.59
7:A:1445:U:H2'	7:A:1446:U:O4'	2.02	0.59
12:F:77:THR:O	12:F:81:ASN:HB2	2.03	0.59
28:a:602:A:N6	28:a:655:A:O2'	2.36	0.59
28:a:2324:U:O2	28:a:2331:G:O6	2.21	0.59
48:u:48:MET:HA	48:u:51:GLN:OE1	2.03	0.59
48:u:79:ARG:HA	48:u:87:GLN:H	1.68	0.59
16:J:27:GLU:O	16:J:31:ARG:HB3	2.03	0.59
28:a:2469:A:N6	28:a:2481:G:O2'	2.35	0.59
7:A:143:A:C2	7:A:220:G:N1	2.59	0.58
25:S:62:VAL:HG12	25:S:64:ASP:H	1.68	0.58
29:b:9:G:O6	29:b:111:U:C2	2.56	0.58
29:b:70:C:H2'	29:b:71:C:H6	1.68	0.58
44:q:3:ALA:O	44:q:14:VAL:N	2.28	0.58
48:u:63:ILE:O	48:u:69:GLU:HA	2.03	0.58
7:A:657:U:H2'	7:A:658:A:H8	1.69	0.58
7:A:1312:A:H3'	7:A:1313:G:H5"	1.85	0.58
28:a:1021:A:H3'	28:a:1022:G:H4'	1.85	0.58
34:g:98:VAL:HG22	34:g:103:ILE:HG23	1.85	0.58
34:g:111:HIS:NE2	34:g:113:VAL:HG22	2.17	0.58
7:A:91:U:H2'	7:A:92:U:C6	2.38	0.58
7:A:1321:C:OP2	25:S:37:ARG:NH1	2.36	0.58
22:P:1:MET:HE2	22:P:1:MET:HA	1.85	0.58
7:A:1240:A:H4'	7:A:1242:G:H1'	1.86	0.58
12:F:5:GLU:OE2	12:F:63:ASN:ND2	2.33	0.58
15:I:118:LEU:HD22	15:I:124:ARG:HG2	1.85	0.58
28:a:602:A:O2'	28:a:604:G:O2'	2.16	0.58
31:d:48:ILE:HD11	31:d:82:PHE:CE1	2.38	0.58
32:e:5:LEU:HB2	32:e:9:GLN:H	1.67	0.58
34:g:94:TYR:HB3	34:g:107:LEU:HD22	1.85	0.58

Continued on next page...

For Manuscript Review

Continued from previous page...

Atom-1	Atom-2	Interatomic distance (Å)	Clash overlap (Å)
43:p:58:ARG:HA	43:p:61:TRP:CD2	2.38	0.58
8:B:10:LEU:HD12	8:B:43:LEU:HD23	1.85	0.58
16:J:22:THR:O	16:J:26:VAL:HG23	2.04	0.58
24:R:48:ARG:HB2	24:R:51:TYR:HD2	1.68	0.58
28:a:1880:U:C2	28:a:1881:C:C5	2.91	0.58
28:a:2745:C:H5'	34:g:146:ALA:HB2	1.84	0.58
38:k:123:ARG:HB2	38:k:143:GLU:OE1	2.03	0.58
46:s:17:SER:OG	46:s:18:GLU:N	2.37	0.58
7:A:1163:C:H2'	7:A:1164:G:C8	2.39	0.58
15:I:55:VAL:HG11	15:I:87:LEU:HD11	1.86	0.58
24:R:42:SER:HB3	24:R:52:GLN:HG3	1.86	0.58
41:n:35:ILE:HB	41:n:102:ARG:NH2	2.18	0.58
44:q:31:GLU:N	44:q:31:GLU:OE2	2.37	0.58
8:B:21:ARG:NH2	8:B:22:TYR:OH	2.36	0.58
17:K:78:GLY:O	17:K:79:ILE:HD13	2.03	0.58
34:g:2:SER:OG	34:g:3:ARG:N	2.34	0.58
12:F:5:GLU:O	12:F:90:MET:N	2.25	0.58
28:a:636:G:OP2	38:k:128:THR:HA	2.03	0.58
7:A:199:C:H2'	7:A:200:A:H8	1.68	0.58
7:A:952:U:H5'	7:A:972:C:H41	1.68	0.58
7:A:1028:C:H1'	7:A:1035:A:N6	2.18	0.58
7:A:1219:U:H2'	7:A:1220:A:C8	2.38	0.58
10:D:125:VAL:HG12	10:D:126:ASN:OD1	2.04	0.58
22:P:17:TYR:CD2	22:P:42:ILE:HD11	2.38	0.58
26:T:35:VAL:O	26:T:39:ILE:HG13	2.04	0.58
43:p:79:PHE:CZ	43:p:109:LEU:HB3	2.39	0.58
1:0:8:LYS:NZ	28:a:2421:G:OP2	2.29	0.58
3:2:28:ASN:O	3:2:36:LYS:NZ	2.37	0.58
28:a:638:G:H2'	28:a:639:U:C6	2.39	0.58
28:a:2319:G:H1'	28:a:2320:U:H5	1.68	0.58
42:o:16:ASP:N	42:o:16:ASP:OD1	2.36	0.58
7:A:1057:G:H8	7:A:1057:G:OP1	1.87	0.57
24:R:35:GLU:OE2	24:R:35:GLU:N	2.25	0.57
28:a:424:G:C6	28:a:425:G:C4	2.92	0.57
41:n:4:LYS:O	41:n:8:ILE:HG12	2.03	0.57
3:2:15:LYS:HE3	28:a:630:G:N7	2.18	0.57
3:2:34:THR:OG1	28:a:2420:C:OP1	2.22	0.57
22:P:41:PRO:C	22:P:42:ILE:HD12	2.28	0.57
11:E:159:LYS:HD3	11:E:163:GLU:HG3	1.86	0.57
12:F:81:ASN:HB3	12:F:84:VAL:HG12	1.86	0.57
28:a:856:G:H2'	28:a:857:G:C8	2.39	0.57

Continued on next page...

For Manuscript Review

Continued from previous page...

Atom-1	Atom-2	Interatomic distance (Å)	Clash overlap (Å)
28:a:2893:A:H1’	28:a:2894:G:C5	2.39	0.57
48:u:30:ILE:HD11	48:u:93:ARG:HE	1.68	0.57
7:A:1140:G:H1’	7:A:1141:G:N7	2.20	0.57
12:F:29:ILE:HD12	12:F:64:VAL:HG22	1.86	0.57
17:K:23:ILE:HB	17:K:86:VAL:HG12	1.85	0.57
28:a:275:C:O2	28:a:362:A:N6	2.38	0.57
7:A:8:A:C6	10:D:206:LYS:HG3	2.39	0.57
15:I:62:ASP:O	15:I:63:LEU:HD23	2.04	0.57
28:a:2541:A:H2’	28:a:2542:A:C8	2.39	0.57
28:a:2762:C:H2’	28:a:2763:G:O4’	2.03	0.57
49:v:72:LYS:CD	49:v:79:PHE:HB2	2.33	0.57
7:A:1190:C:H4’	7:A:1191:G:O5’	2.05	0.57
7:A:1319:A:H4’	25:S:10:PHE:CE1	2.40	0.57
19:M:114:LYS:HD2	19:M:114:LYS:O	2.05	0.57
26:T:22:ALA:O	26:T:26:SER:OG	2.19	0.57
28:a:631:A:HO2’	28:a:632:A:P	2.26	0.57
28:a:2356:U:H2’	28:a:2357:G:O4’	2.04	0.57
29:b:40:U:H1’	29:b:45:A:N6	2.16	0.57
33:f:144:ASP:OD1	33:f:144:ASP:N	2.35	0.57
7:A:7:A:H2’	11:E:124:LEU:HD23	1.85	0.57
7:A:96:U:H2’	7:A:97:G:O4’	2.05	0.57
7:A:272:C:H2’	7:A:273:A:H8	1.69	0.57
14:H:90:ASP:OD1	14:H:90:ASP:N	2.36	0.57
15:I:115:LYS:HB3	15:I:118:LEU:HD12	1.87	0.57
28:a:2305:U:H1’	33:f:133:ARG:HE	1.70	0.57
30:c:146:MET:HE3	30:c:146:MET:HA	1.87	0.57
31:d:2:ILE:HD11	31:d:48:ILE:HD13	1.85	0.57
4:3:15:LYS:HD3	4:3:16:ILE:N	2.19	0.57
7:A:413:A:H5’	7:A:414:A:C8	2.40	0.57
7:A:521:G:O2’	7:A:537:G:OP1	2.23	0.57
7:A:1012:G:N2	7:A:1017:C:OP2	2.36	0.57
7:A:1234:G:OP1	15:I:119:ARG:NH2	2.37	0.57
19:M:30:SER:O	19:M:34:LEU:CB	2.53	0.57
28:a:86:G:C2	28:a:87:U:C4	2.93	0.57
28:a:2479:U:C2	28:a:2480:C:H1’	2.39	0.57
33:f:110:ARG:NE	33:f:137:ILE:O	2.31	0.57
49:v:40:GLN:NE2	49:v:45:PHE:O	2.25	0.57
10:D:44:ARG:O	10:D:45:LYS:HB2	2.03	0.57
28:a:841:G:C6	28:a:842:U:C4	2.93	0.57
28:a:2316:G:H4’	33:f:125:ARG:NH1	2.19	0.57
28:a:2544:G:H2’	28:a:2545:G:O4’	2.05	0.57

Continued on next page...

For Manuscript Review

Continued from previous page...

Atom-1	Atom-2	Interatomic distance (Å)	Clash overlap (Å)
7:A:1159:C:O2'	7:A:1160:U:OP1	2.22	0.56
13:G:50:LEU:HG	13:G:121:ALA:HB1	1.87	0.56
19:M:30:SER:HA	19:M:33:ILE:HG22	1.87	0.56
28:a:636:G:P	38:k:128:THR:HA	2.45	0.56
38:k:127:VAL:HG11	38:k:132:ARG:HB3	1.87	0.56
48:u:44:HIS:CE1	48:u:85:LYS:HB3	2.39	0.56
28:a:1040:A:H2	28:a:1115:G:H1	1.50	0.56
34:g:20:ASN:OD1	34:g:20:ASN:N	2.38	0.56
1:0:39:PHE:HD1	1:0:46:HIS:CG	2.23	0.56
5:4:9:TYR:CE2	33:f:62:GLY:HA3	2.40	0.56
7:A:522:C:H5	18:L:50:ARG:HH12	1.53	0.56
7:A:642:A:O2'	7:A:643:C:H6	1.87	0.56
23:Q:51:ASN:OD1	23:Q:51:ASN:N	2.35	0.56
24:R:11:CYS:HB2	24:R:47:THR:HA	1.88	0.56
28:a:84:A:C3'	47:t:7:ARG:HH22	2.19	0.56
28:a:634:C:C4	28:a:635:C:C4	2.93	0.56
28:a:2327:A:N6	28:a:2388:A:H61	2.03	0.56
29:b:12:C:O2'	49:v:74:PRO:O	2.22	0.56
29:b:52:A:H2	29:b:53:A:H62	1.52	0.56
32:e:2:GLU:OE1	32:e:13:THR:OG1	2.16	0.56
41:n:8:ILE:O	41:n:12:THR:OG1	2.23	0.56
41:n:79:ALA:HB3	41:n:113:ALA:HB3	1.88	0.56
44:q:1:MET:HA	44:q:42:ALA:O	2.04	0.56
48:u:4:ILE:HB	48:u:63:ILE:HA	1.87	0.56
7:A:1015:G:N2	7:A:1220:A:N3	2.54	0.56
7:A:1442:A:N6	7:A:1461:C:O2	2.39	0.56
9:C:82:GLU:HG3	9:C:83:ASP:H	1.69	0.56
13:G:93:PRO:HA	13:G:96:ARG:HD2	1.87	0.56
32:e:147:LEU:HB2	32:e:183:PHE:CD2	2.40	0.56
49:v:71:VAL:HA	49:v:78:LYS:HA	1.87	0.56
9:C:65:ARG:HA	9:C:100:GLN:HB2	1.88	0.56
19:M:57:ARG:HD2	19:M:57:ARG:C	2.31	0.56
19:M:91:HIS:HB2	19:M:97:VAL:HG21	1.86	0.56
28:a:1418:G:N1	28:a:1579:A:N7	2.53	0.56
7:A:198:G:H4'	7:A:199:C:OP1	2.05	0.56
7:A:1133:A:N3	7:A:1143:G:N2	2.53	0.56
10:D:13:ARG:HB3	10:D:38:PRO:HD3	1.87	0.56
10:D:32:CYS:O	10:D:33:LYS:C	2.49	0.56
18:L:99:ARG:HD3	18:L:104:CYS:SG	2.45	0.56
28:a:596:U:H2'	28:a:597:G:H8	1.71	0.56
28:a:2376:A:H61	41:n:92:PHE:HB3	1.71	0.56

Continued on next page...

For Manuscript Review

Continued from previous page...

Atom-1	Atom-2	Interatomic distance (Å)	Clash overlap (Å)
29:b:65:U:H3'	29:b:108:A:H61	1.70	0.56
33:f:4:LEU:HD21	33:f:104:ILE:HD11	1.87	0.56
5:4:42:PRO:HB3	5:4:47:LYS:HB2	1.88	0.56
7:A:608:A:H2'	7:A:609:A:H5'	1.88	0.56
7:A:1163:C:H2'	7:A:1164:G:H8	1.69	0.56
28:a:660:C:H2'	28:a:661:A:N9	2.20	0.56
28:a:1166:G:H2'	28:a:1167:C:H6	1.70	0.56
31:d:3:GLY:HA3	31:d:203:VAL:O	2.05	0.56
34:g:88:GLN:NE2	34:g:165:ALA:O	2.39	0.56
34:g:111:HIS:CE1	34:g:113:VAL:HG22	2.41	0.56
7:A:482:A:H2'	7:A:484:G:H2'	1.88	0.56
7:A:998:C:O4'	7:A:1044:A:H5'	2.05	0.56
16:J:42:LEU:HD13	16:J:71:LEU:N	2.21	0.56
22:P:8:ARG:NH2	22:P:11:ALA:O	2.31	0.56
28:a:1168:G:H2'	28:a:1169:A:C8	2.41	0.56
28:a:2740:A:C4	28:a:2741:A:C8	2.94	0.56
29:b:90:C:H2'	29:b:91:C:H6	1.70	0.56
32:e:171:ASP:OD1	32:e:171:ASP:N	2.37	0.56
33:f:13:VAL:O	33:f:17:MET:HG2	2.06	0.56
36:i:36:LEU:HD21	36:i:54:ILE:HB	1.88	0.56
7:A:652:U:O4	7:A:752:G:O2'	2.19	0.56
7:A:1229:C:H2'	7:A:1230:A:H8	1.71	0.56
16:J:57:VAL:HG22	16:J:58:ASN:H	1.70	0.56
19:M:4:ILE:HG23	19:M:5:ALA:H	1.70	0.56
31:d:33:ARG:NH2	31:d:75:ALA:O	2.38	0.56
33:f:6:ASP:HA	33:f:9:LYS:HE3	1.88	0.56
9:C:134:MET:O	9:C:138:VAL:HG23	2.06	0.56
28:a:279:A:N6	28:a:361:G:H21	2.04	0.56
28:a:938:G:C2'	28:a:939:G:H5'	2.36	0.56
28:a:1527:G:N1	28:a:1544:A:OP2	2.33	0.56
44:q:35:PHE:HD1	44:q:59:ILE:HB	1.70	0.56
7:A:1118:U:O2'	15:I:108:ALA:HB2	2.07	0.55
28:a:544:C:O3'	28:a:545:U:H2'	2.05	0.55
28:a:1026:G:H2'	28:a:1027:A:C8	2.41	0.55
34:g:22:GLN:HA	34:g:37:LEU:HD12	1.89	0.55
5:4:56:ARG:HE	25:S:65:GLU:N	2.04	0.55
7:A:501:C:H2'	7:A:502:A:H8	1.69	0.55
8:B:67:ILE:HG13	8:B:160:ALA:HB3	1.88	0.55
10:D:169:THR:O	10:D:184:ARG:NH1	2.39	0.55
19:M:12:HIS:O	19:M:14:HIS:N	2.39	0.55
20:N:45:VAL:O	20:N:49:GLN:NE2	2.39	0.55

Continued on next page...

For Manuscript Review

Continued from previous page...

Atom-1	Atom-2	Interatomic distance (Å)	Clash overlap (Å)
28:a:879:G:H2'	28:a:880:G:H8	1.70	0.55
28:a:1050:A:H2'	28:a:1051:G:C8	2.39	0.55
28:a:1863:G:C5	28:a:1864:U:C4	2.94	0.55
28:a:1864:U:H2'	28:a:1865:U:C2	2.41	0.55
28:a:1880:U:O4	28:a:1881:C:N4	2.39	0.55
28:a:2327:A:H61	28:a:2388:A:N6	2.04	0.55
31:d:136:ASN:OD1	31:d:137:SER:N	2.39	0.55
32:e:188:MET:N	32:e:188:MET:HE3	2.21	0.55
38:k:112:LEU:HB2	38:k:131:ALA:HA	1.88	0.55
46:s:69:ARG:HB2	46:s:69:ARG:HH11	1.71	0.55
7:A:143:A:H2	7:A:220:G:N1	2.01	0.55
19:M:97:VAL:HG12	19:M:109:ARG:HD3	1.88	0.55
29:b:95:U:O2'	29:b:96:G:H5'	2.06	0.55
53:z:53:LYS:HE2	53:z:53:LYS:HA	1.88	0.55
12:F:62:MET:HE2	12:F:62:MET:HA	1.89	0.55
13:G:138:ARG:HA	13:G:141:VAL:HB	1.87	0.55
16:J:26:VAL:HG12	16:J:30:LYS:HD3	1.88	0.55
28:a:1166:G:H2'	28:a:1167:C:O4'	2.06	0.55
28:a:2318:G:O2'	28:a:2321:U:O4	2.24	0.55
28:a:2382:G:H2'	28:a:2382:G:OP2	2.07	0.55
28:a:2739:U:H3	28:a:2764:A:H62	0.63	0.55
34:g:152:ARG:O	34:g:162:VAL:HG22	2.07	0.55
4:3:30:GLU:HG3	4:3:32:LYS:H	1.70	0.55
7:A:194:C:C2'	7:A:195:A:H4'	2.36	0.55
7:A:1125:U:H5'	16:J:37:ARG:NH1	2.19	0.55
7:A:1372:G:OP1	15:I:14:SER:HB3	2.07	0.55
16:J:27:GLU:HA	16:J:30:LYS:HE2	1.88	0.55
16:J:39:PRO:HB3	16:J:74:VAL:HG22	1.89	0.55
19:M:81:MET:HG3	19:M:81:MET:O	2.07	0.55
7:A:448:A:O2'	7:A:450:G:OP2	2.16	0.55
8:B:32:PHE:HB2	8:B:42:ASN:HB3	1.89	0.55
16:J:7:ARG:HB2	16:J:73:LEU:HD11	1.88	0.55
28:a:2373:G:H2'	28:a:2374:C:C6	2.42	0.55
29:b:68:C:H3'	29:b:69:G:H8	1.72	0.55
32:e:48:THR:OG1	32:e:51:GLU:OE1	2.25	0.55
34:g:144:VAL:O	34:g:148:LEU:HG	2.07	0.55
36:i:95:ARG:HE	36:i:96:ARG:HH12	1.55	0.55
7:A:83:C:H2'	7:A:88:U:H3	1.71	0.55
7:A:451:A:N6	7:A:480:U:O2	2.40	0.55
7:A:1143:G:H3'	7:A:1144:G:H8	1.72	0.55
10:D:124:MET:SD	10:D:127:GLY:HA2	2.47	0.55

Continued on next page...

For Manuscript Review

Continued from previous page...

Atom-1	Atom-2	Interatomic distance (Å)	Clash overlap (Å)
11:E:115:LEU:HD21	11:E:137:VAL:HG12	1.88	0.55
13:G:138:ARG:HD3	13:G:139:GLU:N	2.22	0.55
28:a:1733:G:H2'	28:a:1734:G:H8	1.71	0.55
28:a:1853:A:H2'	28:a:1854:A:C8	2.42	0.55
28:a:2043:C:N4	28:a:2044:C:C2	2.75	0.55
28:a:2741:A:C5	28:a:2742:G:C5	2.95	0.55
29:b:86:G:H21	29:b:87:U:HO2'	1.54	0.55
7:A:201:G:H22	7:A:216:U:H3	1.54	0.55
7:A:602:A:H2'	7:A:603:U:H6	1.71	0.55
7:A:613:C:H2'	7:A:614:U:C6	2.42	0.55
7:A:1518:G:O2'	7:A:1519:MA6:OP1	2.24	0.55
22:P:67:ILE:HG23	22:P:71:VAL:HB	1.89	0.55
28:a:1026:G:O2'	28:a:1027:A:O4'	2.14	0.55
28:a:2297:A:P	28:a:2297:A:H8	2.29	0.55
29:b:76:G:H22	29:b:100:G:H1	1.55	0.55
31:d:32:ASN:O	31:d:95:SER:HA	2.07	0.55
34:g:44:LYS:HB3	34:g:50:LEU:HD22	1.89	0.55
7:A:344:A:H5"	7:A:345:C:H5	1.72	0.55
7:A:1253:A:H3'	7:A:1254:G:C8	2.42	0.55
24:R:48:ARG:HB2	24:R:51:TYR:CD2	2.42	0.55
28:a:282:A:N6	28:a:359:G:O6	2.40	0.55
28:a:596:U:C2	28:a:597:G:N7	2.75	0.55
43:p:65:ILE:HD11	43:p:106:PHE:CE1	2.41	0.55
7:A:1228:A:OP2	19:M:95:LEU:HD11	2.06	0.55
7:A:1331:U:O2'	7:A:1332:G:O4'	2.20	0.55
24:R:71:THR:OG1	24:R:72:ASP:N	2.39	0.55
28:a:2745:C:H2'	28:a:2746:U:C6	2.41	0.55
31:d:37:VAL:H	31:d:92:VAL:CG2	2.18	0.55
34:g:152:ARG:HD3	34:g:161:GLY:HA2	1.89	0.55
2:1:1:MET:N	2:1:1:MET:HE3	2.22	0.54
7:A:9:G:OP2	11:E:126:LYS:NZ	2.40	0.54
7:A:69:G:H22	7:A:98:G:N2	2.05	0.54
7:A:260:G:H2'	7:A:261:U:C4	2.41	0.54
27:U:16:LEU:HG	27:U:17:ARG:HH12	1.71	0.54
28:a:841:G:C5	28:a:842:U:C5	2.94	0.54
28:a:1434:A:H2'	28:a:1435:G:H8	1.71	0.54
28:a:2357:G:H2'	28:a:2359:C:H5	1.72	0.54
29:b:1:U:H2'	29:b:2:G:H8	1.71	0.54
29:b:15:A:H5"	29:b:16:G:N7	2.22	0.54
32:e:168:ASP:HB2	32:e:183:PHE:CE2	2.39	0.54
7:A:10:A:O2'	7:A:507:C:O2'	2.25	0.54

Continued on next page...

For Manuscript Review

Continued from previous page...

Atom-1	Atom-2	Interatomic distance (Å)	Clash overlap (Å)
7:A:720:C:H5'	24:R:41:PRO:HA	1.89	0.54
15:I:129:LYS:HD2	15:I:130:ARG:NH2	2.22	0.54
28:a:2332:C:H5'	49:v:43:THR:OG1	2.07	0.54
29:b:27:C:H2'	29:b:28:C:C6	2.42	0.54
29:b:99:A:H2'	29:b:100:G:C8	2.42	0.54
43:p:81:ASN:HB3	43:p:117:LEU:HD21	1.90	0.54
44:q:10:LYS:NZ	44:q:23:GLU:OE1	2.40	0.54
28:a:1126:A:H4'	28:a:1127:A:H5"	1.88	0.54
34:g:93:GLY:HA2	34:g:95:ARG:NH2	2.23	0.54
43:p:76:TYR:CZ	43:p:80:ILE:HG13	2.42	0.54
44:q:24:LYS:HA	44:q:94:THR:HG23	1.88	0.54
4:3:30:GLU:HG2	4:3:33:HIS:H	1.72	0.54
7:A:194:C:C3'	7:A:195:A:H4'	2.36	0.54
7:A:660:U:H3	7:A:745:G:H1	1.55	0.54
7:A:1106:G:O2'	9:C:169:ARG:NH2	2.38	0.54
28:a:2741:A:C4	28:a:2742:G:C8	2.96	0.54
29:b:29:A:HO2'	29:b:58:A:H61	1.51	0.54
33:f:33:LYS:HA	33:f:96:MET:HE2	1.89	0.54
41:n:35:ILE:HB	41:n:102:ARG:HH21	1.73	0.54
45:r:73:LYS:HB2	45:r:106:VAL:HB	1.89	0.54
48:u:68:LYS:HE3	48:u:70:ILE:HG12	1.89	0.54
7:A:69:G:H1	7:A:98:G:H22	1.56	0.54
16:J:5:ARG:HH21	16:J:7:ARG:HB3	1.73	0.54
28:a:2334:U:C4	41:n:16:ARG:HG2	2.42	0.54
38:k:123:ARG:HG3	38:k:143:GLU:HB2	1.88	0.54
44:q:6:GLN:HB3	44:q:37:GLU:CD	2.32	0.54
48:u:76:ASP:O	48:u:89:ILE:HD12	2.06	0.54
8:B:9:MET:O	8:B:14:VAL:HB	2.08	0.54
9:C:23:PHE:O	9:C:23:PHE:HD2	1.90	0.54
28:a:2377:A:H3'	28:a:2378:A:H8	1.72	0.54
28:a:2759:G:N2	34:g:139:GLN:OE1	2.41	0.54
44:q:5:PHE:HE1	44:q:12:HIS:HB2	1.71	0.54
44:q:6:GLN:HB3	44:q:37:GLU:OE1	2.08	0.54
52:y:31:ARG:HG2	52:y:34:HIS:HB2	1.89	0.54
28:a:2538:C:O2	28:a:2538:C:H2'	2.06	0.54
43:p:58:ARG:HG3	43:p:92:ARG:HD3	1.90	0.54
7:A:105:G:H21	7:A:379:C:H5"	1.73	0.54
8:B:16:PHE:HD1	8:B:38:VAL:HG11	1.73	0.54
28:a:2291:U:H2'	28:a:2292:U:C6	2.42	0.54
29:b:54:G:H2'	29:b:55:U:C6	2.43	0.54
38:k:135:ILE:O	38:k:139:GLY:N	2.41	0.54

Continued on next page...

For Manuscript Review

Continued from previous page...

Atom-1	Atom-2	Interatomic distance (Å)	Clash overlap (Å)
41:n:6:ALA:O	41:n:10:ARG:HB2	2.08	0.54
7:A:778:G:O2'	7:A:779:C:OP1	2.25	0.54
9:C:24:ALA:HB2	9:C:32:ASN:HD22	1.72	0.54
14:H:11:LEU:HD11	14:H:127:CYS:SG	2.48	0.54
24:R:48:ARG:O	24:R:52:GLN:N	2.32	0.54
27:U:65:ALA:HA	27:U:69:ARG:HG2	1.90	0.54
28:a:2748:A:H1'	28:a:2757:A:N6	2.23	0.54
36:i:55:ILE:HG21	36:i:130:HIS:CE1	2.43	0.54
36:i:55:ILE:HG22	36:i:56:VAL:H	1.71	0.54
7:A:195:A:H2'	7:A:196:A:N7	2.23	0.54
8:B:88:ASP:O	8:B:89:GLN:NE2	2.41	0.54
12:F:64:VAL:HB	12:F:71:ILE:HD11	1.90	0.54
15:I:72:ILE:HD12	15:I:73:SER:N	2.23	0.54
28:a:2636:C:OP1	31:d:81:GLU:HB2	2.07	0.54
37:j:88:ASN:ND2	37:j:91:SER:OG	2.35	0.54
7:A:1115:U:N3	7:A:1186:G:O6	2.19	0.53
7:A:1228:A:C8	25:S:84:ALA:HB2	2.43	0.53
22:P:5:ARG:NH2	22:P:23:ASP:O	2.41	0.53
28:a:1023:U:H2'	28:a:1024:G:C5'	2.38	0.53
28:a:2044:C:C2	28:a:2045:C:C6	2.96	0.53
28:a:2324:U:O2	28:a:2331:G:C6	2.61	0.53
29:b:17:C:H2'	29:b:18:G:H8	1.72	0.53
43:p:76:TYR:CE2	43:p:80:ILE:HG13	2.42	0.53
4:3:17:VAL:O	4:3:18:LYS:HD3	2.08	0.53
7:A:166:U:H2'	7:A:167:G:H8	1.72	0.53
9:C:167:TRP:HZ3	9:C:169:ARG:HG3	1.73	0.53
19:M:98:ARG:HD3	19:M:99:GLY:H	1.74	0.53
21:O:72:ARG:NH2	21:O:73:LYS:HA	2.23	0.53
28:a:2529:G:H5'	34:g:175:LYS:HG3	1.90	0.53
31:d:34:VAL:HG13	31:d:50:VAL:HG12	1.89	0.53
31:d:99:GLU:OE1	31:d:99:GLU:N	2.22	0.53
47:t:17:LYS:HG3	47:t:18:ASP:OD2	2.09	0.53
4:3:30:GLU:OE1	4:3:32:LYS:HB2	2.09	0.53
5:4:37:CYS:HB2	5:4:40:CYS:H	1.73	0.53
21:O:24:SER:O	21:O:28:GLN:HG3	2.08	0.53
28:a:1020:A:N6	28:a:1141:U:N1	2.51	0.53
28:a:2331:G:O2'	49:v:42:GLY:HA2	2.08	0.53
29:b:23:G:N7	29:b:56:G:O2'	2.42	0.53
29:b:86:G:N2	29:b:87:U:O2'	2.39	0.53
29:b:94:A:P	48:u:19:ARG:HE	2.30	0.53
33:f:50:LEU:HD21	33:f:67:ILE:HG21	1.90	0.53

Continued on next page...

For Manuscript Review

Continued from previous page...

Atom-1	Atom-2	Interatomic distance (Å)	Clash overlap (Å)
38:k:108:ALA:HB3	38:k:125:LEU:HD13	1.90	0.53
3:2:42:ARG:HH12	28:a:2382:G:N2	2.07	0.53
7:A:73:C:H42	7:A:96:U:H3	0.62	0.53
7:A:196:A:H1'	7:A:221:C:O2'	2.08	0.53
10:D:145:ILE:HG22	10:D:146:ARG:N	2.23	0.53
17:K:89:PRO:HG3	27:U:32:VAL:HG11	1.89	0.53
23:Q:42:THR:OG1	23:Q:43:LYS:N	2.41	0.53
28:a:85:G:C6	28:a:86:G:C2	2.95	0.53
7:A:601:G:H1'	7:A:602:A:H5'	1.89	0.53
7:A:1133:A:HO2'	7:A:1143:G:H1	1.56	0.53
9:C:131:ARG:NH1	9:C:131:ARG:HB3	2.24	0.53
10:D:148:LYS:HG3	10:D:150:LYS:CG	2.39	0.53
12:F:9:MET:HE1	12:F:86:ARG:HD2	1.89	0.53
16:J:35:GLN:NE2	16:J:78:GLU:HB3	2.23	0.53
28:a:2303:G:H1'	33:f:123:ASP:OD1	2.08	0.53
45:r:4:ILE:HG12	45:r:106:VAL:HG22	1.89	0.53
52:y:7:ILE:HD11	52:y:48:ILE:HD11	1.91	0.53
7:A:6:G:H4'	7:A:298:A:H4'	1.90	0.53
7:A:61:G:H22	7:A:105:G:H22	1.57	0.53
7:A:973:G:H3'	7:A:974:A:H5"	1.90	0.53
17:K:53:ARG:HD3	17:K:54:GLY:H	1.73	0.53
19:M:98:ARG:HD2	19:M:100:GLN:CD	2.33	0.53
20:N:36:ALA:HB3	20:N:41:ARG:HH12	1.72	0.53
24:R:53:ARG:O	24:R:57:ARG:HG2	2.08	0.53
28:a:638:G:H2'	28:a:639:U:H6	1.73	0.53
28:a:643:A:H61	28:a:2370:G:C4'	2.20	0.53
28:a:1033:U:O2'	28:a:1034:G:H5"	2.08	0.53
30:c:17:VAL:HB	30:c:204:VAL:HG13	1.90	0.53
41:n:30:ARG:HA	41:n:35:ILE:HG13	1.91	0.53
7:A:472:U:H2'	7:A:473:A:C8	2.44	0.53
7:A:1086:U:H3	7:A:1099:G:H22	1.54	0.53
7:A:1180:A:O2'	7:A:1181:A:OP1	2.26	0.53
13:G:72:THR:O	13:G:72:THR:OG1	2.25	0.53
15:I:42:GLU:O	15:I:46:MET:HG2	2.08	0.53
28:a:358:U:H2'	28:a:359:G:H8	1.73	0.53
28:a:919:U:OP1	29:b:97:C:O2'	2.25	0.53
28:a:2523:G:O2'	28:a:2764:A:H5"	2.09	0.53
39:l:110:GLU:OE1	39:l:114:ARG:HG3	2.09	0.53
41:n:25:ARG:O	41:n:40:ILE:N	2.28	0.53
7:A:326:G:O2'	7:A:327:A:OP1	2.19	0.53
7:A:1086:U:H3	7:A:1099:G:H1	1.55	0.53

Continued on next page...

For Manuscript Review

Continued from previous page...

Atom-1	Atom-2	Interatomic distance (Å)	Clash overlap (Å)
12:F:5:GLU:HA	12:F:62:MET:O	2.08	0.53
28:a:2330:G:C2	28:a:2331:G:H1'	2.44	0.53
28:a:2526:G:O6	28:a:2538:C:N4	2.42	0.53
39:l:118:LYS:HA	39:l:121:ALA:HB3	1.91	0.53
41:n:11:ALA:O	41:n:14:ALA:N	2.42	0.53
4:3:26:ILE:HA	4:3:34:LYS:HG2	1.91	0.53
7:A:437:U:P	10:D:154:ARG:HH12	2.32	0.53
7:A:674:G:H2'	7:A:675:A:H8	1.74	0.53
7:A:1149:U:O2'	7:A:1150:C:H6	1.92	0.53
8:B:16:PHE:O	8:B:38:VAL:HG13	2.09	0.53
15:I:12:ARG:HG3	15:I:13:LYS:H	1.74	0.53
28:a:1223:G:OP1	44:q:68:ARG:NH1	2.41	0.53
28:a:1746:A:H2'	28:a:1747:U:C6	2.44	0.53
28:a:1029:A:N6	28:a:1125:G:H1'	2.22	0.53
32:e:139:LYS:HD2	32:e:139:LYS:C	2.34	0.53
34:g:71:LEU:O	34:g:75:MET:HG3	2.09	0.53
49:v:43:THR:HB	49:v:46:HIS:CE1	2.44	0.53
49:v:71:VAL:HG12	49:v:78:LYS:HG2	1.89	0.53
4:3:1:MET:O	28:a:2538:C:O2'	2.19	0.52
7:A:597:G:H2'	7:A:598:U:H5'	1.90	0.52
7:A:944:G:N1	7:A:1339:G:OP2	2.36	0.52
7:A:1127:G:N7	16:J:7:ARG:NH2	2.56	0.52
14:H:65:TYR:HB3	14:H:69:LYS:HA	1.91	0.52
28:a:997:G:OP1	43:p:91:ASP:HB2	2.09	0.52
28:a:1007:C:OP1	36:i:37:ARG:NH1	2.42	0.52
28:a:2315:G:O2'	33:f:125:ARG:HD3	2.10	0.52
28:a:2471:A:N6	28:a:2480:C:C2	2.78	0.52
32:e:120:VAL:HA	32:e:188:MET:SD	2.49	0.52
37:j:4:GLU:HG2	37:j:23:LYS:HA	1.91	0.52
7:A:105:G:H21	7:A:379:C:C5'	2.21	0.52
8:B:151:ILE:HG23	8:B:154:MET:SD	2.48	0.52
8:B:191:SER:OG	8:B:192:ASP:N	2.40	0.52
11:E:80:THR:HG22	11:E:122:ASN:HB2	1.91	0.52
11:E:90:THR:HG22	11:E:91:GLY:N	2.25	0.52
14:H:74:SER:H	14:H:130:ALA:HB2	1.74	0.52
19:M:5:ALA:HB1	19:M:9:ILE:HB	1.91	0.52
28:a:2377:A:H3'	28:a:2378:A:C8	2.44	0.52
29:b:90:C:C2	29:b:91:C:C5	2.97	0.52
48:u:30:ILE:HG23	48:u:38:LEU:O	2.09	0.52
7:A:259:G:H2'	7:A:260:G:H4'	1.90	0.52
22:P:41:PRO:HG2	22:P:45:GLU:OE1	2.10	0.52

Continued on next page...

For Manuscript Review

Continued from previous page...

Atom-1	Atom-2	Interatomic distance (Å)	Clash overlap (Å)
28:a:599:A:H5"	38:k:9:ALA:HB3	1.91	0.52
28:a:1469:A:H2'	28:a:1470:A:C8	2.45	0.52
28:a:2043:C:C4	28:a:2044:C:C2	2.98	0.52
28:a:2741:A:H62	28:a:2763:G:N2	2.07	0.52
34:g:156:PRO:HB2	34:g:172:LYS:HE2	1.89	0.52
34:g:174:ALA:O	34:g:176:LYS:NZ	2.42	0.52
13:G:143:ARG:O	13:G:147:ALA:HB3	2.10	0.52
28:a:840:C:H2'	28:a:841:G:C8	2.45	0.52
28:a:2071:A:H2'	28:a:2072:C:C6	2.45	0.52
31:d:80:TRP:HB3	31:d:202:ILE:HD11	1.91	0.52
33:f:35:THR:O	33:f:155:THR:N	2.42	0.52
40:m:22:ARG:HG3	40:m:70:THR:HA	1.90	0.52
7:A:402:G:OP1	10:D:71:GLN:NE2	2.32	0.52
7:A:608:A:C2'	7:A:609:A:H5'	2.40	0.52
7:A:718:A:H2	24:R:38:LYS:HZ3	1.58	0.52
22:P:71:VAL:O	22:P:75:ILE:HG13	2.09	0.52
25:S:12:ASP:HB3	25:S:16:LEU:HG	1.90	0.52
32:e:16:GLU:HA	32:e:16:GLU:OE2	2.10	0.52
36:i:16:TYR:O	36:i:54:ILE:HA	2.09	0.52
40:m:106:ASP:OD1	40:m:106:ASP:N	2.42	0.52
7:A:1311:G:H3'	7:A:1312:A:H5"	1.92	0.52
12:F:19:PRO:O	12:F:22:ILE:HG23	2.10	0.52
14:H:41:LYS:NZ	14:H:47:GLU:O	2.42	0.52
19:M:13:LYS:HE3	19:M:15:ALA:HB2	1.91	0.52
28:a:1020:A:C6	28:a:1141:U:C4	2.96	0.52
28:a:1040:A:H4'	48:u:49:ASN:ND2	2.24	0.52
28:a:2356:U:C2	28:a:2357:G:C8	2.97	0.52
28:a:2471:A:N6	28:a:2480:C:O2	2.43	0.52
28:a:2526:G:H2'	28:a:2526:G:N3	2.24	0.52
36:i:54:ILE:O	36:i:122:LEU:HA	2.09	0.52
44:q:2:TYR:CD1	44:q:13:ARG:HD2	2.44	0.52
7:A:501:C:OP1	18:L:114:ARG:NH2	2.35	0.52
7:A:1156:A:O3'	7:A:1157:G:H4'	2.09	0.52
7:A:1440:G:H3'	7:A:1441:U:C6	2.44	0.52
8:B:197:ASP:OD1	8:B:197:ASP:N	2.42	0.52
9:C:55:ILE:HD12	9:C:55:ILE:O	2.10	0.52
28:a:1021:A:C3'	28:a:1022:G:H4'	2.39	0.52
28:a:2479:U:C5	28:a:2480:C:C6	2.97	0.52
32:e:124:PHE:CD2	32:e:187:VAL:HG11	2.45	0.52
43:p:91:ASP:OD2	44:q:11:GLN:NE2	2.42	0.52
51:x:34:SER:O	51:x:34:SER:OG	2.24	0.52

Continued on next page...

For Manuscript Review

Continued from previous page...

Atom-1	Atom-2	Interatomic distance (Å)	Clash overlap (Å)
3:2:42:ARG:HD3	28:a:2349:G:N7	2.25	0.52
7:A:458:U:N3	7:A:475:U:O2	2.42	0.52
7:A:983:A:O2'	7:A:1049:U:OP2	2.26	0.52
7:A:1525:G:H2'	7:A:1526:A:C8	2.43	0.52
12:F:12:PRO:O	12:F:44:ARG:NH2	2.43	0.52
17:K:15:GLN:NE2	17:K:77:TYR:O	2.43	0.52
26:T:33:LYS:HZ2	26:T:37:ALA:HB2	1.74	0.52
26:T:33:LYS:NZ	26:T:37:ALA:HB2	2.25	0.52
28:a:282:A:H2'	28:a:283:G:H8	1.74	0.52
28:a:627:A:H62	38:k:112:LEU:HB3	1.74	0.52
7:A:108:G:H22	7:A:332:G:H5"	1.74	0.52
7:A:1002:A:H2'	7:A:1041:G:C6	2.45	0.52
7:A:1320:A:H4'	7:A:1321:C:OP2	2.09	0.52
20:N:31:ILE:HA	20:N:41:ARG:HH21	1.75	0.52
29:b:64:G:H2'	29:b:65:U:O4'	2.10	0.52
31:d:196:ALA:O	31:d:199:SER:OG	2.27	0.52
34:g:54:PRO:HB3	34:g:61:GLY:C	2.35	0.52
38:k:109:LYS:HG2	38:k:128:THR:HG23	1.91	0.52
49:v:66:LYS:NZ	49:v:66:LYS:HB3	2.24	0.52
7:A:674:G:H2'	7:A:675:A:C8	2.45	0.52
7:A:891:U:O2'	7:A:892:A:H8	1.92	0.52
7:A:1181:A:OP2	15:I:99:ARG:NH2	2.39	0.52
9:C:40:ARG:HG2	9:C:55:ILE:HD11	1.92	0.52
9:C:117:ALA:HB1	9:C:187:SER:HB2	1.92	0.52
10:D:19:LEU:HG	10:D:64:ILE:CD1	2.40	0.52
12:F:8:PHE:CE1	12:F:60:VAL:HG12	2.45	0.52
15:I:22:LYS:HB2	15:I:62:ASP:OD2	2.10	0.52
16:J:53:ILE:O	16:J:54:SER:HB3	2.10	0.52
27:U:54:LYS:O	27:U:58:LYS:HG2	2.09	0.52
28:a:290:U:H3	28:a:350:G:H1	1.58	0.52
28:a:841:G:H2'	28:a:842:U:H6	1.73	0.52
28:a:2528:U:C2	28:a:2536:G:C2	2.98	0.52
28:a:2625:G:H1	28:a:2777:G:H1	1.57	0.52
32:e:147:LEU:HB2	32:e:183:PHE:HD2	1.75	0.52
38:k:127:VAL:HG21	38:k:142:ILE:HD13	1.92	0.52
7:A:255:G:OP1	23:Q:71:LYS:NZ	2.42	0.51
28:a:1028:A:H3'	28:a:1029:A:H8	1.75	0.51
28:a:1434:A:H2'	28:a:1435:G:C8	2.44	0.51
39:l:98:PRO:C	48:u:82:TYR:HB3	2.34	0.51
48:u:57:TYR:HE2	48:u:77:VAL:HB	1.75	0.51
7:A:299:G:H1	7:A:566:A:H61	1.56	0.51

Continued on next page...

For Manuscript Review

Continued from previous page...

Atom-1	Atom-2	Interatomic distance (Å)	Clash overlap (Å)
7:A:1009:A:H61	7:A:1020:U:H3	1.57	0.51
7:A:1066:C:O2'	7:A:1067:A:O5'	2.23	0.51
8:B:43:LEU:HA	8:B:46:THR:HB	1.92	0.51
18:L:110:ARG:NH1	18:L:112:GLN:O	2.43	0.51
28:a:586:A:H5'	32:e:84:THR:HG21	1.91	0.51
28:a:1873:G:H2'	28:a:1874:C:C6	2.45	0.51
31:d:17:GLU:CD	31:d:17:GLU:H	2.17	0.51
31:d:50:VAL:O	31:d:80:TRP:N	2.42	0.51
32:e:119:ILE:O	32:e:188:MET:HE1	2.10	0.51
34:g:63:ALA:O	34:g:67:THR:OG1	2.28	0.51
34:g:101:ASN:OD1	34:g:116:GLN:NE2	2.43	0.51
36:i:15:TRP:HZ3	36:i:55:ILE:HD11	1.74	0.51
36:i:36:LEU:HD11	36:i:54:ILE:HG21	1.91	0.51
7:A:131:A:H3'	7:A:132:U:H5"	1.92	0.51
7:A:415:A:H2'	7:A:416:G:H8	1.75	0.51
10:D:10:LYS:HD2	10:D:38:PRO:HB3	1.93	0.51
22:P:19:VAL:HB	22:P:37:GLY:HA2	1.92	0.51
28:a:1589:U:H2'	28:a:1590:A:H8	1.74	0.51
28:a:2472:G:H5"	28:a:2473:U:H5"	1.92	0.51
31:d:33:ARG:O	31:d:51:THR:HG22	2.11	0.51
34:g:16:ASP:HB2	34:g:27:LYS:HB3	1.92	0.51
34:g:105:LEU:O	34:g:112:PRO:HA	2.11	0.51
43:p:76:TYR:O	43:p:80:ILE:HG12	2.11	0.51
48:u:80:HIS:N	48:u:85:LYS:O	2.43	0.51
7:A:1024:G:H2'	7:A:1025:U:H4'	1.93	0.51
9:C:45:LYS:NZ	9:C:45:LYS:HB3	2.25	0.51
28:a:1418:G:N2	28:a:1580:A:C5	2.78	0.51
29:b:23:G:H2'	29:b:24:G:N7	2.25	0.51
30:c:270:ARG:HG2	30:c:271:ARG:N	2.25	0.51
32:e:151:GLY:N	32:e:192:ALA:HB2	2.25	0.51
34:g:156:PRO:HB2	34:g:172:LYS:CE	2.40	0.51
18:L:75:GLN:OE1	18:L:75:GLN:N	2.43	0.51
23:Q:7:THR:OG1	23:Q:61:ILE:O	2.23	0.51
33:f:147:ASP:OD1	33:f:147:ASP:N	2.43	0.51
34:g:129:THR:HG23	34:g:130:GLU:HG3	1.92	0.51
36:i:54:ILE:HD13	36:i:122:LEU:HD13	1.92	0.51
44:q:25:LEU:H	44:q:94:THR:HG21	1.76	0.51
7:A:458:U:H2'	7:A:459:A:H8	1.76	0.51
7:A:500:G:H2'	7:A:501:C:C6	2.45	0.51
7:A:1014:A:N1	25:S:34:TRP:HB3	2.26	0.51
8:B:112:LYS:HA	8:B:115:LYS:HG2	1.93	0.51

Continued on next page...

For Manuscript Review

Continued from previous page...

Atom-1	Atom-2	Interatomic distance (Å)	Clash overlap (Å)
9:C:25:ASN:O	9:C:27:LYS:N	2.44	0.51
9:C:64:ILE:O	9:C:66:VAL:HG23	2.11	0.51
9:C:135:LYS:O	9:C:139:GLN:HG3	2.10	0.51
10:D:128:ARG:HB3	10:D:128:ARG:CZ	2.38	0.51
19:M:85:CYS:SG	19:M:86:TYR:N	2.84	0.51
25:S:5:LEU:HD23	25:S:5:LEU:O	2.11	0.51
28:a:1028:A:N6	28:a:1126:A:H62	2.08	0.51
28:a:1028:A:H2	28:a:2487:G:H4'	1.73	0.51
33:f:14:LYS:O	33:f:18:THR:OG1	2.28	0.51
34:g:101:ASN:O	34:g:117:LEU:N	2.43	0.51
40:m:32:GLU:OE2	40:m:115:LEU:HD12	2.10	0.51
41:n:83:LEU:HD12	41:n:88:LYS:HD2	1.92	0.51
5:4:56:ARG:HD3	25:S:67:VAL:HB	1.93	0.51
7:A:596:A:H62	7:A:644:U:H3	1.59	0.51
7:A:1444:U:H2'	7:A:1445:U:O4'	2.11	0.51
8:B:96:TRP:HA	8:B:100:MET:SD	2.51	0.51
9:C:5:VAL:HG11	9:C:10:ILE:HG13	1.92	0.51
29:b:48:U:H2'	29:b:49:C:C6	2.45	0.51
33:f:24:SER:HB3	33:f:27:GLN:HG3	1.92	0.51
33:f:126:GLY:O	33:f:158:THR:OG1	2.16	0.51
34:g:48:ASN:OD1	34:g:48:ASN:N	2.43	0.51
38:k:78:ARG:NE	38:k:113:ALA:HB3	2.24	0.51
38:k:112:LEU:HB2	38:k:131:ALA:CA	2.41	0.51
38:k:129:LYS:HA	38:k:132:ARG:CG	2.41	0.51
48:u:20:LEU:HA	48:u:23:ALA:HB3	1.91	0.51
48:u:49:ASN:C	48:u:50:MET:HE2	2.36	0.51
7:A:413:A:H2'	7:A:413:A:N3	2.26	0.51
9:C:40:ARG:CZ	20:N:92:GLU:HG3	2.41	0.51
18:L:21:VAL:HB	18:L:24:LEU:HD23	1.93	0.51
20:N:49:GLN:HE21	25:S:10:PHE:HE2	1.59	0.51
27:U:11:PRO:HB2	27:U:13:ASP:OD1	2.11	0.51
28:a:85:G:C2'	28:a:86:G:H4'	2.36	0.51
28:a:548:G:C5	28:a:549:G:C6	2.99	0.51
28:a:644:A:N1	28:a:2369:A:O2'	2.44	0.51
29:b:92:C:H2'	29:b:93:C:C6	2.45	0.51
30:c:107:PRO:HD2	30:c:110:LEU:HD22	1.93	0.51
32:e:124:PHE:CE2	32:e:138:LEU:HD11	2.46	0.51
36:i:45:THR:HG22	36:i:46:PRO:HD2	1.92	0.51
1:0:13:SER:HA	1:0:49:TYR:HA	1.91	0.51
7:A:73:C:N4	7:A:96:U:C4	2.78	0.51
15:I:85:ARG:O	15:I:88:MET:HG3	2.11	0.51

Continued on next page...

For Manuscript Review

Continued from previous page...

Atom-1	Atom-2	Interatomic distance (Å)	Clash overlap (Å)
29:b:78:A:H2'	29:b:79:G:O4'	2.10	0.51
34:g:44:LYS:HD3	34:g:50:LEU:HA	1.93	0.51
38:k:129:LYS:HA	38:k:132:ARG:HG2	1.93	0.51
51:x:20:ASN:O	51:x:24:GLU:HG2	2.10	0.51
3:2:25:LYS:HD3	38:k:62:PRO:HG2	1.92	0.51
7:A:1049:U:H4'	7:A:1050:G:H4'	1.93	0.51
8:B:83:ALA:HB3	8:B:214:LEU:HD12	1.92	0.51
9:C:2:GLY:O	9:C:4:LYS:N	2.43	0.51
22:P:6:LEU:HG	22:P:17:TYR:HB3	1.93	0.51
25:S:66:MET:HG3	25:S:74:PHE:CZ	2.46	0.51
28:a:279:A:H62	28:a:361:G:H21	1.58	0.51
28:a:2043:C:C5	28:a:2044:C:C5	2.99	0.51
5:4:18:CYS:HB3	5:4:40:CYS:HB3	1.93	0.50
7:A:447:G:N1	7:A:486:U:OP2	2.44	0.50
7:A:600:A:N6	7:A:639:G:O6	2.44	0.50
7:A:1000:A:H2'	7:A:1001:G:C8	2.46	0.50
10:D:168:PRO:O	10:D:170:TRP:N	2.36	0.50
13:G:122:ASN:OD1	13:G:123:GLU:N	2.44	0.50
25:S:21:LYS:O	25:S:21:LYS:HD3	2.11	0.50
28:a:2789:C:N4	28:a:2893:A:C5	2.79	0.50
29:b:63:C:H2'	29:b:64:G:C8	2.42	0.50
29:b:65:U:H3'	29:b:108:A:N6	2.26	0.50
31:d:1:MET:SD	31:d:2:ILE:HG22	2.51	0.50
33:f:70:ALA:O	33:f:82:GLY:N	2.37	0.50
34:g:41:VAL:O	34:g:55:ARG:NH2	2.44	0.50
36:i:138:GLN:N	36:i:138:GLN:OE1	2.44	0.50
37:j:64:ARG:NH2	37:j:100:PHE:O	2.45	0.50
43:p:98:ILE:HG21	43:p:109:LEU:HD12	1.93	0.50
44:q:14:VAL:HB	44:q:20:VAL:HG22	1.93	0.50
48:u:71:LYS:HB3	48:u:73:LYS:HZ1	1.76	0.50
7:A:1285:C:H5"	7:A:1286:A:H2'	1.93	0.50
7:A:1370:C:HO2'	7:A:1371:G:P	2.34	0.50
8:B:19:GLN:OE1	8:B:19:GLN:N	2.33	0.50
24:R:9:LYS:N	24:R:46:GLY:HA3	2.26	0.50
28:a:85:G:C8	28:a:86:G:H1'	2.46	0.50
28:a:661:A:C5	28:a:662:G:C8	3.00	0.50
28:a:879:G:H2'	28:a:880:G:C8	2.46	0.50
30:c:240:PHE:O	30:c:242:LYS:HD2	2.11	0.50
1:0:6:ARG:NH1	28:a:2285:C:OP2	2.40	0.50
7:A:376:G:H2'	7:A:377:G:C8	2.45	0.50
7:A:458:U:H2'	7:A:459:A:C8	2.46	0.50

Continued on next page...

For Manuscript Review

Continued from previous page...

Atom-1	Atom-2	Interatomic distance (Å)	Clash overlap (Å)
7:A:1321:C:H41	25:S:36:ARG:HH11	1.59	0.50
10:D:189:SER:OG	10:D:194:ASP:O	2.29	0.50
28:a:424:G:C6	28:a:425:G:N3	2.79	0.50
29:b:77:U:C4	29:b:99:A:N7	2.76	0.50
32:e:24:ASN:HB3	32:e:27:LEU:HB3	1.93	0.50
43:p:64:ARG:NH1	43:p:100:VAL:HG21	2.27	0.50
1:0:20:PHE:CZ	28:a:2419:U:H1'	2.47	0.50
7:A:465:A:H2'	7:A:465:A:N3	2.26	0.50
7:A:1122:U:H1'	7:A:1123:G:C8	2.47	0.50
15:I:129:LYS:O	15:I:130:ARG:HG3	2.11	0.50
25:S:41:PHE:O	25:S:44:MET:HG2	2.11	0.50
28:a:1167:C:H3'	28:a:1168:G:H8	1.76	0.50
28:a:1720:U:H2'	28:a:1721:G:O4'	2.12	0.50
28:a:1733:G:H2'	28:a:1734:G:C8	2.47	0.50
28:a:2351:G:H22	28:a:2366:A:H5"	1.76	0.50
28:a:2744:G:H21	34:g:143:GLN:NE2	2.07	0.50
36:i:15:TRP:HB3	36:i:137:PRO:HB3	1.93	0.50
36:i:134:ALA:C	36:i:136:GLN:H	2.19	0.50
43:p:61:TRP:HB3	43:p:92:ARG:O	2.11	0.50
47:t:81:ASP:OD1	47:t:82:ARG:N	2.44	0.50
7:A:411:A:H2'	7:A:412:C:C4'	2.41	0.50
7:A:486:U:H2'	7:A:487:A:H8	1.76	0.50
7:A:748:U:O4	7:A:749:A:N6	2.45	0.50
13:G:30:LEU:HD12	13:G:39:ALA:HB1	1.94	0.50
15:I:14:SER:O	15:I:14:SER:OG	2.27	0.50
16:J:40:ILE:HD11	16:J:73:LEU:HB3	1.93	0.50
19:M:34:LEU:HD11	19:M:39:ILE:HB	1.92	0.50
28:a:840:C:C2	28:a:841:G:N7	2.79	0.50
28:a:1020:A:H1'	28:a:1021:A:C2	2.47	0.50
28:a:1433:A:H2'	28:a:1434:A:C8	2.46	0.50
28:a:2304:G:OP1	33:f:121:SER:OG	2.30	0.50
7:A:231:U:H1'	7:A:262:A:H61	1.76	0.50
17:K:50:SER:OG	17:K:69:ARG:NH1	2.44	0.50
20:N:47:LYS:HD2	20:N:47:LYS:C	2.37	0.50
28:a:1042:G:H2'	28:a:1043:C:C6	2.47	0.50
28:a:1183:U:P	52:y:31:ARG:NH2	2.83	0.50
28:a:2335:A:H5'	41:n:13:ARG:NH2	2.27	0.50
28:a:2546:U:H5"	28:a:2547:A:O5'	2.12	0.50
29:b:96:G:H2'	29:b:97:C:O4'	2.11	0.50
32:e:124:PHE:HB3	32:e:148:ILE:HD13	1.92	0.50
43:p:79:PHE:HZ	43:p:109:LEU:HB3	1.77	0.50

Continued on next page...

For Manuscript Review

Continued from previous page...

Atom-1	Atom-2	Interatomic distance (Å)	Clash overlap (Å)
51:x:12:GLU:HG2	51:x:13:GLU:OE1	2.11	0.50
10:D:60:LYS:O	10:D:64:ILE:HD13	2.11	0.50
28:a:2461:A:H2'	28:a:2462:C:C6	2.46	0.50
31:d:37:VAL:HG13	31:d:48:ILE:HG22	1.92	0.50
46:s:3:ARG:HH12	46:s:5:GLU:HG2	1.77	0.50
7:A:66:G:O6	7:A:102:G:C2	2.65	0.50
7:A:618:C:N4	7:A:621:A:OP2	2.41	0.50
7:A:1164:G:H2'	7:A:1165:G:H8	1.77	0.50
12:F:46:GLN:OE1	12:F:46:GLN:N	2.28	0.50
28:a:2324:U:H5"	28:a:2326:C:P	2.52	0.50
28:a:2472:G:N2	28:a:2479:U:O4	2.44	0.50
29:b:74:U:O2	48:u:29:ILE:HD13	2.12	0.50
29:b:92:C:P	48:u:18:ARG:HH12	2.35	0.50
41:n:15:ARG:HE	41:n:95:SER:HA	1.75	0.50
4:3:2:LYS:HE3	28:a:2478:A:H5'	1.93	0.50
7:A:967:5MC:H3'	7:A:968:A:H2'	1.94	0.50
7:A:1130:A:H2'	7:A:1131:G:C8	2.47	0.50
28:a:2741:A:C2'	28:a:2742:G:H8	2.24	0.50
7:A:849:G:H3'	7:A:850:U:H5"	1.94	0.49
8:B:32:PHE:HB3	8:B:40:ILE:HG12	1.94	0.49
10:D:104:ARG:HH11	10:D:104:ARG:HG3	1.77	0.49
28:a:640:C:H2'	28:a:641:U:C6	2.47	0.49
28:a:1126:A:H5"	56:a:6210:SPD:H92	1.94	0.49
28:a:1141:U:H4'	28:a:1142:A:O4'	2.12	0.49
7:A:91:U:H2'	7:A:92:U:H6	1.77	0.49
7:A:260:G:H2'	7:A:261:U:C5	2.47	0.49
7:A:1229:C:H2'	7:A:1230:A:C8	2.45	0.49
28:a:1026:G:C2	28:a:1027:A:C6	3.00	0.49
36:i:55:ILE:HG12	36:i:123:LYS:O	2.12	0.49
43:p:93:LYS:HD3	43:p:94:ILE:HG12	1.94	0.49
7:A:176:C:OP2	26:T:64:LYS:NZ	2.45	0.49
7:A:837:U:H3	7:A:849:G:H1	1.57	0.49
7:A:902:G:H2'	7:A:903:A:H8	1.77	0.49
7:A:1003:G:OP2	7:A:1039:C:N4	2.45	0.49
7:A:1157:G:H5'	7:A:1158:A:O3'	2.11	0.49
9:C:59:ARG:HE	9:C:64:ILE:HD13	1.76	0.49
24:R:50:LYS:HA	24:R:53:ARG:NH1	2.27	0.49
27:U:55:ARG:NH1	27:U:55:ARG:HB3	2.27	0.49
28:a:360:U:H2'	28:a:361:G:C8	2.47	0.49
28:a:424:G:C4	28:a:425:G:H1'	2.47	0.49
28:a:841:G:C6	28:a:938:G:N2	2.80	0.49

Continued on next page...

For Manuscript Review

Continued from previous page...

Atom-1	Atom-2	Interatomic distance (Å)	Clash overlap (Å)
28:a:2399:G:H2'	28:a:2400:G:C8	2.47	0.49
29:b:106:G:H2'	29:b:107:G:O4'	2.12	0.49
4:3:2:LYS:HB2	4:3:35:GLN:CG	2.42	0.49
7:A:127:G:OP1	7:A:605:U:O2'	2.25	0.49
7:A:254:G:O6	7:A:273:A:N6	2.45	0.49
7:A:337:G:H2'	7:A:338:A:C8	2.47	0.49
8:B:213:TYR:O	8:B:217:VAL:HG22	2.12	0.49
9:C:60:PRO:HB3	16:J:93:ALA:HA	1.95	0.49
12:F:8:PHE:HE2	12:F:10:VAL:HG23	1.77	0.49
19:M:98:ARG:HH11	19:M:99:GLY:H	1.60	0.49
25:S:11:ILE:CG2	25:S:38:SER:HA	2.42	0.49
28:a:603:A:H4'	28:a:604:G:C4'	2.42	0.49
28:a:1023:U:C4	28:a:1024:G:C4	3.01	0.49
28:a:2323:G:N1	28:a:2331:G:O6	2.45	0.49
28:a:2830:C:H5"	31:d:56:LYS:HE3	1.94	0.49
36:i:53:TYR:CD2	36:i:121:LYS:HA	2.46	0.49
4:3:4:ARG:HH22	4:3:10:LEU:HD11	1.77	0.49
7:A:1100:C:H5'	8:B:95:ARG:NE	2.28	0.49
8:B:128:LYS:HD2	8:B:128:LYS:N	2.27	0.49
10:D:49:SER:OG	10:D:50:ASP:N	2.44	0.49
19:M:13:LYS:HG3	19:M:15:ALA:HB2	1.94	0.49
19:M:96:PRO:HA	19:M:109:ARG:HB2	1.94	0.49
28:a:2305:U:H2'	28:a:2306:C:O4'	2.13	0.49
28:a:2362:C:C2	28:a:2363:G:N7	2.80	0.49
32:e:5:LEU:HD22	32:e:8:ALA:HB3	1.95	0.49
48:u:44:HIS:O	48:u:48:MET:HB2	2.13	0.49
7:A:502:A:OP1	18:L:115:SER:OG	2.18	0.49
7:A:1017:C:N4	20:N:53:ARG:H	2.11	0.49
7:A:1398:C:O2'	7:A:1403:4OC:OP1	2.31	0.49
12:F:10:VAL:HG13	12:F:83:ALA:HB1	1.94	0.49
17:K:45:ALA:HB3	17:K:70:CYS:HB2	1.94	0.49
23:Q:15:ASP:CG	23:Q:21:ILE:HG22	2.38	0.49
25:S:14:HIS:HD2	25:S:15:LEU:HD23	1.77	0.49
26:T:4:ILE:HG22	26:T:6:SER:H	1.76	0.49
28:a:2356:U:H2'	28:a:2357:G:H5"	1.95	0.49
34:g:88:GLN:N	34:g:163:ARG:O	2.45	0.49
36:i:43:GLU:OE1	36:i:43:GLU:N	2.45	0.49
43:p:79:PHE:CZ	43:p:106:PHE:HE1	2.30	0.49
48:u:47:VAL:HG13	48:u:56:PHE:CZ	2.48	0.49
48:u:77:VAL:HG21	48:u:79:ARG:NH1	2.27	0.49
7:A:194:C:O2'	7:A:196:A:OP1	2.24	0.49

Continued on next page...

For Manuscript Review

Continued from previous page...

Atom-1	Atom-2	Interatomic distance (Å)	Clash overlap (Å)
13:G:131:LYS:HB3	13:G:135:VAL:HG21	1.94	0.49
16:J:6:ILE:HG12	16:J:76:ILE:HG22	1.94	0.49
28:a:2333:A:N3	41:n:9:ARG:NE	2.60	0.49
28:a:2380:C:C2	28:a:2381:A:H1'	2.46	0.49
28:a:2524:G:C6	28:a:2525:G:C8	3.01	0.49
28:a:2896:C:H2'	28:a:2897:U:C6	2.48	0.49
33:f:34:ILE:HD12	33:f:96:MET:HG2	1.93	0.49
33:f:80:ARG:NE	33:f:80:ARG:HA	2.27	0.49
36:i:119:PHE:HD1	36:i:122:LEU:HD23	1.78	0.49
48:u:2:PHE:CE2	48:u:50:MET:HB3	2.47	0.49
48:u:9:ARG:HH12	48:u:17:SER:HA	1.78	0.49
50:w:20:HIS:O	50:w:20:HIS:ND1	2.41	0.49
52:y:38:ARG:NE	52:y:38:ARG:HA	2.26	0.49
7:A:22:G:HO2'	7:A:885:G:H8	1.60	0.49
7:A:108:G:H5'	7:A:109:A:H3'	1.95	0.49
7:A:373:A:H62	7:A:391:G:H1'	1.77	0.49
7:A:1280:A:H5"	16:J:9:ARG:NH2	2.27	0.49
8:B:15:HIS:HA	8:B:40:ILE:HA	1.95	0.49
11:E:126:LYS:HG2	11:E:128:TYR:CZ	2.48	0.49
13:G:59:LEU:HD12	13:G:59:LEU:O	2.13	0.49
25:S:11:ILE:HD12	25:S:12:ASP:N	2.28	0.49
28:a:749:A:N1	28:a:753:A:O2'	2.38	0.49
28:a:2043:C:N4	28:a:2044:C:N3	2.61	0.49
28:a:2591:C:H2'	28:a:2592:G:H8	1.78	0.49
49:v:23:VAL:HA	49:v:38:VAL:HG12	1.95	0.49
49:v:75:LYS:H	49:v:75:LYS:HD2	1.78	0.49
7:A:456:G:H8	7:A:477:A:C2	2.30	0.49
8:B:95:ARG:HG2	8:B:96:TRP:H	1.78	0.49
10:D:25:VAL:HG23	10:D:26:ARG:HG2	1.95	0.49
11:E:106:ILE:O	11:E:106:ILE:HG22	2.12	0.49
13:G:92:ARG:NH2	13:G:93:PRO:HG2	2.28	0.49
15:I:34:SER:OG	15:I:35:LEU:N	2.46	0.49
18:L:55:VAL:HG21	18:L:80:ILE:HD11	1.94	0.49
28:a:219:A:N3	28:a:234:U:O2'	2.43	0.49
28:a:424:G:N1	28:a:425:G:C2	2.80	0.49
28:a:629:G:N7	28:a:630:G:C4	2.80	0.49
28:a:2471:A:N7	28:a:2472:G:C8	2.81	0.49
28:a:2630:G:H1'	28:a:2894:G:C4	2.48	0.49
28:a:2646:C:H2'	28:a:2647:U:O4'	2.13	0.49
33:f:175:PHE:HB3	33:f:177:PHE:HD1	1.78	0.49
43:p:85:LYS:HD3	43:p:86:ALA:N	2.28	0.49

Continued on next page...

For Manuscript Review

Continued from previous page...

Atom-1	Atom-2	Interatomic distance (Å)	Clash overlap (Å)
48:u:80:HIS:HA	48:u:87:GLN:HG3	1.94	0.49
49:v:46:HIS:HB2	49:v:79:PHE:CE1	2.48	0.49
7:A:712:A:H2'	7:A:713:G:C8	2.48	0.49
8:B:134:ALA:O	8:B:138:THR:HG22	2.13	0.49
13:G:88:PRO:HG3	13:G:149:LYS:HD2	1.94	0.49
28:a:424:G:C2	28:a:425:G:H1'	2.46	0.49
28:a:628:G:O4'	28:a:636:G:N2	2.46	0.49
28:a:1880:U:C4	28:a:1881:C:C4	3.01	0.49
28:a:2557:G:H2'	28:a:2558:C:C6	2.48	0.49
28:a:2651:C:O2	28:a:2651:C:H2'	2.13	0.49
28:a:2673:G:N2	28:a:2674:G:O6	2.46	0.49
43:p:85:LYS:HD3	43:p:85:LYS:C	2.38	0.49
1:0:40:ASP:OD1	1:0:42:VAL:HG12	2.14	0.48
7:A:410:G:OP1	10:D:26:ARG:HD3	2.13	0.48
7:A:678:U:H2'	7:A:679:U:C6	2.48	0.48
7:A:1002:A:O2'	7:A:1003:G:N3	2.46	0.48
7:A:1231:C:H2'	7:A:1232:G:H8	1.77	0.48
8:B:72:THR:HG22	8:B:93:ASN:HA	1.94	0.48
9:C:142:MET:SD	9:C:170:GLU:HG2	2.53	0.48
16:J:42:LEU:HD13	16:J:71:LEU:H	1.78	0.48
27:U:69:ARG:O	27:U:70:LEU:HB3	2.13	0.48
28:a:703:U:H2'	28:a:704:G:O4'	2.13	0.48
28:a:1029:A:H2'	28:a:1030:C:C6	2.48	0.48
28:a:2591:C:H2'	28:a:2592:G:C8	2.48	0.48
31:d:181:ASP:OD1	31:d:184:ARG:HB2	2.13	0.48
33:f:63:GLN:HB2	33:f:95:ARG:NH2	2.28	0.48
37:j:34:GLY:N	37:j:37:ASP:OD2	2.46	0.48
4:3:30:GLU:HG2	4:3:33:HIS:N	2.27	0.48
7:A:765:G:H2'	7:A:765:G:N3	2.27	0.48
7:A:1067:A:C5	7:A:1109:C:H5"	2.48	0.48
7:A:1292:U:H2'	7:A:1293:A:H5"	1.95	0.48
9:C:23:PHE:O	9:C:23:PHE:CD2	2.65	0.48
9:C:206:GLU:O	9:C:207:ILE:HD13	2.14	0.48
25:S:19:VAL:HG21	25:S:44:MET:HE1	1.94	0.48
25:S:36:ARG:HB3	25:S:72:GLY:HA3	1.94	0.48
28:a:1022:G:N2	28:a:1141:U:H3	2.11	0.48
28:a:1548:A:H2'	28:a:1549:A:C8	2.48	0.48
28:a:1809:A:H2'	28:a:1810:A:C8	2.47	0.48
28:a:2336:A:H61	49:v:43:THR:HG21	1.78	0.48
28:a:2625:G:C6	28:a:2626:C:C4	3.01	0.48
29:b:50:A:C6	29:b:51:G:N7	2.81	0.48

Continued on next page...

For Manuscript Review

Continued from previous page...

Atom-1	Atom-2	Interatomic distance (Å)	Clash overlap (Å)
29:b:75:G:H2'	29:b:76:G:C8	2.48	0.48
32:e:5:LEU:HA	32:e:120:VAL:HG13	1.95	0.48
34:g:97:ALA:HB3	34:g:104:ASN:HB2	1.95	0.48
1:0:11:LEU:HA	1:0:51:GLU:HA	1.95	0.48
7:A:95:G:H4'	7:A:96:U:C6	2.48	0.48
7:A:721:G:H4'	7:A:722:G:O4'	2.14	0.48
7:A:974:A:H8	7:A:974:A:OP1	1.96	0.48
7:A:1321:C:H1'	7:A:1322:U:OP1	2.13	0.48
8:B:76:ALA:HA	8:B:79:ALA:HB3	1.94	0.48
8:B:94:HIS:C	8:B:94:HIS:CD2	2.92	0.48
28:a:2386:A:H2'	28:a:2387:U:C6	2.48	0.48
34:g:54:PRO:HB3	34:g:61:GLY:HA3	1.94	0.48
8:B:73:LYS:O	8:B:76:ALA:N	2.45	0.48
19:M:2:ALA:N	19:M:9:ILE:HG22	2.28	0.48
28:a:2280:G:O2'	28:a:2327:A:C2	2.65	0.48
28:a:2419:U:H2'	28:a:2420:C:H6	1.78	0.48
29:b:17:C:N4	29:b:109:A:OP2	2.37	0.48
34:g:9:VAL:HG12	34:g:10:VAL:H	1.79	0.48
48:u:29:ILE:HA	48:u:40:ILE:HG12	1.94	0.48
7:A:765:G:C2	7:A:812:G:N3	2.81	0.48
7:A:1112:C:H42	9:C:177:THR:HB	1.78	0.48
7:A:1286:A:H5'	7:A:1287:C:C5	2.49	0.48
10:D:43:ALA:O	10:D:44:ARG:C	2.56	0.48
10:D:97:ARG:O	10:D:101:VAL:HG23	2.14	0.48
13:G:70:ARG:HG2	13:G:71:PRO:O	2.13	0.48
14:H:78:VAL:HG12	14:H:79:SER:H	1.78	0.48
28:a:1800:C:OP2	30:c:182:ARG:NH1	2.34	0.48
28:a:2374:C:H2'	28:a:2375:G:C8	2.48	0.48
29:b:113:C:H2'	29:b:114:C:C6	2.49	0.48
34:g:103:ILE:O	34:g:114:ASP:HA	2.14	0.48
7:A:219:C:H2'	7:A:220:G:H8	1.78	0.48
7:A:1011:C:H42	7:A:1018:G:H1	1.62	0.48
7:A:1336:U:H5'	7:A:1337:C:H5'	1.95	0.48
12:F:9:MET:SD	12:F:86:ARG:HB3	2.54	0.48
16:J:9:ARG:HG2	16:J:73:LEU:HD13	1.96	0.48
28:a:282:A:H2'	28:a:283:G:C8	2.48	0.48
28:a:849:A:H2'	28:a:850:U:C6	2.49	0.48
28:a:1798:U:OP2	30:c:271:ARG:NH2	2.43	0.48
29:b:43:C:N4	29:b:45:A:C6	2.82	0.48
29:b:86:G:N2	29:b:87:U:HO2'	2.11	0.48
39:l:110:GLU:HA	39:l:113:ALA:HB3	1.94	0.48

Continued on next page...

For Manuscript Review

Continued from previous page...

Atom-1	Atom-2	Interatomic distance (Å)	Clash overlap (Å)
40:m:69:ARG:O	40:m:70:THR:OG1	2.27	0.48
7:A:123:C:H2'	7:A:124:C:C6	2.49	0.48
7:A:383:A:N6	7:A:455:U:OP2	2.39	0.48
28:a:595:C:H2'	28:a:596:U:C6	2.48	0.48
28:a:1043:C:H2'	28:a:1044:C:O4'	2.13	0.48
28:a:2524:G:H2'	28:a:2525:G:O4'	2.14	0.48
32:e:48:THR:HG23	32:e:86:ALA:HB3	1.95	0.48
33:f:40:VAL:O	33:f:40:VAL:HG12	2.13	0.48
41:n:23:ALA:C	41:n:42:PRO:HG3	2.38	0.48
7:A:1339:G:H2'	7:A:1340:A:C8	2.49	0.48
7:A:1457:A:H2'	7:A:1458:G:O4'	2.14	0.48
16:J:76:ILE:HD13	16:J:76:ILE:HA	1.71	0.48
28:a:2740:A:H3'	28:a:2741:A:H8	1.78	0.48
41:n:68:LYS:NZ	41:n:101:GLY:HA2	2.29	0.48
7:A:299:G:H2'	7:A:300:A:C8	2.49	0.48
7:A:373:A:C8	7:A:374:A:C8	3.01	0.48
7:A:615:G:C2'	7:A:616:G:H5'	2.43	0.48
7:A:1157:G:O5'	7:A:1158:A:H4'	2.13	0.48
7:A:1164:G:H2'	7:A:1165:G:C8	2.49	0.48
7:A:1356:A:H2'	7:A:1357:A:C8	2.46	0.48
9:C:131:ARG:HB3	9:C:131:ARG:HH11	1.79	0.48
10:D:105:MET:HE3	10:D:105:MET:HB2	1.70	0.48
26:T:47:ALA:HB1	26:T:83:ILE:HD11	1.95	0.48
27:U:4:ILE:HD13	27:U:19:PHE:CE1	2.48	0.48
28:a:2546:U:O2'	28:a:2567:G:OP2	2.27	0.48
31:d:34:VAL:HA	31:d:50:VAL:HA	1.94	0.48
31:d:37:VAL:HA	31:d:47:ALA:O	2.14	0.48
42:o:52:ASN:O	42:o:53:ARG:HD3	2.13	0.48
7:A:744:C:H2'	7:A:745:G:C8	2.49	0.48
7:A:1003:G:H21	7:A:1039:C:H42	1.62	0.48
7:A:1143:G:H3'	7:A:1144:G:C8	2.49	0.48
13:G:140:ASP:HA	13:G:143:ARG:HH21	1.79	0.48
19:M:64:VAL:O	19:M:64:VAL:HG23	2.13	0.48
29:b:15:A:H3'	29:b:16:G:C8	2.49	0.48
36:i:119:PHE:CD1	36:i:122:LEU:HD23	2.49	0.48
48:u:30:ILE:HD11	48:u:93:ARG:NE	2.28	0.48
7:A:157:U:O4	7:A:164:G:O6	2.31	0.47
9:C:122:SER:O	9:C:126:ARG:HG3	2.13	0.47
10:D:101:VAL:O	10:D:105:MET:HE2	2.14	0.47
28:a:263:G:O2'	28:a:429:A:N3	2.44	0.47
28:a:2323:G:O2'	28:a:2337:G:O2'	2.17	0.47

Continued on next page...

For Manuscript Review

Continued from previous page...

Atom-1	Atom-2	Interatomic distance (Å)	Clash overlap (Å)
29:b:4:C:H2'	29:b:5:U:C6	2.49	0.47
33:f:74:VAL:O	33:f:79:ILE:HG22	2.14	0.47
36:i:92:MET:SD	36:i:96:ARG:HD2	2.54	0.47
49:v:45:PHE:CE1	49:v:78:LYS:HB3	2.49	0.47
7:A:1112:C:H3'	7:A:1113:C:H5"	1.96	0.47
10:D:35:GLU:O	10:D:37:ALA:N	2.47	0.47
18:L:77:HIS:ND1	18:L:77:HIS:N	2.62	0.47
19:M:12:HIS:HD2	19:M:44:LYS:NZ	2.12	0.47
24:R:25:ASP:OD1	24:R:26:ILE:N	2.47	0.47
28:a:841:G:H2'	28:a:842:U:C6	2.47	0.47
28:a:2480:C:H2'	28:a:2481:G:O4'	2.14	0.47
28:a:2661:G:H2'	28:a:2662:A:C8	2.48	0.47
38:k:111:ILE:C	38:k:131:ALA:HB2	2.39	0.47
52:y:24:LEU:O	52:y:28:GLY:N	2.46	0.47
1:0:19:HIS:O	1:0:49:TYR:OH	2.29	0.47
5:4:1:MET:N	29:b:35:C:OP2	2.47	0.47
7:A:123:C:H2'	7:A:124:C:H6	1.78	0.47
11:E:14:LYS:HE2	11:E:117:VAL:HG13	1.96	0.47
26:T:61:GLN:OE1	26:T:61:GLN:HA	2.15	0.47
28:a:1027:A:C6	28:a:1126:A:C6	3.03	0.47
44:q:5:PHE:CE1	44:q:12:HIS:HB2	2.48	0.47
7:A:8:A:H4'	7:A:9:G:OP1	2.13	0.47
7:A:384:G:O2'	7:A:385:C:O5'	2.31	0.47
7:A:892:A:H2'	7:A:893:C:H6	1.78	0.47
9:C:202:ILE:HD12	9:C:202:ILE:H	1.80	0.47
10:D:169:THR:O	10:D:169:THR:HG22	2.14	0.47
12:F:18:VAL:HG11	12:F:58:HIS:CE1	2.49	0.47
12:F:20:GLY:HA2	12:F:23:GLU:OE2	2.14	0.47
17:K:27:PHE:HZ	27:U:33:ARG:HH12	1.60	0.47
23:Q:12:VAL:HG12	23:Q:23:VAL:HG12	1.95	0.47
25:S:6:LYS:H	25:S:6:LYS:HD3	1.78	0.47
28:a:1123:C:H2'	28:a:1124:G:C8	2.49	0.47
28:a:2362:C:O2'	28:a:2363:G:H5'	2.14	0.47
29:b:47:C:H2'	29:b:48:U:O4'	2.14	0.47
29:b:97:C:H2'	29:b:98:G:O4'	2.14	0.47
30:c:187:ASP:OD1	30:c:187:ASP:N	2.47	0.47
31:d:34:VAL:HG13	31:d:50:VAL:HA	1.95	0.47
34:g:22:GLN:HE21	34:g:55:ARG:HH22	1.61	0.47
34:g:27:LYS:HD3	34:g:28:GLY:N	2.29	0.47
39:l:111:GLU:CD	39:l:111:GLU:H	2.22	0.47
43:p:92:ARG:O	43:p:95:LEU:HB2	2.13	0.47

Continued on next page...

For Manuscript Review

Continued from previous page...

Atom-1	Atom-2	Interatomic distance (Å)	Clash overlap (Å)
45:r:38:TYR:HE2	53:z:28:LEU:HD21	1.79	0.47
7:A:607:A:H2'	7:A:608:A:O4'	2.14	0.47
7:A:720:C:O2'	24:R:52:GLN:OE1	2.32	0.47
7:A:1253:A:H3'	7:A:1254:G:H8	1.79	0.47
28:a:2373:G:H2'	28:a:2374:C:H6	1.79	0.47
28:a:2378:A:H3'	28:a:2379:G:C8	2.49	0.47
32:e:125:SER:HA	32:e:157:LEU:HD13	1.96	0.47
33:f:135:GLN:N	33:f:150:ARG:O	2.47	0.47
34:g:105:LEU:N	34:g:113:VAL:O	2.47	0.47
37:j:93:GLN:HG3	37:j:94:PRO:HD2	1.97	0.47
39:l:41:LEU:HD22	39:l:45:GLN:HE21	1.80	0.47
49:v:21:LEU:HD21	49:v:41:ARG:HB2	1.97	0.47
49:v:72:LYS:H	49:v:78:LYS:HA	1.80	0.47
7:A:526:C:OP2	18:L:88:LYS:NZ	2.39	0.47
7:A:601:G:C8	7:A:602:A:C8	3.03	0.47
7:A:1159:C:HO2'	7:A:1160:U:P	2.38	0.47
8:B:68:LEU:HD12	8:B:90:PHE:HB2	1.96	0.47
13:G:76:LYS:HG2	13:G:89:VAL:HG21	1.97	0.47
14:H:22:LYS:O	14:H:65:TYR:OH	2.32	0.47
19:M:57:ARG:O	19:M:60:VAL:HG22	2.14	0.47
22:P:41:PRO:O	22:P:43:ALA:N	2.46	0.47
28:a:2326:C:OP2	28:a:2326:C:H6	1.97	0.47
28:a:2418:A:C6	28:a:2419:U:C4	3.02	0.47
28:a:2831:G:OP2	31:d:59:ARG:NH1	2.47	0.47
34:g:22:GLN:NE2	34:g:41:VAL:O	2.46	0.47
36:i:118:MET:O	36:i:121:LYS:HB2	2.14	0.47
41:n:26:LEU:HG	41:n:92:PHE:CD1	2.50	0.47
44:q:56:GLY:O	44:q:58:VAL:HG13	2.14	0.47
5:4:7:PRO:HB2	33:f:62:GLY:HA2	1.95	0.47
7:A:144:G:N2	7:A:144:G:OP2	2.47	0.47
7:A:486:U:H2'	7:A:487:A:C8	2.48	0.47
7:A:602:A:H2'	7:A:603:U:C6	2.50	0.47
7:A:826:C:O2	14:H:16:ASN:ND2	2.48	0.47
9:C:81:GLY:HA3	9:C:85:GLU:HB2	1.97	0.47
12:F:8:PHE:HB2	12:F:84:VAL:HG21	1.96	0.47
14:H:26:THR:OG1	14:H:60:GLU:OE2	2.33	0.47
14:H:92:LEU:HD23	14:H:92:LEU:HA	1.71	0.47
27:U:5:LYS:C	27:U:18:ARG:HH12	2.23	0.47
27:U:54:LYS:HB3	27:U:54:LYS:HE3	1.80	0.47
28:a:891:G:O6	28:a:892:A:N6	2.47	0.47
28:a:1865:U:H3	28:a:1877:A:N6	2.13	0.47

Continued on next page...

For Manuscript Review

Continued from previous page...

Atom-1	Atom-2	Interatomic distance (Å)	Clash overlap (Å)
28:a:2615:U:C2	53:z:4:GLN:HA	2.50	0.47
28:a:2740:A:H2'	28:a:2741:A:O4'	2.14	0.47
31:d:37:VAL:HG22	31:d:48:ILE:HG22	1.97	0.47
38:k:74:THR:HA	38:k:107:PHE:O	2.14	0.47
38:k:93:ASN:OD1	38:k:94:THR:N	2.46	0.47
38:k:95:LEU:HD22	38:k:100:ILE:HD11	1.97	0.47
39:l:14:LYS:O	39:l:71:LYS:NZ	2.47	0.47
43:p:61:TRP:HE3	43:p:92:ARG:HB3	1.80	0.47
51:x:4:LYS:HZ2	51:x:7:ARG:HH11	1.63	0.47
4:3:18:LYS:HD2	4:3:23:ILE:HG13	1.96	0.47
7:A:563:A:HO2'	7:A:566:A:HO2'	1.53	0.47
7:A:599:C:O2'	7:A:600:A:OP1	2.33	0.47
7:A:654:G:H1	7:A:752:G:H21	1.63	0.47
7:A:1071:C:H2'	7:A:1072:G:H8	1.80	0.47
11:E:126:LYS:HG3	11:E:127:ALA:N	2.30	0.47
18:L:87:VAL:HG21	18:L:90:LEU:HD23	1.97	0.47
19:M:91:HIS:CG	19:M:97:VAL:HG11	2.49	0.47
24:R:11:CYS:SG	24:R:47:THR:HG23	2.55	0.47
28:a:1266:G:O2'	28:a:2012:G:O6	2.32	0.47
29:b:24:G:H21	29:b:26:C:N4	2.13	0.47
32:e:58:LYS:HG3	32:e:71:GLY:HA2	1.97	0.47
37:j:113:MET:N	37:j:113:MET:SD	2.87	0.47
47:t:49:VAL:HG13	47:t:52:LEU:O	2.15	0.47
47:t:94:ARG:HE	47:t:103:ILE:HD11	1.79	0.47
7:A:588:G:O2'	7:A:589:U:OP1	2.32	0.47
15:I:54:LEU:HD11	15:I:103:PHE:HE2	1.80	0.47
24:R:70:TYR:HB2	24:R:74:HIS:CE1	2.50	0.47
28:a:886:A:H2'	28:a:887:U:O4'	2.15	0.47
28:a:2314:A:H5"	33:f:33:LYS:NZ	2.30	0.47
28:a:2386:A:H2'	28:a:2387:U:O4'	2.15	0.47
28:a:2479:U:N3	28:a:2480:C:H1'	2.29	0.47
28:a:2740:A:C6	28:a:2741:A:C4	3.03	0.47
28:a:2758:A:C2'	34:g:35:ARG:HH22	2.27	0.47
7:A:120:A:O2'	7:A:122:G:H5'	2.15	0.47
7:A:751:U:H1'	21:O:23:GLY:O	2.15	0.47
7:A:963:G:C6	7:A:964:A:N7	2.83	0.47
19:M:12:HIS:O	19:M:44:LYS:NZ	2.47	0.47
28:a:3:U:H2'	28:a:4:U:C6	2.50	0.47
28:a:2479:U:H5'	28:a:2537:U:H4'	1.97	0.47
31:d:47:ALA:HA	31:d:84:LEU:N	2.29	0.47
32:e:149:ILE:HD11	32:e:188:MET:HB2	1.97	0.47

Continued on next page...

For Manuscript Review

Continued from previous page...

Atom-1	Atom-2	Interatomic distance (Å)	Clash overlap (Å)
47:t:58:ILE:HD12	47:t:58:ILE:O	2.14	0.47
7:A:77:G:O6	7:A:91:U:O2'	2.32	0.46
7:A:757:U:O2'	7:A:879:C:H1'	2.15	0.46
7:A:922:G:H2'	7:A:923:A:C8	2.50	0.46
10:D:145:ILE:C	10:D:146:ARG:HG3	2.39	0.46
17:K:97:ILE:HD11	27:U:16:LEU:HB3	1.97	0.46
28:a:85:G:C6	28:a:86:G:N3	2.83	0.46
28:a:2014:A:H2'	28:a:2015:A:C8	2.49	0.46
28:a:2335:A:H5'	41:n:13:ARG:HH21	1.80	0.46
30:c:182:ARG:HG3	30:c:183:LYS:N	2.30	0.46
33:f:4:LEU:HD12	33:f:100:PHE:HB3	1.96	0.46
43:p:90:ILE:HG12	44:q:39:LEU:O	2.15	0.46
48:u:9:ARG:HH12	48:u:17:SER:CA	2.28	0.46
7:A:135:C:H1'	7:A:325:A:C2	2.50	0.46
7:A:192:A:C2'	7:A:193:C:H5'	2.45	0.46
7:A:323:U:H2'	7:A:324:G:O4'	2.16	0.46
9:C:129:MET:HE3	9:C:129:MET:HB3	1.83	0.46
12:F:5:GLU:N	12:F:90:MET:O	2.48	0.46
17:K:100:LEU:HD23	17:K:100:LEU:HA	1.79	0.46
29:b:14:U:H3'	29:b:15:A:N7	2.29	0.46
29:b:74:U:O2	48:u:29:ILE:HG21	2.15	0.46
34:g:24:ILE:HD12	34:g:72:LEU:HD23	1.96	0.46
37:j:113:MET:SD	37:j:114:LYS:N	2.88	0.46
37:j:114:LYS:HE2	37:j:114:LYS:HB3	1.51	0.46
38:k:29:LYS:HB3	38:k:30:THR:H	1.48	0.46
44:q:35:PHE:CD1	44:q:59:ILE:HB	2.48	0.46
48:u:9:ARG:HG3	48:u:39:ALA:HB1	1.96	0.46
5:4:44:PHE:CZ	33:f:109:PRO:HG3	2.50	0.46
7:A:609:A:O2'	7:A:610:G:H8	1.98	0.46
7:A:673:A:H2'	7:A:674:G:H8	1.78	0.46
7:A:1370:C:O2'	7:A:1371:G:OP1	2.26	0.46
9:C:64:ILE:HG22	9:C:99:ALA:H	1.81	0.46
10:D:105:MET:HB3	10:D:173:VAL:HG11	1.97	0.46
12:F:8:PHE:CE2	12:F:10:VAL:HG23	2.51	0.46
13:G:140:ASP:HA	13:G:143:ARG:NE	2.29	0.46
21:O:88:ARG:HH12	28:a:715:A:H5"	1.80	0.46
28:a:1039:A:N1	28:a:1116:G:C6	2.83	0.46
28:a:1863:G:H2'	28:a:1864:U:C6	2.51	0.46
28:a:2376:A:H61	41:n:92:PHE:CB	2.27	0.46
43:p:62:ILE:HD11	43:p:92:ARG:HE	1.78	0.46
43:p:74:ILE:HG12	43:p:75:SER:H	1.80	0.46

Continued on next page...

For Manuscript Review

Continued from previous page...

Atom-1	Atom-2	Interatomic distance (Å)	Clash overlap (Å)
43:p:81:ASN:O	43:p:85:LYS:CB	2.55	0.46
4:3:2:LYS:H	4:3:35:GLN:HA	1.81	0.46
7:A:33:A:H8	18:L:29:GLN:HE22	1.64	0.46
7:A:79:G:H2'	7:A:80:G:O4'	2.16	0.46
13:G:11:LYS:HG3	13:G:12:ILE:N	2.31	0.46
15:I:75:GLN:O	15:I:79:ILE:HG12	2.16	0.46
16:J:27:GLU:O	16:J:31:ARG:CB	2.64	0.46
27:U:66:ARG:HH11	27:U:67:ARG:NH1	2.09	0.46
28:a:938:G:H2'	28:a:939:G:H5'	1.97	0.46
28:a:1028:A:C2	28:a:1029:A:C4	3.03	0.46
31:d:37:VAL:N	31:d:92:VAL:HG22	2.20	0.46
34:g:41:VAL:HG22	34:g:65:ALA:HA	1.98	0.46
7:A:1324:G:H2'	7:A:1325:A:C8	2.51	0.46
7:A:1449:C:H2'	7:A:1450:C:H6	1.81	0.46
7:A:1478:U:H2'	7:A:1479:U:C6	2.50	0.46
12:F:7:VAL:HB	12:F:88:MET:HG2	1.98	0.46
13:G:136:LYS:HB3	13:G:136:LYS:HE2	1.67	0.46
28:a:629:G:H21	28:a:640:C:H5'	1.79	0.46
28:a:1049:C:H3'	28:a:1050:A:C8	2.51	0.46
28:a:1930:G:O2'	28:a:1968:G:O6	2.30	0.46
28:a:2298:A:N6	28:a:2318:G:H1'	2.31	0.46
31:d:34:VAL:HA	31:d:51:THR:H	1.80	0.46
34:g:66:GLY:O	34:g:69:ARG:HB3	2.15	0.46
34:g:176:LYS:HA	34:g:176:LYS:HD3	1.76	0.46
48:u:83:LYS:HE2	48:u:83:LYS:HB2	1.52	0.46
51:x:13:GLU:OE1	51:x:13:GLU:N	2.40	0.46
5:4:28:VAL:HG21	5:4:32:LEU:HD13	1.97	0.46
7:A:951:G:HO2'	7:A:970:C:HO2'	1.60	0.46
7:A:967:5MC:H2'	7:A:968:A:C8	2.50	0.46
17:K:35:THR:OG1	17:K:36:ASP:N	2.49	0.46
20:N:80:SER:O	20:N:84:VAL:HG13	2.15	0.46
28:a:277:G:H4'	28:a:278:A:C4	2.50	0.46
28:a:2293:G:N3	28:a:2293:G:H2'	2.29	0.46
28:a:2532:G:C2	28:a:2533:U:C5	3.04	0.46
28:a:2669:G:H2'	28:a:2669:G:N3	2.31	0.46
35:h:6:LEU:HD21	35:h:37:VAL:HG13	1.96	0.46
48:u:28:ALA:HB1	48:u:89:ILE:O	2.16	0.46
10:D:104:ARG:HA	10:D:104:ARG:HD3	1.68	0.46
13:G:12:ILE:H	13:G:12:ILE:HG12	1.67	0.46
13:G:132:GLY:C	13:G:134:ALA:H	2.23	0.46
19:M:96:PRO:HB3	19:M:106:ALA:O	2.15	0.46

Continued on next page...

For Manuscript Review

Continued from previous page...

Atom-1	Atom-2	Interatomic distance (Å)	Clash overlap (Å)
22:P:19:VAL:H	22:P:37:GLY:HA3	1.80	0.46
28:a:305:C:H2'	28:a:306:U:C6	2.51	0.46
28:a:549:G:C5	28:a:550:C:C4	3.03	0.46
29:b:24:G:C6	29:b:56:G:C6	3.03	0.46
29:b:108:A:H3'	29:b:109:A:H4'	1.98	0.46
32:e:125:SER:CB	32:e:157:LEU:HB2	2.45	0.46
44:q:16:GLU:CD	44:q:16:GLU:N	2.74	0.46
7:A:106:C:OP1	7:A:106:C:H4'	2.13	0.46
7:A:272:C:H2'	7:A:273:A:C8	2.50	0.46
7:A:549:C:O2'	7:A:550:G:O5'	2.28	0.46
7:A:657:U:H2'	7:A:658:A:C8	2.48	0.46
7:A:998:C:N1	7:A:1044:A:H5'	2.26	0.46
7:A:1190:C:O4'	7:A:1191:G:N2	2.49	0.46
10:D:76:TYR:HA	10:D:90:LEU:HD21	1.97	0.46
17:K:53:ARG:HD3	17:K:54:GLY:N	2.30	0.46
28:a:2527:C:O2	28:a:2527:C:H2'	2.15	0.46
28:a:2533:U:H2'	28:a:2534:A:H5'	1.96	0.46
41:n:100:HIS:O	41:n:103:VAL:HG12	2.16	0.46
7:A:106:C:H5	26:T:10:ARG:HH12	1.64	0.46
7:A:199:C:H2'	7:A:200:A:C8	2.49	0.46
7:A:449:G:H1	22:P:70:ARG:HH22	1.63	0.46
7:A:1030:U:C4	7:A:1032:G:H8	2.34	0.46
23:Q:77:ARG:HH12	23:Q:79:VAL:HA	1.81	0.46
24:R:72:ASP:HB3	27:U:3:VAL:HG23	1.97	0.46
28:a:784:G:H5'	28:a:785:G:OP1	2.15	0.46
28:a:849:A:H2'	28:a:850:U:H6	1.81	0.46
28:a:1005:C:O2'	36:i:30:THR:HG21	2.16	0.46
28:a:1028:A:C2	28:a:1029:A:C5	3.04	0.46
28:a:2745:C:O2'	34:g:142:GLY:HA3	2.16	0.46
28:a:2749:A:C8	28:a:2750:A:H2'	2.51	0.46
28:a:2765:A:N3	28:a:2765:A:H2'	2.31	0.46
28:a:2851:A:N7	56:a:6208:SPD:H92	2.30	0.46
33:f:70:ALA:O	33:f:81:GLN:HG2	2.16	0.46
34:g:24:ILE:O	34:g:35:ARG:N	2.39	0.46
39:l:119:LEU:HA	39:l:119:LEU:HD23	1.69	0.46
43:p:78:LYS:HG3	43:p:117:LEU:HD22	1.98	0.46
7:A:999:C:H4'	7:A:1000:A:O4'	2.16	0.46
7:A:1049:U:O2'	7:A:1215:C:O4'	2.33	0.46
10:D:137:VAL:HG12	10:D:182:PHE:HD2	1.81	0.46
13:G:12:ILE:O	13:G:13:LEU:HB2	2.16	0.46
13:G:66:LEU:O	13:G:70:ARG:NH2	2.47	0.46

Continued on next page...

For Manuscript Review

Continued from previous page...

Atom-1	Atom-2	Interatomic distance (Å)	Clash overlap (Å)
27:U:16:LEU:HG	27:U:17:ARG:NH1	2.31	0.46
28:a:720:U:H2'	28:a:721:A:C8	2.50	0.46
28:a:1042:G:H2'	28:a:1043:C:H6	1.81	0.46
28:a:1508:A:H2'	28:a:1509:A:C8	2.51	0.46
28:a:2524:G:C6	28:a:2525:G:N7	2.84	0.46
28:a:2526:G:N1	28:a:2538:C:C4	2.84	0.46
29:b:32:U:H2'	29:b:33:G:C8	2.52	0.46
32:e:172:ALA:HB2	32:e:192:ALA:HB1	1.98	0.46
33:f:7:TYR:HE2	33:f:173:PHE:HA	1.80	0.46
33:f:56:ASP:O	33:f:60:ILE:HG23	2.16	0.46
34:g:171:THR:O	34:g:171:THR:OG1	2.29	0.46
7:A:64:G:H4'	7:A:65:A:O5'	2.14	0.45
19:M:66:GLU:HB3	19:M:67:GLY:H	1.56	0.45
24:R:37:GLY:O	24:R:63:ARG:NH2	2.49	0.45
28:a:596:U:O2	28:a:662:G:N2	2.48	0.45
28:a:749:A:OP2	56:a:6217:SPD:N10	2.49	0.45
28:a:1027:A:N7	28:a:1126:A:N1	2.63	0.45
28:a:2266:A:H4'	28:a:2267:A:N3	2.32	0.45
29:b:13:G:H5'	29:b:15:A:C2	2.50	0.45
32:e:108:ILE:HA	38:k:1:MET:HE1	1.97	0.45
43:p:66:ASN:HD21	43:p:70:ARG:NH2	2.10	0.45
49:v:46:HIS:HB2	49:v:79:PHE:CZ	2.50	0.45
7:A:782:A:H62	7:A:800:G:H21	1.62	0.45
7:A:1280:A:H2'	7:A:1280:A:N3	2.32	0.45
9:C:55:ILE:HG22	9:C:68:ILE:HG12	1.98	0.45
14:H:126:ILE:HG22	14:H:127:CYS:SG	2.56	0.45
18:L:30:LYS:HE3	18:L:59:ASN:ND2	2.23	0.45
28:a:146:A:H2'	28:a:147:C:C6	2.51	0.45
28:a:632:A:H2'	28:a:633:A:C8	2.51	0.45
28:a:1049:C:H3'	28:a:1050:A:H8	1.80	0.45
28:a:2388:A:OP1	28:a:2388:A:H4'	2.16	0.45
28:a:2739:U:C5	28:a:2763:G:C6	3.04	0.45
41:n:80:GLU:O	41:n:84:GLU:HG2	2.17	0.45
43:p:78:LYS:HB2	43:p:78:LYS:HE3	1.70	0.45
47:t:96:PHE:HD2	47:t:101:GLU:OE2	1.99	0.45
49:v:43:THR:O	49:v:46:HIS:NE2	2.33	0.45
7:A:485:U:H4'	7:A:485:U:OP2	2.15	0.45
9:C:70:THR:HG21	9:C:76:VAL:HG21	1.98	0.45
9:C:74:GLY:O	9:C:76:VAL:N	2.44	0.45
10:D:13:ARG:HG3	10:D:32:CYS:HA	1.98	0.45
25:S:40:ILE:H	25:S:40:ILE:HG12	1.48	0.45

Continued on next page...

For Manuscript Review

Continued from previous page...

Atom-1	Atom-2	Interatomic distance (Å)	Clash overlap (Å)
28:a:145:C:H2'	28:a:146:A:C8	2.52	0.45
28:a:1021:A:C5	28:a:1023:U:C5	3.04	0.45
28:a:2064:C:H2'	28:a:2065:C:C6	2.52	0.45
28:a:2683:C:OP1	42:o:51:ARG:NH2	2.49	0.45
28:a:2751:G:OP1	34:g:3:ARG:HD2	2.16	0.45
7:A:578:C:O2'	7:A:728:A:N3	2.48	0.45
7:A:1012:G:N2	7:A:1013:G:H8	2.14	0.45
28:a:413:C:O2'	28:a:1863:G:N2	2.49	0.45
28:a:2628:C:O2'	28:a:2781:A:H2'	2.16	0.45
28:a:2739:U:C6	28:a:2763:G:C6	3.05	0.45
29:b:71:C:H2'	29:b:72:G:O4'	2.16	0.45
34:g:126:PRO:HD3	34:g:132:VAL:H	1.82	0.45
36:i:96:ARG:HG2	36:i:96:ARG:NH1	2.30	0.45
37:j:53:LYS:HE2	37:j:53:LYS:HB2	1.78	0.45
38:k:95:LEU:HB3	38:k:101:ILE:HG12	1.97	0.45
46:s:14:PRO:HD3	51:x:30:MET:HE3	1.97	0.45
7:A:457:G:H22	7:A:475:U:H2'	1.80	0.45
7:A:539:A:O2'	7:A:540:G:O5'	2.31	0.45
8:B:64:LYS:HD2	8:B:64:LYS:HA	1.68	0.45
10:D:33:LYS:O	10:D:34:ILE:C	2.58	0.45
17:K:97:ILE:HA	17:K:100:LEU:HB2	1.98	0.45
18:L:108:LYS:HB2	18:L:108:LYS:HE3	1.80	0.45
24:R:49:ALA:HB3	24:R:50:LYS:NZ	2.32	0.45
28:a:172:A:H2'	28:a:173:A:C8	2.52	0.45
28:a:841:G:C6	28:a:938:G:C2	3.03	0.45
28:a:1021:A:C4	28:a:1023:U:C5	3.05	0.45
28:a:1754:A:N1	28:a:2716:C:O2'	2.44	0.45
28:a:2739:U:C6	28:a:2763:G:N1	2.84	0.45
29:b:27:C:P	41:n:34:HIS:HE2	2.38	0.45
29:b:68:C:H2'	29:b:69:G:O4'	2.17	0.45
37:j:116:ILE:C	37:j:116:ILE:HD12	2.41	0.45
3:2:26:HIS:CE1	3:2:48:ALA:HB2	2.52	0.45
7:A:1125:U:O2'	7:A:1146:A:N6	2.50	0.45
7:A:1319:A:O2'	25:S:37:ARG:HG2	2.16	0.45
18:L:15:LYS:HE2	18:L:17:ALA:HA	1.99	0.45
19:M:107:ARG:HH11	19:M:107:ARG:HG2	1.81	0.45
23:Q:38:ILE:HD13	23:Q:40:ARG:HD3	1.99	0.45
25:S:15:LEU:O	25:S:19:VAL:HG23	2.16	0.45
28:a:938:G:C2	28:a:939:G:C5	3.04	0.45
28:a:1245:G:OP1	38:k:13:LYS:NZ	2.48	0.45
28:a:2303:G:C6	28:a:2314:A:N6	2.84	0.45

Continued on next page...

For Manuscript Review

Continued from previous page...

Atom-1	Atom-2	Interatomic distance (Å)	Clash overlap (Å)
28:a:2327:A:N6	28:a:2388:A:N6	2.63	0.45
28:a:2471:A:C8	28:a:2472:G:C8	3.05	0.45
28:a:2820:A:O2'	28:a:2821:A:OP1	2.33	0.45
29:b:45:A:C6	29:b:46:A:C8	3.04	0.45
32:e:18:THR:HA	32:e:106:LYS:HG2	1.97	0.45
35:h:8:LYS:HD3	35:h:8:LYS:HA	1.67	0.45
36:i:17:VAL:O	36:i:139:VAL:HG22	2.17	0.45
41:n:39:VAL:HB	41:n:49:VAL:HG13	1.98	0.45
44:q:1:MET:HE1	44:q:41:ILE:HA	1.99	0.45
7:A:1057:G:P	7:A:1057:G:O4'	2.75	0.45
7:A:1377:U:O4	13:G:10:ARG:HD2	2.17	0.45
8:B:79:ALA:HB1	8:B:210:VAL:HG21	1.99	0.45
19:M:90:ARG:HD3	19:M:90:ARG:HA	1.71	0.45
28:a:861:A:OP2	56:a:6218:SPD:N1	2.50	0.45
28:a:1036:G:H3'	28:a:1037:G:C8	2.51	0.45
28:a:2618:G:H21	31:d:155:VAL:HG21	1.81	0.45
28:a:2739:U:C4	28:a:2766:A:N6	2.85	0.45
29:b:86:G:O2'	29:b:87:U:H2'	2.16	0.45
36:i:36:LEU:HD22	36:i:121:LYS:HE3	1.99	0.45
36:i:56:VAL:HB	36:i:124:VAL:HG13	1.98	0.45
46:s:79:ASP:OD1	46:s:79:ASP:N	2.49	0.45
7:A:95:G:H4'	7:A:96:U:C5	2.51	0.45
7:A:1446:U:H1'	7:A:1458:G:H22	1.82	0.45
7:A:1493:A:H3'	7:A:1494:A:H8	1.82	0.45
14:H:47:GLU:HA	14:H:47:GLU:OE1	2.16	0.45
17:K:20:VAL:HA	17:K:83:GLU:HB3	1.99	0.45
25:S:6:LYS:HD3	25:S:6:LYS:N	2.31	0.45
28:a:660:C:N3	28:a:661:A:N6	2.64	0.45
28:a:841:G:C5	28:a:842:U:C4	3.05	0.45
28:a:2296:U:O2'	28:a:2333:A:N6	2.47	0.45
28:a:2370:G:H2'	28:a:2371:G:C8	2.51	0.45
34:g:19:ILE:N	34:g:19:ILE:HD12	2.32	0.45
36:i:55:ILE:HG22	36:i:56:VAL:N	2.31	0.45
44:q:1:MET:HB2	44:q:43:ASN:CG	2.42	0.45
48:u:83:LYS:CB	48:u:85:LYS:HG3	2.43	0.45
2:1:25:LYS:HE2	28:a:1367:A:O2'	2.17	0.45
7:A:78:A:H2'	7:A:79:G:C8	2.52	0.45
7:A:89:C:C4	7:A:90:C:N4	2.85	0.45
7:A:255:G:H2'	7:A:256:U:C6	2.51	0.45
7:A:738:C:OP2	12:F:91:ARG:NH2	2.50	0.45
7:A:1448:A:H3'	7:A:1449:C:C6	2.52	0.45

Continued on next page...

For Manuscript Review

Continued from previous page...

Atom-1	Atom-2	Interatomic distance (Å)	Clash overlap (Å)
7:A:1517:2MG:N2	7:A:1520:MA6:OP2	2.48	0.45
12:F:42:TRP:CE3	12:F:102:MET:HE2	2.52	0.45
28:a:52:A:H2'	28:a:53:A:C8	2.52	0.45
28:a:155:A:H2'	28:a:156:A:C8	2.52	0.45
28:a:621:A:OP2	38:k:99:ASN:ND2	2.50	0.45
28:a:634:C:H2'	28:a:635:C:O4'	2.17	0.45
28:a:1862:G:H1	28:a:1880:U:H3	1.64	0.45
28:a:1873:G:H2'	28:a:1874:C:H6	1.82	0.45
28:a:2251:OMG:H1'	28:a:2251:OMG:HM23	1.56	0.45
28:a:2280:G:H1'	28:a:2327:A:C2	2.51	0.45
28:a:2298:A:H3'	28:a:2299:U:C6	2.52	0.45
28:a:2526:G:C6	28:a:2538:C:C4	3.04	0.45
31:d:36:GLN:O	31:d:48:ILE:HA	2.16	0.45
33:f:30:ARG:O	33:f:159:THR:OG1	2.25	0.45
36:i:32:LEU:HD23	36:i:32:LEU:HA	1.85	0.45
41:n:52:SER:OG	41:n:53:THR:N	2.48	0.45
7:A:219:C:H2'	7:A:220:G:C8	2.52	0.45
7:A:500:G:H2'	7:A:501:C:H6	1.82	0.45
7:A:1024:G:H2'	7:A:1025:U:C4'	2.47	0.45
7:A:1110:A:H3'	7:A:1111:A:H8	1.83	0.45
9:C:183:ASP:N	9:C:183:ASP:OD1	2.50	0.45
13:G:101:MET:O	13:G:105:VAL:HG23	2.17	0.45
15:I:43:THR:O	15:I:47:VAL:HG22	2.17	0.45
28:a:155:A:H2'	28:a:156:A:H8	1.82	0.45
28:a:2625:G:C2	28:a:2626:C:C2	3.05	0.45
56:a:6219:SPD:H22	56:a:6219:SPD:H51	1.78	0.45
31:d:103:ASP:OD2	31:d:103:ASP:N	2.50	0.45
33:f:123:ASP:OD2	33:f:127:ASN:HB2	2.16	0.45
33:f:170:LEU:HB3	33:f:175:PHE:CG	2.52	0.45
41:n:111:ARG:HH21	41:n:117:PHE:C	2.23	0.45
47:t:24:LYS:HB3	47:t:24:LYS:HZ2	1.82	0.45
7:A:74:A:H61	7:A:95:G:P	2.39	0.44
7:A:532:A:N6	7:A:1207:G:O2'	2.47	0.44
7:A:764:C:C2	7:A:765:G:H8	2.35	0.44
9:C:190:HIS:CE1	9:C:195:VAL:HG13	2.52	0.44
18:L:25:GLU:O	18:L:25:GLU:OE2	2.35	0.44
22:P:75:ILE:HD12	22:P:76:LYS:N	2.31	0.44
28:a:918:A:O2'	29:b:79:G:N2	2.50	0.44
28:a:1048:A:H62	28:a:1110:G:H21	1.65	0.44
28:a:1184:U:H2'	28:a:1185:G:H5'	1.99	0.44
28:a:1856:U:H2'	28:a:1857:G:O4'	2.17	0.44

Continued on next page...

For Manuscript Review

Continued from previous page...

Atom-1	Atom-2	Interatomic distance (Å)	Clash overlap (Å)
28:a:2298:A:H5”	33:f:72:LYS:HE2	1.99	0.44
28:a:2419:U:O2’	28:a:2420:C:H5’	2.18	0.44
28:a:2528:U:C2	28:a:2536:G:N1	2.84	0.44
49:v:46:HIS:O	49:v:79:PHE:HA	2.16	0.44
7:A:868:C:H2’	7:A:869:G:O4’	2.17	0.44
7:A:1014:A:H3’	7:A:1015:G:C8	2.53	0.44
7:A:1035:A:O2’	7:A:1036:G:H5”	2.17	0.44
13:G:17:LYS:HE2	15:I:46:MET:SD	2.57	0.44
13:G:63:GLU:HA	13:G:66:LEU:HD23	1.99	0.44
13:G:124:LEU:HD23	13:G:124:LEU:O	2.17	0.44
15:I:129:LYS:C	15:I:129:LYS:HD3	2.41	0.44
25:S:16:LEU:HA	25:S:19:VAL:HB	1.99	0.44
26:T:69:LYS:H	26:T:69:LYS:CD	2.26	0.44
28:a:897:C:H2’	28:a:898:C:H6	1.82	0.44
28:a:2333:A:OP1	49:v:77:ARG:NH1	2.49	0.44
28:a:2579:C:OP1	56:a:6220:SPD:N1	2.50	0.44
28:a:2751:G:P	34:g:3:ARG:HH11	2.40	0.44
7:A:69:G:H1	7:A:98:G:N2	2.14	0.44
7:A:836:G:H2’	7:A:837:U:H5’	1.99	0.44
7:A:1402:G:H2’	7:A:1403:4OC:O4’	2.17	0.44
13:G:38:THR:O	13:G:41:SER:OG	2.28	0.44
16:J:18:ILE:O	16:J:18:ILE:HG13	2.17	0.44
17:K:84:VAL:O	17:K:110:ILE:HA	2.18	0.44
20:N:90:ARG:NH2	20:N:92:GLU:OE2	2.49	0.44
21:O:9:ALA:HA	21:O:12:VAL:HG12	1.99	0.44
26:T:85:LYS:HD2	26:T:85:LYS:O	2.17	0.44
28:a:284:U:O4	28:a:356:G:O6	2.35	0.44
28:a:1019:U:OP1	28:a:1036:G:H4’	2.18	0.44
28:a:1581:G:H2’	28:a:1582:C:H6	1.79	0.44
28:a:2295:C:OP2	41:n:10:ARG:HG3	2.18	0.44
28:a:2327:A:H2’	28:a:2328:A:O4’	2.17	0.44
28:a:2478:A:C6	28:a:2479:U:C4	3.05	0.44
28:a:2533:U:C4	28:a:2534:A:H1’	2.53	0.44
29:b:45:A:H8	29:b:45:A:O5’	1.99	0.44
29:b:66:A:N6	29:b:107:G:H3’	2.32	0.44
32:e:148:ILE:HA	32:e:187:VAL:O	2.18	0.44
34:g:30:ASN:CG	34:g:136:ALA:HB2	2.41	0.44
34:g:87:LEU:HD22	34:g:148:LEU:CB	2.41	0.44
43:p:94:ILE:HD12	44:q:4:VAL:HG21	1.99	0.44
48:u:48:MET:CE	48:u:85:LYS:HA	2.48	0.44
7:A:449:G:N3	7:A:449:G:H2’	2.31	0.44

Continued on next page...

For Manuscript Review

Continued from previous page...

Atom-1	Atom-2	Interatomic distance (Å)	Clash overlap (Å)
9:C:82:GLU:CG	9:C:83:ASP:H	2.31	0.44
11:E:112:ARG:O	11:E:113:ALA:C	2.61	0.44
12:F:55:HIS:O	12:F:55:HIS:ND1	2.51	0.44
19:M:39:ILE:HG22	19:M:40:ALA:H	1.82	0.44
28:a:272:A:H2'	28:a:273:G:H8	1.82	0.44
28:a:2758:A:H1'	34:g:35:ARG:NH2	2.33	0.44
33:f:57:LEU:HA	33:f:60:ILE:HG12	1.99	0.44
34:g:90:VAL:HG12	34:g:160:LYS:HG2	1.99	0.44
38:k:100:ILE:HD12	38:k:101:ILE:HG23	1.99	0.44
40:m:79:LEU:HD12	40:m:83:LEU:HD12	1.99	0.44
44:q:64:VAL:HG22	44:q:95:ASP:O	2.17	0.44
7:A:422:C:H1'	7:A:423:G:C2	2.52	0.44
7:A:973:G:OP1	16:J:59:LYS:HD3	2.18	0.44
7:A:996:A:OP2	7:A:1217:A:H4'	2.17	0.44
10:D:26:ARG:HB2	10:D:27:ALA:H	1.59	0.44
10:D:183:LYS:HB2	10:D:184:ARG:HD3	1.99	0.44
23:Q:36:LYS:HB3	23:Q:36:LYS:HE3	1.83	0.44
28:a:2419:U:C2	28:a:2420:C:C5	3.06	0.44
28:a:2742:G:H2'	28:a:2743:U:C1'	2.47	0.44
34:g:54:PRO:HB3	34:g:61:GLY:CA	2.47	0.44
37:j:108:ARG:HA	37:j:108:ARG:HD2	1.61	0.44
7:A:436:C:H2'	7:A:437:U:C6	2.53	0.44
7:A:513:C:H2'	7:A:514:C:C6	2.53	0.44
7:A:1256:G:H5"	7:A:1357:A:H4'	2.00	0.44
7:A:1320:A:H1'	7:A:1321:C:OP1	2.18	0.44
10:D:167:LYS:HE3	10:D:167:LYS:HB3	1.69	0.44
12:F:39:LEU:HD22	12:F:39:LEU:HA	1.84	0.44
12:F:54:LEU:HD22	12:F:56:LYS:O	2.18	0.44
14:H:18:GLN:OE1	14:H:70:ALA:HA	2.17	0.44
15:I:87:LEU:HD22	15:I:98:LEU:HD11	1.99	0.44
28:a:2296:U:C2'	28:a:2333:A:H61	2.30	0.44
36:i:17:VAL:HB	36:i:55:ILE:HB	1.99	0.44
39:l:111:GLU:O	39:l:114:ARG:HB2	2.18	0.44
41:n:28:VAL:HG11	41:n:103:VAL:HG23	2.00	0.44
43:p:78:LYS:HG3	43:p:117:LEU:HD13	1.99	0.44
49:v:19:LYS:HB3	49:v:19:LYS:HE2	1.77	0.44
7:A:410:G:N2	7:A:432:A:H62	2.15	0.44
7:A:892:A:H2'	7:A:893:C:C6	2.52	0.44
11:E:33:PHE:CG	11:E:57:PRO:HB3	2.52	0.44
11:E:99:ALA:HB2	11:E:124:LEU:HD12	1.99	0.44
15:I:50:GLN:N	15:I:51:PRO:HD2	2.33	0.44

Continued on next page...

For Manuscript Review

Continued from previous page...

Atom-1	Atom-2	Interatomic distance (Å)	Clash overlap (Å)
26:T:70:ASN:O	26:T:74:ARG:HG3	2.18	0.44
28:a:463:G:N2	28:a:466:A:OP2	2.39	0.44
28:a:475:C:O2	28:a:479:A:N6	2.51	0.44
28:a:1020:A:N6	28:a:1141:U:O2	2.41	0.44
28:a:1916:A:H2'	28:a:1917:PSU:C6	2.52	0.44
28:a:2473:U:OP1	28:a:2474:U:H5	2.01	0.44
28:a:2672:U:H2'	28:a:2673:G:H4'	1.99	0.44
29:b:14:U:C4'	29:b:69:G:H22	2.28	0.44
48:u:20:LEU:O	48:u:25:LYS:N	2.50	0.44
52:y:11:ARG:HB2	52:y:54:MET:HB2	1.98	0.44
3:2:15:LYS:HZ1	3:2:23:LYS:HD3	1.81	0.44
3:2:43:HIS:NE2	28:a:2351:G:N7	2.66	0.44
5:4:56:ARG:NH2	25:S:64:ASP:HB3	2.33	0.44
7:A:204:G:O2'	7:A:214:C:O2	2.29	0.44
7:A:235:C:H2'	7:A:236:A:C8	2.53	0.44
7:A:661:G:O2'	7:A:662:U:H5'	2.18	0.44
7:A:736:C:OP1	24:R:61:ARG:NH2	2.50	0.44
10:D:117:LEU:O	10:D:122:ALA:HB3	2.18	0.44
28:a:630:G:C2	28:a:632:A:OP2	2.70	0.44
28:a:861:A:C2	29:b:99:A:H2	2.36	0.44
38:k:95:LEU:HA	38:k:100:ILE:HD11	1.99	0.44
39:l:53:MET:HA	39:l:116:ALA:HB1	2.00	0.44
43:p:62:ILE:HA	43:p:65:ILE:HG22	1.99	0.44
52:y:6:LYS:HA	52:y:6:LYS:HE3	2.00	0.44
7:A:1318:C:H5'	7:A:1319:A:OP2	2.18	0.44
8:B:10:LEU:HA	8:B:14:VAL:HG12	1.98	0.44
9:C:157:LEU:HD12	9:C:164:ARG:HB2	1.99	0.44
15:I:9:THR:HG22	15:I:85:ARG:HH21	1.82	0.44
19:M:34:LEU:CD1	19:M:39:ILE:HB	2.48	0.44
20:N:28:LYS:HA	20:N:31:ILE:HG22	1.99	0.44
28:a:596:U:C2	28:a:662:G:N2	2.86	0.44
28:a:597:G:N1	28:a:661:A:N6	2.66	0.44
28:a:627:A:H62	38:k:112:LEU:H	1.64	0.44
28:a:728:G:OP2	56:a:6215:SPD:N1	2.50	0.44
28:a:973:A:H5'	28:a:1188:U:H1'	2.00	0.44
28:a:1110:G:HO2'	28:a:1111:A:H8	1.58	0.44
28:a:1880:U:N3	28:a:1881:C:N4	2.65	0.44
32:e:111:GLU:HG2	32:e:114:ARG:HH21	1.83	0.44
32:e:128:ALA:O	32:e:130:LYS:N	2.51	0.44
36:i:1:MET:HE3	44:q:12:HIS:HA	1.99	0.44
39:l:33:LEU:HD12	39:l:129:THR:O	2.18	0.44

Continued on next page...

For Manuscript Review

Continued from previous page...

Atom-1	Atom-2	Interatomic distance (Å)	Clash overlap (Å)
39:l:34:LYS:HG3	39:l:131:VAL:HG11	1.99	0.44
43:p:79:PHE:HA	43:p:113:ALA:HB1	2.00	0.44
4:3:4:ARG:O	4:3:37:GLN:HA	2.18	0.43
7:A:120:A:H2'	7:A:122:G:C8	2.44	0.43
13:G:127:ALA:O	13:G:131:LYS:N	2.49	0.43
14:H:9:ASP:N	14:H:9:ASP:OD1	2.49	0.43
18:L:83:ARG:HE	18:L:83:ARG:HB3	1.68	0.43
19:M:52:GLN:HG3	19:M:56:LEU:HB3	2.00	0.43
28:a:661:A:C6	28:a:662:G:C8	3.06	0.43
28:a:2478:A:C6	28:a:2479:U:C5	3.05	0.43
28:a:2766:A:H2'	28:a:2766:A:N3	2.33	0.43
3:2:8:ARG:HD2	3:2:8:ARG:HA	1.70	0.43
7:A:1028:C:H1'	7:A:1035:A:H61	1.82	0.43
8:B:8:ASP:CG	8:B:9:MET:H	2.26	0.43
8:B:9:MET:HB2	8:B:208:ARG:HH21	1.82	0.43
14:H:36:ILE:HG22	14:H:40:LEU:HD23	2.01	0.43
23:Q:30:LYS:HE3	23:Q:30:LYS:HB3	1.62	0.43
27:U:12:PHE:O	27:U:15:ALA:HB3	2.18	0.43
28:a:279:A:H62	28:a:361:G:N2	2.15	0.43
32:e:194:LYS:HE2	32:e:194:LYS:N	2.33	0.43
34:g:101:ASN:CA	34:g:117:LEU:HB2	2.48	0.43
36:i:60:ASP:OD1	36:i:60:ASP:N	2.35	0.43
38:k:14:LYS:HB2	38:k:14:LYS:HE3	1.82	0.43
40:m:8:ARG:HD2	40:m:43:GLU:HG2	2.00	0.43
43:p:57:PHE:O	43:p:61:TRP:CD1	2.71	0.43
44:q:43:ASN:HD22	44:q:103:ALA:HB2	1.82	0.43
7:A:378:G:HO2'	7:A:379:C:H6	1.62	0.43
11:E:110:ALA:O	11:E:111:MET:C	2.61	0.43
13:G:10:ARG:HG3	13:G:11:LYS:N	2.32	0.43
17:K:128:ARG:HH11	17:K:128:ARG:HG3	1.82	0.43
21:O:88:ARG:NH2	21:O:89:ARG:HH22	2.17	0.43
28:a:268:C:H2'	28:a:269:C:H5'	1.99	0.43
28:a:548:G:H2'	28:a:549:G:C8	2.53	0.43
28:a:634:C:N3	28:a:635:C:C4	2.86	0.43
28:a:1018:U:H4'	28:a:1036:G:O2'	2.18	0.43
28:a:1023:U:C4	28:a:1024:G:C5	3.07	0.43
28:a:1045:C:N4	28:a:1111:A:H5"	2.33	0.43
28:a:1799:G:OP1	30:c:258:ARG:NH1	2.47	0.43
28:a:2280:G:H4'	28:a:2327:A:N3	2.34	0.43
28:a:2357:G:H5'	28:a:2358:A:OP2	2.19	0.43
28:a:2369:A:H2'	28:a:2370:G:C8	2.53	0.43

Continued on next page...

For Manuscript Review

Continued from previous page...

Atom-1	Atom-2	Interatomic distance (Å)	Clash overlap (Å)
28:a:2740:A:C8	28:a:2741:A:N7	2.85	0.43
28:a:2743:U:H5'	28:a:2744:G:OP2	2.18	0.43
28:a:2748:A:H1'	28:a:2757:A:H61	1.83	0.43
29:b:80:U:C2	29:b:96:G:O6	2.71	0.43
33:f:57:LEU:HA	33:f:60:ILE:CG1	2.48	0.43
33:f:63:GLN:HE21	33:f:95:ARG:CZ	2.31	0.43
38:k:123:ARG:HG2	38:k:124:GLY:N	2.34	0.43
7:A:166:U:H2'	7:A:167:G:C8	2.52	0.43
7:A:982:U:OP1	7:A:982:U:H6	2.01	0.43
7:A:1369:G:C2'	7:A:1370:C:H5'	2.49	0.43
7:A:1402:G:H3'	7:A:1403:4OC:H6	2.00	0.43
13:G:35:LYS:HB3	13:G:35:LYS:HE2	1.77	0.43
24:R:72:ASP:HB3	27:U:3:VAL:CG2	2.49	0.43
25:S:14:HIS:CD2	25:S:15:LEU:HD23	2.53	0.43
25:S:50:ALA:HB1	25:S:57:HIS:HB3	2.00	0.43
28:a:839:U:C2	28:a:840:C:C5	3.06	0.43
28:a:1027:A:C5	28:a:1126:A:N6	2.86	0.43
28:a:1593:A:H2'	28:a:1594:U:C6	2.54	0.43
28:a:2029:G:H2'	28:a:2031:A:OP1	2.18	0.43
28:a:2303:G:O2'	33:f:129:SER:HB2	2.19	0.43
30:c:261:LYS:HA	30:c:264:ASP:OD2	2.19	0.43
33:f:160:ALA:HB1	33:f:165:GLU:HB2	2.00	0.43
34:g:97:ALA:O	34:g:104:ASN:N	2.47	0.43
41:n:33:ARG:HG3	41:n:34:HIS:CE1	2.53	0.43
7:A:108:G:H5'	7:A:109:A:C3'	2.49	0.43
7:A:239:U:C3'	7:A:240:G:H5"	2.49	0.43
10:D:91:LEU:O	10:D:95:GLU:HB2	2.18	0.43
15:I:118:LEU:CD2	15:I:124:ARG:HG2	2.48	0.43
17:K:109:ASN:C	17:K:110:ILE:HD12	2.43	0.43
28:a:848:C:H2'	28:a:849:A:C8	2.50	0.43
28:a:937:C:N4	28:a:938:G:C5	2.86	0.43
28:a:938:G:N1	28:a:939:G:N7	2.66	0.43
28:a:1416:G:HO2'	28:a:1417:C:P	2.38	0.43
28:a:2361:G:H2'	28:a:2362:C:O4'	2.18	0.43
28:a:2812:G:H2'	28:a:2813:A:C8	2.53	0.43
34:g:3:ARG:HB2	34:g:6:LYS:HE3	2.00	0.43
36:i:54:ILE:HG23	36:i:122:LEU:CA	2.46	0.43
43:p:90:ILE:HG13	43:p:90:ILE:H	1.49	0.43
10:D:145:ILE:CG2	10:D:146:ARG:N	2.81	0.43
11:E:159:LYS:HE3	14:H:43:GLU:HA	2.00	0.43
17:K:64:GLN:O	17:K:68:GLU:HG2	2.18	0.43

Continued on next page...

For Manuscript Review

Continued from previous page...

Atom-1	Atom-2	Interatomic distance (Å)	Clash overlap (Å)
19:M:104:THR:OG1	19:M:105:ASN:N	2.51	0.43
20:N:41:ARG:C	20:N:43:ASN:H	2.26	0.43
21:O:18:ASP:HB2	21:O:19:ALA:H	1.61	0.43
21:O:27:VAL:O	21:O:31:LEU:HD12	2.19	0.43
25:S:13:LEU:O	25:S:17:LYS:HG2	2.18	0.43
28:a:85:G:C6	28:a:98:G:N1	2.87	0.43
28:a:598:U:H2'	28:a:599:A:C8	2.53	0.43
28:a:1000:A:H2'	28:a:1001:A:C8	2.54	0.43
28:a:1865:U:C6	28:a:1875:G:N1	2.86	0.43
28:a:2316:G:H2'	28:a:2317:A:C8	2.53	0.43
28:a:2739:U:C2	28:a:2764:A:N6	2.82	0.43
29:b:42:C:H6	33:f:66:LEU:HD21	1.83	0.43
31:d:35:THR:HA	31:d:93:GLY:H	1.83	0.43
33:f:4:LEU:HD23	33:f:4:LEU:HA	1.78	0.43
34:g:18:LYS:HD3	34:g:19:ILE:N	2.34	0.43
37:j:5:GLN:HA	37:j:20:MET:HE3	2.00	0.43
53:z:31:ASP:HB3	53:z:35:GLY:H	1.82	0.43
4:3:32:LYS:HD3	28:a:2478:A:OP1	2.19	0.43
7:A:1442:A:N6	7:A:1461:C:C2	2.87	0.43
7:A:1464:U:H2'	7:A:1465:U:C6	2.54	0.43
15:I:22:LYS:H	15:I:62:ASP:HB2	1.84	0.43
28:a:1023:U:C5	28:a:1024:G:N7	2.86	0.43
28:a:1412:U:H2'	28:a:1413:A:C8	2.54	0.43
28:a:2523:G:H21	28:a:2764:A:C1'	2.18	0.43
28:a:2680:U:O2'	28:a:2681:C:H5'	2.19	0.43
29:b:90:C:C4	29:b:91:C:H5	2.37	0.43
30:c:146:MET:SD	30:c:154:LEU:HD11	2.58	0.43
34:g:35:ARG:HG3	34:g:36:THR:N	2.33	0.43
37:j:7:MET:HE3	37:j:7:MET:HB3	1.81	0.43
38:k:75:ALA:O	38:k:108:ALA:HB1	2.18	0.43
42:o:43:PHE:CE1	42:o:63:LYS:HD3	2.53	0.43
48:u:71:LYS:HB3	48:u:73:LYS:NZ	2.34	0.43
7:A:536:C:O2'	7:A:537:G:H8	2.02	0.43
7:A:536:C:O2'	7:A:537:G:O5'	2.25	0.43
7:A:1392:U:H2'	7:A:1393:G:C8	2.53	0.43
7:A:1452:U:H1'	7:A:1453:C:OP2	2.18	0.43
9:C:4:LYS:HB2	9:C:4:LYS:HE2	1.74	0.43
11:E:81:LEU:HB2	11:E:98:PRO:HG3	2.01	0.43
14:H:7:ILE:HD11	14:H:32:LEU:HD13	1.99	0.43
19:M:82:ASP:OD1	19:M:83:LEU:HD23	2.19	0.43
25:S:28:LYS:O	25:S:29:LYS:HB2	2.19	0.43

Continued on next page...

For Manuscript Review

Continued from previous page...

Atom-1	Atom-2	Interatomic distance (Å)	Clash overlap (Å)
28:a:760:G:H2'	28:a:761:A:O4'	2.19	0.43
28:a:2247:A:H2'	28:a:2248:C:H6	1.84	0.43
28:a:2419:U:H2'	28:a:2420:C:C6	2.53	0.43
28:a:2527:C:H5"	28:a:2528:U:OP2	2.18	0.43
29:b:26:C:H2'	29:b:27:C:O4'	2.18	0.43
29:b:70:C:C2	29:b:71:C:C5	3.06	0.43
33:f:126:GLY:HA2	33:f:163:ASP:HA	2.00	0.43
36:i:5:THR:HG23	36:i:45:THR:HG21	1.99	0.43
41:n:26:LEU:HD13	41:n:39:VAL:HG22	2.00	0.43
48:u:48:MET:HG2	48:u:51:GLN:HE22	1.82	0.43
49:v:65:GLY:HA3	49:v:85:GLU:H	1.84	0.43
7:A:377:G:H22	7:A:386:C:H5	1.67	0.43
9:C:50:ALA:O	9:C:70:THR:OG1	2.37	0.43
16:J:34:ALA:HB1	16:J:36:VAL:H	1.84	0.43
28:a:58:G:H2'	28:a:59:U:C6	2.54	0.43
28:a:839:U:H2'	28:a:840:C:C6	2.54	0.43
28:a:1027:A:O2'	28:a:1028:A:H5'	2.19	0.43
28:a:1165:A:N3	28:a:1166:G:N7	2.67	0.43
28:a:1183:U:H2'	28:a:1184:U:O4'	2.19	0.43
28:a:2740:A:C8	28:a:2764:A:N6	2.87	0.43
28:a:2741:A:O2'	28:a:2742:G:H5'	2.19	0.43
30:c:80:ARG:NH1	30:c:82:GLU:OE2	2.52	0.43
34:g:156:PRO:HB2	34:g:172:LYS:NZ	2.33	0.43
37:j:76:VAL:HG22	42:o:73:VAL:HG13	2.00	0.43
43:p:80:ILE:HA	43:p:83:LEU:HG	1.99	0.43
7:A:215:C:O4'	7:A:466:A:N6	2.52	0.43
7:A:377:G:H1	7:A:386:C:H41	1.67	0.43
7:A:492:C:H2'	7:A:493:A:H8	1.81	0.43
7:A:555:U:H2'	7:A:556:C:C6	2.54	0.43
7:A:1086:U:H3	7:A:1099:G:N2	2.17	0.43
7:A:1253:A:H62	7:A:1286:A:H62	1.67	0.43
7:A:1317:G:N2	7:A:1319:A:H3'	2.34	0.43
7:A:1474:G:H2'	7:A:1475:U:C6	2.54	0.43
10:D:146:ARG:HB3	10:D:152:GLN:HG2	2.01	0.43
11:E:64:MET:HE3	11:E:64:MET:HB3	1.58	0.43
22:P:39:PHE:CZ	22:P:74:LEU:HB2	2.54	0.43
24:R:12:ARG:HD3	24:R:12:ARG:HA	1.72	0.43
28:a:1416:G:O2'	28:a:1417:C:P	2.77	0.43
28:a:1794:A:H2'	28:a:1795:C:C6	2.54	0.43
28:a:2074:U:H2'	28:a:2075:U:C6	2.54	0.43
28:a:2297:A:N6	28:a:2319:G:H21	2.05	0.43

Continued on next page...

For Manuscript Review

Continued from previous page...

Atom-1	Atom-2	Interatomic distance (Å)	Clash overlap (Å)
28:a:2356:U:H4'	49:v:20:ARG:NE	2.18	0.43
34:g:52:PHE:CG	34:g:69:ARG:HA	2.54	0.43
42:o:36:SER:O	42:o:36:SER:OG	2.33	0.43
47:t:100:SER:O	47:t:100:SER:OG	2.27	0.43
50:w:18:ARG:HA	50:w:23:ASN:O	2.17	0.43
1:0:10:LYS:HG2	1:0:54:ILE:HA	2.00	0.42
4:3:12:ARG:NH2	4:3:13:ASN:HA	2.34	0.42
7:A:599:C:H2'	7:A:600:A:C8	2.40	0.42
7:A:1347:A:H2'	13:G:10:ARG:NH2	2.34	0.42
7:A:1533:U:H1'	7:A:1534:C:H3'	2.01	0.42
8:B:152:LYS:NZ	8:B:153:ASP:OD1	2.37	0.42
16:J:32:THR:HG21	16:J:83:THR:HG22	2.01	0.42
28:a:145:C:H2'	28:a:146:A:H8	1.84	0.42
28:a:1027:A:O2'	28:a:1028:A:O4'	2.29	0.42
28:a:2280:G:H4'	28:a:2327:A:H1'	2.01	0.42
28:a:2294:G:OP2	41:n:10:ARG:HD2	2.18	0.42
28:a:2364:C:H5"	28:a:2365:G:OP2	2.19	0.42
28:a:2386:A:H5"	49:v:55:ARG:HH22	1.84	0.42
28:a:2753:A:OP2	28:a:2754:U:H5	2.02	0.42
29:b:91:C:H3'	29:b:92:C:C6	2.54	0.42
29:b:109:A:H2'	29:b:110:C:O4'	2.19	0.42
32:e:187:VAL:C	32:e:188:MET:HE3	2.44	0.42
34:g:7:ALA:HA	34:g:8:PRO:HD3	1.91	0.42
34:g:15:VAL:HG11	34:g:79:VAL:HB	2.01	0.42
36:i:74:TYR:O	36:i:86:GLN:HA	2.19	0.42
44:q:4:VAL:HA	44:q:12:HIS:O	2.19	0.42
50:w:59:ILE:HG12	50:w:67:VAL:HG21	2.01	0.42
3:2:30:ARG:O	28:a:2419:U:H5	2.02	0.42
5:4:36:VAL:HG11	33:f:110:ARG:HA	2.01	0.42
7:A:62:U:H2'	7:A:63:C:C5	2.53	0.42
7:A:178:C:C2	7:A:179:A:C8	3.08	0.42
7:A:1126:U:H1'	7:A:1127:G:H2'	2.00	0.42
7:A:1259:G:H2'	7:A:1260:C:C6	2.53	0.42
7:A:1288:A:H2	7:A:1354:G:H1'	1.84	0.42
8:B:36:ASN:OD1	8:B:36:ASN:O	2.37	0.42
8:B:96:TRP:HE1	8:B:175:GLU:CD	2.27	0.42
9:C:110:GLU:HG2	9:C:144:LEU:HD13	2.00	0.42
10:D:151:LYS:HZ3	10:D:154:ARG:HD3	1.84	0.42
14:H:54:ASP:C	14:H:56:LYS:H	2.27	0.42
16:J:57:VAL:O	16:J:58:ASN:HB2	2.19	0.42
18:L:50:ARG:HB2	18:L:90:LEU:HD11	2.01	0.42

Continued on next page...

For Manuscript Review

Continued from previous page...

Atom-1	Atom-2	Interatomic distance (Å)	Clash overlap (Å)
20:N:92:GLU:OE1	20:N:92:GLU:N	2.52	0.42
28:a:5:A:H2'	28:a:6:A:C8	2.54	0.42
28:a:711:G:O6	28:a:721:A:N6	2.52	0.42
28:a:1042:G:H1	28:a:1113:U:H3	0.65	0.42
28:a:2530:A:O2'	28:a:2535:G:O6	2.33	0.42
32:e:148:ILE:CG2	32:e:169:VAL:HA	2.49	0.42
43:p:70:ARG:HA	43:p:74:ILE:O	2.18	0.42
48:u:25:LYS:HD2	48:u:25:LYS:HA	1.68	0.42
48:u:30:ILE:HG22	48:u:40:ILE:HD13	2.01	0.42
1:0:10:LYS:HE3	1:0:54:ILE:HA	1.99	0.42
4:3:31:PRO:HB2	28:a:2527:C:H4'	2.00	0.42
5:4:28:VAL:HG21	5:4:32:LEU:HD22	2.01	0.42
10:D:50:ASP:O	10:D:54:GLN:HG3	2.19	0.42
10:D:65:TYR:O	10:D:67:VAL:HG12	2.20	0.42
23:Q:38:ILE:H	23:Q:38:ILE:HG13	1.68	0.42
28:a:84:A:H3'	47:t:7:ARG:NH2	2.27	0.42
28:a:1281:G:H2'	28:a:1282:U:C6	2.54	0.42
28:a:1864:U:H2'	28:a:1865:U:N3	2.34	0.42
28:a:2303:G:O3'	33:f:121:SER:HA	2.19	0.42
29:b:9:G:C6	29:b:111:U:O2	2.70	0.42
29:b:100:G:H2'	29:b:101:A:O4'	2.18	0.42
33:f:5:HIS:O	33:f:9:LYS:HG2	2.20	0.42
39:l:62:LYS:HG2	39:l:64:TRP:CH2	2.54	0.42
40:m:38:LEU:HD13	40:m:111:ALA:HB2	2.02	0.42
47:t:83:VAL:HG22	47:t:96:PHE:CD1	2.54	0.42
48:u:42:LEU:HA	48:u:42:LEU:HD22	1.83	0.42
7:A:132:U:H2'	7:A:133:U:C6	2.54	0.42
7:A:383:A:N7	7:A:455:U:H5'	2.33	0.42
7:A:427:U:H2'	7:A:428:G:N2	2.35	0.42
7:A:533:A:O2'	7:A:536:C:N4	2.52	0.42
7:A:599:C:H4'	14:H:122:GLY:O	2.19	0.42
7:A:1067:A:C4	7:A:1109:C:H5"	2.55	0.42
10:D:8:LYS:C	10:D:10:LYS:H	2.27	0.42
17:K:110:ILE:HG22	27:U:19:PHE:CD1	2.55	0.42
20:N:24:ARG:HG3	20:N:51:LEU:HD12	2.00	0.42
20:N:76:LYS:HG3	20:N:77:PHE:N	2.34	0.42
28:a:273:G:C6	28:a:274:C:N4	2.86	0.42
28:a:630:G:H2'	28:a:631:A:H3'	2.01	0.42
28:a:1326:U:H2'	28:a:1327:A:H8	1.83	0.42
28:a:1597:A:H5"	28:a:1598:A:H5'	2.02	0.42
43:p:109:LEU:HD23	44:q:49:ILE:HD13	2.01	0.42

Continued on next page...

For Manuscript Review

Continued from previous page...

Atom-1	Atom-2	Interatomic distance (Å)	Clash overlap (Å)
44:q:26:ASP:O	44:q:27:ILE:HD12	2.20	0.42
47:t:18:ASP:HB3	47:t:21:LYS:HD3	2.01	0.42
49:v:45:PHE:HA	49:v:78:LYS:O	2.19	0.42
53:z:55:ILE:C	53:z:55:ILE:HD12	2.45	0.42
7:A:122:G:H3'	7:A:123:C:H6	1.85	0.42
7:A:609:A:H1'	7:A:610:G:OP1	2.19	0.42
13:G:25:LYS:HD2	13:G:25:LYS:HA	1.88	0.42
13:G:50:LEU:O	13:G:54:SER:HB2	2.19	0.42
13:G:60:GLU:O	13:G:64:VAL:HG23	2.19	0.42
15:I:98:LEU:HD23	15:I:98:LEU:HA	1.80	0.42
15:I:114:LYS:HG2	15:I:120:LYS:O	2.20	0.42
17:K:80:LYS:HE2	17:K:80:LYS:HB2	1.71	0.42
20:N:61:ARG:HA	20:N:61:ARG:HD3	1.95	0.42
25:S:11:ILE:HG23	25:S:38:SER:HA	2.01	0.42
25:S:29:LYS:HA	25:S:29:LYS:HD2	1.87	0.42
28:a:661:A:C2'	28:a:662:G:H5'	2.43	0.42
28:a:851:C:H2'	28:a:852:U:C6	2.54	0.42
28:a:2384:U:O2'	28:a:2385:C:H5'	2.20	0.42
28:a:2528:U:H2'	28:a:2530:A:H8	1.85	0.42
32:e:22:ASP:CG	32:e:23:PHE:H	2.27	0.42
32:e:194:LYS:HA	32:e:194:LYS:HD3	1.83	0.42
33:f:4:LEU:HG	33:f:101:GLU:HB2	2.02	0.42
43:p:94:ILE:HD12	44:q:4:VAL:CG2	2.50	0.42
44:q:59:ILE:CG2	44:q:98:ILE:HD12	2.50	0.42
7:A:222:U:O2'	7:A:223:A:OP1	2.32	0.42
7:A:437:U:O4'	10:D:154:ARG:NH2	2.45	0.42
7:A:488:C:O2'	7:A:489:C:H5'	2.19	0.42
7:A:596:A:C5	7:A:597:G:C2	3.07	0.42
13:G:131:LYS:HA	13:G:135:VAL:HG11	2.01	0.42
16:J:17:LEU:HD12	16:J:17:LEU:HA	1.70	0.42
19:M:12:HIS:CD2	19:M:12:HIS:C	2.97	0.42
27:U:51:SER:O	27:U:55:ARG:HG3	2.19	0.42
28:a:268:C:C2	28:a:425:G:N2	2.88	0.42
28:a:636:G:OP1	38:k:129:LYS:HG3	2.19	0.42
28:a:2379:G:H5'	41:n:21:LEU:HD21	2.01	0.42
28:a:2741:A:H2'	28:a:2742:G:O4'	2.20	0.42
28:a:2820:A:N3	28:a:2820:A:H2'	2.34	0.42
33:f:73:SER:HB3	33:f:80:ARG:HH21	1.83	0.42
33:f:164:GLU:OE2	33:f:165:GLU:HG2	2.20	0.42
43:p:69:ALA:HB2	43:p:106:PHE:CZ	2.48	0.42
44:q:25:LEU:HD21	44:q:33:VAL:HG21	2.02	0.42

Continued on next page...

For Manuscript Review

Continued from previous page...

Atom-1	Atom-2	Interatomic distance (Å)	Clash overlap (Å)
48:u:40:ILE:HD11	48:u:91:PHE:CD2	2.54	0.42
49:v:81:SER:OG	49:v:82:ILE:N	2.52	0.42
7:A:539:A:H2'	7:A:540:G:C8	2.55	0.42
7:A:778:G:HO2'	7:A:779:C:P	2.42	0.42
7:A:1028:C:H2'	7:A:1029:U:C6	2.55	0.42
8:B:109:GLN:H	8:B:109:GLN:HG3	1.64	0.42
11:E:66:LYS:HB3	11:E:66:LYS:HE2	1.82	0.42
19:M:64:VAL:O	19:M:69:LEU:HG	2.19	0.42
28:a:660:C:N3	28:a:661:A:C6	2.87	0.42
28:a:722:A:H2'	28:a:723:C:C6	2.54	0.42
28:a:1021:A:O3'	28:a:1022:G:H4'	2.19	0.42
28:a:1864:U:H2'	28:a:1865:U:C4	2.55	0.42
28:a:2327:A:C2	28:a:2328:A:C8	3.08	0.42
28:a:2747:G:H5'	34:g:138:LYS:HZ3	1.84	0.42
33:f:95:ARG:HB3	33:f:95:ARG:HE	1.77	0.42
49:v:46:HIS:HB2	49:v:79:PHE:CD1	2.54	0.42
52:y:38:ARG:HA	52:y:38:ARG:HE	1.84	0.42
7:A:193:C:H2'	7:A:194:C:C6	2.55	0.42
7:A:1109:C:H2'	7:A:1110:A:O4'	2.20	0.42
7:A:1188:G:OP1	7:A:1188:G:H4'	2.20	0.42
7:A:1228:A:P	19:M:95:LEU:HD21	2.59	0.42
17:K:26:SER:OG	17:K:27:PHE:N	2.52	0.42
19:M:17:ILE:O	19:M:41:GLU:HG3	2.19	0.42
21:O:70:LEU:HD12	21:O:70:LEU:HA	1.88	0.42
21:O:71:LYS:HB2	21:O:78:TYR:CD1	2.55	0.42
28:a:696:G:O6	57:a:6221:SPM:H81	2.18	0.42
28:a:721:A:H2'	28:a:722:A:C8	2.55	0.42
28:a:1050:A:N6	28:a:1109:C:H42	2.18	0.42
31:d:36:GLN:N	31:d:49:GLN:O	2.52	0.42
36:i:15:TRP:CD1	36:i:53:TYR:HB2	2.55	0.42
36:i:119:PHE:CD1	36:i:122:LEU:HB3	2.55	0.42
37:j:44:LYS:HA	37:j:44:LYS:HD2	1.77	0.42
38:k:20:GLY:HA2	38:k:28:GLY:HA2	2.01	0.42
43:p:68:ALA:C	43:p:106:PHE:HE2	2.28	0.42
49:v:72:LYS:CG	49:v:79:PHE:HB2	2.50	0.42
7:A:447:G:O2'	7:A:448:A:OP1	2.29	0.42
7:A:1104:G:P	7:A:1104:G:O4'	2.78	0.42
7:A:1161:G:C6	7:A:1162:C:C5	3.08	0.42
9:C:74:GLY:C	9:C:76:VAL:H	2.26	0.42
10:D:19:LEU:HB3	10:D:21:LEU:HG	2.02	0.42
11:E:112:ARG:H	11:E:112:ARG:HG2	1.32	0.42

Continued on next page...

For Manuscript Review

Continued from previous page...

Atom-1	Atom-2	Interatomic distance (Å)	Clash overlap (Å)
13:G:126:ASP:O	13:G:131:LYS:HD3	2.20	0.42
16:J:7:ARG:HG2	16:J:101:SER:HB2	2.01	0.42
17:K:93:ARG:O	17:K:97:ILE:HG12	2.20	0.42
19:M:54:ASP:HA	19:M:57:ARG:NE	2.25	0.42
22:P:46:LYS:HE2	22:P:46:LYS:HB2	1.79	0.42
24:R:50:LYS:HA	24:R:53:ARG:HH12	1.85	0.42
28:a:424:G:N3	28:a:425:G:H1'	2.34	0.42
28:a:841:G:C4	28:a:842:U:C6	3.07	0.42
28:a:1019:U:O2'	28:a:1020:A:H5'	2.20	0.42
28:a:2293:G:H4'	41:n:98:GLN:OE1	2.19	0.42
28:a:2404:U:H2'	28:a:2405:G:O4'	2.20	0.42
30:c:124:ILE:O	30:c:125:LYS:HG3	2.20	0.42
48:u:57:TYR:O	48:u:74:ALA:HB3	2.19	0.42
1:0:20:PHE:CE2	28:a:2419:U:H1'	2.55	0.42
4:3:25:VAL:O	4:3:34:LYS:HA	2.20	0.42
7:A:609:A:H8	7:A:609:A:OP2	2.02	0.42
7:A:1192:A:OP2	9:C:4:LYS:HG3	2.19	0.42
8:B:115:LYS:HG3	8:B:116:ASP:N	2.35	0.42
9:C:19:ASN:O	9:C:20:SER:OG	2.32	0.42
10:D:99:ASP:OD2	10:D:115:ARG:HG2	2.20	0.42
28:a:2355:G:H2'	28:a:2356:U:N1	2.35	0.42
28:a:2514:U:H2'	28:a:2515:C:C6	2.55	0.42
28:a:2647:U:H2'	28:a:2648:G:H8	1.85	0.42
28:a:2742:G:H2'	28:a:2743:U:O4'	2.20	0.42
29:b:24:G:N3	29:b:26:C:N4	2.68	0.42
31:d:139:SER:O	31:d:139:SER:OG	2.34	0.42
32:e:23:PHE:HE2	32:e:25:GLU:HG3	1.85	0.42
38:k:135:ILE:O	38:k:140:GLY:N	2.37	0.42
41:n:31:THR:HB	41:n:34:HIS:O	2.19	0.42
48:u:25:LYS:HE2	48:u:41:GLU:O	2.20	0.42
4:3:23:ILE:O	4:3:37:GLN:HG3	2.20	0.41
7:A:1027:C:H2'	7:A:1028:C:C6	2.55	0.41
8:B:165:ASP:HA	8:B:204:ASP:OD1	2.20	0.41
9:C:62:LYS:N	9:C:62:LYS:HD3	2.34	0.41
13:G:15:ASP:HA	13:G:16:PRO:HD3	1.83	0.41
13:G:149:LYS:HD2	13:G:149:LYS:HA	1.87	0.41
15:I:54:LEU:H	15:I:54:LEU:HG	1.53	0.41
15:I:95:ARG:HD3	15:I:95:ARG:H	1.84	0.41
18:L:89:D2T:H7	18:L:89:D2T:H4	1.91	0.41
28:a:172:A:H2'	28:a:173:A:H8	1.85	0.41
28:a:844:A:C2	28:a:935:C:C2	3.08	0.41

Continued on next page...

For Manuscript Review

Continued from previous page...

Atom-1	Atom-2	Interatomic distance (Å)	Clash overlap (Å)
28:a:1027:A:N6	28:a:1126:A:C6	2.88	0.41
28:a:2271:G:H5'	49:v:20:ARG:HD3	2.00	0.41
28:a:2327:A:C6	28:a:2388:A:N1	2.88	0.41
29:b:94:A:P	48:u:19:ARG:HH21	2.42	0.41
29:b:112:G:H2'	29:b:113:C:C6	2.55	0.41
34:g:4:VAL:O	34:g:69:ARG:HG2	2.20	0.41
34:g:50:LEU:HD12	34:g:52:PHE:CZ	2.55	0.41
35:h:12:LEU:O	35:h:12:LEU:HD13	2.20	0.41
43:p:92:ARG:HA	43:p:95:LEU:HD12	2.00	0.41
49:v:65:GLY:CA	49:v:85:GLU:H	2.32	0.41
52:y:56:LYS:HB3	52:y:56:LYS:HE2	1.73	0.41
1:0:38:LYS:C	1:0:46:HIS:HD2	2.28	0.41
7:A:690:G:OP2	17:K:29:ASN:ND2	2.40	0.41
7:A:1071:C:H2'	7:A:1072:G:C8	2.54	0.41
7:A:1411:A:H2'	7:A:1412:C:C6	2.55	0.41
9:C:90:VAL:O	9:C:94:ILE:HD12	2.19	0.41
11:E:59:ALA:O	11:E:61:GLN:HG2	2.20	0.41
16:J:51:VAL:HG11	20:N:84:VAL:HG21	2.02	0.41
25:S:30:PRO:HD3	25:S:48:THR:HB	2.02	0.41
28:a:270:A:C2	28:a:370:G:C4	3.08	0.41
28:a:1023:U:C5	28:a:1024:G:C8	3.08	0.41
28:a:1108:U:C4	28:a:1109:C:H5	2.39	0.41
28:a:2047:C:H2'	28:a:2048:G:H8	1.85	0.41
28:a:2243:U:H2'	28:a:2244:U:C6	2.55	0.41
28:a:2557:G:H2'	28:a:2558:C:H6	1.85	0.41
29:b:91:C:H3'	29:b:92:C:H6	1.85	0.41
32:e:1:MET:SD	32:e:1:MET:N	2.81	0.41
7:A:143:A:N1	7:A:220:G:O6	2.53	0.41
7:A:259:G:C5	7:A:260:G:H1'	2.55	0.41
7:A:373:A:N6	7:A:391:G:H1'	2.34	0.41
7:A:1017:C:H5"	7:A:1017:C:C6	2.56	0.41
9:C:40:ARG:NH1	20:N:92:GLU:HG3	2.35	0.41
9:C:112:ASP:O	9:C:114:LYS:N	2.54	0.41
12:F:29:ILE:HG12	12:F:34:GLY:HA3	2.02	0.41
13:G:140:ASP:CB	13:G:143:ARG:HH21	2.33	0.41
17:K:75:LYS:HD2	17:K:75:LYS:HA	1.73	0.41
19:M:86:TYR:HD1	19:M:86:TYR:HA	1.80	0.41
25:S:66:MET:O	25:S:66:MET:HG2	2.19	0.41
28:a:86:G:N3	28:a:87:U:C5	2.88	0.41
28:a:641:U:H3'	28:a:642:U:C6	2.55	0.41
28:a:842:U:C2	28:a:843:G:C8	3.08	0.41

Continued on next page...

For Manuscript Review

Continued from previous page...

Atom-1	Atom-2	Interatomic distance (Å)	Clash overlap (Å)
28:a:1862:G:C2	28:a:1863:G:N7	2.89	0.41
28:a:1923:U:H2'	28:a:1924:C:C6	2.55	0.41
33:f:52:ASN:ND2	33:f:147:ASP:OD2	2.49	0.41
34:g:72:LEU:O	34:g:76:VAL:HG22	2.20	0.41
44:q:48:LYS:HE3	44:q:48:LYS:HB3	1.84	0.41
48:u:79:ARG:HA	48:u:87:GLN:N	2.34	0.41
5:4:12:ILE:HG12	5:4:26:SER:OG	2.20	0.41
7:A:131:A:N6	7:A:232:G:H21	2.18	0.41
7:A:718:A:H2	24:R:38:LYS:NZ	2.17	0.41
7:A:1080:A:OP2	11:E:50:TYR:OH	2.32	0.41
10:D:64:ILE:HG22	10:D:65:TYR:CD1	2.55	0.41
22:P:6:LEU:HD11	22:P:39:PHE:CD2	2.56	0.41
28:a:6:A:H2'	28:a:7:G:C8	2.55	0.41
28:a:1049:C:H5	28:a:1110:G:N2	2.19	0.41
28:a:1126:A:H4'	28:a:1127:A:O4'	2.20	0.41
28:a:2324:U:H5"	28:a:2326:C:OP2	2.20	0.41
28:a:2343:U:HO2'	28:a:2374:C:H4'	1.85	0.41
28:a:2355:G:C2	28:a:2363:G:C6	3.08	0.41
28:a:2419:U:N3	28:a:2420:C:C4	2.88	0.41
29:b:33:G:O5'	29:b:33:G:H8	2.03	0.41
32:e:125:SER:OG	32:e:126:VAL:N	2.54	0.41
34:g:152:ARG:HG3	34:g:162:VAL:HG13	2.02	0.41
35:h:14:SER:HB3	35:h:15:LEU:H	1.74	0.41
37:j:58:LEU:HD12	37:j:86:LEU:HD13	2.00	0.41
47:t:58:ILE:HD12	47:t:58:ILE:C	2.46	0.41
2:1:4:THR:HG22	28:a:687:C:H1'	2.01	0.41
3:2:17:THR:HA	28:a:629:G:OP2	2.20	0.41
4:3:34:LYS:HB3	4:3:34:LYS:HE2	1.92	0.41
7:A:183:C:H5'	7:A:184:C:OP1	2.20	0.41
7:A:1059:C:H4'	20:N:85:ARG:HH21	1.82	0.41
7:A:1264:A:N6	7:A:1274:G:H1'	2.33	0.41
8:B:184:PHE:CB	8:B:198:PHE:HB2	2.47	0.41
17:K:52:PHE:HA	17:K:56:ARG:NH1	2.35	0.41
18:L:3:THR:OG1	18:L:6:GLN:HG3	2.20	0.41
28:a:7:G:H4'	36:i:15:TRP:HZ2	1.85	0.41
28:a:1432:G:H2'	28:a:1433:A:C8	2.55	0.41
28:a:1486:U:H2'	28:a:1487:U:H6	1.85	0.41
28:a:1570:A:H2'	28:a:1571:A:C8	2.55	0.41
28:a:2295:C:O2'	28:a:2296:U:H5'	2.20	0.41
28:a:2299:U:O5'	28:a:2299:U:H6	2.03	0.41
28:a:2419:U:N3	28:a:2420:C:C5	2.89	0.41

Continued on next page...

For Manuscript Review

Continued from previous page...

Atom-1	Atom-2	Interatomic distance (Å)	Clash overlap (Å)
28:a:2469:A:H4'	39:l:55:ARG:HD3	2.00	0.41
28:a:2519:U:C4	28:a:2541:A:C6	3.08	0.41
36:i:1:MET:CE	44:q:13:ARG:H	2.29	0.41
7:A:473:A:H5"	22:P:76:LYS:NZ	2.36	0.41
8:B:66:LYS:HD2	8:B:66:LYS:N	2.36	0.41
9:C:140:ASN:OD1	9:C:143:ARG:NH1	2.53	0.41
11:E:87:GLY:N	11:E:94:VAL:HG12	2.36	0.41
15:I:52:LEU:H	15:I:52:LEU:HD12	1.84	0.41
17:K:111:THR:HG22	17:K:112:ASP:H	1.85	0.41
20:N:31:ILE:HD12	20:N:41:ARG:HE	1.86	0.41
22:P:19:VAL:HG23	22:P:39:PHE:HB2	2.02	0.41
25:S:25:SER:O	25:S:25:SER:OG	2.33	0.41
28:a:639:U:H2'	28:a:640:C:C6	2.55	0.41
28:a:934:U:H2'	28:a:935:C:C6	2.54	0.41
28:a:1715:G:O2'	28:a:1743:G:O6	2.28	0.41
28:a:2345:G:C2	28:a:2382:G:N7	2.89	0.41
28:a:2365:G:H5'	49:v:58:THR:HG21	2.03	0.41
29:b:22:U:O2	29:b:61:G:O6	2.39	0.41
29:b:29:A:H2'	29:b:30:C:C6	2.55	0.41
32:e:147:LEU:O	32:e:187:VAL:HG12	2.20	0.41
33:f:117:LEU:O	33:f:178:ARG:HG2	2.20	0.41
36:i:32:LEU:HD13	36:i:54:ILE:HD11	2.02	0.41
39:l:36:VAL:HG13	48:u:82:TYR:O	2.20	0.41
43:p:64:ARG:HD3	43:p:100:VAL:HG21	2.02	0.41
43:p:83:LEU:HA	43:p:112:LYS:HZ2	1.85	0.41
44:q:16:GLU:CD	44:q:16:GLU:H	2.28	0.41
48:u:50:MET:HE2	48:u:50:MET:N	2.35	0.41
1:0:26:ASN:ND2	1:0:29:THR:OG1	2.54	0.41
7:A:1162:C:C2'	7:A:1163:C:H5'	2.50	0.41
7:A:1458:G:C2	7:A:1459:G:C8	3.09	0.41
9:C:100:GLN:HB3	9:C:102:ASN:OD1	2.20	0.41
10:D:168:PRO:HB2	10:D:170:TRP:CE2	2.56	0.41
11:E:41:ASP:OD1	11:E:41:ASP:C	2.63	0.41
14:H:11:LEU:HD22	14:H:75:ILE:HG12	2.02	0.41
19:M:98:ARG:HD3	19:M:99:GLY:N	2.36	0.41
21:O:78:TYR:O	21:O:82:ILE:HG23	2.21	0.41
25:S:10:PHE:CD1	25:S:11:ILE:HB	2.56	0.41
25:S:64:ASP:HB3	25:S:65:GLU:H	1.74	0.41
28:a:2044:C:H2'	28:a:2045:C:O4'	2.21	0.41
28:a:2351:G:N2	28:a:2366:A:H5"	2.34	0.41
28:a:2527:C:H3'	28:a:2528:U:C5	2.55	0.41

Continued on next page...

For Manuscript Review

Continued from previous page...

Atom-1	Atom-2	Interatomic distance (Å)	Clash overlap (Å)
29:b:92:C:P	48:u:18:ARG:HH22	2.34	0.41
29:b:94:A:H5”	48:u:19:ARG:NH2	2.35	0.41
31:d:46:ARG:NH2	31:d:86:GLU:O	2.54	0.41
33:f:60:ILE:O	33:f:102:ARG:NH2	2.52	0.41
36:i:102:GLU:HA	36:i:102:GLU:OE2	2.20	0.41
39:l:112:LEU:O	39:l:115:GLU:HB3	2.21	0.41
7:A:285:C:H2’	7:A:286:C:C6	2.56	0.41
7:A:414:A:N6	7:A:431:A:N3	2.69	0.41
7:A:1010:G:H1	7:A:1019:C:H42	1.69	0.41
7:A:1013:G:O6	7:A:1017:C:H5’	2.20	0.41
7:A:1037:C:H4’	7:A:1038:U:H5’	2.03	0.41
7:A:1279:G:H5’	7:A:1280:A:H5’	2.02	0.41
9:C:12:LEU:HD13	9:C:12:LEU:HA	1.91	0.41
9:C:147:LYS:HB2	9:C:203:PHE:CE2	2.56	0.41
10:D:183:LYS:N	10:D:183:LYS:HD2	2.35	0.41
14:H:113:ASP:N	14:H:113:ASP:OD1	2.45	0.41
19:M:113:ARG:HD3	19:M:113:ARG:N	2.31	0.41
28:a:75:G:N3	28:a:75:G:H2’	2.36	0.41
28:a:359:G:C5	28:a:360:U:C4	3.09	0.41
28:a:635:C:O2’	28:a:639:U:H5’	2.20	0.41
28:a:1866:A:C5	28:a:1867:G:H1’	2.55	0.41
28:a:2801:G:H2’	28:a:2802:G:C8	2.55	0.41
29:b:66:A:H2’	29:b:66:A:N3	2.35	0.41
31:d:47:ALA:HB2	31:d:83:ARG:HD2	2.01	0.41
33:f:78:LYS:HE2	33:f:78:LYS:HB2	1.91	0.41
43:p:79:PHE:CE1	43:p:109:LEU:HB3	2.56	0.41
3:2:32:ILE:HA	28:a:2420:C:OP2	2.21	0.41
4:3:2:LYS:HB2	4:3:35:GLN:HG2	2.02	0.41
5:4:34:LEU:HD12	5:4:34:LEU:HA	1.95	0.41
5:4:41:HIS:NE2	33:f:114:PHE:HB3	2.36	0.41
7:A:91:U:H5’	7:A:92:U:C6	2.56	0.41
7:A:229:U:H2’	7:A:230:A:O4’	2.21	0.41
7:A:378:G:O2’	7:A:379:C:H6	2.04	0.41
7:A:490:U:C2	7:A:491:A:C8	3.08	0.41
7:A:621:A:H2’	7:A:622:A:C8	2.56	0.41
7:A:707:U:H4’	17:K:22:HIS:CD2	2.56	0.41
7:A:877:G:HO2’	7:A:878:A:H8	1.68	0.41
7:A:1149:U:O2’	7:A:1150:C:O5’	2.37	0.41
7:A:1219:U:OP2	20:N:9:ARG:NH1	2.54	0.41
7:A:1350:A:H5”	15:I:123:ARG:HB2	2.03	0.41
7:A:1437:C:H2’	7:A:1438:A:H8	1.86	0.41

Continued on next page...

For Manuscript Review

Continued from previous page...

Atom-1	Atom-2	Interatomic distance (Å)	Clash overlap (Å)
8:B:177:ASN:OD1	8:B:178:ASN:N	2.53	0.41
10:D:8:LYS:HG3	10:D:9:LEU:N	2.36	0.41
10:D:171:LEU:HA	10:D:181:THR:O	2.21	0.41
12:F:25:TYR:HD1	12:F:25:TYR:HA	1.70	0.41
12:F:42:TRP:CZ3	12:F:102:MET:HE2	2.56	0.41
12:F:44:ARG:NH1	12:F:58:HIS:HD2	2.18	0.41
12:F:68:GLN:OE1	12:F:68:GLN:N	2.54	0.41
17:K:97:ILE:HD12	27:U:12:PHE:CE1	2.55	0.41
19:M:33:ILE:HD11	19:M:59:GLU:O	2.21	0.41
22:P:52:LEU:HG	22:P:75:ILE:HG22	2.03	0.41
24:R:29:LEU:O	24:R:33:ILE:HG23	2.21	0.41
28:a:274:C:H2'	28:a:275:C:O4'	2.20	0.41
28:a:280:U:C4	28:a:361:G:N2	2.89	0.41
28:a:356:G:H2'	28:a:357:C:C6	2.56	0.41
28:a:633:A:N7	28:a:634:C:C2	2.89	0.41
28:a:634:C:N4	28:a:635:C:N4	2.69	0.41
28:a:937:C:C2	28:a:938:G:H1'	2.56	0.41
28:a:938:G:N1	28:a:939:G:C5	2.89	0.41
28:a:948:C:H1'	28:a:984:A:C8	2.56	0.41
28:a:1036:G:O6	28:a:1119:U:C2	2.71	0.41
28:a:1149:G:H2'	28:a:1150:C:C6	2.55	0.41
28:a:1319:C:O2'	28:a:1320:C:H5'	2.21	0.41
28:a:1880:U:N3	28:a:1881:C:C5	2.88	0.41
28:a:2356:U:N3	28:a:2357:G:C8	2.89	0.41
28:a:2567:G:H2'	28:a:2568:U:C6	2.56	0.41
28:a:2741:A:C2'	28:a:2742:G:C8	2.99	0.41
28:a:2747:G:C6	28:a:2756:U:OP1	2.73	0.41
28:a:2897:U:H2'	28:a:2898:U:C6	2.56	0.41
29:b:78:A:H62	29:b:98:G:H21	0.51	0.41
31:d:51:THR:HA	31:d:80:TRP:CZ3	2.56	0.41
33:f:29:PRO:HB2	33:f:169:LEU:HD22	2.02	0.41
34:g:25:THR:HA	34:g:34:THR:HA	2.02	0.41
34:g:54:PRO:HG3	34:g:62:TRP:CG	2.56	0.41
34:g:118:PRO:HB2	34:g:121:ILE:HD13	2.03	0.41
34:g:154:PRO:HA	34:g:160:LYS:O	2.20	0.41
36:i:1:MET:SD	36:i:1:MET:C	3.04	0.41
36:i:88:THR:OG1	36:i:91:GLU:OE2	2.28	0.41
37:j:90:ASN:OD1	37:j:90:ASN:N	2.54	0.41
41:n:31:THR:HG22	41:n:34:HIS:H	1.86	0.41
43:p:79:PHE:HE1	43:p:109:LEU:C	2.29	0.41
1:0:14:SER:N	1:0:48:ILE:O	2.54	0.41

Continued on next page...

For Manuscript Review

Continued from previous page...

Atom-1	Atom-2	Interatomic distance (Å)	Clash overlap (Å)
1:0:47:VAL:HG23	1:0:49:TYR:CE1	2.56	0.41
7:A:1254:G:H22	7:A:1356:A:H4'	1.85	0.41
7:A:1493:A:H3'	7:A:1494:A:C8	2.55	0.41
8:B:13:GLY:HA2	8:B:16:PHE:HD2	1.85	0.41
8:B:100:MET:HE2	8:B:100:MET:HB3	1.96	0.41
17:K:97:ILE:CD1	27:U:16:LEU:HB3	2.51	0.41
23:Q:29:VAL:CG2	23:Q:40:ARG:HH22	2.33	0.41
26:T:28:MET:O	26:T:32:ILE:HG13	2.21	0.41
28:a:2419:U:C4	28:a:2420:C:N4	2.89	0.41
28:a:2849:U:H4'	28:a:2868:A:C2	2.56	0.41
33:f:29:PRO:HG3	33:f:165:GLU:HB3	2.02	0.41
40:m:24:MET:HE2	40:m:24:MET:HB2	1.91	0.41
44:q:41:ILE:HD13	44:q:103:ALA:HB1	2.03	0.41
47:t:7:ARG:C	47:t:8:ASP:OD1	2.64	0.41
49:v:46:HIS:HB2	49:v:79:PHE:CE2	2.56	0.41
7:A:61:G:H22	7:A:105:G:N2	2.18	0.40
7:A:500:G:H5"	18:L:121:ARG:HH12	1.87	0.40
7:A:836:G:OP1	24:R:51:TYR:OH	2.38	0.40
7:A:1067:A:C6	7:A:1109:C:H5"	2.56	0.40
9:C:107:ARG:NE	9:C:107:ARG:HA	2.37	0.40
18:L:74:LEU:HD12	18:L:74:LEU:HA	1.85	0.40
20:N:76:LYS:HG3	20:N:77:PHE:H	1.85	0.40
26:T:79:LEU:HD13	26:T:79:LEU:HA	1.90	0.40
28:a:2291:U:H4'	28:a:2379:G:H21	1.87	0.40
28:a:2487:G:H2'	28:a:2488:G:C8	2.56	0.40
28:a:2661:G:H8	28:a:2661:G:OP2	2.03	0.40
28:a:2758:A:N3	34:g:35:ARG:CZ	2.84	0.40
29:b:60:C:O5'	29:b:60:C:H6	2.04	0.40
32:e:23:PHE:HB2	32:e:111:GLU:OE1	2.20	0.40
32:e:119:ILE:O	32:e:119:ILE:HG13	2.21	0.40
34:g:134:LYS:NZ	34:g:134:LYS:HB3	2.36	0.40
36:i:124:VAL:HG12	36:i:125:TYR:N	2.36	0.40
47:t:51:ALA:O	47:t:52:LEU:HD13	2.22	0.40
48:u:83:LYS:C	48:u:85:LYS:H	2.29	0.40
50:w:2:SER:HB2	50:w:3:ARG:H	1.59	0.40
7:A:143:A:H2	7:A:220:G:H22	1.69	0.40
7:A:465:A:N6	7:A:466:A:N3	2.70	0.40
7:A:715:A:H2'	7:A:716:A:C8	2.57	0.40
7:A:1134:C:H2'	7:A:1135:G:C8	2.55	0.40
7:A:1187:G:OP1	15:I:115:LYS:NZ	2.30	0.40
10:D:56:ARG:HA	10:D:56:ARG:HD3	1.72	0.40

Continued on next page...

For Manuscript Review

Continued from previous page...

Atom-1	Atom-2	Interatomic distance (Å)	Clash overlap (Å)
13:G:63:GLU:HA	13:G:66:LEU:CD2	2.51	0.40
15:I:90:TYR:C	15:I:90:TYR:CD2	3.00	0.40
16:J:37:ARG:HD3	16:J:37:ARG:HA	1.76	0.40
19:M:18:ALA:HB1	19:M:19:LEU:HD22	2.03	0.40
21:O:17:ARG:HE	21:O:17:ARG:HB3	1.55	0.40
28:a:627:A:H2	28:a:636:G:H2'	1.86	0.40
28:a:851:C:H2'	28:a:852:U:H6	1.86	0.40
28:a:1419:A:O2'	28:a:1421:G:N7	2.49	0.40
28:a:1583:A:HO2'	28:a:1585:C:N4	2.08	0.40
28:a:2323:G:H2'	28:a:2324:U:O4'	2.21	0.40
28:a:2537:U:H2'	28:a:2538:C:H6	1.86	0.40
30:c:175:ARG:NH1	30:c:181:MET:HE1	2.36	0.40
31:d:17:GLU:CD	31:d:17:GLU:N	2.79	0.40
33:f:26:MET:N	33:f:26:MET:SD	2.93	0.40
41:n:7:ARG:HG2	41:n:96:GLY:HA3	2.03	0.40
49:v:37:ILE:HG22	49:v:38:VAL:HG13	2.01	0.40
2:1:1:MET:HE3	2:1:1:MET:H1	1.85	0.40
4:3:25:VAL:N	4:3:35:GLN:O	2.46	0.40
7:A:22:G:O2'	7:A:885:G:H8	2.04	0.40
7:A:411:A:H5"	7:A:411:A:H8	1.87	0.40
7:A:1002:A:H2'	7:A:1002:A:N3	2.36	0.40
7:A:1350:A:P	15:I:121:ALA:HB3	2.61	0.40
12:F:90:MET:SD	12:F:91:ARG:N	2.94	0.40
16:J:6:ILE:HG22	16:J:102:LEU:HA	2.04	0.40
16:J:39:PRO:HA	16:J:73:LEU:O	2.22	0.40
19:M:56:LEU:C	19:M:58:ASP:H	2.29	0.40
27:U:66:ARG:HD3	27:U:67:ARG:HH12	1.87	0.40
28:a:269:C:C5	28:a:424:G:N1	2.90	0.40
28:a:897:C:H2'	28:a:898:C:C6	2.55	0.40
28:a:2273:A:H2'	28:a:2274:A:C8	2.57	0.40
31:d:29:VAL:O	31:d:185:ASN:HB3	2.21	0.40
39:l:126:ILE:H	39:l:126:ILE:HG13	1.83	0.40
41:n:64:TYR:HD1	41:n:65:THR:H	1.69	0.40
43:p:61:TRP:CZ2	43:p:93:LYS:HB2	2.56	0.40
44:q:4:VAL:HG12	44:q:5:PHE:N	2.36	0.40
47:t:39:ILE:HD13	47:t:39:ILE:HA	1.85	0.40
48:u:45:ASP:O	48:u:49:ASN:ND2	2.54	0.40
48:u:72:VAL:HA	48:u:92:VAL:O	2.21	0.40
7:A:86:G:OP2	7:A:86:G:H3'	2.22	0.40
7:A:462:G:H3'	7:A:463:C:C6	2.56	0.40
7:A:1012:G:H3'	7:A:1014:A:O3'	2.22	0.40

Continued on next page...

For Manuscript Review

Continued from previous page...

Atom-1	Atom-2	Interatomic distance (Å)	Clash overlap (Å)
8:B:28:LYS:N	8:B:29:PRO:HD2	2.37	0.40
9:C:37:PHE:CE2	20:N:66:GLN:HG2	2.56	0.40
10:D:35:GLU:HB3	10:D:36:GLN:H	1.57	0.40
10:D:164:GLN:O	10:D:165:ARG:HG3	2.21	0.40
12:F:53:LYS:HE2	12:F:53:LYS:HB2	1.61	0.40
17:K:84:VAL:HB	17:K:110:ILE:HG13	2.03	0.40
19:M:3:ARG:HB3	19:M:54:ASP:CG	2.46	0.40
28:a:660:C:C2	28:a:661:A:C5	3.09	0.40
28:a:1183:U:H4'	52:y:30:ARG:HD2	2.03	0.40
28:a:2455:G:H2'	28:a:2456:C:C6	2.57	0.40
28:a:2488:G:H2'	28:a:2489:U:C6	2.56	0.40
28:a:2741:A:C2'	28:a:2742:G:H5'	2.51	0.40
33:f:35:THR:C	33:f:36:LEU:HD13	2.47	0.40
33:f:170:LEU:HD13	33:f:175:PHE:CE2	2.56	0.40
34:g:22:GLN:NE2	34:g:39:ASP:O	2.54	0.40
34:g:44:LYS:CG	34:g:45:HIS:H	2.33	0.40
34:g:89:LEU:HB2	34:g:94:TYR:HD2	1.87	0.40
34:g:103:ILE:N	34:g:115:HIS:O	2.39	0.40
37:j:113:MET:H	37:j:113:MET:HE3	1.86	0.40
43:p:95:LEU:O	43:p:98:ILE:HB	2.22	0.40
43:p:108:ALA:HB2	44:q:47:VAL:HG21	2.03	0.40
44:q:38:VAL:HG22	44:q:59:ILE:HD11	2.04	0.40
48:u:26:PHE:CE2	48:u:86:LEU:HB2	2.57	0.40
5:4:9:TYR:HB3	5:4:25:ARG:NE	2.36	0.40
7:A:524:G:H2'	7:A:525:C:C6	2.57	0.40
9:C:167:TRP:CZ3	9:C:169:ARG:HG3	2.55	0.40
10:D:189:SER:C	10:D:191:LEU:H	2.30	0.40
12:F:15:SER:HB2	12:F:44:ARG:HH22	1.87	0.40
14:H:55:THR:O	14:H:56:LYS:HG3	2.22	0.40
15:I:46:MET:HE3	15:I:46:MET:HB3	1.81	0.40
16:J:87:LEU:HA	16:J:90:LEU:HD23	2.03	0.40
19:M:57:ARG:O	19:M:59:GLU:N	2.54	0.40
28:a:96:C:H2'	28:a:97:C:C6	2.56	0.40
28:a:984:A:H2'	28:a:984:A:N3	2.37	0.40
28:a:1028:A:N3	28:a:2487:G:H4'	2.36	0.40
28:a:1029:A:H2'	28:a:1030:C:H6	1.86	0.40
28:a:1418:G:H2'	28:a:1419:A:H5"	2.03	0.40
28:a:1790:C:H2'	28:a:1791:A:N7	2.36	0.40
28:a:2747:G:N2	28:a:2757:A:H62	2.20	0.40
28:a:2757:A:H2	34:g:64:GLN:HA	1.87	0.40
33:f:105:THR:O	33:f:106:ILE:HD13	2.22	0.40

Continued on next page...

For Manuscript Review

Continued from previous page...

Atom-1	Atom-2	Interatomic distance (Å)	Clash overlap (Å)
34:g:44:LYS:HD2	34:g:51:THR:N	2.33	0.40
34:g:67:THR:O	34:g:71:LEU:HG	2.21	0.40
36:i:125:TYR:HB2	36:i:130:HIS:CD2	2.55	0.40
43:p:57:PHE:HD2	43:p:61:TRP:HE1	1.68	0.40
43:p:82:GLY:O	43:p:86:ALA:HB3	2.22	0.40
45:r:23:LEU:HD12	45:r:23:LEU:HA	1.91	0.40
48:u:4:ILE:HD13	48:u:4:ILE:HA	1.97	0.40

There are no symmetry-related clashes.

5.3 Torsion angles (i)

5.3.1 Protein backbone (i)

In the following table, the Percentiles column shows the percent Ramachandran outliers of the chain as a percentile score with respect to all PDB entries followed by that with respect to all EM entries.

The Analysed column shows the number of residues for which the backbone conformation was analysed, and the total number of residues.

Mol	Chain	Analysed	Favoured	Allowed	Outliers	Percentiles	
1	0	49/55 (89%)	47 (96%)	2 (4%)	0	100	100
2	1	44/46 (96%)	43 (98%)	1 (2%)	0	100	100
3	2	62/65 (95%)	56 (90%)	6 (10%)	0	100	100
4	3	36/38 (95%)	30 (83%)	6 (17%)	0	100	100
5	4	56/70 (80%)	49 (88%)	7 (12%)	0	100	100
8	B	222/241 (92%)	196 (88%)	24 (11%)	2 (1%)	14	49
9	C	204/233 (88%)	159 (78%)	40 (20%)	5 (2%)	4	24
10	D	203/206 (98%)	150 (74%)	41 (20%)	12 (6%)	1	7
11	E	154/167 (92%)	129 (84%)	24 (16%)	1 (1%)	22	57
12	F	101/135 (75%)	91 (90%)	10 (10%)	0	100	100
13	G	151/179 (84%)	130 (86%)	21 (14%)	0	100	100
14	H	127/130 (98%)	106 (84%)	21 (16%)	0	100	100
15	I	125/130 (96%)	103 (82%)	22 (18%)	0	100	100
16	J	96/103 (93%)	81 (84%)	12 (12%)	3 (3%)	3	19

Continued on next page...

For Manuscript Review

Continued from previous page...

Mol	Chain	Analysed	Favoured	Allowed	Outliers	Percentiles	
17	K	113/129 (88%)	106 (94%)	7 (6%)	0	100	100
18	L	119/124 (96%)	102 (86%)	17 (14%)	0	100	100
19	M	113/118 (96%)	81 (72%)	30 (26%)	2 (2%)	7	32
20	N	98/101 (97%)	88 (90%)	10 (10%)	0	100	100
21	O	86/89 (97%)	81 (94%)	3 (4%)	2 (2%)	5	26
22	P	79/82 (96%)	59 (75%)	18 (23%)	2 (2%)	4	24
23	Q	77/84 (92%)	67 (87%)	10 (13%)	0	100	100
24	R	64/75 (85%)	55 (86%)	9 (14%)	0	100	100
25	S	82/92 (89%)	57 (70%)	23 (28%)	2 (2%)	5	25
26	T	84/87 (97%)	83 (99%)	1 (1%)	0	100	100
27	U	68/71 (96%)	63 (93%)	5 (7%)	0	100	100
30	c	269/273 (98%)	259 (96%)	10 (4%)	0	100	100
31	d	206/209 (99%)	189 (92%)	17 (8%)	0	100	100
32	e	199/201 (99%)	190 (96%)	9 (4%)	0	100	100
33	f	175/179 (98%)	158 (90%)	17 (10%)	0	100	100
34	g	174/177 (98%)	150 (86%)	23 (13%)	1 (1%)	22	57
35	h	39/149 (26%)	34 (87%)	5 (13%)	0	100	100
36	i	140/142 (99%)	131 (94%)	9 (6%)	0	100	100
37	j	121/123 (98%)	117 (97%)	4 (3%)	0	100	100
38	k	142/144 (99%)	135 (95%)	7 (5%)	0	100	100
39	l	132/136 (97%)	127 (96%)	5 (4%)	0	100	100
40	m	116/127 (91%)	108 (93%)	8 (7%)	0	100	100
41	n	114/117 (97%)	107 (94%)	7 (6%)	0	100	100
42	o	112/115 (97%)	109 (97%)	3 (3%)	0	100	100
43	p	115/118 (98%)	106 (92%)	9 (8%)	0	100	100
44	q	101/103 (98%)	89 (88%)	12 (12%)	0	100	100
45	r	108/110 (98%)	105 (97%)	3 (3%)	0	100	100
46	s	91/100 (91%)	83 (91%)	8 (9%)	0	100	100
47	t	100/104 (96%)	92 (92%)	8 (8%)	0	100	100
48	u	92/94 (98%)	83 (90%)	9 (10%)	0	100	100
49	v	76/85 (89%)	65 (86%)	11 (14%)	0	100	100

Continued on next page...

For Manuscript Review

Continued from previous page...

Mol	Chain	Analysed	Favoured	Allowed	Outliers	Percentiles	
50	w	75/78 (96%)	74 (99%)	1 (1%)	0	100	100
51	x	60/63 (95%)	58 (97%)	2 (3%)	0	100	100
52	y	56/59 (95%)	55 (98%)	1 (2%)	0	100	100
53	z	54/57 (95%)	54 (100%)	0	0	100	100
All	All	5480/5913 (93%)	4890 (89%)	558 (10%)	32 (1%)	24	57

All (32) Ramachandran outliers are listed below:

Mol	Chain	Res	Type
8	B	154	MET
9	C	26	THR
10	D	34	ILE
10	D	36	GLN
10	D	40	GLN
10	D	45	LYS
19	M	13	LYS
22	P	16	PHE
9	C	82	GLU
10	D	27	ALA
10	D	44	ARG
10	D	123	ILE
19	M	42	ASP
9	C	3	GLN
10	D	35	GLU
10	D	165	ARG
21	O	18	ASP
25	S	12	ASP
34	g	47	ASP
16	J	54	SER
21	O	17	ARG
8	B	74	ARG
9	C	60	PRO
9	C	196	ILE
10	D	43	ALA
10	D	85	ASN
10	D	129	VAL
11	E	90	THR
16	J	78	GLU
22	P	42	ILE
16	J	57	VAL
25	S	29	LYS

For Manuscript Review

5.3.2 Protein sidechains (i)

In the following table, the Percentiles column shows the percent sidechain outliers of the chain as a percentile score with respect to all PDB entries followed by that with respect to all EM entries.

The Analysed column shows the number of residues for which the sidechain conformation was analysed, and the total number of residues.

Mol	Chain	Analysed	Rotameric	Outliers	Percentiles	
1	0	46/49 (94%)	45 (98%)	1 (2%)	47	76
2	1	38/38 (100%)	38 (100%)	0	100	100
3	2	51/52 (98%)	47 (92%)	4 (8%)	10	36
4	3	34/34 (100%)	30 (88%)	4 (12%)	4	19
5	4	55/62 (89%)	53 (96%)	2 (4%)	30	64
8	B	186/199 (94%)	169 (91%)	17 (9%)	7	30
9	C	170/190 (90%)	155 (91%)	15 (9%)	8	31
10	D	171/173 (99%)	152 (89%)	19 (11%)	5	21
11	E	119/126 (94%)	108 (91%)	11 (9%)	7	29
12	F	90/116 (78%)	83 (92%)	7 (8%)	10	36
13	G	126/147 (86%)	112 (89%)	14 (11%)	5	21
14	H	104/105 (99%)	99 (95%)	5 (5%)	21	55
15	I	105/107 (98%)	98 (93%)	7 (7%)	13	43
16	J	86/90 (96%)	73 (85%)	13 (15%)	2	12
17	K	89/98 (91%)	87 (98%)	2 (2%)	47	76
18	L	102/103 (99%)	87 (85%)	15 (15%)	2	12
19	M	93/96 (97%)	86 (92%)	7 (8%)	11	38
20	N	83/84 (99%)	77 (93%)	6 (7%)	12	39
21	O	76/77 (99%)	68 (90%)	8 (10%)	5	23
22	P	65/65 (100%)	60 (92%)	5 (8%)	10	37
23	Q	73/78 (94%)	61 (84%)	12 (16%)	2	9
24	R	57/65 (88%)	56 (98%)	1 (2%)	54	80
25	S	72/79 (91%)	59 (82%)	13 (18%)	1	7
26	T	65/66 (98%)	61 (94%)	4 (6%)	15	45
27	U	60/61 (98%)	58 (97%)	2 (3%)	33	67
30	c	216/218 (99%)	204 (94%)	12 (6%)	17	49

Continued on next page...

For Manuscript Review

Continued from previous page...

Mol	Chain	Analysed	Rotameric	Outliers	Percentiles	
31	d	163/163 (100%)	152 (93%)	11 (7%)	13	43
32	e	165/165 (100%)	157 (95%)	8 (5%)	21	55
33	f	148/150 (99%)	142 (96%)	6 (4%)	26	60
34	g	137/138 (99%)	121 (88%)	16 (12%)	4	19
35	h	32/114 (28%)	32 (100%)	0	100	100
36	i	116/116 (100%)	109 (94%)	7 (6%)	16	47
37	j	104/104 (100%)	96 (92%)	8 (8%)	10	37
38	k	103/103 (100%)	98 (95%)	5 (5%)	21	54
39	l	107/107 (100%)	103 (96%)	4 (4%)	29	63
40	m	98/103 (95%)	93 (95%)	5 (5%)	20	53
41	n	86/87 (99%)	80 (93%)	6 (7%)	12	41
42	o	99/100 (99%)	93 (94%)	6 (6%)	15	46
43	p	89/90 (99%)	81 (91%)	8 (9%)	8	30
44	q	84/84 (100%)	80 (95%)	4 (5%)	21	55
45	r	93/93 (100%)	90 (97%)	3 (3%)	34	67
46	s	80/84 (95%)	73 (91%)	7 (9%)	8	31
47	t	83/85 (98%)	77 (93%)	6 (7%)	12	39
48	u	78/78 (100%)	73 (94%)	5 (6%)	14	44
49	v	58/63 (92%)	53 (91%)	5 (9%)	8	32
50	w	67/68 (98%)	59 (88%)	8 (12%)	4	19
51	x	54/55 (98%)	51 (94%)	3 (6%)	17	49
52	y	48/49 (98%)	46 (96%)	2 (4%)	25	59
53	z	47/48 (98%)	45 (96%)	2 (4%)	25	58
All	All	4571/4825 (95%)	4230 (92%)	341 (8%)	14	38

All (341) residues with a non-rotameric sidechain are listed below:

Mol	Chain	Res	Type
1	0	35	GLU
3	2	6	THR
3	2	29	LEU
3	2	32	ILE
3	2	38	THR
4	3	16	ILE

Continued on next page...

For Manuscript Review

Continued from previous page...

Mol	Chain	Res	Type
4	3	17	VAL
4	3	26	ILE
4	3	28	SER
5	4	6	HIS
5	4	36	VAL
8	B	14	VAL
8	B	38	VAL
8	B	47	VAL
8	B	60	ILE
8	B	64	LYS
8	B	88	ASP
8	B	92	VAL
8	B	101	LEU
8	B	130	THR
8	B	140	GLU
8	B	159	ASP
8	B	163	VAL
8	B	183	VAL
8	B	191	SER
8	B	197	ASP
8	B	207	ILE
8	B	223	GLU
9	C	12	LEU
9	C	14	ILE
9	C	22	TRP
9	C	27	LYS
9	C	33	LEU
9	C	57	ILE
9	C	63	SER
9	C	67	THR
9	C	69	HIS
9	C	112	ASP
9	C	176	HIS
9	C	177	THR
9	C	183	ASP
9	C	187	SER
9	C	195	VAL
10	D	12	SER
10	D	22	LYS
10	D	25	VAL
10	D	26	ARG
10	D	31	LYS

Continued on next page...

For Manuscript Review

Continued from previous page...

Mol	Chain	Res	Type
10	D	33	LYS
10	D	35	GLU
10	D	36	GLN
10	D	40	GLN
10	D	41	HIS
10	D	45	LYS
10	D	47	ARG
10	D	62	ARG
10	D	117	LEU
10	D	119	SER
10	D	185	LYS
10	D	187	GLU
10	D	188	ARG
10	D	195	ILE
11	E	21	VAL
11	E	30	ILE
11	E	39	VAL
11	E	76	LEU
11	E	100	SER
11	E	103	THR
11	E	111	MET
11	E	112	ARG
11	E	132	ASN
11	E	142	ASP
11	E	156	LYS
12	F	22	ILE
12	F	39	LEU
12	F	60	VAL
12	F	61	LEU
12	F	62	MET
12	F	96	VAL
12	F	103	VAL
13	G	12	ILE
13	G	17	LYS
13	G	29	ILE
13	G	42	ILE
13	G	45	SER
13	G	47	LEU
13	G	49	THR
13	G	66	LEU
13	G	70	ARG
13	G	72	THR

Continued on next page...

For Manuscript Review

Continued from previous page...

Mol	Chain	Res	Type
13	G	77	SER
13	G	87	VAL
13	G	94	VAL
13	G	138	ARG
14	H	2	SER
14	H	40	LEU
14	H	41	LYS
14	H	64	LYS
14	H	101	ILE
15	I	11	ARG
15	I	28	ILE
15	I	54	LEU
15	I	72	ILE
15	I	93	SER
15	I	110	GLN
15	I	114	LYS
16	J	17	LEU
16	J	28	THR
16	J	40	ILE
16	J	63	ASP
16	J	64	GLN
16	J	70	HIS
16	J	75	ASP
16	J	76	ILE
16	J	77	VAL
16	J	80	THR
16	J	81	GLU
16	J	85	ASP
16	J	100	ILE
17	K	33	THR
17	K	81	ASN
18	L	8	VAL
18	L	18	LYS
18	L	35	THR
18	L	39	THR
18	L	41	THR
18	L	47	SER
18	L	51	LYS
18	L	77	HIS
18	L	79	VAL
18	L	93	VAL
18	L	94	ARG

Continued on next page...

For Manuscript Review

Continued from previous page...

Mol	Chain	Res	Type
18	L	107	VAL
18	L	108	LYS
18	L	111	LYS
18	L	112	GLN
19	M	4	ILE
19	M	14	HIS
19	M	48	LEU
19	M	54	ASP
19	M	65	VAL
19	M	85	CYS
19	M	113	ARG
20	N	4	GLN
20	N	39	GLU
20	N	45	VAL
20	N	69	ARG
20	N	90	ARG
20	N	100	SER
21	O	14	GLU
21	O	18	ASP
21	O	20	ASN
21	O	21	ASP
21	O	27	VAL
21	O	52	SER
21	O	67	LEU
21	O	77	ARG
22	P	12	LYS
22	P	31	ARG
22	P	61	VAL
22	P	71	VAL
22	P	76	LYS
23	Q	27	ARG
23	Q	29	VAL
23	Q	30	LYS
23	Q	40	ARG
23	Q	41	THR
23	Q	42	THR
23	Q	51	ASN
23	Q	52	GLU
23	Q	55	ILE
23	Q	57	ASP
23	Q	61	ILE
23	Q	70	THR

Continued on next page...

For Manuscript Review

Continued from previous page...

Mol	Chain	Res	Type
24	R	28	THR
25	S	13	LEU
25	S	25	SER
25	S	34	TRP
25	S	38	SER
25	S	39	THR
25	S	40	ILE
25	S	45	ILE
25	S	51	VAL
25	S	56	GLN
25	S	63	THR
25	S	66	MET
25	S	67	VAL
25	S	83	HIS
26	T	6	SER
26	T	23	SER
26	T	36	TYR
26	T	79	LEU
27	U	6	VAL
27	U	13	ASP
30	c	10	SER
30	c	35	GLU
30	c	69	ARG
30	c	78	VAL
30	c	90	ASN
30	c	129	THR
30	c	139	SER
30	c	140	THR
30	c	157	SER
30	c	164	ILE
30	c	194	GLU
30	c	204	VAL
31	d	14	ILE
31	d	24	VAL
31	d	29	VAL
31	d	51	THR
31	d	79	LEU
31	d	94	GLN
31	d	121	THR
31	d	131	ASP
31	d	137	SER
31	d	193	VAL

Continued on next page...

For Manuscript Review

Continued from previous page...

Mol	Chain	Res	Type
31	d	199	SER
32	e	14	VAL
32	e	43	THR
32	e	75	SER
32	e	105	LEU
32	e	116	ASP
32	e	119	ILE
32	e	121	VAL
32	e	150	THR
33	f	5	HIS
33	f	36	LEU
33	f	89	VAL
33	f	108	VAL
33	f	130	MET
33	f	132	VAL
34	g	4	VAL
34	g	9	VAL
34	g	15	VAL
34	g	20	ASN
34	g	24	ILE
34	g	25	THR
34	g	44	LYS
34	g	60	ASP
34	g	67	THR
34	g	89	LEU
34	g	122	THR
34	g	125	CYS
34	g	127	THR
34	g	134	LYS
34	g	140	VAL
34	g	157	TYR
36	i	45	THR
36	i	53	TYR
36	i	54	ILE
36	i	64	VAL
36	i	65	THR
36	i	131	ASN
36	i	139	VAL
37	j	8	LEU
37	j	20	MET
37	j	28	SER
37	j	63	VAL

Continued on next page...

For Manuscript Review

Continued from previous page...

Mol	Chain	Res	Type
37	j	69	VAL
37	j	76	VAL
37	j	91	SER
37	j	110	GLU
38	k	13	LYS
38	k	23	ILE
38	k	67	THR
38	k	77	ILE
38	k	105	ILE
39	l	3	GLN
39	l	78	LEU
39	l	89	VAL
39	l	114	ARG
40	m	10	LEU
40	m	15	SER
40	m	20	MET
40	m	33	ILE
40	m	98	LEU
41	n	49	VAL
41	n	52	SER
41	n	64	TYR
41	n	74	VAL
41	n	83	LEU
41	n	106	LEU
42	o	4	ILE
42	o	12	GLN
42	o	27	GLU
42	o	28	VAL
42	o	47	VAL
42	o	73	VAL
43	p	22	LYS
43	p	52	GLN
43	p	57	PHE
43	p	75	SER
43	p	78	LYS
43	p	90	ILE
43	p	91	ASP
43	p	104	VAL
44	q	37	GLU
44	q	72	VAL
44	q	96	VAL
44	q	101	ILE

Continued on next page...

For Manuscript Review

Continued from previous page...

Mol	Chain	Res	Type
45	r	13	SER
45	r	99	ARG
45	r	107	VAL
46	s	24	MET
46	s	25	GLU
46	s	28	ASN
46	s	50	LEU
46	s	55	VAL
46	s	78	SER
46	s	79	ASP
47	t	15	THR
47	t	29	LEU
47	t	43	LYS
47	t	49	VAL
47	t	70	VAL
47	t	94	ARG
48	u	20	LEU
48	u	25	LYS
48	u	30	ILE
48	u	41	GLU
48	u	42	LEU
49	v	10	THR
49	v	21	LEU
49	v	30	SER
49	v	58	THR
49	v	78	LYS
50	w	2	SER
50	w	11	ARG
50	w	28	ARG
50	w	35	SER
50	w	40	VAL
50	w	42	SER
50	w	48	THR
50	w	65	ASP
51	x	38	GLN
51	x	40	SER
51	x	53	VAL
52	y	12	SER
52	y	37	GLU
53	z	26	THR
53	z	44	THR

Sometimes sidechains can be flipped to improve hydrogen bonding and reduce clashes. All (44)

For Manuscript Review

such sidechains are listed below:

Mol	Chain	Res	Type
3	2	26	HIS
3	2	31	HIS
4	3	33	HIS
9	C	32	ASN
9	C	190	HIS
10	D	131	ASN
11	E	43	ASN
11	E	97	GLN
13	G	97	ASN
13	G	142	HIS
15	I	31	ASN
16	J	15	HIS
17	K	40	ASN
17	K	101	ASN
18	L	59	ASN
19	M	12	HIS
20	N	4	GLN
20	N	62	ASN
21	O	42	HIS
25	S	14	HIS
30	c	153	GLN
30	c	239	ASN
31	d	140	HIS
31	d	185	ASN
32	e	30	GLN
34	g	104	ASN
35	h	2	GLN
35	h	20	ASN
36	i	40	HIS
36	i	58	ASN
36	i	86	GLN
36	i	132	HIS
37	j	3	GLN
37	j	82	ASN
39	l	45	GLN
41	n	98	GLN
42	o	56	HIS
44	q	43	ASN
45	r	60	HIS
47	t	74	ASN
49	v	57	HIS
51	x	31	GLN

Continued on next page...

For Manuscript Review

Continued from previous page...

Mol	Chain	Res	Type
51	x	58	ASN
53	z	19	HIS

5.3.3 RNA ⓘ

Mol	Chain	Analysed	Backbone Outliers	Pucker Outliers
28	a	2747/2904 (94%)	427 (15%)	0
29	b	118/120 (98%)	36 (30%)	0
6	5	1/2 (50%)	1 (100%)	0
7	A	1513/1517 (99%)	564 (37%)	59 (3%)
All	All	4379/4543 (96%)	1028 (23%)	59 (1%)

All (1028) RNA backbone outliers are listed below:

Mol	Chain	Res	Type
6	5	76	A
7	A	4	U
7	A	6	G
7	A	7	A
7	A	8	A
7	A	9	G
7	A	10	A
7	A	11	G
7	A	15	G
7	A	17	U
7	A	18	C
7	A	22	G
7	A	32	A
7	A	35	G
7	A	37	U
7	A	39	G
7	A	41	G
7	A	42	G
7	A	43	C
7	A	47	C
7	A	48	C
7	A	51	A
7	A	62	U
7	A	63	C
7	A	65	A
7	A	66	G

Continued on next page...

For Manuscript Review

Continued from previous page...

Mol	Chain	Res	Type
7	A	73	C
7	A	75	C
7	A	76	A
7	A	78	A
7	A	79	G
7	A	82	A
7	A	83	C
7	A	84	U
7	A	85	U
7	A	86	G
7	A	87	U
7	A	88	U
7	A	89	C
7	A	90	C
7	A	91	U
7	A	92	U
7	A	93	G
7	A	94	G
7	A	95	G
7	A	96	U
7	A	99	C
7	A	100	G
7	A	106	C
7	A	109	A
7	A	116	A
7	A	120	A
7	A	121	U
7	A	122	G
7	A	123	C
7	A	131	A
7	A	132	U
7	A	134	G
7	A	144	G
7	A	145	G
7	A	146	G
7	A	154	C
7	A	157	U
7	A	162	A
7	A	171	A
7	A	173	U
7	A	177	G
7	A	181	A

Continued on next page...

For Manuscript Review

Continued from previous page...

Mol	Chain	Res	Type
7	A	182	A
7	A	183	C
7	A	184	C
7	A	186	C
7	A	193	C
7	A	195	A
7	A	196	A
7	A	197	A
7	A	198	G
7	A	199	C
7	A	200	A
7	A	215	C
7	A	216	U
7	A	222	U
7	A	223	A
7	A	228	A
7	A	239	U
7	A	240	G
7	A	245	U
7	A	247	G
7	A	251	G
7	A	259	G
7	A	260	G
7	A	261	U
7	A	262	A
7	A	266	G
7	A	267	C
7	A	268	U
7	A	274	A
7	A	280	C
7	A	281	G
7	A	283	U
7	A	289	G
7	A	295	C
7	A	299	G
7	A	300	A
7	A	301	G
7	A	306	A
7	A	325	A
7	A	326	G
7	A	327	A
7	A	328	C

Continued on next page...

For Manuscript Review

Continued from previous page...

Mol	Chain	Res	Type
7	A	330	C
7	A	332	G
7	A	334	C
7	A	338	A
7	A	344	A
7	A	348	G
7	A	351	G
7	A	352	C
7	A	354	G
7	A	367	U
7	A	372	C
7	A	373	A
7	A	377	G
7	A	378	G
7	A	379	C
7	A	381	C
7	A	382	A
7	A	383	A
7	A	384	G
7	A	385	C
7	A	386	C
7	A	397	A
7	A	406	A
7	A	407	U
7	A	410	G
7	A	411	A
7	A	412	C
7	A	413	A
7	A	414	A
7	A	415	A
7	A	422	C
7	A	423	G
7	A	424	G
7	A	428	G
7	A	429	U
7	A	435	A
7	A	436	C
7	A	437	U
7	A	438	U
7	A	440	C
7	A	442	G
7	A	445	G

Continued on next page...

For Manuscript Review

Continued from previous page...

Mol	Chain	Res	Type
7	A	446	G
7	A	448	A
7	A	449	G
7	A	450	G
7	A	451	A
7	A	452	A
7	A	455	U
7	A	458	U
7	A	460	A
7	A	463	C
7	A	465	A
7	A	466	A
7	A	467	U
7	A	468	A
7	A	470	C
7	A	474	A
7	A	475	U
7	A	476	C
7	A	477	A
7	A	480	U
7	A	483	C
7	A	484	G
7	A	485	U
7	A	486	U
7	A	489	C
7	A	490	U
7	A	497	G
7	A	498	A
7	A	499	A
7	A	500	G
7	A	502	A
7	A	505	G
7	A	507	C
7	A	508	U
7	A	511	C
7	A	512	U
7	A	513	C
7	A	516	PSU
7	A	518	C
7	A	521	G
7	A	524	G
7	A	527	G7M

Continued on next page...

For Manuscript Review

Continued from previous page...

Mol	Chain	Res	Type
7	A	532	A
7	A	533	A
7	A	534	U
7	A	536	C
7	A	537	G
7	A	539	A
7	A	540	G
7	A	543	U
7	A	544	G
7	A	547	A
7	A	548	G
7	A	549	C
7	A	550	G
7	A	559	A
7	A	564	C
7	A	573	A
7	A	575	G
7	A	576	C
7	A	577	G
7	A	586	U
7	A	589	U
7	A	592	G
7	A	596	A
7	A	598	U
7	A	599	C
7	A	600	A
7	A	602	A
7	A	607	A
7	A	608	A
7	A	609	A
7	A	610	G
7	A	611	C
7	A	612	C
7	A	613	C
7	A	615	G
7	A	616	G
7	A	617	G
7	A	622	A
7	A	626	G
7	A	632	C
7	A	633	G
7	A	637	U

Continued on next page...

For Manuscript Review

Continued from previous page...

Mol	Chain	Res	Type
7	A	638	U
7	A	639	G
7	A	642	A
7	A	643	C
7	A	644	U
7	A	652	U
7	A	653	A
7	A	662	U
7	A	663	A
7	A	665	A
7	A	687	A
7	A	695	A
7	A	707	U
7	A	720	C
7	A	721	G
7	A	723	U
7	A	724	G
7	A	738	C
7	A	747	A
7	A	749	A
7	A	755	A
7	A	777	A
7	A	779	C
7	A	781	A
7	A	787	A
7	A	793	U
7	A	794	A
7	A	799	G
7	A	802	A
7	A	815	A
7	A	817	C
7	A	818	G
7	A	819	A
7	A	820	U
7	A	821	G
7	A	825	A
7	A	827	U
7	A	831	A
7	A	832	G
7	A	836	G
7	A	838	G
7	A	839	C

Continued on next page...

For Manuscript Review

Continued from previous page...

Mol	Chain	Res	Type
7	A	847	G
7	A	848	C
7	A	850	U
7	A	851	G
7	A	860	A
7	A	872	A
7	A	878	A
7	A	881	G
7	A	887	G
7	A	890	G
7	A	891	U
7	A	892	A
7	A	908	A
7	A	909	A
7	A	912	C
7	A	913	A
7	A	914	A
7	A	922	G
7	A	926	G
7	A	927	G
7	A	934	C
7	A	935	A
7	A	938	A
7	A	953	G
7	A	957	U
7	A	958	A
7	A	959	A
7	A	960	U
7	A	962	C
7	A	963	G
7	A	965	U
7	A	966	2MG
7	A	967	5MC
7	A	969	A
7	A	972	C
7	A	973	G
7	A	974	A
7	A	975	A
7	A	976	G
7	A	978	A
7	A	979	C
7	A	981	U

Continued on next page...

For Manuscript Review

Continued from previous page...

Mol	Chain	Res	Type
7	A	982	U
7	A	989	U
7	A	991	U
7	A	992	U
7	A	993	G
7	A	994	A
7	A	995	C
7	A	996	A
7	A	997	U
7	A	998	C
7	A	999	C
7	A	1000	A
7	A	1001	G
7	A	1002	A
7	A	1003	G
7	A	1004	A
7	A	1005	A
7	A	1006	U
7	A	1008	U
7	A	1009	A
7	A	1010	G
7	A	1011	C
7	A	1012	G
7	A	1013	G
7	A	1014	A
7	A	1015	G
7	A	1016	A
7	A	1017	C
7	A	1018	G
7	A	1021	G
7	A	1022	G
7	A	1023	A
7	A	1024	G
7	A	1025	U
7	A	1026	G
7	A	1027	C
7	A	1028	C
7	A	1030	U
7	A	1031	C
7	A	1032	G
7	A	1034	G
7	A	1035	A

Continued on next page...

For Manuscript Review

Continued from previous page...

Mol	Chain	Res	Type
7	A	1036	G
7	A	1037	C
7	A	1038	U
7	A	1039	C
7	A	1040	U
7	A	1041	G
7	A	1042	A
7	A	1043	G
7	A	1044	A
7	A	1045	C
7	A	1046	A
7	A	1047	G
7	A	1048	G
7	A	1049	U
7	A	1050	G
7	A	1057	G
7	A	1059	C
7	A	1065	U
7	A	1067	A
7	A	1080	A
7	A	1081	A
7	A	1090	U
7	A	1094	G
7	A	1095	U
7	A	1099	G
7	A	1100	C
7	A	1101	A
7	A	1102	A
7	A	1104	G
7	A	1108	G
7	A	1109	C
7	A	1110	A
7	A	1113	C
7	A	1114	C
7	A	1115	U
7	A	1116	U
7	A	1119	C
7	A	1120	C
7	A	1122	U
7	A	1123	G
7	A	1124	U
7	A	1125	U

Continued on next page...

For Manuscript Review

Continued from previous page...

Mol	Chain	Res	Type
7	A	1126	U
7	A	1127	G
7	A	1130	A
7	A	1131	G
7	A	1133	A
7	A	1134	C
7	A	1135	G
7	A	1136	U
7	A	1138	A
7	A	1140	G
7	A	1141	G
7	A	1142	U
7	A	1143	G
7	A	1146	A
7	A	1147	A
7	A	1149	U
7	A	1150	C
7	A	1151	C
7	A	1153	G
7	A	1156	A
7	A	1157	G
7	A	1158	A
7	A	1159	C
7	A	1160	U
7	A	1161	G
7	A	1163	C
7	A	1168	A
7	A	1169	U
7	A	1170	A
7	A	1176	G
7	A	1177	A
7	A	1179	G
7	A	1180	A
7	A	1181	A
7	A	1182	G
7	A	1183	G
7	A	1184	U
7	A	1185	G
7	A	1186	G
7	A	1187	G
7	A	1188	G
7	A	1190	C

Continued on next page...

For Manuscript Review

Continued from previous page...

Mol	Chain	Res	Type
7	A	1191	G
7	A	1192	A
7	A	1193	C
7	A	1197	A
7	A	1198	A
7	A	1199	G
7	A	1200	U
7	A	1201	C
7	A	1202	A
7	A	1203	U
7	A	1212	U
7	A	1213	U
7	A	1214	A
7	A	1215	C
7	A	1216	G
7	A	1226	A
7	A	1227	C
7	A	1228	A
7	A	1235	C
7	A	1239	A
7	A	1241	U
7	A	1242	G
7	A	1243	G
7	A	1244	C
7	A	1246	U
7	A	1247	A
7	A	1249	A
7	A	1250	C
7	A	1251	A
7	A	1253	A
7	A	1254	G
7	A	1256	G
7	A	1257	C
7	A	1258	A
7	A	1259	G
7	A	1275	G
7	A	1276	A
7	A	1277	G
7	A	1280	A
7	A	1281	A
7	A	1282	U
7	A	1283	C

Continued on next page...

For Manuscript Review

Continued from previous page...

Mol	Chain	Res	Type
7	A	1284	U
7	A	1286	A
7	A	1287	C
7	A	1288	A
7	A	1293	A
7	A	1299	U
7	A	1301	G
7	A	1303	C
7	A	1306	G
7	A	1307	A
7	A	1311	G
7	A	1312	A
7	A	1313	G
7	A	1314	U
7	A	1317	G
7	A	1318	C
7	A	1320	A
7	A	1321	C
7	A	1322	U
7	A	1323	C
7	A	1329	C
7	A	1330	A
7	A	1332	G
7	A	1336	U
7	A	1337	C
7	A	1347	A
7	A	1348	G
7	A	1349	U
7	A	1351	A
7	A	1354	G
7	A	1361	A
7	A	1362	G
7	A	1364	A
7	A	1365	U
7	A	1370	C
7	A	1371	G
7	A	1373	U
7	A	1379	C
7	A	1380	G
7	A	1381	U
7	A	1382	U
7	A	1395	A

Continued on next page...

For Manuscript Review

Continued from previous page...

Mol	Chain	Res	Type
7	A	1399	A
7	A	1402	G
7	A	1403	4OC
7	A	1404	C
7	A	1420	A
7	A	1429	A
7	A	1433	G
7	A	1440	G
7	A	1442	A
7	A	1443	G
7	A	1444	U
7	A	1447	A
7	A	1448	A
7	A	1449	C
7	A	1452	U
7	A	1453	C
7	A	1454	G
7	A	1457	A
7	A	1461	C
7	A	1462	G
7	A	1493	A
7	A	1494	A
7	A	1496	U
7	A	1498	G
7	A	1500	A
7	A	1504	A
7	A	1507	U
7	A	1512	C
7	A	1518	G
7	A	1519	MA6
7	A	1528	C
7	A	1530	G
7	A	1531	G
7	A	1532	A
7	A	1534	C
28	a	10	A
28	a	12	U
28	a	34	U
28	a	51	G
28	a	71	A
28	a	74	A
28	a	75	G

Continued on next page...

For Manuscript Review

Continued from previous page...

Mol	Chain	Res	Type
28	a	84	A
28	a	85	G
28	a	86	G
28	a	87	U
28	a	100	U
28	a	101	A
28	a	102	U
28	a	118	A
28	a	119	A
28	a	120	U
28	a	125	A
28	a	139	U
28	a	142	A
28	a	163	C
28	a	181	A
28	a	196	A
28	a	199	A
28	a	215	G
28	a	216	A
28	a	221	A
28	a	222	A
28	a	228	C
28	a	233	A
28	a	248	G
28	a	264	C
28	a	265	A
28	a	268	C
28	a	269	C
28	a	270	A
28	a	271	G
28	a	272	A
28	a	276	U
28	a	278	A
28	a	282	A
28	a	285	G
28	a	287	G
28	a	311	A
28	a	329	G
28	a	330	A
28	a	345	A
28	a	361	G
28	a	362	A

Continued on next page...

For Manuscript Review

Continued from previous page...

Mol	Chain	Res	Type
28	a	385	C
28	a	386	G
28	a	396	G
28	a	404	A
28	a	405	U
28	a	406	G
28	a	411	G
28	a	416	U
28	a	420	C
28	a	425	G
28	a	456	C
28	a	481	G
28	a	490	C
28	a	491	G
28	a	504	A
28	a	505	A
28	a	509	C
28	a	510	C
28	a	530	G
28	a	531	C
28	a	532	A
28	a	545	U
28	a	546	U
28	a	549	G
28	a	551	G
28	a	563	A
28	a	573	U
28	a	574	A
28	a	575	A
28	a	599	A
28	a	600	G
28	a	601	C
28	a	602	A
28	a	615	U
28	a	627	A
28	a	628	G
28	a	629	G
28	a	631	A
28	a	632	A
28	a	636	G
28	a	637	A
28	a	644	A

Continued on next page...

For Manuscript Review

Continued from previous page...

Mol	Chain	Res	Type
28	a	645	C
28	a	647	G
28	a	654	A
28	a	655	A
28	a	656	G
28	a	661	A
28	a	663	G
28	a	686	U
28	a	696	G
28	a	717	C
28	a	730	A
28	a	736	C
28	a	738	G
28	a	746	PSU
28	a	747	5MU
28	a	764	A
28	a	775	G
28	a	776	G
28	a	782	A
28	a	784	G
28	a	785	G
28	a	792	A
28	a	805	G
28	a	806	C
28	a	812	C
28	a	827	U
28	a	828	U
28	a	845	A
28	a	846	U
28	a	847	U
28	a	859	G
28	a	864	G
28	a	883	G
28	a	884	U
28	a	888	C
28	a	890	C
28	a	891	G
28	a	893	C
28	a	895	U
28	a	896	A
28	a	897	C
28	a	910	A

Continued on next page...

For Manuscript Review

Continued from previous page...

Mol	Chain	Res	Type
28	a	934	U
28	a	936	A
28	a	938	G
28	a	939	G
28	a	946	C
28	a	961	C
28	a	971	G
28	a	974	G
28	a	983	A
28	a	996	A
28	a	1009	A
28	a	1011	G
28	a	1012	U
28	a	1013	C
28	a	1022	G
28	a	1023	U
28	a	1024	G
28	a	1025	G
28	a	1026	G
28	a	1028	A
28	a	1029	A
28	a	1032	A
28	a	1033	U
28	a	1047	G
28	a	1048	A
28	a	1110	G
28	a	1111	A
28	a	1112	G
28	a	1116	G
28	a	1127	A
28	a	1130	U
28	a	1132	U
28	a	1135	C
28	a	1136	G
28	a	1141	U
28	a	1142	A
28	a	1168	G
28	a	1169	A
28	a	1179	G
28	a	1180	U
28	a	1184	U
28	a	1185	G

Continued on next page...

For Manuscript Review

Continued from previous page...

Mol	Chain	Res	Type
28	a	1186	G
28	a	1206	G
28	a	1212	G
28	a	1250	G
28	a	1253	A
28	a	1255	U
28	a	1256	G
28	a	1271	G
28	a	1272	A
28	a	1300	G
28	a	1301	A
28	a	1313	U
28	a	1365	A
28	a	1379	U
28	a	1383	A
28	a	1415	U
28	a	1416	G
28	a	1417	C
28	a	1428	C
28	a	1429	G
28	a	1452	G
28	a	1453	A
28	a	1458	U
28	a	1482	G
28	a	1493	C
28	a	1510	G
28	a	1515	A
28	a	1524	G
28	a	1535	A
28	a	1536	C
28	a	1537	G
28	a	1566	A
28	a	1569	A
28	a	1578	U
28	a	1583	A
28	a	1584	U
28	a	1585	C
28	a	1608	A
28	a	1609	A
28	a	1622	G
28	a	1646	C
28	a	1647	U

Continued on next page...

For Manuscript Review

Continued from previous page...

Mol	Chain	Res	Type
28	a	1648	U
28	a	1654	A
28	a	1674	G
28	a	1675	C
28	a	1676	A
28	a	1715	G
28	a	1729	U
28	a	1730	C
28	a	1733	G
28	a	1738	G
28	a	1764	C
28	a	1773	A
28	a	1800	C
28	a	1801	A
28	a	1808	A
28	a	1816	C
28	a	1829	A
28	a	1839	G
28	a	1847	A
28	a	1848	A
28	a	1858	A
28	a	1865	U
28	a	1866	A
28	a	1870	C
28	a	1871	A
28	a	1872	A
28	a	1883	U
28	a	1905	C
28	a	1906	G
28	a	1913	A
28	a	1929	G
28	a	1930	G
28	a	1936	A
28	a	1937	A
28	a	1955	U
28	a	1967	C
28	a	1970	A
28	a	1971	U
28	a	1972	G
28	a	1991	U
28	a	1993	U
28	a	2007	U

Continued on next page...

For Manuscript Review

Continued from previous page...

Mol	Chain	Res	Type
28	a	2023	C
28	a	2027	G
28	a	2030	6MZ
28	a	2031	A
28	a	2033	A
28	a	2035	G
28	a	2036	C
28	a	2043	C
28	a	2046	G
28	a	2055	C
28	a	2056	G
28	a	2060	A
28	a	2061	G
28	a	2062	A
28	a	2069	G7M
28	a	2077	A
28	a	2093	G
28	a	2198	A
28	a	2199	A
28	a	2203	U
28	a	2204	G
28	a	2211	A
28	a	2212	A
28	a	2225	A
28	a	2238	G
28	a	2239	G
28	a	2279	G
28	a	2283	C
28	a	2287	A
28	a	2288	A
28	a	2292	U
28	a	2305	U
28	a	2307	G
28	a	2308	G
28	a	2312	U
28	a	2317	A
28	a	2318	G
28	a	2319	G
28	a	2320	U
28	a	2322	A
28	a	2324	U
28	a	2325	G

Continued on next page...

For Manuscript Review

Continued from previous page...

Mol	Chain	Res	Type
28	a	2326	C
28	a	2327	A
28	a	2331	G
28	a	2333	A
28	a	2334	U
28	a	2335	A
28	a	2336	A
28	a	2338	C
28	a	2339	C
28	a	2345	G
28	a	2347	C
28	a	2348	U
28	a	2349	G
28	a	2351	G
28	a	2352	A
28	a	2357	G
28	a	2358	A
28	a	2361	G
28	a	2362	C
28	a	2363	G
28	a	2364	C
28	a	2366	A
28	a	2367	G
28	a	2372	U
28	a	2376	A
28	a	2379	G
28	a	2381	A
28	a	2382	G
28	a	2383	G
28	a	2385	C
28	a	2388	A
28	a	2389	G
28	a	2402	U
28	a	2403	C
28	a	2406	A
28	a	2417	C
28	a	2418	A
28	a	2421	G
28	a	2425	A
28	a	2429	G
28	a	2441	U
28	a	2448	A

Continued on next page...

For Manuscript Review

Continued from previous page...

Mol	Chain	Res	Type
28	a	2459	A
28	a	2470	G
28	a	2476	A
28	a	2478	A
28	a	2480	C
28	a	2488	G
28	a	2491	U
28	a	2498	OMC
28	a	2502	G
28	a	2505	G
28	a	2513	A
28	a	2518	A
28	a	2520	C
28	a	2528	U
28	a	2530	A
28	a	2531	A
28	a	2534	A
28	a	2535	G
28	a	2542	A
28	a	2543	G
28	a	2547	A
28	a	2566	A
28	a	2567	G
28	a	2582	G
28	a	2585	U
28	a	2586	U
28	a	2602	A
28	a	2603	G
28	a	2609	U
28	a	2613	U
28	a	2615	U
28	a	2624	G
28	a	2628	C
28	a	2629	U
28	a	2646	C
28	a	2649	C
28	a	2651	C
28	a	2652	C
28	a	2653	U
28	a	2661	G
28	a	2667	C
28	a	2670	A

Continued on next page...

For Manuscript Review

Continued from previous page...

Mol	Chain	Res	Type
28	a	2673	G
28	a	2674	G
28	a	2682	A
28	a	2689	U
28	a	2690	U
28	a	2703	C
28	a	2714	G
28	a	2726	A
28	a	2733	A
28	a	2739	U
28	a	2742	G
28	a	2743	U
28	a	2744	G
28	a	2746	U
28	a	2747	G
28	a	2751	G
28	a	2752	C
28	a	2755	C
28	a	2756	U
28	a	2757	A
28	a	2763	G
28	a	2764	A
28	a	2765	A
28	a	2766	A
28	a	2776	A
28	a	2777	G
28	a	2778	A
28	a	2790	U
28	a	2791	G
28	a	2798	U
28	a	2799	A
28	a	2820	A
28	a	2821	A
28	a	2835	A
28	a	2849	U
28	a	2861	U
28	a	2873	A
28	a	2880	C
28	a	2884	U
28	a	2893	A
28	a	2896	C
28	a	2899	A

Continued on next page...

For Manuscript Review

Continued from previous page...

Mol	Chain	Res	Type
29	b	9	G
29	b	12	C
29	b	14	U
29	b	15	A
29	b	24	G
29	b	25	U
29	b	33	G
29	b	35	C
29	b	36	C
29	b	37	C
29	b	41	G
29	b	43	C
29	b	44	G
29	b	45	A
29	b	47	C
29	b	50	A
29	b	53	A
29	b	56	G
29	b	57	A
29	b	68	C
29	b	73	A
29	b	75	G
29	b	77	U
29	b	81	G
29	b	89	U
29	b	90	C
29	b	91	C
29	b	94	A
29	b	95	U
29	b	96	G
29	b	99	A
29	b	100	G
29	b	104	A
29	b	105	G
29	b	108	A
29	b	109	A

All (59) RNA pucker outliers are listed below:

Mol	Chain	Res	Type
7	A	64	G
7	A	87	U

Continued on next page...

For Manuscript Review

Continued from previous page...

Mol	Chain	Res	Type
7	A	93	G
7	A	195	A
7	A	198	G
7	A	222	U
7	A	239	U
7	A	280	C
7	A	326	G
7	A	435	A
7	A	447	G
7	A	450	G
7	A	454	G
7	A	467	U
7	A	484	G
7	A	489	C
7	A	506	G
7	A	538	G
7	A	539	A
7	A	548	G
7	A	588	G
7	A	599	C
7	A	609	A
7	A	778	G
7	A	880	C
7	A	964	A
7	A	994	A
7	A	995	C
7	A	996	A
7	A	999	C
7	A	1004	A
7	A	1010	G
7	A	1012	G
7	A	1017	C
7	A	1036	G
7	A	1040	U
7	A	1043	G
7	A	1044	A
7	A	1066	C
7	A	1100	C
7	A	1115	U
7	A	1122	U
7	A	1133	A
7	A	1156	A

Continued on next page...

For Manuscript Review

Continued from previous page...

Mol	Chain	Res	Type
7	A	1160	U
7	A	1180	A
7	A	1190	C
7	A	1191	G
7	A	1243	G
7	A	1306	G
7	A	1320	A
7	A	1321	C
7	A	1347	A
7	A	1348	G
7	A	1370	C
7	A	1448	A
7	A	1452	U
7	A	1453	C
7	A	1506	G

5.4 Non-standard residues in protein, DNA, RNA chains ⓘ

40 non-standard protein/DNA/RNA residues are modelled in this entry.

In the following table, the Counts columns list the number of bonds (or angles) for which Mogul statistics could be retrieved, the number of bonds (or angles) that are observed in the model and the number of bonds (or angles) that are defined in the Chemical Component Dictionary. The Link column lists molecule types, if any, to which the group is linked. The Z score for a bond length (or angle) is the number of standard deviations the observed value is removed from the expected value. A bond length (or angle) with $|Z| > 2$ is considered an outlier worth inspection. RMSZ is the root-mean-square of all Z scores of the bond lengths (or angles).

Mol	Type	Chain	Res	Link	Bond lengths			Bond angles		
					Counts	RMSZ	$\#\|Z\|>2$	Counts	RMSZ	$\#\|Z\|>2$
39	4D4	l	81	39	9,11,12	2.10	2 (22%)	7,13,15	2.19	3 (42%)
28	PSU	a	2504	28	18,21,22	1.01	1 (5%)	21,30,33	1.92	4 (19%)
28	PSU	a	1917	28	18,21,22	1.00	1 (5%)	21,30,33	1.98	5 (23%)
28	H2U	a	2449	28	18,21,22	0.49	0	19,30,33	1.09	1 (5%)
28	6MZ	a	1618	28	17,25,26	1.31	3 (17%)	15,36,39	2.36	4 (26%)
28	PSU	a	2580	28	18,21,22	1.22	3 (16%)	21,30,33	2.21	5 (23%)
7	5MC	A	967	7	19,22,23	1.28	2 (10%)	26,32,35	1.08	2 (7%)
18	D2T	L	89	18	8,9,10	1.78	2 (25%)	6,11,13	1.80	2 (33%)
28	OMU	a	2552	28	19,22,23	1.11	3 (15%)	25,31,34	2.06	6 (24%)
7	G7M	A	527	7	20,26,27	0.90	1 (5%)	16,39,42	0.59	0

For Manuscript Review

Mol	Type	Chain	Res	Link	Bond lengths			Bond angles		
					Counts	RMSZ	#|Z| > 2	Counts	RMSZ	#|Z| > 2
28	G7M	a	2069	28	20,26,27	0.94	1 (5%)	16,39,42	0.61	0
7	2MG	A	1208	7	18,26,27	1.27	2 (11%)	16,38,41	1.47	3 (18%)
28	2MG	a	1835	28	18,26,27	1.32	2 (11%)	16,38,41	1.66	5 (31%)
28	5MU	a	1939	28	19,22,23	1.21	4 (21%)	27,32,35	2.19	6 (22%)
28	6MZ	a	2030	28	17,25,26	1.29	3 (17%)	15,36,39	2.22	4 (26%)
7	5MC	A	1408	7	19,22,23	1.37	2 (10%)	26,32,35	1.15	3 (11%)
28	PSU	a	746	55,28	18,21,22	1.15	3 (16%)	21,30,33	1.94	4 (19%)
31	MEQ	d	150	31	8,9,10	0.83	0	5,10,12	0.37	0
7	MA6	A	1519	7	19,26,27	1.93	4 (21%)	18,38,41	1.23	2 (11%)
7	2MG	A	966	7	18,26,27	1.23	3 (16%)	16,38,41	1.53	3 (18%)
28	PSU	a	2604	28	18,21,22	1.10	3 (16%)	21,30,33	2.02	4 (19%)
7	4OC	A	1403	7	20,23,24	0.36	0	25,32,35	0.65	1 (4%)
28	OMG	a	2251	28	19,26,27	1.28	2 (10%)	21,38,41	1.39	4 (19%)
28	5MC	a	1962	28	19,22,23	1.53	2 (10%)	26,32,35	1.20	2 (7%)
28	3TD	a	1915	-	19,22,23	1.10	2 (10%)	23,32,35	1.95	3 (13%)
28	2MA	a	2503	55,28	18,25,26	1.27	2 (11%)	20,37,40	2.01	3 (15%)
28	OMC	a	2498	55,28	19,22,23	1.00	1 (5%)	25,31,34	1.28	2 (8%)
28	2MG	a	2445	28	18,26,27	1.48	3 (16%)	16,38,41	1.49	3 (18%)
28	PSU	a	1911	28	18,21,22	0.95	1 (5%)	21,30,33	1.98	4 (19%)
28	1MG	a	745	28	19,26,27	1.40	3 (15%)	18,39,42	1.44	2 (11%)
7	PSU	A	516	7	18,21,22	0.99	1 (5%)	21,30,33	2.00	5 (23%)
39	MS6	l	82	39	5,7,8	0.59	0	2,7,9	1.09	0
7	2MG	A	1517	7	18,26,27	1.32	2 (11%)	16,38,41	1.68	5 (31%)
7	MA6	A	1520	7	19,26,27	1.93	4 (21%)	18,38,41	1.21	2 (11%)
7	UR3	A	1499	7	19,22,23	3.89	6 (31%)	26,32,35	4.65	11 (42%)
28	PSU	a	955	28	18,21,22	1.16	3 (16%)	21,30,33	2.08	4 (19%)
28	PSU	a	2457	28	18,21,22	1.14	4 (22%)	21,30,33	2.14	5 (23%)
28	PSU	a	2605	28	18,21,22	1.13	2 (11%)	21,30,33	1.97	3 (14%)
28	5MU	a	747	28	19,22,23	1.13	3 (15%)	27,32,35	2.16	7 (25%)
17	BH2	K	119	17	5,6,9	0.93	0	1,6,12	0.62	0

In the following table, the Chirals column lists the number of chiral outliers, the number of chiral centers analysed, the number of these observed in the model and the number defined in the Chemical Component Dictionary. Similar counts are reported in the Torsion and Rings columns. '-' means no outliers of that kind were identified.

For Manuscript Review

Mol	Type	Chain	Res	Link	Chirals	Torsions	Rings
39	4D4	l	81	39	-	2/11/12/14	-
28	PSU	a	2504	28	-	2/7/25/26	0/2/2/2
28	PSU	a	1917	28	-	0/7/25/26	0/2/2/2
28	H2U	a	2449	28	-	0/7/38/39	0/2/2/2
28	6MZ	a	1618	28	-	0/5/27/28	0/3/3/3
28	PSU	a	2580	28	-	1/7/25/26	0/2/2/2
7	5MC	A	967	7	-	1/7/25/26	0/2/2/2
18	D2T	L	89	18	-	5/7/12/14	-
28	OMU	a	2552	28	-	0/9/27/28	0/2/2/2
7	G7M	A	527	7	-	2/3/25/26	0/3/3/3
28	G7M	a	2069	28	-	2/3/25/26	0/3/3/3
7	2MG	A	1208	7	-	0/5/27/28	0/3/3/3
28	2MG	a	1835	28	-	0/5/27/28	0/3/3/3
28	5MU	a	1939	28	-	0/7/25/26	0/2/2/2
28	6MZ	a	2030	28	-	2/5/27/28	0/3/3/3
7	5MC	A	1408	7	-	0/7/25/26	0/2/2/2
28	PSU	a	746	55,28	-	1/7/25/26	0/2/2/2
31	MEQ	d	150	31	-	3/8/9/11	-
7	MA6	A	1519	7	-	3/7/29/30	0/3/3/3
7	2MG	A	966	7	-	0/5/27/28	0/3/3/3
28	PSU	a	2604	28	-	1/7/25/26	0/2/2/2
7	4OC	A	1403	7	-	2/9/29/30	0/2/2/2
28	OMG	a	2251	28	-	1/5/27/28	0/3/3/3
28	5MC	a	1962	28	-	0/7/25/26	0/2/2/2
28	3TD	a	1915	-	-	4/7/25/26	0/2/2/2
28	2MA	a	2503	55,28	-	2/3/25/26	0/3/3/3
28	OMC	a	2498	55,28	-	3/9/27/28	0/2/2/2
28	2MG	a	2445	28	-	0/5/27/28	0/3/3/3
28	PSU	a	1911	28	-	0/7/25/26	0/2/2/2
28	1MG	a	745	28	-	0/3/25/26	0/3/3/3
7	PSU	A	516	7	-	1/7/25/26	0/2/2/2
39	MS6	l	82	39	-	2/4/6/8	-
7	2MG	A	1517	7	-	0/5/27/28	0/3/3/3
7	MA6	A	1520	7	-	2/7/29/30	0/3/3/3
7	UR3	A	1499	7	-	2/7/25/26	0/2/2/2
28	PSU	a	955	28	-	0/7/25/26	0/2/2/2
28	PSU	a	2457	28	-	0/7/25/26	0/2/2/2
28	PSU	a	2605	28	-	0/7/25/26	0/2/2/2
28	5MU	a	747	28	-	0/7/25/26	0/2/2/2
17	BH2	K	119	17	1/1/1/4	1/4/5/12	-

For Manuscript Review

All (86) bond length outliers are listed below:

Mol	Chain	Res	Type	Atoms	Z	Observed(Å)	Ideal(Å)
7	A	1499	UR3	C2-N1	13.96	1.57	1.38
7	A	1499	UR3	C6-N1	6.20	1.52	1.38
7	A	1499	UR3	C2-N3	5.71	1.50	1.39
28	a	1962	5MC	C5-C4	-5.35	1.40	1.44
39	l	81	4D4	CZ-NE	5.23	1.43	1.33
7	A	1519	MA6	C6-C5	-5.23	1.36	1.44
7	A	1520	MA6	C6-C5	-5.12	1.37	1.44
7	A	1408	5MC	C5-C4	-4.35	1.40	1.44
7	A	967	5MC	C5-C4	-4.14	1.41	1.44
7	A	1520	MA6	C5-N7	3.69	1.53	1.39
7	A	1519	MA6	C5-N7	3.67	1.53	1.39
7	A	1520	MA6	C6-N6	3.63	1.45	1.37
7	A	1519	MA6	C6-N6	3.54	1.45	1.37
18	L	89	D2T	CB-CA	-3.42	1.53	1.54
28	a	2445	2MG	C5-C6	-3.42	1.40	1.47
28	a	2503	2MA	C6-N1	3.37	1.40	1.33
28	a	2445	2MG	C8-N7	-3.32	1.29	1.34
7	A	1499	UR3	C6-C5	-3.19	1.27	1.35
28	a	2251	OMG	C8-N7	-3.18	1.29	1.34
7	A	1519	MA6	C4-N3	-3.15	1.31	1.35
7	A	1520	MA6	C4-N3	-3.13	1.31	1.35
28	a	745	1MG	C8-N7	-3.13	1.29	1.34
7	A	1517	2MG	C5-C6	-3.11	1.41	1.47
28	a	1835	2MG	C5-C6	-3.06	1.41	1.47
28	a	1618	6MZ	C6-C5	-3.02	1.40	1.44
28	a	2251	OMG	C5-C6	-3.02	1.41	1.47
28	a	2030	6MZ	C6-C5	-3.01	1.40	1.44
28	a	1835	2MG	C8-N7	-2.97	1.30	1.34
7	A	1517	2MG	C8-N7	-2.92	1.30	1.34
7	A	1208	2MG	C5-C6	-2.90	1.41	1.47
7	A	966	2MG	C5-C6	-2.86	1.41	1.47
7	A	1208	2MG	C8-N7	-2.84	1.30	1.34
39	l	81	4D4	CZ-NH1	2.83	1.44	1.34
7	A	966	2MG	C8-N7	-2.82	1.30	1.34
7	A	1408	5MC	C2-N1	-2.81	1.34	1.40
28	a	745	1MG	C6-N1	-2.80	1.34	1.39
7	A	527	G7M	C8-N9	2.78	1.38	1.33
28	a	1939	5MU	C2-N1	-2.76	1.34	1.38
28	a	1962	5MC	C2-N1	-2.73	1.34	1.40
28	a	1915	3TD	C4-N3	-2.72	1.34	1.40
28	a	2069	G7M	C8-N9	2.71	1.38	1.33
28	a	2552	OMU	C2-N1	-2.69	1.34	1.38

Continued on next page...

For Manuscript Review

Continued from previous page...

Mol	Chain	Res	Type	Atoms	Z	Observed(Å)	Ideal(Å)
28	a	747	5MU	C2-N1	-2.66	1.34	1.38
28	a	1618	6MZ	C8-N7	-2.63	1.29	1.34
7	A	967	5MC	C2-N1	-2.58	1.34	1.40
28	a	2498	OMC	C2-N1	-2.56	1.34	1.40
28	a	2580	PSU	C2-N1	-2.54	1.33	1.36
28	a	2030	6MZ	C8-N7	-2.51	1.30	1.34
28	a	2605	PSU	C2-N3	-2.49	1.33	1.37
28	a	746	PSU	O4'-C1'	-2.47	1.40	1.43
28	a	955	PSU	C4-N3	-2.42	1.34	1.38
28	a	2580	PSU	O4'-C1'	-2.42	1.40	1.43
28	a	2552	OMU	C4-N3	-2.38	1.34	1.38
28	a	2604	PSU	C4-N3	-2.37	1.34	1.38
28	a	2605	PSU	C4-N3	-2.36	1.34	1.38
28	a	1939	5MU	C4-C5	-2.35	1.41	1.44
28	a	1939	5MU	C4-N3	-2.32	1.34	1.38
28	a	2580	PSU	C4-N3	-2.31	1.34	1.38
28	a	2030	6MZ	C4-N3	-2.31	1.32	1.35
28	a	1618	6MZ	C4-N3	-2.29	1.32	1.35
28	a	1939	5MU	C2-N3	-2.27	1.34	1.38
28	a	2552	OMU	C2-N3	-2.27	1.34	1.38
28	a	747	5MU	C4-C5	-2.26	1.41	1.44
28	a	746	PSU	C4-N3	-2.26	1.34	1.38
28	a	747	5MU	C4-N3	-2.25	1.34	1.38
28	a	745	1MG	C5-C6	-2.24	1.41	1.47
28	a	1915	3TD	C4-C5	-2.23	1.42	1.47
28	a	2445	2MG	C6-N1	-2.22	1.34	1.37
28	a	2457	PSU	C4-N3	-2.22	1.34	1.38
28	a	2604	PSU	C2-N3	-2.21	1.33	1.37
28	a	2457	PSU	C2-N1	-2.20	1.33	1.36
28	a	955	PSU	C2-N3	-2.17	1.33	1.37
28	a	2457	PSU	O4'-C1'	-2.13	1.40	1.43
28	a	2504	PSU	C4-N3	-2.13	1.34	1.38
28	a	2604	PSU	C2-N1	-2.11	1.33	1.36
7	A	1499	UR3	O4-C4	-2.11	1.19	1.23
7	A	516	PSU	C4-N3	-2.10	1.34	1.38
28	a	1917	PSU	C4-N3	-2.10	1.34	1.38
7	A	1499	UR3	O2-C2	-2.08	1.18	1.22
28	a	1911	PSU	C4-N3	-2.07	1.35	1.38
28	a	2457	PSU	C2-N3	-2.05	1.34	1.37
7	A	966	2MG	C2-N2	2.05	1.37	1.33
18	L	89	D2T	CB1-SB	-2.05	1.75	1.79
28	a	955	PSU	C2-N1	-2.04	1.34	1.36

Continued on next page...

For Manuscript Review

Continued from previous page...

Mol	Chain	Res	Type	Atoms	Z	Observed(Å)	Ideal(Å)
28	a	2503	2MA	C8-N7	-2.03	1.31	1.34
28	a	746	PSU	C2-N3	-2.00	1.34	1.37

All (132) bond angle outliers are listed below:

Mol	Chain	Res	Type	Atoms	Z	Observed(^{o})	Ideal(^{o})
7	A	1499	UR3	C6-N1-C2	-14.80	109.71	121.80
7	A	1499	UR3	C4-N3-C2	-14.23	113.13	124.58
7	A	1499	UR3	C5-C4-N3	7.55	124.99	115.04
28	a	1915	3TD	N1-C2-N3	6.52	120.87	116.13
28	a	2503	2MA	C2-N3-C4	6.13	120.41	115.46
28	a	2580	PSU	N1-C2-N3	6.10	121.60	115.17
28	a	2457	PSU	N1-C2-N3	5.89	121.38	115.17
28	a	1618	6MZ	C2-N1-C6	5.71	121.03	116.60
28	a	955	PSU	N1-C2-N3	5.68	121.16	115.17
28	a	2604	PSU	N1-C2-N3	5.64	121.12	115.17
28	a	2552	OMU	C4-N3-C2	-5.60	119.67	126.61
28	a	1939	5MU	C4-N3-C2	-5.56	120.05	127.34
28	a	1917	PSU	N1-C2-N3	5.49	120.96	115.17
28	a	2030	6MZ	C2-N1-C6	5.48	120.86	116.60
28	a	1911	PSU	N1-C2-N3	5.47	120.94	115.17
28	a	746	PSU	N1-C2-N3	5.42	120.89	115.17
28	a	2504	PSU	N1-C2-N3	5.36	120.82	115.17
28	a	2552	OMU	N3-C2-N1	5.32	121.82	114.89
7	A	516	PSU	N1-C2-N3	5.32	120.78	115.17
28	a	747	5MU	C4-N3-C2	-5.28	120.42	127.34
28	a	2605	PSU	N1-C2-N3	5.21	120.67	115.17
28	a	2503	2MA	C5-C6-N1	-5.10	114.81	120.84
28	a	1939	5MU	N3-C2-N1	4.97	121.36	114.89
28	a	955	PSU	C4-N3-C2	-4.95	119.55	126.37
28	a	2605	PSU	C4-N3-C2	-4.93	119.57	126.37
7	A	1499	UR3	C1'-N1-C2	4.85	124.97	117.04
28	a	2457	PSU	C4-N3-C2	-4.77	119.80	126.37
28	a	1618	6MZ	C9-N6-C6	-4.75	118.44	122.85
28	a	747	5MU	N3-C2-N1	4.71	121.02	114.89
28	a	2604	PSU	C4-N3-C2	-4.70	119.90	126.37
28	a	746	PSU	C4-N3-C2	-4.67	119.94	126.37
28	a	1618	6MZ	N3-C2-N1	-4.64	122.37	128.67
28	a	2580	PSU	C4-N3-C2	-4.63	120.00	126.37
28	a	1911	PSU	C4-N3-C2	-4.62	120.01	126.37
28	a	1939	5MU	C5-C4-N3	4.59	119.31	115.32
28	a	747	5MU	C5-C4-N3	4.55	119.27	115.32

Continued on next page...

For Manuscript Review

Continued from previous page...

Mol	Chain	Res	Type	Atoms	Z	Observed(°)	Ideal(°)
28	a	1917	PSU	C4-N3-C2	-4.52	120.15	126.37
28	a	2504	PSU	C4-N3-C2	-4.38	120.34	126.37
28	a	2030	6MZ	N3-C2-N1	-4.37	122.74	128.67
7	A	516	PSU	C4-N3-C2	-4.37	120.35	126.37
28	a	747	5MU	O4-C4-C5	-4.24	120.06	124.92
28	a	1939	5MU	O4-C4-C5	-4.17	120.15	124.92
7	A	1499	UR3	C3U-N3-C4	4.08	123.53	117.87
28	a	2580	PSU	O2-C2-N1	-4.06	118.60	122.79
7	A	1208	2MG	C8-N7-C5	4.00	109.37	102.55
28	a	745	1MG	C8-N7-C5	3.95	109.27	102.55
28	a	1915	3TD	C4-N3-C2	-3.93	120.45	124.61
7	A	966	2MG	C8-N7-C5	3.93	109.24	102.55
28	a	2251	OMG	C8-N7-C5	3.91	109.21	102.55
28	a	2445	2MG	C8-N7-C5	3.91	109.20	102.55
28	a	1835	2MG	C8-N7-C5	3.89	109.17	102.55
7	A	1517	2MG	C8-N7-C5	3.87	109.14	102.55
39	l	81	4D4	NE-CZ-NH2	3.79	127.19	120.67
28	a	2030	6MZ	C9-N6-C6	-3.77	119.36	122.85
28	a	2457	PSU	O2-C2-N1	-3.72	118.96	122.79
28	a	1962	5MC	C5-C6-N1	-3.70	119.29	123.31
28	a	2449	H2U	C5-C4-N3	-3.65	112.81	116.69
7	A	1408	5MC	C5-C6-N1	-3.60	119.40	123.31
7	A	1519	MA6	C1'-N9-C4	-3.59	120.34	126.64
28	a	1939	5MU	C5-C6-N1	-3.57	119.43	123.31
28	a	1915	3TD	C1'-C5-C4	3.54	122.98	117.61
7	A	967	5MC	C5-C6-N1	-3.54	119.47	123.31
39	l	81	4D4	O-C-CA	-3.53	115.68	124.77
7	A	1520	MA6	C1'-N9-C4	-3.53	120.43	126.64
7	A	1499	UR3	C3U-N3-C2	3.45	123.34	117.33
28	a	2552	OMU	C5-C4-N3	3.40	119.57	114.80
28	a	2604	PSU	O2-C2-N1	-3.32	119.36	122.79
28	a	2552	OMU	CM2-O2'-C2'	-3.30	106.01	114.47
28	a	745	1MG	C5-C6-N1	3.28	118.70	113.96
28	a	2504	PSU	O2-C2-N1	-3.26	119.43	122.79
7	A	1517	2MG	N1-C2-N2	3.25	119.88	116.56
7	A	516	PSU	O2-C2-N1	-3.25	119.44	122.79
28	a	2498	OMC	CM2-O2'-C2'	-3.24	106.15	114.47
28	a	746	PSU	O2-C2-N1	-3.15	119.54	122.79
28	a	1911	PSU	O2-C2-N1	-3.14	119.56	122.79
28	a	2498	OMC	O2-C2-N3	-3.13	117.40	122.33
28	a	955	PSU	O2-C2-N1	-3.12	119.57	122.79
28	a	1917	PSU	O2-C2-N1	-3.11	119.58	122.79

Continued on next page...

For Manuscript Review

Continued from previous page...

Mol	Chain	Res	Type	Atoms	Z	Observed(°)	Ideal(°)
28	a	1835	2MG	N1-C2-N2	3.09	119.71	116.56
28	a	2580	PSU	C6-N1-C2	-2.99	119.91	122.69
28	a	747	5MU	C5-C6-N1	-2.91	120.15	123.31
28	a	2251	OMG	C2-N1-C6	-2.89	119.81	125.11
18	L	89	D2T	O-C-CA	-2.88	117.37	124.77
7	A	1519	MA6	C4-C5-N7	-2.81	106.37	109.34
28	a	2605	PSU	O2-C2-N1	-2.80	119.91	122.79
28	a	2552	OMU	O2-C2-N1	-2.76	119.21	122.80
7	A	966	2MG	N1-C2-N2	2.74	119.36	116.56
28	a	1939	5MU	O2-C2-N1	-2.70	119.28	122.80
7	A	1520	MA6	C4-C5-N7	-2.63	106.56	109.34
28	a	2552	OMU	O4-C4-C5	-2.59	120.70	125.16
28	a	2457	PSU	C6-N1-C2	-2.55	120.32	122.69
28	a	2445	2MG	C5-C6-N1	2.53	118.89	114.07
28	a	1835	2MG	C5-C6-N1	2.50	118.83	114.07
7	A	516	PSU	O4'-C1'-C2'	2.48	108.59	105.15
28	a	747	5MU	C1'-N1-C2	2.43	121.95	117.59
7	A	1499	UR3	O4-C4-C5	-2.39	117.56	124.35
7	A	516	PSU	C6-N1-C2	-2.39	120.47	122.69
7	A	1499	UR3	O2-C2-N3	-2.38	118.04	121.33
7	A	1517	2MG	C5-C6-N1	2.38	118.62	114.07
28	a	1835	2MG	CM2-N2-C2	-2.38	118.54	123.65
28	a	1917	PSU	C6-N1-C2	-2.36	120.50	122.69
28	a	2251	OMG	CM2-O2'-C2'	-2.35	108.45	114.47
7	A	1208	2MG	C5-C6-N1	2.31	118.48	114.07
28	a	2251	OMG	C5-C6-N1	2.31	118.48	114.07
7	A	966	2MG	C5-C6-N1	2.27	118.40	114.07
7	A	1408	5MC	C5-C4-N3	-2.27	119.43	121.75
28	a	2580	PSU	O4'-C1'-C2'	2.25	108.26	105.15
18	L	89	D2T	CB-CA-N	2.22	113.60	109.10
28	a	2030	6MZ	C4-C5-N7	-2.21	107.00	109.34
39	l	81	4D4	NH1-CZ-NE	-2.21	114.24	119.27
7	A	1517	2MG	CM2-N2-C2	-2.21	118.90	123.65
7	A	1208	2MG	N1-C2-N2	2.21	118.82	116.56
28	a	2457	PSU	O4'-C1'-C2'	2.21	108.21	105.15
28	a	2504	PSU	C6-N1-C2	-2.18	120.67	122.69
7	A	1499	UR3	C6-C5-C4	2.17	124.88	120.73
7	A	1499	UR3	O2-C2-N1	-2.16	117.97	122.78
28	a	2445	2MG	CM2-N2-C2	-2.14	119.05	123.65
28	a	1917	PSU	O4'-C1'-C2'	2.13	108.10	105.15
28	a	2604	PSU	C6-N1-C2	-2.13	120.72	122.69
28	a	1911	PSU	C6-N1-C2	-2.13	120.72	122.69

Continued on next page...

For Manuscript Review

Continued from previous page...

Mol	Chain	Res	Type	Atoms	Z	Observed(°)	Ideal(°)
7	A	1499	UR3	C1'-N1-C6	2.12	125.31	120.78
28	a	955	PSU	C6-N1-C2	-2.12	120.72	122.69
28	a	1962	5MC	O2-C2-N3	-2.11	119.01	122.33
7	A	1408	5MC	O2-C2-N3	-2.10	119.01	122.33
28	a	1618	6MZ	C4-C5-N7	-2.08	107.14	109.34
28	a	747	5MU	O2-C2-N1	-2.07	120.10	122.80
28	a	1835	2MG	O6-C6-C5	-2.06	120.24	124.32
7	A	967	5MC	C5-C4-N3	-2.06	119.65	121.75
28	a	2503	2MA	N6-C6-N1	2.05	122.62	117.11
7	A	1403	4OC	O4'-C4'-C3'	-2.04	101.11	105.15
28	a	746	PSU	C6-N1-C2	-2.01	120.82	122.69
7	A	1517	2MG	O6-C6-C5	-2.01	120.33	124.32

All (1) chirality outliers are listed below:

Mol	Chain	Res	Type	Atom
17	K	119	BH2	CB

All (45) torsion outliers are listed below:

Mol	Chain	Res	Type	Atoms
18	L	89	D2T	O-C-CA-CB
18	L	89	D2T	CG-CB-SB-CB1
7	A	527	G7M	O4'-C4'-C5'-O5'
7	A	1519	MA6	C5-C6-N6-C9
17	K	119	BH2	CA-CB-CG-OD1
28	a	1915	3TD	O4'-C1'-C5-C4
28	a	1915	3TD	O4'-C1'-C5-C6
28	a	2251	OMG	C1'-C2'-O2'-CM2
7	A	527	G7M	C3'-C4'-C5'-O5'
7	A	1403	4OC	C3'-C4'-C5'-O5'
28	a	2498	OMC	O4'-C4'-C5'-O5'
39	l	82	MS6	CA-CB-CG-SD
7	A	1519	MA6	O4'-C4'-C5'-O5'
28	a	1915	3TD	C3'-C4'-C5'-O5'
28	a	1915	3TD	O4'-C4'-C5'-O5'
28	a	2030	6MZ	O4'-C4'-C5'-O5'
31	d	150	MEQ	CA-CB-CG-CD
28	a	2030	6MZ	C3'-C4'-C5'-O5'
28	a	2504	PSU	O4'-C4'-C5'-O5'
7	A	516	PSU	C4'-C5'-O5'-P
28	a	2498	OMC	C3'-C4'-C5'-O5'

Continued on next page...

For Manuscript Review

Continued from previous page...

Mol	Chain	Res	Type	Atoms
7	A	1499	UR3	O4'-C4'-C5'-O5'
39	l	82	MS6	CB-CG-SD-CE
31	d	150	MEQ	OE1-CD-CG-CB
7	A	1403	4OC	O4'-C4'-C5'-O5'
31	d	150	MEQ	NE2-CD-CG-CB
28	a	2503	2MA	O4'-C4'-C5'-O5'
39	l	81	4D4	CA-CB-CG-CD
18	L	89	D2T	SB-CB-CG-OD1
7	A	1520	MA6	C5-C6-N6-C9
7	A	1520	MA6	O4'-C4'-C5'-O5'
39	l	81	4D4	OB-CB-CG-CD
28	a	2069	G7M	C4'-C5'-O5'-P
7	A	1499	UR3	C3'-C4'-C5'-O5'
7	A	1519	MA6	C3'-C4'-C5'-O5'
7	A	967	5MC	C4'-C5'-O5'-P
28	a	2504	PSU	C3'-C4'-C5'-O5'
28	a	2580	PSU	O4'-C4'-C5'-O5'
18	L	89	D2T	SB-CB-CG-OD2
28	a	2503	2MA	C3'-C4'-C5'-O5'
28	a	2604	PSU	O4'-C4'-C5'-O5'
28	a	746	PSU	O4'-C1'-C5-C6
18	L	89	D2T	CA-CB-CG-OD2
28	a	2498	OMC	C2'-C1'-N1-C2
28	a	2069	G7M	O4'-C4'-C5'-O5'

There are no ring outliers.

8 monomers are involved in 10 short contacts:

Mol	Chain	Res	Type	Clashes	Symm-Clashes
28	a	1917	PSU	1	0
7	A	967	5MC	2	0
18	L	89	D2T	1	0
7	A	1519	MA6	1	0
7	A	1403	4OC	3	0
28	a	2251	OMG	1	0
7	A	1517	2MG	1	0
7	A	1520	MA6	1	0

5.5 Carbohydrates (i)

There are no oligosaccharides in this entry.

For Manuscript Review

5.6 Ligand geometry (i)

Of 289 ligands modelled in this entry, 274 are monoatomic - leaving 15 for Mogul analysis.

In the following table, the Counts columns list the number of bonds (or angles) for which Mogul statistics could be retrieved, the number of bonds (or angles) that are observed in the model and the number of bonds (or angles) that are defined in the Chemical Component Dictionary. The Link column lists molecule types, if any, to which the group is linked. The Z score for a bond length (or angle) is the number of standard deviations the observed value is removed from the expected value. A bond length (or angle) with $|Z| > 2$ is considered an outlier worth inspection. RMSZ is the root-mean-square of all Z scores of the bond lengths (or angles).

Mol	Type	Chain	Res	Link	Bond lengths			Bond angles		
					Counts	RMSZ	$\#\|Z\| > 2$	Counts	RMSZ	$\#\|Z\| > 2$
56	SPD	a	6211	-	9,9,9	0.32	0	8,8,8	0.76	0
56	SPD	a	6217	-	9,9,9	0.33	0	8,8,8	0.83	0
56	SPD	a	6210	-	9,9,9	0.31	0	8,8,8	0.85	0
56	SPD	a	6218	-	9,9,9	0.29	0	8,8,8	0.83	0
57	SPM	a	6221	-	13,13,13	0.34	0	12,12,12	0.90	0
56	SPD	a	6209	-	9,9,9	0.33	0	8,8,8	0.70	0
56	SPD	a	6215	-	9,9,9	0.33	0	8,8,8	0.82	0
56	SPD	a	6216	-	9,9,9	0.32	0	8,8,8	0.83	0
56	SPD	a	6220	-	9,9,9	0.33	0	8,8,8	0.79	0
56	SPD	a	6213	-	9,9,9	0.32	0	8,8,8	0.83	0
56	SPD	a	6219	-	9,9,9	0.36	0	8,8,8	0.82	0
56	SPD	a	6214	-	9,9,9	0.33	0	8,8,8	0.96	0
56	SPD	a	6207	-	9,9,9	0.33	0	8,8,8	0.81	0
56	SPD	a	6208	-	9,9,9	0.32	0	8,8,8	0.77	0
56	SPD	a	6212	-	9,9,9	0.33	0	8,8,8	0.81	0

In the following table, the Chirals column lists the number of chiral outliers, the number of chiral centers analysed, the number of these observed in the model and the number defined in the Chemical Component Dictionary. Similar counts are reported in the Torsion and Rings columns. '-' means no outliers of that kind were identified.

Mol	Type	Chain	Res	Link	Chirals	Torsions	Rings
56	SPD	a	6211	-	-	0/7/7/7	-
56	SPD	a	6217	-	-	3/7/7/7	-
56	SPD	a	6210	-	-	0/7/7/7	-
56	SPD	a	6218	-	-	1/7/7/7	-
57	SPM	a	6221	-	-	3/11/11/11	-
56	SPD	a	6209	-	-	2/7/7/7	-
56	SPD	a	6215	-	-	2/7/7/7	-
56	SPD	a	6216	-	-	2/7/7/7	-

Continued on next page...

For Manuscript Review

Continued from previous page...

Mol	Type	Chain	Res	Link	Chirals	Torsions	Rings
56	SPD	a	6220	-	-	1/7/7/7	-
56	SPD	a	6213	-	-	1/7/7/7	-
56	SPD	a	6219	-	-	2/7/7/7	-
56	SPD	a	6214	-	-	1/7/7/7	-
56	SPD	a	6207	-	-	3/7/7/7	-
56	SPD	a	6208	-	-	3/7/7/7	-
56	SPD	a	6212	-	-	2/7/7/7	-

There are no bond length outliers.

There are no bond angle outliers.

There are no chirality outliers.

All (26) torsion outliers are listed below:

Mol	Chain	Res	Type	Atoms
57	a	6221	SPM	N10-C11-C12-C13
56	a	6209	SPD	C8-C7-N6-C5
56	a	6215	SPD	C8-C7-N6-C5
57	a	6221	SPM	N5-C6-C7-C8
56	a	6208	SPD	C4-C5-N6-C7
56	a	6208	SPD	N1-C2-C3-C4
56	a	6208	SPD	C2-C3-C4-C5
56	a	6207	SPD	N6-C7-C8-C9
56	a	6216	SPD	N1-C2-C3-C4
56	a	6212	SPD	N1-C2-C3-C4
56	a	6214	SPD	C2-C3-C4-C5
56	a	6216	SPD	C2-C3-C4-C5
56	a	6217	SPD	N6-C7-C8-C9
56	a	6213	SPD	N1-C2-C3-C4
56	a	6217	SPD	C7-C8-C9-N10
57	a	6221	SPM	C11-C12-C13-N14
56	a	6217	SPD	N1-C2-C3-C4
56	a	6218	SPD	N6-C7-C8-C9
56	a	6212	SPD	C2-C3-C4-C5
56	a	6220	SPD	C2-C3-C4-C5
56	a	6215	SPD	C2-C3-C4-C5
56	a	6207	SPD	C4-C5-N6-C7
56	a	6209	SPD	C4-C5-N6-C7
56	a	6219	SPD	C2-C3-C4-C5
56	a	6207	SPD	N1-C2-C3-C4
56	a	6219	SPD	C7-C8-C9-N10

For Manuscript Review

There are no ring outliers.

10 monomers are involved in 13 short contacts:

Mol	Chain	Res	Type	Clashes	Symm-Clashes
56	a	6211	SPD	2	0
56	a	6217	SPD	1	0
56	a	6210	SPD	2	0
56	a	6218	SPD	1	0
57	a	6221	SPM	1	0
56	a	6215	SPD	1	0
56	a	6220	SPD	1	0
56	a	6219	SPD	1	0
56	a	6214	SPD	2	0
56	a	6208	SPD	1	0

5.7 Other polymers (i)

There are no such residues in this entry.

5.8 Polymer linkage issues (i)

The following chains have linkage breaks:

Mol	Chain	Number of breaks
7	A	3
28	a	2
18	L	1

All chain breaks are listed below:

Model	Chain	Residue-1	Atom-1	Residue-2	Atom-2	Distance (Å)
1	A	1267:G	O3'	1271:G	P	15.18
1	A	840:C	O3'	846:G	P	13.53
1	A	204:G	O3'	214:C	P	10.50
1	a	1914:C	O3'	1915:3TD	P	5.23
1	a	1915:3TD	O3'	1916:A	P	4.29
1	L	89:D2T	C	90:LEU	N	3.30

For Manuscript Review

6 Map visualisation (i)

This section contains visualisations of the EMDB entry EMD-75634. These allow visual inspection of the internal detail of the map and identification of artifacts.

Images derived from a raw map, generated by summing the deposited half-maps, are presented below the corresponding image components of the primary map to allow further visual inspection and comparison with those of the primary map.

6.1 Orthogonal projections (i)

6.1.1 Primary map

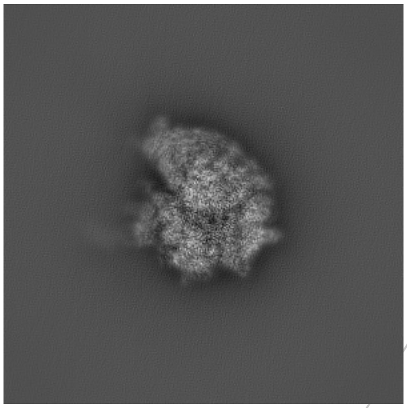

X

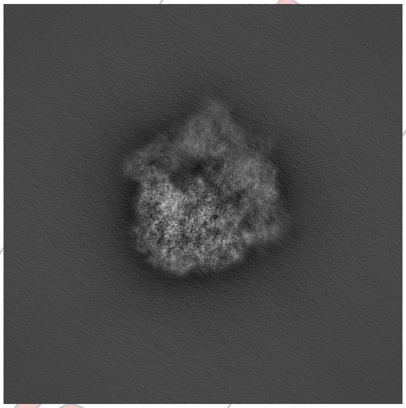

Y

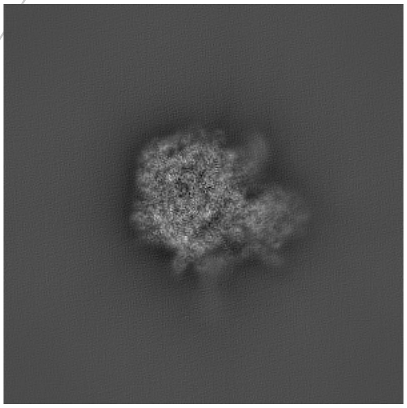

Z

6.1.2 Raw map

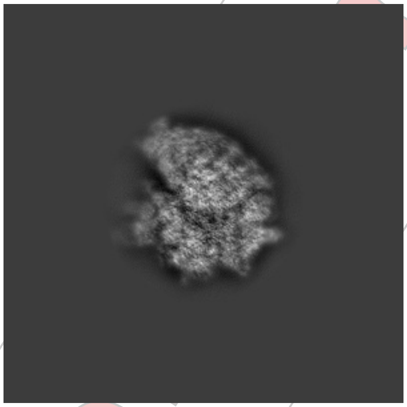

X

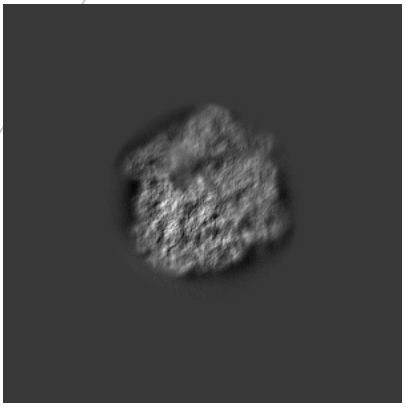

Y

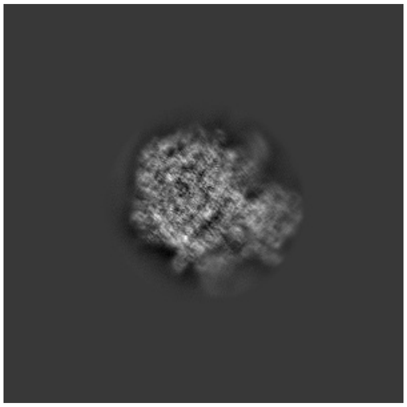

Z

The images above show the map projected in three orthogonal directions.

For Manuscript Review

6.2 Central slices (i)

6.2.1 Primary map

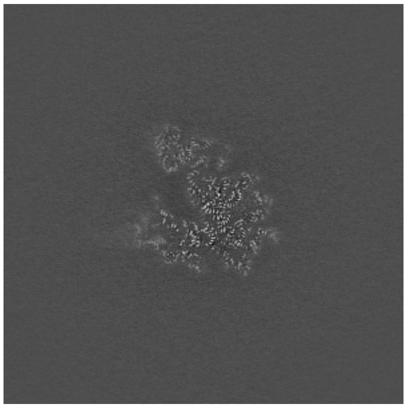

X Index: 240

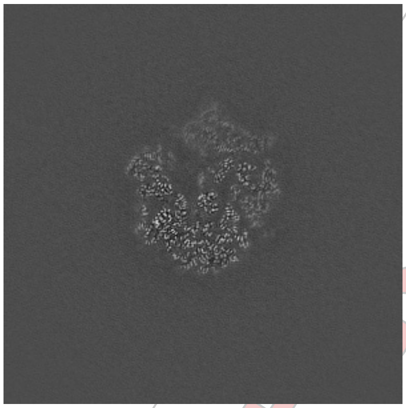

Y Index: 240

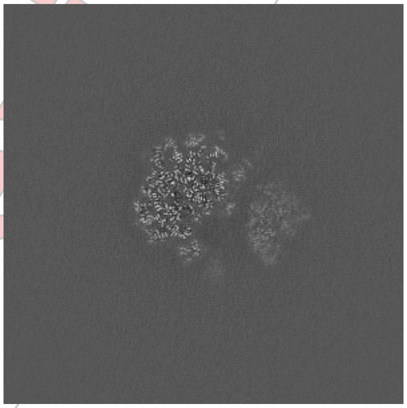

Z Index: 240

6.2.2 Raw map

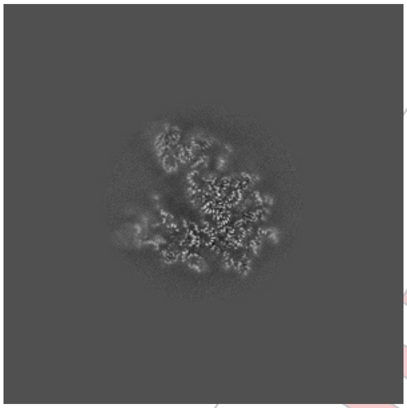

X Index: 240

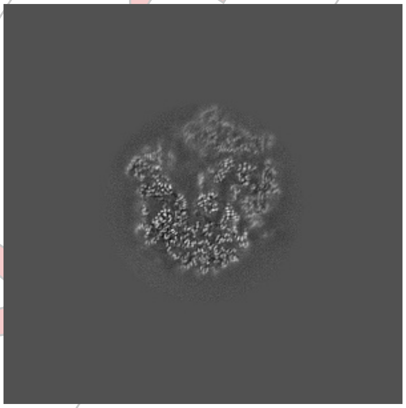

Y Index: 240

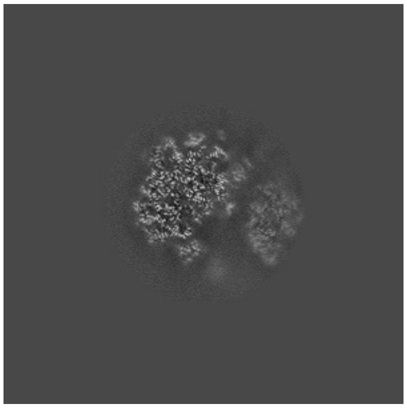

Z Index: 240

The images above show central slices of the map in three orthogonal directions.

For Manuscript Review

6.3 Largest variance slices ⓘ

6.3.1 Primary map

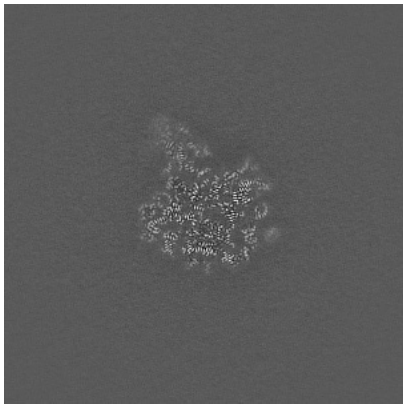
X Index: 218

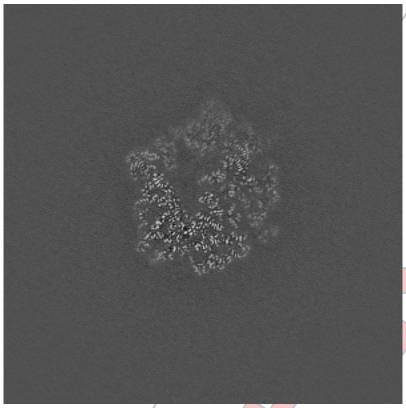
Y Index: 234

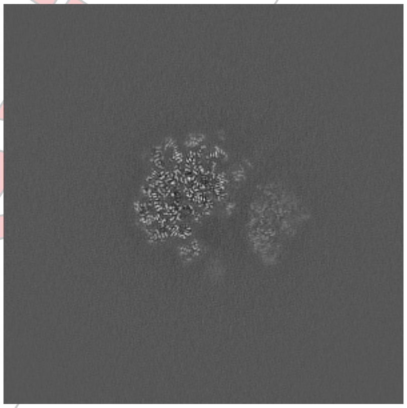
Z Index: 240

6.3.2 Raw map

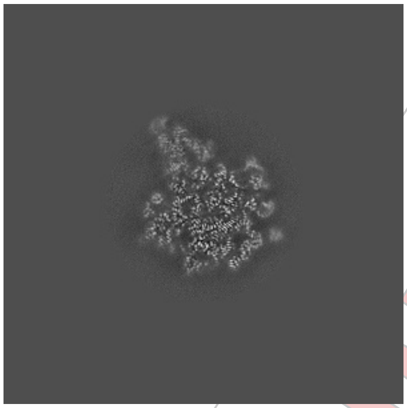
X Index: 224

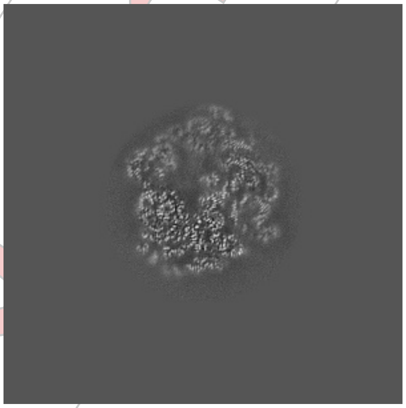
Y Index: 229

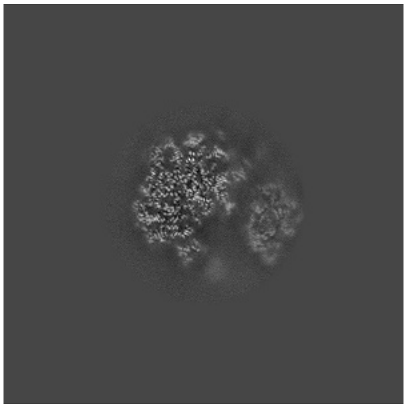
Z Index: 239

The images above show the largest variance slices of the map in three orthogonal directions.

For Manuscript Review

6.4 Orthogonal standard-deviation projections (False-color) (i)

6.4.1 Primary map

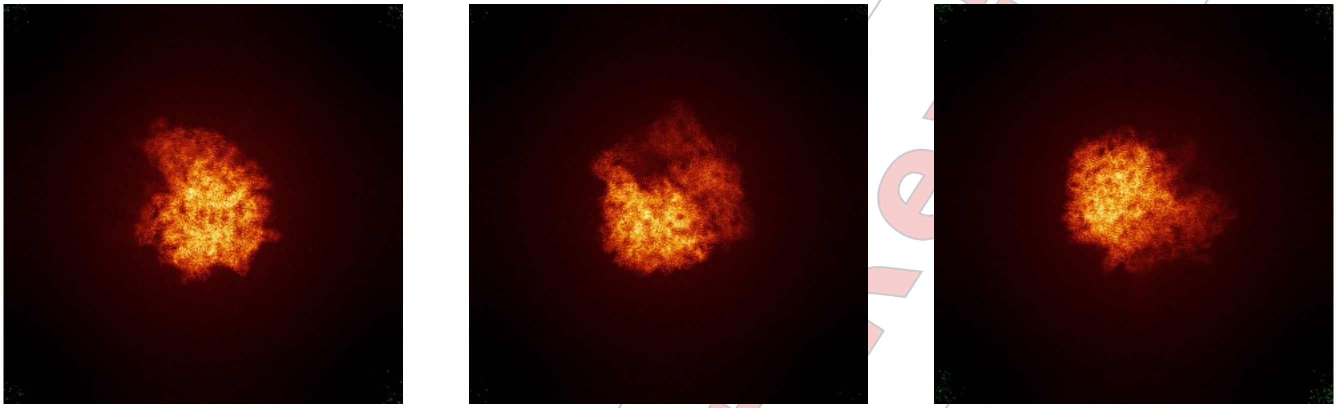

X Y Z

6.4.2 Raw map

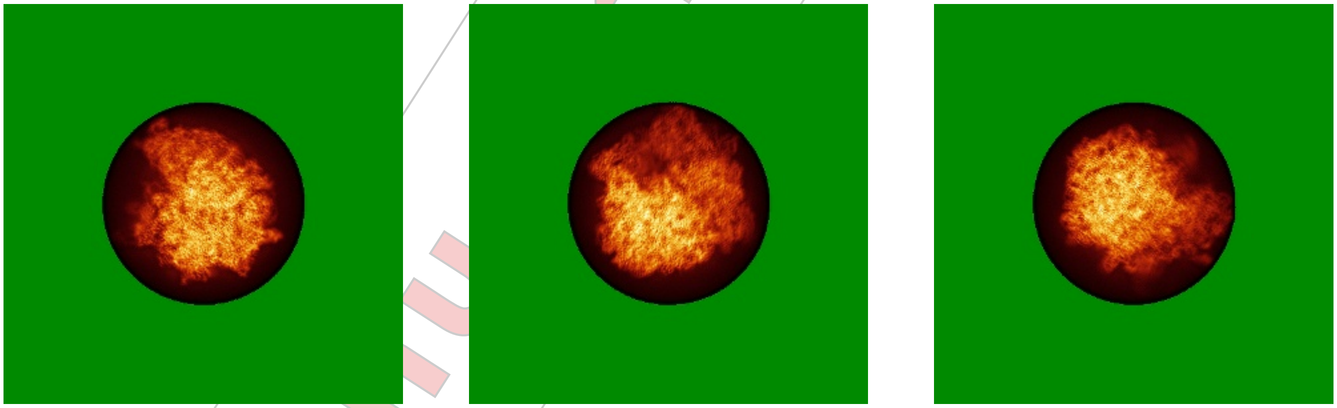

X Y Z

The images above show the map standard deviation projections with false color in three orthogonal directions. Minimum values are shown in green, max in blue, and dark to light orange shades represent small to large values respectively.

For Manuscript Review

6.5 Orthogonal surface views (i)

6.5.1 Primary map

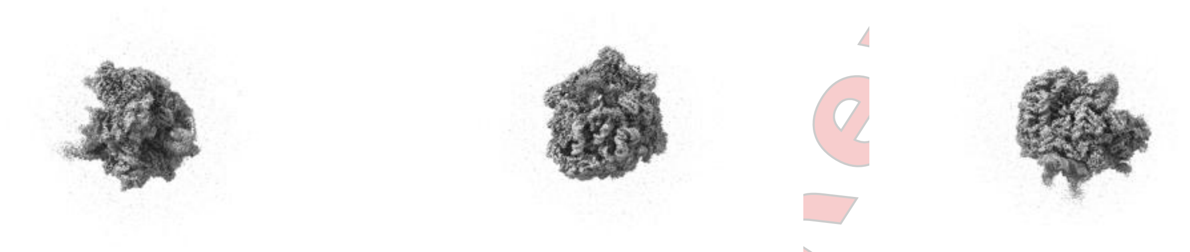

X Y Z

The images above show the 3D surface view of the map at the recommended contour level 2.8. These images, in conjunction with the slice images, may facilitate assessment of whether an appropriate contour level has been provided.

6.5.2 Raw map

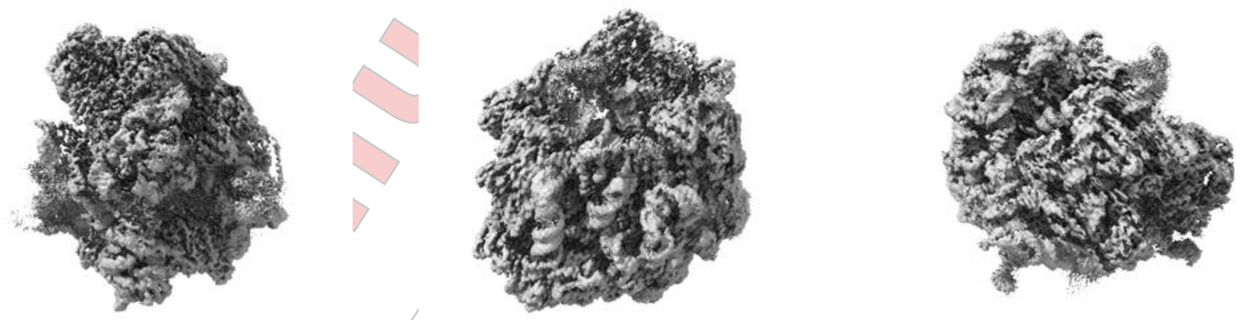

X Y Z

These images show the 3D surface of the raw map. The raw map's contour level was selected so that its surface encloses the same volume as the primary map does at its recommended contour level.

6.6 Mask visualisation (i)

This section was not generated. No masks/segmentation were deposited.

For Manuscript Review

7 Map analysis (i)

This section contains the results of statistical analysis of the map.

7.1 Map-value distribution (i)

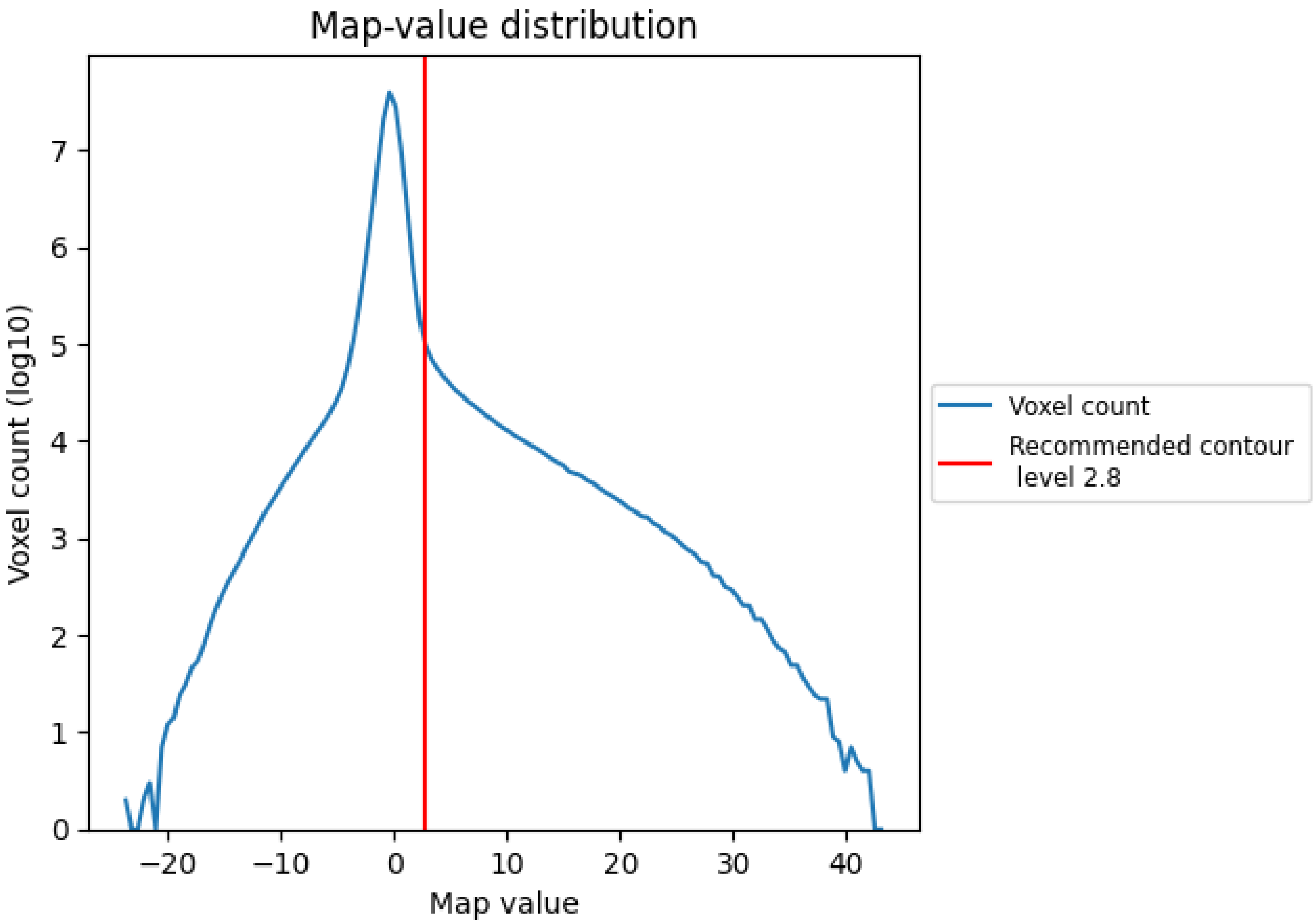

The map-value distribution is plotted in 128 intervals along the x-axis. The y-axis is logarithmic. A spike in this graph at zero usually indicates that the volume has been masked.

For Manuscript Review

7.2 Volume estimate (i)

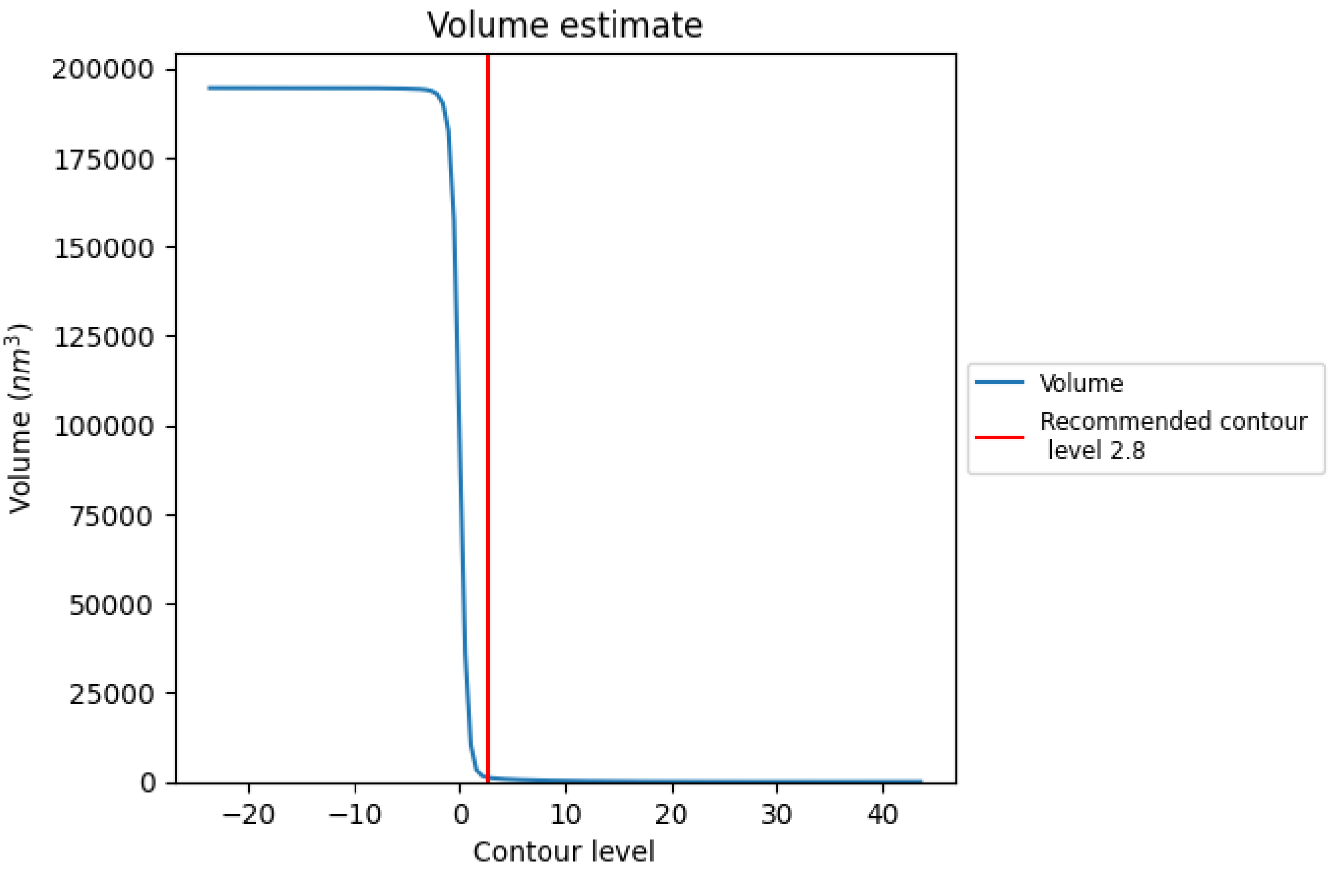

The volume at the recommended contour level is 1155 nm^3; this corresponds to an approximate mass of 1044 kDa.

The volume estimate graph shows how the enclosed volume varies with the contour level. The recommended contour level is shown as a vertical line and the intersection between the line and the curve gives the volume of the enclosed surface at the given level.

For Manuscript Review

7.3 Rotationally averaged power spectrum (i)

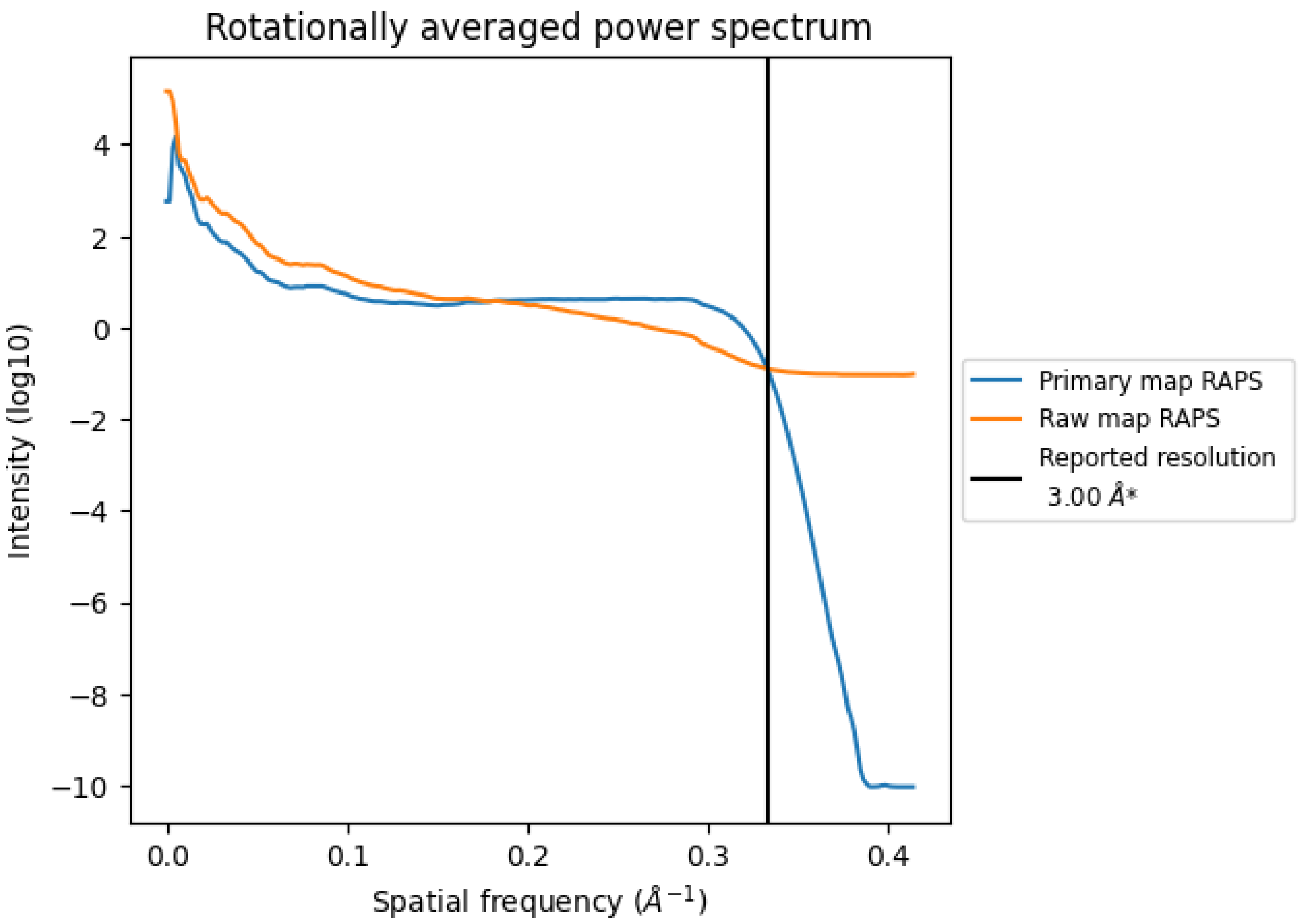

*Reported resolution corresponds to spatial frequency of 0.333 Å^{-1}

For Manuscript Review

8 Fourier-Shell correlation ⓘ

Fourier-Shell Correlation (FSC) is the most commonly used method to estimate the resolution of single-particle and subtomogram-averaged maps. The shape of the curve depends on the imposed symmetry, mask and whether or not the two 3D reconstructions used were processed from a common reference. The reported resolution is shown as a black line. A curve is displayed for the half-bit criterion in addition to lines showing the 0.143 gold standard cut-off and 0.5 cut-off.

8.1 FSC ⓘ

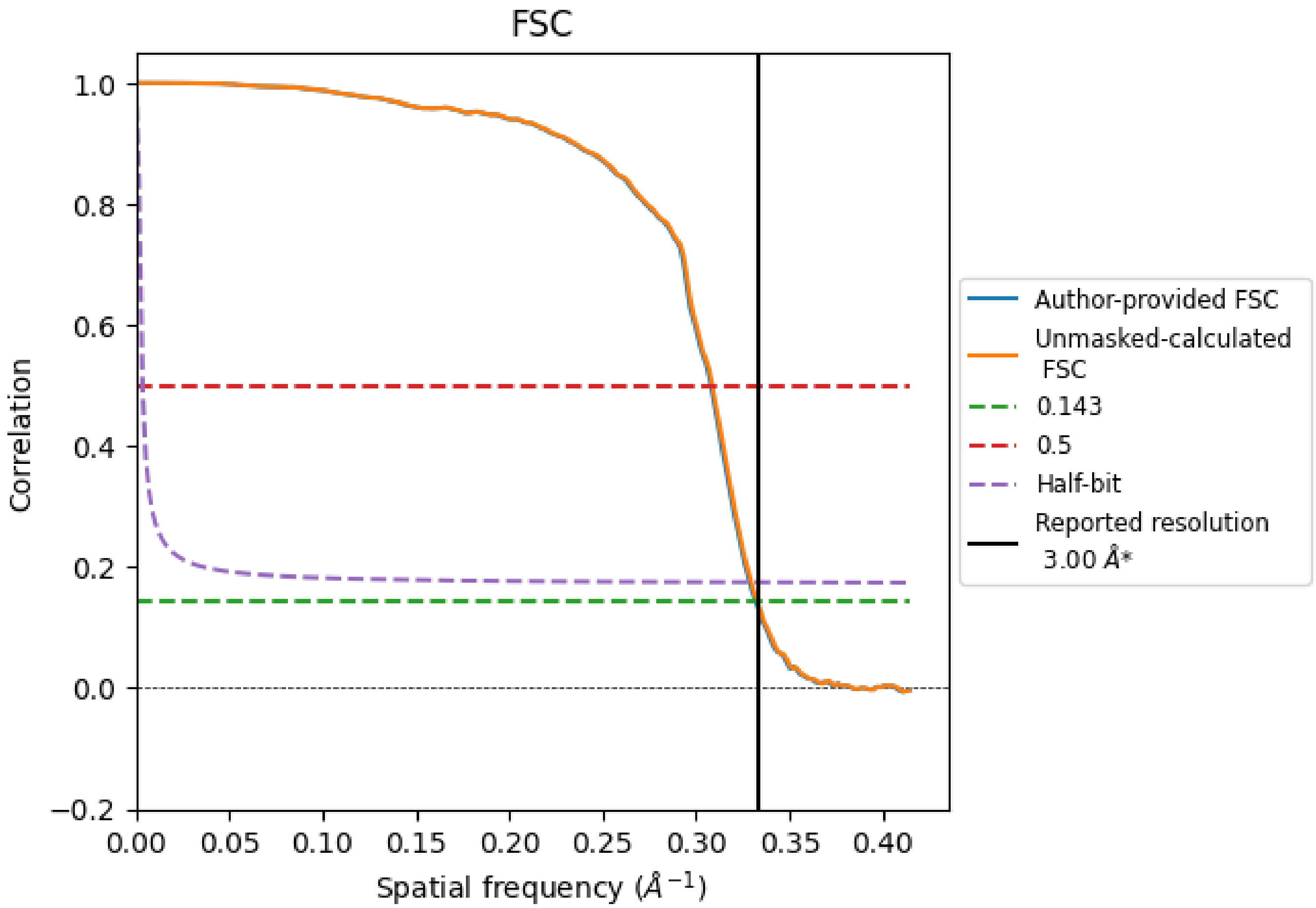

*Reported resolution corresponds to spatial frequency of 0.333 $Å^{-1}$

For Manuscript Review

8.2 Resolution estimates (i)

Resolution estimate (Å)	Estimation criterion (FSC cut-off)		
	0.143	0.5	Half-bit
Reported by author	3.00	-	-
Author-provided FSC curve	3.01	3.25	3.04
Unmasked-calculated*	3.00	3.24	3.03

*Resolution estimate based on FSC curve calculated by comparison of deposited half-maps.

For Manuscript Review

9 Map-model fit (i)

This section contains information regarding the fit between EMDB map EMD-75634 and PDB model 11DG. Per-residue inclusion information can be found in section 3 on page 17.

9.1 Map-model overlay (i)

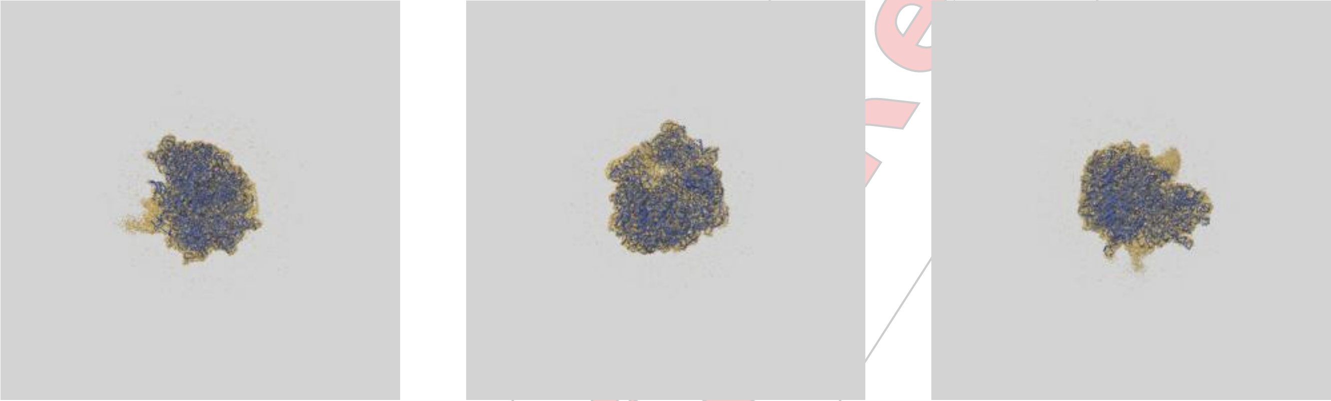

X Y Z

The images above show the 3D surface view of the map at the recommended contour level 2.8 at 50% transparency in yellow overlaid with a ribbon representation of the model coloured in blue. These images allow for the visual assessment of the quality of fit between the atomic model and the map.

For Manuscript Review

9.2 Q-score mapped to coordinate model (i)

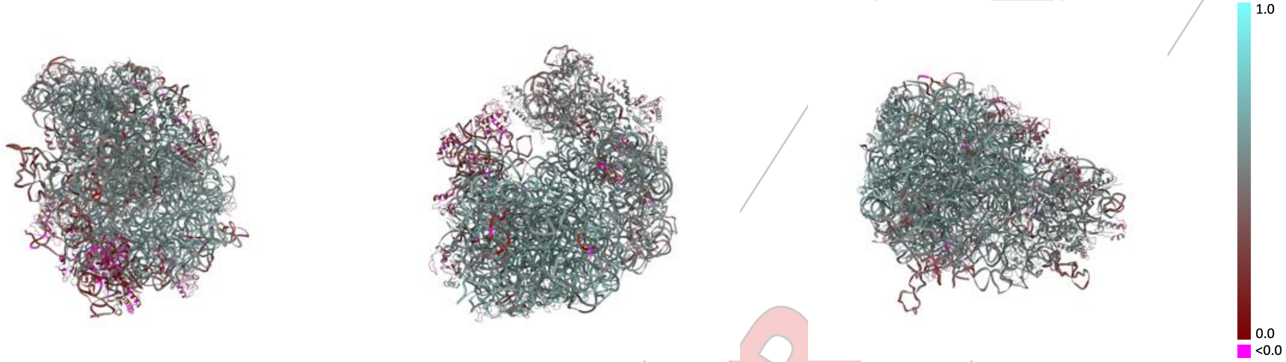

X Y Z

The images above show the model with each residue coloured according its Q-score. This shows their resolvability in the map with higher Q-score values reflecting better resolvability. Please note: Q-score is calculating the resolvability of atoms, and thus high values are only expected at resolutions at which atoms can be resolved. Low Q-score values may therefore be expected for many entries.

9.3 Atom inclusion mapped to coordinate model (i)

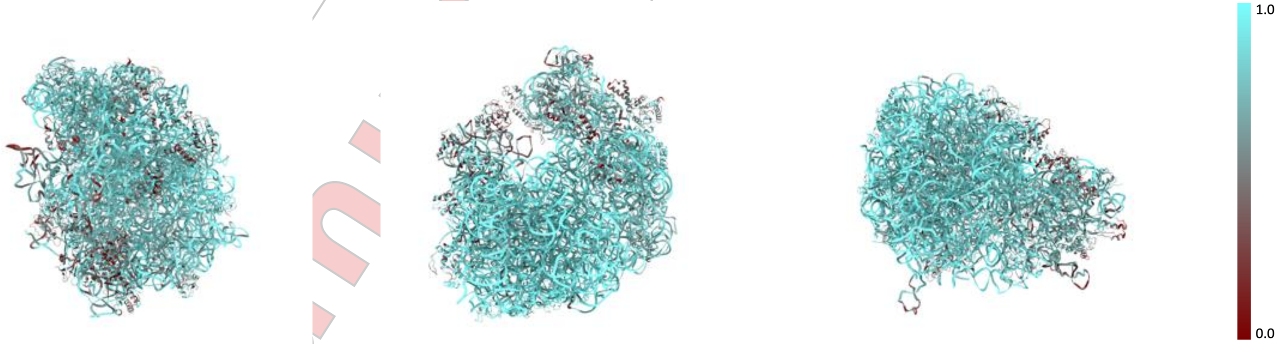

X Y Z

The images above show the model with each residue coloured according to its atom inclusion. This shows to what extent they are inside the map at the recommended contour level (2.8).

For Manuscript Review

9.4 Atom inclusion (i)

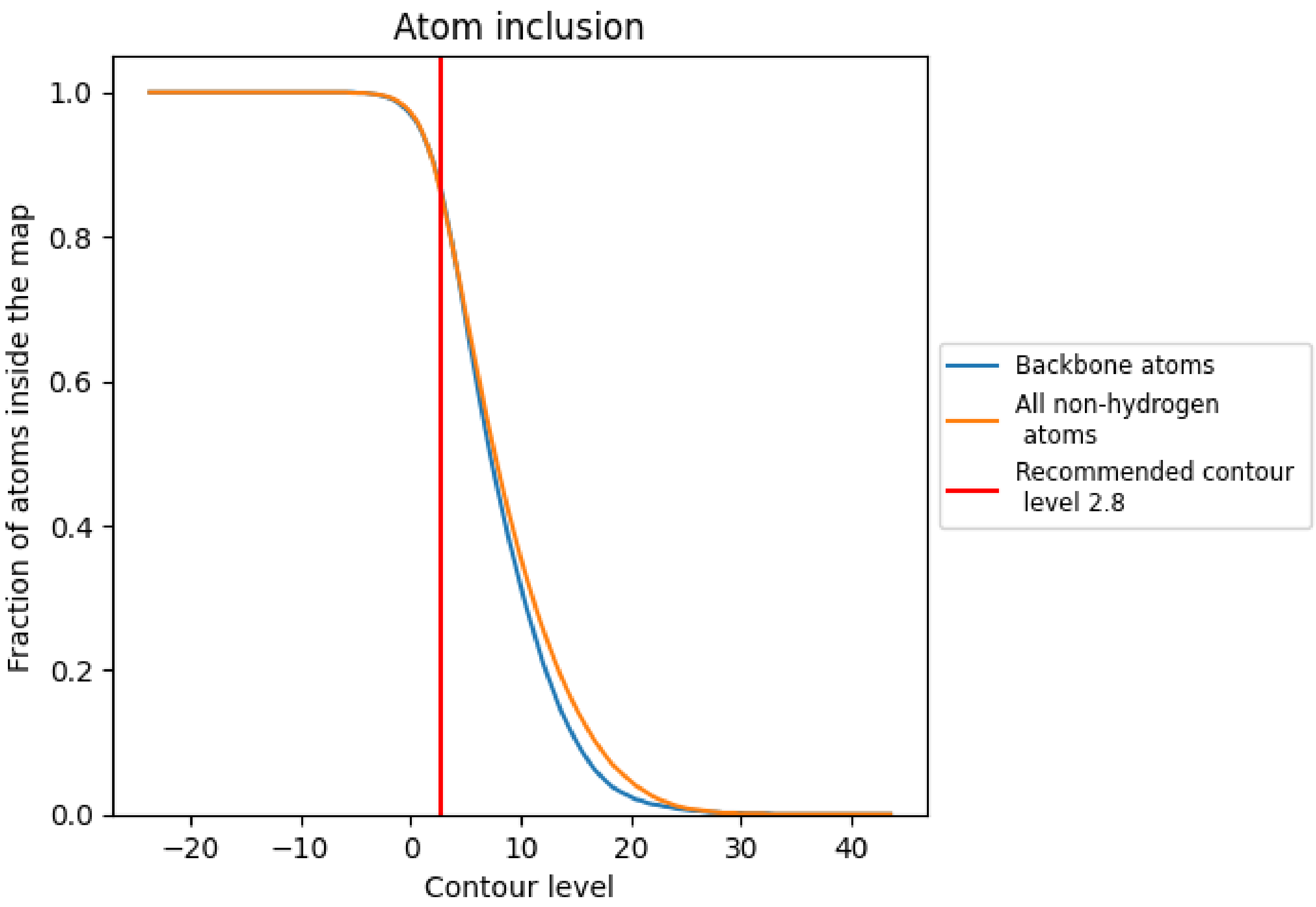

At the recommended contour level, 86% of all backbone atoms, 86% of all non-hydrogen atoms, are inside the map.

For Manuscript Review

9.5 Map-model fit summary (i)

The table lists the average atom inclusion at the recommended contour level (2.8) and Q-score for the entire model and for each chain.

Chain	Atom inclusion	Q-score
All	0.8590	0.5060
0	0.5280	0.2890
1	0.9270	0.6130
2	0.8900	0.5760
3	0.6210	0.2870
4	0.4700	0.2220
5	0.5950	0.5370
A	0.8880	0.5080
B	0.5610	0.4390
C	0.6610	0.4910
D	0.6900	0.4990
E	0.8300	0.5460
F	0.5260	0.2720
G	0.4750	0.4070
H	0.8370	0.5570
I	0.7250	0.4660
J	0.5270	0.4340
K	0.8120	0.5110
L	0.8450	0.5510
M	0.5470	0.3970
N	0.7430	0.5010
O	0.7670	0.4440
P	0.7810	0.5160
Q	0.7870	0.5260
R	0.7250	0.3960
S	0.5910	0.4310
T	0.7940	0.5150
U	0.3560	0.3510
a	0.9390	0.5480
b	0.6440	0.2780
c	0.9470	0.6030
d	0.8730	0.5300
e	0.8300	0.4840
f	0.4530	0.1560
g	0.5260	0.2010

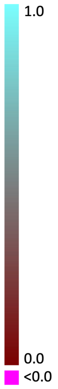

Continued on next page...

For Manuscript Review

Continued from previous page...

Chain	Atom inclusion	Q-score
h	0.7770	0.4560
i	0.8360	0.4950
j	0.8980	0.5870
k	0.8070	0.4540
l	0.8740	0.5310
m	0.9590	0.5990
n	0.4790	0.1840
o	0.9060	0.5830
p	0.8140	0.4450
q	0.7590	0.4290
r	0.8950	0.5820
s	0.9040	0.5680
t	0.8630	0.5180
u	0.5150	0.1950
v	0.7900	0.4710
w	0.9270	0.5860
x	0.8610	0.5300
y	0.8720	0.5210
z	0.9040	0.5920

For Manuscript Review
