# Supplementary material for: Structural adaptations for enhanced translation kinetics in evolved ribosomes
Source: Nucleic Acids Res. 2026 Jul 22;54(14):gkag703. doi: 10.1093/nar/gkag703 (PMC13389312; doi:10.1093/nar/gkag703)
Supplement: gkag703_Supplemental_Files [file gkag703_supplemental_files.zip › PDB_validation.zip!/PA_S3.3_D_1000303769_val-report-full-annotate_P1.pdf]

Full wwPDB EM Validation Report (i)

Mar 3, 2026 – 02:15 PM EST

PDB ID	:	11FV / pdb_000011fv
EMDB ID	:	EMD-75668
Title	:	Chimeric Escherichia coli 70S ribosome containing an evolved 16S rRNA from Pseudomonas aeruginosa (PA-S3.3)
Deposited on	:	2026-02-21
Resolution	:	2.90 Å(reported)

This wwPDB validation report is for manuscript review

For Manuscript Review

This is a Full wwPDB EM Validation Report.

This report is produced by the wwPDB biocuration pipeline after annotation of the structure.

We welcome your comments at *validation@mail.wwpdb.org*
A user guide is available at
https://www.wwpdb.org/validation/2017/EMValidationReportHelp
with specific help available everywhere you see the (i) symbol.

The types of validation reports are described at
http://www.wwpdb.org/validation/2017/FAQs#types.

The following versions of software and data (see references (i)) were used in the production of this report:

EMDB validation analysis	:	0.0.1.dev132
Mogul	:	2022.3.0, CSD as543be (2022)
MolProbity	:	4-5-2 with Phenix2.0
Percentile statistics	:	20231227.v01 (using entries in the PDB archive December 27th 2023)
EM percentile statistics	:	202505.v01 (Using data in the EMDB archive up until May 2025)
MapQ	:	1.9.13
Ideal geometry (proteins)	:	Engh & Huber (2001)
Ideal geometry (DNA, RNA)	:	Parkinson et al. (1996)

1 Overall quality at a glance (i)

The following experimental techniques were used to determine the structure:
ELECTRON MICROSCOPY

The reported resolution of this entry is 2.90 Å.

Percentile scores (ranging between 0-100) for global validation metrics of the entry are shown in the following graphic. The table shows the number of entries on which the scores are based.

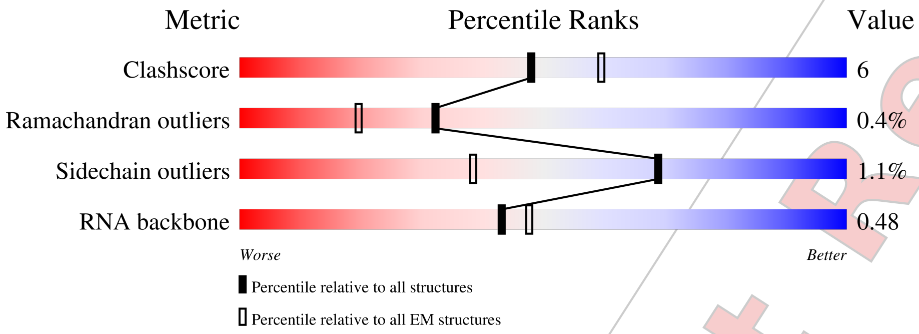

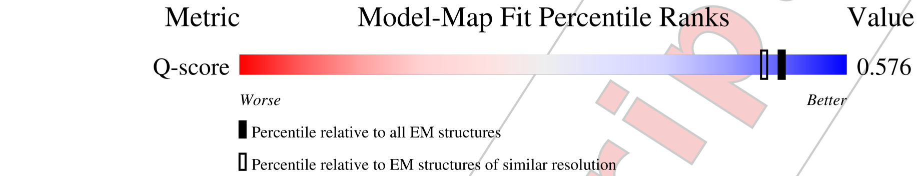

Metric	Whole archive (#Entries)	EM structures (#Entries)	Similar EM resolution (#Entries, resolution range(Å))
Clashscore	210492	15764	-
Ramachandran outliers	207382	16835	-
Sidechain outliers	206894	16415	-
RNA backbone	6643	2191	-
Q-score	-	25397	13054 (2.40 - 3.40)

The table below summarises the geometric issues observed across the polymeric chains and their fit to the map. The red, orange, yellow and green segments of the bar indicate the fraction of residues that contain outliers for >=3, 2, 1 and 0 types of geometric quality criteria respectively. A grey segment represents the fraction of residues that are not modelled. The numeric value for each fraction is indicated below the corresponding segment, with a dot representing fractions <=5% The upper red bar (where present) indicates the fraction of residues that have poor fit to the EM map (all-atom inclusion < 40%). The numeric value is given above the bar.

Mol	Chain	Length	Quality of chain
1	C	1526	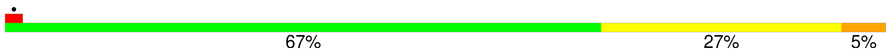

Continued on next page...

For Manuscript Review

Continued from previous page...

Mol	Chain	Length	Quality of chain
2	0	110	• 94% 6%
3	1	81	9% 75% 25%
4	Z	103	• 89% 9% •
5	2	87	• 86% 10% • •
6	3	71	41% 85% 14% •
7	b	100	• 79% 14% 7%
8	c	104	5% 86% 12% • •
9	D	241	28% 67% 23% 10%
10	d	94	87% 13%
11	E	233	• 72% 16% 12%
12	e	85	• 87% • 12%
13	F	206	• 77% 22%
14	f	78	• 91% 8% •
15	G	167	• 78% 11% 10%
16	g	63	5% 89% 10% •
17	H	135	• 62% 12% 26%
18	h	59	• 93% 5% •
19	I	2904	5% 72% 23% 5%
20	i	57	• 82% 16% •
21	J	120	77% 21% • •
22	j	55	73% 58% 31% • 9%
23	K	273	• 84% 15% •
24	k	46	• 98% •
25	L	209	92% 8%
26	l	65	85% 14% •

Continued on next page...

For Manuscript Review

Continued from previous page...

Mol	Chain	Length	Quality of chain
27	M	201	5% / 88% 12%
28	m	38	92% 8%
29	N	179	• / 85% 14% •
30	n	179	15% / 64% 19% • 16%
31	O	177	• / 85% 14% ••
32	o	130	88% 11% •
33	P	149	87% / 64% 36%
34	p	130	14% / 58% 37% • •
35	Q	70	7% / 61% 24% 14%
36	q	103	20% / 21% 42% 32% 5%
37	R	142	85% 15%
38	r	129	• / 66% 25% 9%
39	S	123	88% 11% •
40	t	124	• / 83% 16% •
41	T	144	• / 84% 16%
42	u	118	• / 88% 9% •
43	U	136	• / 90% 10%
44	v	101	13% / 64% 31% 5%
45	V	127	• / 89% 6% 6%
46	w	89	• / 90% 9% •
47	W	117	49% / 37% 62% •
48	x	82	7% / 78% 16% 6%
49	X	115	• / 91% 8% •
50	y	84	6% / 81% 14% 5%
51	Y	118	94% 5% •

Continued on next page...

For Manuscript Review

Continued from previous page...

Mol	Chain	Length	Quality of chain
52	z	75	60% 13% 27%

The following table lists non-polymeric compounds, carbohydrate monomers and non-standard residues in protein, DNA, RNA chains that are outliers for geometric or electron-density-fit criteria:

Mol	Type	Chain	Res	Chirality	Geometry	Clashes	Electron density
53	MG	C	1625	-	-	X	-
53	MG	I	3077	-	-	X	-

For Manuscript Review

2 Entry composition (i)

There are 53 unique types of molecules in this entry. The entry contains 142151 atoms, of which 0 are hydrogens and 0 are deuteriums.

In the tables below, the AltConf column contains the number of residues with at least one atom in alternate conformation and the Trace column contains the number of residues modelled with at most 2 atoms.

- Molecule 1 is a RNA chain called 16S ribosomal RNA.

Mol	Chain	Residues	Atoms					AltConf	Trace
			Total	C	N	O	P		
1	C	1526	32744	14605	6008	10606	1525	0	0

There are 3 discrepancies between the modelled and reference sequences:

Chain	Residue	Modelled	Actual	Comment	Reference
C	434	U	A	conflict	GB 3134256434
C	606	U	C	conflict	GB 3134256434
C	1472	C	U	conflict	GB 3134256434

- Molecule 2 is a protein called Large ribosomal subunit protein uL22.

Mol	Chain	Residues	Atoms					AltConf	Trace
			Total	C	N	O	S		
2	0	110	857	532	166	156	3	0	0

- Molecule 3 is a protein called Small ribosomal subunit protein uS19.

Mol	Chain	Residues	Atoms					AltConf	Trace
			Total	C	N	O	S		
3	1	81	651	416	124	109	2	0	0

- Molecule 4 is a protein called Large ribosomal subunit protein bL21.

Mol	Chain	Residues	Atoms					AltConf	Trace
			Total	C	N	O	S		
4	Z	103	816	516	153	145	2	0	0

- Molecule 5 is a protein called Small ribosomal subunit protein bS20.

Mol	Chain	Residues	Atoms					AltConf	Trace
			Total	C	N	O	S		
5	2	85	665	411	137	114	3	0	0

For Manuscript Review

- Molecule 6 is a protein called Small ribosomal subunit protein bS21.

Mol	Chain	Residues	Atoms					AltConf	Trace
6	3	70	Total 590	C 366	N 125	O 98	S 1	0	0

- Molecule 7 is a protein called 50S ribosomal protein L23.

Mol	Chain	Residues	Atoms					AltConf	Trace
7	b	93	Total 738	C 466	N 139	O 131	S 2	0	0

- Molecule 8 is a protein called 50S ribosomal protein L24.

Mol	Chain	Residues	Atoms				AltConf	Trace
8	c	102	Total 779	C 492	N 146	O 141	0	0

- Molecule 9 is a protein called Small ribosomal subunit protein uS2.

Mol	Chain	Residues	Atoms					AltConf	Trace
9	D	218	Total 1704	C 1081	N 305	O 311	S 7	0	0

- Molecule 10 is a protein called Large ribosomal subunit protein bL25.

Mol	Chain	Residues	Atoms					AltConf	Trace
10	d	94	Total 753	C 479	N 137	O 134	S 3	0	0

- Molecule 11 is a protein called Small ribosomal subunit protein uS3.

Mol	Chain	Residues	Atoms					AltConf	Trace
11	E	206	Total 1624	C 1028	N 305	O 288	S 3	0	0

- Molecule 12 is a protein called 50S ribosomal protein L27.

Mol	Chain	Residues	Atoms					AltConf	Trace
12	e	75	Total 575	C 356	N 116	O 102	S 1	0	0

- Molecule 13 is a protein called Small ribosomal subunit protein uS4.

For Manuscript Review

Mol	Chain	Residues	Atoms					AltConf	Trace
			Total	C	N	O	S		
13	F	205	1643	1026	315	298	4	0	0

- Molecule 14 is a protein called 50S ribosomal protein L28.

Mol	Chain	Residues	Atoms					AltConf	Trace
			Total	C	N	O	S		
14	f	77	625	388	129	106	2	0	0

- Molecule 15 is a protein called Small ribosomal subunit protein uS5.

Mol	Chain	Residues	Atoms					AltConf	Trace
			Total	C	N	O	S		
15	G	150	1105	687	211	201	6	0	0

- Molecule 16 is a protein called Large ribosomal subunit protein uL29.

Mol	Chain	Residues	Atoms					AltConf	Trace
			Total	C	N	O	S		
16	g	62	501	308	98	94	1	0	0

- Molecule 17 is a protein called Small ribosomal subunit protein bS6.

Mol	Chain	Residues	Atoms					AltConf	Trace
			Total	C	N	O	S		
17	H	100	817	515	148	148	6	0	0

- Molecule 18 is a protein called 50S ribosomal protein L30.

Mol	Chain	Residues	Atoms					AltConf	Trace
			Total	C	N	O	S		
18	h	58	449	281	87	79	2	0	0

- Molecule 19 is a RNA chain called 23S ribosomal RNA.

Mol	Chain	Residues	Atoms					AltConf	Trace
			Total	C	N	O	P		
19	I	2898	62271	27787	11455	20129	2900	2	0

- Molecule 20 is a protein called 50S ribosomal protein L32.

For Manuscript Review

Mol	Chain	Residues	Atoms					AltConf	Trace
			Total	C	N	O	S		
20	i	56	444	269	94	80	1	0	0

- Molecule 21 is a RNA chain called 5S ribosomal RNA.

Mol	Chain	Residues	Atoms					AltConf	Trace
			Total	C	N	O	P		
21	J	118	2529	1126	464	821	118	0	0

- Molecule 22 is a protein called 50S ribosomal protein L33.

Mol	Chain	Residues	Atoms				AltConf	Trace
			Total	C	N	O		
22	j	50	409	263	75	71	0	0

- Molecule 23 is a protein called 50S ribosomal protein L2.

Mol	Chain	Residues	Atoms					AltConf	Trace
			Total	C	N	O	S		
23	K	271	2093	1294	427	365	7	1	0

- Molecule 24 is a protein called Large ribosomal subunit protein bL34.

Mol	Chain	Residues	Atoms					AltConf	Trace
			Total	C	N	O	S		
24	k	46	377	228	90	57	2	0	0

- Molecule 25 is a protein called Large ribosomal subunit protein uL3.

Mol	Chain	Residues	Atoms					AltConf	Trace
			Total	C	N	O	S		
25	L	209	1565	979	288	294	4	0	0

- Molecule 26 is a protein called 50S ribosomal protein L35.

Mol	Chain	Residues	Atoms					AltConf	Trace
			Total	C	N	O	S		
26	l	64	504	323	105	74	2	0	0

- Molecule 27 is a protein called Large ribosomal subunit protein uL4.

For Manuscript Review

Mol	Chain	Residues	Atoms					AltConf	Trace
			Total	C	N	O	S		
27	M	201	1552	974	283	290	5	0	0

- Molecule 28 is a protein called Large ribosomal subunit protein bL36A.

Mol	Chain	Residues	Atoms					AltConf	Trace
			Total	C	N	O	S		
28	m	38	302	185	65	48	4	0	0

- Molecule 29 is a protein called Large ribosomal subunit protein uL5.

Mol	Chain	Residues	Atoms					AltConf	Trace
			Total	C	N	O	S		
29	N	177	1410	899	249	256	6	0	0

- Molecule 30 is a protein called Small ribosomal subunit protein uS7.

Mol	Chain	Residues	Atoms					AltConf	Trace
			Total	C	N	O	S		
30	n	151	1181	735	227	215	4	0	0

- Molecule 31 is a protein called Large ribosomal subunit protein uL6.

Mol	Chain	Residues	Atoms					AltConf	Trace
			Total	C	N	O	S		
31	O	176	1323	832	243	246	2	0	0

- Molecule 32 is a protein called Small ribosomal subunit protein uS8.

Mol	Chain	Residues	Atoms					AltConf	Trace
			Total	C	N	O	S		
32	o	129	979	616	173	184	6	0	0

- Molecule 33 is a protein called Large ribosomal subunit protein bL9.

Mol	Chain	Residues	Atoms					AltConf	Trace
			Total	C	N	O	S		
33	P	149	1111	699	197	214	1	0	0

- Molecule 34 is a protein called Small ribosomal subunit protein uS9.

For Manuscript Review

Mol	Chain	Residues	Atoms					AltConf	Trace
			Total	C	N	O	S		
34	p	127	1022	634	206	179	3	0	0

- Molecule 35 is a protein called 50S ribosomal protein L31.

Mol	Chain	Residues	Atoms					AltConf	Trace
			Total	C	N	O	S		
35	Q	60	480	299	90	85	6	0	0

- Molecule 36 is a protein called Small ribosomal subunit protein uS10.

Mol	Chain	Residues	Atoms					AltConf	Trace
			Total	C	N	O	S		
36	q	98	786	493	150	142	1	0	0

- Molecule 37 is a protein called Large ribosomal subunit protein uL13.

Mol	Chain	Residues	Atoms					AltConf	Trace
			Total	C	N	O	S		
37	R	142	1129	714	212	199	4	0	0

- Molecule 38 is a protein called 30S ribosomal protein S11.

Mol	Chain	Residues	Atoms					AltConf	Trace
			Total	C	N	O	S		
38	r	117	877	540	174	160	3	0	0

- Molecule 39 is a protein called Large ribosomal subunit protein uL14.

Mol	Chain	Residues	Atoms					AltConf	Trace
			Total	C	N	O	S		
39	S	122	938	587	180	165	6	0	0

- Molecule 40 is a protein called 30S ribosomal protein S12.

Mol	Chain	Residues	Atoms					AltConf	Trace
			Total	C	N	O	S		
40	t	123	955	590	196	165	4	0	0

- Molecule 41 is a protein called Large ribosomal subunit protein uL15.

For Manuscript Review

Mol	Chain	Residues	Atoms					AltConf	Trace
			Total	C	N	O	S		
41	T	144	1053	654	207	190	2	0	0

- Molecule 42 is a protein called Small ribosomal subunit protein uS13.

Mol	Chain	Residues	Atoms					AltConf	Trace
			Total	C	N	O	S		
42	u	115	891	552	179	157	3	0	0

- Molecule 43 is a protein called Large ribosomal subunit protein uL16.

Mol	Chain	Residues	Atoms					AltConf	Trace
			Total	C	N	O	S		
43	U	136	1075	686	205	178	6	0	0

- Molecule 44 is a protein called Small ribosomal subunit protein uS14.

Mol	Chain	Residues	Atoms					AltConf	Trace
			Total	C	N	O	S		
44	v	96	774	483	160	128	3	0	0

- Molecule 45 is a protein called Large ribosomal subunit protein bL17.

Mol	Chain	Residues	Atoms					AltConf	Trace
			Total	C	N	O	S		
45	V	120	960	593	196	166	5	0	0

- Molecule 46 is a protein called Small ribosomal subunit protein uS15.

Mol	Chain	Residues	Atoms					AltConf	Trace
			Total	C	N	O	S		
46	w	88	710	437	143	129	1	0	0

- Molecule 47 is a protein called Large ribosomal subunit protein uL18.

Mol	Chain	Residues	Atoms				AltConf	Trace
			Total	C	N	O		
47	W	116	892	552	178	162	0	0

- Molecule 48 is a protein called Small ribosomal subunit protein bS16.

For Manuscript Review

Mol	Chain	Residues	Atoms					AltConf	Trace
			Total	C	N	O	S		
48	x	82	649	406	128	114	1	0	0

- Molecule 49 is a protein called Large ribosomal subunit protein bL19.

Mol	Chain	Residues	Atoms					AltConf	Trace
			Total	C	N	O	S		
49	X	114	917	574	179	163	1	0	0

- Molecule 50 is a protein called Small ribosomal subunit protein uS17.

Mol	Chain	Residues	Atoms					AltConf	Trace
			Total	C	N	O	S		
50	y	80	648	411	121	113	3	0	0

- Molecule 51 is a protein called 50S ribosomal protein L20.

Mol	Chain	Residues	Atoms				AltConf	Trace
			Total	C	N	O		
51	Y	117	947	604	192	151	0	0

- Molecule 52 is a protein called Small ribosomal subunit protein bS18.

Mol	Chain	Residues	Atoms				AltConf	Trace
			Total	C	N	O		
52	z	55	455	288	86	81	0	0

- Molecule 53 is MAGNESIUM ION (CCD ID: MG) (formula: Mg).

Mol	Chain	Residues	Atoms		AltConf
			Total	Mg	
53	C	89	89	89	0
53	I	203	203	203	0
53	J	5	5	5	0
53	K	1	1	1	0
53	L	2	2	2	0
53	l	1	1	1	0

Continued on next page...

For Manuscript Review

Continued from previous page...

Mol	Chain	Residues	Atoms		AltConf
53	M	1	Total 1	Mg 1	0
53	p	1	Total 1	Mg 1	0
53	T	1	Total 1	Mg 1	0
53	u	1	Total 1	Mg 1	0
53	V	1	Total 1	Mg 1	0
53	Y	1	Total 1	Mg 1	0

For Manuscript Review

3 Residue-property plots

These plots are drawn for all protein, RNA, DNA and oligosaccharide chains in the entry. The first graphic for a chain summarises the proportions of the various outlier classes displayed in the second graphic. The second graphic shows the sequence view annotated by issues in geometry and atom inclusion in map density. Residues are color-coded according to the number of geometric quality criteria for which they contain at least one outlier: green = 0, yellow = 1, orange = 2 and red = 3 or more. A red diamond above a residue indicates a poor fit to the EM map for this residue (all-atom inclusion < 40%). Stretches of 2 or more consecutive residues without any outlier are shown as a green connector. Residues present in the sample, but not in the model, are shown in grey.

- Molecule 1: 16S ribosomal RNA

Chain C:

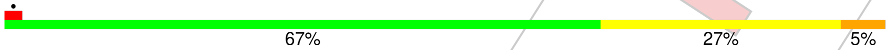

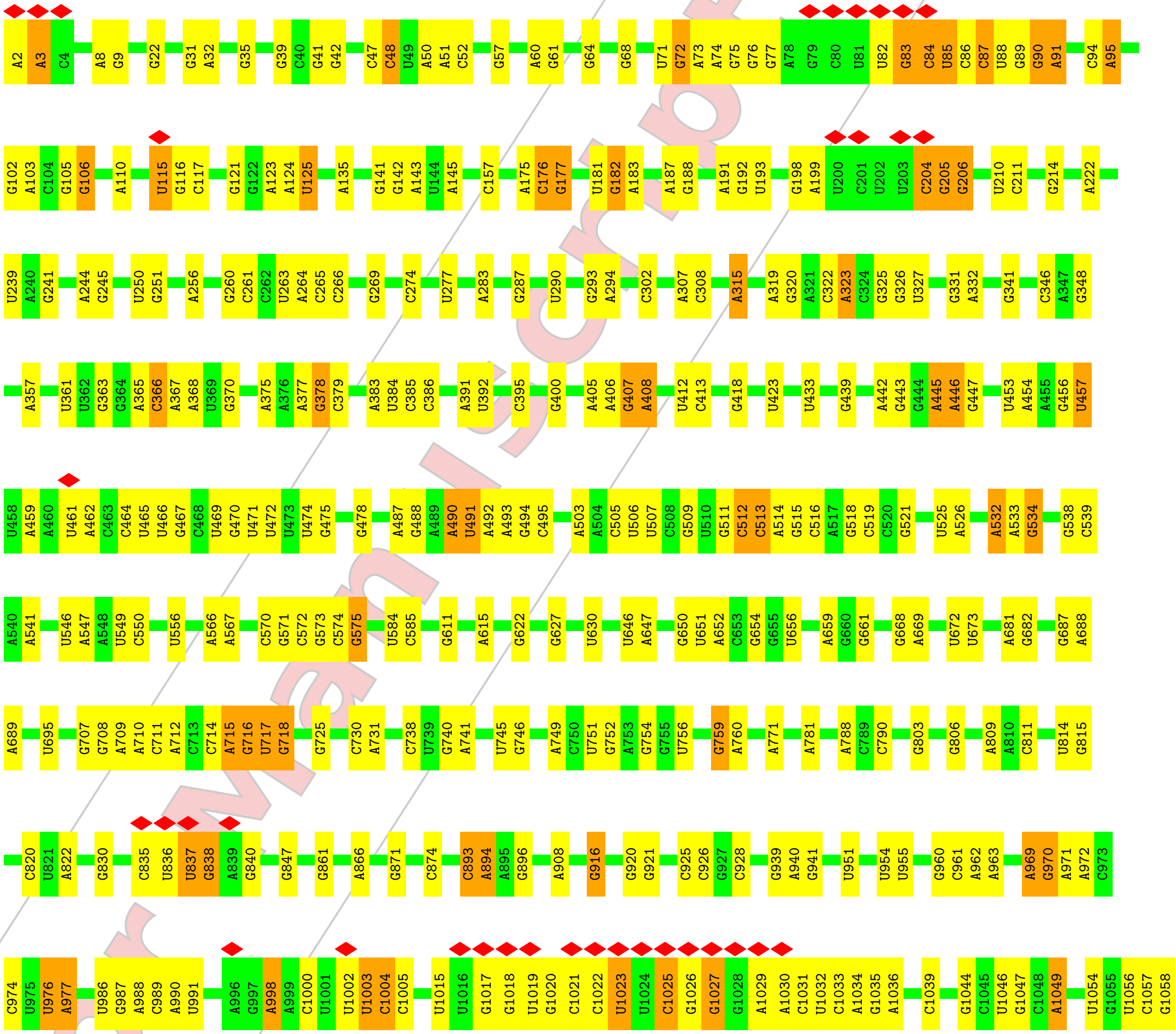

For Manuscript Review

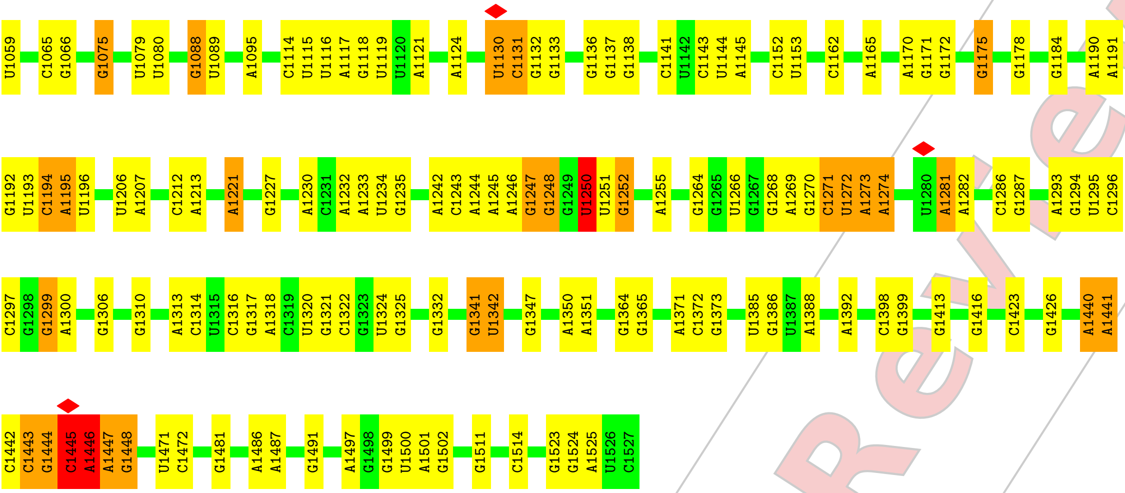

- Molecule 2: Large ribosomal subunit protein uL22

Chain 0:
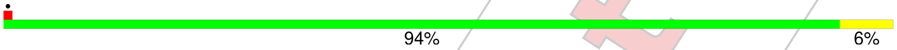

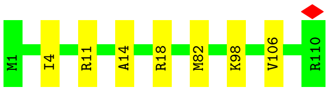

- Molecule 3: Small ribosomal subunit protein uS19

Chain 1:
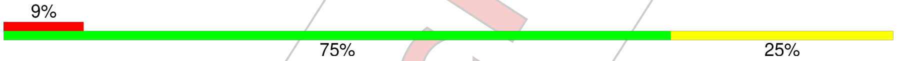

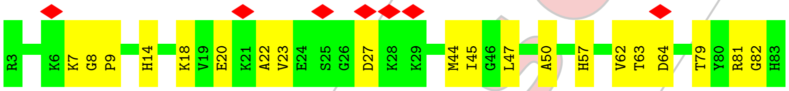

- Molecule 4: Large ribosomal subunit protein bL21

Chain Z:
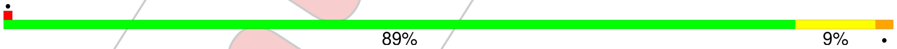

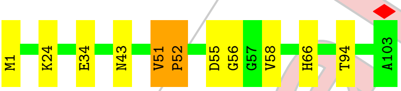

- Molecule 5: Small ribosomal subunit protein bS20

Chain 2:
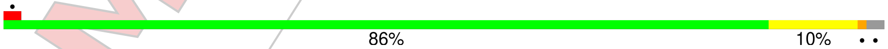

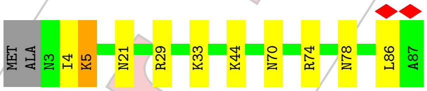

- Molecule 6: Small ribosomal subunit protein bS21

Chain 3:
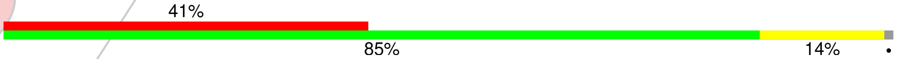

For Manuscript Review

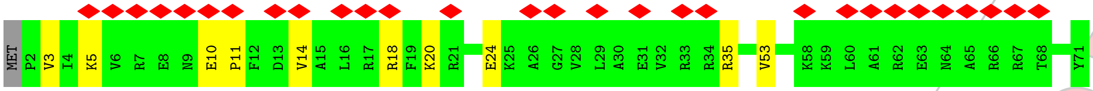

• Molecule 7: 50S ribosomal protein L23

Chain b:

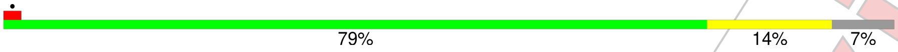

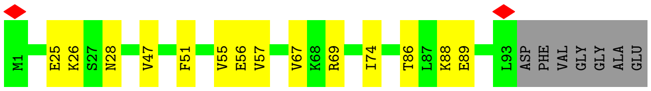

• Molecule 8: 50S ribosomal protein L24

Chain c:

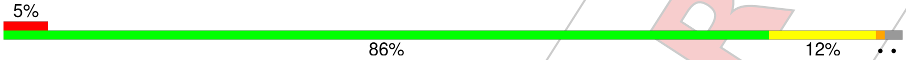

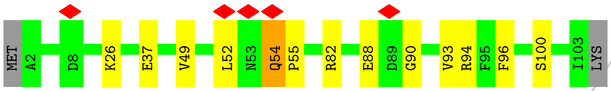

• Molecule 9: Small ribosomal subunit protein uS2

Chain D:

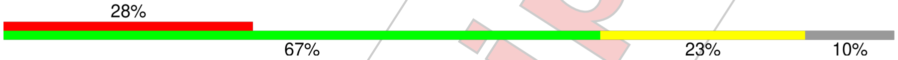

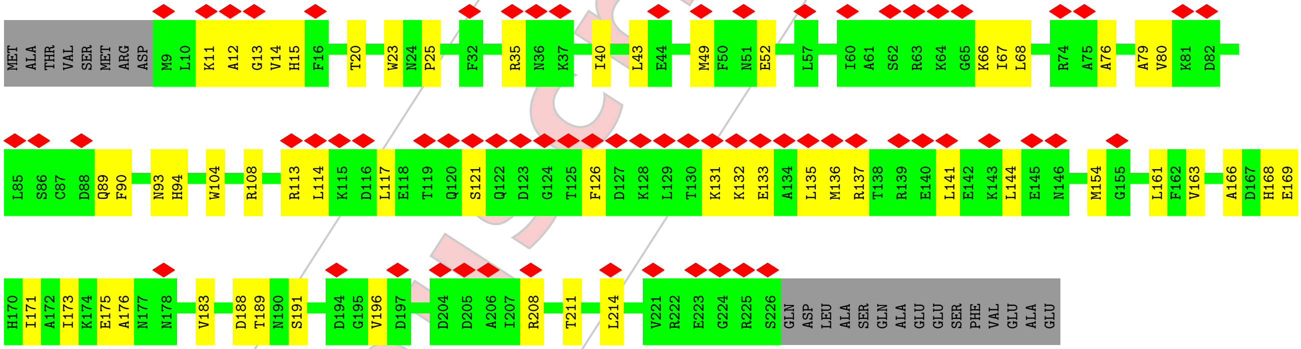

• Molecule 10: Large ribosomal subunit protein bL25

Chain d:

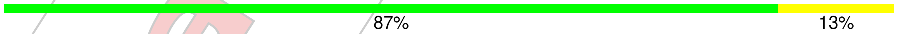

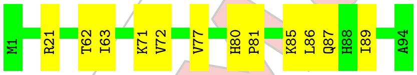

• Molecule 11: Small ribosomal subunit protein uS3

Chain E:

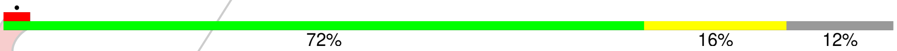

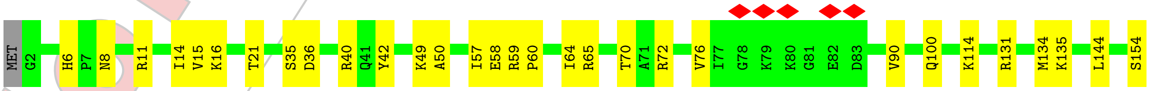

For Manuscript Review

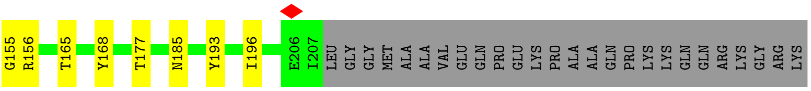

• Molecule 12: 50S ribosomal protein L27

Chain e:

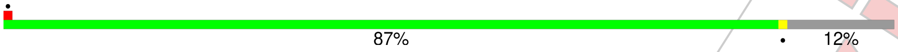

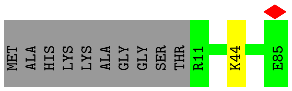

• Molecule 13: Small ribosomal subunit protein uS4

Chain F:

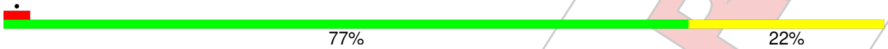

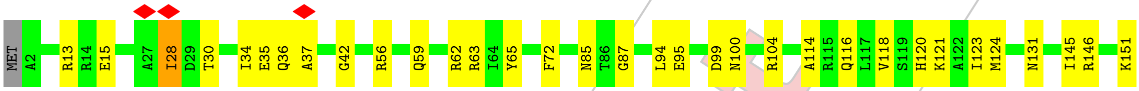

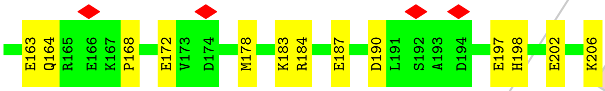

• Molecule 14: 50S ribosomal protein L28

Chain f:

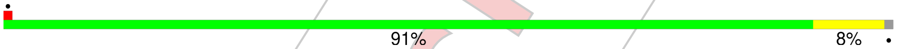

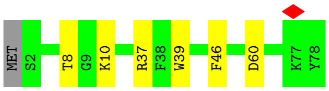

• Molecule 15: Small ribosomal subunit protein uS5

Chain G:

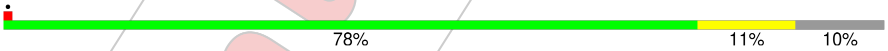

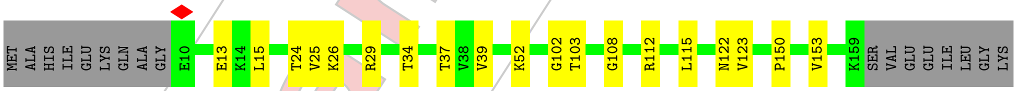

• Molecule 16: Large ribosomal subunit protein uL29

Chain g:

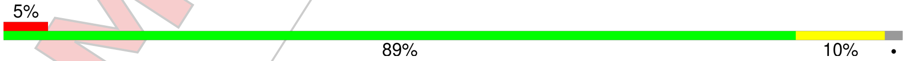

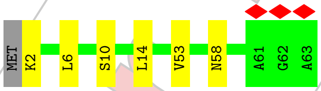

• Molecule 17: Small ribosomal subunit protein bS6

Chain H:

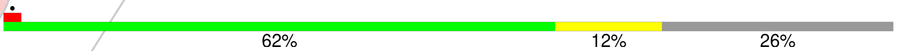

For Manuscript Review

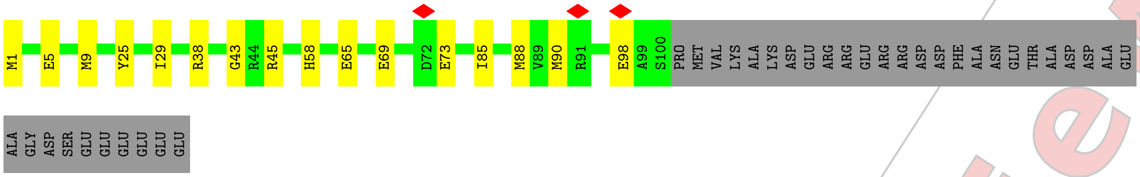

• Molecule 18: 50S ribosomal protein L30

Chain h:

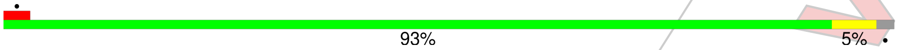

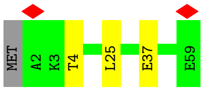

• Molecule 19: 23S ribosomal RNA

Chain I:

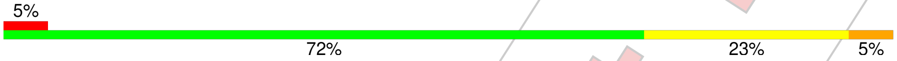

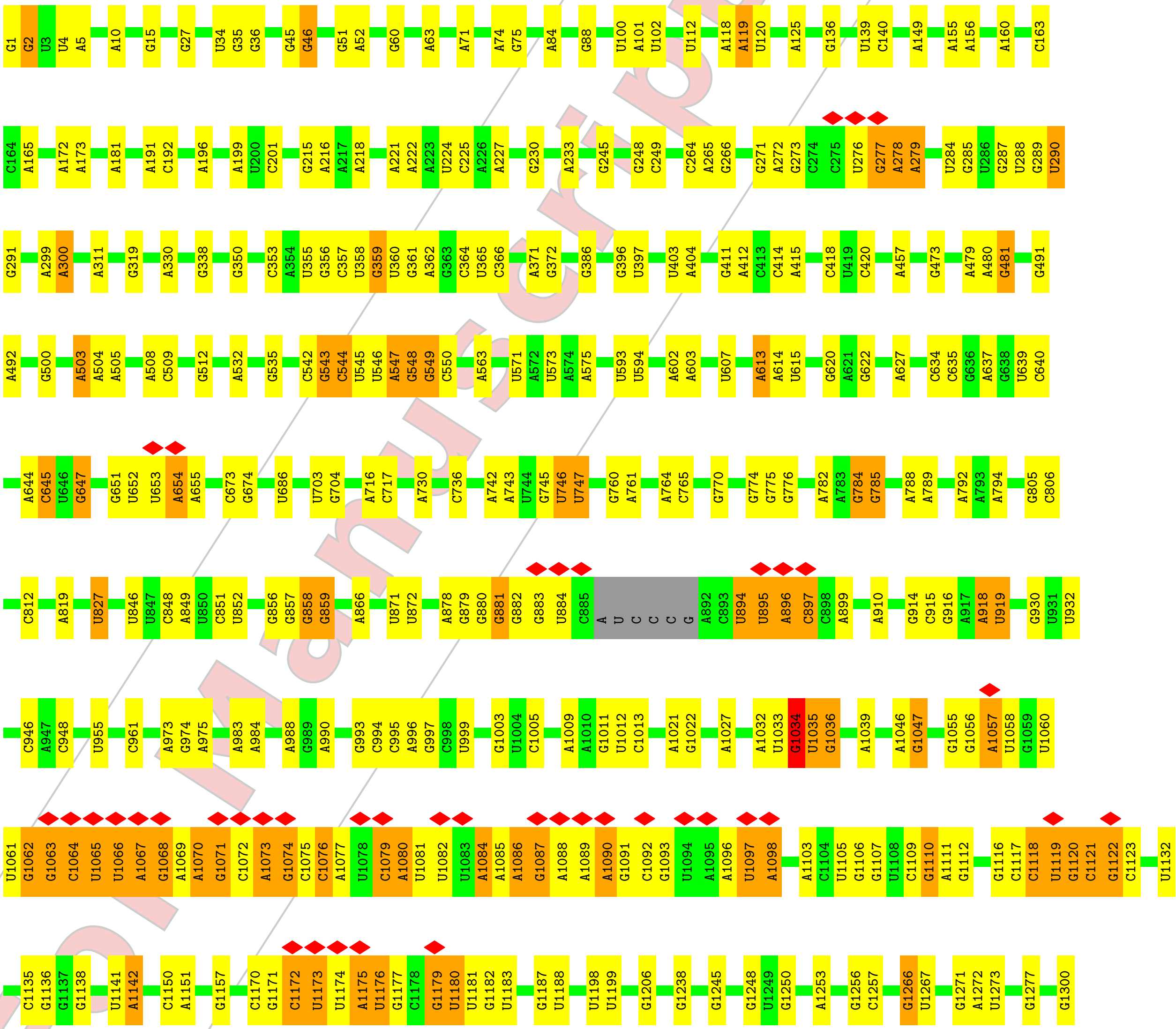

For Manuscript Review

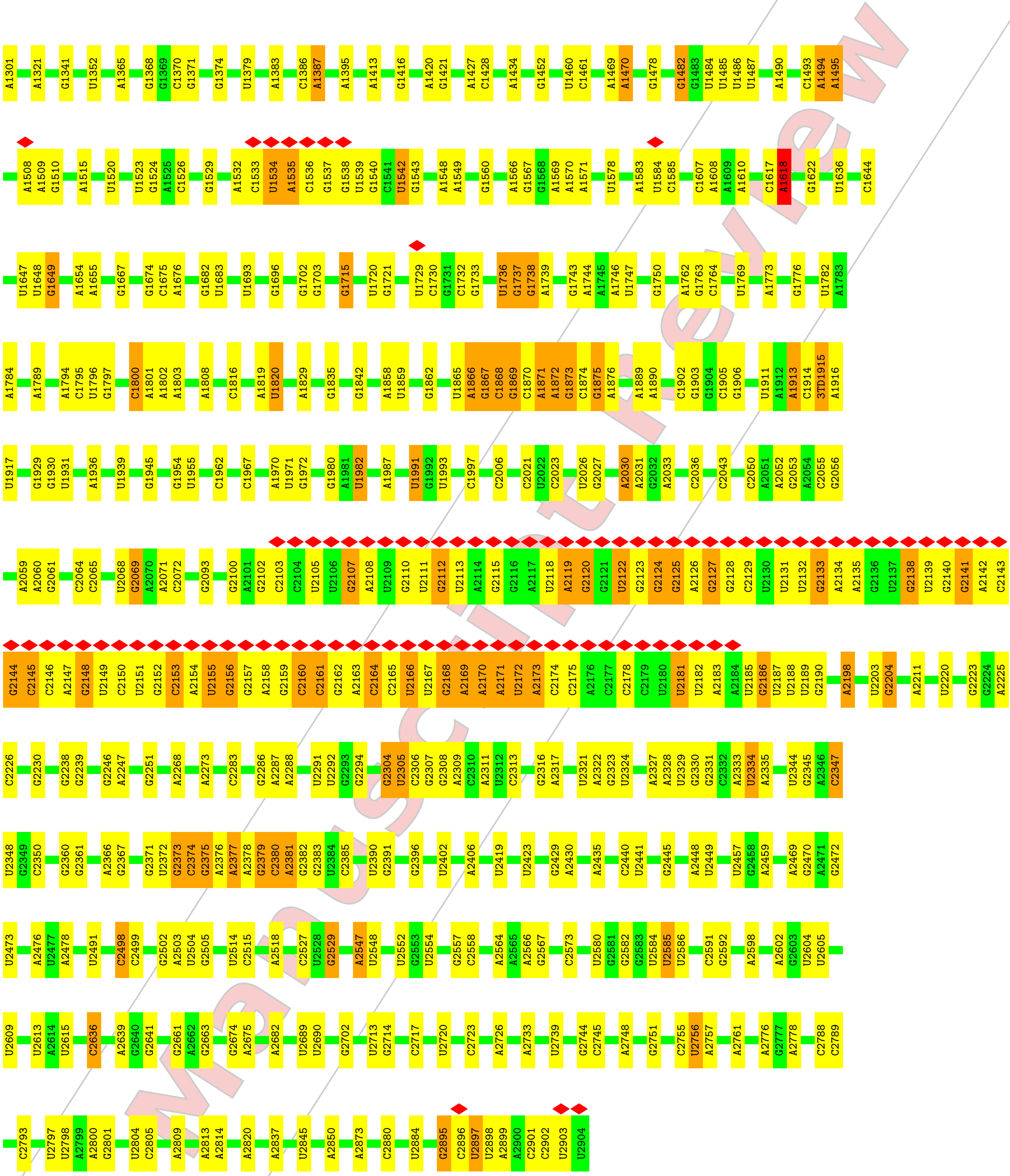

• Molecule 20: 50S ribosomal protein L32

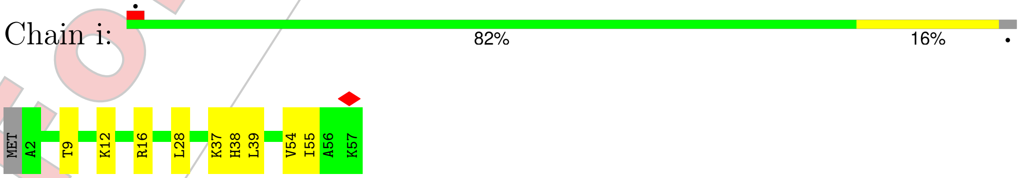

For Manuscript Review

- Molecule 21: 5S ribosomal RNA

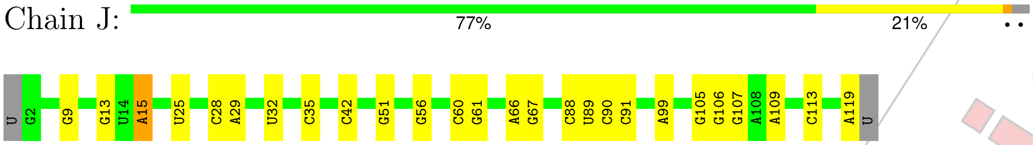

- Molecule 22: 50S ribosomal protein L33

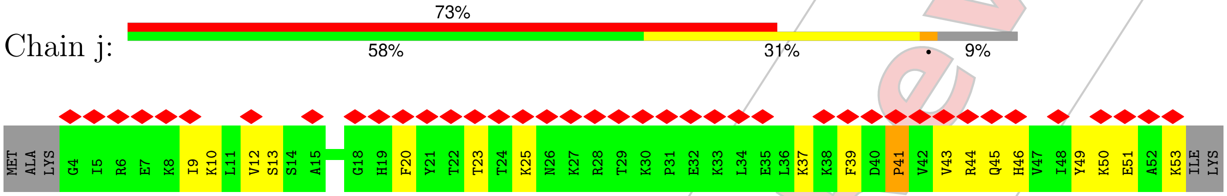

- Molecule 23: 50S ribosomal protein L2

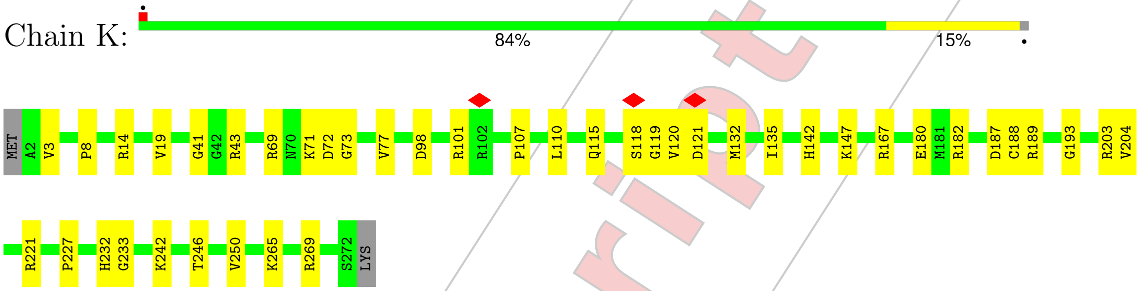

- Molecule 24: Large ribosomal subunit protein bL34

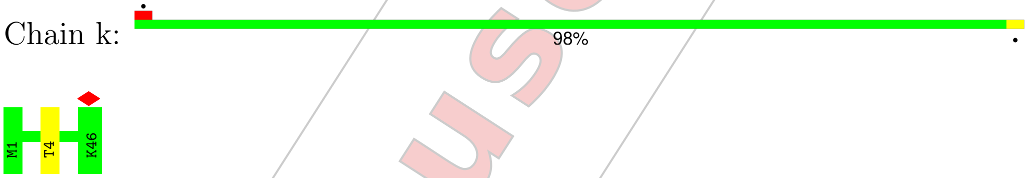

- Molecule 25: Large ribosomal subunit protein uL3

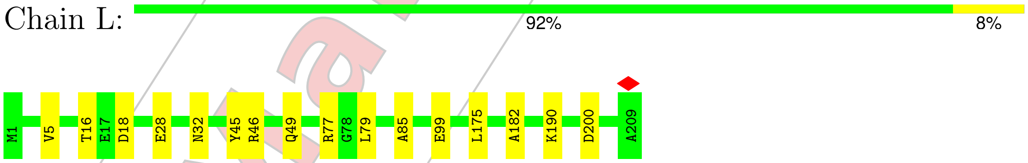

- Molecule 26: 50S ribosomal protein L35

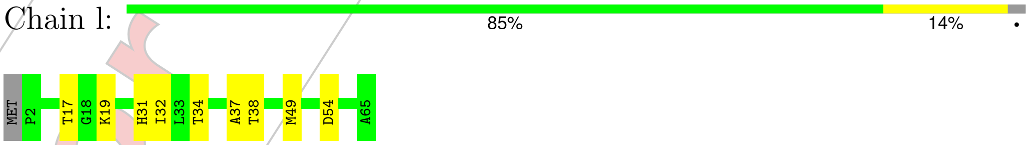

- Molecule 27: Large ribosomal subunit protein uL4

For Manuscript Review

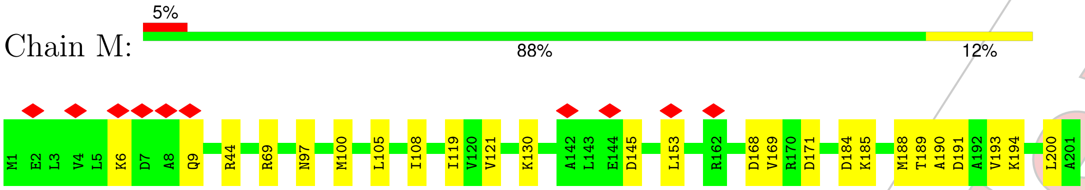

• Molecule 28: Large ribosomal subunit protein bL36A

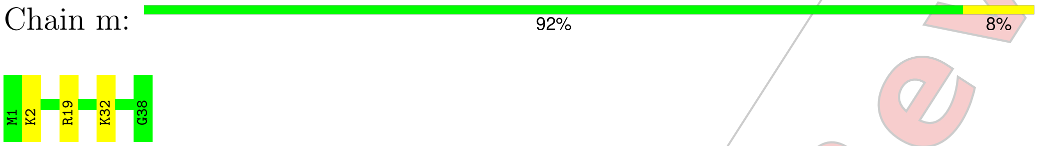

• Molecule 29: Large ribosomal subunit protein uL5

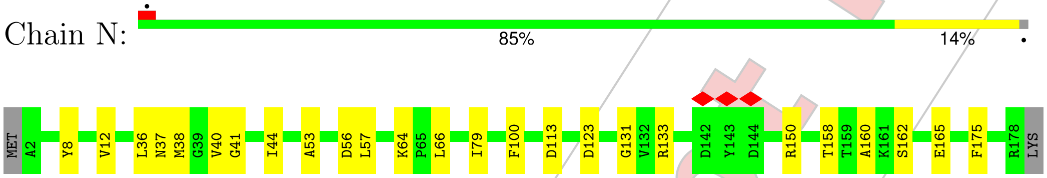

• Molecule 30: Small ribosomal subunit protein uS7

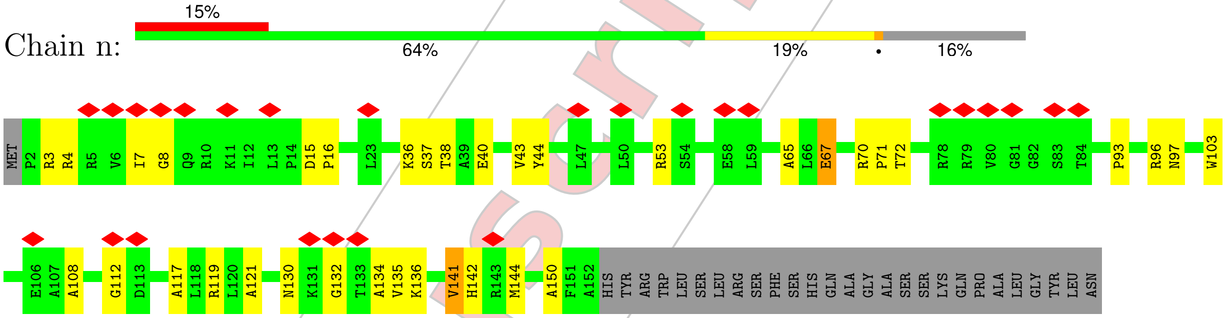

• Molecule 31: Large ribosomal subunit protein uL6

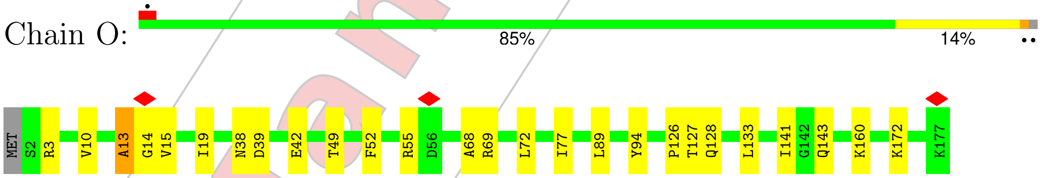

• Molecule 32: Small ribosomal subunit protein uS8

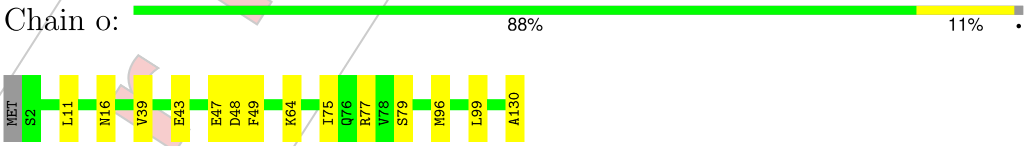

• Molecule 33: Large ribosomal subunit protein bL9

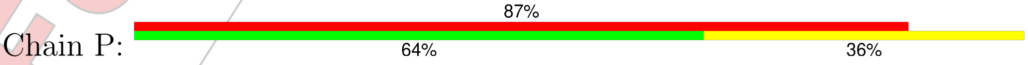

For Manuscript Review

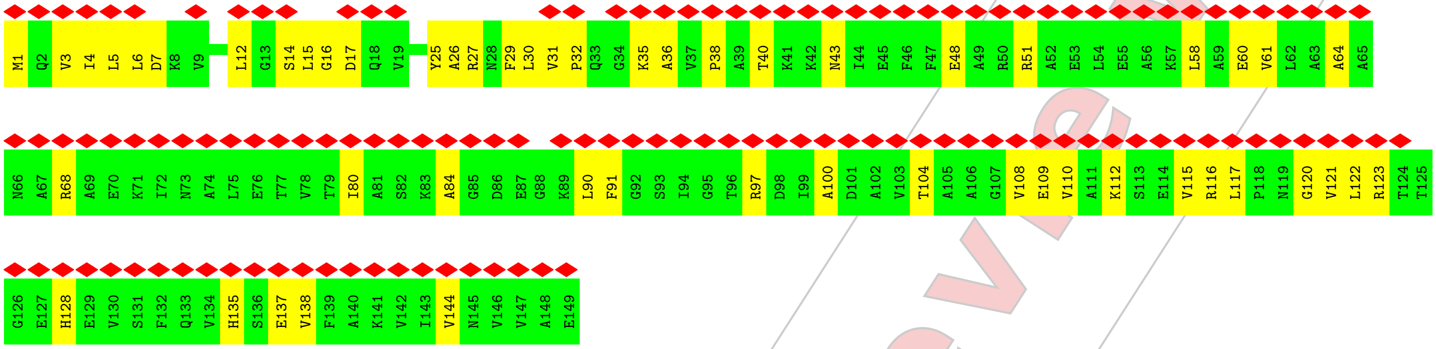

• Molecule 34: Small ribosomal subunit protein uS9

Chain p:

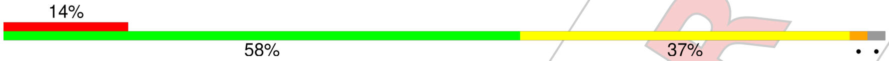

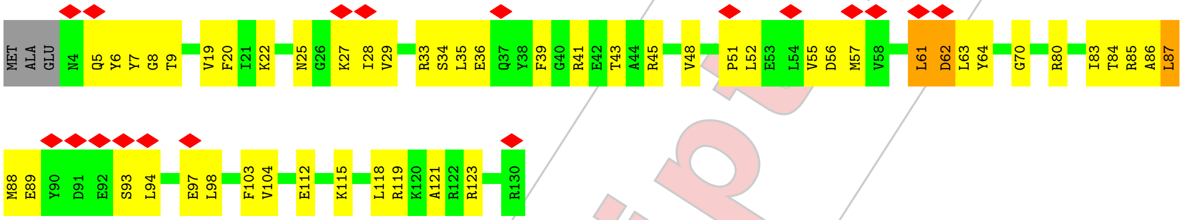

• Molecule 35: 50S ribosomal protein L31

Chain Q:

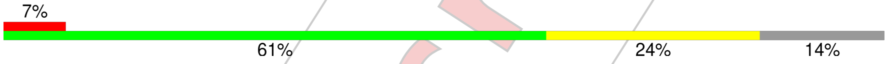

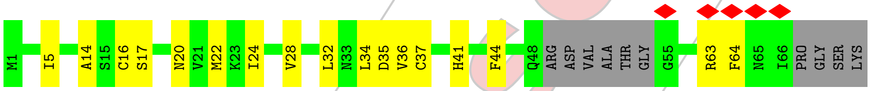

• Molecule 36: Small ribosomal subunit protein uS10

Chain q:

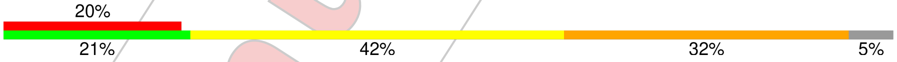

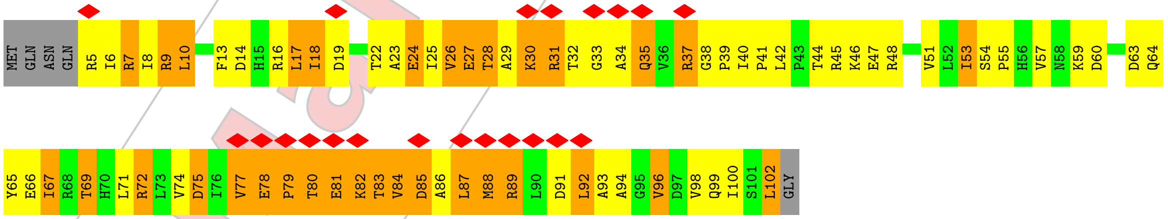

• Molecule 37: Large ribosomal subunit protein uL13

Chain R:

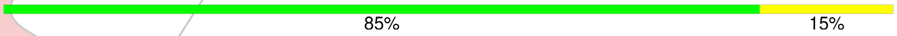

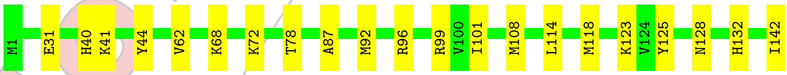

• Molecule 38: 30S ribosomal protein S11

For Manuscript Review

Chain r:

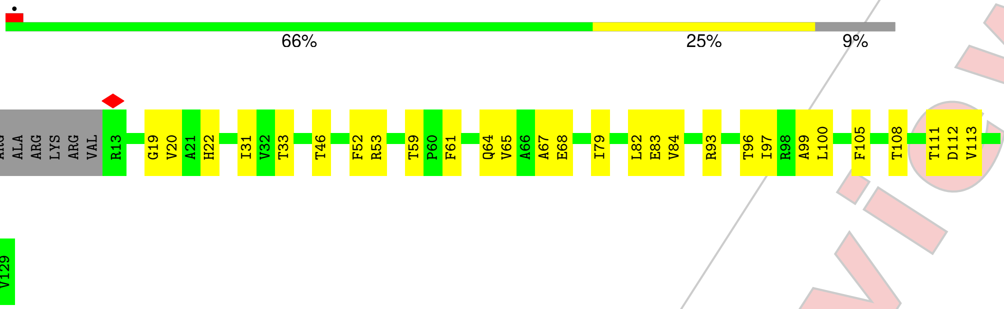

• Molecule 39: Large ribosomal subunit protein uL14

Chain S:

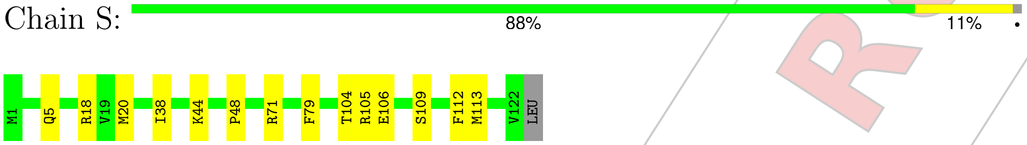

• Molecule 40: 30S ribosomal protein S12

Chain t:

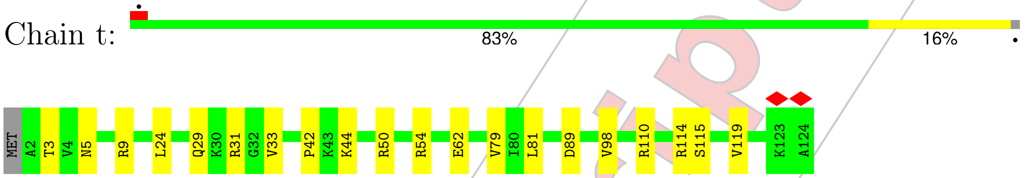

• Molecule 41: Large ribosomal subunit protein uL15

Chain T:

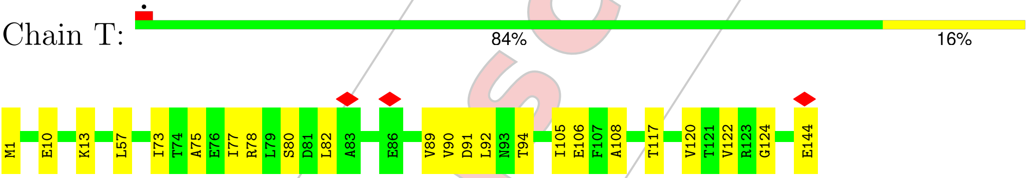

• Molecule 42: Small ribosomal subunit protein uS13

Chain u:

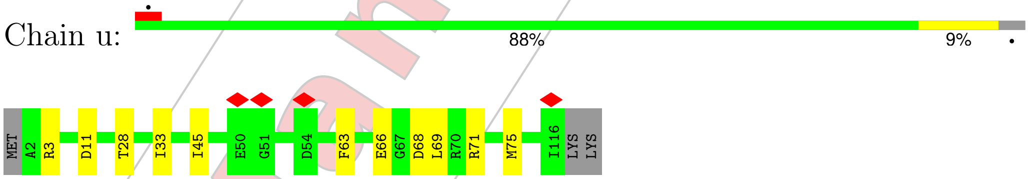

• Molecule 43: Large ribosomal subunit protein uL16

Chain U:

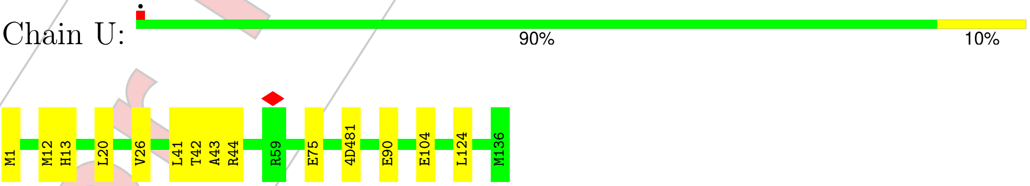

• Molecule 44: Small ribosomal subunit protein uS14

Chain v:

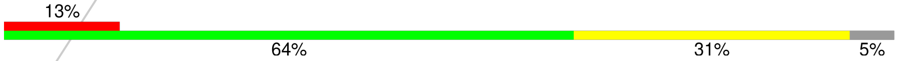

For Manuscript Review

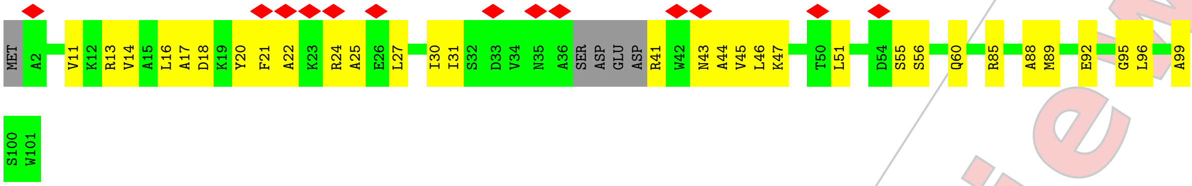

• Molecule 45: Large ribosomal subunit protein bL17

Chain V:
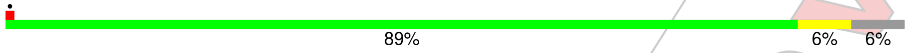

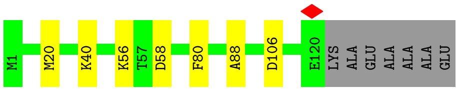

• Molecule 46: Small ribosomal subunit protein uS15

Chain w:
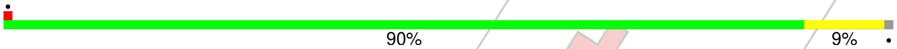

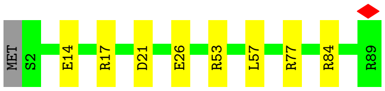

• Molecule 47: Large ribosomal subunit protein uL18

Chain W:
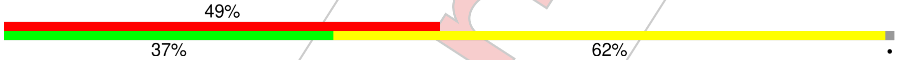

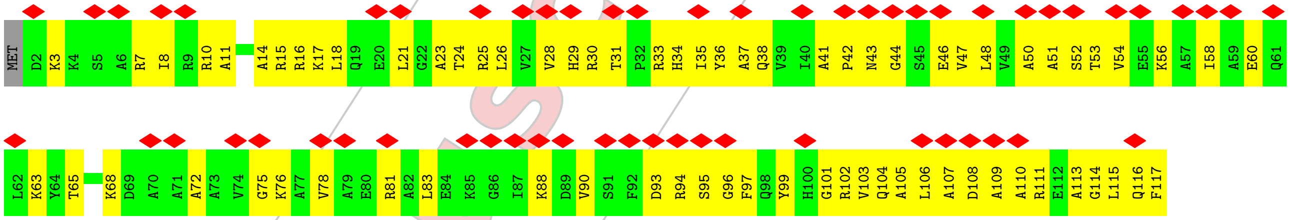

• Molecule 48: Small ribosomal subunit protein bS16

Chain x:
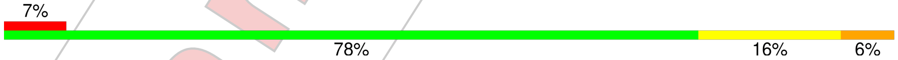

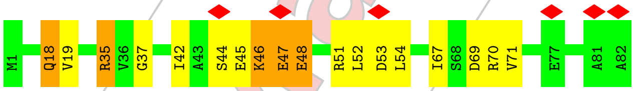

• Molecule 49: Large ribosomal subunit protein bL19

Chain X:
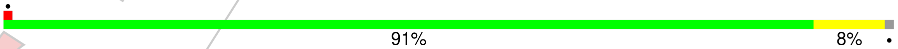

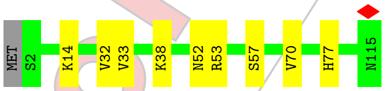

• Molecule 50: Small ribosomal subunit protein uS17

For Manuscript Review

Chain y:

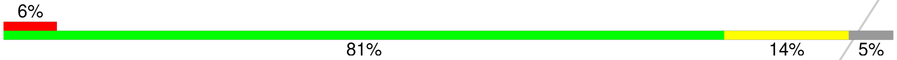

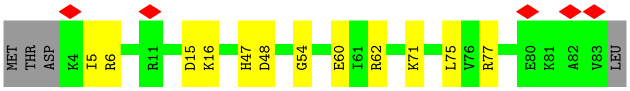

• Molecule 51: 50S ribosomal protein L20

Chain Y:

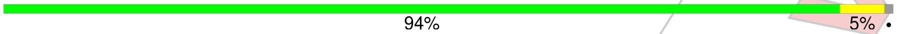

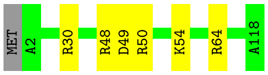

• Molecule 52: Small ribosomal subunit protein bS18

Chain z:

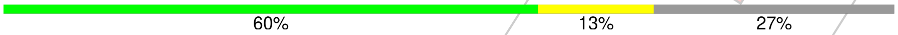

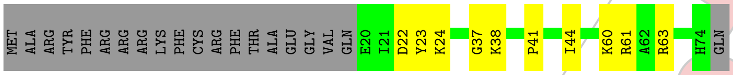

For Manuscript Review

4 Experimental information (i)

Property	Value	Source
EM reconstruction method	SINGLE PARTICLE	Depositor
Imposed symmetry	POINT, Not provided	
Number of particles used	312639	Depositor
Resolution determination method	FSC 0.143 CUT-OFF	Depositor
CTF correction method	PHASE FLIPPING AND AMPLITUDE CORRECTION	Depositor
Microscope	TFS GLACIOS	Depositor
Voltage (kV)	200	Depositor
Electron dose $(e^-/\text{Å}^2)$	60	Depositor
Minimum defocus (nm)	500	Depositor
Maximum defocus (nm)	2500	Depositor
Magnification	Not provided	
Image detector	GATAN K3 (6k x 4k)	Depositor
Maximum map value	35.939	Depositor
Minimum map value	-18.973	Depositor
Average map value	-0.000	Depositor
Map value standard deviation	1.000	Depositor
Recommended contour level	2.5	Depositor
Map size (Å)	455.52, 455.52, 455.52	wwPDB
Map dimensions	480, 480, 480	wwPDB
Map angles (°)	90.0, 90.0, 90.0	wwPDB
Pixel spacing (Å)	0.949, 0.949, 0.949	Depositor

For Manuscript Review

5 Model quality (i)

5.1 Standard geometry (i)

Bond lengths and bond angles in the following residue types are not validated in this section: 2MG, G7M, 5MC, 6MZ, 2MA, H2U, 1MG, OMG, 5MU, OMU, 3TD, MG, OMC, PSU, 4D4

The Z score for a bond length (or angle) is the number of standard deviations the observed value is removed from the expected value. A bond length (or angle) with $|Z| > 5$ is considered an outlier worth inspection. RMSZ is the root-mean-square of all Z scores of the bond lengths (or angles).

Mol	Chain	Bond lengths		Bond angles	
		RMSZ	$\#\|Z\| > 5$	RMSZ	$\#\|Z\| > 5$
1	C	0.29	0/36666	0.39	12/57201 (0.0%)
2	0	0.31	0/864	0.40	0/1156
3	1	0.30	0/667	0.53	0/897
4	Z	0.30	0/829	0.53	0/1107
5	2	0.30	0/671	0.70	1/888 (0.1%)
6	3	0.19	0/598	0.46	0/792
7	b	0.26	0/744	0.51	0/994
8	c	0.27	0/787	0.57	0/1051
9	D	0.21	0/1735	0.44	0/2338
10	d	0.30	0/766	0.49	0/1025
11	E	0.22	0/1651	0.45	0/2225
12	e	0.32	0/582	0.51	0/769
13	F	0.22	0/1665	0.45	0/2227
14	f	0.34	0/635	0.49	0/848
15	G	0.29	0/1118	0.47	0/1504
16	g	0.23	0/502	0.43	0/667
17	H	0.26	0/835	0.55	0/1128
18	h	0.29	0/453	0.48	0/605
19	I	0.36	0/69168	0.38	21/107901 (0.0%)
20	i	0.31	0/450	0.41	0/599
21	J	0.26	0/2828	0.28	0/4410
22	j	0.26	0/416	0.65	0/554
23	K	0.39	0/2132	0.60	0/2866
24	k	0.35	0/380	0.45	0/498
25	L	0.33	0/1586	0.46	0/2134
26	l	0.34	0/513	0.50	0/676
27	M	0.29	0/1571	0.47	0/2113
28	m	0.33	0/303	0.52	0/397
29	N	0.20	0/1434	0.42	0/1926
30	n	0.25	0/1195	0.89	6/1602 (0.4%)
31	O	0.27	0/1343	0.60	1/1816 (0.1%)
32	o	0.24	0/989	0.42	0/1326

For Manuscript Review

Mol	Chain	Bond lengths		Bond angles	
		RMSZ	#|Z| >5	RMSZ	#|Z| >5
33	P	0.19	0/1122	0.46	0/1515
34	p	0.26	0/1034	0.81	1/1375 (0.1%)
35	Q	0.18	0/488	0.37	0/649
36	q	0.91	0/796	1.24	1/1077 (0.1%)
37	R	0.32	0/1152	0.43	0/1551
38	r	0.23	0/893	0.42	0/1205
39	S	0.36	0/947	0.53	0/1268
40	t	0.31	0/969	0.58	0/1300
41	T	0.31	0/1062	0.50	0/1413
42	u	0.19	0/900	0.40	1/1204 (0.1%)
43	U	0.32	0/1081	0.52	2/1443 (0.1%)
44	v	0.22	0/785	0.45	0/1043
45	V	0.33	0/973	0.44	0/1301
46	w	0.27	0/718	0.42	0/959
47	W	0.30	0/902	0.64	0/1209
48	x	0.37	0/659	0.66	0/884
49	X	0.31	0/929	0.44	0/1242
50	y	0.26	0/657	0.51	0/881
51	Y	0.34	0/960	0.37	0/1278
52	z	0.24	0/462	0.41	0/621
All	All	0.33	0/153565	0.43	46/229658 (0.0%)

Chiral center outliers are detected by calculating the chiral volume of a chiral center and verifying if the center is modelled as a planar moiety or with the opposite hand.A planarity outlier is detected by checking planarity of atoms in a peptide group, atoms in a mainchain group or atoms of a sidechain that are expected to be planar.

Mol	Chain	#Chirality outliers	#Planarity outliers
4	Z	0	1
23	K	0	2
34	p	0	1
40	t	0	1
43	U	0	1
All	All	0	6

There are no bond length outliers.

All (46) bond angle outliers are listed below:

Mol	Chain	Res	Type	Atoms	Z	Observed(°)	Ideal(°)
19	I	2717	C	OP1-P-O3'	-9.79	78.63	108.00
19	I	673	C	OP1-P-O3'	-9.20	80.42	108.00
19	I	2598	A	OP1-P-O3'	-8.92	81.24	108.00

Continued on next page...

For Manuscript Review

Continued from previous page...

Mol	Chain	Res	Type	Atoms	Z	Observed(°)	Ideal(°)
19	I	2360	G	OP1-P-O3'	-8.88	81.35	108.00
19	I	2723	C	OP1-P-O3'	-8.88	81.37	108.00
19	I	1655	A	OP2-P-O3'	-8.85	81.46	108.00
19	I	2230	G	OP1-P-O3'	-8.84	81.49	108.00
30	n	108	ALA	N-CA-C	-8.77	104.62	114.62
19	I	2050	C	OP1-P-O3'	-8.69	81.93	108.00
19	I	2674	G	OP1-P-O3'	-8.66	82.02	108.00
19	I	2598	A	OP2-P-O3'	-8.62	82.13	108.00
1	C	325	G	P-O3'-C3'	-8.52	107.42	120.20
19	I	2564	A	OP1-P-O3'	-8.49	82.54	108.00
19	I	2360	G	OP2-P-O3'	-8.45	82.66	108.00
19	I	2564	A	OP2-P-O3'	-8.33	83.02	108.00
19	I	673	C	OP2-P-O3'	-8.31	83.06	108.00
19	I	1655	A	OP1-P-O3'	-8.29	83.12	108.00
19	I	2050	C	OP2-P-O3'	-8.23	83.30	108.00
19	I	2723	C	OP2-P-O3'	-8.20	83.41	108.00
19	I	2674	G	OP2-P-O3'	-8.12	83.63	108.00
19	I	2230	G	OP2-P-O3'	-8.02	83.94	108.00
1	C	307	A	P-O3'-C3'	-7.73	108.61	120.20
19	I	2717	C	OP2-P-O3'	-7.65	85.04	108.00
1	C	1445	C	P-O3'-C3'	-7.42	109.06	120.20
30	n	38	THR	N-CA-C	-7.41	106.17	114.62
30	n	3	ARG	CA-C-N	7.19	133.72	121.14
30	n	3	ARG	C-N-CA	7.19	133.72	121.14
1	C	1250	U	P-O3'-C3'	-6.80	110.00	120.20
1	C	106	G	P-O3'-C3'	-6.66	110.21	120.20
1	C	105	G	P-O3'-C3'	-6.62	110.27	120.20
1	C	1221	A	P-O3'-C3'	-6.09	111.07	120.20
36	q	39	PRO	N-CA-C	-5.83	100.47	112.47
1	C	308	C	P-O3'-C3'	-5.79	111.52	120.20
1	C	1446	A	P-O3'-C3'	-5.78	111.53	120.20
1	C	1443	C	P-O3'-C3'	-5.65	111.72	120.20
19	I	1034	G	C2'-C3'-O3'	5.47	117.71	109.50
42	u	66	GLU	CB-CA-C	-5.44	109.84	117.23
30	n	141	VAL	N-CA-C	-5.27	106.40	112.98
31	O	77	ILE	N-CA-C	-5.25	107.28	113.42
1	C	1445	C	O4'-C1'-C2'	-5.25	102.35	107.60
1	C	525	U	P-O3'-C3'	-5.17	112.45	120.20
5	2	21	ASN	N-CA-C	-5.15	108.26	114.75
34	p	88	MET	N-CA-C	-5.15	108.02	114.56
43	U	12	MET	CA-C-N	-5.12	113.89	123.05
43	U	12	MET	C-N-CA	-5.12	113.89	123.05

Continued on next page...

For Manuscript Review

Continued from previous page...

Mol	Chain	Res	Type	Atoms	Z	Observed(^{o})	Ideal(^{o})
30	n	67	GLU	N-CA-C	-5.09	106.20	114.09

There are no chirality outliers.

All (6) planarity outliers are listed below:

Mol	Chain	Res	Type	Group
23	K	101	ARG	Peptide
23	K	232	HIS	Peptide
43	U	13	HIS	Peptide
4	Z	51	VAL	Peptide
34	p	61	LEU	Peptide
40	t	44	LYS	Peptide

5.2 Too-close contacts (i)

In the following table, the Non-H and H(model) columns list the number of non-hydrogen atoms and hydrogen atoms in the chain respectively. The H(added) column lists the number of hydrogen atoms added and optimized by MolProbity. The Clashes column lists the number of clashes within the asymmetric unit, whereas Symm-Clashes lists symmetry-related clashes.

Mol	Chain	Non-H	H(model)	H(added)	Clashes	Symm-Clashes
1	C	32744	0	16472	247	0
2	0	857	0	922	4	0
3	1	651	0	675	13	0
4	Z	816	0	839	7	0
5	2	665	0	714	6	0
6	3	590	0	629	9	0
7	b	738	0	807	9	0
8	c	779	0	831	9	0
9	D	1704	0	1732	40	0
10	d	753	0	780	7	0
11	E	1624	0	1696	28	0
12	e	575	0	592	3	0
13	F	1643	0	1707	27	0
14	f	625	0	652	7	0
15	G	1105	0	1148	12	0
16	g	501	0	531	4	0
17	H	817	0	808	12	0
18	h	449	0	488	2	0
19	I	62271	0	31340	375	0
20	i	444	0	458	8	0

Continued on next page...

For Manuscript Review

Continued from previous page...

Mol	Chain	Non-H	H(model)	H(added)	Clashes	Symm-Clashes
21	J	2529	0	1281	12	0
22	j	409	0	440	24	0
23	K	2093	0	2166	28	0
24	k	377	0	418	1	0
25	L	1565	0	1616	10	0
26	l	504	0	572	10	0
27	M	1552	0	1619	16	0
28	m	302	0	343	3	0
29	N	1410	0	1444	20	0
30	n	1181	0	1238	27	0
31	O	1323	0	1371	17	0
32	o	979	0	1031	8	0
33	P	1111	0	1148	53	0
34	p	1022	0	1070	48	0
35	Q	480	0	482	13	0
36	q	786	0	828	95	0
37	R	1129	0	1162	14	0
38	r	877	0	887	24	0
39	S	938	0	1012	13	0
40	t	955	0	1016	12	0
41	T	1053	0	1129	16	0
42	u	891	0	952	8	0
43	U	1075	0	1154	7	0
44	v	774	0	824	28	0
45	V	960	0	1000	5	0
46	w	710	0	728	4	0
47	W	892	0	923	87	0
48	x	649	0	666	15	0
49	X	917	0	962	7	0
50	y	648	0	691	8	0
51	Y	947	0	1019	6	0
52	z	455	0	478	8	0
53	C	89	0	0	2	0
53	I	203	0	0	2	0
53	J	5	0	0	0	0
53	K	1	0	0	0	0
53	L	2	0	0	0	0
53	M	1	0	0	0	0
53	T	1	0	0	0	0
53	V	1	0	0	0	0
53	Y	1	0	0	1	0
53	l	1	0	0	0	0

Continued on next page...

For Manuscript Review

Continued from previous page...

Mol	Chain	Non-H	H(model)	H(added)	Clashes	Symm-Clashes
53	p	1	0	0	0	0
53	u	1	0	0	0	0
All	All	142151	0	95491	1341	0

The all-atom clashscore is defined as the number of clashes found per 1000 atoms (including hydrogen atoms). The all-atom clashscore for this structure is 6.

All (1341) close contacts within the same asymmetric unit are listed below, sorted by their clash magnitude.

Atom-1	Atom-2	Interatomic distance (Å)	Clash overlap (Å)
36:q:7:ARG:HH11	36:q:7:ARG:CB	1.36	1.37
19:I:1636:U:OP2	53:I:3077:MG:MG	0.78	1.21
36:q:7:ARG:HH11	36:q:7:ARG:HB3	0.97	1.10
36:q:7:ARG:HB3	36:q:7:ARG:NH1	1.68	1.08
1:C:1273:A:H4'	1:C:1274:A:OP1	1.47	1.07
36:q:24:GLU:N	36:q:24:GLU:OE2	1.89	1.05
36:q:7:ARG:CB	36:q:7:ARG:NH1	2.22	1.00
19:I:1047:G:HO2'	19:I:1110:G:H1	1.07	1.00
36:q:85:ASP:OD2	36:q:89:ARG:HD3	1.62	0.99
48:x:44:SER:C	48:x:46:LYS:H	1.70	0.98
36:q:17:LEU:HD11	36:q:93:ALA:HB3	1.45	0.98
19:I:545:U:O2	19:I:549:G:N1	1.96	0.97
36:q:78:GLU:OE1	36:q:78:GLU:C	2.08	0.96
19:I:1866:A:H3'	19:I:1867:G:H4'	1.49	0.92
4:Z:51:VAL:HG23	4:Z:52:PRO:HD2	1.50	0.92
36:q:27:GLU:O	36:q:29:ALA:N	2.03	0.91
47:W:50:ALA:O	47:W:81:ARG:NH1	2.04	0.90
36:q:27:GLU:C	36:q:29:ALA:H	1.81	0.89
1:C:916:G:H21	1:C:1392:A:H8	1.17	0.89
1:C:1088:G:O2'	53:C:1625:MG:MG	1.12	0.89
1:C:1088:G:HO2'	53:C:1625:MG:MG	0.78	0.88
46:w:26:GLU:OE2	46:w:77:ARG:NH1	2.07	0.88
19:I:1032:A:C2	19:I:1034:G:H8	1.93	0.86
36:q:29:ALA:O	36:q:32:THR:N	2.08	0.86
11:E:60:PRO:HB3	36:q:94:ALA:HB1	1.55	0.86
9:D:132:LYS:HG3	9:D:136:MET:HE2	1.58	0.86
19:I:2744:G:O6	19:I:2761:A:N1	2.01	0.85
19:I:1032:A:H2	19:I:1034:G:H8	1.25	0.84
30:n:72:THR:HA	30:n:96:ARG:HH12	1.42	0.83
36:q:17:LEU:CD1	36:q:93:ALA:HB3	2.08	0.83
34:p:55:VAL:HG23	34:p:94:LEU:HD22	1.59	0.83

Continued on next page...

For Manuscript Review

Continued from previous page...

Atom-1	Atom-2	Interatomic distance (Å)	Clash overlap (Å)
19:I:545:U:O2	19:I:549:G:N2	2.12	0.82
33:P:30:LEU:HA	33:P:35:LYS:HB2	1.61	0.82
9:D:68:LEU:HD12	9:D:154:MET:HE1	1.61	0.82
34:p:51:PRO:HG3	34:p:80:ARG:HG3	1.60	0.82
1:C:1054:U:H5'	36:q:53:ILE:HG22	1.61	0.82
13:F:13:ARG:NH2	13:F:37:ALA:O	2.12	0.82
48:x:44:SER:O	48:x:46:LYS:N	2.13	0.82
19:I:1529:G:H1	19:I:1542:U:H3	1.25	0.81
19:I:545:U:O2	19:I:549:G:C2	2.33	0.81
19:I:2305:U:O2'	29:N:133:ARG:NH1	2.14	0.80
36:q:85:ASP:OD2	36:q:89:ARG:CD	2.28	0.80
19:I:1873:G:C2	19:I:1874:C:H1'	2.16	0.80
47:W:38:GLN:HG2	47:W:51:ALA:H	1.45	0.80
47:W:41:ALA:HB2	47:W:48:LEU:HD21	1.63	0.80
48:x:44:SER:C	48:x:46:LYS:N	2.36	0.80
35:Q:63:ARG:HG3	35:Q:64:PHE:HB2	1.63	0.80
36:q:53:ILE:HD12	44:v:85:ARG:HD2	1.64	0.79
1:C:1444:G:H21	1:C:1447:A:H2	1.31	0.78
19:I:2347:C:N3	19:I:2371:G:N2	2.32	0.77
8:c:26:LYS:HE3	8:c:37:GLU:HG2	1.67	0.77
36:q:78:GLU:OE1	36:q:78:GLU:O	2.02	0.77
15:G:153:VAL:HG11	32:o:99:LEU:HD12	1.67	0.76
26:l:17:THR:HG21	26:l:49:MET:HE1	1.66	0.76
30:n:71:PRO:O	30:n:96:ARG:NH1	2.18	0.76
36:q:17:LEU:CD1	36:q:93:ALA:CB	2.63	0.76
19:I:2151:U:H2'	19:I:2152:G:H8	1.50	0.76
1:C:115:U:O2	1:C:117:C:N4	2.18	0.75
19:I:2162:G:H4'	19:I:2171:A:H2'	1.68	0.75
22:j:44:ARG:O	26:l:38:THR:OG1	2.04	0.75
15:G:103:THR:O	15:G:122:ASN:ND2	2.15	0.75
19:I:1172:C:O2	19:I:1177:G:N2	2.19	0.75
19:I:1913:A:H62	19:I:1915:3TD:H3'	1.49	0.75
1:C:1227:G:OP1	34:p:119:ARG:NH2	2.20	0.75
47:W:26:LEU:HD21	47:W:37:ALA:HB1	1.69	0.75
19:I:2100:G:H1	19:I:2189:U:H3	1.33	0.75
1:C:198:G:C6	1:C:459:A:N6	2.55	0.74
47:W:105:ALA:HA	47:W:108:ASP:HB2	1.67	0.74
36:q:7:ARG:HH11	36:q:7:ARG:CG	1.99	0.74
1:C:204:C:O2'	1:C:206:G:N7	2.19	0.74
19:I:1636:U:P	53:I:3077:MG:MG	1.69	0.74
1:C:1445:C:H1'	1:C:1446:A:H8	1.51	0.74

Continued on next page...

For Manuscript Review

Continued from previous page...

Atom-1	Atom-2	Interatomic distance (Å)	Clash overlap (Å)
6:3:35:ARG:NH2	38:r:117:PRO:O	2.20	0.73
19:I:2166:U:O4	19:I:2170:A:N6	2.20	0.73
22:j:12:VAL:HG13	22:j:50:LYS:HB3	1.70	0.73
19:I:883:G:N1	19:I:894:U:O2	2.17	0.73
19:I:543:G:H2'	19:I:544:C:H5'	1.71	0.73
39:S:71:ARG:NH1	39:S:106:GLU:OE2	2.17	0.73
38:r:59:THR:HG22	38:r:61:PHE:H	1.54	0.73
47:W:34:HIS:O	47:W:102:ARG:NH2	2.22	0.72
19:I:1871:A:HO2'	19:I:1872:A:H8	1.37	0.72
19:I:2348:U:H5'	22:j:46:HIS:CD2	2.24	0.72
19:I:1039:A:N1	19:I:1116:G:O6	2.23	0.72
44:v:41:ARG:HE	44:v:44:ALA:N	1.87	0.72
16:g:2:LYS:N	19:I:102:U:O4	2.22	0.72
19:I:1867:G:H2'	19:I:1868:C:C5	2.24	0.72
19:I:1875:G:H21	19:I:1876:A:H62	1.38	0.72
11:E:14:ILE:HG22	11:E:15:VAL:HG13	1.72	0.72
44:v:20:TYR:O	44:v:24:ARG:N	2.21	0.72
47:W:18:LEU:HD23	47:W:25:ARG:HA	1.69	0.72
47:W:31:THR:HB	47:W:34:HIS:HB2	1.70	0.71
36:q:41:PRO:HA	36:q:72:ARG:HD2	1.72	0.71
23:K:120:VAL:HG22	33:P:91:PHE:HB3	1.72	0.71
1:C:730:C:OP1	52:z:61:ARG:NH2	2.23	0.71
19:I:2107:G:N1	19:I:2182:U:N3	2.37	0.71
9:D:13:GLY:O	9:D:15:HIS:ND1	2.23	0.71
19:I:52:A:H2	19:I:119:A:H62	1.37	0.71
46:w:14:GLU:OE2	46:w:84:ARG:NH2	2.22	0.71
9:D:13:GLY:HA3	9:D:208:ARG:HH12	1.55	0.71
9:D:114:LEU:HD12	9:D:144:LEU:HB3	1.73	0.71
36:q:17:LEU:HD13	36:q:93:ALA:CB	2.22	0.70
36:q:65:TYR:HB3	44:v:96:LEU:HD11	1.72	0.70
36:q:77:VAL:HG22	36:q:78:GLU:N	2.06	0.70
44:v:16:LEU:HG	44:v:55:SER:HB3	1.72	0.70
1:C:969:A:H4'	1:C:970:G:H5"	1.74	0.70
1:C:974:C:O2'	44:v:13:ARG:NH1	2.25	0.70
19:I:278:A:OP2	19:I:361:G:N1	2.23	0.70
19:I:2134:A:H1'	19:I:2159:G:H1'	1.72	0.70
29:N:38:MET:HE3	29:N:53:ALA:HB1	1.74	0.69
1:C:176:C:H1'	1:C:177:G:H2'	1.73	0.69
47:W:28:VAL:O	47:W:95:SER:HB2	1.92	0.69
19:I:2122:U:OP2	19:I:2169:A:O2'	2.09	0.69
1:C:874:C:OP1	40:t:9:ARG:NH2	2.25	0.69

Continued on next page...

For Manuscript Review

Continued from previous page...

Atom-1	Atom-2	Interatomic distance (Å)	Clash overlap (Å)
19:I:896:A:OP2	19:I:897:C:N4	2.25	0.69
1:C:1444:G:H5”	1:C:1445:C:OP2	1.91	0.69
11:E:36:ASP:OD1	11:E:59:ARG:NH2	2.23	0.69
19:I:881:G:N2	19:I:895:U:O2’	2.22	0.69
19:I:1172:C:N3	19:I:1177:G:N1	2.36	0.69
19:I:1913:A:N6	19:I:1916:A:OP2	2.26	0.69
19:I:2895:G:N2	19:I:2896:C:H41	1.90	0.69
17:H:38:ARG:NH1	17:H:98:GLU:O	2.26	0.69
19:I:2291:U:OP1	19:I:2380:C:O2’	2.10	0.69
36:q:34:ALA:CB	36:q:78:GLU:HB3	2.23	0.69
34:p:62:ASP:O	34:p:63:LEU:HD22	1.93	0.68
47:W:7:ARG:HH22	47:W:8:ILE:HD13	1.57	0.68
29:N:158:THR:HG22	29:N:160:ALA:H	1.58	0.68
36:q:53:ILE:HD11	36:q:63:ASP:HB2	1.75	0.68
19:I:1649:G:O2’	45:V:106:ASP:OD2	2.08	0.68
30:n:112:GLY:HA2	30:n:119:ARG:NE	2.08	0.68
9:D:13:GLY:O	9:D:15:HIS:N	2.22	0.68
19:I:1034:G:H3’	19:I:1035:U:C6	2.29	0.68
19:I:290:U:H3	19:I:350:G:H1	0.79	0.68
19:I:1036:G:N2	19:I:1120:G:N3	2.42	0.68
36:q:13:PHE:CD1	36:q:67:ILE:HD11	2.29	0.68
28:m:2:LYS:NZ	28:m:32:LYS:O	2.26	0.67
30:n:16:PRO:HG2	34:p:43:THR:HG22	1.75	0.67
9:D:114:LEU:HB2	9:D:144:LEU:HD23	1.74	0.67
36:q:7:ARG:HH11	36:q:7:ARG:HB2	1.50	0.67
9:D:13:GLY:HA2	9:D:208:ARG:HH22	1.59	0.67
33:P:30:LEU:O	33:P:36:ALA:N	2.27	0.67
47:W:72:ALA:O	47:W:76:LYS:N	2.27	0.67
19:I:1062:G:H3’	19:I:1063:G:H8	1.60	0.67
27:M:108:ILE:HG23	41:T:1:MET:HE1	1.76	0.67
12:e:44:LYS:NZ	19:I:2331:G:OP1	2.29	0.66
22:j:37:LYS:HD3	22:j:46:HIS:HB3	1.77	0.66
36:q:85:ASP:OD2	36:q:89:ARG:NE	2.28	0.66
19:I:2168:G:O2’	19:I:2169:A:O4’	2.13	0.66
33:P:30:LEU:HA	33:P:35:LYS:CB	2.25	0.66
19:I:2306:C:N3	19:I:2307:G:N2	2.43	0.66
19:I:2348:U:H5’	22:j:46:HIS:HD2	1.59	0.66
40:t:54:ARG:HE	40:t:62:GLU:HG3	1.60	0.66
42:u:11:ASP:HA	42:u:45:ILE:HB	1.77	0.66
47:W:34:HIS:ND1	47:W:54:VAL:HG22	2.10	0.66
47:W:99:TYR:OH	47:W:111:ARG:NH2	2.28	0.66

Continued on next page...

For Manuscript Review

Continued from previous page...

Atom-1	Atom-2	Interatomic distance (Å)	Clash overlap (Å)
23:K:121:ASP:HA	33:P:91:PHE:CD2	2.31	0.65
13:F:85:ASN:HB2	15:G:102:GLY:HA3	1.78	0.65
38:r:20:VAL:HG12	38:r:83:GLU:HB2	1.78	0.65
46:w:17:ARG:N	46:w:21:ASP:OD2	2.28	0.65
48:x:18:GLN:HE22	48:x:35:ARG:HH11	1.43	0.65
7:b:25:GLU:HG2	7:b:26:LYS:HD2	1.78	0.65
22:j:9:ILE:HG21	22:j:25:LYS:HE3	1.79	0.65
1:C:1486:A:HO2’	1:C:1487:A:H8	1.45	0.65
11:E:154:SER:HB3	11:E:165:THR:HG23	1.78	0.65
14:f:39:TRP:HB3	33:P:32:PRO:HA	1.77	0.65
19:I:1173:U:O2	19:I:1176:U:N3	2.19	0.65
33:P:4:ILE:HB	33:P:16:GLY:HA2	1.77	0.65
34:p:84:THR:HG23	34:p:98:LEU:HD23	1.79	0.64
47:W:43:ASN:HD21	47:W:46:GLU:HB2	1.62	0.64
19:I:2895:G:H21	19:I:2896:C:H41	1.45	0.64
34:p:28:ILE:HG12	34:p:63:LEU:HD23	1.78	0.64
19:I:547:A:N3	19:I:548:G:N2	2.45	0.64
1:C:998:A:C6	1:C:1020:G:H1’	2.32	0.64
11:E:65:ARG:HG2	11:E:100:GLN:HB3	1.79	0.64
19:I:2204:G:OP2	23:K:147:LYS:NZ	2.27	0.64
33:P:40:THR:H	33:P:43:ASN:HB3	1.62	0.64
19:I:290:U:O4	19:I:350:G:O6	2.16	0.64
1:C:73:A:H8	1:C:90:G:H21	1.46	0.64
1:C:1273:A:OP1	36:q:9:ARG:NH2	2.31	0.64
19:I:355:U:H2’	19:I:356:G:H8	1.63	0.64
19:I:1068:G:O2’	19:I:1096:A:N3	2.29	0.64
41:T:78:ARG:NH1	41:T:80:SER:OG	2.31	0.64
34:p:52:LEU:HD22	34:p:87:LEU:HD13	1.79	0.64
36:q:5:ARG:HH11	36:q:5:ARG:HG2	1.63	0.64
47:W:68:LYS:HA	47:W:102:ARG:HG2	1.80	0.64
7:b:69:ARG:NH2	7:b:74:ILE:HD13	2.13	0.63
19:I:2305:U:HO2’	29:N:133:ARG:NH1	1.95	0.63
19:I:2313:C:O4’	29:N:37:ASN:ND2	2.31	0.63
1:C:1444:G:N2	1:C:1447:A:H2	1.96	0.63
44:v:41:ARG:HE	44:v:44:ALA:H	1.44	0.63
11:E:40:ARG:NH1	44:v:92:GLU:OE1	2.32	0.63
1:C:86:C:H2’	1:C:87:C:C6	2.33	0.63
34:p:28:ILE:HG21	34:p:35:LEU:HD12	1.81	0.63
1:C:1350:A:H2’	1:C:1351:A:C8	2.33	0.63
19:I:2122:U:H2’	19:I:2123:G:H8	1.63	0.63
1:C:1310:G:N1	1:C:1313:A:OP2	2.31	0.63

Continued on next page...

For Manuscript Review

Continued from previous page...

Atom-1	Atom-2	Interatomic distance (Å)	Clash overlap (Å)
2:0:4:ILE:HG22	2:0:106:VAL:HG22	1.81	0.63
34:p:115:LYS:HB2	34:p:118:LEU:HD12	1.80	0.63
36:q:80:THR:O	36:q:83:THR:HG23	1.99	0.63
47:W:31:THR:HG1	47:W:36:TYR:HE1	1.46	0.63
19:I:1913:A:N6	19:I:1915:3TD:O5'	2.32	0.63
27:M:189:THR:HG22	27:M:191:ASP:H	1.63	0.62
47:W:90:VAL:HG21	47:W:115:LEU:HD13	1.80	0.62
9:D:35:ARG:HB3	9:D:40:ILE:HD11	1.81	0.62
1:C:1172:G:N2	1:C:1175:G:OP2	2.32	0.62
19:I:1914:C:O2'	19:I:1915:3TD:OP2	2.18	0.62
23:K:107:PRO:HD2	23:K:110:LEU:HD22	1.82	0.62
44:v:41:ARG:HD2	44:v:43:ASN:H	1.63	0.62
13:F:184:ARG:NH1	13:F:187:GLU:OE1	2.33	0.62
31:O:13:ALA:O	31:O:15:VAL:N	2.33	0.62
1:C:1445:C:H2'	1:C:1446:A:O5'	2.00	0.62
13:F:163:GLU:OE2	13:F:164:GLN:NE2	2.32	0.62
14:f:60:ASP:HB3	33:P:1:MET:HE2	1.81	0.62
19:I:2139:U:O4	19:I:2152:G:O6	2.18	0.62
21:J:113:C:O2'	47:W:47:VAL:HG11	1.99	0.62
9:D:15:HIS:HB2	9:D:40:ILE:HG23	1.81	0.62
50:y:47:HIS:HB2	50:y:71:LYS:HD3	1.82	0.62
1:C:1017:G:O6	1:C:1018:G:O6	2.18	0.61
49:X:33:VAL:HG22	49:X:38:LYS:HG2	1.82	0.61
47:W:94:ARG:NE	47:W:99:TYR:HB2	2.14	0.61
1:C:1444:G:O3'	1:C:1445:C:O4'	2.18	0.61
10:d:80:HIS:HB2	10:d:85:LYS:HB2	1.82	0.61
22:j:39:PHE:CE2	22:j:41:PRO:HA	2.35	0.61
48:x:47:GLU:C	48:x:48:GLU:HG2	2.25	0.61
19:I:2127:G:H21	19:I:2173:A:H1'	1.65	0.61
4:Z:51:VAL:HG23	4:Z:52:PRO:CD	2.26	0.61
15:G:34:THR:HG22	15:G:52:LYS:HG2	1.83	0.61
13:F:37:ALA:HA	13:F:42:GLY:HA3	1.81	0.61
19:I:2756:U:OP2	28:m:19:ARG:NH1	2.31	0.61
23:K:72:ASP:OD1	23:K:119:GLY:HA2	2.01	0.61
19:I:993:G:OP1	51:Y:50:ARG:NH1	2.33	0.61
19:I:1721:G:N2	19:I:1738:G:O2'	2.34	0.61
25:L:5:VAL:H	25:L:32:ASN:HD21	1.48	0.61
27:M:145:ASP:HB3	27:M:184:ASP:HB2	1.82	0.61
44:v:21:PHE:HE1	44:v:51:LEU:HD23	1.65	0.61
19:I:1122:G:N2	19:I:1123:C:H41	1.99	0.61
31:O:10:VAL:HA	31:O:49:THR:HG22	1.81	0.61

Continued on next page...

For Manuscript Review

Continued from previous page...

Atom-1	Atom-2	Interatomic distance (Å)	Clash overlap (Å)
37:R:44:TYR:O	51:Y:64:ARG:NH2	2.32	0.61
38:r:93:ARG:NH2	38:r:112:ASP:OD1	2.34	0.61
1:C:85:U:H2'	1:C:86:C:C6	2.35	0.60
9:D:133:GLU:OE2	9:D:137:ARG:NH2	2.34	0.60
19:I:1175:A:H5'	19:I:1176:U:C6	2.36	0.60
19:I:1277:G:H5'	45:V:20:MET:HE3	1.83	0.60
1:C:1054:U:H5'	36:q:53:ILE:CG2	2.31	0.60
19:I:2144:G:N2	19:I:2147:A:H62	1.99	0.60
19:I:2751:G:OP2	31:O:3:ARG:NH1	2.35	0.60
27:M:168:ASP:OD1	27:M:169:VAL:N	2.34	0.60
19:I:1532:A:H2'	19:I:1533:C:C6	2.37	0.60
4:Z:24:LYS:HA	4:Z:94:THR:HG23	1.82	0.60
19:I:287:G:H2'	19:I:288:U:C6	2.37	0.60
33:P:60:GLU:O	33:P:64:ALA:N	2.31	0.60
9:D:23:TRP:CZ3	9:D:25:PRO:HA	2.37	0.60
1:C:2:A:N7	1:C:3:A:N6	2.49	0.60
19:I:1789:A:OP2	23:K:221:ARG:NH1	2.35	0.60
19:I:2107:G:N2	19:I:2182:U:O2	2.35	0.60
33:P:68:ARG:NH2	33:P:109:GLU:O	2.35	0.60
7:b:56:GLU:HB3	7:b:86:THR:HG23	1.83	0.60
30:n:37:SER:HA	30:n:40:GLU:HG2	1.83	0.60
1:C:538:G:OP1	13:F:56:ARG:NH2	2.34	0.59
19:I:652:U:OP1	19:I:654:A:N6	2.34	0.59
1:C:1272:U:H5'	1:C:1273:A:C8	2.37	0.59
36:q:77:VAL:CG2	36:q:78:GLU:N	2.64	0.59
23:K:41:GLY:O	23:K:43:ARG:NH1	2.36	0.59
30:n:67:GLU:OE2	30:n:70:ARG:NE	2.33	0.59
19:I:1090:A:H2'	19:I:1091:G:C8	2.38	0.59
34:p:29:VAL:HA	34:p:34:SER:HA	1.84	0.59
33:P:15:LEU:HD22	33:P:58:LEU:HD12	1.83	0.59
1:C:1300:A:N6	1:C:1325:G:O2'	2.35	0.59
19:I:1138:G:H21	37:R:108:MET:HE2	1.66	0.59
48:x:69:ASP:OD2	48:x:70:ARG:N	2.35	0.59
1:C:456:G:O2'	1:C:457:U:O5'	2.21	0.58
1:C:1017:G:C6	1:C:1018:G:O6	2.56	0.58
8:c:88:GLU:O	8:c:90:GLY:N	2.36	0.58
23:K:3:VAL:HG12	23:K:19:VAL:HG22	1.85	0.58
33:P:104:THR:HG22	33:P:110:VAL:H	1.68	0.58
1:C:656:U:O2'	1:C:830:G:OP1	2.21	0.58
1:C:961:C:OP2	1:C:962:A:O2'	2.18	0.58
30:n:40:GLU:HA	30:n:43:VAL:HG22	1.85	0.58

Continued on next page...

For Manuscript Review

Continued from previous page...

Atom-1	Atom-2	Interatomic distance (Å)	Clash overlap (Å)
34:p:29:VAL:HG23	34:p:34:SER:H	1.68	0.58
36:q:6:ILE:HD12	36:q:102:LEU:HB3	1.85	0.58
36:q:80:THR:HB	36:q:82:LYS:HG2	1.85	0.58
47:W:109:ALA:O	47:W:113:ALA:N	2.33	0.58
49:X:53:ARG:H	49:X:57:SER:HB3	1.67	0.58
19:I:1105:U:H2'	19:I:1106:G:C8	2.37	0.58
19:I:2168:G:O2'	19:I:2169:A:O5'	2.20	0.58
27:M:105:LEU:HD23	27:M:200:LEU:HD21	1.86	0.58
33:P:7:ASP:HB2	33:P:35:LYS:HG2	1.86	0.58
47:W:43:ASN:OD1	47:W:44:GLY:N	2.37	0.58
2:0:11:ARG:HH22	2:0:98:LYS:HD3	1.68	0.58
15:G:24:THR:HA	15:G:29:ARG:HA	1.83	0.58
33:P:5:LEU:HD12	33:P:14:SER:O	2.04	0.58
33:P:7:ASP:CB	33:P:35:LYS:HG2	2.33	0.58
37:R:87:ALA:HB3	37:R:92:MET:HE2	1.84	0.58
1:C:740:G:H2'	1:C:741:A:C8	2.38	0.58
19:I:52:A:H2	19:I:119:A:N6	2.02	0.58
19:I:1032:A:C2	19:I:1034:G:C8	2.85	0.58
19:I:2151:U:H2'	19:I:2152:G:C8	2.36	0.58
29:N:37:ASN:OD1	29:N:38:MET:N	2.36	0.58
40:t:114:ARG:HG3	40:t:119:VAL:HB	1.84	0.58
36:q:82:LYS:H	36:q:82:LYS:NZ	2.01	0.58
44:v:21:PHE:CD1	44:v:25:ALA:HB2	2.39	0.58
1:C:319:A:C2	1:C:320:G:H2'	2.39	0.58
13:F:198:HIS:O	13:F:202:GLU:HG3	2.03	0.58
19:I:2223:G:O3'	23:K:265:LYS:NZ	2.37	0.58
19:I:2328:A:H2'	19:I:2329:U:C6	2.39	0.58
27:M:153:LEU:HG	27:M:171:ASP:HB2	1.84	0.58
36:q:33:GLY:HA2	36:q:83:THR:HG22	1.85	0.58
47:W:83:LEU:HD11	47:W:88:LYS:HB3	1.85	0.58
1:C:516:C:H41	40:t:50:ARG:NH2	2.01	0.57
47:W:114:GLY:O	47:W:116:GLN:NE2	2.37	0.57
20:i:54:VAL:HG23	20:i:55:ILE:HG12	1.85	0.57
33:P:26:ALA:HA	33:P:30:LEU:HB2	1.85	0.57
37:R:114:LEU:HG	37:R:118:MET:HE3	1.86	0.57
47:W:94:ARG:NH2	47:W:97:PHE:O	2.37	0.57
1:C:446:A:H62	1:C:474:U:H3	1.51	0.57
34:p:84:THR:HG21	34:p:103:PHE:HB3	1.86	0.57
1:C:453:U:H2'	1:C:454:A:H8	1.70	0.57
1:C:1018:G:H2'	1:C:1019:U:C6	2.39	0.57
36:q:24:GLU:OE2	36:q:24:GLU:CA	2.52	0.57

Continued on next page...

For Manuscript Review

Continued from previous page...

Atom-1	Atom-2	Interatomic distance (Å)	Clash overlap (Å)
47:W:68:LYS:NZ	47:W:101:GLY:HA2	2.19	0.57
1:C:1299:G:H22	1:C:1325:G:H2'	1.70	0.57
19:I:674:G:H1'	27:M:69:ARG:HD3	1.86	0.57
38:r:31:ILE:HG12	38:r:46:THR:HG22	1.85	0.57
1:C:940:A:H2'	1:C:941:G:C8	2.39	0.57
17:H:25:TYR:O	17:H:29:ILE:HD12	2.05	0.57
19:I:1122:G:H21	19:I:1123:C:H41	1.52	0.57
1:C:176:C:N4	1:C:188:G:H22	2.02	0.57
1:C:198:G:C2	1:C:459:A:N1	2.73	0.57
52:z:41:PRO:HD2	52:z:44:ILE:HD12	1.87	0.57
1:C:1117:A:O3'	36:q:37:ARG:NH2	2.38	0.57
1:C:1441:A:H5"	1:C:1442:C:H5	1.68	0.57
1:C:1445:C:O2'	1:C:1446:A:O4'	2.21	0.57
30:n:72:THR:HA	30:n:96:ARG:NH1	2.16	0.57
36:q:29:ALA:O	36:q:33:GLY:N	2.38	0.57
36:q:77:VAL:HG22	36:q:78:GLU:H	1.70	0.57
48:x:18:GLN:NE2	48:x:35:ARG:HH11	2.01	0.57
9:D:67:ILE:H	9:D:89:GLN:NE2	2.03	0.57
19:I:4:U:H2'	19:I:5:A:H8	1.70	0.57
19:I:1118:C:C3'	19:I:1119:U:H4'	2.35	0.57
19:I:2139:U:C4	19:I:2152:G:O6	2.58	0.57
19:I:2159:G:H2'	19:I:2160:C:C2	2.40	0.57
30:n:150:ALA:HB1	38:r:59:THR:HG21	1.86	0.57
19:I:1245:G:OP1	41:T:13:LYS:NZ	2.38	0.56
19:I:1913:A:C5	19:I:1915:3TD:N1	2.65	0.56
19:I:1980:G:O2'	19:I:1982:U:OP2	2.22	0.56
29:N:56:ASP:OD2	29:N:150:ARG:NH2	2.38	0.56
1:C:1141:C:O2'	34:p:7:TYR:OH	2.19	0.56
6:3:10:GLU:OE2	6:3:14:VAL:HG13	2.05	0.56
19:I:2107:G:O6	19:I:2182:U:O4	2.23	0.56
23:K:187:ASP:CG	33:P:123:ARG:HH12	2.13	0.56
25:L:49:GLN:NE2	25:L:79:LEU:HD13	2.21	0.56
33:P:80:ILE:HD12	33:P:144:VAL:HG13	1.87	0.56
1:C:357:A:OP2	40:t:31:ARG:NH1	2.37	0.56
1:C:669:A:H1'	38:r:118:HIS:CD2	2.40	0.56
1:C:1281:A:H2'	1:C:1282:A:C8	2.39	0.56
33:P:30:LEU:HD23	33:P:35:LYS:HB3	1.87	0.56
36:q:5:ARG:HD2	36:q:75:ASP:OD1	2.05	0.56
36:q:78:GLU:OE1	36:q:80:THR:N	2.38	0.56
4:Z:55:ASP:OD1	4:Z:56:GLY:N	2.39	0.56
13:F:99:ASP:OD1	13:F:100:ASN:N	2.38	0.56

Continued on next page...

For Manuscript Review

Continued from previous page...

Atom-1	Atom-2	Interatomic distance (Å)	Clash overlap (Å)
37:R:62:VAL:HG11	37:R:101:ILE:HD11	1.88	0.56
1:C:135:A:H1'	1:C:177:G:H22	1.70	0.56
34:p:27:LYS:HB3	34:p:62:ASP:CG	2.30	0.56
1:C:2:A:H3'	1:C:3:A:C2	2.41	0.56
13:F:187:GLU:N	13:F:190:ASP:OD2	2.36	0.56
16:g:10:SER:O	16:g:14:LEU:HD13	2.06	0.56
19:I:1:G:O2'	19:I:2:G:O4'	2.21	0.56
19:I:1715:G:O2'	19:I:1743:G:O6	2.18	0.56
23:K:120:VAL:HB	23:K:189:ARG:HH22	1.70	0.56
47:W:15:ARG:NE	47:W:95:SER:OG	2.22	0.56
1:C:456:G:O2'	1:C:457:U:H6	1.89	0.56
1:C:1445:C:H1'	1:C:1446:A:C8	2.38	0.56
19:I:277:G:H2'	19:I:361:G:C6	2.40	0.56
19:I:1070:A:H4'	19:I:1071:G:O5'	2.05	0.56
19:I:2372:U:O2'	19:I:2373:G:H5'	2.06	0.56
32:o:47:GLU:HG2	32:o:64:LYS:HB3	1.86	0.56
36:q:37:ARG:HA	36:q:37:ARG:NE	2.20	0.56
1:C:1320:U:H2'	1:C:1321:G:H8	1.71	0.56
19:I:2529:G:OP1	31:O:172:LYS:NZ	2.39	0.56
33:P:26:ALA:O	33:P:31:VAL:N	2.38	0.56
44:v:21:PHE:HD2	44:v:56:SER:HA	1.69	0.56
19:I:788:A:H1'	24:k:4:THR:HG21	1.88	0.56
37:R:31:GLU:HG2	37:R:142:ILE:HD12	1.87	0.56
47:W:17:LYS:HE3	47:W:21:LEU:HD11	1.88	0.56
47:W:29:HIS:O	47:W:36:TYR:N	2.39	0.56
21:J:9:G:O2'	47:W:44:GLY:O	2.23	0.55
41:T:73:ILE:HG23	41:T:106:GLU:H	1.70	0.55
19:I:2141:G:H2'	19:I:2142:A:C8	2.41	0.55
19:I:2379:G:O2'	19:I:2380:C:O5'	2.25	0.55
47:W:104:GLN:O	47:W:108:ASP:N	2.39	0.55
19:I:4:U:H2'	19:I:5:A:C8	2.41	0.55
22:j:39:PHE:HB2	22:j:46:HIS:CE1	2.41	0.55
36:q:54:SER:OG	36:q:55:PRO:HD2	2.05	0.55
38:r:82:LEU:HD23	38:r:100:LEU:HD21	1.89	0.55
49:X:14:LYS:HD3	49:X:77:HIS:HA	1.88	0.55
47:W:110:ALA:HA	47:W:113:ALA:HB3	1.88	0.55
19:I:1070:A:O2'	19:I:1098:A:OP2	2.23	0.55
29:N:64:LYS:HD3	35:Q:5:ILE:HD12	1.87	0.55
30:n:7:ILE:HG13	30:n:8:GLY:H	1.72	0.55
47:W:15:ARG:HG2	47:W:93:ASP:OD2	2.06	0.55
1:C:141:G:H2'	1:C:142:G:C8	2.42	0.55

Continued on next page...

For Manuscript Review

Continued from previous page...

Atom-1	Atom-2	Interatomic distance (Å)	Clash overlap (Å)
6:3:5:LYS:NZ	38:r:108:THR:O	2.29	0.55
13:F:15:GLU:OE2	13:F:63:ARG:NH1	2.39	0.55
19:I:544:C:H2'	19:I:545:U:C6	2.42	0.55
1:C:331:G:H2'	1:C:332:A:C8	2.42	0.55
1:C:1020:G:O6	1:C:1029:A:C6	2.60	0.55
11:E:50:ALA:HB1	11:E:76:VAL:HG22	1.88	0.55
13:F:118:VAL:HG22	13:F:123:ILE:HG13	1.88	0.55
22:j:39:PHE:HZ	22:j:44:ARG:HA	1.71	0.55
34:p:86:ALA:O	34:p:87:LEU:HB2	2.05	0.55
47:W:7:ARG:NH2	47:W:8:ILE:HD13	2.21	0.55
1:C:176:C:H42	1:C:188:G:H22	1.54	0.55
34:p:52:LEU:HB2	34:p:55:VAL:HG12	1.89	0.55
47:W:14:ALA:O	47:W:18:LEU:HD13	2.07	0.55
1:C:86:C:H2'	1:C:87:C:H6	1.71	0.55
11:E:57:ILE:HG23	11:E:64:ILE:HD11	1.89	0.55
1:C:1446:A:O2'	1:C:1447:A:O5'	2.25	0.55
10:d:62:THR:HG22	10:d:71:LYS:HD3	1.89	0.55
37:R:68:LYS:HB2	37:R:72:LYS:HG3	1.88	0.55
1:C:1322:C:H5”	42:u:28:THR:HG21	1.89	0.54
17:H:5:GLU:HB3	17:H:90:MET:HB2	1.88	0.54
19:I:878:A:H3'	19:I:879:G:H8	1.72	0.54
19:I:1118:C:H2'	19:I:1119:U:H4'	1.88	0.54
37:R:96:ARG:HD2	37:R:99:ARG:HB2	1.88	0.54
47:W:43:ASN:ND2	47:W:46:GLU:HB2	2.20	0.54
47:W:68:LYS:HG2	47:W:102:ARG:HG3	1.89	0.54
1:C:1295:U:O2'	1:C:1296:C:H5'	2.07	0.54
3:1:8:GLY:C	35:Q:64:PHE:HZ	2.15	0.54
5:2:74:ARG:O	5:2:78:ASN:ND2	2.38	0.54
32:o:48:ASP:OD1	32:o:49:PHE:N	2.38	0.54
47:W:38:GLN:HE21	47:W:50:ALA:HB1	1.71	0.54
1:C:198:G:C2	1:C:199:A:C4	2.95	0.54
19:I:882:G:O6	19:I:894:U:O4	2.25	0.54
19:I:1866:A:N7	19:I:1876:A:N6	2.55	0.54
36:q:18:ILE:O	36:q:22:THR:HG23	2.07	0.54
47:W:26:LEU:O	47:W:93:ASP:HB3	2.06	0.54
19:I:2113:U:O4	19:I:2167:U:O2'	2.26	0.54
27:M:9:GLN:N	27:M:9:GLN:OE1	2.41	0.54
19:I:2125:G:H1	19:I:2170:A:H5”	1.72	0.54
19:I:2163:A:H3'	19:I:2164:C:H5”	1.89	0.54
44:v:27:LEU:HD12	44:v:30:ILE:HD11	1.90	0.54
1:C:708:G:H2'	1:C:709:A:C8	2.42	0.54

Continued on next page...

For Manuscript Review

Continued from previous page...

Atom-1	Atom-2	Interatomic distance (Å)	Clash overlap (Å)
1:C:712:A:C8	38:r:118:HIS:HB3	2.43	0.54
1:C:712:A:H2	52:z:38:LYS:HE2	1.72	0.54
1:C:1244:A:H4'	34:p:70:GLY:H	1.72	0.54
19:I:882:G:N1	19:I:894:U:N3	2.55	0.54
19:I:995:C:OP2	51:Y:54:LYS:NZ	2.41	0.54
19:I:1079:C:HO2'	19:I:1080:A:H8	1.55	0.54
31:O:127:THR:HG22	31:O:128:GLN:H	1.71	0.54
44:v:13:ARG:HB3	44:v:60:GLN:HE21	1.73	0.54
50:y:5:ILE:HD11	50:y:62:ARG:HE	1.72	0.54
19:I:1064:C:H2'	19:I:1065:U:C5	2.42	0.54
52:z:37:GLY:O	52:z:63:ARG:NH2	2.41	0.54
1:C:1446:A:N3	1:C:1446:A:H3'	2.23	0.54
19:I:2745:C:O2	31:O:143:GLN:NE2	2.31	0.54
19:I:2895:G:H21	19:I:2896:C:N4	2.05	0.54
23:K:69:ARG:HH21	23:K:118:SER:HB2	1.72	0.54
23:K:135:ILE:O	23:K:167:ARG:NH2	2.41	0.54
47:W:38:GLN:HG2	47:W:51:ALA:N	2.21	0.54
3:1:50:ALA:HB1	3:1:57:HIS:HB3	1.90	0.54
22:j:43:VAL:HA	26:l:34:THR:O	2.08	0.54
36:q:64:GLN:O	44:v:99:ALA:HB3	2.08	0.54
47:W:99:TYR:HA	47:W:103:VAL:HB	1.89	0.54
1:C:181:U:H2'	1:C:182:G:H3'	1.89	0.53
9:D:20:THR:O	9:D:23:TRP:HD1	1.90	0.53
33:P:122:LEU:HD22	33:P:128:HIS:CG	2.43	0.53
36:q:7:ARG:NH1	36:q:7:ARG:CG	2.62	0.53
25:L:77:ARG:NH2	25:L:200:ASP:OD1	2.35	0.53
30:n:136:LYS:O	30:n:136:LYS:HG2	2.08	0.53
40:t:3:THR:HG22	40:t:5:ASN:H	1.73	0.53
47:W:35:ILE:HG12	47:W:106:LEU:HD12	1.89	0.53
19:I:1120:G:H2'	19:I:1121:C:O4'	2.08	0.53
19:I:1509:A:H2'	19:I:1510:G:C8	2.44	0.53
19:I:2286:G:O6	22:j:41:PRO:HG3	2.09	0.53
36:q:6:ILE:HD12	36:q:102:LEU:HD23	1.90	0.53
19:I:548:G:O2'	19:I:549:G:O4'	2.26	0.53
27:M:188:MET:HE2	27:M:193:VAL:HG22	1.90	0.53
30:n:70:ARG:NH1	30:n:97:ASN:HB3	2.23	0.53
34:p:89:GLU:CD	34:p:89:GLU:H	2.15	0.53
1:C:1445:C:H2'	1:C:1445:C:O2	2.08	0.53
13:F:13:ARG:HG2	13:F:34:ILE:HA	1.89	0.53
17:H:5:GLU:OE1	52:z:24:LYS:NZ	2.28	0.53
19:I:2119:A:N6	19:I:2167:U:O2'	2.32	0.53

Continued on next page...

For Manuscript Review

Continued from previous page...

Atom-1	Atom-2	Interatomic distance (Å)	Clash overlap (Å)
19:I:2372:U:C2	19:I:2373:G:N7	2.76	0.53
36:q:83:THR:O	36:q:84:VAL:C	2.50	0.53
41:T:124:GLY:H	41:T:144:GLU:HA	1.73	0.53
1:C:926:C:OP1	30:n:4:ARG:NH1	2.40	0.53
1:C:1032:U:H2’	1:C:1033:C:H6	1.72	0.53
9:D:49:MET:HA	9:D:52:GLU:HG3	1.91	0.53
11:E:155:GLY:O	11:E:196:ILE:HG12	2.08	0.53
27:M:119:ILE:HD11	27:M:185:LYS:HD3	1.91	0.53
44:v:17:ALA:O	44:v:22:ALA:N	2.40	0.53
47:W:37:ALA:HB2	47:W:106:LEU:HD21	1.90	0.53
1:C:707:G:H2’	1:C:708:G:C8	2.44	0.53
1:C:712:A:H5’	38:r:119:ASN:OD1	2.09	0.53
1:C:759:G:C4	1:C:806:G:C6	2.96	0.53
19:I:1067:A:H8	19:I:1067:A:OP1	1.92	0.53
19:I:1667:G:O2’	19:I:1991:U:O4	2.21	0.53
19:I:1720:U:H2’	19:I:1721:G:O4’	2.08	0.53
19:I:2379:G:HO2’	19:I:2380:C:H6	1.55	0.53
23:K:187:ASP:OD1	33:P:123:ARG:NH1	2.41	0.53
47:W:18:LEU:HB3	47:W:25:ARG:HB3	1.89	0.53
1:C:990:A:H2’	1:C:991:U:C6	2.44	0.53
9:D:211:THR:HA	9:D:214:LEU:HD23	1.91	0.53
10:d:81:PRO:HG2	43:U:20:LEU:HD11	1.90	0.53
30:n:93:PRO:HA	30:n:96:ARG:HG2	1.89	0.53
4:Z:34:GLU:OE2	4:Z:58:VAL:HG13	2.09	0.53
25:L:16:THR:OG1	25:L:18:ASP:OD1	2.23	0.53
1:C:1255:A:N6	1:C:1268:G:H1’	2.24	0.53
3:1:63:THR:HG22	3:1:64:ASP:H	1.73	0.53
19:I:645:C:H2’	19:I:647:G:N7	2.24	0.53
47:W:38:GLN:CG	47:W:51:ALA:H	2.20	0.53
1:C:1152:C:H5	1:C:1175:G:H1	1.57	0.52
11:E:6:HIS:NE2	44:v:89:MET:SD	2.83	0.52
33:P:115:VAL:O	33:P:116:ARG:NH1	2.40	0.52
35:Q:41:HIS:HB3	35:Q:44:PHE:HD2	1.74	0.52
36:q:67:ILE:HD13	44:v:95:GLY:HA3	1.89	0.52
42:u:33:ILE:HD11	42:u:63:PHE:HE2	1.73	0.52
1:C:1035:G:H2’	1:C:1036:A:C8	2.45	0.52
19:I:848:C:H2’	19:I:849:A:H8	1.75	0.52
1:C:210:U:H2’	1:C:211:C:C6	2.44	0.52
1:C:1034:A:H2’	1:C:1035:G:C8	2.45	0.52
19:I:2134:A:H4’	19:I:2159:G:H21	1.75	0.52
27:M:190:ALA:O	27:M:194:LYS:NZ	2.25	0.52

Continued on next page...

For Manuscript Review

Continued from previous page...

Atom-1	Atom-2	Interatomic distance (Å)	Clash overlap (Å)
1:C:8:A:C6	13:F:206:LYS:HB3	2.45	0.52
1:C:175:A:O2’	1:C:176:C:O2	2.27	0.52
9:D:11:LYS:HD3	9:D:12:ALA:N	2.23	0.52
11:E:6:HIS:CE1	11:E:8:ASN:HB3	2.45	0.52
19:I:784:G:H5’	19:I:785:G:OP1	2.10	0.52
19:I:1802:A:H2’	19:I:1803:A:C8	2.44	0.52
41:T:82:LEU:HD21	41:T:120:VAL:HG21	1.91	0.52
47:W:28:VAL:HG11	47:W:103:VAL:HG13	1.92	0.52
1:C:1058:G:H21	1:C:1184:G:H8	1.57	0.52
41:T:117:THR:O	41:T:117:THR:HG22	2.10	0.52
19:I:2304:G:O2’	19:I:2305:U:OP1	2.24	0.52
19:I:856:G:H2’	19:I:857:G:C8	2.44	0.52
19:I:1035:U:O2’	19:I:1036:G:H8	1.91	0.52
23:K:73:GLY:HA2	23:K:121:ASP:OD2	2.09	0.52
36:q:81:GLU:HA	36:q:84:VAL:HB	1.91	0.52
36:q:85:ASP:O	36:q:89:ARG:HG3	2.10	0.52
13:F:87:GLY:HA3	13:F:197:GLU:HG3	1.91	0.52
19:I:848:C:H2’	19:I:849:A:C8	2.45	0.52
19:I:2172:U:H3’	19:I:2173:A:H5’	1.92	0.52
39:S:105:ARG:NH2	49:X:32:VAL:HG21	2.25	0.52
1:C:72:G:H21	1:C:91:A:H62	1.58	0.51
19:I:1047:G:O2’	19:I:1110:G:N1	2.16	0.51
19:I:1532:A:H2’	19:I:1533:C:H6	1.73	0.51
19:I:2144:G:O2’	19:I:2145:C:H3’	2.10	0.51
20:i:38:HIS:ND1	20:i:39:LEU:O	2.37	0.51
30:n:142:HIS:O	30:n:144:MET:N	2.44	0.51
1:C:1271:C:O2’	1:C:1273:A:H8	1.93	0.51
17:H:1:MET:HG3	17:H:65:GLU:OE2	2.10	0.51
19:I:358:U:C2	19:I:359:G:C8	2.98	0.51
33:P:26:ALA:HA	33:P:30:LEU:HD12	1.92	0.51
36:q:85:ASP:O	36:q:86:ALA:C	2.53	0.51
26:l:54:ASP:HB3	41:T:57:LEU:HD22	1.93	0.51
30:n:15:ASP:OD1	30:n:44:TYR:OH	2.27	0.51
36:q:53:ILE:HD12	44:v:85:ARG:CD	2.38	0.51
1:C:1034:A:H2’	1:C:1035:G:H8	1.76	0.51
19:I:2788:C:H2’	19:I:2789:C:C6	2.46	0.51
34:p:83:ILE:HA	34:p:86:ALA:HB3	1.92	0.51
1:C:102:G:H5’	1:C:103:A:H5”	1.91	0.51
1:C:790:C:O3’	38:r:127:ARG:NH1	2.43	0.51
19:I:1141:U:H4’	19:I:1142:A:O4’	2.11	0.51
19:I:2316:G:H2’	19:I:2317:A:H8	1.76	0.51

Continued on next page...

For Manuscript Review

Continued from previous page...

Atom-1	Atom-2	Interatomic distance (Å)	Clash overlap (Å)
20:i:28:LEU:CD1	20:i:37:LYS:HB3	2.41	0.51
19:I:1034:G:O2'	19:I:1035:U:O5'	2.23	0.51
34:p:20:PHE:HB2	34:p:64:TYR:HB3	1.93	0.51
36:q:27:GLU:C	36:q:29:ALA:N	2.48	0.51
41:T:91:ASP:OD1	41:T:92:LEU:N	2.43	0.51
1:C:469:U:H2'	1:C:470:G:H8	1.76	0.51
19:I:299:A:N3	19:I:319:G:O2'	2.40	0.51
19:I:1087:G:N2	19:I:1103:A:N3	2.57	0.51
39:S:18:ARG:HG2	39:S:18:ARG:HH11	1.74	0.51
20:i:28:LEU:HD11	20:i:37:LYS:HD2	1.92	0.51
30:n:53:ARG:NH2	30:n:121:ALA:HB1	2.26	0.51
35:Q:28:VAL:HG11	35:Q:32:LEU:HD11	1.91	0.51
9:D:121:SER:HA	9:D:126:PHE:CD1	2.46	0.51
19:I:2636:C:O2'	25:L:45:TYR:OH	2.26	0.51
48:x:18:GLN:NE2	48:x:35:ARG:HD2	2.26	0.51
1:C:269:G:H5'	50:y:16:LYS:HD2	1.92	0.50
1:C:533:A:H2'	1:C:534:G:C8	2.46	0.50
33:P:40:THR:HG23	33:P:43:ASN:H	1.76	0.50
38:r:84:VAL:HG11	38:r:97:ILE:HD11	1.93	0.50
19:I:1057:A:H2'	19:I:1058:U:C6	2.47	0.50
33:P:135:HIS:ND1	33:P:137:GLU:HG3	2.26	0.50
36:q:22:THR:O	36:q:23:ALA:C	2.54	0.50
7:b:28:ASN:ND2	7:b:88:LYS:O	2.44	0.50
15:G:15:LEU:HD12	15:G:37:THR:HG22	1.92	0.50
19:I:2140:G:H2'	19:I:2141:G:C8	2.45	0.50
19:I:2187:U:H2'	19:I:2188:U:C6	2.46	0.50
33:P:84:ALA:HA	33:P:90:LEU:HA	1.93	0.50
36:q:85:ASP:HA	36:q:88:MET:CE	2.41	0.50
37:R:125:TYR:OH	37:R:132:HIS:NE2	2.40	0.50
1:C:487:A:H2'	1:C:488:G:O4'	2.10	0.50
1:C:512:C:H4'	1:C:513:C:O5'	2.11	0.50
19:I:2162:G:N7	19:I:2164:C:N4	2.60	0.50
19:I:2246:G:H2'	19:I:2247:A:C8	2.47	0.50
19:I:2639:A:H4'	37:R:96:ARG:NH2	2.27	0.50
1:C:470:G:H2'	1:C:471:U:C6	2.46	0.50
14:f:60:ASP:OD2	33:P:27:ARG:NH1	2.44	0.50
19:I:644:A:H2'	19:I:645:C:O4'	2.11	0.50
1:C:972:A:O2'	1:C:1316:C:H5	1.94	0.50
1:C:1026:G:C5	1:C:1027:G:H1'	2.46	0.50
19:I:878:A:H3'	19:I:879:G:C8	2.46	0.50
1:C:1212:C:H2'	1:C:1213:A:C8	2.46	0.50

Continued on next page...

For Manuscript Review

Continued from previous page...

Atom-1	Atom-2	Interatomic distance (Å)	Clash overlap (Å)
19:I:2071:A:H2'	19:I:2072:C:C6	2.47	0.50
48:x:46:LYS:HD3	48:x:48:GLU:O	2.11	0.50
1:C:453:U:H2'	1:C:454:A:C8	2.47	0.49
1:C:1286:C:H2'	1:C:1287:G:H8	1.76	0.49
8:c:93:VAL:HG22	8:c:94:ARG:H	1.76	0.49
22:j:10:LYS:O	22:j:53:LYS:N	2.25	0.49
34:p:22:LYS:O	34:p:61:LEU:N	2.45	0.49
23:K:142:HIS:ND1	23:K:193:GLY:O	2.42	0.49
1:C:646:U:O4	1:C:746:G:O2'	2.27	0.49
19:I:1073:A:H3'	19:I:1074:G:H8	1.77	0.49
19:I:2064:C:H2'	19:I:2065:C:C6	2.47	0.49
19:I:2897:U:H2'	19:I:2898:U:H6	1.77	0.49
47:W:28:VAL:HA	47:W:36:TYR:O	2.12	0.49
48:x:67:ILE:HG23	48:x:71:VAL:HG13	1.93	0.49
19:I:1075:C:H2'	19:I:1076:C:H5"	1.94	0.49
19:I:2126:A:C5	19:I:2163:A:H8	2.31	0.49
19:I:2305:U:H5"	29:N:131:GLY:HA3	1.93	0.49
33:P:30:LEU:CD2	33:P:35:LYS:HB3	2.42	0.49
1:C:1056:U:H2'	1:C:1057:C:C6	2.47	0.49
1:C:1446:A:O2'	1:C:1447:A:N3	2.44	0.49
4:Z:66:HIS:ND1	4:Z:94:THR:HG22	2.28	0.49
1:C:125:U:O2'	1:C:256:A:H8	1.96	0.49
1:C:1250:U:H1'	1:C:1252:G:C6	2.48	0.49
9:D:131:LYS:O	9:D:135:LEU:N	2.41	0.49
11:E:131:ARG:O	11:E:135:LYS:HG2	2.12	0.49
1:C:250:U:H2'	1:C:251:G:H8	1.78	0.49
9:D:67:ILE:H	9:D:89:GLN:HE21	1.61	0.49
9:D:166:ALA:HB3	9:D:191:SER:HG	1.78	0.49
11:E:114:LYS:HB2	11:E:185:ASN:HD22	1.78	0.49
11:E:156:ARG:HH11	11:E:193:TYR:HB3	1.77	0.49
29:N:36:LEU:CD1	29:N:57:LEU:HD11	2.42	0.49
47:W:24:THR:HB	47:W:90:VAL:HA	1.93	0.49
9:D:183:VAL:HG12	9:D:196:VAL:HG23	1.94	0.49
14:f:39:TRP:HB2	14:f:46:PHE:CE1	2.48	0.49
19:I:288:U:H2'	19:I:289:G:C8	2.48	0.49
19:I:613:A:O2'	19:I:614:A:H5'	2.12	0.49
19:I:2641:G:H5"	37:R:78:THR:HB	1.95	0.49
1:C:445:A:N6	1:C:474:U:O2'	2.44	0.49
1:C:549:U:H2'	1:C:550:C:C6	2.48	0.49
1:C:1350:A:H2'	1:C:1351:A:H8	1.77	0.49
9:D:66:LYS:HA	9:D:89:GLN:HE22	1.77	0.49

Continued on next page...

For Manuscript Review

Continued from previous page...

Atom-1	Atom-2	Interatomic distance (Å)	Clash overlap (Å)
9:D:93:ASN:OD1	9:D:94:HIS:ND1	2.45	0.49
19:I:1873:G:N3	19:I:1874:C:H1'	2.27	0.49
47:W:33:ARG:HG2	47:W:65:THR:O	2.13	0.49
19:I:2119:A:O2'	19:I:2120:G:O5'	2.27	0.49
21:J:28:C:OP1	47:W:34:HIS:CD2	2.65	0.49
35:Q:16:CYS:SG	35:Q:17:SER:N	2.86	0.49
36:q:6:ILE:CD1	36:q:102:LEU:HB3	2.43	0.49
38:r:64:GLN:HG2	38:r:99:ALA:HB2	1.95	0.49
1:C:533:A:H2'	1:C:534:G:H8	1.77	0.48
31:O:42:GLU:OE1	31:O:55:ARG:NH2	2.46	0.48
36:q:71:LEU:O	36:q:72:ARG:HD3	2.12	0.48
1:C:83:G:H2'	1:C:84:C:C6	2.48	0.48
1:C:1245:A:H2'	1:C:1246:A:C8	2.47	0.48
5:2:29:ARG:O	5:2:33:LYS:HG3	2.13	0.48
19:I:2804:U:H2'	19:I:2805:C:C6	2.48	0.48
19:I:2897:U:H2'	19:I:2898:U:C6	2.48	0.48
47:W:7:ARG:HD2	47:W:97:PHE:CE1	2.48	0.48
47:W:68:LYS:HZ3	47:W:101:GLY:HA2	1.77	0.48
9:D:76:ALA:O	9:D:80:VAL:HG23	2.13	0.48
19:I:2379:G:N3	19:I:2380:C:C6	2.80	0.48
21:J:42:C:C5	29:N:66:LEU:HD22	2.48	0.48
36:q:35:GLN:HG2	36:q:77:VAL:HG13	1.95	0.48
5:2:44:LYS:HE3	5:2:86:LEU:HB3	1.95	0.48
19:I:1494:A:H2'	19:I:1495:A:C8	2.49	0.48
19:I:2147:A:N7	19:I:2148:G:H1'	2.28	0.48
19:I:2845:U:H5"	49:X:52:ASN:O	2.12	0.48
34:p:33:ARG:O	34:p:33:ARG:HG2	2.13	0.48
36:q:35:GLN:HG2	36:q:35:GLN:O	2.13	0.48
44:v:41:ARG:NE	44:v:43:ASN:HB3	2.28	0.48
50:y:48:ASP:HA	50:y:75:LEU:HD22	1.94	0.48
21:J:106:G:H2'	21:J:107:G:O4'	2.13	0.48
13:F:145:ILE:HD11	13:F:178:MET:SD	2.53	0.48
19:I:1035:U:OP2	19:I:1035:U:H3'	2.13	0.48
36:q:10:LEU:HD11	36:q:98:VAL:HG23	1.96	0.48
36:q:10:LEU:CD1	36:q:98:VAL:HG23	2.44	0.48
12:e:44:LYS:NZ	19:I:2330:G:O3'	2.45	0.48
19:I:1034:G:O3'	19:I:1035:U:H2'	2.13	0.48
19:I:1913:A:H2'	19:I:1914:C:H2'	1.94	0.48
19:I:2153:C:H2'	19:I:2154:A:C8	2.48	0.48
34:p:118:LEU:HB3	34:p:123:ARG:O	2.13	0.48
41:T:73:ILE:CG2	41:T:106:GLU:H	2.26	0.48

Continued on next page...

For Manuscript Review

Continued from previous page...

Atom-1	Atom-2	Interatomic distance (Å)	Clash overlap (Å)
1:C:187:A:H2'	1:C:188:G:C8	2.48	0.48
1:C:893:C:O2'	1:C:894:A:OP1	2.27	0.48
19:I:1118:C:C2'	19:I:1119:U:H4'	2.43	0.48
4:Z:1:MET:HA	4:Z:43:ASN:HA	1.96	0.48
13:F:104:ARG:HG3	13:F:168:PRO:HG3	1.95	0.48
35:Q:35:ASP:OD2	42:u:3:ARG:NH1	2.47	0.48
7:b:56:GLU:HG3	7:b:57:VAL:HG23	1.96	0.48
22:j:43:VAL:HG12	26:l:34:THR:OG1	2.14	0.48
9:D:188:ASP:CG	9:D:189:THR:H	2.22	0.47
19:I:1529:G:O6	19:I:1542:U:O4	2.32	0.47
19:I:1800:C:OP2	23:K:182:ARG:NH2	2.45	0.47
19:I:2327:A:H2'	19:I:2328:A:C8	2.49	0.47
21:J:28:C:OP1	47:W:34:HIS:NE2	2.47	0.47
1:C:407:G:H5"	1:C:408:A:H5'	1.97	0.47
3:1:14:HIS:O	3:1:18:LYS:HG2	2.13	0.47
19:I:1062:G:H3'	19:I:1063:G:C8	2.46	0.47
33:P:3:VAL:HG12	33:P:38:PRO:HA	1.95	0.47
1:C:745:U:H2'	1:C:746:G:O4'	2.15	0.47
3:1:22:ALA:HB1	3:1:27:ASP:HB3	1.95	0.47
9:D:166:ALA:HB3	9:D:191:SER:OG	2.13	0.47
11:E:49:LYS:O	11:E:72:ARG:NH2	2.47	0.47
17:H:43:GLY:HA2	17:H:58:HIS:CD2	2.49	0.47
19:I:45:G:H5'	19:I:46:G:OP1	2.14	0.47
19:I:1248:G:OP1	27:M:44:ARG:NH1	2.40	0.47
29:N:162:SER:OG	29:N:165:GLU:HG3	2.14	0.47
40:t:33:VAL:HG22	40:t:79:VAL:HG12	1.95	0.47
45:V:58:ASP:HB2	45:V:80:PHE:CE1	2.49	0.47
19:I:1105:U:H2'	19:I:1106:G:H8	1.80	0.47
22:j:43:VAL:HG23	22:j:45:GLN:N	2.29	0.47
27:M:97:ASN:HB3	27:M:100:MET:HG2	1.95	0.47
34:p:34:SER:OG	34:p:35:LEU:N	2.45	0.47
47:W:28:VAL:HG13	47:W:106:LEU:HD13	1.96	0.47
48:x:46:LYS:HD3	48:x:48:GLU:H	1.79	0.47
19:I:277:G:H2'	19:I:361:G:C5	2.49	0.47
19:I:2345:G:H21	19:I:2381:A:C2'	2.28	0.47
33:P:5:LEU:HD23	33:P:30:LEU:HD13	1.96	0.47
34:p:87:LEU:O	34:p:87:LEU:HD23	2.15	0.47
36:q:44:THR:HG23	36:q:69:THR:O	2.13	0.47
46:w:53:ARG:O	46:w:57:LEU:HG	2.15	0.47
1:C:204:C:OP2	1:C:205:G:N2	2.48	0.47
1:C:384:U:H2'	1:C:385:C:C6	2.50	0.47

Continued on next page...

For Manuscript Review

Continued from previous page...

Atom-1	Atom-2	Interatomic distance (Å)	Clash overlap (Å)
1:C:1247:G:H2'	1:C:1248:G:H8	1.78	0.47
1:C:1270:G:H2'	1:C:1271:C:O4'	2.15	0.47
11:E:60:PRO:HB3	36:q:94:ALA:CB	2.36	0.47
13:F:28:ILE:HD12	13:F:30:THR:OG1	2.15	0.47
19:I:1086:A:H5"	19:I:1103:A:H2	1.78	0.47
19:I:1119:U:O2	19:I:1120:G:C8	2.68	0.47
22:j:13:SER:HB2	22:j:49:TYR:CZ	2.50	0.47
40:t:42:PRO:HB3	40:t:89:ASP:OD1	2.14	0.47
43:U:1:MET:HE1	43:U:44:ARG:HA	1.96	0.47
1:C:326:G:C2	1:C:327:U:C6	3.02	0.47
13:F:172:GLU:HB2	13:F:183:LYS:HE2	1.97	0.47
14:f:10:LYS:NZ	19:I:397:U:OP2	2.45	0.47
18:h:4:THR:HG23	18:h:37:GLU:OE2	2.14	0.47
19:I:155:A:H2'	19:I:156:A:H8	1.78	0.47
19:I:544:C:O2'	19:I:545:U:O4'	2.32	0.47
19:I:859:G:O2'	19:I:916:G:O6	2.27	0.47
19:I:1092:C:H2'	19:I:1093:G:O4'	2.15	0.47
19:I:1486:U:H2'	19:I:1487:U:C6	2.50	0.47
19:I:1746:A:H2'	19:I:1747:U:C6	2.50	0.47
21:J:66:A:H61	21:J:107:G:H2'	1.79	0.47
23:K:121:ASP:HA	33:P:91:PHE:CE2	2.50	0.47
33:P:137:GLU:CD	33:P:138:VAL:HG13	2.40	0.47
34:p:9:THR:O	34:p:85:ARG:HD2	2.15	0.47
44:v:31:ILE:HG23	44:v:45:VAL:HG21	1.97	0.47
50:y:75:LEU:H	50:y:75:LEU:HD23	1.78	0.47
1:C:951:U:H4'	3:1:79:THR:HG23	1.97	0.47
6:3:11:PRO:HG2	6:3:14:VAL:HG12	1.97	0.47
19:I:479:A:N3	19:I:481:G:H5"	2.30	0.47
19:I:882:G:H21	19:I:896:A:N6	2.13	0.47
30:n:53:ARG:HH22	30:n:121:ALA:HB1	1.80	0.47
40:t:81:LEU:O	40:t:98:VAL:HG12	2.15	0.47
36:q:29:ALA:O	36:q:31:ARG:N	2.48	0.47
36:q:29:ALA:C	36:q:31:ARG:N	2.69	0.47
47:W:11:ALA:HA	47:W:95:SER:C	2.40	0.47
1:C:1250:U:H1'	1:C:1252:G:C5	2.49	0.47
7:b:47:VAL:HG13	7:b:55:VAL:HG21	1.96	0.47
10:d:77:VAL:HG21	10:d:86:LEU:HD22	1.96	0.47
17:H:9:MET:HB2	17:H:85:ILE:O	2.15	0.47
19:I:155:A:H2'	19:I:156:A:C8	2.50	0.47
19:I:1198:U:H2'	19:I:1199:U:C6	2.49	0.47
34:p:52:LEU:HB2	34:p:55:VAL:CG1	2.45	0.47

Continued on next page...

For Manuscript Review

Continued from previous page...

Atom-1	Atom-2	Interatomic distance (Å)	Clash overlap (Å)
38:r:117:PRO:HB2	38:r:119:ASN:O	2.15	0.47
1:C:84:C:H2'	1:C:85:U:C6	2.50	0.46
6:3:3:VAL:HG23	38:r:111:THR:HG22	1.97	0.46
8:c:54:GLN:CB	8:c:55:PRO:HD3	2.45	0.46
9:D:168:HIS:ND1	9:D:169:GLU:OE2	2.42	0.46
19:I:2377:A:H3'	19:I:2378:A:C8	2.50	0.46
19:I:2547:A:H2'	19:I:2548:U:C6	2.50	0.46
15:G:150:PRO:HA	15:G:153:VAL:HG12	1.96	0.46
19:I:1176:U:H2'	19:I:1177:G:H8	1.80	0.46
19:I:2134:A:H4'	19:I:2159:G:N2	2.31	0.46
19:I:2380:C:C4	19:I:2381:A:H1'	2.50	0.46
31:O:19:ILE:O	31:O:19:ILE:HG22	2.15	0.46
47:W:34:HIS:CE1	47:W:54:VAL:HG22	2.50	0.46
1:C:1244:A:H2'	1:C:1245:A:C8	2.51	0.46
19:I:1736:U:H2'	19:I:1737:G:C8	2.50	0.46
34:p:6:TYR:O	34:p:7:TYR:HB2	2.15	0.46
36:q:17:LEU:HD11	36:q:96:VAL:HG12	1.97	0.46
1:C:471:U:H2'	1:C:472:U:C6	2.50	0.46
1:C:1032:U:H2'	1:C:1033:C:C6	2.50	0.46
19:I:1386:C:H2'	19:I:1387:A:C8	2.51	0.46
19:I:1867:G:H2'	19:I:1868:C:H5	1.79	0.46
19:I:2720:U:OP1	49:X:53:ARG:NH2	2.48	0.46
22:j:39:PHE:HB2	22:j:46:HIS:HE1	1.81	0.46
31:O:13:ALA:O	31:O:15:VAL:HG12	2.16	0.46
33:P:117:LEU:HD13	33:P:121:VAL:HG22	1.98	0.46
34:p:80:ARG:O	34:p:84:THR:OG1	2.21	0.46
19:I:871:U:H2'	19:I:872:U:C6	2.50	0.46
19:I:1021:A:O2'	19:I:1123:C:OP1	2.24	0.46
22:j:9:ILE:N	22:j:23:THR:O	2.33	0.46
36:q:59:LYS:HG3	36:q:59:LYS:O	2.14	0.46
45:V:20:MET:HE2	45:V:40:LYS:HD3	1.97	0.46
1:C:182:G:H1'	1:C:183:A:C2	2.49	0.46
1:C:1115:U:H2'	1:C:1116:U:C6	2.51	0.46
1:C:1445:C:C1'	1:C:1446:A:H8	2.23	0.46
39:S:104:THR:HG22	39:S:106:GLU:OE1	2.14	0.46
1:C:574:C:H2'	1:C:575:G:O4'	2.16	0.46
1:C:668:G:H2'	1:C:669:A:H8	1.81	0.46
1:C:1341:G:H4'	1:C:1342:U:O5'	2.15	0.46
3:1:9:PRO:HB3	35:Q:64:PHE:HE2	1.80	0.46
19:I:1034:G:H4'	19:I:1035:U:OP1	2.16	0.46
19:I:1079:C:O2'	19:I:1080:A:H8	1.99	0.46

Continued on next page...

For Manuscript Review

Continued from previous page...

Atom-1	Atom-2	Interatomic distance (Å)	Clash overlap (Å)
19:I:1120:G:C2	19:I:1121:C:C2	3.04	0.46
19:I:1867:G:H2'	19:I:1867:G:N3	2.31	0.46
34:p:39:PHE:O	34:p:45:ARG:NH2	2.48	0.46
36:q:18:ILE:HG23	36:q:19:ASP:N	2.30	0.46
47:W:31:THR:OG1	47:W:36:TYR:HE1	1.98	0.46
1:C:210:U:H2'	1:C:211:C:H6	1.80	0.46
1:C:315:A:N6	1:C:323:A:OP2	2.49	0.46
1:C:319:A:C5	1:C:320:G:C6	3.04	0.46
1:C:378:G:H2'	1:C:379:C:C6	2.51	0.46
1:C:976:U:H4'	1:C:977:A:O5'	2.16	0.46
1:C:1049:A:O2'	11:E:156:ARG:NH2	2.39	0.46
3:1:7:LYS:HD2	3:1:8:GLY:O	2.15	0.46
19:I:1482:G:O6	19:I:1508:A:N6	2.49	0.46
19:I:2323:G:H21	19:I:2324:U:C1'	2.28	0.46
19:I:2377:A:H3'	19:I:2378:A:H8	1.81	0.46
19:I:2472:G:H21	19:I:2478:A:H62	1.62	0.46
27:M:130:LYS:HA	27:M:130:LYS:HD2	1.85	0.46
33:P:5:LEU:HB2	33:P:16:GLY:H	1.81	0.46
39:S:109:SER:O	39:S:113:MET:HG2	2.15	0.46
9:D:117:LEU:HD22	9:D:141:LEU:HD13	1.97	0.46
15:G:25:VAL:HG22	15:G:26:LYS:H	1.81	0.46
19:I:1097:U:H3'	19:I:1098:A:H8	1.81	0.46
19:I:1182:G:H2'	19:I:1183:U:O4'	2.15	0.46
21:J:51:G:OP2	47:W:63:LYS:NZ	2.35	0.46
29:N:40:VAL:HG23	29:N:41:GLY:H	1.81	0.46
34:p:41:ARG:NH1	34:p:43:THR:OG1	2.49	0.46
34:p:94:LEU:HD23	34:p:97:GLU:OE1	2.16	0.46
36:q:33:GLY:HA2	36:q:83:THR:CG2	2.46	0.46
39:S:5:GLN:HA	39:S:20:MET:HE3	1.97	0.46
1:C:94:C:O2'	1:C:95:A:N7	2.44	0.46
1:C:940:A:H2'	1:C:941:G:H8	1.79	0.46
7:b:47:VAL:HG23	7:b:51:PHE:HD2	1.80	0.46
19:I:172:A:H2'	19:I:173:A:H8	1.81	0.46
19:I:880:G:N3	19:I:880:G:H2'	2.31	0.46
19:I:2126:A:N6	19:I:2171:A:OP2	2.49	0.46
36:q:7:ARG:HD2	36:q:75:ASP:OD2	2.16	0.46
48:x:19:VAL:HG12	48:x:37:GLY:C	2.41	0.46
1:C:1124:A:H61	1:C:1138:G:H1'	1.82	0.45
19:I:287:G:H2'	19:I:288:U:H6	1.79	0.45
19:I:703:U:H2'	19:I:704:G:O4'	2.17	0.45
34:p:52:LEU:HD11	34:p:57:MET:HE2	1.98	0.45

Continued on next page...

For Manuscript Review

Continued from previous page...

Atom-1	Atom-2	Interatomic distance (Å)	Clash overlap (Å)
1:C:1445:C:O2	1:C:1445:C:C2'	2.64	0.45
8:c:54:GLN:HB2	8:c:55:PRO:HD3	1.98	0.45
17:H:69:GLU:O	17:H:73:GLU:OE1	2.34	0.45
19:I:878:A:N6	19:I:899:A:O2'	2.49	0.45
19:I:1032:A:H2	19:I:1034:G:C8	2.17	0.45
19:I:1534:U:H5"	19:I:1535:A:OP1	2.15	0.45
19:I:1819:A:H4'	19:I:1820:U:H5"	1.98	0.45
29:N:113:ASP:OD1	42:u:71:ARG:NH2	2.50	0.45
36:q:88:MET:HE3	36:q:88:MET:HB3	1.71	0.45
47:W:108:ASP:OD1	47:W:111:ARG:NE	2.44	0.45
48:x:52:LEU:O	48:x:54:LEU:N	2.50	0.45
1:C:198:G:C6	1:C:459:A:C6	3.04	0.45
1:C:365:A:H2'	1:C:366:C:O4'	2.17	0.45
1:C:1130:U:H3'	1:C:1131:C:H6	1.81	0.45
25:L:49:GLN:HE21	25:L:79:LEU:HD13	1.80	0.45
1:C:1118:G:O2'	1:C:1121:A:N6	2.49	0.45
9:D:161:LEU:HD13	9:D:176:ALA:HB2	1.98	0.45
19:I:1179:G:H3'	19:I:1180:U:C5'	2.46	0.45
29:N:40:VAL:HG23	29:N:41:GLY:N	2.32	0.45
1:C:1022:C:H2'	1:C:1023:U:O4'	2.16	0.45
16:g:58:ASN:ND2	19:I:112:U:H5'	2.31	0.45
19:I:827:U:O2'	19:I:2068:U:C2	2.70	0.45
23:K:8:PRO:HB3	23:K:14:ARG:HG3	1.98	0.45
36:q:65:TYR:CB	44:v:96:LEU:HD11	2.42	0.45
41:T:77:ILE:HD11	41:T:108:ALA:HB1	1.97	0.45
47:W:30:ARG:HA	47:W:35:ILE:HA	1.98	0.45
1:C:73:A:N6	1:C:90:G:H4'	2.32	0.45
19:I:1138:G:N2	37:R:108:MET:HE2	2.32	0.45
19:I:1484:U:H2'	19:I:1485:U:H6	1.82	0.45
20:i:9:THR:HG22	20:i:12:LYS:HG2	1.98	0.45
21:J:28:C:H2'	21:J:29:A:C8	2.52	0.45
36:q:30:LYS:HB3	36:q:30:LYS:HE2	1.65	0.45
47:W:38:GLN:HG2	47:W:50:ALA:HB1	1.98	0.45
1:C:1385:U:H2'	1:C:1386:G:C8	2.51	0.45
1:C:1486:A:O2'	1:C:1487:A:H8	1.97	0.45
19:I:1065:U:O2'	19:I:1066:U:O4'	2.35	0.45
19:I:2163:A:N3	19:I:2163:A:H2'	2.32	0.45
33:P:30:LEU:HB3	33:P:36:ALA:CB	2.47	0.45
34:p:22:LYS:HB2	34:p:61:LEU:HD22	1.98	0.45
39:S:5:GLN:HA	39:S:20:MET:CE	2.46	0.45
1:C:1003:U:H5'	1:C:1004:C:C6	2.52	0.45

Continued on next page...

For Manuscript Review

Continued from previous page...

Atom-1	Atom-2	Interatomic distance (Å)	Clash overlap (Å)
1:C:1371:A:C5	30:n:7:ILE:HD13	2.52	0.45
9:D:79:ALA:HB1	9:D:214:LEU:HD21	1.97	0.45
19:I:278:A:N3	19:I:278:A:H2'	2.32	0.45
19:I:2380:C:OP1	47:W:17:LYS:HE2	2.17	0.45
19:I:2591:C:H2'	19:I:2592:G:C8	2.51	0.45
43:U:41:LEU:HD11	43:U:124:LEU:HD22	1.98	0.45
47:W:41:ALA:HB3	47:W:43:ASN:ND2	2.31	0.45
50:y:60:GLU:OE2	50:y:77:ARG:HG2	2.16	0.45
1:C:836:U:H2'	1:C:837:U:H5"	1.98	0.45
1:C:1445:C:C1'	1:C:1446:A:C8	2.99	0.45
5:2:70:ASN:O	5:2:74:ARG:HG3	2.17	0.45
6:3:5:LYS:O	6:3:18:ARG:NH2	2.49	0.45
7:b:89:GLU:OE2	7:b:89:GLU:N	2.50	0.45
9:D:13:GLY:H	9:D:15:HIS:HE1	1.64	0.45
9:D:171:ILE:O	9:D:175:GLU:HG3	2.17	0.45
19:I:2123:G:H2'	19:I:2124:G:H8	1.81	0.45
19:I:2161:C:H2'	19:I:2162:G:C8	2.52	0.45
19:I:2198:A:N3	33:P:29:PHE:HB2	2.32	0.45
23:K:77:VAL:HG12	23:K:115:GLN:OE1	2.17	0.45
31:O:160:LYS:HB3	31:O:160:LYS:HE3	1.83	0.45
39:S:20:MET:SD	39:S:44:LYS:HE3	2.56	0.45
1:C:1444:G:H3'	1:C:1445:C:H6	1.81	0.45
2:0:11:ARG:NH1	2:0:82:MET:HE2	2.32	0.45
9:D:13:GLY:C	9:D:15:HIS:H	2.16	0.45
19:I:973:A:H5'	19:I:1188:U:H1'	1.99	0.45
19:I:1869:G:N2	19:I:1873:G:N3	2.65	0.45
19:I:2148:G:H2'	19:I:2149:U:C6	2.52	0.45
19:I:2373:G:H2'	19:I:2374:C:C6	2.52	0.45
31:O:133:LEU:HB3	31:O:141:ILE:HD11	1.99	0.45
36:q:37:ARG:NE	36:q:37:ARG:CA	2.79	0.45
36:q:85:ASP:CG	36:q:86:ALA:N	2.75	0.45
42:u:33:ILE:HD11	42:u:63:PHE:CE2	2.50	0.45
13:F:124:MET:SD	13:F:146:ARG:HG2	2.58	0.44
19:I:1056:G:H4'	19:I:1085:A:H2	1.82	0.44
19:I:2379:G:O2'	19:I:2380:C:H6	2.00	0.44
20:i:28:LEU:HD13	20:i:37:LYS:HB3	1.99	0.44
22:j:44:ARG:HE	26:l:37:ALA:HA	1.82	0.44
34:p:112:GLU:OE1	34:p:121:ALA:HB1	2.17	0.44
39:S:79:PHE:CD2	49:X:70:VAL:HG12	2.51	0.44
47:W:7:ARG:O	47:W:10:ARG:N	2.49	0.44
1:C:1416:G:H5"	39:S:48:PRO:HB3	1.99	0.44

Continued on next page...

For Manuscript Review

Continued from previous page...

Atom-1	Atom-2	Interatomic distance (Å)	Clash overlap (Å)
10:d:63:ILE:HD11	10:d:72:VAL:HG21	1.98	0.44
36:q:31:ARG:HE	36:q:31:ARG:HB3	1.52	0.44
1:C:41:G:H2'	1:C:42:G:H8	1.83	0.44
1:C:72:G:N2	1:C:90:G:O2'	2.43	0.44
1:C:1471:U:H2'	1:C:1472:C:C6	2.52	0.44
13:F:114:ALA:O	13:F:118:VAL:HG23	2.18	0.44
19:I:2102:G:H3'	19:I:2103:C:C6	2.52	0.44
19:I:2306:C:H5'	29:N:133:ARG:HH12	1.82	0.44
19:I:2898:U:H2'	19:I:2899:A:C8	2.52	0.44
47:W:7:ARG:HH21	47:W:11:ALA:HB2	1.83	0.44
47:W:94:ARG:CD	47:W:99:TYR:HB2	2.47	0.44
6:3:20:LYS:O	6:3:24:GLU:HG2	2.18	0.44
11:E:21:THR:HG23	11:E:58:GLU:OE1	2.16	0.44
19:I:1913:A:N6	19:I:1915:3TD:H3'	2.26	0.44
19:I:2896:C:H2'	19:I:2897:U:H5'	1.98	0.44
33:P:6:LEU:H	33:P:35:LYS:C	2.26	0.44
34:p:8:GLY:HA3	34:p:19:VAL:HB	1.99	0.44
43:U:75:GLU:HB2	43:U:90:GLU:HG3	2.00	0.44
1:C:630:U:H5'	50:y:6:ARG:HH21	1.82	0.44
1:C:672:U:H2'	1:C:673:U:C6	2.51	0.44
1:C:925:C:O3'	30:n:4:ARG:NH1	2.50	0.44
3:1:63:THR:HG22	3:1:64:ASP:N	2.33	0.44
19:I:1682:G:H2'	19:I:1683:U:C6	2.53	0.44
19:I:2163:A:H5"	19:I:2170:A:O2'	2.16	0.44
47:W:29:HIS:HB3	47:W:36:TYR:CD1	2.52	0.44
1:C:72:G:H2'	1:C:90:G:N2	2.33	0.44
1:C:198:G:N2	1:C:199:A:N3	2.65	0.44
1:C:651:U:H2'	1:C:652:A:H8	1.83	0.44
1:C:1268:G:O2'	1:C:1269:A:H8	2.00	0.44
1:C:1324:U:H2'	1:C:1325:G:O4'	2.18	0.44
1:C:1447:A:H2'	1:C:1448:G:O4'	2.18	0.44
19:I:1508:A:H1'	19:I:1509:A:C8	2.52	0.44
19:I:2804:U:H2'	19:I:2805:C:H6	1.81	0.44
36:q:28:THR:O	36:q:28:THR:HG22	2.18	0.44
36:q:86:ALA:O	36:q:87:LEU:C	2.60	0.44
47:W:52:SER:O	47:W:58:ILE:HD13	2.18	0.44
3:1:45:ILE:HD12	3:1:45:ILE:H	1.83	0.44
11:E:144:LEU:O	11:E:144:LEU:HD23	2.17	0.44
19:I:2128:G:H2'	19:I:2129:C:C6	2.52	0.44
19:I:2344:U:H5"	19:I:2373:G:H4'	1.99	0.44
19:I:2813:A:H2'	19:I:2814:A:C8	2.52	0.44

Continued on next page...

For Manuscript Review

Continued from previous page...

Atom-1	Atom-2	Interatomic distance (Å)	Clash overlap (Å)
34:p:36:GLU:OE2	34:p:45:ARG:NE	2.51	0.44
39:S:38:ILE:HD11	39:S:112:PHE:HZ	1.82	0.44
47:W:30:ARG:HB3	47:W:97:PHE:CG	2.53	0.44
1:C:1020:G:C5	1:C:1021:C:C5	3.06	0.44
1:C:1032:U:C2	1:C:1033:C:C5	3.06	0.44
1:C:1445:C:C2'	1:C:1446:A:C8	3.01	0.44
9:D:104:TRP:HE1	9:D:108:ARG:NH2	2.16	0.44
13:F:121:LYS:HG2	13:F:131:ASN:OD1	2.18	0.44
19:I:414:C:H2'	19:I:415:A:C8	2.53	0.44
19:I:2896:C:C3'	19:I:2897:U:H5'	2.48	0.44
32:o:11:LEU:HD22	32:o:75:ILE:HD11	1.98	0.44
36:q:45:ARG:HG3	36:q:46:LYS:N	2.32	0.44
44:v:18:ASP:HA	44:v:22:ALA:HB2	2.00	0.44
47:W:83:LEU:CD1	47:W:88:LYS:HB3	2.47	0.44
47:W:94:ARG:HD2	47:W:99:TYR:HB2	1.99	0.44
19:I:882:G:H1	19:I:895:U:H1'	1.82	0.44
19:I:2126:A:N3	19:I:2127:G:H1'	2.33	0.44
19:I:2514:U:H2'	19:I:2515:C:C6	2.53	0.44
30:n:132:GLY:O	30:n:134:ALA:N	2.50	0.44
32:o:77:ARG:NE	32:o:79:SER:O	2.51	0.44
34:p:93:SER:C	34:p:94:LEU:HD12	2.43	0.44
35:Q:35:ASP:OD1	35:Q:36:VAL:N	2.50	0.44
38:r:79:ILE:HB	38:r:105:PHE:HE1	1.83	0.44
1:C:1004:C:H2'	1:C:1005:C:C6	2.53	0.43
15:G:115:LEU:HD13	15:G:123:VAL:HG11	2.00	0.43
19:I:593:U:H2'	19:I:594:U:C6	2.54	0.43
36:q:27:GLU:OE2	36:q:31:ARG:HB2	2.18	0.43
43:U:1:MET:HE1	43:U:44:ARG:HD3	2.00	0.43
1:C:331:G:H2'	1:C:332:A:H8	1.82	0.43
1:C:939:G:C2	1:C:940:A:C8	3.06	0.43
13:F:35:GLU:HG2	13:F:36:GLN:HG3	2.00	0.43
19:I:2155:U:H3'	19:I:2156:G:C8	2.54	0.43
34:p:27:LYS:HB3	34:p:62:ASP:OD2	2.19	0.43
36:q:82:LYS:H	36:q:82:LYS:CE	2.31	0.43
36:q:85:ASP:HA	36:q:88:MET:HE1	2.01	0.43
1:C:1049:A:C2'	11:E:156:ARG:HH21	2.30	0.43
7:b:67:VAL:CG1	7:b:74:ILE:HD11	2.48	0.43
18:h:25:LEU:HD21	19:I:930:G:H1'	2.00	0.43
19:I:2152:G:O2'	19:I:2153:C:O4'	2.36	0.43
27:M:121:VAL:O	27:M:189:THR:HA	2.17	0.43
33:P:5:LEU:O	33:P:15:LEU:HD12	2.18	0.43

Continued on next page...

For Manuscript Review

Continued from previous page...

Atom-1	Atom-2	Interatomic distance (Å)	Clash overlap (Å)
33:P:97:ARG:NE	33:P:112:LYS:HD3	2.33	0.43
38:r:113:VAL:O	38:r:113:VAL:HG12	2.18	0.43
19:I:276:U:O3'	19:I:277:G:H4'	2.18	0.43
21:J:13:G:O2'	21:J:15:A:OP2	2.30	0.43
31:O:38:ASN:OD1	31:O:39:ASP:N	2.51	0.43
31:O:52:PHE:CD1	31:O:69:ARG:HG2	2.53	0.43
38:r:84:VAL:HG11	38:r:97:ILE:CD1	2.49	0.43
44:v:43:ASN:HA	44:v:46:LEU:HB3	1.99	0.43
47:W:11:ALA:HA	47:W:96:GLY:N	2.33	0.43
1:C:1075:G:H21	15:G:52:LYS:HE2	1.83	0.43
1:C:1144:U:O4	1:C:1145:A:N6	2.52	0.43
19:I:279:A:N6	19:I:361:G:H1'	2.34	0.43
19:I:1084:A:H2'	19:I:1085:A:C8	2.54	0.43
19:I:2133:G:H2'	19:I:2157:G:N1	2.34	0.43
19:I:2755:C:H3'	28:m:19:ARG:NH1	2.33	0.43
31:O:127:THR:HG22	31:O:128:GLN:N	2.34	0.43
1:C:668:G:H2'	1:C:669:A:C8	2.54	0.43
1:C:1299:G:HO2'	1:C:1300:A:H8	1.65	0.43
1:C:1440:A:HO2'	1:C:1441:A:P	2.41	0.43
19:I:1064:C:H2'	19:I:1065:U:C6	2.54	0.43
23:K:227:PRO:HA	23:K:233:GLY:HA2	2.00	0.43
38:r:52:PHE:O	38:r:53:ARG:HD2	2.17	0.43
41:T:10:GLU:H	41:T:10:GLU:CD	2.27	0.43
47:W:30:ARG:HB3	47:W:97:PHE:CD1	2.53	0.43
47:W:56:LYS:HD2	47:W:60:GLU:HB3	1.99	0.43
51:Y:30:ARG:HG3	53:Y:201:MG:MG	1.44	0.43
17:H:88:MET:HE2	17:H:90:MET:CE	2.49	0.43
19:I:500:G:N1	19:I:503:A:OP2	2.46	0.43
19:I:1120:G:N1	19:I:1121:C:C2	2.87	0.43
19:I:1509:A:O2'	19:I:1510:G:O4'	2.36	0.43
19:I:1538:G:H2'	19:I:1539:U:C6	2.54	0.43
25:L:28:GLU:O	25:L:28:GLU:HG3	2.18	0.43
29:N:123:ASP:OD1	29:N:123:ASP:N	2.44	0.43
30:n:93:PRO:HA	30:n:96:ARG:CG	2.49	0.43
31:O:126:PRO:O	31:O:127:THR:OG1	2.31	0.43
33:P:31:VAL:HA	33:P:36:ALA:O	2.18	0.43
33:P:100:ALA:HB3	33:P:112:LYS:HG2	2.00	0.43
15:G:108:GLY:O	15:G:112:ARG:HG3	2.19	0.43
19:I:918:A:O2'	19:I:919:U:O5'	2.36	0.43
19:I:2813:A:H2'	19:I:2814:A:H8	1.83	0.43
26:l:31:HIS:ND1	26:l:32:ILE:HG13	2.34	0.43

Continued on next page...

For Manuscript Review

Continued from previous page...

Atom-1	Atom-2	Interatomic distance (Å)	Clash overlap (Å)
26:l:32:ILE:O	26:l:32:ILE:HG22	2.17	0.43
33:P:117:LEU:HD22	33:P:120:GLY:O	2.18	0.43
44:v:88:ALA:HB2	44:v:96:LEU:HD23	2.01	0.43
48:x:18:GLN:HE21	48:x:18:GLN:HB3	1.55	0.43
1:C:368:A:H5”	1:C:446:A:C2	2.54	0.43
9:D:15:HIS:HB3	9:D:43:LEU:HD11	2.01	0.43
13:F:59:GLN:OE1	13:F:62:ARG:NH1	2.52	0.43
13:F:116:GLN:HE21	13:F:120:HIS:CE1	2.37	0.43
19:I:172:A:H2’	19:I:173:A:C8	2.54	0.43
19:I:2102:G:H3’	19:I:2103:C:H6	1.83	0.43
27:M:6:LYS:HB2	27:M:119:ILE:HG23	2.00	0.43
1:C:74:A:H3’	1:C:75:G:H8	1.82	0.43
1:C:759:G:C5	1:C:806:G:C5	3.07	0.43
1:C:1116:U:O4	1:C:1117:A:N6	2.51	0.43
6:3:10:GLU:CD	6:3:18:ARG:HH12	2.27	0.43
19:I:2125:G:N1	19:I:2170:A:H5”	2.33	0.43
19:I:2135:A:H61	19:I:2156:G:H1’	1.83	0.43
19:I:2380:C:H2’	19:I:2381:A:C5’	2.48	0.43
19:I:2584:U:H2’	19:I:2585:U:H2’	2.01	0.43
21:J:28:C:OP2	47:W:33:ARG:NH1	2.51	0.43
23:K:72:ASP:HA	23:K:119:GLY:HA2	2.00	0.43
35:Q:22:MET:HG2	35:Q:24:ILE:HG12	2.01	0.43
36:q:48:ARG:HD3	36:q:66:GLU:HB2	1.99	0.43
42:u:71:ARG:O	42:u:75:MET:HG2	2.17	0.43
1:C:319:A:C6	1:C:320:G:C2	3.06	0.42
1:C:998:A:C5	1:C:1020:G:H1’	2.54	0.42
1:C:1242:A:H2’	1:C:1243:C:C6	2.54	0.42
1:C:1398:C:H2’	1:C:1399:G:C8	2.54	0.42
9:D:113:ARG:NH1	9:D:117:LEU:HB2	2.34	0.42
19:I:290:U:O2	19:I:350:G:N2	2.34	0.42
23:K:180:GLU:HG3	23:K:269:ARG:O	2.19	0.42
29:N:8:TYR:HA	29:N:12:VAL:HB	2.01	0.42
32:o:39:VAL:O	32:o:43:GLU:HG2	2.19	0.42
35:Q:20:ASN:OD1	35:Q:37:CYS:SG	2.75	0.42
47:W:3:LYS:O	47:W:7:ARG:HB2	2.19	0.42
1:C:31:G:O2’	1:C:48:C:N4	2.52	0.42
19:I:288:U:H2’	19:I:289:G:H8	1.82	0.42
19:I:365:U:H2’	19:I:366:C:C6	2.55	0.42
19:I:2163:A:H3’	19:I:2164:C:C5’	2.49	0.42
23:K:132:MET:HG2	23:K:188:CYS:O	2.18	0.42
33:P:108:VAL:O	33:P:110:VAL:HG23	2.19	0.42

Continued on next page...

For Manuscript Review

Continued from previous page...

Atom-1	Atom-2	Interatomic distance (Å)	Clash overlap (Å)
36:q:22:THR:OG1	36:q:23:ALA:N	2.51	0.42
44:v:11:VAL:HA	44:v:14:VAL:HG12	2.01	0.42
1:C:717:U:H4'	1:C:718:G:OP2	2.20	0.42
1:C:1443:C:H2'	1:C:1444:G:O4'	2.19	0.42
5:2:4:ILE:O	5:2:5:LYS:HB2	2.19	0.42
19:I:2144:G:HO2'	19:I:2145:C:P	2.41	0.42
19:I:2291:U:H2'	19:I:2292:U:C6	2.55	0.42
19:I:2334:U:C5	47:W:16:ARG:HG3	2.54	0.42
19:I:2367:G:OP2	19:I:2381:A:N6	2.52	0.42
25:L:99:GLU:OE2	25:L:182:ALA:HB2	2.19	0.42
30:n:65:ALA:C	30:n:67:GLU:H	2.27	0.42
31:O:68:ALA:O	31:O:72:LEU:HD13	2.19	0.42
36:q:34:ALA:HB3	36:q:78:GLU:HB3	1.99	0.42
3:1:44:MET:O	3:1:47:LEU:HD23	2.20	0.42
9:D:13:GLY:H	9:D:15:HIS:CE1	2.37	0.42
11:E:58:GLU:O	11:E:64:ILE:HD12	2.20	0.42
19:I:1179:G:H3'	19:I:1180:U:H5"	2.01	0.42
19:I:1570:A:H2'	19:I:1571:A:C8	2.54	0.42
23:K:71:LYS:NZ	23:K:98:ASP:OD2	2.47	0.42
34:p:86:ALA:HA	34:p:89:GLU:OE2	2.19	0.42
36:q:86:ALA:O	36:q:89:ARG:N	2.51	0.42
1:C:1004:C:H2'	1:C:1005:C:H6	1.84	0.42
1:C:1046:U:O2'	1:C:1049:A:OP2	2.29	0.42
11:E:134:MET:HE2	11:E:168:TYR:HD2	1.84	0.42
17:H:45:ARG:HH11	17:H:45:ARG:HB3	1.84	0.42
19:I:1071:G:H4'	19:I:1088:A:O2'	2.19	0.42
19:I:2030:6MZ:H2	19:I:2499:C:H5"	2.01	0.42
22:j:37:LYS:HD3	22:j:46:HIS:CB	2.49	0.42
51:Y:48:ARG:HH21	51:Y:49:ASP:CG	2.27	0.42
1:C:198:G:N2	1:C:199:A:H1'	2.35	0.42
1:C:546:U:C2	1:C:547:A:C8	3.08	0.42
1:C:998:A:N6	1:C:1020:G:H1'	2.33	0.42
1:C:1143:C:H2'	1:C:1144:U:C6	2.55	0.42
19:I:639:U:H2'	19:I:640:C:C6	2.54	0.42
19:I:1032:A:N1	19:I:1034:G:C8	2.88	0.42
19:I:1120:G:C6	19:I:1121:C:C4	3.07	0.42
19:I:1386:C:H2'	19:I:1387:A:H8	1.85	0.42
19:I:1913:A:O2'	19:I:1914:C:H2'	2.19	0.42
47:W:107:ALA:HB1	47:W:117:PHE:CE1	2.54	0.42
47:W:107:ALA:O	47:W:111:ARG:N	2.51	0.42
19:I:1176:U:H2'	19:I:1177:G:C8	2.54	0.42

Continued on next page...

For Manuscript Review

Continued from previous page...

Atom-1	Atom-2	Interatomic distance (Å)	Clash overlap (Å)
19:I:2138:G:H2'	19:I:2139:U:C6	2.54	0.42
1:C:836:U:C5	1:C:838:G:C2	3.07	0.42
1:C:836:U:C5	1:C:838:G:N1	2.88	0.42
1:C:1003:U:H4'	1:C:1004:C:OP2	2.20	0.42
9:D:68:LEU:HA	9:D:90:PHE:O	2.19	0.42
10:d:77:VAL:HG23	10:d:89:ILE:HG12	2.02	0.42
13:F:72:PHE:HE1	13:F:94:LEU:HD11	1.85	0.42
19:I:191:A:H2'	19:I:192:C:C6	2.55	0.42
19:I:620:G:N3	19:I:620:G:H5'	2.35	0.42
19:I:1539:U:H2'	19:I:1540:G:H8	1.83	0.42
22:j:43:VAL:HA	26:l:34:THR:HA	2.00	0.42
30:n:16:PRO:CG	34:p:43:THR:HG22	2.45	0.42
32:o:96:MET:SD	32:o:130:ALA:HB1	2.60	0.42
43:U:42:THR:HG22	43:U:43:ALA:N	2.35	0.42
47:W:18:LEU:HD22	47:W:93:ASP:HB2	2.01	0.42
1:C:714:C:O2	52:z:60:LYS:NZ	2.48	0.42
1:C:717:U:O4	6:3:53:VAL:HG22	2.20	0.42
1:C:1029:A:H2'	1:C:1030:A:H8	1.84	0.42
2:0:14:ALA:O	2:0:18:ARG:HG3	2.20	0.42
19:I:279:A:H61	19:I:361:G:H1'	1.85	0.42
19:I:360:U:H2'	19:I:361:G:C8	2.54	0.42
19:I:542:C:H2'	19:I:543:G:H8	1.85	0.42
19:I:1106:G:H2'	19:I:1107:G:H8	1.83	0.42
19:I:1266:G:OP1	20:i:16:ARG:NE	2.49	0.42
19:I:1902:C:H4'	23:K:242:LYS:O	2.20	0.42
33:P:12:LEU:HD22	33:P:25:TYR:CE2	2.55	0.42
33:P:135:HIS:HB3	33:P:138:VAL:HG22	2.01	0.42
34:p:22:LYS:HE3	34:p:61:LEU:HD22	2.02	0.42
36:q:23:ALA:O	36:q:26:VAL:HG13	2.20	0.42
41:T:77:ILE:CD1	41:T:108:ALA:HB1	2.48	0.42
44:v:43:ASN:O	44:v:47:LYS:HG2	2.20	0.42
47:W:11:ALA:HA	47:W:96:GLY:CA	2.50	0.42
1:C:672:U:H2'	1:C:673:U:H6	1.84	0.42
19:I:896:A:H5"	19:I:897:C:C5	2.54	0.42
19:I:2557:G:H2'	19:I:2558:C:C6	2.55	0.42
38:r:65:VAL:O	38:r:68:GLU:HG2	2.20	0.42
41:T:90:VAL:CG2	41:T:122:VAL:HA	2.49	0.42
43:U:26:VAL:HA	43:U:104:GLU:OE2	2.20	0.42
1:C:465:U:H2'	1:C:466:U:H6	1.85	0.41
1:C:715:A:H4'	1:C:716:G:O5'	2.19	0.41
1:C:1058:G:N2	1:C:1184:G:H2'	2.35	0.41

Continued on next page...

For Manuscript Review

Continued from previous page...

Atom-1	Atom-2	Interatomic distance (Å)	Clash overlap (Å)
1:C:1306:G:OP2	35:Q:63:ARG:NH2	2.50	0.41
8:c:96:PHE:O	8:c:100:SER:HA	2.20	0.41
19:I:651:G:H5’	26:l:19:LYS:HG3	2.02	0.41
19:I:2134:A:N6	19:I:2157:G:O4’	2.53	0.41
19:I:2182:U:H2’	19:I:2183:A:N3	2.35	0.41
19:I:2306:C:N3	19:I:2307:G:C2	2.88	0.41
22:j:10:LYS:HG2	22:j:20:PHE:HB2	2.01	0.41
22:j:12:VAL:HG12	22:j:51:GLU:O	2.20	0.41
33:P:48:GLU:HA	33:P:51:ARG:HB2	2.03	0.41
47:W:75:GLY:HA2	47:W:78:VAL:HG12	2.01	0.41
1:C:263:U:H2’	1:C:264:A:C8	2.56	0.41
19:I:1070:A:C8	19:I:1097:U:H4’	2.55	0.41
19:I:1868:C:H6	19:I:1868:C:O5’	2.03	0.41
19:I:2123:G:H2’	19:I:2124:G:C8	2.55	0.41
34:p:5:GLN:OE1	34:p:5:GLN:N	2.47	0.41
44:v:41:ARG:HD2	44:v:43:ASN:N	2.33	0.41
52:z:22:ASP:OD1	52:z:23:TYR:N	2.54	0.41
1:C:751:U:H2’	1:C:752:G:O4’	2.20	0.41
1:C:1136:G:H2’	1:C:1137:G:O4’	2.21	0.41
14:f:8:THR:OG1	14:f:10:LYS:HG3	2.19	0.41
19:I:994:C:H3’	51:Y:54:LYS:HZ2	1.84	0.41
19:I:2149:U:H2’	19:I:2150:C:C6	2.56	0.41
33:P:58:LEU:HA	33:P:61:VAL:HG12	2.01	0.41
34:p:51:PRO:HA	34:p:103:PHE:CZ	2.55	0.41
47:W:25:ARG:NH1	47:W:44:GLY:HA2	2.35	0.41
19:I:1118:C:H3’	19:I:1119:U:H4’	2.00	0.41
1:C:709:A:H2’	1:C:710:A:C8	2.56	0.41
1:C:820:C:O2	32:o:16:ASN:ND2	2.53	0.41
1:C:1065:C:H2’	1:C:1066:G:H8	1.85	0.41
8:c:52:LEU:C	8:c:54:GLN:H	2.27	0.41
13:F:65:TYR:OH	13:F:95:GLU:OE1	2.29	0.41
19:I:357:C:C2	19:I:358:U:C5	3.08	0.41
19:I:1070:A:H2’	19:I:1097:U:P	2.60	0.41
19:I:2112:G:OP1	19:I:2115:G:N2	2.54	0.41
22:j:39:PHE:CZ	22:j:44:ARG:HA	2.52	0.41
33:P:6:LEU:HG	33:P:36:ALA:HA	2.02	0.41
35:Q:14:ALA:HB1	35:Q:34:LEU:HD23	2.02	0.41
41:T:89:VAL:O	41:T:94:THR:HG21	2.20	0.41
47:W:23:ALA:C	47:W:42:PRO:HG3	2.45	0.41
1:C:41:G:H2’	1:C:42:G:C8	2.55	0.41
1:C:383:A:H3’	1:C:384:U:H6	1.85	0.41

Continued on next page...

For Manuscript Review

Continued from previous page...

Atom-1	Atom-2	Interatomic distance (Å)	Clash overlap (Å)
15:G:13:GLU:HB3	15:G:39:VAL:HG22	2.02	0.41
19:I:948:C:H1’	19:I:984:A:C8	2.56	0.41
19:I:2142:A:H2’	19:I:2143:C:C6	2.56	0.41
29:N:100:PHE:CE1	29:N:175:PHE:HZ	2.39	0.41
48:x:42:ILE:O	48:x:42:ILE:HG22	2.20	0.41
9:D:163:VAL:HG21	9:D:173:ILE:HD11	2.02	0.41
19:I:27:G:N2	19:I:512:G:H1’	2.35	0.41
19:I:1086:A:H3’	19:I:1086:A:N3	2.35	0.41
19:I:1119:U:C2	19:I:1120:G:N7	2.88	0.41
19:I:1548:A:H2’	19:I:1549:A:C8	2.56	0.41
19:I:1794:A:H2’	19:I:1795:C:C6	2.56	0.41
30:n:103:TRP:CH2	30:n:141:VAL:HG21	2.56	0.41
31:O:89:LEU:HD23	31:O:94:TYR:HB3	2.01	0.41
33:P:14:SER:HB3	33:P:17:ASP:OD2	2.20	0.41
40:t:24:LEU:HD22	40:t:29:GLN:HA	2.03	0.41
1:C:35:G:N3	40:t:115:SER:OG	2.54	0.41
1:C:412:U:H2’	1:C:413:C:C6	2.55	0.41
1:C:1170:A:H2’	1:C:1171:G:C8	2.55	0.41
8:c:49:VAL:HG23	8:c:52:LEU:HB3	2.03	0.41
19:I:1370:C:H2’	19:I:1371:G:O4’	2.21	0.41
22:j:10:LYS:CG	22:j:20:PHE:HB2	2.50	0.41
23:K:246:THR:HG23	23:K:250:VAL:O	2.21	0.41
25:L:46:ARG:NH1	25:L:85:ALA:O	2.47	0.41
34:p:22:LYS:C	34:p:61:LEU:HB3	2.46	0.41
39:S:104:THR:HG22	39:S:105:ARG:N	2.35	0.41
1:C:74:A:H3’	1:C:75:G:C8	2.56	0.41
1:C:85:U:H2’	1:C:86:C:H6	1.86	0.41
1:C:293:G:H2’	1:C:294:A:C8	2.56	0.41
1:C:464:C:H2’	1:C:465:U:H6	1.86	0.41
1:C:490:A:O2’	1:C:491:U:OP1	2.36	0.41
1:C:518:G:H2’	1:C:519:C:C6	2.56	0.41
11:E:35:SER:OG	11:E:59:ARG:NH2	2.54	0.41
11:E:70:THR:HG21	11:E:76:VAL:HG21	2.03	0.41
13:F:151:LYS:O	13:F:151:LYS:HG3	2.20	0.41
19:I:634:C:H2’	19:I:635:C:C6	2.56	0.41
19:I:866:A:C8	19:I:914:G:C6	3.09	0.41
19:I:1070:A:H8	19:I:1097:U:OP1	2.04	0.41
19:I:1079:C:H41	19:I:1088:A:P	2.44	0.41
19:I:1484:U:H2’	19:I:1485:U:C6	2.55	0.41
19:I:2171:A:H3’	19:I:2173:A:C8	2.56	0.41
19:I:2185:U:C2	19:I:2186:G:C8	3.09	0.41

Continued on next page...

For Manuscript Review

Continued from previous page...

Atom-1	Atom-2	Interatomic distance (Å)	Clash overlap (Å)
19:I:2347:C:C2	19:I:2371:G:N2	2.88	0.41
25:L:175:LEU:HD22	25:L:190:LYS:O	2.21	0.41
30:n:70:ARG:HH12	30:n:97:ASN:HB3	1.86	0.41
33:P:6:LEU:N	33:P:35:LYS:O	2.49	0.41
34:p:48:VAL:O	34:p:51:PRO:HD2	2.21	0.41
36:q:71:LEU:HD23	36:q:72:ARG:N	2.35	0.41
38:r:22:HIS:HB2	38:r:33:THR:HG23	2.02	0.41
41:T:75:ALA:HB2	41:T:105:ILE:HG21	2.03	0.41
45:V:56:LYS:HE3	45:V:88:ALA:HA	2.03	0.41
1:C:494:G:H2'	1:C:495:C:C6	2.56	0.41
1:C:730:C:H2'	1:C:731:A:C8	2.56	0.41
3:1:20:GLU:O	3:1:23:VAL:HG12	2.21	0.41
12:e:44:LYS:NZ	19:I:2331:G:P	2.94	0.41
17:H:45:ARG:HB3	17:H:45:ARG:NH1	2.35	0.41
17:H:90:MET:HE3	52:z:23:TYR:OH	2.21	0.41
19:I:1796:U:H2'	19:I:1797:G:C8	2.55	0.41
19:I:1866:A:N7	19:I:1867:G:H1'	2.36	0.41
19:I:2181:U:H2'	19:I:2182:U:C6	2.56	0.41
42:u:68:ASP:OD1	42:u:69:LEU:N	2.54	0.41
47:W:34:HIS:ND1	47:W:53:THR:HG23	2.35	0.41
50:y:15:ASP:OD2	50:y:54:GLY:HA2	2.21	0.41
1:C:1114:C:H2'	1:C:1115:U:C6	2.57	0.40
1:C:1268:G:HO2'	1:C:1269:A:H8	1.69	0.40
11:E:6:HIS:HE1	11:E:8:ASN:HB3	1.85	0.40
19:I:1889:A:H2'	19:I:1890:A:C8	2.56	0.40
19:I:2182:U:C2	19:I:2183:A:H2	2.40	0.40
19:I:2374:C:H2'	19:I:2375:G:C8	2.55	0.40
23:K:203[B]:ARG:HG2	23:K:204:VAL:N	2.36	0.40
33:P:6:LEU:HB2	33:P:35:LYS:C	2.46	0.40
1:C:198:G:N1	1:C:459:A:N6	2.69	0.40
5:2:4:ILE:HD12	5:2:4:ILE:H	1.86	0.40
19:I:1469:A:H2'	19:I:1470:A:C8	2.56	0.40
19:I:1607:C:N4	19:I:1622:G:OP2	2.53	0.40
19:I:1913:A:N7	19:I:1915:3TD:C6	2.68	0.40
19:I:2172:U:H3'	19:I:2173:A:C5'	2.51	0.40
34:p:8:GLY:HA3	34:p:86:ALA:HB2	2.03	0.40
37:R:40:HIS:NE2	37:R:41:LYS:HE3	2.36	0.40
38:r:19:GLY:C	38:r:82:LEU:HD12	2.46	0.40
47:W:94:ARG:HD2	47:W:99:TYR:CD1	2.56	0.40
1:C:319:A:H2	1:C:320:G:H2'	1.85	0.40
1:C:465:U:H2'	1:C:466:U:C6	2.57	0.40

Continued on next page...

For Manuscript Review

Continued from previous page...

Atom-1	Atom-2	Interatomic distance (Å)	Clash overlap (Å)
1:C:466:U:C2	1:C:467:G:C2	3.09	0.40
1:C:1194:C:H5"	1:C:1195:A:H3'	2.03	0.40
1:C:1317:G:H2'	1:C:1318:A:C8	2.56	0.40
8:c:82:ARG:NH2	19:I:300:A:OP2	2.53	0.40
10:d:21:ARG:NE	10:d:87:GLN:HB2	2.36	0.40
11:E:11:ARG:NH1	11:E:177:THR:O	2.51	0.40
14:f:37:ARG:HG2	14:f:46:PHE:HB3	2.04	0.40
19:I:279:A:C2	19:I:362:A:H4'	2.56	0.40
19:I:851:C:H2'	19:I:852:U:C6	2.57	0.40
19:I:857:G:H2'	19:I:858:G:O4'	2.22	0.40
19:I:2021:C:OP1	20:i:9:THR:HG21	2.21	0.40
21:J:60:C:H2'	21:J:61:G:H8	1.86	0.40
29:N:44:ILE:HG21	29:N:79:ILE:HG22	2.03	0.40
30:n:130:ASN:HA	30:n:135:VAL:HG21	2.02	0.40
36:q:92:LEU:H	36:q:92:LEU:HD22	1.85	0.40
38:r:67:ALA:HB2	38:r:96:THR:HG23	2.03	0.40
39:S:106:GLU:OE1	39:S:106:GLU:N	2.45	0.40
47:W:28:VAL:HB	47:W:93:ASP:O	2.20	0.40
47:W:35:ILE:O	47:W:53:THR:HG22	2.20	0.40
1:C:395:C:O2'	1:C:615:A:N3	2.50	0.40
1:C:532:A:OP1	40:t:110:ARG:HD2	2.22	0.40
3:1:45:ILE:HA	3:1:62:VAL:HG13	2.04	0.40
11:E:15:VAL:HG23	11:E:16:LYS:HG2	2.03	0.40
19:I:760:G:H2'	19:I:761:A:O4'	2.22	0.40
19:I:1070:A:O2'	19:I:1097:U:H5'	2.22	0.40
19:I:1858:A:H2'	19:I:1859:U:O4'	2.21	0.40
36:q:83:THR:OG1	36:q:84:VAL:N	2.53	0.40
37:R:123:LYS:HE2	37:R:123:LYS:HB3	1.85	0.40
1:C:1023:U:H2'	1:C:1025:C:C4	2.56	0.40
1:C:1192:G:H2'	1:C:1193:U:C6	2.57	0.40
11:E:42:TYR:OH	11:E:90:VAL:HG11	2.22	0.40
16:g:6:LEU:CD2	16:g:53:VAL:HG22	2.52	0.40
19:I:479:A:H1'	19:I:480:A:H5"	2.03	0.40
19:I:742:A:H2'	19:I:743:A:C8	2.57	0.40
19:I:882:G:N1	19:I:895:U:H1'	2.37	0.40
19:I:1063:G:C6	19:I:1064:C:C4	3.09	0.40
19:I:1617:C:O3'	19:I:1618:6MZ:P	2.79	0.40
36:q:42:LEU:HD23	36:q:42:LEU:HA	1.93	0.40
36:q:78:GLU:OE1	36:q:79:PRO:N	2.50	0.40

There are no symmetry-related clashes.

For Manuscript Review

5.3 Torsion angles (i)

5.3.1 Protein backbone (i)

In the following table, the Percentiles column shows the percent Ramachandran outliers of the chain as a percentile score with respect to all PDB entries followed by that with respect to all EM entries.

The Analysed column shows the number of residues for which the backbone conformation was analysed, and the total number of residues.

Mol	Chain	Analysed	Favoured	Allowed	Outliers	Percentiles	
2	0	108/110 (98%)	106 (98%)	2 (2%)	0	100	100
3	1	79/81 (98%)	75 (95%)	3 (4%)	1 (1%)	10	33
4	Z	101/103 (98%)	98 (97%)	2 (2%)	1 (1%)	13	40
5	2	83/87 (95%)	78 (94%)	4 (5%)	1 (1%)	11	35
6	3	68/71 (96%)	68 (100%)	0	0	100	100
7	b	91/100 (91%)	87 (96%)	4 (4%)	0	100	100
8	c	100/104 (96%)	89 (89%)	10 (10%)	1 (1%)	13	40
9	D	216/241 (90%)	208 (96%)	7 (3%)	1 (0%)	25	56
10	d	92/94 (98%)	87 (95%)	5 (5%)	0	100	100
11	E	204/233 (88%)	195 (96%)	9 (4%)	0	100	100
12	e	73/85 (86%)	72 (99%)	1 (1%)	0	100	100
13	F	203/206 (98%)	192 (95%)	10 (5%)	1 (0%)	25	56
14	f	75/78 (96%)	75 (100%)	0	0	100	100
15	G	148/167 (89%)	138 (93%)	10 (7%)	0	100	100
16	g	60/63 (95%)	58 (97%)	2 (3%)	0	100	100
17	H	98/135 (73%)	93 (95%)	5 (5%)	0	100	100
18	h	56/59 (95%)	53 (95%)	3 (5%)	0	100	100
20	i	54/57 (95%)	53 (98%)	1 (2%)	0	100	100
22	j	48/55 (87%)	44 (92%)	3 (6%)	1 (2%)	5	22
23	K	270/273 (99%)	256 (95%)	14 (5%)	0	100	100
24	k	44/46 (96%)	43 (98%)	1 (2%)	0	100	100
25	L	207/209 (99%)	200 (97%)	7 (3%)	0	100	100
26	l	62/65 (95%)	58 (94%)	4 (6%)	0	100	100
27	M	199/201 (99%)	191 (96%)	8 (4%)	0	100	100
28	m	36/38 (95%)	35 (97%)	1 (3%)	0	100	100

Continued on next page...

For Manuscript Review

Continued from previous page...

Mol	Chain	Analysed	Favoured	Allowed	Outliers	Percentiles	
29	N	175/179 (98%)	170 (97%)	5 (3%)	0	100	100
30	n	149/179 (83%)	120 (80%)	27 (18%)	2 (1%)	10	33
31	O	174/177 (98%)	157 (90%)	15 (9%)	2 (1%)	12	37
32	o	127/130 (98%)	125 (98%)	2 (2%)	0	100	100
33	P	147/149 (99%)	144 (98%)	3 (2%)	0	100	100
34	p	125/130 (96%)	98 (78%)	22 (18%)	5 (4%)	2	10
35	Q	56/70 (80%)	56 (100%)	0	0	100	100
36	q	96/103 (93%)	75 (78%)	17 (18%)	4 (4%)	2	9
37	R	140/142 (99%)	137 (98%)	3 (2%)	0	100	100
38	r	115/129 (89%)	111 (96%)	4 (4%)	0	100	100
39	S	120/123 (98%)	117 (98%)	3 (2%)	0	100	100
40	t	121/124 (98%)	112 (93%)	9 (7%)	0	100	100
41	T	142/144 (99%)	135 (95%)	7 (5%)	0	100	100
42	u	113/118 (96%)	108 (96%)	5 (4%)	0	100	100
43	U	133/136 (98%)	128 (96%)	5 (4%)	0	100	100
44	v	92/101 (91%)	89 (97%)	3 (3%)	0	100	100
45	V	118/127 (93%)	115 (98%)	3 (2%)	0	100	100
46	w	86/89 (97%)	83 (96%)	3 (4%)	0	100	100
47	W	114/117 (97%)	103 (90%)	11 (10%)	0	100	100
48	x	80/82 (98%)	76 (95%)	2 (2%)	2 (2%)	4	18
49	X	112/115 (97%)	110 (98%)	2 (2%)	0	100	100
50	y	78/84 (93%)	74 (95%)	4 (5%)	0	100	100
51	Y	115/118 (98%)	115 (100%)	0	0	100	100
52	z	53/75 (71%)	53 (100%)	0	0	100	100
All	All	5556/5902 (94%)	5263 (95%)	271 (5%)	22 (0%)	32	60

All (22) Ramachandran outliers are listed below:

Mol	Chain	Res	Type
5	2	5	LYS
8	c	54	GLN
13	F	28	ILE
31	O	14	GLY
34	p	56	ASP

Continued on next page...

For Manuscript Review

Continued from previous page...

Mol	Chain	Res	Type
34	p	87	LEU
34	p	104	VAL
36	q	28	THR
36	q	57	VAL
48	x	45	GLU
9	D	14	VAL
30	n	117	ALA
48	x	53	ASP
3	1	82	GLY
36	q	38	GLY
31	O	13	ALA
34	p	62	ASP
30	n	36	LYS
34	p	25	ASN
36	q	79	PRO
4	Z	52	PRO
22	j	41	PRO

5.3.2 Protein sidechains (i)

In the following table, the Percentiles column shows the percent sidechain outliers of the chain as a percentile score with respect to all PDB entries followed by that with respect to all EM entries.

The Analysed column shows the number of residues for which the sidechain conformation was analysed, and the total number of residues.

Mol	Chain	Analysed	Rotameric	Outliers	Percentiles	
2	0	93/93 (100%)	93 (100%)	0	100	100
3	1	71/71 (100%)	70 (99%)	1 (1%)	62	86
4	Z	84/84 (100%)	84 (100%)	0	100	100
5	2	65/66 (98%)	65 (100%)	0	100	100
6	3	60/61 (98%)	60 (100%)	0	100	100
7	b	80/84 (95%)	80 (100%)	0	100	100
8	c	83/85 (98%)	83 (100%)	0	100	100
9	D	180/199 (90%)	180 (100%)	0	100	100
10	d	78/78 (100%)	78 (100%)	0	100	100
11	E	170/190 (90%)	170 (100%)	0	100	100
12	e	57/63 (90%)	57 (100%)	0	100	100

Continued on next page...

For Manuscript Review

Continued from previous page...

Mol	Chain	Analysed	Rotameric	Outliers	Percentiles	
13	F	172/173 (99%)	172 (100%)	0	100	100
14	f	67/68 (98%)	67 (100%)	0	100	100
15	G	113/126 (90%)	113 (100%)	0	100	100
16	g	54/55 (98%)	54 (100%)	0	100	100
17	H	87/116 (75%)	87 (100%)	0	100	100
18	h	48/49 (98%)	48 (100%)	0	100	100
20	i	47/48 (98%)	47 (100%)	0	100	100
22	j	45/49 (92%)	45 (100%)	0	100	100
23	K	217/218 (100%)	217 (100%)	0	100	100
24	k	38/38 (100%)	38 (100%)	0	100	100
25	L	164/164 (100%)	164 (100%)	0	100	100
26	l	51/52 (98%)	51 (100%)	0	100	100
27	M	165/165 (100%)	165 (100%)	0	100	100
28	m	34/34 (100%)	34 (100%)	0	100	100
29	N	148/150 (99%)	148 (100%)	0	100	100
30	n	124/147 (84%)	124 (100%)	0	100	100
31	O	137/138 (99%)	137 (100%)	0	100	100
32	o	104/105 (99%)	104 (100%)	0	100	100
33	P	114/114 (100%)	114 (100%)	0	100	100
34	p	105/107 (98%)	105 (100%)	0	100	100
35	Q	55/62 (89%)	55 (100%)	0	100	100
36	q	86/90 (96%)	43 (50%)	43 (50%)	0	0
37	R	116/116 (100%)	115 (99%)	1 (1%)	75	92
38	r	90/99 (91%)	90 (100%)	0	100	100
39	S	103/104 (99%)	103 (100%)	0	100	100
40	t	103/104 (99%)	103 (100%)	0	100	100
41	T	103/103 (100%)	103 (100%)	0	100	100
42	u	93/96 (97%)	93 (100%)	0	100	100
43	U	108/108 (100%)	108 (100%)	0	100	100
44	v	79/84 (94%)	79 (100%)	0	100	100
45	V	100/103 (97%)	100 (100%)	0	100	100

Continued on next page...

For Manuscript Review

Continued from previous page...

Mol	Chain	Analysed	Rotameric	Outliers	Percentiles	
46	w	75/77 (97%)	75 (100%)	0	100	100
47	W	86/87 (99%)	86 (100%)	0	100	100
48	x	65/65 (100%)	59 (91%)	6 (9%)	7	24
49	X	99/100 (99%)	99 (100%)	0	100	100
50	y	74/78 (95%)	74 (100%)	0	100	100
51	Y	89/90 (99%)	89 (100%)	0	100	100
52	z	48/65 (74%)	48 (100%)	0	100	100
All	All	4627/4821 (96%)	4576 (99%)	51 (1%)	69	90

All (51) residues with a non-rotameric sidechain are listed below:

Mol	Chain	Res	Type
3	1	81	ARG
36	q	7	ARG
36	q	8	ILE
36	q	9	ARG
36	q	10	LEU
36	q	14	ASP
36	q	16	ARG
36	q	17	LEU
36	q	18	ILE
36	q	24	GLU
36	q	25	ILE
36	q	26	VAL
36	q	27	GLU
36	q	30	LYS
36	q	31	ARG
36	q	35	GLN
36	q	37	ARG
36	q	40	ILE
36	q	47	GLU
36	q	51	VAL
36	q	53	ILE
36	q	60	ASP
36	q	67	ILE
36	q	69	THR
36	q	72	ARG
36	q	74	VAL
36	q	75	ASP
36	q	77	VAL

Continued on next page...

For Manuscript Review

Continued from previous page...

Mol	Chain	Res	Type
36	q	78	GLU
36	q	80	THR
36	q	81	GLU
36	q	82	LYS
36	q	83	THR
36	q	84	VAL
36	q	85	ASP
36	q	87	LEU
36	q	88	MET
36	q	89	ARG
36	q	91	ASP
36	q	92	LEU
36	q	96	VAL
36	q	99	GLN
36	q	100	ILE
36	q	102	LEU
37	R	128	ASN
48	x	18	GLN
48	x	35	ARG
48	x	46	LYS
48	x	47	GLU
48	x	48	GLU
48	x	51	ARG

Sometimes sidechains can be flipped to improve hydrogen bonding and reduce clashes. All (37) such sidechains are listed below:

Mol	Chain	Res	Type
9	D	58	ASN
9	D	89	GLN
11	E	8	ASN
12	e	50	ASN
13	F	100	ASN
15	G	61	GLN
16	g	58	ASN
17	H	11	HIS
17	H	46	GLN
18	h	20	HIS
23	K	15	HIS
23	K	53	HIS
23	K	70	ASN
23	K	134	ASN
25	L	32	ASN

Continued on next page...

For Manuscript Review

Continued from previous page...

Mol	Chain	Res	Type
25	L	67	HIS
25	L	130	GLN
25	L	173	GLN
27	M	165	HIS
28	m	13	ASN
29	N	5	HIS
29	N	52	ASN
31	O	30	ASN
32	o	4	GLN
33	P	73	ASN
34	p	110	GLN
36	q	35	GLN
36	q	56	HIS
37	R	76	HIS
42	u	52	GLN
44	v	4	GLN
44	v	60	GLN
47	W	100	HIS
47	W	104	GLN
48	x	18	GLN
50	y	9	GLN
51	Y	44	GLN

5.3.3 RNA (i)

Mol	Chain	Analysed	Backbone Outliers	Pucker Outliers
1	C	1525/1526 (99%)	278 (18%)	24 (1%)
19	I	2893/2904 (99%)	529 (18%)	15 (0%)
21	J	117/120 (97%)	14 (11%)	0
All	All	4535/4550 (99%)	821 (18%)	39 (0%)

All (821) RNA backbone outliers are listed below:

Mol	Chain	Res	Type
1	C	3	A
1	C	9	G
1	C	22	G
1	C	32	A
1	C	39	G
1	C	47	C
1	C	48	C

Continued on next page...

For Manuscript Review

Continued from previous page...

Mol	Chain	Res	Type
1	C	50	A
1	C	51	A
1	C	52	C
1	C	57	G
1	C	60	A
1	C	61	G
1	C	64	G
1	C	68	G
1	C	71	U
1	C	72	G
1	C	76	G
1	C	77	G
1	C	82	U
1	C	83	G
1	C	84	C
1	C	85	U
1	C	88	U
1	C	89	G
1	C	90	G
1	C	91	A
1	C	95	A
1	C	106	G
1	C	110	A
1	C	115	U
1	C	116	G
1	C	121	G
1	C	123	A
1	C	124	A
1	C	125	U
1	C	143	A
1	C	145	A
1	C	157	C
1	C	176	C
1	C	177	G
1	C	182	G
1	C	191	A
1	C	192	G
1	C	193	U
1	C	204	C
1	C	205	G
1	C	206	G
1	C	214	G

Continued on next page...

For Manuscript Review

Continued from previous page...

Mol	Chain	Res	Type
1	C	222	A
1	C	239	U
1	C	241	G
1	C	244	A
1	C	245	G
1	C	260	G
1	C	261	C
1	C	265	C
1	C	266	C
1	C	274	C
1	C	277	U
1	C	283	A
1	C	287	G
1	C	290	U
1	C	302	C
1	C	315	A
1	C	322	C
1	C	323	A
1	C	341	G
1	C	346	C
1	C	348	G
1	C	361	U
1	C	363	G
1	C	366	C
1	C	367	A
1	C	370	G
1	C	375	A
1	C	377	A
1	C	378	G
1	C	386	C
1	C	391	A
1	C	392	U
1	C	400	G
1	C	405	A
1	C	406	A
1	C	407	G
1	C	408	A
1	C	418	G
1	C	423	U
1	C	433	U
1	C	439	G
1	C	443	G

Continued on next page...

For Manuscript Review

Continued from previous page...

Mol	Chain	Res	Type
1	C	445	A
1	C	446	A
1	C	447	G
1	C	457	U
1	C	461	U
1	C	462	A
1	C	475	G
1	C	478	G
1	C	490	A
1	C	491	U
1	C	492	A
1	C	493	A
1	C	503	A
1	C	505	C
1	C	507	U
1	C	509	G
1	C	511	G
1	C	512	C
1	C	513	C
1	C	514	A
1	C	515	G
1	C	521	G
1	C	526	A
1	C	532	A
1	C	534	G
1	C	539	C
1	C	541	A
1	C	556	U
1	C	566	A
1	C	567	A
1	C	570	C
1	C	571	G
1	C	572	C
1	C	573	G
1	C	575	G
1	C	584	U
1	C	585	C
1	C	611	G
1	C	622	G
1	C	627	G
1	C	647	A
1	C	650	G

Continued on next page...

For Manuscript Review

Continued from previous page...

Mol	Chain	Res	Type
1	C	654	G
1	C	659	A
1	C	661	G
1	C	681	A
1	C	682	G
1	C	687	G
1	C	688	A
1	C	689	A
1	C	695	U
1	C	711	C
1	C	715	A
1	C	716	G
1	C	717	U
1	C	718	G
1	C	725	G
1	C	738	C
1	C	749	A
1	C	754	G
1	C	756	U
1	C	759	G
1	C	760	A
1	C	771	A
1	C	781	A
1	C	788	A
1	C	803	G
1	C	809	A
1	C	811	C
1	C	814	U
1	C	815	G
1	C	822	A
1	C	835	C
1	C	837	U
1	C	838	G
1	C	840	G
1	C	847	G
1	C	861	G
1	C	866	A
1	C	871	G
1	C	894	A
1	C	896	G
1	C	908	A
1	C	916	G

Continued on next page...

For Manuscript Review

Continued from previous page...

Mol	Chain	Res	Type
1	C	920	G
1	C	921	G
1	C	928	C
1	C	954	U
1	C	955	U
1	C	960	G
1	C	963	A
1	C	969	A
1	C	970	G
1	C	971	A
1	C	977	A
1	C	986	U
1	C	987	G
1	C	988	A
1	C	989	C
1	C	998	A
1	C	1000	C
1	C	1002	U
1	C	1003	U
1	C	1004	C
1	C	1015	U
1	C	1023	U
1	C	1025	C
1	C	1027	G
1	C	1031	C
1	C	1039	C
1	C	1044	G
1	C	1047	G
1	C	1049	A
1	C	1059	U
1	C	1075	G
1	C	1079	U
1	C	1080	U
1	C	1088	G
1	C	1089	U
1	C	1095	A
1	C	1119	U
1	C	1130	U
1	C	1131	C
1	C	1132	G
1	C	1133	G
1	C	1153	U

Continued on next page...

For Manuscript Review

Continued from previous page...

Mol	Chain	Res	Type
1	C	1162	C
1	C	1165	A
1	C	1175	G
1	C	1178	G
1	C	1190	A
1	C	1191	A
1	C	1194	C
1	C	1196	U
1	C	1206	U
1	C	1207	A
1	C	1221	A
1	C	1230	A
1	C	1232	A
1	C	1234	U
1	C	1235	G
1	C	1248	G
1	C	1250	U
1	C	1251	U
1	C	1252	G
1	C	1264	G
1	C	1266	U
1	C	1271	C
1	C	1273	A
1	C	1274	A
1	C	1281	A
1	C	1293	A
1	C	1294	G
1	C	1297	C
1	C	1299	G
1	C	1314	C
1	C	1332	G
1	C	1341	G
1	C	1342	U
1	C	1347	G
1	C	1364	G
1	C	1365	G
1	C	1372	C
1	C	1373	G
1	C	1388	A
1	C	1413	G
1	C	1423	C
1	C	1426	G

Continued on next page...

For Manuscript Review

Continued from previous page...

Mol	Chain	Res	Type
1	C	1440	A
1	C	1441	A
1	C	1444	G
1	C	1445	C
1	C	1446	A
1	C	1447	A
1	C	1448	G
1	C	1481	G
1	C	1491	G
1	C	1497	A
1	C	1499	G
1	C	1500	U
1	C	1501	A
1	C	1502	G
1	C	1511	G
1	C	1514	C
1	C	1523	G
1	C	1524	G
1	C	1525	A
19	I	2	G
19	I	10	A
19	I	15	G
19	I	34	U
19	I	35	G
19	I	36	G
19	I	46	G
19	I	51	G
19	I	60	G
19	I	63	A
19	I	71	A
19	I	74	A
19	I	75	G
19	I	84	A
19	I	88	G
19	I	100	U
19	I	101	A
19	I	118	A
19	I	119	A
19	I	120	U
19	I	125	A
19	I	136	G
19	I	139	U

Continued on next page...

For Manuscript Review

Continued from previous page...

Mol	Chain	Res	Type
19	I	140	C
19	I	149	A
19	I	160	A
19	I	163	C
19	I	165	A
19	I	181	A
19	I	196	A
19	I	199	A
19	I	201	C
19	I	215	G
19	I	216	A
19	I	218	A
19	I	221	A
19	I	222	A
19	I	224	U
19	I	225	C
19	I	227	A
19	I	230	G
19	I	233	A
19	I	245	G
19	I	248	G
19	I	249	C
19	I	264	C
19	I	265	A
19	I	266	G
19	I	271	G
19	I	272	A
19	I	273	G
19	I	277	G
19	I	278	A
19	I	279	A
19	I	284	U
19	I	285	G
19	I	290	U
19	I	291	G
19	I	300	A
19	I	311	A
19	I	330	A
19	I	338	G
19	I	353	C
19	I	359	G
19	I	364	C

Continued on next page...

For Manuscript Review

Continued from previous page...

Mol	Chain	Res	Type
19	I	371	A
19	I	372	G
19	I	386	G
19	I	396	G
19	I	403	U
19	I	404	A
19	I	411	G
19	I	412	A
19	I	418	C
19	I	420	C
19	I	457	A
19	I	473	G
19	I	481	G
19	I	491	G
19	I	492	A
19	I	503	A
19	I	504	A
19	I	505	A
19	I	508	A
19	I	509	C
19	I	532	A
19	I	535	G
19	I	543	G
19	I	544	C
19	I	546	U
19	I	547	A
19	I	548	G
19	I	549	G
19	I	550	C
19	I	563	A
19	I	571	U
19	I	573	U
19	I	575	A
19	I	602	A
19	I	603	A
19	I	607	U
19	I	613	A
19	I	615	U
19	I	622	G
19	I	627	A
19	I	637	A
19	I	645	C

Continued on next page...

For Manuscript Review

Continued from previous page...

Mol	Chain	Res	Type
19	I	647	G
19	I	653	U
19	I	654	A
19	I	655	A
19	I	686	U
19	I	716	A
19	I	717	C
19	I	730	A
19	I	736	C
19	I	746	PSU
19	I	747	5MU
19	I	764	A
19	I	765	C
19	I	770	G
19	I	774	G
19	I	775	G
19	I	776	G
19	I	782	A
19	I	784	G
19	I	785	G
19	I	789	A
19	I	792	A
19	I	794	A
19	I	805	G
19	I	806	C
19	I	812	C
19	I	819	A
19	I	827	U
19	I	846	U
19	I	858	G
19	I	859	G
19	I	881	G
19	I	884	U
19	I	894	U
19	I	895	U
19	I	896	A
19	I	897	C
19	I	910	A
19	I	915	C
19	I	918	A
19	I	919	U
19	I	932	U

Continued on next page...

For Manuscript Review

Continued from previous page...

Mol	Chain	Res	Type
19	I	946	C
19	I	961	C
19	I	974	G
19	I	975	A
19	I	983	A
19	I	988	A
19	I	990	A
19	I	996	A
19	I	997	G
19	I	999	U
19	I	1003	G
19	I	1005	C
19	I	1009	A
19	I	1011	G
19	I	1012	U
19	I	1013	C
19	I	1022	G
19	I	1027	A
19	I	1033	U
19	I	1034	G
19	I	1035	U
19	I	1036	G
19	I	1046	A
19	I	1047	G
19	I	1055	G
19	I	1057	A
19	I	1060	U
19	I	1061	U
19	I	1062	G
19	I	1063	G
19	I	1064	C
19	I	1065	U
19	I	1066	U
19	I	1067	A
19	I	1068	G
19	I	1069	A
19	I	1070	A
19	I	1071	G
19	I	1072	C
19	I	1073	A
19	I	1074	G
19	I	1076	C

Continued on next page...

For Manuscript Review

Continued from previous page...

Mol	Chain	Res	Type
19	I	1077	A
19	I	1079	C
19	I	1080	A
19	I	1081	U
19	I	1082	U
19	I	1084	A
19	I	1086	A
19	I	1087	G
19	I	1089	A
19	I	1090	A
19	I	1097	U
19	I	1098	A
19	I	1109	C
19	I	1110	G
19	I	1111	A
19	I	1112	G
19	I	1118	C
19	I	1119	U
19	I	1120	G
19	I	1121	C
19	I	1122	G
19	I	1132	U
19	I	1135	C
19	I	1136	G
19	I	1142	A
19	I	1150	C
19	I	1151	A
19	I	1157	G
19	I	1170	C
19	I	1171	G
19	I	1172	C
19	I	1173	U
19	I	1174	U
19	I	1175	A
19	I	1176	U
19	I	1179	G
19	I	1180	U
19	I	1181	U
19	I	1187	G
19	I	1206	G
19	I	1238	G
19	I	1250	G

Continued on next page...

For Manuscript Review

Continued from previous page...

Mol	Chain	Res	Type
19	I	1253	A
19	I	1256	G
19	I	1257	C
19	I	1266	G
19	I	1267	U
19	I	1271	G
19	I	1272	A
19	I	1273	U
19	I	1300	G
19	I	1301	A
19	I	1321	A
19	I	1341	G
19	I	1352	U
19	I	1365	A
19	I	1368	G
19	I	1374	G
19	I	1379	U
19	I	1383	A
19	I	1387	A
19	I	1395	A
19	I	1413	A
19	I	1416	G
19	I	1420	A
19	I	1421	G
19	I	1427	A
19	I	1428	C
19	I	1434	A
19	I	1452	G
19	I	1460	U
19	I	1461	C
19	I	1470	A
19	I	1478	G
19	I	1482	G
19	I	1490	A
19	I	1493	C
19	I	1494	A
19	I	1495	A
19	I	1515	A
19	I	1520	U
19	I	1523	U
19	I	1524	G
19	I	1526	C

Continued on next page...

For Manuscript Review

Continued from previous page...

Mol	Chain	Res	Type
19	I	1534	U
19	I	1535	A
19	I	1536	C
19	I	1537	G
19	I	1543	G
19	I	1560	G
19	I	1566	A
19	I	1567	G
19	I	1569	A
19	I	1578	U
19	I	1583	A
19	I	1584	U
19	I	1585	C
19	I	1608	A
19	I	1610	A
19	I	1618	6MZ
19	I	1644	C
19	I	1647	U
19	I	1648	U
19	I	1649	G
19	I	1654	A
19	I	1674	G
19	I	1675	C
19	I	1676	A
19	I	1693	U
19	I	1696	G
19	I	1702	G
19	I	1703	G
19	I	1715	G
19	I	1729	U
19	I	1730	C
19	I	1732	C
19	I	1733	G
19	I	1736	U
19	I	1737	G
19	I	1738	G
19	I	1739	A
19	I	1744	A
19	I	1750	G
19	I	1762	A
19	I	1763	G
19	I	1764	C

Continued on next page...

For Manuscript Review

Continued from previous page...

Mol	Chain	Res	Type
19	I	1769	U
19	I	1773	A
19	I	1776	G
19	I	1782	U
19	I	1784	A
19	I	1800	C
19	I	1801	A
19	I	1808	A
19	I	1816	C
19	I	1820	U
19	I	1829	A
19	I	1842	G
19	I	1862	G
19	I	1866	A
19	I	1867	G
19	I	1868	C
19	I	1869	G
19	I	1870	C
19	I	1871	A
19	I	1872	A
19	I	1873	G
19	I	1875	G
19	I	1903	G
19	I	1905	C
19	I	1906	G
19	I	1913	A
19	I	1929	G
19	I	1930	G
19	I	1931	U
19	I	1936	A
19	I	1945	G
19	I	1954	G
19	I	1955	U
19	I	1967	C
19	I	1970	A
19	I	1971	U
19	I	1972	G
19	I	1982	U
19	I	1987	A
19	I	1991	U
19	I	1993	U
19	I	1997	C

Continued on next page...

For Manuscript Review

Continued from previous page...

Mol	Chain	Res	Type
19	I	2006	C
19	I	2023	C
19	I	2026	U
19	I	2027	G
19	I	2031	A
19	I	2033	A
19	I	2036	C
19	I	2043	C
19	I	2052	A
19	I	2053	G
19	I	2055	C
19	I	2056	G
19	I	2059	A
19	I	2060	A
19	I	2061	G
19	I	2069	G7M
19	I	2093	G
19	I	2105	U
19	I	2107	G
19	I	2108	A
19	I	2110	G
19	I	2111	U
19	I	2112	G
19	I	2118	U
19	I	2119	A
19	I	2120	G
19	I	2122	U
19	I	2124	G
19	I	2125	G
19	I	2127	G
19	I	2131	U
19	I	2132	U
19	I	2133	G
19	I	2138	G
19	I	2141	G
19	I	2144	G
19	I	2145	C
19	I	2146	C
19	I	2148	G
19	I	2153	C
19	I	2155	U
19	I	2156	G

Continued on next page...

For Manuscript Review

Continued from previous page...

Mol	Chain	Res	Type
19	I	2158	A
19	I	2160	C
19	I	2161	C
19	I	2164	C
19	I	2165	C
19	I	2166	U
19	I	2168	G
19	I	2169	A
19	I	2170	A
19	I	2171	A
19	I	2172	U
19	I	2173	A
19	I	2174	C
19	I	2175	C
19	I	2178	C
19	I	2181	U
19	I	2186	G
19	I	2190	G
19	I	2198	A
19	I	2203	U
19	I	2204	G
19	I	2211	A
19	I	2220	U
19	I	2225	A
19	I	2226	C
19	I	2238	G
19	I	2239	G
19	I	2268	A
19	I	2273	A
19	I	2283	C
19	I	2287	A
19	I	2288	A
19	I	2294	G
19	I	2305	U
19	I	2309	A
19	I	2311	A
19	I	2321	U
19	I	2322	A
19	I	2333	A
19	I	2334	U
19	I	2335	A
19	I	2347	C

Continued on next page...

For Manuscript Review

Continued from previous page...

Mol	Chain	Res	Type
19	I	2350	C
19	I	2361	G
19	I	2366	A
19	I	2373	G
19	I	2374	C
19	I	2375	G
19	I	2376	A
19	I	2377	A
19	I	2379	G
19	I	2380	C
19	I	2381	A
19	I	2382	G
19	I	2383	G
19	I	2385	C
19	I	2390	U
19	I	2391	G
19	I	2396	G
19	I	2402	U
19	I	2406	A
19	I	2419	U
19	I	2423	U
19	I	2429	G
19	I	2430	A
19	I	2435	A
19	I	2440	C
19	I	2441	U
19	I	2448	A
19	I	2459	A
19	I	2469	A
19	I	2470	G
19	I	2473	U
19	I	2476	A
19	I	2491	U
19	I	2498	OMC
19	I	2502	G
19	I	2505	G
19	I	2518	A
19	I	2527	C
19	I	2529	G
19	I	2547	A
19	I	2554	U
19	I	2566	A

Continued on next page...

For Manuscript Review

Continued from previous page...

Mol	Chain	Res	Type
19	I	2567	G
19	I	2573	C
19	I	2582	G
19	I	2585	U
19	I	2586	U
19	I	2602	A
19	I	2609	U
19	I	2613	U
19	I	2615	U
19	I	2636	C
19	I	2661	G
19	I	2663	G
19	I	2675	A
19	I	2682	A
19	I	2689	U
19	I	2690	U
19	I	2702	G
19	I	2713	U
19	I	2714	G
19	I	2726	A
19	I	2733	A
19	I	2739	U
19	I	2748	A
19	I	2756	U
19	I	2757	A
19	I	2776	A
19	I	2778	A
19	I	2793	C
19	I	2797	U
19	I	2798	U
19	I	2800	A
19	I	2801	G
19	I	2809	A
19	I	2820	A
19	I	2837	A
19	I	2850	A
19	I	2873	A
19	I	2880	C
19	I	2884	U
19	I	2895	G
19	I	2897	U
19	I	2901	C

Continued on next page...

For Manuscript Review

Continued from previous page...

Mol	Chain	Res	Type
19	I	2902	C
19	I	2903	U
21	J	15	A
21	J	25	U
21	J	32	U
21	J	35	C
21	J	56	G
21	J	67	G
21	J	88	C
21	J	89	U
21	J	90	C
21	J	91	C
21	J	99	A
21	J	105	G
21	J	109	A
21	J	119	A

All (39) RNA pucker outliers are listed below:

Mol	Chain	Res	Type
1	C	87	C
1	C	90	G
1	C	245	G
1	C	265	C
1	C	322	C
1	C	405	A
1	C	442	A
1	C	491	U
1	C	506	U
1	C	512	C
1	C	717	U
1	C	893	C
1	C	954	U
1	C	976	U
1	C	1003	U
1	C	1195	A
1	C	1233	A
1	C	1247	G
1	C	1250	U
1	C	1251	U
1	C	1272	U
1	C	1273	A

Continued on next page...

For Manuscript Review

Continued from previous page...

Mol	Chain	Res	Type
1	C	1341	G
1	C	1440	A
19	I	784	G
19	I	918	A
19	I	1034	G
19	I	1035	U
19	I	1046	A
19	I	1070	A
19	I	1117	C
19	I	1542	U
19	I	1865	U
19	I	1867	G
19	I	1871	A
19	I	2170	A
19	I	2304	G
19	I	2308	G
19	I	2756	U

5.4 Non-standard residues in protein, DNA, RNA chains ⓘ

25 non-standard protein/DNA/RNA residues are modelled in this entry.

In the following table, the Counts columns list the number of bonds (or angles) for which Mogul statistics could be retrieved, the number of bonds (or angles) that are observed in the model and the number of bonds (or angles) that are defined in the Chemical Component Dictionary. The Link column lists molecule types, if any, to which the group is linked. The Z score for a bond length (or angle) is the number of standard deviations the observed value is removed from the expected value. A bond length (or angle) with $|Z| > 2$ is considered an outlier worth inspection. RMSZ is the root-mean-square of all Z scores of the bond lengths (or angles).

Mol	Type	Chain	Res	Link	Bond lengths			Bond angles		
					Counts	RMSZ	$\#\|Z\| > 2$	Counts	RMSZ	$\#\|Z\| > 2$
19	5MU	I	1939	19	19,22,23	1.32	4 (21%)	27,32,35	2.24	6 (22%)
19	H2U	I	2449	19	18,21,22	0.58	0	19,30,33	1.08	1 (5%)
19	6MZ	I	1618	19	17,25,26	1.37	3 (17%)	15,36,39	2.27	4 (26%)
19	PSU	I	2457	19	18,21,22	1.29	4 (22%)	21,30,33	2.17	4 (19%)
19	PSU	I	1911	19	18,21,22	1.07	2 (11%)	21,30,33	1.99	4 (19%)
19	G7M	I	2069	19	20,26,27	0.95	1 (5%)	16,39,42	0.64	0
19	2MG	I	2445	19	18,26,27	1.50	3 (16%)	16,38,41	1.63	4 (25%)
19	5MU	I	747	19	19,22,23	1.24	4 (21%)	27,32,35	2.25	7 (25%)
19	OMU	I	2552	19	19,22,23	1.26	3 (15%)	25,31,34	1.97	6 (24%)

For Manuscript Review

Mol	Type	Chain	Res	Link	Bond lengths			Bond angles		
					Counts	RMSZ	#|Z| > 2	Counts	RMSZ	#|Z| > 2
19	2MG	I	1835	19	18,26,27	1.44	3 (16%)	16,38,41	1.56	3 (18%)
19	PSU	I	2504	19,53	18,21,22	1.19	3 (16%)	21,30,33	1.95	4 (19%)
43	4D4	U	81	43	9,11,12	2.06	2 (22%)	7,13,15	1.92	3 (42%)
19	PSU	I	2580	19	18,21,22	1.43	4 (22%)	21,30,33	2.24	5 (23%)
19	PSU	I	1917	19	18,21,22	1.07	2 (11%)	21,30,33	1.97	4 (19%)
19	PSU	I	955	19	18,21,22	1.23	3 (16%)	21,30,33	1.98	4 (19%)
19	PSU	I	746	19	18,21,22	1.30	4 (22%)	21,30,33	1.95	3 (14%)
19	OMG	I	2251	19	19,26,27	1.40	3 (15%)	21,38,41	1.46	4 (19%)
19	OMC	I	2498	19	19,22,23	1.08	2 (10%)	25,31,34	1.23	2 (8%)
19	PSU	I	2604	19	18,21,22	1.23	3 (16%)	21,30,33	2.16	4 (19%)
19	6MZ	I	2030	19	17,25,26	1.41	3 (17%)	15,36,39	2.27	4 (26%)
19	1MG	I	745	19	19,26,27	1.53	3 (15%)	18,39,42	1.45	2 (11%)
19	PSU	I	2605	19	18,21,22	1.33	3 (16%)	21,30,33	2.00	4 (19%)
19	2MA	I	2503	19,53	18,25,26	1.34	3 (16%)	20,37,40	2.02	3 (15%)
19	3TD	I	1915	19	19,22,23	1.18	2 (10%)	23,32,35	1.56	4 (17%)
19	5MC	I	1962	19	19,22,23	1.74	2 (10%)	26,32,35	1.13	2 (7%)

In the following table, the Chirals column lists the number of chiral outliers, the number of chiral centers analysed, the number of these observed in the model and the number defined in the Chemical Component Dictionary. Similar counts are reported in the Torsion and Rings columns. '-' means no outliers of that kind were identified.

Mol	Type	Chain	Res	Link	Chirals	Torsions	Rings
19	5MU	I	1939	19	-	0/7/25/26	0/2/2/2
19	H2U	I	2449	19	-	0/7/38/39	0/2/2/2
19	6MZ	I	1618	19	-	3/5/27/28	0/3/3/3
19	PSU	I	2457	19	-	0/7/25/26	0/2/2/2
19	PSU	I	1911	19	-	0/7/25/26	0/2/2/2
19	G7M	I	2069	19	-	1/3/25/26	0/3/3/3
19	2MG	I	2445	19	-	0/5/27/28	0/3/3/3
19	5MU	I	747	19	-	0/7/25/26	0/2/2/2
19	OMU	I	2552	19	-	1/9/27/28	0/2/2/2
19	2MG	I	1835	19	-	0/5/27/28	0/3/3/3
19	PSU	I	2504	19,53	-	0/7/25/26	0/2/2/2
43	4D4	U	81	43	-	4/11/12/14	-
19	PSU	I	2580	19	-	2/7/25/26	0/2/2/2
19	PSU	I	1917	19	-	0/7/25/26	0/2/2/2
19	PSU	I	955	19	-	0/7/25/26	0/2/2/2

Continued on next page...

For Manuscript Review

Continued from previous page...

Mol	Type	Chain	Res	Link	Chirals	Torsions	Rings
19	PSU	I	746	19	-	3/7/25/26	0/2/2/2
19	OMG	I	2251	19	-	0/5/27/28	0/3/3/3
19	OMC	I	2498	19	-	2/9/27/28	0/2/2/2
19	PSU	I	2604	19	-	0/7/25/26	0/2/2/2
19	6MZ	I	2030	19	-	2/5/27/28	0/3/3/3
19	1MG	I	745	19	-	0/3/25/26	0/3/3/3
19	PSU	I	2605	19	-	0/7/25/26	0/2/2/2
19	2MA	I	2503	19,53	-	0/3/25/26	0/3/3/3
19	3TD	I	1915	19	-	0/7/25/26	0/2/2/2
19	5MC	I	1962	19	-	0/7/25/26	0/2/2/2

All (69) bond length outliers are listed below:

Mol	Chain	Res	Type	Atoms	Z	Observed(Å)	Ideal(Å)
19	I	1962	5MC	C5-C4	-6.08	1.39	1.44
43	U	81	4D4	CZ-NE	5.04	1.43	1.33
19	I	745	1MG	C8-N7	-3.56	1.29	1.34
19	I	2503	2MA	C6-N1	3.48	1.40	1.33
19	I	2445	2MG	C8-N7	-3.39	1.29	1.34
19	I	2445	2MG	C5-C6	-3.38	1.40	1.47
19	I	2251	OMG	C8-N7	-3.37	1.29	1.34
19	I	1835	2MG	C5-C6	-3.37	1.40	1.47
19	I	2251	OMG	C5-C6	-3.35	1.40	1.47
19	I	1835	2MG	C8-N7	-3.27	1.29	1.34
19	I	745	1MG	C6-N1	-3.25	1.33	1.39
19	I	1962	5MC	C2-N1	-3.23	1.33	1.40
19	I	1618	6MZ	C6-C5	-3.08	1.40	1.44
19	I	2552	OMU	C2-N1	-3.03	1.33	1.38
19	I	2030	6MZ	C6-C5	-3.02	1.40	1.44
19	I	1939	5MU	C2-N1	-2.97	1.33	1.38
19	I	2580	PSU	C2-N1	-2.88	1.32	1.36
19	I	2498	OMC	C2-N1	-2.81	1.34	1.40
19	I	2605	PSU	C4-N3	-2.80	1.33	1.38
43	U	81	4D4	CZ-NH1	2.77	1.44	1.34
19	I	2030	6MZ	C8-N7	-2.76	1.29	1.34
19	I	2605	PSU	C2-N3	-2.74	1.33	1.37
19	I	1618	6MZ	C8-N7	-2.67	1.29	1.34
19	I	746	PSU	C2-N3	-2.66	1.33	1.37
19	I	2580	PSU	O4'-C1'	-2.66	1.40	1.43
19	I	2580	PSU	C4-N3	-2.65	1.33	1.38
19	I	747	5MU	C2-N1	-2.63	1.34	1.38
19	I	2552	OMU	C4-N3	-2.62	1.34	1.38

Continued on next page...

For Manuscript Review

Continued from previous page...

Mol	Chain	Res	Type	Atoms	Z	Observed(Å)	Ideal(Å)
19	I	747	5MU	C4-C5	-2.60	1.40	1.44
19	I	2552	OMU	C2-N3	-2.59	1.33	1.38
19	I	2457	PSU	C4-N3	-2.59	1.34	1.38
19	I	2030	6MZ	C4-N3	-2.57	1.32	1.35
19	I	2457	PSU	C2-N1	-2.56	1.33	1.36
19	I	1939	5MU	C4-N3	-2.55	1.34	1.38
19	I	746	PSU	C4-N3	-2.54	1.34	1.38
19	I	1915	3TD	C4-N3	-2.54	1.35	1.40
19	I	1939	5MU	C4-C5	-2.53	1.40	1.44
19	I	2504	PSU	C4-N3	-2.53	1.34	1.38
19	I	2604	PSU	C2-N3	-2.53	1.33	1.37
19	I	2604	PSU	C4-N3	-2.52	1.34	1.38
19	I	746	PSU	O4'-C1'	-2.51	1.40	1.43
19	I	955	PSU	C4-N3	-2.48	1.34	1.38
19	I	1939	5MU	C2-N3	-2.48	1.33	1.38
19	I	2580	PSU	C2-N3	-2.47	1.33	1.37
19	I	2457	PSU	C2-N3	-2.45	1.33	1.37
19	I	1915	3TD	C4-C5	-2.45	1.42	1.47
19	I	1618	6MZ	C4-N3	-2.44	1.32	1.35
19	I	747	5MU	C4-N3	-2.43	1.34	1.38
19	I	2445	2MG	C6-N1	-2.39	1.34	1.37
19	I	2605	PSU	C2-N1	-2.38	1.33	1.36
19	I	2069	G7M	C8-N9	2.36	1.37	1.33
19	I	2504	PSU	C2-N3	-2.36	1.33	1.37
19	I	745	1MG	C5-C6	-2.35	1.41	1.47
19	I	2604	PSU	C2-N1	-2.34	1.33	1.36
19	I	2504	PSU	C2-N1	-2.34	1.33	1.36
19	I	955	PSU	C2-N1	-2.33	1.33	1.36
19	I	955	PSU	C2-N3	-2.30	1.33	1.37
19	I	2503	2MA	C2-N3	-2.27	1.30	1.34
19	I	2251	OMG	C6-N1	-2.26	1.34	1.37
19	I	1911	PSU	C4-N3	-2.23	1.34	1.38
19	I	747	5MU	C2-N3	-2.22	1.34	1.38
19	I	2503	2MA	C8-N7	-2.19	1.30	1.34
19	I	1835	2MG	C6-N1	-2.17	1.34	1.37
19	I	746	PSU	C2-N1	-2.10	1.33	1.36
19	I	1917	PSU	C4-N3	-2.09	1.34	1.38
19	I	1911	PSU	C2-N3	-2.09	1.34	1.37
19	I	1917	PSU	C2-N1	-2.08	1.34	1.36
19	I	2457	PSU	O4'-C1'	-2.04	1.41	1.43
19	I	2498	OMC	C2-N3	-2.01	1.32	1.36

All (91) bond angle outliers are listed below:

For Manuscript Review

Mol	Chain	Res	Type	Atoms	Z	Observed(°)	Ideal(°)
19	I	2580	PSU	N1-C2-N3	6.30	121.82	115.17
19	I	2457	PSU	N1-C2-N3	6.18	121.69	115.17
19	I	2503	2MA	C2-N3-C4	6.10	120.39	115.46
19	I	2604	PSU	N1-C2-N3	6.08	121.58	115.17
19	I	1939	5MU	C4-N3-C2	-5.82	119.71	127.34
19	I	746	PSU	N1-C2-N3	5.62	121.10	115.17
19	I	2605	PSU	N1-C2-N3	5.61	121.09	115.17
19	I	747	5MU	C4-N3-C2	-5.57	120.03	127.34
19	I	955	PSU	N1-C2-N3	5.53	121.00	115.17
19	I	2030	6MZ	C2-N1-C6	5.52	120.89	116.60
19	I	2504	PSU	N1-C2-N3	5.49	120.96	115.17
19	I	1911	PSU	N1-C2-N3	5.46	120.93	115.17
19	I	1917	PSU	N1-C2-N3	5.44	120.91	115.17
19	I	1939	5MU	N3-C2-N1	5.34	121.84	114.89
19	I	2552	OMU	C4-N3-C2	-5.23	120.12	126.61
19	I	1618	6MZ	C2-N1-C6	5.22	120.65	116.60
19	I	2503	2MA	C5-C6-N1	-5.21	114.67	120.84
19	I	2604	PSU	C4-N3-C2	-4.97	119.52	126.37
19	I	1939	5MU	C5-C4-N3	4.91	119.59	115.32
19	I	746	PSU	C4-N3-C2	-4.86	119.68	126.37
19	I	2552	OMU	N3-C2-N1	4.85	121.21	114.89
19	I	747	5MU	N3-C2-N1	4.84	121.19	114.89
19	I	747	5MU	C5-C4-N3	4.76	119.46	115.32
19	I	2457	PSU	C4-N3-C2	-4.73	119.86	126.37
19	I	2605	PSU	C4-N3-C2	-4.70	119.90	126.37
19	I	2580	PSU	C4-N3-C2	-4.65	119.97	126.37
19	I	1618	6MZ	C9-N6-C6	-4.61	118.57	122.85
19	I	1911	PSU	C4-N3-C2	-4.59	120.04	126.37
19	I	747	5MU	O4-C4-C5	-4.48	119.80	124.92
19	I	1618	6MZ	N3-C2-N1	-4.47	122.60	128.67
19	I	1917	PSU	C4-N3-C2	-4.46	120.23	126.37
19	I	955	PSU	C4-N3-C2	-4.45	120.24	126.37
19	I	2030	6MZ	N3-C2-N1	-4.45	122.63	128.67
19	I	2504	PSU	C4-N3-C2	-4.44	120.25	126.37
19	I	1915	3TD	O2-C2-N3	-4.20	115.90	121.82
19	I	2030	6MZ	C9-N6-C6	-4.15	119.00	122.85
19	I	1939	5MU	O4-C4-C5	-4.07	120.26	124.92
19	I	2251	OMG	C8-N7-C5	3.92	109.22	102.55
19	I	2580	PSU	O2-C2-N1	-3.91	118.76	122.79
19	I	2445	2MG	C8-N7-C5	3.88	109.15	102.55
19	I	745	1MG	C8-N7-C5	3.87	109.14	102.55
19	I	1835	2MG	C8-N7-C5	3.81	109.04	102.55
19	I	2457	PSU	O2-C2-N1	-3.67	119.01	122.79

Continued on next page...

For Manuscript Review

Continued from previous page...

Mol	Chain	Res	Type	Atoms	Z	Observed(°)	Ideal(°)
19	I	2552	OMU	C5-C4-N3	3.65	119.91	114.80
43	U	81	4D4	NE-CZ-NH2	3.46	126.61	120.67
19	I	1962	5MC	C5-C6-N1	-3.45	119.56	123.31
19	I	2604	PSU	O2-C2-N1	-3.43	119.25	122.79
19	I	1939	5MU	C5-C6-N1	-3.41	119.61	123.31
19	I	745	1MG	C5-C6-N1	3.36	118.82	113.96
19	I	2449	H2U	C5-C4-N3	-3.32	113.15	116.69
19	I	747	5MU	C5-C6-N1	-3.32	119.71	123.31
19	I	2552	OMU	CM2-O2'-C2'	-3.25	106.13	114.47
19	I	1911	PSU	O2-C2-N1	-3.23	119.46	122.79
19	I	1917	PSU	O2-C2-N1	-3.20	119.49	122.79
19	I	2580	PSU	C6-N1-C2	-3.10	119.81	122.69
19	I	2504	PSU	O2-C2-N1	-3.07	119.63	122.79
19	I	1915	3TD	C1'-C5-C4	3.03	122.20	117.61
19	I	955	PSU	O2-C2-N1	-2.96	119.73	122.79
19	I	2498	OMC	O2-C2-N3	-2.96	117.67	122.33
19	I	2498	OMC	CM2-O2'-C2'	-2.93	106.97	114.47
19	I	2251	OMG	CM2-O2'-C2'	-2.90	107.03	114.47
19	I	2457	PSU	C6-N1-C2	-2.89	120.01	122.69
19	I	2251	OMG	C2-N1-C6	-2.88	119.84	125.11
19	I	1835	2MG	N1-C2-N2	2.86	119.48	116.56
19	I	2445	2MG	C5-C6-N1	2.75	119.31	114.07
19	I	2445	2MG	N1-C2-N2	2.72	119.34	116.56
19	I	1915	3TD	C4-N3-C2	-2.64	121.81	124.61
19	I	2552	OMU	O4-C4-C5	-2.64	120.61	125.16
19	I	1939	5MU	O2-C2-N1	-2.63	119.38	122.80
19	I	1915	3TD	C5-C6-N1	-2.55	118.61	122.14
19	I	746	PSU	O2-C2-N1	-2.53	120.18	122.79
43	U	81	4D4	O-C-CA	-2.51	118.31	124.77
19	I	955	PSU	C6-N1-C2	-2.50	120.37	122.69
19	I	747	5MU	C1'-N1-C2	2.43	121.95	117.59
19	I	2580	PSU	O4'-C1'-C2'	2.41	108.49	105.15
19	I	2604	PSU	C6-N1-C2	-2.41	120.45	122.69
19	I	1835	2MG	C5-C6-N1	2.41	118.67	114.07
19	I	2445	2MG	CM2-N2-C2	-2.41	118.47	123.65
19	I	2030	6MZ	C4-C5-N7	-2.40	106.80	109.34
19	I	2251	OMG	C5-C6-N1	2.36	118.58	114.07
19	I	1917	PSU	C6-N1-C2	-2.35	120.51	122.69
19	I	1618	6MZ	C4-C5-N7	-2.33	106.87	109.34
19	I	2504	PSU	C6-N1-C2	-2.31	120.54	122.69
19	I	1962	5MC	C5-C4-N3	-2.27	119.43	121.75
19	I	2605	PSU	O2-C2-N1	-2.25	120.47	122.79

Continued on next page...

For Manuscript Review

Continued from previous page...

Mol	Chain	Res	Type	Atoms	Z	Observed(°)	Ideal(°)
19	I	2552	OMU	O2-C2-N1	-2.18	119.96	122.80
19	I	2605	PSU	C6-N1-C2	-2.14	120.70	122.69
19	I	2503	2MA	N6-C6-N1	2.08	122.69	117.11
19	I	1911	PSU	C6-N1-C2	-2.05	120.79	122.69
19	I	747	5MU	C1'-N1-C6	-2.05	117.78	121.15
43	U	81	4D4	NH1-CZ-NE	-2.02	114.67	119.27

There are no chirality outliers.

All (18) torsion outliers are listed below:

Mol	Chain	Res	Type	Atoms
19	I	746	PSU	O4'-C4'-C5'-O5'
19	I	1618	6MZ	O4'-C4'-C5'-O5'
19	I	2498	OMC	O4'-C4'-C5'-O5'
19	I	2580	PSU	O4'-C4'-C5'-O5'
19	I	746	PSU	C3'-C4'-C5'-O5'
19	I	1618	6MZ	C3'-C4'-C5'-O5'
19	I	2030	6MZ	O4'-C4'-C5'-O5'
19	I	2030	6MZ	C3'-C4'-C5'-O5'
19	I	2580	PSU	C3'-C4'-C5'-O5'
43	U	81	4D4	CG-CD-NE-CZ
19	I	2552	OMU	C3'-C2'-O2'-CM2
19	I	1618	6MZ	C4'-C5'-O5'-P
19	I	2498	OMC	C3'-C4'-C5'-O5'
43	U	81	4D4	OB-CB-CG-CD
19	I	746	PSU	O4'-C1'-C5-C6
19	I	2069	G7M	O4'-C4'-C5'-O5'
43	U	81	4D4	O-C-CA-CB
43	U	81	4D4	CA-CB-CG-CD

There are no ring outliers.

3 monomers are involved in 8 short contacts:

Mol	Chain	Res	Type	Clashes	Symm-Clashes
19	I	1618	6MZ	1	0
19	I	2030	6MZ	1	0
19	I	1915	3TD	6	0

5.5 Carbohydrates (i)

There are no oligosaccharides in this entry.

For Manuscript Review

5.6 Ligand geometry (i)

Of 307 ligands modelled in this entry, 307 are monoatomic - leaving 0 for Mogul analysis.

There are no bond length outliers.

There are no bond angle outliers.

There are no chirality outliers.

There are no torsion outliers.

There are no ring outliers.

No monomer is involved in short contacts.

5.7 Other polymers (i)

There are no such residues in this entry.

5.8 Polymer linkage issues (i)

The following chains have linkage breaks:

Mol	Chain	Number of breaks
19	I	2

All chain breaks are listed below:

Model	Chain	Residue-1	Atom-1	Residue-2	Atom-2	Distance (Å)
1	I	1914:C	O3'	1915:3TD	P	5.73
1	I	1617:C	O3'	1618:6MZ	P	2.79

For Manuscript Review

6 Map visualisation (i)

This section contains visualisations of the EMDB entry EMD-75668. These allow visual inspection of the internal detail of the map and identification of artifacts.

Images derived from a raw map, generated by summing the deposited half-maps, are presented below the corresponding image components of the primary map to allow further visual inspection and comparison with those of the primary map.

6.1 Orthogonal projections (i)

6.1.1 Primary map

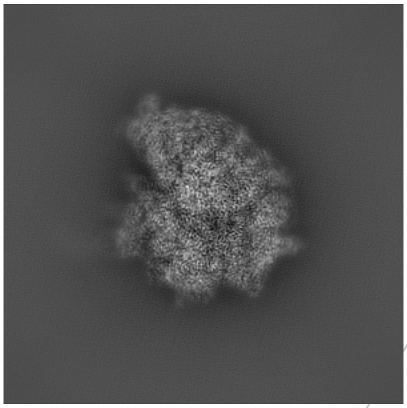
X

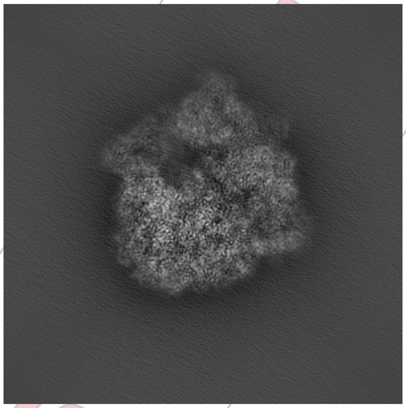
Y

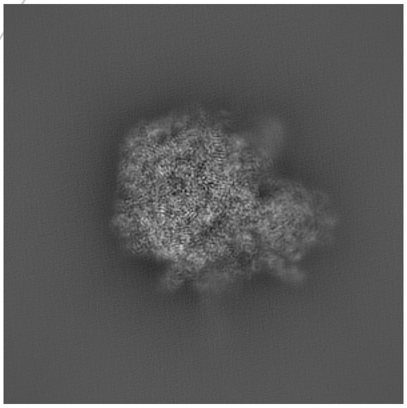
Z

6.1.2 Raw map

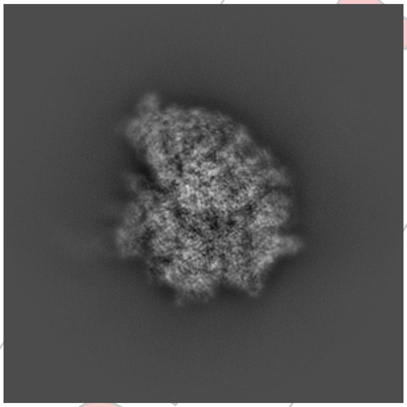
X

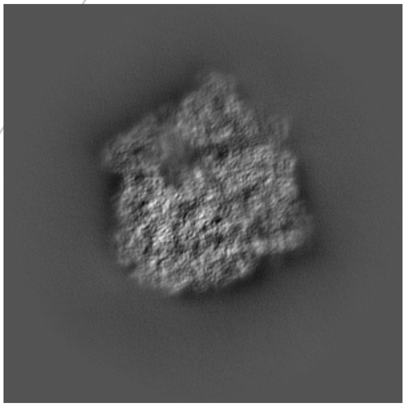
Y

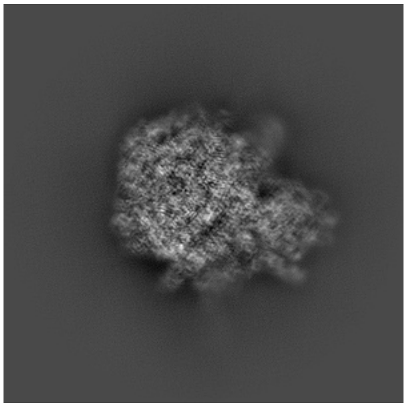
Z

The images above show the map projected in three orthogonal directions.

For Manuscript Review

6.2 Central slices (i)

6.2.1 Primary map

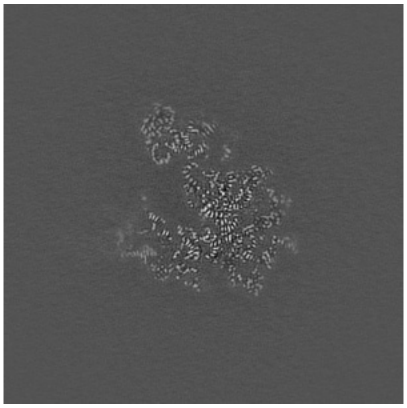

X Index: 240

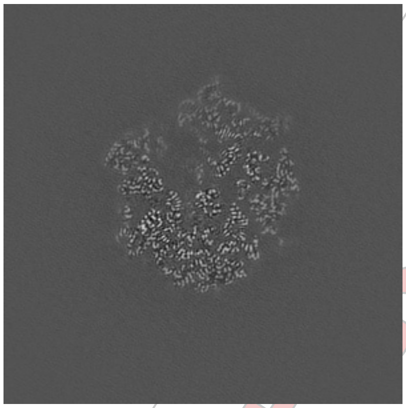

Y Index: 240

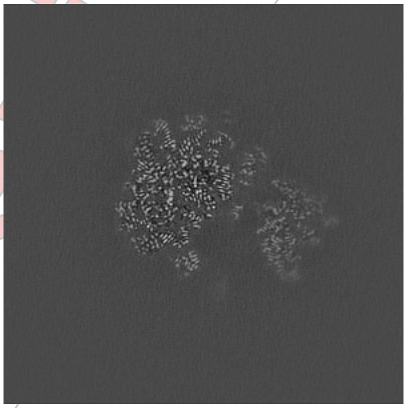

Z Index: 240

6.2.2 Raw map

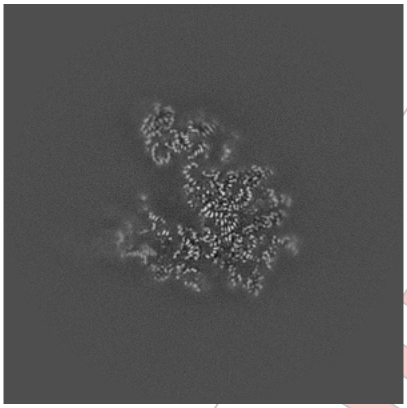

X Index: 240

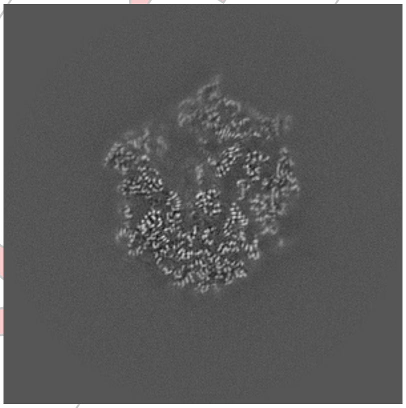

Y Index: 240

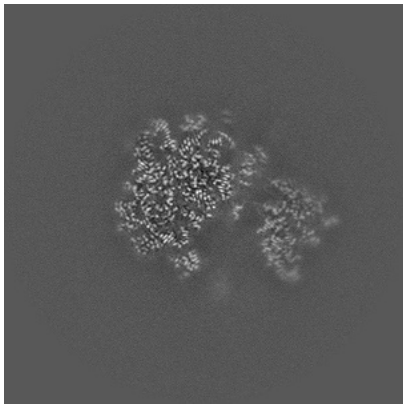

Z Index: 240

The images above show central slices of the map in three orthogonal directions.

For Manuscript Review

6.3 Largest variance slices (i)

6.3.1 Primary map

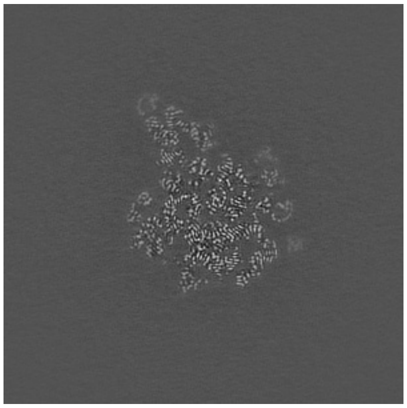
X Index: 220

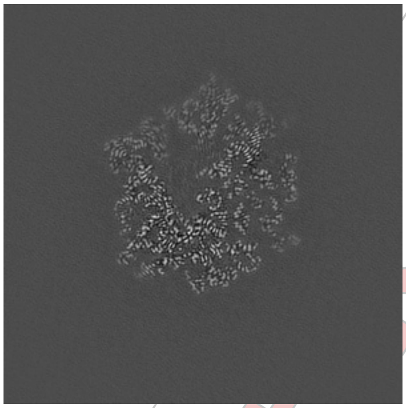
Y Index: 232

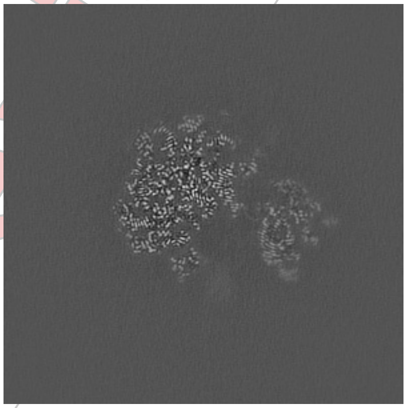
Z Index: 237

6.3.2 Raw map

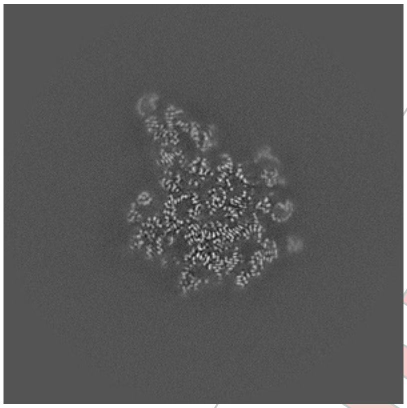
X Index: 220

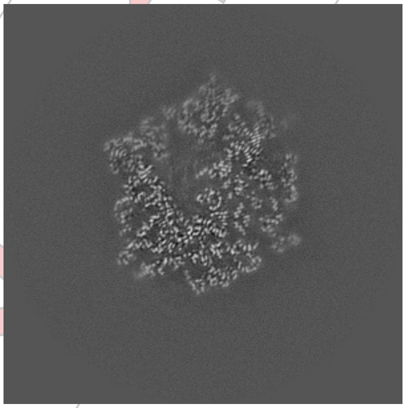
Y Index: 232

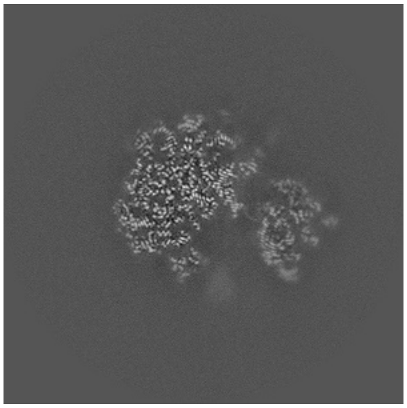
Z Index: 237

The images above show the largest variance slices of the map in three orthogonal directions.

For Manuscript Review

6.4 Orthogonal standard-deviation projections (False-color) (i)

6.4.1 Primary map

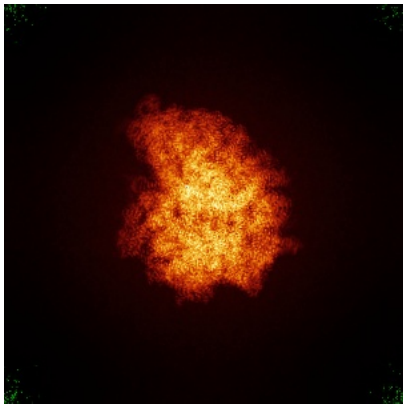

X

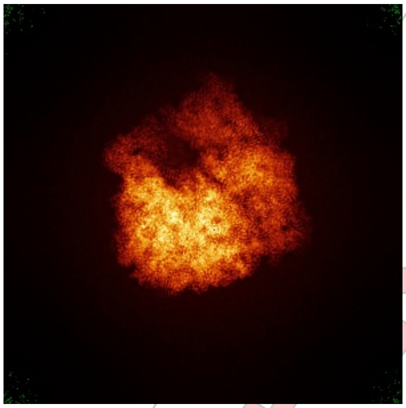

Y

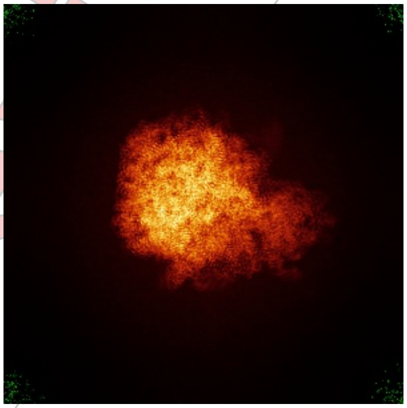

Z

6.4.2 Raw map

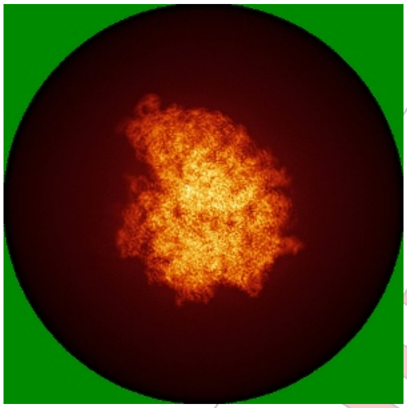

X

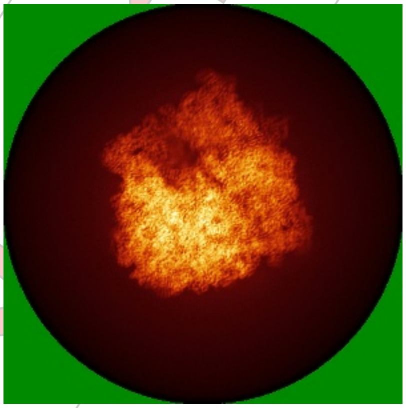

Y

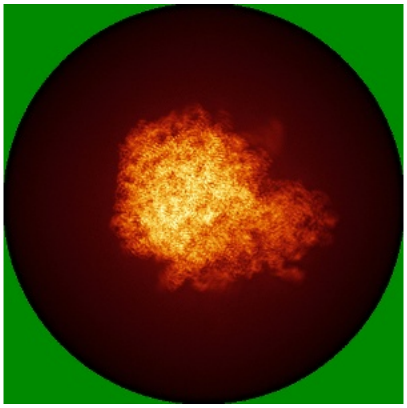

Z

The images above show the map standard deviation projections with false color in three orthogonal directions. Minimum values are shown in green, max in blue, and dark to light orange shades represent small to large values respectively.

For Manuscript Review

6.5 Orthogonal surface views (i)

6.5.1 Primary map

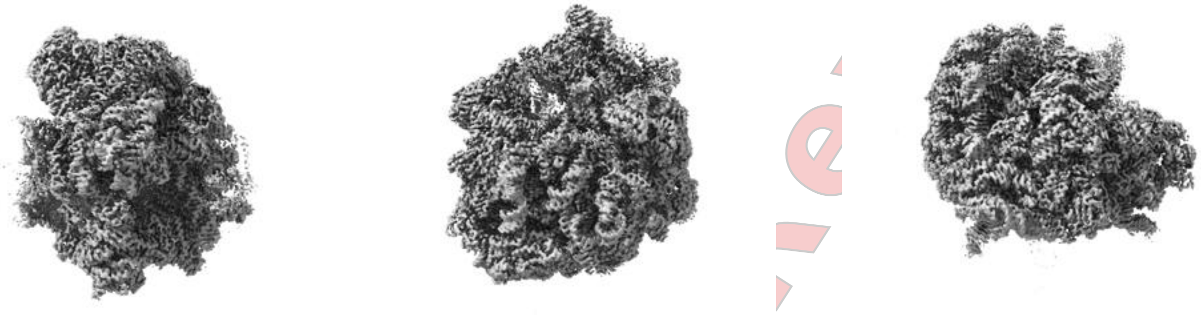

X Y Z

The images above show the 3D surface view of the map at the recommended contour level 2.5. These images, in conjunction with the slice images, may facilitate assessment of whether an appropriate contour level has been provided.

6.5.2 Raw map

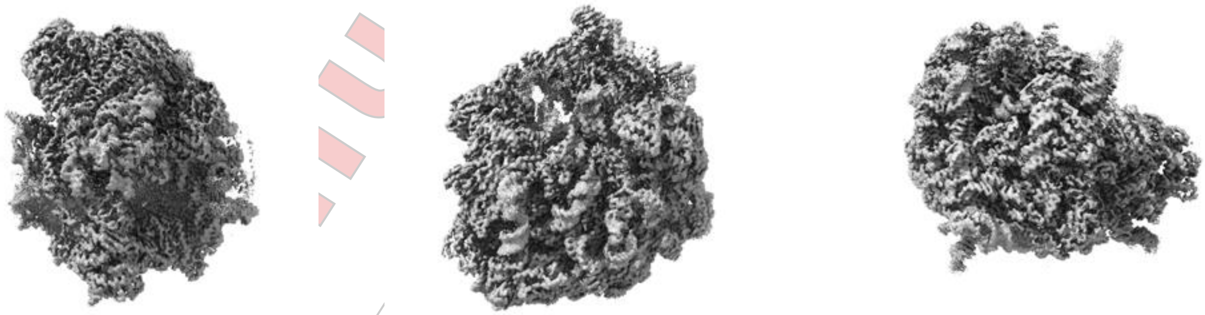

X Y Z

These images show the 3D surface of the raw map. The raw map's contour level was selected so that its surface encloses the same volume as the primary map does at its recommended contour level.

6.6 Mask visualisation (i)

This section was not generated. No masks/segmentation were deposited.

For Manuscript Review

7 Map analysis (i)

This section contains the results of statistical analysis of the map.

7.1 Map-value distribution (i)

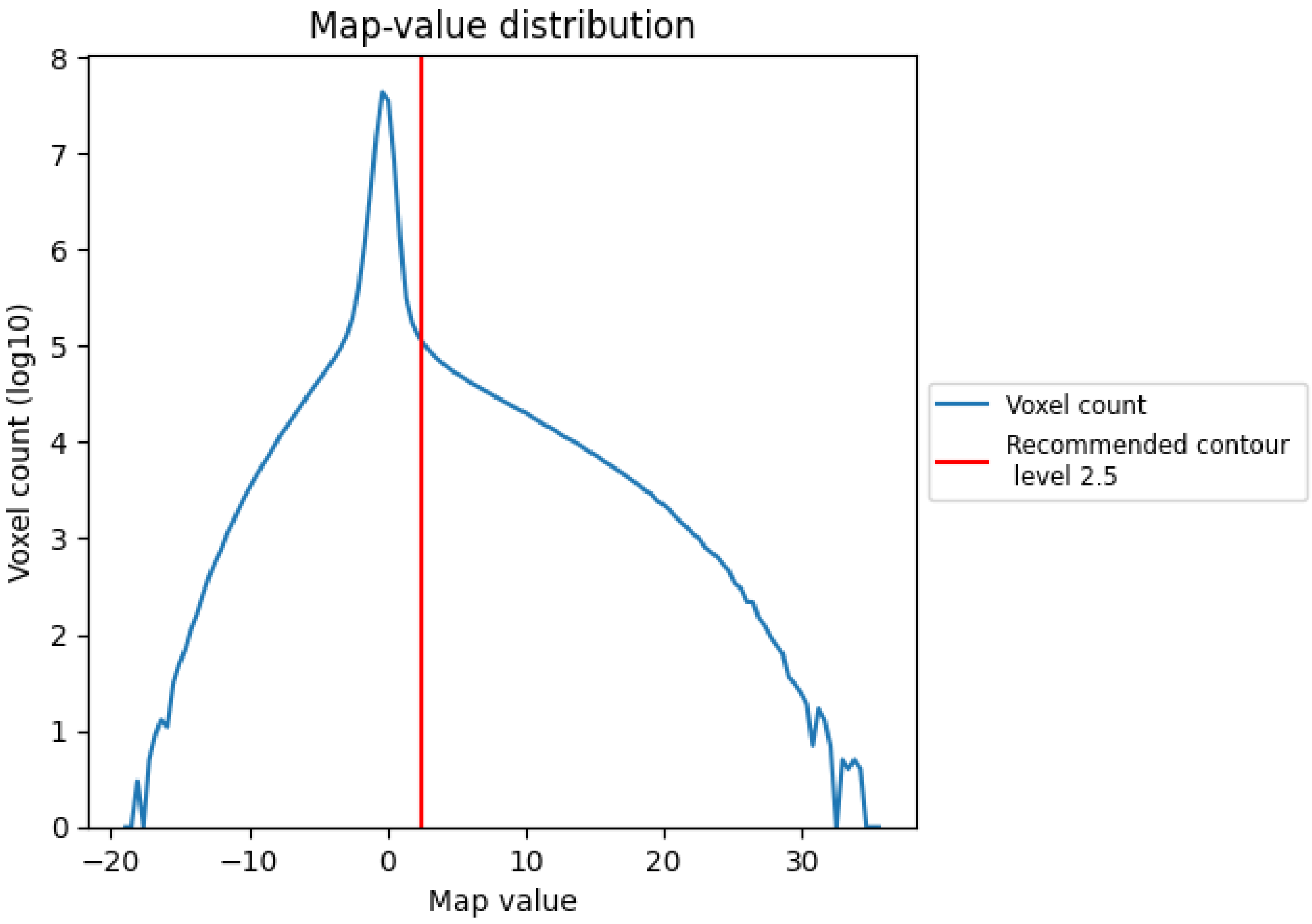

The map-value distribution is plotted in 128 intervals along the x-axis. The y-axis is logarithmic. A spike in this graph at zero usually indicates that the volume has been masked.

For Manuscript Review

7.2 Volume estimate (i)

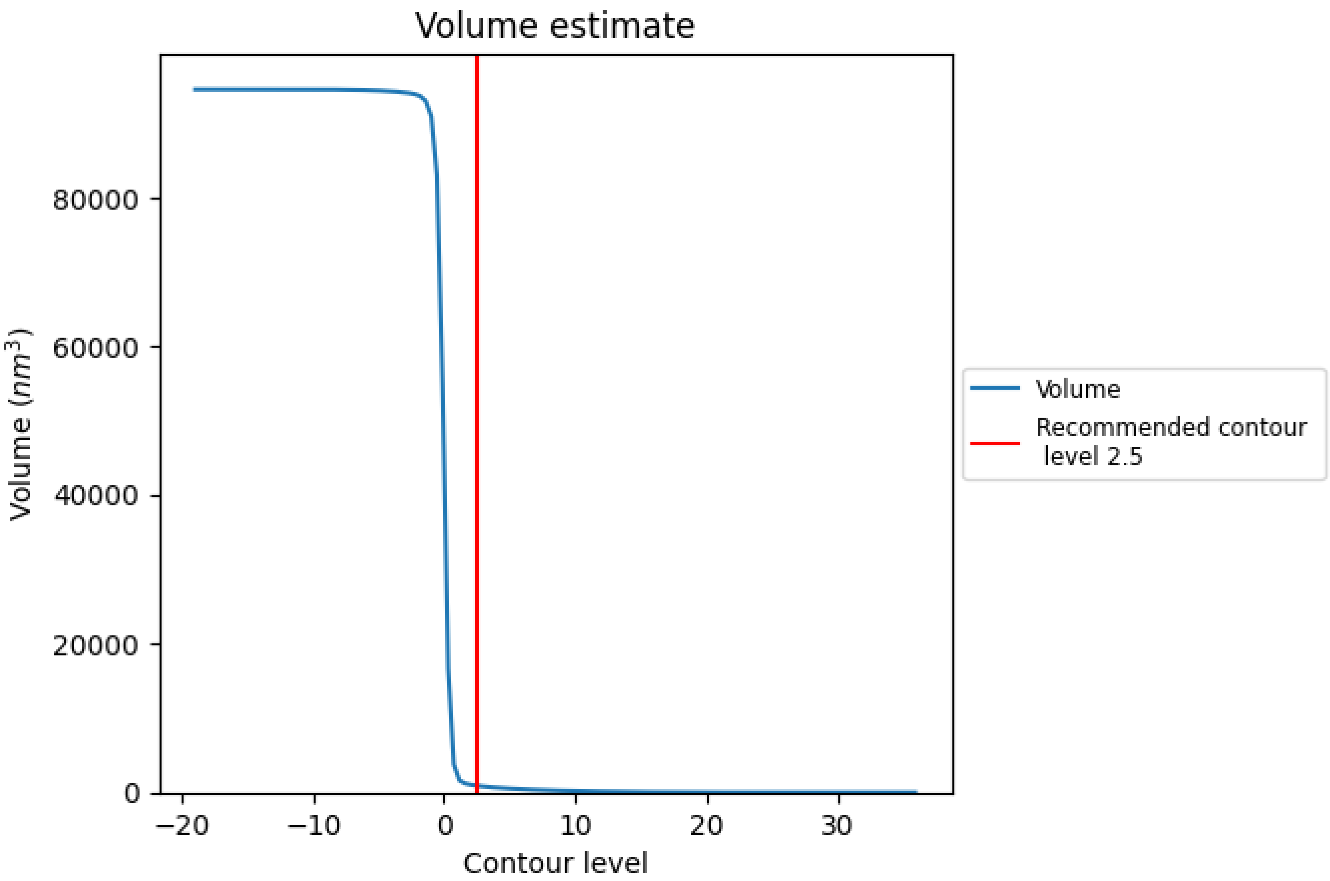

The volume at the recommended contour level is 914 nm^3; this corresponds to an approximate mass of 826 kDa.

The volume estimate graph shows how the enclosed volume varies with the contour level. The recommended contour level is shown as a vertical line and the intersection between the line and the curve gives the volume of the enclosed surface at the given level.

For Manuscript Review

7.3 Rotationally averaged power spectrum (i)

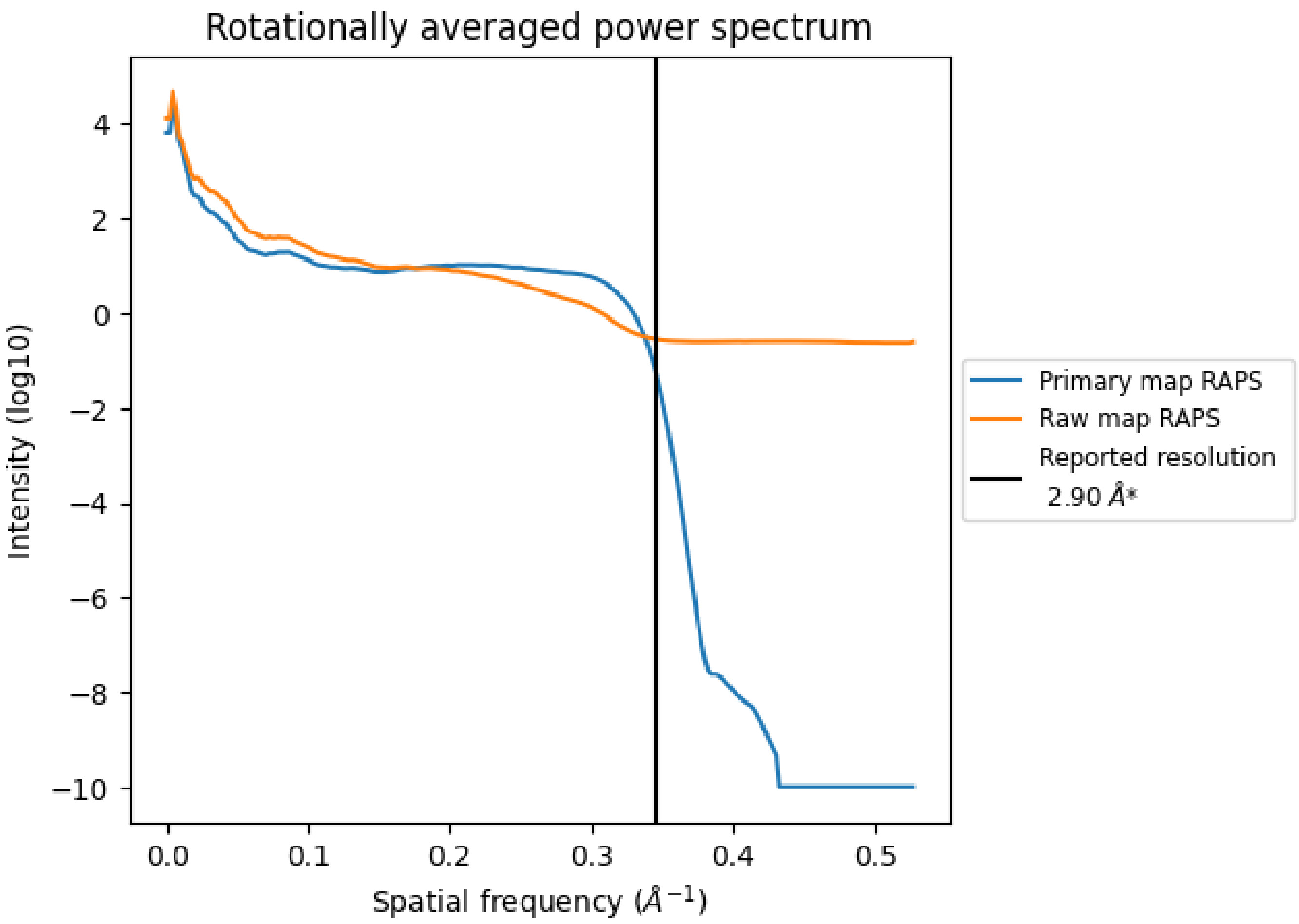

*Reported resolution corresponds to spatial frequency of 0.345 Å^{-1}

For Manuscript Review

8 Fourier-Shell correlation (i)

Fourier-Shell Correlation (FSC) is the most commonly used method to estimate the resolution of single-particle and subtomogram-averaged maps. The shape of the curve depends on the imposed symmetry, mask and whether or not the two 3D reconstructions used were processed from a common reference. The reported resolution is shown as a black line. A curve is displayed for the half-bit criterion in addition to lines showing the 0.143 gold standard cut-off and 0.5 cut-off.

8.1 FSC (i)

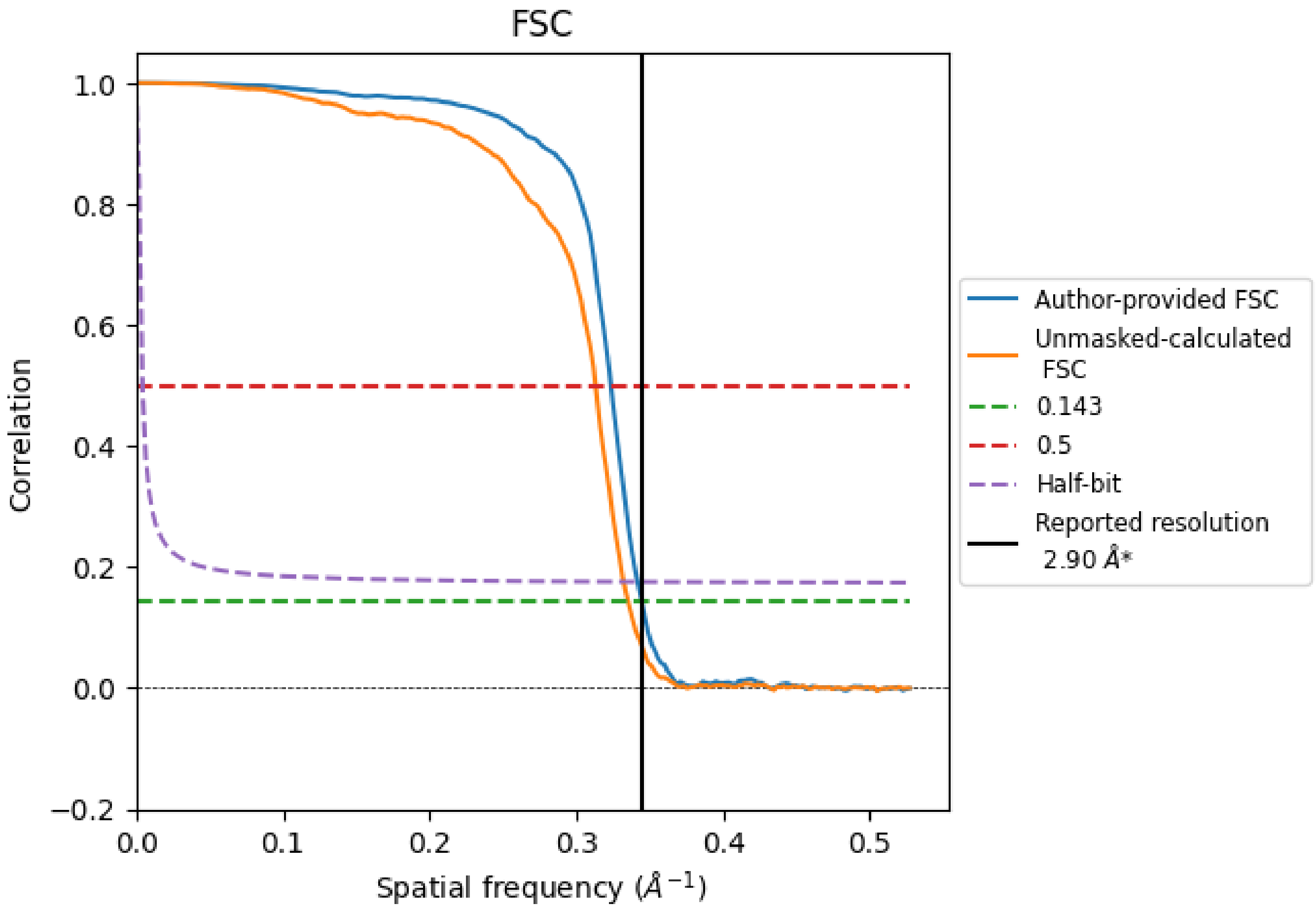

*Reported resolution corresponds to spatial frequency of 0.345 Å^{-1}

For Manuscript Review

8.2 Resolution estimates (i)

Resolution estimate (Å)	Estimation criterion (FSC cut-off)		
	0.143	0.5	Half-bit
Reported by author	2.90	-	-
Author-provided FSC curve	2.90	3.09	2.93
Unmasked-calculated*	2.98	3.19	3.01

*Resolution estimate based on FSC curve calculated by comparison of deposited half-maps.

For Manuscript Review

9 Map-model fit (i)

This section contains information regarding the fit between EMDB map EMD-75668 and PDB model 11FV. Per-residue inclusion information can be found in section 3 on page 15.

9.1 Map-model overlay (i)

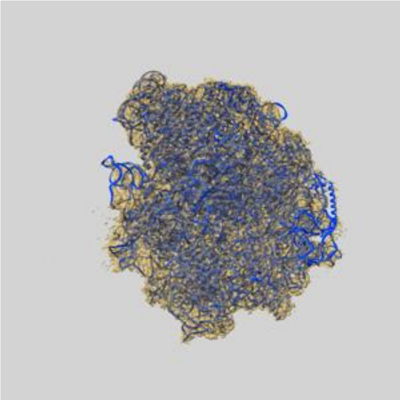

X

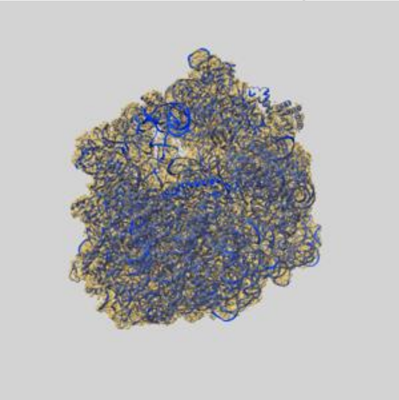

Y

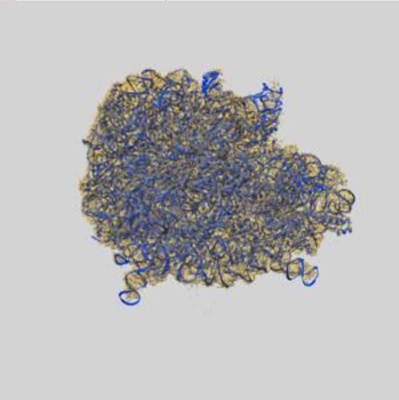

Z

The images above show the 3D surface view of the map at the recommended contour level 2.5 at 50% transparency in yellow overlaid with a ribbon representation of the model coloured in blue. These images allow for the visual assessment of the quality of fit between the atomic model and the map.

For Manuscript Review

9.2 Q-score mapped to coordinate model (i)

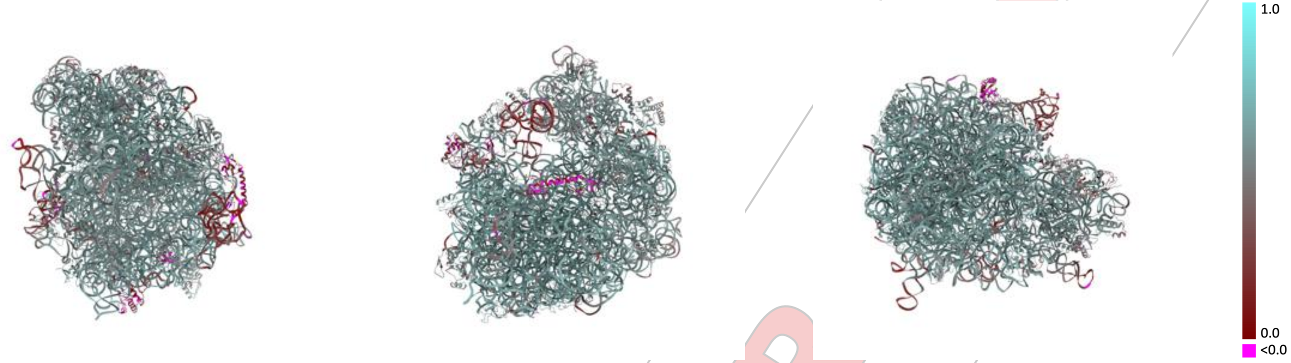

X Y Z

The images above show the model with each residue coloured according its Q-score. This shows their resolvability in the map with higher Q-score values reflecting better resolvability. Please note: Q-score is calculating the resolvability of atoms, and thus high values are only expected at resolutions at which atoms can be resolved. Low Q-score values may therefore be expected for many entries.

9.3 Atom inclusion mapped to coordinate model (i)

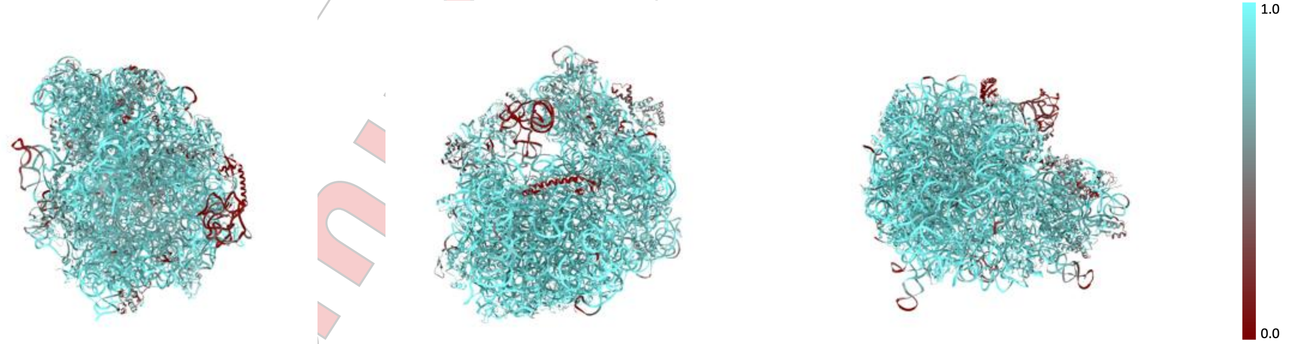

X Y Z

The images above show the model with each residue coloured according to its atom inclusion. This shows to what extent they are inside the map at the recommended contour level (2.5).

For Manuscript Review

9.4 Atom inclusion (i)

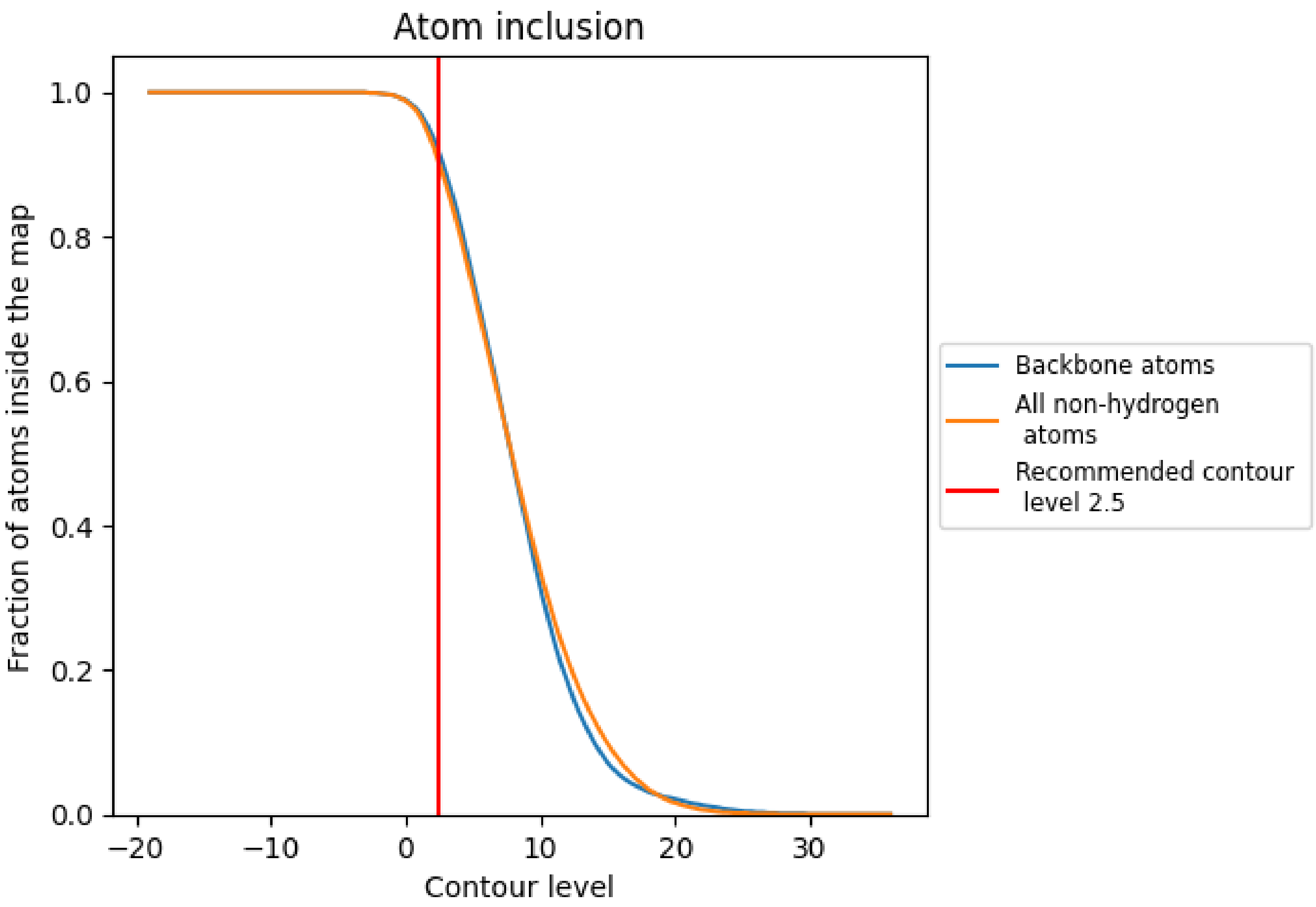

At the recommended contour level, 92% of all backbone atoms, 90% of all non-hydrogen atoms, are inside the map.

For Manuscript Review

9.5 Map-model fit summary (i)

The table lists the average atom inclusion at the recommended contour level (2.5) and Q-score for the entire model and for each chain.

Chain	Atom inclusion	Q-score
All	0.9000	0.5760
0	0.9160	0.6130
1	0.7590	0.4840
2	0.8880	0.5760
3	0.4860	0.4210
C	0.9430	0.5850
D	0.5210	0.4760
E	0.7840	0.5410
F	0.7920	0.5450
G	0.9060	0.5870
H	0.7950	0.5070
I	0.9360	0.5940
J	0.9750	0.6030
K	0.9480	0.6130
L	0.9490	0.6190
M	0.8440	0.5510
N	0.8050	0.5440
O	0.8600	0.5620
P	0.1460	0.1470
Q	0.7220	0.5010
R	0.9440	0.6220
S	0.9550	0.6180
T	0.9000	0.5870
U	0.9370	0.6050
V	0.9570	0.6120
W	0.4390	0.1610
X	0.9350	0.6180
Y	0.9700	0.6230
Z	0.9120	0.5950
b	0.8910	0.5830
c	0.8620	0.5670
d	0.8930	0.5820
e	0.9460	0.6200
f	0.9230	0.6030
g	0.8430	0.5450

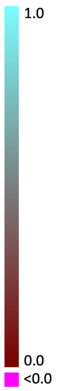

Continued on next page...

For Manuscript Review

Continued from previous page...

Chain	Atom inclusion	Q-score
h	0.9060	0.5980
i	0.9390	0.6210
j	0.1950	0.1140
k	0.9750	0.6340
l	0.9630	0.6270
m	0.9490	0.6090
n	0.5970	0.5090
o	0.9050	0.5980
p	0.6880	0.4900
q	0.6620	0.5410
r	0.8640	0.5450
t	0.9000	0.5890
u	0.7800	0.5380
v	0.7380	0.4690
w	0.9100	0.5790
x	0.8530	0.5760
y	0.8150	0.5230
z	0.9060	0.5710

For Manuscript Review
