# Supplementary material for: Structural adaptations for enhanced translation kinetics in evolved ribosomes
Source: Nucleic Acids Res. 2026 Jul 22;54(14):gkag703. doi: 10.1093/nar/gkag703 (PMC13389312; doi:10.1093/nar/gkag703)
Supplement: gkag703_Supplemental_Files [file gkag703_supplemental_files.zip › PDB_validation.zip!/VC_ST_D_1000305288_val-report-full-annotate_P1.pdf]

wwPDB EM Validation Summary Report (i)

Feb 25, 2026 – 01:12 PM EST

PDB ID	:	11EL / pdb_000011el
EMDB ID	:	EMD-75650
Title	:	Chimeric Escherichia coli 70S ribosome containing an evolved 16S rRNA from Vibrio cholerae (VC-ST)
Deposited on	:	2026-02-19
Resolution	:	3.20 Å(reported)

This wwPDB validation report is for manuscript review

This is a wwPDB EM Validation Summary Report.

This report is produced by the wwPDB biocuration pipeline after annotation of the structure.

We welcome your comments at *validation@mail.wwpdb.org*
A user guide is available at
https://www.wwpdb.org/validation/2017/EMValidationReportHelp
with specific help available everywhere you see the (i) symbol.

The types of validation reports are described at
http://www.wwpdb.org/validation/2017/FAQs#types.

For Manuscript Review

The following versions of software and data (see references (i)) were used in the production of this report:

EMDB validation analysis	:	0.0.1.dev132
Mogul	:	2022.3.0, CSD as543be (2022)
MolProbity	:	4-5-2 with Phenix2.0
Percentile statistics	:	20231227.v01 (using entries in the PDB archive December 27th 2023)
EM percentile statistics	:	202505.v01 (Using data in the EMDB archive up until May 2025)
MapQ	:	1.9.13
Ideal geometry (proteins)	:	Engh & Huber (2001)
Ideal geometry (DNA, RNA)	:	Parkinson et al. (1996)

1 Overall quality at a glance (i)

The following experimental techniques were used to determine the structure:
ELECTRON MICROSCOPY

The reported resolution of this entry is 3.20 Å.

Percentile scores (ranging between 0-100) for global validation metrics of the entry are shown in the following graphic. The table shows the number of entries on which the scores are based.

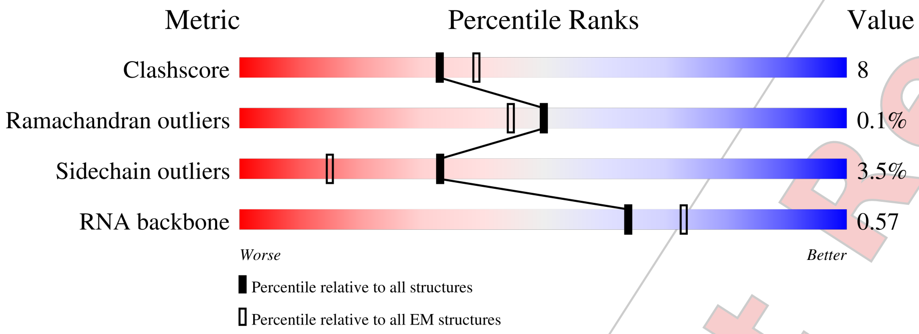

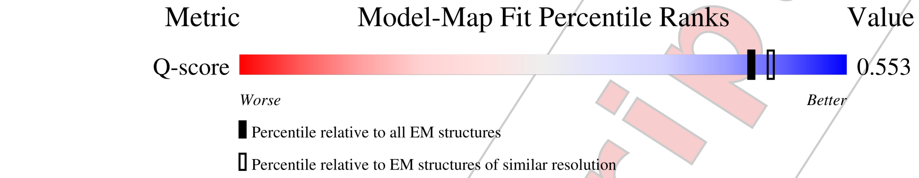

Metric	Whole archive (#Entries)	EM structures (#Entries)	Similar EM resolution (#Entries, resolution range(Å))
Clashscore	210492	15764	-
Ramachandran outliers	207382	16835	-
Sidechain outliers	206894	16415	-
RNA backbone	6643	2191	-
Q-score	-	25397	15020 (2.70 - 3.70)

The table below summarises the geometric issues observed across the polymeric chains and their fit to the map. The red, orange, yellow and green segments of the bar indicate the fraction of residues that contain outliers for >=3, 2, 1 and 0 types of geometric quality criteria respectively. A grey segment represents the fraction of residues that are not modelled. The numeric value for each fraction is indicated below the corresponding segment, with a dot representing fractions <=5% The upper red bar (where present) indicates the fraction of residues that have poor fit to the EM map (all-atom inclusion < 40%). The numeric value is given above the bar.

Mol	Chain	Length	Quality of chain
1	0	55	11% / 75% 18% 7%

Continued on next page...

For Manuscript Review

Continued from previous page...

Mol	Chain	Length	Quality of chain
2	1	46	• 83% 17%
3	2	65	77% 22% •
4	3	38	• 82% 18%
5	4	70	6% 51% 33% • 14%
6	5	2	100% 50% 50%
7	A	1532	• 52% 37% 9% •
8	B	241	12% 69% 23% 7%
9	C	233	5% 60% 26% •• 12%
10	D	206	5% 68% 30% •
11	E	167	• 74% 19% • 7%
12	F	135	50% 24% • 24%
13	G	179	8% 62% 20% • 15%
14	H	130	• 72% 28% •
15	I	130	9% 60% 37% ••
16	J	103	28% 63% 28% • 5%
17	K	129	• 60% 29% • 9%
18	L	124	• 67% 31% ••
19	M	118	5% 72% 25% •
20	N	101	• 61% 38% •
21	O	89	• 80% 19% •
22	P	82	27% 43% 56% •
23	Q	84	• 61% 31% •• 6%
24	R	75	5% 63% 24% • 12%
25	S	92	• 60% 32% 9%
26	T	87	• 69% 26% ••

Continued on next page...

For Manuscript Review

Continued from previous page...

Mol	Chain	Length	Quality of chain
27	U	71	31% 70% 28% •
28	a	2904	• 60% 32% • 5%
29	b	120	• 72% 25% ••
30	c	273	79% 19% •
31	d	209	83% 16%
32	e	201	• 78% 21%
33	f	179	• 69% 30% ••
34	g	177	• 67% 29% ••
35	h	149	• 19% 8% 72%
36	i	142	77% 23%
37	j	123	70% 28% •
38	k	144	• 83% 17%
39	l	136	80% 19% •
40	m	127	76% 16% • 7%
41	n	117	82% 17% •
42	o	115	• 83% 16% ••
43	p	118	• 88% 11% •
44	q	103	• 77% 22% •
45	r	110	• 76% 23% •
46	s	100	71% 22% 7%
47	t	104	• 72% 25% ••
48	u	94	• 81% 18% •
49	v	85	• 64% 27% • 8%
50	w	78	83% 15% •
51	x	63	• 76% 21% ••

Continued on next page...

For Manuscript Review

Continued from previous page...

Mol	Chain	Length	Quality of chain
52	y	59	85% 12%
53	z	57	68% 30%

For Manuscript Review

2 Entry composition (i)

There are 57 unique types of molecules in this entry. The entry contains 138605 atoms, of which 0 are hydrogens and 0 are deuteriums.

In the tables below, the AltConf column contains the number of residues with at least one atom in alternate conformation and the Trace column contains the number of residues modelled with at most 2 atoms.

- Molecule 1 is a protein called 50S ribosomal protein L33.

Mol	Chain	Residues	Atoms				AltConf	Trace
			Total	C	N	O		
1	0	51	417	269	76	72	0	0

- Molecule 2 is a protein called Large ribosomal subunit protein bL34.

Mol	Chain	Residues	Atoms					AltConf	Trace
			Total	C	N	O	S		
2	1	46	377	228	90	57	2	0	0

- Molecule 3 is a protein called 50S ribosomal protein L35.

Mol	Chain	Residues	Atoms					AltConf	Trace
			Total	C	N	O	S		
3	2	64	504	323	105	74	2	0	0

- Molecule 4 is a protein called Large ribosomal subunit protein bL36A.

Mol	Chain	Residues	Atoms					AltConf	Trace
			Total	C	N	O	S		
4	3	38	302	185	65	48	4	0	0

- Molecule 5 is a protein called 50S ribosomal protein L31.

Mol	Chain	Residues	Atoms					AltConf	Trace
			Total	C	N	O	S		
5	4	60	480	299	90	85	6	0	0

- Molecule 6 is a RNA chain called E-site tRNA.

Mol	Chain	Residues	Atoms					AltConf	Trace
			Total	C	N	O	P		
6	5	2	42	19	8	13	2	0	0

For Manuscript Review

- Molecule 7 is a RNA chain called 16S ribosomal RNA.

Mol	Chain	Residues	Atoms					AltConf	Trace
			Total	C	N	O	P		
7	A	1520	32632	14563	5988	10561	1520	0	0

There are 14 discrepancies between the modelled and reference sequences:

Chain	Residue	Modelled	Actual	Comment	Reference
A	193	C	G	conflict	GB 3166933093
A	412	C	A	conflict	GB 3166933093
A	459	A	U	conflict	GB 3166933093
A	626	G	A	conflict	GB 3166933093
A	641	U	A	conflict	GB 3166933093
A	711	U	A	conflict	GB 3166933093
A	745	G	A	conflict	GB 3166933093
A	746	A	G	conflict	GB 3166933093
A	?	-	A	deletion	GB 3166933093
A	?	-	U	deletion	GB 3166933093
A	852	A	G	conflict	GB 3166933093
A	904	C	U	conflict	GB 3166933093
A	1416	A	G	conflict	GB 3166933093
A	1420	A	G	conflict	GB 3166933093

- Molecule 8 is a protein called Small ribosomal subunit protein uS2.

Mol	Chain	Residues	Atoms					AltConf	Trace
			Total	C	N	O	S		
8	B	224	1753	1109	315	321	8	0	0

- Molecule 9 is a protein called Small ribosomal subunit protein uS3.

Mol	Chain	Residues	Atoms					AltConf	Trace
			Total	C	N	O	S		
9	C	206	1624	1028	305	288	3	0	0

- Molecule 10 is a protein called Small ribosomal subunit protein uS4.

Mol	Chain	Residues	Atoms					AltConf	Trace
			Total	C	N	O	S		
10	D	205	1643	1026	315	298	4	0	0

- Molecule 11 is a protein called Small ribosomal subunit protein uS5.

For Manuscript Review

Mol	Chain	Residues	Atoms					AltConf	Trace
			Total	C	N	O	S		
11	E	156	1152	717	217	212	6	0	0

- Molecule 12 is a protein called Small ribosomal subunit protein bS6.

Mol	Chain	Residues	Atoms					AltConf	Trace
			Total	C	N	O	S		
12	F	103	839	530	151	151	7	0	0

- Molecule 13 is a protein called Small ribosomal subunit protein uS7.

Mol	Chain	Residues	Atoms					AltConf	Trace
			Total	C	N	O	S		
13	G	153	1203	750	231	218	4	0	0

- Molecule 14 is a protein called Small ribosomal subunit protein uS8.

Mol	Chain	Residues	Atoms					AltConf	Trace
			Total	C	N	O	S		
14	H	129	979	616	173	184	6	0	0

- Molecule 15 is a protein called Small ribosomal subunit protein uS9.

Mol	Chain	Residues	Atoms					AltConf	Trace
			Total	C	N	O	S		
15	I	127	1022	634	206	179	3	0	0

- Molecule 16 is a protein called Small ribosomal subunit protein uS10.

Mol	Chain	Residues	Atoms					AltConf	Trace
			Total	C	N	O	S		
16	J	98	786	493	150	142	1	0	0

- Molecule 17 is a protein called Small ribosomal subunit protein uS11.

Mol	Chain	Residues	Atoms					AltConf	Trace
			Total	C	N	O	S		
17	K	117	877	540	173	161	3	0	0

There is a discrepancy between the modelled and reference sequences:

For Manuscript Review

Chain	Residue	Modelled	Actual	Comment	Reference
K	119	IAS	ASN	engineered mutation	UNP A7ZSI6

- Molecule 18 is a protein called Small ribosomal subunit protein uS12.

Mol	Chain	Residues	Atoms					AltConf	Trace
			Total	C	N	O	S		
18	L	123	957	591	196	165	5	0	0

- Molecule 19 is a protein called Small ribosomal subunit protein uS13.

Mol	Chain	Residues	Atoms					AltConf	Trace
			Total	C	N	O	S		
19	M	115	891	552	179	157	3	0	0

- Molecule 20 is a protein called Small ribosomal subunit protein uS14.

Mol	Chain	Residues	Atoms					AltConf	Trace
			Total	C	N	O	S		
20	N	100	805	499	164	139	3	0	0

- Molecule 21 is a protein called Small ribosomal subunit protein uS15.

Mol	Chain	Residues	Atoms					AltConf	Trace
			Total	C	N	O	S		
21	O	88	714	439	144	130	1	0	0

- Molecule 22 is a protein called Small ribosomal subunit protein bS16.

Mol	Chain	Residues	Atoms					AltConf	Trace
			Total	C	N	O	S		
22	P	81	643	403	127	112	1	0	0

- Molecule 23 is a protein called Small ribosomal subunit protein uS17.

Mol	Chain	Residues	Atoms					AltConf	Trace
			Total	C	N	O	S		
23	Q	79	641	406	120	112	3	0	0

- Molecule 24 is a protein called Small ribosomal subunit protein bS18.

For Manuscript Review

Mol	Chain	Residues	Atoms					AltConf	Trace
			Total	C	N	O	S		
24	R	66	544	345	102	96	1	0	0

- Molecule 25 is a protein called Small ribosomal subunit protein uS19.

Mol	Chain	Residues	Atoms					AltConf	Trace
			Total	C	N	O	S		
25	S	84	668	427	127	112	2	0	0

- Molecule 26 is a protein called Small ribosomal subunit protein bS20.

Mol	Chain	Residues	Atoms					AltConf	Trace
			Total	C	N	O	S		
26	T	86	670	414	138	115	3	0	0

- Molecule 27 is a protein called Small ribosomal subunit protein bS21.

Mol	Chain	Residues	Atoms					AltConf	Trace
			Total	C	N	O	S		
27	U	70	589	366	125	97	1	0	0

- Molecule 28 is a RNA chain called 23S ribosomal RNA.

Mol	Chain	Residues	Atoms					AltConf	Trace
			Total	C	N	O	P		
28	a	2753	59130	26384	10897	19096	2753	0	0

- Molecule 29 is a RNA chain called 5S ribosomal RNA.

Mol	Chain	Residues	Atoms					AltConf	Trace
			Total	C	N	O	P		
29	b	119	2549	1135	466	829	119	0	0

- Molecule 30 is a protein called 50S ribosomal protein L2.

Mol	Chain	Residues	Atoms					AltConf	Trace
			Total	C	N	O	S		
30	c	271	2082	1288	423	364	7	0	0

- Molecule 31 is a protein called 50S ribosomal protein L3.

For Manuscript Review

Mol	Chain	Residues	Atoms					AltConf	Trace
			Total	C	N	O	S		
31	d	209	1566	980	288	294	4	0	0

- Molecule 32 is a protein called Large ribosomal subunit protein uL4.

Mol	Chain	Residues	Atoms					AltConf	Trace
			Total	C	N	O	S		
32	e	201	1552	974	283	290	5	0	0

- Molecule 33 is a protein called Large ribosomal subunit protein uL5.

Mol	Chain	Residues	Atoms					AltConf	Trace
			Total	C	N	O	S		
33	f	177	1410	899	249	256	6	0	0

- Molecule 34 is a protein called Large ribosomal subunit protein uL6.

Mol	Chain	Residues	Atoms					AltConf	Trace
			Total	C	N	O	S		
34	g	176	1323	832	243	246	2	0	0

- Molecule 35 is a protein called Large ribosomal subunit protein bL9.

Mol	Chain	Residues	Atoms					AltConf	Trace
			Total	C	N	O	S		
35	h	41	303	194	54	54	1	0	0

- Molecule 36 is a protein called Large ribosomal subunit protein uL13.

Mol	Chain	Residues	Atoms					AltConf	Trace
			Total	C	N	O	S		
36	i	142	1129	714	212	199	4	0	0

- Molecule 37 is a protein called Large ribosomal subunit protein uL14.

Mol	Chain	Residues	Atoms					AltConf	Trace
			Total	C	N	O	S		
37	j	123	946	593	181	166	6	0	0

- Molecule 38 is a protein called Large ribosomal subunit protein uL15.

For Manuscript Review

Mol	Chain	Residues	Atoms					AltConf	Trace
			Total	C	N	O	S		
38	k	144	1053	654	207	190	2	0	0

- Molecule 39 is a protein called Large ribosomal subunit protein uL16.

Mol	Chain	Residues	Atoms					AltConf	Trace
			Total	C	N	O	S		
39	l	136	1075	686	205	177	7	0	0

- Molecule 40 is a protein called Large ribosomal subunit protein bL17.

Mol	Chain	Residues	Atoms					AltConf	Trace
			Total	C	N	O	S		
40	m	118	945	585	194	161	5	0	0

- Molecule 41 is a protein called Large ribosomal subunit protein uL18.

Mol	Chain	Residues	Atoms				AltConf	Trace
			Total	C	N	O		
41	n	116	892	552	178	162	0	0

- Molecule 42 is a protein called Large ribosomal subunit protein bL19.

Mol	Chain	Residues	Atoms					AltConf	Trace
			Total	C	N	O	S		
42	o	114	917	574	179	163	1	0	0

- Molecule 43 is a protein called 50S ribosomal protein L20.

Mol	Chain	Residues	Atoms				AltConf	Trace
			Total	C	N	O		
43	p	117	947	604	192	151	0	0

- Molecule 44 is a protein called Large ribosomal subunit protein bL21.

Mol	Chain	Residues	Atoms					AltConf	Trace
			Total	C	N	O	S		
44	q	103	816	516	153	145	2	0	0

- Molecule 45 is a protein called Large ribosomal subunit protein uL22.

For Manuscript Review

Mol	Chain	Residues	Atoms					AltConf	Trace
			Total	C	N	O	S		
45	r	110	857	532	166	156	3	0	0

- Molecule 46 is a protein called 50S ribosomal protein L23.

Mol	Chain	Residues	Atoms					AltConf	Trace
			Total	C	N	O	S		
46	s	93	738	466	139	131	2	0	0

- Molecule 47 is a protein called 50S ribosomal protein L24.

Mol	Chain	Residues	Atoms				AltConf	Trace
			Total	C	N	O		
47	t	102	779	492	146	141	0	0

- Molecule 48 is a protein called Large ribosomal subunit protein bL25.

Mol	Chain	Residues	Atoms					AltConf	Trace
			Total	C	N	O	S		
48	u	94	753	479	137	134	3	0	0

- Molecule 49 is a protein called 50S ribosomal protein L27.

Mol	Chain	Residues	Atoms					AltConf	Trace
			Total	C	N	O	S		
49	v	78	586	362	116	107	1	0	0

- Molecule 50 is a protein called 50S ribosomal protein L28.

Mol	Chain	Residues	Atoms					AltConf	Trace
			Total	C	N	O	S		
50	w	77	625	388	129	106	2	0	0

- Molecule 51 is a protein called Large ribosomal subunit protein uL29.

Mol	Chain	Residues	Atoms					AltConf	Trace
			Total	C	N	O	S		
51	x	62	501	308	98	94	1	0	0

- Molecule 52 is a protein called 50S ribosomal protein L30.

For Manuscript Review

Mol	Chain	Residues	Atoms					AltConf	Trace
			Total	C	N	O	S		
52	y	58	449	281	87	79	2	0	0

- Molecule 53 is a protein called 50S ribosomal protein L32.

Mol	Chain	Residues	Atoms					AltConf	Trace
			Total	C	N	O	S		
53	z	56	444	269	94	80	1	0	0

- Molecule 54 is ZINC ION (CCD ID: ZN) (formula: Zn).

Mol	Chain	Residues	Atoms		AltConf
			Total	Zn	
54	3	1	1	1	0
54	4	1	1	1	0

- Molecule 55 is MAGNESIUM ION (CCD ID: MG) (formula: Mg).

Mol	Chain	Residues	Atoms		AltConf
			Total	Mg	
55	A	91	91	91	0
55	D	1	1	1	0
55	a	207	207	207	0
55	b	5	5	5	0
55	c	1	1	1	0
55	m	1	1	1	0
55	p	1	1	1	0
55	z	1	1	1	0

- Molecule 56 is SPERMIDINE (CCD ID: SPD) (formula: $C_7H_{19}N_3$).

For Manuscript Review

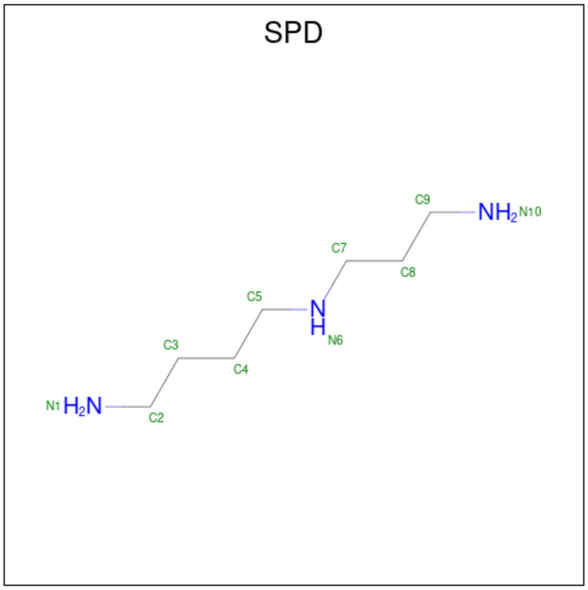

Mol	Chain	Residues	Atoms	AltConf
56	A	1	Total C N<br>10 7 3	0
56	A	1	Total C N<br>10 7 3	0
56	a	1	Total C N<br>10 7 3	0
56	a	1	Total C N<br>10 7 3	0
56	a	1	Total C N<br>10 7 3	0
56	a	1	Total C N<br>10 7 3	0
56	a	1	Total C N<br>10 7 3	0
56	a	1	Total C N<br>10 7 3	0
56	a	1	Total C N<br>10 7 3	0
56	a	1	Total C N<br>10 7 3	0
56	a	1	Total C N<br>10 7 3	0
56	a	1	Total C N<br>10 7 3	0
56	a	1	Total C N<br>10 7 3	0
56	a	1	Total C N<br>10 7 3	0

Continued on next page...

For Manuscript Review

Continued from previous page...

Mol	Chain	Residues	Atoms	AltConf
56	a	1	Total C N<br>10 7 3	0
56	a	1	Total C N<br>10 7 3	0

- Molecule 57 is SPERMINE (CCD ID: SPM) (formula: $C_{10}H_{26}N_4$).

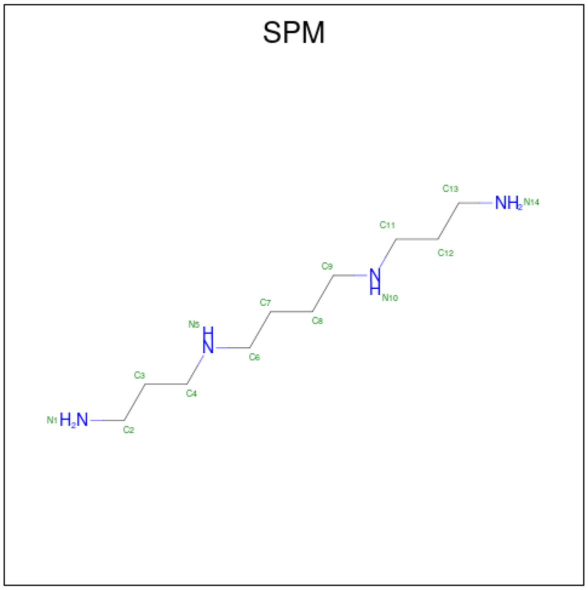

Mol	Chain	Residues	Atoms	AltConf
57	a	1	Total C N<br>14 10 4	0

For Manuscript Review

3 Residue-property plots ⓘ

These plots are drawn for all protein, RNA, DNA and oligosaccharide chains in the entry. The first graphic for a chain summarises the proportions of the various outlier classes displayed in the second graphic. The second graphic shows the sequence view annotated by issues in geometry and atom inclusion in map density. Residues are color-coded according to the number of geometric quality criteria for which they contain at least one outlier: green = 0, yellow = 1, orange = 2 and red = 3 or more. A red diamond above a residue indicates a poor fit to the EM map for this residue (all-atom inclusion < 40%). Stretches of 2 or more consecutive residues without any outlier are shown as a green connector. Residues present in the sample, but not in the model, are shown in grey.

- Molecule 1: 50S ribosomal protein L33

Chain 0:

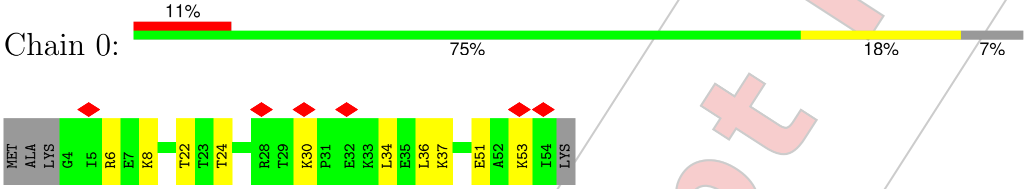

- Molecule 2: Large ribosomal subunit protein bL34

Chain 1:

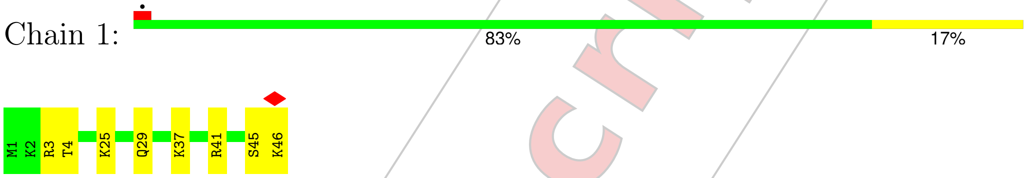

- Molecule 3: 50S ribosomal protein L35

Chain 2:

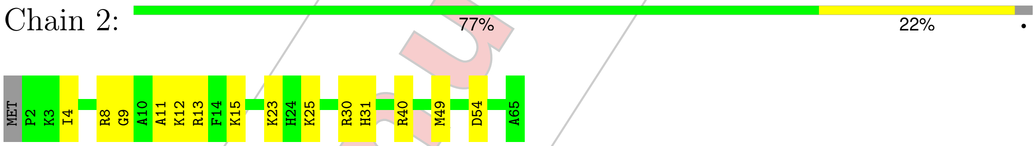

- Molecule 4: Large ribosomal subunit protein bL36A

Chain 3:

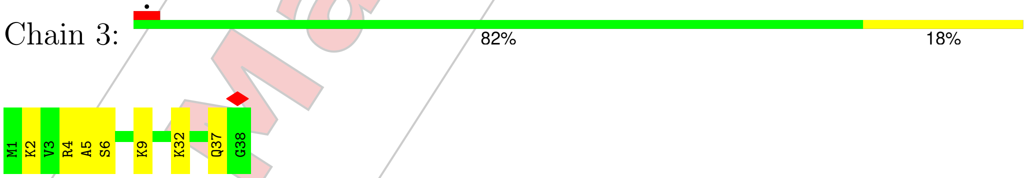

- Molecule 5: 50S ribosomal protein L31

Chain 4:

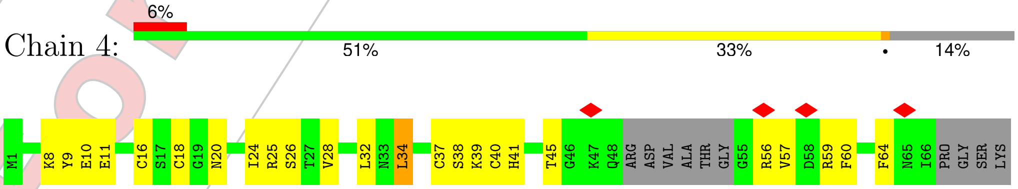

For Manuscript Review

- Molecule 6: E-site tRNA

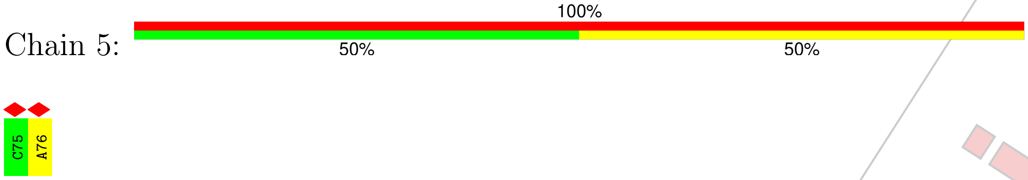

- Molecule 7: 16S ribosomal RNA

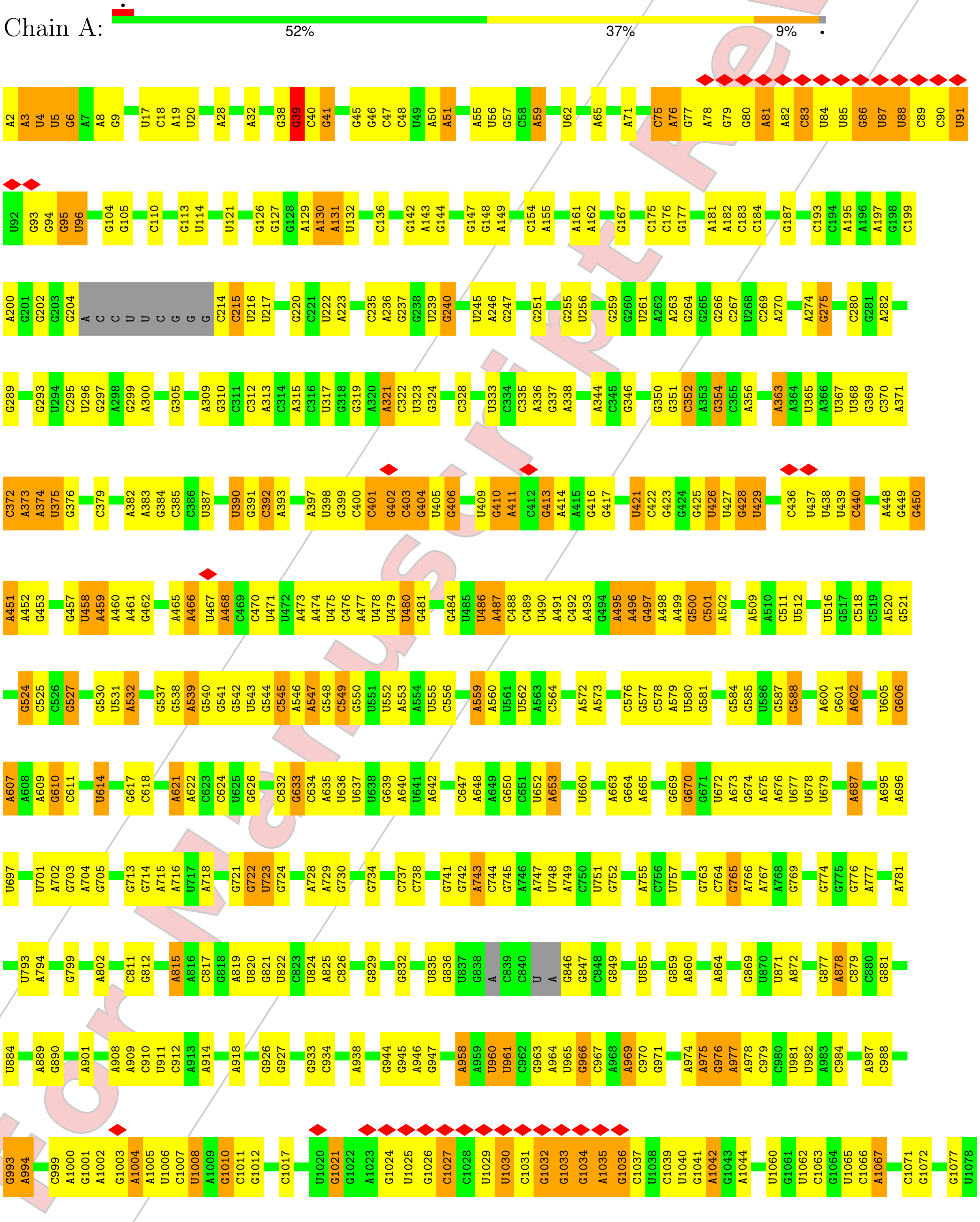

For Manuscript Review

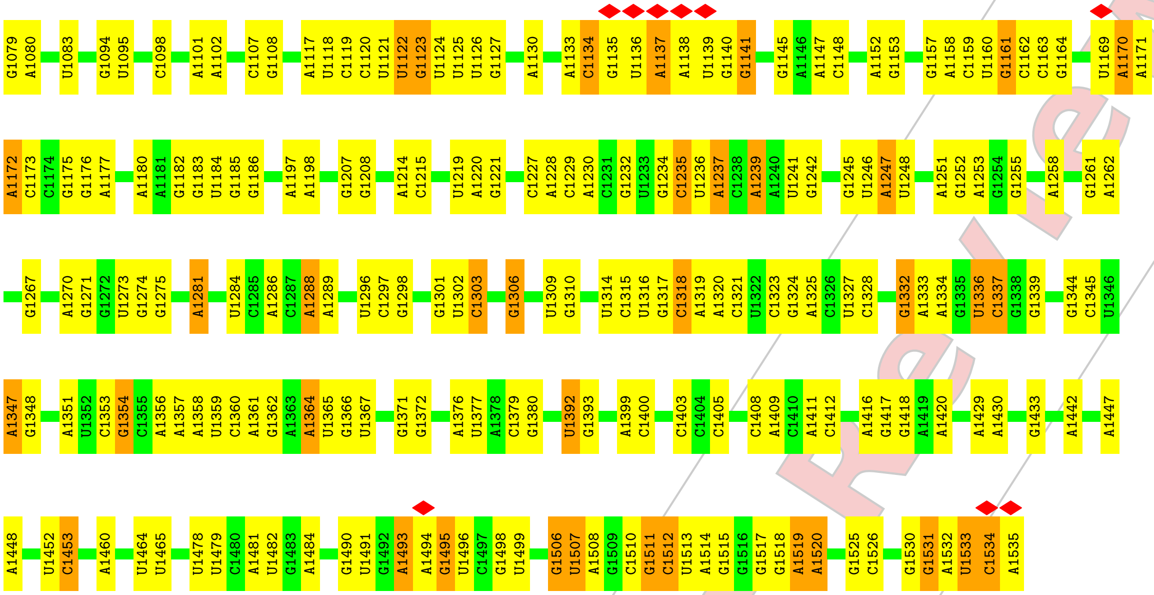

- Molecule 8: Small ribosomal subunit protein uS2

Chain B:

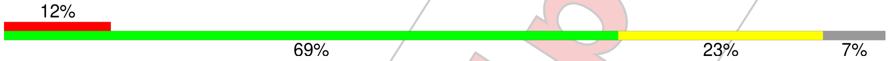

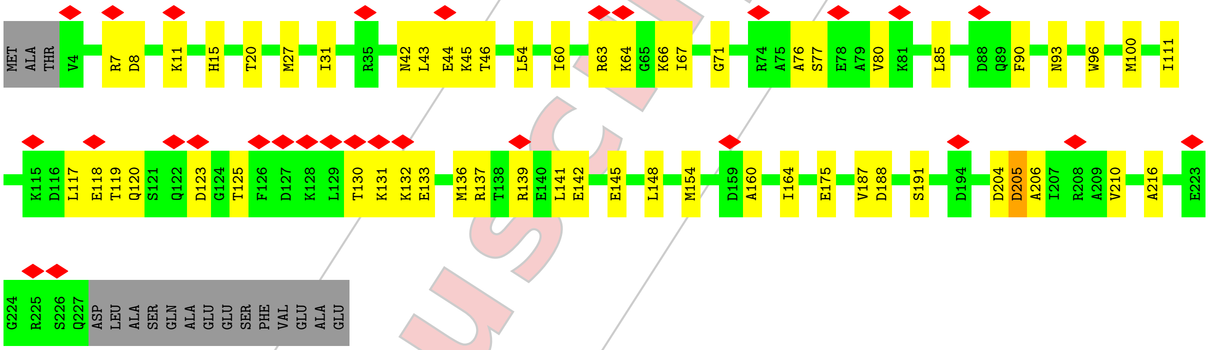

- Molecule 9: Small ribosomal subunit protein uS3

Chain C:

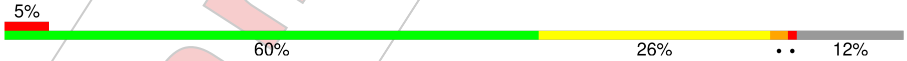

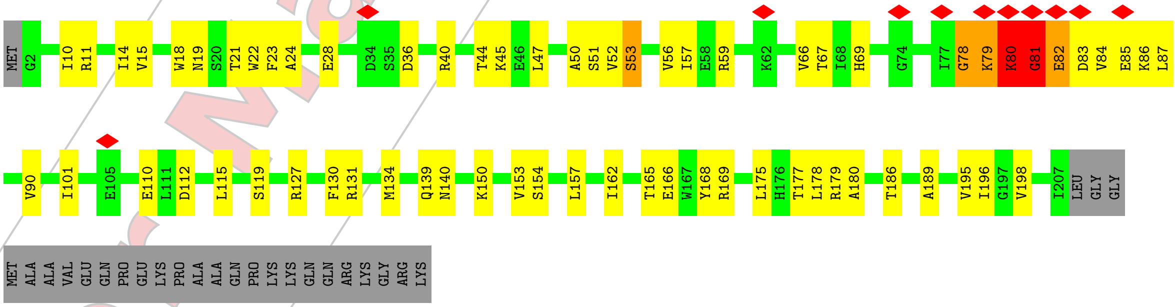

- Molecule 10: Small ribosomal subunit protein uS4

Chain D:

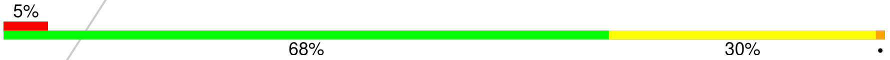

For Manuscript Review

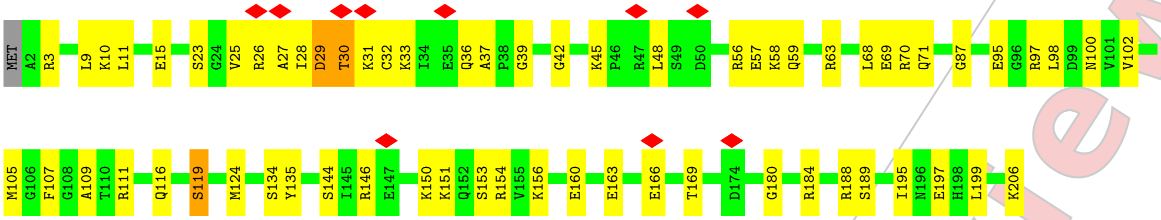

• Molecule 11: Small ribosomal subunit protein uS5

Chain E:

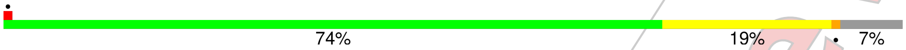

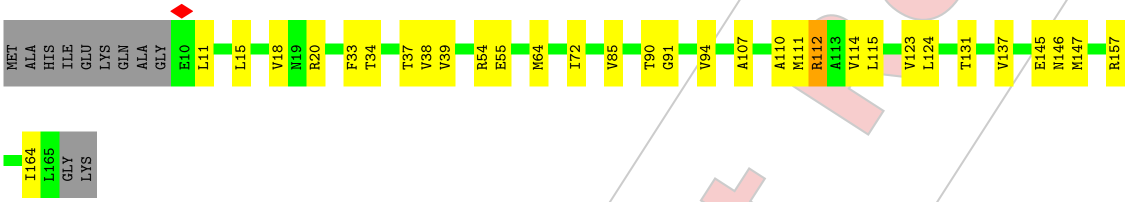

• Molecule 12: Small ribosomal subunit protein bS6

Chain F:

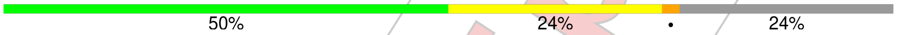

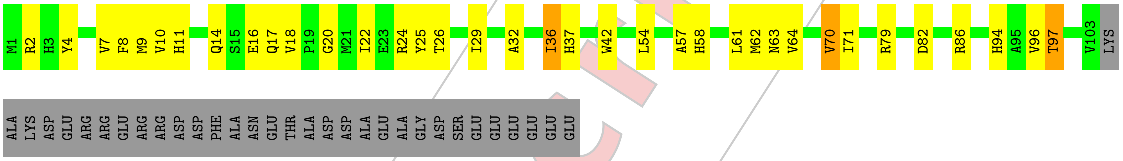

• Molecule 13: Small ribosomal subunit protein uS7

Chain G:

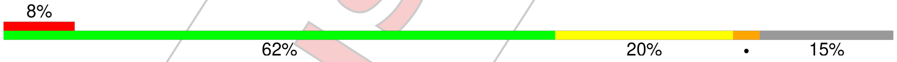

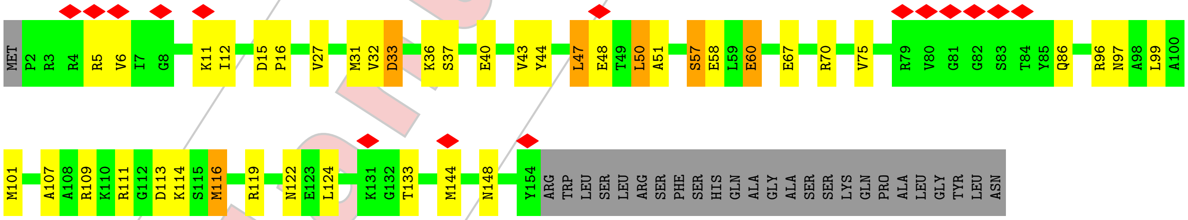

• Molecule 14: Small ribosomal subunit protein uS8

Chain H:

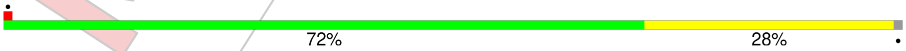

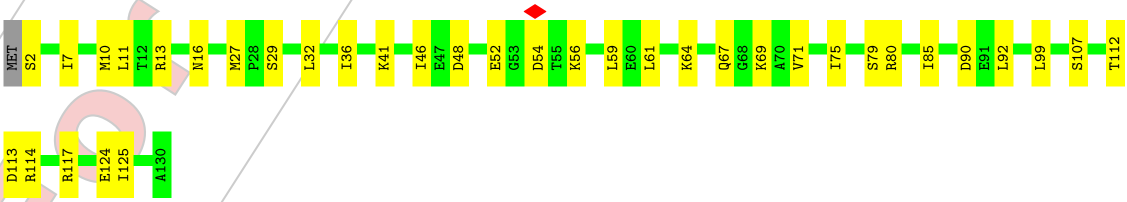

• Molecule 15: Small ribosomal subunit protein uS9

For Manuscript Review

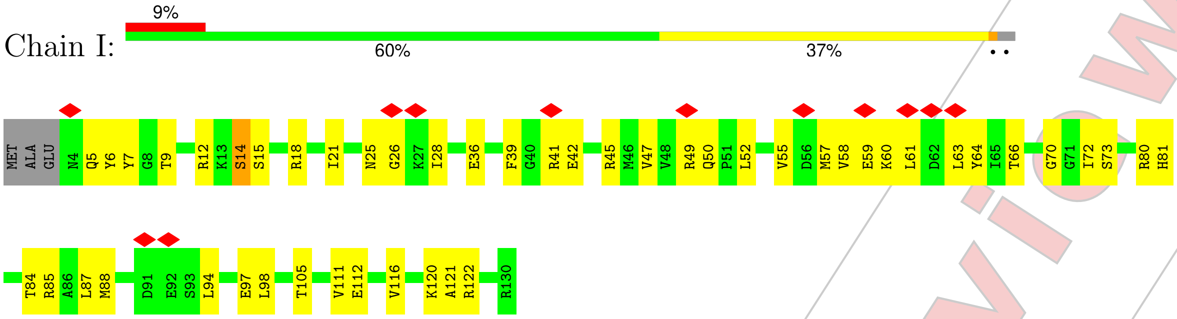

• Molecule 16: Small ribosomal subunit protein uS10

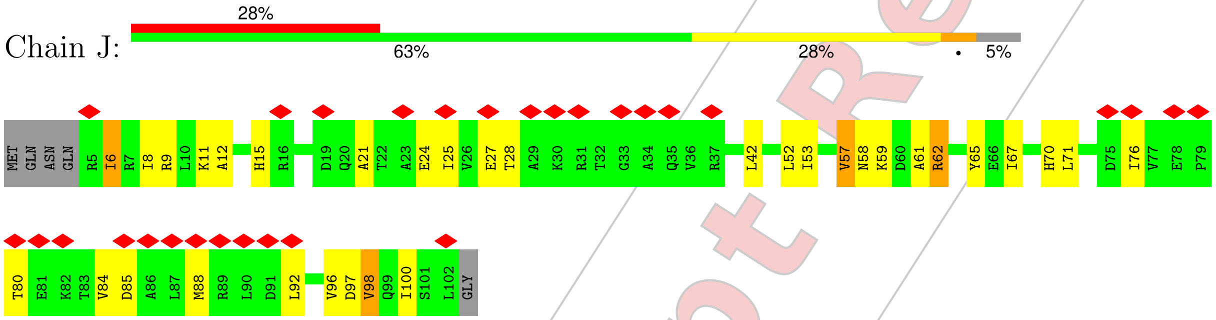

• Molecule 17: Small ribosomal subunit protein uS11

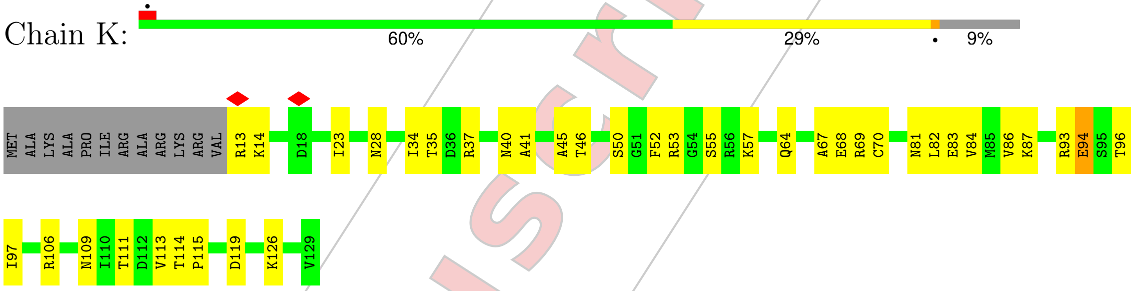

• Molecule 18: Small ribosomal subunit protein uS12

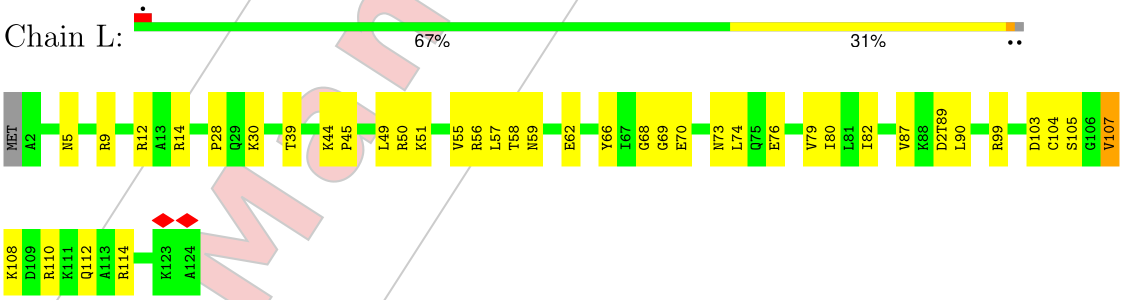

• Molecule 19: Small ribosomal subunit protein uS13

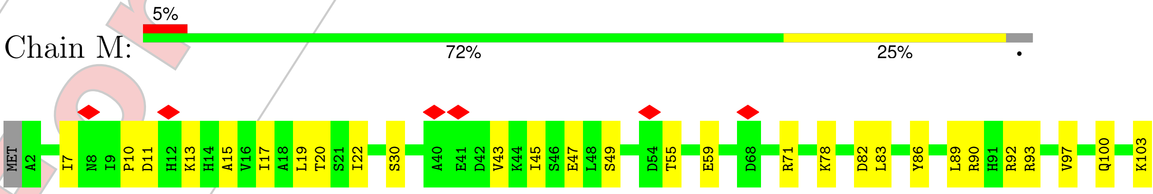

For Manuscript Review

T104 K110 I116 LYS LYS

• Molecule 20: Small ribosomal subunit protein uS14

Chain N: 61% 38%

MET A2 K3 Q4 S5 M6 K7 A8 R9 R13 D18 K19 Y20 R24 L27 K28 A29 I30 I31 S32 D33 E39 D40 R41 V45 L46 K47 R53 D54 S58 R59 Q60 R61 R69 P70 R75 S80 R81 I82 K83 V84 R85 M89 R90 G91 E92 L96

K97 K98 W101

• Molecule 21: Small ribosomal subunit protein uS15

Chain O: 80% 19%

MET S2 L3 K10 I11 V12 S13 E14 D18 A19 N20 D21 T22 L32 I36 K48 R54 Y78 I82 R88 R89

• Molecule 22: Small ribosomal subunit protein bS16

Chain P: 27% 43% 56%

M1 V2 T3 I4 R5 R8 H9 G10 A11 K12 K13 R14 P15 F16 Y17 Q18 V19 V20 V21 R25 R28 R35 V36 G37 F38 F39 N40 P41 I42 A43 S44 E45 K46 E47 E48 G49 T50 R51 L52 D53 L54 D55 R56 I57 A58 H59 W60 Q63 I67 S68 D69 R70 V71 A72

A73 L74 I75 K76 E77 V78 N79 K80 A81 ALA

• Molecule 23: Small ribosomal subunit protein uS17

Chain Q: 61% 31% 6%

MET THR ASP K4 I5 R6 T7 L8 V12 D15 K16 I21 V22 V23 A24 I25 T41 T42 V46 H47 D48 N51 E52 C53 G54 I55 G56 D57 V58 I61 R62 R65 P66 L67 S68 K71 T74 L75 E80 K81 A82 VAL LEU

• Molecule 24: Small ribosomal subunit protein bS18

Chain R: 5% 63% 24% 12%

MET ALA ARG TYR PHE ARG ARG ARG K9 F10 A15 E20 I21 D22 Y23 K24 T34 K38 I39 V40 P41 S42 T45 G46 T47 Q52 R53 Q54 R57 R63 S66 Y70 T71 D72 R73 H74 GLN

• Molecule 25: Small ribosomal subunit protein uS19

For Manuscript Review

Chain S:

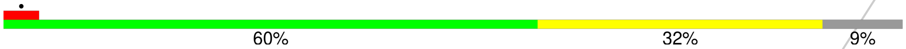

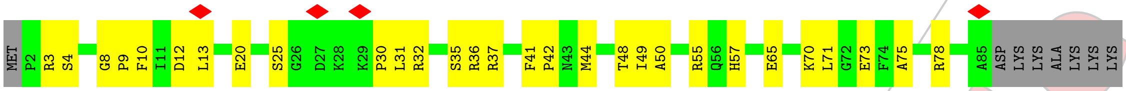

• Molecule 26: Small ribosomal subunit protein bS20

Chain T:

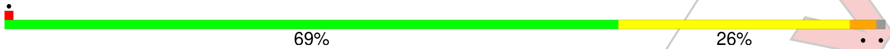

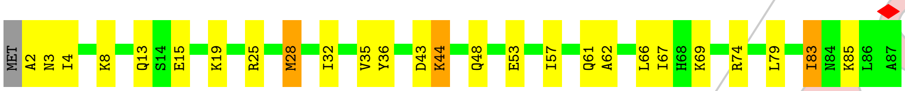

• Molecule 27: Small ribosomal subunit protein bS21

Chain U:

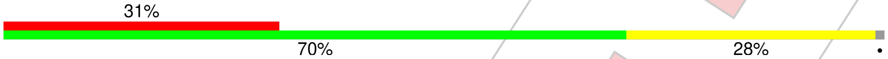

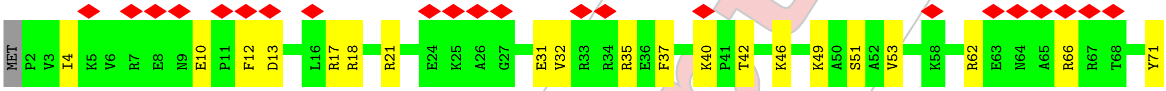

• Molecule 28: 23S ribosomal RNA

Chain a:

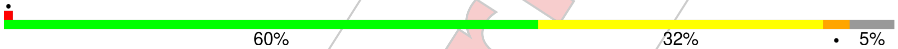

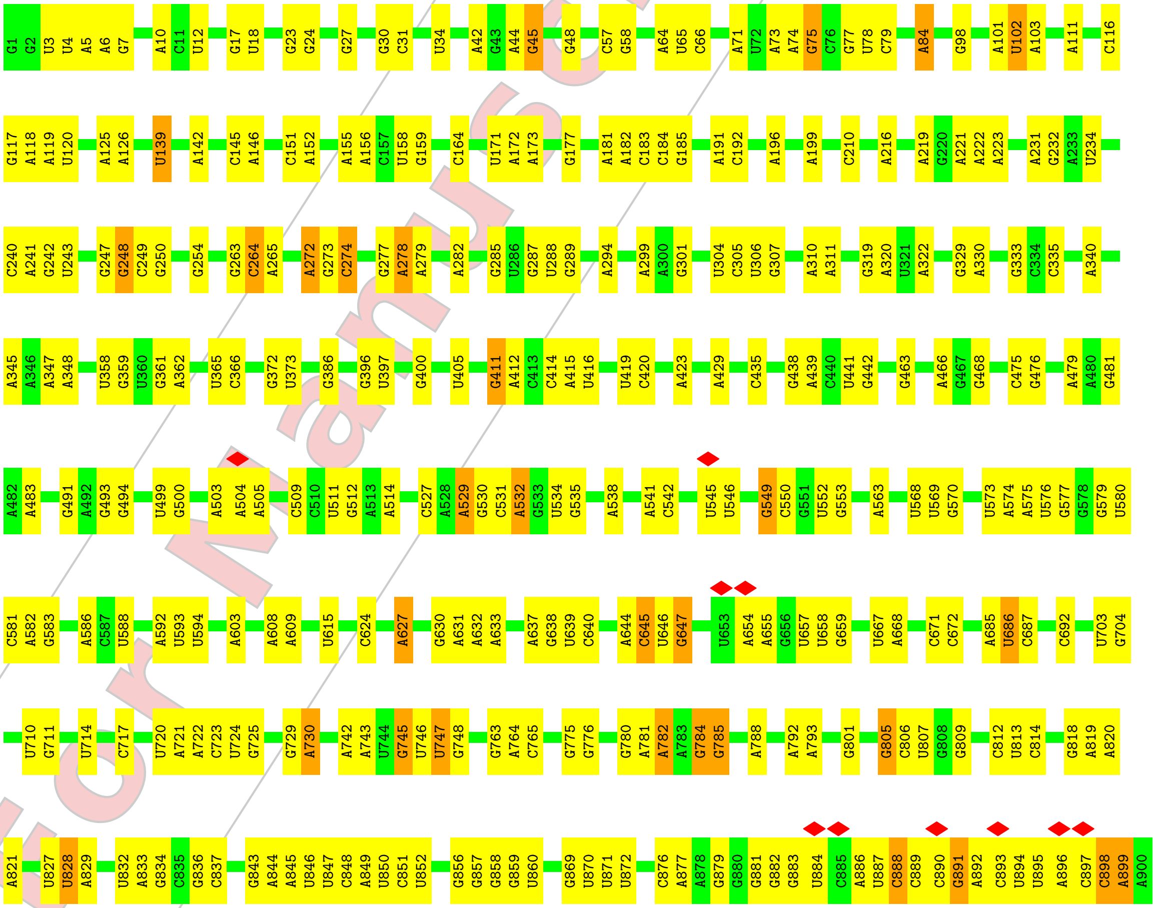

For Manuscript Review

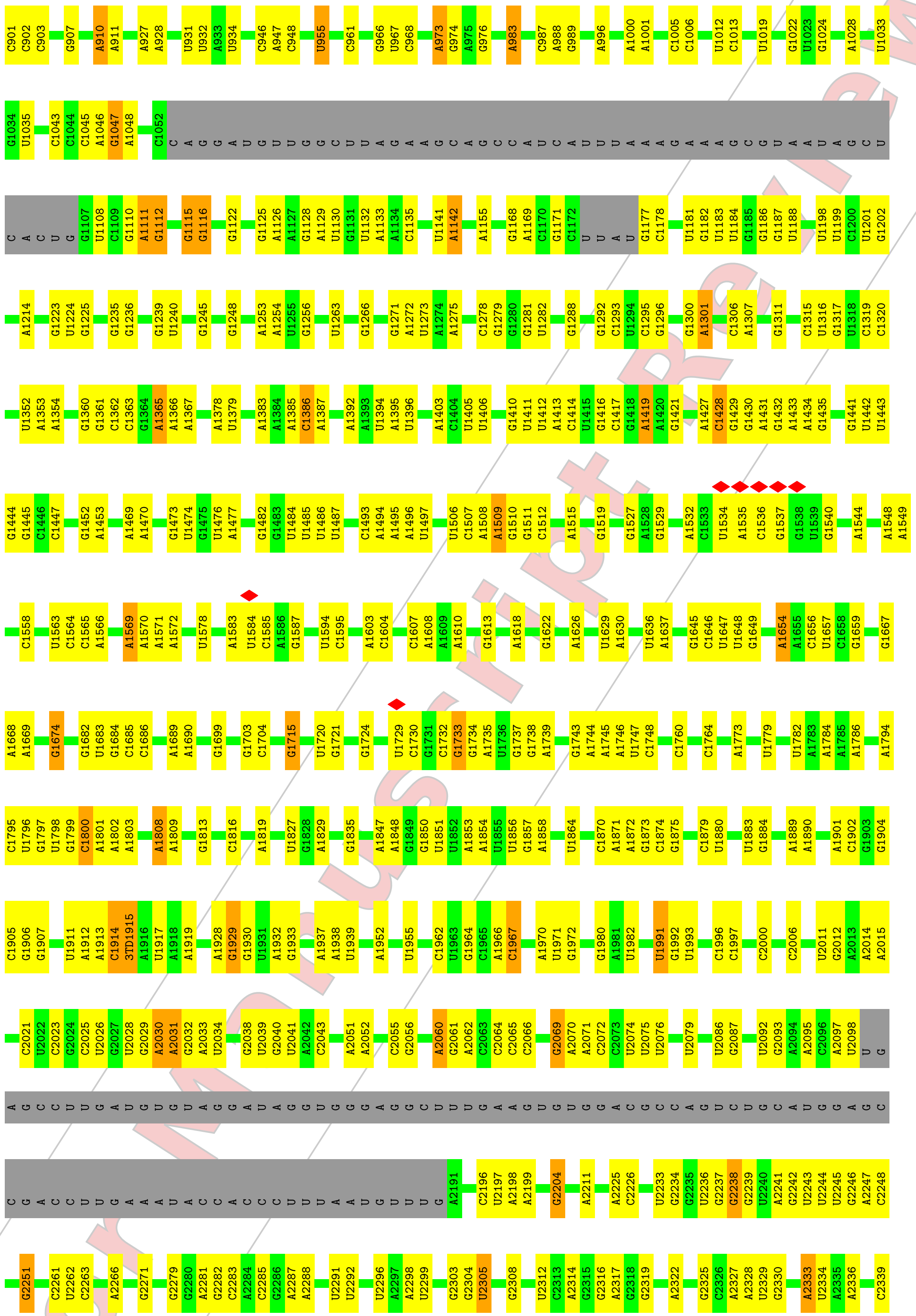

For Manuscript Review

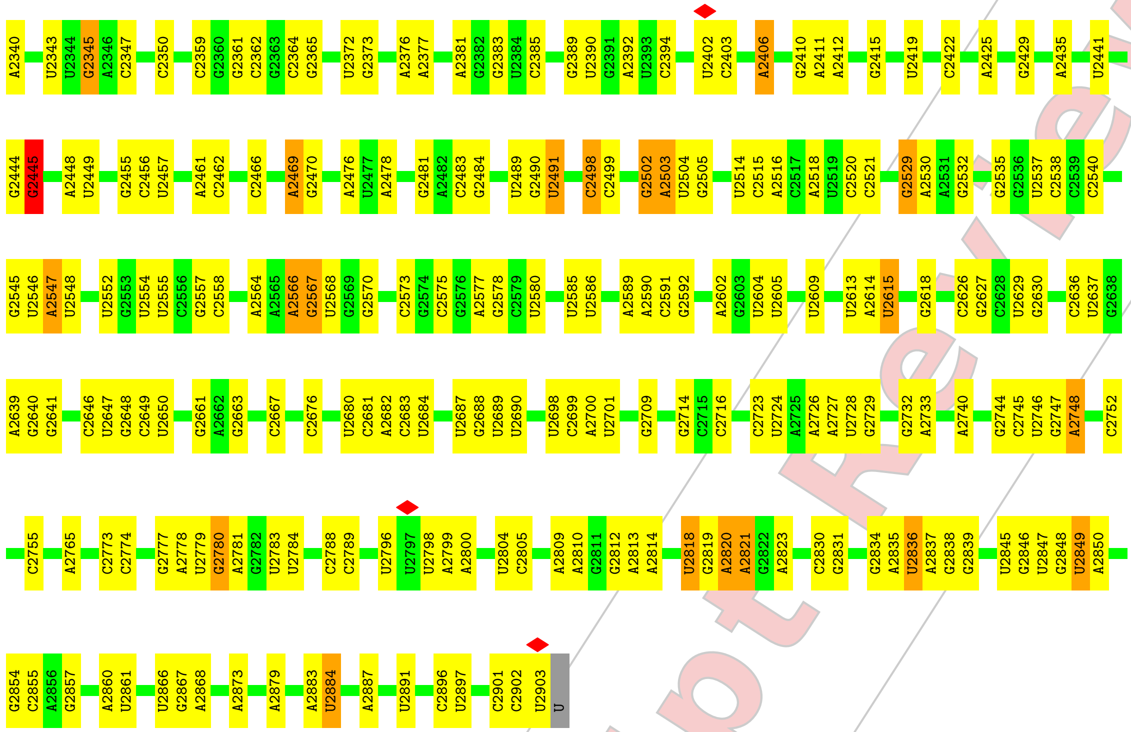

- Molecule 29: 5S ribosomal RNA

Chain b:

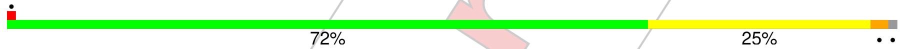

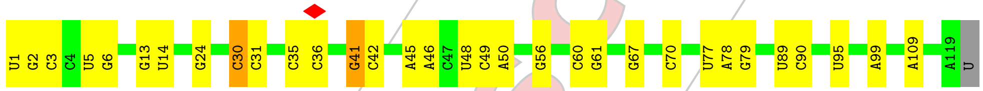

- Molecule 30: 50S ribosomal protein L2

Chain c:

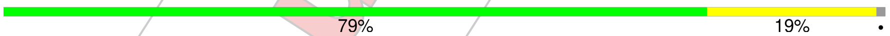

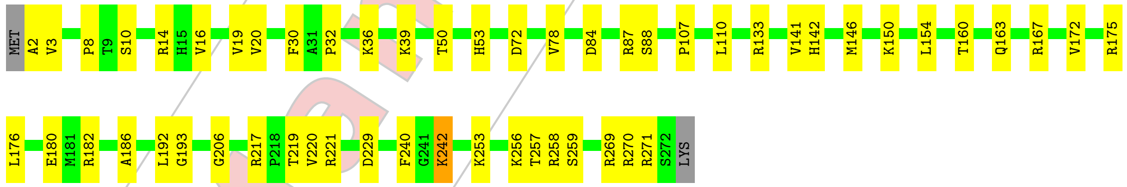

- Molecule 31: 50S ribosomal protein L3

Chain d:

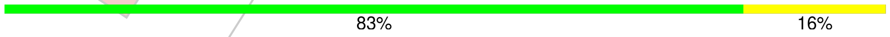

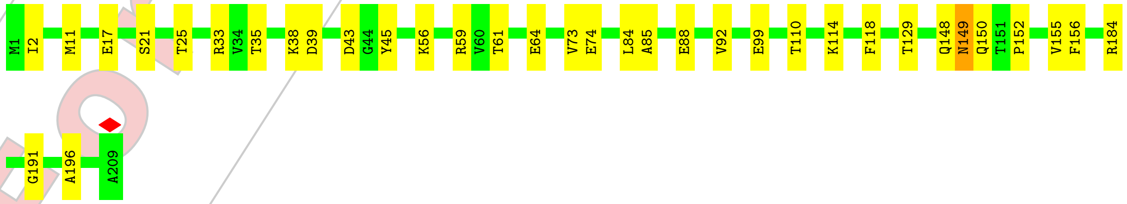

For Manuscript Review

• Molecule 32: Large ribosomal subunit protein uL4

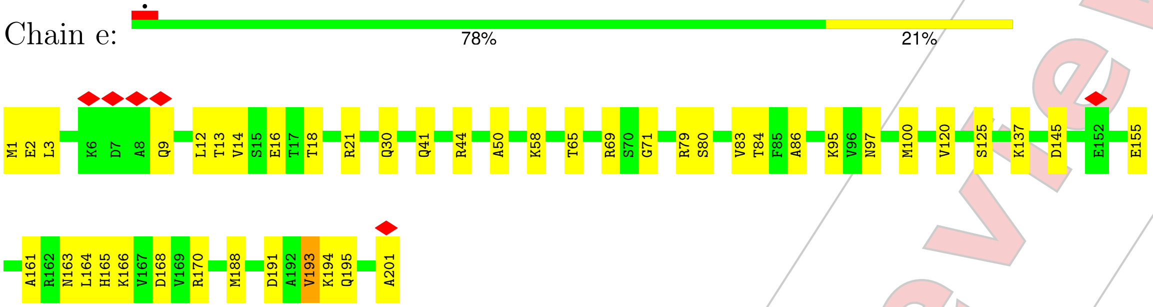

• Molecule 33: Large ribosomal subunit protein uL5

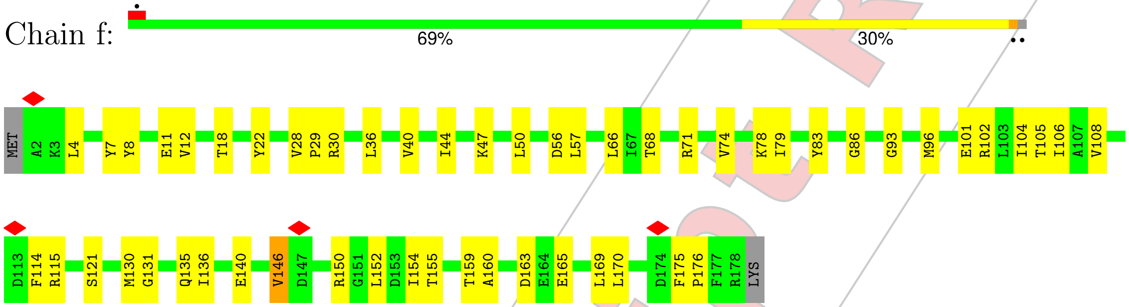

• Molecule 34: Large ribosomal subunit protein uL6

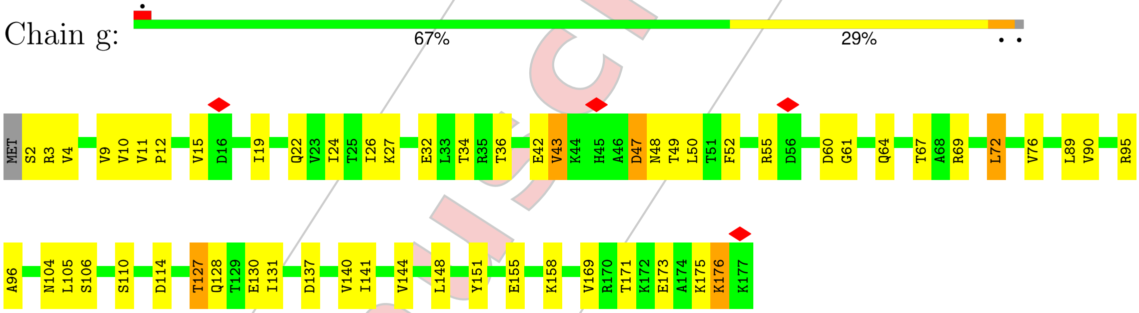

• Molecule 35: Large ribosomal subunit protein bL9

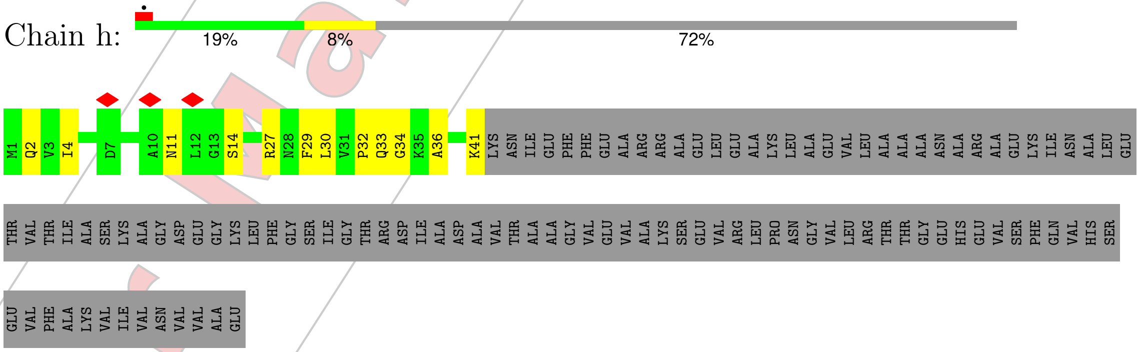

• Molecule 36: Large ribosomal subunit protein uL13

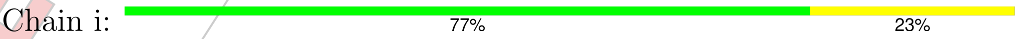

For Manuscript Review

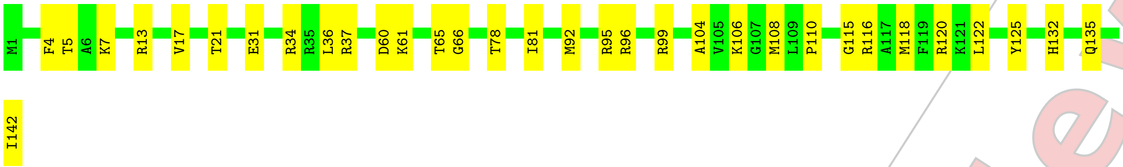

• Molecule 37: Large ribosomal subunit protein uL14

Chain j:

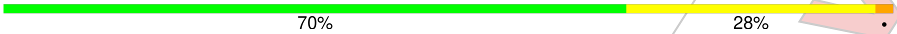

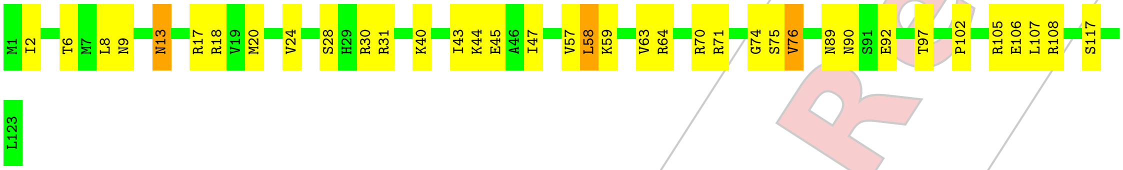

• Molecule 38: Large ribosomal subunit protein uL15

Chain k:

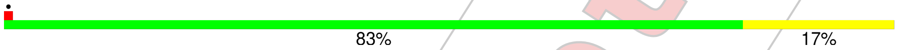

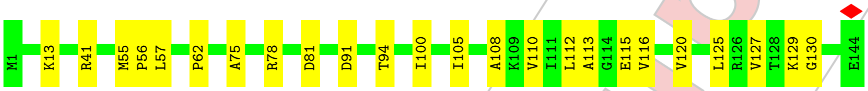

• Molecule 39: Large ribosomal subunit protein uL16

Chain l:

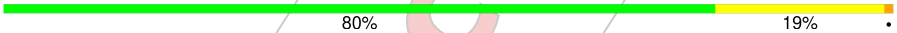

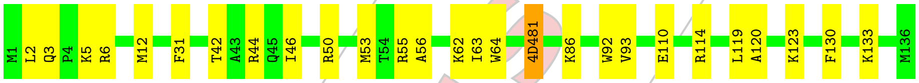

• Molecule 40: Large ribosomal subunit protein bL17

Chain m:

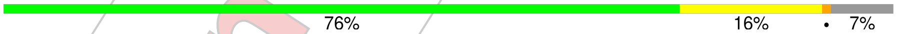

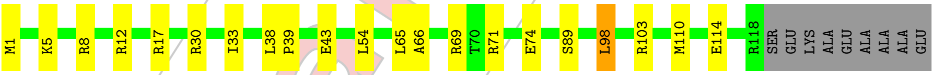

• Molecule 41: Large ribosomal subunit protein uL18

Chain n:

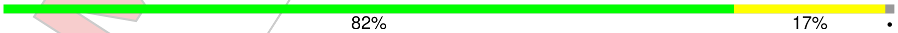

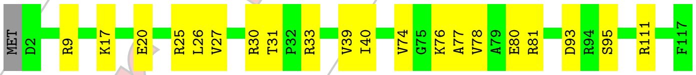

• Molecule 42: Large ribosomal subunit protein bL19

Chain o:

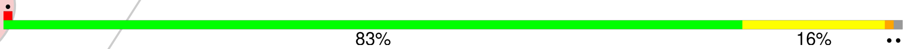

For Manuscript Review

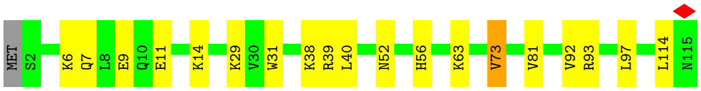

• Molecule 43: 50S ribosomal protein L20

Chain p:
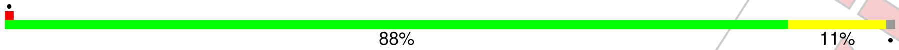

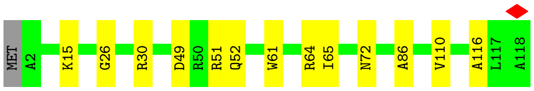

• Molecule 44: Large ribosomal subunit protein bL21

Chain q:
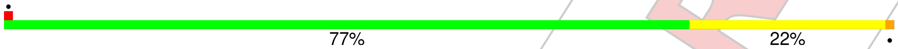

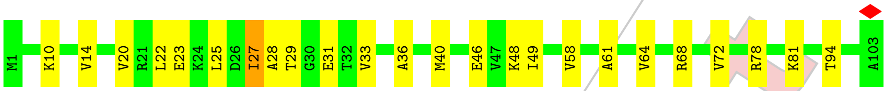

• Molecule 45: Large ribosomal subunit protein uL22

Chain r:
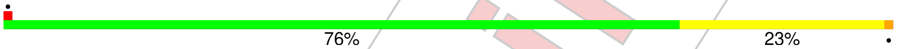

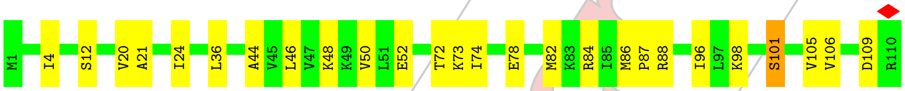

• Molecule 46: 50S ribosomal protein L23

Chain s:
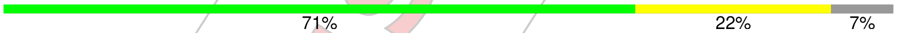

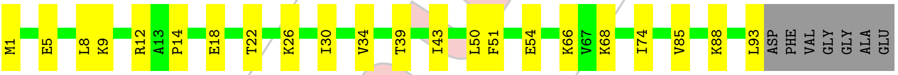

• Molecule 47: 50S ribosomal protein L24

Chain t:
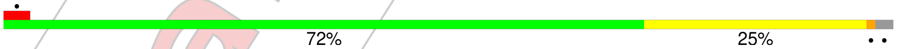

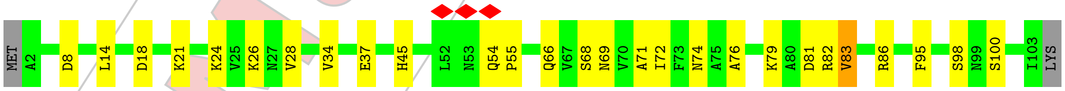

• Molecule 48: Large ribosomal subunit protein bL25

Chain u:
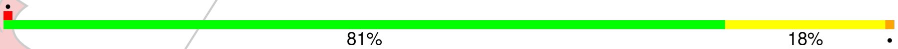

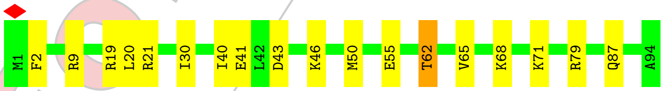

• Molecule 49: 50S ribosomal protein L27

Chain v:

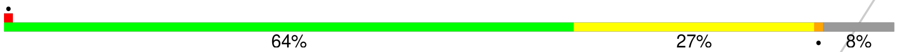

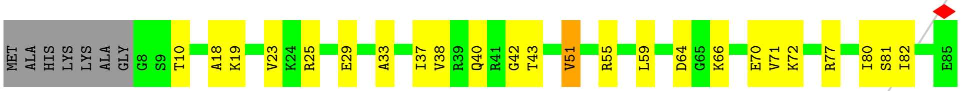

- Molecule 50: 50S ribosomal protein L28

Chain w:

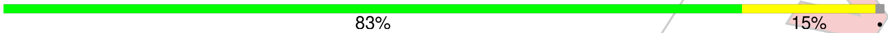

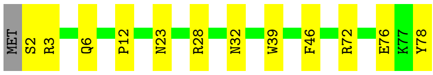

- Molecule 51: Large ribosomal subunit protein uL29

Chain x:

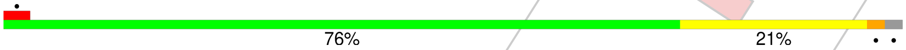

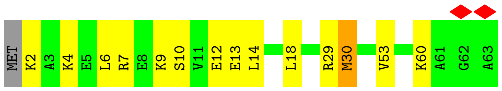

- Molecule 52: 50S ribosomal protein L30

Chain y:

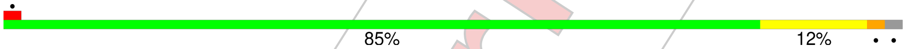

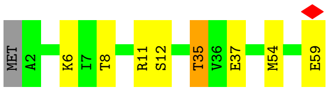

- Molecule 53: 50S ribosomal protein L32

Chain z:

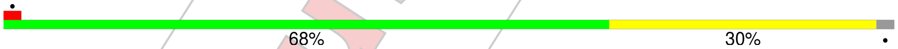

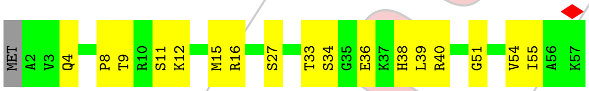

For Manuscript Review

4 Experimental information (i)

Property	Value	Source
EM reconstruction method	SINGLE PARTICLE	Depositor
Imposed symmetry	POINT, Not provided	
Number of particles used	577128	Depositor
Resolution determination method	FSC 0.143 CUT-OFF	Depositor
CTF correction method	PHASE FLIPPING AND AMPLITUDE CORRECTION	Depositor
Microscope	TFS KRIOS	Depositor
Voltage (kV)	300	Depositor
Electron dose $(e^-/\text{Å}^2)$	60	Depositor
Minimum defocus (nm)	500	Depositor
Maximum defocus (nm)	2500	Depositor
Magnification	Not provided	
Image detector	GATAN K3 BIOQUANTUM (6k x 4k)	Depositor
Maximum map value	27.798	Depositor
Minimum map value	-18.382	Depositor
Average map value	0.000	Depositor
Map value standard deviation	1.000	Depositor
Recommended contour level	2.5	Depositor
Map size (Å)	425.984, 425.984, 425.984	wwPDB
Map dimensions	512, 512, 512	wwPDB
Map angles (°)	90.0, 90.0, 90.0	wwPDB
Pixel spacing (Å)	0.832, 0.832, 0.832	Depositor

For Manuscript Review

5 Model quality (i)

5.1 Standard geometry (i)

Bond lengths and bond angles in the following residue types are not validated in this section: 5MC, MS6, 2MA, OMC, MG, IAS, 6MZ, 3TD, SPM, 1MG, ZN, G7M, PSU, UR3, OMU, 4D4, OMG, SPD, MA6, 5MU, MEQ, H2U, 4OC, 2MG, D2T

The Z score for a bond length (or angle) is the number of standard deviations the observed value is removed from the expected value. A bond length (or angle) with $|Z| > 5$ is considered an outlier worth inspection. RMSZ is the root-mean-square of all Z scores of the bond lengths (or angles).

Mol	Chain	Bond lengths		Bond angles	
		RMSZ	#|Z| >5	RMSZ	#|Z| >5
1	0	0.15	0/424	0.31	0/565
2	1	0.28	0/380	0.31	0/498
3	2	0.24	0/513	0.29	0/676
4	3	0.27	0/303	0.32	0/397
5	4	0.18	0/488	0.41	0/649
6	5	0.08	0/46	0.08	0/69
7	A	0.25	2/36260 (0.0%)	0.33	2/56556 (0.0%)
8	B	0.17	0/1784	0.36	0/2403
9	C	0.22	0/1651	0.42	0/2225
10	D	0.20	0/1665	0.40	0/2227
11	E	0.29	0/1165	0.45	0/1568
12	F	0.22	0/858	0.46	0/1160
13	G	0.17	0/1219	0.36	0/1635
14	H	0.21	0/989	0.30	0/1326
15	I	0.21	0/1034	0.45	0/1375
16	J	0.22	0/796	0.41	0/1077
17	K	0.22	0/884	0.39	0/1191
18	L	0.23	0/960	0.38	0/1286
19	M	0.15	0/900	0.32	0/1204
20	N	0.20	0/817	0.41	0/1088
21	O	0.21	0/722	0.36	0/964
22	P	0.26	0/653	0.56	0/877
23	Q	0.24	0/650	0.55	1/871 (0.1%)
24	R	0.20	0/553	0.34	0/742
25	S	0.17	0/685	0.33	0/922
26	T	0.23	0/676	0.39	0/895
27	U	0.16	0/597	0.30	0/792
28	a	0.28	0/65651	0.29	0/102413
29	b	0.21	0/2850	0.23	0/4444
30	c	0.28	0/2121	0.32	0/2852
31	d	0.26	0/1576	0.31	0/2119

For Manuscript Review

Mol	Chain	Bond lengths		Bond angles	
		RMSZ	#|Z| >5	RMSZ	#|Z| >5
32	e	0.22	0/1571	0.31	0/2113
33	f	0.18	0/1434	0.36	0/1926
34	g	0.19	0/1343	0.45	0/1816
35	h	0.19	0/306	0.49	0/413
36	i	0.24	0/1152	0.27	0/1551
37	j	0.27	0/955	0.31	0/1279
38	k	0.24	0/1062	0.31	0/1413
39	l	0.25	0/1073	0.29	0/1433
40	m	0.26	0/958	0.32	0/1281
41	n	0.19	0/902	0.35	0/1209
42	o	0.25	0/929	0.29	0/1242
43	p	0.27	0/960	0.27	0/1278
44	q	0.23	0/829	0.29	0/1107
45	r	0.24	0/864	0.26	0/1156
46	s	0.21	0/744	0.32	0/994
47	t	0.19	0/787	0.38	0/1051
48	u	0.20	0/766	0.29	0/1025
49	v	0.26	0/593	0.30	0/785
50	w	0.25	0/635	0.27	0/848
51	x	0.19	0/502	0.40	0/667
52	y	0.25	0/453	0.32	0/605
53	z	0.25	0/450	0.34	0/599
All	All	0.25	2/149138 (0.0%)	0.32	3/222857 (0.0%)

Chiral center outliers are detected by calculating the chiral volume of a chiral center and verifying if the center is modelled as a planar moiety or with the opposite hand.A planarity outlier is detected by checking planarity of atoms in a peptide group, atoms in a mainchain group or atoms of a sidechain that are expected to be planar.

Mol	Chain	#Chirality outliers	#Planarity outliers
9	C	0	5
11	E	0	1
23	Q	0	1
34	g	0	1
39	l	0	1
All	All	0	9

All (2) bond length outliers are listed below:

Mol	Chain	Res	Type	Atoms	Z	Observed(Å)	Ideal(Å)
7	A	39	G	C2-N2	-11.49	1.11	1.34
7	A	39	G	C6-N1	8.28	1.56	1.39

For Manuscript Review

All (3) bond angle outliers are listed below:

Mol	Chain	Res	Type	Atoms	Z	Observed(^{o})	Ideal(^{o})
7	A	39	G	N1-C2-N2	-28.21	31.57	116.20
7	A	39	G	N3-C2-N2	-10.84	87.38	119.90
23	Q	16	LYS	CA-CB-CG	6.43	126.96	114.10

There are no chirality outliers.

5 of 9 planarity outliers are listed below:

Mol	Chain	Res	Type	Group
9	C	78	GLY	Peptide
9	C	80	LYS	Mainchain,Peptide
9	C	81	GLY	Peptide
9	C	82	GLU	Peptide
11	E	112	ARG	Sidechain

5.2 Too-close contacts (i)

In the following table, the Non-H and H(model) columns list the number of non-hydrogen atoms and hydrogen atoms in the chain respectively. The H(added) column lists the number of hydrogen atoms added and optimized by MolProbity. The Clashes column lists the number of clashes within the asymmetric unit, whereas Symm-Clashes lists symmetry-related clashes.

Mol	Chain	Non-H	H(model)	H(added)	Clashes	Symm-Clashes
1	0	417	0	451	6	0
2	1	377	0	418	8	0
3	2	504	0	572	15	0
4	3	302	0	340	6	0
5	4	480	0	478	20	0
6	5	42	0	23	0	0
7	A	32632	0	16431	474	0
8	B	1753	0	1780	40	0
9	C	1624	0	1696	50	0
10	D	1643	0	1707	46	0
11	E	1152	0	1196	21	0
12	F	839	0	833	26	0
13	G	1203	0	1254	28	0
14	H	979	0	1031	23	0
15	I	1022	0	1070	40	0
16	J	786	0	828	25	0
17	K	877	0	882	28	0
18	L	957	0	1017	29	0

Continued on next page...

For Manuscript Review

Continued from previous page...

Mol	Chain	Non-H	H(model)	H(added)	Clashes	Symm-Clashes
19	M	891	0	952	18	0
20	N	805	0	844	32	0
21	O	714	0	734	13	0
22	P	643	0	661	44	0
23	Q	641	0	682	21	0
24	R	544	0	565	14	0
25	S	668	0	693	22	0
26	T	670	0	719	19	0
27	U	589	0	629	15	0
28	a	59130	0	29769	583	0
29	b	2549	0	1291	20	0
30	c	2082	0	2154	40	0
31	d	1566	0	1618	28	0
32	e	1552	0	1619	29	0
33	f	1410	0	1444	39	0
34	g	1323	0	1371	29	0
35	h	303	0	327	10	0
36	i	1129	0	1162	23	0
37	j	946	0	1023	25	0
38	k	1053	0	1129	19	0
39	l	1075	0	1145	19	0
40	m	945	0	989	11	0
41	n	892	0	923	11	0
42	o	917	0	962	15	0
43	p	947	0	1019	6	0
44	q	816	0	839	14	0
45	r	857	0	922	18	0
46	s	738	0	807	16	0
47	t	779	0	831	18	0
48	u	753	0	780	15	0
49	v	586	0	596	15	0
50	w	625	0	652	8	0
51	x	501	0	531	13	0
52	y	449	0	488	4	0
53	z	444	0	458	15	0
54	3	1	0	0	0	0
54	4	1	0	0	0	0
55	A	91	0	0	0	0
55	D	1	0	0	0	0
55	a	207	0	0	0	0
55	b	5	0	0	0	0
55	c	1	0	0	0	0

Continued on next page...

For Manuscript Review

Continued from previous page...

Mol	Chain	Non-H	H(model)	H(added)	Clashes	Symm-Clashes
55	m	1	0	0	0	0
55	p	1	0	0	0	0
55	z	1	0	0	0	0
56	A	20	0	36	1	0
56	a	140	0	266	11	0
57	a	14	0	26	0	0
All	All	138605	0	93663	1877	0

The all-atom clashscore is defined as the number of clashes found per 1000 atoms (including hydrogen atoms). The all-atom clashscore for this structure is 8.

The worst 5 of 1877 close contacts within the same asymmetric unit are listed below, sorted by their clash magnitude.

Atom-1	Atom-2	Interatomic distance (Å)	Clash overlap (Å)
9:C:80:LYS:O	9:C:82:GLU:N	1.71	1.23
7:A:1135:G:O6	7:A:1141:G:C2	2.21	0.92
24:R:42:SER:HB2	24:R:52:GLN:HG3	1.53	0.90
8:B:131:LYS:O	8:B:131:LYS:NZ	2.05	0.89
7:A:1135:G:C6	7:A:1141:G:N2	2.43	0.87

There are no symmetry-related clashes.

5.3 Torsion angles (i)

5.3.1 Protein backbone (i)

In the following table, the Percentiles column shows the percent Ramachandran outliers of the chain as a percentile score with respect to all PDB entries followed by that with respect to all EM entries.

The Analysed column shows the number of residues for which the backbone conformation was analysed, and the total number of residues.

Mol	Chain	Analysed	Favoured	Allowed	Outliers	Percentiles	
1	0	49/55 (89%)	49 (100%)	0	0	100	100
2	1	44/46 (96%)	43 (98%)	1 (2%)	0	100	100
3	2	62/65 (95%)	60 (97%)	2 (3%)	0	100	100
4	3	36/38 (95%)	35 (97%)	1 (3%)	0	100	100
5	4	56/70 (80%)	56 (100%)	0	0	100	100

Continued on next page...

For Manuscript Review

Continued from previous page...

Mol	Chain	Analysed	Favoured	Allowed	Outliers	Percentiles	
8	B	222/241 (92%)	208 (94%)	14 (6%)	0	100	100
9	C	204/233 (88%)	191 (94%)	11 (5%)	2 (1%)	13	47
10	D	203/206 (98%)	185 (91%)	18 (9%)	0	100	100
11	E	154/167 (92%)	148 (96%)	6 (4%)	0	100	100
12	F	101/135 (75%)	95 (94%)	6 (6%)	0	100	100
13	G	151/179 (84%)	136 (90%)	15 (10%)	0	100	100
14	H	127/130 (98%)	121 (95%)	6 (5%)	0	100	100
15	I	125/130 (96%)	120 (96%)	5 (4%)	0	100	100
16	J	96/103 (93%)	92 (96%)	3 (3%)	1 (1%)	13	47
17	K	113/129 (88%)	105 (93%)	8 (7%)	0	100	100
18	L	120/124 (97%)	113 (94%)	7 (6%)	0	100	100
19	M	113/118 (96%)	109 (96%)	4 (4%)	0	100	100
20	N	98/101 (97%)	94 (96%)	4 (4%)	0	100	100
21	O	86/89 (97%)	84 (98%)	2 (2%)	0	100	100
22	P	79/82 (96%)	65 (82%)	14 (18%)	0	100	100
23	Q	77/84 (92%)	72 (94%)	5 (6%)	0	100	100
24	R	64/75 (85%)	59 (92%)	5 (8%)	0	100	100
25	S	82/92 (89%)	80 (98%)	2 (2%)	0	100	100
26	T	84/87 (97%)	84 (100%)	0	0	100	100
27	U	68/71 (96%)	66 (97%)	2 (3%)	0	100	100
30	c	269/273 (98%)	265 (98%)	4 (2%)	0	100	100
31	d	206/209 (99%)	200 (97%)	5 (2%)	1 (0%)	25	60
32	e	199/201 (99%)	194 (98%)	5 (2%)	0	100	100
33	f	175/179 (98%)	169 (97%)	6 (3%)	0	100	100
34	g	174/177 (98%)	159 (91%)	14 (8%)	1 (1%)	22	57
35	h	39/149 (26%)	34 (87%)	5 (13%)	0	100	100
36	i	140/142 (99%)	139 (99%)	1 (1%)	0	100	100
37	j	121/123 (98%)	116 (96%)	5 (4%)	0	100	100
38	k	142/144 (99%)	139 (98%)	3 (2%)	0	100	100
39	l	132/136 (97%)	131 (99%)	1 (1%)	0	100	100
40	m	116/127 (91%)	109 (94%)	7 (6%)	0	100	100

Continued on next page...

For Manuscript Review

Continued from previous page...

Mol	Chain	Analysed	Favoured	Allowed	Outliers	Percentiles	
41	n	114/117 (97%)	109 (96%)	5 (4%)	0	100	100
42	o	112/115 (97%)	107 (96%)	5 (4%)	0	100	100
43	p	115/118 (98%)	114 (99%)	1 (1%)	0	100	100
44	q	101/103 (98%)	96 (95%)	5 (5%)	0	100	100
45	r	108/110 (98%)	105 (97%)	3 (3%)	0	100	100
46	s	91/100 (91%)	87 (96%)	4 (4%)	0	100	100
47	t	100/104 (96%)	94 (94%)	6 (6%)	0	100	100
48	u	92/94 (98%)	91 (99%)	1 (1%)	0	100	100
49	v	76/85 (89%)	74 (97%)	2 (3%)	0	100	100
50	w	75/78 (96%)	74 (99%)	1 (1%)	0	100	100
51	x	60/63 (95%)	57 (95%)	3 (5%)	0	100	100
52	y	56/59 (95%)	52 (93%)	4 (7%)	0	100	100
53	z	54/57 (95%)	53 (98%)	1 (2%)	0	100	100
All	All	5481/5913 (93%)	5238 (96%)	238 (4%)	5 (0%)	50	80

All (5) Ramachandran outliers are listed below:

Mol	Chain	Res	Type
9	C	81	GLY
31	d	149	ASN
16	J	57	VAL
34	g	47	ASP
9	C	80	LYS

5.3.2 Protein sidechains (i)

In the following table, the Percentiles column shows the percent sidechain outliers of the chain as a percentile score with respect to all PDB entries followed by that with respect to all EM entries.

The Analysed column shows the number of residues for which the sidechain conformation was analysed, and the total number of residues.

Mol	Chain	Analysed	Rotameric	Outliers	Percentiles	
1	0	46/49 (94%)	44 (96%)	2 (4%)	25	57
2	1	38/38 (100%)	38 (100%)	0	100	100

Continued on next page...

For Manuscript Review

Continued from previous page...

Mol	Chain	Analysed	Rotameric	Outliers	Percentiles	
3	2	51/52 (98%)	51 (100%)	0	100	100
4	3	34/34 (100%)	34 (100%)	0	100	100
5	4	55/62 (89%)	53 (96%)	2 (4%)	30	62
8	B	186/199 (94%)	183 (98%)	3 (2%)	58	79
9	C	170/190 (90%)	161 (95%)	9 (5%)	19	52
10	D	172/173 (99%)	161 (94%)	11 (6%)	14	46
11	E	119/126 (94%)	116 (98%)	3 (2%)	42	71
12	F	90/116 (78%)	85 (94%)	5 (6%)	17	50
13	G	126/147 (86%)	115 (91%)	11 (9%)	8	32
14	H	104/105 (99%)	100 (96%)	4 (4%)	28	60
15	I	105/107 (98%)	100 (95%)	5 (5%)	21	55
16	J	86/90 (96%)	82 (95%)	4 (5%)	22	55
17	K	89/98 (91%)	84 (94%)	5 (6%)	17	50
18	L	102/103 (99%)	98 (96%)	4 (4%)	27	60
19	M	93/96 (97%)	87 (94%)	6 (6%)	14	45
20	N	83/84 (99%)	81 (98%)	2 (2%)	44	71
21	O	76/77 (99%)	76 (100%)	0	100	100
22	P	65/65 (100%)	63 (97%)	2 (3%)	35	66
23	Q	73/78 (94%)	71 (97%)	2 (3%)	40	69
24	R	57/65 (88%)	54 (95%)	3 (5%)	19	52
25	S	72/79 (91%)	71 (99%)	1 (1%)	62	82
26	T	65/66 (98%)	61 (94%)	4 (6%)	15	47
27	U	60/61 (98%)	57 (95%)	3 (5%)	20	54
30	c	216/218 (99%)	212 (98%)	4 (2%)	52	76
31	d	163/163 (100%)	158 (97%)	5 (3%)	35	66
32	e	165/165 (100%)	160 (97%)	5 (3%)	36	66
33	f	148/150 (99%)	143 (97%)	5 (3%)	32	63
34	g	137/138 (99%)	124 (90%)	13 (10%)	7	28
35	h	32/114 (28%)	31 (97%)	1 (3%)	35	66
36	i	116/116 (100%)	113 (97%)	3 (3%)	41	70
37	j	104/104 (100%)	98 (94%)	6 (6%)	17	49

Continued on next page...

For Manuscript Review

Continued from previous page...

Mol	Chain	Analysed	Rotameric	Outliers	Percentiles	
38	k	103/103 (100%)	102 (99%)	1 (1%)	73	87
39	l	107/107 (100%)	107 (100%)	0	100	100
40	m	98/103 (95%)	95 (97%)	3 (3%)	35	66
41	n	86/87 (99%)	85 (99%)	1 (1%)	67	85
42	o	99/100 (99%)	98 (99%)	1 (1%)	73	87
43	p	89/90 (99%)	87 (98%)	2 (2%)	47	73
44	q	84/84 (100%)	81 (96%)	3 (4%)	30	62
45	r	93/93 (100%)	92 (99%)	1 (1%)	70	86
46	s	80/84 (95%)	79 (99%)	1 (1%)	65	83
47	t	83/85 (98%)	81 (98%)	2 (2%)	44	71
48	u	78/78 (100%)	76 (97%)	2 (3%)	41	70
49	v	58/63 (92%)	53 (91%)	5 (9%)	8	33
50	w	67/68 (98%)	66 (98%)	1 (2%)	60	81
51	x	54/55 (98%)	53 (98%)	1 (2%)	52	76
52	y	48/49 (98%)	46 (96%)	2 (4%)	25	58
53	z	47/48 (98%)	46 (98%)	1 (2%)	48	74
All	All	4572/4825 (95%)	4412 (96%)	160 (4%)	33	63

5 of 160 residues with a non-rotameric sidechain are listed below:

Mol	Chain	Res	Type
34	g	90	VAL
44	q	72	VAL
34	g	131	ILE
37	j	76	VAL
49	v	10	THR

Sometimes sidechains can be flipped to improve hydrogen bonding and reduce clashes. 5 of 40 such sidechains are listed below:

Mol	Chain	Res	Type
35	h	11	ASN
48	u	75	GLN
36	i	135	GLN
41	n	104	GLN
50	w	36	HIS

For Manuscript Review

5.3.3 RNA (i)

Mol	Chain	Analysed	Backbone Outliers	Pucker Outliers
28	a	2748/2904 (94%)	314 (11%)	0
29	b	118/120 (98%)	12 (10%)	0
6	5	1/2 (50%)	1 (100%)	0
7	A	1517/1532 (99%)	311 (20%)	18 (1%)
All	All	4384/4558 (96%)	638 (14%)	18 (0%)

5 of 638 RNA backbone outliers are listed below:

Mol	Chain	Res	Type
6	5	76	A
7	A	3	A
7	A	4	U
7	A	5	U
7	A	6	G

5 of 18 RNA pucker outliers are listed below:

Mol	Chain	Res	Type
7	A	1031	C
7	A	1533	U
7	A	1506	G
7	A	495	A
7	A	960	U

5.4 Non-standard residues in protein, DNA, RNA chains (i)

40 non-standard protein/DNA/RNA residues are modelled in this entry.

In the following table, the Counts columns list the number of bonds (or angles) for which Mogul statistics could be retrieved, the number of bonds (or angles) that are observed in the model and the number of bonds (or angles) that are defined in the Chemical Component Dictionary. The Link column lists molecule types, if any, to which the group is linked. The Z score for a bond length (or angle) is the number of standard deviations the observed value is removed from the expected value. A bond length (or angle) with $|Z| > 2$ is considered an outlier worth inspection. RMSZ is the root-mean-square of all Z scores of the bond lengths (or angles).

Mol	Type	Chain	Res	Link	Bond lengths			Bond angles		
					Counts	RMSZ	$\#\|Z\| > 2$	Counts	RMSZ	$\#\|Z\| > 2$
28	5MU	a	1939	28	19,22,23	1.22	4 (21%)	27,32,35	2.22	6 (22%)
28	PSU	a	2457	28	18,21,22	1.11	2 (11%)	21,30,33	2.11	5 (23%)
17	IAS	K	119	17	6,7,8	1.75	1 (16%)	3,8,10	3.41	1 (33%)

For Manuscript Review

Mol	Type	Chain	Res	Link	Bond lengths			Bond angles		
					Counts	RMSZ	#|Z| > 2	Counts	RMSZ	#|Z| > 2
28	2MG	a	1835	28	18,26,27	1.38	2 (11%)	16,38,41	1.66	4 (25%)
28	OMU	a	2552	28	19,22,23	1.13	3 (15%)	25,31,34	1.95	6 (24%)
28	PSU	a	2604	28	18,21,22	1.10	2 (11%)	21,30,33	2.02	4 (19%)
39	4D4	l	81	39	9,11,12	2.05	2 (22%)	7,13,15	2.04	3 (42%)
7	2MG	A	1517	7	18,26,27	1.33	2 (11%)	16,38,41	1.70	4 (25%)
28	5MU	a	747	28	19,22,23	1.23	4 (21%)	27,32,35	2.20	6 (22%)
28	5MC	a	1962	28	19,22,23	1.43	2 (10%)	26,32,35	1.19	3 (11%)
7	5MC	A	967	7	19,22,23	1.33	2 (10%)	26,32,35	1.11	2 (7%)
31	MEQ	d	150	31	8,9,10	0.90	0	5,10,12	0.53	0
28	H2U	a	2449	28	18,21,22	0.56	0	19,30,33	1.07	1 (5%)
28	OMC	a	2498	55,28	19,22,23	1.02	1 (5%)	25,31,34	1.17	2 (8%)
7	2MG	A	1208	7	18,26,27	1.29	2 (11%)	16,38,41	1.49	3 (18%)
7	UR3	A	1499	7	19,22,23	3.83	6 (31%)	26,32,35	4.61	10 (38%)
28	2MA	a	2503	55,28	18,25,26	1.28	1 (5%)	20,37,40	2.01	2 (10%)
28	1MG	a	745	28	19,26,27	1.41	3 (15%)	18,39,42	1.43	3 (16%)
28	PSU	a	2580	28	18,21,22	1.18	3 (16%)	21,30,33	2.05	5 (23%)
28	PSU	a	955	28	18,21,22	1.13	3 (16%)	21,30,33	2.01	4 (19%)
7	4OC	A	1403	7	20,23,24	2.39	4 (20%)	25,32,35	1.00	1 (4%)
7	G7M	A	527	7	20,26,27	0.93	1 (5%)	16,39,42	0.58	0
39	MS6	l	82	39	5,7,8	0.57	0	2,7,9	1.21	0
28	PSU	a	2504	28	18,21,22	1.05	2 (11%)	21,30,33	2.00	4 (19%)
28	2MG	a	2445	28	18,26,27	1.40	2 (11%)	16,38,41	1.67	4 (25%)
28	3TD	a	1915	28	19,22,23	1.14	2 (10%)	23,32,35	1.89	3 (13%)
7	MA6	A	1520	7	19,26,27	1.93	4 (21%)	18,38,41	1.26	2 (11%)
28	6MZ	a	1618	28	17,25,26	1.29	3 (17%)	15,36,39	2.27	3 (20%)
28	PSU	a	2605	28	18,21,22	1.14	3 (16%)	21,30,33	1.97	4 (19%)
28	OMG	a	2251	28	19,26,27	1.30	2 (10%)	21,38,41	1.44	4 (19%)
7	PSU	A	516	7,55	18,21,22	1.08	2 (11%)	21,30,33	1.99	5 (23%)
18	D2T	L	89	18	8,9,10	1.94	2 (25%)	6,11,13	1.48	1 (16%)
28	PSU	a	746	55,28	18,21,22	1.07	2 (11%)	21,30,33	1.94	4 (19%)
28	PSU	a	1911	28	18,21,22	0.99	1 (5%)	21,30,33	2.02	4 (19%)
7	MA6	A	1519	7	19,26,27	1.93	4 (21%)	18,38,41	1.25	2 (11%)
7	2MG	A	966	7	18,26,27	1.28	2 (11%)	16,38,41	1.68	5 (31%)
7	5MC	A	1408	7	19,22,23	1.49	2 (10%)	26,32,35	1.10	3 (11%)
28	6MZ	a	2030	28	17,25,26	1.32	3 (17%)	15,36,39	2.49	4 (26%)

For Manuscript Review

Mol	Type	Chain	Res	Link	Bond lengths			Bond angles		
					Counts	RMSZ	#|Z| > 2	Counts	RMSZ	#|Z| > 2
28	G7M	a	2069	28	20,26,27	0.98	1 (5%)	16,39,42	0.67	0
28	PSU	a	1917	28	18,21,22	1.03	2 (11%)	21,30,33	1.92	4 (19%)

In the following table, the Chirals column lists the number of chiral outliers, the number of chiral centers analysed, the number of these observed in the model and the number defined in the Chemical Component Dictionary. Similar counts are reported in the Torsion and Rings columns. '-' means no outliers of that kind were identified.

Mol	Type	Chain	Res	Link	Chirals	Torsions	Rings
28	5MU	a	1939	28	-	0/7/25/26	0/2/2/2
28	PSU	a	2457	28	-	0/7/25/26	0/2/2/2
17	IAS	K	119	17	-	3/7/7/8	-
28	2MG	a	1835	28	-	2/5/27/28	0/3/3/3
28	OMU	a	2552	28	-	1/9/27/28	0/2/2/2
28	PSU	a	2604	28	-	0/7/25/26	0/2/2/2
39	4D4	l	81	39	-	2/11/12/14	-
7	2MG	A	1517	7	-	0/5/27/28	0/3/3/3
28	5MU	a	747	28	-	0/7/25/26	0/2/2/2
28	5MC	a	1962	28	-	0/7/25/26	0/2/2/2
7	5MC	A	967	7	-	0/7/25/26	0/2/2/2
31	MEQ	d	150	31	-	2/8/9/11	-
28	H2U	a	2449	28	-	1/7/38/39	0/2/2/2
28	OMC	a	2498	55,28	-	0/9/27/28	0/2/2/2
7	2MG	A	1208	7	-	0/5/27/28	0/3/3/3
7	UR3	A	1499	7	-	0/7/25/26	0/2/2/2
28	2MA	a	2503	55,28	-	1/3/25/26	0/3/3/3
28	1MG	a	745	28	-	0/3/25/26	0/3/3/3
28	PSU	a	2580	28	-	2/7/25/26	0/2/2/2
28	PSU	a	955	28	-	0/7/25/26	0/2/2/2
7	4OC	A	1403	7	-	0/9/29/30	0/2/2/2
7	G7M	A	527	7	-	2/3/25/26	0/3/3/3
39	MS6	l	82	39	-	4/4/6/8	-
28	PSU	a	2504	28	-	2/7/25/26	0/2/2/2
28	2MG	a	2445	28	-	2/5/27/28	0/3/3/3
28	3TD	a	1915	28	-	2/7/25/26	0/2/2/2
7	MA6	A	1520	7	-	2/7/29/30	0/3/3/3
28	6MZ	a	1618	28	-	2/5/27/28	0/3/3/3
28	PSU	a	2605	28	-	0/7/25/26	0/2/2/2
28	OMG	a	2251	28	-	0/5/27/28	0/3/3/3

Continued on next page...

For Manuscript Review

Continued from previous page...

Mol	Type	Chain	Res	Link	Chirals	Torsions	Rings
7	PSU	A	516	7,55	-	0/7/25/26	0/2/2/2
18	D2T	L	89	18	-	1/7/12/14	-
28	PSU	a	746	55,28	-	1/7/25/26	0/2/2/2
28	PSU	a	1911	28	-	0/7/25/26	0/2/2/2
7	MA6	A	1519	7	-	0/7/29/30	0/3/3/3
7	2MG	A	966	7	-	2/5/27/28	0/3/3/3
7	5MC	A	1408	7	-	2/7/25/26	0/2/2/2
28	6MZ	a	2030	28	-	1/5/27/28	0/3/3/3
28	G7M	a	2069	28	-	1/3/25/26	0/3/3/3
28	PSU	a	1917	28	-	0/7/25/26	0/2/2/2

The worst 5 of 89 bond length outliers are listed below:

Mol	Chain	Res	Type	Atoms	Z	Observed(Å)	Ideal(Å)
7	A	1499	UR3	C2-N1	13.84	1.57	1.38
7	A	1403	4OC	O2-C2	8.42	1.39	1.23
7	A	1499	UR3	C6-N1	5.91	1.52	1.38
7	A	1499	UR3	C2-N3	5.34	1.49	1.39
7	A	1519	MA6	C6-C5	-5.08	1.37	1.44

The worst 5 of 131 bond angle outliers are listed below:

Mol	Chain	Res	Type	Atoms	Z	Observed(^{o})	Ideal(^{o})
7	A	1499	UR3	C4-N3-C2	-14.52	112.89	124.58
7	A	1499	UR3	C6-N1-C2	-14.12	110.26	121.80
7	A	1499	UR3	C5-C4-N3	7.71	125.20	115.04
28	a	1915	3TD	N1-C2-N3	6.67	120.98	116.13
28	a	2503	2MA	C2-N3-C4	5.97	120.28	115.46

There are no chirality outliers.

5 of 38 torsion outliers are listed below:

Mol	Chain	Res	Type	Atoms
7	A	966	2MG	O4'-C4'-C5'-O5'
7	A	1520	MA6	O4'-C4'-C5'-O5'
39	l	81	4D4	NE-CD-CG-CB
17	K	119	IAS	N-CA-CB-CG
28	a	1618	6MZ	N1-C6-N6-C9

There are no ring outliers.

8 monomers are involved in 10 short contacts:

For Manuscript Review

Mol	Chain	Res	Type	Clashes	Symm-Clashes
31	d	150	MEQ	1	0
28	a	2503	2MA	1	0
28	a	745	1MG	1	0
28	a	955	PSU	1	0
28	a	2445	2MG	1	0
28	a	1915	3TD	1	0
28	a	2251	OMG	1	0
28	a	2030	6MZ	3	0

5.5 Carbohydrates (i)

There are no oligosaccharides in this entry.

5.6 Ligand geometry (i)

Of 327 ligands modelled in this entry, 310 are monoatomic - leaving 17 for Mogul analysis.

In the following table, the Counts columns list the number of bonds (or angles) for which Mogul statistics could be retrieved, the number of bonds (or angles) that are observed in the model and the number of bonds (or angles) that are defined in the Chemical Component Dictionary. The Link column lists molecule types, if any, to which the group is linked. The Z score for a bond length (or angle) is the number of standard deviations the observed value is removed from the expected value. A bond length (or angle) with $|Z| > 2$ is considered an outlier worth inspection. RMSZ is the root-mean-square of all Z scores of the bond lengths (or angles).

Mol	Type	Chain	Res	Link	Bond lengths			Bond angles		
					Counts	RMSZ	$\#\|Z\| > 2$	Counts	RMSZ	$\#\|Z\| > 2$
56	SPD	A	1692	-	9,9,9	0.32	0	8,8,8	0.83	0
56	SPD	a	6210	-	9,9,9	0.32	0	8,8,8	0.85	0
56	SPD	A	1693	7	9,9,9	0.33	0	8,8,8	0.75	0
56	SPD	a	6207	-	9,9,9	0.32	0	8,8,8	0.85	0
56	SPD	a	6211	-	9,9,9	0.31	0	8,8,8	0.75	0
56	SPD	a	6214	-	9,9,9	0.32	0	8,8,8	0.86	0
57	SPM	a	6221	-	13,13,13	0.35	0	12,12,12	0.92	0
56	SPD	a	6218	-	9,9,9	0.30	0	8,8,8	0.90	0
56	SPD	a	6215	-	9,9,9	0.33	0	8,8,8	0.88	0
56	SPD	a	6217	-	9,9,9	0.32	0	8,8,8	0.81	0
56	SPD	a	6219	-	9,9,9	0.32	0	8,8,8	0.83	0
56	SPD	a	6212	-	9,9,9	0.33	0	8,8,8	0.82	0
56	SPD	a	6213	-	9,9,9	0.32	0	8,8,8	0.79	0
56	SPD	a	6220	-	9,9,9	0.33	0	8,8,8	0.79	0
56	SPD	a	6208	-	9,9,9	0.31	0	8,8,8	0.73	0
56	SPD	a	6216	-	9,9,9	0.33	0	8,8,8	0.88	0

For Manuscript Review

Mol	Type	Chain	Res	Link	Bond lengths			Bond angles		
					Counts	RMSZ	#|Z| > 2	Counts	RMSZ	#|Z| > 2
56	SPD	a	6209	-	9,9,9	0.32	0	8,8,8	0.77	0

In the following table, the Chirals column lists the number of chiral outliers, the number of chiral centers analysed, the number of these observed in the model and the number defined in the Chemical Component Dictionary. Similar counts are reported in the Torsion and Rings columns. '-' means no outliers of that kind were identified.

Mol	Type	Chain	Res	Link	Chirals	Torsions	Rings
56	SPD	A	1692	-	-	2/7/7/7	-
56	SPD	a	6210	-	-	1/7/7/7	-
56	SPD	A	1693	7	-	2/7/7/7	-
56	SPD	a	6207	-	-	5/7/7/7	-
56	SPD	a	6211	-	-	3/7/7/7	-
56	SPD	a	6214	-	-	4/7/7/7	-
57	SPM	a	6221	-	-	3/11/11/11	-
56	SPD	a	6218	-	-	5/7/7/7	-
56	SPD	a	6215	-	-	4/7/7/7	-
56	SPD	a	6217	-	-	2/7/7/7	-
56	SPD	a	6219	-	-	2/7/7/7	-
56	SPD	a	6212	-	-	2/7/7/7	-
56	SPD	a	6213	-	-	1/7/7/7	-
56	SPD	a	6220	-	-	2/7/7/7	-
56	SPD	a	6208	-	-	5/7/7/7	-
56	SPD	a	6216	-	-	4/7/7/7	-
56	SPD	a	6209	-	-	3/7/7/7	-

There are no bond length outliers.

There are no bond angle outliers.

There are no chirality outliers.

5 of 50 torsion outliers are listed below:

Mol	Chain	Res	Type	Atoms
56	a	6214	SPD	N6-C7-C8-C9
56	A	1692	SPD	C3-C4-C5-N6
56	a	6217	SPD	C3-C4-C5-N6
56	a	6218	SPD	N6-C7-C8-C9
56	a	6220	SPD	N6-C7-C8-C9

For Manuscript Review

There are no ring outliers.

8 monomers are involved in 12 short contacts:

Mol	Chain	Res	Type	Clashes	Symm-Clashes
56	A	1692	SPD	1	0
56	a	6211	SPD	1	0
56	a	6214	SPD	1	0
56	a	6218	SPD	1	0
56	a	6215	SPD	1	0
56	a	6217	SPD	2	0
56	a	6212	SPD	2	0
56	a	6208	SPD	3	0

5.7 Other polymers (i)

There are no such residues in this entry.

5.8 Polymer linkage issues (i)

The following chains have linkage breaks:

Mol	Chain	Number of breaks
28	a	1

All chain breaks are listed below:

Model	Chain	Residue-1	Atom-1	Residue-2	Atom-2	Distance (Å)
1	a	1914:C	O3'	1915:3TD	P	4.29

For Manuscript Review

6 Map visualisation (i)

This section contains visualisations of the EMDB entry EMD-75650. These allow visual inspection of the internal detail of the map and identification of artifacts.

Images derived from a raw map, generated by summing the deposited half-maps, are presented below the corresponding image components of the primary map to allow further visual inspection and comparison with those of the primary map.

6.1 Orthogonal projections (i)

6.1.1 Primary map

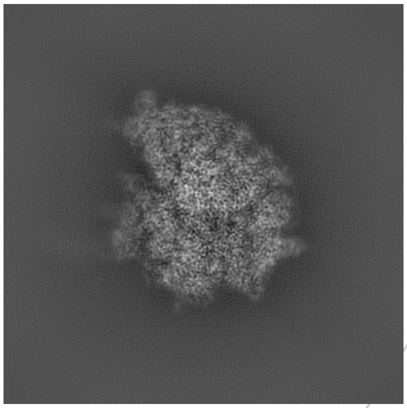
X

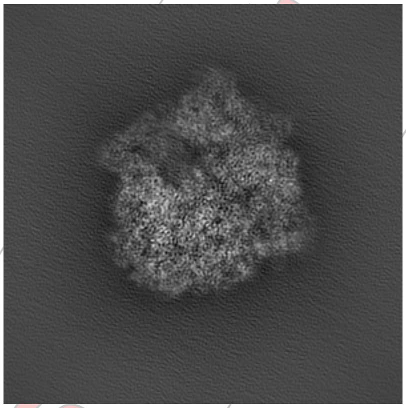
Y

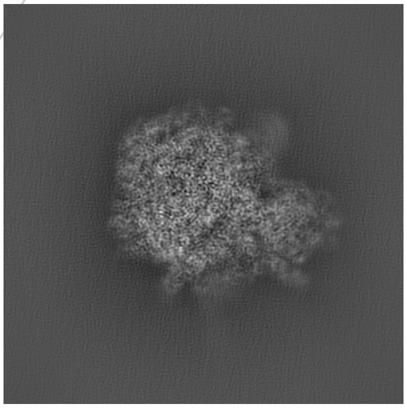
Z

6.1.2 Raw map

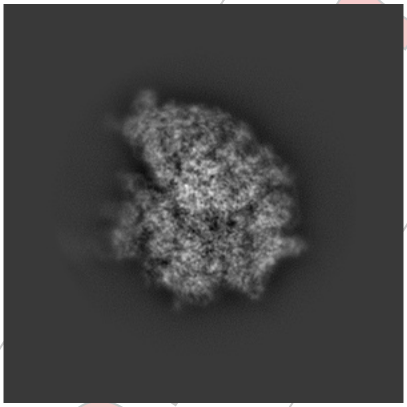
X

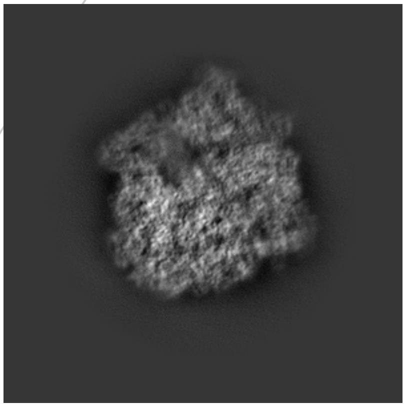
Y

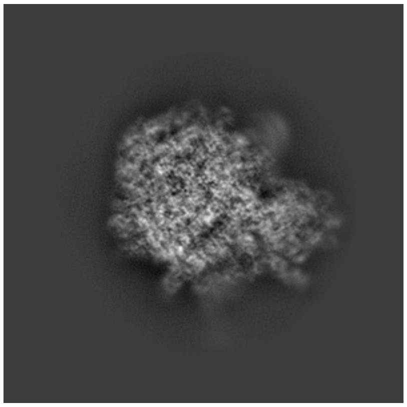
Z

The images above show the map projected in three orthogonal directions.

For Manuscript Review

6.2 Central slices (i)

6.2.1 Primary map

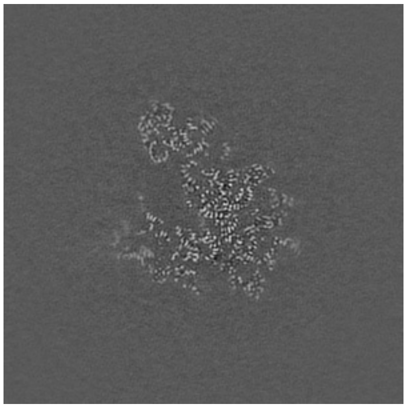
X Index: 256

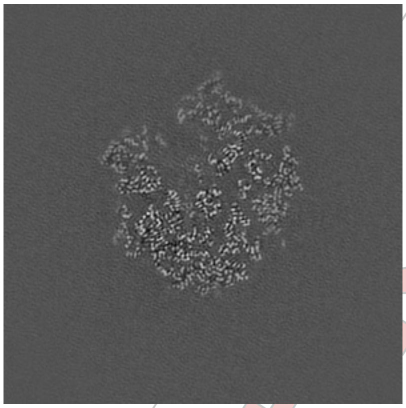
Y Index: 256

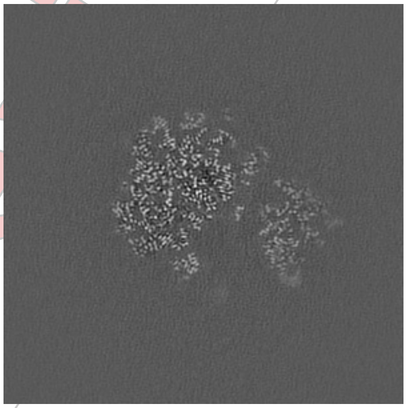
Z Index: 256

6.2.2 Raw map

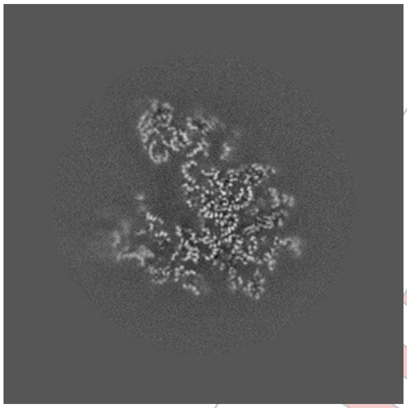
X Index: 256

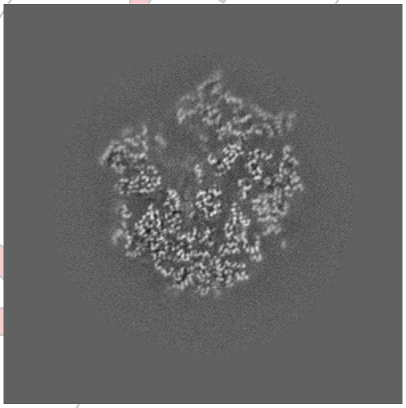
Y Index: 256

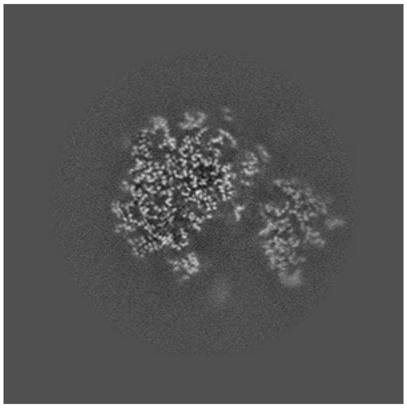
Z Index: 256

The images above show central slices of the map in three orthogonal directions.

For Manuscript Review

6.3 Largest variance slices (i)

6.3.1 Primary map

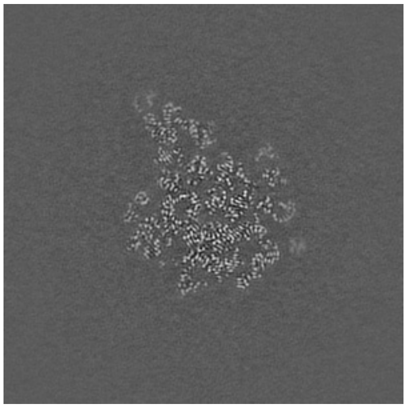
X Index: 234

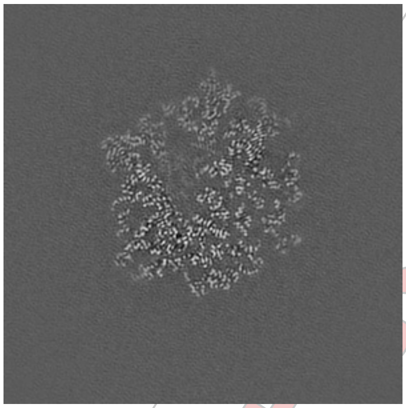
Y Index: 247

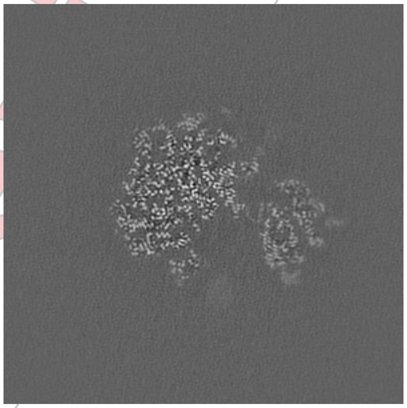
Z Index: 253

6.3.2 Raw map

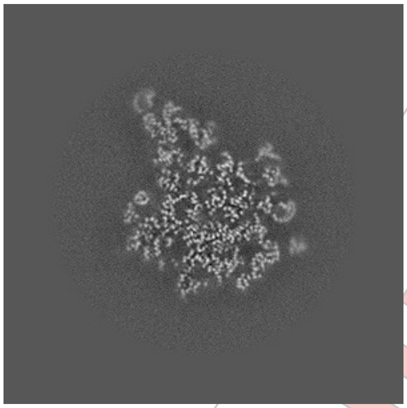
X Index: 234

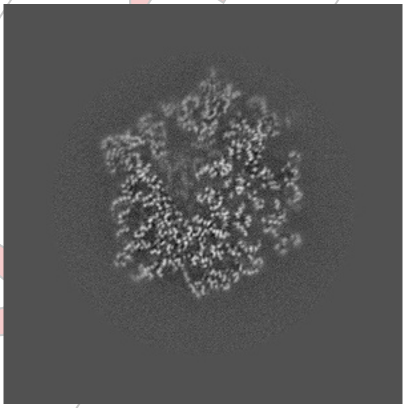
Y Index: 247

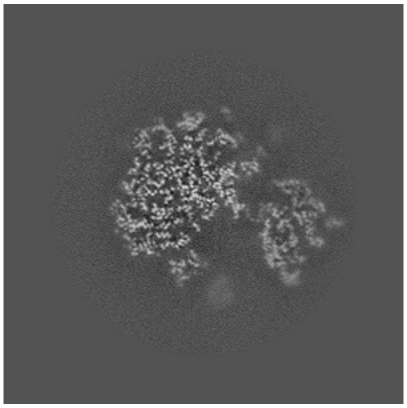
Z Index: 253

The images above show the largest variance slices of the map in three orthogonal directions.

For Manuscript Review

6.4 Orthogonal standard-deviation projections (False-color) (i)

6.4.1 Primary map

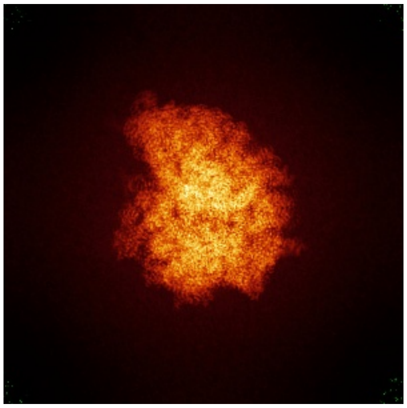
X

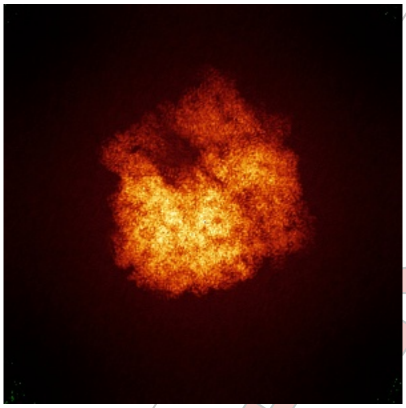
Y

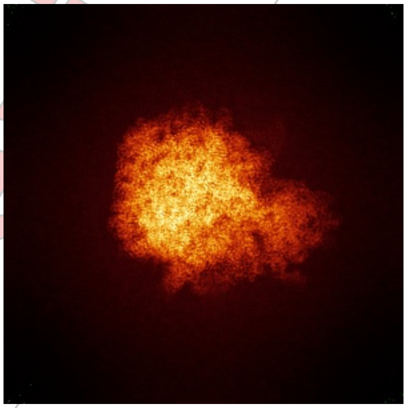
Z

6.4.2 Raw map

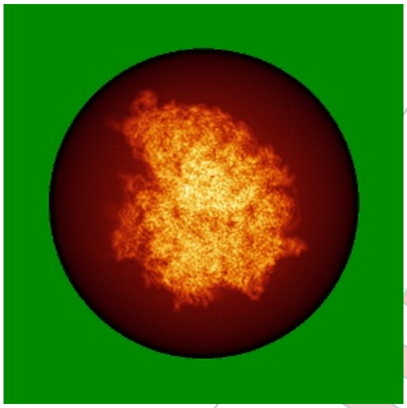
X

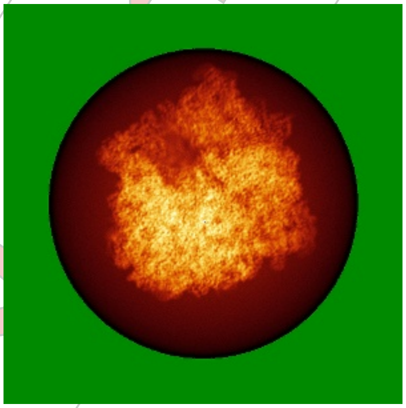
Y

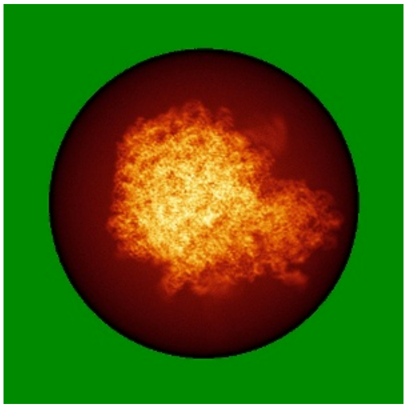
Z

The images above show the map standard deviation projections with false color in three orthogonal directions. Minimum values are shown in green, max in blue, and dark to light orange shades represent small to large values respectively.

For Manuscript Review

6.5 Orthogonal surface views (i)

6.5.1 Primary map

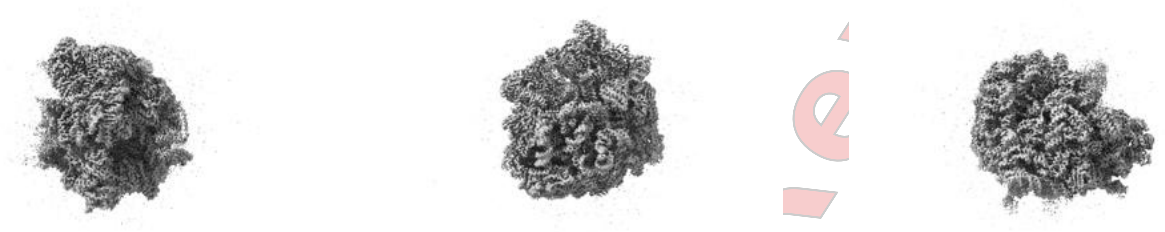

X Y Z

The images above show the 3D surface view of the map at the recommended contour level 2.5. These images, in conjunction with the slice images, may facilitate assessment of whether an appropriate contour level has been provided.

6.5.2 Raw map

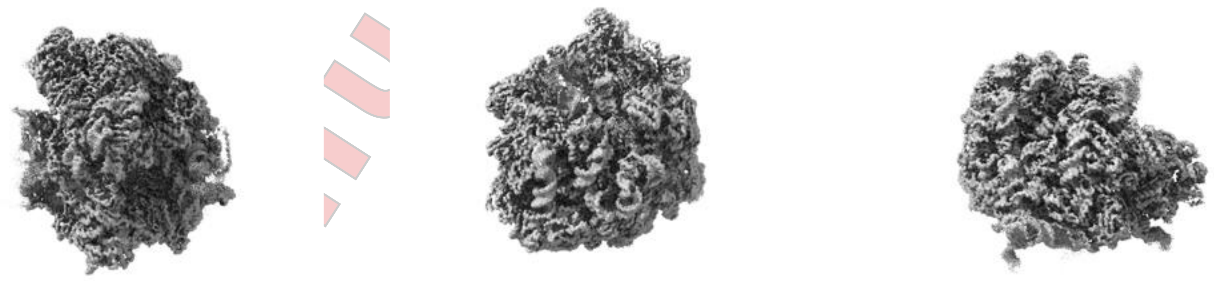

X Y Z

These images show the 3D surface of the raw map. The raw map's contour level was selected so that its surface encloses the same volume as the primary map does at its recommended contour level.

6.6 Mask visualisation (i)

This section was not generated. No masks/segmentation were deposited.

For Manuscript Review

7 Map analysis (i)

This section contains the results of statistical analysis of the map.

7.1 Map-value distribution (i)

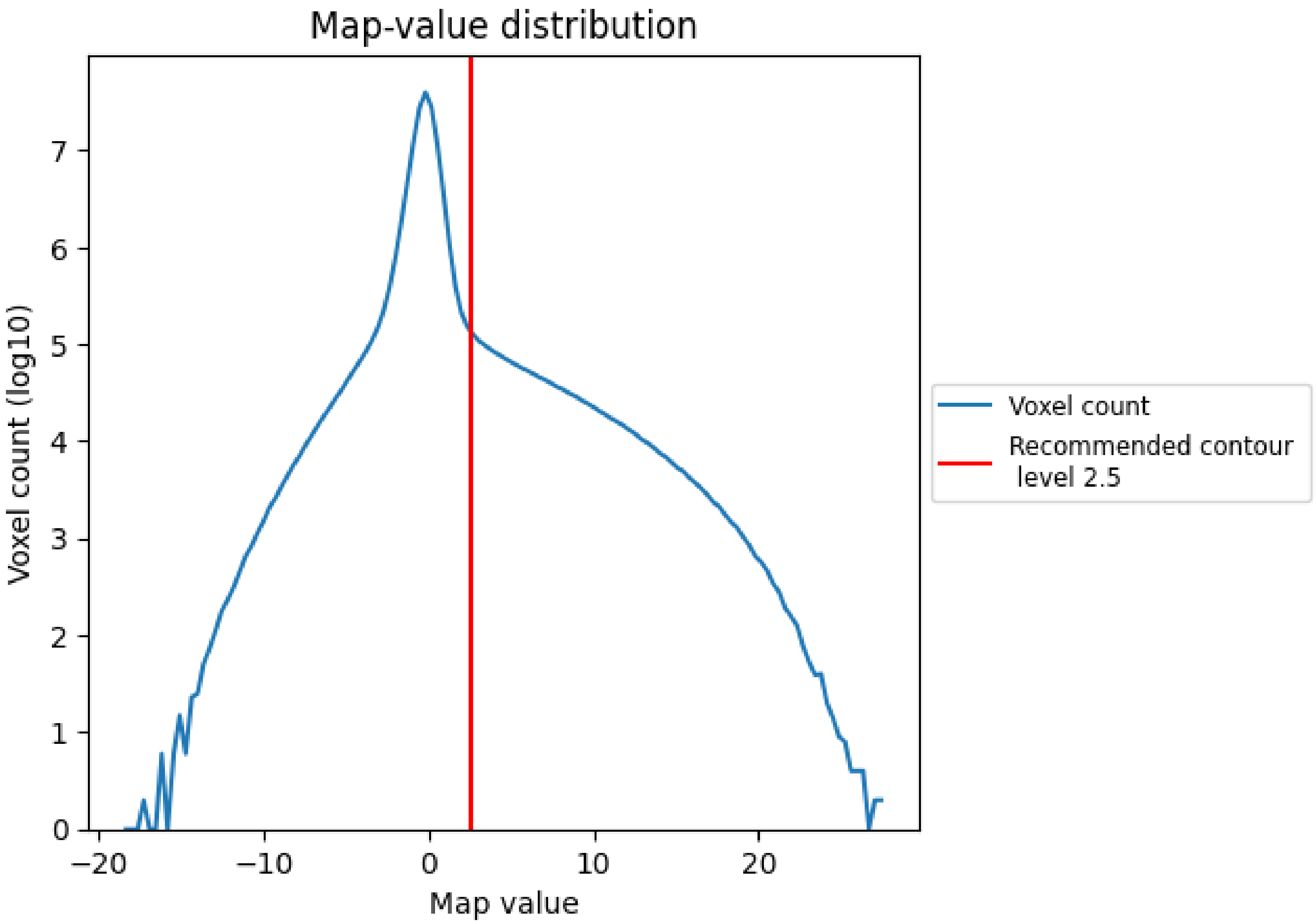

The map-value distribution is plotted in 128 intervals along the x-axis. The y-axis is logarithmic. A spike in this graph at zero usually indicates that the volume has been masked.

For Manuscript Review

7.2 Volume estimate (i)

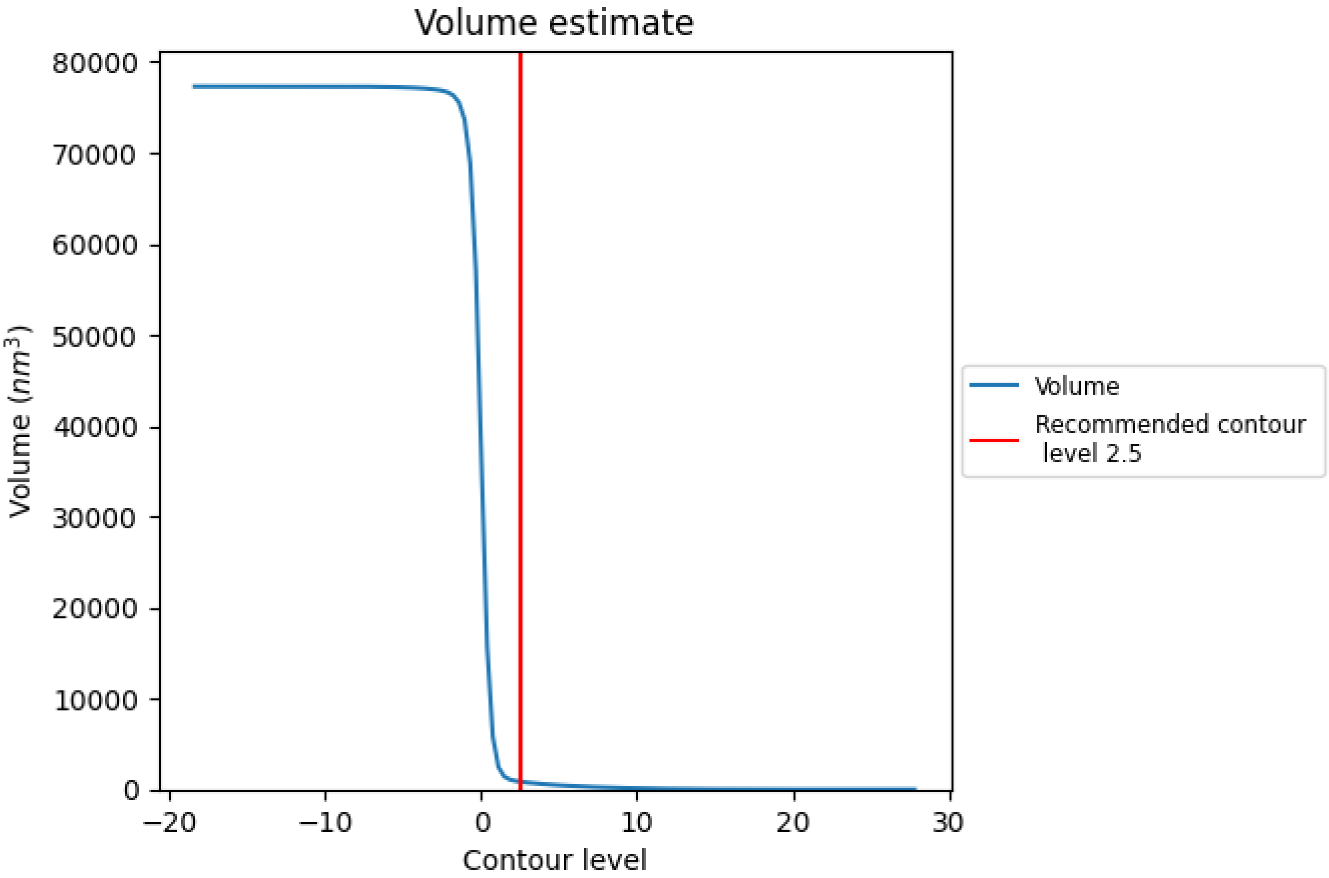

The volume at the recommended contour level is 852 nm^3; this corresponds to an approximate mass of 769 kDa.

The volume estimate graph shows how the enclosed volume varies with the contour level. The recommended contour level is shown as a vertical line and the intersection between the line and the curve gives the volume of the enclosed surface at the given level.

For Manuscript Review

7.3 Rotationally averaged power spectrum ⓘ

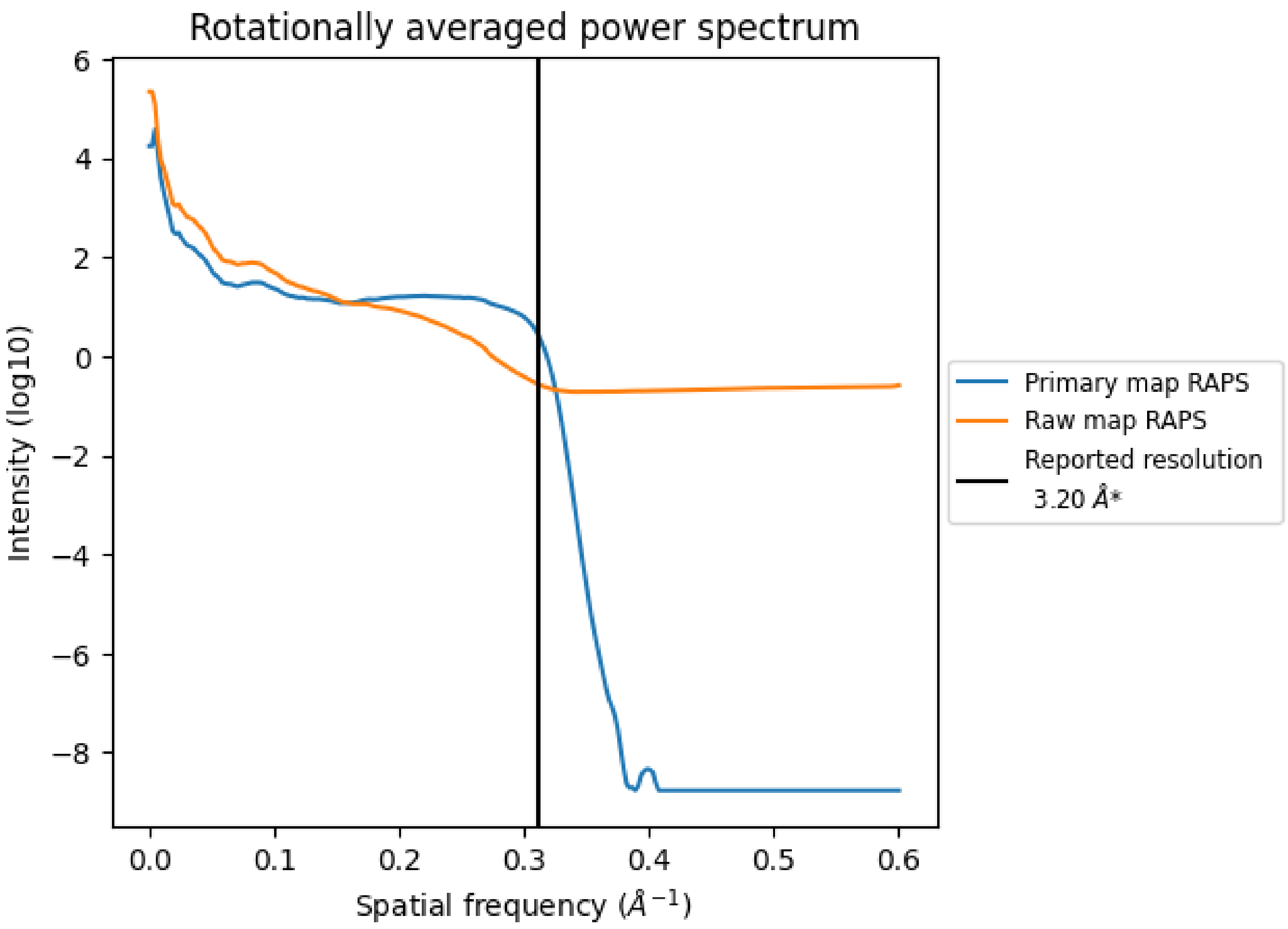

*Reported resolution corresponds to spatial frequency of 0.312 $Å^{-1}$

For Manuscript Review

8 Fourier-Shell correlation (i)

Fourier-Shell Correlation (FSC) is the most commonly used method to estimate the resolution of single-particle and subtomogram-averaged maps. The shape of the curve depends on the imposed symmetry, mask and whether or not the two 3D reconstructions used were processed from a common reference. The reported resolution is shown as a black line. A curve is displayed for the half-bit criterion in addition to lines showing the 0.143 gold standard cut-off and 0.5 cut-off.

8.1 FSC (i)

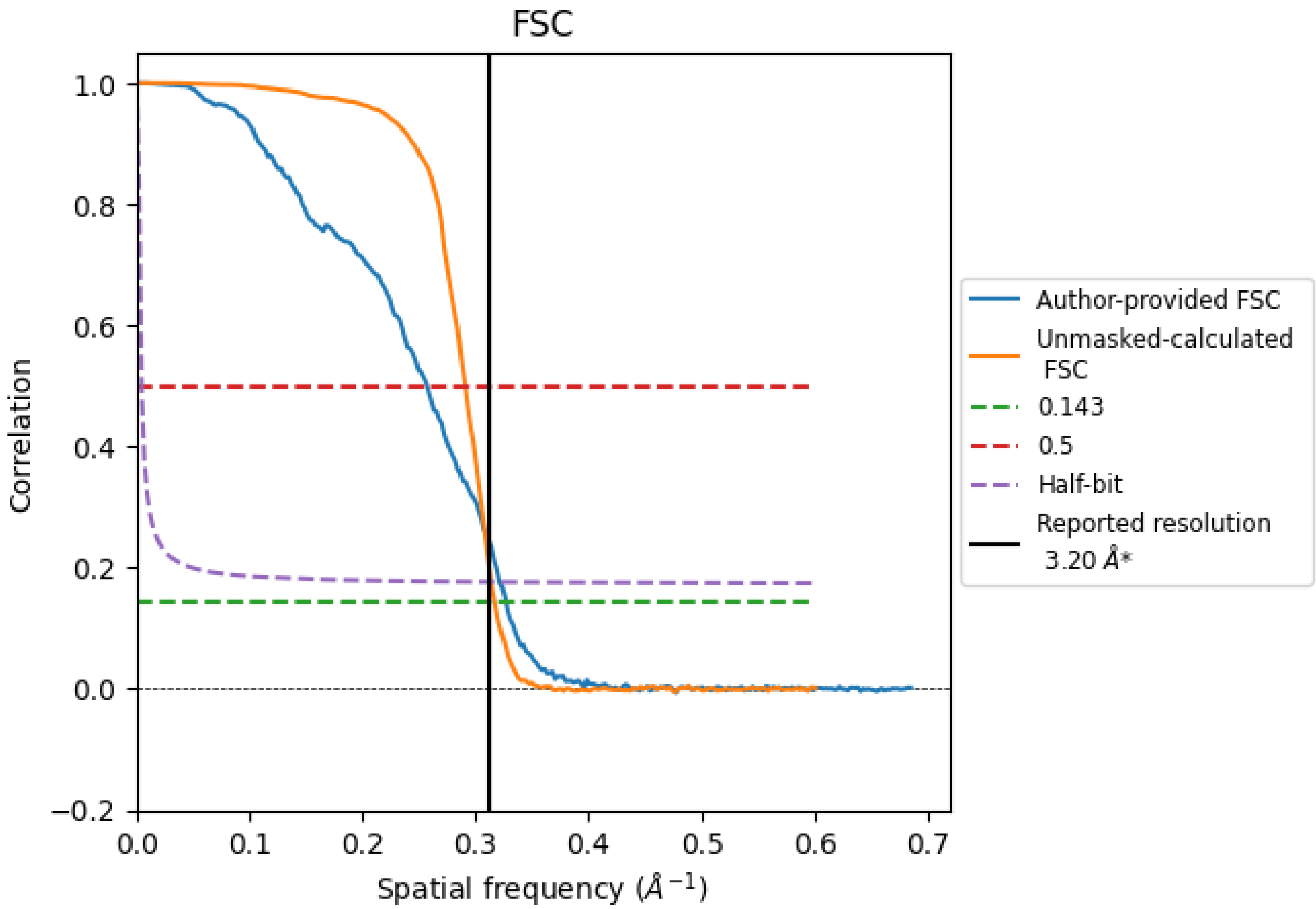

*Reported resolution corresponds to spatial frequency of 0.312 Å^{-1}

For Manuscript Review

8.2 Resolution estimates (i)

Resolution estimate (Å)	Estimation criterion (FSC cut-off)		
	0.143	0.5	Half-bit
Reported by author	3.20	-	-
Author-provided FSC curve	3.06	3.89	3.11
Unmasked-calculated*	3.15	3.44	3.18

*Resolution estimate based on FSC curve calculated by comparison of deposited half-maps.

For Manuscript Review

9 Map-model fit (i)

This section contains information regarding the fit between EMDB map EMD-75650 and PDB model 11EL. Per-residue inclusion information can be found in section 3 on page 17.

9.1 Map-model overlay (i)

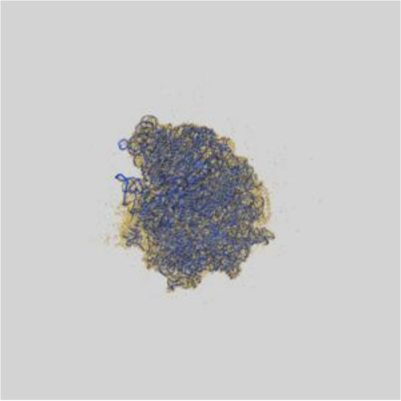

X

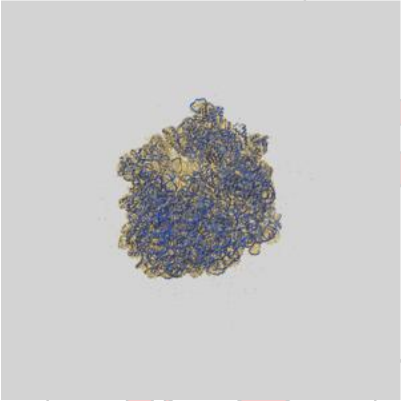

Y

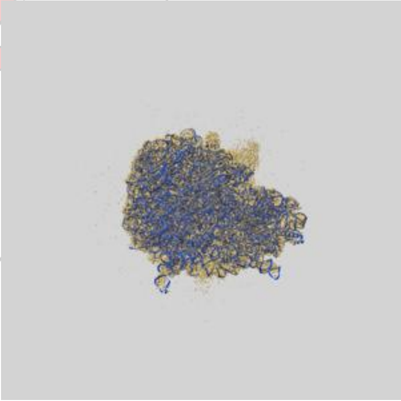

Z

The images above show the 3D surface view of the map at the recommended contour level 2.5 at 50% transparency in yellow overlaid with a ribbon representation of the model coloured in blue. These images allow for the visual assessment of the quality of fit between the atomic model and the map.

For Manuscript Review

9.2 Q-score mapped to coordinate model (i)

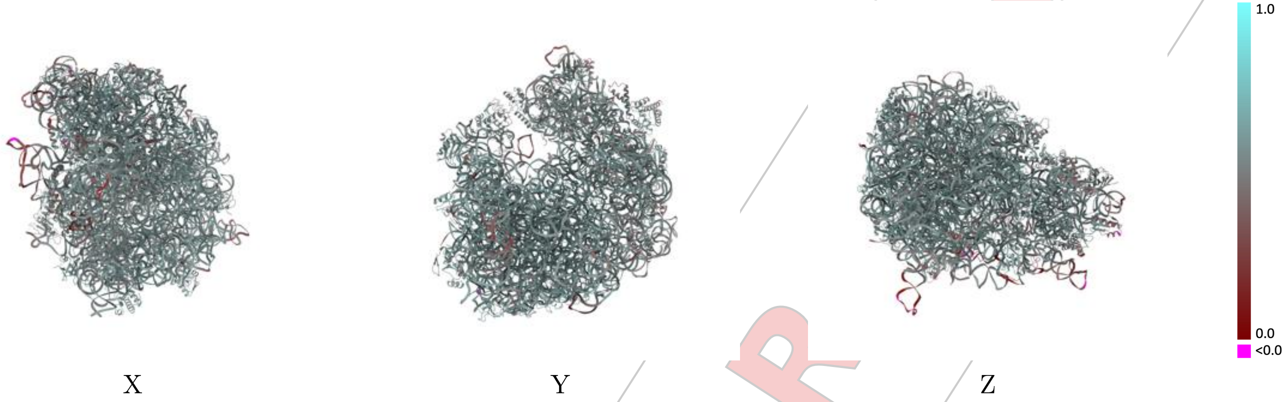

The images above show the model with each residue coloured according its Q-score. This shows their resolvability in the map with higher Q-score values reflecting better resolvability. Please note: Q-score is calculating the resolvability of atoms, and thus high values are only expected at resolutions at which atoms can be resolved. Low Q-score values may therefore be expected for many entries.

9.3 Atom inclusion mapped to coordinate model (i)

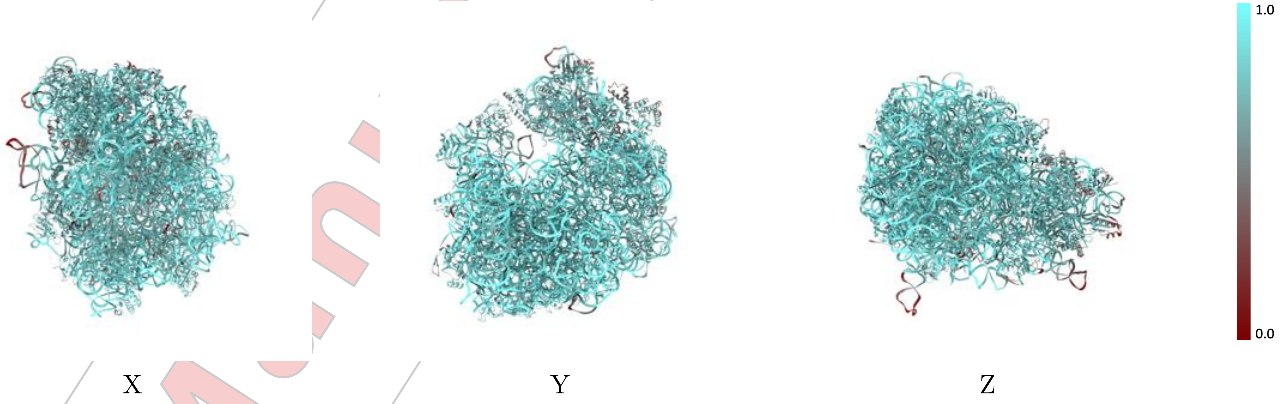

The images above show the model with each residue coloured according to its atom inclusion. This shows to what extent they are inside the map at the recommended contour level (2.5).

For Manuscript Review

9.4 Atom inclusion (i)

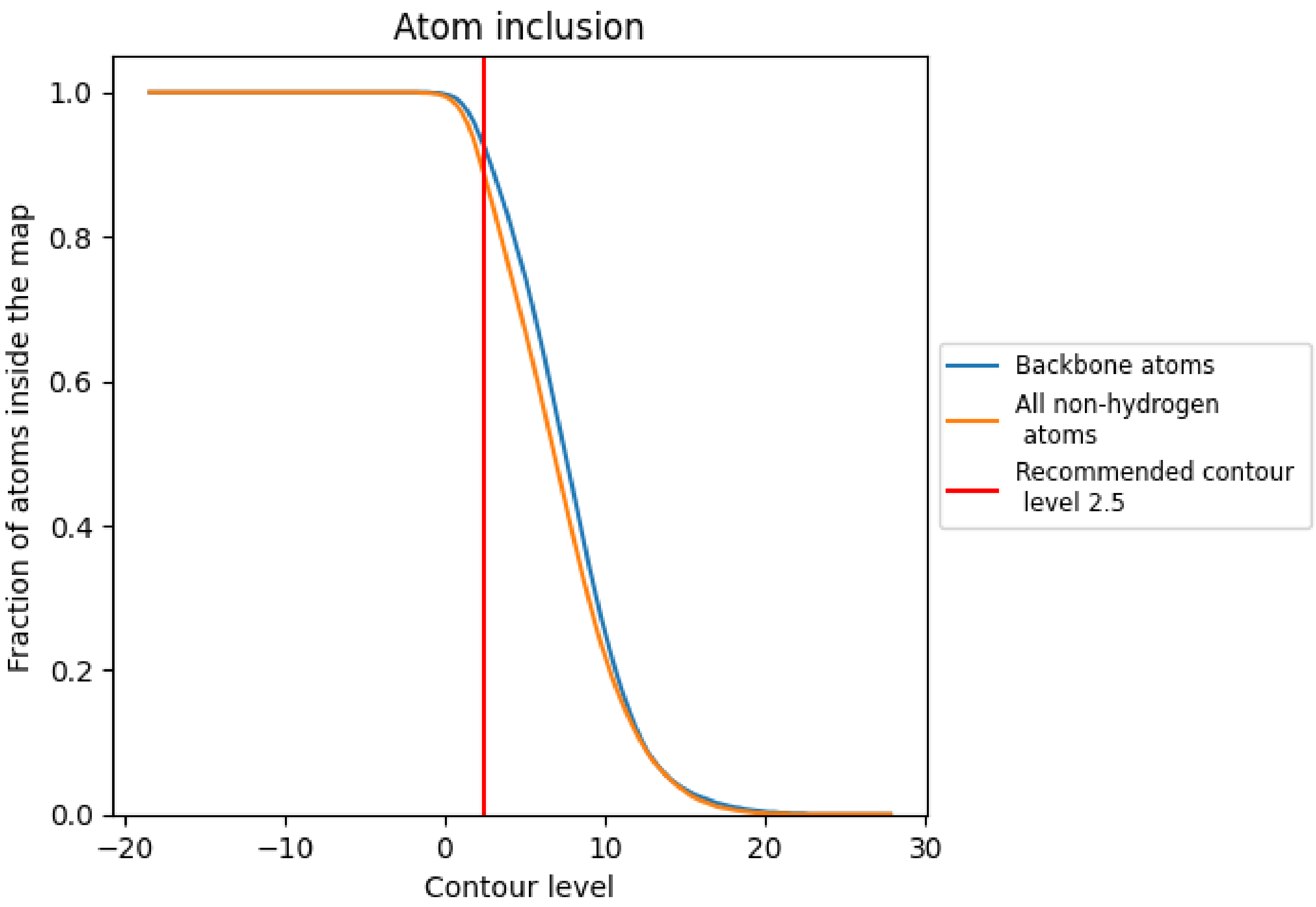

At the recommended contour level, 92% of all backbone atoms, 88% of all non-hydrogen atoms, are inside the map.

For Manuscript Review

9.5 Map-model fit summary (i)

The table lists the average atom inclusion at the recommended contour level (2.5) and Q-score for the entire model and for each chain.

Chain	Atom inclusion	Q-score
All	0.8840	0.5530
0	0.6430	0.5550
1	0.8990	0.5950
2	0.9080	0.6010
3	0.9010	0.6050
4	0.6990	0.4980
5	0.0950	0.4890
A	0.8890	0.5310
B	0.6410	0.5150
C	0.7470	0.5440
D	0.7480	0.5340
E	0.8530	0.5730
F	0.8180	0.5420
G	0.6630	0.4960
H	0.8400	0.5740
I	0.6920	0.5260
J	0.5830	0.4860
K	0.8420	0.5540
L	0.8820	0.5760
M	0.7650	0.5390
N	0.7440	0.5340
O	0.8580	0.5670
P	0.6060	0.3790
Q	0.8060	0.5300
R	0.8280	0.5500
S	0.7640	0.5230
T	0.8170	0.5460
U	0.5040	0.4910
a	0.9350	0.5630
b	0.9160	0.5460
c	0.9190	0.5980
d	0.9030	0.5930
e	0.8120	0.5680
f	0.7490	0.5350
g	0.7800	0.5290

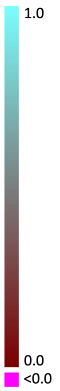

Continued on next page...

For Manuscript Review

Continued from previous page...

Chain	Atom inclusion	Q-score
h	0.7330	0.5130
i	0.9040	0.5940
j	0.8950	0.5890
k	0.8790	0.5810
l	0.8890	0.5810
m	0.9010	0.5900
n	0.8290	0.5660
o	0.8860	0.5940
p	0.8880	0.5900
q	0.8670	0.5920
r	0.8600	0.5820
s	0.8420	0.5690
t	0.8180	0.5670
u	0.8480	0.5780
v	0.8990	0.5900
w	0.8950	0.5840
x	0.7890	0.5470
y	0.8490	0.5790
z	0.8790	0.5830

For Manuscript Review
