# Supplementary material for: Structural adaptations for enhanced translation kinetics in evolved ribosomes
Source: Nucleic Acids Res. 2026 Jul 22;54(14):gkag703. doi: 10.1093/nar/gkag703 (PMC13389312; doi:10.1093/nar/gkag703)
Supplement: gkag703_Supplemental_Files [file gkag703_supplemental_files.zip › PDB_validation.zip!/PA_ST_D_1000303768_val-report-full-annotate_P1.pdf]

Full wwPDB EM Validation Report (i)

Feb 25, 2026 – 04:34 PM EST

PDB ID	:	11GG / pdb_000011gg
EMDB ID	:	EMD-75676
Title	:	Chimeric Escherichia coli 70S ribosome containing an evolved 16S rRNA from Pseudomonas aeruginosa (PA-ST)
Deposited on	:	2026-02-22
Resolution	:	3.02 Å(reported)

This wwPDB validation report is for manuscript review

This is a Full wwPDB EM Validation Report.

This report is produced by the wwPDB biocuration pipeline after annotation of the structure.

We welcome your comments at *validation@mail.wwpdb.org*
A user guide is available at
https://www.wwpdb.org/validation/2017/EMValidationReportHelp
with specific help available everywhere you see the (i) symbol.

The types of validation reports are described at
http://www.wwpdb.org/validation/2017/FAQs#types.

For Manuscript Review

The following versions of software and data (see references (i)) were used in the production of this report:

EMDB validation analysis	:	0.0.1.dev132
Mogul	:	2022.3.0, CSD as543be (2022)
MolProbity	:	4-5-2 with Phenix2.0
Percentile statistics	:	20231227.v01 (using entries in the PDB archive December 27th 2023)
EM percentile statistics	:	202505.v01 (Using data in the EMDB archive up until May 2025)
MapQ	:	1.9.13
Ideal geometry (proteins)	:	Engh & Huber (2001)
Ideal geometry (DNA, RNA)	:	Parkinson et al. (1996)

1 Overall quality at a glance (i)

The following experimental techniques were used to determine the structure:
ELECTRON MICROSCOPY

The reported resolution of this entry is 3.02 Å.

Percentile scores (ranging between 0-100) for global validation metrics of the entry are shown in the following graphic. The table shows the number of entries on which the scores are based.

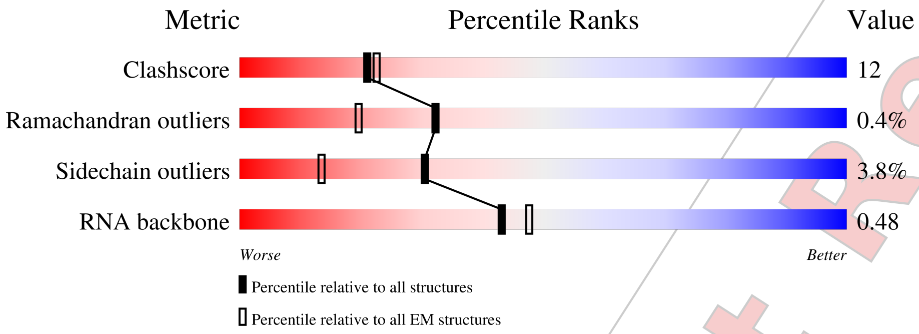

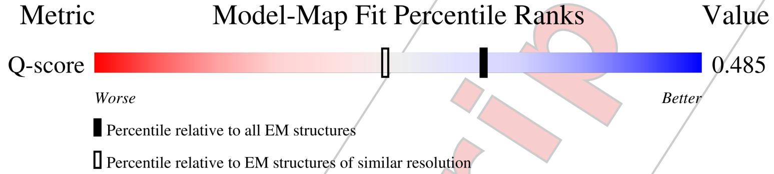

Metric	Whole archive (#Entries)	EM structures (#Entries)	Similar EM resolution (#Entries, resolution range(Å))
Clashscore	210492	15764	-
Ramachandran outliers	207382	16835	-
Sidechain outliers	206894	16415	-
RNA backbone	6643	2191	-
Q-score	-	25397	13913 (2.52 - 3.52)

The table below summarises the geometric issues observed across the polymeric chains and their fit to the map. The red, orange, yellow and green segments of the bar indicate the fraction of residues that contain outliers for >=3, 2, 1 and 0 types of geometric quality criteria respectively. A grey segment represents the fraction of residues that are not modelled. The numeric value for each fraction is indicated below the corresponding segment, with a dot representing fractions <=5% The upper red bar (where present) indicates the fraction of residues that have poor fit to the EM map (all-atom inclusion < 40%). The numeric value is given above the bar.

Mol	Chain	Length	Quality of chain
1	C	1536	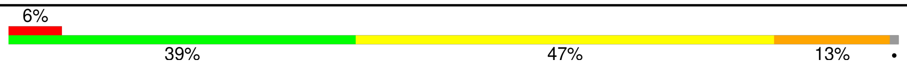

Continued on next page...

Validation Pipeline (wwPDB-VP) : 2.48.1

For Manuscript Review

Continued from previous page...

Mol	Chain	Length	Quality of chain
2	1	92	13% / 51% 30% 7% 12%
3	Z	103	29% / 82% 16% •
4	2	87	9% / 60% 32% 5% •
5	4	110	5% / 73% 26% •
6	3	71	45% / 72% 25% ••
7	b	100	6% / 72% 20% • 7%
8	c	104	29% / 66% 30% • •
9	D	241	81% / 66% 23% • 10%
10	d	94	57% / 61% 37% •
11	E	233	6% / 63% 25% 12%
12	e	85	22% / 64% 24% • 12%
13	F	206	28% / 61% 34% •
14	f	78	71% 27% ••
15	G	167	• / 67% 22% • 10%
16	g	63	17% / 81% 17% •
17	H	135	• / 50% 23% • 26%
18	h	59	19% / 73% 24% • •
19	I	2904	13% / 47% 44% 9%
20	i	57	• / 72% 25% • •
21	J	120	39% / 35% 48% 15% •
22	j	55	84% / 69% 22% 9%
23	K	273	• / 72% 26% ••
24	k	46	• / 85% 15%
25	L	209	9% / 80% 19% •
26	l	65	• / 58% 40% •

Continued on next page...

For Manuscript Review

Continued from previous page...

Mol	Chain	Length	Quality of chain
27	M	201	24% / 78% 22%
28	m	38	• / 71% 26% •
29	N	179	66% / 66% 31% ••
30	n	179	14% / 55% 28% • 16%
31	O	177	67% / 71% 28% ••
32	o	130	• / 73% 24% ••
33	P	149	76% / 79% 21% •
34	p	130	14% / 58% 36% • •
35	Q	70	70% / 59% 27% 14%
36	q	103	39% / 61% 30% • 5%
37	R	142	• / 78% 22%
38	r	129	• / 70% 19% • 9%
39	S	123	• / 74% 24% ••
40	t	124	• / 61% 35% ••
41	T	144	28% / 76% 22% •
42	u	118	5% / 68% 27% • •
43	U	136	7% / 65% 34% •
44	v	101	13% / 57% 38% 5%
45	V	127	• / 69% 25% • 6%
46	w	89	• / 82% 16% ••
47	W	117	• / 73% 26% ••
48	x	82	26% / 55% 43% ••
49	X	115	• / 74% 24% ••
50	y	84	14% / 60% 33% • 5%
51	Y	118	10% / 80% 19% ••

Continued on next page...

For Manuscript Review

Continued from previous page...

Mol	Chain	Length	Quality of chain
52	z	75	47% 25% • 27%

For Manuscript Review

2 Entry composition (i)

There are 53 unique types of molecules in this entry. The entry contains 142146 atoms, of which 0 are hydrogens and 0 are deuteriums.

In the tables below, the AltConf column contains the number of residues with at least one atom in alternate conformation and the Trace column contains the number of residues modelled with at most 2 atoms.

- Molecule 1 is a RNA chain called 16S ribosomal RNA.

Mol	Chain	Residues	Atoms					AltConf	Trace
			Total	C	N	O	P		
1	C	1526	32745	14606	6011	10603	1525	0	0

- Molecule 2 is a protein called Small ribosomal subunit protein uS19.

Mol	Chain	Residues	Atoms					AltConf	Trace
			Total	C	N	O	S		
2	1	81	651	416	124	109	2	0	0

- Molecule 3 is a protein called Ribosomal protein L21.

Mol	Chain	Residues	Atoms					AltConf	Trace
			Total	C	N	O	S		
3	Z	103	816	516	153	145	2	0	0

- Molecule 4 is a protein called Small ribosomal subunit protein bS20.

Mol	Chain	Residues	Atoms					AltConf	Trace
			Total	C	N	O	S		
4	2	84	659	408	136	112	3	0	0

- Molecule 5 is a protein called Large ribosomal subunit protein uL22.

Mol	Chain	Residues	Atoms					AltConf	Trace
			Total	C	N	O	S		
5	4	110	857	532	166	156	3	0	0

- Molecule 6 is a protein called Small ribosomal subunit protein bS21.

Mol	Chain	Residues	Atoms					AltConf	Trace
			Total	C	N	O	S		
6	3	70	590	366	125	98	1	0	0

For Manuscript Review

- Molecule 7 is a protein called 50S ribosomal protein L23.

Mol	Chain	Residues	Atoms					AltConf	Trace
7	b	93	Total 738	C 466	N 139	O 131	S 2	0	0

- Molecule 8 is a protein called 50S ribosomal protein L24.

Mol	Chain	Residues	Atoms				AltConf	Trace
8	c	102	Total 779	C 492	N 146	O 141	0	0

- Molecule 9 is a protein called Small ribosomal subunit protein uS2.

Mol	Chain	Residues	Atoms					AltConf	Trace
9	D	218	Total 1704	C 1081	N 305	O 311	S 7	0	0

- Molecule 10 is a protein called Large ribosomal subunit protein bL25.

Mol	Chain	Residues	Atoms					AltConf	Trace
10	d	94	Total 753	C 479	N 137	O 134	S 3	0	0

- Molecule 11 is a protein called Small ribosomal subunit protein uS3.

Mol	Chain	Residues	Atoms					AltConf	Trace
11	E	206	Total 1624	C 1028	N 305	O 288	S 3	0	0

- Molecule 12 is a protein called 50S ribosomal protein L27.

Mol	Chain	Residues	Atoms					AltConf	Trace
12	e	75	Total 575	C 356	N 116	O 102	S 1	0	0

- Molecule 13 is a protein called Small ribosomal subunit protein uS4.

Mol	Chain	Residues	Atoms					AltConf	Trace
13	F	205	Total 1643	C 1026	N 315	O 298	S 4	0	0

- Molecule 14 is a protein called 50S ribosomal protein L28.

For Manuscript Review

Mol	Chain	Residues	Atoms					AltConf	Trace
			Total	C	N	O	S		
14	f	77	625	388	129	106	2	0	0

- Molecule 15 is a protein called Small ribosomal subunit protein uS5.

Mol	Chain	Residues	Atoms					AltConf	Trace
			Total	C	N	O	S		
15	G	150	1105	687	211	201	6	0	0

- Molecule 16 is a protein called Large ribosomal subunit protein uL29.

Mol	Chain	Residues	Atoms					AltConf	Trace
			Total	C	N	O	S		
16	g	62	501	308	98	94	1	0	0

- Molecule 17 is a protein called Small ribosomal subunit protein bS6.

Mol	Chain	Residues	Atoms					AltConf	Trace
			Total	C	N	O	S		
17	H	100	817	515	148	148	6	0	0

- Molecule 18 is a protein called 50S ribosomal protein L30.

Mol	Chain	Residues	Atoms					AltConf	Trace
			Total	C	N	O	S		
18	h	58	449	281	87	79	2	0	0

- Molecule 19 is a RNA chain called 23S ribosomal RNA.

Mol	Chain	Residues	Atoms					AltConf	Trace
			Total	C	N	O	P		
19	I	2898	62271	27787	11455	20129	2900	2	0

- Molecule 20 is a protein called 50S ribosomal protein L32.

Mol	Chain	Residues	Atoms					AltConf	Trace
			Total	C	N	O	S		
20	i	56	444	269	94	80	1	0	0

- Molecule 21 is a RNA chain called 5S ribosomal RNA.

For Manuscript Review

Mol	Chain	Residues	Atoms					AltConf	Trace
			Total	C	N	O	P		
21	J	118	2529	1126	464	821	118	0	0

- Molecule 22 is a protein called 50S ribosomal protein L33.

Mol	Chain	Residues	Atoms				AltConf	Trace
			Total	C	N	O		
22	j	50	409	263	75	71	0	0

- Molecule 23 is a protein called 50S ribosomal protein L2.

Mol	Chain	Residues	Atoms					AltConf	Trace
			Total	C	N	O	S		
23	K	271	2093	1294	427	365	7	1	0

- Molecule 24 is a protein called 50S ribosomal protein L34.

Mol	Chain	Residues	Atoms					AltConf	Trace
			Total	C	N	O	S		
24	k	46	377	228	90	57	2	0	0

- Molecule 25 is a protein called Large ribosomal subunit protein uL3.

Mol	Chain	Residues	Atoms					AltConf	Trace
			Total	C	N	O	S		
25	L	209	1565	979	288	294	4	0	0

- Molecule 26 is a protein called 50S ribosomal protein L35.

Mol	Chain	Residues	Atoms					AltConf	Trace
			Total	C	N	O	S		
26	l	64	504	323	105	74	2	0	0

- Molecule 27 is a protein called Large ribosomal subunit protein uL4.

Mol	Chain	Residues	Atoms					AltConf	Trace
			Total	C	N	O	S		
27	M	201	1552	974	283	290	5	0	0

- Molecule 28 is a protein called 50S ribosomal protein L36.

For Manuscript Review

Mol	Chain	Residues	Atoms					AltConf	Trace
			Total	C	N	O	S		
28	m	38	302	185	65	48	4	0	0

- Molecule 29 is a protein called Large ribosomal subunit protein uL5.

Mol	Chain	Residues	Atoms					AltConf	Trace
			Total	C	N	O	S		
29	N	177	1410	899	249	256	6	0	0

- Molecule 30 is a protein called Small ribosomal subunit protein uS7.

Mol	Chain	Residues	Atoms					AltConf	Trace
			Total	C	N	O	S		
30	n	151	1181	735	227	215	4	0	0

- Molecule 31 is a protein called Large ribosomal subunit protein uL6.

Mol	Chain	Residues	Atoms					AltConf	Trace
			Total	C	N	O	S		
31	O	176	1323	832	243	246	2	0	0

- Molecule 32 is a protein called Small ribosomal subunit protein uS8.

Mol	Chain	Residues	Atoms					AltConf	Trace
			Total	C	N	O	S		
32	o	129	979	616	173	184	6	0	0

- Molecule 33 is a protein called Large ribosomal subunit protein bL9.

Mol	Chain	Residues	Atoms					AltConf	Trace
			Total	C	N	O	S		
33	P	149	1111	699	197	214	1	0	0

- Molecule 34 is a protein called Small ribosomal subunit protein uS9.

Mol	Chain	Residues	Atoms					AltConf	Trace
			Total	C	N	O	S		
34	p	127	1022	634	206	179	3	0	0

- Molecule 35 is a protein called 50S ribosomal protein L31.

For Manuscript Review

Mol	Chain	Residues	Atoms					AltConf	Trace
			Total	C	N	O	S		
35	Q	60	480	299	90	85	6	0	0

- Molecule 36 is a protein called Small ribosomal subunit protein uS10.

Mol	Chain	Residues	Atoms					AltConf	Trace
			Total	C	N	O	S		
36	q	98	786	493	150	142	1	0	0

- Molecule 37 is a protein called Large ribosomal subunit protein uL13.

Mol	Chain	Residues	Atoms					AltConf	Trace
			Total	C	N	O	S		
37	R	142	1129	714	212	199	4	0	0

- Molecule 38 is a protein called 30S ribosomal protein S11.

Mol	Chain	Residues	Atoms					AltConf	Trace
			Total	C	N	O	S		
38	r	117	877	540	174	160	3	0	0

- Molecule 39 is a protein called Large ribosomal subunit protein uL14.

Mol	Chain	Residues	Atoms					AltConf	Trace
			Total	C	N	O	S		
39	S	122	938	587	180	165	6	0	0

- Molecule 40 is a protein called 30S ribosomal protein S12.

Mol	Chain	Residues	Atoms					AltConf	Trace
			Total	C	N	O	S		
40	t	123	955	590	196	165	4	0	0

- Molecule 41 is a protein called Large ribosomal subunit protein uL15.

Mol	Chain	Residues	Atoms					AltConf	Trace
			Total	C	N	O	S		
41	T	144	1053	654	207	190	2	0	0

- Molecule 42 is a protein called Small ribosomal subunit protein uS13.

For Manuscript Review

Mol	Chain	Residues	Atoms					AltConf	Trace
			Total	C	N	O	S		
42	u	115	891	552	179	157	3	0	0

- Molecule 43 is a protein called Large ribosomal subunit protein uL16.

Mol	Chain	Residues	Atoms					AltConf	Trace
			Total	C	N	O	S		
43	U	136	1075	686	205	178	6	0	0

- Molecule 44 is a protein called Small ribosomal subunit protein uS14.

Mol	Chain	Residues	Atoms					AltConf	Trace
			Total	C	N	O	S		
44	v	96	774	483	160	128	3	0	0

- Molecule 45 is a protein called Large ribosomal subunit protein bL17.

Mol	Chain	Residues	Atoms					AltConf	Trace
			Total	C	N	O	S		
45	V	120	960	593	196	166	5	0	0

- Molecule 46 is a protein called Small ribosomal subunit protein uS15.

Mol	Chain	Residues	Atoms					AltConf	Trace
			Total	C	N	O	S		
46	w	88	710	437	143	129	1	0	0

- Molecule 47 is a protein called Large ribosomal subunit protein uL18.

Mol	Chain	Residues	Atoms				AltConf	Trace
			Total	C	N	O		
47	W	116	892	552	178	162	0	0

- Molecule 48 is a protein called Small ribosomal subunit protein bS16.

Mol	Chain	Residues	Atoms					AltConf	Trace
			Total	C	N	O	S		
48	x	82	649	406	128	114	1	0	0

- Molecule 49 is a protein called Large ribosomal subunit protein bL19.

For Manuscript Review

Mol	Chain	Residues	Atoms					AltConf	Trace
			Total	C	N	O	S		
49	X	114	917	574	179	163	1	0	0

- Molecule 50 is a protein called Small ribosomal subunit protein uS17.

Mol	Chain	Residues	Atoms					AltConf	Trace
			Total	C	N	O	S		
50	y	80	648	411	121	113	3	0	0

- Molecule 51 is a protein called 50S ribosomal protein L20.

Mol	Chain	Residues	Atoms				AltConf	Trace
			Total	C	N	O		
51	Y	117	947	604	192	151	0	0

- Molecule 52 is a protein called Small ribosomal subunit protein bS18.

Mol	Chain	Residues	Atoms				AltConf	Trace
			Total	C	N	O		
52	z	55	455	288	86	81	0	0

- Molecule 53 is MAGNESIUM ION (CCD ID: MG) (formula: Mg).

Mol	Chain	Residues	Atoms		AltConf
			Total	Mg	
53	C	90	90	90	0
53	I	204	204	204	0
53	i	1	1	1	0
53	J	5	5	5	0
53	K	1	1	1	0
53	L	1	1	1	0
53	l	1	1	1	0
53	q	1	1	1	0
53	V	2	2	2	0

Continued on next page...

For Manuscript Review

Continued from previous page...

Mol	Chain	Residues	Atoms		AltConf
			Total	Mg	
53	Y	1	1	1	0

For Manuscript Review

3 Residue-property plots (i)

These plots are drawn for all protein, RNA, DNA and oligosaccharide chains in the entry. The first graphic for a chain summarises the proportions of the various outlier classes displayed in the second graphic. The second graphic shows the sequence view annotated by issues in geometry and atom inclusion in map density. Residues are color-coded according to the number of geometric quality criteria for which they contain at least one outlier: green = 0, yellow = 1, orange = 2 and red = 3 or more. A red diamond above a residue indicates a poor fit to the EM map for this residue (all-atom inclusion < 40%). Stretches of 2 or more consecutive residues without any outlier are shown as a green connector. Residues present in the sample, but not in the model, are shown in grey.

- Molecule 1: 16S ribosomal RNA

Chain C:

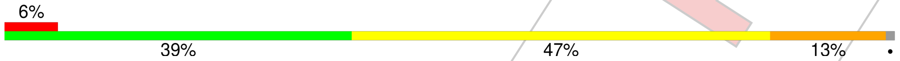

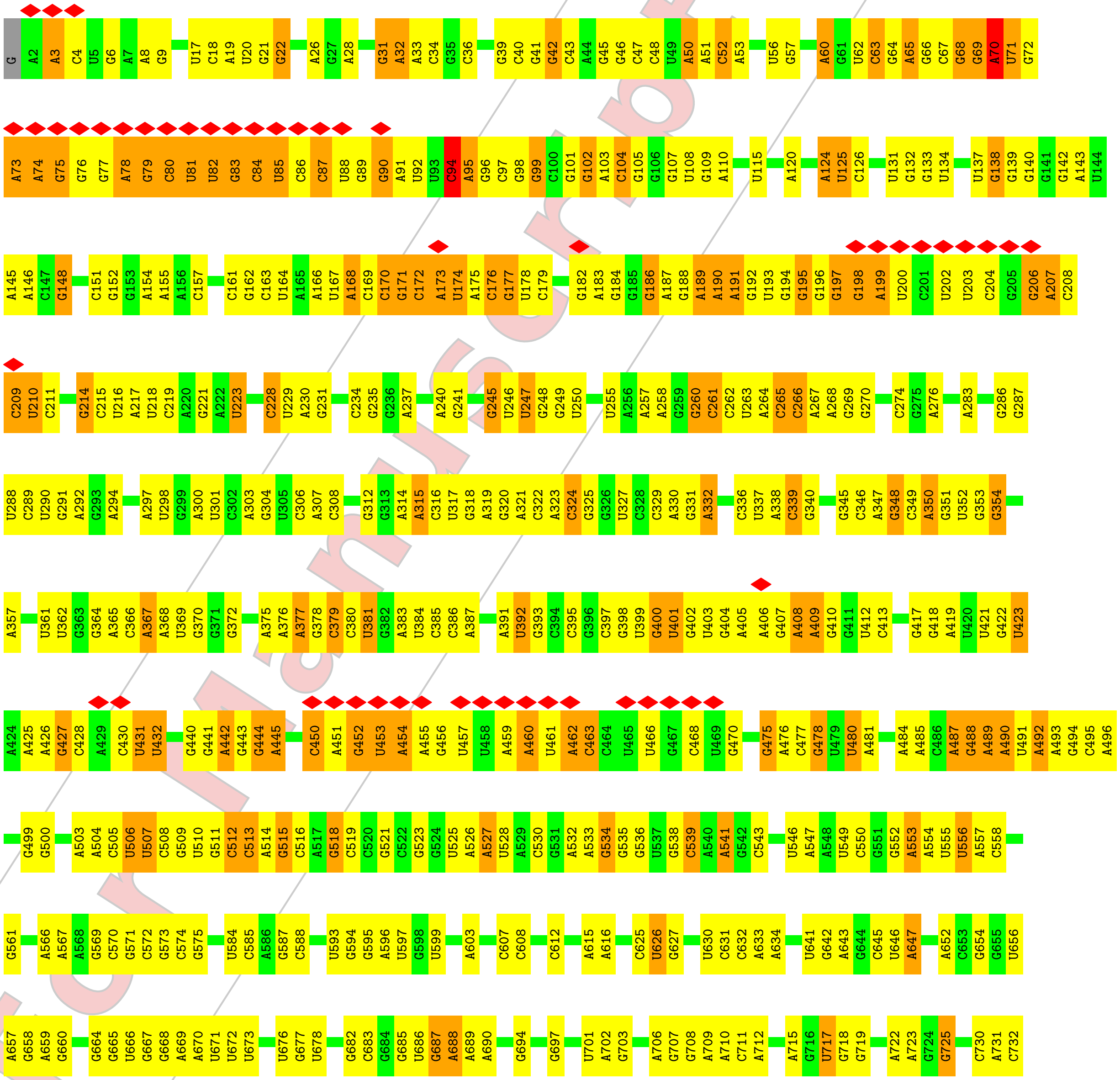

For Manuscript Review

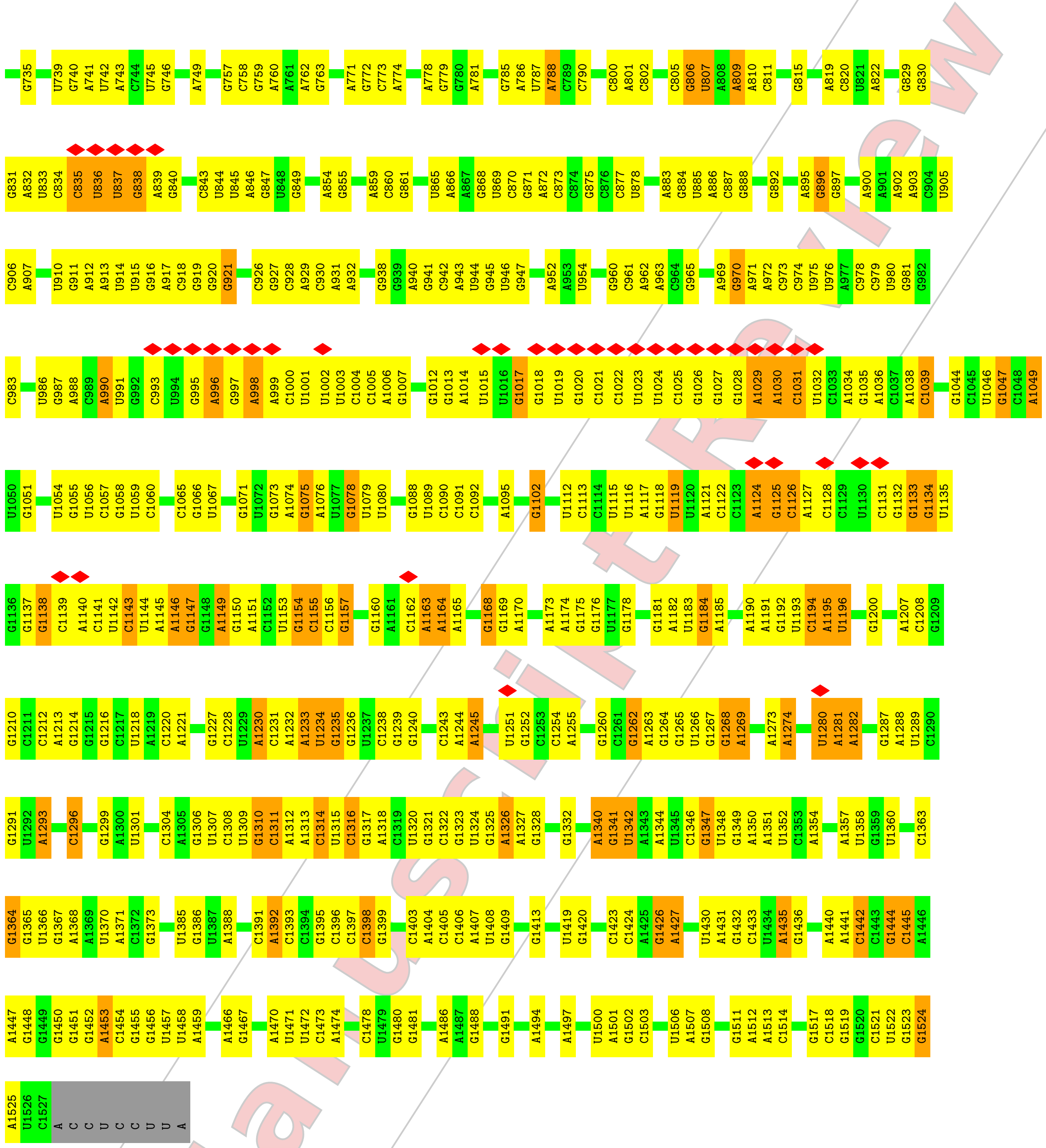

• Molecule 2: Small ribosomal subunit protein uS19

Chain 1:

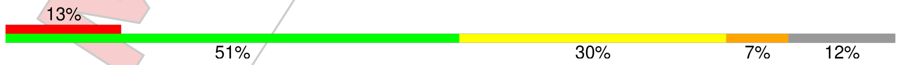

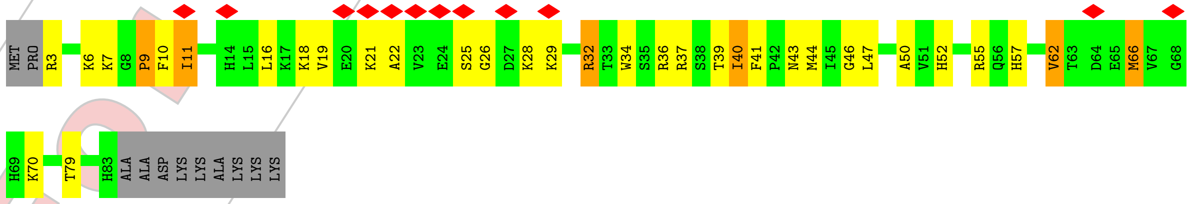

• Molecule 3: Ribosomal protein L21

For Manuscript Review

Chain Z:

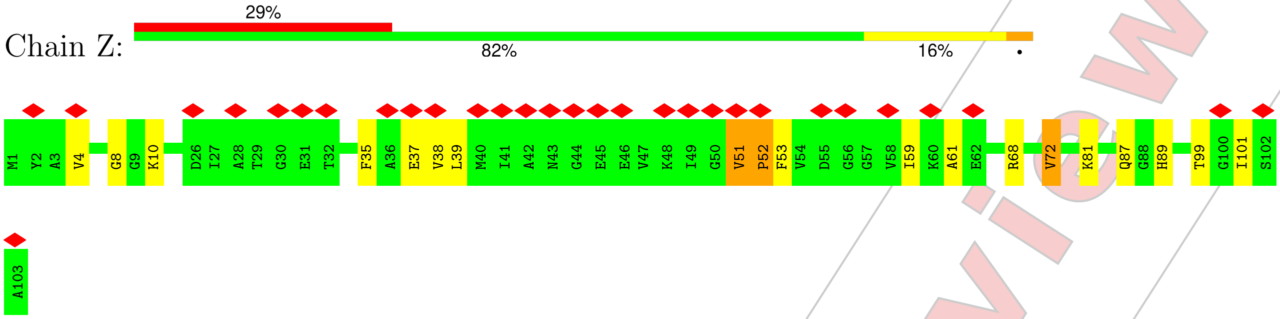

• Molecule 4: Small ribosomal subunit protein bS20

Chain 2:

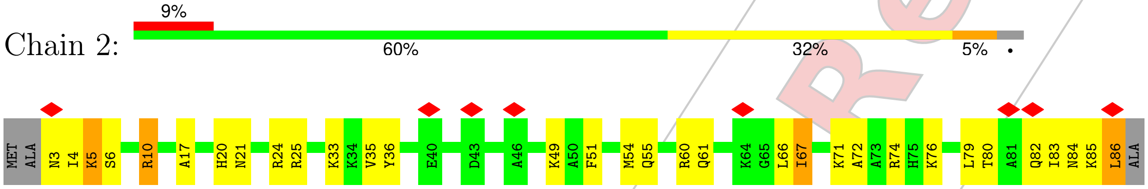

• Molecule 5: Large ribosomal subunit protein uL22

Chain 4:

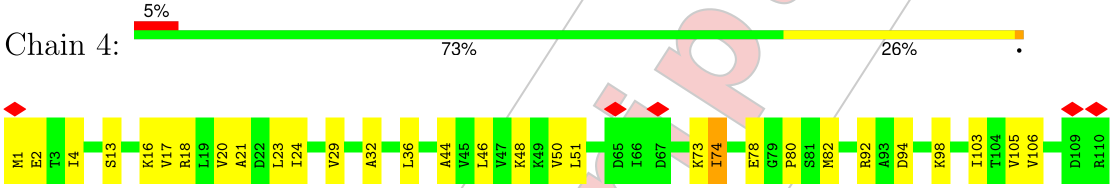

• Molecule 6: Small ribosomal subunit protein bS21

Chain 3:

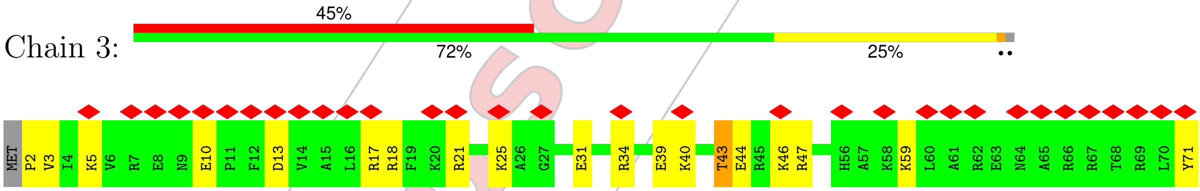

• Molecule 7: 50S ribosomal protein L23

Chain b:

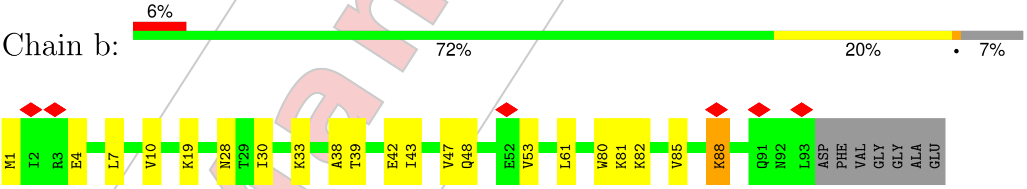

• Molecule 8: 50S ribosomal protein L24

Chain c:

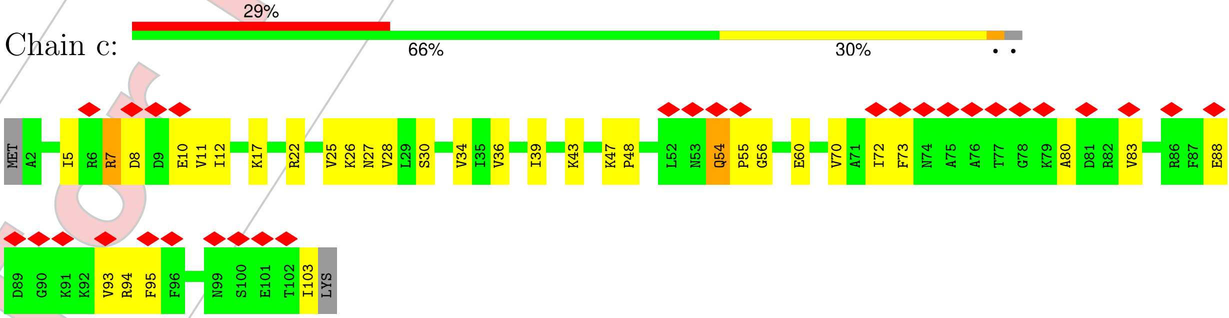

For Manuscript Review

• Molecule 9: Small ribosomal subunit protein uS2

Chain D:

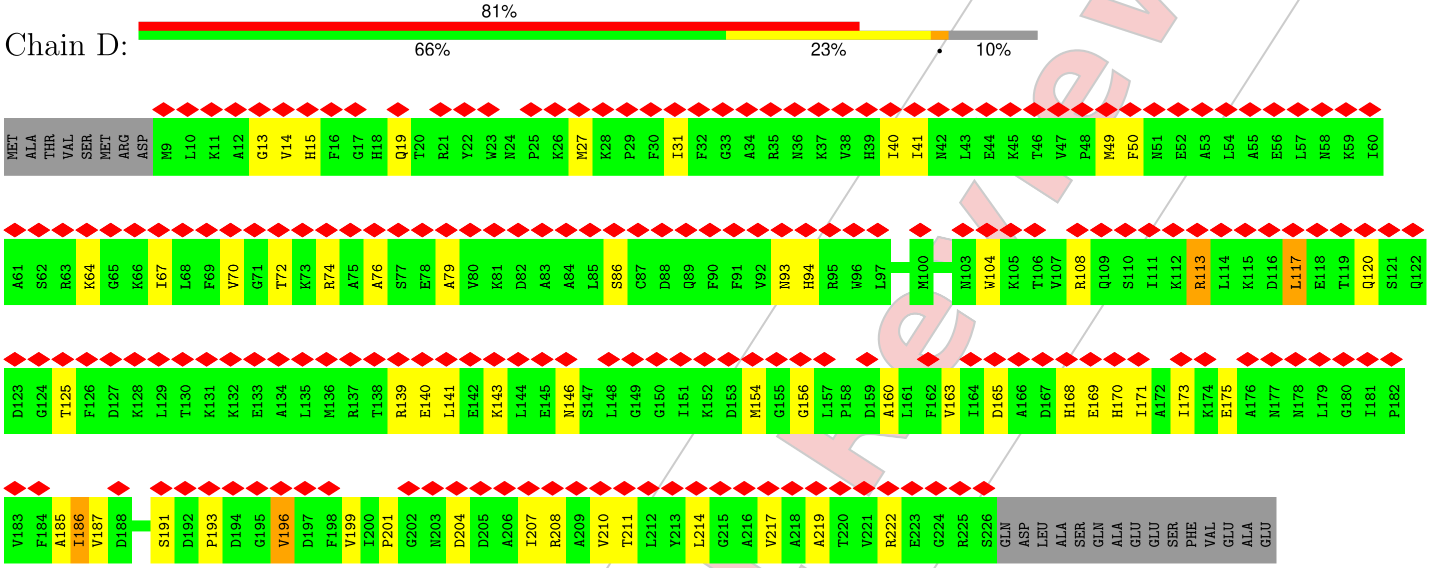

• Molecule 10: Large ribosomal subunit protein bL25

Chain d:

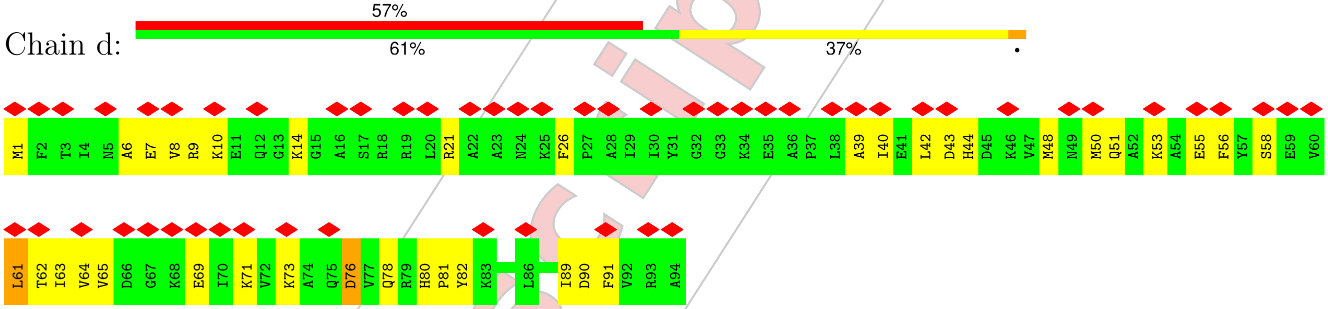

• Molecule 11: Small ribosomal subunit protein uS3

Chain E:

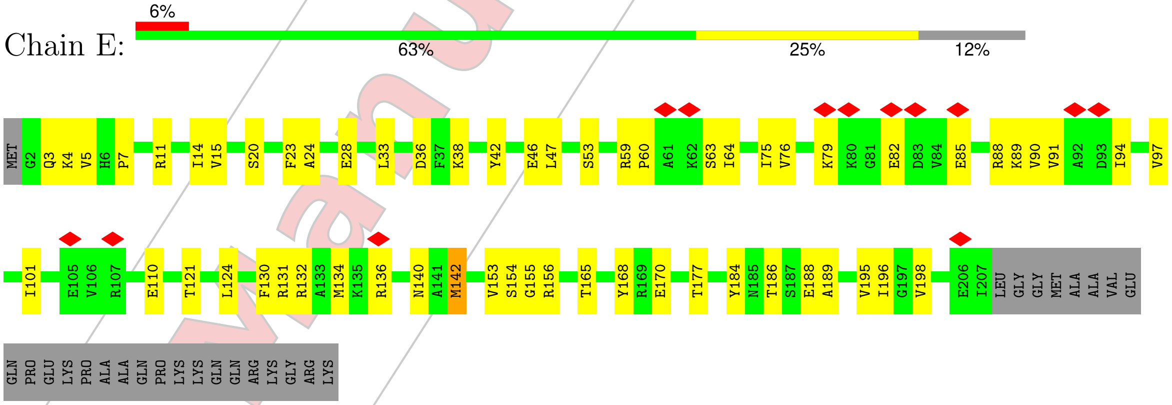

• Molecule 12: 50S ribosomal protein L27

Chain e:

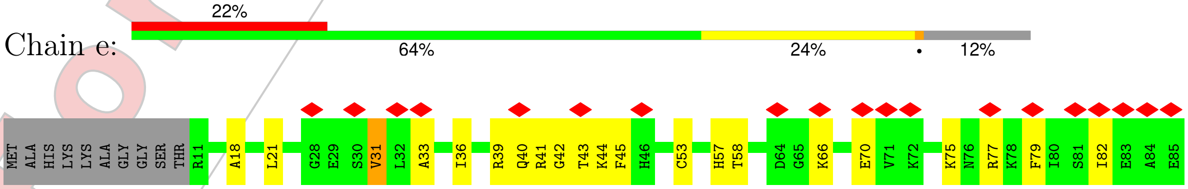

For Manuscript Review

• Molecule 13: Small ribosomal subunit protein uS4

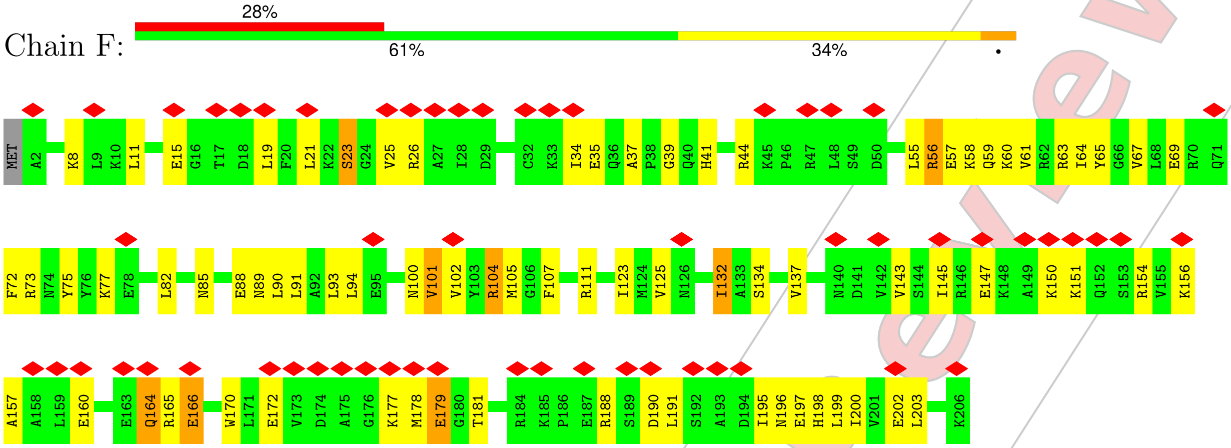

• Molecule 14: 50S ribosomal protein L28

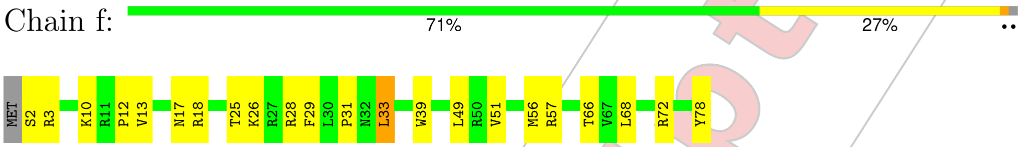

• Molecule 15: Small ribosomal subunit protein uS5

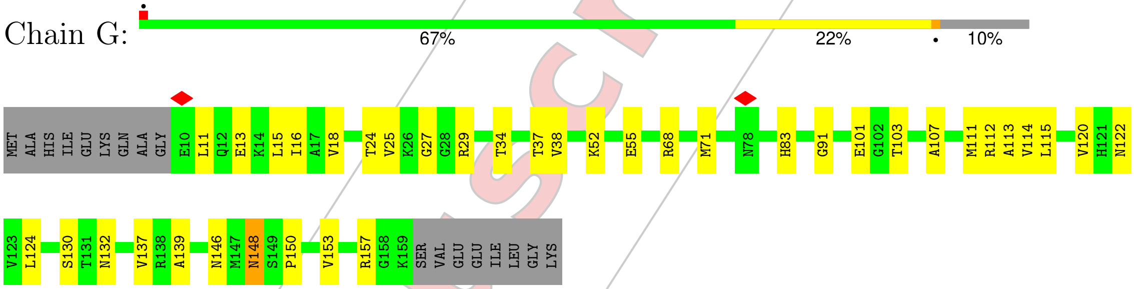

• Molecule 16: Large ribosomal subunit protein uL29

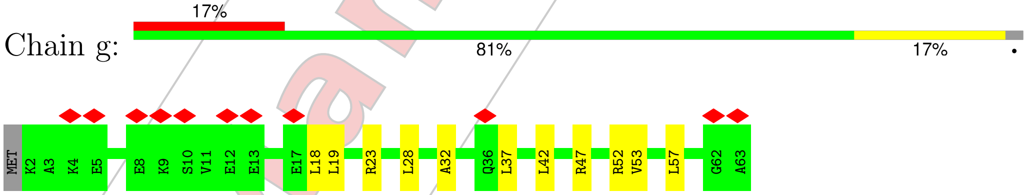

• Molecule 17: Small ribosomal subunit protein bS6

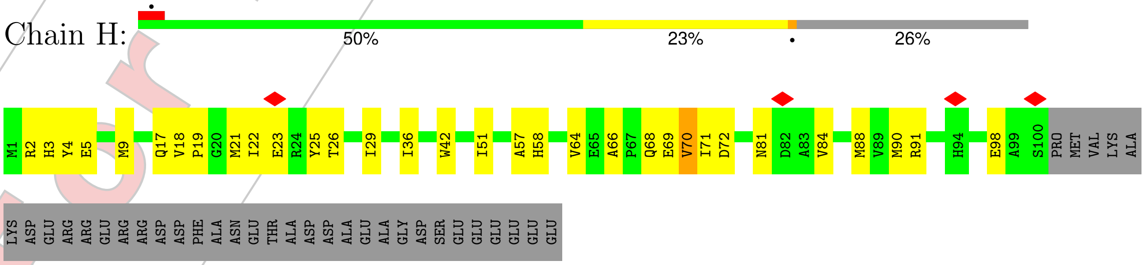

For Manuscript Review

• Molecule 18: 50S ribosomal protein L30

Chain h: 19% 73% 24% • •

MET A2 K3 T4 I7 A13 R16 L24 L25 G26 L27 V36 E37 R38 E39 D40 T41 P42 A43 I44 R45 G46 M47 I48 M54 E58 E59

• Molecule 19: 23S ribosomal RNA

Chain I: 13% 47% 44% 9%

G1 G2 U3 U4 A5 C8 G9 U12 G15 C16 G17 U18 A19 C20 A21 G24 U25 G26 G27 A28 U29 G30 C31 C32 C33 U34 G35 C36 C37 A38 G39 U40 C41 A44 G45 G46 C47 G51 A52 G60 A63 A64 U65 C66 A71 U72 A73 A74 G75 C76 G77

U78 C79 G80 G81 U82 A83 A84 G85 G86 U87 G88 U92 G93 A94 A95 C96 C97 G98 U99 U100 A101 U102 A103 C106 G107 G117 A118 A119 U120 G121 G122 A125 A131 G132 U133 G134 U135 G136 U137 U138 U139 C140 G141 A142 C143 A144 C145 A146 C147 U148 A152 U153 U154 A155

A160 A161 U162 C163 C164 A165 U166 A167 U171 A172 G177 G178 A181 A182 C183 C184 G185 G189 A190 A191 C192 A196 A197 C198 A199 U200 C201 C210 A213 G214 G215 A216 A217 A218 A221 A222 A223 U224 C228 C229 G230 A231 G232 A233 C239 C240 A241 A244

G245 G248 C249 G250 A251 G263 C264 A265 G266 C267 C268 C269 A270 G271 A272 G273 C274 C275 U276 G277 A278 A279 A282 G283 U284 G285 U286 G287 U288 G289 U290 G291 G295 U296 G297 G298 A299 C302 G303 U304 C305 U306 G307 G308 A309 A310 A311 G319 A320 A330 G338

A345 A346 A347 A348 U349 G350 C351 A352 C353 A354 U355 G356 C357 U358 G359 U360 G361 A362 G363 C364 U365 C366 G367 A371 G372 U373 A374 G375 G376 C377 G386 A391 U395 G396 U399 G400 A401 U402 U403 A404 U405 G406 G411 A412 C413 C414 A415 U416 C417 C418 U419 C420 C421 A422

A423 G424 G425 A428 A429 A432 C435 G438 A439 C445 G446 U451 A454 A457 G458 G463 A466 G469 C475 G476 A477 A478 A479 A480 G481 G491 A492 U499 G500 A503 A504 A505 C509 C510 U511 G512 A513 A514 A515 U521 A522 A529

G530 C531 A532 G533 U534 A538 G539 G543 C544 U545 U546 A547 G548 G549 C550 G551 U552 G553 G561 U562 A563 C564 C565 U566 U567 U568 U569 G570 U571 A572 U573 A574 A575 U576 G577 G578 G579 U580 C581 A582 G583 C584 G585 A586 C587 U588 U589 A590 U591 A592 U593 U594 C595 U596 G597 U598 A599

G600 C601 A602 A603 G604 G605 U606 U607 A613 A614 U615 G620 A621 G622 A627 G628 G629 G630 A631 A632 C633 C634 C635 G636 A637 G638 U639 C640 U641 U642 A643 A644 C645 U646 G647 U652 U653 A654 A655 G656 U657 U658 G659 C660 A661 G664 U665 A666 U667 A668 C671 C672 C673 G674

A675 A676 C680 G681 G682 A685 U686 A689 G690 A693 C698 U703 G704 A705 A706 G707 U710 G711 G712 G713 U714 A715 A716 C717 A718 A721 A722 C723 U724 G725 G726 A727 G728 G729 A730 A734 A742 A743 U744 G745 U746 U747 G748 A753 U754 U755 A756 G757

G760 A761 U762 G763 A764 C765 G774 G775 G776 A782 A783 G784 G785 C786 C787 A788 A789 U790 C791 A792 A793 C796 G797 A802 G805 C806 U807 G808 G809 C812 U813 C814 C815 C816 C817 G818 A819 A820 U827 U828 A829 G830 G831 U832 A833 G834 C835 G836 U839 C840 G841 U842

G843 A844 A845 U846 U847 C848 A849 U850 C851 G856 G857 G858 G859 A863 G864 U872 C873 G874 G875 C876 A877 A878 G879 G880 G881 G882 G883 U884 C885 A U C C C G A892 C893 U894 U895 A896 C897 C898 A899 C903 G904 G907 A910 A911 G914 C915 G916 A917 A918

For Manuscript Review

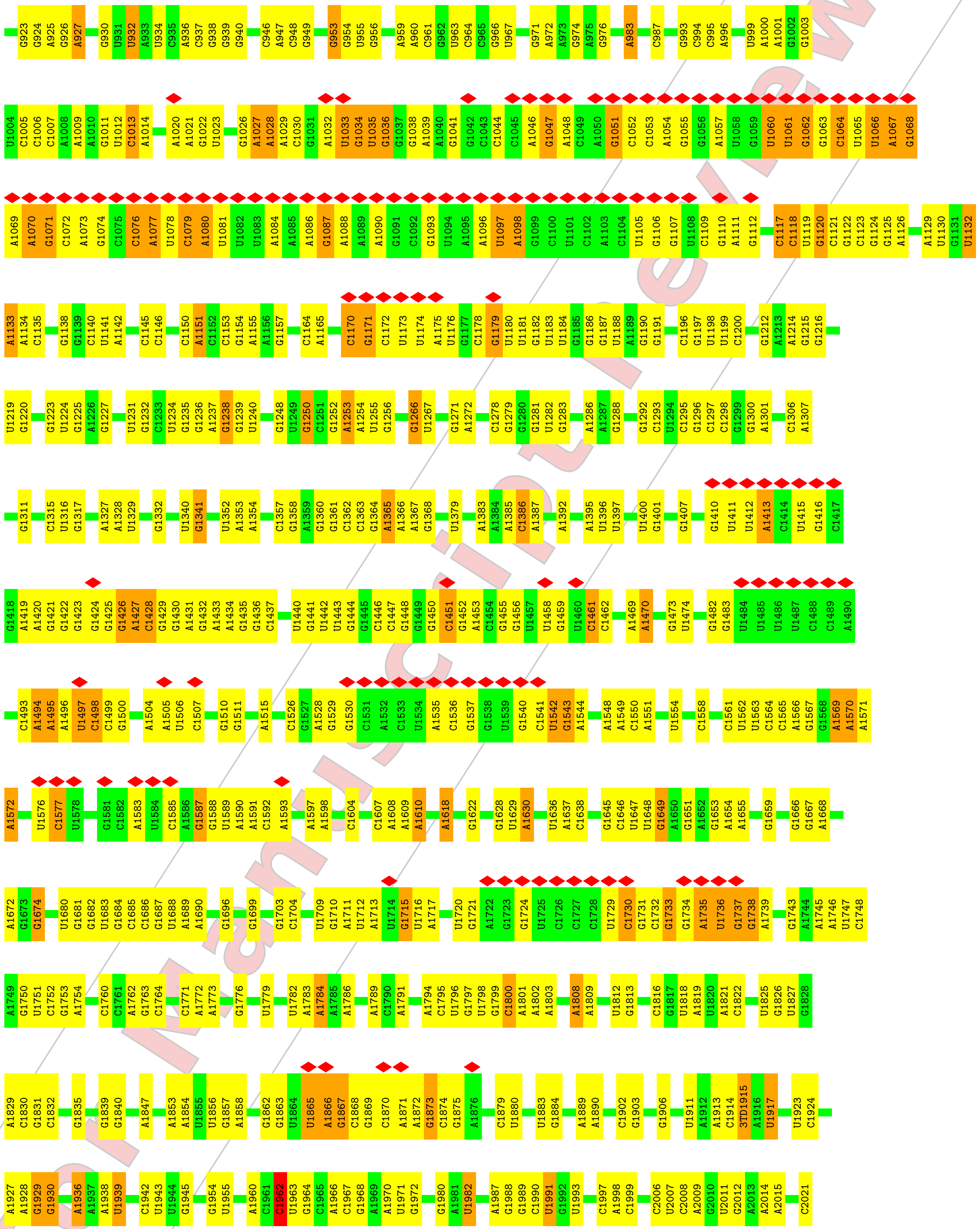

For Manuscript Review

U2022 C2023 G2024 C2025 U2026 A2030 A2031 G2032 A2033 U2034 G2035 C2036 A2037 G2038 U2039 G2040 C2043 C2047 G2048 G2049 C2050 A2051 C2055 G2056 A2059 A2060 G2061 C2064 C2065 C2066 G2067 U2068 G2069 A2070 A2071 C2072 C2073 U2074 U2075 C2078 U2079 A2080 U2081 A2082 U2086 G2087 A2088 G2093 C2096 A2097

U2098 U2099 G2100 A2101 G2102 C2103 C2104 U2105 U2106 G2107 A2108 U2109 G2110 U2111 G2112 U2113 A2114 G2115 G2116 A2117 U2118 A2119 G2120 C2121 U2122 G2123 G2124 G2125 A2126 G2127 G2128 C2129 U2130 U2131 U2132 G2133 A2134 A2135 G2136 U2137 G2138 U2139 G2140 G2141 A2142 C2143 G2144 C2145 C2146 A2147 G2148 U2149 C2150 U2151 G2152 C2153 A2154 U2155 G2156 G2157

A2158 G2159 C2160 C2161 G2162 A2163 C2164 C2165 U2166 U2167 G2168 A2169 A2170 A2171 U2172 A2173 C2174 C2175 A2176 C2177 C2178 C2179 U2180 U2181 U2182 A2183 A2184 U2185 G2186 U2187 U2188 U2189 G2190 A2191 U2192 G2193 U2194 U2195 C2196 U2197 A2198 G2204 C2208 A2211 C2215 G2216 A2225 C2226 A2227 G2228 U2229 G2230 U2231 C2232 U2233

G2234 G2235 U2236 G2237 G2238 G2239 U2240 A2241 G2242 U2243 U2244 U2245 G2246 A2247 C2248 G2251 C2258 U2262 C2263 A2266 U2267 A2268 G2269 A2270 G2271 U2272 A2273 A2274 G2279 G2282 C2283 G2286 A2287 A2288 G2289 G2290 U2291 U2292 G2293 G2294 C2295 U2296 A2297 A2298 U2299 C2300 C2301 U2302 G2303 G2304 U2305 C2306 G2307

G2308 A2309 C2310 A2311 U2312 G2315 G2316 A2317 G2318 G2319 U2320 U2321 A2322 G2323 U2324 G2330 G2331 C2332 A2333 U2334 A2335 A2336 G2337 C2338 C2339 A2340 U2343 U2344 G2345 A2346 C2347 U2348 G2349 C2350 G2351 G2352 G2353 C2354 G2355 U2356 G2357 G2360 G2361 C2362 G2363 C2364 G2365 A2366 G2367 C2368 A2369 G2370 G2371 U2372 G2373 C2374

G2375 A2376 A2377 A2378 G2379 C2380 A2381 G2382 G2383 U2384 C2385 A2386 U2387 A2388 G2389 U2390 G2391 A2392 U2393 C2394 C2395 G2396 G2397 U2398 G2399 G2400 U2401 U2402 G2405 A2406 A2411 A2412 A2418 U2419 C2420 U2423 C2427 G2428 G2429 A2430 U2431 A2432 A2433 A2434 A2435 U2441 C2442 C2443 G2444 G2445 A2448 U2449

U2457 G2458 A2459 A2468 A2469 G2470 A2471 G2472 U2473 U2474 C2475 A2476 U2477 A2478 U2479 C2483 G2484 G2485 C2486 U2491 G2494 G2495 C2498 C2499 U2500 C2501 G2502 A2503 U2504 G2505 U2506 C2507 G2508 A2513 U2514 C2515 A2516 C2517 A2518 U2519 C2520 C2521 U2522 G2523 G2524 C2527 U2528 G2529 A2530 A2531 G2532 U2533 A2534

G2535 G2536 U2537 C2538 C2539 A2542 G2543 G2544 G2545 U2546 A2547 U2548 U2552 G2553 U2554 U2555 C2556 G2557 C2558 U2563 A2564 A2565 A2566 G2567 U2568 A2572 C2575 G2576 A2577 G2578 C2579 U2580 G2581 G2582 G2583 U2584 U2585 U2586 A2589 U2590 C2591 G2592 G2595 A2598 A2602 G2603 U2604 U2605 C2606 G2607 G2608

U2609 U2613 A2614 U2615 C2616 U2617 G2618 C2626 G2627 C2628 U2629 G2630 G2631 A2632 G2633 A2634 A2635 C2636 U2637 G2638 G2641 A2642 G2643 G2644 G2645 C2646 U2647 C2648 C2649 U2650 C2651 C2652 U2653 A2654 G2655 C2658 G2659 A2660 G2661 A2662 G2663 C2664 G2669 A2670 C2671 U2672 G2673 G2674 G2677 C2678 A2679 U2680 C2683

U2684 G2685 G2686 U2687 G2688 U2689 U2690 U2698 C2699 A2700 U2701 G2702 C2703 C2704 A2705 A2706 G2709 C2710 A2711 G2714 C2715 C2716 C2717 C2718 G2719 U2720 A2721 G2722 C2723 U2724 A2725 A2726 A2727 U2728 G2729 C2730 G2731 G2732 A2733 G2737 A2738 U2739 A2740 A2741 G2742 U2743 G2744 C2745 U2746 G2747 A2748 A2749 A2750 G2751 C2752 A2753

U2754 C2755 U2756 A2757 A2758 G2759 C2760 A2761 C2762 G2763 A2764 C2771 C2772 C2773 C2774 G2775 A2776 G2777 A2778 A2781 U2784 C2785 C2788 C2789 U2790 G2791 A2792 C2793 U2796 U2797 U2798 A2799 A2800 G2801 G2802 G2803 U2804 C2805 A2809 G2812 A2813 A2814 U2818 G2819 A2820 A2821 G2822 A2823 C2830 G2831 U2832

U2833 A2837 G2838 G2839 C2840 C2841 G2842 U2845 G2846 U2847 G2848 U2849 G2852 C2853 G2854 C2855 A2856 G2857 C2858 G2859 A2860 G2867 A2868 G2869 A2872 A2873 G2876 C2880 U2881 A2882 A2883 U2884 A2887 U2891 G2892 A2893 G2894 G2895 C2896 U2897 U2898 A2899 A2900 C2901 C2902 U2903 U2904

• Molecule 20: 50S ribosomal protein L32

Chain i: 72% 25%

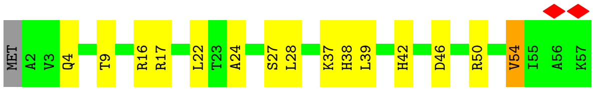

• Molecule 21: 5S ribosomal RNA

Chain J:

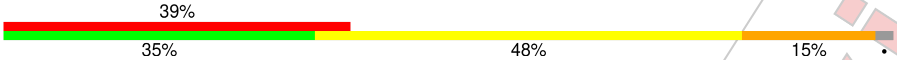

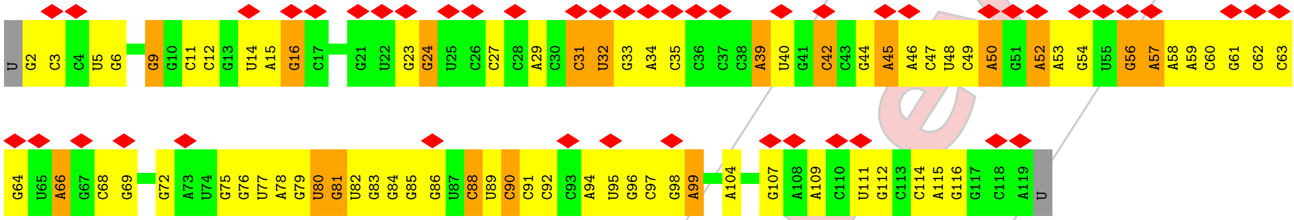

• Molecule 22: 50S ribosomal protein L33

Chain j:

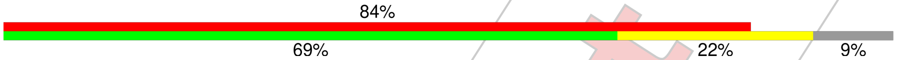

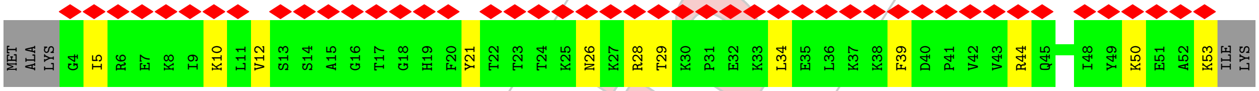

• Molecule 23: 50S ribosomal protein L2

Chain K:

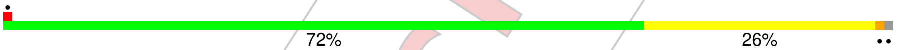

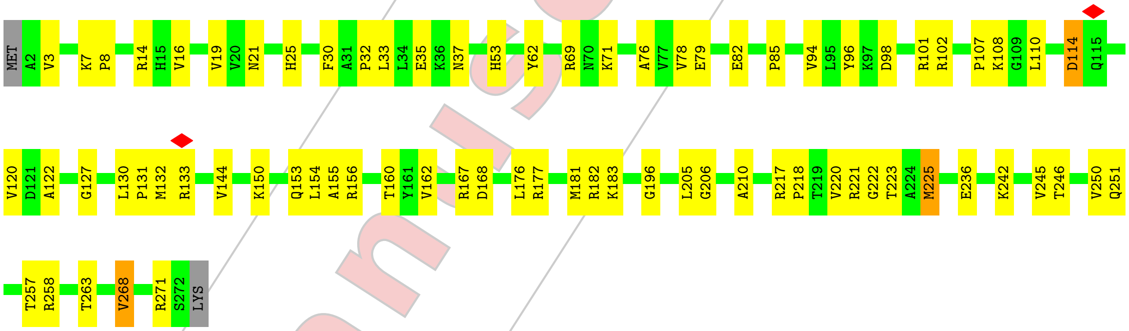

• Molecule 24: 50S ribosomal protein L34

Chain k:

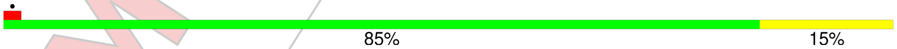

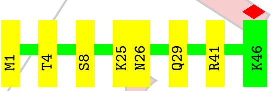

• Molecule 25: Large ribosomal subunit protein uL3

Chain L:

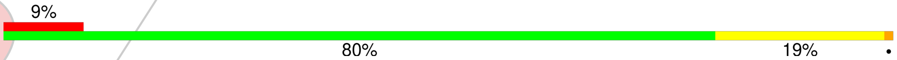

For Manuscript Review

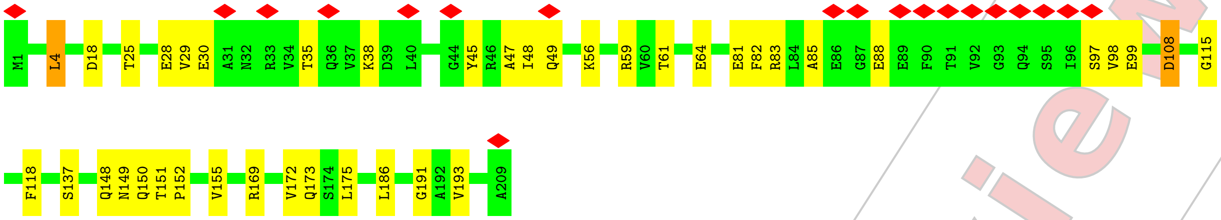

• Molecule 26: 50S ribosomal protein L35

Chain l:

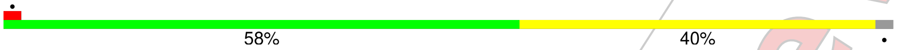

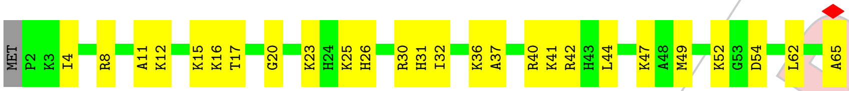

• Molecule 27: Large ribosomal subunit protein uL4

Chain M:

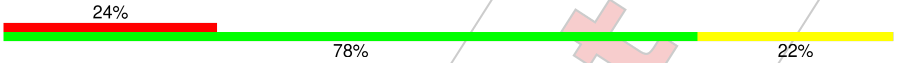

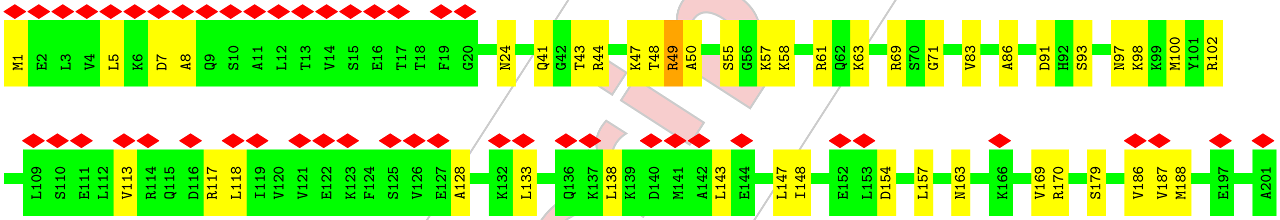

• Molecule 28: 50S ribosomal protein L36

Chain m:

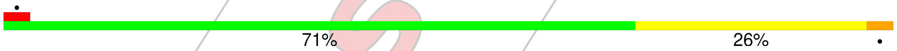

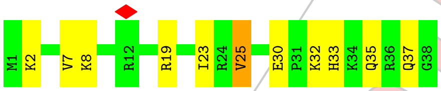

• Molecule 29: Large ribosomal subunit protein uL5

Chain N:

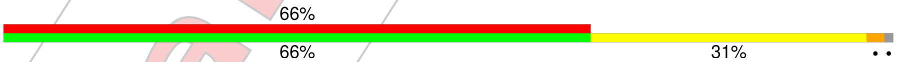

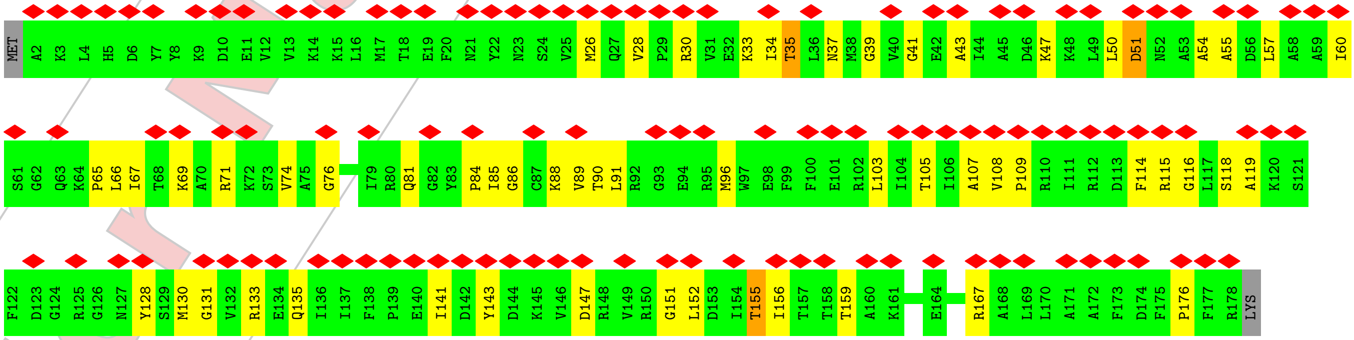

• Molecule 30: Small ribosomal subunit protein uS7

For Manuscript Review

Chain n:

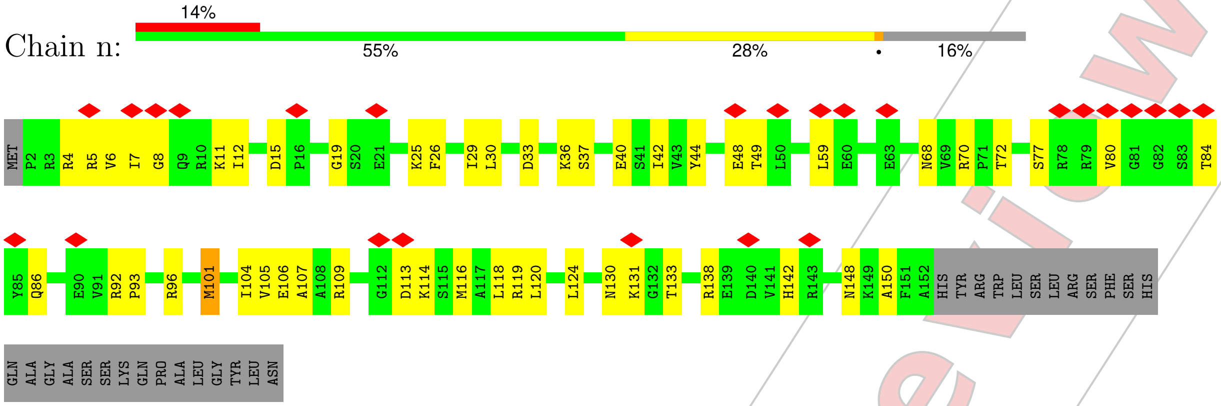

• Molecule 31: Large ribosomal subunit protein uL6

Chain O:

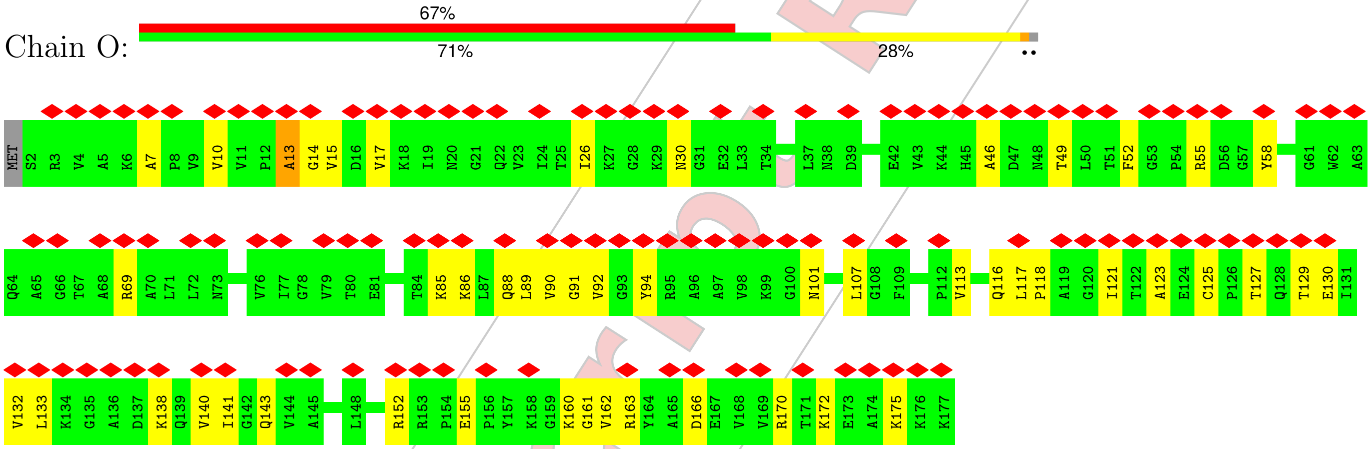

• Molecule 32: Small ribosomal subunit protein uS8

Chain o:

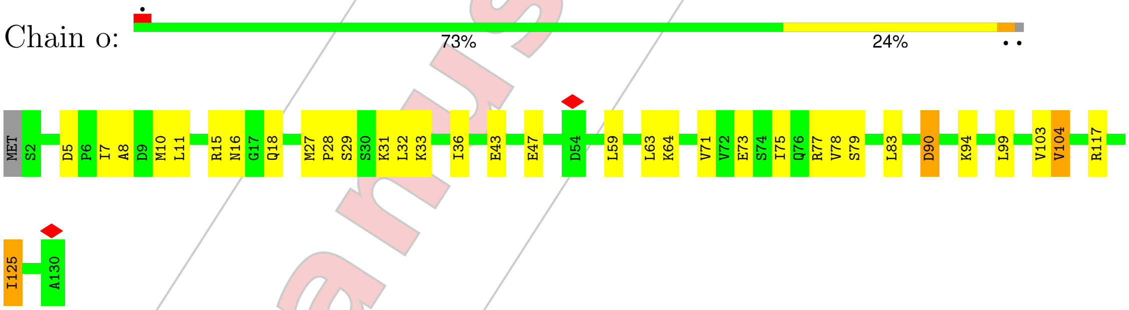

• Molecule 33: Large ribosomal subunit protein bL9

Chain P:

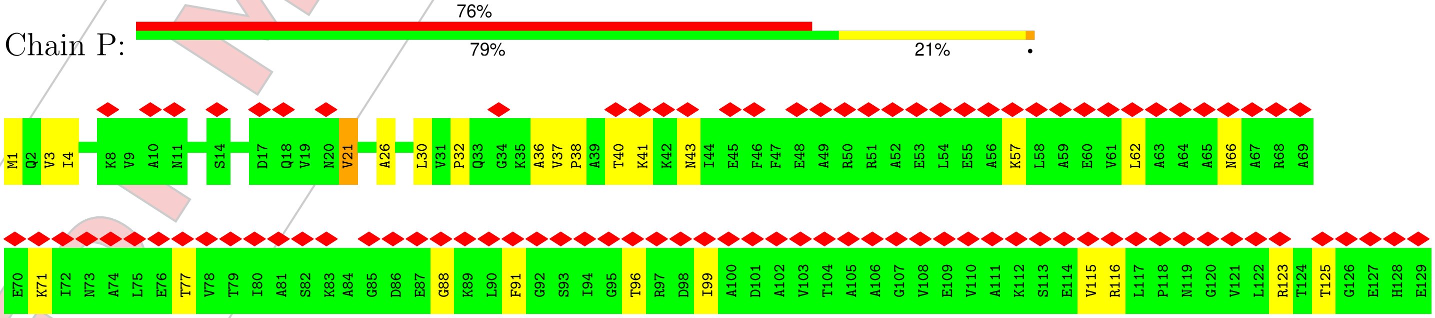

For Manuscript Review

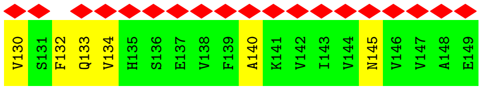

• Molecule 34: Small ribosomal subunit protein uS9

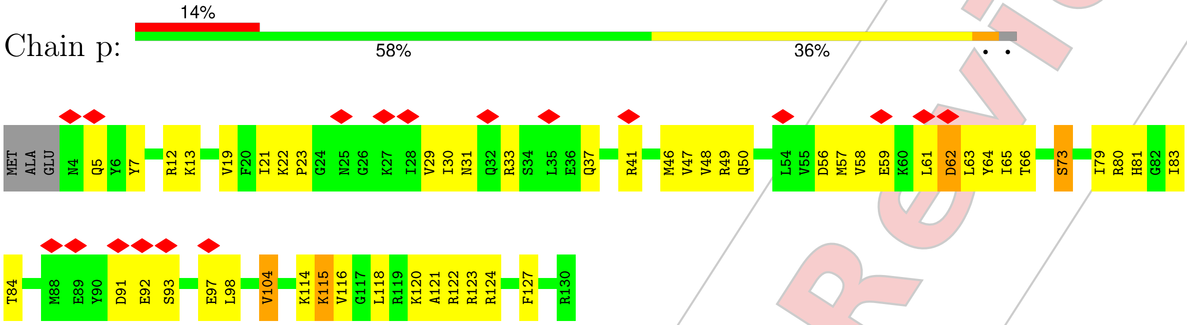

• Molecule 35: 50S ribosomal protein L31

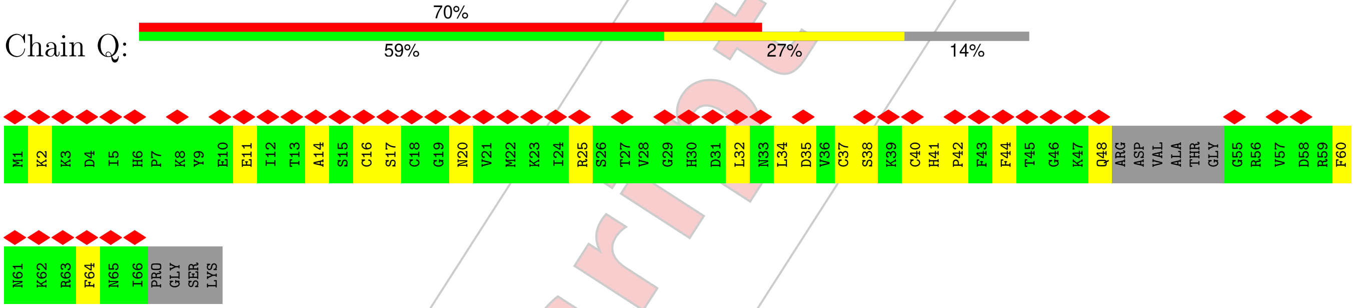

• Molecule 36: Small ribosomal subunit protein uS10

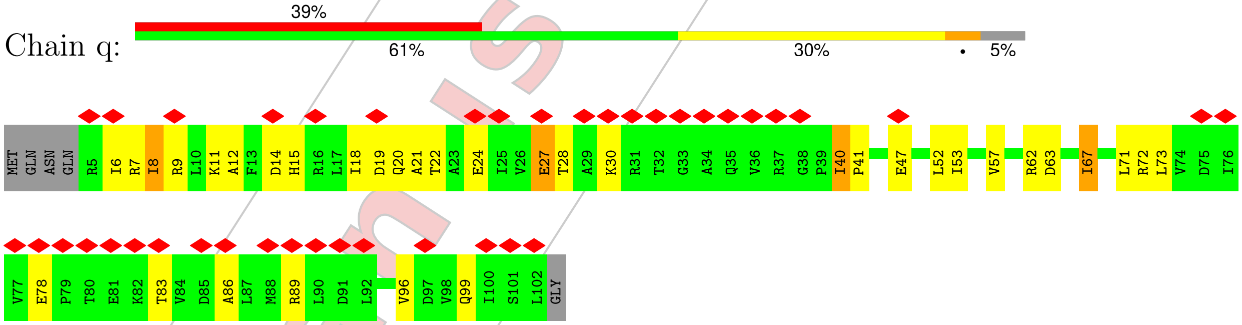

• Molecule 37: Large ribosomal subunit protein uL13

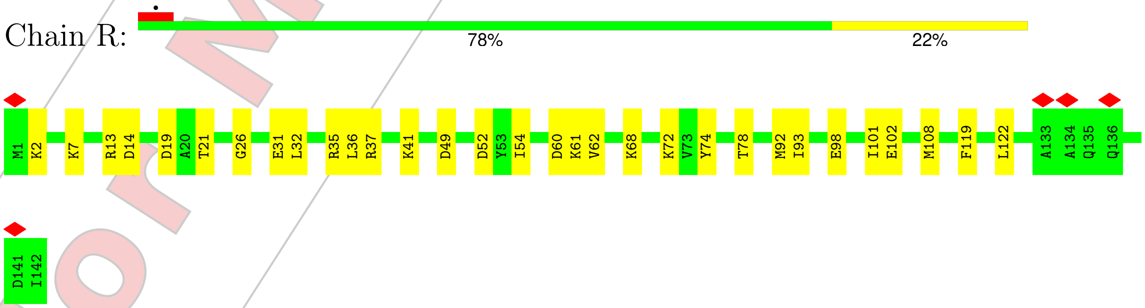

• Molecule 38: 30S ribosomal protein S11

For Manuscript Review

Chain r:

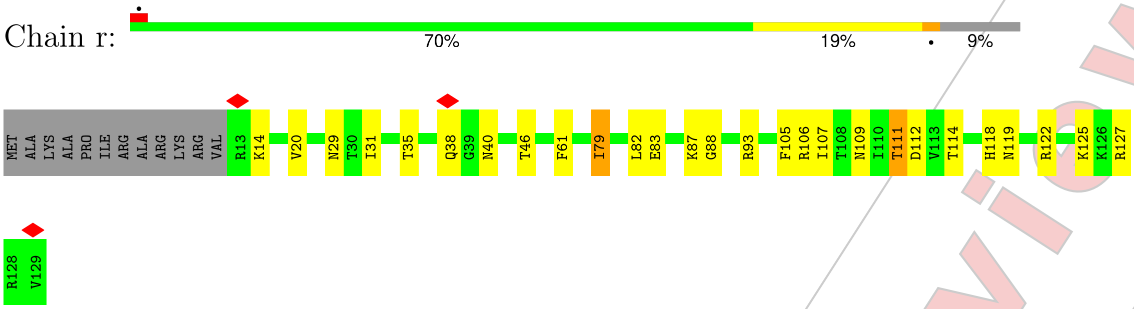

• Molecule 39: Large ribosomal subunit protein uL14

Chain S:

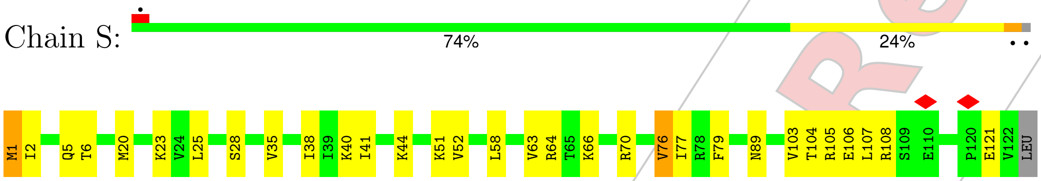

• Molecule 40: 30S ribosomal protein S12

Chain t:

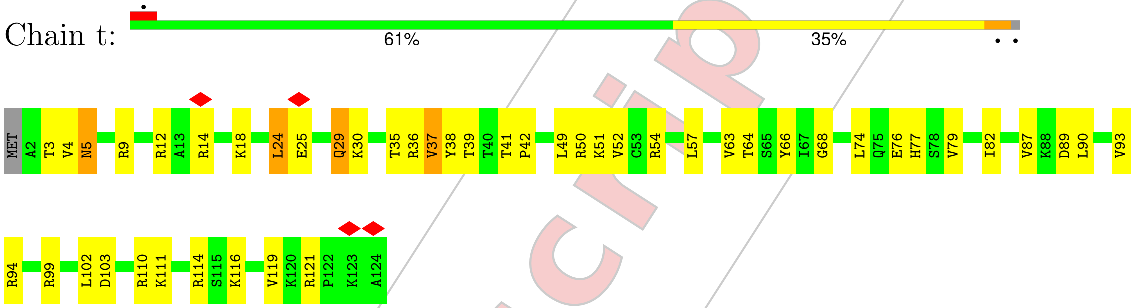

• Molecule 41: Large ribosomal subunit protein uL15

Chain T:

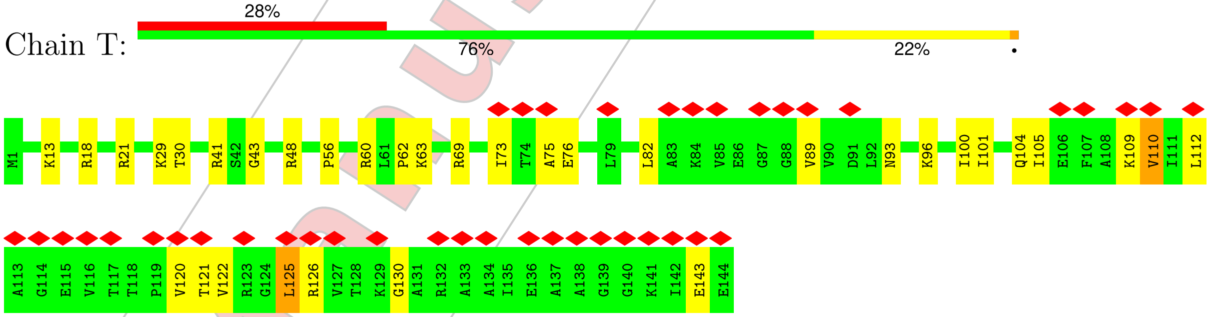

• Molecule 42: Small ribosomal subunit protein uS13

Chain u:

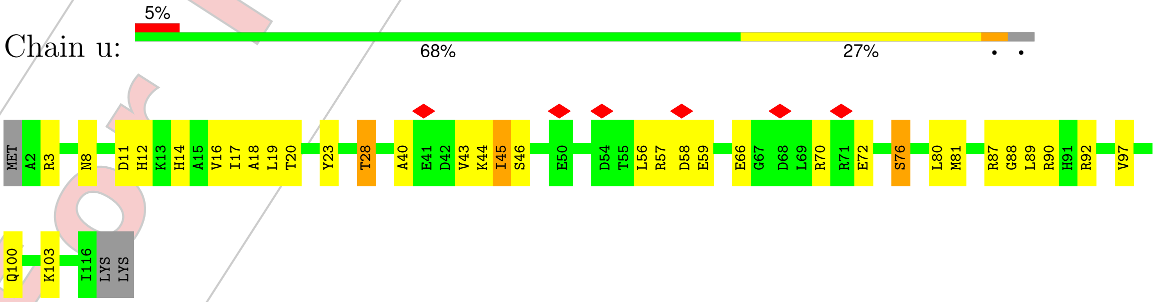

For Manuscript Review

- Molecule 43: Large ribosomal subunit protein uL16

Chain U:

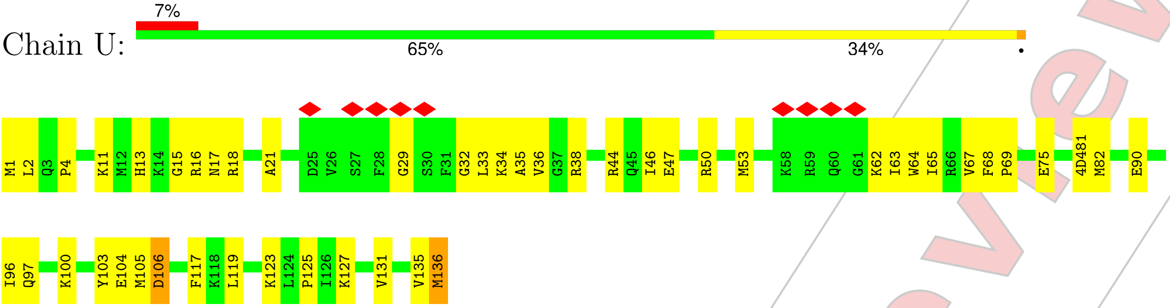

- Molecule 44: Small ribosomal subunit protein uS14

Chain v:

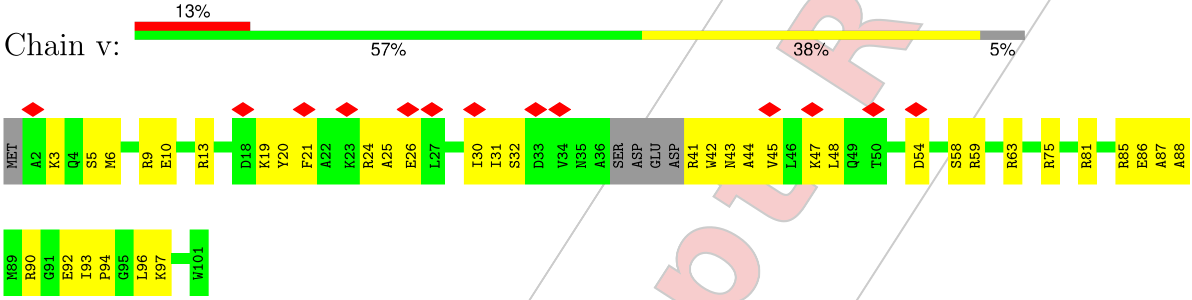

- Molecule 45: Large ribosomal subunit protein bL17

Chain V:

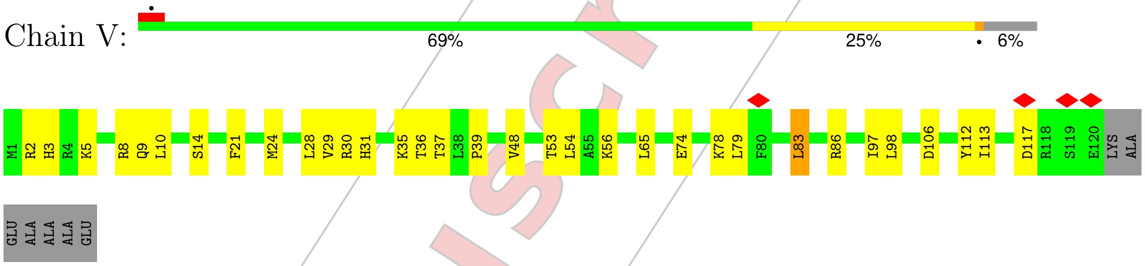

- Molecule 46: Small ribosomal subunit protein uS15

Chain w:

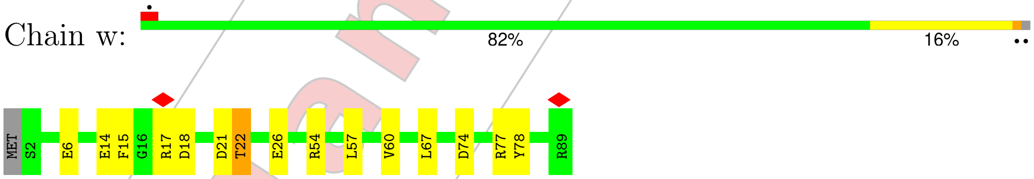

- Molecule 47: Large ribosomal subunit protein uL18

Chain W:

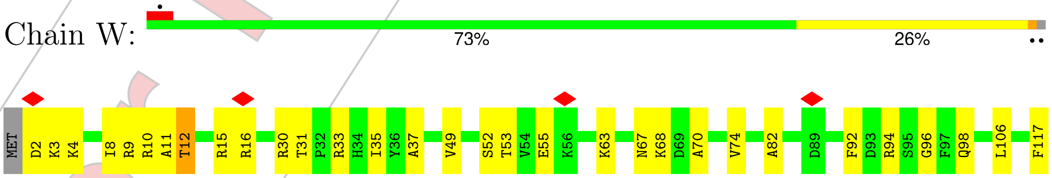

- Molecule 48: Small ribosomal subunit protein bS16

Chain x:

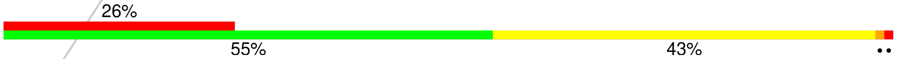

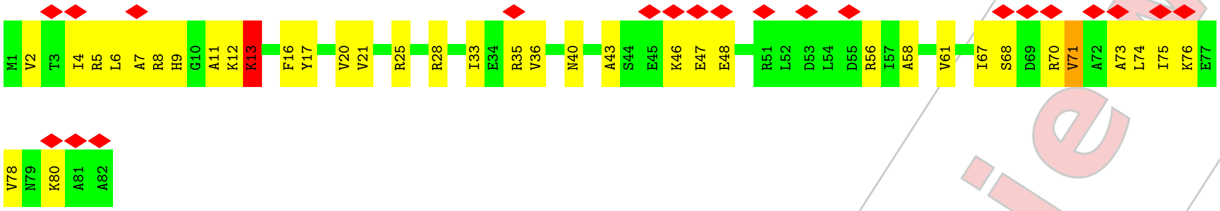

• Molecule 49: Large ribosomal subunit protein bL19

Chain X:

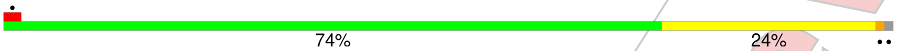

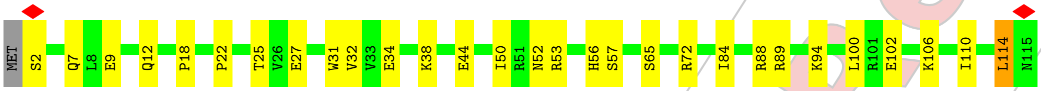

• Molecule 50: Small ribosomal subunit protein uS17

Chain y:

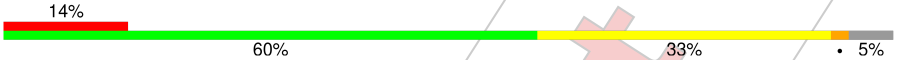

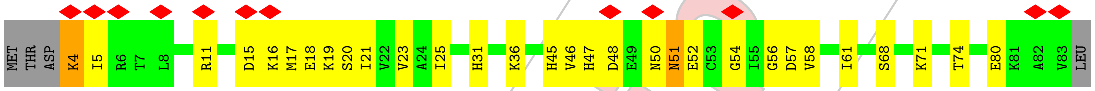

• Molecule 51: 50S ribosomal protein L20

Chain Y:

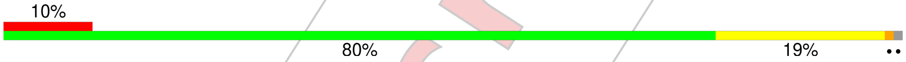

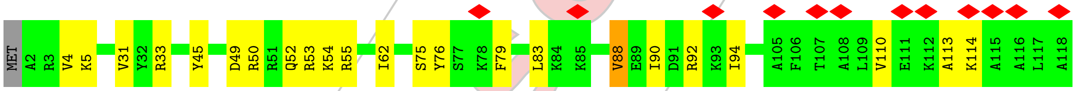

• Molecule 52: Small ribosomal subunit protein bS18

Chain z:

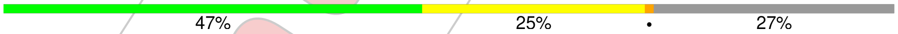

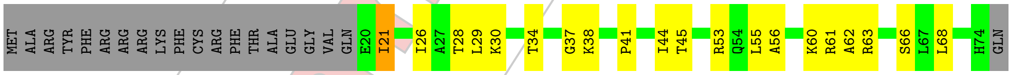

For Manuscript Review

4 Experimental information (i)

Property	Value	Source
EM reconstruction method	SINGLE PARTICLE	Depositor
Imposed symmetry	POINT, Not provided	
Number of particles used	560442	Depositor
Resolution determination method	FSC 0.143 CUT-OFF	Depositor
CTF correction method	PHASE FLIPPING AND AMPLITUDE CORRECTION	Depositor
Microscope	TFS KRIOS	Depositor
Voltage (kV)	300	Depositor
Electron dose ($e^-/\text{Å}^2$)	60	Depositor
Minimum defocus (nm)	500	Depositor
Maximum defocus (nm)	2500	Depositor
Magnification	Not provided	
Image detector	FEI FALCON IV (4k x 4k)	Depositor
Maximum map value	30.211	Depositor
Minimum map value	-15.495	Depositor
Average map value	0.000	Depositor
Map value standard deviation	1.000	Depositor
Recommended contour level	3	Depositor
Map size (Å)	425.984, 425.984, 425.984	wwPDB
Map dimensions	512, 512, 512	wwPDB
Map angles (°)	90.0, 90.0, 90.0	wwPDB
Pixel spacing (Å)	0.832, 0.832, 0.832	Depositor

For Manuscript Review

5 Model quality (i)

5.1 Standard geometry (i)

Bond lengths and bond angles in the following residue types are not validated in this section: 5MC, OMU, PSU, G7M, 5MU, 3TD, H2U, 1MG, OMG, 4D4, 2MG, MG, 6MZ, OMC, 2MA

The Z score for a bond length (or angle) is the number of standard deviations the observed value is removed from the expected value. A bond length (or angle) with $|Z| > 5$ is considered an outlier worth inspection. RMSZ is the root-mean-square of all Z scores of the bond lengths (or angles).

Mol	Chain	Bond lengths		Bond angles	
		RMSZ	#|Z| >5	RMSZ	#|Z| >5
1	C	0.14	0/36668	0.33	9/57204 (0.0%)
2	1	0.24	0/667	0.58	0/897
3	Z	0.15	0/829	0.39	0/1107
4	2	0.19	0/665	0.40	0/881
5	4	0.12	0/864	0.29	0/1156
6	3	0.12	0/598	0.32	0/792
7	b	0.12	0/744	0.33	0/994
8	c	0.15	0/787	0.37	0/1051
9	D	0.14	0/1735	0.36	0/2338
10	d	0.16	0/766	0.42	0/1025
11	E	0.12	0/1651	0.32	0/2225
12	e	0.12	0/582	0.38	0/769
13	F	0.16	0/1665	0.44	0/2227
14	f	0.11	0/635	0.29	0/848
15	G	0.15	0/1118	0.41	0/1504
16	g	0.13	0/502	0.29	0/667
17	H	0.17	0/835	0.45	0/1128
18	h	0.12	0/453	0.30	0/605
19	I	0.12	0/69168	0.30	20/107901 (0.0%)
20	i	0.09	0/450	0.23	0/599
21	J	0.12	0/2828	0.29	0/4410
22	j	0.08	0/416	0.25	0/554
23	K	0.14	0/2132	0.32	0/2866
24	k	0.11	0/380	0.29	0/498
25	L	0.13	0/1586	0.32	0/2134
26	l	0.10	0/513	0.25	0/676
27	M	0.12	0/1571	0.29	0/2113
28	m	0.16	0/303	0.37	0/397
29	N	0.16	0/1434	0.39	0/1926
30	n	0.15	0/1195	0.41	0/1602
31	O	0.13	0/1343	0.36	0/1816
32	o	0.12	0/989	0.27	0/1326

For Manuscript Review

Mol	Chain	Bond lengths		Bond angles	
		RMSZ	#|Z| >5	RMSZ	#|Z| >5
33	P	0.12	0/1122	0.36	0/1515
34	p	0.22	0/1034	0.59	0/1375
35	Q	0.10	0/488	0.31	0/649
36	q	0.13	0/796	0.36	0/1077
37	R	0.11	0/1152	0.31	0/1551
38	r	0.14	0/893	0.35	0/1205
39	S	0.14	0/947	0.34	0/1268
40	t	0.16	0/969	0.44	2/1300 (0.2%)
41	T	0.10	0/1062	0.33	0/1413
42	u	0.16	0/900	0.49	0/1204
43	U	0.13	0/1081	0.34	0/1443
44	v	0.19	0/785	0.36	0/1043
45	V	0.10	0/973	0.27	0/1301
46	w	0.13	0/718	0.28	0/959
47	W	0.11	0/902	0.28	0/1209
48	x	0.21	0/659	0.68	0/884
49	X	0.13	0/929	0.29	0/1242
50	y	0.18	0/657	0.54	0/881
51	Y	0.11	0/960	0.24	0/1278
52	z	0.13	0/462	0.28	0/621
All	All	0.13	0/153561	0.33	31/229654 (0.0%)

There are no bond length outliers.

All (31) bond angle outliers are listed below:

Mol	Chain	Res	Type	Atoms	Z	Observed(^{o})	Ideal(^{o})
1	C	69	G	OP2-P-O3'	-11.99	72.03	108.00
1	C	70	A	O5'-P-OP2	-11.55	73.35	108.00
1	C	94	C	OP1-P-O3'	-9.34	79.98	108.00
19	I	673	C	OP1-P-O3'	-9.03	80.92	108.00
19	I	2723	C	OP1-P-O3'	-9.02	80.94	108.00
19	I	2230	G	OP1-P-O3'	-8.91	81.25	108.00
19	I	2674	G	OP1-P-O3'	-8.91	81.28	108.00
19	I	2050	C	OP1-P-O3'	-8.86	81.43	108.00
19	I	2360	G	OP1-P-O3'	-8.84	81.47	108.00
19	I	1655	A	OP1-P-O3'	-8.75	81.75	108.00
19	I	2598	A	OP1-P-O3'	-8.74	81.78	108.00
19	I	2717	C	OP1-P-O3'	-8.72	81.85	108.00
19	I	2564	A	OP1-P-O3'	-8.66	82.00	108.00
19	I	2717	C	OP2-P-O3'	-8.45	82.66	108.00
19	I	2598	A	OP2-P-O3'	-8.43	82.71	108.00
19	I	1655	A	OP2-P-O3'	-8.39	82.83	108.00

Continued on next page...

For Manuscript Review

Continued from previous page...

Mol	Chain	Res	Type	Atoms	Z	Observed(^{o})	Ideal(^{o})
19	I	2564	A	OP2-P-O3'	-8.38	82.85	108.00
19	I	2360	G	OP2-P-O3'	-8.36	82.92	108.00
19	I	673	C	OP2-P-O3'	-8.24	83.28	108.00
19	I	2723	C	OP2-P-O3'	-8.14	83.58	108.00
1	C	69	G	OP1-P-O3'	-8.09	83.72	108.00
19	I	2230	G	OP2-P-O3'	-8.05	83.85	108.00
19	I	2050	C	OP2-P-O3'	-7.97	84.09	108.00
19	I	2674	G	OP2-P-O3'	-7.94	84.19	108.00
1	C	94	C	OP2-P-O3'	-7.05	86.86	108.00
1	C	70	A	OP1-P-OP2	6.62	139.47	119.60
1	C	70	A	O5'-P-OP1	-6.62	88.15	108.00
1	C	69	G	O3'-P-O5'	6.46	113.69	104.00
1	C	379	C	C4'-C3'-C2'	-5.19	97.41	102.60
40	t	74	LEU	CA-C-N	5.05	130.79	121.85
40	t	74	LEU	C-N-CA	5.05	130.79	121.85

There are no chirality outliers.

There are no planarity outliers.

5.2 Too-close contacts (i)

In the following table, the Non-H and H(model) columns list the number of non-hydrogen atoms and hydrogen atoms in the chain respectively. The H(added) column lists the number of hydrogen atoms added and optimized by MolProbity. The Clashes column lists the number of clashes within the asymmetric unit, whereas Symm-Clashes lists symmetry-related clashes.

Mol	Chain	Non-H	H(model)	H(added)	Clashes	Symm-Clashes
1	C	32745	0	16474	700	0
2	1	651	0	675	27	0
3	Z	816	0	839	14	0
4	2	659	0	709	26	0
5	4	857	0	922	20	0
6	3	590	0	629	13	0
7	b	738	0	807	12	0
8	c	779	0	831	25	0
9	D	1704	0	1732	38	0
10	d	753	0	780	23	0
11	E	1624	0	1696	38	0
12	e	575	0	592	19	0
13	F	1643	0	1707	54	0
14	f	625	0	652	19	0

Continued on next page...

For Manuscript Review

Continued from previous page...

Mol	Chain	Non-H	H(model)	H(added)	Clashes	Symm-Clashes
15	G	1105	0	1148	28	0
16	g	501	0	531	7	0
17	H	817	0	808	24	0
18	h	449	0	488	9	0
19	I	62271	0	31340	1060	0
20	i	444	0	458	12	0
21	J	2529	0	1281	80	0
22	j	409	0	440	8	0
23	K	2093	0	2166	56	0
24	k	377	0	418	6	0
25	L	1565	0	1616	29	0
26	l	504	0	572	22	0
27	M	1552	0	1619	34	0
28	m	302	0	343	8	0
29	N	1410	0	1444	44	0
30	n	1181	0	1238	34	0
31	O	1323	0	1371	37	0
32	o	979	0	1031	27	0
33	P	1111	0	1148	21	0
34	p	1022	0	1070	36	0
35	Q	480	0	482	14	0
36	q	786	0	828	23	0
37	R	1129	0	1162	23	0
38	r	877	0	887	29	0
39	S	938	0	1012	23	0
40	t	955	0	1016	31	0
41	T	1053	0	1129	29	0
42	u	891	0	952	24	0
43	U	1075	0	1154	30	0
44	v	774	0	824	30	0
45	V	960	0	1000	23	0
46	w	710	0	728	12	0
47	W	892	0	923	27	0
48	x	649	0	666	33	0
49	X	917	0	962	19	0
50	y	648	0	691	26	0
51	Y	947	0	1019	18	0
52	z	455	0	478	13	0
53	C	90	0	0	0	0
53	I	204	0	0	0	0
53	J	5	0	0	0	0
53	K	1	0	0	0	0

Continued on next page...

For Manuscript Review

Continued from previous page...

Mol	Chain	Non-H	H(model)	H(added)	Clashes	Symm-Clashes
53	L	1	0	0	0	0
53	V	2	0	0	0	0
53	Y	1	0	0	0	0
53	i	1	0	0	0	0
53	l	1	0	0	0	0
53	q	1	0	0	0	0
All	All	142146	0	95488	2724	0

The all-atom clashscore is defined as the number of clashes found per 1000 atoms (including hydrogen atoms). The all-atom clashscore for this structure is 12.

All (2724) close contacts within the same asymmetric unit are listed below, sorted by their clash magnitude.

Atom-1	Atom-2	Interatomic distance (Å)	Clash overlap (Å)
19:I:2747:G:H21	19:I:2757:A:N6	1.38	1.20
19:I:2747:G:N2	19:I:2757:A:H62	1.41	1.18
19:I:2741:A:H62	19:I:2763:G:N2	1.44	1.13
19:I:2741:A:N6	19:I:2763:G:H21	1.47	1.12
21:J:72:G:N2	21:J:104:A:H62	1.56	1.03
19:I:707:G:H1	19:I:724:U:H3	1.02	1.02
21:J:72:G:H21	21:J:104:A:N6	1.58	1.01
21:J:78:A:H62	21:J:98:G:N2	1.58	1.01
19:I:2144:G:H21	19:I:2147:A:H62	1.00	0.96
21:J:72:G:H21	21:J:104:A:H62	0.95	0.94
19:I:2351:G:N2	19:I:2366:A:H62	1.66	0.93
19:I:1529:G:H1	19:I:1542:U:H3	1.00	0.92
19:I:2351:G:H21	19:I:2366:A:N6	1.68	0.91
21:J:78:A:H62	21:J:98:G:H21	0.93	0.89
21:J:78:A:N6	21:J:98:G:H21	1.68	0.89
19:I:1123:C:H2'	19:I:1124:G:H8	1.37	0.88
1:C:1020:G:H1	1:C:1029:A:H2	1.21	0.88
19:I:2351:G:H21	19:I:2366:A:H62	0.90	0.88
1:C:63:C:H5"	1:C:377:A:H61	1.39	0.87
19:I:1483:G:H1	19:I:1506:U:H3	1.21	0.87
19:I:2144:G:N2	19:I:2147:A:H62	1.74	0.85
19:I:2144:G:H21	19:I:2147:A:N6	1.74	0.84
1:C:658:G:H22	1:C:735:G:H1	1.22	0.83
19:I:1724:G:H1	19:I:1736:U:H3	0.88	0.83
1:C:916:G:H21	1:C:1392:A:H8	1.24	0.82
19:I:1421:G:H2'	19:I:1422:G:H8	1.46	0.81
19:I:2728:U:HO2'	19:I:2729:G:H8	1.26	0.80

Continued on next page...

For Manuscript Review

Continued from previous page...

Atom-1	Atom-2	Interatomic distance (Å)	Clash overlap (Å)
19:I:177:G:OP2	19:I:177:G:N2	2.12	0.80
1:C:1311:C:O2	2:1:37:ARG:NH2	2.14	0.80
4:2:66:LEU:HD23	4:2:67:ILE:HD13	1.63	0.79
19:I:9:G:N2	19:I:2800:A:H61	1.80	0.79
36:q:47:GLU:HB2	36:q:67:ILE:HB	1.62	0.79
1:C:1020:G:O6	1:C:1029:A:N1	2.16	0.79
19:I:2529:G:H5'	31:O:175:LYS:HB2	1.64	0.78
47:W:12:THR:HA	47:W:15:ARG:HB2	1.64	0.78
19:I:820:A:H4'	19:I:836:G:H22	1.47	0.78
1:C:1153:U:H3	1:C:1175:G:H1	1.32	0.77
8:c:7:ARG:NH2	19:I:85:G:OP1	2.18	0.77
19:I:9:G:H21	19:I:2800:A:N6	1.83	0.77
1:C:431:U:H3	1:C:489:A:H2	1.32	0.77
9:D:67:ILE:HG13	9:D:160:ALA:HB3	1.67	0.76
47:W:31:THR:HG22	47:W:33:ARG:H	1.51	0.76
50:y:15:ASP:HA	50:y:21:ILE:HG22	1.68	0.76
19:I:976:G:HO2'	19:I:1155:A:HO2'	1.34	0.76
19:I:2153:C:H2'	19:I:2154:A:H8	1.51	0.76
21:J:46:A:H4'	47:W:3:LYS:HE3	1.66	0.76
44:v:54:ASP:OD1	44:v:59:ARG:NH1	2.18	0.76
21:J:42:C:H42	29:N:90:THR:HG22	1.49	0.75
1:C:926:C:H5"	30:n:4:ARG:HH21	1.51	0.75
17:H:26:THR:HG23	17:H:36:ILE:HD12	1.67	0.75
21:J:60:C:H2'	21:J:61:G:H8	1.51	0.75
19:I:1779:U:OP2	19:I:1784:A:N6	2.16	0.75
12:e:33:ALA:O	19:I:2353:G:O2'	2.03	0.75
7:b:1:MET:N	19:I:142:A:O2'	2.19	0.74
1:C:1391:C:H5"	1:C:1392:A:H5"	1.69	0.74
19:I:475:C:O2	19:I:479:A:N6	2.20	0.74
4:2:80:THR:HA	4:2:85:LYS:HB3	1.68	0.74
48:x:71:VAL:HA	48:x:74:LEU:HB2	1.70	0.74
1:C:667:G:H2'	1:C:668:G:C8	2.23	0.74
19:I:374:A:H3'	19:I:375:G:H8	1.52	0.74
1:C:1235:G:H2'	1:C:1236:G:H8	1.53	0.73
1:C:475:G:O2'	1:C:477:C:N4	2.21	0.73
3:Z:35:PHE:HB2	3:Z:59:ILE:HB	1.71	0.73
34:p:22:LYS:HB2	34:p:61:LEU:HB3	1.71	0.73
19:I:2139:U:H3	19:I:2152:G:H1	1.35	0.73
1:C:190:A:N7	1:C:215:C:O2'	2.19	0.73
1:C:1441:A:H3'	1:C:1442:C:H5"	1.70	0.73
19:I:807:U:OP2	41:T:41:ARG:NH2	2.21	0.73

Continued on next page...

For Manuscript Review

Continued from previous page...

Atom-1	Atom-2	Interatomic distance (Å)	Clash overlap (Å)
19:I:1034:G:O6	19:I:1122:G:N2	2.18	0.73
19:I:1288:G:OP2	19:I:1288:G:N2	2.18	0.73
19:I:938:G:H2'	19:I:939:G:H8	1.54	0.73
1:C:1342:U:H4'	34:p:122:ARG:HD2	1.71	0.73
12:e:31:VAL:HG21	12:e:82:ILE:HD12	1.69	0.73
30:n:113:ASP:HB3	30:n:119:ARG:HG2	1.70	0.72
19:I:290:U:H3	19:I:350:G:H1	0.81	0.72
19:I:1311:G:OP2	19:I:1311:G:N2	2.16	0.72
19:I:266:G:H1	19:I:425:G:H22	1.35	0.72
19:I:1071:G:H22	19:I:1090:A:H62	1.37	0.72
27:M:187:VAL:C	27:M:188:MET:HE2	2.15	0.72
19:I:2618:G:H21	25:L:155:VAL:HG21	1.54	0.72
1:C:73:A:H8	1:C:90:G:H21	1.37	0.72
1:C:442:A:N6	1:C:478:G:O6	2.23	0.72
19:I:1123:C:H2'	19:I:1124:G:C8	2.23	0.72
1:C:532:A:H5'	40:t:111:LYS:HB2	1.71	0.72
1:C:707:G:H2'	1:C:708:G:C8	2.25	0.72
19:I:2635:A:O2'	25:L:81:GLU:OE2	2.08	0.72
1:C:453:U:H2'	1:C:454:A:C8	2.24	0.71
15:G:157:ARG:NH2	32:o:99:LEU:O	2.22	0.71
19:I:1461:C:O2'	19:I:2702:G:N2	2.22	0.71
1:C:1322:C:H5"	42:u:28:THR:HG21	1.70	0.71
1:C:573:G:O2'	46:w:54:ARG:NH1	2.24	0.71
19:I:2474:U:O4	19:I:2529:G:N2	2.21	0.71
15:G:24:THR:HA	15:G:29:ARG:HA	1.71	0.71
1:C:740:G:H2'	1:C:741:A:C8	2.26	0.71
33:P:38:PRO:O	33:P:43:ASN:ND2	2.23	0.71
19:I:1528:A:N6	19:I:1543:G:O2'	2.22	0.71
1:C:730:C:H2'	1:C:731:A:H8	1.54	0.70
17:H:69:GLU:OE1	17:H:69:GLU:N	2.23	0.70
11:E:154:SER:HB2	11:E:165:THR:HG23	1.73	0.70
19:I:9:G:N2	19:I:2800:A:N6	2.38	0.70
1:C:668:G:H2'	1:C:669:A:H8	1.56	0.70
19:I:414:C:O2'	19:I:1863:G:N2	2.24	0.70
25:L:38:LYS:HG3	25:L:47:ALA:HB3	1.72	0.70
34:p:46:MET:O	34:p:50:GLN:N	2.24	0.70
19:I:2683:C:O2	39:S:70:ARG:NH2	2.22	0.70
19:I:598:U:O4	19:I:659:G:O6	2.10	0.70
19:I:1798:U:OP2	23:K:271:ARG:NH2	2.25	0.70
19:I:1250:G:N7	41:T:18:ARG:NH2	2.40	0.70
33:P:1:MET:N	33:P:21:VAL:O	2.24	0.70

Continued on next page...

For Manuscript Review

Continued from previous page...

Atom-1	Atom-2	Interatomic distance (Å)	Clash overlap (Å)
18:h:44:ILE:HG23	18:h:47:MET:HE2	1.73	0.70
19:I:2292:U:H2’	19:I:2293:G:C8	2.27	0.70
21:J:86:G:H3’	21:J:88:C:H41	1.57	0.70
32:o:18:GLN:HG2	32:o:63:LEU:HD13	1.73	0.69
1:C:249:G:H4’	50:y:19:LYS:HD3	1.75	0.69
1:C:255:U:OP2	4:2:74:ARG:NH1	2.24	0.69
1:C:719:G:OP1	1:C:847:G:N2	2.22	0.69
48:x:7:ALA:HB1	48:x:28:ARG:HG3	1.75	0.69
19:I:883:G:O6	19:I:894:U:O2	2.11	0.69
19:I:2700:A:H61	19:I:2706:A:H61	1.41	0.69
19:I:2848:G:O2’	19:I:2867:G:N2	2.26	0.69
1:C:538:G:OP1	13:F:56:ARG:NH2	2.26	0.69
19:I:1433:A:H2’	19:I:1434:A:C8	2.26	0.69
1:C:329:C:H2’	1:C:330:A:H8	1.58	0.69
13:F:90:LEU:O	13:F:94:LEU:HD12	1.93	0.69
19:I:411:G:OP2	19:I:2406:A:O2’	2.10	0.69
19:I:581:C:H2’	19:I:582:A:H8	1.57	0.69
19:I:932:U:O2’	19:I:934:U:O4	2.11	0.69
19:I:2134:A:H1’	19:I:2159:G:H1’	1.74	0.69
48:x:47:GLU:HG3	48:x:48:GLU:N	2.08	0.69
1:C:291:G:N2	1:C:294:A:OP2	2.23	0.69
1:C:1320:U:H2’	1:C:1321:G:H8	1.58	0.69
1:C:269:G:OP2	50:y:16:LYS:NZ	2.25	0.68
1:C:1065:C:H2’	1:C:1066:G:H8	1.57	0.68
1:C:1169:G:H2’	1:C:1170:A:H8	1.58	0.68
19:I:1248:G:OP1	27:M:44:ARG:NH1	2.24	0.68
19:I:2312:U:O2’	29:N:37:ASN:OD1	2.12	0.68
19:I:2291:U:H2’	19:I:2292:U:C6	2.28	0.68
19:I:1592:C:H2’	19:I:1593:A:C8	2.28	0.68
42:u:11:ASP:HB2	42:u:46:SER:HB3	1.75	0.68
19:I:1789:A:OP2	23:K:221:ARG:NH1	2.27	0.68
21:J:44:G:OP2	21:J:45:A:N6	2.26	0.68
19:I:2898:U:H2’	19:I:2899:A:C8	2.29	0.68
22:j:12:VAL:HG13	22:j:50:LYS:HB3	1.75	0.68
19:I:882:G:N3	19:I:896:A:N6	2.41	0.68
34:p:30:ILE:N	34:p:33:ARG:O	2.26	0.68
9:D:72:THR:O	9:D:74:ARG:NH1	2.27	0.68
19:I:2292:U:H2’	19:I:2293:G:H8	1.59	0.68
19:I:1443:U:H2’	19:I:1444:G:H8	1.59	0.68
30:n:68:ASN:OD1	30:n:130:ASN:ND2	2.27	0.68
31:O:170:ARG:HH22	31:O:172:LYS:HB2	1.57	0.68

Continued on next page...

For Manuscript Review

Continued from previous page...

Atom-1	Atom-2	Interatomic distance (Å)	Clash overlap (Å)
25:L:30:GLU:N	25:L:30:GLU:OE2	2.27	0.67
8:c:43:LYS:HE2	19:I:499:U:H5”	1.75	0.67
1:C:138:G:N2	1:C:173:A:OP2	2.27	0.67
1:C:708:G:H2’	1:C:709:A:C8	2.29	0.67
19:I:1724:G:O6	19:I:1736:U:O4	2.13	0.67
19:I:1730:C:O2	19:I:1731:G:N1	2.28	0.67
19:I:2750:A:O2’	19:I:2753:A:N6	2.26	0.67
1:C:384:U:O2’	48:x:28:ARG:NH2	2.28	0.67
1:C:499:G:H2’	1:C:500:G:H8	1.60	0.67
19:I:874:G:N1	19:I:904:G:O6	2.28	0.67
1:C:526:A:N6	1:C:1200:G:O2’	2.27	0.67
12:e:36:ILE:H	19:I:2354:C:HO2’	1.40	0.67
19:I:2756:U:OP2	28:m:19:ARG:NH1	2.27	0.67
1:C:421:U:O2’	1:C:535:G:OP1	2.12	0.67
1:C:1245:A:N3	1:C:1363:C:O2’	2.28	0.67
1:C:1524:G:N7	6:3:46:LYS:NZ	2.43	0.67
19:I:601:C:H2’	19:I:602:A:O4’	1.94	0.67
19:I:1063:G:O6	19:I:1077:A:N6	2.29	0.66
19:I:2522:U:O2’	19:I:2647:U:OP1	2.11	0.66
39:S:104:THR:HG22	39:S:106:GLU:H	1.60	0.66
1:C:140:G:N2	1:C:172:C:OP1	2.29	0.66
26:l:32:ILE:O	26:l:36:LYS:NZ	2.26	0.66
38:r:93:ARG:NH2	38:r:112:ASP:OD1	2.28	0.66
19:I:993:G:OP1	51:Y:50:ARG:NH1	2.28	0.66
19:I:2122:U:H2’	19:I:2123:G:H8	1.60	0.66
6:3:17:ARG:O	6:3:21:ARG:HG2	1.96	0.66
19:I:2648:G:H2’	19:I:2649:C:C6	2.30	0.66
1:C:60:A:N1	1:C:101:G:O2’	2.29	0.66
1:C:1054:U:OP1	44:v:85:ARG:NH2	2.29	0.66
1:C:1281:A:H2’	1:C:1282:A:C8	2.31	0.66
47:W:10:ARG:NH1	47:W:96:GLY:O	2.28	0.66
43:U:50:ARG:HG3	43:U:65:ILE:HD11	1.76	0.66
48:x:9:HIS:O	48:x:16:PHE:N	2.28	0.66
30:n:107:ALA:HB1	30:n:133:THR:HB	1.78	0.66
1:C:84:C:H2’	1:C:85:U:C6	2.31	0.66
19:I:994:C:OP1	51:Y:53:ARG:NH2	2.28	0.66
1:C:21:G:H2’	1:C:22:G:C8	2.31	0.66
13:F:69:GLU:N	13:F:69:GLU:OE2	2.24	0.66
21:J:50:A:O5’	47:W:63:LYS:NZ	2.29	0.66
1:C:495:C:H2’	1:C:496:A:H8	1.61	0.65
19:I:2641:G:H5”	37:R:78:THR:HB	1.78	0.65

Continued on next page...

For Manuscript Review

Continued from previous page...

Atom-1	Atom-2	Interatomic distance (Å)	Clash overlap (Å)
1:C:138:G:H22	1:C:172:C:H3'	1.60	0.65
9:D:117:LEU:HD22	9:D:141:LEU:HD21	1.78	0.65
40:t:79:VAL:HG22	40:t:102:LEU:HD23	1.77	0.65
1:C:820:C:O2	32:o:16:ASN:ND2	2.29	0.65
19:I:568:U:H1'	19:I:2030:6MZ:H9C1	1.77	0.65
19:I:2127:G:H21	19:I:2173:A:H1'	1.61	0.65
14:f:10:LYS:NZ	19:I:396:G:OP2	2.28	0.65
19:I:2645:G:OP2	19:I:2645:G:N2	2.21	0.65
52:z:37:GLY:O	52:z:63:ARG:NH2	2.29	0.65
1:C:612:C:N4	1:C:615:A:OP2	2.28	0.65
21:J:5:U:OP1	21:J:61:G:O2'	2.11	0.65
48:x:6:LEU:HD22	48:x:17:TYR:HB3	1.78	0.65
8:c:48:PRO:HG3	8:c:56:GLY:H	1.61	0.65
13:F:104:ARG:O	13:F:165:ARG:NH2	2.30	0.65
1:C:1124:A:C5	1:C:1125:G:H1'	2.32	0.65
2:1:62:VAL:HA	2:1:66:MET:HE2	1.78	0.65
11:E:60:PRO:O	11:E:63:SER:OG	2.15	0.65
19:I:1591:A:H2'	19:I:1592:C:C6	2.31	0.65
17:H:2:ARG:NH1	17:H:68:GLN:OE1	2.28	0.65
1:C:286:G:O2'	1:C:603:A:N6	2.29	0.64
1:C:709:A:H2'	1:C:710:A:C8	2.32	0.64
19:I:598:U:H3	19:I:659:G:H1	1.44	0.64
1:C:914:U:O2'	1:C:1075:G:O2'	2.14	0.64
1:C:1017:G:H2'	1:C:1018:G:H8	1.61	0.64
1:C:1426:G:H5'	49:X:106:LYS:HG3	1.78	0.64
12:e:43:THR:HG1	19:I:2331:G:HO2'	1.44	0.64
19:I:1070:A:O2'	19:I:1098:A:OP2	2.14	0.64
19:I:1434:A:H2'	19:I:1435:G:H8	1.63	0.64
19:I:2298:A:OP1	29:N:71:ARG:NH1	2.30	0.64
1:C:269:G:P	50:y:16:LYS:HZ3	2.20	0.64
1:C:932:A:N3	1:C:1370:U:O2'	2.27	0.64
1:C:1160:G:H22	1:C:1165:A:H2	1.45	0.64
12:e:43:THR:OG1	19:I:2331:G:O2'	2.15	0.64
19:I:171:U:H2'	19:I:172:A:H8	1.62	0.64
19:I:1038:G:H2'	19:I:1039:A:C8	2.32	0.64
19:I:2566:A:N1	39:S:28:SER:OG	2.30	0.64
19:I:1051:G:H5'	19:I:2752:C:H1'	1.80	0.64
19:I:2405:G:O2'	19:I:2411:A:N6	2.29	0.64
19:I:599:A:H2'	19:I:600:G:C8	2.32	0.64
19:I:1170:C:N4	19:I:1171:G:O6	2.31	0.64
19:I:2006:C:O2'	19:I:2823:A:N3	2.31	0.64

Continued on next page...

For Manuscript Review

Continued from previous page...

Atom-1	Atom-2	Interatomic distance (Å)	Clash overlap (Å)
19:I:2322:A:H61	19:I:2333:A:H61	1.45	0.64
1:C:546:U:H2’	1:C:547:A:H8	1.62	0.64
1:C:1138:G:H21	1:C:1141:C:H41	1.45	0.64
11:E:11:ARG:NH1	11:E:177:THR:O	2.31	0.64
12:e:36:ILE:N	19:I:2354:C:O2’	2.30	0.64
7:b:28:ASN:ND2	7:b:88:LYS:O	2.31	0.64
19:I:47:C:HO2’	19:I:52:A:HO2’	1.45	0.64
19:I:581:C:H2’	19:I:582:A:C8	2.32	0.64
35:Q:41:HIS:HB3	35:Q:44:PHE:HB2	1.80	0.64
19:I:576:U:H2’	19:I:577:G:C8	2.33	0.64
19:I:1432:G:H2’	19:I:1433:A:C8	2.32	0.64
1:C:303:A:H2’	1:C:304:G:H8	1.63	0.64
8:c:10:GLU:HB3	8:c:73:PHE:HB3	1.77	0.64
19:I:2788:C:O2’	19:I:2809:A:N3	2.31	0.64
31:O:94:TYR:OH	31:O:152:ARG:NH1	2.31	0.64
1:C:1371:A:OP1	30:n:92:ARG:NH2	2.32	0.63
3:Z:59:ILE:HG12	3:Z:101:ILE:HG23	1.79	0.63
14:f:17:ASN:OD1	14:f:25:THR:OG1	2.15	0.63
1:C:914:U:HO2’	1:C:1075:G:HO2’	1.45	0.63
1:C:1156:C:H2’	1:C:1157:G:C8	2.33	0.63
19:I:1411:U:H2’	19:I:1412:U:C6	2.33	0.63
19:I:2289:G:O6	19:I:2343:U:O2	2.16	0.63
31:O:118:PRO:HD2	31:O:121:ILE:HB	1.80	0.63
12:e:40:GLN:OE1	12:e:45:PHE:N	2.31	0.63
19:I:373:U:O2’	19:I:423:A:N3	2.30	0.63
40:t:35:THR:HG22	40:t:36:ARG:HG3	1.79	0.63
11:E:36:ASP:OD1	11:E:59:ARG:NH1	2.31	0.63
9:D:186:ILE:HD11	9:D:204:ASP:HB3	1.81	0.63
29:N:35:THR:HG23	29:N:155:THR:HG23	1.81	0.63
30:n:116:MET:HA	30:n:119:ARG:HB2	1.79	0.63
1:C:829:G:OP1	52:z:53:ARG:NH2	2.30	0.63
13:F:26:ARG:HH12	13:F:164:GLN:HE22	1.46	0.63
21:J:75:G:H2’	21:J:76:G:C8	2.33	0.63
25:L:35:THR:OG1	25:L:49:GLN:O	2.16	0.63
19:I:639:U:H2’	19:I:640:C:C6	2.33	0.63
21:J:63:C:H2’	21:J:64:G:C8	2.34	0.63
23:K:33:LEU:HD21	23:K:102:ARG:HA	1.80	0.63
30:n:101:MET:HA	30:n:104:ILE:HD12	1.80	0.63
1:C:1354:A:C8	44:v:58:SER:HB2	2.34	0.62
27:M:48:THR:HG23	27:M:50:ALA:H	1.64	0.62
31:O:85:LYS:HB2	31:O:133:LEU:HB2	1.81	0.62

Continued on next page...

For Manuscript Review

Continued from previous page...

Atom-1	Atom-2	Interatomic distance (Å)	Clash overlap (Å)
31:O:91:GLY:HA2	31:O:160:LYS:HG2	1.79	0.62
1:C:1116:U:O4	1:C:1117:A:N6	2.32	0.62
10:d:14:LYS:NZ	21:J:80:U:O4	2.24	0.62
19:I:856:G:H2'	19:I:857:G:C8	2.34	0.62
19:I:1068:G:O2'	19:I:1096:A:N3	2.32	0.62
19:I:2626:C:H2'	19:I:2627:G:C8	2.34	0.62
29:N:130:MET:HG3	29:N:131:GLY:H	1.63	0.62
34:p:115:LYS:NZ	34:p:121:ALA:O	2.30	0.62
19:I:1962:5MC:O2'	19:I:1964:G:OP2	2.17	0.62
21:J:83:G:H2'	21:J:84:G:C8	2.34	0.62
31:O:140:VAL:HA	31:O:143:GLN:HB2	1.80	0.62
1:C:191:A:O2'	1:C:214:G:N2	2.31	0.62
13:F:19:LEU:HG	13:F:64:ILE:HG12	1.80	0.62
23:K:107:PRO:HD2	23:K:110:LEU:HD22	1.80	0.62
1:C:171:G:O6	1:C:1442:C:O2'	2.15	0.62
1:C:384:U:H2'	1:C:385:C:C6	2.34	0.62
1:C:1441:A:O2'	1:C:1442:C:OP1	2.13	0.62
3:Z:81:LYS:NZ	19:I:568:U:O4	2.32	0.62
19:I:1130:U:N3	19:I:2025:C:OP1	2.26	0.62
33:P:3:VAL:HG12	33:P:38:PRO:HA	1.80	0.62
41:T:112:LEU:HD12	41:T:130:GLY:HA3	1.82	0.62
9:D:93:ASN:O	9:D:93:ASN:ND2	2.27	0.62
47:W:8:ILE:H	47:W:8:ILE:HD12	1.65	0.62
19:I:2478:A:H5'	19:I:2479:U:H5	1.64	0.62
19:I:885:C:H1'	19:I:892:A:H1'	1.80	0.62
19:I:1007:C:OP1	37:R:37:ARG:NH1	2.32	0.62
19:I:1592:C:H2'	19:I:1593:A:H8	1.64	0.62
1:C:940:A:O2'	1:C:1327:A:N3	2.32	0.62
19:I:704:G:O2'	19:I:726:G:N2	2.30	0.62
1:C:593:U:H2'	1:C:594:G:H8	1.65	0.61
1:C:1017:G:H2'	1:C:1018:G:C8	2.35	0.61
5:4:82:MET:HG3	5:4:98:LYS:HB2	1.82	0.61
19:I:841:G:H2'	19:I:842:U:C6	2.35	0.61
21:J:40:U:N3	21:J:44:G:OP2	2.33	0.61
47:W:67:ASN:H	47:W:70:ALA:HB3	1.65	0.61
1:C:327:U:OP1	4:2:3:ASN:ND2	2.26	0.61
1:C:683:C:OP1	38:r:29:ASN:ND2	2.28	0.61
10:d:9:ARG:HD3	10:d:39:ALA:HB1	1.82	0.61
18:h:45:ARG:HA	18:h:48:ILE:HD12	1.80	0.61
19:I:569:U:O2'	19:I:983:A:N1	2.33	0.61
19:I:1447:C:O2'	19:I:1544:A:N3	2.33	0.61

Continued on next page...

For Manuscript Review

Continued from previous page...

Atom-1	Atom-2	Interatomic distance (Å)	Clash overlap (Å)
21:J:49:C:OP1	47:W:68:LYS:N	2.30	0.61
1:C:383:A:H3'	1:C:384:U:H6	1.65	0.61
19:I:1026:G:H2'	19:I:1027:A:H8	1.66	0.61
1:C:28:A:O2'	1:C:290:U:OP1	2.17	0.61
1:C:1218:U:O2'	1:C:1316:C:OP1	2.13	0.61
10:d:76:ASP:OD1	10:d:76:ASP:N	2.32	0.61
13:F:41:HIS:HA	13:F:44:ARG:HH21	1.64	0.61
19:I:1022:G:N2	19:I:1023:U:O4	2.32	0.61
19:I:1332:G:N7	19:I:1609:A:O2'	2.32	0.61
19:I:2700:A:H61	19:I:2706:A:N6	1.98	0.61
1:C:905:U:OP2	40:t:94:ARG:NH2	2.33	0.61
9:D:76:ALA:HB1	9:D:79:ALA:HB3	1.82	0.61
16:g:18:LEU:HB2	16:g:53:VAL:HG11	1.81	0.61
19:I:605:G:O2'	19:I:657:U:O2'	2.04	0.61
19:I:2139:U:O4	19:I:2152:G:O6	2.18	0.61
21:J:39:A:H1'	21:J:46:A:H61	1.66	0.61
35:Q:14:ALA:HB1	35:Q:34:LEU:HD23	1.82	0.61
19:I:131:A:H2'	19:I:132:G:C8	2.35	0.61
19:I:674:G:H1'	27:M:69:ARG:HD3	1.83	0.61
19:I:882:G:H1'	19:I:896:A:H61	1.64	0.61
34:p:81:HIS:NE2	34:p:104:VAL:O	2.33	0.61
35:Q:11:GLU:HA	35:Q:25:ARG:HA	1.82	0.61
1:C:196:G:H2'	1:C:197:G:C8	2.36	0.61
1:C:1113:C:H42	1:C:1149:A:H61	1.47	0.61
32:o:104:VAL:HG12	32:o:125:ILE:HG22	1.82	0.61
34:p:48:VAL:HG13	34:p:79:ILE:HG21	1.83	0.61
39:S:121:GLU:OE2	49:X:65:SER:OG	2.18	0.61
8:c:28:VAL:HG22	8:c:34:VAL:HG12	1.83	0.61
13:F:196:ASN:HB2	13:F:199:LEU:HD23	1.83	0.61
19:I:463:G:N2	19:I:466:A:OP2	2.29	0.61
19:I:2515:C:H2'	19:I:2516:A:H8	1.66	0.61
43:U:53:MET:HE3	43:U:117:PHE:HE1	1.64	0.61
1:C:1403:C:H2'	1:C:1404:A:H8	1.64	0.61
19:I:715:A:OP1	46:w:60:VAL:HG13	2.01	0.61
19:I:1496:A:H2'	19:I:1498:C:C5	2.35	0.61
21:J:52:A:N1	47:W:33:ARG:NH2	2.48	0.61
1:C:961:C:OP2	1:C:962:A:O2'	2.15	0.60
19:I:561:G:HO2'	51:Y:45:TYR:HH	1.45	0.60
19:I:1433:A:H2'	19:I:1434:A:H8	1.64	0.60
19:I:2144:G:N2	19:I:2146:C:OP2	2.34	0.60
27:M:91:ASP:OD2	27:M:93:SER:OG	2.17	0.60

Continued on next page...

For Manuscript Review

Continued from previous page...

Atom-1	Atom-2	Interatomic distance (Å)	Clash overlap (Å)
1:C:916:G:H2'	1:C:917:A:C8	2.37	0.60
45:V:86:ARG:NH2	45:V:117:ASP:O	2.32	0.60
15:G:25:VAL:HG13	15:G:27:GLY:H	1.64	0.60
16:g:47:ARG:NH2	19:I:74:A:OP1	2.32	0.60
19:I:833:A:H2'	19:I:834:G:C8	2.36	0.60
19:I:874:G:H2'	19:I:875:G:C8	2.36	0.60
19:I:2747:G:H21	19:I:2757:A:H62	0.68	0.60
31:O:107:LEU:HD12	31:O:113:VAL:HG11	1.83	0.60
43:U:21:ALA:HB1	43:U:100:LYS:HG2	1.83	0.60
47:W:8:ILE:HA	47:W:11:ALA:HB3	1.82	0.60
1:C:81:U:H1'	1:C:82:U:H5	1.67	0.60
1:C:194:G:H2'	1:C:195:G:H8	1.66	0.60
1:C:263:U:H2'	1:C:264:A:C8	2.36	0.60
1:C:940:A:H2'	1:C:941:G:C8	2.36	0.60
1:C:998:A:OP1	1:C:1019:U:O2'	2.15	0.60
19:I:476:G:N1	19:I:479:A:OP2	2.34	0.60
21:J:31:C:H2'	21:J:32:U:C6	2.36	0.60
1:C:1244:A:H2'	1:C:1245:A:C8	2.37	0.60
19:I:2354:C:H2'	19:I:2355:G:C8	2.35	0.60
29:N:69:LYS:HA	29:N:84:PRO:HA	1.83	0.60
29:N:74:VAL:HG13	29:N:76:GLY:H	1.66	0.60
38:r:88:GLY:O	38:r:93:ARG:NH1	2.34	0.60
1:C:706:A:H2'	1:C:707:G:C8	2.37	0.60
19:I:213:A:H2'	19:I:214:G:C8	2.37	0.60
19:I:1645:G:H5"	19:I:1646:C:H5'	1.84	0.60
23:K:181:MET:HB2	23:K:268:VAL:HG22	1.83	0.60
1:C:397:C:H5'	13:F:132:ILE:HG22	1.84	0.60
1:C:539:C:OP2	13:F:59:GLN:NE2	2.31	0.60
2:1:39:THR:HG22	2:1:70:LYS:HD2	1.84	0.60
19:I:181:A:H1'	19:I:435:C:H5'	1.83	0.60
19:I:2637:U:H5"	25:L:83:ARG:HH12	1.66	0.60
19:I:2741:A:H62	19:I:2763:G:H21	0.71	0.60
5:4:17:VAL:HG11	5:4:103:ILE:HG12	1.83	0.60
19:I:1:G:N2	19:I:2:G:N7	2.50	0.60
23:K:122:ALA:HB3	23:K:130:LEU:HG	1.83	0.60
29:N:115:ARG:NH1	35:Q:48:GLN:OE1	2.35	0.60
1:C:1021:C:H2'	1:C:1022:C:H6	1.67	0.60
11:E:153:VAL:HG22	11:E:198:VAL:HG22	1.82	0.60
12:e:39:ARG:NH1	12:e:40:GLN:O	2.34	0.60
28:m:2:LYS:HG3	28:m:35:GLN:HB3	1.82	0.60
19:I:290:U:O4	19:I:350:G:O6	2.20	0.59

Continued on next page...

For Manuscript Review

Continued from previous page...

Atom-1	Atom-2	Interatomic distance (Å)	Clash overlap (Å)
19:I:674:G:OP2	27:M:49:ARG:NH2	2.35	0.59
19:I:1009:A:N3	19:I:1153:C:O2'	2.35	0.59
19:I:1429:G:H2'	19:I:1430:G:H8	1.66	0.59
19:I:2162:G:N7	19:I:2164:C:N4	2.50	0.59
43:U:15:GLY:O	43:U:16:ARG:NH1	2.35	0.59
1:C:230:A:H2'	1:C:231:G:H8	1.67	0.59
7:b:38:ALA:O	7:b:81:LYS:NZ	2.34	0.59
19:I:1529:G:O6	19:I:1542:U:O4	2.20	0.59
19:I:2857:G:N2	19:I:2860:A:OP2	2.25	0.59
43:U:34:LYS:HE3	43:U:131:VAL:HG11	1.84	0.59
1:C:1349:G:H2'	1:C:1350:A:C8	2.37	0.59
3:Z:68:ARG:NH2	19:I:1224:U:OP2	2.34	0.59
19:I:793:A:OP2	19:I:2071:A:O2'	2.19	0.59
19:I:832:U:H2'	19:I:833:A:H8	1.67	0.59
19:I:2032:G:N2	25:L:151:THR:OG1	2.35	0.59
19:I:2350:C:H3'	19:I:2351:G:H8	1.67	0.59
48:x:67:ILE:HD12	48:x:68:SER:H	1.66	0.59
1:C:86:C:H2'	1:C:87:C:H6	1.67	0.59
1:C:370:G:H1	1:C:381:U:H3	1.51	0.59
7:b:43:ILE:O	7:b:47:VAL:HG23	2.02	0.59
14:f:3:ARG:NH1	19:I:1365:A:OP1	2.33	0.59
19:I:2758:A:H2'	19:I:2759:G:H8	1.67	0.59
4:2:4:ILE:HG22	4:2:5:LYS:HD2	1.83	0.59
29:N:37:ASN:HD22	29:N:88:LYS:HB2	1.68	0.59
41:T:122:VAL:HB	41:T:125:LEU:HD21	1.83	0.59
1:C:263:U:H2'	1:C:264:A:H8	1.67	0.59
19:I:875:G:H2'	19:I:876:C:C6	2.38	0.59
19:I:1386:C:H2'	19:I:1387:A:H8	1.67	0.59
45:V:83:LEU:HD12	45:V:86:ARG:HD3	1.84	0.59
12:e:40:GLN:NE2	12:e:42:GLY:O	2.36	0.59
15:G:148:ASN:N	15:G:148:ASN:OD1	2.35	0.59
19:I:807:U:O2'	19:I:2060:A:N1	2.35	0.59
29:N:51:ASP:OD1	29:N:51:ASP:N	2.33	0.59
1:C:8:A:N6	13:F:202:GLU:O	2.35	0.59
1:C:18:C:OP1	15:G:132:ASN:ND2	2.34	0.59
1:C:1317:G:H2'	1:C:1318:A:C8	2.38	0.59
23:K:30:PHE:HD2	23:K:33:LEU:HB2	1.68	0.59
1:C:702:A:H2'	1:C:703:G:H8	1.68	0.59
1:C:1349:G:H2'	1:C:1350:A:H8	1.64	0.59
19:I:1266:G:O2'	19:I:2012:G:O6	2.19	0.59
19:I:1712:U:OP2	19:I:1713:A:O2'	2.18	0.59

Continued on next page...

For Manuscript Review

Continued from previous page...

Atom-1	Atom-2	Interatomic distance (Å)	Clash overlap (Å)
23:K:155:ALA:HB2	23:K:162:VAL:HG23	1.84	0.59
27:M:154:ASP:HB3	27:M:157:LEU:HB2	1.84	0.59
1:C:197:G:O2'	1:C:460:A:N6	2.36	0.59
1:C:730:C:H2'	1:C:731:A:C8	2.35	0.59
19:I:1026:G:H2'	19:I:1027:A:C8	2.38	0.59
19:I:1386:C:H2'	19:I:1387:A:C8	2.38	0.59
7:b:19:LYS:NZ	19:I:1340:U:OP1	2.35	0.58
19:I:1827:U:OP2	23:K:221:ARG:NE	2.35	0.58
19:I:2528:U:O5'	31:O:172:LYS:NZ	2.33	0.58
19:I:2771:C:O2'	25:L:173:GLN:NE2	2.32	0.58
31:O:107:LEU:HB2	31:O:113:VAL:HG21	1.85	0.58
1:C:1021:C:H2'	1:C:1022:C:C6	2.38	0.58
8:c:36:VAL:HB	8:c:39:ILE:HB	1.85	0.58
48:x:9:HIS:N	48:x:16:PHE:O	2.34	0.58
1:C:73:A:C5	1:C:91:A:H1'	2.37	0.58
13:F:65:TYR:O	13:F:67:VAL:HG12	2.03	0.58
19:I:2136:G:H1	19:I:2155:U:H3	1.52	0.58
27:M:186:VAL:HG13	27:M:188:MET:HE3	1.85	0.58
28:m:30:GLU:OE1	28:m:32:LYS:HB2	2.02	0.58
29:N:57:LEU:HA	29:N:60:ILE:HB	1.85	0.58
1:C:195:G:H1	1:C:210:U:H3	1.51	0.58
1:C:1308:C:H2'	1:C:1309:U:C6	2.38	0.58
4:2:82:GLN:O	4:2:86:LEU:N	2.34	0.58
19:I:1589:U:H2'	19:I:1590:A:C8	2.38	0.58
1:C:1112:U:H2'	1:C:1113:C:H6	1.69	0.58
19:I:2392:A:OP2	26:l:31:HIS:NE2	2.34	0.58
30:n:4:ARG:HG3	30:n:5:ARG:H	1.69	0.58
41:T:110:VAL:HG22	41:T:125:LEU:HD12	1.85	0.58
1:C:1238:C:H2'	1:C:1239:G:C8	2.39	0.58
1:C:1313:A:OP1	2:1:3:ARG:HD2	2.04	0.58
12:e:39:ARG:HH22	12:e:41:ARG:HG2	1.68	0.58
19:I:1638:C:O2	19:I:2698:U:O2'	2.22	0.58
38:r:79:ILE:HB	38:r:105:PHE:HE2	1.68	0.58
41:T:109:LYS:HB2	41:T:126:ARG:HE	1.68	0.58
45:V:53:THR:HA	45:V:56:LYS:HD3	1.86	0.58
1:C:998:A:H2'	1:C:999:A:O4'	2.03	0.58
1:C:1350:A:H2'	1:C:1351:A:C8	2.39	0.58
1:C:1507:A:H2'	1:C:1508:G:C8	2.38	0.58
4:2:84:ASN:OD1	4:2:85:LYS:N	2.37	0.58
19:I:729:G:O2'	19:I:763:G:H4'	2.04	0.58
19:I:1752:C:H2'	19:I:1753:G:C8	2.38	0.58

Continued on next page...

For Manuscript Review

Continued from previous page...

Atom-1	Atom-2	Interatomic distance (Å)	Clash overlap (Å)
48:x:40:ASN:HB3	48:x:43:ALA:HB2	1.85	0.58
48:x:47:GLU:HG3	48:x:48:GLU:H	1.66	0.58
1:C:178:U:H3	1:C:187:A:H61	1.50	0.58
1:C:805:C:O2'	1:C:895:A:N1	2.34	0.58
9:D:168:HIS:ND1	9:D:169:GLU:OE2	2.28	0.58
19:I:842:U:H2'	19:I:843:G:H8	1.68	0.58
19:I:1153:C:OP1	51:Y:92:ARG:NH2	2.37	0.58
1:C:495:C:H2'	1:C:496:A:C8	2.38	0.58
1:C:975:U:OP1	44:v:9:ARG:NH1	2.36	0.58
1:C:1022:C:H2'	1:C:1023:U:O4'	2.04	0.58
1:C:1233:A:H4'	1:C:1234:U:O5'	2.03	0.58
4:2:67:ILE:HG23	4:2:71:LYS:HB3	1.84	0.58
10:d:6:ALA:HB1	10:d:40:ILE:HB	1.86	0.58
13:F:101:VAL:O	13:F:105:MET:HG2	2.04	0.58
17:H:5:GLU:HB2	17:H:90:MET:HB3	1.84	0.58
19:I:4:U:H2'	19:I:5:A:C8	2.39	0.58
19:I:265:A:H62	19:I:428:A:H1'	1.68	0.58
19:I:743:A:O2'	19:I:1659:G:OP1	2.20	0.58
19:I:1434:A:H2'	19:I:1435:G:C8	2.38	0.58
19:I:2471:A:N1	19:I:2478:A:N6	2.51	0.58
19:I:2537:U:H2'	19:I:2538:C:C6	2.39	0.58
21:J:50:A:OP1	47:W:67:ASN:ND2	2.35	0.58
40:t:37:VAL:HG23	40:t:76:GLU:HG2	1.86	0.58
1:C:1073:G:H2'	1:C:1074:A:C8	2.38	0.58
19:I:2591:C:H2'	19:I:2592:G:C8	2.39	0.58
1:C:677:G:H21	38:r:40:ASN:HA	1.68	0.57
13:F:60:LYS:HE3	13:F:195:ILE:HG22	1.86	0.57
21:J:82:U:H2'	21:J:83:G:C8	2.38	0.57
44:v:19:LYS:HE3	44:v:20:TYR:CE1	2.39	0.57
1:C:917:A:O2'	1:C:1393:C:OP2	2.19	0.57
2:1:50:ALA:HB1	2:1:57:HIS:HB3	1.84	0.57
19:I:8:C:H2'	19:I:9:G:H8	1.68	0.57
19:I:1215:G:H1	19:I:1234:U:H3	1.52	0.57
19:I:1415:U:H3	19:I:1587:G:H1	1.52	0.57
19:I:2047:C:H2'	19:I:2048:G:H8	1.70	0.57
19:I:2151:U:H2'	19:I:2152:G:H8	1.68	0.57
39:S:40:LYS:NZ	39:S:89:ASN:OD1	2.37	0.57
1:C:1255:A:OP2	1:C:1268:G:N2	2.37	0.57
5:4:74:ILE:HD12	5:4:105:VAL:HG22	1.85	0.57
11:E:91:VAL:HA	11:E:94:ILE:HG22	1.85	0.57
1:C:997:G:N2	1:C:998:A:O2'	2.37	0.57

Continued on next page...

For Manuscript Review

Continued from previous page...

Atom-1	Atom-2	Interatomic distance (Å)	Clash overlap (Å)
3:Z:51:VAL:HG23	3:Z:52:PRO:HD3	1.85	0.57
11:E:14:ILE:HG13	11:E:15:VAL:HG22	1.86	0.57
13:F:72:PHE:HE1	13:F:94:LEU:HD21	1.69	0.57
19:I:827:U:O2'	19:I:2068:U:N3	2.37	0.57
36:q:22:THR:HG21	36:q:72:ARG:HG2	1.86	0.57
1:C:376:A:H2'	1:C:377:A:C8	2.40	0.57
1:C:1141:C:H2'	1:C:1142:U:O4'	2.05	0.57
1:C:1431:A:H2'	1:C:1432:G:H8	1.69	0.57
19:I:707:G:O6	19:I:724:U:O4	2.22	0.57
19:I:1800:C:OP2	23:K:182:ARG:NH2	2.36	0.57
1:C:884:G:O2'	1:C:900:A:N6	2.38	0.57
6:3:43:THR:O	6:3:47:ARG:HG3	2.04	0.57
19:I:184:C:H2'	19:I:185:G:H8	1.68	0.57
19:I:1097:U:H3'	19:I:1098:A:H8	1.70	0.57
19:I:2134:A:H2'	19:I:2135:A:H8	1.70	0.57
19:I:2151:U:H2'	19:I:2152:G:C8	2.40	0.57
19:I:698:C:O2'	19:I:734:A:N6	2.35	0.57
19:I:1550:C:OP1	19:I:1720:U:O2'	2.17	0.57
19:I:1796:U:H2'	19:I:1797:G:C8	2.39	0.57
21:J:81:G:O6	21:J:95:U:O2	2.21	0.57
50:y:21:ILE:HG12	50:y:48:ASP:HB3	1.86	0.57
1:C:1406:C:H2'	1:C:1407:A:C8	2.40	0.57
19:I:705:A:H4'	23:K:7:LYS:HD2	1.86	0.57
19:I:2233:U:H2'	19:I:2234:G:C8	2.39	0.57
34:p:22:LYS:O	34:p:61:LEU:N	2.38	0.57
37:R:102:GLU:HB3	37:R:119:PHE:HZ	1.70	0.57
1:C:995:G:HO2'	1:C:996:A:H8	1.51	0.57
19:I:2615:U:C2	20:i:4:GLN:HA	2.40	0.57
1:C:209:C:O2'	1:C:210:U:OP1	2.22	0.57
1:C:352:U:H2'	1:C:353:G:H8	1.70	0.57
1:C:480:U:H2'	1:C:481:A:H8	1.69	0.57
4:2:35:VAL:HG21	4:2:54:MET:HE3	1.86	0.57
41:T:100:ILE:HG22	41:T:101:ILE:HG23	1.86	0.57
5:4:92:ARG:NH2	5:4:94:ASP:OD2	2.37	0.56
19:I:2086:U:H2'	19:I:2087:G:C8	2.40	0.56
19:I:2646:C:OP2	19:I:2732:G:O2'	2.23	0.56
21:J:82:U:H2'	21:J:83:G:H8	1.70	0.56
44:v:88:ALA:HB2	44:v:96:LEU:HD23	1.86	0.56
1:C:103:A:N6	1:C:319:A:N1	2.53	0.56
1:C:892:G:N2	1:C:895:A:OP2	2.34	0.56
15:G:16:ILE:HD11	15:G:113:ALA:HB3	1.87	0.56

Continued on next page...

For Manuscript Review

Continued from previous page...

Atom-1	Atom-2	Interatomic distance (Å)	Clash overlap (Å)
44:v:44:ALA:HA	44:v:47:LYS:HE3	1.85	0.56
1:C:556:U:H5”	1:C:557:A:C5	2.40	0.56
3:Z:51:VAL:HG23	3:Z:52:PRO:CD	2.36	0.56
13:F:61:VAL:HG21	13:F:200:ILE:HD11	1.87	0.56
19:I:1443:U:H2’	19:I:1444:G:C8	2.40	0.56
19:I:2152:G:O2’	19:I:2153:C:O4’	2.24	0.56
19:I:2258:C:O2’	19:I:2427:C:OP2	2.20	0.56
19:I:2293:G:H2’	19:I:2294:G:C8	2.41	0.56
23:K:8:PRO:HB3	23:K:14:ARG:HG3	1.87	0.56
24:k:41:ARG:HB3	24:k:41:ARG:NH1	2.20	0.56
33:P:40:THR:HG22	33:P:43:ASN:ND2	2.20	0.56
37:R:60:ASP:OD2	37:R:61:LYS:NZ	2.39	0.56
47:W:49:VAL:HG21	47:W:82:ALA:HA	1.88	0.56
50:y:15:ASP:OD2	50:y:54:GLY:HA2	2.05	0.56
1:C:869:U:O2’	32:o:15:ARG:NH1	2.32	0.56
1:C:1112:U:H2’	1:C:1113:C:C6	2.41	0.56
1:C:1137:G:H2’	1:C:1138:G:C8	2.41	0.56
1:C:1344:A:OP2	34:p:120:LYS:NZ	2.27	0.56
19:I:582:A:H2’	19:I:583:G:H8	1.71	0.56
19:I:710:U:N3	19:I:711:G:N7	2.54	0.56
19:I:2291:U:OP1	19:I:2380:C:O2’	2.23	0.56
35:Q:37:CYS:H	35:Q:40:CYS:HG	1.52	0.56
1:C:790:C:O3’	38:r:127:ARG:NH1	2.38	0.56
1:C:906:C:H2’	1:C:907:A:C8	2.40	0.56
1:C:1216:G:OP1	1:C:1315:U:O2’	2.21	0.56
10:d:42:LEU:HD21	10:d:63:ILE:HD11	1.88	0.56
13:F:172:GLU:HB3	13:F:181:THR:HB	1.87	0.56
19:I:2528:U:O2’	19:I:2530:A:OP1	2.17	0.56
27:M:7:ASP:OD1	27:M:7:ASP:N	2.38	0.56
49:X:89:ARG:HG3	49:X:89:ARG:HH11	1.69	0.56
1:C:194:G:H2’	1:C:195:G:C8	2.41	0.56
1:C:230:A:H2’	1:C:231:G:C8	2.40	0.56
1:C:1398:C:H2’	1:C:1399:G:C8	2.41	0.56
11:E:142:MET:SD	11:E:170:GLU:HG2	2.46	0.56
19:I:574:A:N6	19:I:2034:U:OP1	2.35	0.56
19:I:1746:A:H2’	19:I:1747:U:C6	2.41	0.56
1:C:17:U:H2’	1:C:18:C:C6	2.41	0.56
1:C:329:C:H2’	1:C:330:A:C8	2.39	0.56
1:C:668:G:H2’	1:C:669:A:C8	2.38	0.56
19:I:1266:G:OP1	20:i:16:ARG:NE	2.39	0.56
19:I:2483:C:N3	43:U:123:LYS:NZ	2.50	0.56

Continued on next page...

For Manuscript Review

Continued from previous page...

Atom-1	Atom-2	Interatomic distance (Å)	Clash overlap (Å)
19:I:2642:G:H2’	19:I:2643:G:H8	1.71	0.56
22:j:44:ARG:HH21	26:l:37:ALA:HA	1.69	0.56
1:C:306:C:H2’	1:C:307:A:H8	1.69	0.56
1:C:489:A:O2’	1:C:490:A:OP2	2.21	0.56
1:C:1314:C:H42	2:1:36:ARG:HB2	1.70	0.56
19:I:676:A:HO2’	19:I:2442:C:HO2’	1.54	0.56
19:I:917:A:C2	21:J:80:U:H4’	2.41	0.56
19:I:1607:C:N4	19:I:1622:G:OP2	2.35	0.56
19:I:2168:G:O2’	19:I:2169:A:O4’	2.23	0.56
33:P:30:LEU:HB3	33:P:36:ALA:HB3	1.87	0.56
39:S:20:MET:HE3	39:S:44:LYS:HE3	1.87	0.56
40:t:68:GLY:O	40:t:99:ARG:NH1	2.39	0.56
45:V:29:VAL:O	45:V:78:LYS:NZ	2.35	0.56
1:C:1157:G:H22	1:C:1168:G:H22	1.54	0.56
19:I:586:A:N1	19:I:809:G:O2’	2.37	0.56
19:I:818:G:N1	19:I:1188:U:OP2	2.29	0.56
19:I:1796:U:H2’	19:I:1797:G:H8	1.69	0.56
21:J:44:G:H5”	21:J:45:A:N7	2.20	0.56
21:J:63:C:H2’	21:J:64:G:H8	1.70	0.56
23:K:71:LYS:NZ	23:K:98:ASP:OD2	2.37	0.56
30:n:40:GLU:OE2	34:p:41:ARG:NH1	2.38	0.56
32:o:11:LEU:HD22	32:o:75:ILE:HD11	1.87	0.56
36:q:15:HIS:HA	36:q:18:ILE:HG22	1.88	0.56
1:C:70:A:H2’	1:C:71:U:H5’	1.88	0.56
1:C:533:A:H2’	1:C:534:G:C8	2.41	0.56
1:C:995:G:O2’	1:C:996:A:H8	1.89	0.56
18:h:7:ILE:HG23	18:h:36:VAL:HG23	1.87	0.56
19:I:272:A:H2’	19:I:273:G:C8	2.41	0.56
19:I:911:A:N6	43:U:11:LYS:O	2.38	0.56
51:Y:49:ASP:OD1	51:Y:52:GLN:NE2	2.39	0.56
1:C:1233:A:H62	1:C:1293:A:N6	2.04	0.55
13:F:177:LYS:HE2	13:F:179:GLU:HG2	1.88	0.55
17:H:17:GLN:O	17:H:21:MET:HG3	2.06	0.55
19:I:2246:G:H2’	19:I:2247:A:H8	1.72	0.55
21:J:42:C:OP2	35:Q:2:LYS:NZ	2.29	0.55
35:Q:16:CYS:SG	35:Q:17:SER:N	2.79	0.55
36:q:24:GLU:OE1	36:q:24:GLU:N	2.36	0.55
40:t:24:LEU:HD21	40:t:82:ILE:HG22	1.88	0.55
47:W:37:ALA:HB2	47:W:106:LEU:HD11	1.88	0.55
1:C:188:G:H4’	4:2:60:ARG:HG2	1.88	0.55
1:C:741:A:H2’	1:C:742:U:C6	2.40	0.55

Continued on next page...

For Manuscript Review

Continued from previous page...

Atom-1	Atom-2	Interatomic distance (Å)	Clash overlap (Å)
8:c:5:ILE:HG22	8:c:7:ARG:HH12	1.71	0.55
9:D:196:VAL:HG13	9:D:199:VAL:HG22	1.88	0.55
19:I:500:G:H22	19:I:503:A:H5'	1.70	0.55
1:C:584:U:OP1	32:o:31:LYS:N	2.36	0.55
1:C:1244:A:N3	1:C:1364:G:O2'	2.35	0.55
13:F:102:VAL:HG13	13:F:107:PHE:HB2	1.87	0.55
19:I:2246:G:H2'	19:I:2247:A:C8	2.42	0.55
19:I:2312:U:H4'	29:N:39:GLY:HA3	1.89	0.55
19:I:2649:C:H2'	19:I:2650:U:C6	2.41	0.55
27:M:83:VAL:HB	27:M:86:ALA:HB2	1.89	0.55
28:m:25:VAL:HG22	28:m:35:GLN:HG2	1.88	0.55
39:S:35:VAL:HG11	39:S:104:THR:HB	1.88	0.55
44:v:3:LYS:HG3	44:v:6:MET:HG2	1.88	0.55
1:C:306:C:H2'	1:C:307:A:C8	2.42	0.55
1:C:970:G:OP2	1:C:1352:U:O2'	2.23	0.55
11:E:38:LYS:HB3	11:E:94:ILE:HD11	1.87	0.55
19:I:742:A:H2'	19:I:743:A:H8	1.71	0.55
1:C:192:G:H2'	1:C:193:U:C6	2.42	0.55
1:C:940:A:H2'	1:C:941:G:H8	1.71	0.55
1:C:1341:G:H4'	1:C:1342:U:O5'	2.05	0.55
5:4:16:LYS:NZ	19:I:2012:G:N7	2.52	0.55
13:F:150:LYS:HG2	13:F:151:LYS:HD2	1.89	0.55
17:H:71:ILE:HD12	17:H:72:ASP:N	2.22	0.55
19:I:995:C:H42	37:R:2:LYS:HD3	1.72	0.55
19:I:2377:A:H2'	19:I:2378:A:C8	2.41	0.55
23:K:25:HIS:HB3	23:K:82:GLU:HB3	1.89	0.55
29:N:119:ALA:O	29:N:167:ARG:NH2	2.40	0.55
48:x:36:VAL:HG11	48:x:56:ARG:HG2	1.89	0.55
1:C:317:U:OP1	4:2:25:ARG:NH2	2.30	0.55
19:I:1084:A:N3	19:I:1105:U:O2'	2.36	0.55
39:S:52:VAL:HG21	39:S:58:LEU:HD11	1.89	0.55
1:C:368:A:H5"	1:C:445:A:C2	2.42	0.55
1:C:515:G:OP2	40:t:51:LYS:NZ	2.34	0.55
9:D:120:GLN:O	9:D:125:THR:OG1	2.25	0.55
11:E:130:PHE:O	11:E:134:MET:HG3	2.06	0.55
17:H:9:MET:HB2	17:H:57:ALA:HB1	1.88	0.55
19:I:146:A:H2'	19:I:147:C:C6	2.41	0.55
19:I:1316:U:H2'	19:I:1317:G:H8	1.71	0.55
19:I:1636:U:H2'	19:I:1637:A:C8	2.42	0.55
19:I:1721:G:N2	19:I:1738:G:O2'	2.39	0.55
19:I:2378:A:H2'	19:I:2379:G:H5'	1.88	0.55

Continued on next page...

For Manuscript Review

Continued from previous page...

Atom-1	Atom-2	Interatomic distance (Å)	Clash overlap (Å)
24:k:25:LYS:NZ	24:k:26:ASN:OD1	2.36	0.55
29:N:91:LEU:HD13	29:N:96:MET:HA	1.88	0.55
1:C:74:A:H62	1:C:89:G:H21	1.54	0.55
1:C:499:G:H2'	1:C:500:G:C8	2.39	0.55
2:1:21:LYS:O	2:1:25:SER:OG	2.24	0.55
19:I:355:U:H2'	19:I:356:G:H8	1.71	0.55
19:I:1281:G:H2'	19:I:1282:U:C6	2.42	0.55
19:I:1415:U:O2	19:I:1587:G:N2	2.38	0.55
19:I:1812:U:H2'	19:I:1813:G:H8	1.72	0.55
19:I:2100:G:H1	19:I:2189:U:H3	1.55	0.55
19:I:2575:C:H5'	25:L:149:ASN:HB2	1.88	0.55
19:I:2589:A:H2'	19:I:2590:A:H8	1.71	0.55
38:r:14:LYS:N	38:r:14:LYS:HE2	2.22	0.55
39:S:105:ARG:NH1	39:S:105:ARG:HB2	2.21	0.55
1:C:364:G:H1	1:C:385:C:H42	1.53	0.55
1:C:1367:G:H5"	30:n:36:LYS:HD2	1.89	0.55
4:2:4:ILE:O	4:2:5:LYS:HB2	2.06	0.55
15:G:11:LEU:HD11	15:G:71:MET:HE1	1.89	0.55
19:I:797:G:OP1	27:M:55:SER:OG	2.22	0.55
19:I:1062:G:N7	19:I:1076:C:N4	2.55	0.55
19:I:1315:C:O2'	19:I:1392:A:N3	2.40	0.55
19:I:2153:C:H2'	19:I:2154:A:C8	2.36	0.55
1:C:914:U:H2'	1:C:915:U:C6	2.42	0.55
1:C:990:A:H2'	1:C:991:U:C6	2.42	0.55
13:F:188:ARG:HA	13:F:191:LEU:HD23	1.88	0.55
17:H:25:TYR:O	17:H:29:ILE:HG22	2.06	0.55
19:I:1441:G:H2'	19:I:1442:U:C6	2.41	0.55
19:I:1587:G:H2'	19:I:1588:G:H8	1.71	0.55
19:I:2305:U:O2'	29:N:133:ARG:NH1	2.39	0.55
21:J:52:A:N6	47:W:33:ARG:HH21	2.05	0.55
43:U:47:GLU:OE2	43:U:50:ARG:NH2	2.40	0.55
1:C:234:C:H2'	1:C:235:G:H8	1.72	0.54
19:I:1130:U:C2	19:I:2025:C:H5"	2.42	0.54
19:I:2514:U:H2'	19:I:2515:C:C6	2.41	0.54
23:K:144:VAL:HB	23:K:154:LEU:HB2	1.89	0.54
29:N:103:LEU:HA	29:N:107:ALA:HB3	1.88	0.54
37:R:31:GLU:OE2	37:R:35:ARG:NH1	2.41	0.54
1:C:78:A:H3'	1:C:79:G:H8	1.73	0.54
1:C:1385:U:H2'	1:C:1386:G:C8	2.42	0.54
8:c:47:LYS:HD3	8:c:48:PRO:HD2	1.88	0.54
13:F:60:LYS:O	13:F:64:ILE:HG13	2.06	0.54

Continued on next page...

For Manuscript Review

Continued from previous page...

Atom-1	Atom-2	Interatomic distance (Å)	Clash overlap (Å)
21:J:91:C:O2	21:J:91:C:H2’	2.07	0.54
23:K:132:MET:O	23:K:167:ARG:NH1	2.40	0.54
37:R:41:LYS:NZ	37:R:52:ASP:OD1	2.41	0.54
46:w:67:LEU:HD22	46:w:78:TYR:HE2	1.73	0.54
1:C:331:G:H2’	1:C:332:A:C8	2.42	0.54
1:C:1065:C:H2’	1:C:1066:G:C8	2.42	0.54
1:C:1183:U:H5”	11:E:5:VAL:HG11	1.89	0.54
1:C:1507:A:H2’	1:C:1508:G:H8	1.72	0.54
13:F:85:ASN:HB3	13:F:88:GLU:HB2	1.89	0.54
19:I:192:C:O2	19:I:802:A:O2’	2.23	0.54
19:I:1306:C:H2’	19:I:1307:A:H8	1.71	0.54
19:I:1473:G:H2’	19:I:1474:U:H6	1.72	0.54
19:I:2122:U:OP2	19:I:2169:A:O2’	2.25	0.54
38:r:20:VAL:HG13	38:r:35:THR:HB	1.90	0.54
1:C:725:G:H5’	1:C:760:A:H4’	1.88	0.54
13:F:59:GLN:O	13:F:63:ARG:HG3	2.06	0.54
19:I:2547:A:H2’	19:I:2548:U:C6	2.42	0.54
19:I:2655:G:O2’	19:I:2664:G:O6	2.25	0.54
32:o:7:ILE:HG13	32:o:32:LEU:HD23	1.89	0.54
46:w:57:LEU:HA	46:w:60:VAL:HB	1.89	0.54
1:C:85:U:H2’	1:C:86:C:H6	1.71	0.54
1:C:168:A:N6	1:C:171:G:OP1	2.40	0.54
19:I:1442:U:H2’	19:I:1443:U:C6	2.42	0.54
19:I:1548:A:H2’	19:I:1549:A:C8	2.43	0.54
19:I:2037:A:H2’	19:I:2038:G:C8	2.42	0.54
19:I:652:U:OP1	19:I:654:A:N6	2.41	0.54
19:I:859:G:O2’	19:I:916:G:O6	2.24	0.54
1:C:41:G:H2’	1:C:42:G:C8	2.42	0.54
1:C:85:U:H2’	1:C:86:C:C6	2.43	0.54
1:C:105:G:H1	1:C:324:C:H42	1.56	0.54
1:C:151:C:H2’	1:C:152:G:H8	1.73	0.54
1:C:364:G:H2’	1:C:365:A:C8	2.43	0.54
11:E:85:GLU:OE2	11:E:88:ARG:NH1	2.40	0.54
13:F:56:ARG:HH12	13:F:63:ARG:HH21	1.54	0.54
14:f:28:ARG:NH2	19:I:1365:A:OP1	2.39	0.54
19:I:30:G:O2’	19:I:1214:A:N3	2.33	0.54
19:I:630:G:N2	19:I:633:A:OP2	2.29	0.54
19:I:873:C:N4	19:I:874:G:O6	2.41	0.54
19:I:1423:G:H2’	19:I:1424:G:C8	2.42	0.54
19:I:1818:U:O2’	23:K:153:GLN:O	2.23	0.54
19:I:2065:C:H1’	19:I:2449:H2U:HN3	1.73	0.54

Continued on next page...

For Manuscript Review

Continued from previous page...

Atom-1	Atom-2	Interatomic distance (Å)	Clash overlap (Å)
47:W:8:ILE:O	47:W:12:THR:OG1	2.24	0.54
1:C:41:G:H2'	1:C:42:G:H8	1.73	0.54
1:C:190:A:H3'	1:C:190:A:N3	2.23	0.54
19:I:1819:A:H5"	23:K:160:THR:HG21	1.89	0.54
42:u:87:ARG:HG3	42:u:87:ARG:HH11	1.73	0.54
43:U:75:GLU:HB2	43:U:90:GLU:HG3	1.90	0.54
49:X:100:LEU:HD11	49:X:110:ILE:HD11	1.88	0.54
8:c:83:VAL:HG21	8:c:103:ILE:HD11	1.88	0.54
10:d:44:HIS:NE2	10:d:48:MET:SD	2.80	0.54
19:I:2025:C:H2'	19:I:2026:U:C6	2.43	0.54
19:I:2112:G:OP1	19:I:2114:A:N6	2.36	0.54
50:y:19:LYS:HB3	50:y:47:HIS:HE1	1.73	0.54
19:I:664:G:O2'	19:I:940:G:OP1	2.23	0.54
19:I:1715:G:O2'	19:I:1743:G:O6	2.21	0.54
19:I:1808:A:H3'	19:I:1809:A:C8	2.43	0.54
19:I:2134:A:H4'	19:I:2159:G:H21	1.72	0.54
19:I:2163:A:H3'	19:I:2164:C:H5"	1.88	0.54
19:I:2168:G:O2'	19:I:2169:A:O5'	2.23	0.54
31:O:127:THR:HB	31:O:130:GLU:HB2	1.90	0.54
34:p:29:VAL:HG13	34:p:64:TYR:HA	1.90	0.54
45:V:2:ARG:NH1	45:V:5:LYS:O	2.41	0.54
51:Y:110:VAL:O	51:Y:114:LYS:N	2.38	0.54
1:C:800:C:H2'	1:C:801:A:H8	1.72	0.53
33:P:62:LEU:O	33:P:66:ASN:ND2	2.41	0.53
40:t:30:LYS:O	40:t:82:ILE:HB	2.08	0.53
43:U:32:GLY:HA2	43:U:104:GLU:HA	1.90	0.53
45:V:28:LEU:HD23	45:V:48:VAL:HG21	1.90	0.53
1:C:88:U:H2'	1:C:89:G:O4'	2.08	0.53
1:C:587:G:H2'	1:C:588:C:H6	1.73	0.53
1:C:1185:A:H5"	11:E:4:LYS:HE3	1.88	0.53
19:I:244:A:OP2	26:l:8:ARG:NH2	2.34	0.53
19:I:910:A:H2'	19:I:911:A:C8	2.42	0.53
19:I:1361:G:H2'	19:I:1362:C:C6	2.44	0.53
21:J:83:G:H2'	21:J:84:G:H8	1.71	0.53
33:P:71:LYS:HD3	33:P:71:LYS:C	2.32	0.53
44:v:31:ILE:HG23	44:v:45:VAL:HG21	1.89	0.53
1:C:102:G:H5"	1:C:103:A:C2	2.43	0.53
1:C:1142:U:H4'	34:p:7:TYR:CE2	2.43	0.53
1:C:1212:C:H2'	1:C:1213:A:C8	2.43	0.53
15:G:38:VAL:HG11	15:G:114:VAL:HG22	1.91	0.53
19:I:599:A:H4'	27:M:24:ASN:HD21	1.73	0.53

Continued on next page...

For Manuscript Review

Continued from previous page...

Atom-1	Atom-2	Interatomic distance (Å)	Clash overlap (Å)
19:I:863:A:H2’	19:I:864:G:H8	1.71	0.53
19:I:2136:G:O6	19:I:2155:U:O4	2.26	0.53
19:I:2530:A:H62	31:O:172:LYS:HD2	1.72	0.53
25:L:25:THR:OG1	25:L:191:GLY:O	2.26	0.53
30:n:15:ASP:OD1	30:n:19:GLY:N	2.30	0.53
3:Z:72:VAL:HG13	3:Z:89:HIS:HB3	1.90	0.53
19:I:248:G:O2’	19:I:2432:A:OP1	2.26	0.53
19:I:832:U:H2’	19:I:833:A:C8	2.43	0.53
19:I:2114:A:H62	19:I:2119:A:H62	1.57	0.53
19:I:2134:A:H2’	19:I:2135:A:C8	2.43	0.53
21:J:60:C:H2’	21:J:61:G:C8	2.39	0.53
29:N:30:ARG:N	29:N:159:THR:OG1	2.40	0.53
37:R:68:LYS:HG2	37:R:72:LYS:HG3	1.90	0.53
1:C:19:A:H2’	1:C:20:U:C6	2.44	0.53
10:d:53:LYS:HG2	10:d:55:GLU:HB3	1.90	0.53
19:I:548:G:O2’	19:I:549:G:O4’	2.25	0.53
19:I:577:G:O2’	19:I:1254:A:OP1	2.27	0.53
19:I:814:C:H1’	19:I:1225:G:H21	1.71	0.53
19:I:1563:U:H2’	19:I:1564:C:C6	2.43	0.53
21:J:9:G:O6	21:J:111:U:O2	2.26	0.53
25:L:4:LEU:HD13	25:L:29:VAL:HG11	1.89	0.53
34:p:33:ARG:NH1	34:p:37:GLN:O	2.41	0.53
1:C:573:G:H2’	1:C:574:C:C6	2.44	0.53
1:C:910:U:H2’	1:C:911:G:H8	1.73	0.53
1:C:1051:G:H5’	11:E:155:GLY:HA3	1.90	0.53
1:C:1326:A:H2’	1:C:1327:A:C8	2.44	0.53
1:C:1458:U:H2’	1:C:1459:A:H8	1.74	0.53
13:F:100:ASN:OD1	13:F:111:ARG:NE	2.35	0.53
19:I:2502:G:H5”	19:I:2503:2MA:H5”	1.91	0.53
29:N:118:SER:O	29:N:128:TYR:OH	2.18	0.53
32:o:83:LEU:HG	40:t:4:VAL:HG21	1.88	0.53
1:C:331:G:H2’	1:C:332:A:H8	1.73	0.53
19:I:2023:C:H2’	19:I:2024:G:H8	1.74	0.53
31:O:17:VAL:HG13	31:O:26:ILE:HD11	1.91	0.53
1:C:444:G:N3	1:C:445:A:N6	2.57	0.53
1:C:506:U:O2’	1:C:507:U:OP1	2.27	0.53
5:4:1:MET:HE3	5:4:2:GLU:N	2.23	0.53
9:D:15:HIS:HB2	9:D:40:ILE:HG23	1.91	0.53
9:D:94:HIS:ND1	9:D:146:ASN:O	2.39	0.53
19:I:1422:G:H2’	19:I:1423:G:C8	2.43	0.53
19:I:2291:U:H2’	19:I:2292:U:H6	1.73	0.53

Continued on next page...

For Manuscript Review

Continued from previous page...

Atom-1	Atom-2	Interatomic distance (Å)	Clash overlap (Å)
19:I:2876:G:OP1	49:X:2:SER:N	2.41	0.53
1:C:1051:G:O2'	11:E:188:GLU:OE2	2.21	0.53
1:C:1212:C:H2'	1:C:1213:A:H8	1.73	0.53
1:C:1310:G:N1	1:C:1313:A:OP2	2.41	0.53
1:C:1350:A:H2'	1:C:1351:A:H8	1.72	0.53
1:C:1403:C:H2'	1:C:1404:A:C8	2.44	0.53
7:b:4:GLU:OE1	16:g:23:ARG:NH1	2.42	0.53
13:F:72:PHE:CE1	13:F:94:LEU:HD21	2.44	0.53
19:I:134:G:H2'	19:I:135:U:C6	2.44	0.53
19:I:2688:G:N1	19:I:2720:U:OP2	2.25	0.53
24:k:25:LYS:HG3	24:k:26:ASN:N	2.22	0.53
29:N:103:LEU:HG	29:N:108:VAL:HG23	1.91	0.53
1:C:32:A:H2'	1:C:33:A:C8	2.44	0.53
1:C:83:G:O2'	1:C:84:C:OP2	2.22	0.53
1:C:206:G:C2	1:C:207:A:H1'	2.44	0.53
1:C:806:G:O2'	1:C:807:U:O4'	2.27	0.53
19:I:210:C:OP1	24:k:29:GLN:NE2	2.40	0.53
19:I:414:C:H2'	19:I:415:A:C8	2.44	0.53
19:I:580:U:H2'	19:I:581:C:C6	2.43	0.53
19:I:742:A:H2'	19:I:743:A:C8	2.43	0.53
19:I:788:A:OP1	19:I:791:C:N4	2.35	0.53
19:I:1794:A:H2'	19:I:1795:C:C6	2.44	0.53
19:I:2162:G:H4'	19:I:2171:A:H2'	1.91	0.53
19:I:2742:G:H3'	19:I:2755:C:H41	1.74	0.53
31:O:89:LEU:HG	31:O:162:VAL:HG22	1.89	0.53
33:P:71:LYS:HD3	33:P:71:LYS:O	2.09	0.53
37:R:32:LEU:O	37:R:36:LEU:HG	2.09	0.53
45:V:30:ARG:NH1	45:V:74:GLU:OE1	2.42	0.53
1:C:248:G:OP1	50:y:68:SER:OG	2.26	0.52
1:C:911:G:H2'	1:C:912:A:C8	2.44	0.52
1:C:1370:U:H2'	1:C:1371:A:C8	2.44	0.52
1:C:1471:U:H2'	1:C:1472:U:C6	2.44	0.52
19:I:857:G:N2	19:I:2269:G:O4'	2.42	0.52
19:I:2301:C:H2'	19:I:2302:U:C6	2.43	0.52
27:M:148:ILE:HB	27:M:169:VAL:HG22	1.91	0.52
1:C:120:A:OP1	1:C:599:U:O2'	2.25	0.52
1:C:368:A:H5"	1:C:445:A:H2	1.74	0.52
1:C:495:C:H1'	1:C:543:C:H1'	1.91	0.52
1:C:896:G:H2'	1:C:897:G:H8	1.73	0.52
1:C:1124:A:H5'	34:p:64:TYR:HE2	1.74	0.52
1:C:1157:G:H1	1:C:1168:G:H22	1.57	0.52

Continued on next page...

For Manuscript Review

Continued from previous page...

Atom-1	Atom-2	Interatomic distance (Å)	Clash overlap (Å)
1:C:1348:U:H2'	1:C:1349:G:H8	1.74	0.52
1:C:1408:U:H2'	1:C:1409:G:H8	1.72	0.52
6:3:13:ASP:O	6:3:17:ARG:NH2	2.41	0.52
9:D:113:ARG:HH12	9:D:117:LEU:HD13	1.73	0.52
14:f:49:LEU:HB3	14:f:51:VAL:HG13	1.90	0.52
15:G:15:LEU:HD12	15:G:37:THR:HG22	1.90	0.52
18:h:25:LEU:HD21	19:I:930:G:H1'	1.89	0.52
19:I:52:A:H2	19:I:119:A:H62	1.57	0.52
19:I:729:G:H5'	19:I:730:A:H5"	1.90	0.52
19:I:918:A:O2'	21:J:81:G:O4'	2.19	0.52
19:I:953:G:H2'	19:I:954:G:H8	1.74	0.52
19:I:1079:C:HO2'	19:I:1080:A:H8	1.58	0.52
19:I:2337:G:H2'	19:I:2338:C:C6	2.44	0.52
30:n:6:VAL:O	30:n:8:GLY:N	2.33	0.52
31:O:117:LEU:HD21	31:O:123:ALA:HB2	1.90	0.52
42:u:72:GLU:O	42:u:76:SER:OG	2.27	0.52
1:C:33:A:H2'	1:C:34:C:C6	2.43	0.52
1:C:67:C:H2'	1:C:68:G:C8	2.45	0.52
1:C:270:G:H5'	50:y:17:MET:SD	2.49	0.52
1:C:972:A:C2	1:C:1313:A:C4	2.96	0.52
1:C:1281:A:H2	1:C:1347:G:H1'	1.73	0.52
6:3:39:GLU:HG2	6:3:44:GLU:HG2	1.92	0.52
9:D:13:GLY:HA3	9:D:208:ARG:HH12	1.74	0.52
19:I:64:A:H2'	19:I:65:U:C6	2.44	0.52
1:C:73:A:H5"	1:C:90:G:N2	2.24	0.52
19:I:842:U:H2'	19:I:843:G:C8	2.45	0.52
31:O:166:ASP:OD1	31:O:166:ASP:N	2.42	0.52
46:w:6:GLU:OE1	46:w:6:GLU:N	2.25	0.52
1:C:938:G:N1	1:C:1332:G:OP2	2.38	0.52
4:2:25:ARG:HG3	4:2:66:LEU:HD21	1.92	0.52
19:I:154:U:C2	19:I:155:A:C8	2.98	0.52
19:I:1682:G:H2'	19:I:1683:U:C6	2.45	0.52
19:I:2064:C:H2'	19:I:2065:C:H6	1.74	0.52
19:I:2521:C:O2'	19:I:2564:A:N3	2.40	0.52
19:I:2812:G:H2'	19:I:2813:A:C8	2.45	0.52
30:n:26:PHE:HE2	30:n:120:LEU:HD21	1.75	0.52
31:O:88:GLN:O	31:O:163:ARG:N	2.25	0.52
32:o:10:MET:HG3	32:o:33:LYS:HD3	1.92	0.52
1:C:314:A:H2'	1:C:315:A:C8	2.43	0.52
15:G:107:ALA:HB1	15:G:111:MET:HB2	1.92	0.52
19:I:511:U:H4'	19:I:1235:G:H4'	1.91	0.52

Continued on next page...

For Manuscript Review

Continued from previous page...

Atom-1	Atom-2	Interatomic distance (Å)	Clash overlap (Å)
19:I:1084:A:H1'	19:I:1106:G:H5'	1.92	0.52
19:I:1654:A:O2'	25:L:118:PHE:O	2.22	0.52
19:I:2149:U:H2'	19:I:2150:C:C6	2.44	0.52
19:I:2301:C:H2'	19:I:2302:U:H6	1.75	0.52
19:I:2784:U:H2'	19:I:2785:C:H6	1.74	0.52
19:I:87:U:H3	19:I:94:A:H61	1.55	0.52
19:I:1353:A:H2'	19:I:1354:A:H8	1.75	0.52
19:I:1385:A:O2'	19:I:1396:U:O2	2.25	0.52
19:I:2233:U:H2'	19:I:2234:G:H8	1.73	0.52
19:I:2243:U:H2'	19:I:2244:U:C6	2.44	0.52
19:I:2315:G:H2'	19:I:2316:G:C8	2.45	0.52
21:J:56:G:H21	21:J:59:A:H2	1.48	0.52
30:n:113:ASP:OD1	30:n:114:LYS:N	2.34	0.52
1:C:494:G:H2'	1:C:495:C:C6	2.45	0.52
1:C:1501:A:H2'	1:C:1502:G:C8	2.43	0.52
19:I:631:A:OP2	26:l:23:LYS:NZ	2.43	0.52
19:I:1065:U:O2'	19:I:1066:U:O4'	2.28	0.52
19:I:1124:G:H1'	28:m:23:ILE:HD12	1.91	0.52
21:J:86:G:N2	21:J:91:C:H1'	2.24	0.52
43:U:36:VAL:HB	43:U:127:LYS:HB3	1.92	0.52
1:C:351:G:O2'	1:C:362:U:O2	2.26	0.52
1:C:646:U:O4	1:C:746:G:O2'	2.25	0.52
11:E:23:PHE:CZ	36:q:11:LYS:HG2	2.45	0.52
19:I:704:G:H1'	19:I:727:A:N6	2.25	0.52
19:I:2122:U:H2'	19:I:2123:G:C8	2.44	0.52
19:I:2636:C:O2'	25:L:45:TYR:OH	2.21	0.52
19:I:2701:U:H3'	19:I:2702:G:C5'	2.40	0.52
23:K:120:VAL:HG12	33:P:91:PHE:HB3	1.91	0.52
43:U:46:ILE:HD12	43:U:69:PRO:HG3	1.91	0.52
1:C:40:C:H2'	1:C:41:G:C8	2.45	0.52
19:I:419:U:H2'	19:I:420:C:C6	2.45	0.52
19:I:1424:G:H3'	19:I:1425:G:H8	1.74	0.52
19:I:1737:G:H2'	19:I:1738:G:C4	2.45	0.52
19:I:2339:C:H2'	19:I:2340:A:C8	2.45	0.52
19:I:2377:A:O2'	47:W:117:PHE:O	2.24	0.52
23:K:245:VAL:HG12	23:K:251:GLN:HA	1.92	0.52
50:y:23:VAL:HG21	50:y:61:ILE:HG21	1.92	0.52
1:C:557:A:H2'	1:C:561:G:C8	2.46	0.51
1:C:1235:G:H2'	1:C:1236:G:C8	2.40	0.51
1:C:1322:C:C2	1:C:1323:G:C8	2.98	0.51
1:C:1478:C:O2'	19:I:1960:A:O2'	2.22	0.51

Continued on next page...

For Manuscript Review

Continued from previous page...

Atom-1	Atom-2	Interatomic distance (Å)	Clash overlap (Å)
19:I:106:C:H2'	19:I:107:G:H8	1.74	0.51
19:I:1410:G:H2'	19:I:1411:U:C6	2.45	0.51
19:I:2315:G:H2'	19:I:2316:G:H8	1.75	0.51
26:l:17:THR:HG21	26:l:49:MET:HE1	1.92	0.51
29:N:43:ALA:HB2	29:N:50:LEU:HD11	1.92	0.51
52:z:62:ALA:HB3	52:z:68:LEU:HD12	1.92	0.51
1:C:451:A:H2'	1:C:452:G:H8	1.75	0.51
1:C:512:C:H5"	1:C:513:C:C6	2.45	0.51
2:1:22:ALA:HB1	2:1:26:GLY:HA2	1.92	0.51
3:Z:8:GLY:O	3:Z:10:LYS:NZ	2.43	0.51
10:d:64:VAL:HG22	10:d:69:GLU:HG2	1.92	0.51
13:F:156:LYS:HE2	13:F:156:LYS:HA	1.92	0.51
19:I:863:A:H2'	19:I:864:G:C8	2.45	0.51
19:I:1007:C:H5"	37:R:37:ARG:NH1	2.25	0.51
21:J:16:G:N1	21:J:69:G:H1'	2.25	0.51
27:M:147:LEU:O	27:M:188:MET:HE1	2.10	0.51
1:C:367:A:O2'	1:C:444:G:N2	2.44	0.51
4:2:82:GLN:O	4:2:85:LYS:N	2.41	0.51
14:f:18:ARG:NH1	19:I:201:C:OP1	2.36	0.51
19:I:31:C:O2'	19:I:1238:G:OP1	2.28	0.51
19:I:306:U:H2'	19:I:307:G:O4'	2.10	0.51
19:I:685:A:N1	19:I:787:C:H1'	2.25	0.51
19:I:1710:G:H2'	19:I:1711:A:C8	2.45	0.51
19:I:1936:A:OP2	19:I:1962:5MC:N4	2.40	0.51
19:I:638:G:H2'	19:I:639:U:C6	2.45	0.51
19:I:1667:G:O2'	19:I:1991:U:O4	2.26	0.51
19:I:2589:A:H2'	19:I:2590:A:C8	2.46	0.51
25:L:25:THR:HG21	25:L:193:VAL:HG22	1.92	0.51
44:v:10:GLU:HG3	44:v:63:ARG:HD2	1.91	0.51
1:C:845:U:H2'	1:C:846:A:C8	2.46	0.51
1:C:1030:A:H3'	1:C:1031:C:C6	2.46	0.51
19:I:2812:G:H2'	19:I:2813:A:H8	1.76	0.51
39:S:25:LEU:HD12	39:S:38:ILE:HG22	1.93	0.51
46:w:26:GLU:OE2	46:w:77:ARG:NH2	2.43	0.51
48:x:58:ALA:HA	48:x:61:VAL:HG12	1.92	0.51
1:C:757:G:H2'	1:C:758:C:C6	2.45	0.51
9:D:207:ILE:HA	9:D:210:VAL:HG12	1.93	0.51
19:I:415:A:H2'	19:I:416:U:C6	2.46	0.51
19:I:1316:U:H2'	19:I:1317:G:C8	2.45	0.51
19:I:2304:G:H22	19:I:2312:U:H3	1.57	0.51
23:K:16:VAL:HG22	23:K:206:GLY:HA3	1.92	0.51

Continued on next page...

For Manuscript Review

Continued from previous page...

Atom-1	Atom-2	Interatomic distance (Å)	Clash overlap (Å)
3:Z:4:VAL:HG23	3:Z:39:LEU:HB2	1.92	0.51
9:D:207:ILE:O	9:D:211:THR:HG23	2.10	0.51
10:d:43:ASP:OD1	10:d:43:ASP:N	2.42	0.51
18:h:13:ALA:HA	18:h:16:ARG:HG2	1.93	0.51
19:I:282:A:H2'	19:I:283:G:C8	2.45	0.51
26:l:12:LYS:HD2	41:T:63:LYS:HE3	1.92	0.51
26:l:25:LYS:HB3	26:l:47:LYS:HE3	1.92	0.51
30:n:93:PRO:HA	30:n:96:ARG:HD3	1.93	0.51
34:p:5:GLN:HA	34:p:22:LYS:NZ	2.25	0.51
1:C:392:U:H2'	1:C:393:G:H8	1.76	0.51
15:G:148:ASN:ND2	32:o:73:GLU:OE1	2.44	0.51
19:I:132:G:H2'	19:I:133:U:C6	2.46	0.51
19:I:303:G:H2'	19:I:304:U:C6	2.46	0.51
19:I:1296:G:OP1	19:I:2709:G:O2'	2.19	0.51
19:I:1819:A:H3'	23:K:177:ARG:HG2	1.92	0.51
22:j:10:LYS:O	22:j:53:LYS:N	2.37	0.51
44:v:41:ARG:HD3	44:v:43:ASN:H	1.75	0.51
1:C:806:G:OP2	1:C:896:G:N2	2.43	0.51
19:I:629:G:H1'	19:I:639:U:H1'	1.93	0.51
19:I:1469:A:H2'	19:I:1470:A:H8	1.76	0.51
19:I:1629:U:O4	19:I:1630:A:N6	2.44	0.51
19:I:2703:C:H2'	19:I:2704:C:H6	1.76	0.51
21:J:97:C:H2'	21:J:98:G:O4'	2.11	0.51
33:P:132:PHE:HB2	33:P:140:ALA:HB3	1.93	0.51
34:p:19:VAL:HG22	34:p:65:ILE:HG23	1.92	0.51
1:C:86:C:H2'	1:C:87:C:C6	2.46	0.51
1:C:423:U:H3	1:C:425:A:H62	1.58	0.51
1:C:686:U:O2'	1:C:688:A:N7	2.38	0.51
1:C:806:G:O2'	1:C:807:U:O5'	2.27	0.51
1:C:855:G:O2'	1:C:868:G:O2'	2.27	0.51
1:C:1268:G:O2'	1:C:1269:A:OP1	2.28	0.51
11:E:131:ARG:HH11	11:E:131:ARG:HG3	1.76	0.51
19:I:191:A:H2'	19:I:192:C:H6	1.76	0.51
19:I:629:G:N3	19:I:639:U:O2'	2.44	0.51
19:I:1672:A:C2	19:I:2582:G:H5'	2.46	0.51
19:I:2081:U:H2'	19:I:2082:A:H8	1.75	0.51
19:I:2229:U:H2'	19:I:2230:G:H8	1.76	0.51
19:I:2308:G:O2'	19:I:2310:C:OP2	2.29	0.51
29:N:105:THR:HA	35:Q:38:SER:HB3	1.92	0.51
34:p:84:THR:HG23	34:p:98:LEU:HD13	1.93	0.51
37:R:62:VAL:HG11	37:R:101:ILE:HD11	1.93	0.51

Continued on next page...

For Manuscript Review

Continued from previous page...

Atom-1	Atom-2	Interatomic distance (Å)	Clash overlap (Å)
42:u:57:ARG:C	42:u:59:GLU:H	2.19	0.51
1:C:427:G:H2'	1:C:428:C:C6	2.47	0.50
19:I:374:A:C8	19:I:400:G:N2	2.72	0.50
19:I:995:C:N4	37:R:2:LYS:HD3	2.26	0.50
19:I:2014:A:H2'	19:I:2015:A:C8	2.46	0.50
23:K:3:VAL:HG22	23:K:19:VAL:HG22	1.93	0.50
29:N:26:MET:HE2	29:N:26:MET:O	2.11	0.50
38:r:111:THR:OG1	38:r:112:ASP:N	2.44	0.50
46:w:17:ARG:N	46:w:21:ASP:OD1	2.44	0.50
1:C:108:U:H2'	1:C:109:G:C8	2.46	0.50
1:C:641:U:H2'	1:C:642:G:H8	1.76	0.50
1:C:763:G:H4'	1:C:1507:A:H4'	1.93	0.50
1:C:916:G:H2'	1:C:917:A:H8	1.73	0.50
1:C:1144:U:C2	1:C:1145:A:C8	3.00	0.50
19:I:643:A:H3'	19:I:644:A:C8	2.46	0.50
19:I:987:C:O2'	19:I:1000:A:N3	2.36	0.50
19:I:2126:A:N6	19:I:2163:A:O4'	2.39	0.50
32:o:47:GLU:HG2	32:o:64:LYS:HB3	1.93	0.50
36:q:19:ASP:OD1	36:q:20:GLN:N	2.43	0.50
49:X:27:GLU:HG3	49:X:44:GLU:HB2	1.94	0.50
50:y:31:HIS:N	50:y:36:LYS:O	2.35	0.50
1:C:391:A:N7	1:C:541:A:O2'	2.41	0.50
1:C:587:G:H2'	1:C:588:C:C6	2.46	0.50
1:C:1113:C:H42	1:C:1149:A:N6	2.09	0.50
2:1:32:ARG:HH21	2:1:34:TRP:HH2	1.58	0.50
4:2:21:ASN:HD22	4:2:66:LEU:HD12	1.77	0.50
8:c:88:GLU:HG2	8:c:93:VAL:HG11	1.93	0.50
13:F:101:VAL:HG13	13:F:105:MET:HE3	1.93	0.50
19:I:374:A:H4'	19:I:422:A:C2	2.46	0.50
19:I:2071:A:H2'	19:I:2072:C:C6	2.46	0.50
19:I:2193:G:H2'	19:I:2194:U:C6	2.45	0.50
19:I:2398:U:N3	19:I:2399:G:N7	2.59	0.50
19:I:2845:U:H5"	49:X:52:ASN:O	2.11	0.50
30:n:42:ILE:HD13	30:n:116:MET:HB3	1.93	0.50
1:C:249:G:H2'	1:C:250:U:C6	2.47	0.50
1:C:574:C:H2'	1:C:575:G:O4'	2.12	0.50
1:C:1244:A:H2'	1:C:1245:A:H8	1.74	0.50
15:G:13:GLU:OE1	15:G:68:ARG:NH1	2.42	0.50
19:I:152:A:H2'	19:I:153:U:H6	1.76	0.50
19:I:589:U:H2'	19:I:590:A:H8	1.77	0.50
19:I:600:G:H22	19:I:605:G:H4'	1.76	0.50

Continued on next page...

For Manuscript Review

Continued from previous page...

Atom-1	Atom-2	Interatomic distance (Å)	Clash overlap (Å)
19:I:1604:C:O2'	19:I:1610:A:N1	2.35	0.50
19:I:2591:C:H2'	19:I:2592:G:H8	1.76	0.50
21:J:115:A:H2'	21:J:116:G:C8	2.45	0.50
1:C:198:G:N3	1:C:199:A:H1'	2.27	0.50
1:C:395:C:O2'	1:C:615:A:N3	2.43	0.50
1:C:1304:C:OP2	42:u:87:ARG:NH2	2.45	0.50
3:Z:37:GLU:OE1	3:Z:37:GLU:N	2.42	0.50
6:3:5:LYS:HD2	38:r:109:ASN:HB3	1.91	0.50
9:D:27:MET:O	9:D:31:ILE:HG13	2.11	0.50
17:H:98:GLU:H	17:H:98:GLU:CD	2.18	0.50
19:I:64:A:H2'	19:I:65:U:H6	1.76	0.50
19:I:1026:G:OP2	19:I:1134:A:O2'	2.23	0.50
19:I:1052:C:H2'	19:I:1053:C:C6	2.45	0.50
35:Q:64:PHE:CG	35:Q:64:PHE:O	2.64	0.50
1:C:512:C:H4'	1:C:513:C:O5'	2.12	0.50
1:C:668:G:H21	38:r:118:HIS:HB2	1.75	0.50
1:C:1433:C:OP1	4:2:33:LYS:HD3	2.11	0.50
5:4:20:VAL:HG11	5:4:44:ALA:HA	1.93	0.50
19:I:1734:G:H2'	19:I:1735:A:H8	1.77	0.50
19:I:1798:U:O2'	19:I:1802:A:N3	2.42	0.50
1:C:1238:C:H2'	1:C:1239:G:H8	1.75	0.50
19:I:305:C:H2'	19:I:306:U:C6	2.46	0.50
19:I:589:U:H2'	19:I:590:A:C8	2.47	0.50
19:I:938:G:H2'	19:I:939:G:C8	2.39	0.50
19:I:1857:G:N2	19:I:1884:G:N3	2.60	0.50
19:I:2731:G:H2'	19:I:2732:G:C8	2.47	0.50
39:S:5:GLN:HA	39:S:20:MET:SD	2.52	0.50
1:C:418:G:H2'	1:C:419:A:H8	1.77	0.50
1:C:912:A:H2'	1:C:913:A:C8	2.47	0.50
1:C:1344:A:O2'	30:n:33:ASP:OD2	2.29	0.50
6:3:2:PRO:HG2	38:r:112:ASP:HB3	1.93	0.50
10:d:26:PHE:CE1	10:d:42:LEU:HB2	2.47	0.50
10:d:90:ASP:OD1	10:d:91:PHE:N	2.45	0.50
19:I:290:U:O2	19:I:350:G:N2	2.34	0.50
19:I:514:A:N3	19:I:581:C:O2'	2.37	0.50
19:I:532:A:H4'	19:I:533:G:C8	2.47	0.50
19:I:745:1MG:O2'	19:I:748:G:H1'	2.12	0.50
19:I:814:C:H1'	19:I:1225:G:N2	2.27	0.50
19:I:926:G:H2'	19:I:927:A:C8	2.47	0.50
19:I:1450:G:H2'	19:I:1451:C:C6	2.47	0.50
19:I:2215:C:H2'	19:I:2216:G:H8	1.77	0.50

Continued on next page...

For Manuscript Review

Continued from previous page...

Atom-1	Atom-2	Interatomic distance (Å)	Clash overlap (Å)
22:j:28:ARG:HG3	22:j:29:THR:HG23	1.94	0.50
1:C:549:U:H2'	1:C:550:C:C6	2.46	0.50
12:e:70:GLU:HG2	12:e:79:PHE:HB2	1.93	0.50
19:I:438:G:H2'	19:I:439:A:C8	2.47	0.50
19:I:587:C:OP2	41:T:21:ARG:NH2	2.45	0.50
19:I:1278:C:H2'	19:I:1279:G:H8	1.76	0.50
19:I:2096:C:H2'	19:I:2097:A:H8	1.77	0.50
19:I:2352:A:H3'	19:I:2353:G:H8	1.77	0.50
52:z:56:ALA:O	52:z:60:LYS:HG3	2.12	0.50
1:C:40:C:H2'	1:C:41:G:H8	1.77	0.49
1:C:248:G:O2'	50:y:18:GLU:O	2.30	0.49
1:C:1346:C:H2'	1:C:1347:G:C8	2.47	0.49
13:F:107:PHE:CG	13:F:145:ILE:HD11	2.47	0.49
19:I:302:C:H2'	19:I:303:G:H8	1.77	0.49
19:I:2411:A:H2'	19:I:2412:A:H8	1.76	0.49
44:v:90:ARG:HG3	44:v:92:GLU:HG2	1.93	0.49
1:C:45:G:H4'	1:C:300:A:H2	1.77	0.49
1:C:975:U:H2'	1:C:976:U:C5	2.47	0.49
1:C:1419:U:H2'	1:C:1420:G:H8	1.77	0.49
11:E:24:ALA:HB1	11:E:28:GLU:HG2	1.92	0.49
19:I:347:A:H2'	19:I:348:A:C8	2.47	0.49
19:I:882:G:H2'	19:I:883:G:C8	2.47	0.49
19:I:2128:G:N3	19:I:2173:A:O2'	2.44	0.49
19:I:2241:A:H2'	19:I:2242:G:C8	2.47	0.49
42:u:81:MET:HE3	42:u:92:ARG:HG3	1.93	0.49
5:4:23:LEU:HD11	20:i:22:LEU:HB2	1.94	0.49
19:I:160:A:N3	19:I:2208:C:O2'	2.35	0.49
19:I:676:A:O2'	19:I:2442:C:O2'	2.26	0.49
19:I:1266:G:OP2	20:i:17:ARG:NE	2.36	0.49
19:I:1812:U:H2'	19:I:1813:G:C8	2.47	0.49
31:O:90:VAL:O	31:O:161:GLY:N	2.33	0.49
1:C:65:A:H2'	1:C:375:A:N1	2.27	0.49
1:C:1054:U:H4'	36:q:53:ILE:HG22	1.94	0.49
1:C:1071:G:N2	1:C:1074:A:OP2	2.40	0.49
19:I:177:G:H3'	19:I:178:G:H8	1.78	0.49
19:I:632:A:H2'	19:I:633:A:C8	2.46	0.49
19:I:1353:A:H2'	19:I:1354:A:C8	2.47	0.49
19:I:1853:A:N3	19:I:2233:U:O2'	2.42	0.49
19:I:2266:A:H4'	19:I:2267:A:N3	2.27	0.49
27:M:97:ASN:HB2	27:M:100:MET:HG3	1.94	0.49
38:r:106:ARG:HG2	38:r:107:ILE:N	2.26	0.49

Continued on next page...

For Manuscript Review

Continued from previous page...

Atom-1	Atom-2	Interatomic distance (Å)	Clash overlap (Å)
48:x:46:LYS:HD3	48:x:47:GLU:N	2.27	0.49
51:Y:75:SER:OG	51:Y:76:TYR:N	2.41	0.49
1:C:103:A:H62	1:C:318:G:H21	1.58	0.49
1:C:138:G:N2	1:C:172:C:H3'	2.26	0.49
1:C:1360:U:O2'	36:q:62:ARG:NH1	2.41	0.49
2:1:40:ILE:HA	2:1:44:MET:SD	2.52	0.49
8:c:60:GLU:OE1	8:c:60:GLU:HA	2.12	0.49
17:H:88:MET:HE3	17:H:90:MET:HE2	1.94	0.49
19:I:77:G:H2'	19:I:78:U:C6	2.47	0.49
19:I:828:U:H2'	19:I:829:A:C8	2.48	0.49
19:I:1129:A:N6	19:I:2491:U:OP1	2.46	0.49
19:I:1794:A:H2'	19:I:1795:C:H6	1.78	0.49
19:I:2845:U:H2'	19:I:2846:G:C8	2.47	0.49
25:L:48:ILE:HD11	25:L:82:PHE:HD1	1.78	0.49
36:q:27:GLU:OE2	36:q:28:THR:N	2.46	0.49
1:C:451:A:H2'	1:C:452:G:C8	2.48	0.49
1:C:759:G:N2	1:C:807:U:OP2	2.31	0.49
2:1:10:PHE:CD1	2:1:11:ILE:N	2.80	0.49
8:c:5:ILE:O	8:c:7:ARG:NH2	2.42	0.49
13:F:156:LYS:HE3	13:F:178:MET:HE2	1.94	0.49
19:I:184:C:H2'	19:I:185:G:C8	2.46	0.49
19:I:666:A:H2'	19:I:667:U:C6	2.46	0.49
19:I:813:U:H2'	19:I:814:C:C6	2.47	0.49
19:I:1052:C:H2'	19:I:1053:C:H6	1.77	0.49
19:I:2133:G:H2'	19:I:2157:G:N1	2.27	0.49
27:M:128:ALA:HB3	27:M:133:LEU:HD12	1.95	0.49
1:C:45:G:H2'	1:C:46:G:H8	1.77	0.49
1:C:316:C:H2'	1:C:317:U:C6	2.47	0.49
1:C:1020:G:C6	1:C:1029:A:N1	2.79	0.49
1:C:1518:C:H2'	1:C:1519:G:C8	2.47	0.49
19:I:593:U:H2'	19:I:594:U:C6	2.48	0.49
19:I:1219:U:H2'	19:I:1220:G:H8	1.78	0.49
19:I:1506:U:H2'	19:I:1507:C:C6	2.48	0.49
19:I:2821:A:OP2	25:L:115:GLY:N	2.46	0.49
21:J:80:U:O2	21:J:96:G:O6	2.30	0.49
23:K:222:GLY:HA2	23:K:225:MET:HG3	1.94	0.49
32:o:11:LEU:HB3	32:o:75:ILE:HG12	1.95	0.49
46:w:18:ASP:OD2	46:w:18:ASP:N	2.44	0.49
1:C:45:G:H2'	1:C:46:G:C8	2.47	0.49
1:C:998:A:H3'	1:C:1018:G:H22	1.78	0.49
1:C:1301:U:OP1	42:u:100:GLN:NE2	2.46	0.49

Continued on next page...

For Manuscript Review

Continued from previous page...

Atom-1	Atom-2	Interatomic distance (Å)	Clash overlap (Å)
7:b:30:ILE:HG13	7:b:85:VAL:HB	1.95	0.49
8:c:54:GLN:CB	8:c:55:PRO:HD3	2.42	0.49
19:I:52:A:OP2	19:I:117:G:N1	2.43	0.49
19:I:1124:G:N3	28:m:37:GLN:NE2	2.61	0.49
30:n:86:GLN:HB2	30:n:148:ASN:HD21	1.78	0.49
42:u:19:LEU:HD11	42:u:56:LEU:HD22	1.95	0.49
45:V:8:ARG:HG3	45:V:10:LEU:HG	1.95	0.49
1:C:480:U:H2'	1:C:481:A:C8	2.48	0.49
1:C:1308:C:H2'	1:C:1309:U:H6	1.75	0.49
6:3:5:LYS:O	6:3:18:ARG:NH2	2.43	0.49
13:F:25:VAL:HG23	13:F:26:ARG:H	1.78	0.49
15:G:157:ARG:NH1	32:o:43:GLU:OE2	2.46	0.49
19:I:8:C:H2'	19:I:9:G:C8	2.46	0.49
19:I:162:U:H1'	19:I:163:C:H5	1.78	0.49
19:I:263:G:O2'	19:I:429:A:N3	2.39	0.49
39:S:103:VAL:HB	39:S:107:LEU:HD12	1.93	0.49
40:t:57:LEU:HD11	40:t:63:VAL:HG22	1.94	0.49
1:C:533:A:H2'	1:C:534:G:H8	1.77	0.49
14:f:57:ARG:HH22	19:I:399:U:H5	1.59	0.49
19:I:1423:G:H2'	19:I:1424:G:H8	1.77	0.49
19:I:1510:G:H2'	19:I:1511:G:H8	1.77	0.49
19:I:2008:C:H2'	19:I:2009:A:H8	1.78	0.49
19:I:2064:C:H2'	19:I:2065:C:C6	2.48	0.49
21:J:5:U:H2'	21:J:6:G:H8	1.77	0.49
1:C:709:A:H2'	1:C:710:A:H8	1.75	0.48
1:C:778:A:H2'	1:C:779:G:C8	2.48	0.48
1:C:1157:G:H1	1:C:1168:G:H1	1.61	0.48
1:C:1506:U:H2'	1:C:1507:A:C8	2.48	0.48
3:Z:38:VAL:HG22	3:Z:59:ILE:HG13	1.95	0.48
9:D:139:ARG:HH22	9:D:143:LYS:HB2	1.76	0.48
11:E:7:PRO:HD2	11:E:184:TYR:CD2	2.48	0.48
11:E:79:LYS:O	11:E:82:GLU:HG2	2.13	0.48
15:G:24:THR:HG23	15:G:24:THR:O	2.13	0.48
19:I:78:U:H2'	19:I:79:C:C6	2.48	0.48
19:I:1027:A:H2	19:I:1028:A:H62	1.61	0.48
19:I:1689:A:H2'	19:I:1690:A:C8	2.48	0.48
19:I:2228:G:H2'	19:I:2229:U:C6	2.48	0.48
19:I:2557:G:H2'	19:I:2558:C:C6	2.47	0.48
21:J:52:A:H61	47:W:33:ARG:HH21	1.60	0.48
25:L:85:ALA:N	25:L:88:GLU:OE1	2.41	0.48
32:o:90:ASP:OD1	32:o:90:ASP:N	2.43	0.48

Continued on next page...

For Manuscript Review

Continued from previous page...

Atom-1	Atom-2	Interatomic distance (Å)	Clash overlap (Å)
38:r:20:VAL:HG23	38:r:83:GLU:O	2.12	0.48
1:C:739:U:OP1	1:C:845:U:O2'	2.18	0.48
1:C:1143:C:H3'	1:C:1144:U:H6	1.78	0.48
19:I:936:A:H2'	19:I:937:C:C6	2.48	0.48
19:I:1497:U:O2'	19:I:1577:C:H5"	2.13	0.48
19:I:2847:U:H2'	19:I:2848:G:O4'	2.12	0.48
29:N:39:GLY:HA2	29:N:86:GLY:HA3	1.95	0.48
29:N:135:GLN:HE22	29:N:152:LEU:HD21	1.79	0.48
34:p:114:LYS:NZ	34:p:118:LEU:O	2.46	0.48
35:Q:16:CYS:HB3	35:Q:20:ASN:HB2	1.95	0.48
37:R:14:ASP:OD1	37:R:14:ASP:N	2.41	0.48
42:u:66:GLU:OE1	42:u:70:ARG:NH2	2.46	0.48
1:C:513:C:N4	1:C:523:G:O2'	2.40	0.48
1:C:666:U:H2'	1:C:667:G:H8	1.78	0.48
1:C:677:G:N2	38:r:40:ASN:HA	2.28	0.48
10:d:6:ALA:HB3	10:d:65:VAL:HG12	1.94	0.48
14:f:2:SER:O	14:f:2:SER:OG	2.20	0.48
19:I:2332:C:O2'	19:I:2335:A:H1'	2.13	0.48
19:I:2567:G:H2'	19:I:2568:U:C6	2.49	0.48
21:J:5:U:H2'	21:J:6:G:C8	2.48	0.48
30:n:37:SER:OG	34:p:41:ARG:NE	2.46	0.48
1:C:633:A:H2'	1:C:634:A:H8	1.77	0.48
1:C:836:U:O2'	1:C:837:U:O2	2.21	0.48
1:C:1020:G:N1	1:C:1029:A:C2	2.72	0.48
1:C:1296:C:C5	42:u:17:ILE:HG13	2.48	0.48
1:C:1457:U:H2'	1:C:1458:U:C6	2.49	0.48
9:D:187:VAL:HG13	9:D:191:SER:HB3	1.94	0.48
17:H:2:ARG:HE	17:H:91:ARG:HH11	1.60	0.48
19:I:171:U:H2'	19:I:172:A:C8	2.45	0.48
19:I:1797:G:O2'	23:K:257:THR:OG1	2.24	0.48
19:I:2051:A:H5'	19:I:2578:G:O4'	2.12	0.48
19:I:2181:U:H2'	19:I:2182:U:C6	2.48	0.48
19:I:2273:A:H2'	19:I:2274:A:C8	2.49	0.48
19:I:2531:A:H61	19:I:2662:A:H61	1.60	0.48
21:J:47:C:H5'	47:W:30:ARG:HH12	1.79	0.48
22:j:5:ILE:HD12	22:j:26:ASN:HD21	1.78	0.48
27:M:98:LYS:O	27:M:102:ARG:HD3	2.14	0.48
41:T:69:ARG:HG2	41:T:69:ARG:HH11	1.77	0.48
43:U:2:LEU:HD23	43:U:2:LEU:H	1.78	0.48
1:C:375:A:H2'	1:C:376:A:O4'	2.13	0.48
1:C:408:A:H1'	1:C:409:A:N3	2.28	0.48

Continued on next page...

For Manuscript Review

Continued from previous page...

Atom-1	Atom-2	Interatomic distance (Å)	Clash overlap (Å)
1:C:1115:U:H3	1:C:1147:G:H1	1.61	0.48
1:C:1470:A:H2'	1:C:1471:U:C6	2.49	0.48
13:F:198:HIS:CE1	13:F:199:LEU:HD22	2.48	0.48
19:I:874:G:H2'	19:I:875:G:H8	1.77	0.48
19:I:1286:A:H1'	19:I:1288:G:OP2	2.14	0.48
19:I:1866:A:N7	19:I:1867:G:O2'	2.42	0.48
19:I:2113:U:O4	19:I:2167:U:O2'	2.31	0.48
19:I:2368:C:H2'	19:I:2369:A:C8	2.49	0.48
19:I:2595:G:N2	19:I:2598:A:OP2	2.31	0.48
43:U:29:GLY:HA3	43:U:106:ASP:OD1	2.14	0.48
1:C:96:G:H2'	1:C:97:C:C6	2.48	0.48
1:C:307:A:H2'	1:C:308:C:C6	2.49	0.48
1:C:656:U:H2'	1:C:657:A:C8	2.48	0.48
1:C:1312:A:H5"	2:1:3:ARG:HH12	1.77	0.48
1:C:1518:C:H2'	1:C:1519:G:H8	1.78	0.48
19:I:675:A:N3	19:I:2443:C:O2'	2.44	0.48
23:K:76:ALA:HB2	23:K:96:TYR:CD2	2.49	0.48
27:M:41:GLN:NE2	27:M:43:THR:OG1	2.44	0.48
29:N:114:PHE:HZ	29:N:176:PRO:HG3	1.79	0.48
51:Y:83:LEU:HB3	51:Y:88:VAL:HG12	1.95	0.48
1:C:652:A:H1'	46:w:22:THR:HG21	1.95	0.48
19:I:712:G:H2'	19:I:713:G:C8	2.48	0.48
19:I:1120:G:H2'	19:I:1121:C:C6	2.48	0.48
19:I:1721:G:H1'	19:I:1739:A:N6	2.29	0.48
19:I:2868:A:H2'	19:I:2869:G:C8	2.49	0.48
32:o:64:LYS:HG3	32:o:71:VAL:HG21	1.95	0.48
40:t:3:THR:HG22	40:t:5:ASN:H	1.78	0.48
1:C:418:G:H2'	1:C:419:A:C8	2.49	0.48
1:C:672:U:H2'	1:C:673:U:H6	1.78	0.48
19:I:1495:A:H2'	19:I:1496:A:C8	2.49	0.48
19:I:1528:A:OP2	19:I:1543:G:N2	2.46	0.48
19:I:1721:G:N2	19:I:1738:G:N3	2.61	0.48
19:I:1802:A:H2'	19:I:1803:A:C8	2.48	0.48
19:I:2538:C:H2'	19:I:2539:C:C6	2.49	0.48
19:I:2615:U:H2'	19:I:2616:C:H6	1.78	0.48
29:N:65:PRO:HA	29:N:89:VAL:HG22	1.94	0.48
31:O:7:ALA:O	31:O:69:ARG:NE	2.47	0.48
47:W:52:SER:OG	47:W:53:THR:N	2.47	0.48
1:C:83:G:C4	1:C:84:C:C5	3.02	0.48
1:C:234:C:H2'	1:C:235:G:C8	2.49	0.48
1:C:387:A:H5'	1:C:477:C:O2'	2.13	0.48

Continued on next page...

For Manuscript Review

Continued from previous page...

Atom-1	Atom-2	Interatomic distance (Å)	Clash overlap (Å)
1:C:687:G:OP1	38:r:127:ARG:NH2	2.47	0.48
1:C:1145:A:O2'	1:C:1146:A:H5'	2.14	0.48
1:C:1473:C:H2'	1:C:1474:A:C8	2.48	0.48
9:D:113:ARG:NH1	9:D:117:LEU:HD13	2.27	0.48
19:I:18:U:H2'	19:I:19:A:C8	2.48	0.48
19:I:1357:C:H2'	19:I:1358:G:O4'	2.13	0.48
19:I:2293:G:H5"	47:W:94:ARG:NH1	2.29	0.48
19:I:2316:G:H2'	19:I:2317:A:H8	1.79	0.48
19:I:2338:C:H2'	19:I:2339:C:C6	2.49	0.48
22:j:34:LEU:HD23	22:j:34:LEU:H	1.79	0.48
48:x:71:VAL:O	48:x:75:ILE:HG13	2.14	0.48
1:C:317:U:H2'	1:C:318:G:O4'	2.13	0.48
1:C:1047:G:N7	1:C:1194:C:H5'	2.29	0.48
9:D:13:GLY:C	9:D:15:HIS:H	2.22	0.48
12:e:66:LYS:O	12:e:82:ILE:HA	2.14	0.48
17:H:4:TYR:HB2	17:H:64:VAL:HG22	1.96	0.48
18:h:24:LEU:HD11	18:h:54:MET:HE1	1.96	0.48
19:I:18:U:H2'	19:I:19:A:H8	1.78	0.48
19:I:414:C:H2'	19:I:415:A:H8	1.78	0.48
19:I:878:A:H3'	19:I:879:G:H8	1.79	0.48
19:I:2074:U:H2'	19:I:2075:U:C6	2.49	0.48
19:I:2262:U:OP1	19:I:2387:U:O2'	2.32	0.48
19:I:2290:G:H2'	19:I:2291:U:C6	2.49	0.48
21:J:16:G:C6	21:J:69:G:H1'	2.48	0.48
39:S:77:ILE:HG12	49:X:72:ARG:HG3	1.95	0.48
50:y:11:ARG:NH2	50:y:56:GLY:O	2.43	0.48
1:C:161:C:H2'	1:C:162:G:H8	1.79	0.47
1:C:632:C:C2	1:C:633:A:C8	3.02	0.47
4:2:72:ALA:O	4:2:76:LYS:HG3	2.13	0.47
15:G:103:THR:O	15:G:122:ASN:ND2	2.32	0.47
19:I:661:A:H1'	41:T:13:LYS:HA	1.95	0.47
19:I:1853:A:H2'	19:I:1854:A:C8	2.48	0.47
19:I:2086:U:H2'	19:I:2087:G:H8	1.77	0.47
19:I:2527:C:H2'	19:I:2528:U:O4'	2.14	0.47
19:I:2679:A:H2'	19:I:2680:U:C6	2.48	0.47
23:K:69:ARG:NH1	23:K:127:GLY:O	2.41	0.47
25:L:97:SER:OG	25:L:98:VAL:N	2.47	0.47
1:C:56:U:H2'	1:C:57:G:H8	1.79	0.47
1:C:552:G:OP2	1:C:553:A:O2'	2.23	0.47
1:C:902:A:H2'	1:C:903:A:H8	1.79	0.47
1:C:1314:C:N4	2:1:36:ARG:HB2	2.29	0.47

Continued on next page...

For Manuscript Review

Continued from previous page...

Atom-1	Atom-2	Interatomic distance (Å)	Clash overlap (Å)
19:I:375:G:H1	19:I:399:U:H3	1.62	0.47
19:I:566:U:H5"	41:T:29:LYS:HE3	1.95	0.47
19:I:658:U:O2	27:M:97:ASN:ND2	2.46	0.47
19:I:1106:G:H2'	19:I:1107:G:C8	2.49	0.47
41:T:101:ILE:HB	41:T:105:ILE:HG13	1.96	0.47
1:C:507:U:H2'	1:C:508:C:C6	2.48	0.47
1:C:1006:A:H2'	1:C:1007:G:C8	2.49	0.47
1:C:1049:A:H1'	11:E:156:ARG:HH21	1.79	0.47
4:2:83:ILE:HD12	4:2:84:ASN:N	2.29	0.47
13:F:154:ARG:N	13:F:154:ARG:HD2	2.28	0.47
19:I:1504:A:H2'	19:I:1505:A:C8	2.50	0.47
19:I:1540:G:H2'	19:I:1541:C:C6	2.49	0.47
19:I:1791:A:H5"	23:K:205:LEU:HD12	1.95	0.47
19:I:1856:U:H2'	19:I:1857:G:O4'	2.14	0.47
19:I:2061:G:H2'	19:I:2501:C:O2'	2.14	0.47
19:I:2651:C:N4	19:I:2652:C:N4	2.62	0.47
22:j:10:LYS:HB3	22:j:53:LYS:HA	1.95	0.47
23:K:30:PHE:CE2	23:K:32:PRO:HG2	2.49	0.47
33:P:145:ASN:OD1	33:P:145:ASN:N	2.47	0.47
1:C:722:A:H2'	1:C:723:A:C8	2.49	0.47
4:2:36:TYR:CZ	4:2:79:LEU:HD21	2.49	0.47
5:4:4:ILE:HG22	5:4:106:VAL:HG13	1.96	0.47
19:I:948:C:H2'	19:I:949:G:H8	1.80	0.47
19:I:1122:G:N2	19:I:1123:C:H41	2.12	0.47
19:I:2007:U:C2	19:I:2008:C:C5	3.02	0.47
19:I:2532:G:O2'	19:I:2663:G:N2	2.47	0.47
26:l:12:LYS:HB3	41:T:63:LYS:HG2	1.95	0.47
32:o:94:LYS:HG2	32:o:117:ARG:HH22	1.79	0.47
35:Q:35:ASP:OD1	35:Q:35:ASP:N	2.42	0.47
1:C:677:G:H2'	1:C:678:U:H6	1.80	0.47
1:C:712:A:H2	52:z:38:LYS:HD3	1.79	0.47
1:C:854:A:H3'	1:C:855:G:H8	1.80	0.47
19:I:65:U:H2'	19:I:66:C:H6	1.80	0.47
19:I:376:G:H2'	19:I:376:G:N3	2.30	0.47
19:I:667:U:H2'	19:I:668:A:O4'	2.15	0.47
19:I:705:A:C2	19:I:727:A:H1'	2.49	0.47
19:I:1224:U:H2'	19:I:1225:G:C8	2.49	0.47
19:I:2441:U:OP2	19:I:2586:U:O2'	2.31	0.47
19:I:2685:G:H2'	19:I:2686:G:H8	1.80	0.47
21:J:42:C:C6	29:N:66:LEU:HD13	2.49	0.47
37:R:36:LEU:HD23	37:R:54:ILE:HD12	1.96	0.47

Continued on next page...

For Manuscript Review

Continued from previous page...

Atom-1	Atom-2	Interatomic distance (Å)	Clash overlap (Å)
48:x:76:LYS:O	48:x:80:LYS:N	2.38	0.47
1:C:676:U:H2'	1:C:677:G:H8	1.80	0.47
1:C:774:A:H5"	38:r:125:LYS:HD3	1.95	0.47
1:C:1213:A:H2'	1:C:1214:G:C8	2.50	0.47
10:d:80:HIS:CD2	10:d:81:PRO:HD2	2.50	0.47
19:I:903:C:H2'	19:I:904:G:C8	2.50	0.47
19:I:1079:C:O2'	19:I:1080:A:H8	1.98	0.47
19:I:1387:A:H5'	19:I:1469:A:H1'	1.96	0.47
19:I:1826:G:OP1	23:K:223:THR:N	2.47	0.47
19:I:1930:G:O2'	19:I:1968:G:O6	2.29	0.47
19:I:2349:G:O5'	26:l:42:ARG:NH2	2.47	0.47
19:I:2357:G:N2	19:I:2360:G:OP2	2.36	0.47
27:M:117:ARG:O	27:M:186:VAL:HB	2.14	0.47
32:o:8:ALA:HB2	32:o:77:ARG:HD2	1.97	0.47
42:u:23:TYR:H	42:u:66:GLU:HG2	1.78	0.47
1:C:288:U:H2'	1:C:289:C:C6	2.49	0.47
1:C:536:G:H5'	13:F:39:GLY:HA2	1.96	0.47
1:C:595:G:H2'	1:C:596:A:H8	1.80	0.47
1:C:772:G:H21	38:r:122:ARG:HD2	1.79	0.47
3:Z:37:GLU:H	3:Z:37:GLU:CD	2.23	0.47
5:4:36:LEU:HD13	5:4:48:LYS:HA	1.97	0.47
19:I:1198:U:H2'	19:I:1199:U:C6	2.49	0.47
19:I:1587:G:H2'	19:I:1588:G:C8	2.49	0.47
19:I:2070:A:H2'	19:I:2071:A:C8	2.50	0.47
19:I:2322:A:H61	19:I:2333:A:N6	2.11	0.47
19:I:2514:U:H2'	19:I:2515:C:H6	1.79	0.47
19:I:2529:G:H5"	19:I:2530:A:H5"	1.97	0.47
19:I:2651:C:C4	19:I:2652:C:N4	2.83	0.47
23:K:168:ASP:OD1	23:K:168:ASP:N	2.46	0.47
26:l:16:LYS:HE2	26:l:20:GLY:HA2	1.96	0.47
31:O:101:ASN:O	31:O:116:GLN:NE2	2.44	0.47
34:p:12:ARG:HG3	34:p:13:LYS:HG3	1.96	0.47
34:p:62:ASP:O	34:p:63:LEU:HD22	2.15	0.47
41:T:82:LEU:HD21	41:T:120:VAL:HG21	1.96	0.47
42:u:44:LYS:O	42:u:45:ILE:HG22	2.15	0.47
44:v:26:GLU:O	44:v:30:ILE:HG12	2.15	0.47
1:C:6:G:H4'	1:C:292:A:H4'	1.96	0.47
1:C:1154:G:H21	1:C:1156:C:H41	1.63	0.47
9:D:163:VAL:HG12	9:D:165:ASP:H	1.80	0.47
11:E:88:ARG:O	11:E:91:VAL:HG12	2.15	0.47
19:I:362:A:H3'	19:I:363:G:H8	1.79	0.47

Continued on next page...

For Manuscript Review

Continued from previous page...

Atom-1	Atom-2	Interatomic distance (Å)	Clash overlap (Å)
19:I:1086:A:O2'	19:I:1087:G:N7	2.48	0.47
19:I:2627:G:N2	19:I:2781:A:H2'	2.29	0.47
26:l:11:ALA:HA	26:l:62:LEU:HD21	1.97	0.47
43:U:62:LYS:HG3	43:U:63:ILE:N	2.29	0.47
50:y:19:LYS:HB3	50:y:47:HIS:CE1	2.49	0.47
1:C:43:C:H5"	48:x:11:ALA:O	2.15	0.47
1:C:919:G:C2	1:C:921:G:C8	3.03	0.47
1:C:1220:C:N4	42:u:103:LYS:HD3	2.29	0.47
1:C:1312:A:H5"	2:1:3:ARG:NH1	2.30	0.47
17:H:19:PRO:O	17:H:23:GLU:HG2	2.14	0.47
19:I:45:G:H5'	19:I:46:G:OP1	2.15	0.47
19:I:796:C:H2'	19:I:797:G:C8	2.50	0.47
21:J:66:A:H1'	21:J:68:C:H41	1.80	0.47
23:K:108:LYS:HA	23:K:196:GLY:HA2	1.97	0.47
31:O:13:ALA:O	31:O:15:VAL:N	2.48	0.47
34:p:47:VAL:O	34:p:80:ARG:HG3	2.14	0.47
39:S:1:MET:HE3	39:S:1:MET:HB2	1.69	0.47
42:u:44:LYS:C	42:u:46:SER:H	2.21	0.47
1:C:538:G:H2'	1:C:539:C:O4'	2.14	0.47
1:C:642:G:H2'	1:C:643:A:H8	1.80	0.47
1:C:917:A:H2'	1:C:918:C:C6	2.49	0.47
1:C:1092:C:O2'	6:3:71:TYR:O	2.21	0.47
9:D:214:LEU:HA	9:D:217:VAL:HG12	1.97	0.47
19:I:17:G:H2'	19:I:18:U:C6	2.50	0.47
19:I:843:G:H2'	19:I:844:A:O4'	2.15	0.47
19:I:1047:G:O2'	19:I:1110:G:O6	2.33	0.47
19:I:1219:U:H2'	19:I:1220:G:C8	2.48	0.47
19:I:2701:U:H3'	19:I:2702:G:H5"	1.97	0.47
20:i:54:VAL:HG21	45:V:98:LEU:HD22	1.97	0.47
21:J:53:A:H2'	21:J:54:G:C8	2.49	0.47
31:O:46:ALA:O	31:O:49:THR:OG1	2.31	0.47
48:x:12:LYS:O	48:x:13:LYS:NZ	2.45	0.47
1:C:72:G:H21	1:C:91:A:H8	1.63	0.46
1:C:268:A:H4'	1:C:269:G:O5'	2.14	0.46
1:C:516:C:H41	40:t:50:ARG:NH2	2.13	0.46
1:C:572:C:O2'	1:C:722:A:N3	2.41	0.46
1:C:573:G:H5'	1:C:722:A:H1'	1.97	0.46
1:C:809:A:N7	1:C:1503:C:O2'	2.40	0.46
19:I:272:A:H2'	19:I:273:G:H8	1.79	0.46
19:I:594:U:H2'	19:I:595:C:C6	2.50	0.46
19:I:1747:U:H2'	19:I:1748:C:C6	2.50	0.46

Continued on next page...

For Manuscript Review

Continued from previous page...

Atom-1	Atom-2	Interatomic distance (Å)	Clash overlap (Å)
19:I:2776:A:H4'	19:I:2777:G:O5'	2.15	0.46
21:J:32:U:H2'	21:J:33:G:C8	2.50	0.46
27:M:113:VAL:HG22	27:M:118:LEU:HD22	1.98	0.46
29:N:34:ILE:HG13	29:N:96:MET:HE3	1.97	0.46
40:t:52:VAL:HG12	40:t:66:TYR:HA	1.97	0.46
48:x:12:LYS:O	48:x:12:LYS:HD3	2.16	0.46
1:C:228:C:H2'	1:C:229:U:H6	1.80	0.46
1:C:509:G:H2'	1:C:510:U:H6	1.81	0.46
9:D:27:MET:SD	9:D:193:PRO:HG3	2.55	0.46
9:D:171:ILE:O	9:D:175:GLU:HG3	2.16	0.46
15:G:83:HIS:NE2	15:G:148:ASN:OD1	2.47	0.46
19:I:1597:A:H5"	19:I:1598:A:H5'	1.97	0.46
19:I:1771:C:H2'	19:I:1772:A:H8	1.80	0.46
19:I:2289:G:H2'	19:I:2290:G:C8	2.50	0.46
19:I:2881:U:H2'	19:I:2882:A:C8	2.50	0.46
21:J:115:A:H2'	21:J:116:G:H8	1.80	0.46
27:M:147:LEU:HD12	27:M:148:ILE:H	1.79	0.46
31:O:92:VAL:HG13	31:O:160:LYS:HD2	1.97	0.46
39:S:66:LYS:HE2	39:S:66:LYS:HB3	1.65	0.46
44:v:21:PHE:HD1	44:v:25:ALA:HB2	1.80	0.46
49:X:88:ARG:HE	49:X:88:ARG:HB3	1.58	0.46
1:C:630:U:H2'	1:C:631:C:C6	2.50	0.46
1:C:712:A:H5'	38:r:119:ASN:OD1	2.15	0.46
1:C:717:U:O2	1:C:849:G:H4'	2.15	0.46
1:C:1519:G:OP2	6:3:40:LYS:NZ	2.46	0.46
2:1:3:ARG:HH21	2:1:7:LYS:HD3	1.80	0.46
12:e:39:ARG:HG2	12:e:58:THR:HG22	1.97	0.46
17:H:66:ALA:HB1	17:H:70:VAL:HG11	1.96	0.46
19:I:395:U:O2'	19:I:396:G:N7	2.40	0.46
19:I:656:G:H2'	19:I:657:U:C6	2.50	0.46
19:I:682:G:H5'	24:k:26:ASN:CG	2.41	0.46
19:I:1447:C:H2'	19:I:1448:G:H8	1.80	0.46
19:I:2245:U:H5"	19:I:2246:G:H5'	1.96	0.46
31:O:55:ARG:HB3	31:O:58:TYR:CE2	2.51	0.46
32:o:125:ILE:O	32:o:125:ILE:HD12	2.16	0.46
38:r:112:ASP:OD2	38:r:114:THR:HG22	2.15	0.46
50:y:19:LYS:O	50:y:47:HIS:ND1	2.46	0.46
1:C:68:G:H1	1:C:95:A:H61	1.63	0.46
1:C:511:G:H1	1:C:527:A:P	2.38	0.46
1:C:672:U:H2'	1:C:673:U:C6	2.51	0.46
1:C:731:A:H2'	1:C:732:C:C6	2.49	0.46

Continued on next page...

For Manuscript Review

Continued from previous page...

Atom-1	Atom-2	Interatomic distance (Å)	Clash overlap (Å)
1:C:979:C:H2'	1:C:980:U:C6	2.50	0.46
1:C:1154:G:H21	1:C:1156:C:N4	2.13	0.46
1:C:1281:A:C2	1:C:1347:G:H1'	2.51	0.46
2:1:44:MET:O	2:1:47:LEU:HB2	2.15	0.46
19:I:1093:G:H21	19:I:1098:A:H62	1.63	0.46
19:I:1469:A:H2'	19:I:1470:A:C8	2.50	0.46
19:I:1685:C:H2'	19:I:1686:C:H6	1.80	0.46
19:I:2039:U:H2'	19:I:2040:G:C8	2.49	0.46
19:I:2070:A:H2'	19:I:2071:A:H8	1.81	0.46
21:J:2:G:H2'	21:J:3:C:C6	2.51	0.46
30:n:6:VAL:C	30:n:8:GLY:H	2.23	0.46
36:q:9:ARG:HB2	36:q:99:GLN:HB2	1.98	0.46
1:C:161:C:H2'	1:C:162:G:C8	2.51	0.46
1:C:487:A:H3'	1:C:488:G:C8	2.50	0.46
1:C:869:U:O2'	32:o:15:ARG:HD2	2.16	0.46
1:C:1115:U:H2'	1:C:1116:U:C6	2.51	0.46
1:C:1169:G:H2'	1:C:1170:A:C8	2.46	0.46
1:C:1452:G:H2'	1:C:1453:A:C8	2.50	0.46
8:c:22:ARG:NH1	8:c:73:PHE:HB2	2.31	0.46
16:g:28:LEU:HD11	16:g:42:LEU:HB3	1.98	0.46
17:H:29:ILE:CD1	17:H:64:VAL:HB	2.45	0.46
19:I:81:G:HO2'	19:I:295:G:HO2'	1.63	0.46
19:I:671:C:OP1	41:T:43:GLY:N	2.34	0.46
19:I:703:U:H2'	19:I:704:G:O4'	2.15	0.46
19:I:755:U:H2'	19:I:756:A:C8	2.51	0.46
19:I:1013:C:H2'	19:I:1014:A:H8	1.79	0.46
19:I:1023:U:O2	19:I:1023:U:H2'	2.15	0.46
19:I:1283:G:N1	19:I:1286:A:OP2	2.47	0.46
19:I:1865:U:H4'	19:I:1866:A:OP1	2.15	0.46
19:I:1980:G:O2'	19:I:1982:U:OP2	2.28	0.46
19:I:2345:G:H4'	19:I:2347:C:OP2	2.15	0.46
50:y:57:ASP:HB3	50:y:80:GLU:O	2.15	0.46
1:C:209:C:HO2'	1:C:210:U:P	2.38	0.46
1:C:1506:U:H2'	1:C:1507:A:H8	1.79	0.46
2:1:19:VAL:HA	2:1:22:ALA:HB2	1.97	0.46
11:E:20:SER:HB2	44:v:94:PRO:HG3	1.97	0.46
19:I:291:G:C6	19:I:350:G:C6	3.04	0.46
19:I:307:G:N1	19:I:310:A:OP2	2.28	0.46
19:I:552:U:H2'	19:I:553:G:H8	1.81	0.46
19:I:1733:G:H2'	19:I:1734:G:H8	1.81	0.46
19:I:2531:A:H61	19:I:2662:A:N6	2.14	0.46

Continued on next page...

For Manuscript Review

Continued from previous page...

Atom-1	Atom-2	Interatomic distance (Å)	Clash overlap (Å)
19:I:2744:G:H2’	19:I:2745:C:C6	2.50	0.46
25:L:38:LYS:HB2	25:L:47:ALA:H	1.80	0.46
1:C:198:G:C2	1:C:199:A:H1’	2.50	0.46
1:C:460:A:H5”	1:C:462:A:C2	2.51	0.46
1:C:596:A:H2’	1:C:597:U:C6	2.51	0.46
1:C:990:A:N1	1:C:1039:C:O2’	2.45	0.46
1:C:1521:C:H2’	1:C:1522:U:C6	2.50	0.46
7:b:61:LEU:HD11	7:b:82:LYS:HE2	1.97	0.46
9:D:86:SER:HB3	9:D:222:ARG:HH21	1.80	0.46
11:E:134:MET:HE2	11:E:168:TYR:CD2	2.51	0.46
19:I:592:A:H2	26:l:4:ILE:HD11	1.80	0.46
19:I:2818:U:H2’	19:I:2819:G:C8	2.51	0.46
21:J:9:G:C5	21:J:112:G:C6	3.04	0.46
21:J:90:C:H5’	43:U:18:ARG:HG2	1.97	0.46
33:P:88:GLY:O	33:P:125:THR:OG1	2.30	0.46
36:q:8:ILE:O	36:q:73:LEU:HD12	2.15	0.46
42:u:18:ALA:C	42:u:20:THR:H	2.24	0.46
43:U:53:MET:HE1	43:U:103:TYR:CG	2.51	0.46
49:X:31:TRP:CE3	49:X:38:LYS:HG2	2.50	0.46
1:C:173:A:O5’	1:C:174:U:H5”	2.15	0.46
1:C:262:C:H2’	1:C:263:U:H6	1.80	0.46
1:C:773:C:H1’	38:r:122:ARG:HD3	1.97	0.46
19:I:355:U:H2’	19:I:356:G:C8	2.51	0.46
19:I:640:C:H2’	19:I:641:U:O4’	2.15	0.46
19:I:1077:A:H2	19:I:1088:A:H62	1.64	0.46
19:I:1440:U:H2’	19:I:1441:G:C8	2.51	0.46
19:I:1510:G:H2’	19:I:1511:G:C8	2.50	0.46
19:I:2011:U:H2’	19:I:2012:G:O4’	2.16	0.46
19:I:2687:U:H2’	19:I:2688:G:O4’	2.15	0.46
20:i:28:LEU:HD12	20:i:37:LYS:HD2	1.97	0.46
21:J:115:A:H4’	47:W:55:GLU:HG2	1.98	0.46
30:n:25:LYS:HB2	30:n:25:LYS:HE2	1.59	0.46
1:C:352:U:C2	1:C:353:G:C8	3.04	0.46
1:C:944:U:H2’	1:C:945:G:C8	2.50	0.46
1:C:1366:U:H5”	34:p:73:SER:HB2	1.98	0.46
1:C:1472:U:H2’	1:C:1473:C:C6	2.51	0.46
5:4:46:LEU:O	5:4:50:VAL:HG23	2.16	0.46
15:G:15:LEU:HD21	15:G:18:VAL:HG23	1.98	0.46
19:I:153:U:H2’	19:I:154:U:H6	1.81	0.46
19:I:1038:G:H2’	19:I:1039:A:H8	1.77	0.46
19:I:2584:U:H2’	19:I:2585:U:H2’	1.98	0.46

Continued on next page...

For Manuscript Review

Continued from previous page...

Atom-1	Atom-2	Interatomic distance (Å)	Clash overlap (Å)
25:L:172:VAL:HB	25:L:175:LEU:HD11	1.97	0.46
29:N:33:LYS:HD2	29:N:35:THR:HB	1.98	0.46
29:N:128:TYR:O	29:N:156:ILE:N	2.49	0.46
30:n:124:LEU:HA	30:n:124:LEU:HD23	1.77	0.46
1:C:707:G:H2'	1:C:708:G:H8	1.78	0.46
2:1:43:ASN:OD1	2:1:43:ASN:C	2.58	0.46
17:H:90:MET:SD	52:z:61:ARG:NH2	2.74	0.46
19:I:796:C:H2'	19:I:797:G:H8	1.80	0.46
19:I:925:A:H2'	19:I:926:G:H8	1.80	0.46
19:I:1432:G:H2'	19:I:1433:A:H8	1.77	0.46
19:I:1771:C:H2'	19:I:1772:A:C8	2.51	0.46
19:I:2530:A:N7	31:O:172:LYS:NZ	2.42	0.46
19:I:2818:U:H2'	19:I:2819:G:H8	1.80	0.46
29:N:116:GLY:HA2	29:N:176:PRO:HB2	1.98	0.46
1:C:488:G:H2'	1:C:490:A:H8	1.81	0.45
1:C:657:A:O3'	52:z:53:ARG:NH1	2.48	0.45
1:C:708:G:H2'	1:C:709:A:H8	1.78	0.45
1:C:1452:G:H2'	1:C:1453:A:H8	1.81	0.45
7:b:39:THR:OG1	7:b:42:GLU:OE1	2.27	0.45
12:e:21:LEU:HD22	12:e:39:ARG:NE	2.31	0.45
17:H:51:ILE:HD11	52:z:66:SER:HB2	1.98	0.45
19:I:277:G:H2'	19:I:361:G:C6	2.51	0.45
19:I:858:G:H3'	19:I:859:G:C8	2.51	0.45
19:I:1295:C:C2	19:I:1296:G:C8	3.04	0.45
19:I:1422:G:N2	19:I:1498:C:O2	2.47	0.45
19:I:2530:A:N6	31:O:172:LYS:HD2	2.29	0.45
21:J:114:C:H2'	21:J:115:A:H8	1.81	0.45
23:K:258:ARG:HH22	23:K:263:THR:HB	1.81	0.45
1:C:43:C:P	48:x:12:LYS:HD2	2.56	0.45
1:C:317:U:H4'	4:2:17:ALA:HB3	1.99	0.45
1:C:412:U:H2'	1:C:413:C:C6	2.51	0.45
1:C:556:U:H1'	40:t:12:ARG:HB3	1.98	0.45
1:C:630:U:H2'	1:C:631:C:H6	1.81	0.45
1:C:739:U:H5'	1:C:829:G:N2	2.31	0.45
1:C:1307:U:H2'	1:C:1308:C:H6	1.82	0.45
5:4:29:VAL:HG12	5:4:51:LEU:HD11	1.98	0.45
5:4:78:GLU:O	19:I:24:G:O2'	2.25	0.45
13:F:58:LYS:HD3	13:F:203:LEU:HD12	1.98	0.45
13:F:202:GLU:OE1	15:G:112:ARG:NH2	2.41	0.45
19:I:796:C:OP1	27:M:57:LYS:HD2	2.15	0.45
19:I:807:U:C2	19:I:808:G:C8	3.04	0.45

Continued on next page...

For Manuscript Review

Continued from previous page...

Atom-1	Atom-2	Interatomic distance (Å)	Clash overlap (Å)
19:I:1494:A:H2'	19:I:1495:A:C8	2.51	0.45
19:I:1571:A:H2'	19:I:1572:A:O4'	2.16	0.45
19:I:2282:G:H4'	19:I:2389:G:O2'	2.16	0.45
19:I:2345:G:H21	19:I:2381:A:H1'	1.82	0.45
19:I:2350:C:H3'	19:I:2351:G:C8	2.48	0.45
19:I:2641:G:H2'	19:I:2642:G:H8	1.81	0.45
19:I:2813:A:H2'	19:I:2814:A:H8	1.81	0.45
36:q:12:ALA:HB2	36:q:96:VAL:HG12	1.98	0.45
1:C:102:G:C5	4:2:10:ARG:HD3	2.51	0.45
1:C:265:C:HO2'	1:C:266:C:P	2.39	0.45
1:C:647:A:H2	32:o:28:PRO:HB3	1.81	0.45
1:C:885:U:H2'	1:C:886:A:H8	1.82	0.45
1:C:952:A:C6	2:1:55:ARG:HB2	2.52	0.45
1:C:1471:U:H2'	1:C:1472:U:H6	1.81	0.45
10:d:7:GLU:OE2	10:d:10:LYS:NZ	2.48	0.45
13:F:34:ILE:HG23	13:F:35:GLU:HG2	1.99	0.45
19:I:374:A:H3'	19:I:375:G:C8	2.42	0.45
19:I:833:A:H2'	19:I:834:G:H8	1.79	0.45
19:I:1079:C:H41	19:I:1088:A:P	2.40	0.45
19:I:1253:A:OP1	51:Y:33:ARG:NH2	2.49	0.45
19:I:1825:U:H2'	19:I:1826:G:C8	2.50	0.45
19:I:2375:G:H5'	19:I:2376:A:OP2	2.16	0.45
19:I:2700:A:N6	19:I:2706:A:H61	2.10	0.45
21:J:29:A:H2	21:J:57:A:H61	1.64	0.45
21:J:62:C:H2'	21:J:63:C:C6	2.51	0.45
29:N:54:ALA:HB1	29:N:65:PRO:HG3	1.99	0.45
48:x:73:ALA:HA	48:x:76:LYS:HE3	1.99	0.45
1:C:45:G:H5"	1:C:301:U:H1'	1.99	0.45
1:C:104:C:O2'	48:x:25:ARG:O	2.33	0.45
1:C:189:A:O4'	1:C:216:U:O2'	2.30	0.45
1:C:442:A:H3'	1:C:443:G:H21	1.82	0.45
1:C:1341:G:O6	34:p:12:ARG:NH2	2.41	0.45
17:H:18:VAL:HG11	17:H:58:HIS:CD2	2.52	0.45
19:I:582:A:H2'	19:I:583:G:C8	2.49	0.45
19:I:1035:U:O2'	19:I:1036:G:H5"	2.17	0.45
19:I:1198:U:H2'	19:I:1199:U:H6	1.82	0.45
19:I:1297:C:H2'	19:I:1298:C:H6	1.81	0.45
19:I:1435:G:H2'	19:I:1436:G:H8	1.80	0.45
19:I:1499:C:C2	19:I:1500:G:C8	3.04	0.45
19:I:2485:G:O3'	43:U:125:PRO:HB3	2.15	0.45
30:n:72:THR:HG22	30:n:142:HIS:CE1	2.52	0.45

Continued on next page...

For Manuscript Review

Continued from previous page...

Atom-1	Atom-2	Interatomic distance (Å)	Clash overlap (Å)
52:z:21:ILE:HD11	52:z:55:LEU:HA	1.99	0.45
52:z:41:PRO:HD2	52:z:44:ILE:HD12	1.99	0.45
1:C:999:A:H5”	1:C:1000:C:C5	2.51	0.45
19:I:3:U:H2’	19:I:4:U:C6	2.51	0.45
19:I:244:A:H2’	19:I:245:G:O4’	2.16	0.45
19:I:547:A:N3	19:I:548:G:N2	2.47	0.45
19:I:722:A:H2’	19:I:723:C:C6	2.51	0.45
19:I:1360:G:C8	19:I:1361:G:C8	3.04	0.45
19:I:1363:C:O2’	19:I:1809:A:N3	2.41	0.45
19:I:1751:U:H2’	19:I:1752:C:C6	2.51	0.45
19:I:1862:G:H2’	19:I:1863:G:H8	1.81	0.45
19:I:1928:A:H2’	19:I:1929:G:O4’	2.17	0.45
19:I:2352:A:H3’	19:I:2353:G:C8	2.51	0.45
23:K:78:VAL:HG22	23:K:94:VAL:HG12	1.99	0.45
36:q:27:GLU:HA	36:q:30:LYS:HG2	1.98	0.45
40:t:50:ARG:HG2	40:t:66:TYR:CE1	2.51	0.45
1:C:171:G:O2’	1:C:172:C:P	2.74	0.45
1:C:245:G:N2	1:C:260:G:O6	2.49	0.45
1:C:329:C:O2’	1:C:1427:A:N3	2.42	0.45
1:C:404:G:H2’	1:C:423:U:C4	2.51	0.45
1:C:546:U:H2’	1:C:547:A:C8	2.47	0.45
1:C:952:A:OP1	2:1:55:ARG:NH1	2.47	0.45
1:C:996:A:H2’	1:C:997:G:C8	2.51	0.45
1:C:1054:U:H2’	1:C:1055:G:H8	1.81	0.45
1:C:1260:G:N2	1:C:1262:G:O2’	2.50	0.45
1:C:1307:U:H2’	1:C:1308:C:C6	2.50	0.45
13:F:8:LYS:HB3	13:F:21:LEU:HB3	1.98	0.45
19:I:239:C:O2’	19:I:622:G:O2’	2.23	0.45
19:I:1199:U:H2’	19:I:1200:C:H6	1.81	0.45
19:I:1292:G:H2’	19:I:1293:C:C6	2.51	0.45
19:I:2670:A:H2’	19:I:2671:G:C8	2.51	0.45
21:J:34:A:N6	21:J:44:G:H1’	2.30	0.45
43:U:4:PRO:HG3	43:U:68:PHE:HE2	1.81	0.45
47:W:92:PHE:HB2	47:W:117:PHE:CD1	2.51	0.45
1:C:75:G:H1	1:C:88:U:H3	1.65	0.45
1:C:397:C:OP1	13:F:134:SER:OG	2.28	0.45
1:C:902:A:H2’	1:C:903:A:C8	2.51	0.45
1:C:1480:G:H2’	1:C:1481:G:O4’	2.17	0.45
6:3:31:GLU:OE1	6:3:34:ARG:NH2	2.50	0.45
13:F:15:GLU:HG3	13:F:19:LEU:HD21	1.99	0.45
19:I:914:G:H5’	19:I:915:C:OP2	2.17	0.45

Continued on next page...

For Manuscript Review

Continued from previous page...

Atom-1	Atom-2	Interatomic distance (Å)	Clash overlap (Å)
19:I:936:A:C6	19:I:937:C:N4	2.85	0.45
19:I:947:A:H2'	19:I:948:C:C6	2.51	0.45
19:I:1077:A:O2'	19:I:1078:U:O4'	2.35	0.45
19:I:1190:G:H2'	19:I:1191:G:H8	1.82	0.45
19:I:1564:C:H2'	19:I:1565:C:C6	2.51	0.45
19:I:1649:G:O2'	45:V:106:ASP:OD2	2.29	0.45
19:I:1736:U:C4	19:I:1737:G:C6	3.05	0.45
27:M:61:ARG:HG3	27:M:63:LYS:O	2.17	0.45
29:N:143:TYR:OH	42:u:11:ASP:OD2	2.25	0.45
43:U:67:VAL:HG21	43:U:96:ILE:HD11	1.99	0.45
47:W:12:THR:HG22	47:W:16:ARG:HG2	1.99	0.45
1:C:19:A:H5"	15:G:91:GLY:HA3	1.98	0.45
1:C:94:C:O2'	1:C:95:A:C8	2.69	0.45
1:C:216:U:H2'	1:C:217:A:C8	2.52	0.45
1:C:516:C:OP2	40:t:66:TYR:OH	2.22	0.45
1:C:573:G:H2'	1:C:574:C:H6	1.82	0.45
10:d:80:HIS:CG	10:d:81:PRO:HD2	2.51	0.45
18:h:44:ILE:HA	18:h:47:MET:HG2	1.98	0.45
19:I:117:G:OP2	19:I:119:A:O2'	2.24	0.45
19:I:721:A:H2'	19:I:722:A:C8	2.52	0.45
19:I:1006:C:O2'	37:R:108:MET:O	2.33	0.45
19:I:2891:U:H2'	19:I:2892:G:C8	2.52	0.45
33:P:116:ARG:NH2	33:P:133:GLN:OE1	2.49	0.45
1:C:229:U:H2'	1:C:230:A:C8	2.52	0.45
1:C:364:G:H2'	1:C:365:A:H8	1.81	0.45
1:C:507:U:H2'	1:C:508:C:H6	1.82	0.45
1:C:668:G:N2	38:r:118:HIS:HB2	2.32	0.45
1:C:930:C:C4	1:C:931:A:N7	2.85	0.45
1:C:1181:G:H2'	1:C:1182:A:C8	2.52	0.45
1:C:1181:G:H2'	1:C:1182:A:H8	1.82	0.45
19:I:16:C:H2'	19:I:17:G:H8	1.82	0.45
19:I:693:A:O2'	19:I:1353:A:N3	2.45	0.45
19:I:782:A:N7	23:K:220:VAL:HG21	2.31	0.45
19:I:1032:A:O2'	19:I:1033:U:H5'	2.16	0.45
19:I:1063:G:H2'	19:I:1064:C:O4'	2.17	0.45
19:I:1117:C:HO2'	19:I:1118:C:H6	1.65	0.45
19:I:1341:G:OP1	19:I:1397:U:N3	2.47	0.45
19:I:1400:U:H2'	19:I:1401:G:C8	2.52	0.45
19:I:1529:G:H2'	19:I:1530:G:C8	2.52	0.45
19:I:1750:G:H2'	19:I:1751:U:C6	2.52	0.45
19:I:1927:A:H2'	19:I:1928:A:C8	2.52	0.45

Continued on next page...

For Manuscript Review

Continued from previous page...

Atom-1	Atom-2	Interatomic distance (Å)	Clash overlap (Å)
48:x:6:LEU:HD12	48:x:70:ARG:HB3	1.99	0.45
49:X:22:PRO:HD3	49:X:50:ILE:HD12	1.99	0.45
52:z:26:ILE:HG22	52:z:30:LYS:HG2	1.97	0.45
1:C:125:U:H2’	1:C:126:C:C6	2.52	0.45
1:C:454:A:H2’	1:C:455:A:O4’	2.16	0.45
2:1:46:GLY:O	2:1:47:LEU:HD22	2.16	0.45
19:I:500:G:N1	19:I:503:A:OP2	2.49	0.45
19:I:674:G:P	27:M:49:ARG:HH22	2.40	0.45
19:I:2096:C:H2’	19:I:2097:A:C8	2.51	0.45
19:I:2323:G:H2’	19:I:2323:G:N3	2.32	0.45
19:I:2820:A:H4’	45:V:3:HIS:CD2	2.52	0.45
38:r:31:ILE:HG23	38:r:46:THR:HG22	1.99	0.45
47:W:4:LYS:O	47:W:8:ILE:HD12	2.17	0.45
1:C:349:C:H2’	1:C:350:A:O4’	2.17	0.44
1:C:859:A:H2’	1:C:860:C:C6	2.52	0.44
1:C:871:G:O2’	32:o:5:ASP:OD1	2.28	0.44
1:C:1239:G:H2’	1:C:1240:G:H8	1.82	0.44
11:E:91:VAL:HG11	11:E:101:ILE:HD11	1.98	0.44
19:I:37:C:H2’	19:I:38:A:C8	2.52	0.44
19:I:152:A:H2’	19:I:153:U:C6	2.52	0.44
19:I:266:G:H1	19:I:425:G:N2	2.09	0.44
19:I:712:G:C6	19:I:713:G:C6	3.06	0.44
19:I:1703:G:H2’	19:I:1704:C:C6	2.52	0.44
19:I:2300:C:H2’	19:I:2301:C:C6	2.52	0.44
19:I:2347:C:H2’	19:I:2348:U:H6	1.83	0.44
19:I:2508:G:H1	19:I:2580:PSU:HN3	1.65	0.44
23:K:133:ARG:NE	33:P:123:ARG:HH11	2.15	0.44
29:N:135:GLN:HG3	29:N:141:ILE:HD11	1.98	0.44
30:n:29:ILE:HG22	30:n:105:VAL:HG21	1.99	0.44
36:q:53:ILE:HD11	36:q:63:ASP:CG	2.42	0.44
37:R:74:TYR:CG	37:R:92:MET:HE2	2.52	0.44
38:r:82:LEU:HD23	38:r:82:LEU:HA	1.77	0.44
45:V:97:ILE:HG12	45:V:113:ILE:HG12	1.99	0.44
8:c:26:LYS:HG3	8:c:27:ASN:OD1	2.17	0.44
14:f:13:VAL:HG23	14:f:29:PHE:HB2	2.00	0.44
14:f:56:MET:HE3	14:f:56:MET:HB2	1.77	0.44
14:f:72:ARG:NE	14:f:78:TYR:OH	2.35	0.44
19:I:143:C:H2’	19:I:144:A:C8	2.52	0.44
19:I:417:C:H2’	19:I:418:C:H6	1.81	0.44
19:I:538:A:H2’	19:I:539:G:O4’	2.17	0.44
19:I:2345:G:N1	19:I:2373:G:O6	2.50	0.44

Continued on next page...

For Manuscript Review

Continued from previous page...

Atom-1	Atom-2	Interatomic distance (Å)	Clash overlap (Å)
19:I:2690:U:OP1	45:V:14:SER:OG	2.27	0.44
19:I:2901:C:H3'	19:I:2902:C:H6	1.82	0.44
21:J:94:A:H2'	21:J:95:U:O4'	2.16	0.44
25:L:148:GLN:HB2	25:L:152:PRO:HG3	1.99	0.44
26:l:15:LYS:HB2	26:l:23:LYS:HE3	2.00	0.44
32:o:43:GLU:OE1	32:o:43:GLU:C	2.61	0.44
40:t:30:LYS:HA	40:t:30:LYS:HD2	1.90	0.44
41:T:21:ARG:HA	41:T:21:ARG:HD3	1.82	0.44
44:v:45:VAL:O	44:v:48:LEU:HD12	2.18	0.44
50:y:4:LYS:HG2	50:y:5:ILE:H	1.81	0.44
2:1:28:LYS:HB3	2:1:28:LYS:HE2	1.67	0.44
15:G:137:VAL:C	15:G:139:ALA:H	2.25	0.44
18:h:27:LEU:O	18:h:38:ARG:NH2	2.32	0.44
19:I:643:A:H2'	19:I:643:A:N3	2.33	0.44
19:I:898:C:H2'	19:I:899:A:O4'	2.17	0.44
19:I:959:A:H2'	19:I:960:A:C8	2.52	0.44
19:I:1462:C:O2'	19:I:2702:G:O2'	2.18	0.44
19:I:2215:C:H2'	19:I:2216:G:C8	2.52	0.44
19:I:2651:C:H2'	19:I:2652:C:C6	2.52	0.44
19:I:2773:C:H2'	19:I:2774:C:H6	1.82	0.44
19:I:2784:U:H2'	19:I:2785:C:C6	2.51	0.44
19:I:2841:C:H2'	19:I:2842:G:H8	1.82	0.44
23:K:120:VAL:HG13	23:K:131:PRO:HG2	1.98	0.44
23:K:205:LEU:HB3	23:K:210:ALA:HB3	1.99	0.44
29:N:143:TYR:CD2	42:u:8:ASN:HB3	2.51	0.44
45:V:35:LYS:HB2	45:V:112:TYR:CE1	2.52	0.44
48:x:74:LEU:O	48:x:78:VAL:HG23	2.18	0.44
1:C:124:A:H1'	1:C:257:A:O2'	2.18	0.44
1:C:312:G:C6	1:C:330:A:C6	3.06	0.44
1:C:490:A:H61	1:C:492:A:N6	2.16	0.44
1:C:1027:G:H2'	1:C:1028:G:O4'	2.17	0.44
1:C:1404:A:H2'	1:C:1405:C:C6	2.52	0.44
10:d:89:ILE:HD12	10:d:89:ILE:HA	1.79	0.44
13:F:11:LEU:HD13	13:F:63:ARG:HD3	1.99	0.44
13:F:170:TRP:CZ3	13:F:190:ASP:HB3	2.53	0.44
19:I:755:U:H2'	19:I:756:A:H8	1.82	0.44
19:I:1297:C:H2'	19:I:1298:C:C6	2.53	0.44
19:I:1426:G:H2'	19:I:1427:A:N7	2.33	0.44
27:M:186:VAL:CG1	27:M:188:MET:HE3	2.46	0.44
42:u:3:ARG:HE	42:u:3:ARG:HB3	1.58	0.44
45:V:83:LEU:HA	45:V:86:ARG:HD3	1.99	0.44

Continued on next page...

For Manuscript Review

Continued from previous page...

Atom-1	Atom-2	Interatomic distance (Å)	Clash overlap (Å)
51:Y:90:ILE:HD11	51:Y:94:ILE:HG21	1.97	0.44
52:z:29:LEU:HD23	52:z:68:LEU:HD11	1.98	0.44
1:C:91:A:H2'	1:C:91:A:N3	2.33	0.44
1:C:370:G:H4'	48:x:5:ARG:HG3	1.99	0.44
5:4:80:PRO:HD3	19:I:25:U:H5"	1.99	0.44
19:I:214:G:H1'	19:I:217:A:H5'	2.00	0.44
19:I:415:A:H2'	19:I:416:U:H6	1.82	0.44
19:I:817:C:O2'	19:I:839:U:OP1	2.27	0.44
19:I:1053:C:H2'	19:I:1054:A:C8	2.51	0.44
19:I:1060:U:H1'	19:I:1061:U:H5'	2.00	0.44
19:I:1412:U:H3	19:I:1591:A:H2	1.66	0.44
19:I:1939:5MU:OP1	19:I:2604:PSU:O2'	2.30	0.44
19:I:2333:A:H4'	19:I:2334:U:H3'	1.98	0.44
19:I:2468:A:H2'	19:I:2476:A:C6	2.52	0.44
19:I:2590:A:H2'	19:I:2591:C:H6	1.81	0.44
19:I:2760:C:H3'	19:I:2761:A:H8	1.83	0.44
21:J:24:G:H1'	21:J:27:C:N4	2.32	0.44
34:p:23:PRO:C	34:p:61:LEU:HB2	2.42	0.44
37:R:19:ASP:OD2	37:R:21:THR:HG23	2.17	0.44
41:T:56:PRO:O	41:T:60:ARG:HG3	2.18	0.44
42:u:81:MET:HE2	42:u:81:MET:HB3	1.87	0.44
50:y:17:MET:HE1	50:y:45:HIS:HD2	1.82	0.44
1:C:56:U:H2'	1:C:57:G:C8	2.52	0.44
1:C:407:G:H3'	1:C:408:A:H3'	1.99	0.44
1:C:786:A:H1'	1:C:788:A:N7	2.32	0.44
1:C:944:U:H2'	1:C:945:G:H8	1.81	0.44
1:C:961:C:H4'	34:p:127:PHE:HE2	1.82	0.44
1:C:1000:C:H2'	1:C:1001:U:C6	2.53	0.44
1:C:1134:G:H2'	1:C:1135:U:C6	2.52	0.44
2:1:18:LYS:HD3	2:1:18:LYS:HA	1.82	0.44
11:E:134:MET:HE2	11:E:168:TYR:HD2	1.83	0.44
19:I:538:A:H5"	37:R:7:LYS:HE2	1.98	0.44
19:I:562:U:H1'	19:I:2035:G:O2'	2.18	0.44
19:I:1431:A:H2'	19:I:1432:G:H8	1.82	0.44
19:I:1529:G:H2'	19:I:1530:G:H8	1.82	0.44
19:I:1567:G:H2'	23:K:85:PRO:HG3	1.99	0.44
19:I:1628:G:H2'	19:I:1629:U:C6	2.53	0.44
19:I:1682:G:OP2	19:I:1699:G:N2	2.48	0.44
19:I:1873:G:H2'	19:I:1874:C:H6	1.83	0.44
19:I:1883:U:H2'	19:I:1884:G:O4'	2.17	0.44
19:I:2148:G:H2'	19:I:2149:U:C6	2.53	0.44

Continued on next page...

For Manuscript Review

Continued from previous page...

Atom-1	Atom-2	Interatomic distance (Å)	Clash overlap (Å)
19:I:2266:A:H4'	19:I:2267:A:C2	2.53	0.44
19:I:2364:C:H2'	19:I:2365:G:O4'	2.18	0.44
25:L:28:GLU:HG3	25:L:28:GLU:O	2.17	0.44
25:L:108:ASP:OD1	25:L:173:GLN:HG2	2.17	0.44
26:l:26:HIS:HB3	26:l:44:LEU:HD22	1.99	0.44
28:m:7:VAL:HG12	28:m:35:GLN:HG3	2.00	0.44
29:N:34:ILE:HG12	29:N:156:ILE:HD13	1.99	0.44
32:o:77:ARG:NE	32:o:79:SER:O	2.50	0.44
38:r:38:GLN:CD	38:r:38:GLN:N	2.76	0.44
49:X:9:GLU:OE2	49:X:56:HIS:NE2	2.50	0.44
1:C:74:A:H62	1:C:89:G:N2	2.14	0.44
1:C:79:G:H2'	1:C:80:C:O4'	2.18	0.44
1:C:216:U:H2'	1:C:217:A:H8	1.82	0.44
1:C:757:G:H2'	1:C:758:C:H6	1.82	0.44
1:C:910:U:H2'	1:C:911:G:C8	2.52	0.44
1:C:1056:U:H2'	1:C:1057:C:C6	2.53	0.44
16:g:32:ALA:HB2	16:g:37:LEU:HD22	1.99	0.44
19:I:81:G:H2'	19:I:82:U:O4'	2.16	0.44
19:I:372:G:H4'	19:I:373:U:H5'	2.00	0.44
19:I:417:C:H2'	19:I:418:C:C6	2.53	0.44
19:I:561:G:O2'	51:Y:45:TYR:OH	2.23	0.44
22:j:39:PHE:CZ	22:j:44:ARG:HA	2.53	0.44
29:N:47:LYS:HA	29:N:50:LEU:HD13	1.98	0.44
39:S:70:ARG:HD3	39:S:76:VAL:HB	2.00	0.44
45:V:28:LEU:HD23	45:V:48:VAL:HG11	2.00	0.44
1:C:265:C:O2'	1:C:266:C:OP1	2.30	0.44
1:C:297:A:H2'	1:C:298:U:O4'	2.18	0.44
1:C:315:A:H2'	1:C:316:C:H6	1.83	0.44
1:C:785:G:O6	1:C:786:A:N6	2.49	0.44
1:C:1146:A:O2'	1:C:1147:G:H8	2.01	0.44
4:2:20:HIS:CD2	4:2:24:ARG:HE	2.35	0.44
4:2:61:GLN:OE1	4:2:61:GLN:HA	2.17	0.44
19:I:451:U:H4'	27:M:47:LYS:NZ	2.32	0.44
19:I:814:C:H2'	19:I:815:C:H6	1.83	0.44
19:I:966:G:H2'	19:I:967:U:C6	2.53	0.44
19:I:1429:G:H2'	19:I:1430:G:C8	2.51	0.44
19:I:1588:G:H2'	19:I:1589:U:C6	2.53	0.44
19:I:2831:G:N2	19:I:2884:U:OP2	2.51	0.44
40:t:39:THR:OG1	40:t:49:LEU:O	2.35	0.44
41:T:122:VAL:O	41:T:143:GLU:HB2	2.18	0.44
49:X:25:THR:HB	49:X:88:ARG:HB2	1.98	0.44

Continued on next page...

For Manuscript Review

Continued from previous page...

Atom-1	Atom-2	Interatomic distance (Å)	Clash overlap (Å)
1:C:20:U:H2'	1:C:21:G:O4'	2.18	0.44
1:C:151:C:H2'	1:C:152:G:C8	2.51	0.44
1:C:186:G:H2'	1:C:186:G:N3	2.33	0.44
1:C:999:A:C5	1:C:1000:C:H1'	2.52	0.44
1:C:1195:A:H1'	1:C:1196:U:OP2	2.18	0.44
1:C:1435:A:C6	49:X:114:LEU:HD21	2.53	0.44
5:4:1:MET:HE3	5:4:2:GLU:H	1.83	0.44
19:I:27:G:N1	19:I:512:G:H1'	2.33	0.44
19:I:647:G:H2'	19:I:647:G:N3	2.32	0.44
19:I:1361:G:H2'	19:I:1362:C:H6	1.81	0.44
19:I:1830:C:H2'	19:I:1831:G:H8	1.82	0.44
19:I:2347:C:H2'	19:I:2348:U:C6	2.53	0.44
19:I:2445:2MG:OP1	27:M:69:ARG:NH1	2.40	0.44
21:J:57:A:H2'	21:J:58:A:C8	2.52	0.44
37:R:13:ARG:NH2	37:R:49:ASP:O	2.30	0.44
39:S:64:ARG:HB2	39:S:79:PHE:CD1	2.52	0.44
49:X:53:ARG:H	49:X:57:SER:HB3	1.83	0.44
1:C:642:G:H2'	1:C:643:A:C8	2.52	0.43
1:C:1293:A:H2'	1:C:1293:A:N3	2.33	0.43
7:b:33:LYS:HG2	7:b:80:TRP:CZ3	2.53	0.43
14:f:39:TRP:CE3	33:P:32:PRO:HA	2.53	0.43
15:G:115:LEU:O	15:G:120:VAL:HG22	2.18	0.43
19:I:87:U:H3	19:I:94:A:N6	2.16	0.43
19:I:282:A:N6	19:I:359:G:O6	2.51	0.43
19:I:287:G:H2'	19:I:288:U:C6	2.53	0.43
19:I:1668:A:O2'	19:I:1674:G:N7	2.39	0.43
19:I:1818:U:H2'	23:K:156:ARG:HB2	2.00	0.43
20:i:46:ASP:OD1	20:i:46:ASP:N	2.41	0.43
38:r:79:ILE:HB	38:r:105:PHE:CE2	2.52	0.43
40:t:50:ARG:HG2	40:t:66:TYR:HE1	1.83	0.43
1:C:21:G:H2'	1:C:22:G:H8	1.77	0.43
1:C:218:U:H2'	1:C:219:C:C6	2.53	0.43
1:C:508:C:H2'	1:C:509:G:H8	1.82	0.43
1:C:701:U:H2'	1:C:702:A:C8	2.53	0.43
2:1:32:ARG:HD2	2:1:57:HIS:CG	2.53	0.43
4:2:55:GLN:HG3	4:2:76:LYS:HD2	2.00	0.43
13:F:147:GLU:H	13:F:147:GLU:CD	2.26	0.43
19:I:403:U:H4'	19:I:404:A:H8	1.83	0.43
19:I:760:G:H2'	19:I:761:A:O4'	2.18	0.43
19:I:925:A:H2'	19:I:926:G:C8	2.53	0.43
19:I:1561:C:H2'	19:I:1562:U:C6	2.53	0.43

Continued on next page...

For Manuscript Review

Continued from previous page...

Atom-1	Atom-2	Interatomic distance (Å)	Clash overlap (Å)
19:I:1709:U:H2'	19:I:1710:G:H8	1.83	0.43
19:I:1710:G:H2'	19:I:1711:A:H8	1.83	0.43
19:I:2669:G:H2'	19:I:2670:A:H8	1.82	0.43
19:I:2838:G:C4	19:I:2839:G:C8	3.06	0.43
21:J:66:A:H1'	21:J:68:C:N4	2.33	0.43
23:K:21:ASN:OD1	23:K:21:ASN:N	2.51	0.43
30:n:77:SER:OG	30:n:86:GLN:OE1	2.21	0.43
31:O:52:PHE:CD2	31:O:69:ARG:HG2	2.53	0.43
1:C:176:C:H3'	1:C:177:G:C2	2.53	0.43
1:C:260:G:O2'	1:C:261:C:H3'	2.18	0.43
1:C:399:U:H3'	1:C:400:G:H5'	2.00	0.43
1:C:745:U:H2'	1:C:746:G:O4'	2.17	0.43
1:C:1405:C:H2'	1:C:1406:C:C6	2.54	0.43
19:I:20:C:H2'	19:I:21:A:H8	1.83	0.43
19:I:299:A:N3	19:I:319:G:O2'	2.42	0.43
19:I:345:A:N3	19:I:347:A:N6	2.66	0.43
19:I:723:C:H2'	19:I:724:U:C6	2.53	0.43
19:I:807:U:H2'	19:I:808:G:H8	1.83	0.43
19:I:1315:C:H2'	19:I:1316:U:C6	2.54	0.43
19:I:2305:U:O4	29:N:151:GLY:N	2.51	0.43
19:I:2411:A:H2'	19:I:2412:A:C8	2.52	0.43
19:I:2543:G:H2'	19:I:2544:G:C8	2.54	0.43
19:I:2710:C:H2'	19:I:2711:A:C8	2.53	0.43
21:J:57:A:H2'	21:J:58:A:H8	1.83	0.43
50:y:11:ARG:HD2	50:y:58:VAL:HG22	1.99	0.43
1:C:615:A:H2'	1:C:616:A:C8	2.53	0.43
1:C:625:C:H5"	1:C:626:U:H6	1.83	0.43
1:C:739:U:H2'	1:C:740:G:C8	2.52	0.43
1:C:1324:U:H2'	1:C:1325:G:O4'	2.18	0.43
1:C:1395:G:H2'	1:C:1396:C:O4'	2.18	0.43
8:c:7:ARG:HD3	8:c:25:VAL:HB	2.00	0.43
9:D:163:VAL:HG21	9:D:173:ILE:HD11	2.01	0.43
9:D:185:ALA:H	9:D:196:VAL:HG21	1.84	0.43
13:F:166:GLU:H	13:F:166:GLU:HG2	1.60	0.43
19:I:458:G:O2'	19:I:469:G:O6	2.34	0.43
19:I:513:A:H5'	19:I:1216:G:HO2'	1.83	0.43
19:I:580:U:H2'	19:I:581:C:H6	1.83	0.43
19:I:963:U:H2'	19:I:964:C:C6	2.52	0.43
19:I:1153:C:H5"	51:Y:62:ILE:HD13	1.99	0.43
19:I:1239:G:H2'	19:I:1240:U:O4'	2.18	0.43
19:I:1791:A:H4'	23:K:205:LEU:HB2	1.99	0.43

Continued on next page...

For Manuscript Review

Continued from previous page...

Atom-1	Atom-2	Interatomic distance (Å)	Clash overlap (Å)
19:I:1865:U:H1'	19:I:1866:A:O5'	2.17	0.43
19:I:1873:G:H2'	19:I:1874:C:C6	2.54	0.43
19:I:1942:C:OP2	19:I:1943:U:O2'	2.31	0.43
19:I:2241:A:H2'	19:I:2242:G:H8	1.82	0.43
19:I:2322:A:N6	19:I:2333:A:H61	2.14	0.43
21:J:23:G:H2'	21:J:24:G:C5	2.53	0.43
32:o:29:SER:HB2	32:o:59:LEU:HB2	1.99	0.43
33:P:4:ILE:HD11	33:P:37:VAL:HG22	2.00	0.43
35:Q:60:PHE:O	35:Q:64:PHE:HB3	2.18	0.43
42:u:88:GLY:O	42:u:89:LEU:HB2	2.18	0.43
1:C:248:G:H5"	50:y:71:LYS:HD2	1.99	0.43
1:C:553:A:H4'	1:C:554:A:H3'	1.99	0.43
1:C:942:C:H2'	1:C:943:A:H8	1.83	0.43
1:C:1288:A:H2'	1:C:1289:U:C6	2.54	0.43
17:H:81:ASN:HD21	17:H:84:VAL:HG23	1.84	0.43
19:I:289:G:C6	19:I:352:A:C6	3.07	0.43
19:I:971:G:H2'	19:I:972:A:O4'	2.18	0.43
19:I:1182:G:H2'	19:I:1183:U:O4'	2.18	0.43
19:I:2065:C:H2'	19:I:2066:C:H6	1.82	0.43
19:I:2139:U:C4	19:I:2152:G:O6	2.71	0.43
19:I:2228:G:H2'	19:I:2229:U:H6	1.82	0.43
19:I:2632:A:H2'	19:I:2633:G:H8	1.84	0.43
41:T:69:ARG:HG2	41:T:69:ARG:NH1	2.34	0.43
51:Y:79:PHE:O	51:Y:83:LEU:HG	2.19	0.43
1:C:3:A:H2'	1:C:4:C:C6	2.54	0.43
1:C:139:G:H1	1:C:140:G:N2	2.15	0.43
1:C:166:A:N6	1:C:168:A:O2'	2.52	0.43
1:C:484:A:H2'	1:C:485:A:C8	2.54	0.43
1:C:489:A:HO2'	1:C:490:A:P	2.40	0.43
1:C:819:A:H2'	1:C:820:C:C6	2.53	0.43
1:C:1175:G:O2'	1:C:1176:G:N7	2.45	0.43
1:C:1317:G:H2'	1:C:1318:A:H8	1.81	0.43
1:C:1473:C:H2'	1:C:1474:A:H8	1.84	0.43
13:F:75:TYR:CD1	13:F:93:LEU:HB3	2.53	0.43
19:I:132:G:H2'	19:I:133:U:H6	1.82	0.43
19:I:515:A:H1'	19:I:581:C:H1'	1.99	0.43
19:I:1196:C:C2	19:I:1197:G:C8	3.07	0.43
19:I:1252:G:N2	51:Y:33:ARG:HB3	2.34	0.43
19:I:1754:A:C8	49:X:94:LYS:HE2	2.54	0.43
21:J:79:G:H2'	21:J:80:U:O4'	2.17	0.43
45:V:36:THR:OG1	45:V:37:THR:N	2.52	0.43

Continued on next page...

For Manuscript Review

Continued from previous page...

Atom-1	Atom-2	Interatomic distance (Å)	Clash overlap (Å)
1:C:207:A:H3'	1:C:208:C:C5	2.54	0.43
1:C:702:A:H2'	1:C:703:G:C8	2.51	0.43
1:C:1126:C:H2'	1:C:1127:A:H8	1.84	0.43
1:C:1323:G:C5	1:C:1324:U:C5	3.07	0.43
11:E:124:LEU:HD21	11:E:130:PHE:HB3	2.00	0.43
13:F:157:ALA:O	13:F:160:GLU:HG3	2.18	0.43
16:g:19:LEU:HD12	16:g:19:LEU:HA	1.91	0.43
19:I:197:A:H2	19:I:2434:A:H62	1.67	0.43
19:I:222:A:N6	19:I:232:G:H1'	2.34	0.43
19:I:848:C:H2'	19:I:849:A:C8	2.53	0.43
19:I:994:C:OP2	51:Y:54:LYS:NZ	2.39	0.43
19:I:1125:G:H3'	19:I:1126:A:C8	2.54	0.43
19:I:2038:G:H2'	19:I:2039:U:O4'	2.19	0.43
19:I:2247:A:H2'	19:I:2248:C:H6	1.84	0.43
19:I:2311:A:O2'	19:I:2312:U:H6	2.01	0.43
19:I:2641:G:H2'	19:I:2642:G:C8	2.54	0.43
19:I:2737:G:H2'	19:I:2738:A:C8	2.53	0.43
19:I:2848:G:H1'	19:I:2868:A:N6	2.34	0.43
34:p:46:MET:HA	34:p:49:ARG:HG2	2.00	0.43
38:r:87:LYS:HG3	38:r:114:THR:HA	2.00	0.43
43:U:33:LEU:HG	43:U:34:LYS:H	1.83	0.43
43:U:64:TRP:HB2	43:U:104:GLU:HB2	2.01	0.43
1:C:19:A:H2'	1:C:20:U:H6	1.82	0.43
1:C:73:A:N6	1:C:90:G:O2'	2.52	0.43
1:C:262:C:H2'	1:C:263:U:C6	2.54	0.43
1:C:337:U:H2'	1:C:339:C:N3	2.34	0.43
1:C:926:C:H2'	1:C:927:G:C8	2.53	0.43
1:C:1213:A:H2'	1:C:1214:G:H8	1.83	0.43
1:C:1450:G:H3'	1:C:1451:G:H8	1.84	0.43
2:1:9:PRO:HG2	2:1:41:PHE:CE2	2.53	0.43
14:f:26:LYS:HD2	19:I:190:A:OP2	2.19	0.43
19:I:548:G:H2'	19:I:548:G:N3	2.33	0.43
19:I:645:C:H2'	19:I:647:G:N7	2.33	0.43
19:I:1145:C:N3	19:I:1146:C:C5	2.87	0.43
19:I:2370:G:H2'	19:I:2371:G:O4'	2.18	0.43
19:I:2545:G:H2'	19:I:2546:U:O4'	2.19	0.43
19:I:2677:G:H2'	19:I:2678:C:C6	2.54	0.43
27:M:58:LYS:HG2	27:M:71:GLY:HA2	2.00	0.43
31:O:89:LEU:HB2	31:O:129:THR:HB	2.01	0.43
34:p:123:ARG:NH1	34:p:124:ARG:O	2.49	0.43
41:T:48:ARG:HE	41:T:48:ARG:HB2	1.72	0.43

Continued on next page...

For Manuscript Review

Continued from previous page...

Atom-1	Atom-2	Interatomic distance (Å)	Clash overlap (Å)
52:z:26:ILE:HA	52:z:29:LEU:HB2	2.00	0.43
1:C:17:U:H2'	1:C:18:C:H6	1.83	0.43
1:C:731:A:H2'	1:C:732:C:H6	1.84	0.43
1:C:1078:G:OP2	1:C:1078:G:H8	2.01	0.43
1:C:1133:G:H4'	1:C:1134:G:H5'	2.00	0.43
1:C:1153:U:C5	1:C:1155:C:H1'	2.53	0.43
9:D:49:MET:HG3	9:D:50:PHE:N	2.32	0.43
10:d:62:THR:HG22	10:d:71:LYS:HD3	2.01	0.43
19:I:266:G:H22	19:I:425:G:H22	1.66	0.43
19:I:2334:U:OP2	47:W:9:ARG:HA	2.19	0.43
19:I:2362:C:OP1	26:l:40:ARG:NE	2.51	0.43
23:K:160:THR:HA	23:K:177:ARG:HD3	2.01	0.43
34:p:31:ASN:HD21	34:p:66:THR:HG23	1.84	0.43
39:S:23:LYS:HD3	39:S:23:LYS:HA	1.84	0.43
1:C:64:G:H4'	1:C:65:A:H5'	2.01	0.43
1:C:875:G:P	40:t:9:ARG:HH22	2.42	0.43
1:C:1119:U:H4'	36:q:7:ARG:NH1	2.34	0.43
1:C:1146:A:O4'	36:q:41:PRO:HB2	2.19	0.43
1:C:1227:G:H2'	1:C:1228:C:C6	2.53	0.43
11:E:42:TYR:OH	11:E:46:GLU:OE1	2.28	0.43
11:E:168:TYR:OH	15:G:55:GLU:OE2	2.19	0.43
14:f:31:PRO:HB2	14:f:33:LEU:HD13	2.01	0.43
15:G:29:ARG:HG2	15:G:29:ARG:O	2.19	0.43
19:I:578:G:OP1	19:I:1255:U:O2'	2.37	0.43
19:I:937:C:OP1	26:l:52:LYS:HD2	2.18	0.43
19:I:1186:G:H2'	19:I:1187:G:O4'	2.18	0.43
19:I:2030:6MZ:C2	19:I:2499:C:H5"	2.48	0.43
19:I:2651:C:H2'	19:I:2652:C:H6	1.84	0.43
19:I:2669:G:H2'	19:I:2670:A:C8	2.54	0.43
21:J:33:G:H2'	21:J:34:A:C8	2.54	0.43
42:u:88:GLY:C	42:u:90:ARG:H	2.26	0.43
46:w:14:GLU:HG2	46:w:15:PHE:CD1	2.54	0.43
1:C:52:C:H2'	1:C:53:A:C8	2.54	0.42
1:C:98:G:C2	1:C:99:G:C5	3.07	0.42
1:C:194:G:O2'	1:C:195:G:H5'	2.19	0.42
1:C:402:G:O3'	13:F:23:SER:OG	2.36	0.42
1:C:1163:A:H2'	1:C:1164:A:C8	2.54	0.42
1:C:1348:U:H2'	1:C:1349:G:C8	2.54	0.42
5:4:18:ARG:HA	5:4:21:ALA:HB3	2.01	0.42
5:4:73:LYS:HB3	5:4:106:VAL:HB	2.01	0.42
8:c:48:PRO:HB3	8:c:55:PRO:HA	2.01	0.42

Continued on next page...

For Manuscript Review

Continued from previous page...

Atom-1	Atom-2	Interatomic distance (Å)	Clash overlap (Å)
13:F:125:VAL:HB	13:F:143:VAL:HG22	2.00	0.42
19:I:363:G:H2'	19:I:364:C:C6	2.54	0.42
19:I:917:A:H2	21:J:80:U:H4'	1.80	0.42
19:I:1153:C:H2'	19:I:1154:G:O4'	2.19	0.42
19:I:1862:G:O6	19:I:1880:U:O2	2.36	0.42
19:I:2530:A:P	19:I:2535:G:H22	2.42	0.42
19:I:2742:G:H3'	19:I:2755:C:N4	2.34	0.42
23:K:53:HIS:HA	23:K:217:ARG:HB2	2.01	0.42
40:t:79:VAL:O	40:t:103:ASP:HB2	2.19	0.42
43:U:17:ASN:O	43:U:38:ARG:NH1	2.52	0.42
1:C:50:A:O2'	1:C:354:G:N2	2.52	0.42
1:C:172:C:O2'	1:C:173:A:H5"	2.19	0.42
1:C:306:C:C2	1:C:307:A:C8	3.07	0.42
1:C:450:C:H2'	1:C:451:A:C8	2.54	0.42
1:C:1020:G:C2	1:C:1030:A:C5	3.07	0.42
1:C:1118:G:O2'	1:C:1121:A:N6	2.52	0.42
1:C:1472:U:H2'	1:C:1473:C:H6	1.84	0.42
8:c:83:VAL:HG13	8:c:95:PHE:O	2.19	0.42
10:d:58:SER:O	10:d:73:LYS:NZ	2.52	0.42
19:I:181:A:H2'	19:I:182:A:C8	2.54	0.42
19:I:419:U:H2'	19:I:420:C:H6	1.84	0.42
19:I:1183:U:H2'	19:I:1184:U:C6	2.54	0.42
19:I:1680:U:H2'	19:I:1681:G:O4'	2.19	0.42
19:I:1831:G:H2'	19:I:1832:C:C6	2.55	0.42
19:I:2195:U:H2'	19:I:2196:C:H6	1.84	0.42
19:I:2204:G:H4'	23:K:150:LYS:HG3	2.01	0.42
19:I:2287:A:O2'	19:I:2288:A:O5'	2.34	0.42
27:M:5:LEU:HD12	27:M:8:ALA:HB3	2.00	0.42
32:o:10:MET:HG2	32:o:27:MET:SD	2.59	0.42
51:Y:110:VAL:HA	51:Y:113:ALA:HB3	2.02	0.42
1:C:89:G:H5'	1:C:90:G:OP2	2.20	0.42
1:C:288:U:H2'	1:C:289:C:H6	1.83	0.42
1:C:593:U:H2'	1:C:594:G:C8	2.50	0.42
1:C:835:C:N4	1:C:838:G:O6	2.52	0.42
1:C:1112:U:H3	1:C:1150:G:H1	1.67	0.42
1:C:1173:A:H2'	1:C:1174:A:O4'	2.19	0.42
1:C:1327:A:H2'	1:C:1328:G:O4'	2.19	0.42
2:1:6:LYS:HB2	2:1:6:LYS:HE2	1.73	0.42
11:E:47:LEU:HD22	11:E:76:VAL:HG22	2.01	0.42
13:F:82:LEU:HB2	13:F:89:ASN:HD22	1.83	0.42
18:h:3:LYS:HG2	18:h:40:ASP:OD2	2.20	0.42

Continued on next page...

For Manuscript Review

Continued from previous page...

Atom-1	Atom-2	Interatomic distance (Å)	Clash overlap (Å)
19:I:145:C:H2'	19:I:146:A:C8	2.53	0.42
19:I:584:C:N4	19:I:585:G:O6	2.52	0.42
19:I:713:G:H2'	19:I:714:U:C6	2.54	0.42
19:I:917:A:H5"	19:I:2268:A:H61	1.83	0.42
19:I:1327:A:H2'	19:I:1328:A:O4'	2.18	0.42
19:I:1412:U:H3'	19:I:1413:A:H5"	2.00	0.42
19:I:1653:G:C6	45:V:9:GLN:HB3	2.55	0.42
19:I:2087:G:H2'	19:I:2088:A:H8	1.85	0.42
19:I:2133:G:H2'	19:I:2157:G:H1	1.85	0.42
19:I:2523:G:H2'	19:I:2523:G:N3	2.33	0.42
19:I:2532:G:H2'	19:I:2533:U:C6	2.55	0.42
19:I:2637:U:H2'	19:I:2638:G:O4'	2.20	0.42
19:I:2830:C:OP2	25:L:59:ARG:NH1	2.52	0.42
23:K:114:ASP:OD1	23:K:114:ASP:N	2.46	0.42
30:n:26:PHE:CE2	30:n:120:LEU:HD21	2.52	0.42
31:O:125:CYS:C	31:O:127:THR:H	2.27	0.42
50:y:23:VAL:HG12	50:y:25:ILE:HG13	2.00	0.42
1:C:246:U:H2'	1:C:247:U:C5	2.54	0.42
1:C:352:U:H2'	1:C:353:G:C8	2.51	0.42
1:C:453:U:H2'	1:C:454:A:H8	1.81	0.42
1:C:607:C:H2'	1:C:608:C:C6	2.54	0.42
1:C:1320:U:H2'	1:C:1321:G:C8	2.47	0.42
1:C:1409:G:C6	1:C:1480:G:C6	3.08	0.42
3:Z:61:ALA:HA	3:Z:99:THR:HG23	2.01	0.42
9:D:139:ARG:HG3	9:D:140:GLU:N	2.34	0.42
9:D:219:ALA:HA	9:D:222:ARG:HG2	2.00	0.42
13:F:69:GLU:O	13:F:73:ARG:HB3	2.20	0.42
15:G:146:ASN:C	15:G:146:ASN:OD1	2.62	0.42
19:I:320:A:N3	27:M:163:ASN:ND2	2.68	0.42
19:I:764:A:O2'	19:I:765:C:H5'	2.20	0.42
19:I:1011:G:C6	19:I:1151:A:C6	3.08	0.42
19:I:1295:C:H2'	19:I:1296:G:H8	1.85	0.42
19:I:1889:A:H2'	19:I:1890:A:C8	2.54	0.42
19:I:2235:G:H2'	19:I:2236:U:C6	2.55	0.42
19:I:2445:2MG:P	27:M:69:ARG:HH22	2.42	0.42
19:I:2472:G:H3'	19:I:2473:U:C5'	2.50	0.42
19:I:2478:A:H1'	19:I:2529:G:C8	2.53	0.42
19:I:2634:A:H2'	19:I:2635:A:C8	2.54	0.42
19:I:2636:C:H2'	19:I:2637:U:H6	1.85	0.42
23:K:120:VAL:HA	23:K:131:PRO:HD3	2.00	0.42
23:K:246:THR:HG23	23:K:250:VAL:O	2.19	0.42

Continued on next page...

For Manuscript Review

Continued from previous page...

Atom-1	Atom-2	Interatomic distance (Å)	Clash overlap (Å)
32:o:36:ILE:HG23	32:o:103:VAL:HG11	2.02	0.42
48:x:21:VAL:O	48:x:33:ILE:HB	2.20	0.42
1:C:462:A:H2'	1:C:463:C:O4'	2.18	0.42
1:C:515:G:O2'	1:C:530:C:O2'	2.36	0.42
1:C:1312:A:H8	1:C:1312:A:O5'	2.02	0.42
1:C:1512:A:H2'	1:C:1513:A:C8	2.54	0.42
14:f:3:ARG:O	14:f:12:PRO:HD3	2.18	0.42
19:I:216:A:C8	19:I:432:A:C6	3.07	0.42
19:I:402:A:H2'	19:I:403:U:O4'	2.20	0.42
19:I:576:U:H2'	19:I:577:G:H8	1.78	0.42
19:I:1000:A:H2'	19:I:1001:A:C8	2.53	0.42
19:I:1435:G:H2'	19:I:1436:G:C8	2.54	0.42
19:I:1446:C:H2'	19:I:1447:C:C6	2.54	0.42
19:I:1710:G:H4'	19:I:2858:C:O2	2.19	0.42
19:I:1998:A:H2'	19:I:1999:C:H6	1.84	0.42
19:I:2163:A:H5"	19:I:2170:A:O2'	2.20	0.42
19:I:2563:U:H2'	19:I:2565:A:OP2	2.20	0.42
19:I:2688:G:H1'	19:I:2721:A:H61	1.85	0.42
19:I:2728:U:O2'	19:I:2729:G:H8	1.94	0.42
19:I:2756:U:H1'	19:I:2757:A:H5"	2.00	0.42
19:I:2846:G:H2'	19:I:2847:U:C6	2.55	0.42
19:I:2887:A:N3	20:i:27:SER:OG	2.46	0.42
21:J:11:C:H2'	21:J:12:C:O4'	2.19	0.42
21:J:77:U:H2'	21:J:78:A:C8	2.55	0.42
34:p:97:GLU:H	34:p:97:GLU:CD	2.28	0.42
39:S:2:ILE:HD13	39:S:6:THR:HG21	2.02	0.42
43:U:136:MET:HE2	43:U:136:MET:HB3	1.93	0.42
44:v:32:SER:HA	44:v:42:TRP:HB2	2.01	0.42
48:x:46:LYS:NZ	48:x:47:GLU:HB3	2.34	0.42
1:C:6:G:C4	15:G:124:LEU:HD11	2.55	0.42
1:C:17:U:O2'	1:C:1073:G:H1'	2.18	0.42
1:C:268:A:H1'	1:C:269:G:C8	2.55	0.42
1:C:369:U:H2'	1:C:370:G:H8	1.85	0.42
1:C:1005:C:H2'	1:C:1006:A:H8	1.84	0.42
4:2:51:PHE:CD1	4:2:51:PHE:C	2.98	0.42
11:E:33:LEU:HD21	44:v:93:ILE:HG12	2.02	0.42
11:E:89:LYS:HB3	11:E:89:LYS:HE3	1.74	0.42
19:I:347:A:H2'	19:I:348:A:H8	1.85	0.42
19:I:1989:G:H2'	19:I:1990:C:O4'	2.20	0.42
19:I:2030:6MZ:N3	19:I:2499:C:H5"	2.34	0.42
19:I:2287:A:C6	19:I:2289:G:C8	3.07	0.42

Continued on next page...

For Manuscript Review

Continued from previous page...

Atom-1	Atom-2	Interatomic distance (Å)	Clash overlap (Å)
19:I:2854:G:H2'	19:I:2855:C:C6	2.55	0.42
34:p:91:ASP:O	34:p:93:SER:N	2.52	0.42
44:v:87:ALA:HB1	44:v:92:GLU:HB2	2.01	0.42
47:W:2:ASP:OD1	47:W:2:ASP:N	2.53	0.42
48:x:21:VAL:O	48:x:21:VAL:HG23	2.19	0.42
50:y:68:SER:HB3	50:y:71:LYS:HD3	2.02	0.42
1:C:369:U:O2'	48:x:7:ALA:O	2.37	0.42
1:C:645:C:H2'	1:C:646:U:C6	2.55	0.42
1:C:773:C:H2'	1:C:774:A:O4'	2.19	0.42
8:c:93:VAL:HG22	8:c:94:ARG:H	1.85	0.42
14:f:57:ARG:HB3	19:I:372:G:N7	2.33	0.42
16:g:47:ARG:NH1	19:I:60:G:H5"	2.35	0.42
19:I:35:G:H1'	19:I:454:A:C4	2.55	0.42
19:I:106:C:H2'	19:I:107:G:C8	2.53	0.42
19:I:224:U:O4	19:I:419:U:O2'	2.37	0.42
19:I:680:C:H2'	19:I:681:G:C8	2.54	0.42
19:I:948:C:H2'	19:I:949:G:C8	2.54	0.42
19:I:1196:C:H2'	19:I:1197:G:H8	1.85	0.42
19:I:2804:U:H2'	19:I:2805:C:H6	1.85	0.42
21:J:24:G:H1'	21:J:27:C:H42	1.85	0.42
21:J:32:U:H2'	21:J:33:G:O4'	2.19	0.42
21:J:84:G:N2	21:J:85:G:H1'	2.34	0.42
29:N:81:GLN:H	29:N:81:GLN:HG2	1.66	0.42
34:p:57:MET:HE3	34:p:57:MET:HB3	1.91	0.42
40:t:14:ARG:HD3	40:t:14:ARG:HA	1.84	0.42
40:t:35:THR:N	40:t:54:ARG:O	2.47	0.42
41:T:75:ALA:HB2	41:T:105:ILE:HG21	2.01	0.42
51:Y:52:GLN:HA	51:Y:55:ARG:HB2	2.01	0.42
1:C:73:A:H3'	1:C:74:A:H8	1.85	0.42
1:C:229:U:H2'	1:C:230:A:H8	1.84	0.42
1:C:240:A:C2	1:C:276:A:C5	3.07	0.42
1:C:450:C:H2'	1:C:451:A:O4'	2.20	0.42
1:C:996:A:H2'	1:C:997:G:O4'	2.20	0.42
1:C:1090:C:H2'	1:C:1091:C:C6	2.54	0.42
6:3:59:LYS:H	6:3:59:LYS:HG2	1.61	0.42
8:c:12:ILE:O	8:c:70:VAL:HA	2.19	0.42
10:d:50:MET:HE1	10:d:56:PHE:HB2	2.01	0.42
17:H:18:VAL:O	17:H:22:ILE:HG13	2.19	0.42
19:I:1442:U:H2'	19:I:1443:U:H6	1.84	0.42
19:I:1561:C:H2'	19:I:1562:U:H6	1.84	0.42
19:I:1783:A:H5'	19:I:2608:G:H4'	2.02	0.42

Continued on next page...

For Manuscript Review

Continued from previous page...

Atom-1	Atom-2	Interatomic distance (Å)	Clash overlap (Å)
19:I:2078:C:H2'	19:I:2079:U:C6	2.54	0.42
19:I:2389:G:H5"	19:I:2390:U:O4'	2.19	0.42
19:I:2484:G:OP1	43:U:44:ARG:HD2	2.20	0.42
19:I:2804:U:H2'	19:I:2805:C:C6	2.54	0.42
39:S:108:ARG:NE	49:X:34:GLU:OE2	2.53	0.42
1:C:73:A:H8	1:C:90:G:N2	2.10	0.42
1:C:105:G:H1	1:C:324:C:N4	2.16	0.42
1:C:170:C:H1'	1:C:171:G:H5'	2.02	0.42
1:C:192:G:H2'	1:C:193:U:H6	1.83	0.42
1:C:678:U:H1'	38:r:40:ASN:HB3	2.02	0.42
1:C:973:C:H2'	44:v:59:ARG:HH21	1.85	0.42
1:C:1230:A:H2'	1:C:1231:C:C6	2.54	0.42
1:C:1466:A:H2'	1:C:1467:G:C8	2.54	0.42
3:Z:39:LEU:HG	3:Z:53:PHE:CD1	2.55	0.42
13:F:57:GLU:HG2	13:F:199:LEU:HB2	2.01	0.42
17:H:90:MET:HE3	17:H:90:MET:HB2	1.71	0.42
19:I:44:A:H2'	19:I:45:G:O4'	2.20	0.42
19:I:414:C:H4'	19:I:1879:C:O2	2.20	0.42
19:I:689:A:H2'	19:I:690:G:C8	2.54	0.42
19:I:1637:A:H4'	19:I:2711:A:O2'	2.20	0.42
27:M:170:ARG:NH2	27:M:179:SER:OG	2.50	0.42
44:v:96:LEU:HD12	44:v:96:LEU:HA	1.93	0.42
50:y:46:VAL:HG13	50:y:74:THR:HA	2.01	0.42
1:C:94:C:O2'	1:C:95:A:H8	2.01	0.42
1:C:739:U:H5'	1:C:829:G:H21	1.85	0.42
1:C:1054:U:P	44:v:85:ARG:HH22	2.43	0.42
1:C:1121:A:H1'	1:C:1274:A:C6	2.54	0.42
1:C:1494:A:H5"	1:C:1502:G:H5"	2.01	0.42
7:b:10:VAL:HG21	7:b:42:GLU:HB3	2.02	0.42
14:f:2:SER:N	19:I:1364:G:H3'	2.35	0.42
19:I:27:G:N2	19:I:512:G:H1'	2.35	0.42
19:I:30:G:H2'	19:I:31:C:C6	2.55	0.42
19:I:371:A:H8	19:I:371:A:OP2	2.03	0.42
19:I:592:A:C2	26:l:4:ILE:HD11	2.54	0.42
19:I:634:C:H2'	19:I:635:C:C6	2.55	0.42
19:I:753:A:H2'	19:I:754:U:H6	1.85	0.42
19:I:954:G:H5"	43:U:13:HIS:CG	2.55	0.42
19:I:1064:C:H2'	19:I:1065:U:C4	2.54	0.42
19:I:1278:C:H2'	19:I:1279:G:C8	2.55	0.42
19:I:1588:G:H2'	19:I:1589:U:H6	1.84	0.42
19:I:2300:C:H2'	19:I:2301:C:H6	1.85	0.42

Continued on next page...

For Manuscript Review

Continued from previous page...

Atom-1	Atom-2	Interatomic distance (Å)	Clash overlap (Å)
19:I:2605:PSU:H2’	19:I:2606:C:C6	2.54	0.42
24:k:41:ARG:HB3	24:k:41:ARG:HH11	1.85	0.42
39:S:63:VAL:HG12	39:S:107:LEU:HD11	2.02	0.42
1:C:392:U:H2’	1:C:393:G:C8	2.55	0.41
1:C:427:G:H2’	1:C:428:C:H6	1.84	0.41
1:C:1021:C:C2	1:C:1022:C:C5	3.07	0.41
1:C:1354:A:OP2	44:v:75:ARG:NH2	2.53	0.41
1:C:1423:C:H2’	1:C:1424:C:H6	1.85	0.41
4:2:49:LYS:O	4:2:49:LYS:HD3	2.20	0.41
19:I:135:U:H2’	19:I:136:G:O4’	2.20	0.41
19:I:479:A:N3	19:I:481:G:H5”	2.35	0.41
19:I:716:A:H3’	19:I:717:C:H5”	2.02	0.41
19:I:1250:G:OP2	41:T:21:ARG:NH1	2.53	0.41
19:I:1550:C:H2’	19:I:1551:A:C8	2.55	0.41
19:I:1938:A:C6	19:I:2590:A:H1’	2.55	0.41
19:I:2192:U:H2’	19:I:2193:G:H8	1.84	0.41
19:I:2247:A:H2’	19:I:2248:C:C6	2.55	0.41
19:I:2720:U:C2	19:I:2721:A:C8	3.08	0.41
19:I:2732:G:H3’	19:I:2733:A:O4’	2.19	0.41
25:L:61:THR:OG1	25:L:64:GLU:HG3	2.20	0.41
30:n:150:ALA:HA	38:r:61:PHE:HB3	2.02	0.41
34:p:22:LYS:CB	34:p:61:LEU:HB3	2.44	0.41
40:t:116:LYS:HE3	40:t:116:LYS:HB2	1.62	0.41
49:X:18:PRO:HG3	49:X:84:ILE:O	2.19	0.41
1:C:303:A:H2’	1:C:304:G:C8	2.51	0.41
1:C:488:G:O2’	1:C:490:A:H1’	2.19	0.41
1:C:778:A:H2’	1:C:779:G:H8	1.84	0.41
11:E:110:GLU:HG2	11:E:140:ASN:HB3	2.02	0.41
19:I:32:C:H2’	19:I:33:C:C6	2.55	0.41
19:I:240:C:OP2	19:I:241:A:O2’	2.37	0.41
19:I:956:G:O2’	43:U:82:MET:HE1	2.20	0.41
19:I:1044:C:O2	19:I:1048:A:O2’	2.38	0.41
19:I:1282:U:H2’	19:I:1283:G:O4’	2.19	0.41
19:I:1570:A:H2’	19:I:1570:A:N3	2.36	0.41
33:P:4:ILE:O	33:P:4:ILE:HD12	2.20	0.41
33:P:40:THR:OG1	33:P:41:LYS:N	2.50	0.41
34:p:124:ARG:HG3	34:p:124:ARG:NH1	2.35	0.41
36:q:14:ASP:OD1	36:q:14:ASP:N	2.52	0.41
48:x:7:ALA:O	48:x:8:ARG:HB2	2.19	0.41
1:C:26:A:H61	1:C:552:G:H1’	1.84	0.41
1:C:31:G:O2’	1:C:32:A:O5’	2.32	0.41

Continued on next page...

For Manuscript Review

Continued from previous page...

Atom-1	Atom-2	Interatomic distance (Å)	Clash overlap (Å)
1:C:82:U:H1'	1:C:83:G:O4'	2.20	0.41
1:C:146:A:N6	1:C:163:C:C2	2.88	0.41
1:C:257:A:H2'	1:C:258:A:C8	2.54	0.41
1:C:689:A:H2'	1:C:690:A:C8	2.55	0.41
1:C:1075:G:C5	1:C:1076:A:C8	3.08	0.41
2:1:36:ARG:HD2	2:1:52:HIS:O	2.19	0.41
9:D:41:ILE:HG21	9:D:201:PRO:HB2	2.03	0.41
11:E:155:GLY:O	11:E:196:ILE:HG12	2.21	0.41
17:H:21:MET:HE3	17:H:25:TYR:OH	2.21	0.41
19:I:153:U:H2'	19:I:154:U:C6	2.56	0.41
19:I:565:C:H2'	19:I:566:U:C6	2.55	0.41
19:I:566:U:H2'	19:I:567:U:C6	2.55	0.41
19:I:1140:C:H5'	37:R:26:GLY:HA3	2.01	0.41
19:I:1666:G:H4'	39:S:6:THR:HG23	2.01	0.41
19:I:2193:G:H2'	19:I:2194:U:H6	1.85	0.41
19:I:2506[A]:U:OP2	19:I:2576:G:N1	2.39	0.41
19:I:2649:C:H2'	19:I:2650:U:H6	1.85	0.41
19:I:2773:C:H5"	25:L:169:ARG:HG2	2.02	0.41
23:K:183:LYS:HE3	23:K:183:LYS:HB2	1.69	0.41
30:n:131:LYS:HA	30:n:131:LYS:HD3	1.75	0.41
31:O:10:VAL:HA	31:O:49:THR:HG22	2.01	0.41
36:q:71:LEU:HD23	36:q:71:LEU:HA	1.84	0.41
37:R:68:LYS:HB2	37:R:68:LYS:HE3	1.91	0.41
47:W:35:ILE:HG23	47:W:74:VAL:HG11	2.03	0.41
1:C:193:U:H2'	1:C:194:G:H8	1.84	0.41
1:C:431:U:H4'	1:C:432:U:OP1	2.20	0.41
1:C:833:U:H2'	1:C:834:C:C6	2.55	0.41
1:C:1144:U:H2'	1:C:1145:A:C8	2.55	0.41
1:C:1192:G:H2'	1:C:1193:U:C6	2.55	0.41
5:4:23:LEU:HD22	20:i:24:ALA:HB2	2.02	0.41
9:D:64:LYS:HA	9:D:64:LYS:HD2	1.90	0.41
19:I:428:A:H2'	19:I:429:A:C8	2.56	0.41
19:I:579:G:H2'	19:I:580:U:C6	2.55	0.41
19:I:713:G:H21	19:I:718:A:N6	2.18	0.41
19:I:1027:A:N1	19:I:1126:A:C5	2.88	0.41
19:I:1047:G:H4'	19:I:1048:A:H5'	2.02	0.41
19:I:1164:C:H2'	19:I:1165:A:H8	1.85	0.41
19:I:1234:U:C4	19:I:1235:G:C5	3.08	0.41
19:I:1713:A:H61	19:I:1745:A:H61	1.68	0.41
19:I:2531:A:C2	19:I:2659:G:H1'	2.55	0.41
23:K:176:LEU:HD23	23:K:176:LEU:HA	1.88	0.41

Continued on next page...

For Manuscript Review

Continued from previous page...

Atom-1	Atom-2	Interatomic distance (Å)	Clash overlap (Å)
26:l:30:ARG:HH21	41:T:62:PRO:HB2	1.86	0.41
31:O:92:VAL:HG22	31:O:160:LYS:HZ3	1.85	0.41
31:O:140:VAL:C	31:O:143:GLN:H	2.28	0.41
42:u:80:LEU:HD13	42:u:87:ARG:HB3	2.02	0.41
44:v:86:GLU:O	44:v:90:ARG:HG2	2.21	0.41
49:X:32:VAL:HG12	49:X:34:GLU:HG2	2.03	0.41
1:C:101:G:C6	1:C:102:G:H1'	2.55	0.41
1:C:107:G:O2'	1:C:348:G:H5'	2.20	0.41
1:C:494:G:H2'	1:C:495:C:H6	1.84	0.41
1:C:806:G:HO2'	1:C:807:U:P	2.43	0.41
1:C:1470:A:H2'	1:C:1471:U:H6	1.84	0.41
11:E:132:ARG:O	11:E:136:ARG:HG2	2.20	0.41
15:G:150:PRO:HA	15:G:153:VAL:HG12	2.03	0.41
19:I:307:G:N2	19:I:309:A:H3'	2.35	0.41
19:I:534:U:O2'	51:Y:49:ASP:OD2	2.36	0.41
19:I:652:U:P	19:I:654:A:H61	2.44	0.41
19:I:715:A:H2'	19:I:716:A:H8	1.85	0.41
19:I:828:U:H4'	19:I:831:G:C6	2.55	0.41
19:I:1178:C:H5"	19:I:1179:G:OP1	2.19	0.41
19:I:1366:A:H2'	19:I:1367:A:O4'	2.20	0.41
19:I:1576:U:O4	19:I:1577:C:N4	2.54	0.41
19:I:1799:G:C5	23:K:176:LEU:HD13	2.56	0.41
19:I:1902:C:H4'	23:K:242:LYS:O	2.20	0.41
19:I:2192:U:H2'	19:I:2193:G:C8	2.56	0.41
19:I:2845:U:H2'	19:I:2846:G:H8	1.86	0.41
28:m:33:HIS:O	28:m:35:GLN:NE2	2.51	0.41
29:N:41:GLY:HA2	29:N:85:ILE:HG23	2.01	0.41
48:x:13:LYS:HA	48:x:13:LYS:HD3	1.85	0.41
1:C:223:U:H4'	48:x:33:ILE:HD13	2.03	0.41
1:C:265:C:H2'	1:C:266:C:C6	2.56	0.41
1:C:409:A:C4	1:C:410:G:C8	3.09	0.41
1:C:762:A:H4'	1:C:1517:G:N2	2.36	0.41
1:C:1058:G:H21	1:C:1184:G:H2'	1.86	0.41
1:C:1430:U:O4	1:C:1431:A:N6	2.53	0.41
8:c:54:GLN:HB2	8:c:55:PRO:HD3	2.03	0.41
9:D:117:LEU:HA	9:D:120:GLN:HB2	2.02	0.41
13:F:73:ARG:HH11	13:F:77:LYS:HE2	1.86	0.41
19:I:39:G:H2'	19:I:40:U:C6	2.56	0.41
19:I:656:G:H2'	19:I:657:U:N1	2.36	0.41
19:I:813:U:H2'	19:I:814:C:H6	1.85	0.41
19:I:1070:A:H2'	19:I:1097:U:OP1	2.19	0.41

Continued on next page...

For Manuscript Review

Continued from previous page...

Atom-1	Atom-2	Interatomic distance (Å)	Clash overlap (Å)
19:I:1132:U:H2’	19:I:1133:A:C8	2.56	0.41
19:I:1292:G:H2’	19:I:1293:C:H6	1.86	0.41
19:I:1441:G:H2’	19:I:1442:U:H6	1.85	0.41
19:I:1637:A:H5’	19:I:1760:C:O2’	2.20	0.41
19:I:1839:G:C2	19:I:1840:G:C8	3.08	0.41
19:I:2087:G:H2’	19:I:2088:A:C8	2.55	0.41
31:O:30:ASN:C	31:O:30:ASN:OD1	2.63	0.41
31:O:138:LYS:O	31:O:141:ILE:HG22	2.21	0.41
33:P:26:ALA:HA	33:P:30:LEU:HB2	2.01	0.41
33:P:57:LYS:HE2	33:P:57:LYS:HB2	1.81	0.41
35:Q:42:PRO:HB2	35:Q:48:GLN:HE21	1.86	0.41
44:v:19:LYS:HE3	44:v:20:TYR:CD1	2.55	0.41
1:C:33:A:N3	40:t:29:GLN:NE2	2.65	0.41
1:C:518:G:H2’	1:C:519:C:C6	2.55	0.41
1:C:554:A:H4’	1:C:555:U:H5”	2.02	0.41
1:C:742:U:H2’	1:C:743:A:C8	2.56	0.41
1:C:831:G:H2’	1:C:832:A:H8	1.85	0.41
1:C:974:C:H2’	1:C:975:U:O4’	2.21	0.41
15:G:111:MET:HB3	15:G:111:MET:HE2	1.71	0.41
17:H:42:TRP:CD1	17:H:42:TRP:N	2.88	0.41
19:I:84:A:N6	19:I:98:G:O2’	2.52	0.41
19:I:607:U:O4	19:I:620:G:H5”	2.21	0.41
19:I:896:A:H5”	19:I:897:C:H5	1.84	0.41
19:I:1138:G:N3	37:R:108:MET:HE2	2.36	0.41
19:I:1636:U:H2’	19:I:1637:A:H8	1.84	0.41
19:I:1684:G:H2’	19:I:1685:C:C6	2.56	0.41
21:J:39:A:H1’	21:J:46:A:N6	2.34	0.41
23:K:35:GLU:N	23:K:62:TYR:O	2.52	0.41
23:K:79:GLU:OE2	23:K:101:ARG:NH1	2.42	0.41
1:C:264:A:H2’	1:C:265:C:C6	2.56	0.41
1:C:398:G:O2’	1:C:492:A:N1	2.53	0.41
1:C:495:C:O2	1:C:543:C:O2’	2.29	0.41
1:C:670:A:H2’	1:C:671:U:H6	1.86	0.41
1:C:872:A:H2’	1:C:873:C:C6	2.55	0.41
5:4:13:SER:O	5:4:17:VAL:HG23	2.20	0.41
10:d:26:PHE:CZ	10:d:42:LEU:HD12	2.56	0.41
12:e:53:CYS:SG	12:e:57:HIS:HA	2.61	0.41
19:I:362:A:H3’	19:I:363:G:C8	2.55	0.41
19:I:445:C:H2’	19:I:446:G:C8	2.56	0.41
19:I:1422:G:H22	19:I:1498:C:H1’	1.86	0.41
19:I:1428:C:C5	19:I:1569:A:H5”	2.55	0.41

Continued on next page...

For Manuscript Review

Continued from previous page...

Atom-1	Atom-2	Interatomic distance (Å)	Clash overlap (Å)
19:I:1638:C:H1’	19:I:2698:U:O2’	2.21	0.41
19:I:2262:U:H2’	19:I:2263:C:H6	1.86	0.41
21:J:66:A:N6	21:J:107:G:H2’	2.36	0.41
25:L:186:LEU:HD12	25:L:186:LEU:HA	1.97	0.41
30:n:44:TYR:O	30:n:48:GLU:HG2	2.20	0.41
41:T:76:GLU:N	41:T:76:GLU:OE1	2.54	0.41
45:V:54:LEU:HD21	45:V:65:LEU:HB3	2.01	0.41
1:C:138:G:H1	1:C:172:C:C5’	2.33	0.41
1:C:154:A:H2’	1:C:155:A:O4’	2.21	0.41
1:C:208:C:H2’	1:C:209:C:C5	2.56	0.41
1:C:312:G:O6	1:C:330:A:N6	2.54	0.41
1:C:460:A:H2’	1:C:462:A:N3	2.36	0.41
1:C:595:G:H2’	1:C:596:A:C8	2.55	0.41
1:C:664:G:H2’	1:C:665:G:H8	1.84	0.41
1:C:887:C:C2	1:C:888:G:C8	3.09	0.41
1:C:1049:A:C6	1:C:1200:G:C5	3.09	0.41
1:C:1075:G:N2	15:G:52:LYS:HZ1	2.18	0.41
1:C:1144:U:H2’	1:C:1145:A:H8	1.86	0.41
1:C:1157:G:H22	1:C:1168:G:N2	2.19	0.41
1:C:1340:A:N1	1:C:1368:A:H5”	2.36	0.41
1:C:1396:C:H2’	1:C:1397:C:O4’	2.21	0.41
5:4:24:ILE:HG23	5:4:32:ALA:HB1	2.03	0.41
6:3:25:LYS:HA	6:3:25:LYS:HD2	1.94	0.41
9:D:70:VAL:HB	9:D:163:VAL:HA	2.01	0.41
9:D:113:ARG:NH2	9:D:140:GLU:HG2	2.36	0.41
10:d:8:VAL:HA	10:d:39:ALA:O	2.21	0.41
10:d:21:ARG:HG2	10:d:26:PHE:HA	2.03	0.41
10:d:61:LEU:HD21	10:d:73:LYS:HA	2.03	0.41
12:e:75:LYS:HG3	12:e:77:ARG:HG3	2.03	0.41
19:I:249:C:O2	26:l:12:LYS:NZ	2.47	0.41
19:I:250:G:H2’	19:I:251:A:C8	2.56	0.41
19:I:552:U:C2	19:I:553:G:C8	3.09	0.41
19:I:588:U:H2’	19:I:589:U:C6	2.56	0.41
19:I:878:A:H3’	19:I:879:G:C8	2.56	0.41
19:I:1028:A:N3	19:I:2486:C:O2’	2.49	0.41
19:I:1231:U:H2’	19:I:1232:G:H8	1.86	0.41
19:I:1687:G:H2’	19:I:1688:U:C6	2.56	0.41
19:I:1733:G:N3	19:I:1734:G:C8	2.89	0.41
19:I:2298:A:H2’	19:I:2299:U:H5’	2.03	0.41
19:I:2419:U:OP1	26:l:41:LYS:NZ	2.50	0.41
19:I:2494:G:C2	19:I:2495:G:C8	3.08	0.41

Continued on next page...

For Manuscript Review

Continued from previous page...

Atom-1	Atom-2	Interatomic distance (Å)	Clash overlap (Å)
19:I:2531:A:OP2	31:O:175:LYS:N	2.42	0.41
19:I:2703:C:H2’	19:I:2704:C:C6	2.55	0.41
19:I:2803:G:H2’	19:I:2804:U:H6	1.86	0.41
19:I:2830:C:H5”	25:L:56:LYS:HE3	2.02	0.41
20:i:38:HIS:ND1	20:i:39:LEU:O	2.53	0.41
26:l:62:LEU:HB3	26:l:65:ALA:HB2	2.03	0.41
31:O:86:LYS:HD3	31:O:132:VAL:HG22	2.01	0.41
34:p:19:VAL:HG11	34:p:83:ILE:HA	2.03	0.41
39:S:51:LYS:HZ3	39:S:51:LYS:HG3	1.76	0.41
41:T:29:LYS:HB3	41:T:30:THR:H	1.71	0.41
43:U:53:MET:HG2	43:U:105:MET:HE1	2.03	0.41
45:V:79:LEU:HD23	45:V:83:LEU:HD23	2.03	0.41
46:w:21:ASP:O	46:w:21:ASP:OD2	2.39	0.41
50:y:51:ASN:OD1	50:y:51:ASN:N	2.54	0.41
1:C:257:A:H2’	1:C:258:A:H8	1.86	0.41
1:C:331:G:C2	1:C:332:A:C5	3.09	0.41
1:C:400:G:H2’	1:C:401:U:C6	2.56	0.41
1:C:641:U:H2’	1:C:642:G:C8	2.55	0.41
1:C:974:C:O2’	44:v:13:ARG:NH1	2.54	0.41
1:C:1210:G:H5”	44:v:5:SER:OG	2.21	0.41
1:C:1282:A:N1	1:C:1365:G:H1’	2.36	0.41
19:I:40:U:H2’	19:I:41:C:C6	2.56	0.41
19:I:249:C:OP2	19:I:2394:C:O2’	2.24	0.41
19:I:521:U:H2’	19:I:522:A:C8	2.55	0.41
19:I:543:G:H2’	19:I:544:C:H5’	2.03	0.41
19:I:686:U:H6	19:I:788:A:N1	2.19	0.41
19:I:689:A:H2’	19:I:690:G:H8	1.86	0.41
19:I:1421:G:H2’	19:I:1422:G:C8	2.38	0.41
19:I:1754:A:N1	19:I:2716:C:O2’	2.42	0.41
19:I:2478:A:H5’	19:I:2479:U:C5	2.51	0.41
19:I:2533:U:H2’	19:I:2534:A:O4’	2.20	0.41
19:I:2777:G:H21	19:I:2781:A:H2	1.68	0.41
25:L:149:ASN:OD1	25:L:150:GLN:N	2.54	0.41
27:M:138:LEU:HD23	27:M:143:LEU:HB2	2.03	0.41
30:n:30:LEU:HD12	30:n:105:VAL:HG13	2.03	0.41
30:n:70:ARG:O	30:n:138:ARG:NH1	2.53	0.41
31:O:92:VAL:HG22	31:O:160:LYS:NZ	2.36	0.41
33:P:96:THR:HA	33:P:99:ILE:HG22	2.03	0.41
36:q:52:LEU:HB2	44:v:81:ARG:HD2	2.03	0.41
41:T:89:VAL:HG22	41:T:121:THR:HB	2.02	0.41
48:x:2:VAL:HG11	48:x:5:ARG:HB2	2.03	0.41

Continued on next page...

For Manuscript Review

Continued from previous page...

Atom-1	Atom-2	Interatomic distance (Å)	Clash overlap (Å)
48:x:20:VAL:HA	48:x:35:ARG:HA	2.02	0.41
1:C:877:C:H2'	1:C:878:U:C6	2.56	0.40
1:C:1066:G:H2'	1:C:1067:U:C6	2.57	0.40
1:C:1156:C:H2'	1:C:1157:G:H8	1.85	0.40
1:C:1266:U:H2'	1:C:1267:G:C8	2.56	0.40
8:c:72:ILE:O	8:c:80:ALA:HA	2.21	0.40
9:D:154:MET:HE2	9:D:156:GLY:C	2.45	0.40
11:E:75:ILE:H	11:E:75:ILE:HG12	1.69	0.40
13:F:11:LEU:HD22	13:F:63:ARG:NH1	2.36	0.40
19:I:1196:C:H2'	19:I:1197:G:C8	2.56	0.40
19:I:1410:G:C6	19:I:1593:A:N6	2.89	0.40
19:I:1716:U:H2'	19:I:1717:A:H8	1.86	0.40
19:I:1923:U:H2'	19:I:1924:C:C6	2.56	0.40
19:I:2399:G:H2'	19:I:2400:G:C8	2.57	0.40
19:I:2554:U:H2'	19:I:2555:U:C6	2.56	0.40
19:I:2679:A:H2'	19:I:2680:U:H6	1.85	0.40
20:i:38:HIS:ND1	20:i:42:HIS:O	2.54	0.40
29:N:66:LEU:HG	29:N:67:ILE:H	1.86	0.40
40:t:42:PRO:HB3	40:t:89:ASP:OD2	2.21	0.40
43:U:21:ALA:HB2	43:U:97:GLN:HB2	2.03	0.40
1:C:96:G:H2'	1:C:97:C:H6	1.85	0.40
1:C:148:G:C6	1:C:162:G:C6	3.09	0.40
1:C:494:G:OP1	40:t:121:ARG:NH2	2.54	0.40
1:C:656:U:O2'	1:C:830:G:OP1	2.39	0.40
1:C:685:G:H2'	1:C:686:U:C6	2.56	0.40
8:c:17:LYS:H	8:c:17:LYS:HG2	1.73	0.40
8:c:83:VAL:HG11	8:c:94:ARG:HB3	2.02	0.40
9:D:104:TRP:HE1	9:D:108:ARG:NH2	2.19	0.40
11:E:121:THR:HG23	11:E:189:ALA:HB2	2.03	0.40
14:f:26:LYS:HD3	19:I:189:G:OP1	2.22	0.40
14:f:68:LEU:HD23	14:f:68:LEU:HA	1.83	0.40
17:H:3:HIS:CD2	17:H:3:HIS:N	2.89	0.40
19:I:598:U:O4	19:I:599:A:N6	2.55	0.40
19:I:1212:G:H1'	19:I:1237:A:N6	2.36	0.40
19:I:1410:G:N1	19:I:1593:A:N6	2.69	0.40
19:I:1651:G:H4'	45:V:39:PRO:HG3	2.03	0.40
19:I:1734:G:N3	19:I:1735:A:C8	2.89	0.40
19:I:2171:A:O2'	19:I:2172:U:H3'	2.21	0.40
19:I:2230:G:H2'	19:I:2231:U:C6	2.56	0.40
19:I:2883:A:OP2	20:i:50:ARG:NH1	2.54	0.40
23:K:37:ASN:HB2	23:K:62:TYR:HB2	2.03	0.40

Continued on next page...

For Manuscript Review

Continued from previous page...

Atom-1	Atom-2	Interatomic distance (Å)	Clash overlap (Å)
29:N:109:PRO:HG3	35:Q:44:PHE:CE2	2.55	0.40
36:q:40:ILE:HD11	36:q:73:LEU:HB3	2.03	0.40
40:t:110:ARG:HD2	40:t:110:ARG:HA	1.94	0.40
40:t:114:ARG:HG2	40:t:119:VAL:O	2.21	0.40
44:v:21:PHE:CD1	44:v:25:ALA:HB2	2.55	0.40
50:y:50:ASN:O	50:y:52:GLU:N	2.53	0.40
1:C:506:U:H2'	1:C:507:U:C6	2.56	0.40
1:C:1454:C:C2	1:C:1455:G:C8	3.10	0.40
8:c:8:ASP:OD1	8:c:8:ASP:N	2.54	0.40
12:e:40:GLN:O	12:e:57:HIS:HB2	2.21	0.40
12:e:66:LYS:HE2	12:e:66:LYS:HB3	1.91	0.40
19:I:2:G:N3	19:I:2:G:H2'	2.35	0.40
19:I:29:U:H2'	19:I:30:G:C8	2.56	0.40
19:I:391:A:H1'	19:I:411:G:O4'	2.21	0.40
19:I:577:G:H2'	19:I:578:G:C8	2.56	0.40
19:I:604:G:O2'	19:I:656:G:N2	2.53	0.40
19:I:764:A:H2	23:K:218:PRO:HG3	1.86	0.40
19:I:784:G:H5'	19:I:785:G:OP1	2.21	0.40
19:I:1067:A:H8	19:I:1067:A:OP1	2.03	0.40
19:I:1431:A:H2'	19:I:1432:G:C8	2.55	0.40
21:J:56:G:O2'	21:J:59:A:N6	2.54	0.40
21:J:98:G:O2'	21:J:99:A:H5"	2.21	0.40
36:q:40:ILE:O	36:q:40:ILE:HD12	2.21	0.40
37:R:102:GLU:HB3	37:R:119:PHE:CZ	2.53	0.40
45:V:30:ARG:HD2	45:V:31:HIS:NE2	2.36	0.40
1:C:350:A:N3	1:C:362:U:O2'	2.37	0.40
1:C:801:A:H2'	1:C:802:C:C6	2.56	0.40
1:C:905:U:H2'	1:C:906:C:C6	2.55	0.40
1:C:946:U:H2'	1:C:947:G:H8	1.86	0.40
1:C:1405:C:H2'	1:C:1406:C:H6	1.87	0.40
1:C:1444:G:H3'	1:C:1445:C:H5"	2.04	0.40
7:b:48:GLN:HA	7:b:53:VAL:O	2.22	0.40
9:D:74:ARG:HA	9:D:74:ARG:CZ	2.51	0.40
11:E:42:TYR:OH	11:E:90:VAL:HG11	2.20	0.40
19:I:1190:G:H2'	19:I:1191:G:C8	2.57	0.40
19:I:1987:A:H2'	19:I:1988:G:H8	1.84	0.40
21:J:14:U:O3'	21:J:107:G:O2'	2.40	0.40
36:q:18:ILE:HA	36:q:21:ALA:HB3	2.03	0.40
40:t:87:VAL:HG23	40:t:90:LEU:H	1.86	0.40
41:T:93:ASN:HA	41:T:96:LYS:HD2	2.03	0.40
43:U:34:LYS:HG3	43:U:35:ALA:N	2.36	0.40

Continued on next page...

For Manuscript Review

Continued from previous page...

Atom-1	Atom-2	Interatomic distance (Å)	Clash overlap (Å)
44:v:30:ILE:HG13	44:v:31:ILE:N	2.37	0.40
50:y:46:VAL:HG21	50:y:61:ILE:HG21	2.02	0.40
1:C:315:A:H2'	1:C:316:C:C6	2.56	0.40
1:C:664:G:H2'	1:C:665:G:C8	2.56	0.40
1:C:895:A:O2'	1:C:1507:A:OP1	2.25	0.40
1:C:1046:U:O2'	1:C:1049:A:OP2	2.26	0.40
1:C:1102:G:N3	1:C:1102:G:H2'	2.36	0.40
1:C:1280:U:O2'	1:C:1281:A:H4'	2.22	0.40
1:C:1340:A:OP1	34:p:122:ARG:NH1	2.55	0.40
12:e:18:ALA:HB1	19:I:2271:G:OP1	2.21	0.40
19:I:296:U:H2'	19:I:297:G:C8	2.57	0.40
19:I:713:G:H21	19:I:718:A:H62	1.69	0.40
19:I:923:G:H2'	19:I:924:G:H8	1.86	0.40
19:I:1197:G:H2'	19:I:1198:U:H6	1.86	0.40
19:I:1223:G:C6	19:I:1227:G:C6	3.10	0.40
19:I:1422:G:H2'	19:I:1423:G:H8	1.86	0.40
19:I:1689:A:H2'	19:I:1690:A:H8	1.84	0.40
19:I:1724:G:C6	19:I:1737:G:C2	3.10	0.40
19:I:1821:A:H2'	19:I:1822:C:C6	2.57	0.40
19:I:2034:U:O2'	19:I:2035:G:H5'	2.21	0.40
19:I:2718:G:O2'	19:I:2847:U:OP1	2.39	0.40
21:J:85:G:C2	21:J:86:G:C8	3.09	0.40
29:N:51:ASP:O	29:N:55:ALA:N	2.38	0.40
29:N:91:LEU:HA	29:N:91:LEU:HD23	1.74	0.40
30:n:25:LYS:HG3	30:n:29:ILE:HG13	2.03	0.40
30:n:86:GLN:HB2	30:n:148:ASN:ND2	2.36	0.40
30:n:105:VAL:O	30:n:109:ARG:HG3	2.21	0.40
36:q:86:ALA:HA	36:q:89:ARG:HE	1.87	0.40
41:T:73:ILE:HD12	41:T:104:GLN:O	2.21	0.40
42:u:40:ALA:HB3	42:u:43:VAL:HG13	2.04	0.40
45:V:21:PHE:HA	45:V:24:MET:HB3	2.02	0.40
46:w:74:ASP:HB2	46:w:77:ARG:HD3	2.04	0.40

There are no symmetry-related clashes.

5.3 Torsion angles (i)

5.3.1 Protein backbone (i)

In the following table, the Percentiles column shows the percent Ramachandran outliers of the chain as a percentile score with respect to all PDB entries followed by that with respect to all EM

For Manuscript Review

entries.

The Analysed column shows the number of residues for which the backbone conformation was analysed, and the total number of residues.

Mol	Chain	Analysed	Favoured	Allowed	Outliers	Percentiles	
2	1	79/92 (86%)	72 (91%)	5 (6%)	2 (2%)	4	22
3	Z	101/103 (98%)	99 (98%)	1 (1%)	1 (1%)	13	44
4	2	82/87 (94%)	79 (96%)	2 (2%)	1 (1%)	11	39
5	4	108/110 (98%)	108 (100%)	0	0	100	100
6	3	68/71 (96%)	68 (100%)	0	0	100	100
7	b	91/100 (91%)	90 (99%)	1 (1%)	0	100	100
8	c	100/104 (96%)	91 (91%)	8 (8%)	1 (1%)	13	44
9	D	216/241 (90%)	207 (96%)	8 (4%)	1 (0%)	25	59
10	d	92/94 (98%)	86 (94%)	6 (6%)	0	100	100
11	E	204/233 (88%)	197 (97%)	7 (3%)	0	100	100
12	e	73/85 (86%)	68 (93%)	5 (7%)	0	100	100
13	F	203/206 (98%)	184 (91%)	18 (9%)	1 (0%)	25	59
14	f	75/78 (96%)	74 (99%)	1 (1%)	0	100	100
15	G	148/167 (89%)	142 (96%)	6 (4%)	0	100	100
16	g	60/63 (95%)	58 (97%)	2 (3%)	0	100	100
17	H	98/135 (73%)	95 (97%)	3 (3%)	0	100	100
18	h	56/59 (95%)	50 (89%)	6 (11%)	0	100	100
20	i	54/57 (95%)	54 (100%)	0	0	100	100
22	j	48/55 (87%)	44 (92%)	4 (8%)	0	100	100
23	K	270/273 (99%)	259 (96%)	11 (4%)	0	100	100
24	k	44/46 (96%)	43 (98%)	1 (2%)	0	100	100
25	L	207/209 (99%)	197 (95%)	10 (5%)	0	100	100
26	l	62/65 (95%)	60 (97%)	2 (3%)	0	100	100
27	M	199/201 (99%)	191 (96%)	8 (4%)	0	100	100
28	m	36/38 (95%)	36 (100%)	0	0	100	100
29	N	175/179 (98%)	165 (94%)	10 (6%)	0	100	100
30	n	149/179 (83%)	137 (92%)	10 (7%)	2 (1%)	10	37
31	O	174/177 (98%)	157 (90%)	15 (9%)	2 (1%)	12	42
32	o	127/130 (98%)	123 (97%)	4 (3%)	0	100	100

Continued on next page...

For Manuscript Review

Continued from previous page...

Mol	Chain	Analysed	Favoured	Allowed	Outliers	Percentiles	
33	P	147/149 (99%)	141 (96%)	6 (4%)	0	100	100
34	p	125/130 (96%)	99 (79%)	20 (16%)	6 (5%)	2	10
35	Q	56/70 (80%)	53 (95%)	3 (5%)	0	100	100
36	q	96/103 (93%)	93 (97%)	2 (2%)	1 (1%)	13	44
37	R	140/142 (99%)	136 (97%)	4 (3%)	0	100	100
38	r	115/129 (89%)	111 (96%)	4 (4%)	0	100	100
39	S	120/123 (98%)	117 (98%)	3 (2%)	0	100	100
40	t	121/124 (98%)	111 (92%)	10 (8%)	0	100	100
41	T	142/144 (99%)	138 (97%)	4 (3%)	0	100	100
42	u	113/118 (96%)	95 (84%)	15 (13%)	3 (3%)	4	21
43	U	133/136 (98%)	127 (96%)	6 (4%)	0	100	100
44	v	92/101 (91%)	89 (97%)	3 (3%)	0	100	100
45	V	118/127 (93%)	115 (98%)	3 (2%)	0	100	100
46	w	86/89 (97%)	82 (95%)	4 (5%)	0	100	100
47	W	114/117 (97%)	113 (99%)	1 (1%)	0	100	100
48	x	80/82 (98%)	63 (79%)	16 (20%)	1 (1%)	10	37
49	X	112/115 (97%)	109 (97%)	3 (3%)	0	100	100
50	y	78/84 (93%)	70 (90%)	8 (10%)	0	100	100
51	Y	115/118 (98%)	114 (99%)	1 (1%)	0	100	100
52	z	53/75 (71%)	52 (98%)	1 (2%)	0	100	100
All	All	5555/5913 (94%)	5262 (95%)	271 (5%)	22 (0%)	32	64

All (22) Ramachandran outliers are listed below:

Mol	Chain	Res	Type
2	1	9	PRO
4	2	5	LYS
8	c	54	GLN
30	n	59	LEU
34	p	73	SER
42	u	12	HIS
42	u	28	THR
42	u	45	ILE
34	p	58	VAL
34	p	92	GLU

Continued on next page...

For Manuscript Review

Continued from previous page...

Mol	Chain	Res	Type
36	q	57	VAL
48	x	13	LYS
30	n	7	ILE
9	D	14	VAL
31	O	14	GLY
34	p	56	ASP
34	p	62	ASP
31	O	13	ALA
2	1	29	LYS
13	F	37	ALA
3	Z	52	PRO
34	p	104	VAL

5.3.2 Protein sidechains (i)

In the following table, the Percentiles column shows the percent sidechain outliers of the chain as a percentile score with respect to all PDB entries followed by that with respect to all EM entries.

The Analysed column shows the number of residues for which the sidechain conformation was analysed, and the total number of residues.

Mol	Chain	Analysed	Rotameric	Outliers	Percentiles	
2	1	71/79 (90%)	64 (90%)	7 (10%)	6	24
3	Z	84/84 (100%)	81 (96%)	3 (4%)	30	62
4	2	65/66 (98%)	61 (94%)	4 (6%)	15	44
5	4	93/93 (100%)	92 (99%)	1 (1%)	70	87
6	3	60/61 (98%)	57 (95%)	3 (5%)	20	52
7	b	80/84 (95%)	78 (98%)	2 (2%)	42	72
8	c	83/85 (98%)	80 (96%)	3 (4%)	30	62
9	D	180/199 (90%)	174 (97%)	6 (3%)	33	65
10	d	78/78 (100%)	72 (92%)	6 (8%)	10	35
11	E	170/190 (90%)	163 (96%)	7 (4%)	26	58
12	e	57/63 (90%)	55 (96%)	2 (4%)	31	64
13	F	172/173 (99%)	159 (92%)	13 (8%)	11	36
14	f	67/68 (98%)	65 (97%)	2 (3%)	36	68
15	G	113/126 (90%)	109 (96%)	4 (4%)	31	64

Continued on next page...

For Manuscript Review

Continued from previous page...

Mol	Chain	Analysed	Rotameric	Outliers	Percentiles	
16	g	54/55 (98%)	52 (96%)	2 (4%)	29	62
17	H	87/116 (75%)	86 (99%)	1 (1%)	70	87
18	h	48/49 (98%)	47 (98%)	1 (2%)	48	76
20	i	47/48 (98%)	45 (96%)	2 (4%)	25	57
22	j	45/49 (92%)	44 (98%)	1 (2%)	47	75
23	K	217/218 (100%)	213 (98%)	4 (2%)	54	79
24	k	38/38 (100%)	35 (92%)	3 (8%)	10	34
25	L	164/164 (100%)	159 (97%)	5 (3%)	36	68
26	l	51/52 (98%)	50 (98%)	1 (2%)	50	77
27	M	165/165 (100%)	163 (99%)	2 (1%)	67	85
28	m	34/34 (100%)	32 (94%)	2 (6%)	16	46
29	N	148/150 (99%)	143 (97%)	5 (3%)	32	65
30	n	124/147 (84%)	116 (94%)	8 (6%)	14	42
31	O	137/138 (99%)	136 (99%)	1 (1%)	81	91
32	o	104/105 (99%)	100 (96%)	4 (4%)	28	61
33	P	114/114 (100%)	109 (96%)	5 (4%)	24	56
34	p	105/107 (98%)	101 (96%)	4 (4%)	28	61
35	Q	55/62 (89%)	54 (98%)	1 (2%)	54	79
36	q	86/90 (96%)	79 (92%)	7 (8%)	9	33
37	R	116/116 (100%)	113 (97%)	3 (3%)	41	71
38	r	90/99 (91%)	88 (98%)	2 (2%)	47	75
39	S	103/104 (99%)	100 (97%)	3 (3%)	37	69
40	t	103/104 (99%)	92 (89%)	11 (11%)	5	21
41	T	103/103 (100%)	101 (98%)	2 (2%)	52	78
42	u	93/96 (97%)	88 (95%)	5 (5%)	18	49
43	U	108/108 (100%)	103 (95%)	5 (5%)	23	55
44	v	79/84 (94%)	77 (98%)	2 (2%)	42	72
45	V	100/103 (97%)	99 (99%)	1 (1%)	73	88
46	w	75/77 (97%)	74 (99%)	1 (1%)	65	84
47	W	86/87 (99%)	84 (98%)	2 (2%)	45	74
48	x	65/65 (100%)	62 (95%)	3 (5%)	23	55

Continued on next page...

For Manuscript Review

Continued from previous page...

Mol	Chain	Analysed	Rotameric	Outliers	Percentiles	
49	X	99/100 (99%)	95 (96%)	4 (4%)	27	59
50	y	74/78 (95%)	71 (96%)	3 (4%)	26	58
51	Y	89/90 (99%)	85 (96%)	4 (4%)	23	56
52	z	48/65 (74%)	44 (92%)	4 (8%)	9	32
All	All	4627/4829 (96%)	4450 (96%)	177 (4%)	30	61

All (177) residues with a non-rotameric sidechain are listed below:

Mol	Chain	Res	Type
2	1	11	ILE
2	1	16	LEU
2	1	32	ARG
2	1	40	ILE
2	1	62	VAL
2	1	66	MET
2	1	79	THR
3	Z	51	VAL
3	Z	72	VAL
3	Z	87	GLN
4	2	6	SER
4	2	10	ARG
4	2	67	ILE
4	2	86	LEU
5	4	74	ILE
6	3	3	VAL
6	3	10	GLU
6	3	43	THR
7	b	7	LEU
7	b	88	LYS
8	c	7	ARG
8	c	11	VAL
8	c	30	SER
9	D	19	GLN
9	D	113	ARG
9	D	117	LEU
9	D	170	HIS
9	D	186	ILE
9	D	196	VAL
10	d	1	MET
10	d	51	GLN
10	d	61	LEU

Continued on next page...

For Manuscript Review

Continued from previous page...

Mol	Chain	Res	Type
10	d	76	ASP
10	d	78	GLN
10	d	82	TYR
11	E	3	GLN
11	E	53	SER
11	E	64	ILE
11	E	97	VAL
11	E	142	MET
11	E	186	THR
11	E	195	VAL
12	e	31	VAL
12	e	44	LYS
13	F	23	SER
13	F	55	LEU
13	F	56	ARG
13	F	91	LEU
13	F	101	VAL
13	F	104	ARG
13	F	123	ILE
13	F	132	ILE
13	F	137	VAL
13	F	164	GLN
13	F	166	GLU
13	F	179	GLU
13	F	197	GLU
14	f	33	LEU
14	f	66	THR
15	G	34	THR
15	G	101	GLU
15	G	130	SER
15	G	148	ASN
16	g	52	ARG
16	g	57	LEU
17	H	70	VAL
18	h	7	ILE
20	i	9	THR
20	i	54	VAL
22	j	21	TYR
23	K	114	ASP
23	K	225	MET
23	K	236	GLU
23	K	268	VAL

Continued on next page...

For Manuscript Review

Continued from previous page...

Mol	Chain	Res	Type
24	k	1	MET
24	k	4	THR
24	k	8	SER
25	L	4	LEU
25	L	18	ASP
25	L	99	GLU
25	L	108	ASP
25	L	137	SER
26	l	54	ASP
27	M	1	MET
27	M	49	ARG
28	m	8	LYS
28	m	25	VAL
29	N	28	VAL
29	N	35	THR
29	N	51	ASP
29	N	147	ASP
29	N	155	THR
30	n	11	LYS
30	n	12	ILE
30	n	49	THR
30	n	80	VAL
30	n	84	THR
30	n	101	MET
30	n	106	GLU
30	n	118	LEU
31	O	155	GLU
32	o	78	VAL
32	o	90	ASP
32	o	104	VAL
32	o	125	ILE
33	P	21	VAL
33	P	77	THR
33	P	115	VAL
33	P	130	VAL
33	P	134	VAL
34	p	21	ILE
34	p	59	GLU
34	p	115	LYS
34	p	116	VAL
35	Q	32	LEU
36	q	6	ILE

Continued on next page...

For Manuscript Review

Continued from previous page...

Mol	Chain	Res	Type
36	q	8	ILE
36	q	27	GLU
36	q	40	ILE
36	q	67	ILE
36	q	78	GLU
36	q	83	THR
37	R	93	ILE
37	R	98	GLU
37	R	122	LEU
38	r	79	ILE
38	r	111	THR
39	S	1	MET
39	S	41	ILE
39	S	76	VAL
40	t	5	ASN
40	t	18	LYS
40	t	24	LEU
40	t	25	GLU
40	t	29	GLN
40	t	37	VAL
40	t	38	TYR
40	t	41	THR
40	t	64	THR
40	t	77	HIS
40	t	93	VAL
41	T	110	VAL
41	T	125	LEU
42	u	14	HIS
42	u	16	VAL
42	u	58	ASP
42	u	76	SER
42	u	97	VAL
43	U	1	MET
43	U	106	ASP
43	U	119	LEU
43	U	135	VAL
43	U	136	MET
44	v	24	ARG
44	v	97	LYS
45	V	83	LEU
46	w	22	THR
47	W	12	THR

Continued on next page...

For Manuscript Review

Continued from previous page...

Mol	Chain	Res	Type
47	W	98	GLN
48	x	4	ILE
48	x	13	LYS
48	x	71	VAL
49	X	7	GLN
49	X	12	GLN
49	X	102	GLU
49	X	114	LEU
50	y	4	LYS
50	y	20	SER
50	y	51	ASN
51	Y	4	VAL
51	Y	5	LYS
51	Y	31	VAL
51	Y	88	VAL
52	z	21	ILE
52	z	28	THR
52	z	34	THR
52	z	45	THR

Sometimes sidechains can be flipped to improve hydrogen bonding and reduce clashes. All (27) such sidechains are listed below:

Mol	Chain	Res	Type
4	2	13	GLN
10	d	12	GLN
11	E	8	ASN
11	E	123	GLN
11	E	190	HIS
12	e	76	ASN
13	F	40	GLN
13	F	136	GLN
14	f	16	ASN
16	g	41	HIS
17	H	17	GLN
23	K	60	GLN
23	K	70	ASN
25	L	150	GLN
26	l	28	ASN
27	M	24	ASN
30	n	148	ASN
33	P	2	GLN
36	q	56	HIS

Continued on next page...

For Manuscript Review

Continued from previous page...

Mol	Chain	Res	Type
38	r	64	GLN
38	r	118	HIS
39	S	90	ASN
42	u	105	ASN
47	W	38	GLN
48	x	59	HIS
49	X	66	ASN
51	Y	44	GLN

5.3.3 RNA ⓘ

Mol	Chain	Analysed	Backbone Outliers	Pucker Outliers
1	C	1525/1536 (99%)	398 (26%)	20 (1%)
19	I	2894/2904 (99%)	564 (19%)	11 (0%)
21	J	117/120 (97%)	24 (20%)	0
All	All	4536/4560 (99%)	986 (21%)	31 (0%)

All (986) RNA backbone outliers are listed below:

Mol	Chain	Res	Type
1	C	3	A
1	C	9	G
1	C	22	G
1	C	31	G
1	C	32	A
1	C	36	C
1	C	39	G
1	C	42	G
1	C	47	C
1	C	48	C
1	C	50	A
1	C	51	A
1	C	52	C
1	C	60	A
1	C	62	U
1	C	63	C
1	C	65	A
1	C	66	G
1	C	68	G
1	C	69	G
1	C	70	A

Continued on next page...

For Manuscript Review

Continued from previous page...

Mol	Chain	Res	Type
1	C	71	U
1	C	73	A
1	C	74	A
1	C	75	G
1	C	76	G
1	C	77	G
1	C	78	A
1	C	79	G
1	C	80	C
1	C	81	U
1	C	82	U
1	C	83	G
1	C	84	C
1	C	85	U
1	C	87	C
1	C	90	G
1	C	92	U
1	C	94	C
1	C	95	A
1	C	99	G
1	C	102	G
1	C	104	C
1	C	110	A
1	C	115	U
1	C	124	A
1	C	125	U
1	C	131	U
1	C	132	G
1	C	133	G
1	C	134	U
1	C	137	U
1	C	138	G
1	C	142	G
1	C	143	A
1	C	145	A
1	C	148	G
1	C	157	C
1	C	164	U
1	C	167	U
1	C	168	A
1	C	169	C
1	C	170	C

Continued on next page...

For Manuscript Review

Continued from previous page...

Mol	Chain	Res	Type
1	C	171	G
1	C	172	C
1	C	173	A
1	C	174	U
1	C	175	A
1	C	176	C
1	C	177	G
1	C	179	C
1	C	182	G
1	C	183	A
1	C	184	G
1	C	186	G
1	C	189	A
1	C	190	A
1	C	191	A
1	C	195	G
1	C	197	G
1	C	198	G
1	C	199	A
1	C	200	U
1	C	202	U
1	C	203	U
1	C	204	C
1	C	206	G
1	C	207	A
1	C	209	C
1	C	210	U
1	C	211	C
1	C	214	G
1	C	221	G
1	C	223	U
1	C	228	C
1	C	237	A
1	C	241	G
1	C	245	G
1	C	247	U
1	C	260	G
1	C	261	C
1	C	266	C
1	C	267	A
1	C	274	C
1	C	283	A

Continued on next page...

For Manuscript Review

Continued from previous page...

Mol	Chain	Res	Type
1	C	287	G
1	C	315	A
1	C	320	G
1	C	321	A
1	C	322	C
1	C	323	A
1	C	324	C
1	C	325	G
1	C	332	A
1	C	336	C
1	C	338	A
1	C	339	C
1	C	340	G
1	C	345	G
1	C	346	C
1	C	347	A
1	C	348	G
1	C	350	A
1	C	354	G
1	C	357	A
1	C	361	U
1	C	366	C
1	C	367	A
1	C	372	G
1	C	377	A
1	C	378	G
1	C	380	C
1	C	381	U
1	C	386	C
1	C	392	U
1	C	400	G
1	C	401	U
1	C	403	U
1	C	405	A
1	C	406	A
1	C	408	A
1	C	409	A
1	C	417	G
1	C	422	G
1	C	423	U
1	C	426	A
1	C	427	G

Continued on next page...

For Manuscript Review

Continued from previous page...

Mol	Chain	Res	Type
1	C	430	C
1	C	432	U
1	C	440	G
1	C	441	G
1	C	442	A
1	C	444	G
1	C	445	A
1	C	450	C
1	C	452	G
1	C	453	U
1	C	454	A
1	C	456	G
1	C	457	U
1	C	459	A
1	C	460	A
1	C	461	U
1	C	462	A
1	C	463	C
1	C	466	U
1	C	468	C
1	C	470	G
1	C	475	G
1	C	476	A
1	C	478	G
1	C	480	U
1	C	487	A
1	C	488	G
1	C	489	A
1	C	490	A
1	C	491	U
1	C	492	A
1	C	493	A
1	C	503	A
1	C	504	A
1	C	505	C
1	C	507	U
1	C	512	C
1	C	513	C
1	C	514	A
1	C	515	G
1	C	518	G
1	C	521	G

Continued on next page...

For Manuscript Review

Continued from previous page...

Mol	Chain	Res	Type
1	C	525	U
1	C	527	A
1	C	528	U
1	C	534	G
1	C	539	C
1	C	541	A
1	C	553	A
1	C	556	U
1	C	558	C
1	C	566	A
1	C	567	A
1	C	569	G
1	C	570	C
1	C	571	G
1	C	585	C
1	C	626	U
1	C	627	G
1	C	647	A
1	C	654	G
1	C	659	A
1	C	660	G
1	C	682	G
1	C	687	G
1	C	688	A
1	C	694	G
1	C	697	G
1	C	711	C
1	C	715	A
1	C	717	U
1	C	718	G
1	C	725	G
1	C	749	A
1	C	771	A
1	C	781	A
1	C	787	U
1	C	788	A
1	C	806	G
1	C	807	U
1	C	809	A
1	C	810	A
1	C	811	C
1	C	815	G

Continued on next page...

For Manuscript Review

Continued from previous page...

Mol	Chain	Res	Type
1	C	822	A
1	C	835	C
1	C	837	U
1	C	838	G
1	C	839	A
1	C	840	G
1	C	843	C
1	C	844	U
1	C	861	G
1	C	865	U
1	C	866	A
1	C	870	C
1	C	883	A
1	C	896	G
1	C	920	G
1	C	921	G
1	C	928	C
1	C	929	A
1	C	954	U
1	C	960	G
1	C	963	A
1	C	965	G
1	C	969	A
1	C	970	G
1	C	971	A
1	C	978	C
1	C	981	G
1	C	983	C
1	C	986	U
1	C	987	G
1	C	988	A
1	C	990	A
1	C	993	C
1	C	996	A
1	C	998	A
1	C	1002	U
1	C	1003	U
1	C	1004	C
1	C	1012	G
1	C	1013	G
1	C	1014	A
1	C	1015	U

Continued on next page...

For Manuscript Review

Continued from previous page...

Mol	Chain	Res	Type
1	C	1017	G
1	C	1024	U
1	C	1025	C
1	C	1026	G
1	C	1029	A
1	C	1030	A
1	C	1031	C
1	C	1032	U
1	C	1034	A
1	C	1035	G
1	C	1036	A
1	C	1038	A
1	C	1039	C
1	C	1044	G
1	C	1047	G
1	C	1049	A
1	C	1059	U
1	C	1060	C
1	C	1075	G
1	C	1078	G
1	C	1079	U
1	C	1080	U
1	C	1088	G
1	C	1089	U
1	C	1095	A
1	C	1102	G
1	C	1119	U
1	C	1122	C
1	C	1124	A
1	C	1125	G
1	C	1126	C
1	C	1128	C
1	C	1131	C
1	C	1132	G
1	C	1133	G
1	C	1134	G
1	C	1138	G
1	C	1139	C
1	C	1140	A
1	C	1143	C
1	C	1146	A
1	C	1147	G

Continued on next page...

For Manuscript Review

Continued from previous page...

Mol	Chain	Res	Type
1	C	1149	A
1	C	1151	A
1	C	1154	G
1	C	1155	C
1	C	1157	G
1	C	1162	C
1	C	1163	A
1	C	1164	A
1	C	1168	G
1	C	1178	G
1	C	1184	G
1	C	1190	A
1	C	1191	A
1	C	1194	C
1	C	1196	U
1	C	1207	A
1	C	1208	C
1	C	1221	A
1	C	1230	A
1	C	1232	A
1	C	1234	U
1	C	1235	G
1	C	1243	C
1	C	1245	A
1	C	1251	U
1	C	1252	G
1	C	1254	C
1	C	1262	G
1	C	1263	A
1	C	1264	G
1	C	1265	G
1	C	1268	G
1	C	1269	A
1	C	1273	A
1	C	1274	A
1	C	1280	U
1	C	1281	A
1	C	1282	A
1	C	1287	G
1	C	1291	G
1	C	1293	A
1	C	1296	C

Continued on next page...

For Manuscript Review

Continued from previous page...

Mol	Chain	Res	Type
1	C	1299	G
1	C	1306	G
1	C	1310	G
1	C	1311	C
1	C	1314	C
1	C	1316	C
1	C	1326	A
1	C	1340	A
1	C	1341	G
1	C	1342	U
1	C	1347	G
1	C	1357	A
1	C	1358	U
1	C	1364	G
1	C	1373	G
1	C	1388	A
1	C	1392	A
1	C	1398	C
1	C	1413	G
1	C	1426	G
1	C	1427	A
1	C	1435	A
1	C	1436	G
1	C	1440	A
1	C	1442	C
1	C	1444	G
1	C	1445	C
1	C	1447	A
1	C	1448	G
1	C	1453	A
1	C	1456	G
1	C	1486	A
1	C	1488	G
1	C	1491	G
1	C	1497	A
1	C	1500	U
1	C	1511	G
1	C	1514	C
1	C	1523	G
1	C	1524	G
1	C	1525	A
19	I	2	G

Continued on next page...

For Manuscript Review

Continued from previous page...

Mol	Chain	Res	Type
19	I	12	U
19	I	15	G
19	I	27	G
19	I	34	U
19	I	36	G
19	I	46	G
19	I	51	G
19	I	60	G
19	I	63	A
19	I	71	A
19	I	73	A
19	I	74	A
19	I	75	G
19	I	88	G
19	I	92	U
19	I	93	G
19	I	99	U
19	I	100	U
19	I	101	A
19	I	103	A
19	I	118	A
19	I	120	U
19	I	121	G
19	I	122	G
19	I	125	A
19	I	131	A
19	I	137	U
19	I	139	U
19	I	140	C
19	I	141	G
19	I	148	U
19	I	152	A
19	I	160	A
19	I	163	C
19	I	165	A
19	I	167	A
19	I	196	A
19	I	199	A
19	I	216	A
19	I	218	A
19	I	221	A
19	I	222	A

Continued on next page...

For Manuscript Review

Continued from previous page...

Mol	Chain	Res	Type
19	I	228	C
19	I	230	G
19	I	233	A
19	I	248	G
19	I	264	C
19	I	265	A
19	I	266	G
19	I	267	C
19	I	268	C
19	I	271	G
19	I	272	A
19	I	275	C
19	I	277	G
19	I	278	A
19	I	279	A
19	I	284	U
19	I	285	G
19	I	290	U
19	I	291	G
19	I	311	A
19	I	330	A
19	I	338	G
19	I	353	C
19	I	364	C
19	I	371	A
19	I	372	G
19	I	377	G
19	I	386	G
19	I	396	G
19	I	404	A
19	I	406	G
19	I	411	G
19	I	413	C
19	I	435	C
19	I	457	A
19	I	477	A
19	I	481	G
19	I	491	G
19	I	492	A
19	I	503	A
19	I	505	A
19	I	509	C

Continued on next page...

For Manuscript Review

Continued from previous page...

Mol	Chain	Res	Type
19	I	529	A
19	I	530	G
19	I	531	C
19	I	532	A
19	I	543	G
19	I	546	U
19	I	547	A
19	I	548	G
19	I	549	G
19	I	550	C
19	I	563	A
19	I	571	U
19	I	573	U
19	I	575	A
19	I	596	U
19	I	597	G
19	I	603	A
19	I	607	U
19	I	613	A
19	I	615	U
19	I	627	A
19	I	637	A
19	I	642	U
19	I	643	A
19	I	645	C
19	I	646	U
19	I	647	G
19	I	654	A
19	I	655	A
19	I	668	A
19	I	686	U
19	I	705	A
19	I	706	A
19	I	713	G
19	I	714	U
19	I	715	A
19	I	717	C
19	I	726	G
19	I	730	A
19	I	746	PSU
19	I	747	5MU
19	I	757	G

Continued on next page...

For Manuscript Review

Continued from previous page...

Mol	Chain	Res	Type
19	I	764	A
19	I	765	C
19	I	774	G
19	I	775	G
19	I	776	G
19	I	782	A
19	I	784	G
19	I	785	G
19	I	789	A
19	I	805	G
19	I	812	C
19	I	819	A
19	I	827	U
19	I	839	U
19	I	840	C
19	I	844	A
19	I	845	A
19	I	846	U
19	I	850	U
19	I	851	C
19	I	858	G
19	I	859	G
19	I	872	U
19	I	873	C
19	I	877	A
19	I	882	G
19	I	884	U
19	I	894	U
19	I	895	U
19	I	896	A
19	I	897	C
19	I	907	G
19	I	910	A
19	I	914	G
19	I	915	C
19	I	927	A
19	I	932	U
19	I	946	C
19	I	953	G
19	I	961	C
19	I	974	G
19	I	983	A

Continued on next page...

For Manuscript Review

Continued from previous page...

Mol	Chain	Res	Type
19	I	996	A
19	I	999	U
19	I	1003	G
19	I	1005	C
19	I	1012	U
19	I	1013	C
19	I	1020	A
19	I	1021	A
19	I	1027	A
19	I	1028	A
19	I	1029	A
19	I	1030	C
19	I	1033	U
19	I	1034	G
19	I	1035	U
19	I	1036	G
19	I	1041	G
19	I	1046	A
19	I	1047	G
19	I	1051	G
19	I	1055	G
19	I	1057	A
19	I	1060	U
19	I	1061	U
19	I	1062	G
19	I	1064	C
19	I	1066	U
19	I	1067	A
19	I	1068	G
19	I	1069	A
19	I	1070	A
19	I	1071	G
19	I	1072	C
19	I	1073	A
19	I	1074	G
19	I	1076	C
19	I	1077	A
19	I	1079	C
19	I	1080	A
19	I	1081	U
19	I	1087	G
19	I	1097	U

Continued on next page...

For Manuscript Review

Continued from previous page...

Mol	Chain	Res	Type
19	I	1098	A
19	I	1109	C
19	I	1111	A
19	I	1112	G
19	I	1118	C
19	I	1119	U
19	I	1120	G
19	I	1132	U
19	I	1133	A
19	I	1135	C
19	I	1141	U
19	I	1142	A
19	I	1150	C
19	I	1151	A
19	I	1157	G
19	I	1170	C
19	I	1171	G
19	I	1172	C
19	I	1173	U
19	I	1174	U
19	I	1175	A
19	I	1176	U
19	I	1179	G
19	I	1180	U
19	I	1181	U
19	I	1236	G
19	I	1238	G
19	I	1250	G
19	I	1253	A
19	I	1256	G
19	I	1266	G
19	I	1267	U
19	I	1271	G
19	I	1272	A
19	I	1300	G
19	I	1301	A
19	I	1329	U
19	I	1341	G
19	I	1352	U
19	I	1365	A
19	I	1368	G
19	I	1379	U

Continued on next page...

For Manuscript Review

Continued from previous page...

Mol	Chain	Res	Type
19	I	1383	A
19	I	1386	C
19	I	1395	A
19	I	1407	G
19	I	1413	A
19	I	1416	G
19	I	1419	A
19	I	1420	A
19	I	1426	G
19	I	1427	A
19	I	1428	C
19	I	1437	C
19	I	1451	C
19	I	1452	G
19	I	1453	A
19	I	1455	G
19	I	1456	G
19	I	1458	U
19	I	1459	G
19	I	1461	C
19	I	1470	A
19	I	1482	G
19	I	1493	C
19	I	1494	A
19	I	1495	A
19	I	1497	U
19	I	1498	C
19	I	1515	A
19	I	1526	C
19	I	1535	A
19	I	1536	C
19	I	1537	G
19	I	1543	G
19	I	1554	U
19	I	1558	C
19	I	1566	A
19	I	1569	A
19	I	1570	A
19	I	1572	A
19	I	1577	C
19	I	1583	A
19	I	1585	C

Continued on next page...

For Manuscript Review

Continued from previous page...

Mol	Chain	Res	Type
19	I	1587	G
19	I	1608	A
19	I	1610	A
19	I	1618	6MZ
19	I	1630	A
19	I	1647	U
19	I	1648	U
19	I	1649	G
19	I	1674	G
19	I	1696	G
19	I	1715	G
19	I	1729	U
19	I	1730	C
19	I	1732	C
19	I	1733	G
19	I	1735	A
19	I	1736	U
19	I	1737	G
19	I	1738	G
19	I	1762	A
19	I	1763	G
19	I	1764	C
19	I	1773	A
19	I	1776	G
19	I	1782	U
19	I	1784	A
19	I	1786	A
19	I	1800	C
19	I	1801	A
19	I	1808	A
19	I	1816	C
19	I	1829	A
19	I	1847	A
19	I	1858	A
19	I	1866	A
19	I	1867	G
19	I	1868	C
19	I	1869	G
19	I	1870	C
19	I	1872	A
19	I	1873	G
19	I	1875	G

Continued on next page...

For Manuscript Review

Continued from previous page...

Mol	Chain	Res	Type
19	I	1903	G
19	I	1906	G
19	I	1913	A
19	I	1914	C
19	I	1915	3TD
19	I	1917	PSU
19	I	1929	G
19	I	1930	G
19	I	1936	A
19	I	1945	G
19	I	1954	G
19	I	1955	U
19	I	1962	5MC
19	I	1963	U
19	I	1966	A
19	I	1967	C
19	I	1970	A
19	I	1971	U
19	I	1972	G
19	I	1982	U
19	I	1991	U
19	I	1993	U
19	I	1997	C
19	I	2021	C
19	I	2023	C
19	I	2030	6MZ
19	I	2031	A
19	I	2033	A
19	I	2035	G
19	I	2043	C
19	I	2055	C
19	I	2056	G
19	I	2059	A
19	I	2060	A
19	I	2061	G
19	I	2069	G7M
19	I	2080	A
19	I	2093	G
19	I	2097	A
19	I	2102	G
19	I	2105	U
19	I	2108	A

Continued on next page...

For Manuscript Review

Continued from previous page...

Mol	Chain	Res	Type
19	I	2110	G
19	I	2111	U
19	I	2112	G
19	I	2116	G
19	I	2118	U
19	I	2119	A
19	I	2121	G
19	I	2122	U
19	I	2124	G
19	I	2125	G
19	I	2126	A
19	I	2127	G
19	I	2131	U
19	I	2132	U
19	I	2133	G
19	I	2138	G
19	I	2141	G
19	I	2144	G
19	I	2146	C
19	I	2148	G
19	I	2153	C
19	I	2158	A
19	I	2160	C
19	I	2161	C
19	I	2164	C
19	I	2165	C
19	I	2166	U
19	I	2167	U
19	I	2168	G
19	I	2169	A
19	I	2170	A
19	I	2171	A
19	I	2172	U
19	I	2174	C
19	I	2177	C
19	I	2178	C
19	I	2181	U
19	I	2183	A
19	I	2186	G
19	I	2190	G
19	I	2198	A
19	I	2204	G

Continued on next page...

For Manuscript Review

Continued from previous page...

Mol	Chain	Res	Type
19	I	2211	A
19	I	2225	A
19	I	2226	C
19	I	2238	G
19	I	2239	G
19	I	2279	G
19	I	2283	C
19	I	2286	G
19	I	2287	A
19	I	2290	G
19	I	2295	C
19	I	2299	U
19	I	2304	G
19	I	2305	U
19	I	2309	A
19	I	2311	A
19	I	2320	U
19	I	2322	A
19	I	2324	U
19	I	2330	G
19	I	2333	A
19	I	2334	U
19	I	2335	A
19	I	2336	A
19	I	2337	G
19	I	2338	C
19	I	2343	U
19	I	2346	A
19	I	2347	C
19	I	2349	G
19	I	2350	C
19	I	2354	C
19	I	2356	U
19	I	2357	G
19	I	2366	A
19	I	2372	U
19	I	2373	G
19	I	2375	G
19	I	2379	G
19	I	2380	C
19	I	2381	A
19	I	2382	G

Continued on next page...

For Manuscript Review

Continued from previous page...

Mol	Chain	Res	Type
19	I	2383	G
19	I	2385	C
19	I	2395	C
19	I	2396	G
19	I	2399	G
19	I	2406	A
19	I	2418	A
19	I	2419	U
19	I	2420	C
19	I	2423	U
19	I	2428	G
19	I	2429	G
19	I	2430	A
19	I	2435	A
19	I	2441	U
19	I	2448	A
19	I	2459	A
19	I	2470	G
19	I	2472	G
19	I	2473	U
19	I	2474	U
19	I	2475	C
19	I	2476	A
19	I	2478	A
19	I	2479	U
19	I	2491	U
19	I	2494	G
19	I	2498	OMC
19	I	2502	G
19	I	2505	G
19	I	2513	A
19	I	2518	A
19	I	2520	C
19	I	2524	G
19	I	2529	G
19	I	2530	A
19	I	2532	G
19	I	2542	A
19	I	2547	A
19	I	2554	U
19	I	2566	A
19	I	2567	G

Continued on next page...

For Manuscript Review

Continued from previous page...

Mol	Chain	Res	Type
19	I	2572	A
19	I	2602	A
19	I	2609	U
19	I	2613	U
19	I	2615	U
19	I	2626	C
19	I	2627	G
19	I	2629	U
19	I	2630	G
19	I	2631	G
19	I	2636	C
19	I	2646	C
19	I	2648	G
19	I	2654	A
19	I	2669	G
19	I	2673	G
19	I	2689	U
19	I	2690	U
19	I	2702	G
19	I	2703	C
19	I	2714	G
19	I	2718	G
19	I	2725	A
19	I	2726	A
19	I	2732	G
19	I	2733	A
19	I	2739	U
19	I	2740	A
19	I	2744	G
19	I	2748	A
19	I	2752	C
19	I	2753	A
19	I	2754	U
19	I	2755	C
19	I	2757	A
19	I	2764	A
19	I	2776	A
19	I	2777	G
19	I	2778	A
19	I	2789	C
19	I	2791	G
19	I	2793	C

Continued on next page...

For Manuscript Review

Continued from previous page...

Mol	Chain	Res	Type
19	I	2797	U
19	I	2798	U
19	I	2800	A
19	I	2801	G
19	I	2818	U
19	I	2820	A
19	I	2833	U
19	I	2837	A
19	I	2849	U
19	I	2852	G
19	I	2869	G
19	I	2872	A
19	I	2873	A
19	I	2880	C
19	I	2884	U
19	I	2895	G
19	I	2903	U
21	J	9	G
21	J	15	A
21	J	16	G
21	J	24	G
21	J	31	C
21	J	32	U
21	J	35	C
21	J	39	A
21	J	42	C
21	J	45	A
21	J	48	U
21	J	50	A
21	J	52	A
21	J	56	G
21	J	57	A
21	J	66	A
21	J	80	U
21	J	81	G
21	J	88	C
21	J	89	U
21	J	90	C
21	J	92	C
21	J	99	A
21	J	109	A

All (31) RNA pucker outliers are listed below:

For Manuscript Review

Mol	Chain	Res	Type
1	C	81	U
1	C	83	G
1	C	94	C
1	C	171	G
1	C	209	C
1	C	260	G
1	C	265	C
1	C	322	C
1	C	339	C
1	C	379	C
1	C	431	U
1	C	506	U
1	C	512	C
1	C	806	G
1	C	836	U
1	C	1195	A
1	C	1233	A
1	C	1268	G
1	C	1340	A
1	C	1341	G
19	I	784	G
19	I	1046	A
19	I	1070	A
19	I	1117	C
19	I	1542	U
19	I	1865	U
19	I	1871	A
19	I	1962	5MC
19	I	2170	A
19	I	2349	G
19	I	2756	U

5.4 Non-standard residues in protein, DNA, RNA chains (i)

25 non-standard protein/DNA/RNA residues are modelled in this entry.

In the following table, the Counts columns list the number of bonds (or angles) for which Mogul statistics could be retrieved, the number of bonds (or angles) that are observed in the model and the number of bonds (or angles) that are defined in the Chemical Component Dictionary. The Link column lists molecule types, if any, to which the group is linked. The Z score for a bond length (or angle) is the number of standard deviations the observed value is removed from the expected value. A bond length (or angle) with $|Z| > 2$ is considered an outlier worth inspection. RMSZ is the root-mean-square of all Z scores of the bond lengths (or angles).

For Manuscript Review

Mol	Type	Chain	Res	Link	Bond lengths			Bond angles		
					Counts	RMSZ	#|Z| > 2	Counts	RMSZ	#|Z| > 2
19	5MU	I	1939	19	19,22,23	1.05	3 (15%)	27,32,35	2.14	6 (22%)
19	G7M	I	2069	19	20,26,27	0.94	1 (5%)	16,39,42	0.60	0
43	4D4	U	81	43	9,11,12	0.88	0	7,13,15	1.75	2 (28%)
19	OMG	I	2251	19	19,26,27	1.20	2 (10%)	21,38,41	1.45	4 (19%)
19	H2U	I	2449	19	18,21,22	0.45	0	19,30,33	1.10	1 (5%)
19	PSU	I	2605	19	18,21,22	0.95	0	21,30,33	1.90	4 (19%)
19	6MZ	I	1618	19	17,25,26	1.17	2 (11%)	15,36,39	2.14	3 (20%)
19	PSU	I	746	53,19	18,21,22	1.00	2 (11%)	21,30,33	1.88	4 (19%)
19	2MG	I	2445	19	18,26,27	1.28	2 (11%)	16,38,41	1.55	4 (25%)
19	PSU	I	1917	19	18,21,22	0.91	0	21,30,33	1.91	5 (23%)
19	2MG	I	1835	19	18,26,27	1.27	2 (11%)	16,38,41	1.46	3 (18%)
19	OMU	I	2552	19	19,22,23	0.97	2 (10%)	25,31,34	1.93	6 (24%)
19	6MZ	I	2030	19	17,25,26	1.16	2 (11%)	15,36,39	2.47	4 (26%)
19	PSU	I	955	19	18,21,22	0.95	0	21,30,33	1.95	4 (19%)
19	PSU	I	2580	19	18,21,22	1.07	1 (5%)	21,30,33	1.99	4 (19%)
19	3TD	I	1915	19	19,22,23	1.06	2 (10%)	23,32,35	1.95	3 (13%)
19	5MU	I	747	19	19,22,23	1.02	2 (10%)	27,32,35	2.10	5 (18%)
19	5MC	I	1962	19	19,22,23	1.25	2 (10%)	26,32,35	1.13	2 (7%)
19	OMC	I	2498	53,19	19,22,23	0.89	1 (5%)	25,31,34	1.07	2 (8%)
19	PSU	I	2604	19	18,21,22	0.93	0	21,30,33	1.87	4 (19%)
19	1MG	I	745	19	19,26,27	1.26	3 (15%)	18,39,42	1.38	2 (11%)
19	PSU	I	2504	19	18,21,22	0.92	1 (5%)	21,30,33	2.02	4 (19%)
19	PSU	I	2457	19	18,21,22	0.96	0	21,30,33	1.98	5 (23%)
19	PSU	I	1911	19	18,21,22	0.91	1 (5%)	21,30,33	1.96	4 (19%)
19	2MA	I	2503	53,19	18,25,26	1.29	1 (5%)	20,37,40	1.99	3 (15%)

In the following table, the Chirals column lists the number of chiral outliers, the number of chiral centers analysed, the number of these observed in the model and the number defined in the Chemical Component Dictionary. Similar counts are reported in the Torsion and Rings columns. '-' means no outliers of that kind were identified.

Mol	Type	Chain	Res	Link	Chirals	Torsions	Rings
19	5MU	I	1939	19	-	0/7/25/26	0/2/2/2
19	G7M	I	2069	19	-	0/3/25/26	0/3/3/3
43	4D4	U	81	43	-	1/11/12/14	-
19	OMG	I	2251	19	-	0/5/27/28	0/3/3/3
19	H2U	I	2449	19	-	0/7/38/39	0/2/2/2

Continued on next page...

For Manuscript Review

Continued from previous page...

Mol	Type	Chain	Res	Link	Chirals	Torsions	Rings
19	PSU	I	2605	19	-	0/7/25/26	0/2/2/2
19	6MZ	I	1618	19	-	2/5/27/28	0/3/3/3
19	PSU	I	746	53,19	-	1/7/25/26	0/2/2/2
19	2MG	I	2445	19	-	1/5/27/28	0/3/3/3
19	PSU	I	1917	19	-	2/7/25/26	0/2/2/2
19	2MG	I	1835	19	-	1/5/27/28	0/3/3/3
19	OMU	I	2552	19	-	0/9/27/28	0/2/2/2
19	6MZ	I	2030	19	-	2/5/27/28	0/3/3/3
19	PSU	I	955	19	-	0/7/25/26	0/2/2/2
19	PSU	I	2580	19	-	1/7/25/26	0/2/2/2
19	3TD	I	1915	19	-	5/7/25/26	0/2/2/2
19	5MU	I	747	19	-	0/7/25/26	0/2/2/2
19	5MC	I	1962	19	-	0/7/25/26	0/2/2/2
19	OMC	I	2498	53,19	-	0/9/27/28	0/2/2/2
19	PSU	I	2604	19	-	0/7/25/26	0/2/2/2
19	1MG	I	745	19	-	0/3/25/26	0/3/3/3
19	PSU	I	2504	19	-	1/7/25/26	0/2/2/2
19	PSU	I	2457	19	-	0/7/25/26	0/2/2/2
19	PSU	I	1911	19	-	0/7/25/26	0/2/2/2
19	2MA	I	2503	53,19	-	3/3/25/26	0/3/3/3

All (32) bond length outliers are listed below:

Mol	Chain	Res	Type	Atoms	Z	Observed(Å)	Ideal(Å)
19	I	1962	5MC	C5-C4	-4.02	1.41	1.44
19	I	2503	2MA	C6-N1	3.59	1.40	1.33
19	I	745	1MG	C8-N7	-3.03	1.30	1.34
19	I	1835	2MG	C5-C6	-2.97	1.41	1.47
19	I	2445	2MG	C5-C6	-2.95	1.41	1.47
19	I	2251	OMG	C5-C6	-2.91	1.41	1.47
19	I	2069	G7M	C8-N9	2.88	1.38	1.33
19	I	1835	2MG	C8-N7	-2.86	1.30	1.34
19	I	2445	2MG	C8-N7	-2.84	1.30	1.34
19	I	2251	OMG	C8-N7	-2.82	1.30	1.34
19	I	1618	6MZ	C6-C5	-2.75	1.40	1.44
19	I	2030	6MZ	C6-C5	-2.69	1.40	1.44
19	I	2580	PSU	O4'-C1'	-2.68	1.40	1.43
19	I	745	1MG	C6-N1	-2.57	1.34	1.39
19	I	1939	5MU	C2-N1	-2.54	1.34	1.38
19	I	1962	5MC	C2-N1	-2.53	1.34	1.40
19	I	2498	OMC	C2-N1	-2.52	1.34	1.40

Continued on next page...

For Manuscript Review

Continued from previous page...

Mol	Chain	Res	Type	Atoms	Z	Observed(Å)	Ideal(Å)
19	I	1915	3TD	C4-N3	-2.52	1.35	1.40
19	I	2552	OMU	C2-N1	-2.46	1.34	1.38
19	I	1618	6MZ	C8-N7	-2.34	1.30	1.34
19	I	747	5MU	C2-N1	-2.34	1.34	1.38
19	I	746	PSU	O4'-C1'	-2.23	1.40	1.43
19	I	2030	6MZ	C8-N7	-2.17	1.30	1.34
19	I	1915	3TD	C4-C5	-2.14	1.42	1.47
19	I	747	5MU	C4-N3	-2.11	1.34	1.38
19	I	2552	OMU	C4-N3	-2.10	1.35	1.38
19	I	1939	5MU	C4-N3	-2.08	1.35	1.38
19	I	2504	PSU	C4-N3	-2.07	1.35	1.38
19	I	745	1MG	C5-C6	-2.04	1.41	1.47
19	I	1939	5MU	C4-C5	-2.03	1.41	1.44
19	I	746	PSU	C4-N3	-2.02	1.35	1.38
19	I	1911	PSU	C4-N3	-2.01	1.35	1.38

All (88) bond angle outliers are listed below:

Mol	Chain	Res	Type	Atoms	Z	Observed(^{o})	Ideal(^{o})
19	I	1915	3TD	N1-C2-N3	6.47	120.84	116.13
19	I	2030	6MZ	C2-N1-C6	5.96	121.23	116.60
19	I	1618	6MZ	C2-N1-C6	5.79	121.09	116.60
19	I	2503	2MA	C2-N3-C4	5.74	120.09	115.46
19	I	2504	PSU	N1-C2-N3	5.66	121.13	115.17
19	I	955	PSU	N1-C2-N3	5.48	120.94	115.17
19	I	1939	5MU	C4-N3-C2	-5.47	120.17	127.34
19	I	1911	PSU	N1-C2-N3	5.45	120.92	115.17
19	I	2457	PSU	N1-C2-N3	5.44	120.91	115.17
19	I	2580	PSU	N1-C2-N3	5.42	120.88	115.17
19	I	746	PSU	N1-C2-N3	5.42	120.88	115.17
19	I	1917	PSU	N1-C2-N3	5.37	120.84	115.17
19	I	2503	2MA	C5-C6-N1	-5.35	114.51	120.84
19	I	747	5MU	C4-N3-C2	-5.29	120.40	127.34
19	I	2604	PSU	N1-C2-N3	5.21	120.67	115.17
19	I	2605	PSU	N1-C2-N3	5.13	120.58	115.17
19	I	2552	OMU	C4-N3-C2	-5.13	120.24	126.61
19	I	2030	6MZ	C9-N6-C6	-5.01	118.20	122.85
19	I	2552	OMU	N3-C2-N1	4.86	121.22	114.89
19	I	1939	5MU	C5-C4-N3	4.75	119.46	115.32
19	I	1939	5MU	N3-C2-N1	4.73	121.04	114.89
19	I	2030	6MZ	N3-C2-N1	-4.72	122.26	128.67
19	I	747	5MU	N3-C2-N1	4.64	120.94	114.89

Continued on next page...

For Manuscript Review

Continued from previous page...

Mol	Chain	Res	Type	Atoms	Z	Observed(°)	Ideal(°)
19	I	1618	6MZ	N3-C2-N1	-4.62	122.40	128.67
19	I	747	5MU	C5-C4-N3	4.56	119.28	115.32
19	I	2504	PSU	C4-N3-C2	-4.54	120.12	126.37
19	I	1911	PSU	C4-N3-C2	-4.46	120.23	126.37
19	I	2457	PSU	C4-N3-C2	-4.44	120.25	126.37
19	I	955	PSU	C4-N3-C2	-4.43	120.27	126.37
19	I	2605	PSU	C4-N3-C2	-4.36	120.36	126.37
19	I	746	PSU	C4-N3-C2	-4.34	120.39	126.37
19	I	1917	PSU	C4-N3-C2	-4.26	120.50	126.37
19	I	2604	PSU	C4-N3-C2	-4.21	120.57	126.37
19	I	1939	5MU	O4-C4-C5	-4.18	120.13	124.92
19	I	2580	PSU	C4-N3-C2	-4.16	120.65	126.37
19	I	1915	3TD	C1'-C5-C4	4.03	123.72	117.61
19	I	747	5MU	O4-C4-C5	-4.02	120.32	124.92
19	I	745	1MG	C8-N7-C5	3.97	109.31	102.55
19	I	2251	OMG	C8-N7-C5	3.97	109.31	102.55
19	I	1835	2MG	C8-N7-C5	3.90	109.19	102.55
19	I	2445	2MG	C8-N7-C5	3.88	109.16	102.55
19	I	2580	PSU	O2-C2-N1	-3.84	118.82	122.79
19	I	1915	3TD	C4-N3-C2	-3.75	120.65	124.61
19	I	2449	H2U	C5-C4-N3	-3.64	112.81	116.69
19	I	1962	5MC	C5-C6-N1	-3.53	119.47	123.31
19	I	1939	5MU	C5-C6-N1	-3.53	119.48	123.31
43	U	81	4D4	NE-CZ-NH2	3.43	126.56	120.67
19	I	2457	PSU	O2-C2-N1	-3.40	119.28	122.79
19	I	2605	PSU	O2-C2-N1	-3.27	119.42	122.79
19	I	2552	OMU	C5-C4-N3	3.23	119.33	114.80
19	I	747	5MU	C5-C6-N1	-3.23	119.81	123.31
19	I	955	PSU	O2-C2-N1	-3.16	119.53	122.79
19	I	1911	PSU	O2-C2-N1	-3.11	119.58	122.79
19	I	2604	PSU	O2-C2-N1	-3.11	119.58	122.79
19	I	746	PSU	O2-C2-N1	-3.08	119.61	122.79
19	I	745	1MG	C5-C6-N1	3.06	118.39	113.96
19	I	1917	PSU	O2-C2-N1	-3.05	119.64	122.79
19	I	2552	OMU	CM2-O2'-C2'	-3.05	106.64	114.47
19	I	2504	PSU	O2-C2-N1	-3.02	119.67	122.79
19	I	2498	OMC	CM2-O2'-C2'	-2.96	106.89	114.47
19	I	2251	OMG	CM2-O2'-C2'	-2.85	107.15	114.47
19	I	2552	OMU	O4-C4-C5	-2.80	120.34	125.16
19	I	1939	5MU	O2-C2-N1	-2.74	119.23	122.80
19	I	2580	PSU	C6-N1-C2	-2.74	120.15	122.69
19	I	1618	6MZ	C9-N6-C6	-2.69	120.35	122.85

Continued on next page...

For Manuscript Review

Continued from previous page...

Mol	Chain	Res	Type	Atoms	Z	Observed(°)	Ideal(°)
19	I	2251	OMG	C2-N1-C6	-2.65	120.25	125.11
19	I	2445	2MG	N1-C2-N2	2.65	119.26	116.56
19	I	2552	OMU	O2-C2-N1	-2.53	119.51	122.80
19	I	1917	PSU	C6-N1-C2	-2.42	120.45	122.69
19	I	2504	PSU	C6-N1-C2	-2.41	120.46	122.69
19	I	2498	OMC	O2-C2-N3	-2.39	118.56	122.33
19	I	2030	6MZ	C4-C5-N7	-2.36	106.84	109.34
19	I	746	PSU	C6-N1-C2	-2.35	120.51	122.69
19	I	955	PSU	C6-N1-C2	-2.35	120.51	122.69
19	I	2604	PSU	C6-N1-C2	-2.34	120.52	122.69
43	U	81	4D4	NH1-CZ-NE	-2.33	113.97	119.27
19	I	2445	2MG	CM2-N2-C2	-2.31	118.69	123.65
19	I	2457	PSU	C6-N1-C2	-2.31	120.55	122.69
19	I	2251	OMG	C5-C6-N1	2.28	118.42	114.07
19	I	2605	PSU	C6-N1-C2	-2.27	120.59	122.69
19	I	1835	2MG	C5-C6-N1	2.26	118.38	114.07
19	I	2445	2MG	C5-C6-N1	2.23	118.32	114.07
19	I	1835	2MG	N1-C2-N2	2.22	118.82	116.56
19	I	1911	PSU	C6-N1-C2	-2.21	120.64	122.69
19	I	2457	PSU	O4'-C1'-C2'	2.17	108.15	105.15
19	I	2503	2MA	N6-C6-N1	2.06	122.65	117.11
19	I	1962	5MC	C5-C4-N3	-2.05	119.65	121.75
19	I	1917	PSU	O4'-C1'-C2'	2.05	107.98	105.15

There are no chirality outliers.

All (20) torsion outliers are listed below:

Mol	Chain	Res	Type	Atoms
19	I	746	PSU	O4'-C1'-C5-C6
19	I	1618	6MZ	O4'-C4'-C5'-O5'
19	I	1618	6MZ	C3'-C4'-C5'-O5'
19	I	1915	3TD	O4'-C1'-C5-C4
19	I	1915	3TD	O4'-C1'-C5-C6
19	I	1915	3TD	O4'-C4'-C5'-O5'
19	I	1917	PSU	O4'-C4'-C5'-O5'
19	I	1915	3TD	C3'-C4'-C5'-O5'
19	I	1917	PSU	C3'-C4'-C5'-O5'
19	I	2030	6MZ	O4'-C4'-C5'-O5'
19	I	2030	6MZ	C3'-C4'-C5'-O5'
19	I	2503	2MA	O4'-C4'-C5'-O5'
19	I	2503	2MA	C3'-C4'-C5'-O5'
19	I	2504	PSU	O4'-C4'-C5'-O5'

Continued on next page...

For Manuscript Review

Continued from previous page...

Mol	Chain	Res	Type	Atoms
19	I	2445	2MG	C3'-C4'-C5'-O5'
19	I	2580	PSU	O4'-C4'-C5'-O5'
43	U	81	4D4	OB-CB-CG-CD
19	I	1915	3TD	C2'-C1'-C5-C6
19	I	2503	2MA	C4'-C5'-O5'-P
19	I	1835	2MG	C3'-C4'-C5'-O5'

There are no ring outliers.

10 monomers are involved in 13 short contacts:

Mol	Chain	Res	Type	Clashes	Symm-Clashes
19	I	1939	5MU	1	0
19	I	2449	H2U	1	0
19	I	2605	PSU	1	0
19	I	2445	2MG	2	0
19	I	2030	6MZ	3	0
19	I	2580	PSU	1	0
19	I	1962	5MC	2	0
19	I	2604	PSU	1	0
19	I	745	1MG	1	0
19	I	2503	2MA	1	0

5.5 Carbohydrates (i)

There are no oligosaccharides in this entry.

5.6 Ligand geometry (i)

Of 307 ligands modelled in this entry, 307 are monoatomic - leaving 0 for Mogul analysis.

There are no bond length outliers.

There are no bond angle outliers.

There are no chirality outliers.

There are no torsion outliers.

There are no ring outliers.

No monomer is involved in short contacts.

For Manuscript Review

5.7 Other polymers (i)

There are no such residues in this entry.

5.8 Polymer linkage issues (i)

There are no chain breaks in this entry.

For Manuscript Review

6 Map visualisation (i)

This section contains visualisations of the EMDB entry EMD-75676. These allow visual inspection of the internal detail of the map and identification of artifacts.

Images derived from a raw map, generated by summing the deposited half-maps, are presented below the corresponding image components of the primary map to allow further visual inspection and comparison with those of the primary map.

6.1 Orthogonal projections (i)

6.1.1 Primary map

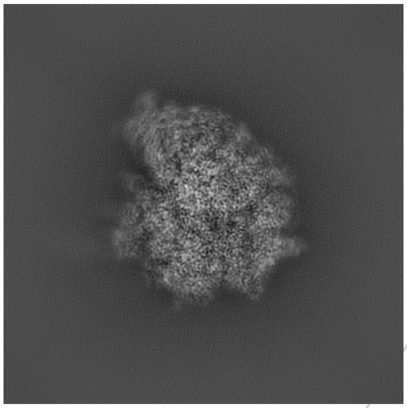
X

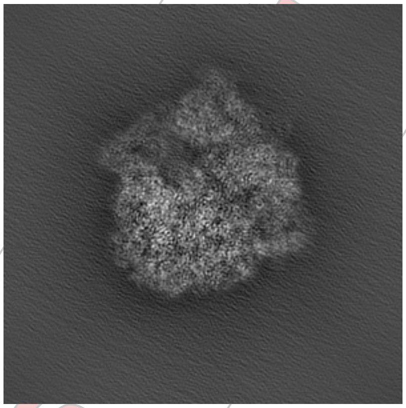
Y

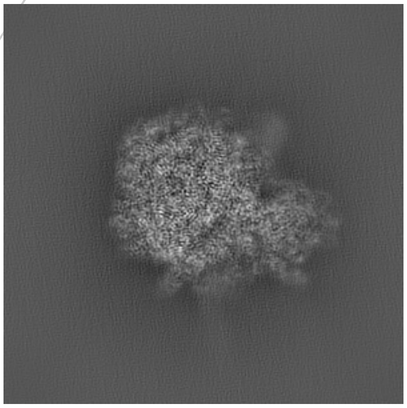
Z

6.1.2 Raw map

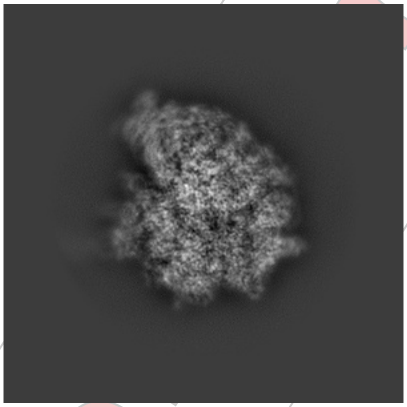
X

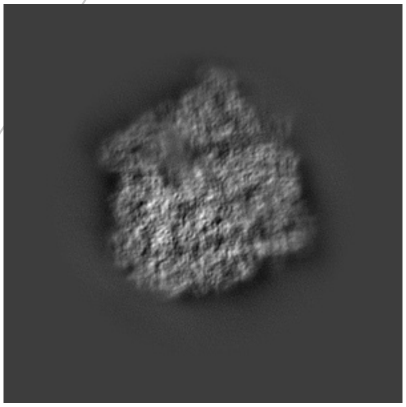
Y

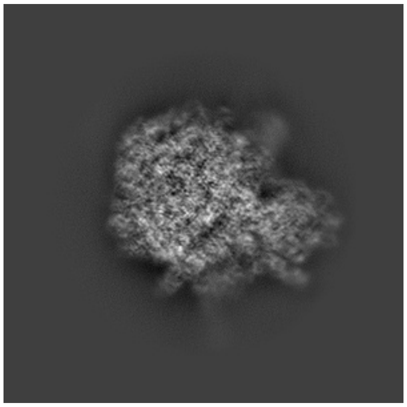
Z

The images above show the map projected in three orthogonal directions.

For Manuscript Review

6.2 Central slices (i)

6.2.1 Primary map

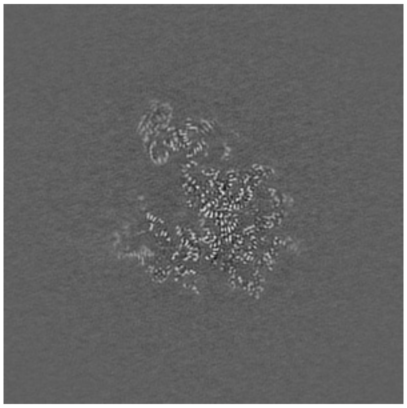
X Index: 256

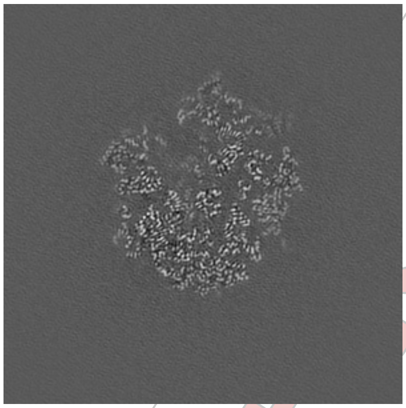
Y Index: 256

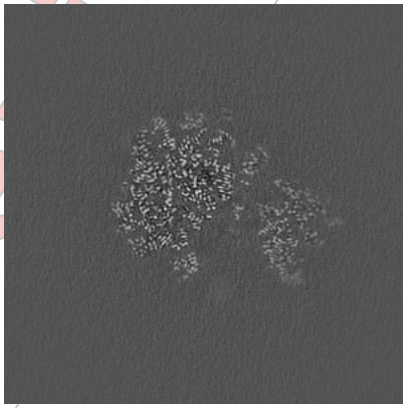
Z Index: 256

6.2.2 Raw map

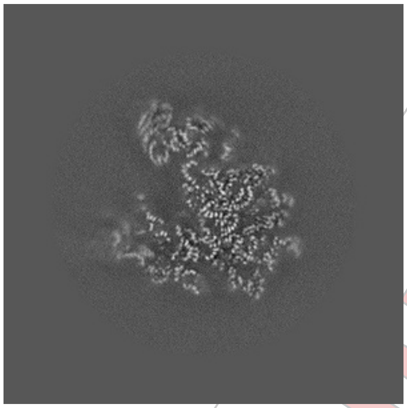
X Index: 256

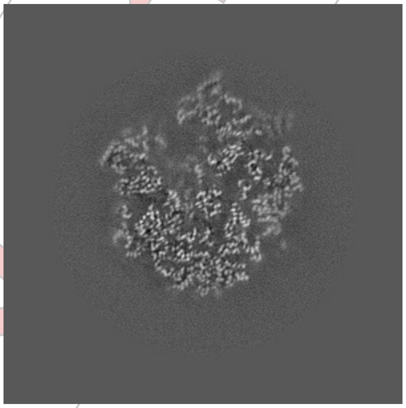
Y Index: 256

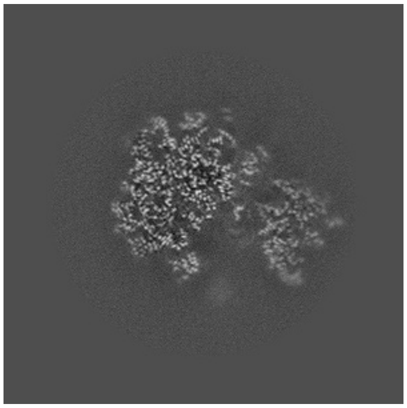
Z Index: 256

The images above show central slices of the map in three orthogonal directions.

For Manuscript Review

6.3 Largest variance slices (i)

6.3.1 Primary map

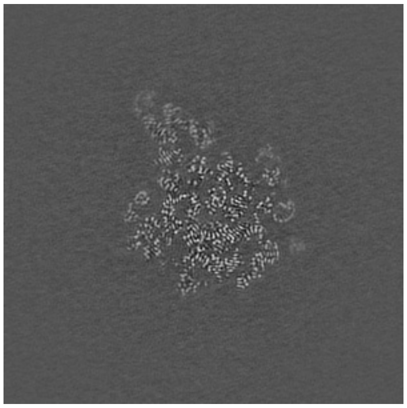

X Index: 234

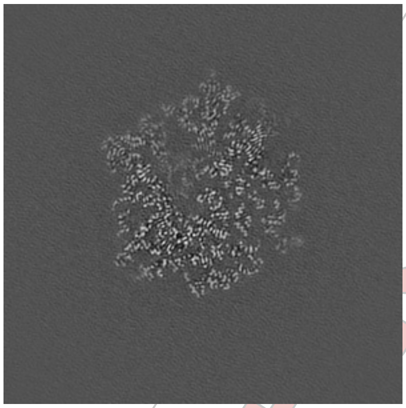

Y Index: 247

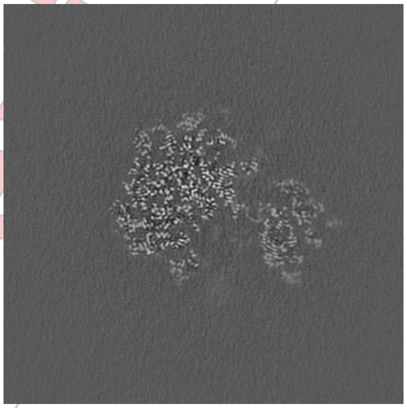

Z Index: 253

6.3.2 Raw map

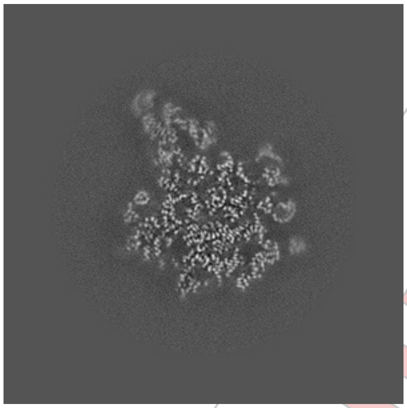

X Index: 234

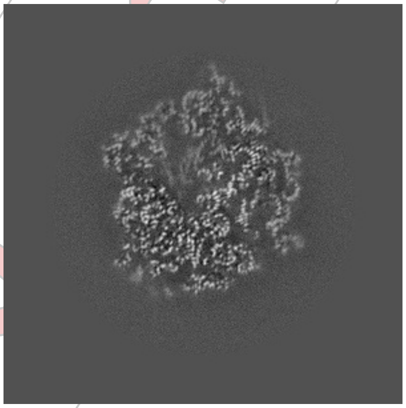

Y Index: 241

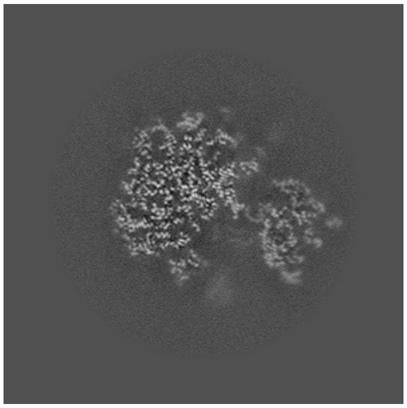

Z Index: 253

The images above show the largest variance slices of the map in three orthogonal directions.

For Manuscript Review

6.4 Orthogonal standard-deviation projections (False-color) (i)

6.4.1 Primary map

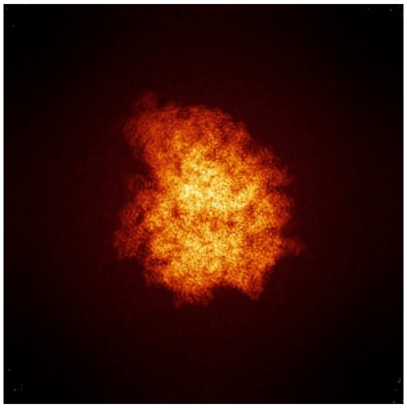

X

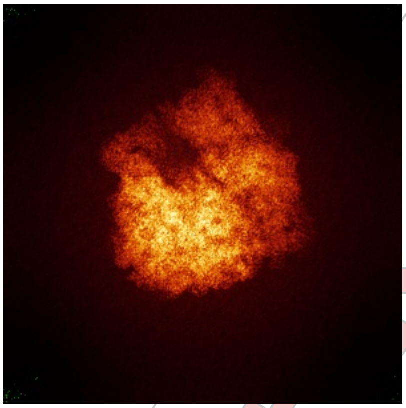

Y

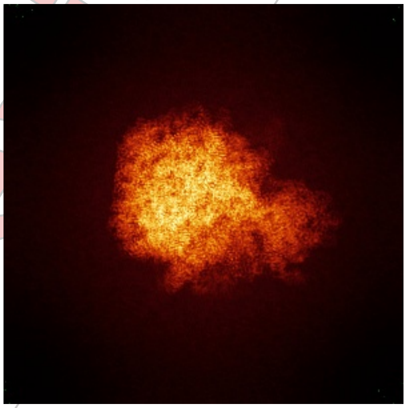

Z

6.4.2 Raw map

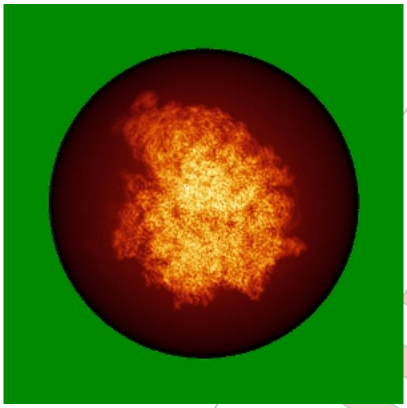

X

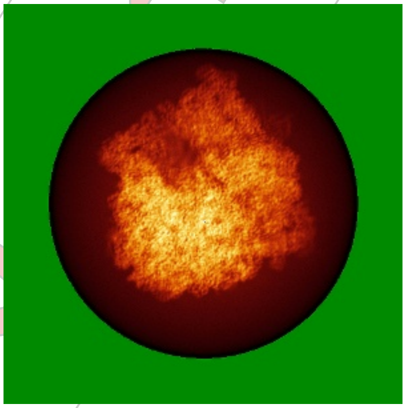

Y

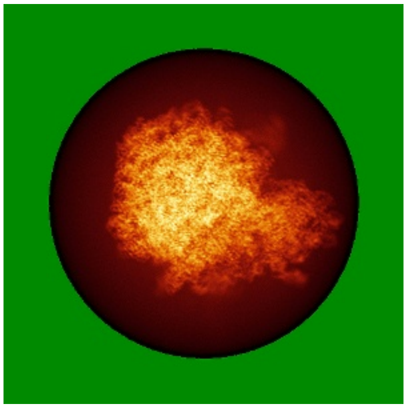

Z

The images above show the map standard deviation projections with false color in three orthogonal directions. Minimum values are shown in green, max in blue, and dark to light orange shades represent small to large values respectively.

For Manuscript Review

6.5 Orthogonal surface views (i)

6.5.1 Primary map

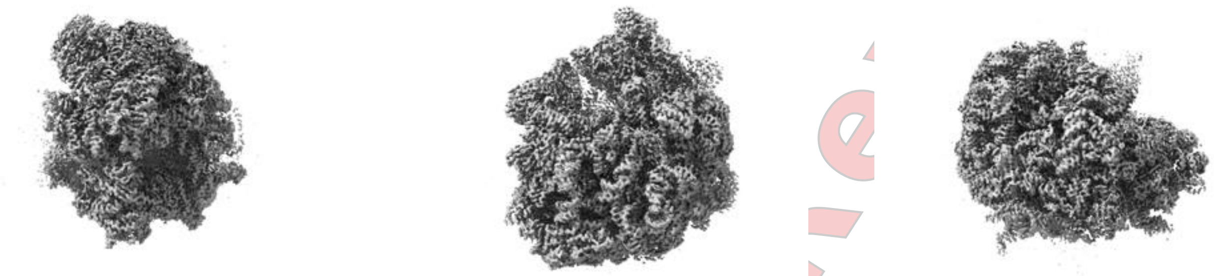

X Y Z

The images above show the 3D surface view of the map at the recommended contour level 3.0. These images, in conjunction with the slice images, may facilitate assessment of whether an appropriate contour level has been provided.

6.5.2 Raw map

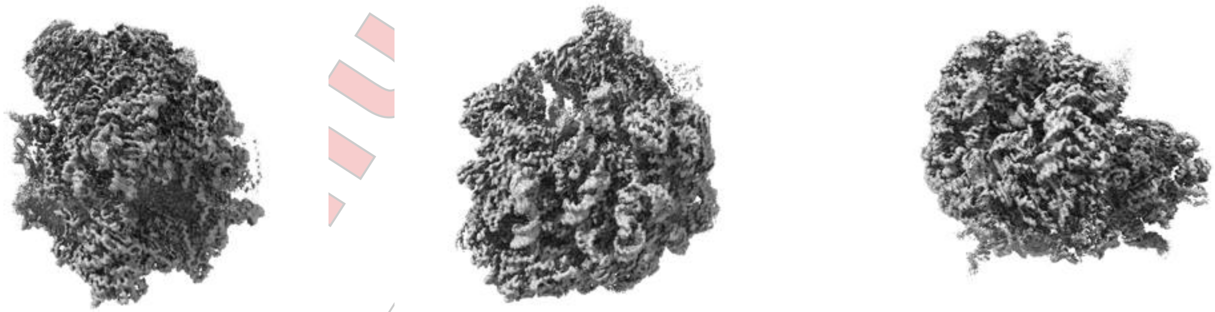

X Y Z

These images show the 3D surface of the raw map. The raw map's contour level was selected so that its surface encloses the same volume as the primary map does at its recommended contour level.

6.6 Mask visualisation (i)

This section was not generated. No masks/segmentation were deposited.

For Manuscript Review

7 Map analysis (i)

This section contains the results of statistical analysis of the map.

7.1 Map-value distribution (i)

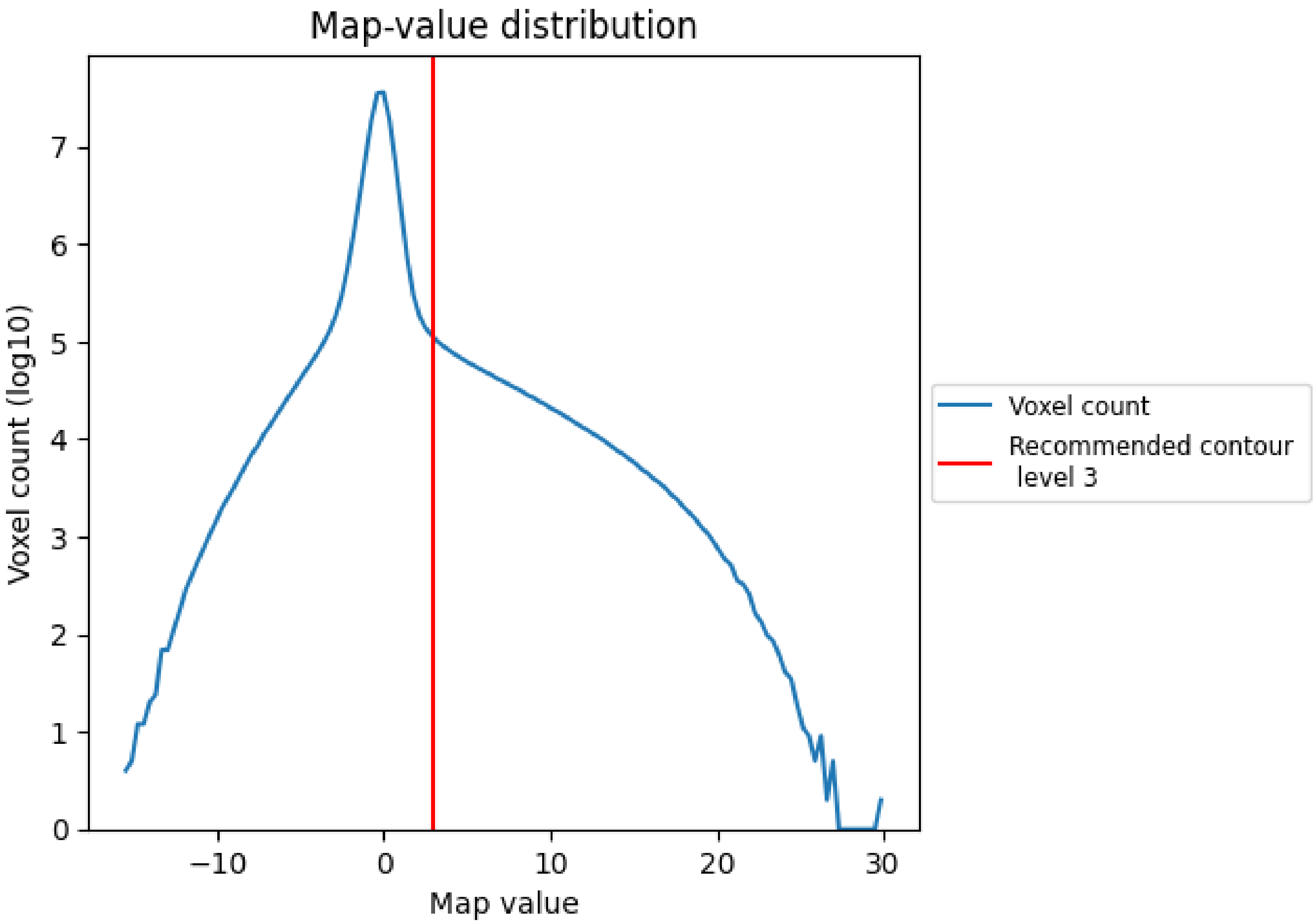

The map-value distribution is plotted in 128 intervals along the x-axis. The y-axis is logarithmic. A spike in this graph at zero usually indicates that the volume has been masked.

For Manuscript Review

7.2 Volume estimate ⓘ

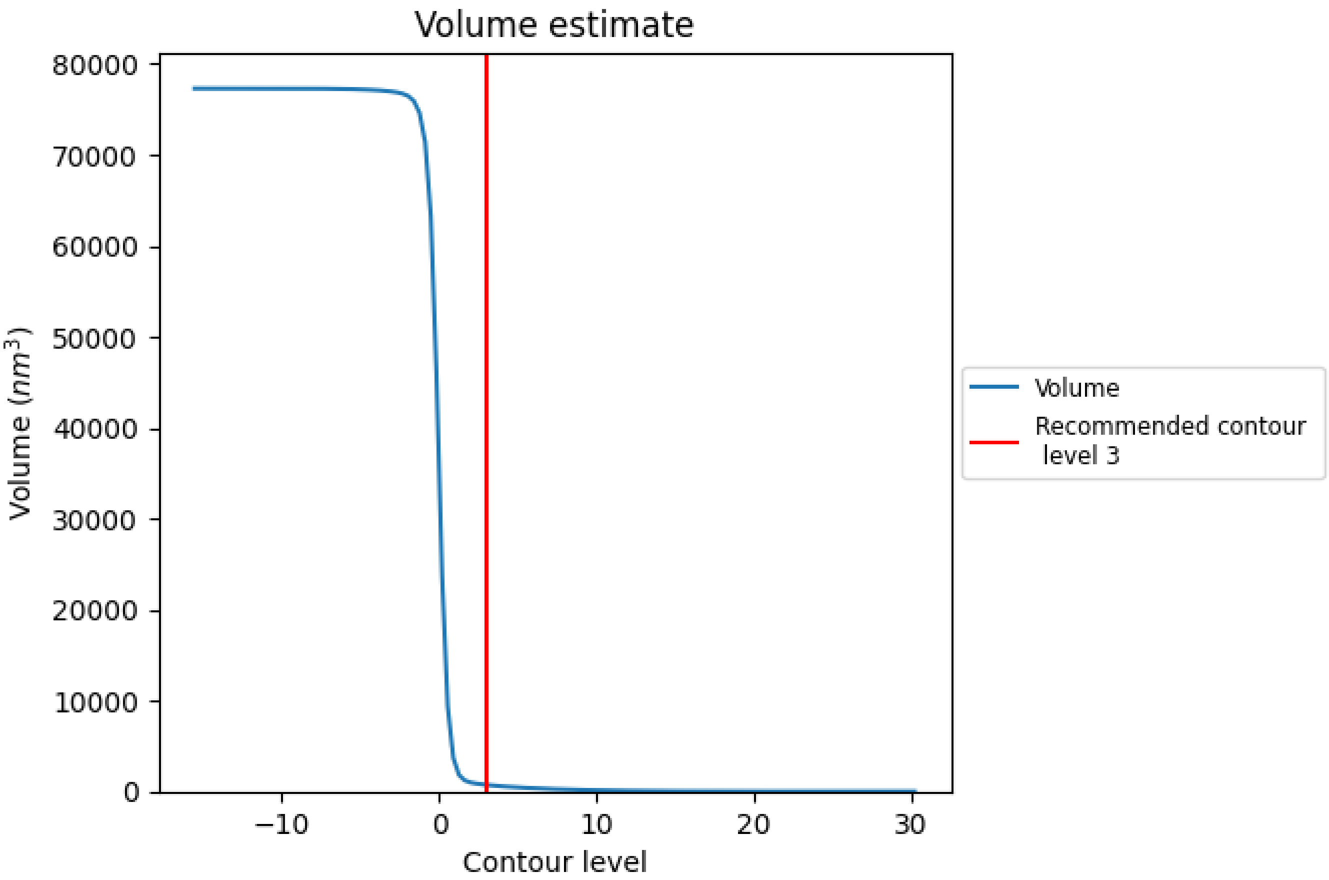

The volume at the recommended contour level is 720 nm^3; this corresponds to an approximate mass of 651 kDa.

The volume estimate graph shows how the enclosed volume varies with the contour level. The recommended contour level is shown as a vertical line and the intersection between the line and the curve gives the volume of the enclosed surface at the given level.

For Manuscript Review

7.3 Rotationally averaged power spectrum (i)

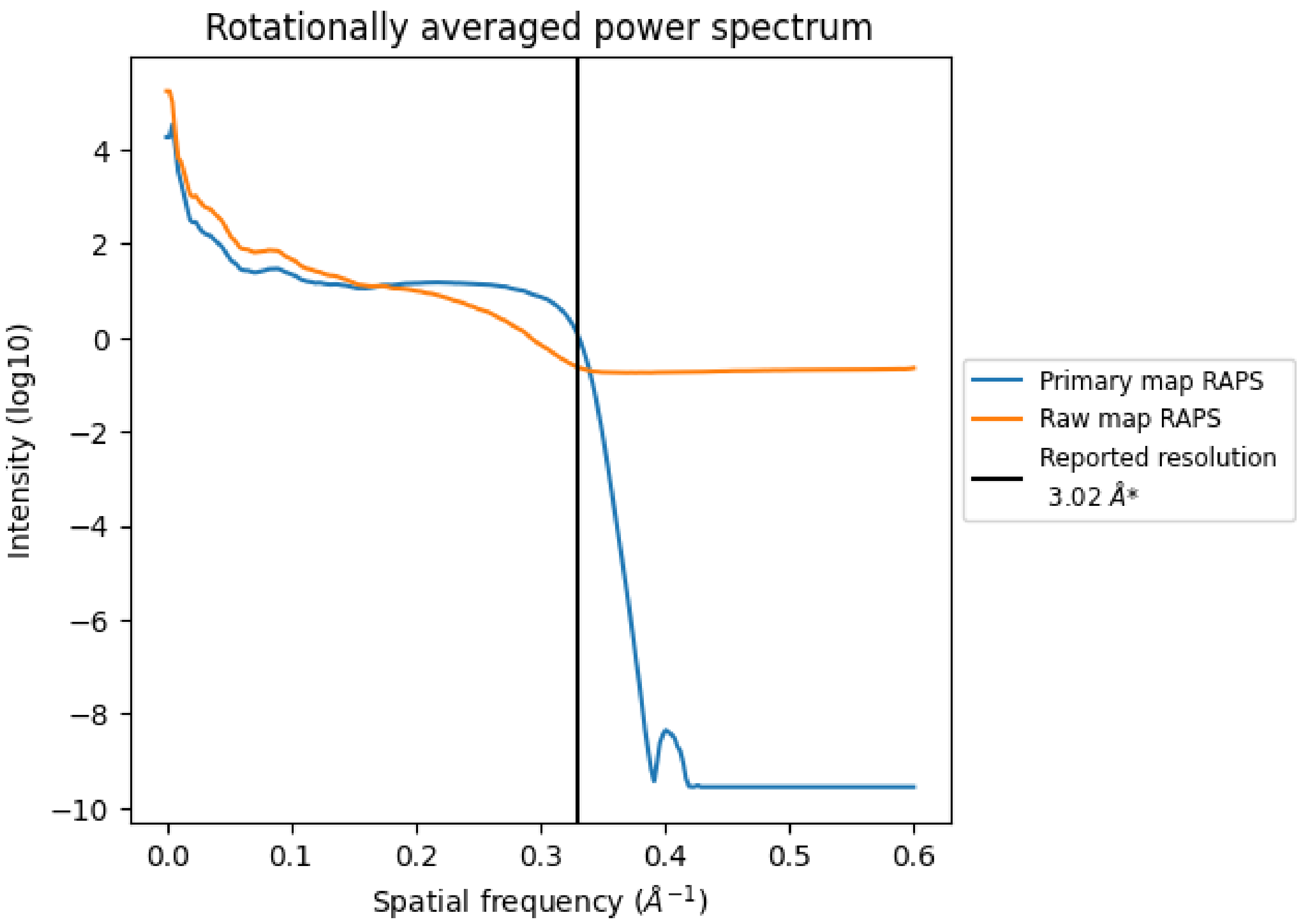

*Reported resolution corresponds to spatial frequency of 0.331 Å^{-1}

For Manuscript Review

8 Fourier-Shell correlation (i)

Fourier-Shell Correlation (FSC) is the most commonly used method to estimate the resolution of single-particle and subtomogram-averaged maps. The shape of the curve depends on the imposed symmetry, mask and whether or not the two 3D reconstructions used were processed from a common reference. The reported resolution is shown as a black line. A curve is displayed for the half-bit criterion in addition to lines showing the 0.143 gold standard cut-off and 0.5 cut-off.

8.1 FSC (i)

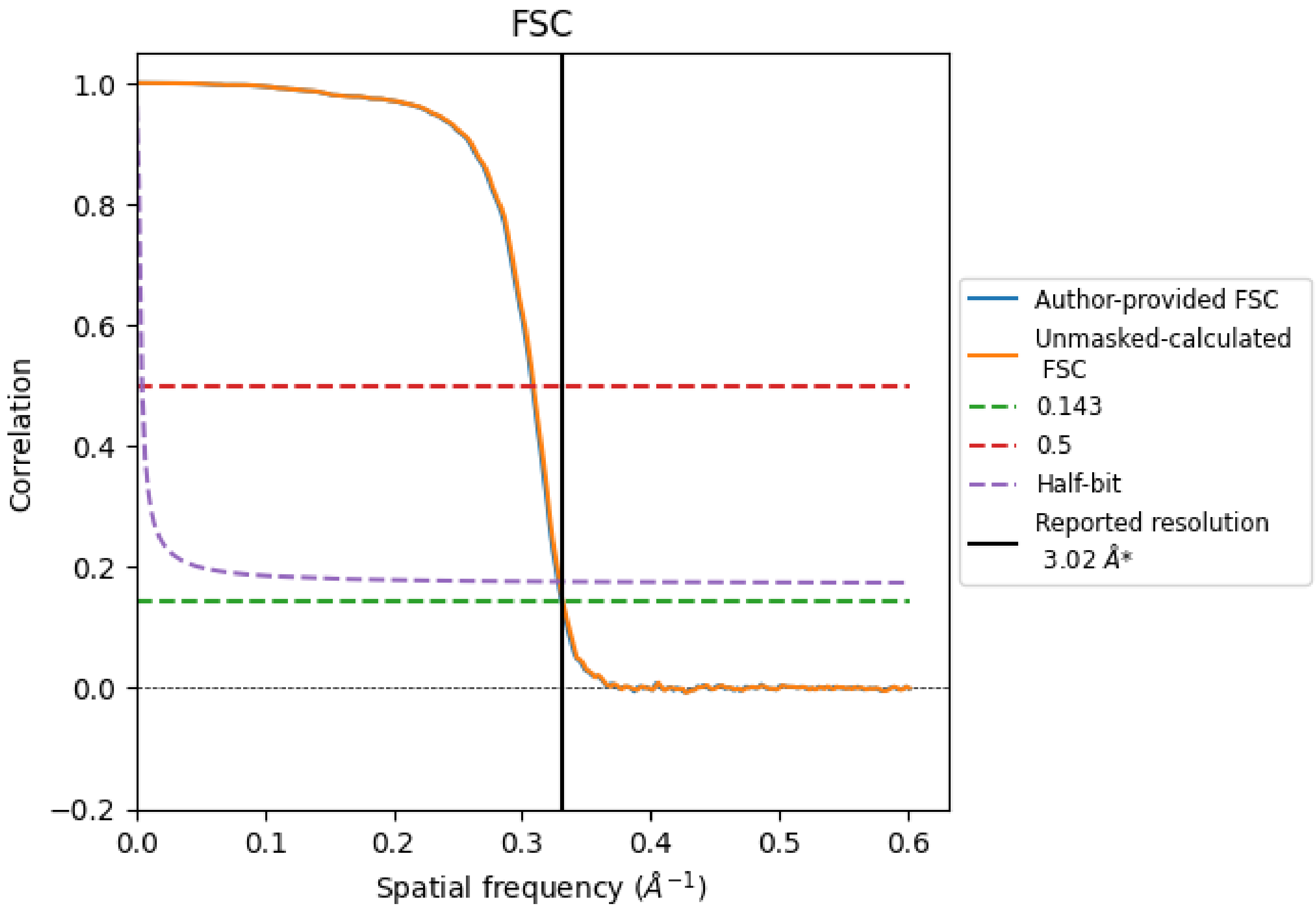

*Reported resolution corresponds to spatial frequency of 0.331 Å^{-1}

For Manuscript Review

8.2 Resolution estimates (i)

Resolution estimate (Å)	Estimation criterion (FSC cut-off)		
	0.143	0.5	Half-bit
Reported by author	3.02	-	-
Author-provided FSC curve	3.02	3.25	3.05
Unmasked-calculated*	3.01	3.24	3.04

*Resolution estimate based on FSC curve calculated by comparison of deposited half-maps.

For Manuscript Review

9 Map-model fit (i)

This section contains information regarding the fit between EMDB map EMD-75676 and PDB model 11GG. Per-residue inclusion information can be found in section 3 on page 15.

9.1 Map-model overlay (i)

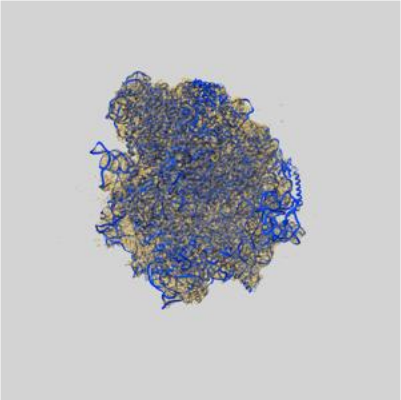

X

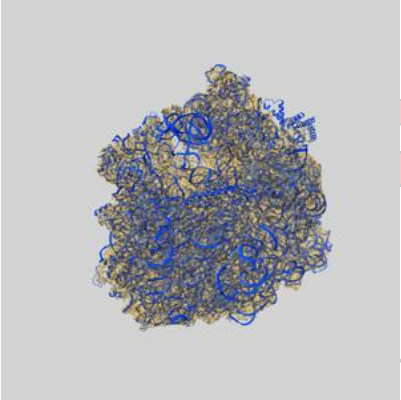

Y

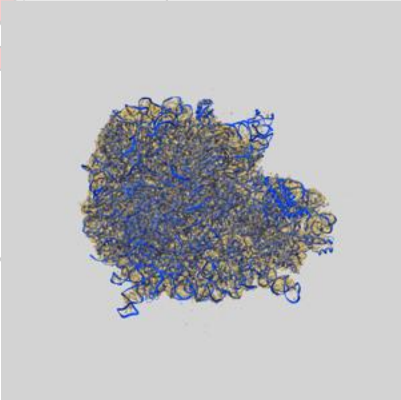

Z

The images above show the 3D surface view of the map at the recommended contour level 3.0 at 50% transparency in yellow overlaid with a ribbon representation of the model coloured in blue. These images allow for the visual assessment of the quality of fit between the atomic model and the map.

For Manuscript Review

9.2 Q-score mapped to coordinate model (i)

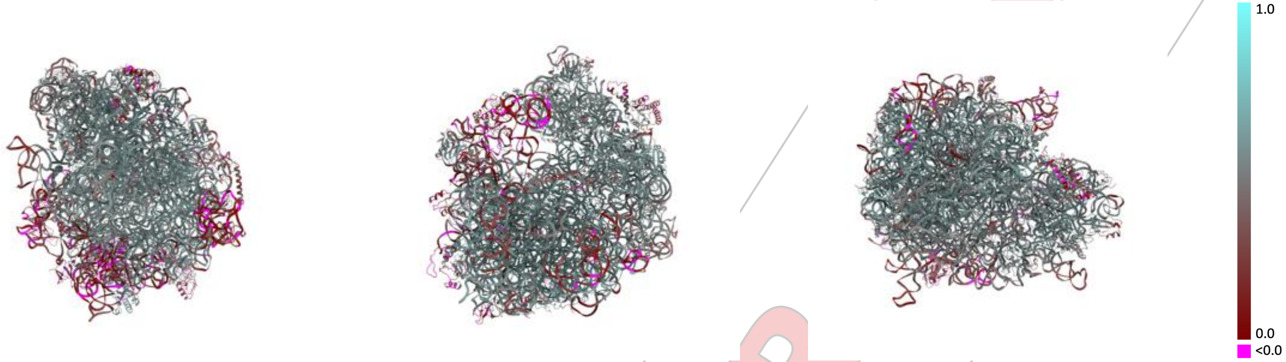

X Y Z

The images above show the model with each residue coloured according its Q-score. This shows their resolvability in the map with higher Q-score values reflecting better resolvability. Please note: Q-score is calculating the resolvability of atoms, and thus high values are only expected at resolutions at which atoms can be resolved. Low Q-score values may therefore be expected for many entries.

9.3 Atom inclusion mapped to coordinate model (i)

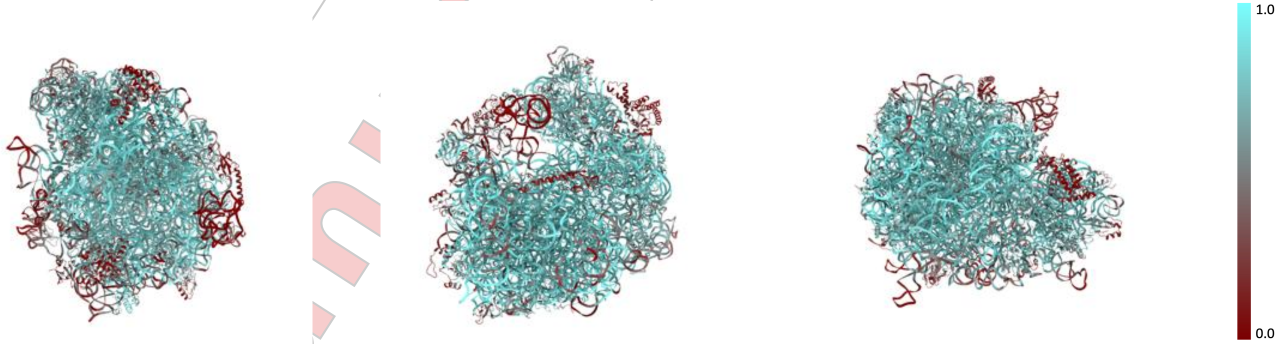

X Y Z

The images above show the model with each residue coloured according to its atom inclusion. This shows to what extent they are inside the map at the recommended contour level (3).

For Manuscript Review

9.4 Atom inclusion (i)

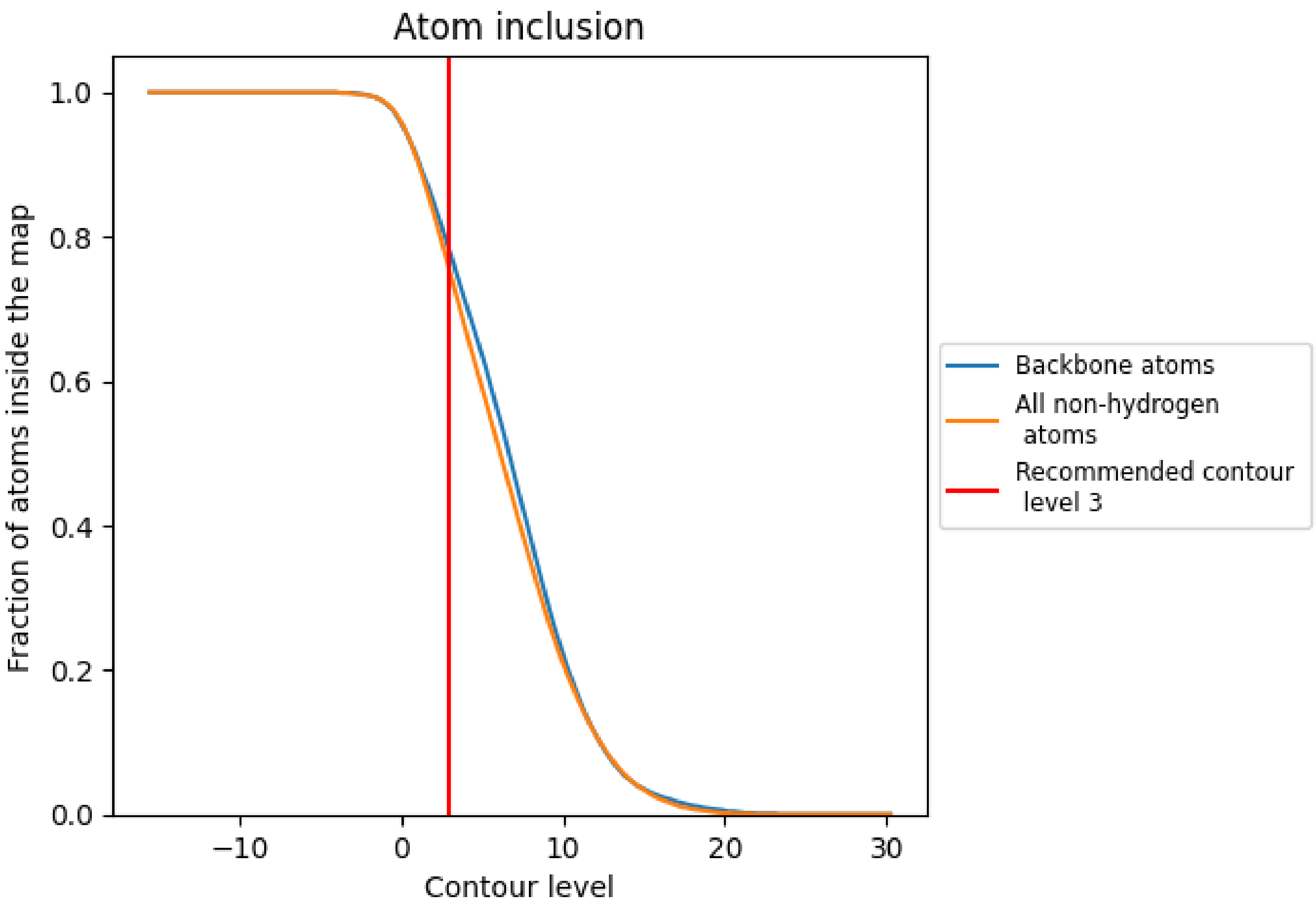

At the recommended contour level, 78% of all backbone atoms, 75% of all non-hydrogen atoms, are inside the map.

For Manuscript Review

9.5 Map-model fit summary (i)

The table lists the average atom inclusion at the recommended contour level (3) and Q-score for the entire model and for each chain.

Chain	Atom inclusion	Q-score
All	0.7530	0.4850
1	0.6690	0.5010
2	0.6910	0.5140
3	0.4230	0.4620
4	0.8230	0.5830
C	0.8220	0.5230
D	0.1490	0.3410
E	0.7160	0.5490
F	0.5380	0.5120
G	0.7670	0.5590
H	0.7060	0.5180
I	0.8030	0.4840
J	0.4450	0.2020
K	0.8770	0.5860
L	0.7940	0.5350
M	0.6480	0.4210
N	0.2910	0.1390
O	0.2880	0.1360
P	0.2610	0.3880
Q	0.1800	0.1440
R	0.8380	0.5640
S	0.8470	0.5840
T	0.6480	0.3860
U	0.7970	0.5350
V	0.8700	0.5860
W	0.7580	0.5360
X	0.8450	0.6030
Y	0.7870	0.5110
Z	0.6150	0.3710
b	0.7510	0.5480
c	0.5500	0.3680
d	0.3560	0.1850
e	0.6310	0.3790
f	0.8590	0.5850
g	0.6360	0.4360

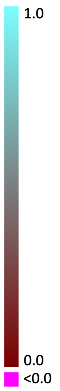

Continued on next page...

For Manuscript Review

Continued from previous page...

Chain	Atom inclusion	Q-score
h	0.6960	0.4400
i	0.8460	0.5970
j	0.1200	0.1290
k	0.9070	0.6160
l	0.8920	0.6110
m	0.8360	0.5460
n	0.5870	0.5160
o	0.7790	0.5690
p	0.6460	0.5170
q	0.4950	0.4560
r	0.7540	0.5450
t	0.7360	0.5170
u	0.6990	0.5330
v	0.7030	0.5180
w	0.7940	0.5510
x	0.6120	0.4740
y	0.6770	0.5250
z	0.8190	0.5510

For Manuscript Review
