# Supplementary material for: Structural adaptations for enhanced translation kinetics in evolved ribosomes
Source: Nucleic Acids Res. 2026 Jul 22;54(14):gkag703. doi: 10.1093/nar/gkag703 (PMC13389312; doi:10.1093/nar/gkag703)
Supplement: gkag703_Supplemental_Files [file gkag703_supplemental_files.zip › PDB_validation.zip!/EC_S3.5_D_1000303865_val-report-full-annotate_P1.pdf]

Full wwPDB EM Validation Report (i)

Feb 19, 2026 – 11:28 AM EST

PDB ID	:	11BQ / pdb_000011bq
EMDB ID	:	EMD-75605
Title	:	Escherichia coli 70S ribosome containing an evolved 16S rRNA (EC-S3.5)
Deposited on	:	2026-02-16
Resolution	:	3.00 Å(reported)

This wwPDB validation report is for manuscript review

For Manuscript Review

This is a Full wwPDB EM Validation Report.

This report is produced by the wwPDB biocuration pipeline after annotation of the structure.

We welcome your comments at *validation@mail.wwpdb.org*
A user guide is available at
https://www.wwpdb.org/validation/2017/EMValidationReportHelp
with specific help available everywhere you see the (i) symbol.

The types of validation reports are described at
http://www.wwpdb.org/validation/2017/FAQs#types.

The following versions of software and data (see references (i)) were used in the production of this report:

EMDB validation analysis	:	0.0.1.dev132
Mogul	:	2022.3.0, CSD as543be (2022)
MolProbity	:	4-5-2 with Phenix2.0
buster-report	:	1.1.7 (2018)
Percentile statistics	:	20231227.v01 (using entries in the PDB archive December 27th 2023)
EM percentile statistics	:	202505.v01 (Using data in the EMDB archive up until May 2025)
MapQ	:	1.9.13
Ideal geometry (proteins)	:	Engh & Huber (2001)
Ideal geometry (DNA, RNA)	:	Parkinson et al. (1996)

1 Overall quality at a glance (i)

The following experimental techniques were used to determine the structure:
ELECTRON MICROSCOPY

The reported resolution of this entry is 3.00 Å.

Percentile scores (ranging between 0-100) for global validation metrics of the entry are shown in the following graphic. The table shows the number of entries on which the scores are based.

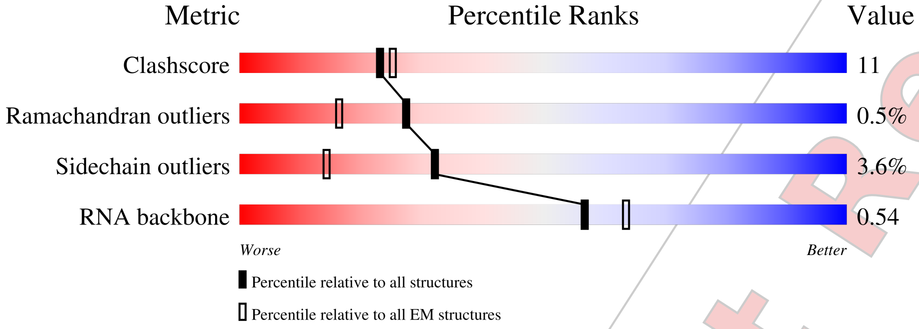

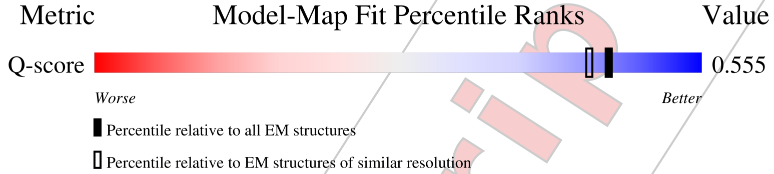

Metric	Whole archive (#Entries)	EM structures (#Entries)	Similar EM resolution (#Entries, resolution range(Å))
Clashscore	210492	15764	-
Ramachandran outliers	207382	16835	-
Sidechain outliers	206894	16415	-
RNA backbone	6643	2191	-
Q-score	-	25397	14081 (2.50 - 3.50)

The table below summarises the geometric issues observed across the polymeric chains and their fit to the map. The red, orange, yellow and green segments of the bar indicate the fraction of residues that contain outliers for >=3, 2, 1 and 0 types of geometric quality criteria respectively. A grey segment represents the fraction of residues that are not modelled. The numeric value for each fraction is indicated below the corresponding segment, with a dot representing fractions <=5% The upper red bar (where present) indicates the fraction of residues that have poor fit to the EM map (all-atom inclusion < 40%). The numeric value is given above the bar.

Mol	Chain	Length	Quality of chain
1	0	55	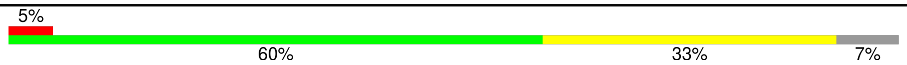

Continued on next page...

For Manuscript Review

Continued from previous page...

Mol	Chain	Length	Quality of chain
2	1	46	• / 80% 20%
3	2	65	78% 20% •
4	3	38	68% 32%
5	4	70	16% / 34% 50% • 14%
6	5	2	50% 50%
7	A	1542	• / 42% 40% 15% • •
8	B	241	29% / 60% 32% • 7%
9	C	233	13% / 55% 31% • 12%
10	D	206	5% / 62% 34% •
11	E	167	• / 65% 26% • 7%
12	F	135	• / 44% 30% • 24%
13	G	179	37% / 50% 35% • 15%
14	H	130	• / 72% 25% • •
15	I	130	28% / 51% 47% •
16	J	103	52% / 55% 38% • 5%
17	K	129	• / 67% 23% 9%
18	L	124	• / 63% 31% 5% • •
19	M	118	32% / 52% 44% • •
20	N	101	18% / 36% 34% 22% 8% •
21	O	89	• / 71% 27% • •
22	P	82	12% / 21% 48% 26% 5% •
23	Q	84	6% / 44% 45% 5% 6%
24	R	75	• / 59% 25% • 12%
25	S	92	33% / 58% 30% • 9%
26	T	87	• / 53% 41% 5% •

Continued on next page...

For Manuscript Review

Continued from previous page...

Mol	Chain	Length	Quality of chain
27	U	71	58% 63% 35% •
28	a	2753	• 65% 32% •
29	b	119	• 66% 34%
30	c	273	83% 15% ••
31	d	209	80% 20%
32	e	201	• 83% 16% •
33	f	179	11% 63% 34% ••
34	g	177	5% 68% 29% ••
35	h	149	• 17% 9% • 72%
36	i	142	82% 18%
37	j	123	76% 22% •
38	k	144	• 86% 14%
39	l	136	• 82% 18% •
40	m	127	76% 17% 7%
41	n	117	79% 21% •
42	o	115	• 73% 25% ••
43	p	118	86% 14% •
44	q	103	• 72% 28%
45	r	110	• 80% 20%
46	s	100	66% 27% 7%
47	t	104	• 71% 26% ••
48	u	94	• 66% 34%
49	v	85	• 82% 9% 8%
50	w	78	82% 17% •
51	x	63	• 78% 19% ••

Continued on next page...

For Manuscript Review

Continued from previous page...

Mol	Chain	Length	Quality of chain
52	y	59	• 66% 31% • •
53	z	57	• 81% 18% •

The following table lists non-polymeric compounds, carbohydrate monomers and non-standard residues in protein, DNA, RNA chains that are outliers for geometric or electron-density-fit criteria:

Mol	Type	Chain	Res	Chirality	Geometry	Clashes	Electron density
56	SPD	a	6209	-	-	X	-

For Manuscript Review

2 Entry composition (i)

There are 59 unique types of molecules in this entry. The entry contains 138618 atoms, of which 0 are hydrogens and 0 are deuteriums.

In the tables below, the AltConf column contains the number of residues with at least one atom in alternate conformation and the Trace column contains the number of residues modelled with at most 2 atoms.

- Molecule 1 is a protein called 50S ribosomal protein L33.

Mol	Chain	Residues	Atoms				AltConf	Trace
			Total	C	N	O		
1	0	51	417	269	76	72	0	0

- Molecule 2 is a protein called Large ribosomal subunit protein bL34.

Mol	Chain	Residues	Atoms					AltConf	Trace
			Total	C	N	O	S		
2	1	46	377	228	90	57	2	0	0

- Molecule 3 is a protein called 50S ribosomal protein L35.

Mol	Chain	Residues	Atoms					AltConf	Trace
			Total	C	N	O	S		
3	2	64	504	323	105	74	2	0	0

- Molecule 4 is a protein called Large ribosomal subunit protein bL36A.

Mol	Chain	Residues	Atoms					AltConf	Trace
			Total	C	N	O	S		
4	3	38	302	185	65	48	4	0	0

- Molecule 5 is a protein called 50S ribosomal protein L31.

Mol	Chain	Residues	Atoms					AltConf	Trace
			Total	C	N	O	S		
5	4	60	480	299	90	85	6	0	0

- Molecule 6 is a RNA chain called E-site tRNA.

Mol	Chain	Residues	Atoms					AltConf	Trace
			Total	C	N	O	P		
6	5	2	42	19	8	13	2	0	0

For Manuscript Review

- Molecule 7 is a RNA chain called 16S ribosomal RNA.

Mol	Chain	Residues	Atoms					AltConf	Trace
			Total	C	N	O	P		
7	A	1519	32607	14551	5985	10552	1519	0	0

- Molecule 8 is a protein called Small ribosomal subunit protein uS2.

Mol	Chain	Residues	Atoms					AltConf	Trace
			Total	C	N	O	S		
8	B	224	1753	1109	315	321	8	0	0

- Molecule 9 is a protein called 30S ribosomal protein S3.

Mol	Chain	Residues	Atoms					AltConf	Trace
			Total	C	N	O	S		
9	C	206	1624	1028	305	288	3	0	0

- Molecule 10 is a protein called Small ribosomal subunit protein uS4.

Mol	Chain	Residues	Atoms					AltConf	Trace
			Total	C	N	O	S		
10	D	205	1643	1026	315	298	4	0	0

- Molecule 11 is a protein called Small ribosomal subunit protein uS5.

Mol	Chain	Residues	Atoms					AltConf	Trace
			Total	C	N	O	S		
11	E	156	1152	717	217	212	6	0	0

- Molecule 12 is a protein called Small ribosomal subunit protein bS6.

Mol	Chain	Residues	Atoms					AltConf	Trace
			Total	C	N	O	S		
12	F	103	839	530	151	151	7	0	0

- Molecule 13 is a protein called Small ribosomal subunit protein uS7.

Mol	Chain	Residues	Atoms					AltConf	Trace
			Total	C	N	O	S		
13	G	153	1203	750	231	218	4	0	0

- Molecule 14 is a protein called Small ribosomal subunit protein uS8.

For Manuscript Review

Mol	Chain	Residues	Atoms					AltConf	Trace
			Total	C	N	O	S		
14	H	129	979	616	173	184	6	0	0

- Molecule 15 is a protein called Small ribosomal subunit protein uS9.

Mol	Chain	Residues	Atoms					AltConf	Trace
			Total	C	N	O	S		
15	I	127	1022	634	206	179	3	0	0

- Molecule 16 is a protein called Small ribosomal subunit protein uS10.

Mol	Chain	Residues	Atoms					AltConf	Trace
			Total	C	N	O	S		
16	J	98	786	493	150	142	1	0	0

- Molecule 17 is a protein called Small ribosomal subunit protein uS11.

Mol	Chain	Residues	Atoms					AltConf	Trace
			Total	C	N	O	S		
17	K	117	877	540	173	161	3	0	0

There is a discrepancy between the modelled and reference sequences:

Chain	Residue	Modelled	Actual	Comment	Reference
K	119	IAS	ASN	conflict	UNP A7ZSI6

- Molecule 18 is a protein called Small ribosomal subunit protein uS12.

Mol	Chain	Residues	Atoms					AltConf	Trace
			Total	C	N	O	S		
18	L	123	962	595	198	164	5	1	0

There is a discrepancy between the modelled and reference sequences:

Chain	Residue	Modelled	Actual	Comment	Reference
L	89	CYS	ASP	conflict	UNP A7ZSL7

- Molecule 19 is a protein called Small ribosomal subunit protein uS13.

Mol	Chain	Residues	Atoms					AltConf	Trace
			Total	C	N	O	S		
19	M	115	891	552	179	157	3	0	0

For Manuscript Review

- Molecule 20 is a protein called Small ribosomal subunit protein uS14.

Mol	Chain	Residues	Atoms					AltConf	Trace
			Total	C	N	O	S		
20	N	100	805	499	164	139	3	0	0

- Molecule 21 is a protein called Small ribosomal subunit protein uS15.

Mol	Chain	Residues	Atoms					AltConf	Trace
			Total	C	N	O	S		
21	O	88	714	439	144	130	1	0	0

- Molecule 22 is a protein called Small ribosomal subunit protein bS16.

Mol	Chain	Residues	Atoms					AltConf	Trace
			Total	C	N	O	S		
22	P	81	643	403	127	112	1	0	0

- Molecule 23 is a protein called Small ribosomal subunit protein uS17.

Mol	Chain	Residues	Atoms					AltConf	Trace
			Total	C	N	O	S		
23	Q	79	641	406	120	112	3	0	0

- Molecule 24 is a protein called Small ribosomal subunit protein bS18.

Mol	Chain	Residues	Atoms					AltConf	Trace
			Total	C	N	O	S		
24	R	66	544	345	102	96	1	0	0

- Molecule 25 is a protein called Small ribosomal subunit protein uS19.

Mol	Chain	Residues	Atoms					AltConf	Trace
			Total	C	N	O	S		
25	S	84	668	427	127	112	2	0	0

- Molecule 26 is a protein called Small ribosomal subunit protein bS20.

Mol	Chain	Residues	Atoms					AltConf	Trace
			Total	C	N	O	S		
26	T	86	670	414	138	115	3	0	0

- Molecule 27 is a protein called Small ribosomal subunit protein bS21.

For Manuscript Review

Mol	Chain	Residues	Atoms					AltConf	Trace
			Total	C	N	O	S		
27	U	70	589	366	125	97	1	0	0

- Molecule 28 is a RNA chain called 23S ribosomal RNA.

Mol	Chain	Residues	Atoms					AltConf	Trace
			Total	C	N	O	P		
28	a	2753	59130	26384	10897	19096	2753	0	0

- Molecule 29 is a RNA chain called 5S ribosomal RNA.

Mol	Chain	Residues	Atoms					AltConf	Trace
			Total	C	N	O	P		
29	b	119	2549	1135	466	829	119	0	0

- Molecule 30 is a protein called 50S ribosomal protein L2.

Mol	Chain	Residues	Atoms					AltConf	Trace
			Total	C	N	O	S		
30	c	271	2082	1288	423	364	7	0	0

- Molecule 31 is a protein called 50S ribosomal protein L3.

Mol	Chain	Residues	Atoms					AltConf	Trace
			Total	C	N	O	S		
31	d	209	1566	980	288	294	4	0	0

- Molecule 32 is a protein called Large ribosomal subunit protein uL4.

Mol	Chain	Residues	Atoms					AltConf	Trace
			Total	C	N	O	S		
32	e	201	1552	974	283	290	5	0	0

- Molecule 33 is a protein called Large ribosomal subunit protein uL5.

Mol	Chain	Residues	Atoms					AltConf	Trace
			Total	C	N	O	S		
33	f	177	1410	899	249	256	6	0	0

- Molecule 34 is a protein called Large ribosomal subunit protein uL6.

For Manuscript Review

Mol	Chain	Residues	Atoms					AltConf	Trace
			Total	C	N	O	S		
34	g	176	1323	832	243	246	2	0	0

- Molecule 35 is a protein called Large ribosomal subunit protein bL9.

Mol	Chain	Residues	Atoms					AltConf	Trace
			Total	C	N	O	S		
35	h	41	303	194	54	54	1	0	0

- Molecule 36 is a protein called Large ribosomal subunit protein uL13.

Mol	Chain	Residues	Atoms					AltConf	Trace
			Total	C	N	O	S		
36	i	142	1129	714	212	199	4	0	0

- Molecule 37 is a protein called Large ribosomal subunit protein uL14.

Mol	Chain	Residues	Atoms					AltConf	Trace
			Total	C	N	O	S		
37	j	123	957	599	185	167	6	1	0

- Molecule 38 is a protein called Large ribosomal subunit protein uL15.

Mol	Chain	Residues	Atoms					AltConf	Trace
			Total	C	N	O	S		
38	k	144	1053	654	207	190	2	0	0

- Molecule 39 is a protein called Large ribosomal subunit protein uL16.

Mol	Chain	Residues	Atoms					AltConf	Trace
			Total	C	N	O	S		
39	l	136	1075	686	205	177	7	0	0

- Molecule 40 is a protein called Large ribosomal subunit protein bL17.

Mol	Chain	Residues	Atoms					AltConf	Trace
			Total	C	N	O	S		
40	m	118	945	585	194	161	5	0	0

- Molecule 41 is a protein called Large ribosomal subunit protein uL18.

For Manuscript Review

Mol	Chain	Residues	Atoms				AltConf	Trace
			Total	C	N	O		
41	n	116	892	552	178	162	0	0

- Molecule 42 is a protein called Large ribosomal subunit protein bL19.

Mol	Chain	Residues	Atoms					AltConf	Trace
			Total	C	N	O	S		
42	o	114	917	574	179	163	1	0	0

- Molecule 43 is a protein called 50S ribosomal protein L20.

Mol	Chain	Residues	Atoms				AltConf	Trace
			Total	C	N	O		
43	p	117	947	604	192	151	0	0

- Molecule 44 is a protein called Large ribosomal subunit protein bL21.

Mol	Chain	Residues	Atoms					AltConf	Trace
			Total	C	N	O	S		
44	q	103	816	516	153	145	2	0	0

- Molecule 45 is a protein called Large ribosomal subunit protein uL22.

Mol	Chain	Residues	Atoms					AltConf	Trace
			Total	C	N	O	S		
45	r	110	857	532	166	156	3	0	0

- Molecule 46 is a protein called 50S ribosomal protein L23.

Mol	Chain	Residues	Atoms					AltConf	Trace
			Total	C	N	O	S		
46	s	93	738	466	139	131	2	0	0

- Molecule 47 is a protein called 50S ribosomal protein L24.

Mol	Chain	Residues	Atoms				AltConf	Trace
			Total	C	N	O		
47	t	102	779	492	146	141	0	0

- Molecule 48 is a protein called Large ribosomal subunit protein bL25.

For Manuscript Review

Mol	Chain	Residues	Atoms					AltConf	Trace
			Total	C	N	O	S		
48	u	94	753	479	137	134	3	0	0

- Molecule 49 is a protein called 50S ribosomal protein L27.

Mol	Chain	Residues	Atoms					AltConf	Trace
			Total	C	N	O	S		
49	v	78	586	362	116	107	1	0	0

- Molecule 50 is a protein called 50S ribosomal protein L28.

Mol	Chain	Residues	Atoms					AltConf	Trace
			Total	C	N	O	S		
50	w	77	625	388	129	106	2	0	0

- Molecule 51 is a protein called Large ribosomal subunit protein uL29.

Mol	Chain	Residues	Atoms					AltConf	Trace
			Total	C	N	O	S		
51	x	62	501	308	98	94	1	0	0

- Molecule 52 is a protein called 50S ribosomal protein L30.

Mol	Chain	Residues	Atoms					AltConf	Trace
			Total	C	N	O	S		
52	y	58	449	281	87	79	2	0	0

- Molecule 53 is a protein called 50S ribosomal protein L32.

Mol	Chain	Residues	Atoms					AltConf	Trace
			Total	C	N	O	S		
53	z	56	444	269	94	80	1	0	0

- Molecule 54 is ZINC ION (CCD ID: ZN) (formula: Zn).

Mol	Chain	Residues	Atoms		AltConf
			Total	Zn	
54	3	1	1	1	0
			Total	Zn	
54	4	1	1	1	0

- Molecule 55 is MAGNESIUM ION (CCD ID: MG) (formula: Mg).

For Manuscript Review

Mol	Chain	Residues	Atoms		AltConf
55	A	89	Total 89	Mg 89	0
55	N	1	Total 1	Mg 1	0
55	Q	1	Total 1	Mg 1	0
55	a	208	Total 208	Mg 208	0
55	b	5	Total 5	Mg 5	0
55	c	1	Total 1	Mg 1	0
55	p	1	Total 1	Mg 1	0
55	z	1	Total 1	Mg 1	0

- Molecule 56 is SPERMIDINE (CCD ID: SPD) (formula: $C_7H_{19}N_3$).

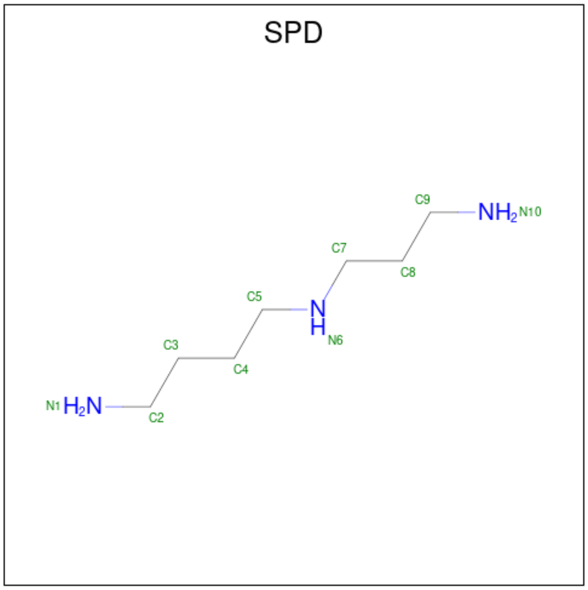

Mol	Chain	Residues	Atoms			AltConf
56	A	1	Total 10	C 7	N 3	0
56	a	1	Total 10	C 7	N 3	0
56	a	1	Total 10	C 7	N 3	0
56	a	1	Total 10	C 7	N 3	0

Continued on next page...

For Manuscript Review

Continued from previous page...

Mol	Chain	Residues	Atoms	AltConf
56	a	1	Total C N<br>10 7 3	0
56	a	1	Total C N<br>10 7 3	0
56	a	1	Total C N<br>10 7 3	0
56	a	1	Total C N<br>10 7 3	0
56	a	1	Total C N<br>10 7 3	0
56	a	1	Total C N<br>10 7 3	0
56	a	1	Total C N<br>10 7 3	0
56	a	1	Total C N<br>10 7 3	0
56	a	1	Total C N<br>10 7 3	0
56	a	1	Total C N<br>10 7 3	0

- Molecule 57 is SPERMINE (CCD ID: SPM) (formula: $C_{10}H_{26}N_4$).

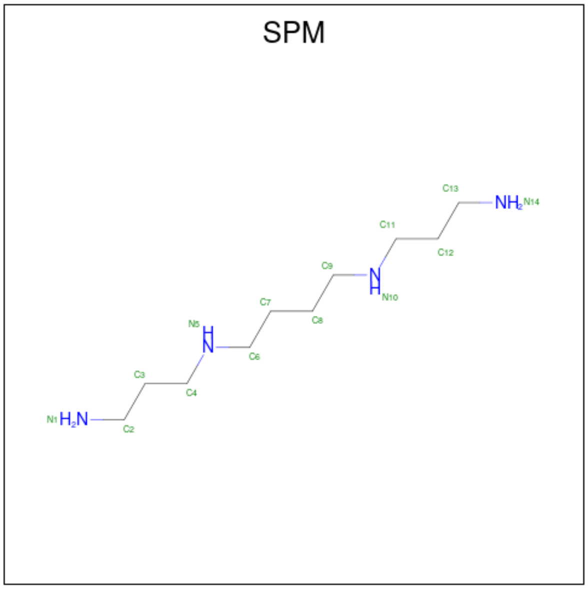

Mol	Chain	Residues	Atoms	AltConf
57	a	1	Total C N<br>14 10 4	0

For Manuscript Review

- Molecule 58 is CYTIDINE-5'-MONOPHOSPHATE (CCD ID: C) (formula: $C_9H_{14}N_3O_8P$).

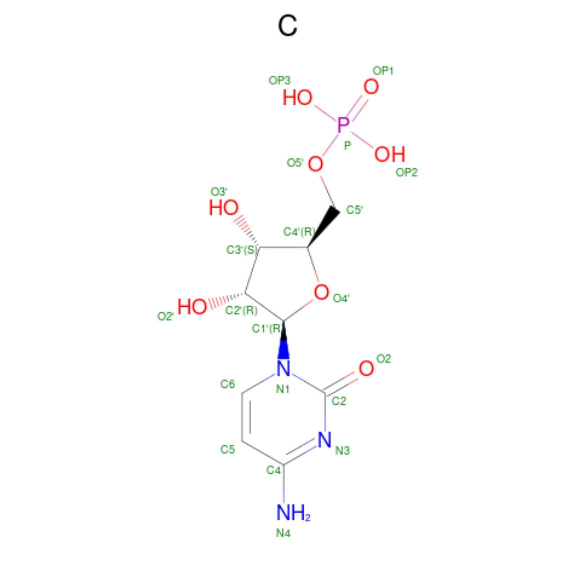

Mol	Chain	Residues	Atoms	AltConf
58	a	1	Total C N O P<br>20 9 3 7 1	0

- Molecule 59 is GUANOSINE-5'-MONOPHOSPHATE (CCD ID: G) (formula: $C_{10}H_{14}N_5O_8P$).

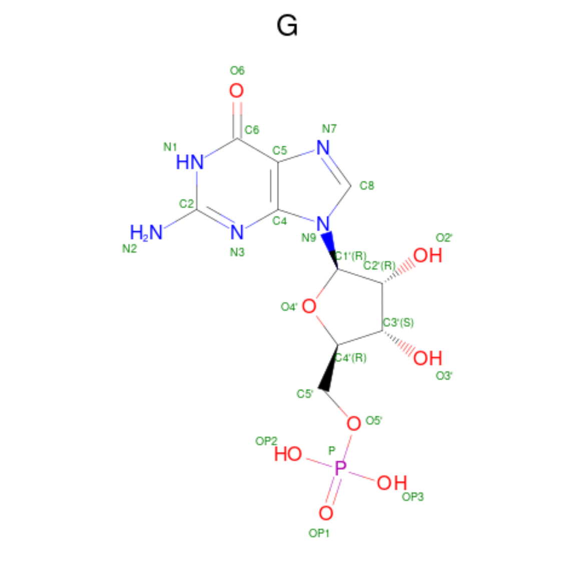

Mol	Chain	Residues	Atoms	AltConf
59	a	1	Total C N O P<br>23 10 5 7 1	0

For Manuscript Review

3 Residue-property plots (i)

These plots are drawn for all protein, RNA, DNA and oligosaccharide chains in the entry. The first graphic for a chain summarises the proportions of the various outlier classes displayed in the second graphic. The second graphic shows the sequence view annotated by issues in geometry and atom inclusion in map density. Residues are color-coded according to the number of geometric quality criteria for which they contain at least one outlier: green = 0, yellow = 1, orange = 2 and red = 3 or more. A red diamond above a residue indicates a poor fit to the EM map for this residue (all-atom inclusion < 40%). Stretches of 2 or more consecutive residues without any outlier are shown as a green connector. Residues present in the sample, but not in the model, are shown in grey.

- Molecule 1: 50S ribosomal protein L33

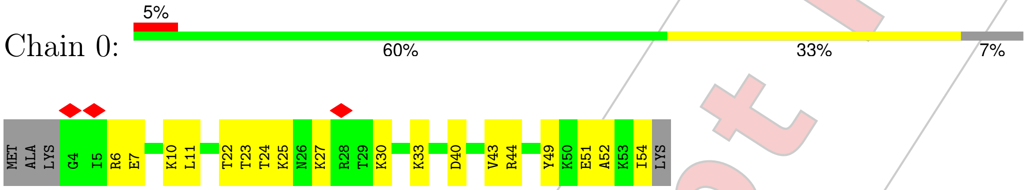

- Molecule 2: Large ribosomal subunit protein bL34

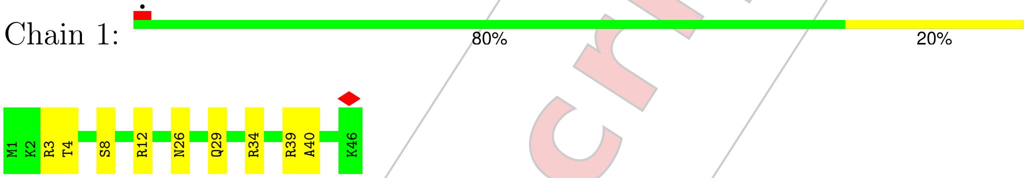

- Molecule 3: 50S ribosomal protein L35

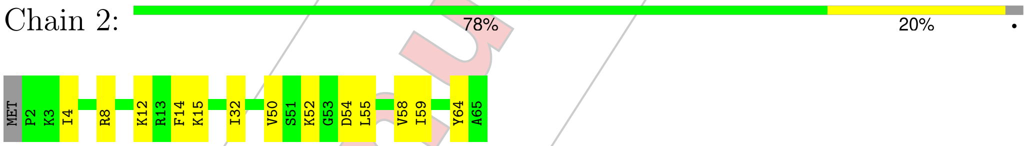

- Molecule 4: Large ribosomal subunit protein bL36A

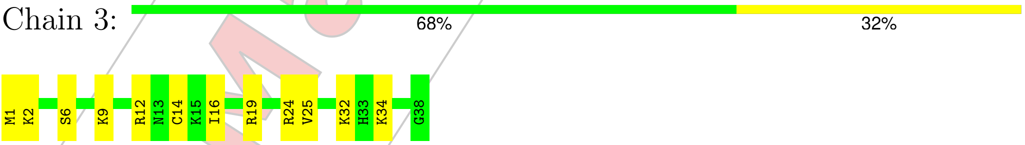

- Molecule 5: 50S ribosomal protein L31

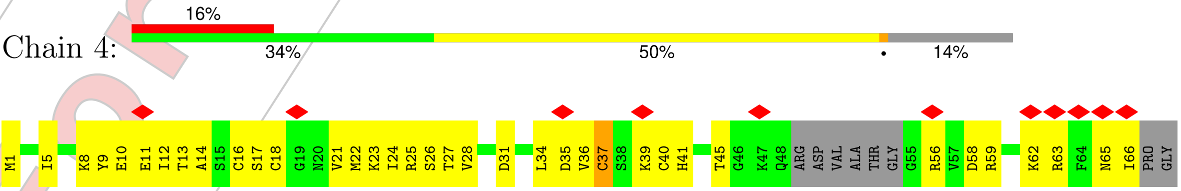

For Manuscript Review

SER LYS

- Molecule 6: E-site tRNA

Chain 5: 50% 50%

C75 A76

- Molecule 7: 16S ribosomal RNA

Chain A: 42% 40% 15%

A A2 A3 U4 U5 G6 A7 A8 G9 G15 A16 U17 C18 G21 G22 A28 G31 A32 A33 C34 G35 C36 U37 G38 G39 C40 G41 G45 G46 C47 C48 U49 A50 A51 A55 U56 G57 C58 A59 A60 G61 U62 A65 A71 A72 C73 A74 G75 G76 A77 A78 G79

A80 A81 G82 C83 U84 U85 G86 C87 U88 U89 C90 U91 U92 U93 G94 C95 U96 G97 A98 C99 G104 G107 C110 G111 G112 G113 U114 G115 A116 A119 A120 U121 G122 U123 C124 G127 G128 A129 A130 A131 C132 U137 G141 G142 A143 G144 G145 G146 G147 G148 A149 U150

U154 A155 C156 U157 A161 A162 C163 G164 G165 U166 A167 A171 C175 C176 G177 C178 A179 U180 A181 A182 C183 G187 C188 A189 A190 C193 C194 A195 A196 A197 G200 G201 G202 G203 G204 A C C U U C G G G C214 C215 U216 C217 G220 C221 C222 A223 U224

C225 G226 C233 C234 C235 A236 G237 A243 U244 U245 A246 G247 G251 U252 A253 U261 C264 G265 G266 C267 U268 C269 A270 C271 C272 U273 C280 G281 A282 G289 C290 U291 G292 G293 U294 C295 U296 G297 A298 G299 A300 G305 A306 C307 C308 A309 G310 C311 A315 C316 U317

G318 G319 A320 A321 C322 U323 G324 A327 C328 C335 A336 G337 A338 C339 A344 C345 G346 C352 A353 G354 C355 A356 G361 G362 A363 A364 U365 A366 U367 U368 A371 C372 A373 A374 U375 G376 G377 G378 C379 G380 C381 A382 A383 G384 C385 G388 A389 U390 G391 C392 A393 G394

A397 U398 G399 C400 C401 G402 C403 G404 U405 G406 U407 A408 U409 G410 A411 C412 G413 A414 A415 G416 G417 U420 U421 C422 G423 G424 G425 U426 U427 G428 U429 A430 A431 A432 G433 U434 A435 C436 U437 U438 U439 C440 A441 A448 G449 G450 A451 A452 G453 G454 G455 A456 G457 U458 A459 A460 A461 G462

U463 U464 A465 A466 U467 A468 C469 C470 U471 U472 U473 G474 C475 U476 C477 A478 U479 U480 G481 G484 U485 U486 A487 C488 C489 C490 G491 C492 A493 G494 A495 A496 G497 A498 A499 G500 C501 A502 C503 C504 G505 G506 A509 A510 C511 U512 C513 C514 G515 U516 G517 C518 G521 C522 A523 G524 C525

C526 G527 C528 G529 G530 U531 A532 A533 C536 G541 G542 U543 G544 C545 A546 A547 G548 C549 G550 U551 U552 A553 A554 U555 C556 A559 A560 U561 U562 A563 C564 U565 G566 G570 U571 A572 A573 C576 G577 C578 A579 C580 G581 G584 G585 U590 G591 G592 U593 A596 C599

A600 G601 A602 U603 G604 U605 G606 A607 A608 A609 U610 C611 C612 C613 C614 G615 G616 G617 C618 U619 C620 A621 A622 G627 G628 G633 C634 A635 U636 G639 A642 A649 G650 C651 U652 U653 U657 A663 G664 A665 G669 G670 G671 U672 A673 G674 A675 A676 U677 U678 C679

G685 U686 A687 G693 A694 A695 A696 U697 G703 A704 G713 G714 A715 A716 U717 A718 G721 C722 U723 G724 A728 A729 G734 C735 C736 C737 C738 G741 G742 A743 C744 G745 A746 A747 G748 A749 C750 U751 G752 G755 C756 U757 C764 G765 A766 G769 G774 A777

G778 C779 A780 A781 A792 U793 A794 C795 C796 G799 C811 A815 A816 C817 U820 G821 A825 C826 G829 G830 A831 G832 G836 U837 G838 C839 C840 C U U G A G846 G847 C848 G849 U850 G859 A860 G861 A864 C868 G869 A872 U875 C876 G877

For Manuscript Review

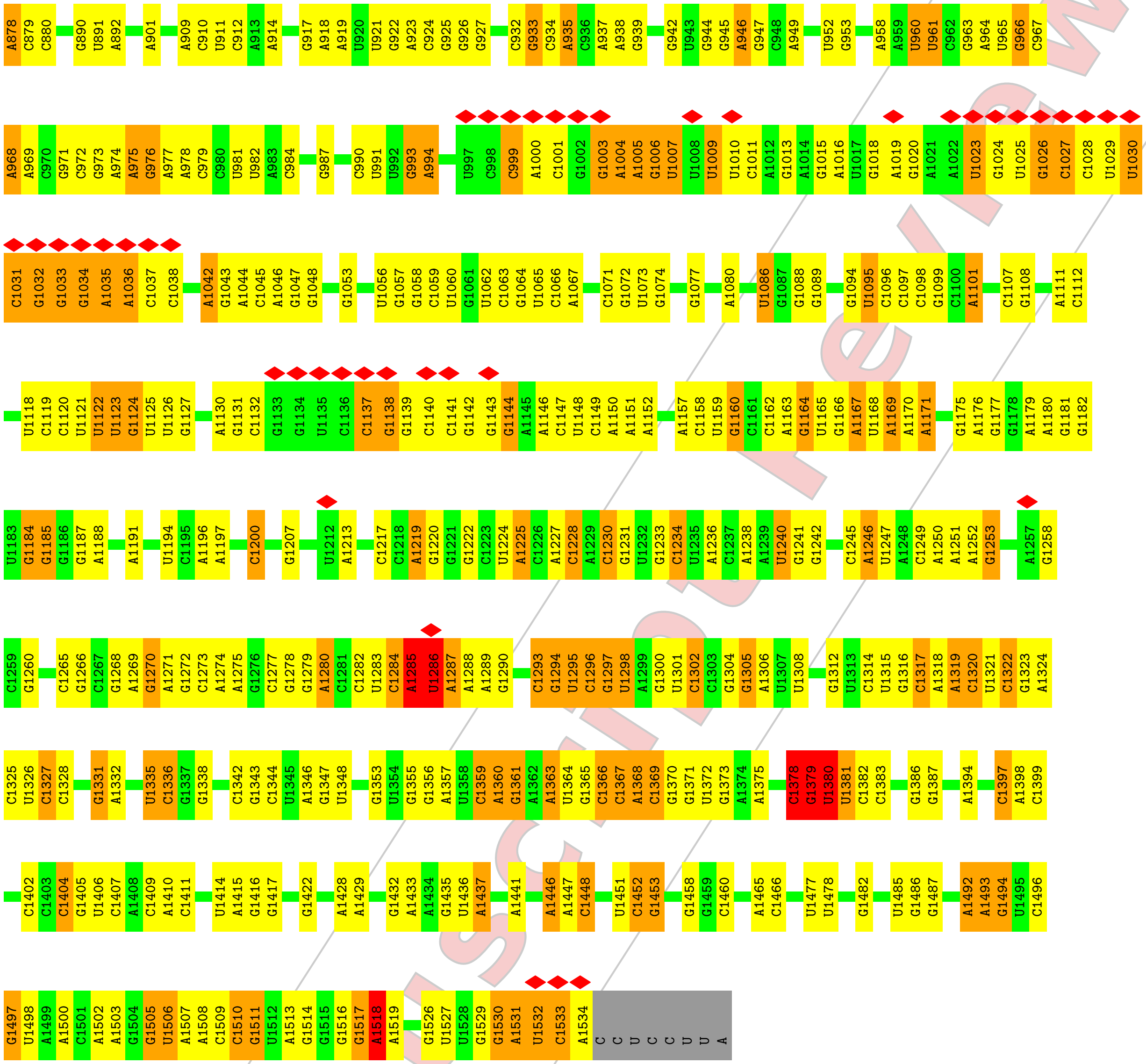

• Molecule 8: Small ribosomal subunit protein uS2

Chain B:

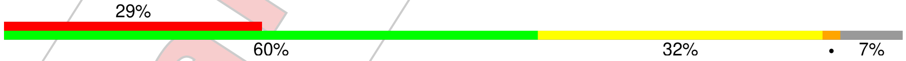

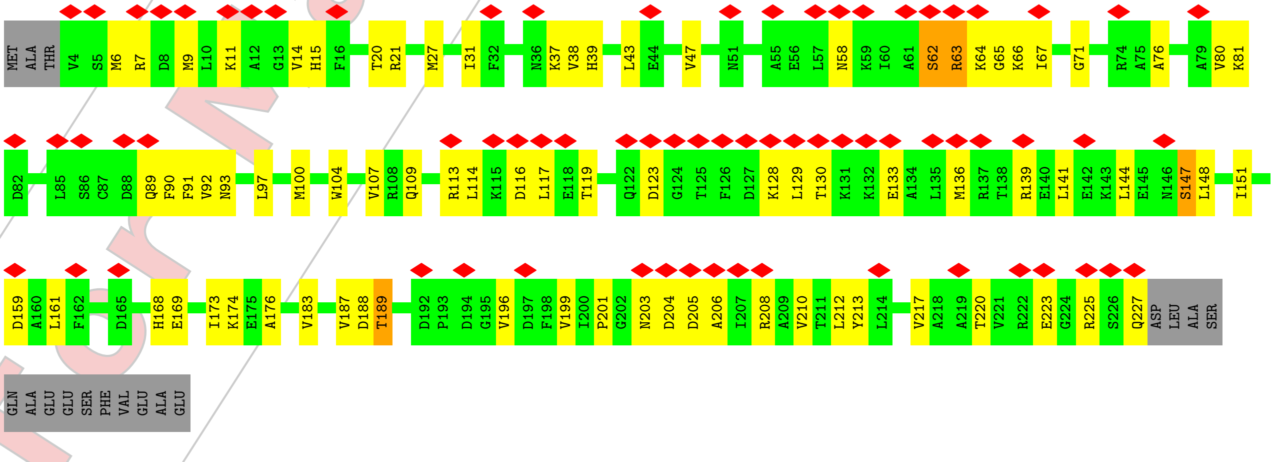

For Manuscript Review

• Molecule 9: 30S ribosomal protein S3

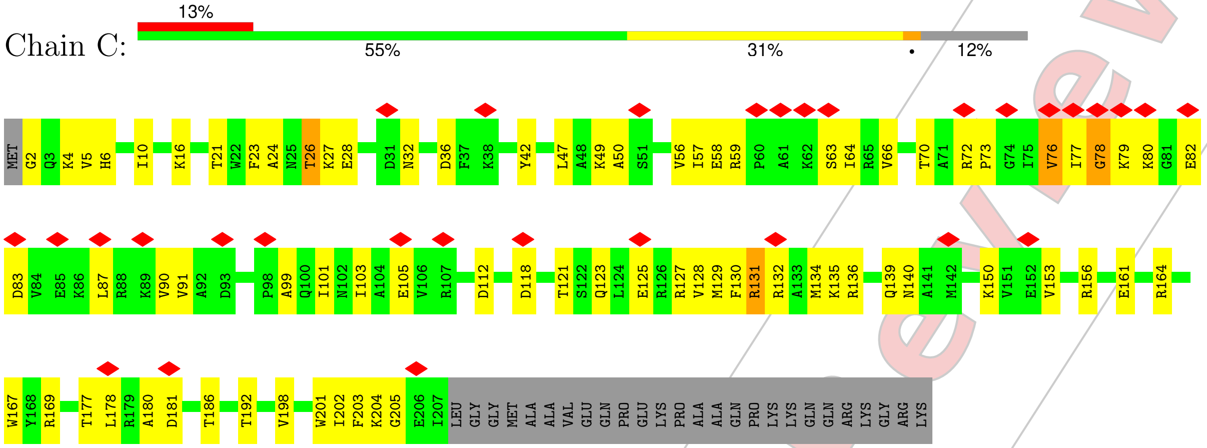

• Molecule 10: Small ribosomal subunit protein uS4

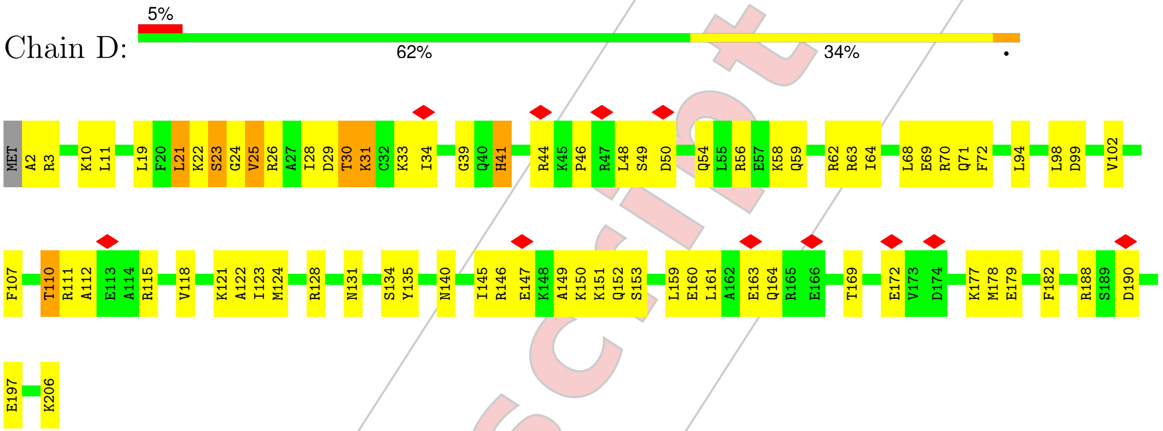

• Molecule 11: Small ribosomal subunit protein uS5

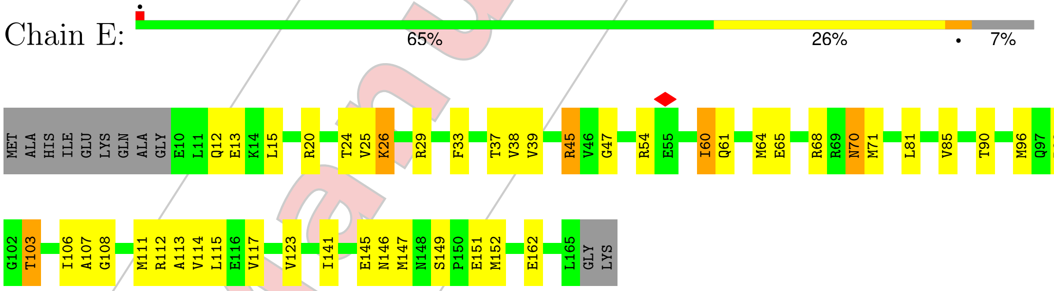

• Molecule 12: Small ribosomal subunit protein bS6

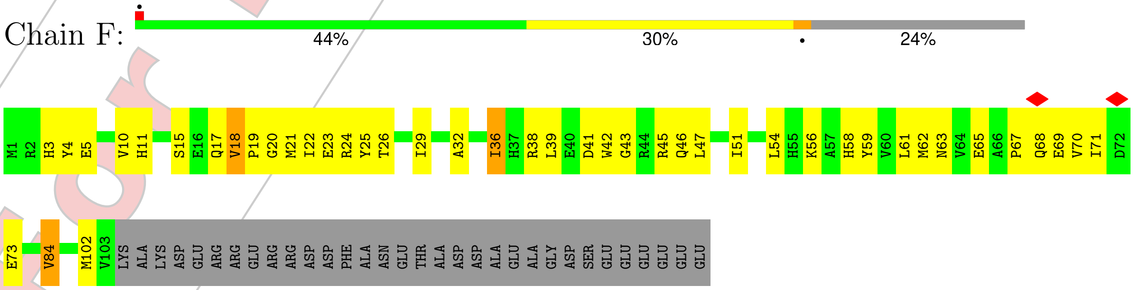

For Manuscript Review

- Molecule 13: Small ribosomal subunit protein uS7

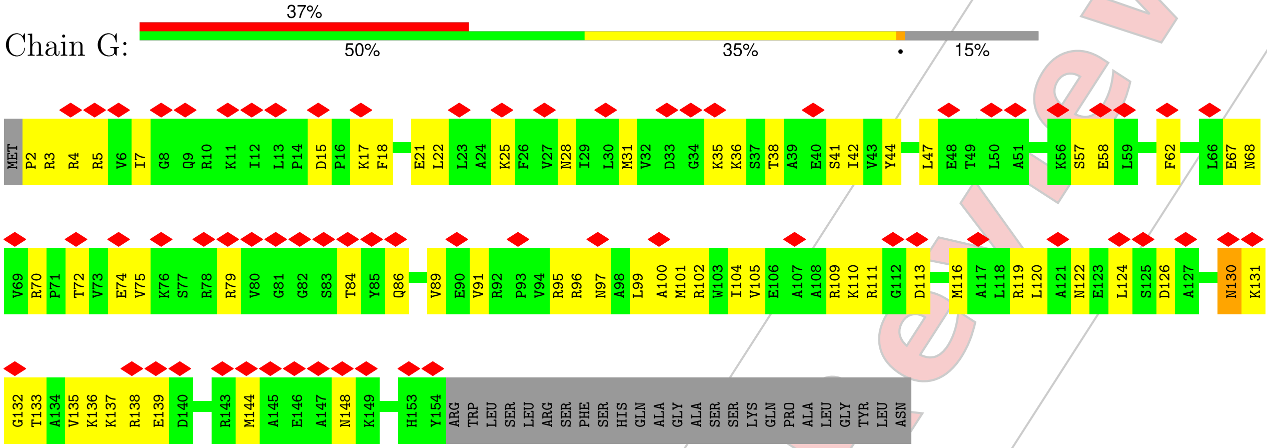

- Molecule 14: Small ribosomal subunit protein uS8

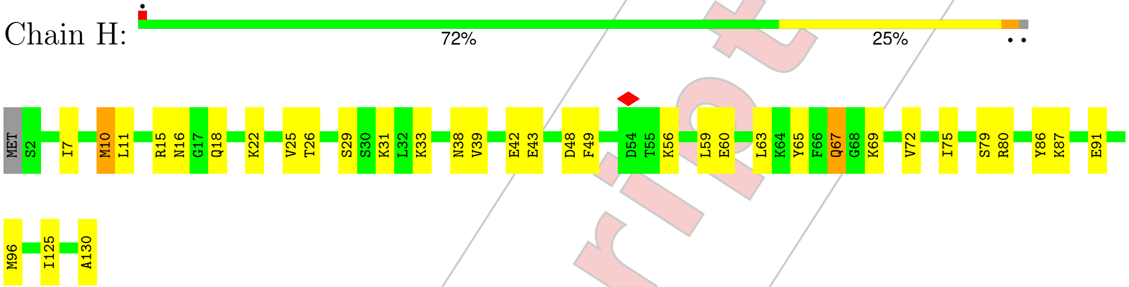

- Molecule 15: Small ribosomal subunit protein uS9

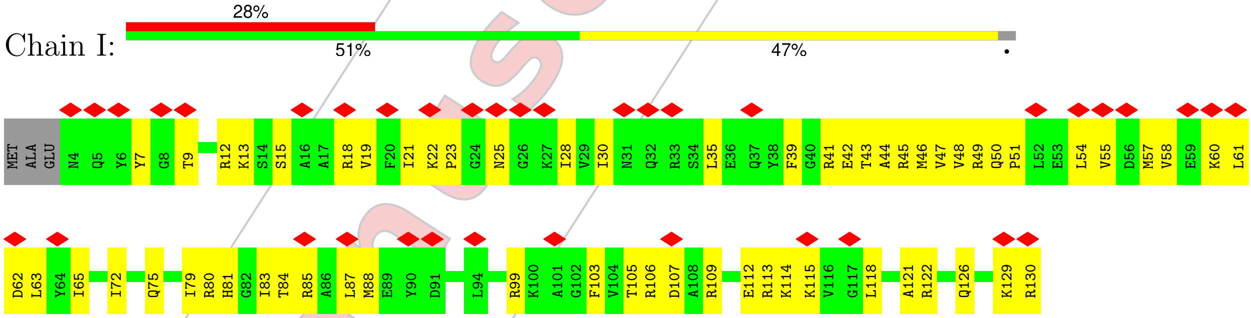

- Molecule 16: Small ribosomal subunit protein uS10

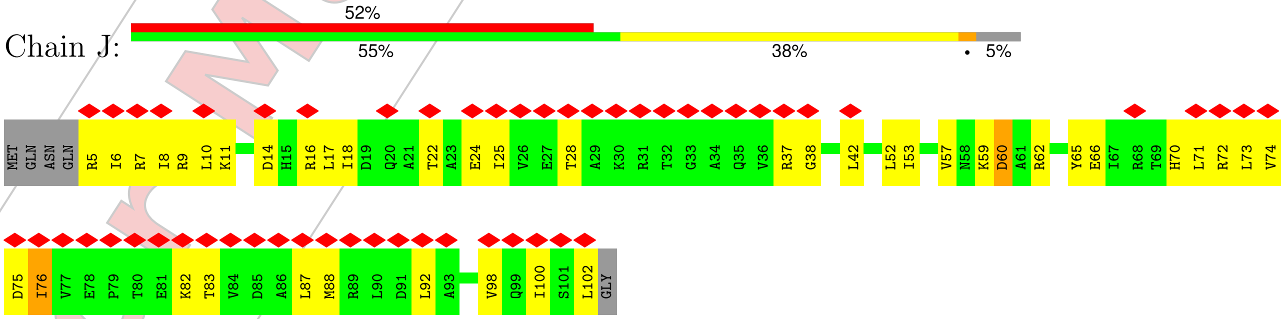

- Molecule 17: Small ribosomal subunit protein uS11

For Manuscript Review

Chain K:

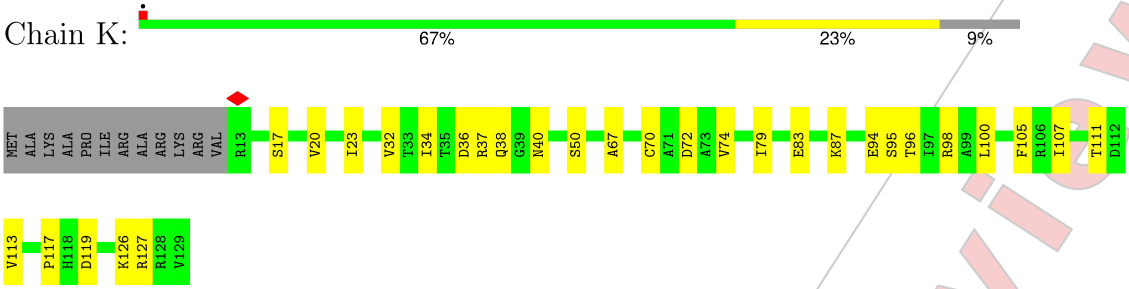

• Molecule 18: Small ribosomal subunit protein uS12

Chain L:

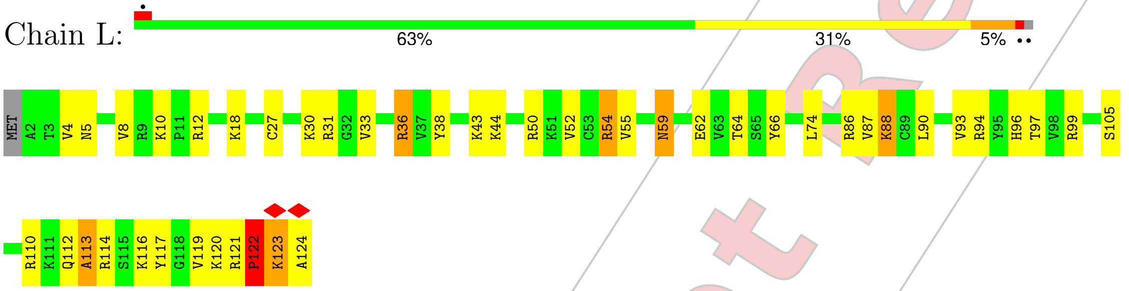

• Molecule 19: Small ribosomal subunit protein uS13

Chain M:

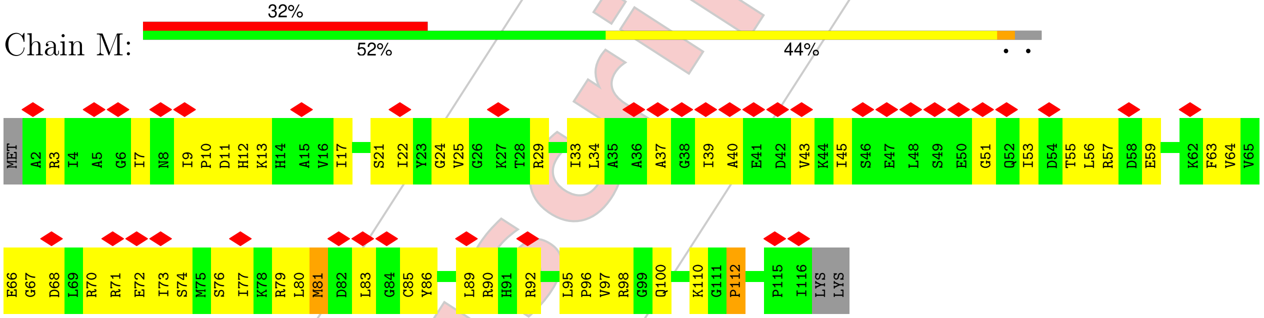

• Molecule 20: Small ribosomal subunit protein uS14

Chain N:

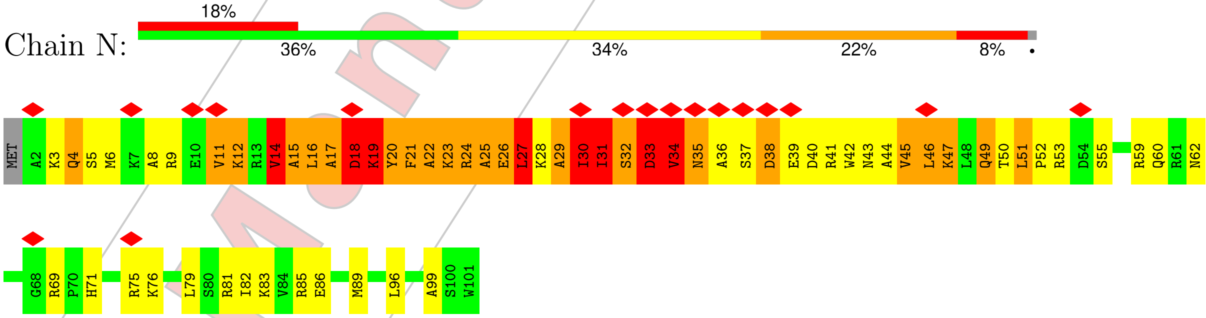

• Molecule 21: Small ribosomal subunit protein uS15

Chain O:

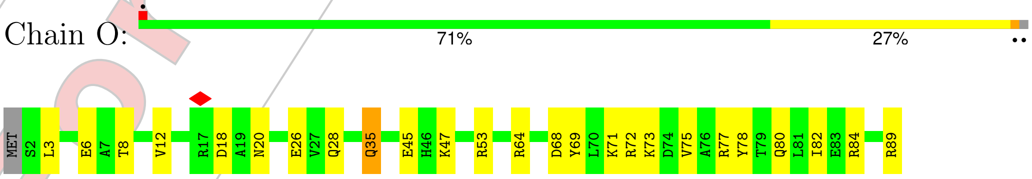

• Molecule 22: Small ribosomal subunit protein bS16

For Manuscript Review

Chain P:

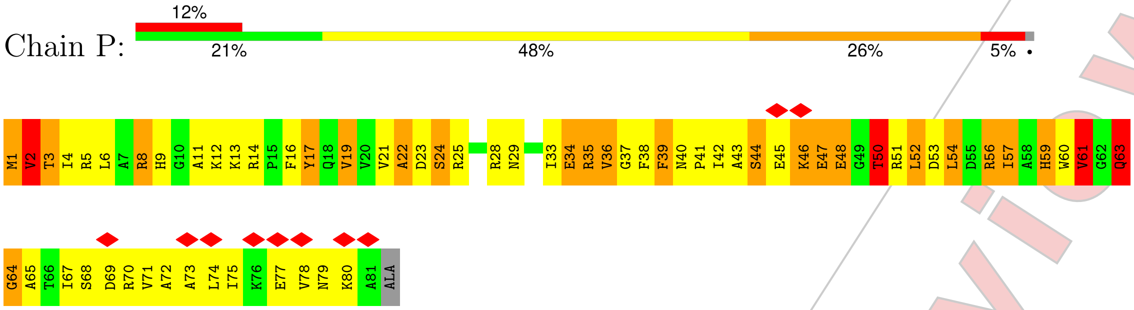

• Molecule 23: Small ribosomal subunit protein uS17

Chain Q:

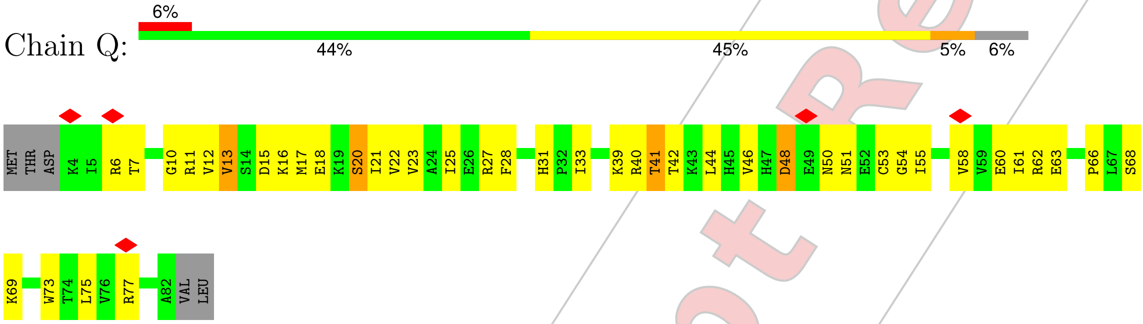

• Molecule 24: Small ribosomal subunit protein bS18

Chain R:

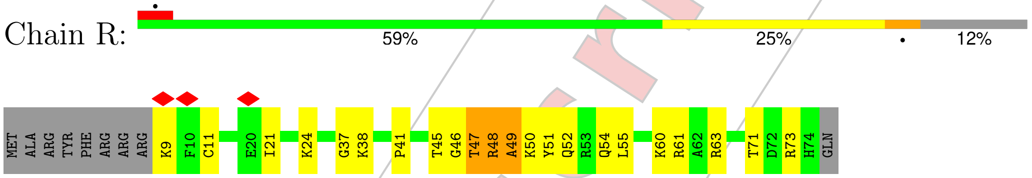

• Molecule 25: Small ribosomal subunit protein uS19

Chain S:

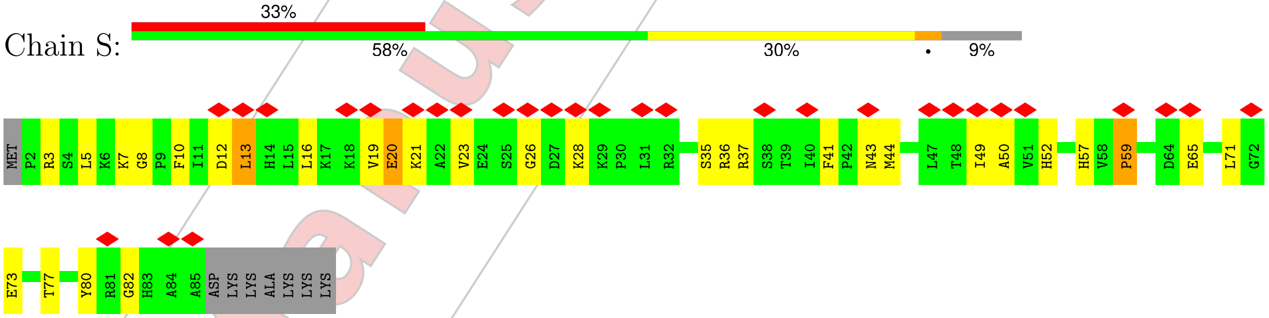

• Molecule 26: Small ribosomal subunit protein bS20

Chain T:

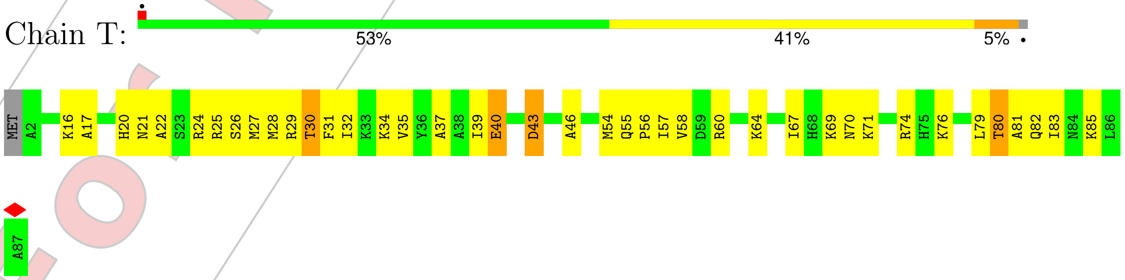

For Manuscript Review

- Molecule 27: Small ribosomal subunit protein bS21

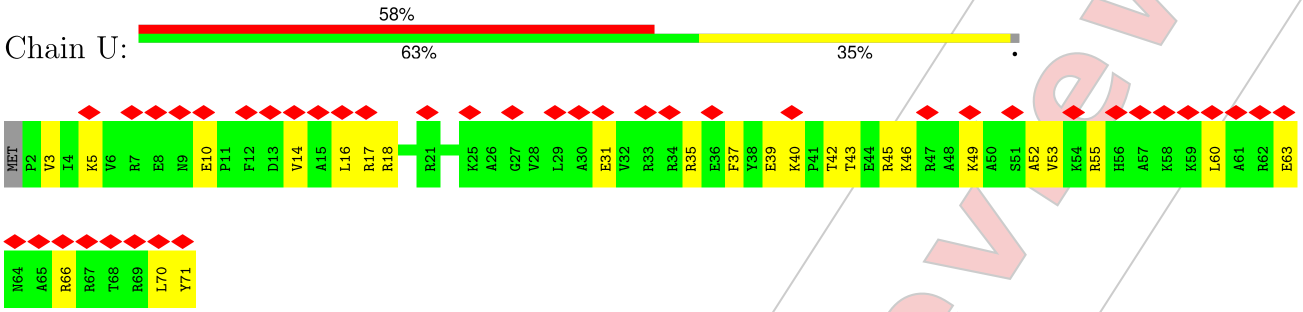

- Molecule 28: 23S ribosomal RNA

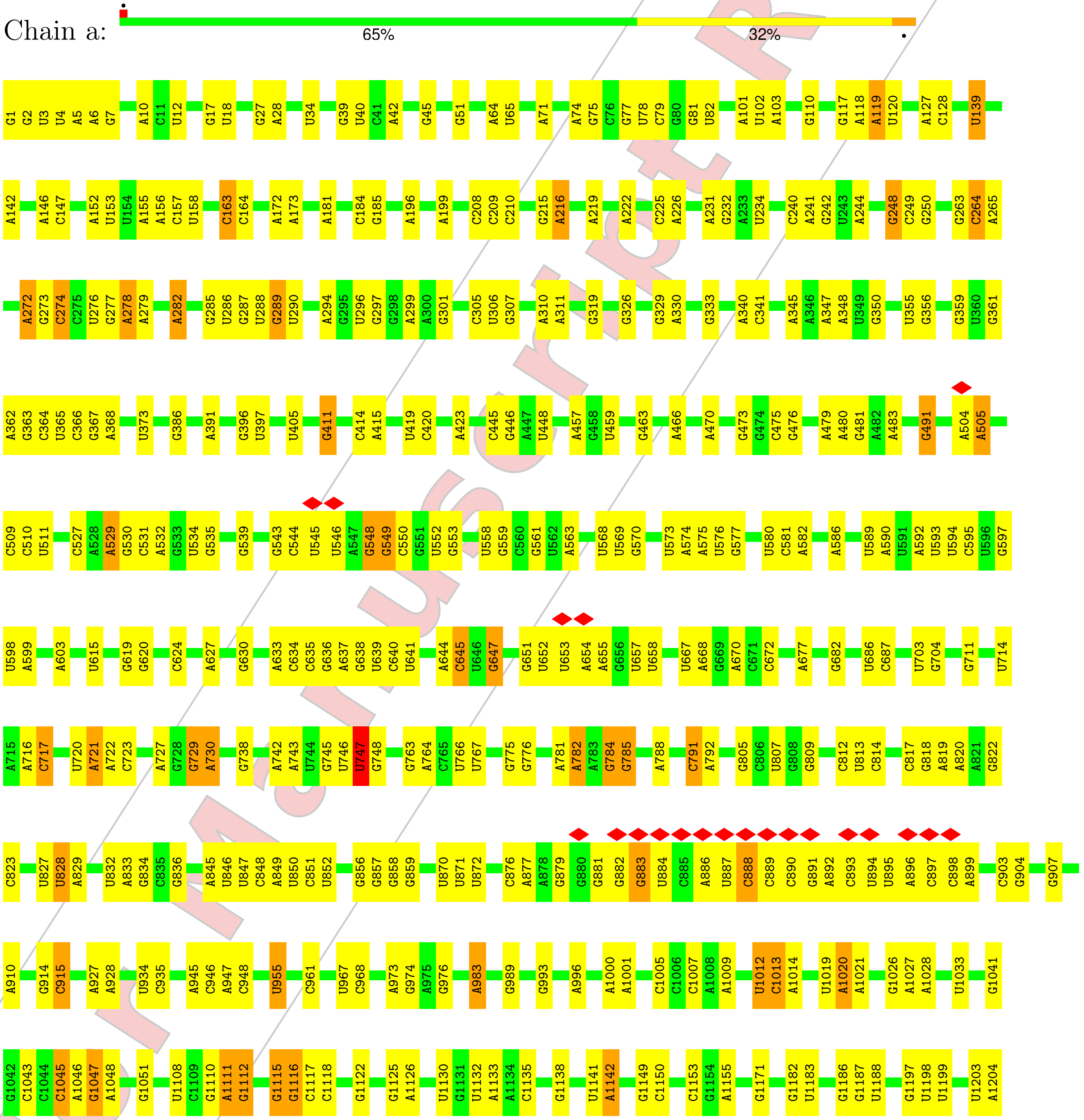

For Manuscript Review

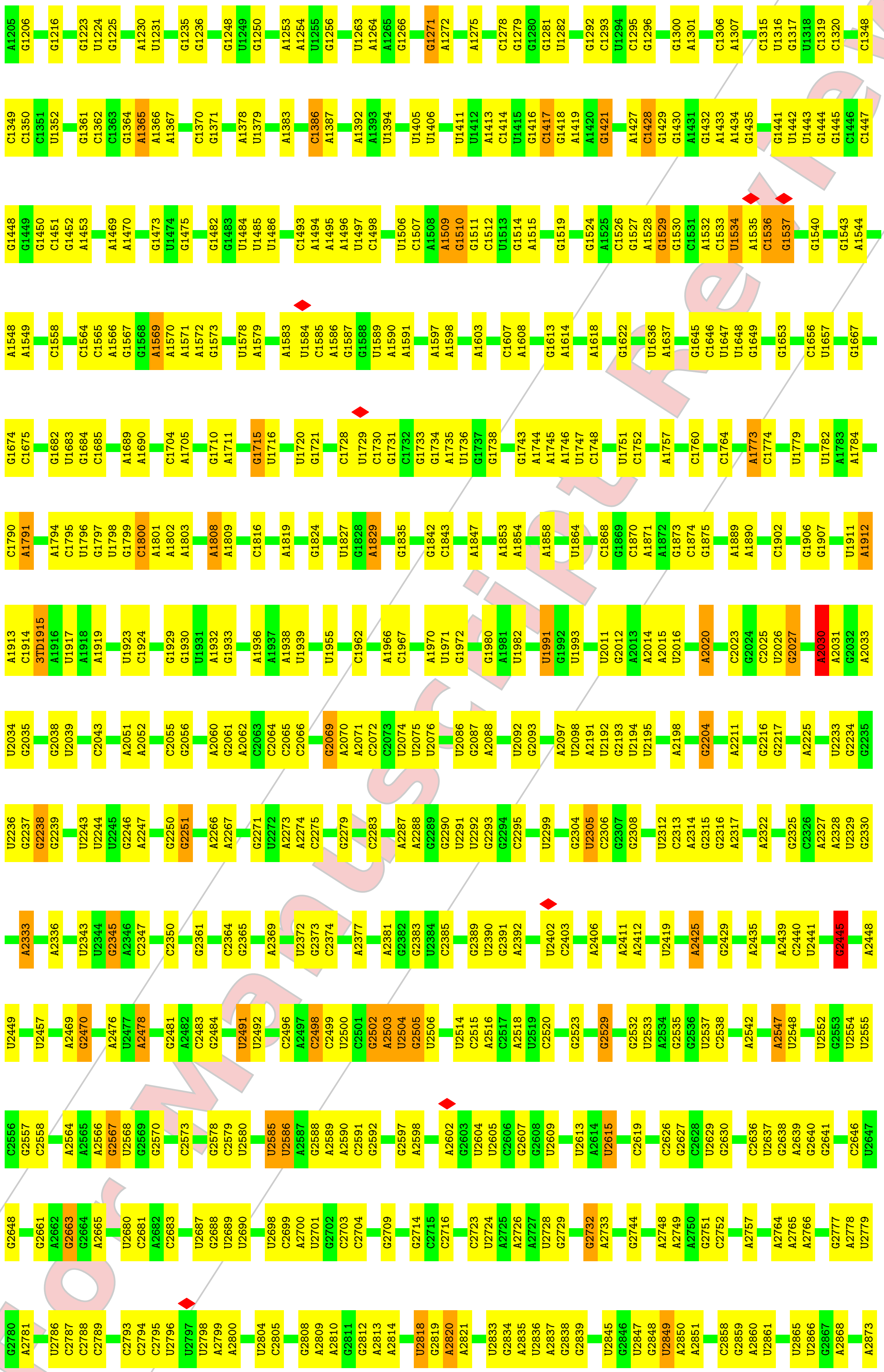

For Manuscript Review

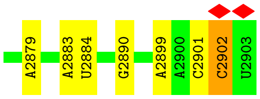

• Molecule 29: 5S ribosomal RNA

Chain b:

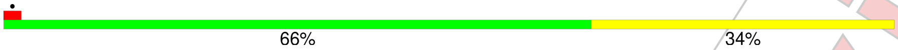

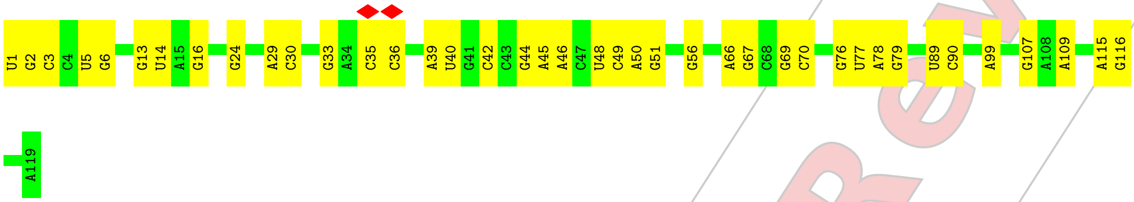

• Molecule 30: 50S ribosomal protein L2

Chain c:

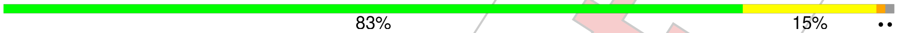

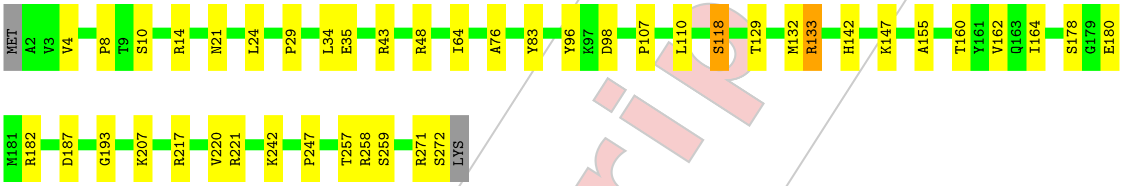

• Molecule 31: 50S ribosomal protein L3

Chain d:

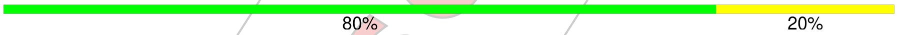

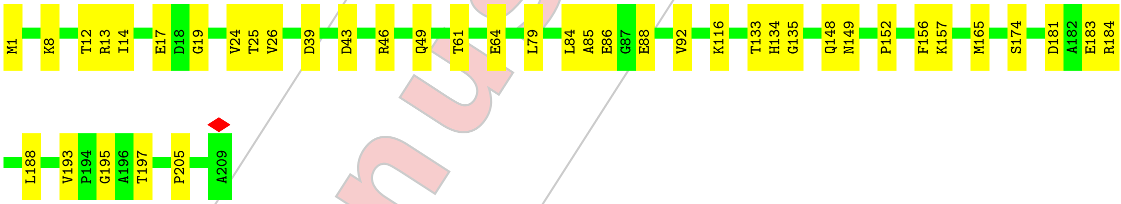

• Molecule 32: Large ribosomal subunit protein uL4

Chain e:

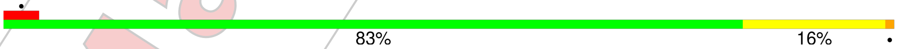

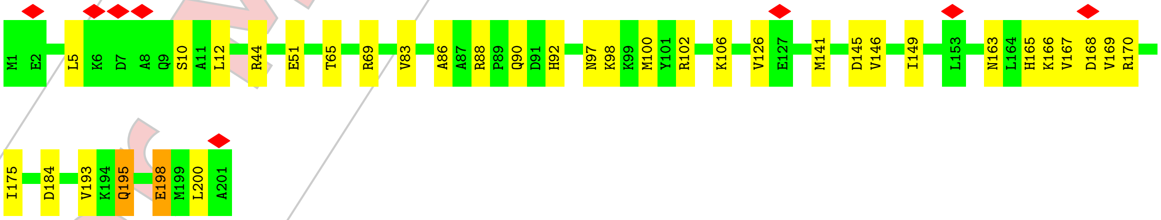

• Molecule 33: Large ribosomal subunit protein uL5

Chain f:

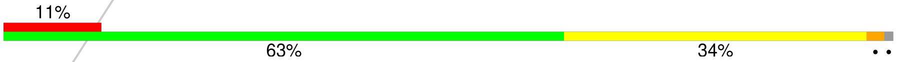

For Manuscript Review

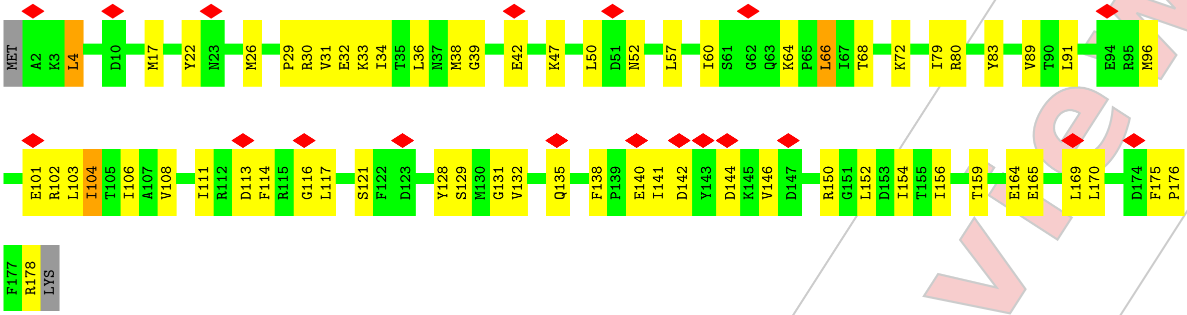

• Molecule 34: Large ribosomal subunit protein uL6

Chain g: 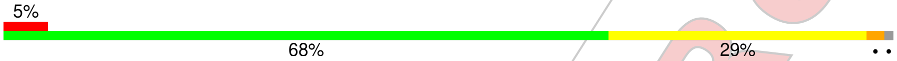

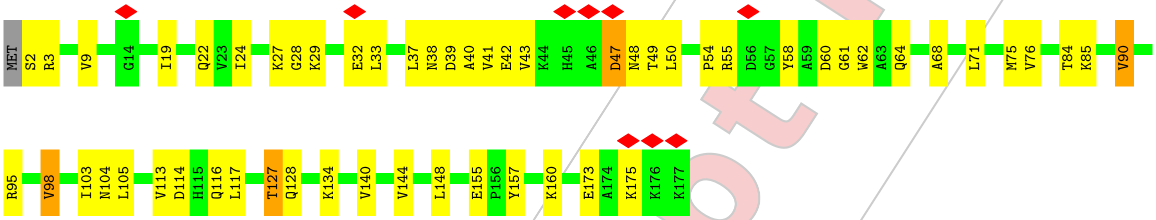

• Molecule 35: Large ribosomal subunit protein bL9

Chain h: 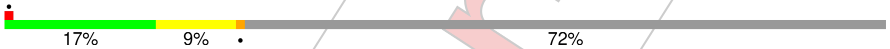

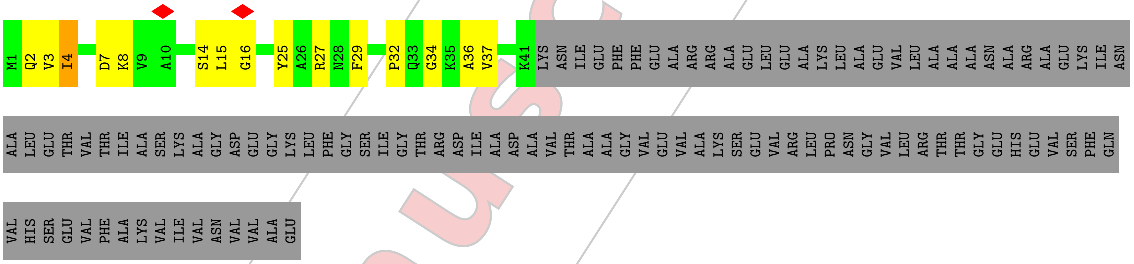

• Molecule 36: Large ribosomal subunit protein uL13

Chain i: 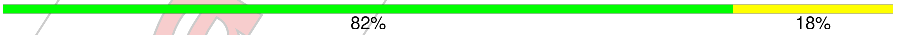

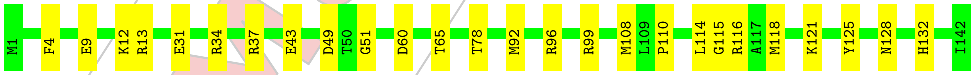

• Molecule 37: Large ribosomal subunit protein uL14

Chain j: 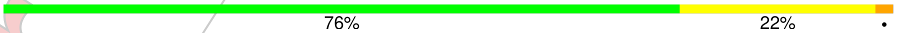

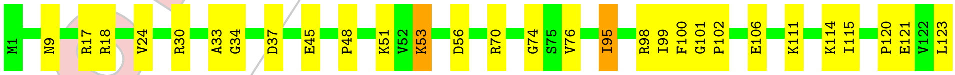

• Molecule 38: Large ribosomal subunit protein uL15

For Manuscript Review

Chain k:

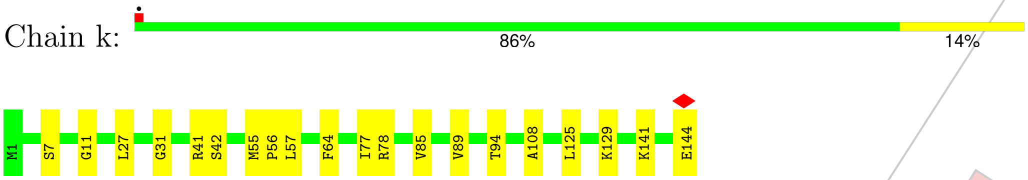

• Molecule 39: Large ribosomal subunit protein uL16

Chain l:

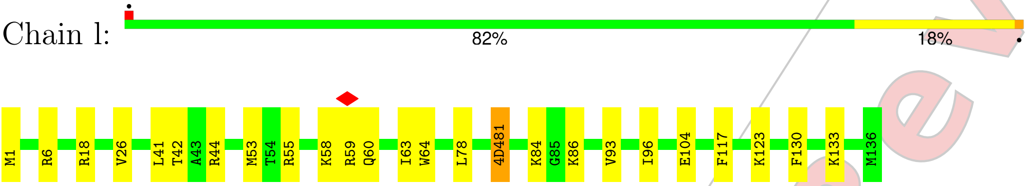

• Molecule 40: Large ribosomal subunit protein bL17

Chain m:

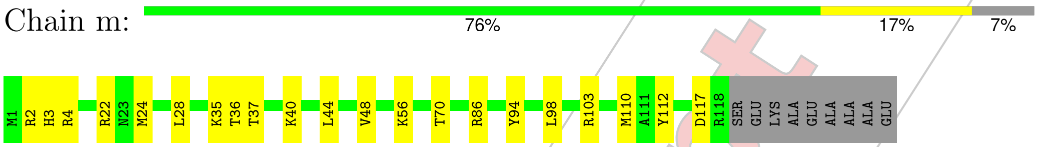

• Molecule 41: Large ribosomal subunit protein uL18

Chain n:

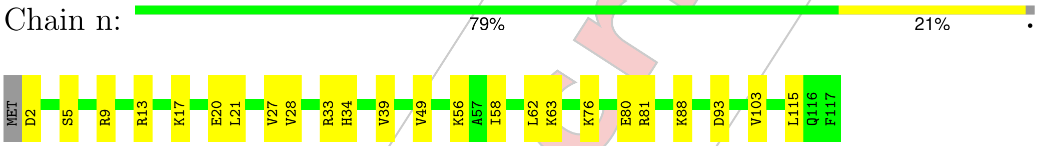

• Molecule 42: Large ribosomal subunit protein bL19

Chain o:

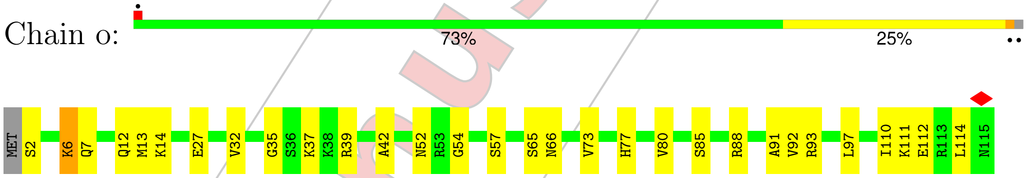

• Molecule 43: 50S ribosomal protein L20

Chain p:

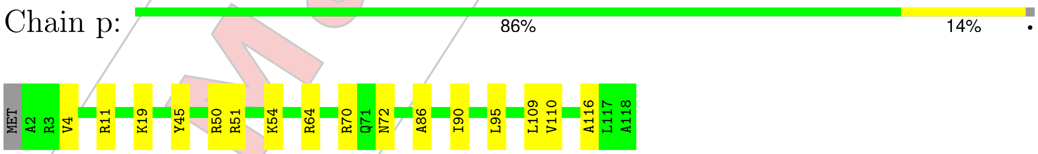

• Molecule 44: Large ribosomal subunit protein bL21

Chain q:

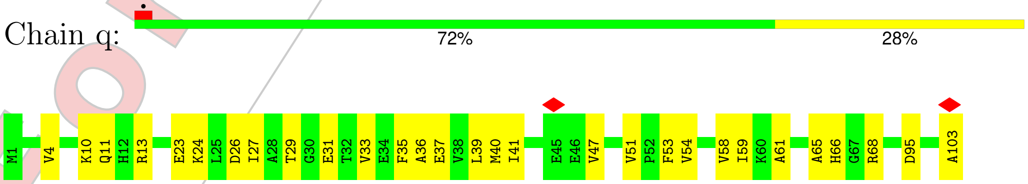

For Manuscript Review

• Molecule 45: Large ribosomal subunit protein uL22

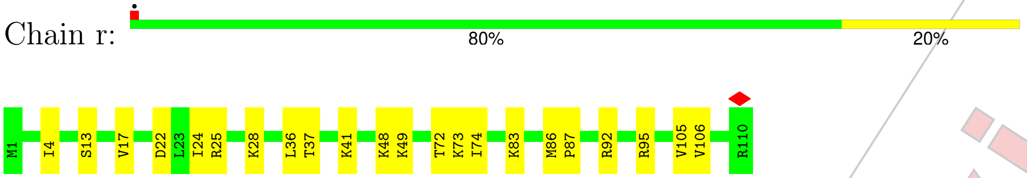

• Molecule 46: 50S ribosomal protein L23

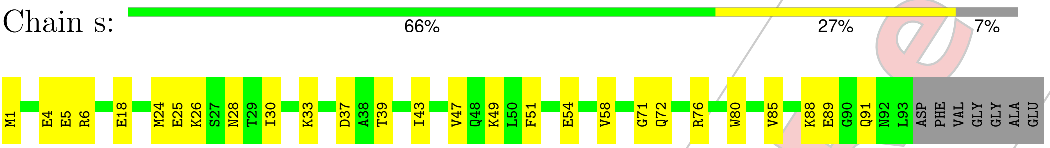

• Molecule 47: 50S ribosomal protein L24

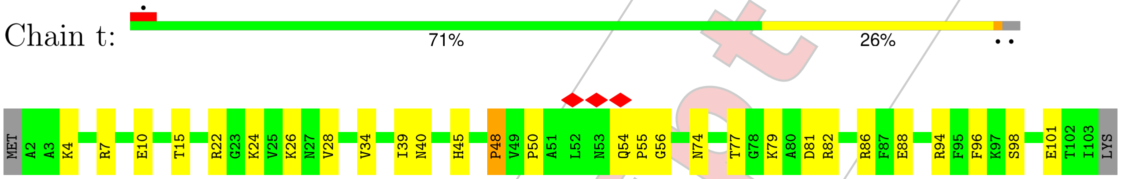

• Molecule 48: Large ribosomal subunit protein bL25

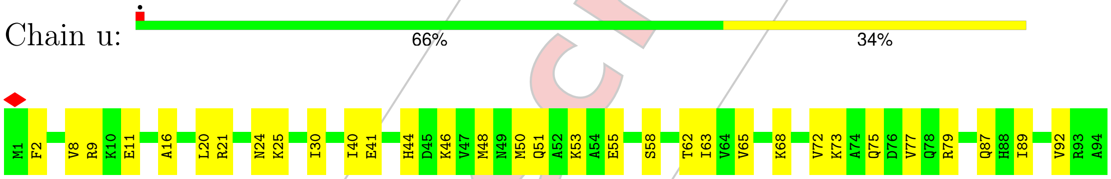

• Molecule 49: 50S ribosomal protein L27

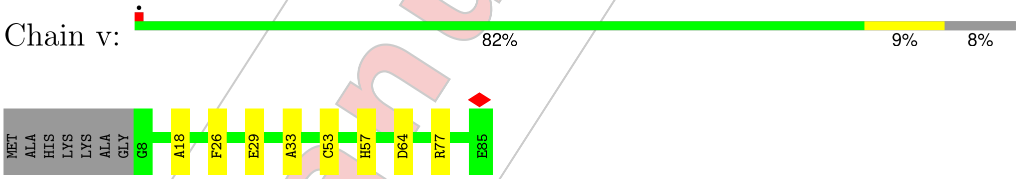

• Molecule 50: 50S ribosomal protein L28

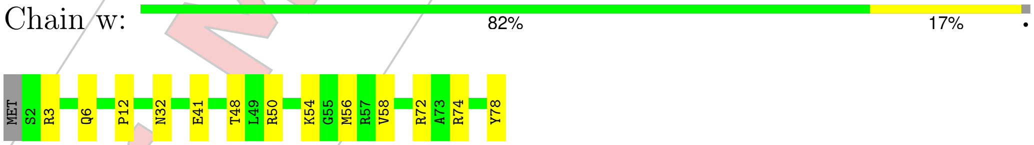

• Molecule 51: Large ribosomal subunit protein uL29

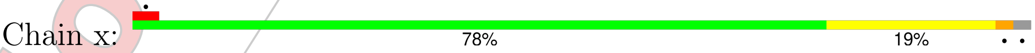

For Manuscript Review

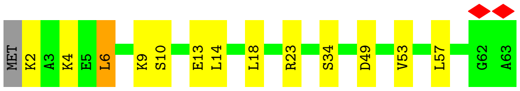

• Molecule 52: 50S ribosomal protein L30

Chain y:

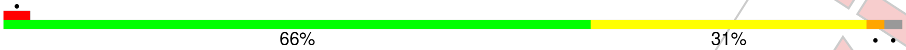

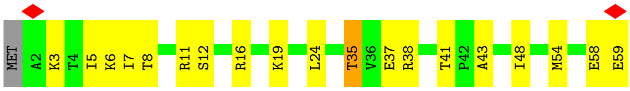

• Molecule 53: 50S ribosomal protein L32

Chain z:

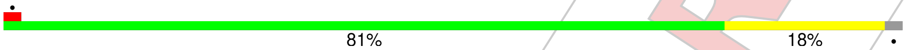

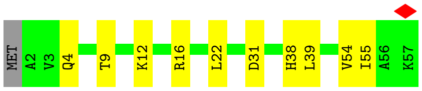

For Manuscript Review

4 Experimental information (i)

Property	Value	Source
EM reconstruction method	SINGLE PARTICLE	Depositor
Imposed symmetry	POINT, Not provided	
Number of particles used	592601	Depositor
Resolution determination method	FSC 0.143 CUT-OFF	Depositor
CTF correction method	PHASE FLIPPING AND AMPLITUDE CORRECTION	Depositor
Microscope	TFS KRIOS	Depositor
Voltage (kV)	300	Depositor
Electron dose $(e^-/\text{Å}^2)$	60	Depositor
Minimum defocus (nm)	500	Depositor
Maximum defocus (nm)	2500	Depositor
Magnification	Not provided	
Image detector	GATAN K3 BIOQUANTUM (6k x 4k)	Depositor
Maximum map value	28.928	Depositor
Minimum map value	-15.076	Depositor
Average map value	0.000	Depositor
Map value standard deviation	1.000	Depositor
Recommended contour level	2.5	Depositor
Map size (Å)	399.36002, 399.36002, 399.36002	wwPDB
Map dimensions	480, 480, 480	wwPDB
Map angles (°)	90.0, 90.0, 90.0	wwPDB
Pixel spacing (Å)	0.832, 0.832, 0.832	Depositor

For Manuscript Review

5 Model quality (i)

5.1 Standard geometry (i)

Bond lengths and bond angles in the following residue types are not validated in this section: 5MU, OMU, 4OC, IAS, SPD, PSU, 6MZ, MG, OMG, 2MG, SPM, MA6, ZN, G7M, MEQ, 5MC, H2U, UR3, 1MG, 3TD, OMC, 4D4, MS6, 2MA

The Z score for a bond length (or angle) is the number of standard deviations the observed value is removed from the expected value. A bond length (or angle) with $|Z| > 5$ is considered an outlier worth inspection. RMSZ is the root-mean-square of all Z scores of the bond lengths (or angles).

Mol	Chain	Bond lengths		Bond angles	
		RMSZ	#$\|Z\|$ >5	RMSZ	#$\|Z\|$ >5
1	0	0.25	0/424	0.42	0/565
2	1	0.33	0/380	0.35	0/498
3	2	0.30	0/513	0.40	0/676
4	3	0.32	0/303	0.39	0/397
5	4	0.16	0/488	0.47	0/649
6	5	0.17	0/46	0.11	0/69
7	A	0.30	0/36230	0.50	82/56509 (0.1%)
8	B	0.20	0/1784	0.51	2/2403 (0.1%)
9	C	0.22	0/1651	0.52	0/2225
10	D	0.33	0/1665	0.61	2/2227 (0.1%)
11	E	0.31	0/1165	0.58	0/1568
12	F	0.25	0/858	0.49	0/1160
13	G	0.18	0/1219	0.44	0/1635
14	H	0.26	0/989	0.43	0/1326
15	I	0.23	0/1034	0.56	0/1375
16	J	0.22	0/796	0.49	0/1077
17	K	0.23	0/884	0.45	0/1191
18	L	0.47	1/976 (0.1%)	0.82	1/1308 (0.1%)
19	M	0.26	0/900	0.66	3/1204 (0.2%)
20	N	1.20	11/817 (1.3%)	1.29	15/1088 (1.4%)
21	O	0.23	0/722	0.36	0/964
22	P	0.76	0/653	1.35	9/877 (1.0%)
23	Q	0.26	0/650	0.53	0/871
24	R	0.32	0/553	0.51	0/742
25	S	0.39	1/685 (0.1%)	0.79	4/922 (0.4%)
26	T	0.25	0/676	0.56	0/895
27	U	0.17	0/597	0.37	0/792
28	a	0.35	0/65651	0.31	0/102413
29	b	0.24	0/2850	0.26	0/4444
30	c	0.36	0/2121	0.40	0/2852
31	d	0.34	0/1576	0.41	0/2119

For Manuscript Review

Mol	Chain	Bond lengths		Bond angles	
		RMSZ	#|Z| >5	RMSZ	#|Z| >5
32	e	0.29	0/1571	0.40	0/2113
33	f	0.24	0/1434	0.51	0/1926
34	g	0.25	0/1343	0.56	0/1816
35	h	0.23	0/306	0.66	0/413
36	i	0.31	0/1152	0.37	0/1551
37	j	0.35	0/966	0.49	1/1293 (0.1%)
38	k	0.31	0/1062	0.42	0/1413
39	l	0.36	0/1073	0.44	0/1433
40	m	0.34	0/958	0.42	0/1281
41	n	0.22	0/902	0.44	0/1209
42	o	0.33	0/929	0.41	0/1242
43	p	0.33	0/960	0.35	0/1278
44	q	0.30	0/829	0.47	0/1107
45	r	0.30	0/864	0.35	0/1156
46	s	0.27	0/744	0.44	0/994
47	t	0.26	0/787	0.52	2/1051 (0.2%)
48	u	0.27	0/766	0.44	0/1025
49	v	0.32	0/593	0.37	0/785
50	w	0.36	0/635	0.38	0/848
51	x	0.22	0/502	0.43	0/667
52	y	0.30	0/453	0.47	0/605
53	z	0.32	0/450	0.40	0/599
All	All	0.33	13/149135 (0.0%)	0.43	121/222846 (0.1%)

Chiral center outliers are detected by calculating the chiral volume of a chiral center and verifying if the center is modelled as a planar moiety or with the opposite hand.A planarity outlier is detected by checking planarity of atoms in a peptide group, atoms in a mainchain group or atoms of a sidechain that are expected to be planar.

Mol	Chain	#Chirality outliers	#Planarity outliers
9	C	0	1
34	g	0	1
39	l	0	1
All	All	0	3

All (13) bond length outliers are listed below:

Mol	Chain	Res	Type	Atoms	Z	Observed(Å)	Ideal(Å)
20	N	19	LYS	C-O	12.49	1.39	1.24
20	N	30	ILE	C-O	11.27	1.37	1.24
20	N	18	ASP	C-O	8.94	1.35	1.24
20	N	11	VAL	C-O	6.80	1.31	1.24

Continued on next page...

For Manuscript Review

Continued from previous page...

Mol	Chain	Res	Type	Atoms	Z	Observed(Å)	Ideal(Å)
20	N	22	ALA	C-O	6.60	1.32	1.24
25	S	59	PRO	CG-CD	-6.17	1.29	1.50
20	N	34	VAL	N-CA	5.74	1.53	1.46
20	N	15	ALA	C-O	5.40	1.30	1.24
20	N	23	LYS	N-CA	5.30	1.52	1.46
18	L	113	ALA	CA-CB	-5.29	1.45	1.53
20	N	14	VAL	C-O	5.18	1.30	1.24
20	N	27	LEU	C-O	5.18	1.30	1.24
20	N	26	GLU	C-O	5.00	1.29	1.24

All (121) bond angle outliers are listed below:

Mol	Chain	Res	Type	Atoms	Z	Observed(^{o})	Ideal(^{o})
19	M	112	PRO	CA-N-CD	-11.29	96.19	112.00
25	S	59	PRO	CA-N-CD	-11.03	96.56	112.00
7	A	412	C	P-O3'-C3'	-10.77	104.05	120.20
25	S	59	PRO	N-CD-CG	-10.47	87.49	103.20
7	A	402	G	P-O3'-C3'	-10.09	105.07	120.20
7	A	1381	U	P-O3'-C3'	-10.07	105.09	120.20
7	A	551	U	P-O3'-C3'	-10.01	105.19	120.20
7	A	1378	C	P-O3'-C3'	-9.30	106.25	120.20
7	A	90	C	P-O3'-C3'	-9.30	106.25	120.20
7	A	37	U	P-O3'-C3'	-9.28	106.29	120.20
7	A	433	G	P-O3'-C3'	-9.22	106.37	120.20
7	A	77	A	P-O3'-C3'	-9.21	106.38	120.20
7	A	78	A	P-O3'-C3'	-9.18	106.43	120.20
7	A	608	A	P-O3'-C3'	-9.14	106.49	120.20
7	A	81	A	P-O3'-C3'	-9.06	106.61	120.20
7	A	1368	A	P-O3'-C3'	-9.02	106.68	120.20
7	A	435	A	P-O3'-C3'	-8.84	106.94	120.20
7	A	401	C	P-O3'-C3'	-8.74	107.09	120.20
7	A	496	A	P-O3'-C3'	-8.72	107.12	120.20
7	A	431	A	P-O3'-C3'	-8.53	107.41	120.20
7	A	437	U	P-O3'-C3'	-8.50	107.45	120.20
7	A	764	C	P-O3'-C3'	-8.47	107.49	120.20
7	A	409	U	P-O3'-C3'	-8.43	107.56	120.20
7	A	40	C	P-O3'-C3'	-8.42	107.57	120.20
7	A	550	G	P-O3'-C3'	-8.42	107.57	120.20
7	A	1379	G	P-O3'-C3'	-8.40	107.60	120.20
7	A	91	U	P-O3'-C3'	-8.31	107.74	120.20
7	A	552	U	P-O3'-C3'	-8.25	107.83	120.20
7	A	408	A	P-O3'-C3'	-8.21	107.89	120.20

Continued on next page...

For Manuscript Review

Continued from previous page...

Mol	Chain	Res	Type	Atoms	Z	Observed(^{o})	Ideal(^{o})
7	A	39	G	P-O3'-C3'	-8.13	108.01	120.20
7	A	403	C	P-O3'-C3'	-8.08	108.08	120.20
7	A	432	A	P-O3'-C3'	-7.94	108.30	120.20
7	A	605	U	P-O3'-C3'	-7.80	108.50	120.20
22	P	59	HIS	CA-C-N	-7.78	108.81	122.26
22	P	59	HIS	C-N-CA	-7.78	108.81	122.26
7	A	1380	U	P-O3'-C3'	-7.76	108.56	120.20
7	A	88	U	P-O3'-C3'	-7.70	108.64	120.20
7	A	87	C	P-O3'-C3'	-7.68	108.67	120.20
7	A	41	G	P-O3'-C3'	-7.56	108.86	120.20
7	A	430	A	P-O3'-C3'	-7.56	108.86	120.20
7	A	1284	C	P-O3'-C3'	-7.50	108.95	120.20
7	A	501	C	P-O3'-C3'	-7.48	108.97	120.20
7	A	609	A	P-O3'-C3'	-7.44	109.04	120.20
7	A	413	G	P-O3'-C3'	-7.37	109.15	120.20
7	A	407	U	P-O3'-C3'	-7.33	109.20	120.20
7	A	438	U	P-O3'-C3'	-7.10	109.56	120.20
7	A	1367	C	P-O3'-C3'	-7.02	109.67	120.20
7	A	440	C	P-O3'-C3'	-7.01	109.68	120.20
7	A	530	G	P-O3'-C3'	-6.85	109.92	120.20
7	A	1366	C	P-O3'-C3'	-6.72	110.12	120.20
47	t	48	PRO	CA-N-CD	-6.66	102.68	112.00
7	A	38	G	P-O3'-C3'	-6.61	110.28	120.20
7	A	89	U	P-O3'-C3'	-6.59	110.31	120.20
8	B	63	ARG	CB-CG-CD	6.56	126.39	111.30
7	A	79	G	P-O3'-C3'	-6.54	110.40	120.20
7	A	436	C	P-O3'-C3'	-6.47	110.49	120.20
25	S	59	PRO	CA-CB-CG	-6.46	92.23	104.50
7	A	1414	U	P-O3'-C3'	-6.41	110.58	120.20
7	A	1517	G	C2'-C3'-O3'	6.40	123.30	113.70
7	A	494	G	P-O3'-C3'	-6.39	110.62	120.20
7	A	411	A	P-O3'-C3'	-6.37	110.65	120.20
7	A	120	A	P-O3'-C3'	-6.36	110.65	120.20
7	A	1286	U	P-O3'-C3'	-6.36	110.66	120.20
7	A	1516	2MG	P-O3'-C3'	-6.36	110.65	120.20
7	A	92	U	P-O3'-C3'	-6.30	110.75	120.20
7	A	82	G	P-O3'-C3'	-6.24	110.85	120.20
7	A	434	U	P-O3'-C3'	-6.21	110.89	120.20
7	A	80	A	P-O3'-C3'	-6.21	110.89	120.20
18	L	122	PRO	N-CA-CB	-6.20	96.74	103.25
7	A	406	G	P-O3'-C3'	-6.20	110.91	120.20
20	N	21	PHE	CA-C-N	-6.18	109.74	121.54

Continued on next page...

For Manuscript Review

Continued from previous page...

Mol	Chain	Res	Type	Atoms	Z	Observed(^{o})	Ideal(^{o})
20	N	21	PHE	C-N-CA	-6.18	109.74	121.54
20	N	25	ALA	N-CA-C	-6.14	103.53	111.02
22	P	57	ILE	N-CA-C	-6.12	104.37	110.62
7	A	121	U	P-O3'-C3'	-6.12	111.02	120.20
7	A	611	C	P-O3'-C3'	-6.11	111.03	120.20
7	A	1287	A	P-O3'-C3'	-6.10	111.05	120.20
7	A	495	A	C4'-C3'-O3'	6.08	118.52	109.40
19	M	112	PRO	N-CD-CG	-6.07	94.10	103.20
22	P	61	VAL	N-CA-C	-6.07	104.94	110.82
7	A	549	C	P-O3'-C3'	-6.02	111.17	120.20
7	A	517	G	P-O3'-C3'	-6.01	111.18	120.20
7	A	610	U	P-O3'-C3'	-5.91	111.33	120.20
7	A	548	G	C2'-C3'-O3'	5.84	122.47	113.70
7	A	531	U	P-O3'-C3'	-5.82	111.47	120.20
22	P	17	TYR	CB-CA-C	5.75	119.48	109.65
10	D	23	SER	CB-CA-C	-5.75	109.93	116.54
7	A	1517	G	C4'-C3'-C2'	-5.72	96.88	102.60
7	A	498	A	P-O3'-C3'	-5.69	111.66	120.20
22	P	63	GLN	CA-C-N	-5.66	110.32	121.41
22	P	63	GLN	C-N-CA	-5.66	110.32	121.41
37	j	95	ILE	N-CA-C	-5.65	106.88	111.91
25	S	20	GLU	N-CA-C	-5.64	105.05	111.14
7	A	439	U	P-O3'-C3'	-5.64	111.74	120.20
20	N	33	ASP	CA-C-N	-5.63	111.84	121.97
20	N	33	ASP	C-N-CA	-5.63	111.84	121.97
20	N	19	LYS	CA-C-N	-5.62	110.81	121.54
20	N	19	LYS	C-N-CA	-5.62	110.81	121.54
20	N	21	PHE	CB-CA-C	-5.61	99.25	110.42
22	P	56	ARG	N-CA-C	-5.60	107.10	114.04
7	A	410	G	P-O3'-C3'	-5.57	111.85	120.20
10	D	150	LYS	N-CA-C	-5.52	104.29	111.74
7	A	85	U	P-O3'-C3'	-5.49	111.97	120.20
20	N	34	VAL	CA-C-O	-5.31	114.14	120.78
20	N	34	VAL	CA-C-N	-5.27	111.47	121.54
20	N	34	VAL	C-N-CA	-5.27	111.47	121.54
20	N	14	VAL	CA-C-N	-5.27	113.27	120.44
20	N	14	VAL	C-N-CA	-5.27	113.27	120.44
7	A	1285	A	P-O3'-C3'	-5.24	112.34	120.20
19	M	66	GLU	CB-CA-C	-5.22	110.13	117.23
7	A	604	G	P-O3'-C3'	-5.21	112.38	120.20
7	A	548	G	C4'-C3'-C2'	-5.19	97.41	102.60
8	B	63	ARG	CA-CB-CG	5.19	124.47	114.10

Continued on next page...

For Manuscript Review

Continued from previous page...

Mol	Chain	Res	Type	Atoms	Z	Observed(°)	Ideal(°)
7	A	536	C	P-O3'-C3'	-5.16	112.46	120.20
7	A	500	G	C4'-C3'-C2'	-5.13	97.47	102.60
7	A	1369	C	P-O3'-C3'	-5.12	112.52	120.20
20	N	4	GLN	N-CA-CB	-5.05	102.45	110.28
22	P	39	PHE	CB-CA-C	-5.05	102.61	110.74
7	A	493	A	P-O3'-C3'	-5.04	112.63	120.20
20	N	19	LYS	CA-C-O	5.04	127.72	120.51
47	t	48	PRO	N-CD-CG	-5.02	95.67	103.20

There are no chirality outliers.

All (3) planarity outliers are listed below:

Mol	Chain	Res	Type	Group
9	C	78	GLY	Peptide
34	g	47	ASP	Peptide
39	l	81	4D4	Mainchain

5.2 Too-close contacts (i)

In the following table, the Non-H and H(model) columns list the number of non-hydrogen atoms and hydrogen atoms in the chain respectively. The H(added) column lists the number of hydrogen atoms added and optimized by MolProbity. The Clashes column lists the number of clashes within the asymmetric unit, whereas Symm-Clashes lists symmetry-related clashes.

Mol	Chain	Non-H	H(model)	H(added)	Clashes	Symm-Clashes
1	0	417	0	451	14	0
2	1	377	0	418	9	0
3	2	504	0	572	11	0
4	3	302	0	340	9	0
5	4	480	0	478	28	0
6	5	42	0	23	0	0
7	A	32607	0	16431	723	0
8	B	1753	0	1780	62	0
9	C	1624	0	1696	52	0
10	D	1643	0	1707	68	0
11	E	1152	0	1196	40	0
12	F	839	0	833	30	0
13	G	1203	0	1254	48	0
14	H	979	0	1031	24	0
15	I	1022	0	1070	64	0
16	J	786	0	828	36	0

Continued on next page...

For Manuscript Review

Continued from previous page...

Mol	Chain	Non-H	H(model)	H(added)	Clashes	Symm-Clashes
17	K	877	0	882	23	0
18	L	962	0	1029	36	0
19	M	891	0	952	45	0
20	N	805	0	844	89	0
21	O	714	0	734	19	0
22	P	643	0	661	100	0
23	Q	641	0	682	30	0
24	R	544	0	565	25	0
25	S	668	0	693	34	0
26	T	670	0	719	33	0
27	U	589	0	629	16	0
28	a	59130	0	29768	566	0
29	b	2549	0	1291	23	0
30	c	2082	0	2154	32	0
31	d	1566	0	1618	29	0
32	e	1552	0	1619	20	0
33	f	1410	0	1444	61	0
34	g	1323	0	1371	39	0
35	h	303	0	327	13	0
36	i	1129	0	1162	23	0
37	j	957	0	1035	23	0
38	k	1053	0	1129	14	0
39	l	1075	0	1145	21	0
40	m	945	0	989	14	0
41	n	892	0	923	17	0
42	o	917	0	962	22	0
43	p	947	0	1019	12	0
44	q	816	0	839	18	0
45	r	857	0	922	17	0
46	s	738	0	807	20	0
47	t	779	0	831	16	0
48	u	753	0	780	23	0
49	v	586	0	596	6	0
50	w	625	0	652	9	0
51	x	501	0	531	14	0
52	y	449	0	488	12	0
53	z	444	0	458	12	0
54	3	1	0	0	0	0
54	4	1	0	0	0	0
55	A	89	0	0	0	0
55	N	1	0	0	0	0
55	Q	1	0	0	0	0

Continued on next page...

For Manuscript Review

Continued from previous page...

Mol	Chain	Non-H	H(model)	H(added)	Clashes	Symm-Clashes
55	a	208	0	0	0	0
55	b	5	0	0	0	0
55	c	1	0	0	0	0
55	p	1	0	0	0	0
55	z	1	0	0	0	0
56	A	10	0	19	2	0
56	a	130	0	247	20	0
57	a	14	0	26	1	0
58	a	20	0	12	0	0
59	a	23	0	12	0	0
All	All	138618	0	93674	2490	0

The all-atom clashscore is defined as the number of clashes found per 1000 atoms (including hydrogen atoms). The all-atom clashscore for this structure is 11.

All (2490) close contacts within the same asymmetric unit are listed below, sorted by their clash magnitude.

Atom-1	Atom-2	Interatomic distance (Å)	Clash overlap (Å)
20:N:18:ASP:O	20:N:21:PHE:HB3	1.59	1.00
7:A:39:G:H1	7:A:403:C:H5	1.08	0.96
37:j:121:GLU:OE1	42:o:66:ASN:ND2	1.99	0.95
11:E:70:ASN:O	11:E:70:ASN:ND2	2.03	0.92
7:A:83:C:H5'	7:A:84:U:H3'	1.52	0.92
22:P:39:PHE:CG	22:P:74:LEU:HD11	2.05	0.91
7:A:458:U:H3	7:A:474:G:H1	1.15	0.90
7:A:83:C:H5"	7:A:86:G:H1	1.37	0.89
7:A:78:A:H2'	7:A:79:G:C8	2.08	0.88
20:N:27:LEU:HD21	20:N:47:LYS:HB3	1.57	0.87
10:D:28:ILE:HG21	10:D:34:ILE:HD13	1.53	0.87
7:A:392:C:H5"	22:P:13:LYS:HE2	1.57	0.87
18:L:36:ARG:HB3	18:L:36:ARG:HH11	1.40	0.86
20:N:11:VAL:O	20:N:14:VAL:HG12	1.76	0.86
20:N:17:ALA:O	20:N:18:ASP:C	2.19	0.86
22:P:39:PHE:CZ	22:P:41:PRO:HA	2.11	0.86
22:P:39:PHE:CD2	22:P:74:LEU:HD11	2.12	0.85
15:I:21:ILE:HD13	15:I:63:LEU:HD13	1.59	0.84
7:A:1242:G:H1	7:A:1295:U:H3	1.22	0.84
7:A:413:G:H1'	7:A:428:G:H21	1.39	0.84
47:t:74:ASN:HD22	47:t:77:THR:HG22	1.41	0.84
7:A:664:G:H22	7:A:741:G:H1	1.24	0.84
7:A:500:G:H2'	7:A:501:C:C6	2.14	0.83

Continued on next page...

For Manuscript Review

Continued from previous page...

Atom-1	Atom-2	Interatomic distance (Å)	Clash overlap (Å)
7:A:81:A:H2'	7:A:82:G:C8	2.14	0.83
7:A:437:U:H4'	10:D:152:GLN:HE22	1.43	0.83
28:a:2469:A:H4'	39:l:55:ARG:HD3	1.60	0.82
11:E:70:ASN:HD22	11:E:70:ASN:C	1.81	0.82
32:e:97:ASN:HB2	32:e:100:MET:HG3	1.61	0.82
16:J:82:LYS:H	16:J:82:LYS:HD2	1.44	0.81
24:R:47:THR:HG22	24:R:48:ARG:H	1.45	0.81
10:D:48:LEU:HD21	10:D:56:ARG:HG3	1.62	0.81
39:l:64:TRP:HB2	39:l:104:GLU:HB2	1.60	0.81
14:H:10:MET:HE2	14:H:33:LYS:HG2	1.63	0.81
28:a:1047:G:HO2'	28:a:1110:G:H1	1.21	0.80
20:N:19:LYS:C	20:N:21:PHE:H	1.88	0.80
22:P:39:PHE:HB2	22:P:74:LEU:HD13	1.62	0.79
34:g:104:ASN:ND2	34:g:114:ASP:OD2	2.16	0.79
16:J:8:ILE:HB	16:J:74:VAL:HG23	1.64	0.79
22:P:39:PHE:HB2	22:P:74:LEU:CD1	2.13	0.79
28:a:1043:C:O2	28:a:1112:G:N2	2.13	0.78
7:A:1397:C:OP2	11:E:29:ARG:NH2	2.17	0.78
7:A:392:C:H2'	7:A:393:A:H8	1.48	0.78
22:P:1:MET:O	22:P:2:VAL:C	2.26	0.78
9:C:36:ASP:OD1	9:C:59:ARG:NH2	2.17	0.77
22:P:4:ILE:HD12	22:P:65:ALA:HB3	1.65	0.77
28:a:2683:C:O2	37:j:70:ARG:NH2	2.17	0.77
7:A:999:C:N3	7:A:1042:A:N6	2.32	0.77
26:T:76:LYS:O	26:T:80:THR:OG1	2.02	0.77
15:I:57:MET:HE2	15:I:60:LYS:HG2	1.67	0.77
19:M:34:LEU:HD22	19:M:39:ILE:HD11	1.66	0.77
20:N:19:LYS:C	20:N:21:PHE:N	2.40	0.77
9:C:21:THR:HG23	9:C:58:GLU:HG2	1.67	0.77
23:Q:18:GLU:N	23:Q:18:GLU:OE2	2.18	0.76
41:n:39:VAL:HB	41:n:49:VAL:HG22	1.66	0.76
7:A:977:A:HO2'	7:A:981:U:H3	1.33	0.76
7:A:1118:U:OP1	15:I:106:ARG:NH1	2.18	0.76
7:A:564:C:OP1	18:L:12:ARG:NH1	2.18	0.76
7:A:736:C:OP1	24:R:61:ARG:NH1	2.18	0.76
15:I:112:GLU:HG2	15:I:121:ALA:HB1	1.67	0.76
22:P:19:VAL:HG13	22:P:37:GLY:C	2.09	0.76
7:A:428:G:OP2	10:D:10:LYS:NZ	2.17	0.76
7:A:1060:U:H5"	16:J:53:ILE:HG13	1.67	0.76
7:A:494:G:H2'	7:A:496:A:H8	1.48	0.76
13:G:144:MET:O	13:G:148:ASN:ND2	2.16	0.76

Continued on next page...

For Manuscript Review

Continued from previous page...

Atom-1	Atom-2	Interatomic distance (Å)	Clash overlap (Å)
36:i:128:ASN:O	36:i:128:ASN:ND2	2.15	0.76
7:A:1086:U:H3	7:A:1099:G:H22	1.33	0.76
22:P:23:ASP:O	22:P:25:ARG:N	2.19	0.76
22:P:60:TRP:HA	22:P:63:GLN:HB2	1.67	0.76
42:o:35:GLY:HA3	42:o:37:LYS:HZ3	1.50	0.76
20:N:34:VAL:O	20:N:36:ALA:N	2.19	0.76
28:a:1728:C:O2	28:a:1731:G:N1	2.19	0.75
7:A:80:A:H61	7:A:89:U:H3	1.31	0.75
9:C:134:MET:HE2	9:C:153:VAL:HB	1.66	0.75
28:a:2392:A:C2	38:k:55:MET:HE1	2.21	0.75
7:A:85:U:H4’	7:A:86:G:C2	2.22	0.75
5:4:16:CYS:SG	5:4:37:CYS:HB3	2.26	0.75
7:A:437:U:C6	7:A:437:U:H5”	2.21	0.75
9:C:135:LYS:O	9:C:139:GLN:HG3	1.87	0.75
13:G:130:ASN:O	13:G:130:ASN:ND2	2.20	0.75
7:A:1047:G:H5”	20:N:4:GLN:HE21	1.53	0.74
14:H:48:ASP:OD1	14:H:49:PHE:N	2.21	0.74
32:e:149:ILE:HD12	32:e:175:ILE:HG22	1.68	0.74
22:P:39:PHE:CG	22:P:74:LEU:CD1	2.70	0.74
28:a:1607:C:N4	28:a:1622:G:OP2	2.20	0.74
8:B:63:ARG:NE	8:B:63:ARG:O	2.20	0.73
48:u:11:GLU:N	48:u:11:GLU:OE2	2.21	0.73
48:u:63:ILE:HD12	48:u:72:VAL:HG21	1.70	0.73
20:N:17:ALA:O	20:N:19:LYS:N	2.21	0.73
22:P:75:ILE:O	22:P:79:ASN:HB2	1.88	0.73
36:i:125:TYR:HH	36:i:132:HIS:HE2	1.34	0.73
30:c:142:HIS:ND1	30:c:193:GLY:O	2.19	0.73
7:A:83:C:H4’	7:A:84:U:OP2	1.88	0.73
11:E:108:GLY:O	11:E:112:ARG:HG3	1.89	0.73
7:A:460:A:H2’	7:A:461:A:C8	2.24	0.73
7:A:673:A:H2’	7:A:674:G:C8	2.22	0.73
19:M:112:PRO:HD2	19:M:112:PRO:O	1.89	0.72
20:N:45:VAL:O	20:N:49:GLN:HG3	1.89	0.72
40:m:22:ARG:HG3	40:m:70:THR:HA	1.71	0.72
7:A:80:A:H2’	7:A:81:A:C8	2.25	0.72
7:A:1071:C:H2’	7:A:1072:G:H8	1.54	0.72
17:K:38:GLN:OE1	17:K:38:GLN:N	2.22	0.72
3:2:55:LEU:O	3:2:59:ILE:HD12	1.89	0.72
28:a:2250:G:OP1	39:l:84:LYS:NZ	2.22	0.72
8:B:11:LYS:O	8:B:208:ARG:NH2	2.22	0.72
25:S:59:PRO:HD2	25:S:59:PRO:O	1.87	0.72

Continued on next page...

For Manuscript Review

Continued from previous page...

Atom-1	Atom-2	Interatomic distance (Å)	Clash overlap (Å)
11:E:149:SER:H	11:E:152:MET:HE2	1.55	0.71
7:A:531:U:H5’	7:A:531:U:H6	1.53	0.71
7:A:1147:C:HO2’	15:I:7:TYR:HH	1.38	0.71
7:A:677:U:H3	7:A:713:G:H22	1.36	0.71
12:F:10:VAL:HG12	12:F:58:HIS:HB3	1.71	0.71
31:d:1:MET:HB3	31:d:205:PRO:HG2	1.71	0.71
32:e:51:GLU:OE1	32:e:88:ARG:NH2	2.23	0.71
48:u:21:ARG:NH1	48:u:87:GLN:O	2.23	0.71
13:G:113:ASP:HB2	13:G:119:ARG:HG2	1.72	0.71
28:a:2505:G:N2	28:a:2506:U:O4	2.23	0.71
34:g:9:VAL:HG21	34:g:50:LEU:HD12	1.72	0.71
41:n:2:ASP:OD1	41:n:5:SER:OG	2.08	0.71
7:A:1356:G:H2’	7:A:1357:A:C8	2.25	0.71
22:P:4:ILE:H	22:P:65:ALA:HB1	1.56	0.71
46:s:47:VAL:HG23	46:s:51:PHE:HD1	1.55	0.71
48:u:24:ASN:O	48:u:24:ASN:ND2	2.24	0.71
7:A:147:G:H2’	7:A:148:G:C8	2.26	0.71
30:c:107:PRO:HD2	30:c:110:LEU:HD22	1.73	0.71
7:A:1130:A:H61	7:A:1144:G:H1’	1.55	0.70
29:b:51:G:OP1	41:n:63:LYS:NZ	2.19	0.70
36:i:43:GLU:N	36:i:43:GLU:OE2	2.23	0.70
29:b:77:U:OP1	48:u:21:ARG:NH2	2.23	0.70
7:A:1279:G:O2’	7:A:1282:C:N4	2.24	0.70
23:Q:13:VAL:HG13	23:Q:22:VAL:HG23	1.72	0.70
9:C:153:VAL:HG22	9:C:198:VAL:HG22	1.74	0.70
35:h:7:ASP:OD1	35:h:8:LYS:N	2.23	0.70
31:d:183:GLU:OE2	31:d:183:GLU:N	2.20	0.70
5:4:9:TYR:OH	33:f:102:ARG:NH1	2.24	0.69
5:4:63:ARG:NH2	7:A:1312:G:OP2	2.24	0.69
20:N:26:GLU:O	20:N:29:ALA:HB3	1.92	0.69
7:A:87:C:H2’	7:A:88:U:H6	1.58	0.69
15:I:43:THR:O	15:I:47:VAL:HG23	1.92	0.69
24:R:21:ILE:HD13	24:R:54:GLN:HB3	1.73	0.69
11:E:64:MET:HA	11:E:64:MET:HE3	1.73	0.69
15:I:60:LYS:O	15:I:60:LYS:NZ	2.26	0.69
7:A:90:C:H2’	7:A:91:U:N3	2.07	0.69
10:D:121:LYS:O	10:D:146:ARG:NH1	2.25	0.69
26:T:39:ILE:HD13	26:T:82:GLN:HG2	1.75	0.69
28:a:1392:A:N6	46:s:18:GLU:OE2	2.24	0.69
7:A:413:G:H1’	7:A:428:G:N2	2.06	0.69
28:a:1779:U:OP2	28:a:1784:A:N6	2.24	0.69

Continued on next page...

For Manuscript Review

Continued from previous page...

Atom-1	Atom-2	Interatomic distance (Å)	Clash overlap (Å)
7:A:1301:U:O2’	7:A:1302:C:OP1	2.11	0.68
33:f:116:GLY:HA3	33:f:178:ARG:HB3	1.75	0.68
5:4:18:CYS:SG	5:4:40:CYS:HB3	2.33	0.68
7:A:831:A:H5’	8:B:21:ARG:HD3	1.75	0.68
22:P:38:PHE:O	22:P:50:THR:HA	1.92	0.68
10:D:44:ARG:HA	10:D:44:ARG:NH1	2.08	0.68
22:P:42:ILE:HG13	22:P:43:ALA:H	1.58	0.68
10:D:102:VAL:HG13	10:D:107:PHE:HB2	1.75	0.68
39:l:41:LEU:HG	39:l:96:ILE:CD1	2.24	0.68
7:A:1147:C:O2	15:I:18:ARG:NH1	2.26	0.68
13:G:131:LYS:O	13:G:136:LYS:NZ	2.25	0.68
17:K:111:THR:HG23	27:U:3:VAL:HG12	1.76	0.68
18:L:99:ARG:NH1	18:L:105:SER:O	2.27	0.68
20:N:8:ALA:O	20:N:11:VAL:HG12	1.94	0.68
21:O:26:GLU:OE2	21:O:26:GLU:N	2.20	0.68
15:I:39:PHE:O	15:I:45:ARG:NH2	2.26	0.68
33:f:52:ASN:HB3	33:f:150:ARG:HH12	1.59	0.68
8:B:114:LEU:HG	8:B:144:LEU:HB3	1.74	0.67
29:b:1:U:H2’	29:b:2:G:H8	1.58	0.67
7:A:1152:A:OP1	16:J:70:HIS:ND1	2.26	0.67
8:B:66:LYS:HB3	8:B:90:PHE:HE1	1.59	0.67
13:G:101:MET:O	13:G:105:VAL:HG23	1.94	0.67
20:N:21:PHE:O	20:N:22:ALA:C	2.37	0.67
34:g:33:LEU:HB3	34:g:75:MET:HE2	1.74	0.67
48:u:55:GLU:OE2	48:u:55:GLU:N	2.18	0.67
7:A:1318:A:OP1	25:S:3:ARG:NH2	2.27	0.67
10:D:23:SER:HB3	10:D:110:THR:HB	1.76	0.67
20:N:33:ASP:O	20:N:34:VAL:HB	1.93	0.67
22:P:2:VAL:HG21	22:P:60:TRP:HE3	1.60	0.67
22:P:47:GLU:HG3	22:P:48:GLU:N	2.07	0.67
21:O:89:ARG:NH2	28:a:714:U:OP2	2.28	0.67
5:4:14:ALA:HB1	5:4:34:LEU:HD11	1.77	0.67
7:A:502:A:C2	7:A:544:G:C2	2.82	0.67
8:B:130:THR:OG1	8:B:133:GLU:OE2	2.13	0.67
20:N:19:LYS:O	20:N:21:PHE:N	2.28	0.67
22:P:8:ARG:HB3	22:P:28:ARG:NH1	2.10	0.67
28:a:219:A:N3	28:a:234:U:O2’	2.28	0.67
28:a:463:G:N2	28:a:466:A:OP2	2.25	0.67
7:A:104:G:OP1	26:T:16:LYS:NZ	2.29	0.66
7:A:511:C:O2’	7:A:512:U:O5’	2.13	0.66
7:A:1249:C:O2’	15:I:75:GLN:NE2	2.27	0.66

Continued on next page...

For Manuscript Review

Continued from previous page...

Atom-1	Atom-2	Interatomic distance (Å)	Clash overlap (Å)
28:a:2304:G:H22	28:a:2312:U:H3	1.43	0.66
31:d:184:ARG:NH1	42:o:7:GLN:HE22	1.93	0.66
7:A:269:C:H2’	7:A:270:A:H8	1.60	0.66
26:T:26:SER:O	26:T:30:THR:OG1	2.13	0.66
38:k:108:ALA:HB3	38:k:125:LEU:HD22	1.76	0.66
8:B:58:ASN:ND2	8:B:223:GLU:OE2	2.27	0.66
20:N:32:SER:O	20:N:34:VAL:N	2.29	0.66
5:4:13:THR:OG1	5:4:31:ASP:OD2	2.14	0.66
7:A:744:C:H2’	7:A:745:G:H8	1.60	0.66
7:A:202:G:O2’	7:A:468:A:N3	2.28	0.66
7:A:297:G:N2	7:A:300:A:OP2	2.29	0.66
10:D:11:LEU:HD13	10:D:63:ARG:HG2	1.78	0.66
28:a:2484:G:OP1	39:l:44:ARG:NH1	2.28	0.66
7:A:1323:G:H2’	7:A:1324:A:C8	2.31	0.66
22:P:8:ARG:HB3	22:P:28:ARG:HH11	1.61	0.66
34:g:38:ASN:ND2	34:g:64:GLN:OE1	2.26	0.66
7:A:960:U:H4’	7:A:961:U:OP2	1.95	0.66
7:A:1323:G:H2’	7:A:1324:A:H8	1.61	0.66
20:N:30:ILE:O	20:N:33:ASP:HB3	1.96	0.66
16:J:18:ILE:O	16:J:22:THR:HG23	1.95	0.65
22:P:44:SER:HB3	22:P:47:GLU:HB3	1.77	0.65
7:A:1003:G:N2	7:A:1004:A:O2’	2.29	0.65
15:I:28:ILE:HG13	15:I:63:LEU:HB2	1.79	0.65
23:Q:46:VAL:HG21	23:Q:61:ILE:HD13	1.79	0.65
25:S:49:ILE:HG21	25:S:71:LEU:HD21	1.79	0.65
34:g:60:ASP:OD2	34:g:61:GLY:N	2.25	0.65
46:s:5:GLU:N	46:s:5:GLU:OE1	2.30	0.65
47:t:86:ARG:NH1	47:t:88:GLU:OE1	2.28	0.65
7:A:714:G:H2’	7:A:715:A:C8	2.31	0.65
7:A:1124:G:OP1	16:J:38:GLY:N	2.29	0.65
28:a:1800:C:OP2	30:c:182:ARG:NH2	2.30	0.65
46:s:25:GLU:HG3	46:s:26:LYS:HD3	1.79	0.65
15:I:79:ILE:O	15:I:83:ILE:HG13	1.97	0.65
26:T:17:ALA:O	26:T:21:ASN:ND2	2.27	0.65
28:a:788:A:OP1	28:a:791:C:N4	2.27	0.65
7:A:1359:C:OP2	20:N:75:ARG:NH1	2.30	0.65
9:C:129:MET:HB2	9:C:132:ARG:HG3	1.78	0.65
4:3:2:LYS:NZ	4:3:32:LYS:O	2.27	0.65
7:A:87:C:H2’	7:A:88:U:C6	2.32	0.65
7:A:1348:U:H4’	15:I:122:ARG:HG3	1.77	0.65
13:G:5:ARG:NH2	13:G:7:ILE:O	2.30	0.65

Continued on next page...

For Manuscript Review

Continued from previous page...

Atom-1	Atom-2	Interatomic distance (Å)	Clash overlap (Å)
20:N:20:TYR:HD1	20:N:51:LEU:HD22	1.62	0.65
17:K:34:ILE:HD12	17:K:74:VAL:HG21	1.79	0.64
20:N:20:TYR:HB3	20:N:51:LEU:HD13	1.78	0.64
28:a:1115:G:O2'	28:a:1116:G:O5'	2.14	0.64
28:a:2092:U:OP2	35:h:27:ARG:NH1	2.30	0.64
7:A:451:A:N6	7:A:480:U:O2'	2.26	0.64
7:A:460:A:H2'	7:A:461:A:H8	1.62	0.64
9:C:129:MET:HA	9:C:129:MET:HE3	1.78	0.64
28:a:529:A:OP2	36:i:116:ARG:NH2	2.30	0.64
33:f:4:LEU:HD13	33:f:101:GLU:HG2	1.79	0.64
28:a:2469:A:N6	28:a:2481:G:O2'	2.30	0.64
7:A:1279:G:OP2	16:J:11:LYS:NZ	2.30	0.64
7:A:86:G:H4'	7:A:87:C:O5'	1.98	0.64
45:r:72:THR:HG22	45:r:73:LYS:HG3	1.77	0.64
7:A:363:A:N6	18:L:27:CYS:SG	2.69	0.64
7:A:421:U:H3	9:C:127:ARG:HH12	1.44	0.64
7:A:674:G:H2'	7:A:675:A:H8	1.63	0.64
28:a:1419:A:O2'	28:a:1421:G:N7	2.23	0.64
36:i:49:ASP:OD1	36:i:121:LYS:NZ	2.31	0.64
7:A:77:A:H2'	7:A:78:A:H5"	1.79	0.64
7:A:127:G:N2	23:Q:63:GLU:OE2	2.29	0.64
20:N:32:SER:O	20:N:33:ASP:C	2.41	0.64
51:x:2:LYS:HG2	51:x:6:LEU:HD13	1.79	0.64
5:4:16:CYS:HA	5:4:34:LEU:HB2	1.79	0.64
15:I:72:ILE:H	15:I:72:ILE:HD12	1.61	0.64
20:N:27:LEU:CD2	20:N:47:LYS:HB3	2.26	0.64
37:j:18[B]:ARG:NH1	37:j:45:GLU:OE1	2.30	0.64
28:a:568:U:H1'	28:a:2030:6MZ:H9C1	1.79	0.64
13:G:137:LYS:HB3	13:G:137:LYS:NZ	2.13	0.63
52:y:8:THR:HG23	52:y:35:THR:HG22	1.79	0.63
7:A:713:G:H2'	7:A:714:G:C8	2.34	0.63
7:A:1191:A:OP2	9:C:2:GLY:N	2.32	0.63
19:M:79:ARG:NH2	25:S:65:GLU:O	2.31	0.63
19:M:90:ARG:HB2	19:M:97:VAL:HG12	1.80	0.63
51:x:13:GLU:OE1	51:x:13:GLU:N	2.18	0.63
7:A:400:C:OP1	10:D:70:ARG:NH2	2.30	0.63
7:A:1266:G:N2	7:A:1269:A:OP2	2.23	0.63
7:A:5:U:H5'	7:A:6:G:C4	2.34	0.63
7:A:150:U:H3	7:A:171:A:H62	1.47	0.63
19:M:97:VAL:HG23	19:M:98:ARG:HG2	1.80	0.63
40:m:86:ARG:NE	40:m:117:ASP:OD1	2.31	0.63

Continued on next page...

For Manuscript Review

Continued from previous page...

Atom-1	Atom-2	Interatomic distance (Å)	Clash overlap (Å)
47:t:10:GLU:OE1	47:t:22:ARG:NH1	2.31	0.63
7:A:1025:U:H4'	7:A:1026:G:H8	1.63	0.63
28:a:2849:U:HO2'	56:a:6209:SPD:HN11	1.46	0.63
7:A:82:G:N7	7:A:83:C:H1'	2.13	0.63
28:a:639:U:H2'	28:a:640:C:C6	2.33	0.63
28:a:1433:A:H2'	28:a:1434:A:C8	2.34	0.63
28:a:1667:G:O2'	28:a:1991:U:O4	2.17	0.63
52:y:7:ILE:HD11	52:y:48:ILE:HD11	1.80	0.63
7:A:398:U:H2'	7:A:399:G:H8	1.63	0.63
7:A:1071:C:H2'	7:A:1072:G:C8	2.33	0.63
9:C:16:LYS:NZ	9:C:181:ASP:OD1	2.31	0.63
18:L:36:ARG:HH11	18:L:36:ARG:CB	2.11	0.63
34:g:173:GLU:N	34:g:173:GLU:OE2	2.29	0.63
7:A:416:G:H2'	7:A:417:G:H8	1.63	0.63
28:a:411:G:OP2	28:a:2406:A:O2'	2.17	0.63
28:a:721:A:H2'	28:a:722:A:H8	1.64	0.63
28:a:2343:U:HO2'	28:a:2373:G:HO2'	1.42	0.63
39:l:53:MET:HG2	39:l:63:ILE:HD12	1.80	0.63
8:B:67:ILE:HG12	8:B:89:GLN:HE21	1.64	0.63
28:a:2305:U:H5"	33:f:131:GLY:HA3	1.79	0.63
34:g:19:ILE:HG12	34:g:24:ILE:HD12	1.81	0.63
19:M:11:ASP:OD1	19:M:12:HIS:N	2.32	0.62
28:a:2291:U:H2'	28:a:2292:U:C6	2.33	0.62
7:A:299:G:H2'	7:A:300:A:C8	2.34	0.62
7:A:657:U:O4'	21:O:28:GLN:NE2	2.32	0.62
9:C:64:ILE:HG22	9:C:99:ALA:HA	1.81	0.62
19:M:72:GLU:OE1	19:M:73:ILE:HG23	1.98	0.62
25:S:50:ALA:HB1	25:S:57:HIS:HB3	1.80	0.62
28:a:2328:A:H2'	28:a:2329:U:C6	2.34	0.62
28:a:2547:A:H2'	28:a:2548:U:C6	2.34	0.62
22:P:28:ARG:HD2	22:P:29:ASN:OD1	1.99	0.62
7:A:81:A:H8	7:A:81:A:O5'	1.82	0.62
7:A:375:U:OP1	22:P:70:ARG:NH1	2.33	0.62
7:A:421:U:H3	9:C:127:ARG:NH1	1.97	0.62
7:A:479:U:O4	56:A:1690:SPD:N6	2.32	0.62
15:I:12:ARG:O	15:I:15:SER:OG	2.17	0.62
15:I:113:ARG:HG3	15:I:115:LYS:HE3	1.81	0.62
22:P:39:PHE:CE1	22:P:74:LEU:HD21	2.34	0.62
29:b:76:G:OP1	48:u:9:ARG:NH2	2.31	0.62
7:A:269:C:H2'	7:A:270:A:C8	2.34	0.62
7:A:663:A:H61	7:A:743:A:H61	1.47	0.62

Continued on next page...

For Manuscript Review

Continued from previous page...

Atom-1	Atom-2	Interatomic distance (Å)	Clash overlap (Å)
22:P:2:VAL:HG21	22:P:60:TRP:CE3	2.35	0.62
28:a:820:A:H4'	28:a:836:G:H22	1.65	0.62
1:0:33:LYS:HE2	1:0:51:GLU:HG2	1.81	0.62
7:A:189:A:H2'	7:A:190:A:C8	2.34	0.62
28:a:1012:U:OP2	43:p:70:ARG:NH2	2.32	0.62
7:A:392:C:H2'	7:A:393:A:C8	2.34	0.62
22:P:22:ALA:HA	22:P:33:ILE:HG13	1.82	0.62
40:m:56:LYS:NZ	40:m:94:TYR:OH	2.32	0.62
7:A:1027:C:H2'	7:A:1028:C:C6	2.35	0.62
13:G:38:THR:O	13:G:42:ILE:HD12	2.00	0.62
23:Q:68:SER:OG	23:Q:69:LYS:N	2.30	0.62
47:t:48:PRO:HD3	47:t:56:GLY:HA3	1.82	0.62
20:N:34:VAL:HG12	20:N:35:ASN:N	2.15	0.62
28:a:1434:A:H2'	28:a:1435:G:H8	1.65	0.62
28:a:2851:A:N7	56:a:6209:SPD:H92	2.15	0.62
7:A:78:A:H61	7:A:91:U:H3	1.47	0.61
7:A:513:C:H2'	7:A:514:C:H6	1.65	0.61
15:I:114:LYS:NZ	15:I:118:LEU:O	2.31	0.61
28:a:1715:G:O2'	28:a:1743:G:O6	2.18	0.61
46:s:39:THR:O	46:s:43:ILE:HD12	2.00	0.61
7:A:28:A:O2'	7:A:296:U:OP1	2.16	0.61
7:A:1004:A:C2	7:A:1005:A:H1'	2.35	0.61
41:n:27:VAL:HA	41:n:93:ASP:HB3	1.80	0.61
7:A:796:C:O3'	17:K:127:ARG:NH1	2.33	0.61
7:A:1505:G:O2'	7:A:1506:U:OP2	2.19	0.61
33:f:34:ILE:HB	33:f:91:LEU:HB2	1.81	0.61
28:a:630:G:N2	28:a:633:A:OP2	2.31	0.61
33:f:156:ILE:HG21	33:f:170:LEU:HD21	1.81	0.61
36:i:128:ASN:HD22	36:i:128:ASN:C	2.04	0.61
12:F:19:PRO:O	12:F:23:GLU:HG2	2.00	0.61
19:M:24:GLY:O	19:M:29:ARG:NH1	2.33	0.61
7:A:80:A:N6	7:A:89:U:H3	1.96	0.61
7:A:1297:G:N2	7:A:1298:U:O4	2.32	0.61
25:S:12:ASP:OD2	25:S:35:SER:OG	2.12	0.61
28:a:1980:G:O2'	28:a:1982:U:OP2	2.19	0.61
20:N:15:ALA:O	20:N:16:LEU:C	2.41	0.61
21:O:8:THR:O	21:O:12:VAL:HG13	2.00	0.61
28:a:505:A:OP2	56:a:6211:SPD:N1	2.33	0.61
31:d:14:ILE:HD11	31:d:188:LEU:HD12	1.82	0.61
7:A:39:G:N1	7:A:403:C:H5	1.89	0.61
34:g:38:ASN:OD1	34:g:39:ASP:N	2.34	0.61

Continued on next page...

For Manuscript Review

Continued from previous page...

Atom-1	Atom-2	Interatomic distance (Å)	Clash overlap (Å)
50:w:72:ARG:NH1	50:w:78:TYR:OH	2.33	0.61
15:I:57:MET:HA	15:I:57:MET:HE3	1.83	0.61
23:Q:48:ASP:OD2	23:Q:51:ASN:HA	2.01	0.61
24:R:73:ARG:HH11	24:R:73:ARG:HG3	1.66	0.61
34:g:2:SER:OG	34:g:3:ARG:N	2.18	0.61
7:A:391:G:O2'	7:A:392:C:O5'	2.17	0.60
7:A:451:A:O5'	22:P:70:ARG:NH2	2.29	0.60
22:P:4:ILE:N	22:P:65:ALA:HB1	2.15	0.60
22:P:48:GLU:O	22:P:48:GLU:HG3	2.00	0.60
7:A:513:C:H2'	7:A:514:C:C6	2.36	0.60
22:P:56:ARG:HA	22:P:59:HIS:HB3	1.82	0.60
2:1:12:ARG:HH11	2:1:12:ARG:HG3	1.66	0.60
7:A:76:G:H2'	7:A:77:A:H5'	1.82	0.60
8:B:9:MET:HE2	8:B:14:VAL:HG21	1.82	0.60
13:G:100:ALA:O	13:G:104:ILE:HG13	2.01	0.60
46:s:43:ILE:O	46:s:47:VAL:HG12	2.01	0.60
8:B:15:HIS:HB3	8:B:43:LEU:HD21	1.81	0.60
16:J:37:ARG:HB2	16:J:75:ASP:HB2	1.83	0.60
7:A:201:G:HO2'	7:A:469:C:HO2'	1.50	0.60
7:A:391:G:HO2'	7:A:392:C:P	2.25	0.60
7:A:1290:G:OP1	13:G:35:LYS:NZ	2.35	0.60
24:R:47:THR:HG22	24:R:48:ARG:N	2.16	0.60
28:a:1141:U:OP2	36:i:65:THR:OG1	2.19	0.60
12:F:22:ILE:HD13	12:F:39:LEU:HD11	1.83	0.60
13:G:137:LYS:HB3	13:G:137:LYS:HZ2	1.67	0.60
20:N:19:LYS:O	20:N:20:TYR:C	2.40	0.60
28:a:569:U:O2'	28:a:983:A:N1	2.32	0.60
4:3:16:ILE:HG12	4:3:25:VAL:HG22	1.83	0.60
7:A:410:G:H2'	7:A:429:U:C5	2.37	0.60
15:I:46:MET:N	15:I:46:MET:SD	2.74	0.60
5:4:59:ARG:HA	5:4:62:LYS:HE3	1.83	0.60
7:A:90:C:H2'	7:A:91:U:H3	1.67	0.60
7:A:1230:C:H2'	7:A:1231:G:H8	1.65	0.60
9:C:136:ARG:HD3	9:C:140:ASN:HD21	1.65	0.60
7:A:693:G:OP1	17:K:127:ARG:NH2	2.35	0.60
22:P:39:PHE:CB	22:P:74:LEU:CD1	2.79	0.60
20:N:30:ILE:O	20:N:31:ILE:C	2.45	0.59
28:a:729:G:H5"	28:a:730:A:H5"	1.83	0.59
36:i:31:GLU:OE2	36:i:34:ARG:NH1	2.35	0.59
7:A:451:A:O2'	56:A:1690:SPD:N1	2.35	0.59
16:J:7:ARG:HD2	16:J:73:LEU:HD11	1.84	0.59

Continued on next page...

For Manuscript Review

Continued from previous page...

Atom-1	Atom-2	Interatomic distance (Å)	Clash overlap (Å)
16:J:8:ILE:HD13	16:J:100:ILE:HG12	1.82	0.59
20:N:28:LYS:HA	20:N:31:ILE:HG13	1.82	0.59
28:a:549:G:H2’	28:a:550:C:C6	2.37	0.59
28:a:1528:A:OP2	28:a:1543:G:N2	2.35	0.59
7:A:1026:G:O2’	7:A:1027:C:OP1	2.19	0.59
10:D:44:ARG:HA	10:D:44:ARG:HH11	1.66	0.59
22:P:36:VAL:CG1	22:P:53:ASP:HB3	2.32	0.59
52:y:41:THR:HG22	52:y:43:ALA:H	1.67	0.59
7:A:85:U:O2’	7:A:86:G:H5”	2.03	0.59
7:A:1162:C:H2’	7:A:1163:A:H8	1.67	0.59
11:E:100:SER:O	11:E:103:THR:OG1	2.20	0.59
19:M:67:GLY:O	19:M:71:ARG:HG3	2.03	0.59
28:a:301:G:OP2	47:t:82:ARG:NH2	2.33	0.59
28:a:1417:C:HO2’	28:a:1587:G:HO2’	1.51	0.59
3:2:12:LYS:NZ	28:a:249:C:O2	2.33	0.59
7:A:362:G:N2	7:A:364:A:O2’	2.36	0.59
15:I:45:ARG:HD3	15:I:49:ARG:HH22	1.68	0.59
21:O:47:LYS:O	21:O:53:ARG:NH2	2.36	0.59
22:P:2:VAL:O	22:P:65:ALA:HA	2.03	0.59
7:A:406:G:O6	7:A:495:A:H2’	2.02	0.59
37:j:106:GLU:N	37:j:106:GLU:OE1	2.35	0.59
45:r:74:ILE:HD12	45:r:105:VAL:HG22	1.83	0.59
7:A:1363:A:O2’	7:A:1365:G:N7	2.27	0.59
10:D:28:ILE:CG2	10:D:34:ILE:HD13	2.31	0.59
36:i:125:TYR:OH	36:i:132:HIS:NE2	2.24	0.59
28:a:1045:C:O2	28:a:1111:A:N6	2.34	0.59
31:d:46:ARG:NH2	31:d:85:ALA:O	2.36	0.59
7:A:113:G:H1’	7:A:354:G:H5’	1.85	0.59
7:A:1301:U:H3’	7:A:1302:C:H5”	1.84	0.59
7:A:1305:G:N2	7:A:1331:G:O2’	2.32	0.59
11:E:25:VAL:HG12	11:E:26:LYS:H	1.68	0.59
22:P:9:HIS:O	22:P:16:PHE:HB3	2.03	0.59
28:a:721:A:H2’	28:a:722:A:C8	2.38	0.59
3:2:54:ASP:HB3	38:k:57:LEU:HD22	1.84	0.59
22:P:36:VAL:HG12	22:P:53:ASP:HB3	1.85	0.59
28:a:1430:G:H5”	57:a:6221:SPM:H21	1.83	0.59
28:a:1548:A:H2’	28:a:1549:A:C8	2.37	0.59
45:r:22:ASP:OD2	45:r:25:ARG:NH1	2.27	0.59
22:P:61:VAL:C	22:P:63:GLN:N	2.59	0.58
7:A:741:G:C4	7:A:742:G:H8	2.21	0.58
7:A:746:A:H2’	7:A:747:A:C8	2.38	0.58

Continued on next page...

For Manuscript Review

Continued from previous page...

Atom-1	Atom-2	Interatomic distance (Å)	Clash overlap (Å)
8:B:213:TYR:O	8:B:217:VAL:HG23	2.03	0.58
20:N:20:TYR:CD1	20:N:51:LEU:HD22	2.38	0.58
20:N:21:PHE:CD2	20:N:22:ALA:N	2.71	0.58
22:P:45:GLU:CD	22:P:45:GLU:H	2.11	0.58
37:j:70:ARG:NH1	37:j:74:GLY:O	2.36	0.58
1:0:44:ARG:NH1	1:0:44:ARG:HG3	2.17	0.58
7:A:339:C:OP2	37:j:98:ARG:NH1	2.36	0.58
23:Q:12:VAL:HG12	23:Q:55:ILE:HA	1.85	0.58
31:d:181:ASP:OD2	31:d:184:ARG:HD2	2.03	0.58
7:A:635:A:O2'	23:Q:6:ARG:NH1	2.37	0.58
48:u:11:GLU:HG2	48:u:16:ALA:HB1	1.86	0.58
51:x:9:LYS:HB3	51:x:13:GLU:HG2	1.85	0.58
17:K:17:SER:HA	17:K:79:ILE:HA	1.86	0.58
20:N:32:SER:O	20:N:34:VAL:HG23	2.04	0.58
37:j:34:GLY:N	37:j:37:ASP:OD2	2.37	0.58
7:A:398:U:H2'	7:A:399:G:C8	2.38	0.58
7:A:993:G:O2'	7:A:994:A:N7	2.37	0.58
7:A:1167:A:O2'	7:A:1169:A:N7	2.37	0.58
12:F:20:GLY:O	12:F:24:ARG:HG3	2.03	0.58
19:M:40:ALA:HB3	19:M:43:VAL:HG23	1.86	0.58
22:P:39:PHE:CZ	22:P:74:LEU:HD21	2.39	0.58
22:P:60:TRP:CA	22:P:63:GLN:HB2	2.33	0.58
7:A:1130:A:H2'	7:A:1131:G:H8	1.69	0.58
28:a:2646:C:OP2	28:a:2732:G:O2'	2.21	0.58
33:f:38:MET:HE2	33:f:57:LEU:HD13	1.86	0.58
48:u:46:LYS:O	48:u:50:MET:HG3	2.03	0.58
5:4:35:ASP:OD2	19:M:3:ARG:NH1	2.37	0.58
7:A:216:U:H2'	7:A:217:C:C6	2.39	0.58
7:A:1355:G:H2'	7:A:1356:G:C8	2.38	0.58
28:a:272:A:H2'	28:a:273:G:H8	1.68	0.58
7:A:71:A:N1	7:A:99:C:O2'	2.35	0.58
7:A:461:A:H2'	7:A:462:G:H8	1.69	0.58
7:A:477:C:H2'	7:A:478:A:C8	2.37	0.58
7:A:811:C:O2'	7:A:901:A:N1	2.36	0.58
7:A:1122:U:O2'	7:A:1123:U:O5'	2.21	0.58
28:a:2204:G:OP2	30:c:147:LYS:NZ	2.35	0.58
41:n:28:VAL:HG21	41:n:103:VAL:HG13	1.85	0.58
47:t:7:ARG:NH1	47:t:26:LYS:O	2.37	0.58
18:L:87:VAL:HG22	18:L:96:HIS:HE2	1.69	0.57
20:N:31:ILE:O	20:N:41:ARG:HD3	2.04	0.57
22:P:44:SER:CB	22:P:47:GLU:HB3	2.33	0.57

Continued on next page...

For Manuscript Review

Continued from previous page...

Atom-1	Atom-2	Interatomic distance (Å)	Clash overlap (Å)
28:a:2304:G:O4'	33:f:129:SER:OG	2.22	0.57
5:4:9:TYR:CE2	5:4:25:ARG:HG3	2.39	0.57
16:J:66:GLU:HB3	20:N:99:ALA:HB2	1.85	0.57
17:K:34:ILE:HG13	17:K:70:CYS:SG	2.44	0.57
24:R:45:THR:HG1	24:R:47:THR:HG1	1.52	0.57
28:a:748:G:OP2	56:a:6217:SPD:N10	2.36	0.57
28:a:848:C:H2'	28:a:849:A:H8	1.68	0.57
43:p:90:ILE:HG22	43:p:95:LEU:HG	1.86	0.57
7:A:935:A:O2'	7:A:1383:C:O2	2.21	0.57
8:B:225:ARG:HB2	8:B:225:ARG:HH11	1.69	0.57
22:P:4:ILE:HD12	22:P:65:ALA:CB	2.34	0.57
28:a:781:A:OP1	30:c:217:ARG:NH2	2.37	0.57
36:i:114:LEU:O	36:i:118:MET:HG3	2.04	0.57
7:A:517:G:N2	7:A:533:A:OP2	2.33	0.57
10:D:124:MET:HG2	10:D:146:ARG:HG2	1.85	0.57
11:E:81:LEU:HD11	11:E:96:MET:HB3	1.86	0.57
12:F:41:ASP:OD1	12:F:58:HIS:NE2	2.36	0.57
20:N:30:ILE:HG22	20:N:31:ILE:N	2.18	0.57
28:a:807:U:OP2	38:k:41:ARG:NH2	2.37	0.57
28:a:1434:A:H2'	28:a:1435:G:C8	2.39	0.57
7:A:384:G:H2'	7:A:385:C:C6	2.39	0.57
7:A:1016:A:O2'	7:A:1217:C:O2'	2.21	0.57
7:A:1356:G:H2'	7:A:1357:A:H8	1.67	0.57
17:K:20:VAL:HG13	17:K:83:GLU:HB2	1.85	0.57
28:a:299:A:N3	28:a:319:G:O2'	2.35	0.57
40:m:103:ARG:HD3	40:m:110:MET:HE3	1.85	0.57
7:A:374:A:H5"	7:A:451:A:H8	1.69	0.57
11:E:107:ALA:HB1	11:E:111:MET:HB2	1.85	0.57
14:H:38:ASN:O	14:H:42:GLU:HG2	2.05	0.57
22:P:21:VAL:O	22:P:22:ALA:HB2	2.04	0.57
22:P:60:TRP:O	22:P:64:GLY:N	2.33	0.57
28:a:1469:A:H2'	28:a:1470:A:C8	2.40	0.57
28:a:2016:U:O2	53:z:4:GLN:NE2	2.38	0.57
1:0:22:THR:HG21	28:a:2419:U:H4'	1.85	0.57
7:A:430:A:P	10:D:22:LYS:NZ	2.78	0.57
7:A:437:U:H4'	10:D:152:GLN:NE2	2.18	0.57
7:A:826:C:O2	14:H:16:ASN:ND2	2.38	0.57
8:B:62:SER:OG	8:B:227:GLN:OE1	2.23	0.57
8:B:129:LEU:HB3	8:B:133:GLU:HG3	1.86	0.57
7:A:324:G:N1	7:A:327:A:OP2	2.38	0.57
11:E:61:GLN:O	11:E:65:GLU:HG2	2.05	0.57

Continued on next page...

For Manuscript Review

For Manuscript Review

Continued from previous page...

Atom-1	Atom-2	Interatomic distance (Å)	Clash overlap (Å)
53:z:38:HIS:ND1	53:z:39:LEU:O	2.37	0.57
7:A:363:A:OP2	18:L:31:ARG:NH1	2.37	0.57
7:A:451:A:H61	7:A:480:U:HO2'	1.50	0.57
9:C:87:LEU:O	9:C:91:VAL:HG12	2.05	0.57
16:J:92:LEU:HG	16:J:98:VAL:HG11	1.87	0.57
28:a:473:G:OP2	56:a:6216:SPD:N10	2.36	0.57
28:a:549:G:H2'	28:a:550:C:H6	1.69	0.57
28:a:2850:A:OP2	56:a:6209:SPD:N10	2.37	0.57
40:m:24:MET:HG2	40:m:44:LEU:HD22	1.86	0.57
41:n:76:LYS:O	41:n:80:GLU:HG3	2.05	0.57
7:A:413:G:OP1	7:A:413:G:C8	2.58	0.57
7:A:743:A:H2'	7:A:743:A:N3	2.20	0.57
11:E:113:ALA:O	11:E:117:VAL:HG22	2.04	0.57
20:N:37:SER:HB2	20:N:40:ASP:CG	2.29	0.57
34:g:127:THR:OG1	34:g:128:GLN:N	2.38	0.57
7:A:1271:A:H2'	7:A:1272:G:H8	1.69	0.56
21:O:71:LYS:HD2	21:O:78:TYR:CE1	2.40	0.56
26:T:22:ALA:O	26:T:26:SER:OG	2.23	0.56
28:a:5:A:H2'	28:a:6:A:H8	1.69	0.56
28:a:593:U:H2'	28:a:594:U:C6	2.41	0.56
31:d:174:SER:O	31:d:174:SER:OG	2.21	0.56
1:0:10:LYS:HG3	1:0:54:ILE:HD12	1.86	0.56
1:0:23:THR:OG1	1:0:24:THR:N	2.38	0.56
7:A:1005:A:H3'	7:A:1006:G:H8	1.69	0.56
7:A:1013:G:N2	7:A:1016:A:OP2	2.30	0.56
10:D:177:LYS:HB2	10:D:179:GLU:HG2	1.85	0.56
28:a:5:A:H2'	28:a:6:A:C8	2.40	0.56
28:a:742:A:H2'	28:a:743:A:C8	2.41	0.56
28:a:856:G:H2'	28:a:857:G:C8	2.40	0.56
28:a:2327:A:H2'	28:a:2328:A:C8	2.41	0.56
28:a:2627:G:N2	28:a:2777:G:OP2	2.38	0.56
7:A:82:G:C8	7:A:83:C:H1'	2.41	0.56
7:A:490:C:H2'	7:A:491:G:H8	1.70	0.56
7:A:663:A:H61	7:A:743:A:N6	2.04	0.56
7:A:1169:A:H2'	7:A:1170:A:C8	2.40	0.56
7:A:1317:C:C4	20:N:53:ARG:HG2	2.40	0.56
22:P:72:ALA:HA	22:P:75:ILE:HG22	1.87	0.56
26:T:43:ASP:OD1	26:T:46:ALA:N	2.38	0.56
27:U:14:VAL:HA	27:U:17:ARG:HE	1.70	0.56
33:f:17:MET:HE1	33:f:22:TYR:HB2	1.86	0.56
44:q:31:GLU:N	44:q:31:GLU:OE2	2.39	0.56

Continued on next page...

Continued from previous page...

Atom-1	Atom-2	Interatomic distance (Å)	Clash overlap (Å)
7:A:1492:A:H2’	7:A:1493:A:C4	2.40	0.56
8:B:104:TRP:HA	8:B:107:VAL:HG22	1.88	0.56
9:C:50:ALA:O	9:C:70:THR:OG1	2.24	0.56
10:D:62:ARG:NH1	10:D:69:GLU:OE2	2.38	0.56
28:a:672:C:OP2	38:k:42:SER:OG	2.22	0.56
32:e:168:ASP:OD2	32:e:170:ARG:NH1	2.38	0.56
7:A:451:A:P	22:P:70:ARG:HH22	2.29	0.56
9:C:105:GLU:HA	9:C:105:GLU:OE2	2.06	0.56
11:E:47:GLY:HA3	11:E:71:MET:HA	1.88	0.56
16:J:6:ILE:HB	16:J:76:ILE:HG22	1.87	0.56
19:M:70:ARG:O	19:M:73:ILE:HD12	2.06	0.56
26:T:82:GLN:HA	26:T:85:LYS:NZ	2.20	0.56
28:a:483:A:C8	47:t:45:HIS:HD2	2.24	0.56
28:a:727:A:OP2	56:a:6215:SPD:N6	2.34	0.56
28:a:1819:A:H5”	30:c:160:THR:HG21	1.86	0.56
5:4:66:ILE:HA	20:N:38:ASP:OD1	2.05	0.56
7:A:96:U:H2’	7:A:97:G:C8	2.40	0.56
7:A:559:A:H4’	7:A:560:A:H3’	1.87	0.56
10:D:122:ALA:C	10:D:123:ILE:HD13	2.30	0.56
28:a:2850:A:N7	28:a:2868:A:O2’	2.33	0.56
30:c:21:ASN:HB3	30:c:24:LEU:HG	1.88	0.56
48:u:68:LYS:HE2	48:u:68:LYS:HA	1.85	0.56
51:x:4:LYS:H	51:x:4:LYS:HD2	1.69	0.56
19:M:51:GLY:O	19:M:55:THR:HG23	2.05	0.56
22:P:34:GLU:HG2	22:P:36:VAL:HG22	1.88	0.56
34:g:24:ILE:HD11	34:g:43:VAL:HG21	1.88	0.56
7:A:1000:A:H2’	7:A:1001:C:C6	2.40	0.56
8:B:63:ARG:O	8:B:63:ARG:CZ	2.54	0.56
10:D:118:VAL:HA	10:D:123:ILE:HG12	1.88	0.56
51:x:14:LEU:HD23	51:x:57:LEU:HD23	1.88	0.56
7:A:963:G:C2	7:A:964:A:C8	2.94	0.56
14:H:11:LEU:HD22	14:H:75:ILE:HD11	1.88	0.56
35:h:25:TYR:HE1	35:h:29:PHE:HD2	1.53	0.56
7:A:79:G:H2’	7:A:80:A:C1’	2.36	0.55
9:C:57:ILE:HG12	9:C:66:VAL:HG22	1.88	0.55
10:D:98:LEU:HB2	10:D:135:TYR:HB3	1.88	0.55
16:J:9:ARG:HH21	16:J:42:LEU:HD13	1.70	0.55
22:P:70:ARG:HD2	22:P:73:ALA:HB3	1.88	0.55
28:a:871:U:H2’	28:a:872:U:C6	2.41	0.55
7:A:17:U:H2’	7:A:18:C:C6	2.41	0.55
7:A:264:C:O2’	23:Q:66:PRO:O	2.23	0.55

Continued on next page...

For Manuscript Review

Continued from previous page...

Atom-1	Atom-2	Interatomic distance (Å)	Clash overlap (Å)
7:A:374:A:H5"	7:A:451:A:C8	2.41	0.55
8:B:206:ALA:O	8:B:210:VAL:HG23	2.07	0.55
23:Q:61:ILE:HG22	23:Q:73:TRP:HE3	1.71	0.55
28:a:1047:G:O2'	28:a:1110:G:N1	2.21	0.55
33:f:31:VAL:HG22	33:f:96:MET:HE2	1.88	0.55
7:A:83:C:H5"	7:A:86:G:N1	2.16	0.55
7:A:459:A:H2'	7:A:460:A:H8	1.71	0.55
7:A:859:G:H2'	7:A:860:A:C8	2.42	0.55
7:A:1098:C:O2'	27:U:71:TYR:O	2.24	0.55
7:A:1251:A:N3	7:A:1369:C:O2'	2.38	0.55
28:a:2038:G:H2'	28:a:2039:U:O4'	2.06	0.55
28:a:2076:U:OP2	28:a:2238:G:N2	2.29	0.55
5:4:5:ILE:HD11	33:f:64:LYS:HD2	1.88	0.55
7:A:80:A:H8	7:A:80:A:O5'	1.89	0.55
8:B:58:ASN:ND2	8:B:220:THR:O	2.38	0.55
13:G:72:THR:HA	13:G:96:ARG:HE	1.72	0.55
28:a:1138:G:N3	36:i:108:MET:HE2	2.21	0.55
28:a:1746:A:H2'	28:a:1747:U:C6	2.41	0.55
7:A:718:A:H2	24:R:38:LYS:NZ	2.04	0.55
12:F:70:VAL:HA	12:F:73:GLU:OE1	2.06	0.55
28:a:1386:C:H2'	28:a:1387:A:C8	2.42	0.55
28:a:2316:G:H2'	28:a:2317:A:H8	1.71	0.55
7:A:83:C:C5'	7:A:86:G:H22	2.20	0.55
7:A:155:A:H2'	7:A:156:C:H6	1.71	0.55
7:A:344:A:H5"	7:A:345:C:H5	1.71	0.55
7:A:815:A:N7	7:A:1509:C:O2'	2.38	0.55
7:A:744:C:H2'	7:A:745:G:C8	2.41	0.55
16:J:25:ILE:HD11	16:J:92:LEU:HD11	1.89	0.55
23:Q:11:ARG:HH11	23:Q:11:ARG:HG3	1.72	0.55
46:s:4:GLU:OE2	51:x:23:ARG:NH1	2.40	0.55
7:A:499:A:C6	7:A:547:A:C8	2.95	0.55
20:N:20:TYR:HB2	20:N:55:SER:HB2	1.89	0.55
28:a:1475:G:O2'	28:a:1514:G:O6	2.24	0.55
44:q:10:LYS:NZ	44:q:23:GLU:OE2	2.39	0.55
7:A:473:U:H2'	7:A:474:G:H8	1.71	0.55
7:A:1120:C:H2'	7:A:1121:U:H6	1.72	0.55
15:I:45:ARG:O	15:I:49:ARG:HG3	2.07	0.55
22:P:50:THR:HG21	22:P:78:VAL:HG21	1.89	0.55
28:a:2329:U:H2'	28:a:2330:G:C8	2.42	0.55
34:g:42:GLU:OE1	34:g:55:ARG:NE	2.40	0.55
7:A:572:A:H5"	7:A:917:G:H4'	1.89	0.55

Continued on next page...

For Manuscript Review

Continued from previous page...

Atom-1	Atom-2	Interatomic distance (Å)	Clash overlap (Å)
7:A:1347:G:O6	15:I:12:ARG:NH2	2.29	0.55
30:c:155:ALA:HB2	30:c:162:VAL:HG23	1.88	0.55
7:A:531:U:H5'	7:A:531:U:C6	2.40	0.54
18:L:52:VAL:HG12	18:L:66:TYR:HD1	1.72	0.54
25:S:20:GLU:HA	25:S:23:VAL:HG12	1.89	0.54
44:q:65:ALA:HB3	44:q:95:ASP:HB2	1.89	0.54
7:A:486:U:H2'	7:A:487:A:H8	1.71	0.54
28:a:1794:A:H2'	28:a:1795:C:C6	2.43	0.54
52:y:6:LYS:HB2	52:y:58:GLU:HB2	1.90	0.54
7:A:674:G:H2'	7:A:675:A:C8	2.42	0.54
7:A:1137:C:O2	7:A:1138:G:N2	2.40	0.54
19:M:92:ARG:HB3	28:a:888:C:OP1	2.07	0.54
27:U:45:ARG:O	27:U:49:LYS:HG3	2.07	0.54
28:a:288:U:H2'	28:a:289:G:C8	2.42	0.54
28:a:2086:U:H2'	28:a:2087:G:C8	2.42	0.54
44:q:41:ILE:HD13	44:q:103:ALA:HA	1.89	0.54
45:r:37:THR:OG1	45:r:48:LYS:NZ	2.31	0.54
7:A:235:C:H2'	7:A:236:A:C8	2.43	0.54
7:A:451:A:N6	7:A:480:U:HO2'	2.04	0.54
7:A:974:A:OP1	20:N:69:ARG:NH2	2.39	0.54
7:A:1118:U:H1'	7:A:1179:A:C8	2.42	0.54
7:A:1277:C:H2'	7:A:1278:G:H5"	1.89	0.54
16:J:52:LEU:HD23	16:J:62:ARG:HD3	1.89	0.54
20:N:31:ILE:HG22	25:S:7:LYS:NZ	2.22	0.54
25:S:3:ARG:HD3	25:S:8:GLY:O	2.08	0.54
28:a:955:PSU:OP1	39:l:86:LYS:NZ	2.32	0.54
31:d:86:GLU:OE2	31:d:86:GLU:N	2.32	0.54
32:e:106:LYS:HE3	32:e:200:LEU:HB3	1.88	0.54
44:q:29:THR:HG22	44:q:66:HIS:HD2	1.72	0.54
7:A:1250:A:H2'	7:A:1251:A:C8	2.41	0.54
28:a:1365:A:OP1	50:w:3:ARG:NH1	2.32	0.54
39:l:117:PHE:HD2	39:l:130:PHE:HD1	1.55	0.54
40:m:103:ARG:HB2	40:m:110:MET:HE3	1.88	0.54
47:t:54:GLN:HG2	47:t:55:PRO:HD3	1.90	0.54
7:A:80:A:O5'	7:A:80:A:C8	2.61	0.54
7:A:83:C:H5'	7:A:84:U:H5"	1.90	0.54
28:a:172:A:H2'	28:a:173:A:C8	2.42	0.54
28:a:644:A:H2'	28:a:645:C:O4'	2.07	0.54
28:a:2788:C:H2'	28:a:2789:C:C6	2.43	0.54
37:j:9:ASN:OD1	37:j:18[A]:ARG:NH1	2.41	0.54
7:A:121:U:C2	7:A:121:U:H5"	2.43	0.54

Continued on next page...

For Manuscript Review

Continued from previous page...

Atom-1	Atom-2	Interatomic distance (Å)	Clash overlap (Å)
22:P:12:LYS:HG2	22:P:13:LYS:HG2	1.88	0.54
23:Q:27:ARG:HG3	23:Q:40:ARG:HB2	1.89	0.54
28:a:307:G:N1	28:a:310:A:OP2	2.33	0.54
28:a:1548:A:H2’	28:a:1549:A:H8	1.73	0.54
28:a:1570:A:H2’	28:a:1571:A:C8	2.42	0.54
28:a:1796:U:H2’	28:a:1797:G:H8	1.73	0.54
28:a:1802:A:H2’	28:a:1803:A:C8	2.43	0.54
33:f:108:VAL:HG12	33:f:114:PHE:CE1	2.43	0.54
1:0:40:ASP:HB3	1:0:43:VAL:HG12	1.90	0.54
24:R:48:ARG:O	24:R:50:LYS:N	2.41	0.54
26:T:35:VAL:HG21	26:T:54:MET:HE3	1.88	0.54
44:q:4:VAL:HG22	44:q:13:ARG:HB2	1.90	0.54
7:A:131:A:H2’	7:A:132:C:C6	2.43	0.54
7:A:493:A:H2’	7:A:494:G:C1’	2.38	0.54
7:A:769:G:H4’	7:A:1513:A:H4’	1.89	0.54
7:A:1025:U:H4’	7:A:1026:G:C8	2.41	0.54
7:A:1121:U:C2	7:A:1122:U:C5	2.95	0.54
7:A:1240:U:O4	13:G:109:ARG:NE	2.33	0.54
7:A:1380:U:C4’	7:A:1381:U:H5	2.21	0.54
8:B:205:ASP:OD2	8:B:205:ASP:N	2.39	0.54
12:F:32:ALA:HB2	12:F:70:VAL:HG11	1.89	0.54
28:a:1197:G:H2’	28:a:1198:U:H6	1.73	0.54
29:b:1:U:H2’	29:b:2:G:C8	2.39	0.54
47:t:98:SER:O	47:t:98:SER:OG	2.25	0.54
7:A:84:U:H3’	7:A:86:G:H22	1.73	0.53
7:A:1288:A:N1	7:A:1371:G:H1’	2.23	0.53
7:A:1415:A:H2’	7:A:1416:G:H8	1.73	0.53
13:G:111:ARG:HB3	13:G:119:ARG:HD3	1.90	0.53
15:I:23:PRO:HA	15:I:60:LYS:NZ	2.23	0.53
22:P:48:GLU:OE2	22:P:51:ARG:HD3	2.08	0.53
28:a:78:U:H2’	28:a:79:C:C6	2.43	0.53
8:B:225:ARG:HB2	8:B:225:ARG:NH1	2.23	0.53
12:F:61:LEU:HD12	12:F:62:MET:N	2.22	0.53
19:M:77:ILE:O	19:M:81:MET:HG2	2.08	0.53
22:P:74:LEU:HA	22:P:77:GLU:HG2	1.91	0.53
28:a:813:U:H2’	28:a:814:C:H6	1.73	0.53
28:a:993:G:OP2	43:p:51:ARG:NH2	2.36	0.53
28:a:1028:A:N6	28:a:1125:G:H2’	2.23	0.53
28:a:1683:U:H2’	28:a:1684:G:H8	1.71	0.53
28:a:1853:A:H2’	28:a:1854:A:C8	2.43	0.53
28:a:2866:U:O4	56:a:6209:SPD:N10	2.41	0.53

Continued on next page...

For Manuscript Review

Continued from previous page...

Atom-1	Atom-2	Interatomic distance (Å)	Clash overlap (Å)
47:t:50:PRO:HA	47:t:54:GLN:HB3	1.90	0.53
7:A:83:C:C5'	7:A:84:U:H5"	2.38	0.53
7:A:203:G:O2'	7:A:465:A:N1	2.41	0.53
7:A:459:A:H2'	7:A:460:A:C8	2.43	0.53
7:A:1191:A:OP1	9:C:4:LYS:NZ	2.41	0.53
9:C:79:LYS:HB2	9:C:83:ASP:HB2	1.90	0.53
16:J:65:TYR:HB3	20:N:96:LEU:HD11	1.90	0.53
28:a:2793:C:H2'	28:a:2794:C:C6	2.43	0.53
29:b:14:U:OP2	29:b:70:C:O2'	2.26	0.53
36:i:96:ARG:HG3	36:i:99:ARG:HB2	1.90	0.53
42:o:91:ALA:HB3	42:o:111:LYS:HG3	1.89	0.53
7:A:78:A:N6	7:A:90:C:C2	2.76	0.53
7:A:253:A:OP1	23:Q:69:LYS:NZ	2.33	0.53
7:A:437:U:O2	7:A:437:U:H2'	2.09	0.53
7:A:875:U:O2'	14:H:15:ARG:NH1	2.38	0.53
7:A:1175:G:H2'	7:A:1176:A:H8	1.74	0.53
12:F:10:VAL:HA	12:F:84:VAL:HA	1.89	0.53
12:F:25:TYR:O	12:F:29:ILE:HD13	2.08	0.53
18:L:36:ARG:CZ	18:L:38:TYR:HD1	2.22	0.53
28:a:2233:U:H2'	28:a:2234:G:C8	2.43	0.53
7:A:56:U:H2'	7:A:57:G:H8	1.74	0.53
7:A:545:C:OP1	10:D:58:LYS:NZ	2.42	0.53
7:A:652:U:O4	7:A:752:G:O2'	2.25	0.53
7:A:715:A:H2'	7:A:716:A:C8	2.44	0.53
7:A:1273:C:H2'	7:A:1274:A:O4'	2.07	0.53
7:A:1355:G:H2'	7:A:1356:G:H8	1.73	0.53
11:E:141:ILE:O	11:E:145:GLU:HG3	2.08	0.53
21:O:64:ARG:NH1	21:O:68:ASP:OD2	2.39	0.53
22:P:50:THR:CB	22:P:78:VAL:HG21	2.39	0.53
22:P:56:ARG:HA	22:P:59:HIS:CB	2.39	0.53
25:S:20:GLU:O	25:S:21:LYS:C	2.50	0.53
35:h:2:GLN:OE1	35:h:2:GLN:HA	2.08	0.53
3:2:32:ILE:O	3:2:32:ILE:HG22	2.07	0.53
7:A:155:A:H2'	7:A:156:C:C6	2.44	0.53
7:A:405:U:OP1	7:A:406:G:O2'	2.25	0.53
7:A:1163:A:H2'	7:A:1164:G:C8	2.43	0.53
7:A:1224:U:O2'	7:A:1322:C:OP1	2.23	0.53
7:A:1532:U:H4'	7:A:1533:C:OP1	2.08	0.53
13:G:126:ASP:OD2	13:G:132:GLY:N	2.41	0.53
28:a:813:U:H2'	28:a:814:C:C6	2.44	0.53
34:g:105:LEU:HB2	34:g:113:VAL:HB	1.91	0.53

Continued on next page...

For Manuscript Review

Continued from previous page...

Atom-1	Atom-2	Interatomic distance (Å)	Clash overlap (Å)
34:g:116:GLN:OE1	34:g:117:LEU:N	2.42	0.53
7:A:878:A:OP1	14:H:80:ARG:HB2	2.09	0.53
52:y:16:ARG:HG3	52:y:54:MET:HE1	1.90	0.53
9:C:131:ARG:HB3	9:C:131:ARG:HH11	1.74	0.53
24:R:9:LYS:HA	24:R:46:GLY:HA3	1.91	0.53
51:x:4:LYS:H	51:x:4:LYS:CD	2.20	0.53
7:A:1121:U:H2'	7:A:1122:U:C6	2.44	0.53
15:I:35:LEU:HD11	15:I:48:VAL:HG21	1.91	0.53
15:I:50:GLN:O	15:I:54:LEU:HD22	2.08	0.53
19:M:7:ILE:HD11	19:M:22:ILE:HA	1.91	0.53
7:A:405:U:H5'	7:A:406:G:C4'	2.39	0.53
7:A:1486:G:H2'	7:A:1487:G:C8	2.44	0.53
17:K:79:ILE:HB	17:K:105:PHE:HE1	1.74	0.53
28:a:414:C:H2'	28:a:415:A:C8	2.44	0.53
28:a:2751:G:C5	34:g:3:ARG:NH2	2.77	0.53
7:A:391:G:O2'	7:A:392:C:H6	1.92	0.52
7:A:413:G:O3'	7:A:428:G:N2	2.42	0.52
7:A:1122:U:HO2'	7:A:1123:U:C5'	2.21	0.52
7:A:1314:C:H2'	7:A:1315:U:C6	2.44	0.52
12:F:18:VAL:HG22	12:F:19:PRO:HD3	1.91	0.52
23:Q:17:MET:HB2	23:Q:20:SER:O	2.09	0.52
28:a:365:U:H2'	28:a:366:C:C6	2.44	0.52
28:a:639:U:H2'	28:a:640:C:H6	1.73	0.52
28:a:1278:C:H2'	28:a:1279:G:H8	1.74	0.52
28:a:2796:U:H3	28:a:2799:A:H61	1.57	0.52
42:o:54:GLY:O	42:o:57:SER:OG	2.20	0.52
15:I:80:ARG:O	15:I:84:THR:HG23	2.09	0.52
28:a:586:A:N1	28:a:809:G:O2'	2.38	0.52
28:a:1494:A:H2'	28:a:1495:A:C8	2.44	0.52
30:c:118:SER:HB3	30:c:129:THR:HB	1.91	0.52
40:m:28:LEU:HD23	40:m:48:VAL:HG21	1.90	0.52
8:B:21:ARG:O	8:B:21:ARG:HG3	2.09	0.52
17:K:37:ARG:NH1	17:K:83:GLU:OE2	2.42	0.52
20:N:31:ILE:HG22	25:S:7:LYS:HZ3	1.73	0.52
28:a:139:U:C4	46:s:1:MET:HE1	2.44	0.52
33:f:57:LEU:O	33:f:60:ILE:HG22	2.09	0.52
34:g:22:GLN:OE1	34:g:55:ARG:NH1	2.43	0.52
34:g:29:LYS:H	34:g:29:LYS:HD2	1.75	0.52
2:1:4:THR:HG22	28:a:687:C:H1'	1.91	0.52
2:1:12:ARG:HG3	2:1:12:ARG:NH1	2.25	0.52
5:4:23:LYS:O	5:4:24:ILE:HD13	2.10	0.52

Continued on next page...

For Manuscript Review

Continued from previous page...

Atom-1	Atom-2	Interatomic distance (Å)	Clash overlap (Å)
7:A:337:G:H2'	7:A:338:A:C8	2.44	0.52
7:A:397:A:N7	7:A:547:A:O2'	2.41	0.52
7:A:1530:G:O6	27:U:46:LYS:NZ	2.41	0.52
15:I:25:ASN:N	15:I:62:ASP:OD1	2.42	0.52
20:N:34:VAL:C	20:N:36:ALA:N	2.67	0.52
28:a:1141:U:H4'	28:a:1142:A:O4'	2.09	0.52
5:4:58:ASP:O	5:4:62:LYS:HG3	2.08	0.52
7:A:411:A:H1'	7:A:413:G:OP1	2.08	0.52
7:A:1222:G:OP2	7:A:1322:C:N4	2.40	0.52
8:B:66:LYS:N	8:B:159:ASP:OD1	2.28	0.52
21:O:64:ARG:O	21:O:64:ARG:HD3	2.09	0.52
28:a:1266:G:O2'	28:a:2012:G:O6	2.24	0.52
7:A:429:U:O4'	7:A:430:A:C8	2.62	0.52
7:A:1005:A:H2'	7:A:1006:G:O4'	2.10	0.52
7:A:1162:C:H2'	7:A:1163:A:C8	2.44	0.52
13:G:42:ILE:HG21	13:G:116:MET:HG3	1.92	0.52
34:g:155:GLU:HG2	34:g:157:TYR:H	1.74	0.52
51:x:10:SER:H	51:x:13:GLU:CD	2.17	0.52
11:E:85:VAL:HG11	11:E:147:MET:HG3	1.91	0.52
13:G:111:ARG:HH21	13:G:122:ASN:HB3	1.75	0.52
15:I:129:LYS:HD2	15:I:130:ARG:NH1	2.25	0.52
28:a:414:C:H2'	28:a:415:A:H8	1.74	0.52
28:a:818:G:N1	28:a:1188:U:OP2	2.35	0.52
28:a:2749:A:H5"	34:g:2:SER:HB3	1.92	0.52
28:a:2849:U:OP1	42:o:93:ARG:NH2	2.27	0.52
42:o:112:GLU:HG2	42:o:114:LEU:HG	1.92	0.52
7:A:280:C:N3	23:Q:41:THR:HG23	2.25	0.52
7:A:1278:G:H2'	7:A:1278:G:N3	2.24	0.52
15:I:19:VAL:HG22	15:I:65:ILE:HG13	1.92	0.52
33:f:29:PRO:HB2	33:f:169:LEU:HD22	1.92	0.52
7:A:384:G:H2'	7:A:385:C:H6	1.75	0.52
7:A:607:A:C2	7:A:608:A:C4	2.98	0.52
7:A:939:G:OP1	13:G:102:ARG:NH1	2.31	0.52
7:A:1228:C:OP2	19:M:110:LYS:NZ	2.41	0.52
20:N:23:LYS:O	20:N:26:GLU:HG2	2.10	0.52
21:O:78:TYR:O	21:O:82:ILE:HG23	2.09	0.52
22:P:40:ASN:O	22:P:43:ALA:HB2	2.10	0.52
25:S:19:VAL:O	25:S:23:VAL:HG12	2.08	0.52
26:T:25:ARG:O	26:T:29:ARG:HG3	2.10	0.52
28:a:1536:C:H4'	28:a:1537:G:C4	2.45	0.52
28:a:2564:A:OP1	28:a:2648:G:O2'	2.25	0.52

Continued on next page...

For Manuscript Review

Continued from previous page...

Atom-1	Atom-2	Interatomic distance (Å)	Clash overlap (Å)
28:a:2615:U:C2	53:z:4:GLN:HA	2.45	0.52
28:a:2796:U:H3	28:a:2799:A:N6	2.07	0.52
33:f:104:ILE:HD11	33:f:175:PHE:CD2	2.45	0.52
41:n:13:ARG:HG2	41:n:13:ARG:HH11	1.75	0.52
7:A:846:G:N2	7:A:847:G:C2	2.78	0.52
7:A:1124:G:N2	7:A:1125:U:O4	2.33	0.52
22:P:41:PRO:HG2	22:P:42:ILE:H	1.75	0.52
28:a:64:A:H2'	28:a:65:U:C6	2.45	0.52
28:a:157:C:H2'	28:a:158:U:O4'	2.10	0.52
28:a:2292:U:H2'	28:a:2293:G:H8	1.74	0.52
31:d:8:LYS:NZ	31:d:195:GLY:O	2.39	0.52
35:h:34:GLY:O	35:h:36:ALA:N	2.43	0.51
43:p:72:ASN:HB3	43:p:110:VAL:HG11	1.92	0.51
52:y:11:ARG:HB2	52:y:54:MET:HB2	1.91	0.51
7:A:193:C:H2'	7:A:194:C:C6	2.46	0.51
7:A:877:G:O2'	7:A:878:A:OP2	2.21	0.51
7:A:1062:U:H2'	7:A:1063:C:C6	2.46	0.51
9:C:26:THR:HG23	20:N:76:LYS:HD2	1.91	0.51
20:N:19:LYS:HE3	20:N:20:TYR:CE2	2.44	0.51
28:a:1683:U:H2'	28:a:1684:G:C8	2.45	0.51
7:A:56:U:H2'	7:A:57:G:C8	2.45	0.51
7:A:496:A:N3	7:A:496:A:H2'	2.24	0.51
15:I:46:MET:O	15:I:50:GLN:HG3	2.11	0.51
28:a:2514:U:H2'	28:a:2515:C:C6	2.44	0.51
4:3:1:MET:HE2	4:3:34:LYS:HG2	1.92	0.51
7:A:8:A:C6	10:D:206:LYS:HB2	2.46	0.51
7:A:399:G:H2'	7:A:400:C:C6	2.45	0.51
7:A:981:U:H2'	7:A:982:U:C5	2.44	0.51
7:A:1372:U:H2'	7:A:1373:G:O4'	2.10	0.51
7:A:1452:C:H4'	7:A:1453:G:H5"	1.93	0.51
8:B:67:ILE:H	8:B:89:GLN:NE2	2.09	0.51
13:G:111:ARG:NH2	13:G:122:ASN:HB3	2.25	0.51
19:M:25:VAL:HG13	19:M:29:ARG:HB3	1.91	0.51
28:a:475:C:O2	28:a:479:A:N6	2.43	0.51
28:a:527:C:O2'	28:a:2779:U:O2'	2.27	0.51
28:a:1292:G:H2'	28:a:1293:C:C6	2.46	0.51
28:a:1796:U:H2'	28:a:1797:G:C8	2.45	0.51
28:a:2216:G:H2'	28:a:2217:G:H8	1.76	0.51
28:a:2533:U:OP1	28:a:2665:A:O2'	2.28	0.51
34:g:9:VAL:HG22	34:g:50:LEU:H	1.75	0.51
7:A:79:G:H3'	7:A:80:A:H8	1.76	0.51

Continued on next page...

For Manuscript Review

Continued from previous page...

Atom-1	Atom-2	Interatomic distance (Å)	Clash overlap (Å)
7:A:427:U:O2’	7:A:541:G:OP1	2.27	0.51
7:A:1249:C:H4’	15:I:75:GLN:HE22	1.74	0.51
14:H:96:MET:SD	14:H:130:ALA:HB1	2.49	0.51
15:I:106:ARG:HD2	15:I:107:ASP:N	2.25	0.51
19:M:96:PRO:HB2	19:M:100:GLN:OE1	2.09	0.51
28:a:2786:U:H2’	28:a:2787:C:H6	1.75	0.51
33:f:142:ASP:OD1	33:f:144:ASP:N	2.39	0.51
28:a:155:A:H2’	28:a:156:A:C8	2.46	0.51
28:a:729:G:OP1	30:c:10:SER:OG	2.28	0.51
28:a:1043:C:N3	28:a:1112:G:N1	2.38	0.51
28:a:2812:G:H2’	28:a:2813:A:C8	2.45	0.51
31:d:183:GLU:H	31:d:183:GLU:CD	2.12	0.51
32:e:165:HIS:CD2	32:e:166:LYS:HG2	2.46	0.51
33:f:159:THR:O	33:f:159:THR:OG1	2.29	0.51
47:t:28:VAL:HG22	47:t:34:VAL:HG12	1.93	0.51
7:A:437:U:O5’	10:D:152:GLN:NE2	2.44	0.51
7:A:1118:U:H1’	7:A:1179:A:C5	2.46	0.51
15:I:21:ILE:HD12	15:I:62:ASP:O	2.10	0.51
15:I:85:ARG:HA	15:I:88:MET:HE2	1.92	0.51
18:L:122:PRO:O	18:L:124:ALA:N	2.44	0.51
20:N:16:LEU:O	20:N:17:ALA:O	2.28	0.51
22:P:68:SER:OG	22:P:69:ASP:N	2.42	0.51
28:a:742:A:H2’	28:a:743:A:H8	1.75	0.51
32:e:146:VAL:HG12	32:e:167:VAL:HG22	1.91	0.51
39:l:41:LEU:HG	39:l:96:ILE:HD11	1.93	0.51
47:t:4:LYS:O	47:t:94:ARG:NH1	2.40	0.51
7:A:323:U:H2’	7:A:324:G:O4’	2.10	0.51
7:A:429:U:O4’	7:A:430:A:H8	1.94	0.51
7:A:978:A:C2	7:A:1319:A:C4	2.98	0.51
7:A:1380:U:H4’	7:A:1381:U:H5	1.75	0.51
9:C:27:LYS:HB3	9:C:28:GLU:OE1	2.10	0.51
14:H:22:LYS:O	14:H:65:TYR:OH	2.24	0.51
28:a:2243:U:H2’	28:a:2244:U:C6	2.45	0.51
5:4:41:HIS:O	5:4:45:THR:HG23	2.10	0.51
28:a:17:G:H2’	28:a:18:U:C6	2.46	0.51
28:a:1720:U:H2’	28:a:1721:G:O4’	2.11	0.51
31:d:116:LYS:HB2	31:d:165:MET:HB3	1.93	0.51
8:B:63:ARG:HH21	8:B:65:GLY:HA2	1.76	0.51
11:E:65:GLU:OE1	11:E:68:ARG:NH1	2.43	0.51
15:I:21:ILE:HD11	15:I:61:LEU:HG	1.93	0.51
25:S:19:VAL:HG13	25:S:20:GLU:OE1	2.11	0.51

Continued on next page...

For Manuscript Review

Continued from previous page...

Atom-1	Atom-2	Interatomic distance (Å)	Clash overlap (Å)
26:T:30:THR:O	26:T:34:LYS:HG2	2.11	0.51
28:a:1734:G:H2'	28:a:1735:A:H8	1.76	0.51
31:d:26:VAL:HG22	31:d:188:LEU:HD22	1.92	0.51
33:f:116:GLY:O	33:f:178:ARG:NH2	2.40	0.51
12:F:26:THR:HG23	12:F:36:ILE:HD12	1.93	0.50
18:L:87:VAL:O	18:L:88:LYS:C	2.54	0.50
28:a:286:U:H2'	28:a:287:G:H8	1.75	0.50
33:f:103:LEU:O	33:f:108:VAL:HG23	2.11	0.50
2:1:29:GLN:NE2	28:a:210:C:OP1	2.39	0.50
7:A:81:A:H2'	7:A:82:G:N7	2.25	0.50
7:A:880:C:OP1	18:L:5:ASN:ND2	2.44	0.50
7:A:1063:C:OP2	7:A:1064:G:O2'	2.26	0.50
18:L:30:LYS:HD2	18:L:59:ASN:HD21	1.76	0.50
27:U:52:ALA:HA	27:U:55:ARG:HD2	1.93	0.50
28:a:240:C:OP2	28:a:241:A:O2'	2.26	0.50
28:a:2364:C:H2'	28:a:2365:G:O4'	2.11	0.50
37:j:24:VAL:HG12	37:j:30:ARG:HD3	1.92	0.50
41:n:33:ARG:HG2	41:n:34:HIS:CD2	2.46	0.50
7:A:201:G:H1	7:A:216:U:H3	1.59	0.50
7:A:381:C:H2'	7:A:382:A:O4'	2.11	0.50
7:A:1031:C:H4'	7:A:1032:G:N7	2.26	0.50
27:U:63:GLU:HG2	27:U:66:ARG:HE	1.76	0.50
28:a:2591:C:H2'	28:a:2592:G:C8	2.46	0.50
44:q:26:ASP:O	44:q:27:ILE:HD13	2.12	0.50
7:A:293:G:C6	7:A:305:G:N1	2.78	0.50
7:A:555:U:H2'	7:A:556:C:C6	2.47	0.50
7:A:745:G:H2'	7:A:746:A:H8	1.74	0.50
7:A:1015:G:H2'	7:A:1016:A:C8	2.46	0.50
28:a:1013:C:H2'	28:a:1014:A:H8	1.75	0.50
30:c:76:ALA:HB2	30:c:96:TYR:CD1	2.46	0.50
7:A:451:A:H1'	7:A:452:A:C2	2.46	0.50
7:A:678:U:H2'	7:A:679:C:H6	1.76	0.50
9:C:130:PHE:HZ	9:C:164:ARG:HH22	1.59	0.50
28:a:326:G:O6	56:a:6214:SPD:N6	2.38	0.50
28:a:2845:U:H5"	42:o:52:ASN:O	2.12	0.50
33:f:60:ILE:HD11	33:f:138:PHE:CE2	2.47	0.50
33:f:114:PHE:C	33:f:114:PHE:CD2	2.90	0.50
33:f:132:VAL:HB	33:f:152:LEU:HD23	1.92	0.50
44:q:41:ILE:HD12	44:q:54:VAL:HG11	1.93	0.50
7:A:222:C:H2'	7:A:223:A:H8	1.75	0.50
7:A:1005:A:C4	7:A:1006:G:H1'	2.47	0.50

Continued on next page...

For Manuscript Review

Continued from previous page...

Atom-1	Atom-2	Interatomic distance (Å)	Clash overlap (Å)
24:R:73:ARG:HG2	24:R:73:ARG:O	2.11	0.50
28:a:589:U:H2'	28:a:590:A:C8	2.46	0.50
28:a:594:U:H2'	28:a:595:C:C6	2.47	0.50
28:a:2071:A:H2'	28:a:2072:C:C6	2.46	0.50
28:a:2469:A:H2'	28:a:2470:G:O4'	2.12	0.50
28:a:2698:U:H2'	28:a:2699:C:C6	2.45	0.50
45:r:24:ILE:HD13	45:r:36:LEU:HD11	1.93	0.50
45:r:83:LYS:HD2	45:r:95:ARG:HD3	1.93	0.50
51:x:10:SER:O	51:x:14:LEU:HB2	2.12	0.50
7:A:157:U:H1'	7:A:165:G:N2	2.26	0.50
7:A:373:A:C2'	7:A:374:A:H5'	2.42	0.50
7:A:579:A:H2'	7:A:580:C:C6	2.47	0.50
7:A:619:U:C1'	10:D:128:ARG:HH12	2.25	0.50
7:A:979:C:O2	20:N:59:ARG:NH1	2.45	0.50
11:E:20:ARG:HG3	11:E:33:PHE:CE2	2.46	0.50
19:M:10:PRO:HB2	19:M:13:LYS:HG3	1.94	0.50
34:g:90:VAL:HG23	34:g:160:LYS:HA	1.93	0.50
48:u:30:ILE:HD11	48:u:40:ILE:HD13	1.92	0.50
1:0:44:ARG:HG3	1:0:44:ARG:HH11	1.77	0.50
7:A:95:C:HO2'	7:A:96:U:H6	1.58	0.50
7:A:563:A:O2'	7:A:566:G:O2'	2.25	0.50
7:A:1271:A:H2'	7:A:1272:G:C8	2.46	0.50
9:C:202:ILE:HG22	9:C:204:LYS:HG2	1.94	0.50
24:R:21:ILE:HD13	24:R:54:GLN:CB	2.41	0.50
28:a:848:C:H2'	28:a:849:A:C8	2.47	0.50
28:a:1432:G:H2'	28:a:1433:A:C8	2.47	0.50
28:a:2030:6MZ:C2	28:a:2499:C:H5"	2.42	0.50
44:q:36:ALA:HA	44:q:58:VAL:HG23	1.92	0.50
46:s:33:LYS:HG2	46:s:80:TRP:CE3	2.47	0.50
7:A:83:C:C6	7:A:86:G:C6	2.99	0.50
7:A:578:C:O2'	7:A:728:A:N3	2.43	0.50
7:A:859:G:H2'	7:A:860:A:H8	1.76	0.50
7:A:944:G:N1	7:A:1338:G:OP2	2.41	0.50
7:A:999:C:H2'	7:A:1000:A:H8	1.77	0.50
7:A:1422:G:H5'	37:j:48:PRO:HG3	1.93	0.50
7:A:1496:C:H2'	7:A:1497:G:O4'	2.12	0.50
9:C:77:ILE:HG12	9:C:103:ILE:HG13	1.94	0.50
10:D:2:ALA:HA	10:D:68:LEU:HD11	1.93	0.50
11:E:60:ILE:O	11:E:64:MET:HG2	2.12	0.50
13:G:22:LEU:HD22	13:G:62:PHE:CE1	2.47	0.50
20:N:40:ASP:O	20:N:43:ASN:HB2	2.12	0.50

Continued on next page...

For Manuscript Review

Continued from previous page...

Atom-1	Atom-2	Interatomic distance (Å)	Clash overlap (Å)
32:e:195:GLN:O	32:e:198:GLU:HG3	2.12	0.50
33:f:34:ILE:CD1	33:f:156:ILE:HG13	2.42	0.50
37:j:76:VAL:HG12	42:o:73:VAL:HB	1.94	0.50
42:o:88:ARG:NH2	42:o:110:ILE:O	2.45	0.50
7:A:549:C:H2'	7:A:550:G:O4'	2.12	0.49
13:G:28:ASN:HA	13:G:31:MET:HE3	1.94	0.49
28:a:2025:C:H2'	28:a:2026:U:C6	2.47	0.49
28:a:2836:U:H2'	28:a:2837:A:C8	2.47	0.49
33:f:104:ILE:HD11	33:f:175:PHE:HD2	1.76	0.49
5:4:22:MET:HE3	33:f:106:ILE:HD11	1.94	0.49
7:A:382:A:H2'	7:A:383:A:C8	2.47	0.49
7:A:718:A:H2	24:R:38:LYS:HZ2	1.60	0.49
7:A:1004:A:H2'	7:A:1005:A:O4'	2.11	0.49
13:G:4:ARG:HA	13:G:4:ARG:NH1	2.26	0.49
22:P:19:VAL:HG13	22:P:37:GLY:CA	2.42	0.49
28:a:1271:G:O6	56:a:6217:SPD:N1	2.45	0.49
28:a:1733:G:H2'	28:a:1734:G:H8	1.76	0.49
28:a:1799:G:O2'	30:c:180:GLU:OE2	2.23	0.49
56:a:6217:SPD:H32	45:r:86:MET:HE2	1.94	0.49
31:d:61:THR:OG1	31:d:64:GLU:HG3	2.13	0.49
34:g:42:GLU:OE1	34:g:42:GLU:HA	2.12	0.49
37:j:17:ARG:NH1	37:j:17:ARG:HB3	2.27	0.49
52:y:37:GLU:O	52:y:38:ARG:NH1	2.45	0.49
7:A:461:A:H2'	7:A:462:G:C8	2.47	0.49
7:A:493:A:H2'	7:A:494:G:N9	2.27	0.49
7:A:1492:A:OP1	18:L:44:LYS:HE2	2.12	0.49
15:I:54:LEU:HD23	15:I:103:PHE:HE1	1.77	0.49
19:M:33:ILE:HD11	19:M:63:PHE:CE1	2.47	0.49
20:N:44:ALA:O	20:N:45:VAL:C	2.55	0.49
20:N:50:THR:CG2	25:S:13:LEU:HG	2.42	0.49
22:P:12:LYS:O	22:P:13:LYS:HB2	2.12	0.49
28:a:667:U:H2'	28:a:668:A:O4'	2.13	0.49
33:f:89:VAL:HG12	33:f:91:LEU:HD22	1.94	0.49
53:z:54:VAL:HG12	53:z:55:ILE:HG23	1.94	0.49
7:A:421:U:H5'	7:A:422:C:C6	2.48	0.49
7:A:1458:G:H5"	26:T:26:SER:HB2	1.94	0.49
11:E:38:VAL:HG11	11:E:114:VAL:HG22	1.95	0.49
12:F:67:PRO:HG2	12:F:70:VAL:HG22	1.94	0.49
14:H:25:VAL:HG13	14:H:63:LEU:HD11	1.94	0.49
16:J:5:ARG:HA	16:J:76:ILE:O	2.12	0.49
24:R:37:GLY:O	24:R:63:ARG:NH2	2.44	0.49

Continued on next page...

For Manuscript Review

Continued from previous page...

Atom-1	Atom-2	Interatomic distance (Å)	Clash overlap (Å)
28:a:2328:A:H2’	28:a:2329:U:H6	1.77	0.49
30:c:259:SER:O	30:c:259:SER:OG	2.29	0.49
39:l:117:PHE:HD2	39:l:130:PHE:CD1	2.30	0.49
7:A:416:G:H2’	7:A:417:G:C8	2.46	0.49
7:A:846:G:N2	7:A:847:G:N3	2.60	0.49
8:B:6:MET:HE3	8:B:47:VAL:HG11	1.95	0.49
10:D:59:GLN:OE1	10:D:62:ARG:NH1	2.45	0.49
16:J:82:LYS:HD2	16:J:82:LYS:N	2.22	0.49
28:a:624:C:O2’	28:a:657:U:OP1	2.29	0.49
40:m:35:LYS:HB2	40:m:112:TYR:CE2	2.47	0.49
7:A:1230:C:H2’	7:A:1231:G:C8	2.45	0.49
13:G:120:LEU:O	13:G:124:LEU:HD12	2.13	0.49
18:L:54:ARG:HH11	18:L:62:GLU:HG2	1.76	0.49
33:f:33:LYS:HA	33:f:96:MET:HE3	1.94	0.49
33:f:91:LEU:HB3	33:f:96:MET:HA	1.94	0.49
22:P:50:THR:HB	22:P:78:VAL:HG21	1.93	0.49
26:T:28:MET:O	26:T:32:ILE:HG13	2.12	0.49
28:a:703:U:H2’	28:a:704:G:O4’	2.13	0.49
33:f:47:LYS:HE2	33:f:50:LEU:HD12	1.94	0.49
34:g:48:ASN:O	34:g:49:THR:HG23	2.12	0.49
7:A:524:G:H2’	7:A:525:C:C6	2.47	0.49
7:A:613:C:C2	7:A:614:C:C5	3.00	0.49
8:B:161:LEU:HD22	8:B:176:ALA:HB2	1.93	0.49
20:N:22:ALA:O	20:N:25:ALA:HB3	2.12	0.49
28:a:638:G:H2’	28:a:639:U:C6	2.47	0.49
28:a:2250:G:O2’	28:a:2496:C:OP1	2.18	0.49
7:A:1295:U:O2’	7:A:1302:C:N4	2.46	0.49
7:A:1320:C:H2’	7:A:1321:U:C6	2.47	0.49
7:A:1366:C:O5’	7:A:1366:C:H6	1.96	0.49
7:A:1513:A:H2’	7:A:1514:G:C8	2.47	0.49
8:B:113:ARG:NH1	8:B:116:ASP:OD1	2.46	0.49
15:I:45:ARG:O	15:I:48:VAL:HG22	2.13	0.49
19:M:33:ILE:HD11	19:M:63:PHE:HE1	1.77	0.49
28:a:2859:G:H2’	28:a:2860:A:C8	2.48	0.49
48:u:9:ARG:HG2	48:u:41:GLU:HG2	1.94	0.49
9:C:150:LYS:HG2	9:C:201:TRP:CE3	2.47	0.49
13:G:79:ARG:HB2	13:G:84:THR:HG22	1.95	0.49
28:a:1248:G:OP1	32:e:44:ARG:NH1	2.35	0.49
28:a:2591:C:H2’	28:a:2592:G:H8	1.78	0.49
4:3:9:LYS:HG2	4:3:14:CYS:HB2	1.94	0.48
7:A:473:U:H2’	7:A:474:G:C8	2.47	0.48

Continued on next page...

For Manuscript Review

Continued from previous page...

Atom-1	Atom-2	Interatomic distance (Å)	Clash overlap (Å)
17:K:36:ASP:OD1	17:K:40:ASN:HB2	2.13	0.48
26:T:67:ILE:HB	26:T:71:LYS:HD3	1.95	0.48
28:a:581:C:H2'	28:a:582:A:C8	2.48	0.48
28:a:784:G:H5'	28:a:785:G:OP1	2.13	0.48
28:a:2799:A:O2'	28:a:2800:A:H5"	2.13	0.48
32:e:98:LYS:O	32:e:102:ARG:HG3	2.12	0.48
33:f:80:ARG:HB2	33:f:83:TYR:CZ	2.48	0.48
34:g:134:LYS:HB3	34:g:134:LYS:HE2	1.62	0.48
7:A:89:U:H2'	7:A:90:C:C6	2.49	0.48
7:A:500:G:H4'	7:A:501:C:OP1	2.14	0.48
7:A:547:A:H4'	7:A:548:G:O5'	2.14	0.48
7:A:974:A:H4'	7:A:975:A:H3'	1.95	0.48
7:A:976:G:P	20:N:71:HIS:HD1	2.36	0.48
9:C:24:ALA:HB2	9:C:32:ASN:HD22	1.78	0.48
16:J:52:LEU:HB2	20:N:81:ARG:HH11	1.78	0.48
24:R:49:ALA:O	24:R:52:GLN:N	2.46	0.48
33:f:135:GLN:HE22	33:f:146:VAL:HG13	1.78	0.48
7:A:223:A:H2'	7:A:224:U:C6	2.48	0.48
7:A:498:A:H8	7:A:498:A:OP2	1.97	0.48
7:A:673:A:H2'	7:A:674:G:H8	1.74	0.48
7:A:1118:U:H2'	7:A:1119:C:H6	1.78	0.48
7:A:1446:A:H8	7:A:1446:A:OP1	1.96	0.48
12:F:26:THR:HA	12:F:36:ILE:HD11	1.95	0.48
21:O:18:ASP:OD1	21:O:20:ASN:N	2.40	0.48
28:a:1386:C:H2'	28:a:1387:A:H8	1.76	0.48
28:a:1405:U:H2'	28:a:1406:U:C6	2.47	0.48
28:a:2064:C:H2'	28:a:2065:C:C6	2.49	0.48
37:j:17:ARG:HB3	37:j:17:ARG:CZ	2.42	0.48
3:2:50:VAL:HG11	3:2:58:VAL:HG21	1.96	0.48
7:A:580:C:H2'	7:A:581:G:O4'	2.13	0.48
7:A:840:C:O2	7:A:847:G:N2	2.46	0.48
7:A:1124:G:OP2	16:J:37:ARG:NH2	2.47	0.48
7:A:1380:U:O4	13:G:2:PRO:C	2.57	0.48
26:T:82:GLN:HA	26:T:85:LYS:HZ3	1.78	0.48
28:a:2483:C:N3	39:l:123:LYS:NZ	2.59	0.48
31:d:39:ASP:OD1	31:d:39:ASP:N	2.47	0.48
7:A:235:C:H2'	7:A:236:A:H8	1.77	0.48
7:A:270:A:H2'	7:A:271:C:C6	2.48	0.48
7:A:736:C:H2'	7:A:737:C:H6	1.78	0.48
7:A:949:A:OP1	19:M:100:GLN:HB2	2.12	0.48
7:A:1366:C:O3'	16:J:62:ARG:NH2	2.46	0.48

Continued on next page...

For Manuscript Review

Continued from previous page...

Atom-1	Atom-2	Interatomic distance (Å)	Clash overlap (Å)
7:A:1507:A:H2'	7:A:1508:A:C8	2.48	0.48
20:N:79:LEU:HD22	20:N:83:LYS:HB3	1.96	0.48
22:P:56:ARG:C	22:P:59:HIS:H	2.22	0.48
23:Q:25:ILE:HB	23:Q:42:THR:HG23	1.95	0.48
31:d:152:PRO:HG3	31:d:156:PHE:CZ	2.48	0.48
7:A:200:G:H2'	7:A:201:G:H8	1.78	0.48
7:A:405:U:H5'	7:A:406:G:H4'	1.95	0.48
28:a:305:C:H2'	28:a:306:U:C6	2.48	0.48
28:a:363:G:H2'	28:a:364:C:H6	1.79	0.48
28:a:1496:A:H2'	28:a:1498:C:C5	2.48	0.48
28:a:1794:A:H2'	28:a:1795:C:H6	1.77	0.48
28:a:1797:G:HO2'	30:c:257:THR:HG1	1.58	0.48
28:a:2292:U:H2'	28:a:2293:G:C8	2.48	0.48
28:a:2314:A:H2'	28:a:2315:G:C8	2.49	0.48
36:i:4:PHE:O	43:p:64:ARG:NH1	2.39	0.48
7:A:466:A:H4'	7:A:467:U:H5	1.78	0.48
7:A:1187:G:H5'	15:I:115:LYS:HE2	1.96	0.48
7:A:1494:G:HO2'	28:a:1912:A:HO2'	1.52	0.48
13:G:17:LYS:HE2	13:G:18:PHE:CZ	2.47	0.48
13:G:47:LEU:HD22	13:G:58:GLU:HB3	1.96	0.48
15:I:129:LYS:HD2	15:I:130:ARG:HH11	1.79	0.48
19:M:53:ILE:O	19:M:57:ARG:HG2	2.12	0.48
19:M:73:ILE:HD12	19:M:74:SER:N	2.29	0.48
20:N:14:VAL:O	20:N:17:ALA:HB3	2.14	0.48
20:N:24:ARG:O	20:N:25:ALA:C	2.57	0.48
26:T:31:PHE:O	26:T:35:VAL:HG23	2.14	0.48
27:U:70:LEU:HD23	27:U:71:TYR:HD2	1.79	0.48
28:a:155:A:H2'	28:a:156:A:H8	1.78	0.48
28:a:2313:C:H2'	28:a:2314:A:H8	1.79	0.48
28:a:2639:A:H2'	28:a:2640:G:O4'	2.14	0.48
7:A:193:C:H2'	7:A:194:C:H6	1.79	0.48
7:A:1095:U:H2'	7:A:1096:C:C6	2.49	0.48
7:A:1120:C:C2	7:A:1121:U:C5	3.02	0.48
9:C:6:HIS:CG	20:N:89:MET:HB3	2.48	0.48
9:C:42:TYR:CZ	9:C:90:VAL:HG11	2.48	0.48
11:E:15:LEU:HA	11:E:37:THR:HG22	1.96	0.48
12:F:38:ARG:HB3	12:F:63:ASN:HB2	1.96	0.48
20:N:20:TYR:CD1	20:N:52:PRO:CD	2.96	0.48
28:a:1315:C:O2'	28:a:1392:A:N3	2.45	0.48
36:i:60:ASP:OD1	36:i:60:ASP:N	2.46	0.48
46:s:76:ARG:HG2	46:s:76:ARG:HH11	1.79	0.48

Continued on next page...

For Manuscript Review

Continued from previous page...

Atom-1	Atom-2	Interatomic distance (Å)	Clash overlap (Å)
50:w:6:GLN:O	50:w:74:ARG:NH2	2.47	0.48
7:A:403:C:H2'	7:A:404:G:C8	2.48	0.48
7:A:421:U:N3	9:C:127:ARG:NH1	2.62	0.48
7:A:500:G:HO2'	7:A:501:C:P	2.37	0.48
7:A:909:A:OP1	18:L:18:LYS:NZ	2.40	0.48
16:J:14:ASP:OD2	16:J:16:ARG:NH1	2.46	0.48
17:K:117:PRO:HD2	27:U:35:ARG:HD3	1.96	0.48
20:N:41:ARG:O	20:N:42:TRP:C	2.55	0.48
26:T:80:THR:O	26:T:83:ILE:HG13	2.13	0.48
28:a:1306:C:H2'	28:a:1307:A:H8	1.78	0.48
28:a:1682:G:H2'	28:a:1683:U:C6	2.49	0.48
28:a:1842:G:H2'	28:a:1843:C:C6	2.49	0.48
46:s:76:ARG:HG2	46:s:76:ARG:NH1	2.28	0.48
7:A:976:G:OP1	20:N:71:HIS:ND1	2.45	0.48
7:A:1222:G:OP1	7:A:1321:U:O2'	2.30	0.48
15:I:47:VAL:HG12	15:I:80:ARG:HE	1.78	0.48
18:L:54:ARG:HG3	18:L:55:VAL:N	2.29	0.48
23:Q:7:THR:HG22	23:Q:62:ARG:HB3	1.95	0.48
26:T:81:ALA:O	26:T:85:LYS:HG3	2.13	0.48
28:a:363:G:H2'	28:a:364:C:C6	2.48	0.48
28:a:1413:A:H2'	28:a:1414:C:C6	2.48	0.48
28:a:1636:U:H2'	28:a:1637:A:C8	2.49	0.48
28:a:1808:A:H3'	28:a:1809:A:C8	2.48	0.48
28:a:2502:G:H5"	28:a:2503:2MA:H5"	1.94	0.48
35:h:3:VAL:HG23	35:h:37:VAL:O	2.13	0.48
48:u:20:LEU:HD22	48:u:25:LYS:HB2	1.96	0.48
7:A:1435:G:H2'	7:A:1436:U:C6	2.49	0.47
28:a:561:G:O2'	43:p:45:TYR:OH	2.19	0.47
28:a:1744:A:H3'	28:a:1745:A:H8	1.79	0.47
28:a:2818:U:H2'	28:a:2819:G:H8	1.78	0.47
34:g:27:LYS:HB2	34:g:32:GLU:OE1	2.15	0.47
7:A:590:U:OP1	14:H:31:LYS:HG3	2.14	0.47
8:B:183:VAL:HG12	8:B:196:VAL:HG13	1.96	0.47
10:D:23:SER:CB	10:D:110:THR:HB	2.42	0.47
24:R:49:ALA:O	24:R:50:LYS:C	2.56	0.47
28:a:535:G:C6	28:a:559:G:C6	3.02	0.47
28:a:1597:A:H5"	28:a:1598:A:H5'	1.94	0.47
28:a:1684:G:H2'	28:a:1685:C:H6	1.79	0.47
31:d:84:LEU:HD22	31:d:88:GLU:HB2	1.96	0.47
33:f:140:GLU:OE2	33:f:140:GLU:N	2.34	0.47
7:A:33:A:H2'	7:A:34:C:C6	2.50	0.47

Continued on next page...

For Manuscript Review

Continued from previous page...

Atom-1	Atom-2	Interatomic distance (Å)	Clash overlap (Å)
7:A:137:U:H3	7:A:226:G:H1	1.63	0.47
7:A:346:G:OP1	42:o:39:ARG:NH1	2.40	0.47
7:A:403:C:H2'	7:A:404:G:H8	1.80	0.47
7:A:522:C:H41	18:L:50:ARG:NH2	2.13	0.47
7:A:911:U:H2'	7:A:912:C:C6	2.49	0.47
7:A:945:G:C2	7:A:946:A:C8	3.03	0.47
7:A:1144:G:N2	7:A:1146:A:H62	2.12	0.47
9:C:23:PHE:C	9:C:23:PHE:CD1	2.92	0.47
20:N:50:THR:O	20:N:51:LEU:O	2.32	0.47
22:P:39:PHE:CB	22:P:74:LEU:HD13	2.37	0.47
28:a:272:A:H2'	28:a:273:G:C8	2.49	0.47
28:a:1263:U:O2'	53:z:4:GLN:OE1	2.30	0.47
37:j:102:PRO:HB3	37:j:123:LEU:HD11	1.96	0.47
37:j:111:LYS:NZ	37:j:111:LYS:HB3	2.28	0.47
7:A:1074:G:O2'	7:A:1101:A:N1	2.46	0.47
9:C:177:THR:HG22	9:C:180:ALA:H	1.79	0.47
13:G:21:GLU:N	13:G:21:GLU:OE1	2.48	0.47
16:J:24:GLU:O	16:J:28:THR:HG22	2.14	0.47
19:M:33:ILE:HD12	19:M:59:GLU:HB3	1.96	0.47
28:a:419:U:H2'	28:a:420:C:C6	2.50	0.47
28:a:832:U:H2'	28:a:833:A:C8	2.49	0.47
28:a:1443:U:H2'	28:a:1444:G:H8	1.78	0.47
48:u:2:PHE:HD1	48:u:50:MET:HE2	1.80	0.47
48:u:2:PHE:HE2	48:u:55:GLU:HG2	1.79	0.47
1:0:30:LYS:HD3	1:0:30:LYS:HA	1.68	0.47
7:A:544:G:H2'	7:A:545:C:O4'	2.15	0.47
7:A:545:C:H5'	10:D:69:GLU:HB2	1.96	0.47
7:A:546:A:O2'	7:A:548:G:O2'	2.22	0.47
7:A:932:C:H2'	7:A:933:G:C8	2.49	0.47
7:A:932:C:H2'	7:A:933:G:H8	1.79	0.47
7:A:1096:C:H2'	7:A:1097:C:H6	1.80	0.47
26:T:56:PRO:HG2	26:T:57:ILE:HD12	1.96	0.47
28:a:989:G:OP2	52:y:12:SER:HB2	2.14	0.47
28:a:1874:C:H2'	28:a:1875:G:O4'	2.14	0.47
28:a:2246:G:H2'	28:a:2247:A:C8	2.49	0.47
7:A:492:C:H2'	7:A:493:A:C8	2.49	0.47
7:A:592:G:H2'	7:A:593:U:C6	2.50	0.47
7:A:619:U:H4'	10:D:128:ARG:NH2	2.30	0.47
7:A:687:A:C2	7:A:704:A:C5	3.03	0.47
9:C:72:ARG:O	9:C:76:VAL:HG12	2.14	0.47
9:C:123:GLN:HB3	9:C:128:VAL:HB	1.97	0.47

Continued on next page...

For Manuscript Review

Continued from previous page...

Atom-1	Atom-2	Interatomic distance (Å)	Clash overlap (Å)
11:E:61:GLN:OE1	11:E:61:GLN:HA	2.14	0.47
11:E:101:GLU:H	11:E:101:GLU:CD	2.22	0.47
22:P:19:VAL:HG11	22:P:52:LEU:CD1	2.45	0.47
28:a:282:A:N6	28:a:359:G:O6	2.47	0.47
28:a:644:A:C2	28:a:2369:A:H1'	2.50	0.47
28:a:1149:G:H2'	28:a:1150:C:C6	2.50	0.47
28:a:1428:C:C5	28:a:1569:A:H5"	2.50	0.47
28:a:2818:U:H2'	28:a:2819:G:C8	2.50	0.47
33:f:135:GLN:HG3	33:f:150:ARG:O	2.14	0.47
37:j:99:ILE:HG12	37:j:115:ILE:HG23	1.96	0.47
1:0:10:LYS:O	1:0:52:ALA:N	2.42	0.47
5:4:37:CYS:SG	5:4:39:LYS:N	2.86	0.47
7:A:2:A:N3	7:A:2:A:H2'	2.29	0.47
7:A:236:A:H2'	7:A:237:G:C8	2.50	0.47
7:A:270:A:H2'	7:A:271:C:H6	1.80	0.47
7:A:272:C:H2'	7:A:273:U:H6	1.80	0.47
7:A:401:C:O2'	7:A:621:A:N3	2.46	0.47
7:A:678:U:H2'	7:A:679:C:C6	2.50	0.47
7:A:792:A:H1'	7:A:794:A:N7	2.30	0.47
7:A:922:G:H2'	7:A:923:A:C8	2.50	0.47
7:A:1023:U:H2'	7:A:1024:G:O4'	2.14	0.47
13:G:25:LYS:HB3	13:G:101:MET:HE1	1.97	0.47
25:S:3:ARG:NH1	25:S:10:PHE:HB2	2.30	0.47
26:T:37:ALA:HA	26:T:40:GLU:OE1	2.13	0.47
28:a:857:G:H2'	28:a:858:G:O4'	2.15	0.47
28:a:871:U:H2'	28:a:872:U:H6	1.80	0.47
28:a:1223:G:OP1	44:q:68:ARG:NH1	2.48	0.47
28:a:1281:G:H2'	28:a:1282:U:C6	2.50	0.47
31:d:14:ILE:HD12	31:d:24:VAL:HG11	1.95	0.47
34:g:40:ALA:HB2	34:g:58:TYR:CD2	2.50	0.47
36:i:13:ARG:HD2	36:i:51:GLY:O	2.14	0.47
7:A:78:A:N6	7:A:90:C:N3	2.63	0.47
7:A:1163:A:H2'	7:A:1164:G:H8	1.79	0.47
18:L:64:THR:HG22	18:L:93:VAL:HG22	1.97	0.47
28:a:1441:G:H2'	28:a:1442:U:C6	2.50	0.47
4:3:19:ARG:HB2	4:3:24:ARG:HD2	1.96	0.47
5:4:56:ARG:O	5:4:59:ARG:HG3	2.15	0.47
7:A:495:A:H4'	7:A:496:A:OP1	2.15	0.47
19:M:39:ILE:HD12	19:M:39:ILE:O	2.15	0.47
28:a:580:U:H2'	28:a:581:C:C6	2.50	0.47
28:a:1292:G:H2'	28:a:1293:C:H6	1.80	0.47

Continued on next page...

For Manuscript Review

Continued from previous page...

Atom-1	Atom-2	Interatomic distance (Å)	Clash overlap (Å)
28:a:1902:C:H4'	30:c:242:LYS:O	2.14	0.47
28:a:1914:C:H2'	28:a:1915:3TD:H6	1.96	0.47
28:a:2820:A:N3	28:a:2820:A:H2'	2.30	0.47
33:f:36:LEU:HD22	33:f:154:ILE:HG12	1.97	0.47
34:g:84:THR:OG1	34:g:134:LYS:HE3	2.15	0.47
39:l:78:LEU:HD12	39:l:78:LEU:HA	1.81	0.47
42:o:27:GLU:OE1	42:o:42:ALA:HB1	2.15	0.47
3:2:64:TYR:CE1	28:a:242:G:H5"	2.49	0.47
5:4:11:GLU:OE1	5:4:23:LYS:HB3	2.16	0.47
7:A:406:G:O6	7:A:495:A:C2'	2.62	0.47
7:A:451:A:O2'	7:A:452:A:OP2	2.27	0.47
7:A:599:C:H2'	7:A:600:A:H8	1.80	0.47
21:O:69:TYR:OH	21:O:73:LYS:HE3	2.15	0.47
25:S:26:GLY:O	25:S:28:LYS:HD2	2.14	0.47
28:a:276:U:H2'	28:a:277:G:C8	2.50	0.47
28:a:1130:U:C2	28:a:2025:C:H5"	2.50	0.47
28:a:1589:U:H2'	28:a:1590:A:C8	2.50	0.47
28:a:1798:U:H5"	30:c:258:ARG:HB2	1.97	0.47
28:a:2406:A:H1'	56:a:6212:SPD:H51	1.96	0.47
28:a:2557:G:H2'	28:a:2558:C:C6	2.50	0.47
32:e:149:ILE:CD1	32:e:175:ILE:HG22	2.43	0.47
39:l:58:LYS:O	39:l:60:GLN:NE2	2.39	0.47
44:q:11:GLN:NE2	44:q:39:LEU:HD11	2.30	0.47
3:2:52:LYS:HE3	3:2:52:LYS:HB3	1.55	0.46
5:4:26:SER:OG	5:4:27:THR:N	2.44	0.46
7:A:31:G:N1	7:A:48:C:H5"	2.30	0.46
7:A:83:C:C5	7:A:87:C:N4	2.82	0.46
7:A:96:U:H2'	7:A:97:G:H8	1.78	0.46
7:A:181:A:N6	7:A:195:A:OP2	2.49	0.46
7:A:430:A:P	10:D:22:LYS:HZ1	2.38	0.46
7:A:1125:U:C4	7:A:1127:G:C4	3.03	0.46
7:A:1314:C:H2'	7:A:1315:U:H6	1.80	0.46
7:A:1378:C:H2'	7:A:1379:G:O4'	2.15	0.46
8:B:66:LYS:HB3	8:B:90:PHE:CE1	2.44	0.46
10:D:121:LYS:HG3	10:D:131:ASN:HB3	1.97	0.46
12:F:3:HIS:NE2	12:F:65:GLU:OE1	2.49	0.46
19:M:86:TYR:OH	19:M:90:ARG:NH1	2.48	0.46
22:P:50:THR:CG2	22:P:78:VAL:HG21	2.44	0.46
24:R:48:ARG:O	24:R:49:ALA:C	2.58	0.46
28:a:634:C:H2'	28:a:635:C:C6	2.50	0.46
28:a:851:C:H2'	28:a:852:U:H6	1.80	0.46

Continued on next page...

For Manuscript Review

Continued from previous page...

Atom-1	Atom-2	Interatomic distance (Å)	Clash overlap (Å)
28:a:1349:C:C2	28:a:1350:C:C5	3.03	0.46
28:a:2250:G:OP1	28:a:2275:C:O2'	2.28	0.46
28:a:2809:A:H2'	28:a:2810:A:C8	2.50	0.46
7:A:405:U:H5'	7:A:406:G:O4'	2.15	0.46
7:A:451:A:HO2'	7:A:452:A:P	2.37	0.46
7:A:751:U:H2'	7:A:752:G:O4'	2.15	0.46
7:A:968:A:C8	7:A:1062:U:H4'	2.50	0.46
7:A:1404:C:H2'	7:A:1405:G:C8	2.50	0.46
11:E:54:ARG:HG3	11:E:54:ARG:HH11	1.80	0.46
20:N:62:ASN:OD1	20:N:62:ASN:N	2.49	0.46
22:P:60:TRP:C	22:P:63:GLN:HB2	2.40	0.46
28:a:577:G:O2'	28:a:1254:A:OP1	2.33	0.46
28:a:782:A:N7	30:c:220:VAL:HG21	2.31	0.46
28:a:1199:U:H1'	43:p:4:VAL:HG22	1.96	0.46
28:a:2597:G:H2'	28:a:2598:A:C8	2.51	0.46
28:a:2834:G:H2'	28:a:2879:A:H61	1.80	0.46
34:g:37:LEU:HD23	34:g:68:ALA:HB1	1.97	0.46
7:A:83:C:H3'	7:A:86:G:N2	2.30	0.46
7:A:649:A:H2'	7:A:650:G:O4'	2.16	0.46
7:A:1125:U:O2'	7:A:1126:U:O5'	2.31	0.46
14:H:69:LYS:HE2	14:H:69:LYS:HB2	1.72	0.46
18:L:54:ARG:HH11	18:L:62:GLU:CG	2.29	0.46
20:N:29:ALA:O	20:N:32:SER:OG	2.34	0.46
28:a:146:A:H2'	28:a:147:C:C6	2.50	0.46
28:a:883:G:N2	28:a:894:U:O2	2.48	0.46
28:a:1447:C:O2'	28:a:1544:A:N3	2.41	0.46
28:a:2014:A:H2'	28:a:2015:A:C8	2.49	0.46
28:a:2515:C:H2'	28:a:2516:A:H8	1.80	0.46
28:a:2680:U:O2'	28:a:2681:C:H5'	2.15	0.46
33:f:135:GLN:HB2	33:f:141:ILE:CD1	2.45	0.46
35:h:34:GLY:C	35:h:36:ALA:H	2.23	0.46
49:v:26:PHE:N	49:v:29:GLU:HG3	2.30	0.46
51:x:49:ASP:O	51:x:53:VAL:HG23	2.15	0.46
7:A:78:A:C4	7:A:92:U:N3	2.83	0.46
7:A:1158:C:H2'	7:A:1160:G:H5'	1.97	0.46
26:T:57:ILE:HD12	26:T:57:ILE:H	1.80	0.46
28:a:548:G:H2'	28:a:549:G:H1'	1.98	0.46
28:a:1590:A:H2'	28:a:1591:A:C8	2.51	0.46
46:s:54:GLU:HB2	46:s:91:GLN:NE2	2.31	0.46
5:4:63:ARG:HD3	25:S:5:LEU:HD11	1.97	0.46
7:A:57:G:H2'	7:A:58:C:C6	2.50	0.46

Continued on next page...

For Manuscript Review

Continued from previous page...

Atom-1	Atom-2	Interatomic distance (Å)	Clash overlap (Å)
7:A:363:A:O2'	7:A:364:A:H5'	2.16	0.46
7:A:470:C:H2'	7:A:471:U:C6	2.51	0.46
7:A:635:A:H2'	7:A:636:U:C6	2.51	0.46
7:A:937:A:O2'	7:A:1378:C:N4	2.48	0.46
7:A:1142:G:C5	7:A:1143:G:H1'	2.51	0.46
7:A:1295:U:O2'	7:A:1296:C:H5'	2.16	0.46
7:A:1326:U:H2'	7:A:1327:C:H6	1.81	0.46
7:A:1343:G:H2'	7:A:1344:C:C6	2.50	0.46
16:J:14:ASP:OD1	16:J:17:LEU:N	2.35	0.46
23:Q:28:PHE:HE1	23:Q:39:LYS:HG3	1.80	0.46
28:a:851:C:H2'	28:a:852:U:C6	2.51	0.46
28:a:1567:G:OP2	30:c:83:TYR:OH	2.24	0.46
28:a:2020:A:H5'	53:z:9:THR:CG2	2.46	0.46
28:a:2266:A:H4'	28:a:2267:A:N3	2.31	0.46
29:b:16:G:N2	29:b:69:G:H1'	2.29	0.46
34:g:140:VAL:O	34:g:144:VAL:HG23	2.15	0.46
37:j:121:GLU:OE2	42:o:65:SER:OG	2.29	0.46
7:A:450:G:O2'	7:A:481:G:N1	2.49	0.46
7:A:745:G:H2'	7:A:746:A:C8	2.50	0.46
15:I:81:HIS:O	15:I:84:THR:OG1	2.28	0.46
18:L:50:ARG:HB3	18:L:90:LEU:HD11	1.98	0.46
22:P:42:ILE:HG13	22:P:43:ALA:N	2.28	0.46
24:R:21:ILE:HD11	24:R:51:TYR:HD1	1.81	0.46
28:a:2:G:H2'	28:a:3:U:C6	2.50	0.46
28:a:476:G:N1	28:a:479:A:OP2	2.42	0.46
32:e:5:LEU:HD13	32:e:10:SER:HB2	1.98	0.46
34:g:144:VAL:O	34:g:148:LEU:HD12	2.15	0.46
51:x:14:LEU:HD12	51:x:14:LEU:HA	1.78	0.46
2:1:3:ARG:HD2	28:a:1613:G:O2'	2.15	0.46
7:A:466:A:OP2	7:A:466:A:H8	1.97	0.46
7:A:736:C:H2'	7:A:737:C:C6	2.50	0.46
7:A:1327:C:H2'	7:A:1328:C:H6	1.81	0.46
10:D:72:PHE:HE1	10:D:94:LEU:HD11	1.80	0.46
11:E:13:GLU:HG2	11:E:39:VAL:HG12	1.96	0.46
20:N:45:VAL:HG12	20:N:46:LEU:HD23	1.97	0.46
22:P:8:ARG:CB	22:P:28:ARG:HH11	2.28	0.46
27:U:40:LYS:O	27:U:43:THR:OG1	2.29	0.46
7:A:236:A:H5"	23:Q:44:LEU:HD11	1.97	0.46
8:B:37:LYS:HA	8:B:37:LYS:HD3	1.71	0.46
9:C:47:LEU:HB3	9:C:50:ALA:HB3	1.97	0.46
14:H:31:LYS:HB2	14:H:31:LYS:HE3	1.64	0.46

Continued on next page...

For Manuscript Review

Continued from previous page...

Atom-1	Atom-2	Interatomic distance (Å)	Clash overlap (Å)
16:J:60:ASP:OD2	16:J:60:ASP:N	2.48	0.46
19:M:9:ILE:HG22	19:M:21:SER:OG	2.15	0.46
20:N:3:LYS:HB2	20:N:6:MET:HG2	1.98	0.46
22:P:23:ASP:C	22:P:25:ARG:N	2.72	0.46
28:a:903:C:H2'	28:a:904:G:H8	1.81	0.46
28:a:1529:G:H2'	28:a:1530:G:H8	1.81	0.46
28:a:1842:G:H2'	28:a:1843:C:H6	1.80	0.46
28:a:1889:A:H2'	28:a:1890:A:C8	2.51	0.46
28:a:2445:2MG:OP1	32:e:69:ARG:NH1	2.43	0.46
34:g:95:ARG:NH1	34:g:95:ARG:HB3	2.30	0.46
37:j:95:ILE:O	37:j:95:ILE:HG12	2.16	0.46
39:l:44:ARG:HG2	39:l:44:ARG:HH11	1.81	0.46
51:x:10:SER:OG	51:x:13:GLU:OE1	2.29	0.46
7:A:204:G:C6	7:A:465:A:C8	3.04	0.46
7:A:406:G:O6	7:A:495:A:O2'	2.33	0.46
7:A:503:C:H2'	7:A:504:C:C6	2.51	0.46
7:A:946:A:H2'	7:A:947:G:C8	2.51	0.46
7:A:1077:G:N2	7:A:1080:A:OP2	2.45	0.46
8:B:20:THR:HA	8:B:39:HIS:CD2	2.51	0.46
10:D:172:GLU:HA	10:D:172:GLU:OE2	2.15	0.46
13:G:22:LEU:HD22	13:G:62:PHE:HE1	1.80	0.46
14:H:86:TYR:C	14:H:87:LYS:HD2	2.40	0.46
17:K:107:ILE:HD12	17:K:107:ILE:O	2.16	0.46
19:M:64:VAL:HG13	19:M:68:ASP:HB2	1.98	0.46
20:N:20:TYR:CD1	20:N:52:PRO:HD3	2.51	0.46
20:N:29:ALA:O	20:N:30:ILE:C	2.58	0.46
20:N:30:ILE:HD13	20:N:30:ILE:N	2.31	0.46
21:O:26:GLU:OE1	21:O:77:ARG:NE	2.49	0.46
28:a:1296:G:OP1	28:a:2709:G:O2'	2.21	0.46
28:a:2523:G:HO2'	28:a:2764:A:HO2'	1.62	0.46
48:u:58:SER:O	48:u:73:LYS:NZ	2.49	0.46
7:A:123:U:OP1	7:A:311:C:O2'	2.33	0.46
7:A:757:U:O2'	7:A:879:C:O2	2.34	0.46
7:A:1005:A:N6	7:A:1024:G:O2'	2.49	0.46
7:A:1176:A:H2'	7:A:1177:G:C8	2.51	0.46
8:B:136:MET:HE2	8:B:136:MET:HA	1.98	0.46
9:C:164:ARG:HG3	9:C:164:ARG:HH11	1.81	0.46
9:C:203:PHE:HE2	9:C:205:GLY:C	2.23	0.46
20:N:82:ILE:O	20:N:86:GLU:HG3	2.16	0.46
28:a:78:U:H2'	28:a:79:C:H6	1.80	0.46
28:a:927:A:H2'	28:a:928:A:C8	2.51	0.46

Continued on next page...

For Manuscript Review

Continued from previous page...

Atom-1	Atom-2	Interatomic distance (Å)	Clash overlap (Å)
28:a:1417:C:O2'	28:a:1587:G:O2'	2.21	0.46
28:a:1751:U:H2'	28:a:1752:C:C6	2.51	0.46
28:a:2194:U:H2'	28:a:2195:U:H6	1.81	0.46
28:a:2567:G:H2'	28:a:2568:U:C6	2.51	0.46
33:f:26:MET:HE3	33:f:26:MET:HB3	1.80	0.46
33:f:33:LYS:CA	33:f:96:MET:HE3	2.46	0.46
7:A:1451:U:H3'	7:A:1452:C:H6	1.81	0.45
15:I:22:LYS:O	15:I:61:LEU:HD12	2.16	0.45
19:M:81:MET:HE2	28:a:888:C:C2	2.51	0.45
28:a:2579:C:OP1	56:a:6220:SPD:N1	2.35	0.45
36:i:121:LYS:HB2	36:i:121:LYS:HE2	1.80	0.45
43:p:86:ALA:HB2	43:p:116:ALA:HB2	1.97	0.45
44:q:11:GLN:HE21	44:q:39:LEU:HD11	1.81	0.45
7:A:1305:G:O2'	7:A:1306:A:OP2	2.32	0.45
7:A:1316:G:N2	7:A:1318:A:H3'	2.31	0.45
7:A:1325:C:H2'	7:A:1326:U:H6	1.81	0.45
7:A:1380:U:C4	13:G:3:ARG:HG2	2.52	0.45
9:C:121:THR:O	9:C:125:GLU:HG2	2.16	0.45
10:D:140:ASN:N	10:D:182:PHE:O	2.39	0.45
13:G:75:VAL:CG2	13:G:86:GLN:HB3	2.46	0.45
18:L:112:GLN:O	18:L:113:ALA:HB3	2.16	0.45
20:N:14:VAL:CG1	20:N:15:ALA:N	2.79	0.45
28:a:77:G:H2'	28:a:78:U:C6	2.51	0.45
33:f:80:ARG:HB2	33:f:83:TYR:CE1	2.51	0.45
33:f:117:LEU:HD12	33:f:176:PRO:O	2.16	0.45
38:k:77:ILE:CD1	38:k:108:ALA:HB1	2.46	0.45
42:o:2:SER:O	42:o:6:LYS:HB2	2.16	0.45
46:s:88:LYS:C	46:s:89:GLU:HG3	2.42	0.45
10:D:3:ARG:HG3	10:D:3:ARG:O	2.16	0.45
10:D:188:ARG:HD2	10:D:188:ARG:HA	1.76	0.45
20:N:31:ILE:HA	20:N:41:ARG:HD3	1.98	0.45
22:P:2:VAL:HG23	22:P:21:VAL:HG13	1.99	0.45
28:a:1484:U:H2'	28:a:1485:U:C6	2.52	0.45
28:a:1932:A:H2'	28:a:1933:G:O4'	2.16	0.45
28:a:2291:U:H2'	28:a:2292:U:H6	1.78	0.45
28:a:2813:A:H2'	28:a:2814:A:C8	2.51	0.45
29:b:5:U:H2'	29:b:6:G:H8	1.82	0.45
30:c:29:PRO:HG2	30:c:34:LEU:HD11	1.98	0.45
40:m:36:THR:OG1	40:m:37:THR:N	2.50	0.45
1:0:6:ARG:HG2	1:0:24:THR:HB	1.98	0.45
4:3:32:LYS:HE2	28:a:2478:A:H5'	1.98	0.45

Continued on next page...

For Manuscript Review

Continued from previous page...

Atom-1	Atom-2	Interatomic distance (Å)	Clash overlap (Å)
7:A:83:C:H5'	7:A:86:G:H22	1.82	0.45
7:A:414:A:H3'	7:A:415:A:H8	1.82	0.45
7:A:1170:A:H2'	7:A:1171:A:O4'	2.16	0.45
12:F:67:PRO:HB2	12:F:69:GLU:HG3	1.97	0.45
20:N:11:VAL:HG13	20:N:12:LYS:N	2.31	0.45
20:N:50:THR:HG22	25:S:13:LEU:HG	1.98	0.45
28:a:355:U:H2'	28:a:356:G:H8	1.81	0.45
28:a:1364:G:OP2	50:w:50:ARG:NH1	2.45	0.45
28:a:2087:G:H2'	28:a:2088:A:C8	2.52	0.45
28:a:2491:U:H4'	28:a:2492:U:OP1	2.16	0.45
28:a:2627:G:O2'	28:a:2781:A:N1	2.44	0.45
30:c:133:ARG:NH1	30:c:187:ASP:OD2	2.48	0.45
1:0:44:ARG:HA	1:0:44:ARG:HD2	1.85	0.45
7:A:620:C:H2'	7:A:621:A:C8	2.51	0.45
7:A:764:C:O5'	7:A:764:C:H6	1.99	0.45
7:A:779:C:H2'	7:A:780:A:O4'	2.17	0.45
7:A:1157:A:H4'	7:A:1158:C:O4'	2.17	0.45
7:A:1315:U:H2'	7:A:1316:G:O4'	2.17	0.45
8:B:128:LYS:H	8:B:128:LYS:HD2	1.82	0.45
8:B:196:VAL:HB	8:B:199:VAL:HG12	1.97	0.45
23:Q:10:GLY:O	23:Q:58:VAL:HA	2.16	0.45
24:R:47:THR:CG2	24:R:48:ARG:H	2.23	0.45
27:U:5:LYS:HA	27:U:5:LYS:HD2	1.64	0.45
28:a:828:U:H2'	28:a:829:A:C8	2.51	0.45
28:a:1747:U:H2'	28:a:1748:C:C6	2.51	0.45
28:a:2193:G:H2'	28:a:2194:U:C6	2.51	0.45
28:a:2345:G:N3	28:a:2381:A:H2'	2.32	0.45
28:a:2523:G:O2'	28:a:2764:A:O2'	2.32	0.45
33:f:30:ARG:H	33:f:159:THR:HG23	1.82	0.45
39:l:18:ARG:CB	39:l:18:ARG:HH11	2.30	0.45
48:u:53:LYS:HB3	48:u:55:GLU:OE2	2.17	0.45
49:v:26:PHE:H	49:v:29:GLU:HG3	1.82	0.45
5:4:65:ASN:N	5:4:65:ASN:OD1	2.49	0.45
7:A:175:C:N4	7:A:176:C:H41	2.15	0.45
7:A:337:G:H2'	7:A:338:A:H8	1.80	0.45
7:A:494:G:O2'	7:A:496:A:H1'	2.16	0.45
7:A:604:G:H2'	7:A:605:U:O4'	2.17	0.45
7:A:1270:G:H2'	7:A:1271:A:H8	1.82	0.45
7:A:1342:C:O2'	15:I:126:GLN:HG2	2.16	0.45
10:D:31:LYS:HE3	10:D:31:LYS:HB3	1.64	0.45
10:D:145:ILE:HG22	10:D:149:ALA:HB2	1.99	0.45

Continued on next page...

For Manuscript Review

Continued from previous page...

Atom-1	Atom-2	Interatomic distance (Å)	Clash overlap (Å)
18:L:66:TYR:HB3	18:L:96:HIS:CD2	2.52	0.45
28:a:367:G:H2'	28:a:368:A:H8	1.81	0.45
28:a:511:U:H4'	28:a:1235:G:H4'	1.98	0.45
28:a:552:U:H2'	28:a:553:G:H8	1.82	0.45
28:a:1684:G:H2'	28:a:1685:C:C6	2.50	0.45
28:a:2070:A:H2'	28:a:2071:A:C8	2.52	0.45
35:h:29:PHE:O	35:h:32:PRO:HD2	2.17	0.45
48:u:11:GLU:HG2	48:u:16:ALA:CB	2.46	0.45
52:y:7:ILE:O	52:y:35:THR:HA	2.15	0.45
53:z:54:VAL:HG12	53:z:55:ILE:CG2	2.47	0.45
7:A:426:U:H5'	7:A:427:U:OP2	2.16	0.45
7:A:562:U:H1'	18:L:12:ARG:HD2	1.98	0.45
7:A:718:A:C2	24:R:38:LYS:NZ	2.84	0.45
7:A:831:A:H2'	7:A:832:G:O4'	2.17	0.45
7:A:1010:U:H2'	7:A:1011:C:H6	1.81	0.45
7:A:1217:C:P	20:N:9:ARG:HH21	2.40	0.45
7:A:1409:C:H2'	7:A:1410:A:H8	1.82	0.45
10:D:197:GLU:OE2	10:D:197:GLU:N	2.45	0.45
11:E:54:ARG:HG3	11:E:54:ARG:NH1	2.31	0.45
18:L:110:ARG:HB2	18:L:119:VAL:HG21	1.99	0.45
28:a:290:U:H3	28:a:350:G:H1	1.65	0.45
28:a:581:C:H2'	28:a:582:A:H8	1.81	0.45
28:a:817:C:H2'	28:a:818:G:O4'	2.16	0.45
28:a:1429:G:H2'	28:a:1430:G:H8	1.81	0.45
28:a:1923:U:H2'	28:a:1924:C:C6	2.51	0.45
28:a:2097:A:H2'	28:a:2098:U:C6	2.52	0.45
28:a:2439:A:N6	28:a:2585:U:H4'	2.31	0.45
56:a:6217:SPD:H52	45:r:86:MET:HE2	1.99	0.45
35:h:15:LEU:HD23	35:h:16:GLY:N	2.31	0.45
44:q:35:PHE:HB2	44:q:59:ILE:HB	1.98	0.45
7:A:83:C:H5'	7:A:84:U:C3'	2.35	0.45
7:A:406:G:H1	7:A:436:C:H42	1.65	0.45
7:A:414:A:H2'	7:A:414:A:N3	2.32	0.45
7:A:494:G:O5'	7:A:494:G:H8	2.00	0.45
7:A:1302:C:C4	19:M:17:ILE:HD11	2.51	0.45
10:D:159:LEU:HD12	10:D:160:GLU:N	2.31	0.45
13:G:68:ASN:O	13:G:138:ARG:NH2	2.47	0.45
22:P:56:ARG:O	22:P:60:TRP:N	2.47	0.45
24:R:11:CYS:SG	24:R:48:ARG:HG3	2.57	0.45
25:S:80:TYR:CZ	25:S:82:GLY:HA2	2.51	0.45
28:a:163:C:H2'	28:a:164:C:C6	2.52	0.45

Continued on next page...

For Manuscript Review

Continued from previous page...

Atom-1	Atom-2	Interatomic distance (Å)	Clash overlap (Å)
28:a:1443:U:H2’	28:a:1444:G:C8	2.52	0.45
28:a:1704:C:H2’	28:a:1705:A:C8	2.51	0.45
34:g:95:ARG:HB3	34:g:95:ARG:HH11	1.82	0.45
42:o:37:LYS:HD3	42:o:37:LYS:N	2.32	0.45
7:A:2:A:N6	7:A:628:G:H1’	2.32	0.45
7:A:271:C:H2’	7:A:272:C:H6	1.80	0.45
7:A:1107:C:C4	7:A:1108:G:C8	3.04	0.45
7:A:1366:C:H2’	7:A:1367:C:O4’	2.17	0.45
9:C:78:GLY:C	9:C:80:LYS:N	2.72	0.45
17:K:23:ILE:HG12	17:K:96:THR:HG21	1.98	0.45
19:M:90:ARG:HH21	19:M:95:LEU:HD13	1.81	0.45
25:S:41:PHE:N	25:S:44:MET:SD	2.71	0.45
28:a:4:U:H2’	28:a:5:A:H8	1.82	0.45
28:a:1710:G:H2’	28:a:1711:A:C8	2.52	0.45
28:a:2191:A:H2’	28:a:2192:U:C6	2.52	0.45
28:a:2329:U:H2’	28:a:2330:G:H8	1.81	0.45
37:j:24:VAL:HG13	37:j:33:ALA:HB2	1.99	0.45
7:A:31:G:O2’	7:A:48:C:N4	2.50	0.45
7:A:83:C:H3’	7:A:86:G:C2	2.52	0.45
7:A:600:A:H2’	7:A:601:G:H8	1.82	0.45
7:A:1386:G:H2’	7:A:1387:G:H8	1.82	0.45
8:B:92:VAL:HG22	8:B:151:ILE:HD11	1.99	0.45
15:I:50:GLN:HB2	15:I:51:PRO:HD3	1.97	0.45
18:L:4:VAL:O	18:L:8:VAL:HG13	2.16	0.45
22:P:19:VAL:HG13	22:P:37:GLY:O	2.16	0.45
25:S:41:PHE:HB2	25:S:44:MET:HE3	1.98	0.45
28:a:2:G:H2’	28:a:3:U:H6	1.82	0.45
28:a:172:A:H2’	28:a:173:A:H8	1.82	0.45
28:a:1026:G:H2’	28:a:1027:A:C8	2.52	0.45
28:a:1484:U:H2’	28:a:1485:U:H6	1.82	0.45
28:a:1485:U:H2’	28:a:1486:U:H6	1.82	0.45
28:a:1571:A:H2’	28:a:1572:A:C8	2.52	0.45
28:a:1653:G:H3’	40:m:2:ARG:HG2	1.99	0.45
28:a:2030:6MZ:N3	28:a:2499:C:H5”	2.32	0.45
31:d:12:THR:OG1	31:d:13:ARG:N	2.50	0.45
38:k:78:ARG:HG3	38:k:78:ARG:HH11	1.82	0.45
5:4:1:MET:HE3	5:4:1:MET:HB3	1.76	0.44
7:A:129:A:H1’	7:A:130:A:C8	2.52	0.44
7:A:261:U:OP2	26:T:71:LYS:NZ	2.48	0.44
7:A:518:C:C5	7:A:530:G:H5’	2.52	0.44
7:A:1147:C:H4’	15:I:7:TYR:CE2	2.52	0.44

Continued on next page...

For Manuscript Review

Continued from previous page...

Atom-1	Atom-2	Interatomic distance (Å)	Clash overlap (Å)
10:D:50:ASP:O	10:D:54:GLN:HG3	2.17	0.44
14:H:67:GLN:C	14:H:69:LYS:H	2.25	0.44
14:H:79:SER:HB3	14:H:125:ILE:O	2.17	0.44
22:P:6:LEU:HD23	22:P:19:VAL:HA	1.99	0.44
28:a:558:U:H2'	28:a:559:G:H8	1.81	0.44
28:a:833:A:H2'	28:a:834:G:C8	2.52	0.44
28:a:1026:G:H2'	28:a:1027:A:H8	1.81	0.44
28:a:1216:G:OP1	43:p:11:ARG:NH2	2.49	0.44
49:v:33:ALA:N	49:v:64:ASP:OD1	2.50	0.44
50:w:56:MET:HE3	50:w:56:MET:HB2	1.72	0.44
52:y:5:ILE:HG22	52:y:59:GLU:HA	1.99	0.44
7:A:85:U:O3'	7:A:86:G:N3	2.49	0.44
7:A:868:C:H2'	7:A:869:G:O4'	2.18	0.44
7:A:1030:U:O4	7:A:1033:G:C5	2.71	0.44
7:A:1268:G:H2'	7:A:1269:A:C8	2.52	0.44
7:A:1327:C:H2'	7:A:1328:C:C6	2.52	0.44
7:A:1381:U:H2'	7:A:1382:C:O4'	2.18	0.44
7:A:1531:A:C6	7:A:1533:C:C2	3.05	0.44
8:B:116:ASP:O	8:B:119:THR:OG1	2.25	0.44
8:B:187:VAL:O	8:B:201:PRO:HA	2.17	0.44
19:M:73:ILE:HD12	19:M:74:SER:H	1.82	0.44
21:O:45:GLU:O	21:O:45:GLU:HG2	2.17	0.44
22:P:59:HIS:O	22:P:63:GLN:CG	2.65	0.44
28:a:819:A:H5"	28:a:973:A:N1	2.33	0.44
28:a:1203:U:OP2	28:a:1204:A:O2'	2.25	0.44
28:a:2087:G:H2'	28:a:2088:A:H8	1.82	0.44
7:A:146:G:N2	7:A:177:G:C8	2.86	0.44
7:A:439:U:O2	7:A:439:U:O4'	2.34	0.44
7:A:1326:U:O2'	7:A:1327:C:H5'	2.17	0.44
10:D:110:THR:OG1	10:D:111:ARG:N	2.50	0.44
21:O:80:GLN:O	21:O:84:ARG:HG3	2.18	0.44
28:a:887:U:O2'	28:a:889:C:OP2	2.32	0.44
28:a:1278:C:H2'	28:a:1279:G:C8	2.52	0.44
28:a:2615:U:H1'	53:z:4:GLN:HB3	1.99	0.44
32:e:12:LEU:HD12	32:e:12:LEU:HA	1.83	0.44
33:f:113:ASP:CG	33:f:113:ASP:O	2.61	0.44
36:i:110:PRO:O	36:i:115:GLY:HA3	2.17	0.44
45:r:4:ILE:HG12	45:r:106:VAL:HG22	2.00	0.44
48:u:75:GLN:HB2	48:u:92:VAL:HG23	1.99	0.44
4:3:1:MET:HE3	4:3:1:MET:HB2	1.85	0.44
7:A:375:U:OP2	22:P:70:ARG:HB2	2.18	0.44

Continued on next page...

For Manuscript Review

Continued from previous page...

Atom-1	Atom-2	Interatomic distance (Å)	Clash overlap (Å)
7:A:1285:A:H8	7:A:1285:A:O5'	2.00	0.44
8:B:97:LEU:H	8:B:100:MET:HE1	1.82	0.44
8:B:117:LEU:HB3	8:B:141:LEU:HD23	2.00	0.44
8:B:168:HIS:ND1	8:B:169:GLU:OE1	2.51	0.44
10:D:28:ILE:C	10:D:30:THR:N	2.74	0.44
11:E:149:SER:N	11:E:152:MET:HE2	2.29	0.44
18:L:36:ARG:NH1	18:L:38:TYR:HB3	2.32	0.44
28:a:448:U:OP2	56:a:6216:SPD:H91	2.16	0.44
28:a:1444:G:H2'	28:a:1445:G:H8	1.82	0.44
28:a:1509:A:N3	28:a:1510:G:C8	2.86	0.44
28:a:2193:G:H2'	28:a:2194:U:H6	1.82	0.44
29:b:2:G:H2'	29:b:3:C:C6	2.52	0.44
31:d:49:GLN:NE2	31:d:79:LEU:HD13	2.32	0.44
33:f:135:GLN:HB2	33:f:141:ILE:HD12	1.99	0.44
39:l:44:ARG:NH1	39:l:44:ARG:HG2	2.32	0.44
41:n:115:LEU:HD23	41:n:115:LEU:HA	1.84	0.44
1:0:10:LYS:HG2	1:0:22:THR:HG22	2.00	0.44
7:A:86:G:H4'	7:A:87:C:O4'	2.18	0.44
7:A:960:U:H2'	7:A:1225:A:H62	1.82	0.44
7:A:1266:G:N2	7:A:1268:G:H3'	2.33	0.44
7:A:1285:A:H4'	7:A:1286:U:OP1	2.16	0.44
7:A:1447:A:O2'	7:A:1448:C:H5'	2.18	0.44
9:C:73:PRO:HD3	9:C:105:GLU:OE2	2.17	0.44
11:E:70:ASN:ND2	11:E:70:ASN:C	2.55	0.44
16:J:72:ARG:HA	16:J:72:ARG:HD3	1.67	0.44
17:K:72:ASP:C	17:K:72:ASP:OD1	2.61	0.44
18:L:121:ARG:O	18:L:122:PRO:C	2.61	0.44
22:P:46:LYS:HE3	22:P:46:LYS:HB2	1.37	0.44
23:Q:12:VAL:HG22	23:Q:23:VAL:HG22	2.00	0.44
24:R:24:LYS:HE2	24:R:24:LYS:HB2	1.79	0.44
28:a:598:U:H2'	28:a:599:A:H8	1.82	0.44
28:a:2793:C:H2'	28:a:2794:C:H6	1.81	0.44
33:f:104:ILE:HD13	33:f:104:ILE:HA	1.86	0.44
33:f:140:GLU:H	33:f:140:GLU:CD	2.22	0.44
36:i:9:GLU:CD	36:i:9:GLU:H	2.25	0.44
39:l:26:VAL:CG2	39:l:133:LYS:HA	2.47	0.44
7:A:430:A:P	10:D:22:LYS:HZ2	2.39	0.44
7:A:458:U:H3'	7:A:459:A:H8	1.83	0.44
7:A:1149:C:H2'	7:A:1150:A:C8	2.53	0.44
7:A:1289:A:H61	15:I:72:ILE:HD11	1.82	0.44
7:A:1318:A:O2'	25:S:37:ARG:HD3	2.16	0.44

Continued on next page...

For Manuscript Review

Continued from previous page...

Atom-1	Atom-2	Interatomic distance (Å)	Clash overlap (Å)
7:A:1436:U:H2'	7:A:1437:A:H8	1.82	0.44
10:D:99:ASP:OD1	10:D:99:ASP:N	2.49	0.44
20:N:21:PHE:CG	20:N:22:ALA:N	2.82	0.44
20:N:39:GLU:OE2	20:N:43:ASN:ND2	2.51	0.44
20:N:85:ARG:O	20:N:89:MET:HG2	2.17	0.44
22:P:2:VAL:HA	22:P:22:ALA:O	2.17	0.44
22:P:8:ARG:CB	22:P:28:ARG:NH1	2.80	0.44
28:a:457:A:N1	28:a:470:A:H5"	2.33	0.44
28:a:1645:G:H5"	28:a:1646:C:H5'	1.98	0.44
28:a:2637:U:H2'	28:a:2638:G:O4'	2.18	0.44
29:b:48:U:H2'	29:b:49:C:C6	2.52	0.44
33:f:108:VAL:HG13	33:f:111:ILE:HD12	1.99	0.44
41:n:88:LYS:HE3	41:n:88:LYS:HB3	1.76	0.44
7:A:178:C:C2	7:A:179:A:C8	3.06	0.44
7:A:222:C:H2'	7:A:223:A:C8	2.52	0.44
7:A:1004:A:N6	7:A:1026:G:H1'	2.33	0.44
7:A:1280:A:P	16:J:9:ARG:HH22	2.41	0.44
7:A:1518:MA6:H8	7:A:1518:MA6:O5'	2.17	0.44
12:F:4:TYR:CD2	12:F:71:ILE:HG13	2.53	0.44
15:I:21:ILE:CG1	15:I:61:LEU:HG	2.48	0.44
28:a:184:C:H2'	28:a:185:G:C8	2.53	0.44
28:a:364:C:H2'	28:a:365:U:H6	1.82	0.44
28:a:1295:C:C2	28:a:1296:G:C8	3.06	0.44
28:a:1572:A:H2'	28:a:1573:G:H8	1.82	0.44
29:b:2:G:H2'	29:b:3:C:H6	1.82	0.44
35:h:8:LYS:HD3	35:h:14:SER:HA	1.99	0.44
49:v:53:CYS:SG	49:v:57:HIS:HA	2.57	0.44
7:A:600:A:H2'	7:A:601:G:C8	2.53	0.44
7:A:1409:C:H2'	7:A:1410:A:C8	2.51	0.44
10:D:23:SER:OG	10:D:24:GLY:N	2.51	0.44
10:D:151:LYS:C	10:D:153:SER:H	2.25	0.44
11:E:96:MET:HB2	11:E:96:MET:HE3	1.75	0.44
12:F:51:ILE:O	12:F:54:LEU:HB2	2.17	0.44
16:J:83:THR:O	16:J:87:LEU:HD12	2.18	0.44
18:L:122:PRO:O	18:L:123:LYS:C	2.60	0.44
28:a:27:G:O2'	28:a:28:A:OP2	2.34	0.44
28:a:1590:A:H2'	28:a:1591:A:H8	1.83	0.44
28:a:1637:A:H5'	28:a:1760:C:O2'	2.18	0.44
28:a:2537:U:H2'	28:a:2538:C:C6	2.53	0.44
48:u:77:VAL:HG23	48:u:89:ILE:HG12	2.00	0.44
52:y:24:LEU:HD11	52:y:54:MET:SD	2.57	0.44

Continued on next page...

For Manuscript Review

Continued from previous page...

Atom-1	Atom-2	Interatomic distance (Å)	Clash overlap (Å)
2:1:34:ARG:NH2	2:1:39:ARG:HD3	2.33	0.44
7:A:45:G:H2'	7:A:46:G:H8	1.83	0.44
7:A:83:C:H5	7:A:87:C:N4	2.16	0.44
7:A:91:U:O2	7:A:91:U:H2'	2.17	0.44
7:A:154:U:H2'	7:A:155:A:C8	2.53	0.44
7:A:292:G:O2'	7:A:609:A:N6	2.51	0.44
7:A:309:A:H2'	7:A:310:G:H8	1.83	0.44
7:A:669:G:O2'	7:A:670:G:H8	2.00	0.44
7:A:1510:C:HO2'	7:A:1511:G:H8	1.62	0.44
8:B:188:ASP:HB2	8:B:204:ASP:OD2	2.17	0.44
10:D:25:VAL:HG13	10:D:161:LEU:HG	1.99	0.44
13:G:75:VAL:HG21	13:G:86:GLN:HB3	1.99	0.44
22:P:39:PHE:CE1	22:P:74:LEU:CD2	3.00	0.44
28:a:6:A:H2'	28:a:7:G:C8	2.53	0.44
28:a:117:G:OP2	28:a:119:A:O2'	2.24	0.44
28:a:139:U:O4	46:s:1:MET:HE1	2.18	0.44
28:a:248:G:H5'	28:a:250:G:N7	2.33	0.44
28:a:445:C:H2'	28:a:446:G:O4'	2.18	0.44
28:a:934:U:H2'	28:a:935:C:C6	2.52	0.44
28:a:1509:A:HO2'	28:a:1510:G:H8	1.62	0.44
28:a:2723:C:H2'	28:a:2724:U:O4'	2.17	0.44
29:b:42:C:C5	33:f:66:LEU:HD13	2.53	0.44
29:b:115:A:H2'	29:b:116:G:H8	1.83	0.44
39:l:59:ARG:HE	39:l:60:GLN:HE22	1.66	0.44
43:p:109:LEU:HD11	44:q:40:MET:HE1	1.99	0.44
44:q:24:LYS:HZ2	44:q:24:LYS:HB3	1.83	0.44
3:2:8:ARG:NH2	28:a:244:A:OP2	2.36	0.43
7:A:410:G:C2	7:A:429:U:C2	3.05	0.43
7:A:1009:U:C2	7:A:1010:U:C5	3.06	0.43
7:A:1095:U:H2'	7:A:1096:C:H6	1.83	0.43
7:A:1371:G:H2'	7:A:1372:U:C6	2.53	0.43
8:B:9:MET:HE3	8:B:212:LEU:HD21	2.00	0.43
15:I:21:ILE:HG13	15:I:61:LEU:HG	2.00	0.43
17:K:126:LYS:NZ	17:K:126:LYS:HB3	2.33	0.43
20:N:19:LYS:HE3	20:N:20:TYR:CD2	2.53	0.43
21:O:3:LEU:HD23	21:O:3:LEU:HA	1.80	0.43
22:P:56:ARG:O	22:P:59:HIS:N	2.50	0.43
26:T:43:ASP:CG	26:T:46:ALA:H	2.25	0.43
28:a:543:G:H2'	28:a:544:C:O4'	2.18	0.43
28:a:594:U:H2'	28:a:595:C:H6	1.83	0.43
28:a:2216:G:H2'	28:a:2217:G:C8	2.53	0.43

Continued on next page...

For Manuscript Review

Continued from previous page...

Atom-1	Atom-2	Interatomic distance (Å)	Clash overlap (Å)
28:a:2590:A:H2'	28:a:2591:C:C6	2.53	0.43
28:a:2804:U:H2'	28:a:2805:C:H6	1.82	0.43
28:a:2820:A:H4'	40:m:3:HIS:CD2	2.53	0.43
1:0:7:GLU:HG2	1:0:27:LYS:HE3	2.00	0.43
7:A:264:C:O2'	7:A:265:G:OP1	2.36	0.43
7:A:323:U:OP1	26:T:25:ARG:NH2	2.50	0.43
7:A:474:G:H2'	7:A:475:C:C6	2.53	0.43
7:A:1265:C:H2'	7:A:1266:G:C8	2.53	0.43
8:B:27:MET:O	8:B:31:ILE:HG13	2.18	0.43
9:C:24:ALA:HB1	9:C:28:GLU:HG2	2.01	0.43
22:P:33:ILE:O	22:P:34:GLU:HB2	2.18	0.43
25:S:20:GLU:HA	25:S:23:VAL:CG1	2.47	0.43
26:T:20:HIS:CE1	26:T:24:ARG:HD2	2.53	0.43
28:a:849:A:H2'	28:a:850:U:C6	2.53	0.43
28:a:1316:U:H2'	28:a:1317:G:H8	1.84	0.43
28:a:1534:U:H2'	28:a:1536:C:C6	2.53	0.43
28:a:2236:U:H2'	28:a:2237:G:O4'	2.18	0.43
29:b:40:U:N3	29:b:44:G:OP2	2.42	0.43
33:f:79:ILE:HD12	33:f:79:ILE:O	2.18	0.43
45:r:41:LYS:HE3	53:z:22:LEU:HD11	2.00	0.43
7:A:121:U:O2	7:A:121:U:C2'	2.64	0.43
7:A:201:G:O2'	7:A:469:C:O2'	2.24	0.43
7:A:373:A:H2'	7:A:374:A:H5'	2.00	0.43
7:A:392:C:H5"	22:P:13:LYS:CE	2.37	0.43
7:A:653:U:OP1	14:H:56:LYS:NZ	2.52	0.43
7:A:734:G:O2'	24:R:60:LYS:HD3	2.18	0.43
7:A:1057:G:H2'	7:A:1058:G:O4'	2.18	0.43
15:I:42:GLU:O	15:I:46:MET:HG2	2.18	0.43
28:a:548:G:H2'	28:a:549:G:C1'	2.48	0.43
28:a:636:G:OP1	38:k:129:LYS:HG2	2.18	0.43
28:a:1198:U:H2'	28:a:1199:U:C6	2.53	0.43
28:a:1704:C:H2'	28:a:1705:A:H8	1.82	0.43
28:a:2372:U:H2'	28:a:2373:G:H8	1.84	0.43
28:a:2619:C:H5"	31:d:157:LYS:HG2	1.99	0.43
29:b:45:A:C4	29:b:46:A:C8	3.06	0.43
30:c:132:MET:HB2	30:c:132:MET:HE3	1.84	0.43
33:f:30:ARG:O	33:f:159:THR:HG22	2.18	0.43
7:A:406:G:H8	7:A:406:G:OP2	2.01	0.43
7:A:465:A:H2'	7:A:466:A:C8	2.53	0.43
7:A:488:C:H2'	7:A:489:C:C6	2.53	0.43
7:A:1033:G:C6	7:A:1034:G:C4	3.06	0.43

Continued on next page...

For Manuscript Review

Continued from previous page...

Atom-1	Atom-2	Interatomic distance (Å)	Clash overlap (Å)
8:B:113:ARG:HA	8:B:116:ASP:OD2	2.18	0.43
12:F:42:TRP:CE3	12:F:102:MET:HE1	2.53	0.43
12:F:45:ARG:HB2	12:F:59:TYR:CE1	2.53	0.43
18:L:114:ARG:O	18:L:117:TYR:N	2.46	0.43
22:P:19:VAL:HG11	22:P:52:LEU:HD12	1.99	0.43
28:a:208:C:H2'	28:a:209:C:H6	1.83	0.43
28:a:1733:G:H2'	28:a:1734:G:C8	2.52	0.43
28:a:2306:C:N4	33:f:39:GLY:O	2.41	0.43
37:j:98:ARG:NH2	37:j:100:PHE:HE2	2.15	0.43
46:s:6:ARG:NH2	46:s:37:ASP:O	2.51	0.43
5:4:8:LYS:HA	5:4:8:LYS:HD3	1.64	0.43
7:A:393:A:C2	7:A:394:G:C8	3.07	0.43
7:A:420:U:N3	7:A:422:C:N3	2.67	0.43
7:A:614:C:H2'	7:A:615:G:O4'	2.18	0.43
7:A:627:G:H2'	7:A:628:G:O4'	2.18	0.43
7:A:1086:U:H3	7:A:1099:G:N2	2.09	0.43
7:A:1410:A:H2'	7:A:1411:C:C6	2.53	0.43
7:A:1415:A:H2'	7:A:1416:G:C8	2.51	0.43
12:F:43:GLY:HA2	12:F:58:HIS:CE1	2.53	0.43
14:H:7:ILE:O	14:H:11:LEU:HG	2.19	0.43
14:H:26:THR:HG22	14:H:60:GLU:HG3	2.00	0.43
15:I:63:LEU:HB3	15:I:65:ILE:HD13	2.00	0.43
19:M:76:SER:O	19:M:80:LEU:HD13	2.17	0.43
20:N:5:SER:O	20:N:8:ALA:HB3	2.18	0.43
23:Q:16:LYS:HD3	23:Q:16:LYS:HA	1.82	0.43
28:a:273:G:C6	28:a:274:C:N4	2.87	0.43
28:a:333:G:H4'	56:a:6214:SPD:H41	2.00	0.43
28:a:576:U:H2'	28:a:577:G:C8	2.53	0.43
33:f:22:TYR:OH	33:f:165:GLU:OE1	2.34	0.43
7:A:233:C:H2'	7:A:234:C:H6	1.83	0.43
7:A:390:U:O2'	22:P:28:ARG:NH2	2.52	0.43
7:A:467:U:H3'	7:A:468:A:C5'	2.48	0.43
7:A:864:A:H4'	11:E:90:THR:HG23	2.00	0.43
7:A:924:C:H2'	7:A:925:G:H8	1.83	0.43
7:A:1428:A:H2'	7:A:1429:A:O4'	2.18	0.43
10:D:164:GLN:H	10:D:164:GLN:HG2	1.64	0.43
10:D:197:GLU:H	10:D:197:GLU:CD	2.24	0.43
13:G:36:LYS:HG2	15:I:41:ARG:NH2	2.33	0.43
15:I:44:ALA:O	15:I:48:VAL:HG13	2.19	0.43
18:L:122:PRO:C	18:L:124:ALA:N	2.76	0.43
19:M:7:ILE:HD11	19:M:22:ILE:HG13	2.01	0.43

Continued on next page...

For Manuscript Review

Continued from previous page...

Atom-1	Atom-2	Interatomic distance (Å)	Clash overlap (Å)
28:a:1442:U:H2'	28:a:1443:U:C6	2.54	0.43
28:a:1506:U:H2'	28:a:1507:C:C6	2.53	0.43
28:a:1589:U:H2'	28:a:1590:A:H8	1.83	0.43
28:a:2273:A:H2'	28:a:2274:A:C8	2.53	0.43
28:a:2315:G:H2'	28:a:2316:G:H8	1.83	0.43
28:a:2700:A:H2'	28:a:2701:U:H6	1.84	0.43
28:a:2804:U:H2'	28:a:2805:C:C6	2.53	0.43
41:n:21:LEU:HD23	41:n:21:LEU:HA	1.84	0.43
7:A:204:G:H5'	7:A:465:A:C2	2.54	0.43
7:A:459:A:N1	7:A:474:G:C6	2.87	0.43
7:A:696:A:H2'	7:A:697:U:H6	1.84	0.43
8:B:203:ASN:C	8:B:203:ASN:OD1	2.62	0.43
9:C:156:ARG:NH2	9:C:161:GLU:OE2	2.52	0.43
13:G:72:THR:O	13:G:91:VAL:HG22	2.19	0.43
13:G:130:ASN:HA	13:G:135:VAL:HG11	2.00	0.43
28:a:12:U:O2	28:a:2626:C:H4'	2.18	0.43
28:a:668:A:H2'	28:a:670:A:H62	1.83	0.43
28:a:1115:G:HO2'	28:a:1116:G:P	2.40	0.43
28:a:1319:C:O2'	28:a:1320:C:H5'	2.19	0.43
28:a:2373:G:H2'	28:a:2374:C:C6	2.54	0.43
29:b:49:C:H2'	29:b:50:A:C8	2.53	0.43
29:b:49:C:H2'	29:b:50:A:H8	1.83	0.43
29:b:66:A:N6	29:b:107:G:H2'	2.33	0.43
32:e:168:ASP:OD1	32:e:169:VAL:N	2.50	0.43
41:n:17:LYS:NZ	41:n:20:GLU:OE2	2.40	0.43
41:n:58:ILE:H	41:n:58:ILE:HD12	1.83	0.43
42:o:13:MET:HE3	42:o:77:HIS:CE1	2.54	0.43
48:u:79:ARG:HG2	48:u:79:ARG:HH11	1.83	0.43
8:B:43:LEU:H	8:B:43:LEU:HD22	1.84	0.43
10:D:48:LEU:CD2	10:D:56:ARG:HG3	2.42	0.43
14:H:39:VAL:O	14:H:43:GLU:HG2	2.18	0.43
17:K:50:SER:O	17:K:50:SER:OG	2.29	0.43
18:L:66:TYR:O	18:L:97:THR:HG23	2.19	0.43
19:M:37:ALA:HB2	19:M:59:GLU:OE1	2.19	0.43
20:N:37:SER:HB2	20:N:40:ASP:CB	2.49	0.43
27:U:10:GLU:CD	27:U:18:ARG:HH21	2.27	0.43
28:a:1:G:H2'	28:a:2:G:C8	2.54	0.43
28:a:597:G:O2'	38:k:11:GLY:O	2.28	0.43
28:a:619:G:OP2	28:a:620:G:N2	2.51	0.43
28:a:1529:G:H2'	28:a:1530:G:C8	2.53	0.43
28:a:1614:A:C6	45:r:87:PRO:HB3	2.54	0.43

Continued on next page...

For Manuscript Review

Continued from previous page...

Atom-1	Atom-2	Interatomic distance (Å)	Clash overlap (Å)
28:a:2813:A:H2'	28:a:2814:A:H8	1.82	0.43
38:k:141:LYS:HE2	38:k:141:LYS:HB3	1.79	0.43
7:A:90:C:H2'	7:A:90:C:O2	2.18	0.43
7:A:344:A:H5"	7:A:345:C:C5	2.51	0.43
7:A:452:A:O2'	22:P:73:ALA:HB1	2.18	0.43
7:A:542:G:H5'	10:D:39:GLY:HA2	2.00	0.43
7:A:1048:G:P	20:N:4:GLN:NE2	2.92	0.43
7:A:1293:C:H2'	7:A:1294:G:C8	2.54	0.43
7:A:1382:C:H4'	13:G:79:ARG:HH21	1.83	0.43
8:B:139:ARG:NE	8:B:139:ARG:HA	2.34	0.43
10:D:149:ALA:HB1	10:D:178:MET:HE2	2.01	0.43
15:I:41:ARG:O	15:I:45:ARG:HG2	2.19	0.43
17:K:34:ILE:HB	17:K:74:VAL:HG11	2.01	0.43
22:P:40:ASN:HB3	22:P:43:ALA:HB2	2.01	0.43
28:a:657:U:H2'	28:a:658:U:C6	2.54	0.43
28:a:976:G:HO2'	28:a:1155:A:HO2'	1.61	0.43
28:a:2542:A:H5"	28:a:2766:A:O2'	2.18	0.43
33:f:150:ARG:HH11	33:f:150:ARG:HG3	1.84	0.43
41:n:58:ILE:HD11	41:n:81:ARG:NH2	2.34	0.43
47:t:24:LYS:HE3	47:t:24:LYS:HB3	1.70	0.43
7:A:86:G:H1'	7:A:87:C:OP2	2.18	0.43
7:A:352:C:H4'	7:A:354:G:OP1	2.18	0.43
7:A:371:A:H2'	7:A:372:C:O4'	2.18	0.43
7:A:1217:C:OP2	20:N:9:ARG:NH2	2.51	0.43
7:A:1417:G:C6	7:A:1482:G:C6	3.06	0.43
9:C:118:ASP:O	9:C:121:THR:HG22	2.19	0.43
13:G:4:ARG:HA	13:G:4:ARG:HH11	1.83	0.43
13:G:15:ASP:OD2	13:G:44:TYR:OH	2.37	0.43
20:N:16:LEU:HD13	20:N:16:LEU:HA	1.72	0.43
20:N:38:ASP:CG	20:N:39:GLU:N	2.76	0.43
22:P:23:ASP:C	22:P:25:ARG:H	2.27	0.43
26:T:27:MET:SD	26:T:57:ILE:HG21	2.59	0.43
26:T:55:GLN:HA	26:T:58:VAL:HG22	2.01	0.43
28:a:743:A:OP1	31:d:135:GLY:HA2	2.18	0.43
28:a:947:A:H2'	28:a:948:C:C6	2.54	0.43
28:a:2491:U:H5'	28:a:2570:G:H5"	2.01	0.43
34:g:60:ASP:O	34:g:62:TRP:N	2.51	0.43
34:g:85:LYS:HE3	34:g:85:LYS:HB3	1.94	0.43
42:o:14:LYS:HD2	42:o:77:HIS:HA	2.00	0.43
7:A:90:C:O5'	7:A:90:C:H6	2.02	0.42
7:A:224:U:H2'	7:A:225:C:C6	2.53	0.42

Continued on next page...

For Manuscript Review

Continued from previous page...

Atom-1	Atom-2	Interatomic distance (Å)	Clash overlap (Å)
7:A:488:C:H4'	7:A:489:C:OP1	2.18	0.42
7:A:607:A:H5'	7:A:607:A:H8	1.82	0.42
7:A:860:A:H2'	7:A:861:G:O4'	2.19	0.42
7:A:923:A:H2'	7:A:924:C:H6	1.84	0.42
7:A:938:A:N6	7:A:939:G:C6	2.87	0.42
7:A:1312:G:H5'	25:S:5:LEU:HD22	2.00	0.42
12:F:17:GLN:O	12:F:21:MET:HG3	2.19	0.42
22:P:6:LEU:HD13	22:P:17:TYR:HB3	2.01	0.42
22:P:52:LEU:N	22:P:52:LEU:HD13	2.34	0.42
28:a:225:C:H2'	28:a:226:A:O4'	2.19	0.42
28:a:367:G:H2'	28:a:368:A:C8	2.54	0.42
28:a:876:C:H2'	28:a:877:A:O4'	2.19	0.42
28:a:914:G:H5'	28:a:915:C:OP2	2.19	0.42
40:m:24:MET:HE1	40:m:40:LYS:HB3	2.01	0.42
45:r:92:ARG:HE	45:r:92:ARG:HB3	1.62	0.42
2:1:26:ASN:CG	28:a:682:G:H5'	2.44	0.42
5:4:56:ARG:HG3	25:S:65:GLU:HA	2.01	0.42
7:A:246:A:C2	7:A:282:A:C5	3.06	0.42
7:A:837:U:H2'	7:A:838:G:H8	1.84	0.42
7:A:1004:A:C5'	7:A:1024:G:H22	2.31	0.42
7:A:1034:G:O2'	7:A:1035:A:H5'	2.18	0.42
7:A:1119:C:H2'	7:A:1120:C:H6	1.83	0.42
7:A:1147:C:H2'	7:A:1148:U:H6	1.84	0.42
7:A:1360:A:H2'	7:A:1361:G:C8	2.54	0.42
11:E:115:LEU:HD13	11:E:123:VAL:HG11	2.00	0.42
12:F:5:GLU:OE2	12:F:38:ARG:NH2	2.51	0.42
14:H:87:LYS:HG3	14:H:91:GLU:HG3	2.01	0.42
27:U:31:GLU:OE2	27:U:35:ARG:NE	2.36	0.42
28:a:598:U:H2'	28:a:599:A:C8	2.54	0.42
28:a:1000:A:H2'	28:a:1001:A:C8	2.54	0.42
28:a:1734:G:H2'	28:a:1735:A:C8	2.54	0.42
28:a:1774:C:O2	28:a:1774:C:H2'	2.19	0.42
28:a:1790:C:H2'	28:a:1791:A:C5	2.54	0.42
31:d:184:ARG:CZ	42:o:7:GLN:HE22	2.32	0.42
33:f:164:GLU:HG2	33:f:165:GLU:N	2.34	0.42
5:4:16:CYS:SG	5:4:17:SER:N	2.93	0.42
5:4:36:VAL:HG21	5:4:41:HIS:HD2	1.83	0.42
7:A:361:G:H2'	7:A:362:G:O4'	2.20	0.42
7:A:503:C:H2'	7:A:504:C:H6	1.83	0.42
7:A:1130:A:H2'	7:A:1131:G:C8	2.50	0.42
7:A:1147:C:H1'	15:I:18:ARG:HH12	1.85	0.42

Continued on next page...

For Manuscript Review

Continued from previous page...

Atom-1	Atom-2	Interatomic distance (Å)	Clash overlap (Å)
7:A:1382:C:H2'	7:A:1383:C:C6	2.55	0.42
9:C:5:VAL:HG21	9:C:10:ILE:HD11	2.01	0.42
10:D:21:LEU:HD23	10:D:21:LEU:HA	1.86	0.42
22:P:11:ALA:O	22:P:12:LYS:C	2.62	0.42
28:a:277:G:H1'	28:a:278:A:C5	2.55	0.42
28:a:364:C:H2'	28:a:365:U:C6	2.54	0.42
28:a:570:G:H2'	28:a:2030:6MZ:N7	2.35	0.42
28:a:1115:G:N3	28:a:1116:G:C8	2.87	0.42
28:a:1125:G:OP2	28:a:1126:A:O2'	2.29	0.42
28:a:1824:G:O3'	30:c:247:PRO:HD3	2.20	0.42
28:a:2233:U:H2'	28:a:2234:G:H8	1.83	0.42
28:a:2411:A:H2'	28:a:2412:A:C8	2.55	0.42
28:a:2786:U:H2'	28:a:2787:C:C6	2.53	0.42
33:f:121:SER:HB3	33:f:128:TYR:CE1	2.54	0.42
38:k:89:VAL:O	38:k:94:THR:HG21	2.19	0.42
41:n:13:ARG:HG2	41:n:13:ARG:NH1	2.33	0.42
7:A:45:G:H2'	7:A:46:G:C8	2.54	0.42
7:A:59:A:H1'	7:A:354:G:N2	2.35	0.42
7:A:441:A:O5'	7:A:441:A:H8	2.01	0.42
7:A:619:U:H4'	10:D:128:ARG:HH22	1.84	0.42
7:A:1252:A:H2'	7:A:1253:G:O4'	2.19	0.42
9:C:79:LYS:HB3	9:C:82:GLU:HG2	2.01	0.42
10:D:107:PHE:HB3	10:D:145:ILE:HD11	1.99	0.42
11:E:81:LEU:HB2	11:E:98:PRO:HG3	2.01	0.42
18:L:10:LYS:HB3	18:L:10:LYS:HE3	1.79	0.42
21:O:64:ARG:HH22	21:O:89:ARG:C	2.28	0.42
22:P:79:ASN:C	22:P:80:LYS:HD3	2.44	0.42
26:T:79:LEU:O	26:T:83:ILE:HG12	2.20	0.42
28:a:534:U:H2'	28:a:535:G:H8	1.84	0.42
28:a:677:A:OP1	56:a:6219:SPD:N6	2.52	0.42
28:a:722:A:H2'	28:a:723:C:C6	2.54	0.42
28:a:967:U:H2'	28:a:968:C:C6	2.54	0.42
28:a:1013:C:H2'	28:a:1014:A:C8	2.54	0.42
28:a:1295:C:H2'	28:a:1296:G:H8	1.85	0.42
28:a:2641:G:H5"	36:i:78:THR:HB	2.02	0.42
28:a:2700:A:H2'	28:a:2701:U:C6	2.54	0.42
35:h:4:ILE:HD12	35:h:4:ILE:O	2.19	0.42
37:j:114:LYS:HE3	37:j:114:LYS:HB3	1.85	0.42
43:p:50:ARG:O	43:p:54:LYS:NZ	2.45	0.42
7:A:37:U:H4'	7:A:501:C:OP1	2.19	0.42
7:A:85:U:H4'	7:A:86:G:N2	2.33	0.42

Continued on next page...

For Manuscript Review

Continued from previous page...

Atom-1	Atom-2	Interatomic distance (Å)	Clash overlap (Å)
7:A:124:C:H4'	7:A:291:U:O2'	2.20	0.42
7:A:180:U:H2'	7:A:181:A:H5'	2.02	0.42
7:A:458:U:C2	7:A:459:A:N7	2.87	0.42
7:A:515:G:C6	7:A:516:PSU:C2	3.07	0.42
7:A:728:A:H2'	7:A:729:A:C8	2.55	0.42
7:A:1277:C:C2'	7:A:1278:G:H5"	2.49	0.42
7:A:1282:C:H2'	7:A:1283:U:C6	2.54	0.42
7:A:1335:U:H5'	7:A:1336:C:H5"	2.01	0.42
9:C:130:PHE:O	9:C:134:MET:HG3	2.19	0.42
22:P:23:ASP:O	22:P:24:SER:C	2.63	0.42
28:a:645:C:H2'	28:a:647:G:C8	2.54	0.42
28:a:1117:C:H2'	28:a:1118:C:C6	2.55	0.42
28:a:1361:G:H2'	28:a:1362:C:C6	2.55	0.42
28:a:1827:U:OP2	30:c:221:ARG:HD2	2.19	0.42
28:a:2065:C:H2'	28:a:2066:C:H6	1.84	0.42
28:a:2529:G:H5'	34:g:175:LYS:HB3	2.01	0.42
45:r:28:LYS:HE3	45:r:28:LYS:HB3	1.82	0.42
2:1:34:ARG:CZ	2:1:39:ARG:HD3	2.50	0.42
3:2:4:ILE:HD11	28:a:592:A:C2	2.55	0.42
7:A:404:G:C2	7:A:405:U:C2	3.08	0.42
7:A:409:U:C4	7:A:410:G:C5	3.08	0.42
7:A:422:C:H1'	7:A:423:G:N2	2.34	0.42
7:A:584:G:H2'	7:A:585:G:H8	1.84	0.42
7:A:923:A:H2'	7:A:924:C:C6	2.54	0.42
7:A:1004:A:C6	7:A:1026:G:H1'	2.55	0.42
7:A:1006:G:H2'	7:A:1007:U:C6	2.55	0.42
7:A:1394:A:N1	7:A:1500:A:O2'	2.48	0.42
7:A:1508:A:H2'	7:A:1509:C:O4'	2.19	0.42
22:P:39:PHE:CB	22:P:74:LEU:HD11	2.44	0.42
23:Q:31:HIS:HE1	23:Q:33:ILE:HD12	1.85	0.42
25:S:36:ARG:HE	25:S:36:ARG:HB3	1.71	0.42
28:a:163:C:H2'	28:a:164:C:H6	1.83	0.42
28:a:2849:U:H4'	28:a:2868:A:C2	2.55	0.42
29:b:66:A:H61	29:b:107:G:H2'	1.85	0.42
31:d:133:THR:O	31:d:134:HIS:HB2	2.20	0.42
32:e:83:VAL:HB	32:e:86:ALA:HB2	2.02	0.42
7:A:57:G:H2'	7:A:58:C:H6	1.83	0.42
7:A:73:C:O2'	7:A:74:A:H5'	2.19	0.42
7:A:315:A:H5"	7:A:317:U:OP2	2.19	0.42
7:A:377:G:H2'	7:A:378:G:H8	1.85	0.42
7:A:390:U:H4'	22:P:28:ARG:NH2	2.35	0.42

Continued on next page...

For Manuscript Review

Continued from previous page...

Atom-1	Atom-2	Interatomic distance (Å)	Clash overlap (Å)
7:A:737:C:H2'	7:A:738:C:C6	2.54	0.42
7:A:1315:U:O2'	7:A:1360:A:O2'	2.25	0.42
9:C:56:VAL:O	9:C:66:VAL:HA	2.20	0.42
10:D:72:PHE:CE1	10:D:94:LEU:HD11	2.55	0.42
10:D:163:GLU:OE1	10:D:163:GLU:HA	2.20	0.42
10:D:190:ASP:OD1	10:D:190:ASP:N	2.53	0.42
18:L:114:ARG:C	18:L:116:LYS:N	2.78	0.42
25:S:36:ARG:HD3	25:S:52:HIS:O	2.19	0.42
28:a:286:U:C2	28:a:287:G:C8	3.08	0.42
28:a:634:C:H2'	28:a:635:C:H6	1.85	0.42
28:a:1405:U:H2'	28:a:1406:U:H6	1.85	0.42
28:a:1799:G:N7	30:c:178:SER:OG	2.51	0.42
28:a:2074:U:H2'	28:a:2075:U:C6	2.55	0.42
36:i:12:LYS:HD2	36:i:12:LYS:HA	1.78	0.42
44:q:33:VAL:HG13	44:q:61:ALA:HB3	2.01	0.42
7:A:80:A:C2	7:A:81:A:C5	3.06	0.42
7:A:90:C:H2'	7:A:91:U:C4	2.54	0.42
7:A:161:A:H2'	7:A:162:A:C8	2.55	0.42
7:A:612:C:C4	7:A:613:C:N4	2.88	0.42
7:A:737:C:H2'	7:A:738:C:H6	1.84	0.42
7:A:891:U:H2'	7:A:892:A:H8	1.83	0.42
7:A:1035:A:O2'	7:A:1036:A:H5"	2.19	0.42
8:B:203:ASN:OD1	8:B:204:ASP:N	2.53	0.42
12:F:47:LEU:HD13	12:F:51:ILE:HD12	2.02	0.42
13:G:74:GLU:OE2	13:G:95:ARG:NE	2.43	0.42
13:G:135:VAL:O	13:G:139:GLU:HG2	2.20	0.42
16:J:11:LYS:HE3	16:J:71:LEU:HD21	2.00	0.42
19:M:90:ARG:HH11	19:M:90:ARG:HG3	1.85	0.42
20:N:50:THR:C	20:N:51:LEU:O	2.62	0.42
26:T:55:GLN:N	26:T:56:PRO:HD2	2.34	0.42
28:a:574:A:N6	28:a:2034:U:OP1	2.51	0.42
28:a:747:5MU:O2	28:a:2014:A:H1'	2.20	0.42
28:a:2251:OMG:HM23	28:a:2251:OMG:H1'	1.72	0.42
28:a:2290:G:H2'	28:a:2291:U:C6	2.55	0.42
28:a:2532:G:N2	28:a:2663:G:O2'	2.53	0.42
35:h:3:VAL:CG2	35:h:36:ALA:HB1	2.50	0.42
46:s:30:ILE:HG13	46:s:85:VAL:HB	2.02	0.42
4:3:6:SER:O	4:3:6:SER:OG	2.37	0.42
7:A:21:G:H2'	7:A:22:G:C8	2.54	0.42
7:A:82:G:H5"	7:A:83:C:O2	2.20	0.42
7:A:88:U:H3'	7:A:89:U:H6	1.84	0.42

Continued on next page...

For Manuscript Review

Continued from previous page...

Atom-1	Atom-2	Interatomic distance (Å)	Clash overlap (Å)
7:A:234:C:H2’	7:A:235:C:H6	1.85	0.42
7:A:1121:U:H2’	7:A:1122:U:H6	1.84	0.42
7:A:1436:U:C2	7:A:1437:A:C8	3.08	0.42
7:A:1509:C:C2’	7:A:1510:C:H5’	2.50	0.42
21:O:6:GLU:H	21:O:6:GLU:CD	2.28	0.42
23:Q:15:ASP:OD2	23:Q:54:GLY:HA2	2.18	0.42
23:Q:61:ILE:HG22	23:Q:73:TRP:CE3	2.54	0.42
26:T:69:LYS:HE3	26:T:69:LYS:HB3	1.80	0.42
28:a:3:U:H2’	28:a:4:U:C6	2.55	0.42
28:a:347:A:H2’	28:a:348:A:C8	2.55	0.42
28:a:589:U:H2’	28:a:590:A:H8	1.84	0.42
28:a:1348:C:C5	28:a:1349:C:C5	3.08	0.42
28:a:1387:A:H5’	28:a:1469:A:H1’	2.00	0.42
28:a:2314:A:H2’	28:a:2315:G:H8	1.83	0.42
28:a:2585:U:O2’	28:a:2586:U:H5’	2.20	0.42
29:b:115:A:H2’	29:b:116:G:C8	2.55	0.42
37:j:101:GLY:O	37:j:120:PRO:HD2	2.20	0.42
53:z:31:ASP:OD1	53:z:31:ASP:C	2.63	0.42
4:3:12:ARG:NH1	4:3:12:ARG:HB3	2.35	0.42
7:A:60:A:N1	7:A:107:G:O2’	2.40	0.42
7:A:110:C:H2’	7:A:111:G:O4’	2.19	0.42
7:A:1053:G:N7	7:A:1200:C:H5’	2.35	0.42
7:A:1073:U:C2	7:A:1074:G:C8	3.07	0.42
7:A:1118:U:H1’	7:A:1179:A:N7	2.35	0.42
7:A:1180:A:OP2	15:I:99:ARG:NH2	2.53	0.42
7:A:1465:A:H2’	7:A:1466:C:C6	2.54	0.42
7:A:1530:G:O2’	7:A:1533:C:N4	2.53	0.42
8:B:64:LYS:HB2	8:B:64:LYS:HE2	1.55	0.42
17:K:113:VAL:HG12	24:R:73:ARG:HD2	2.02	0.42
25:S:7:LYS:HE2	25:S:7:LYS:HA	2.01	0.42
28:a:720:U:H2’	28:a:721:A:C8	2.54	0.42
28:a:1009:A:N3	28:a:1153:C:O2’	2.49	0.42
28:a:1798:U:OP2	30:c:271:ARG:NH1	2.47	0.42
28:a:2026:U:H2’	28:a:2027:G:O4’	2.20	0.42
28:a:2333:A:OP2	49:v:77:ARG:NH2	2.32	0.42
28:a:2554:U:H2’	28:a:2555:U:C6	2.55	0.42
28:a:2728:U:O2’	28:a:2729:G:H8	2.03	0.42
28:a:2865:U:O4	56:a:6209:SPD:H22	2.20	0.42
41:n:63:LYS:HD2	41:n:63:LYS:C	2.44	0.42
43:p:19:LYS:HZ2	43:p:19:LYS:HG2	1.76	0.42
7:A:404:G:N2	7:A:405:U:C2	2.87	0.41

Continued on next page...

For Manuscript Review

Continued from previous page...

Atom-1	Atom-2	Interatomic distance (Å)	Clash overlap (Å)
7:A:490:C:H2’	7:A:491:G:C8	2.52	0.41
7:A:505:G:H2’	7:A:506:G:C8	2.55	0.41
7:A:613:C:HO2’	7:A:614:C:H5	1.66	0.41
7:A:677:U:O2	7:A:777:A:O2’	2.32	0.41
7:A:1165:U:H2’	7:A:1166:G:O4’	2.20	0.41
7:A:1233:G:H2’	7:A:1234:C:C6	2.55	0.41
7:A:1308:U:OP1	19:M:97:VAL:HG22	2.20	0.41
7:A:1366:C:H2’	7:A:1367:C:C6	2.54	0.41
12:F:42:TRP:HB2	12:F:59:TYR:HB2	2.02	0.41
14:H:26:THR:HA	14:H:59:LEU:O	2.20	0.41
17:K:67:ALA:HB1	17:K:100:LEU:HD22	2.02	0.41
19:M:85:CYS:HB2	25:S:73:GLU:HB3	2.01	0.41
28:a:39:G:H2’	28:a:40:U:C6	2.55	0.41
28:a:729:G:C6	30:c:207:LYS:HB2	2.55	0.41
28:a:1656:C:H2’	28:a:1657:U:H6	1.84	0.41
28:a:1735:A:H2’	28:a:1736:U:C6	2.55	0.41
29:b:39:A:H2’	29:b:40:U:C6	2.55	0.41
30:c:35:GLU:HG3	30:c:64:ILE:HD11	2.02	0.41
33:f:135:GLN:HG2	33:f:150:ARG:HB2	2.00	0.41
46:s:71:GLY:O	46:s:72:GLN:HG2	2.20	0.41
47:t:39:ILE:HG22	47:t:40:ASN:N	2.35	0.41
50:w:41:GLU:HA	50:w:41:GLU:OE1	2.19	0.41
2:1:40:ALA:N	28:a:459:U:OP1	2.50	0.41
3:2:14:PHE:C	3:2:15:LYS:HD2	2.44	0.41
7:A:2:A:C6	7:A:3:A:C2	3.08	0.41
7:A:81:A:N6	7:A:87:C:H42	2.18	0.41
7:A:427:U:H4’	7:A:541:G:H5”	2.02	0.41
7:A:1023:U:C2	7:A:1024:G:C8	3.08	0.41
7:A:1151:A:HO2’	7:A:1152:A:H8	1.63	0.41
7:A:1485:U:H2’	7:A:1486:G:H8	1.85	0.41
8:B:27:MET:HG2	8:B:189:THR:HA	2.02	0.41
10:D:19:LEU:HD22	10:D:64:ILE:HG13	2.02	0.41
11:E:12:GLN:HG2	11:E:117:VAL:HG12	2.02	0.41
15:I:84:THR:HG21	15:I:103:PHE:HB3	2.01	0.41
15:I:115:LYS:HD3	15:I:115:LYS:HA	1.83	0.41
19:M:89:LEU:HD23	19:M:89:LEU:HA	1.93	0.41
23:Q:28:PHE:CE1	23:Q:39:LYS:HG3	2.54	0.41
25:S:19:VAL:HG21	25:S:44:MET:HG2	2.02	0.41
28:a:373:U:O2’	28:a:423:A:H1’	2.20	0.41
28:a:391:A:H1’	28:a:411:G:O4’	2.20	0.41
28:a:711:G:C6	28:a:721:A:C6	3.08	0.41

Continued on next page...

For Manuscript Review

Continued from previous page...

Atom-1	Atom-2	Interatomic distance (Å)	Clash overlap (Å)
28:a:729:G:H4’	28:a:763:G:O5’	2.20	0.41
28:a:892:A:H2’	28:a:893:C:C6	2.54	0.41
28:a:1186:G:H2’	28:a:1187:G:O4’	2.20	0.41
28:a:1370:C:H2’	28:a:1371:G:O4’	2.20	0.41
28:a:1532:A:H2’	28:a:1533:C:H6	1.85	0.41
28:a:2271:G:OP1	49:v:18:ALA:HB1	2.20	0.41
28:a:2411:A:H2’	28:a:2412:A:H8	1.85	0.41
28:a:2590:A:H2’	28:a:2591:C:H6	1.85	0.41
28:a:2808:G:O2’	28:a:2890:G:O6	2.28	0.41
28:a:2838:G:C4	28:a:2839:G:C8	3.08	0.41
28:a:2847:U:H2’	28:a:2848:G:O4’	2.19	0.41
30:c:98:ASP:C	30:c:98:ASP:OD1	2.63	0.41
7:A:78:A:C5	7:A:92:U:N3	2.88	0.41
7:A:188:C:H2’	7:A:189:A:O4’	2.20	0.41
7:A:618:C:N4	7:A:621:A:OP2	2.54	0.41
7:A:919:A:O2’	7:A:1080:A:N1	2.50	0.41
7:A:1147:C:H2’	7:A:1148:U:C6	2.55	0.41
7:A:1477:U:H2’	7:A:1478:U:C6	2.54	0.41
7:A:1510:C:O2’	7:A:1511:G:H8	2.03	0.41
8:B:76:ALA:O	8:B:80:VAL:HG12	2.20	0.41
8:B:81:LYS:HG3	8:B:91:PHE:CE2	2.56	0.41
8:B:147:SER:HB3	8:B:148:LEU:HD12	2.02	0.41
9:C:58:GLU:OE1	9:C:59:ARG:N	2.53	0.41
10:D:21:LEU:C	10:D:23:SER:N	2.75	0.41
13:G:99:LEU:HA	13:G:99:LEU:HD23	1.83	0.41
17:K:94:GLU:HA	17:K:94:GLU:OE1	2.20	0.41
23:Q:60:GLU:HB2	23:Q:77:ARG:HG2	2.02	0.41
28:a:277:G:H4’	28:a:278:A:O5’	2.21	0.41
28:a:886:A:H2’	28:a:887:U:O4’	2.20	0.41
28:a:1182:G:H2’	28:a:1183:U:O4’	2.20	0.41
30:c:132:MET:HG2	30:c:164:ILE:HD11	2.03	0.41
45:r:83:LYS:HB3	45:r:95:ARG:HD3	2.01	0.41
48:u:44:HIS:CE1	48:u:48:MET:HG3	2.54	0.41
51:x:14:LEU:HB3	51:x:57:LEU:HD21	2.01	0.41
7:A:113:G:H2’	7:A:114:U:C6	2.55	0.41
7:A:190:A:H8	7:A:190:A:O5’	2.03	0.41
7:A:1219:A:H2’	7:A:1220:G:C8	2.55	0.41
7:A:1326:U:H2’	7:A:1327:C:C6	2.55	0.41
8:B:37:LYS:H	8:B:37:LYS:HE2	1.85	0.41
8:B:123:ASP:OD2	8:B:123:ASP:C	2.63	0.41
8:B:173:ILE:HG22	8:B:174:LYS:HD2	2.01	0.41

Continued on next page...

For Manuscript Review

Continued from previous page...

Atom-1	Atom-2	Interatomic distance (Å)	Clash overlap (Å)
11:E:162:GLU:OE1	11:E:162:GLU:HA	2.21	0.41
12:F:46:GLN:HA	12:F:56:LYS:HD3	2.02	0.41
22:P:1:MET:O	22:P:3:THR:N	2.53	0.41
23:Q:11:ARG:HG3	23:Q:11:ARG:NH1	2.35	0.41
28:a:397:U:H5"	50:w:32:ASN:HB2	2.02	0.41
28:a:1019:U:O2'	28:a:1021:A:N7	2.52	0.41
28:a:1394:U:H4'	28:a:1603:A:H4'	2.02	0.41
28:a:1469:A:H2'	28:a:1470:A:H8	1.85	0.41
28:a:1710:G:H4'	28:a:2858:C:O2	2.20	0.41
56:a:6209:SPD:H101	56:a:6209:SPD:H72	1.59	0.41
29:b:29:A:H2'	29:b:30:C:C6	2.56	0.41
31:d:14:ILE:HA	42:o:12:GLN:HE22	1.84	0.41
33:f:42:GLU:C	33:f:42:GLU:OE1	2.63	0.41
50:w:54:LYS:O	50:w:58:VAL:HG23	2.20	0.41
51:x:18:LEU:HB2	51:x:53:VAL:HG11	2.02	0.41
7:A:455:G:C2	7:A:478:A:C2	3.09	0.41
7:A:496:A:C2	7:A:497:G:C5	3.08	0.41
7:A:825:A:H2'	7:A:826:C:C6	2.56	0.41
7:A:921:U:O2	11:E:24:THR:HB	2.20	0.41
7:A:1004:A:H5'	7:A:1024:G:H22	1.85	0.41
7:A:1111:A:H2'	7:A:1112:C:C6	2.56	0.41
12:F:11:HIS:CD2	12:F:54:LEU:HD21	2.55	0.41
13:G:97:ASN:O	13:G:101:MET:HG3	2.20	0.41
15:I:55:VAL:HG11	15:I:87:LEU:HD13	2.03	0.41
28:a:215:G:H4'	28:a:216:A:H4'	2.02	0.41
28:a:479:A:H4'	28:a:480:A:OP1	2.21	0.41
28:a:1418:G:N2	28:a:1579:A:N7	2.69	0.41
56:a:6217:SPD:H72	45:r:87:PRO:O	2.20	0.41
30:c:43:ARG:HA	30:c:48:ARG:O	2.20	0.41
31:d:19:GLY:HA3	42:o:80:VAL:HG23	2.03	0.41
39:l:18:ARG:NH1	39:l:18:ARG:HB3	2.35	0.41
45:r:13:SER:O	45:r:17:VAL:HG23	2.20	0.41
7:A:5:U:H5'	7:A:6:G:C5	2.56	0.41
7:A:504:C:C2	7:A:542:G:N2	2.88	0.41
7:A:511:C:O4'	10:D:41:HIS:NE2	2.53	0.41
7:A:1347:G:N7	15:I:13:LYS:HE2	2.36	0.41
8:B:217:VAL:O	8:B:220:THR:HG22	2.20	0.41
9:C:112:ASP:OD2	9:C:112:ASP:C	2.63	0.41
11:E:111:MET:HE2	11:E:111:MET:HB3	1.79	0.41
12:F:10:VAL:HG11	12:F:18:VAL:HG12	2.02	0.41
15:I:84:THR:O	15:I:88:MET:HE2	2.21	0.41

Continued on next page...

For Manuscript Review

Continued from previous page...

Atom-1	Atom-2	Interatomic distance (Å)	Clash overlap (Å)
18:L:52:VAL:HG11	18:L:90:LEU:HD13	2.01	0.41
23:Q:53:CYS:SG	23:Q:75:LEU:HD11	2.60	0.41
25:S:16:LEU:O	25:S:19:VAL:HG12	2.21	0.41
28:a:4:U:H2'	28:a:5:A:C8	2.56	0.41
28:a:81:G:H2'	28:a:82:U:O4'	2.19	0.41
28:a:340:A:H2'	28:a:341:C:O4'	2.21	0.41
28:a:1526:C:H2'	28:a:1527:G:O4'	2.20	0.41
28:a:2052:A:H4'	31:d:148:GLN:O	2.19	0.41
32:e:145:ASP:HB3	32:e:184:ASP:HB2	2.01	0.41
7:A:36:C:H2'	7:A:37:U:O4'	2.21	0.41
7:A:46:G:O2'	7:A:365:U:H1'	2.21	0.41
7:A:176:C:O2'	7:A:177:G:H5"	2.21	0.41
7:A:408:A:OP1	10:D:112:ALA:HB3	2.21	0.41
7:A:590:U:H2'	7:A:591:U:C6	2.54	0.41
7:A:671:G:H2'	7:A:672:U:C6	2.56	0.41
7:A:1380:U:H4'	7:A:1381:U:C5	2.54	0.41
8:B:7:ARG:NH2	8:B:11:LYS:HE2	2.36	0.41
11:E:39:VAL:HG23	11:E:71:MET:SD	2.61	0.41
16:J:6:ILE:HB	16:J:76:ILE:CG2	2.51	0.41
19:M:39:ILE:HD12	19:M:39:ILE:C	2.45	0.41
28:a:152:A:H2'	28:a:153:U:C6	2.56	0.41
28:a:766:U:H2'	28:a:767:U:C6	2.55	0.41
28:a:1007:C:H5"	36:i:37:ARG:HH11	1.86	0.41
28:a:1689:A:H2'	28:a:1690:A:C8	2.56	0.41
28:a:2295:C:OP2	41:n:9:ARG:NH2	2.51	0.41
28:a:2389:G:H5"	28:a:2390:U:O4'	2.21	0.41
29:b:78:A:H2'	29:b:79:G:O4'	2.21	0.41
31:d:25:THR:HG21	31:d:193:VAL:HG22	2.03	0.41
34:g:39:ASP:OD1	34:g:58:TYR:OH	2.37	0.41
47:t:81:ASP:OD1	47:t:96:PHE:HB3	2.20	0.41
7:A:462:G:C5	7:A:463:U:C4	3.09	0.41
7:A:502:A:H2'	7:A:503:C:O4'	2.21	0.41
7:A:1004:A:C4	7:A:1026:G:C5	3.08	0.41
7:A:1030:U:O4	7:A:1033:G:C4	2.74	0.41
7:A:1305:G:HO2'	7:A:1306:A:P	2.43	0.41
13:G:110:LYS:HD3	13:G:110:LYS:HA	1.60	0.41
17:K:126:LYS:HD2	27:U:37:PHE:HB2	2.03	0.41
25:S:36:ARG:HH12	25:S:77:THR:HG22	1.84	0.41
28:a:64:A:H2'	28:a:65:U:H6	1.85	0.41
28:a:231:A:H2'	28:a:232:G:O4'	2.20	0.41
28:a:306:U:H2'	28:a:307:G:O4'	2.21	0.41

Continued on next page...

For Manuscript Review

Continued from previous page...

Atom-1	Atom-2	Interatomic distance (Å)	Clash overlap (Å)
28:a:414:C:H1'	28:a:1864:U:H1'	2.03	0.41
28:a:491:G:O6	45:r:49:LYS:NZ	2.40	0.41
28:a:640:C:H2'	28:a:641:U:C6	2.55	0.41
28:a:1773:A:C8	28:a:1829:A:C8	3.09	0.41
36:i:92:MET:HE3	36:i:92:MET:HB3	1.93	0.41
38:k:55:MET:HG3	38:k:56:PRO:HD2	2.03	0.41
42:o:92:VAL:HG21	42:o:97:LEU:HD21	2.03	0.41
46:s:24:MET:HE2	46:s:30:ILE:HG23	2.02	0.41
3:2:15:LYS:HD3	38:k:64:PHE:CZ	2.56	0.41
7:A:392:C:C2	7:A:393:A:C8	3.08	0.41
7:A:621:A:H2'	7:A:622:A:C8	2.56	0.41
7:A:952:U:H2'	7:A:953:G:H8	1.86	0.41
7:A:971:G:H1'	7:A:1365:G:O2'	2.20	0.41
7:A:1142:G:C4	7:A:1143:G:H1'	2.55	0.41
7:A:1382:C:O5'	7:A:1382:C:H6	2.04	0.41
8:B:71:GLY:O	8:B:93:ASN:HA	2.21	0.41
8:B:109:GLN:H	8:B:109:GLN:HG2	1.67	0.41
9:C:167:TRP:HZ3	9:C:169:ARG:HG2	1.86	0.41
10:D:26:ARG:NH2	10:D:30:THR:O	2.54	0.41
10:D:147:GLU:C	10:D:149:ALA:H	2.27	0.41
14:H:42:GLU:HG2	14:H:42:GLU:H	1.71	0.41
15:I:30:ILE:HG12	15:I:65:ILE:HB	2.02	0.41
15:I:114:LYS:HG2	15:I:115:LYS:H	1.86	0.41
16:J:7:ARG:HD3	16:J:75:ASP:OD1	2.20	0.41
16:J:24:GLU:C	16:J:24:GLU:OE1	2.64	0.41
19:M:89:LEU:HD23	19:M:92:ARG:HD2	2.03	0.41
20:N:19:LYS:HE3	20:N:19:LYS:HB3	1.85	0.41
22:P:2:VAL:HG23	22:P:21:VAL:CG1	2.51	0.41
23:Q:15:ASP:HA	23:Q:21:ILE:HG22	2.02	0.41
28:a:651:G:C2	28:a:652:U:C6	3.09	0.41
28:a:716:A:H2'	28:a:717:C:O4'	2.21	0.41
28:a:1224:U:H2'	28:a:1225:G:C8	2.56	0.41
28:a:1366:A:H2'	28:a:1367:A:O4'	2.21	0.41
28:a:1450:G:O2'	28:a:1451:C:H5'	2.21	0.41
28:a:1532:A:H2'	28:a:1533:C:C6	2.56	0.41
28:a:2051:A:H5'	28:a:2578:G:O4'	2.21	0.41
28:a:2636:C:H2'	28:a:2637:U:C6	2.56	0.41
28:a:2703:C:C2	28:a:2704:C:C5	3.08	0.41
28:a:2820:A:N6	31:d:197:THR:HB	2.35	0.41
32:e:163:ASN:OD1	32:e:163:ASN:O	2.39	0.41
33:f:34:ILE:HD13	33:f:156:ILE:HA	2.02	0.41

Continued on next page...

For Manuscript Review

Continued from previous page...

Atom-1	Atom-2	Interatomic distance (Å)	Clash overlap (Å)
34:g:27:LYS:HD2	34:g:28:GLY:N	2.36	0.41
38:k:27:LEU:O	38:k:31:GLY:HA2	2.21	0.41
40:m:4:ARG:HH11	40:m:4:ARG:HG2	1.86	0.41
44:q:31:GLU:N	44:q:31:GLU:CD	2.78	0.41
52:y:19:LYS:HB2	52:y:19:LYS:HE2	1.91	0.41
53:z:12:LYS:HD3	53:z:12:LYS:HA	1.84	0.41
7:A:355:C:O2’	7:A:388:G:N3	2.43	0.41
7:A:836:G:H1	7:A:850:U:H3	1.68	0.41
7:A:999:C:H2’	7:A:1000:A:C8	2.56	0.41
7:A:1130:A:C8	7:A:1146:A:N1	2.89	0.41
7:A:1245:C:H2’	7:A:1246:A:C8	2.56	0.41
7:A:1405:G:H2’	7:A:1406:U:H6	1.86	0.41
7:A:1526:G:H2’	7:A:1527:U:C6	2.56	0.41
10:D:28:ILE:HG21	10:D:34:ILE:CD1	2.38	0.41
13:G:5:ARG:O	13:G:5:ARG:HG3	2.21	0.41
17:K:95:SER:O	17:K:98:ARG:HG2	2.21	0.41
18:L:86:ARG:HA	18:L:94:ARG:HA	2.03	0.41
19:M:81:MET:O	19:M:92:ARG:NH2	2.46	0.41
20:N:28:LYS:O	20:N:29:ALA:C	2.64	0.41
22:P:6:LEU:HD23	22:P:6:LEU:HA	1.93	0.41
22:P:67:ILE:CG2	22:P:71:VAL:HG13	2.51	0.41
25:S:5:LEU:HA	25:S:5:LEU:HD12	1.78	0.41
26:T:57:ILE:HD12	26:T:57:ILE:N	2.35	0.41
28:a:208:C:H2’	28:a:209:C:C6	2.55	0.41
28:a:296:U:H2’	28:a:297:G:C8	2.56	0.41
28:a:870:U:OP1	39:l:6:ARG:NH1	2.54	0.41
28:a:1473:G:C6	28:a:1519:G:C6	3.09	0.41
28:a:2588:G:O6	28:a:2607:G:C6	2.74	0.41
34:g:98:VAL:HG23	34:g:103:ILE:HG12	2.03	0.41
35:h:29:PHE:C	35:h:32:PRO:HD2	2.45	0.41
37:j:53:LYS:NZ	37:j:56:ASP:OD1	2.55	0.41
50:w:3:ARG:O	50:w:12:PRO:HD3	2.20	0.41
1:0:11:LEU:HB3	1:0:49:TYR:HB3	2.03	0.40
7:A:335:C:O2’	7:A:1433:A:N3	2.48	0.40
7:A:415:A:H2’	7:A:416:G:H8	1.85	0.40
7:A:1026:G:HO2’	7:A:1027:C:P	2.43	0.40
7:A:1036:A:H2’	7:A:1037:C:O4’	2.19	0.40
7:A:1166:G:O2’	7:A:1169:A:N6	2.54	0.40
8:B:100:MET:HB2	8:B:100:MET:HE2	1.84	0.40
9:C:66:VAL:HB	9:C:101:ILE:HD13	2.03	0.40
11:E:146:ASN:OD1	11:E:146:ASN:C	2.63	0.40

Continued on next page...

For Manuscript Review

Continued from previous page...

Atom-1	Atom-2	Interatomic distance (Å)	Clash overlap (Å)
16:J:88:MET:HE2	16:J:88:MET:HB2	1.89	0.40
19:M:83:LEU:HD21	25:S:65:GLU:HB2	2.02	0.40
22:P:39:PHE:O	22:P:41:PRO:HD3	2.20	0.40
24:R:41:PRO:O	24:R:45:THR:HG23	2.22	0.40
24:R:71:THR:HG23	24:R:73:ARG:H	1.85	0.40
28:a:263:G:H2’	28:a:264:C:O4’	2.21	0.40
28:a:1197:G:H2’	28:a:1198:U:C6	2.54	0.40
28:a:1532:A:C6	28:a:1540:G:C6	3.09	0.40
28:a:1564:C:H2’	28:a:1565:C:C6	2.55	0.40
28:a:2011:U:H2’	28:a:2012:G:O4’	2.21	0.40
32:e:141:MET:HE2	32:e:141:MET:HB3	1.99	0.40
33:f:57:LEU:HD12	33:f:57:LEU:HA	1.87	0.40
46:s:4:GLU:HG3	46:s:49:LYS:HE2	2.03	0.40
5:4:12:ILE:HD13	5:4:28:VAL:HG21	2.04	0.40
7:A:272:C:H2’	7:A:273:U:C6	2.56	0.40
7:A:672:U:H2’	7:A:673:A:C8	2.55	0.40
7:A:925:G:H1’	7:A:1502:A:C4	2.56	0.40
7:A:1184:G:H2’	7:A:1185:G:H8	1.86	0.40
8:B:97:LEU:H	8:B:97:LEU:HD22	1.86	0.40
9:C:129:MET:HB2	9:C:132:ARG:HD2	2.04	0.40
13:G:67:GLU:HG2	13:G:70:ARG:HH21	1.86	0.40
16:J:10:LEU:HD13	16:J:22:THR:HG22	2.03	0.40
21:O:35:GLN:NE2	21:O:35:GLN:HA	2.36	0.40
27:U:16:LEU:HD12	27:U:16:LEU:HA	1.89	0.40
28:a:6:A:H2’	28:a:7:G:H8	1.85	0.40
28:a:1230:A:H2’	28:a:1231:U:C6	2.56	0.40
28:a:2299:U:OP1	33:f:72:LYS:HE3	2.21	0.40
28:a:2391:G:O6	28:a:2425:A:H8	2.04	0.40
28:a:2589:A:H2’	28:a:2590:A:C8	2.55	0.40
28:a:2687:U:H2’	28:a:2688:G:O4’	2.22	0.40
39:l:42:THR:HG22	39:l:93:VAL:HG12	2.04	0.40
40:m:4:ARG:HG2	40:m:4:ARG:NH1	2.36	0.40
42:o:6:LYS:HB3	42:o:6:LYS:HE3	1.83	0.40
44:q:37:GLU:HB3	44:q:53:PHE:CE1	2.56	0.40
7:A:15:G:H4’	11:E:29:ARG:NH1	2.35	0.40
7:A:404:G:OP2	10:D:115:ARG:NH1	2.54	0.40
7:A:627:G:OP2	22:P:35:ARG:NH2	2.54	0.40
7:A:723:U:O4	27:U:53:VAL:HG22	2.21	0.40
7:A:990:C:H2’	7:A:991:U:C6	2.57	0.40
7:A:1138:G:C5	7:A:1140:C:H1’	2.56	0.40
10:D:94:LEU:HD23	10:D:94:LEU:HA	1.93	0.40

Continued on next page...

For Manuscript Review

For Manuscript Review

Continued from previous page...

Atom-1	Atom-2	Interatomic distance (Å)	Clash overlap (Å)
11:E:45:ARG:HH21	11:E:71:MET:HE1	1.87	0.40
15:I:75:GLN:O	15:I:79:ILE:HG12	2.22	0.40
15:I:107:ASP:OD1	15:I:109:ARG:NH2	2.40	0.40
26:T:70:ASN:O	26:T:74:ARG:HG3	2.21	0.40
28:a:822:G:H2'	28:a:823:C:C6	2.57	0.40
28:a:1019:U:H2'	28:a:1020:A:C8	2.56	0.40
30:c:272:SER:O	30:c:272:SER:OG	2.32	0.40
47:t:79:LYS:HE2	47:t:79:LYS:HB3	1.66	0.40
7:A:79:G:N2	7:A:91:U:O4	2.54	0.40
7:A:474:G:H2'	7:A:475:C:H6	1.86	0.40
7:A:502:A:H2'	7:A:503:C:C6	2.56	0.40
7:A:514:C:C2	7:A:515:G:C8	3.10	0.40
7:A:973:G:OP1	16:J:59:LYS:NZ	2.33	0.40
7:A:1034:G:H2'	7:A:1035:A:C8	2.57	0.40
13:G:31:MET:HE3	13:G:31:MET:HB2	1.67	0.40
14:H:18:GLN:HE21	14:H:72:VAL:H	1.68	0.40
20:N:47:LYS:HZ2	20:N:47:LYS:HA	1.86	0.40
22:P:72:ALA:O	22:P:75:ILE:HG22	2.22	0.40
26:T:60:ARG:O	26:T:64:LYS:HG2	2.21	0.40
28:a:1511:G:H2'	28:a:1512:C:C6	2.55	0.40
28:a:1715:G:O2'	28:a:1716:U:OP2	2.39	0.40
28:a:2901:C:H2'	28:a:2902:C:C6	2.57	0.40
30:c:8:PRO:HB3	30:c:14:ARG:HG3	2.03	0.40
31:d:17:GLU:H	31:d:17:GLU:HG2	1.56	0.40
33:f:32:GLU:OE1	33:f:32:GLU:C	2.65	0.40
34:g:54:PRO:HD3	34:g:62:TRP:CZ3	2.57	0.40
7:A:62:U:OP1	7:A:385:C:O2'	2.38	0.40
7:A:453:G:C4	7:A:454:G:C8	3.10	0.40
7:A:553:A:N6	7:A:554:A:N6	2.70	0.40
7:A:1271:A:C2	7:A:1272:G:C5	3.09	0.40
11:E:151:GLU:OE2	11:E:151:GLU:HA	2.22	0.40
12:F:71:ILE:HD13	12:F:71:ILE:HA	1.85	0.40
20:N:49:GLN:O	20:N:50:THR:C	2.64	0.40
21:O:72:ARG:HG2	21:O:72:ARG:O	2.21	0.40
22:P:54:LEU:HD12	22:P:54:LEU:HA	1.76	0.40
28:a:127:A:H5"	28:a:128:C:O4'	2.22	0.40
28:a:539:G:H5'	36:i:9:GLU:OE2	2.21	0.40
28:a:881:G:H2'	28:a:882:G:C8	2.57	0.40
28:a:1264:A:OP1	53:z:16:ARG:NH2	2.40	0.40
28:a:1447:C:H2'	28:a:1448:G:C8	2.56	0.40
28:a:1636:U:H2'	28:a:1637:A:H8	1.86	0.40

Continued on next page...

Continued from previous page...

Atom-1	Atom-2	Interatomic distance (Å)	Clash overlap (Å)
28:a:1733:G:C2	28:a:1734:G:C5	3.09	0.40
28:a:2500:U:O2'	28:a:2504:PSU:OP1	2.38	0.40
28:a:2585:U:H6	28:a:2585:U:H2'	1.68	0.40
32:e:90:GLN:HG3	32:e:92:HIS:CE1	2.56	0.40
33:f:60:ILE:HD11	33:f:138:PHE:CD2	2.56	0.40
46:s:58:VAL:HG22	46:s:85:VAL:HG13	2.03	0.40
48:u:48:MET:O	48:u:51:GLN:HG3	2.22	0.40

There are no symmetry-related clashes.

5.3 Torsion angles ⓘ

5.3.1 Protein backbone ⓘ

In the following table, the Percentiles column shows the percent Ramachandran outliers of the chain as a percentile score with respect to all PDB entries followed by that with respect to all EM entries.

The Analysed column shows the number of residues for which the backbone conformation was analysed, and the total number of residues.

Mol	Chain	Analysed	Favoured	Allowed	Outliers	Percentiles	
1	0	49/55 (89%)	49 (100%)	0	0	100	100
2	1	44/46 (96%)	44 (100%)	0	0	100	100
3	2	62/65 (95%)	60 (97%)	2 (3%)	0	100	100
4	3	36/38 (95%)	36 (100%)	0	0	100	100
5	4	56/70 (80%)	53 (95%)	3 (5%)	0	100	100
8	B	222/241 (92%)	210 (95%)	12 (5%)	0	100	100
9	C	204/233 (88%)	192 (94%)	12 (6%)	0	100	100
10	D	203/206 (98%)	186 (92%)	15 (7%)	2 (1%)	13	46
11	E	154/167 (92%)	151 (98%)	3 (2%)	0	100	100
12	F	101/135 (75%)	97 (96%)	4 (4%)	0	100	100
13	G	151/179 (84%)	139 (92%)	12 (8%)	0	100	100
14	H	127/130 (98%)	121 (95%)	6 (5%)	0	100	100
15	I	125/130 (96%)	121 (97%)	4 (3%)	0	100	100
16	J	96/103 (93%)	92 (96%)	3 (3%)	1 (1%)	13	46

Continued on next page...

For Manuscript Review

Continued from previous page...

Mol	Chain	Analysed	Favoured	Allowed	Outliers	Percentiles	
17	K	113/129 (88%)	106 (94%)	7 (6%)	0	100	100
18	L	122/124 (98%)	107 (88%)	14 (12%)	1 (1%)	16	51
19	M	113/118 (96%)	105 (93%)	8 (7%)	0	100	100
20	N	98/101 (97%)	77 (79%)	8 (8%)	13 (13%)	0	1
21	O	86/89 (97%)	84 (98%)	2 (2%)	0	100	100
22	P	79/82 (96%)	60 (76%)	13 (16%)	6 (8%)	1	4
23	Q	77/84 (92%)	73 (95%)	4 (5%)	0	100	100
24	R	64/75 (85%)	56 (88%)	6 (9%)	2 (3%)	3	19
25	S	82/92 (89%)	81 (99%)	1 (1%)	0	100	100
26	T	84/87 (97%)	82 (98%)	2 (2%)	0	100	100
27	U	68/71 (96%)	67 (98%)	1 (2%)	0	100	100
30	c	269/273 (98%)	262 (97%)	7 (3%)	0	100	100
31	d	206/209 (99%)	197 (96%)	8 (4%)	1 (0%)	25	61
32	e	199/201 (99%)	196 (98%)	3 (2%)	0	100	100
33	f	175/179 (98%)	169 (97%)	6 (3%)	0	100	100
34	g	174/177 (98%)	156 (90%)	17 (10%)	1 (1%)	22	57
35	h	39/149 (26%)	34 (87%)	5 (13%)	0	100	100
36	i	140/142 (99%)	139 (99%)	1 (1%)	0	100	100
37	j	122/123 (99%)	118 (97%)	4 (3%)	0	100	100
38	k	142/144 (99%)	139 (98%)	3 (2%)	0	100	100
39	l	132/136 (97%)	127 (96%)	5 (4%)	0	100	100
40	m	116/127 (91%)	111 (96%)	5 (4%)	0	100	100
41	n	114/117 (97%)	109 (96%)	5 (4%)	0	100	100
42	o	112/115 (97%)	107 (96%)	5 (4%)	0	100	100
43	p	115/118 (98%)	114 (99%)	1 (1%)	0	100	100
44	q	101/103 (98%)	96 (95%)	5 (5%)	0	100	100
45	r	108/110 (98%)	107 (99%)	1 (1%)	0	100	100
46	s	91/100 (91%)	85 (93%)	6 (7%)	0	100	100
47	t	100/104 (96%)	93 (93%)	7 (7%)	0	100	100
48	u	92/94 (98%)	91 (99%)	1 (1%)	0	100	100
49	v	76/85 (89%)	74 (97%)	2 (3%)	0	100	100

Continued on next page...

For Manuscript Review

Continued from previous page...

Mol	Chain	Analysed	Favoured	Allowed	Outliers	Percentiles	
50	w	75/78 (96%)	75 (100%)	0	0	100	100
51	x	60/63 (95%)	56 (93%)	4 (7%)	0	100	100
52	y	56/59 (95%)	54 (96%)	2 (4%)	0	100	100
53	z	54/57 (95%)	53 (98%)	1 (2%)	0	100	100
All	All	5484/5913 (93%)	5211 (95%)	246 (4%)	27 (0%)	27	61

All (27) Ramachandran outliers are listed below:

Mol	Chain	Res	Type
20	N	17	ALA
20	N	18	ASP
20	N	33	ASP
20	N	35	ASN
22	P	24	SER
34	g	47	ASP
16	J	57	VAL
20	N	30	ILE
20	N	31	ILE
20	N	32	SER
20	N	51	LEU
24	R	47	THR
24	R	49	ALA
31	d	149	ASN
18	L	123	LYS
20	N	29	ALA
20	N	20	TYR
20	N	34	VAL
20	N	49	GLN
22	P	50	THR
22	P	22	ALA
22	P	34	GLU
10	D	21	LEU
22	P	64	GLY
10	D	46	PRO
22	P	2	VAL
20	N	45	VAL

For Manuscript Review

5.3.2 Protein sidechains (i)

In the following table, the Percentiles column shows the percent sidechain outliers of the chain as a percentile score with respect to all PDB entries followed by that with respect to all EM entries.

The Analysed column shows the number of residues for which the sidechain conformation was analysed, and the total number of residues.

Mol	Chain	Analysed	Rotameric	Outliers	Percentiles	
1	0	46/49 (94%)	45 (98%)	1 (2%)	47	76
2	1	38/38 (100%)	37 (97%)	1 (3%)	41	72
3	2	51/52 (98%)	51 (100%)	0	100	100
4	3	34/34 (100%)	34 (100%)	0	100	100
5	4	55/62 (89%)	52 (94%)	3 (6%)	18	50
8	B	186/199 (94%)	182 (98%)	4 (2%)	47	76
9	C	170/190 (90%)	162 (95%)	8 (5%)	22	56
10	D	172/173 (99%)	161 (94%)	11 (6%)	14	44
11	E	119/126 (94%)	113 (95%)	6 (5%)	20	53
12	F	90/116 (78%)	85 (94%)	5 (6%)	17	49
13	G	126/147 (86%)	121 (96%)	5 (4%)	27	61
14	H	104/105 (99%)	101 (97%)	3 (3%)	37	70
15	I	105/107 (98%)	102 (97%)	3 (3%)	37	70
16	J	86/90 (96%)	83 (96%)	3 (4%)	31	65
17	K	89/98 (91%)	87 (98%)	2 (2%)	47	76
18	L	104/104 (100%)	94 (90%)	10 (10%)	7	27
19	M	93/96 (97%)	90 (97%)	3 (3%)	34	67
20	N	83/84 (99%)	70 (84%)	13 (16%)	2	10
21	O	76/77 (99%)	74 (97%)	2 (3%)	41	72
22	P	65/65 (100%)	46 (71%)	19 (29%)	0	1
23	Q	73/78 (94%)	68 (93%)	5 (7%)	13	42
24	R	57/65 (88%)	55 (96%)	2 (4%)	31	65
25	S	72/79 (91%)	70 (97%)	2 (3%)	38	70
26	T	65/66 (98%)	61 (94%)	4 (6%)	15	45
27	U	60/61 (98%)	57 (95%)	3 (5%)	20	53
30	c	216/218 (99%)	213 (99%)	3 (1%)	62	83

Continued on next page...

For Manuscript Review

Continued from previous page...

Mol	Chain	Analysed	Rotameric	Outliers	Percentiles	
31	d	163/163 (100%)	161 (99%)	2 (1%)	67	86
32	e	165/165 (100%)	160 (97%)	5 (3%)	36	69
33	f	148/150 (99%)	144 (97%)	4 (3%)	40	71
34	g	137/138 (99%)	131 (96%)	6 (4%)	24	58
35	h	32/114 (28%)	31 (97%)	1 (3%)	35	68
36	i	116/116 (100%)	116 (100%)	0	100	100
37	j	105/104 (101%)	103 (98%)	2 (2%)	52	79
38	k	103/103 (100%)	100 (97%)	3 (3%)	37	70
39	l	107/107 (100%)	106 (99%)	1 (1%)	75	89
40	m	98/103 (95%)	97 (99%)	1 (1%)	73	88
41	n	86/87 (99%)	84 (98%)	2 (2%)	45	75
42	o	99/100 (99%)	96 (97%)	3 (3%)	36	69
43	p	89/90 (99%)	89 (100%)	0	100	100
44	q	84/84 (100%)	82 (98%)	2 (2%)	44	74
45	r	93/93 (100%)	93 (100%)	0	100	100
46	s	80/84 (95%)	79 (99%)	1 (1%)	65	85
47	t	83/85 (98%)	81 (98%)	2 (2%)	44	74
48	u	78/78 (100%)	75 (96%)	3 (4%)	28	62
49	v	58/63 (92%)	58 (100%)	0	100	100
50	w	67/68 (98%)	66 (98%)	1 (2%)	60	83
51	x	54/55 (98%)	52 (96%)	2 (4%)	29	63
52	y	48/49 (98%)	46 (96%)	2 (4%)	25	59
53	z	47/48 (98%)	47 (100%)	0	100	100
All	All	4575/4826 (95%)	4411 (96%)	164 (4%)	32	64

All (164) residues with a non-rotameric sidechain are listed below:

Mol	Chain	Res	Type
1	0	25	LYS
2	1	8	SER
5	4	10	GLU
5	4	21	VAL
5	4	37	CYS
8	B	38	VAL

Continued on next page...

For Manuscript Review

Continued from previous page...

Mol	Chain	Res	Type
8	B	62	SER
8	B	147	SER
8	B	189	THR
9	C	26	THR
9	C	49	LYS
9	C	63	SER
9	C	76	VAL
9	C	131	ARG
9	C	178	LEU
9	C	186	THR
9	C	192	THR
10	D	25	VAL
10	D	29	ASP
10	D	30	THR
10	D	31	LYS
10	D	33	LYS
10	D	41	HIS
10	D	49	SER
10	D	71	GLN
10	D	110	THR
10	D	134	SER
10	D	169	THR
11	E	26	LYS
11	E	45	ARG
11	E	60	ILE
11	E	70	ASN
11	E	103	THR
11	E	106	ILE
12	F	15	SER
12	F	18	VAL
12	F	36	ILE
12	F	68	GLN
12	F	84	VAL
13	G	41	SER
13	G	57	SER
13	G	89	VAL
13	G	130	ASN
13	G	133	THR
14	H	10	MET
14	H	29	SER
14	H	67	GLN
15	I	9	THR

Continued on next page...

For Manuscript Review

Continued from previous page...

Mol	Chain	Res	Type
15	I	58	VAL
15	I	105	THR
16	J	60	ASP
16	J	76	ILE
16	J	102	LEU
17	K	32	VAL
17	K	87	LYS
18	L	33	VAL
18	L	36	ARG
18	L	43[A]	LYS
18	L	43[B]	LYS
18	L	54	ARG
18	L	59	ASN
18	L	74	LEU
18	L	88	LYS
18	L	120	LYS
18	L	122	PRO
19	M	45	ILE
19	M	56	LEU
19	M	81	MET
20	N	12	LYS
20	N	14	VAL
20	N	16	LEU
20	N	19	LYS
20	N	24	ARG
20	N	27	LEU
20	N	30	ILE
20	N	31	ILE
20	N	34	VAL
20	N	38	ASP
20	N	46	LEU
20	N	47	LYS
20	N	60	GLN
21	O	35	GLN
21	O	75	VAL
22	P	1	MET
22	P	2	VAL
22	P	3	THR
22	P	5	ARG
22	P	8	ARG
22	P	14	ARG
22	P	19	VAL

Continued on next page...

For Manuscript Review

Continued from previous page...

Mol	Chain	Res	Type
22	P	35	ARG
22	P	36	VAL
22	P	44	SER
22	P	46	LYS
22	P	47	GLU
22	P	48	GLU
22	P	50	THR
22	P	52	LEU
22	P	54	LEU
22	P	57	ILE
22	P	61	VAL
22	P	63	GLN
23	Q	13	VAL
23	Q	20	SER
23	Q	41	THR
23	Q	48	ASP
23	Q	50	ASN
24	R	48	ARG
24	R	55	LEU
25	S	13	LEU
25	S	43	ASN
26	T	30	THR
26	T	40	GLU
26	T	43	ASP
26	T	80	THR
27	U	39	GLU
27	U	42	THR
27	U	60	LEU
30	c	4	VAL
30	c	118	SER
30	c	133	ARG
31	d	43	ASP
31	d	92	VAL
32	e	65	THR
32	e	126	VAL
32	e	193	VAL
32	e	195	GLN
32	e	198	GLU
33	f	4	LEU
33	f	66	LEU
33	f	68	THR
33	f	104	ILE

Continued on next page...

For Manuscript Review

Continued from previous page...

Mol	Chain	Res	Type
34	g	41	VAL
34	g	71	LEU
34	g	76	VAL
34	g	90	VAL
34	g	98	VAL
34	g	127	THR
35	h	4	ILE
37	j	51	LYS
37	j	53	LYS
38	k	7	SER
38	k	85	VAL
38	k	144	GLU
39	l	1	MET
40	m	98	LEU
41	n	56	LYS
41	n	62	LEU
42	o	6	LYS
42	o	32	VAL
42	o	85	SER
44	q	47	VAL
44	q	51	VAL
46	s	28	ASN
47	t	15	THR
47	t	101	GLU
48	u	8	VAL
48	u	62	THR
48	u	65	VAL
50	w	48	THR
51	x	6	LEU
51	x	34	SER
52	y	3	LYS
52	y	35	THR

Sometimes sidechains can be flipped to improve hydrogen bonding and reduce clashes. All (54) such sidechains are listed below:

Mol	Chain	Res	Type
4	3	35	GLN
5	4	41	HIS
8	B	15	HIS
8	B	89	GLN
9	C	32	ASN
9	C	140	ASN

Continued on next page...

For Manuscript Review

Continued from previous page...

Mol	Chain	Res	Type
10	D	74	ASN
10	D	152	GLN
10	D	196	ASN
11	E	70	ASN
12	F	37	HIS
12	F	55	HIS
14	H	4	GLN
15	I	25	ASN
15	I	75	GLN
18	L	5	ASN
18	L	6	GLN
20	N	4	GLN
20	N	43	ASN
21	O	35	GLN
22	P	63	GLN
24	R	31	ASN
26	T	3	ASN
26	T	13	GLN
27	U	56	HIS
30	c	25	HIS
30	c	90	ASN
30	c	128	ASN
30	c	134	ASN
30	c	143	ASN
30	c	260	ASN
31	d	185	ASN
32	e	165	HIS
33	f	52	ASN
34	g	30	ASN
34	g	48	ASN
34	g	104	ASN
34	g	128	GLN
34	g	139	GLN
38	k	4	ASN
40	m	18	GLN
41	n	100	HIS
42	o	7	GLN
43	p	20	GLN
43	p	44	GLN
45	r	9	HIS
45	r	40	ASN
46	s	70	HIS

Continued on next page...

For Manuscript Review

Continued from previous page...

Mol	Chain	Res	Type
47	t	74	ASN
48	u	24	ASN
50	w	20	HIS
50	w	36	HIS
51	x	15	ASN
51	x	31	GLN

5.3.3 RNA ⓘ

Mol	Chain	Analysed	Backbone Outliers	Pucker Outliers
28	a	2748/2753 (99%)	321 (11%)	0
29	b	118/119 (99%)	11 (9%)	0
6	5	1/2 (50%)	1 (100%)	0
7	A	1516/1542 (98%)	394 (25%)	26 (1%)
All	All	4383/4416 (99%)	727 (16%)	26 (0%)

All (727) RNA backbone outliers are listed below:

Mol	Chain	Res	Type
6	5	76	A
7	A	3	A
7	A	4	U
7	A	5	U
7	A	6	G
7	A	9	G
7	A	38	G
7	A	39	G
7	A	47	C
7	A	48	C
7	A	50	A
7	A	51	A
7	A	55	A
7	A	65	A
7	A	72	A
7	A	74	A
7	A	76	G
7	A	78	A
7	A	80	A
7	A	81	A
7	A	82	G
7	A	84	U

Continued on next page...

For Manuscript Review

Continued from previous page...

Mol	Chain	Res	Type
7	A	85	U
7	A	86	G
7	A	87	C
7	A	90	C
7	A	91	U
7	A	92	U
7	A	93	U
7	A	95	C
7	A	96	U
7	A	99	C
7	A	116	A
7	A	119	A
7	A	121	U
7	A	122	G
7	A	127	G
7	A	130	A
7	A	131	A
7	A	141	G
7	A	142	G
7	A	143	A
7	A	144	G
7	A	149	A
7	A	155	A
7	A	162	A
7	A	164	G
7	A	167	A
7	A	177	G
7	A	181	A
7	A	182	A
7	A	183	C
7	A	187	G
7	A	197	A
7	A	202	G
7	A	204	G
7	A	215	C
7	A	216	U
7	A	220	G
7	A	226	G
7	A	243	A
7	A	245	U
7	A	247	G
7	A	251	G

Continued on next page...

For Manuscript Review

Continued from previous page...

Mol	Chain	Res	Type
7	A	253	A
7	A	264	C
7	A	265	G
7	A	266	G
7	A	267	C
7	A	289	G
7	A	292	G
7	A	295	C
7	A	306	A
7	A	307	C
7	A	319	G
7	A	321	A
7	A	328	C
7	A	344	A
7	A	352	C
7	A	354	G
7	A	356	A
7	A	363	A
7	A	364	A
7	A	365	U
7	A	367	U
7	A	368	U
7	A	372	C
7	A	374	A
7	A	375	U
7	A	380	G
7	A	389	A
7	A	390	U
7	A	392	C
7	A	401	C
7	A	406	G
7	A	407	U
7	A	408	A
7	A	413	G
7	A	414	A
7	A	415	A
7	A	421	U
7	A	422	C
7	A	423	G
7	A	424	G
7	A	426	U
7	A	429	U

Continued on next page...

For Manuscript Review

Continued from previous page...

Mol	Chain	Res	Type
7	A	431	A
7	A	436	C
7	A	437	U
7	A	438	U
7	A	439	U
7	A	448	A
7	A	449	G
7	A	451	A
7	A	453	G
7	A	457	G
7	A	458	U
7	A	459	A
7	A	463	U
7	A	464	U
7	A	465	A
7	A	466	A
7	A	467	U
7	A	468	A
7	A	469	C
7	A	472	U
7	A	474	G
7	A	475	C
7	A	476	U
7	A	477	C
7	A	478	A
7	A	479	U
7	A	480	U
7	A	481	G
7	A	484	G
7	A	486	U
7	A	487	A
7	A	489	C
7	A	491	G
7	A	492	C
7	A	496	A
7	A	497	G
7	A	498	A
7	A	499	A
7	A	500	G
7	A	501	C
7	A	509	A
7	A	511	C

Continued on next page...

For Manuscript Review

Continued from previous page...

Mol	Chain	Res	Type
7	A	512	U
7	A	513	C
7	A	518	C
7	A	521	G
7	A	524	G
7	A	527	G7M
7	A	528	C
7	A	530	G
7	A	531	U
7	A	532	A
7	A	545	C
7	A	547	A
7	A	548	G
7	A	549	C
7	A	552	U
7	A	559	A
7	A	563	A
7	A	570	G
7	A	572	A
7	A	573	A
7	A	576	C
7	A	577	G
7	A	578	C
7	A	596	A
7	A	603	U
7	A	607	A
7	A	608	A
7	A	609	A
7	A	610	U
7	A	611	C
7	A	614	C
7	A	615	G
7	A	616	G
7	A	621	A
7	A	633	G
7	A	639	G
7	A	642	A
7	A	650	G
7	A	653	U
7	A	665	A
7	A	670	G
7	A	685	G

Continued on next page...

For Manuscript Review

Continued from previous page...

Mol	Chain	Res	Type
7	A	687	A
7	A	694	A
7	A	695	A
7	A	703	G
7	A	721	G
7	A	723	U
7	A	724	G
7	A	734	G
7	A	743	A
7	A	747	A
7	A	748	G
7	A	749	A
7	A	755	G
7	A	764	C
7	A	766	A
7	A	774	G
7	A	777	A
7	A	781	A
7	A	792	A
7	A	793	U
7	A	794	A
7	A	799	G
7	A	815	A
7	A	817	C
7	A	820	U
7	A	821	G
7	A	829	G
7	A	832	G
7	A	836	G
7	A	848	C
7	A	849	G
7	A	872	A
7	A	877	G
7	A	878	A
7	A	890	G
7	A	910	C
7	A	914	A
7	A	918	A
7	A	926	G
7	A	927	G
7	A	933	G
7	A	934	C

Continued on next page...

For Manuscript Review

Continued from previous page...

Mol	Chain	Res	Type
7	A	935	A
7	A	942	G
7	A	946	A
7	A	958	A
7	A	960	U
7	A	961	U
7	A	965	U
7	A	966	2MG
7	A	968	A
7	A	969	A
7	A	972	C
7	A	975	A
7	A	976	G
7	A	984	C
7	A	987	G
7	A	993	G
7	A	994	A
7	A	999	C
7	A	1003	G
7	A	1004	A
7	A	1005	A
7	A	1006	G
7	A	1007	U
7	A	1009	U
7	A	1018	G
7	A	1019	A
7	A	1020	G
7	A	1023	U
7	A	1026	G
7	A	1027	C
7	A	1029	U
7	A	1030	U
7	A	1031	C
7	A	1032	G
7	A	1033	G
7	A	1034	G
7	A	1035	A
7	A	1036	A
7	A	1038	C
7	A	1042	A
7	A	1043	G
7	A	1044	A

Continued on next page...

For Manuscript Review

Continued from previous page...

Mol	Chain	Res	Type
7	A	1045	C
7	A	1046	A
7	A	1056	U
7	A	1059	C
7	A	1065	U
7	A	1066	C
7	A	1067	A
7	A	1086	U
7	A	1088	G
7	A	1089	G
7	A	1094	G
7	A	1095	U
7	A	1101	A
7	A	1122	U
7	A	1123	U
7	A	1124	G
7	A	1132	C
7	A	1137	C
7	A	1138	G
7	A	1139	G
7	A	1141	C
7	A	1144	G
7	A	1159	U
7	A	1160	G
7	A	1164	G
7	A	1167	A
7	A	1168	U
7	A	1169	A
7	A	1171	A
7	A	1181	G
7	A	1182	G
7	A	1184	G
7	A	1185	G
7	A	1188	A
7	A	1194	U
7	A	1196	A
7	A	1197	A
7	A	1200	C
7	A	1213	A
7	A	1219	A
7	A	1225	A
7	A	1227	A

Continued on next page...

For Manuscript Review

Continued from previous page...

Mol	Chain	Res	Type
7	A	1228	C
7	A	1230	C
7	A	1234	C
7	A	1236	A
7	A	1238	A
7	A	1240	U
7	A	1241	G
7	A	1246	A
7	A	1247	U
7	A	1253	G
7	A	1258	G
7	A	1260	G
7	A	1270	G
7	A	1275	A
7	A	1280	A
7	A	1284	C
7	A	1285	A
7	A	1286	U
7	A	1287	A
7	A	1293	C
7	A	1294	G
7	A	1295	U
7	A	1296	C
7	A	1297	G
7	A	1298	U
7	A	1300	G
7	A	1302	C
7	A	1304	G
7	A	1305	G
7	A	1317	C
7	A	1319	A
7	A	1320	C
7	A	1322	C
7	A	1327	C
7	A	1331	G
7	A	1332	A
7	A	1335	U
7	A	1336	C
7	A	1346	A
7	A	1353	G
7	A	1359	C
7	A	1360	A

Continued on next page...

For Manuscript Review

Continued from previous page...

Mol	Chain	Res	Type
7	A	1361	G
7	A	1363	A
7	A	1364	U
7	A	1368	A
7	A	1370	G
7	A	1375	A
7	A	1378	C
7	A	1379	G
7	A	1380	U
7	A	1397	C
7	A	1398	A
7	A	1399	C
7	A	1404	C
7	A	1432	G
7	A	1437	A
7	A	1441	A
7	A	1446	A
7	A	1448	C
7	A	1452	C
7	A	1453	G
7	A	1460	C
7	A	1492	A
7	A	1493	A
7	A	1494	G
7	A	1497	G
7	A	1503	A
7	A	1506	U
7	A	1510	C
7	A	1511	G
7	A	1517	G
7	A	1518	MA6
7	A	1529	G
7	A	1530	G
7	A	1531	A
7	A	1532	U
7	A	1533	C
7	A	1534	A
28	a	10	A
28	a	34	U
28	a	42	A
28	a	45	G
28	a	51	G

Continued on next page...

For Manuscript Review

Continued from previous page...

Mol	Chain	Res	Type
28	a	71	A
28	a	74	A
28	a	75	G
28	a	101	A
28	a	102	U
28	a	103	A
28	a	110	G
28	a	118	A
28	a	119	A
28	a	120	U
28	a	139	U
28	a	142	A
28	a	163	C
28	a	181	A
28	a	196	A
28	a	199	A
28	a	216	A
28	a	222	A
28	a	248	G
28	a	264	C
28	a	265	A
28	a	272	A
28	a	274	C
28	a	278	A
28	a	279	A
28	a	282	A
28	a	285	G
28	a	289	G
28	a	294	A
28	a	311	A
28	a	329	G
28	a	330	A
28	a	345	A
28	a	361	G
28	a	362	A
28	a	386	G
28	a	396	G
28	a	405	U
28	a	411	G
28	a	481	G
28	a	491	G
28	a	504	A

Continued on next page...

For Manuscript Review

Continued from previous page...

Mol	Chain	Res	Type
28	a	505	A
28	a	509	C
28	a	510	C
28	a	529	A
28	a	530	G
28	a	531	C
28	a	532	A
28	a	545	U
28	a	546	U
28	a	548	G
28	a	549	G
28	a	563	A
28	a	573	U
28	a	575	A
28	a	603	A
28	a	615	U
28	a	627	A
28	a	637	A
28	a	645	C
28	a	647	G
28	a	653	U
28	a	654	A
28	a	655	A
28	a	686	U
28	a	717	C
28	a	721	A
28	a	729	G
28	a	730	A
28	a	738	G
28	a	747	5MU
28	a	764	A
28	a	775	G
28	a	776	G
28	a	782	A
28	a	784	G
28	a	785	G
28	a	791	C
28	a	792	A
28	a	805	G
28	a	812	C
28	a	827	U
28	a	828	U

Continued on next page...

For Manuscript Review

Continued from previous page...

Mol	Chain	Res	Type
28	a	845	A
28	a	846	U
28	a	847	U
28	a	859	G
28	a	879	G
28	a	883	G
28	a	884	U
28	a	888	C
28	a	890	C
28	a	891	G
28	a	895	U
28	a	896	A
28	a	897	C
28	a	898	C
28	a	899	A
28	a	907	G
28	a	910	A
28	a	915	C
28	a	945	A
28	a	946	C
28	a	961	C
28	a	974	G
28	a	983	A
28	a	996	A
28	a	1005	C
28	a	1012	U
28	a	1013	C
28	a	1020	A
28	a	1033	U
28	a	1041	G
28	a	1045	C
28	a	1046	A
28	a	1047	G
28	a	1048	A
28	a	1051	G
28	a	1108	U
28	a	1111	A
28	a	1112	G
28	a	1115	G
28	a	1116	G
28	a	1122	G
28	a	1132	U

Continued on next page...

For Manuscript Review

Continued from previous page...

Mol	Chain	Res	Type
28	a	1133	A
28	a	1135	C
28	a	1142	A
28	a	1171	G
28	a	1206	G
28	a	1236	G
28	a	1250	G
28	a	1253	A
28	a	1256	G
28	a	1271	G
28	a	1272	A
28	a	1275	A
28	a	1300	G
28	a	1301	A
28	a	1352	U
28	a	1365	A
28	a	1378	A
28	a	1379	U
28	a	1383	A
28	a	1386	C
28	a	1411	U
28	a	1416	G
28	a	1417	C
28	a	1421	G
28	a	1427	A
28	a	1428	C
28	a	1452	G
28	a	1453	A
28	a	1482	G
28	a	1493	C
28	a	1497	U
28	a	1509	A
28	a	1510	G
28	a	1515	A
28	a	1524	G
28	a	1529	G
28	a	1534	U
28	a	1535	A
28	a	1536	C
28	a	1537	G
28	a	1558	C
28	a	1566	A

Continued on next page...

For Manuscript Review

Continued from previous page...

Mol	Chain	Res	Type
28	a	1569	A
28	a	1578	U
28	a	1583	A
28	a	1584	U
28	a	1585	C
28	a	1586	A
28	a	1608	A
28	a	1647	U
28	a	1648	U
28	a	1649	G
28	a	1674	G
28	a	1675	C
28	a	1715	G
28	a	1729	U
28	a	1730	C
28	a	1738	G
28	a	1757	A
28	a	1764	C
28	a	1773	A
28	a	1782	U
28	a	1791	A
28	a	1800	C
28	a	1801	A
28	a	1808	A
28	a	1816	C
28	a	1829	A
28	a	1847	A
28	a	1858	A
28	a	1868	C
28	a	1870	C
28	a	1871	A
28	a	1873	G
28	a	1906	G
28	a	1907	G
28	a	1912	A
28	a	1913	A
28	a	1919	A
28	a	1929	G
28	a	1930	G
28	a	1936	A
28	a	1938	A
28	a	1955	U

Continued on next page...

For Manuscript Review

Continued from previous page...

Mol	Chain	Res	Type
28	a	1966	A
28	a	1967	C
28	a	1970	A
28	a	1971	U
28	a	1972	G
28	a	1991	U
28	a	1993	U
28	a	2020	A
28	a	2023	C
28	a	2027	G
28	a	2030	6MZ
28	a	2031	A
28	a	2033	A
28	a	2035	G
28	a	2043	C
28	a	2055	C
28	a	2056	G
28	a	2060	A
28	a	2061	G
28	a	2062	A
28	a	2069	G7M
28	a	2093	G
28	a	2198	A
28	a	2204	G
28	a	2211	A
28	a	2225	A
28	a	2238	G
28	a	2239	G
28	a	2279	G
28	a	2283	C
28	a	2287	A
28	a	2288	A
28	a	2305	U
28	a	2308	G
28	a	2322	A
28	a	2325	G
28	a	2333	A
28	a	2336	A
28	a	2345	G
28	a	2347	C
28	a	2350	C
28	a	2361	G

Continued on next page...

For Manuscript Review

Continued from previous page...

Mol	Chain	Res	Type
28	a	2377	A
28	a	2383	G
28	a	2385	C
28	a	2402	U
28	a	2403	C
28	a	2425	A
28	a	2429	G
28	a	2435	A
28	a	2440	C
28	a	2441	U
28	a	2445	2MG
28	a	2448	A
28	a	2470	G
28	a	2476	A
28	a	2478	A
28	a	2491	U
28	a	2498	OMC
28	a	2502	G
28	a	2505	G
28	a	2518	A
28	a	2520	C
28	a	2529	G
28	a	2535	G
28	a	2547	A
28	a	2566	A
28	a	2567	G
28	a	2573	C
28	a	2585	U
28	a	2586	U
28	a	2602	A
28	a	2609	U
28	a	2613	U
28	a	2615	U
28	a	2629	U
28	a	2630	G
28	a	2661	G
28	a	2663	G
28	a	2689	U
28	a	2690	U
28	a	2714	G
28	a	2716	C
28	a	2726	A

Continued on next page...

For Manuscript Review

Continued from previous page...

Mol	Chain	Res	Type
28	a	2732	G
28	a	2733	A
28	a	2744	G
28	a	2748	A
28	a	2752	C
28	a	2757	A
28	a	2765	A
28	a	2778	A
28	a	2795	C
28	a	2798	U
28	a	2818	U
28	a	2820	A
28	a	2821	A
28	a	2833	U
28	a	2835	A
28	a	2849	U
28	a	2861	U
28	a	2873	A
28	a	2883	A
28	a	2884	U
28	a	2899	A
28	a	2902	C
29	b	13	G
29	b	24	G
29	b	33	G
29	b	35	C
29	b	36	C
29	b	56	G
29	b	67	G
29	b	89	U
29	b	90	C
29	b	99	A
29	b	109	A

All (26) RNA pucker outliers are listed below:

Mol	Chain	Res	Type
7	A	4	U
7	A	80	A
7	A	83	C
7	A	86	G
7	A	92	U

Continued on next page...

For Manuscript Review

Continued from previous page...

Mol	Chain	Res	Type
7	A	121	U
7	A	264	C
7	A	374	A
7	A	414	A
7	A	437	U
7	A	438	U
7	A	452	A
7	A	488	C
7	A	495	A
7	A	500	G
7	A	512	U
7	A	531	U
7	A	547	A
7	A	548	G
7	A	722	G
7	A	960	U
7	A	1026	G
7	A	1035	A
7	A	1285	A
7	A	1505	G
7	A	1532	U

5.4 Non-standard residues in protein, DNA, RNA chains ⓘ

39 non-standard protein/DNA/RNA residues are modelled in this entry.

In the following table, the Counts columns list the number of bonds (or angles) for which Mogul statistics could be retrieved, the number of bonds (or angles) that are observed in the model and the number of bonds (or angles) that are defined in the Chemical Component Dictionary. The Link column lists molecule types, if any, to which the group is linked. The Z score for a bond length (or angle) is the number of standard deviations the observed value is removed from the expected value. A bond length (or angle) with $|Z| > 2$ is considered an outlier worth inspection. RMSZ is the root-mean-square of all Z scores of the bond lengths (or angles).

Mol	Type	Chain	Res	Link	Bond lengths			Bond angles		
					Counts	RMSZ	$\#\|Z\| > 2$	Counts	RMSZ	$\#\|Z\| > 2$
28	PSU	a	2605	28	18,21,22	1.23	3 (16%)	21,30,33	1.97	4 (19%)
7	G7M	A	527	7	20,26,27	1.16	3 (15%)	16,39,42	0.42	0
7	5MC	A	967	7	19,22,23	1.34	2 (10%)	26,32,35	1.10	2 (7%)
28	G7M	a	2069	28	20,26,27	0.96	1 (5%)	16,39,42	0.67	0
28	PSU	a	2580	28	18,21,22	1.28	4 (22%)	21,30,33	2.09	5 (23%)
28	2MG	a	1835	28	18,26,27	1.42	3 (16%)	16,38,41	1.64	4 (25%)

For Manuscript Review

Mol	Type	Chain	Res	Link	Bond lengths			Bond angles		
					Counts	RMSZ	#|Z| > 2	Counts	RMSZ	#|Z| > 2
7	4OC	A	1402	7	20,23,24	2.35	4 (20%)	25,32,35	1.00	1 (4%)
28	6MZ	a	1618	28	17,25,26	1.35	3 (17%)	15,36,39	2.11	4 (26%)
28	5MU	a	747	28	19,22,23	1.26	4 (21%)	27,32,35	2.23	9 (33%)
39	4D4	l	81	39	9,11,12	2.04	2 (22%)	7,13,15	2.08	2 (28%)
28	2MG	a	2445	28	18,26,27	1.41	3 (16%)	16,38,41	1.73	5 (31%)
28	PSU	a	2457	28	18,21,22	1.17	4 (22%)	21,30,33	1.98	5 (23%)
17	IAS	K	119	17	6,7,8	1.75	1 (16%)	3,8,10	3.34	1 (33%)
7	5MC	A	1407	7	19,22,23	1.60	2 (10%)	26,32,35	1.20	3 (11%)
28	3TD	a	1915	28	19,22,23	1.17	3 (15%)	23,32,35	1.93	3 (13%)
28	2MA	a	2503	28,55	18,25,26	1.30	3 (16%)	20,37,40	1.97	3 (15%)
31	MEQ	d	150	31	8,9,10	0.89	0	5,10,12	0.53	0
28	OMU	a	2552	28	19,22,23	1.21	3 (15%)	25,31,34	2.04	6 (24%)
28	PSU	a	955	28	18,21,22	1.17	3 (16%)	21,30,33	2.05	4 (19%)
28	PSU	a	2504	28	18,21,22	1.14	3 (16%)	21,30,33	1.92	4 (19%)
7	PSU	A	516	7	18,21,22	1.07	2 (11%)	21,30,33	2.01	5 (23%)
28	5MC	a	1962	28	19,22,23	1.62	2 (10%)	26,32,35	1.20	3 (11%)
28	PSU	a	746	28,55	18,21,22	1.20	3 (16%)	21,30,33	1.92	4 (19%)
28	6MZ	a	2030	28	17,25,26	1.40	3 (17%)	15,36,39	2.40	4 (26%)
28	OMG	a	2251	28	19,26,27	1.31	2 (10%)	21,38,41	1.44	4 (19%)
28	5MU	a	1939	28	19,22,23	1.33	4 (21%)	27,32,35	2.23	6 (22%)
28	OMC	a	2498	28,55	19,22,23	1.06	1 (5%)	25,31,34	1.16	2 (8%)
28	PSU	a	1917	28	18,21,22	1.06	1 (5%)	21,30,33	1.93	4 (19%)
39	MS6	l	82	39	5,7,8	0.66	0	2,7,9	1.20	0
7	2MG	A	1516	7	18,26,27	1.26	3 (16%)	16,38,41	1.27	2 (12%)
7	2MG	A	966	7	18,26,27	1.25	2 (11%)	16,38,41	1.61	4 (25%)
7	MA6	A	1518	7	19,26,27	2.95	10 (52%)	18,38,41	1.50	2 (11%)
28	PSU	a	2604	28	18,21,22	1.17	3 (16%)	21,30,33	2.02	4 (19%)
7	2MG	A	1207	7	18,26,27	1.24	2 (11%)	16,38,41	1.56	4 (25%)
28	1MG	a	745	28	19,26,27	1.50	3 (15%)	18,39,42	1.33	2 (11%)
28	PSU	a	1911	28	18,21,22	1.04	2 (11%)	21,30,33	1.96	4 (19%)
7	MA6	A	1519	7	19,26,27	1.92	4 (21%)	18,38,41	1.28	2 (11%)
7	UR3	A	1498	7	19,22,23	3.81	6 (31%)	26,32,35	4.60	10 (38%)
28	H2U	a	2449	28	18,21,22	0.60	0	19,30,33	1.03	1 (5%)

In the following table, the Chirals column lists the number of chiral outliers, the number of chiral centers analysed, the number of these observed in the model and the number defined in the

For Manuscript Review

Chemical Component Dictionary. Similar counts are reported in the Torsion and Rings columns. '-' means no outliers of that kind were identified.

Mol	Type	Chain	Res	Link	Chirals	Torsions	Rings
28	PSU	a	2605	28	-	0/7/25/26	0/2/2/2
7	G7M	A	527	7	-	3/3/25/26	0/3/3/3
7	5MC	A	967	7	-	0/7/25/26	0/2/2/2
28	G7M	a	2069	28	-	0/3/25/26	0/3/3/3
28	PSU	a	2580	28	-	1/7/25/26	0/2/2/2
28	2MG	a	1835	28	-	0/5/27/28	0/3/3/3
7	4OC	A	1402	7	-	1/9/29/30	0/2/2/2
28	6MZ	a	1618	28	-	0/5/27/28	0/3/3/3
28	5MU	a	747	28	-	1/7/25/26	0/2/2/2
39	4D4	l	81	39	-	3/11/12/14	-
28	2MG	a	2445	28	-	2/5/27/28	0/3/3/3
28	PSU	a	2457	28	-	0/7/25/26	0/2/2/2
17	IAS	K	119	17	-	2/7/7/8	-
7	5MC	A	1407	7	-	0/7/25/26	0/2/2/2
28	3TD	a	1915	28	-	2/7/25/26	0/2/2/2
28	2MA	a	2503	28,55	-	0/3/25/26	0/3/3/3
31	MEQ	d	150	31	-	2/8/9/11	-
28	OMU	a	2552	28	-	2/9/27/28	0/2/2/2
28	PSU	a	955	28	-	0/7/25/26	0/2/2/2
28	PSU	a	2504	28	-	2/7/25/26	0/2/2/2
7	PSU	A	516	7	-	0/7/25/26	0/2/2/2
28	5MC	a	1962	28	-	0/7/25/26	0/2/2/2
28	PSU	a	746	28,55	-	1/7/25/26	0/2/2/2
28	6MZ	a	2030	28	-	2/5/27/28	0/3/3/3
28	OMG	a	2251	28	-	0/5/27/28	0/3/3/3
28	5MU	a	1939	28	-	0/7/25/26	0/2/2/2
28	OMC	a	2498	28,55	-	0/9/27/28	0/2/2/2
28	PSU	a	1917	28	-	0/7/25/26	0/2/2/2
39	MS6	l	82	39	-	3/4/6/8	-
7	2MG	A	1516	7	-	2/5/27/28	0/3/3/3
7	2MG	A	966	7	-	2/5/27/28	0/3/3/3
7	MA6	A	1518	7	-	2/7/29/30	0/3/3/3
28	PSU	a	2604	28	-	0/7/25/26	0/2/2/2
7	2MG	A	1207	7	-	0/5/27/28	0/3/3/3
28	1MG	a	745	28	-	0/3/25/26	0/3/3/3
28	PSU	a	1911	28	-	0/7/25/26	0/2/2/2
7	MA6	A	1519	7	-	3/7/29/30	0/3/3/3
7	UR3	A	1498	7	-	0/7/25/26	0/2/2/2

Continued on next page...

For Manuscript Review

Continued from previous page...

Mol	Type	Chain	Res	Link	Chirals	Torsions	Rings
28	H2U	a	2449	28	-	0/7/38/39	0/2/2/2

All (107) bond length outliers are listed below:

Mol	Chain	Res	Type	Atoms	Z	Observed(Å)	Ideal(Å)
7	A	1498	UR3	C2-N1	13.67	1.57	1.38
7	A	1402	4OC	O2-C2	8.30	1.39	1.23
7	A	1518	MA6	C6-C5	-7.06	1.34	1.44
7	A	1498	UR3	C6-N1	5.88	1.52	1.38
28	a	1962	5MC	C5-C4	-5.81	1.39	1.44
7	A	1518	MA6	C4-N3	-5.55	1.28	1.35
7	A	1407	5MC	C5-C4	-5.40	1.40	1.44
7	A	1498	UR3	C2-N3	5.34	1.49	1.39
7	A	1519	MA6	C6-C5	-5.15	1.37	1.44
39	l	81	4D4	CZ-NE	4.96	1.42	1.33
7	A	967	5MC	C5-C4	-4.25	1.40	1.44
7	A	1402	4OC	C4-N4	4.25	1.44	1.36
7	A	1518	MA6	O4'-C1'	4.24	1.46	1.40
7	A	1518	MA6	C1'-N9	-3.83	1.40	1.49
7	A	1519	MA6	C5-N7	3.65	1.53	1.39
17	K	119	IAS	OD1-CG	3.55	1.40	1.20
7	A	1498	UR3	C6-C5	-3.46	1.27	1.35
7	A	1402	4OC	C2-N3	3.42	1.43	1.36
7	A	1519	MA6	C4-N3	-3.38	1.31	1.35
7	A	1519	MA6	C6-N6	3.38	1.45	1.37
28	a	1835	2MG	C5-C6	-3.36	1.40	1.47
28	a	2503	2MA	C6-N1	3.35	1.39	1.33
7	A	527	G7M	C8-N9	3.33	1.39	1.33
28	a	745	1MG	C8-N7	-3.30	1.29	1.34
28	a	745	1MG	C6-N1	-3.26	1.33	1.39
28	a	1939	5MU	C2-N1	-3.23	1.33	1.38
7	A	1518	MA6	C3'-C4'	-3.21	1.44	1.53
28	a	2445	2MG	C8-N7	-3.19	1.29	1.34
7	A	1518	MA6	C3'-C2'	-3.17	1.44	1.53
28	a	2552	OMU	C2-N1	-3.14	1.33	1.38
28	a	1835	2MG	C8-N7	-3.13	1.29	1.34
28	a	2445	2MG	C5-C6	-3.12	1.41	1.47
28	a	2251	OMG	C8-N7	-3.11	1.30	1.34
28	a	2251	OMG	C5-C6	-3.10	1.41	1.47
28	a	2030	6MZ	C6-C5	-3.07	1.40	1.44
7	A	1516	2MG	C5-C6	-3.01	1.41	1.47
28	a	1618	6MZ	C6-C5	-2.98	1.40	1.44

Continued on next page...

For Manuscript Review

Continued from previous page...

Mol	Chain	Res	Type	Atoms	Z	Observed(Å)	Ideal(Å)
28	a	2498	OMC	C2-N1	-2.89	1.34	1.40
7	A	966	2MG	C5-C6	-2.89	1.41	1.47
28	a	747	5MU	C2-N1	-2.87	1.34	1.38
7	A	1407	5MC	C2-N1	-2.84	1.34	1.40
7	A	967	5MC	C2-N1	-2.81	1.34	1.40
7	A	1207	2MG	C5-C6	-2.81	1.41	1.47
28	a	1915	3TD	C4-N3	-2.79	1.34	1.40
7	A	966	2MG	C8-N7	-2.79	1.30	1.34
39	l	81	4D4	CZ-NH1	2.79	1.44	1.34
7	A	1207	2MG	C8-N7	-2.78	1.30	1.34
28	a	2580	PSU	O4'-C1'	-2.78	1.40	1.43
7	A	1518	MA6	C5-N7	2.78	1.49	1.39
28	a	1962	5MC	C2-N1	-2.74	1.34	1.40
28	a	2030	6MZ	C8-N7	-2.72	1.29	1.34
28	a	1618	6MZ	C8-N7	-2.69	1.29	1.34
28	a	2030	6MZ	C4-N3	-2.66	1.32	1.35
28	a	1939	5MU	C4-C5	-2.52	1.40	1.44
28	a	2069	G7M	C8-N9	2.52	1.37	1.33
28	a	2552	OMU	C4-N3	-2.51	1.34	1.38
28	a	1618	6MZ	C4-N3	-2.50	1.32	1.35
28	a	1939	5MU	C4-N3	-2.48	1.34	1.38
7	A	1516	2MG	C5-C4	-2.48	1.37	1.43
28	a	2604	PSU	C4-N3	-2.45	1.34	1.38
28	a	955	PSU	C4-N3	-2.44	1.34	1.38
28	a	1915	3TD	C4-C5	-2.44	1.42	1.47
7	A	1516	2MG	C8-N7	-2.44	1.31	1.34
28	a	2605	PSU	C4-N3	-2.43	1.34	1.38
28	a	2605	PSU	C2-N3	-2.43	1.33	1.37
28	a	2504	PSU	C4-N3	-2.42	1.34	1.38
28	a	747	5MU	C4-N3	-2.41	1.34	1.38
28	a	2580	PSU	C4-N3	-2.40	1.34	1.38
28	a	745	1MG	C5-C6	-2.40	1.40	1.47
28	a	746	PSU	C4-N3	-2.39	1.34	1.38
28	a	1939	5MU	C2-N3	-2.38	1.33	1.38
28	a	2457	PSU	C4-N3	-2.36	1.34	1.38
28	a	747	5MU	C4-C5	-2.36	1.41	1.44
7	A	516	PSU	O4'-C1'	-2.34	1.40	1.43
7	A	1518	MA6	C6-N1	-2.34	1.29	1.32
28	a	747	5MU	C2-N3	-2.32	1.33	1.38
28	a	2552	OMU	C2-N3	-2.31	1.33	1.38
28	a	746	PSU	O4'-C1'	-2.31	1.40	1.43
7	A	1498	UR3	O4-C4	-2.29	1.18	1.23

Continued on next page...

For Manuscript Review

Continued from previous page...

Mol	Chain	Res	Type	Atoms	Z	Observed(Å)	Ideal(Å)
28	a	2605	PSU	C2-N1	-2.28	1.33	1.36
7	A	527	G7M	C5-C6	-2.27	1.39	1.45
28	a	2457	PSU	C2-N1	-2.26	1.33	1.36
28	a	2604	PSU	C2-N3	-2.25	1.33	1.37
28	a	2580	PSU	C2-N1	-2.23	1.33	1.36
7	A	527	G7M	C8-N7	2.22	1.37	1.33
28	a	1911	PSU	C4-N3	-2.22	1.34	1.38
28	a	955	PSU	C2-N1	-2.21	1.33	1.36
7	A	1402	4OC	C6-C5	2.21	1.40	1.35
28	a	2503	2MA	C2-N3	-2.20	1.30	1.34
28	a	955	PSU	C2-N3	-2.19	1.33	1.37
28	a	2580	PSU	C2-N3	-2.19	1.33	1.37
28	a	2445	2MG	C6-N1	-2.18	1.34	1.37
28	a	2457	PSU	C2-N3	-2.17	1.33	1.37
28	a	2504	PSU	C2-N3	-2.16	1.33	1.37
28	a	746	PSU	C2-N3	-2.15	1.33	1.37
28	a	1917	PSU	C4-N3	-2.14	1.34	1.38
7	A	1518	MA6	O3'-C3'	2.13	1.48	1.43
28	a	2504	PSU	C2-N1	-2.13	1.33	1.36
7	A	1498	UR3	O2-C2	-2.12	1.18	1.22
28	a	2457	PSU	O4'-C1'	-2.11	1.40	1.43
28	a	2604	PSU	C2-N1	-2.07	1.34	1.36
7	A	1518	MA6	O5'-C5'	-2.06	1.38	1.44
28	a	2503	2MA	C8-N7	-2.04	1.31	1.34
28	a	1835	2MG	C6-N1	-2.03	1.34	1.37
28	a	1911	PSU	C2-N3	-2.03	1.34	1.37
28	a	1915	3TD	C2-N1	-2.03	1.34	1.37
7	A	516	PSU	C4-N3	-2.02	1.35	1.38

All (132) bond angle outliers are listed below:

Mol	Chain	Res	Type	Atoms	Z	Observed(°)	Ideal(°)
7	A	1498	UR3	C4-N3-C2	-14.54	112.88	124.58
7	A	1498	UR3	C6-N1-C2	-13.81	110.51	121.80
7	A	1498	UR3	C5-C4-N3	7.94	125.50	115.04
28	a	1915	3TD	N1-C2-N3	6.93	121.17	116.13
28	a	2503	2MA	C2-N3-C4	5.89	120.22	115.46
28	a	955	PSU	N1-C2-N3	5.84	121.33	115.17
28	a	2580	PSU	N1-C2-N3	5.78	121.26	115.17
28	a	1939	5MU	C4-N3-C2	-5.71	119.85	127.34
28	a	2604	PSU	N1-C2-N3	5.65	121.12	115.17
28	a	747	5MU	C4-N3-C2	-5.64	119.94	127.34

Continued on next page...

For Manuscript Review

Continued from previous page...

Mol	Chain	Res	Type	Atoms	Z	Observed(°)	Ideal(°)
28	a	2552	OMU	C4-N3-C2	-5.58	119.69	126.61
28	a	2457	PSU	N1-C2-N3	5.56	121.03	115.17
7	A	516	PSU	N1-C2-N3	5.54	121.02	115.17
28	a	2504	PSU	N1-C2-N3	5.50	120.97	115.17
28	a	2030	6MZ	C2-N1-C6	5.46	120.84	116.60
28	a	1911	PSU	N1-C2-N3	5.42	120.89	115.17
28	a	2605	PSU	N1-C2-N3	5.41	120.87	115.17
17	K	119	IAS	OD1-CG-CB	-5.36	109.78	125.38
28	a	746	PSU	N1-C2-N3	5.36	120.82	115.17
28	a	1917	PSU	N1-C2-N3	5.36	120.82	115.17
28	a	1939	5MU	C5-C4-N3	5.13	119.78	115.32
28	a	2503	2MA	C5-C6-N1	-5.08	114.83	120.84
28	a	2552	OMU	N3-C2-N1	5.07	121.50	114.89
7	A	1498	UR3	C1'-N1-C2	5.00	125.23	117.04
28	a	2030	6MZ	C9-N6-C6	-4.95	118.26	122.85
28	a	747	5MU	C5-C4-N3	4.94	119.61	115.32
28	a	1618	6MZ	C2-N1-C6	4.82	120.34	116.60
28	a	747	5MU	N3-C2-N1	4.72	121.04	114.89
28	a	1939	5MU	N3-C2-N1	4.72	121.03	114.89
28	a	2030	6MZ	N3-C2-N1	-4.59	122.44	128.67
28	a	1911	PSU	C4-N3-C2	-4.58	120.06	126.37
28	a	2605	PSU	C4-N3-C2	-4.57	120.07	126.37
28	a	2604	PSU	C4-N3-C2	-4.56	120.08	126.37
28	a	746	PSU	C4-N3-C2	-4.56	120.09	126.37
28	a	955	PSU	C4-N3-C2	-4.49	120.19	126.37
28	a	2580	PSU	C4-N3-C2	-4.45	120.24	126.37
28	a	2457	PSU	C4-N3-C2	-4.41	120.30	126.37
28	a	1917	PSU	C4-N3-C2	-4.39	120.32	126.37
28	a	1939	5MU	O4-C4-C5	-4.34	119.95	124.92
28	a	2504	PSU	C4-N3-C2	-4.32	120.42	126.37
28	a	1618	6MZ	N3-C2-N1	-4.26	122.89	128.67
7	A	516	PSU	C4-N3-C2	-4.25	120.52	126.37
28	a	1618	6MZ	C9-N6-C6	-4.20	118.95	122.85
7	A	1498	UR3	C3U-N3-C4	4.14	123.60	117.87
7	A	1207	2MG	C8-N7-C5	4.07	109.48	102.55
28	a	2445	2MG	C8-N7-C5	4.05	109.44	102.55
28	a	747	5MU	O4-C4-C5	-3.97	120.38	124.92
7	A	966	2MG	C8-N7-C5	3.96	109.29	102.55
28	a	1835	2MG	C8-N7-C5	3.89	109.17	102.55
28	a	2251	OMG	C8-N7-C5	3.87	109.14	102.55
28	a	1915	3TD	C4-N3-C2	-3.85	120.54	124.61
28	a	745	1MG	C8-N7-C5	3.84	109.09	102.55

Continued on next page...

For Manuscript Review

Continued from previous page...

Mol	Chain	Res	Type	Atoms	Z	Observed(°)	Ideal(°)
28	a	1939	5MU	C5-C6-N1	-3.84	119.14	123.31
7	A	967	5MC	C5-C6-N1	-3.68	119.31	123.31
7	A	1519	MA6	C1'-N9-C4	-3.68	120.18	126.64
7	A	1407	5MC	C5-C6-N1	-3.59	119.41	123.31
7	A	1498	UR3	C3U-N3-C2	3.55	123.52	117.33
39	l	81	4D4	O-C-CA	-3.48	115.83	124.77
28	a	747	5MU	C5-C6-N1	-3.46	119.56	123.31
28	a	2552	OMU	C5-C4-N3	3.45	119.64	114.80
28	a	1962	5MC	C5-C6-N1	-3.40	119.62	123.31
7	A	516	PSU	O2-C2-N1	-3.38	119.30	122.79
28	a	2449	H2U	C5-C4-N3	-3.32	113.15	116.69
39	l	81	4D4	NE-CZ-NH2	3.32	126.38	120.67
28	a	2552	OMU	CM2-O2'-C2'	-3.30	106.01	114.47
28	a	955	PSU	O2-C2-N1	-3.27	119.42	122.79
28	a	2498	OMC	CM2-O2'-C2'	-3.14	106.41	114.47
28	a	2251	OMG	CM2-O2'-C2'	-3.14	106.42	114.47
28	a	2457	PSU	O2-C2-N1	-3.12	119.57	122.79
28	a	2580	PSU	O2-C2-N1	-3.08	119.61	122.79
28	a	745	1MG	C5-C6-N1	3.07	118.40	113.96
28	a	1917	PSU	O2-C2-N1	-3.04	119.66	122.79
28	a	2445	2MG	N1-C2-N2	2.97	119.59	116.56
7	A	966	2MG	N1-C2-N2	2.94	119.56	116.56
28	a	2445	2MG	CM2-N2-C2	-2.93	117.35	123.65
28	a	746	PSU	O2-C2-N1	-2.92	119.78	122.79
28	a	2604	PSU	O2-C2-N1	-2.89	119.81	122.79
7	A	1519	MA6	C4-C5-N7	-2.86	106.31	109.34
7	A	1518	MA6	C4-C5-N7	-2.84	106.34	109.34
28	a	2605	PSU	O2-C2-N1	-2.83	119.87	122.79
28	a	2552	OMU	O2-C2-N1	-2.83	119.11	122.80
28	a	955	PSU	C6-N1-C2	-2.81	120.09	122.69
28	a	2580	PSU	C6-N1-C2	-2.80	120.09	122.69
28	a	1835	2MG	N1-C2-N2	2.78	119.40	116.56
28	a	2251	OMG	C2-N1-C6	-2.78	120.02	125.11
7	A	1407	5MC	O2-C2-N3	-2.78	117.95	122.33
28	a	1939	5MU	O2-C2-N1	-2.76	119.21	122.80
28	a	1911	PSU	O2-C2-N1	-2.74	119.97	122.79
28	a	2504	PSU	O2-C2-N1	-2.72	119.98	122.79
28	a	2552	OMU	O4-C4-C5	-2.68	120.54	125.16
28	a	2498	OMC	O2-C2-N3	-2.62	118.19	122.33
7	A	516	PSU	C6-N1-C2	-2.62	120.26	122.69
28	a	1915	3TD	C1'-C5-C4	2.60	121.55	117.61
28	a	2504	PSU	C6-N1-C2	-2.57	120.30	122.69

Continued on next page...

For Manuscript Review

Continued from previous page...

Mol	Chain	Res	Type	Atoms	Z	Observed(°)	Ideal(°)
7	A	1516	2MG	C2'-C3'-C4'	-2.55	97.69	102.61
7	A	1402	4OC	C6-C5-C4	2.53	120.05	117.00
28	a	1835	2MG	CM2-N2-C2	-2.52	118.23	123.65
7	A	1518	MA6	O2'-C2'-C3'	-2.50	103.79	111.82
7	A	1207	2MG	N1-C2-N2	2.48	119.09	116.56
28	a	1835	2MG	C5-C6-N1	2.46	118.77	114.07
7	A	1498	UR3	O2-C2-N3	-2.46	117.94	121.33
7	A	1498	UR3	O4-C4-C5	-2.45	117.41	124.35
28	a	2457	PSU	C6-N1-C2	-2.42	120.45	122.69
28	a	2604	PSU	C6-N1-C2	-2.41	120.45	122.69
28	a	2445	2MG	C5-C6-N1	2.40	118.64	114.07
28	a	2030	6MZ	C4-C5-N7	-2.39	106.81	109.34
28	a	1917	PSU	C6-N1-C2	-2.38	120.48	122.69
7	A	1407	5MC	C5-C4-N3	-2.37	119.32	121.75
7	A	1207	2MG	C5-C6-N1	2.30	118.46	114.07
28	a	2580	PSU	O4'-C1'-C2'	2.30	108.33	105.15
28	a	1962	5MC	O2-C2-N3	-2.29	118.71	122.33
7	A	516	PSU	O4'-C1'-C2'	2.22	108.22	105.15
28	a	2251	OMG	C5-C6-N1	2.21	118.29	114.07
28	a	746	PSU	C6-N1-C2	-2.21	120.64	122.69
7	A	967	5MC	C5-C4-N3	-2.21	119.49	121.75
28	a	747	5MU	C1'-N1-C2	2.19	121.53	117.59
7	A	966	2MG	C5-C6-N1	2.18	118.24	114.07
28	a	2605	PSU	C6-N1-C2	-2.17	120.67	122.69
7	A	966	2MG	CM2-N2-C2	-2.14	119.04	123.65
28	a	2445	2MG	N2-C2-N3	-2.13	117.80	120.51
7	A	1498	UR3	O4-C4-N3	-2.12	117.08	119.66
7	A	1498	UR3	C6-C5-C4	2.11	124.77	120.73
28	a	747	5MU	C1'-N1-C6	-2.10	117.69	121.15
28	a	1618	6MZ	C4-C5-N7	-2.10	107.11	109.34
28	a	1911	PSU	C6-N1-C2	-2.10	120.74	122.69
28	a	2457	PSU	O4'-C1'-C2'	2.08	108.03	105.15
7	A	1207	2MG	CM2-N2-C2	-2.08	119.19	123.65
28	a	747	5MU	C5M-C5-C6	-2.05	120.07	122.85
28	a	747	5MU	O2-C2-N1	-2.05	120.13	122.80
7	A	1516	2MG	CM2-N2-C2	-2.02	119.31	123.65
28	a	2503	2MA	N6-C6-N1	2.01	122.52	117.11
28	a	1962	5MC	C1'-N1-C6	-2.00	117.85	121.15

There are no chirality outliers.

All (36) torsion outliers are listed below:

For Manuscript Review

Mol	Chain	Res	Type	Atoms
39	l	81	4D4	NE-CD-CG-CB
7	A	966	2MG	O4'-C4'-C5'-O5'
7	A	1518	MA6	O4'-C4'-C5'-O5'
7	A	1519	MA6	C5-C6-N6-C9
17	K	119	IAS	N-CA-CB-CG
28	a	1915	3TD	O4'-C4'-C5'-O5'
7	A	527	G7M	C3'-C4'-C5'-O5'
7	A	1516	2MG	O4'-C4'-C5'-O5'
7	A	1519	MA6	O4'-C4'-C5'-O5'
28	a	1915	3TD	C3'-C4'-C5'-O5'
28	a	2504	PSU	O4'-C4'-C5'-O5'
39	l	82	MS6	CA-CB-CG-SD
28	a	2030	6MZ	O4'-C4'-C5'-O5'
28	a	2445	2MG	C3'-C4'-C5'-O5'
7	A	966	2MG	C3'-C4'-C5'-O5'
28	a	2030	6MZ	C3'-C4'-C5'-O5'
31	d	150	MEQ	NE2-CD-CG-CB
31	d	150	MEQ	OE1-CD-CG-CB
7	A	527	G7M	O4'-C4'-C5'-O5'
28	a	2445	2MG	O4'-C4'-C5'-O5'
28	a	2504	PSU	C3'-C4'-C5'-O5'
39	l	82	MS6	CB-CG-SD-CE
7	A	1519	MA6	C3'-C4'-C5'-O5'
7	A	1518	MA6	C3'-C4'-C5'-O5'
7	A	1402	4OC	C1'-C2'-O2'-CM2
17	K	119	IAS	CA-CB-CG-OD1
28	a	2552	OMU	C3'-C2'-O2'-CM2
7	A	1516	2MG	C3'-C4'-C5'-O5'
7	A	527	G7M	C4'-C5'-O5'-P
28	a	2580	PSU	O4'-C4'-C5'-O5'
39	l	81	4D4	CG-CD-NE-CZ
28	a	2552	OMU	C1'-C2'-O2'-CM2
28	a	746	PSU	O4'-C1'-C5-C6
28	a	747	5MU	C3'-C4'-C5'-O5'
39	l	82	MS6	C-CA-CB-CG
39	l	81	4D4	O-C-CA-CB

There are no ring outliers.

10 monomers are involved in 13 short contacts:

Mol	Chain	Res	Type	Clashes	Symm-Clashes
28	a	747	5MU	1	0
28	a	2445	2MG	1	0

Continued on next page...

For Manuscript Review

Continued from previous page...

Mol	Chain	Res	Type	Clashes	Symm-Clashes
28	a	1915	3TD	1	0
28	a	2503	2MA	1	0
28	a	955	PSU	1	0
28	a	2504	PSU	1	0
7	A	516	PSU	1	0
28	a	2030	6MZ	4	0
28	a	2251	OMG	1	0
7	A	1518	MA6	1	0

5.5 Carbohydrates (i)

There are no oligosaccharides in this entry.

5.6 Ligand geometry (i)

Of 326 ligands modelled in this entry, 309 are monoatomic - leaving 17 for Mogul analysis.

In the following table, the Counts columns list the number of bonds (or angles) for which Mogul statistics could be retrieved, the number of bonds (or angles) that are observed in the model and the number of bonds (or angles) that are defined in the Chemical Component Dictionary. The Link column lists molecule types, if any, to which the group is linked. The Z score for a bond length (or angle) is the number of standard deviations the observed value is removed from the expected value. A bond length (or angle) with $|Z| > 2$ is considered an outlier worth inspection. RMSZ is the root-mean-square of all Z scores of the bond lengths (or angles).

Mol	Type	Chain	Res	Link	Bond lengths			Bond angles		
					Counts	RMSZ	#$|Z| > 2$	Counts	RMSZ	#$|Z| > 2$
57	SPM	a	6221	-	13,13,13	0.36	0	12,12,12	0.94	0
58	C	a	6223	-	18,21,22	0.29	0	25,30,33	0.36	0
56	SPD	a	6208	-	9,9,9	0.30	0	8,8,8	0.79	0
56	SPD	a	6218	-	9,9,9	0.29	0	8,8,8	0.91	0
56	SPD	a	6216	-	9,9,9	0.35	0	8,8,8	0.82	0
56	SPD	a	6220	-	9,9,9	0.33	0	8,8,8	0.77	0
56	SPD	a	6210	-	9,9,9	0.33	0	8,8,8	0.85	0
56	SPD	a	6214	-	9,9,9	0.32	0	8,8,8	0.81	0
56	SPD	a	6217	-	9,9,9	0.33	0	8,8,8	0.71	0
56	SPD	a	6215	-	9,9,9	0.33	0	8,8,8	0.85	0
56	SPD	a	6209	-	9,9,9	0.30	0	8,8,8	0.85	0
56	SPD	a	6212	-	9,9,9	0.33	0	8,8,8	0.84	0
56	SPD	A	1690	-	9,9,9	0.32	0	8,8,8	0.81	0
56	SPD	a	6219	-	9,9,9	0.33	0	8,8,8	0.80	0
59	G	a	6224	-	18,25,26	1.05	2 (11%)	17,37,40	0.74	1 (5%)

For Manuscript Review

Mol	Type	Chain	Res	Link	Bond lengths			Bond angles		
					Counts	RMSZ	#|Z| > 2	Counts	RMSZ	#|Z| > 2
56	SPD	a	6213	-	9,9,9	0.33	0	8,8,8	0.77	0
56	SPD	a	6211	-	9,9,9	0.32	0	8,8,8	0.79	0

In the following table, the Chirals column lists the number of chiral outliers, the number of chiral centers analysed, the number of these observed in the model and the number defined in the Chemical Component Dictionary. Similar counts are reported in the Torsion and Rings columns. '-' means no outliers of that kind were identified.

Mol	Type	Chain	Res	Link	Chirals	Torsions	Rings
57	SPM	a	6221	-	-	3/11/11/11	-
58	C	a	6223	-	-	0/7/25/26	0/2/2/2
56	SPD	a	6208	-	-	1/7/7/7	-
56	SPD	a	6218	-	-	3/7/7/7	-
56	SPD	a	6216	-	-	2/7/7/7	-
56	SPD	a	6220	-	-	3/7/7/7	-
56	SPD	a	6210	-	-	2/7/7/7	-
56	SPD	a	6214	-	-	3/7/7/7	-
56	SPD	a	6217	-	-	4/7/7/7	-
56	SPD	a	6215	-	-	3/7/7/7	-
56	SPD	a	6209	-	-	2/7/7/7	-
56	SPD	a	6212	-	-	3/7/7/7	-
56	SPD	A	1690	-	-	3/7/7/7	-
56	SPD	a	6219	-	-	0/7/7/7	-
59	G	a	6224	-	-	0/3/25/26	0/3/3/3
56	SPD	a	6213	-	-	0/7/7/7	-
56	SPD	a	6211	-	-	3/7/7/7	-

All (2) bond length outliers are listed below:

Mol	Chain	Res	Type	Atoms	Z	Observed(Å)	Ideal(Å)
59	a	6224	G	C5-C6	-2.57	1.42	1.47
59	a	6224	G	C8-N7	-2.49	1.30	1.34

All (1) bond angle outliers are listed below:

Mol	Chain	Res	Type	Atoms	Z	Observed(^{o})	Ideal(^{o})
59	a	6224	G	O6-C6-C5	2.13	128.55	124.32

There are no chirality outliers.

For Manuscript Review

All (35) torsion outliers are listed below:

Mol	Chain	Res	Type	Atoms
56	a	6215	SPD	N6-C7-C8-C9
56	a	6212	SPD	C3-C4-C5-N6
56	a	6217	SPD	C3-C4-C5-N6
56	a	6210	SPD	C3-C4-C5-N6
56	a	6211	SPD	C3-C4-C5-N6
56	a	6214	SPD	C3-C4-C5-N6
57	a	6221	SPM	C7-C8-C9-N10
56	A	1690	SPD	C3-C4-C5-N6
56	a	6211	SPD	N6-C7-C8-C9
56	a	6214	SPD	C8-C7-N6-C5
56	a	6218	SPD	C8-C7-N6-C5
56	a	6218	SPD	N6-C7-C8-C9
56	a	6220	SPD	C4-C5-N6-C7
56	a	6215	SPD	C3-C4-C5-N6
56	a	6216	SPD	C2-C3-C4-C5
56	a	6209	SPD	C2-C3-C4-C5
57	a	6221	SPM	C8-C9-N10-C11
56	a	6212	SPD	C4-C5-N6-C7
56	a	6220	SPD	N6-C7-C8-C9
56	a	6218	SPD	C3-C4-C5-N6
56	a	6216	SPD	N1-C2-C3-C4
56	a	6210	SPD	N1-C2-C3-C4
56	A	1690	SPD	C7-C8-C9-N10
56	a	6209	SPD	C7-C8-C9-N10
56	a	6217	SPD	C8-C7-N6-C5
56	a	6208	SPD	C8-C7-N6-C5
56	a	6211	SPD	C8-C7-N6-C5
56	a	6215	SPD	C8-C7-N6-C5
56	A	1690	SPD	N6-C7-C8-C9
56	a	6217	SPD	C4-C5-N6-C7
57	a	6221	SPM	N10-C11-C12-C13
56	a	6214	SPD	C2-C3-C4-C5
56	a	6220	SPD	C2-C3-C4-C5
56	a	6217	SPD	C7-C8-C9-N10
56	a	6212	SPD	C8-C7-N6-C5

There are no ring outliers.

11 monomers are involved in 23 short contacts:

Mol	Chain	Res	Type	Clashes	Symm-Clashes
57	a	6221	SPM	1	0

Continued on next page...

For Manuscript Review

Continued from previous page...

Mol	Chain	Res	Type	Clashes	Symm-Clashes
56	a	6216	SPD	2	0
56	a	6220	SPD	1	0
56	a	6214	SPD	2	0
56	a	6217	SPD	5	0
56	a	6215	SPD	1	0
56	a	6209	SPD	6	0
56	a	6212	SPD	1	0
56	A	1690	SPD	2	0
56	a	6219	SPD	1	0
56	a	6211	SPD	1	0

The following is a two-dimensional graphical depiction of Mogul quality analysis of bond lengths, bond angles, torsion angles, and ring geometry for all instances of the Ligand of Interest. In addition, ligands with molecular weight > 250 and outliers as shown on the validation Tables will also be included. For torsion angles, if less then 5% of the Mogul distribution of torsion angles is within 10 degrees of the torsion angle in question, then that torsion angle is considered an outlier. Any bond that is central to one or more torsion angles identified as an outlier by Mogul will be highlighted in the graph. For rings, the root-mean-square deviation (RMSD) between the ring in question and similar rings identified by Mogul is calculated over all ring torsion angles. If the average RMSD is greater than 60 degrees and the minimal RMSD between the ring in question and any Mogul-identified rings is also greater than 60 degrees, then that ring is considered an outlier. The outliers are highlighted in purple. The color gray indicates Mogul did not find sufficient equivalents in the CSD to analyse the geometry.

Ligand G a 6224

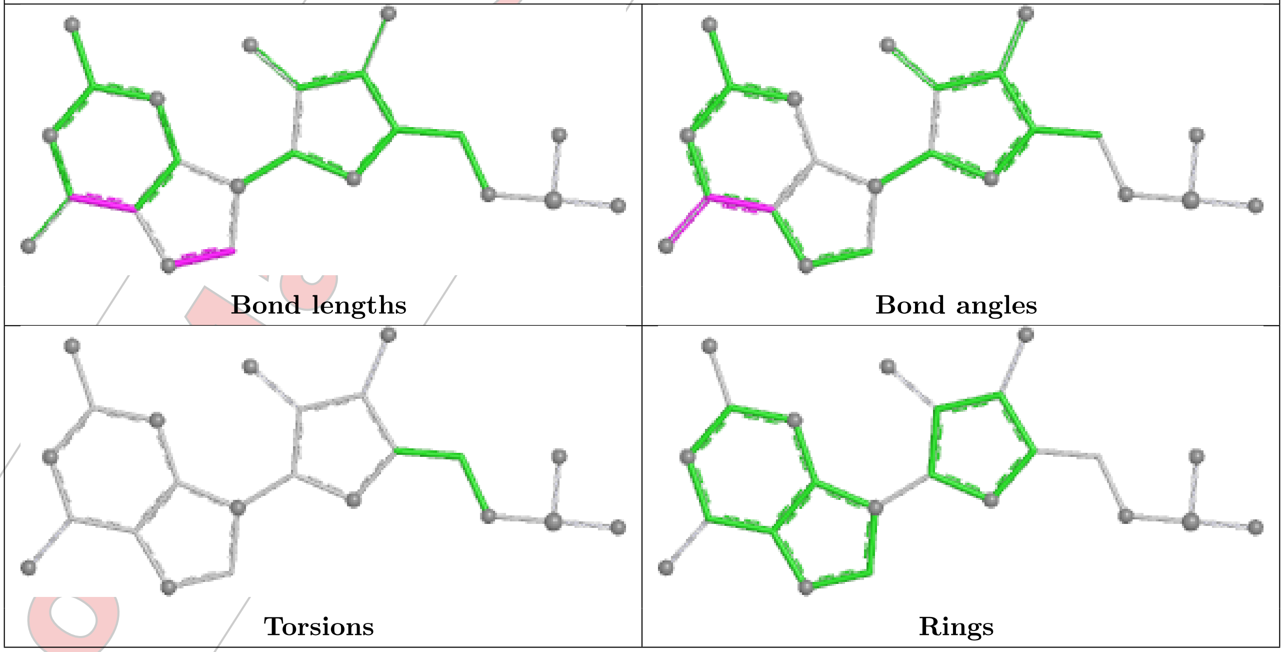

For Manuscript Review

5.7 Other polymers (i)

There are no such residues in this entry.

5.8 Polymer linkage issues (i)

The following chains have linkage breaks:

Mol	Chain	Number of breaks
28	a	4

All chain breaks are listed below:

Model	Chain	Residue-1	Atom-1	Residue-2	Atom-2	Distance (Å)
1	a	1052:C	O3'	1107:G	P	17.83
1	a	2098:U	O3'	2191:A	P	17.80
1	a	1172:C	O3'	1177:G	P	15.94
1	a	1914:C	O3'	1915:3TD	P	4.71

For Manuscript Review

6 Map visualisation (i)

This section contains visualisations of the EMDB entry EMD-75605. These allow visual inspection of the internal detail of the map and identification of artifacts.

Images derived from a raw map, generated by summing the deposited half-maps, are presented below the corresponding image components of the primary map to allow further visual inspection and comparison with those of the primary map.

6.1 Orthogonal projections (i)

6.1.1 Primary map

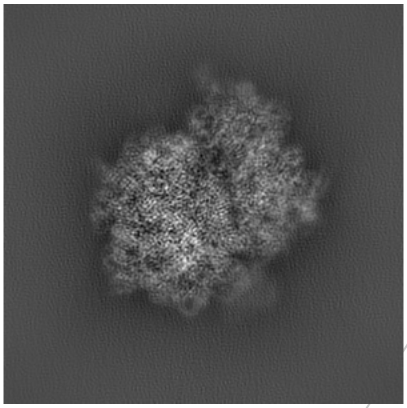
X

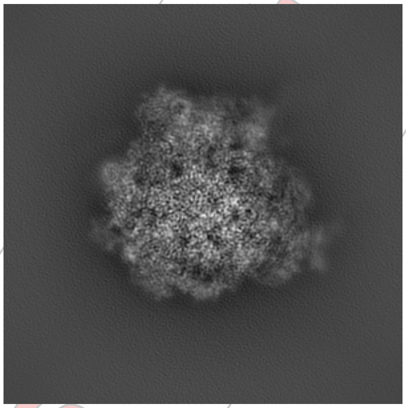
Y

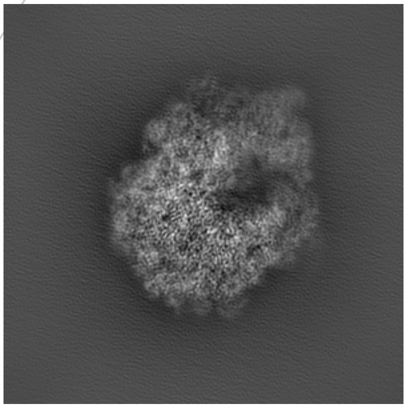
Z

6.1.2 Raw map

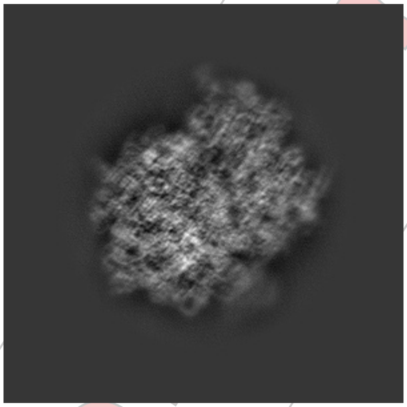
X

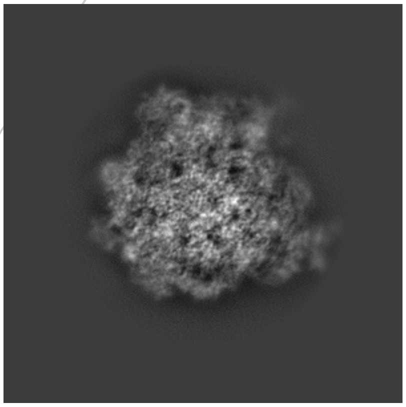
Y

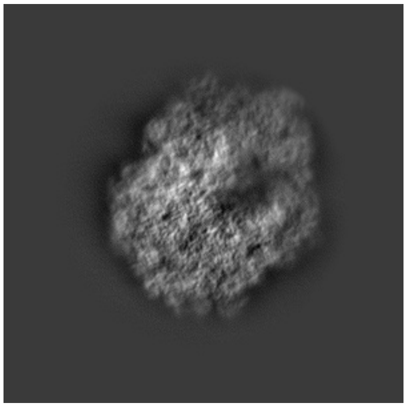
Z

The images above show the map projected in three orthogonal directions.

For Manuscript Review

6.2 Central slices (i)

6.2.1 Primary map

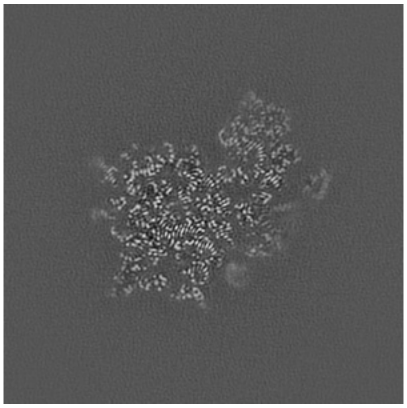
X Index: 240

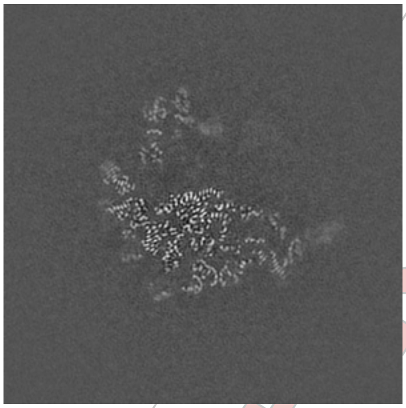
Y Index: 240

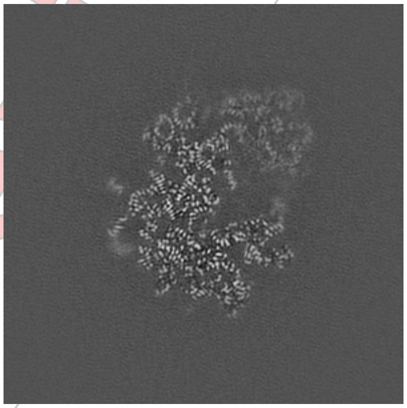
Z Index: 240

6.2.2 Raw map

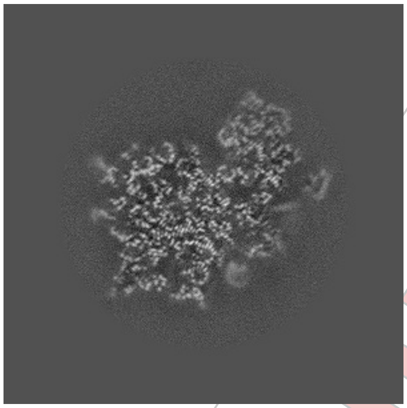
X Index: 240

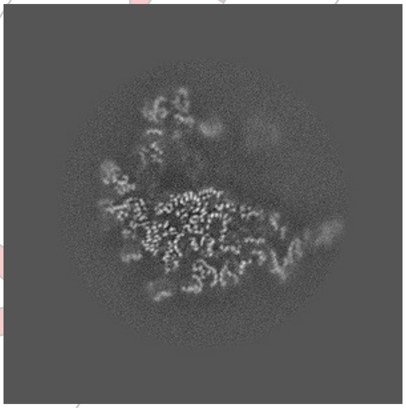
Y Index: 240

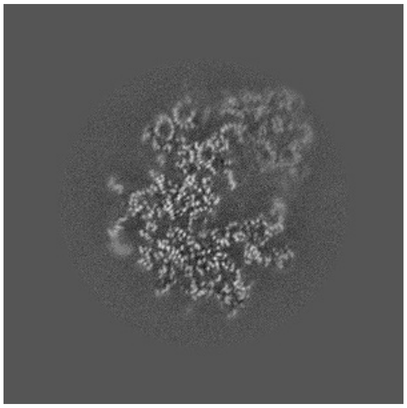
Z Index: 240

The images above show central slices of the map in three orthogonal directions.

For Manuscript Review

6.3 Largest variance slices ⓘ

6.3.1 Primary map

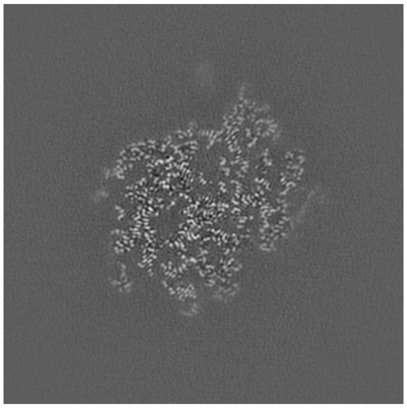
X Index: 217

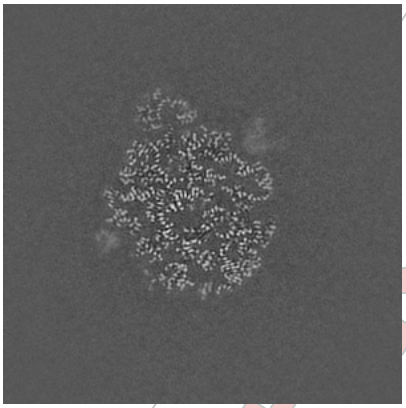
Y Index: 207

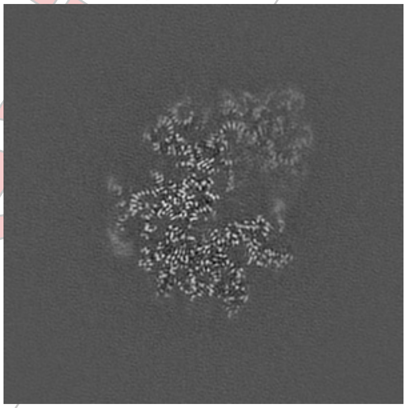
Z Index: 238

6.3.2 Raw map

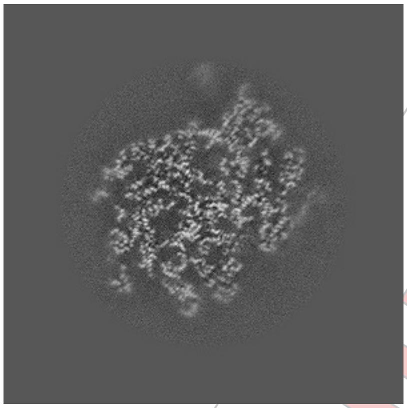
X Index: 216

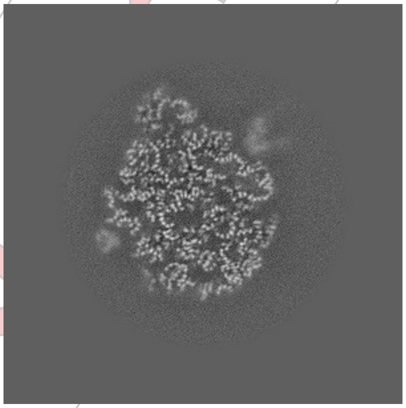
Y Index: 207

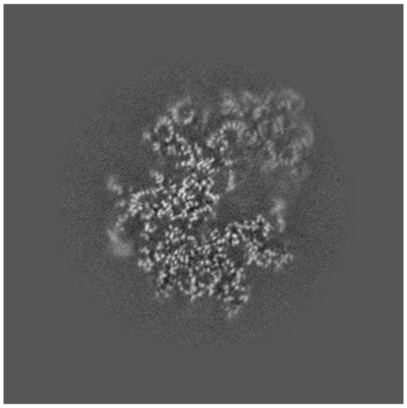
Z Index: 238

The images above show the largest variance slices of the map in three orthogonal directions.

For Manuscript Review

6.4 Orthogonal standard-deviation projections (False-color) (i)

6.4.1 Primary map

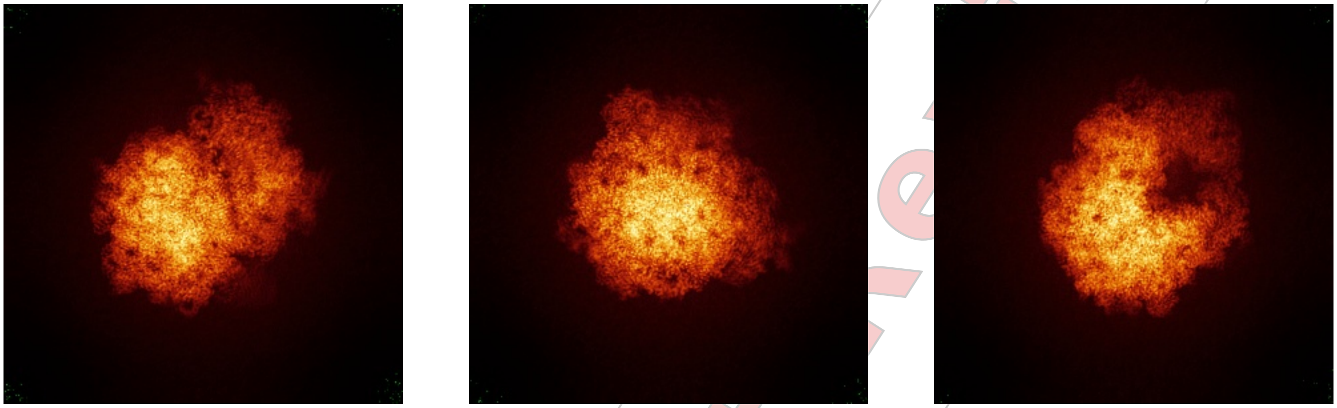

X Y Z

6.4.2 Raw map

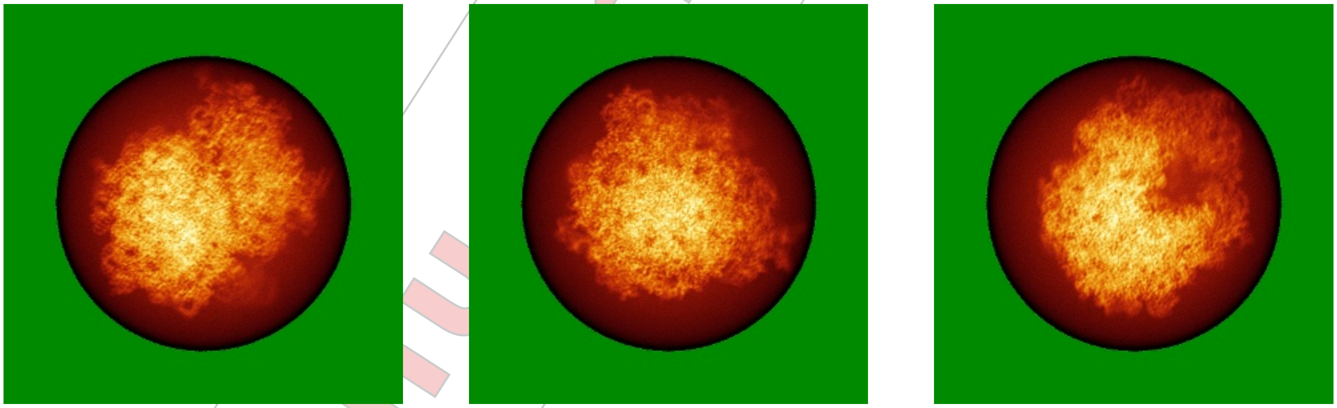

X Y Z

The images above show the map standard deviation projections with false color in three orthogonal directions. Minimum values are shown in green, max in blue, and dark to light orange shades represent small to large values respectively.

For Manuscript Review

6.5 Orthogonal surface views (i)

6.5.1 Primary map

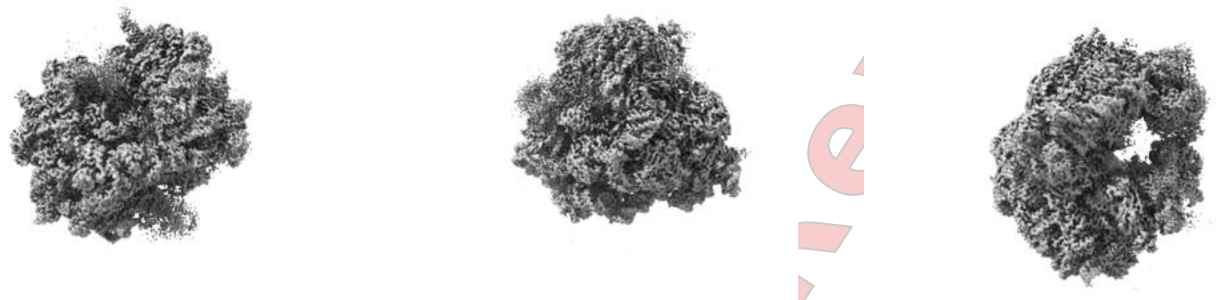

X Y Z

The images above show the 3D surface view of the map at the recommended contour level 2.5. These images, in conjunction with the slice images, may facilitate assessment of whether an appropriate contour level has been provided.

6.5.2 Raw map

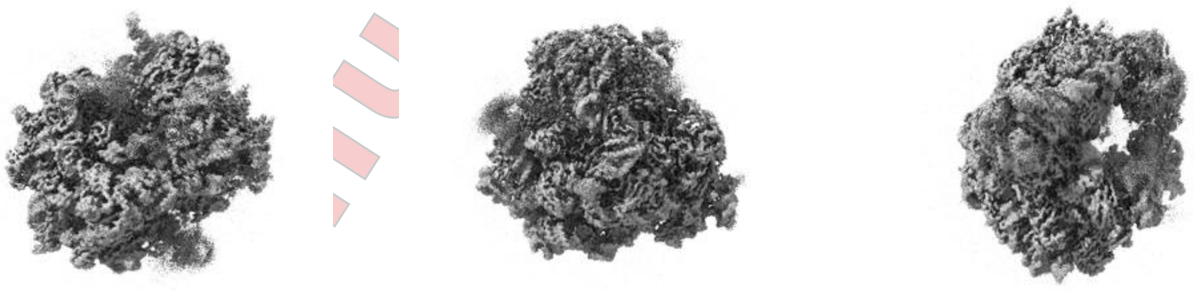

X Y Z

These images show the 3D surface of the raw map. The raw map's contour level was selected so that its surface encloses the same volume as the primary map does at its recommended contour level.

6.6 Mask visualisation (i)

This section was not generated. No masks/segmentation were deposited.

For Manuscript Review

7 Map analysis (i)

This section contains the results of statistical analysis of the map.

7.1 Map-value distribution (i)

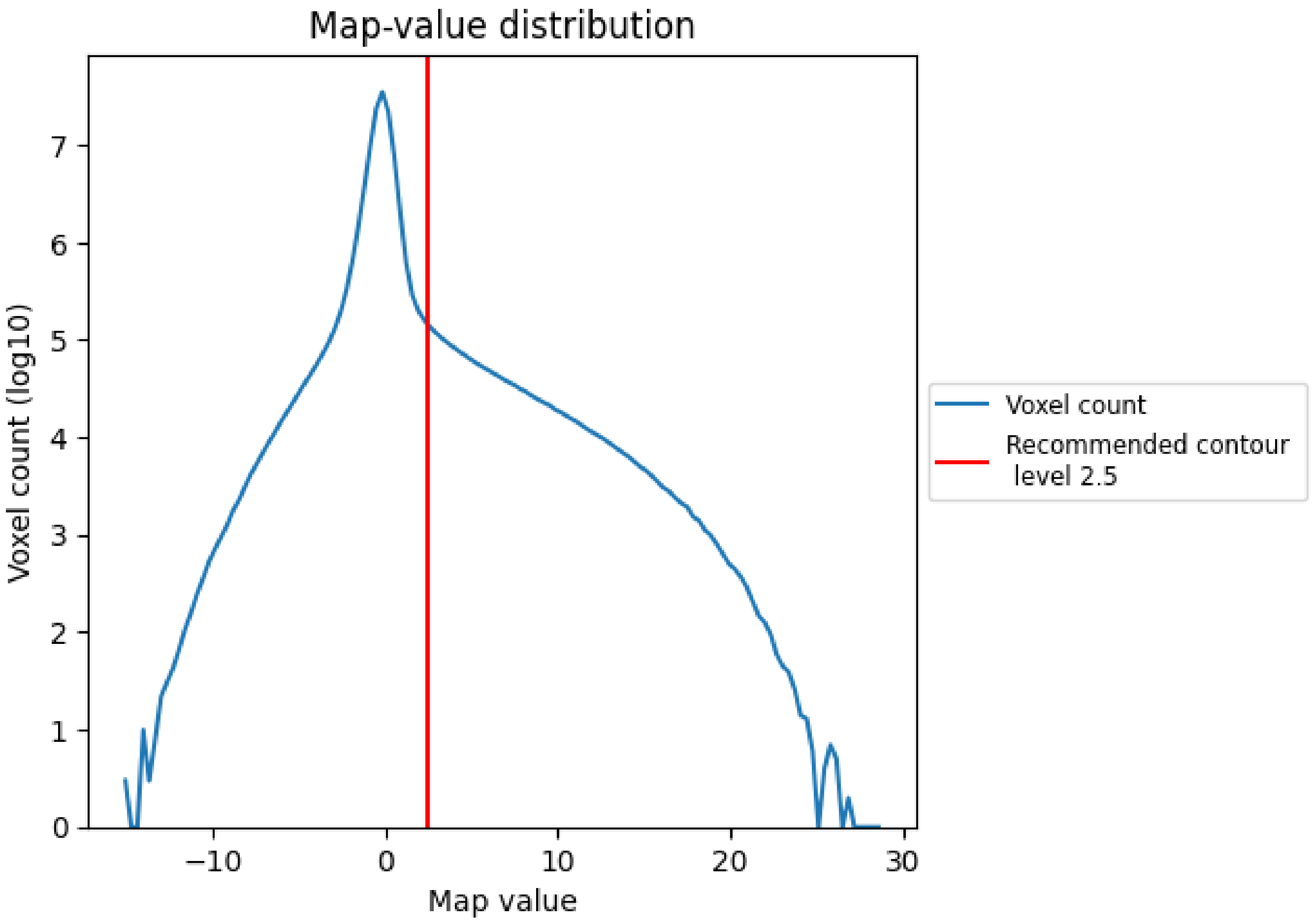

The map-value distribution is plotted in 128 intervals along the x-axis. The y-axis is logarithmic. A spike in this graph at zero usually indicates that the volume has been masked.

For Manuscript Review

7.2 Volume estimate (i)

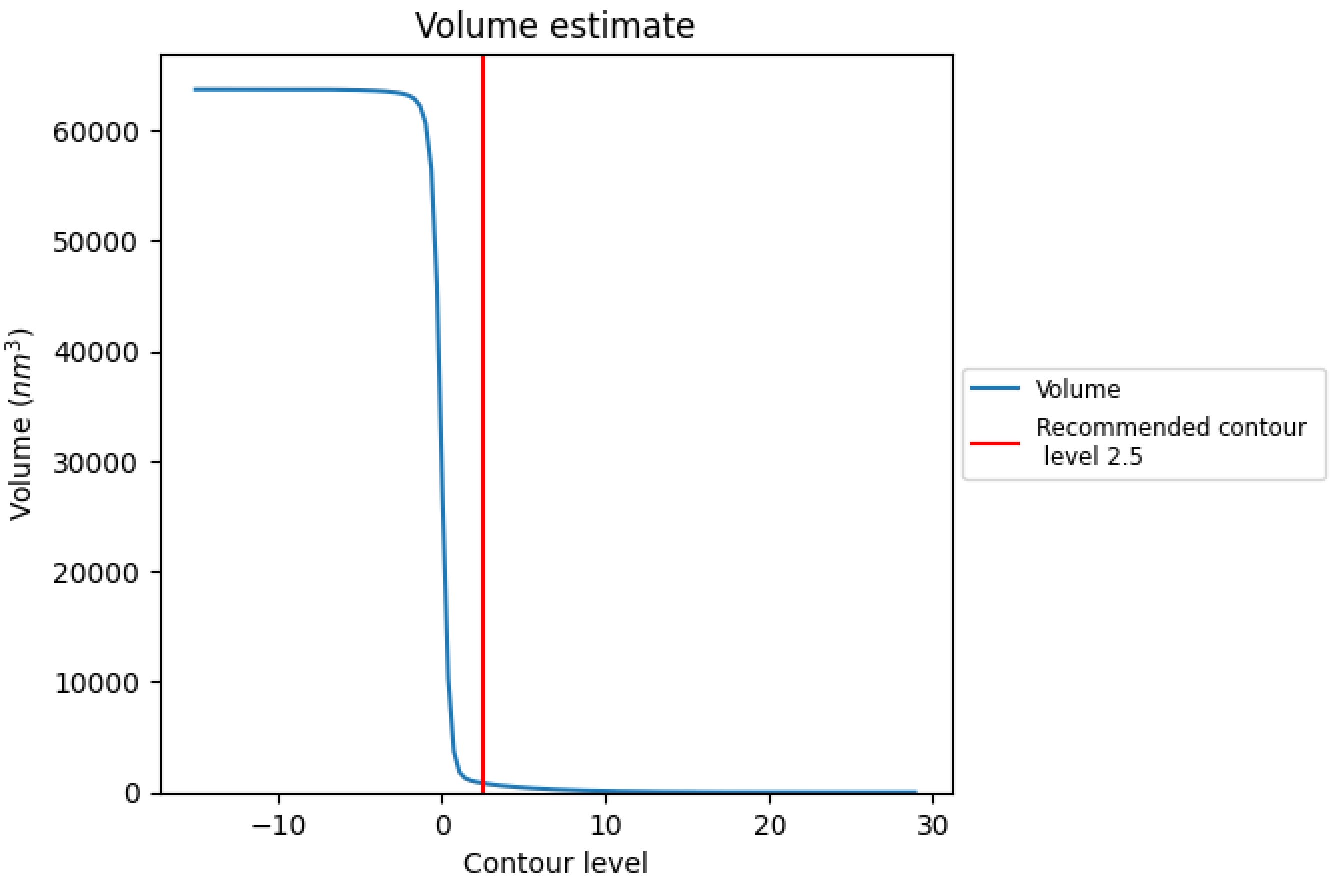

The volume at the recommended contour level is 837 nm^3; this corresponds to an approximate mass of 756 kDa.

The volume estimate graph shows how the enclosed volume varies with the contour level. The recommended contour level is shown as a vertical line and the intersection between the line and the curve gives the volume of the enclosed surface at the given level.

For Manuscript Review

7.3 Rotationally averaged power spectrum (i)

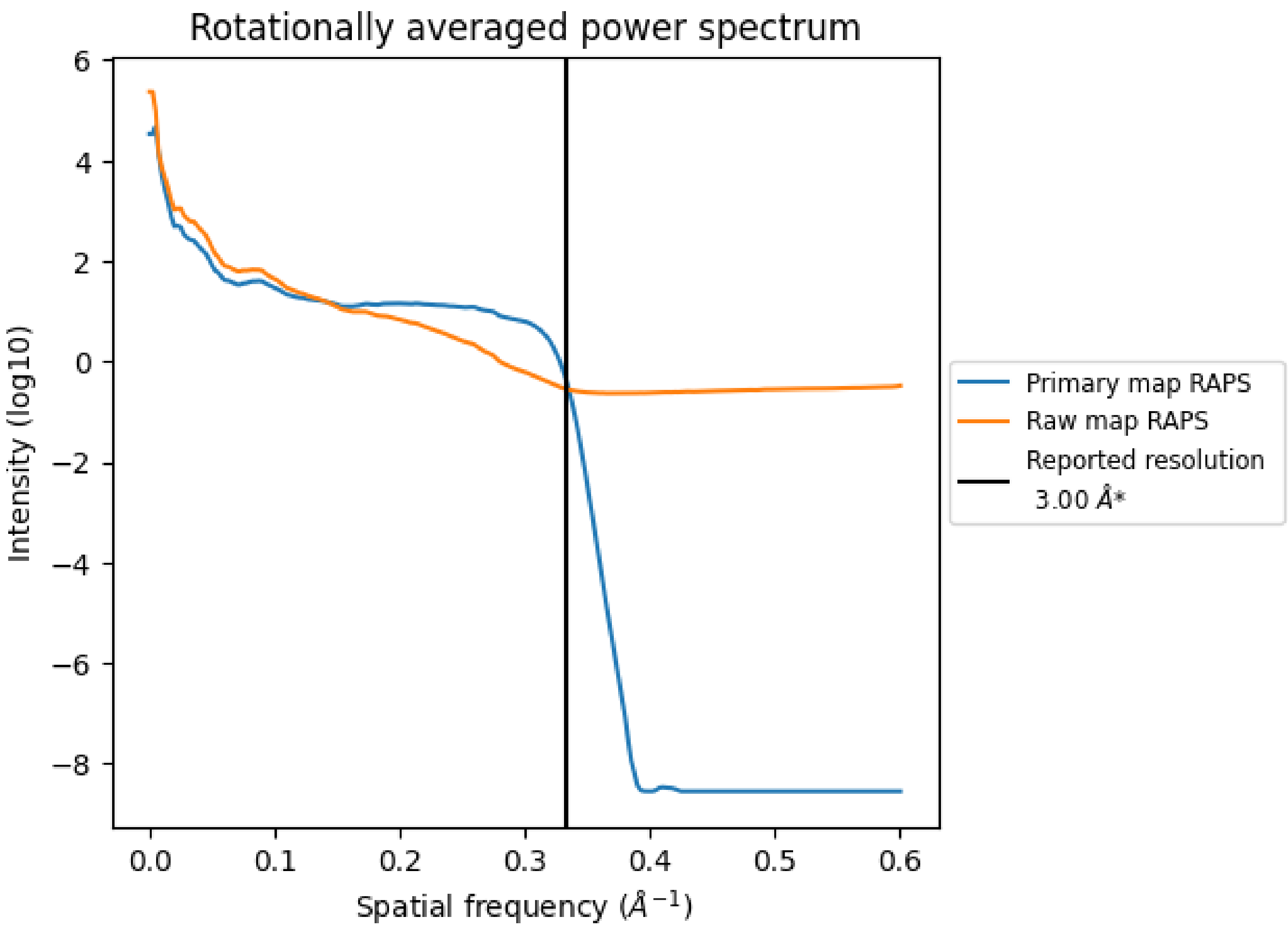

*Reported resolution corresponds to spatial frequency of 0.333 Å^{-1}

For Manuscript Review

8 Fourier-Shell correlation ⓘ

Fourier-Shell Correlation (FSC) is the most commonly used method to estimate the resolution of single-particle and subtomogram-averaged maps. The shape of the curve depends on the imposed symmetry, mask and whether or not the two 3D reconstructions used were processed from a common reference. The reported resolution is shown as a black line. A curve is displayed for the half-bit criterion in addition to lines showing the 0.143 gold standard cut-off and 0.5 cut-off.

8.1 FSC ⓘ

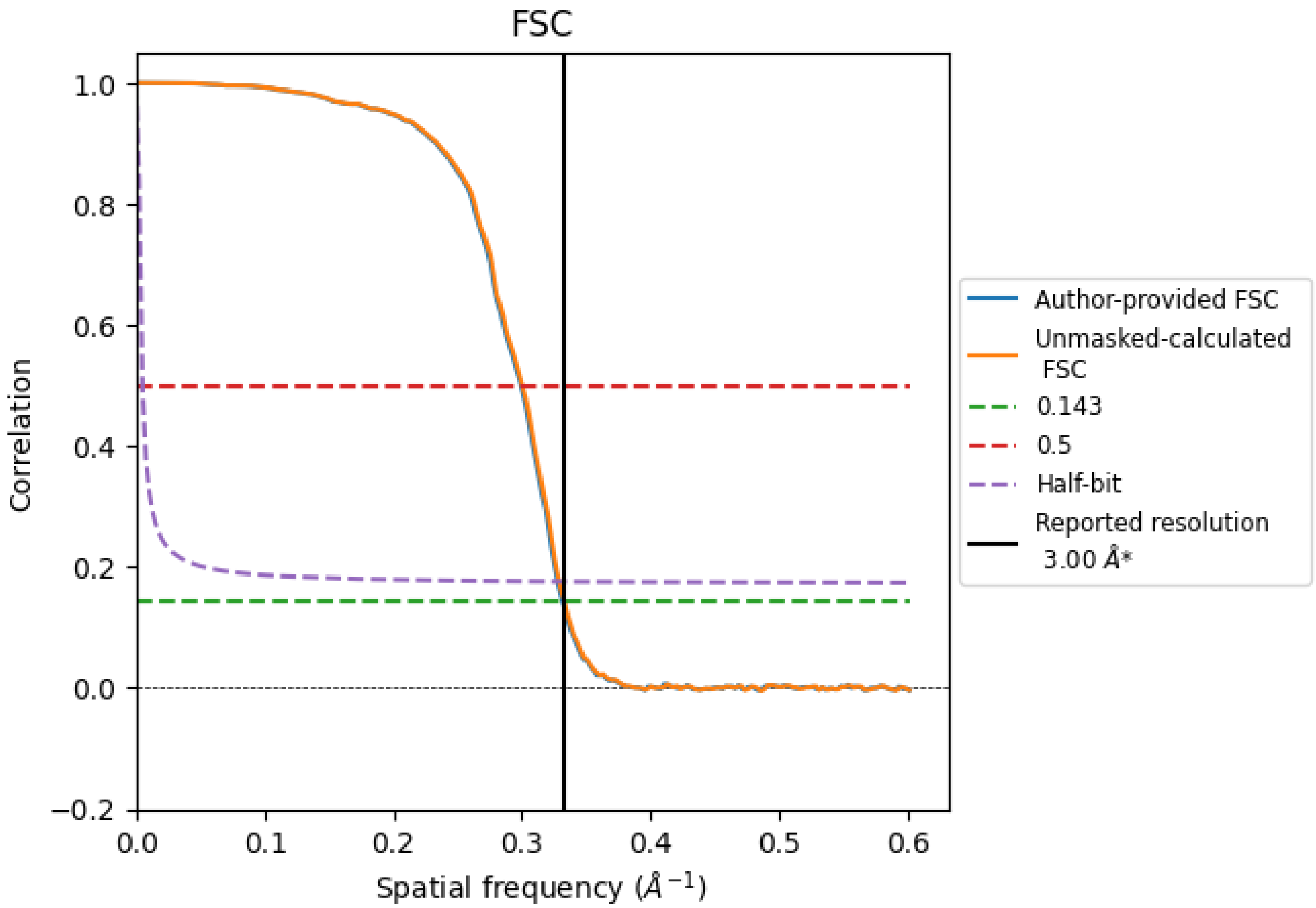

*Reported resolution corresponds to spatial frequency of 0.333 Å^{-1}

For Manuscript Review

8.2 Resolution estimates (i)

Resolution estimate (Å)	Estimation criterion (FSC cut-off)		
	0.143	0.5	Half-bit
Reported by author	3.00	-	-
Author-provided FSC curve	3.02	3.34	3.05
Unmasked-calculated*	3.00	3.33	3.04

*Resolution estimate based on FSC curve calculated by comparison of deposited half-maps.

For Manuscript Review

9 Map-model fit (i)

This section contains information regarding the fit between EMDB map EMD-75605 and PDB model 11BQ. Per-residue inclusion information can be found in section 3 on page 17.

9.1 Map-model overlay (i)

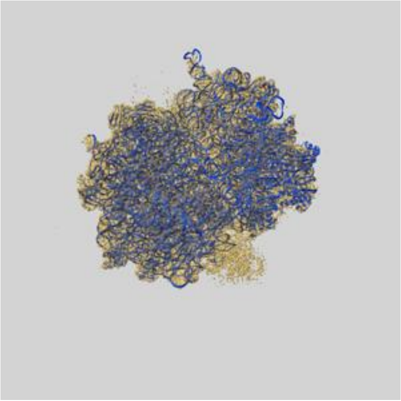

X

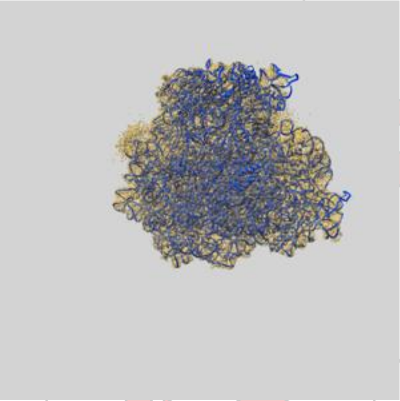

Y

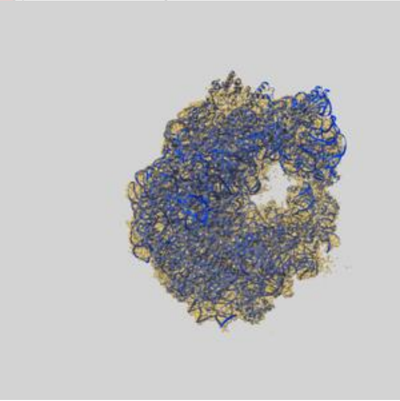

Z

The images above show the 3D surface view of the map at the recommended contour level 2.5 at 50% transparency in yellow overlaid with a ribbon representation of the model coloured in blue. These images allow for the visual assessment of the quality of fit between the atomic model and the map.

For Manuscript Review

9.2 Q-score mapped to coordinate model (i)

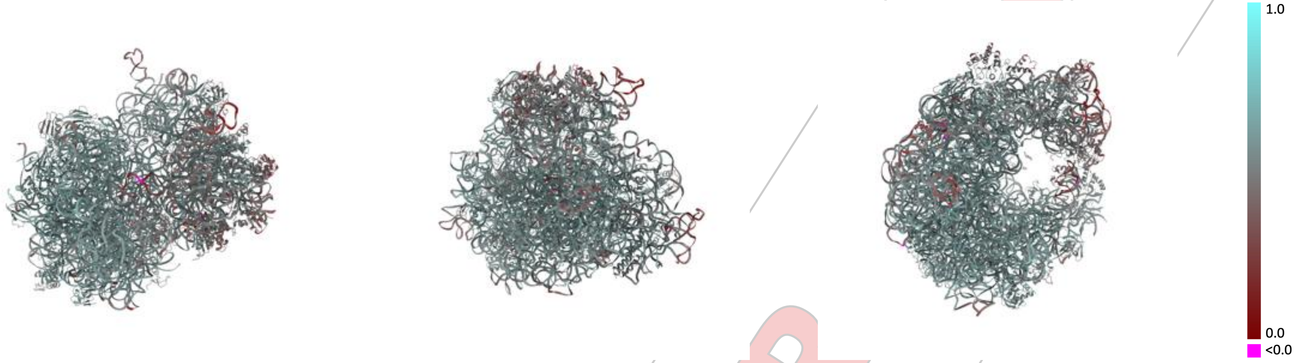

X Y Z

The images above show the model with each residue coloured according its Q-score. This shows their resolvability in the map with higher Q-score values reflecting better resolvability. Please note: Q-score is calculating the resolvability of atoms, and thus high values are only expected at resolutions at which atoms can be resolved. Low Q-score values may therefore be expected for many entries.

9.3 Atom inclusion mapped to coordinate model (i)

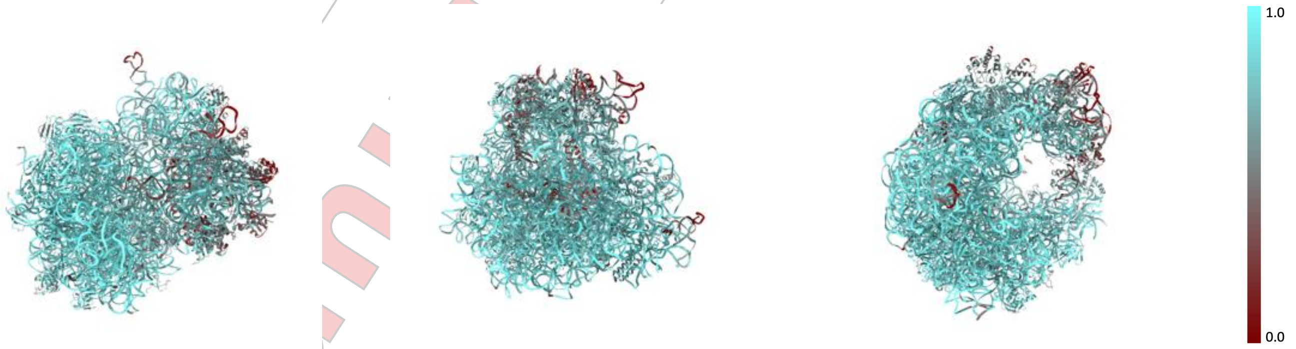

X Y Z

The images above show the model with each residue coloured according to its atom inclusion. This shows to what extent they are inside the map at the recommended contour level (2.5).

For Manuscript Review

9.4 Atom inclusion (i)

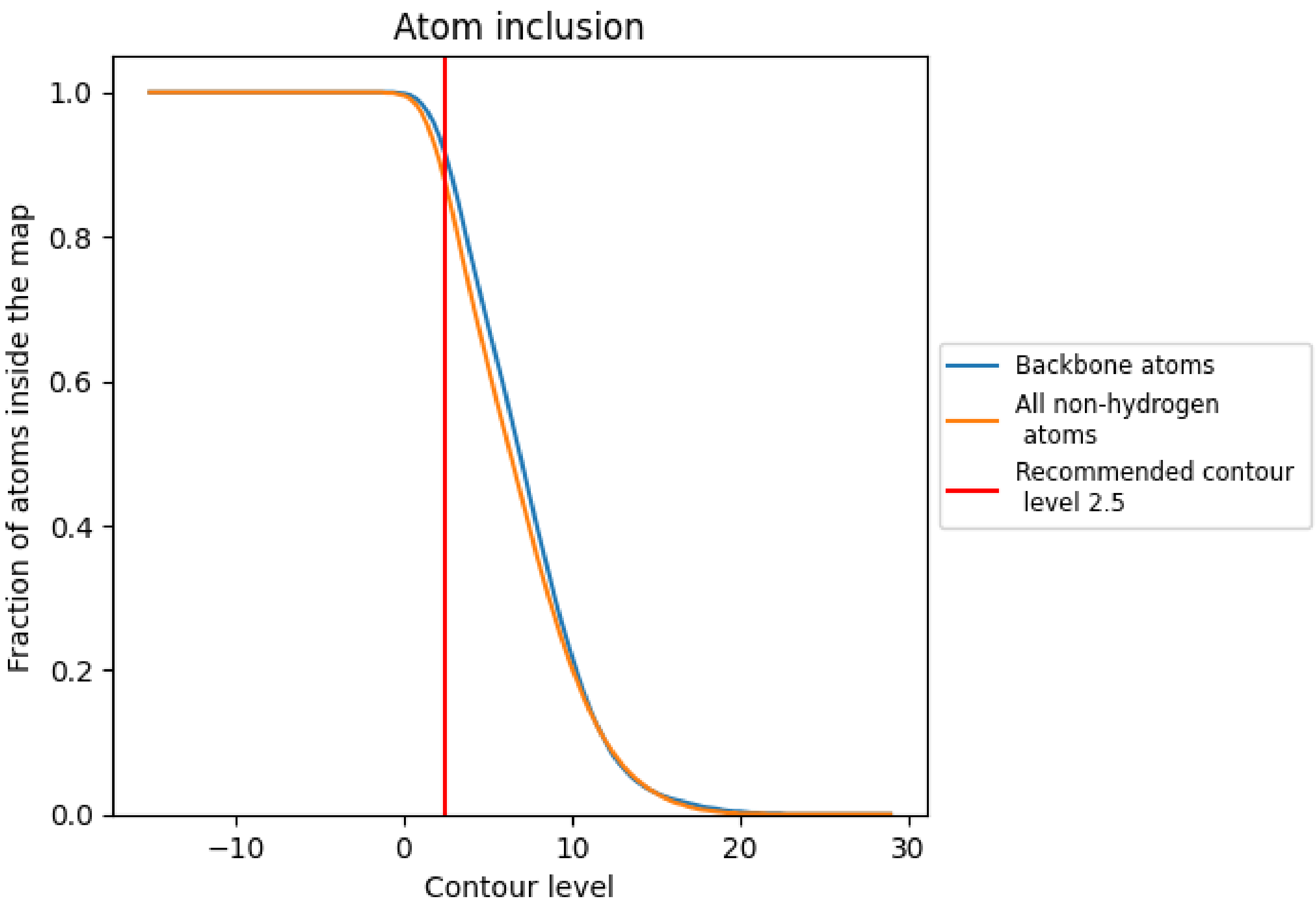

At the recommended contour level, 91% of all backbone atoms, 87% of all non-hydrogen atoms, are inside the map.

For Manuscript Review

9.5 Map-model fit summary (i)

The table lists the average atom inclusion at the recommended contour level (2.5) and Q-score for the entire model and for each chain.

Chain	Atom inclusion	Q-score
All	0.8730	0.5550
0	0.7430	0.5710
1	0.9320	0.6060
2	0.9210	0.6160
3	0.9150	0.6010
4	0.5640	0.4420
5	0.6430	0.5640
A	0.8640	0.5260
B	0.5130	0.4710
C	0.6170	0.5160
D	0.6880	0.5330
E	0.8030	0.5620
F	0.7900	0.5240
G	0.4370	0.4270
H	0.8030	0.5660
I	0.5310	0.4650
J	0.3930	0.4480
K	0.8140	0.5430
L	0.8590	0.5750
M	0.5380	0.4490
N	0.6050	0.5040
O	0.8540	0.5570
P	0.7010	0.4940
Q	0.6980	0.4510
R	0.7920	0.5360
S	0.4970	0.4610
T	0.7570	0.5330
U	0.3900	0.4530
a	0.9530	0.5780
b	0.9270	0.5520
c	0.9380	0.6060
d	0.9190	0.6040
e	0.8200	0.5710
f	0.6580	0.4810
g	0.7560	0.5130

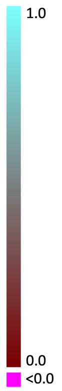

Continued on next page...

For Manuscript Review

Continued from previous page...

Chain	Atom inclusion	Q-score
h	0.7300	0.5090
i	0.9150	0.5980
j	0.9070	0.6000
k	0.8780	0.5860
l	0.9020	0.5890
m	0.9350	0.6040
n	0.8230	0.5590
o	0.8940	0.5990
p	0.8980	0.6020
q	0.8720	0.5940
r	0.8820	0.5980
s	0.8520	0.5800
t	0.7940	0.5590
u	0.8440	0.5720
v	0.8850	0.5990
w	0.9070	0.5960
x	0.7730	0.5360
y	0.8600	0.5800
z	0.8790	0.5890

For Manuscript Review
